# A correlation-based feature analysis of physical examination indicators can help predict the overall underlying health status using machine learning

**DOI:** 10.1038/s41598-022-20474-3

**Published:** 2022-11-15

**Authors:** Haixin Wang, Ping Shuai, Yanhui Deng, Jiyun Yang, Yi Shi, Dongyu Li, Tao Yong, Yuping Liu, Lulin Huang

**Affiliations:** 1 Sichuan Provincial Key Laboratory for Human Disease Gene Study, Center for Medical Genetics, Sichuan Academy of Medical Sciences & Sichuan Provincial People′s Hospital, University of Electronic Science and Technology of China, Chengdu, China; 2 Research Unit for Blindness Prevention of Chinese Academy of Medical Sciences (2019RU026), Sichuan Academy of Medical Sciences, 32 The First Ring Road West 2, Chengdu, 610072 Sichuan China; 3 Health Management Center and Physical Examination Center of Sichuan Provincial People’s Hospital, School of Medicine, University of Electronic Science and Technology of China, Chengdu, China; 4 Medical Information Center of Sichuan Provincial People’s Hospital, School of Medicine, University of Electronic Science and Technology of China, Chengdu, China

**Keywords:** Bioinformatics, Disease prevention

## Abstract

As a systematic investigation of the correlations between physical examination indicators (PEIs) is lacking, most PEIs are currently independently used for disease warning. This results in the general physical examination having limited diagnostic values. Here, we systematically analyzed the correlations in 221 PEIs between healthy and 34 unhealthy statuses in 803,614 individuals in China. Specifically, the study population included 711,928 healthy participants, 51,341 patients with hypertension, 12,878 patients with diabetes, and 34,997 patients with other unhealthy statuses. We found rich relevance between PEIs in the healthy physical status (7662 significant correlations, 31.5%). However, in the disease conditions, the PEI correlations changed. We focused on the difference in PEIs between healthy and 35 unhealthy physical statuses and found 1239 significant PEI differences, suggesting that they could be candidate disease markers. Finally, we established machine learning algorithms to predict health status using 15–16% of the PEIs through feature extraction, reaching a 66–99% accurate prediction, depending on the physical status. This new reference of the PEI correlation provides rich information for chronic disease diagnosis. The developed machine learning algorithms can fundamentally affect the practice of general physical examinations.

## Introduction

A comprehensive primary healthcare system has a broader effect on human health than does clinical medical treatment^1^. Health examinations help healthy individuals improve their understanding of their physical functions and maintain their health status, as well as inform individuals of the health benefits of changing unhealthy habits and avoiding risk factors that can lead to disease^2^. Physical examinations can help minimize the distress of diseases^3^. As the population size grows and ages, healthcare needs continuously increase, and healthcare provisions become more sophisticated and thus more costly^4^.

Health examinations are common elements of healthcare in developed countries^5^. They consist of a general blood examination, urine examination, blood glucose examination, blood lipid examination, and renal function examination, among others. However, currently, a physical examination report is assessed mainly based on one or two independent physical examination indicators (PEIs), which can provide limited information to physical examiners about their health condition or disease diagnosis^6^. The correlations between PEI in different physical states (i.e., healthy, hypertension, and diabetes) have not been systematically investigated, even though they are expected to provide valuable information on public healthcare, for example, by defining a small set of easily measurable PEIs that can be used in the accurate diagnosis of a disease before the disease phenogenesis.

The recent explosion of available health data promises to transform healthcare by improving care quality and, therefore, population health while constraining escalating costs^7^. Health examination centers generate systematic big data that can reveal otherwise undetected underlying health issues^8^. In clinical practice, there is a growing investment in developing big data applications for medical care, such as those based on artificial intelligence (AI), to diagnose diseases based on clinical images^9^. Although artificial intelligence can save costs and improve efficiency, especially for the early diagnosis and prevention of chronic diseases^10^, currently, no prediction models have been generated for physical status predictions based on PEIs because of insufficient systematic analyses of PEIs in physical status.

As healthcare reform has made impressive progress in the expansion of insurance coverage, the general physical examination industry has accumulated big data^11^. By using a large dataset of general health examinations of the Chinese population, the present study aimed to determine the correlations in PEIs between healthy and unhealthy (i.e., those with underlying chronic diseases) patients, to elucidate the relationship between chronic disorders and normal individuals for these PEIs to discover candidate disease markers, and to develop machine learning (ML) models that could predict individual health statuses using a refined set of PEIs. To address these points, we used physical examination data from 80,3614 individuals who visited one health examination center between 2013 and 2018 and data from 221 PEIs associated with 35 physical conditions, with the majority of unhealthy physical states being due to chronic diseases.

## Results

### Study population

We included 811,244 individuals who attended the Health Management Center and Physical Examination Center between 2013 and 2019. These subjects were enrolled in Sichuan Province, with most of them from Chengdu City. The enrolled subjects accounted for about 1% of the demographics of Sichuan Province and 5% of the demographics of Chengdu City. The participants represented 35 healthy states based on either a healthy status or the presence of an underlying disease condition (unhealthy status). Specifically, the study population included 711,928 healthy participants, 46,981 patients with hypertension, 11,745 patients with diabetes, and 32,960 patients with other unhealthy statuses (mainly chronic diseases) (Table 1). Moreover, 7630 samples with 12 diseases in replication for prediction were also enrolled in 2019 as a separate dataset. We included 221 PEIs, which comprised patient demographic information (age and sex) and lifestyle indicators (alcohol consumption, tobacco use, etc.), in our analysis.

**Table 1:** Summary of the study sample’s detected correlations and the different PEIs between a healthy status and an unhealthy status.

Body status	Sample (N)	Age (Range)	Female %	Sig. correlation (a)	Sig. correlation % (b)	Different PEIs (c)
Normal condition	711,928	41.4 (4–105)	45.7	7662	31.5	–
Cholecystolithiasis	993	50.09 (19–97)	47.8	1622	6.9	28
Hypertension	46,981	62.0 (20–102)	36.1	4413	18.3	112
Hypertension + diabetes	8586	67.3 (34–99)	67.1	3008	12.6	100
Hypertension + coronary	2074	74.0 (34–98)	40.4	1920	7.7	53
Hypertensive + diabetes +coronary	928	73.5 (37–98)	35	2014	8.7	56
Hyperlipidemia	1722	65.0 (29–96)	33	2256	9.5	51
Coronary heart disease	1325	68.7 (27–93)	24.6	1925	8.3	36
Coronary + diabetes	280	70 (44–89)	20.4	1335	6.2	40
Rhinallergosis	156	39 (18–92)	39.7	1278	6.1	12
Hypothyroidism	1661	46.7 (16–94)	84.6	1703	7.1	33
Hyperthyroidism	767	45.2 (15–89)	66.6	1234	5.2	16
Cervical spondylopathy	318	53.7 (27–87)	48.7	1589	7	14
Rheumatoid arthritis	396	55.0 (24–86)	74.7	1039	4.5	29
Chronic rhinitis	320	38.6 (21–85)	30.6	969	4.5	8
Nephropathy	76	48.6 (23–91)	42	1771	7.8	31
Diabetes	11,745	59.3 (14–96)	24.9	2972	12.3	91
Gout	2138	51.8 (20–97)	2	1813	7.8	52
Parkinson's syndrome	197	70.4 (40–92)	26.4	1659	8.1	28
Stomach trouble	1296	49.5 (19–95)	39.2	1523	6.5	37
Chronic pharyngitis	782	40.2 (18–90)	32.7	1487	6.2	14
Lumbar disc protrusion	385	56.8 (25–91)	35.6	982	4.3	12
Hepatitis B	706	45.3 (22–82)	22.8	1084	4.5	34
Hypertension + other diseases	2409	64.6 (28–94)	38.1	1750	7.1	67
Coronary + others	101	69.3 (36–92)	33.7	569	3.3	12
Diabetes + others	373	63 (29–90)	26.8	1045	4.6	35
Bronchial disease	574	60.7 (19–95)	33.2	1276	5.6	19
Other disease conditions	2777	51.4 (17–100)	43.8	2066	8.5	38
Brain diseases	257	69.9 (27–98)	29.5	1023	4.7	25
Hepatic adipose infiltration	274	44.1 (21–77)	12.4	1139	5.3	36
Asthma	280	51.0 (12–91)	57.9	1388	5.9	23
Other cardiac diseases	344	60.0 (26–90)	55.9	749	3.6	19
Heart disease	229	69.7 (26–96)	42.6	698	3.5	28
Hepatopathy	180	51.6 (25–83)	38.7	702	3.6	25
Pregnant	56	29.7 (24–36)	100	1015	8.5	25

(a) Significant correlations, which are the number of correlations with *P* values calculated by PCC adjusted by all the correlations (*P* < 0.05/24,322 PEI pairs = 2 × 10^–6^).

(b) Significant correlation %, which is the percentage of significant correlations in all the correlation pairs.

Detailed information on the correlations described in (a–b) is provided in Tables S1–35.

(c) The number of PEIs was significantly different between a normal physical status and a non-normal physical status (*P* < 0.05/34 non-normal status = 1.4 × 10^–3^). A linear regression model was used to compare the PEIs between a normal physical status and a non-normal physical status, adjusted for gender and age. Detailed information on this summary is provided in Table S36.

### PEI correlations in participants with healthy physical status

**Figure 1:**
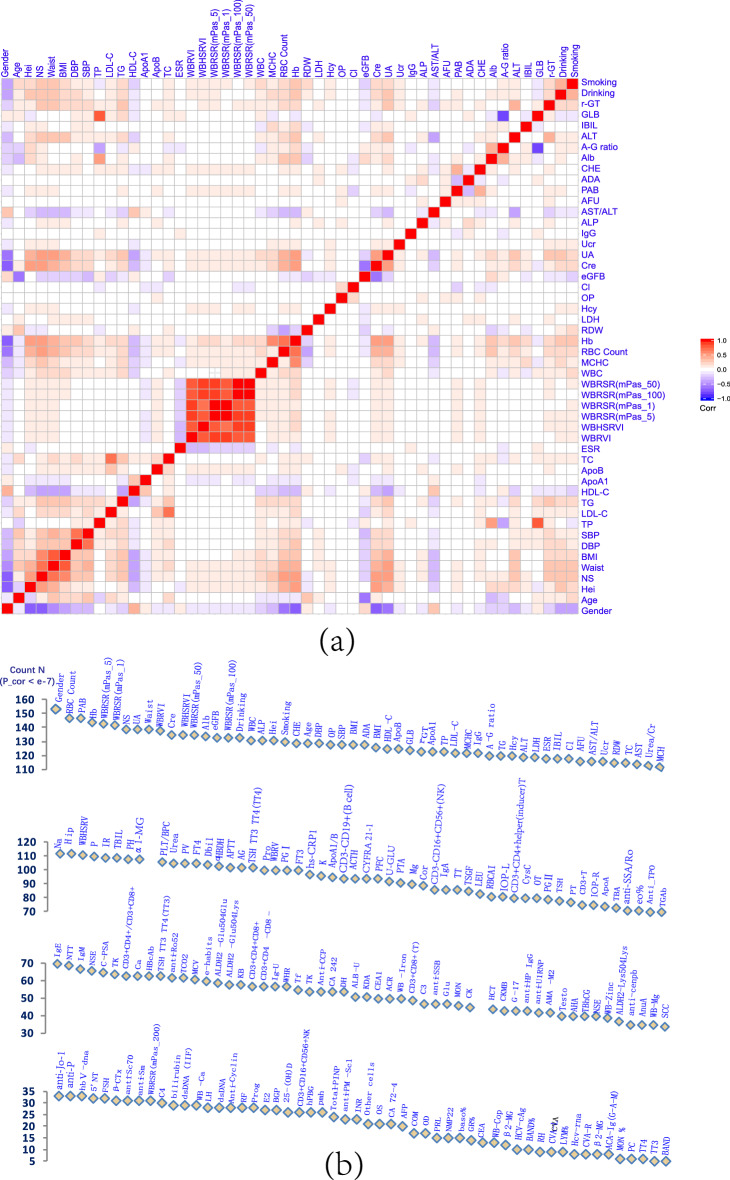
PEI correlations detected in the healthy cohort. (**a**) A correlation map of the top 50 correlated PEIs, with each having > 114 significant correlations with other PEIs (FDR < 0.05). (**b**) The number of statistically significant correlations detected in the healthy population for each PEI.

We explored the PEI correlations in participants with a healthy physical status to provide a background. Among 221 PEIs, 7662 significant correlations (*P* < 0.05/24,322 PEI pairs = 2 × 10^−6^) were found in 24,322 PEI pair correlations (31.5%) (Table 1, Table S1) in those with a healthy physical status (*N* = 711,928, mean age 41.4, female = 45.7%). This finding suggests a wide range of correlations between PEIs (Fig. 1). The top 50 correlated PEIs included sex, age, red blood cell count, prealbumin, history of alcohol intake (alcohol consumption and drinking), alkaline phosphatase level, and tobacco use (smoking), among others (Fig. 1a). Among the 221 PEIs, the number of significantly correlated PEIs also suggested rich correlations between PEIs (Fig. 1b). Some of the identified correlations between PEIs in a healthy status were consistent with the reported literature, but most of them were newly discovered in this study.

**Figure 2:**
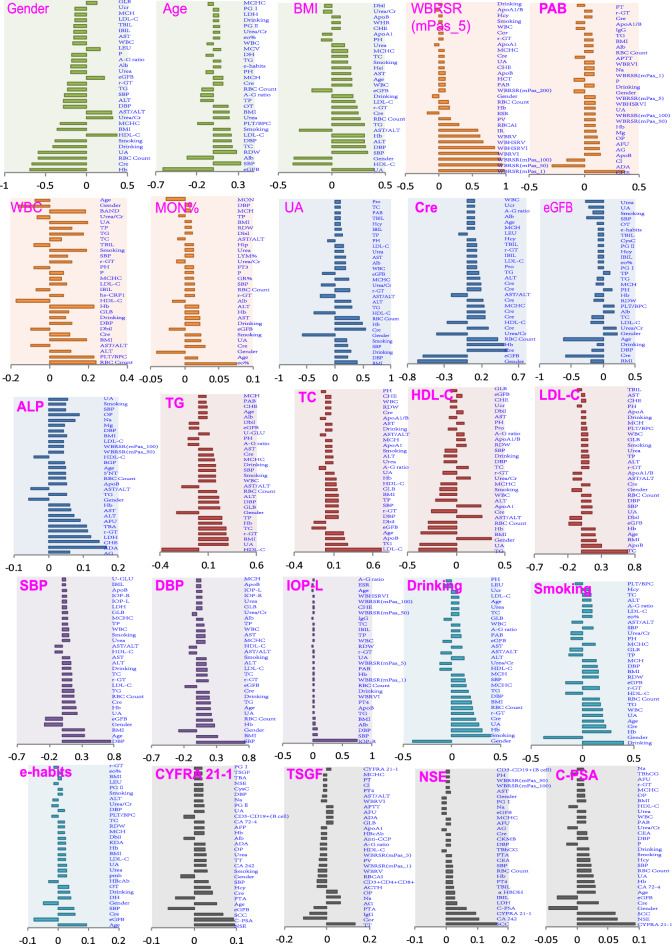
Correlation directions of typical PEIs in healthy physical conditions. The r values were calculated using the PCC method. See Table 1 for detailed information on the PEIs.

General inspection PEIs showed rich relevance to each other or to other PEIs. For example, sex showed the richest PEI correlations (151 PEI pairs, males vs. females), including hemoglobin, creatinine, uric acid, drinking, smoking, and body mass index (BMI), which reflect the differences in body shape, physique, and living habits between males and females (Figs. 1 and 2, Table S1). Age also showed strong PEI correlations (125 PEI pairs), such as estimated glomerular filtration rate (eGFB), systolic pressure (SBP), diastolic pressure (DBP), albumin (Alb), and low-density lipoprotein (LDL-C). These findings suggest that body functions systematically change with increasing age (Figs. 1 and 2, Table S1). We also found 124 PEI correlations with BMI, which reflects a strong influence of body shape on PEIs, including uric acid, high-density lipoprotein (HDL-C), systolic pressure, and diastolic pressure (Figs. 1 and 2, Table S1). Blood pressure (BP) has many physiological meanings. We identified a set of PEIs that correlated with BP, including 125 PEIs for diastolic pressure and 124 PEIs for systolic pressure (Figs. 1 and 2, Table S1). Intraocular pressure (IOP) is an important factor in the diagnosis of glaucoma^12^. We found 79 PEIs that were weakly correlated with intraocular pressure of the left eye (IOP-L), including intraocular pressure of the right eye (IOP-R), systolic pressure, diastolic pressure, albumin, BMI, triglycerides (TG), ApoB, drinking, and total cholesterol (TC). Similar to IOP-L, 73 PEIs were weakly correlated with IOP-R (Figs. 1 and 2, Table S1).

**Figure 3:**
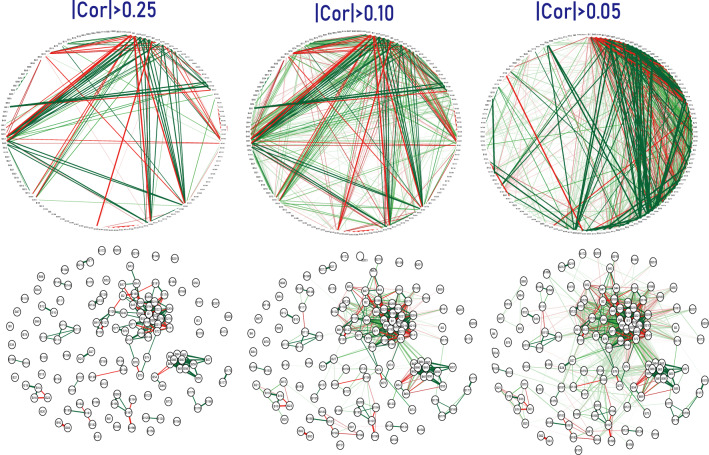
PEI networks in a healthy physical status. In the weighted graphs, the green edges indicate positive weights, and the red edges indicate negative weights. The color saturation and width of the edges correspond to the absolute weight and scale relative to the strongest weight in the graph, respectively. The circular layout shows how well the data conform to the model, while the force-oriented layout shows how each node (connected and unconnected) repulses the other and how connected nodes attract each other.

As expected, blood lipid PEIs showed many correlations. For example, 119 PEIs were correlated with triglyceride (Figs. 1 and 3, Table S1), and 122 PEIs were correlated with high-density lipoprotein, with many negative correlations, including triglyceride, uric acid, and BMI (Figs. 1 and 2, Table S2). The correlation patterns between low-density lipoprotein and high-density lipoprotein showed a specific opposite trend (Figs. 1 and 2, Table S1). Clearly, health habits have a profound impact on physical health. We detected 130 PEIs that were correlated with drinking, such as sex, smoking, hemoglobin, and uric acid (Figs. 1 and 2, Table S1), and 128 PEIs were correlated with smoking, including drinking, sex, and age (Figs. 1 and 2, Table S1). We also detected 58 PEIs that weakly correlated with exercise habits (e-habits), including age, estimated glomerular filtration rate, and systolic pressure (Figs. 1 and 2, Table S1). Tumor marker expression can indicate the occurrence and development of tumors. We detected weak correlations between several tumor markers and PEIs. For example, 88 PEIs were correlated with the cytokeratin-19-fragment CYFRA21-1 (CYFRA 21-1); 83 PEIs were correlated with tumor-supplied group factors; 64 PEIs were correlated with neuron-specific enolase; and 64 PEIs were correlated with complex prostate-specific antigen (C-PSA) (Figs. 1 and 2, Table S1).

### PEI correlations in individuals with unhealthy physical status

We then examined the PEI correlations and identified rich correlations in 34 unhealthy physical statuses (Table 1). Unlike in the healthy physical status, we found fewer significant correlations in PEIs in subjects with an unhealthy physical status. This could have been caused by the sample size effect (Table 1, Tables S2–S35). Each unhealthy physical state had its own correlation spectrum, and most of them were newly discovered in this study. For example, in the hypertension population, we found 4413 significant correlations in the 221 PEIs of 24,322 PEI pairs (18.3%) (Table S2). The PEIs with increased correlations included monocytes (MON) (70 in hypertension vs. 6 in the healthy physical state; the same as below), quantitative detection of hepatitis B virus DNA (HBV-DNA) (76 vs. 33), and quantitative detection of hepatitis C virus RNA (HCV-RNA) (49 vs. 8) (Table S2). Subjects with both hypertension and coronary heart disease (hypertension + coronary) had an increased correlation with the RH blood group compared to the healthy cohort (41 vs. 9 in normal). Conversely, the number of correlations in homocysteine (Hcy) greatly decreased in unhealthy versus healthy patients (2 vs. 120). In diabetes, 10 PEI pairs increased, while the remaining 195 PEI pairs decreased. The increased PEIs included monocytes (41 vs. 6), hepatitis C virus RNA (42 vs. 8), anti-Sc70 (59 vs. 31), and hepatitis C core antigen (HCV-cAg) (35 vs. 10) (Table S17). These results suggest that under unhealthy status, PEIs changed systematically. Each disease had its own specific PEI spectrum.

**Figure 4:**
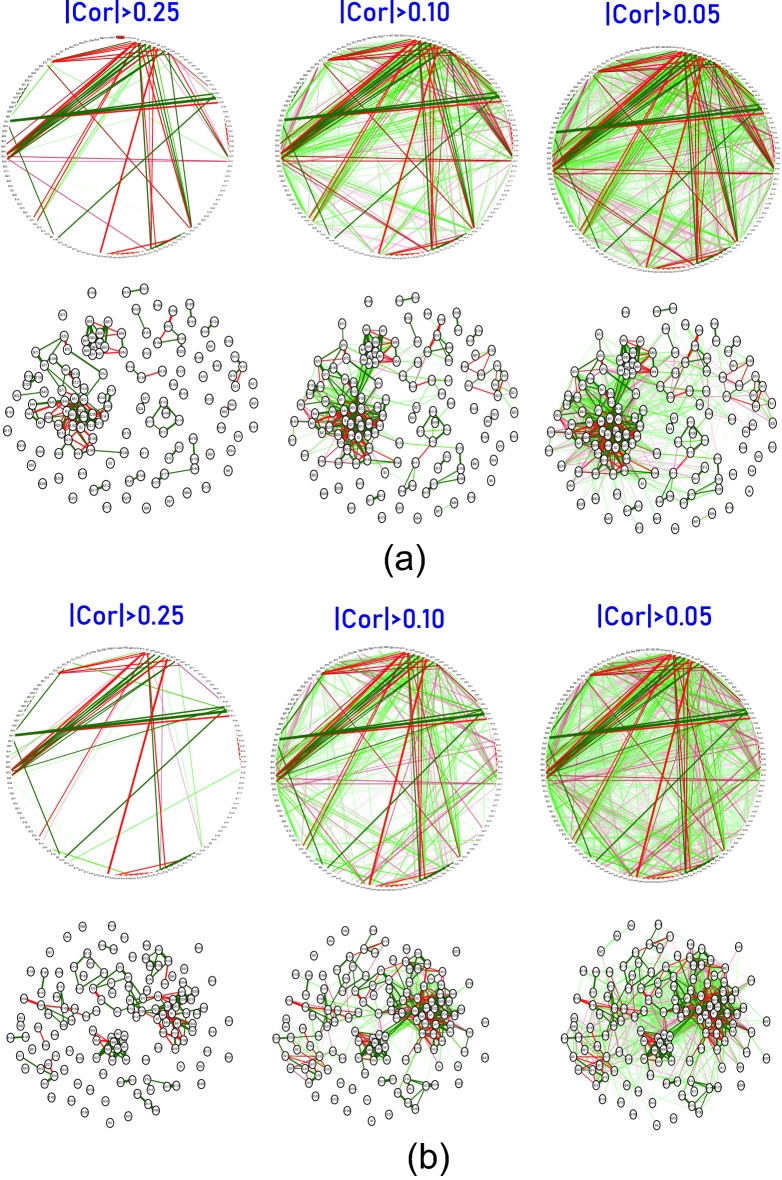
PEI networks in hypertension (**a**) and diabetes (**b**). In the weighted graphs, the green edges indicate positive weights, and the red edges indicate negative weights. The color saturation and width of the edges correspond to the absolute weight and scale relative to the strongest weight in the graph, respectively. At a minimum, the edge with an absolute weight at this value is omitted. The circular layout is convenient for seeing how well the data conform to a model, but another layout is more appropriate for showing how the data are clustered. A force-oriented layout was created by specifying layout = “spring.”. In principle, through this function, each node (connected and unconnected) repulses the other, and the connected nodes also attract each other.

We explored the correlation networks between the PEIs using a qgraph^13^, which shows the LinkMode among the PEIs. In a healthy state, the PEIs showed rich interactions in both positive and negative directions (Fig. 3). In unhealthy physical states, each state showed its unique interaction networks with the PEIs. Figure 4 illustrates the network of hypertension and diabetes. These results show a dependency relationship between multiple indicators in each physical state, which can be used in combination in the assessment of physical health.

### Candidate PEI markers for unhealthy physical status

**Figure 5:**
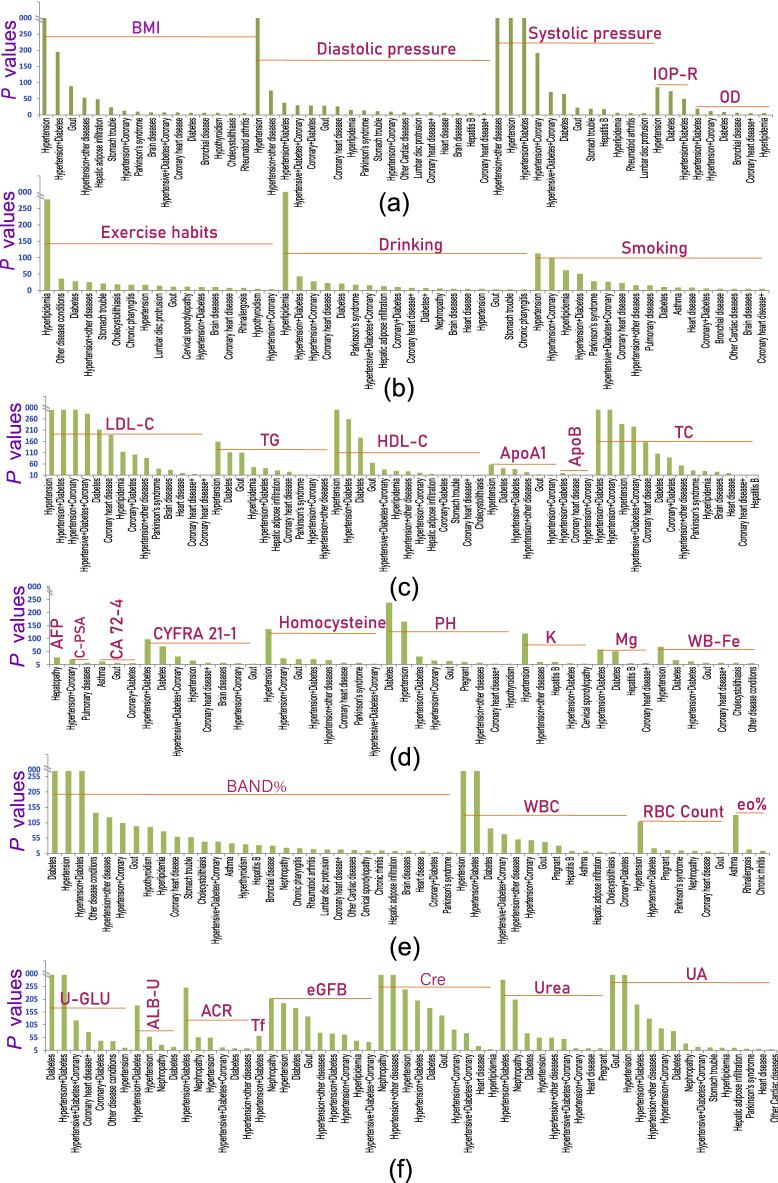
Representative candidate markers for an unhealthy physical status. A linear regression model was used to compare PEIs between healthy and unhealthy physical statuses after adjusting for sex and age. Significantly different PEIs (*P* < 0.05) after a Bonferroni adjustment (*P* < 0.05/34 unhealthy states = 1.4 × 10^−3^) are shown. See also Table S36.

To discover and verify new candidate biomarkers or the effects of living habits on the early diagnosis of diseases, we calculated the differences in each of the 221 PEIs between healthy and unhealthy physical states. In total, we found 1239 significantly different PEI pairs in the healthy status and 34 unhealthy physical statuses (*P* < 0.05/34 = 0.0014, adjusted for 34 unhealthy physical statuses) (Table 1, Fig. 5, Table S36). For example, 112 PEIs were significantly different between patients with hypertension and healthy individuals, 100 PEIs were different between hypertension + diabetes and healthy individuals, and 91 PEIs were different between diabetes and healthy individuals. Some of the results were consistent with previous findings, and some were newly discovered.

**Figure 6:**
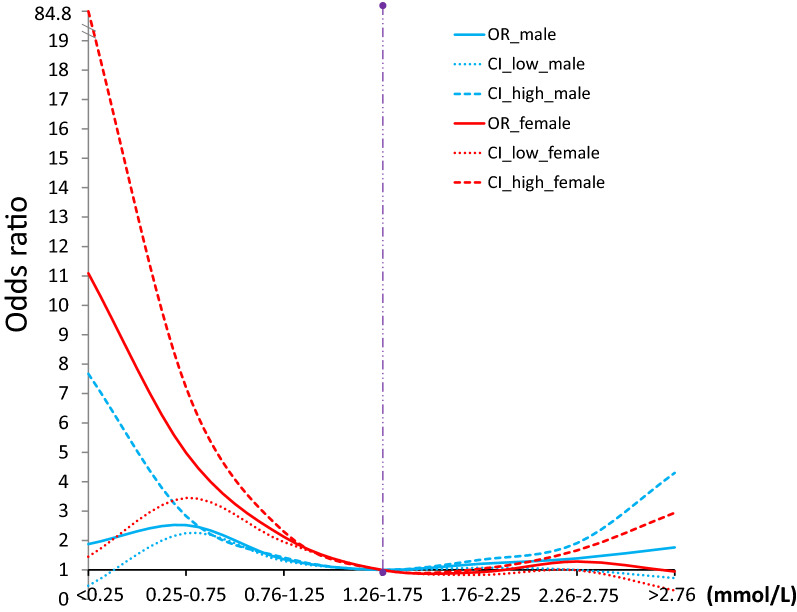
Odds ratios for the HDL-C concentration in plasma from subjects with normal physical status and subjects with diabetes. Both male and female subjects were included in this study.

For most of the 221 PEIs, we detected a difference between healthy and unhealthy physical statuses, especially in the PEIs involved in physique, lifestyle, and blood lipids (Fig. 5, Table S36). For example, for BMI, we found differences between healthy and unhealthy physical statuses in 16 of the 34 unhealthy physical statuses, including in patients with hypertension (*P* = 0) and gout (*P* = 6.48 × 10^−90^). Exercise habits (E-habits) showed 19 differences between healthy and unhealthy statuses, including hyperlipidemia (*P* = 1.28 × 10^−277^) and diabetes (*P* = 4.20 × 10^−29^). Dietary habits also showed differences in 10 unhealthy statuses, including chronic pharyngitis (*P* = 2.59 × 10^−19^) and cholecystolithiasis (*P* = 9.43 × 10^−18^). We detected differences in alcohol intake habits in 20 unhealthy statuses, including hyperlipidemia (*P* = 0), coronary heart disease (*P* = 4.06 × 10^−24^), diabetes (*P* = 1.09 × 10^−22^), and Parkinson’s syndrome (*P* = 1.43 × 10^−17^). We also observed differences in smoking habits in 18 unhealthy statuses compared to the healthy condition, including hypertension (*P* = 2.74 × 10^−114^), hyperlipidemia (*P* = 2.69 × 10^−62^), and Parkinson’s syndrome (*P* = 5.12 × 10^−29^). We found differences for intraocular pressure of the right eye in five unhealthy statuses compared to the healthy status, including hypertension (*P* = 3.63 × 10^−85^) and diabetes (*P* = 2.01 × 10^−73^); similar findings were found for intraocular pressure of the left eye (Fig. 5, Table S36). For the lipid PEIs, we observed differences between 34 unhealthy and healthy statuses. For example, low-density lipoprotein was detected in 21 unhealthy statuses, including hypertension (*P* = 0) and diabetes (*P* = 2.95 × 10^−212^). High-density lipoprotein was detected in 17 unhealthy statuses, including diabetes (*P* = 1.92 × 10^−177^) (Fig. 5, Table S36). We conducted a detailed analysis of high-density lipoprotein and diabetes and found that those with low high-density lipoprotein level showed a significantly higher risk of developing diabetes than those with average values (1.26–1.75 mmol/L) in this population. Notably, those with high-density lipoprotein levels also showed an elevated risk of developing diabetes (Fig. 6).

Tumor-associated antigens showed significant differences between healthy and unhealthy statuses. For example, cytokeratin-19-fragment CYFRA21-1 was detected in 10 unhealthy statuses, including hypertension + diabetes (*P* = 3.71 × 10^−97)^ and diabetes (*P* = 4.52 × 10^−70^). Carcinoembryonic antigen 1 (CEA1) was detected in 12 unhealthy statuses, including hypertension + coronary (*P* = 9.59 × 10^−29^) and diabetes (*P* = 1.73 × 10^−18^). Alpha-fetoprotein (AFP) was detected in hepatopathy (*P* = 1.08 × 10^−28^), and complex prostate-specific antigen was found in hypertension + coronary (*P* = 8.38 × 10^−20^). The carbohydrate antigen CA724 (CA 72-4) was detected in asthma (*P* = 9.92 × 10^−13^), gout (*P* = 3.53 × 10^−7^), and coronary + diabetes (*P* = 4.06 × 10^−5^) (Fig. 5, Table S36). We found significant differences between healthy and unhealthy statuses in other PEIs. For example, differences were found in urine sugar levels (U-GLU) in nine unhealthy statuses, including diabetes and its associated diseases. The eosinophil rate (eo%) was found in five unhealthy statuses, including asthma (*P* = 1.38 × 10^−129^) and rhinallergosis (*P* = 4.05 × 10^−18^). Whole blood iron levels (WB-Fe) were found in 11 unhealthy statuses, including hypertension (*P* = 2.52 × 10^−69^). We detected PH in 11 unhealthy status, including diabetes (*P* = 1.97 × 10^−239^), hypertension (*P* = 2.41 × 10^−166^), hypertension + diabetes (*P* = 9.90 × 10^−32^), and gout (*P* = 9.82 × 10^−15^). We found potassium (K+) in five unhealthy statuses, including hypertension (*P* = 1.98 × 10^−119^) and hepatitis B (*P* = 3.13 × 10^−10^). We detected differences in magnesium (Mg2+) in hypertension + diabetes (*P* = 3.14 × 10^−58^) and diabetes (*P* = 5.10 × 10^−52^). Hcy, which is an indicator of cardiovascular disease, was detected in eight unhealthy statuses, including hypertension (*P* = 1.97 × 10^−136^) and Parkinson’s syndrome (*P* = 1.76 × 10^−7^) (Fig. 5, Table S36). These results provide a set of candidate markers for the early diagnosis of chronic diseases.

### ML to predict healthy and unhealthy physical statuses from PEIs

A key objective of this study was to apply PEI data and machine learning technology to develop algorithms that could predict a common disease based on a general physical examination. We used three machine learning models: kernelized support vector machine (SVM), multilayer perceptron (MLP), and random forest (RF). The MLP prediction models only resulted in a low f1_score, recall, and precision in our initial training data. It took 10 h for the support vector machine model to perform binary classification; thus, we excluded the MLP and support vector machine prediction models for further training. We found that RF was more suitable for our data. Binary classification only took 2–3 min, and the prediction effect of RF was much better than that of MLP and support vector machine. However, RF could not give good performance in the multi-class classification of all physical statuses. Finally, we used binary classification to classify each pair of healthy and unhealthy physical statuses (e.g., hypertension and healthy people; Parkinson’s syndrome and healthy people), and we obtained relatively better performance than multi-class classification. We then optimized this prediction algorithm. As the data were characterized using serious category imbalance, a random under-sampling method was adopted that balanced the data by randomly selecting the data subset of the target class. For each physical status, the top 15% or 16% representative PEIs were extracted for prediction through feature extraction. The advantage of this method is that it is usually fast and completely independent of the model applied after feature selection.

**Figure 7:**
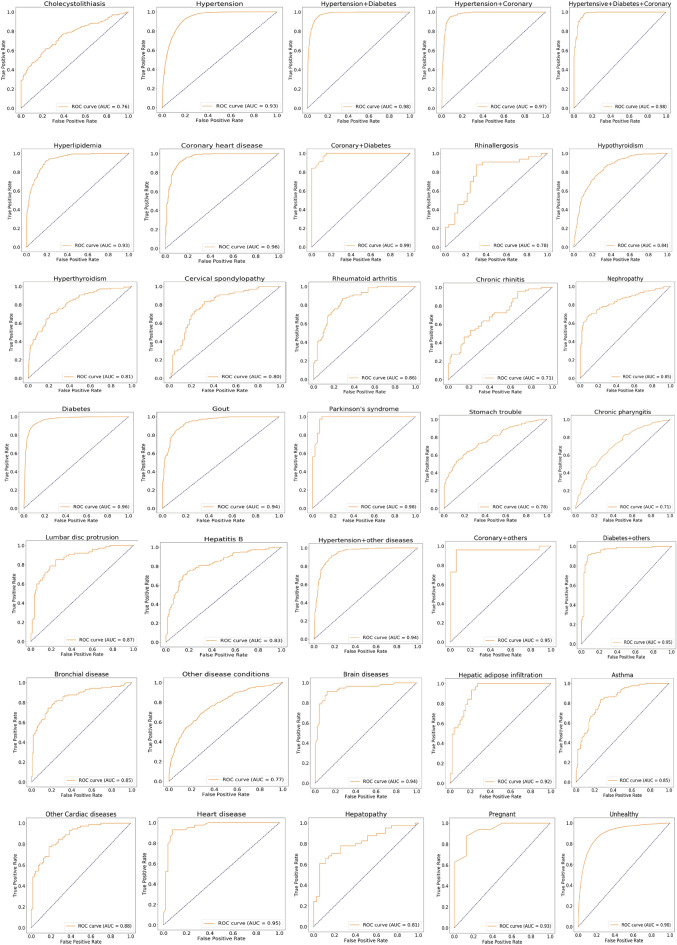
Machine learning prediction of 35 physical statuses using a random forest algorithm. The receiver operating characteristic (ROC) curve takes the false-positive rate as the horizontal axis and the true-positive rate as the vertical axis. The horizontal axis represents the proportion of the actual negative instances in the positive class predicted by the classifier to all negative instances. The vertical axis represents the proportion of the actual positive instances in the positive class predicted by the classifier to all positive instances. The AUC is the area under the ROC curve.

Finally, in the RF algorithm prediction of each pair of healthy and unhealthy physical statuses, the area under the curve (AUC) of the receiver operating characteristic (ROC) curve reached 66–99%, depending on the unhealthy physical status (average 87.6%) (Fig. 7, Table 2, and Tables S37 and S38). In the classification, AUC values of more than 90% indicated excellent performance, and values of 80–90% indicated good performance. Our algorithm provided high-precision predictions in 18 of the 34 unhealthy physical statuses (AUC > 90%) and good performance in nine unhealthy physical statuses (90% > AUC > 80%). In our algorithm, patients with heart-related diseases showed excellent performance. For example, by extracting 30 PEI features (i.e., age, leukocyte count, monocytes, Mon%, mean corpuscular volume, red blood cell count, red cell distribution width, lymphocyte rate, platelet count, low-density lipoprotein, high-density lipoprotein, total cholesterol, carcinoembryonic antigen 1, albumin, albumin–globulin, cystatin c, glucose, urine sugar, urine creatinine, estimated glomerular filtration rate, creatinine, urea, waistline, waist–hip ratio, BMI, operation history, systolic pressure, height, neck size, and anamnesis), hypertensive + diabetes + coronary heart disease provided 99% AUC by just using 909 training samples and 387 validation samples [f1-score (95%CI) 0.96 (0.95–0.96); accuracy (95%CI) 0.95 (0.94–0.97); specificity (95%CI) 0.95 (0.94–0.95); recall (sensitivity) (95%CI) 0.95 (0.94–0.97)]. In our algorithm, patients with Parkinson’s syndrome showed 97% AUC using 192 training samples and 83 validation samples [f1-score (95%CI) 0.91 (0.90–0.91); accuracy (95%CI) 0.90 (0.89–0.90); specificity (95%CI) 0.87 (0.79–0.94); recall (95%CI) 0.90 (0.89–0.91)]. For hepatic adipose infiltration, our algorithm also showed good prediction performance using 803 training samples and 115 validation samples [f1-score (95%CI) 0.82 (0.78–0.87); accuracy (95%CI) 0.81 (0.76–0.86); specificity (95%CI) 0.75 (0.67–0.82); recall (95%CI) 0.82 (0.77–0.87); AUC (95%CI) 0.92 (0.89–0.94)]. For chronic rhinitis, we obtained the lowest prediction performance in this study [AUC (95%CI) 0.66 (0.60–0.72)]. When all the unhealthy physical statuses were classified as one “unhealthy” status together, our algorithm also provided good predictions: f1-score (95%CI) 0.83 (0.83–0.83); accuracy (95%CI) 0.82 (0.82–0.82); specificity (95%CI) 0.81 (0.81–0.81); sensitivity (95%CI) 0.84 (0.84–0.84); AUC (95%CI) 0.9 (0.90–0.90). These results suggest that by performing feature extraction on the PEIs (15–16% of all 221 PEIs) using only a small number of samples, the proposed RF algorithm provided good performance for the majority of the unhealthy physical status predictions.

**Table 2:** Predictive validity of models.

	Training set (sample size)	Validation set (sample size)	F1-score (95%CI)	Specificity (95%CI)	Recall (sensitivity) (95%CI)	ROC (AUC) (95%CI)
Cholecystolithiasis	963	413	0.69 (0.66–0.71)	0.73 (0.70–0.76)	0.70 (0.67–0.72)	0.77 (0.75–0.79)
Hypertension	46,171	17,987	0.86 (0.86–0.86)	0.82 (0.82–0.82)	0.85 (0.85–0.86)	0.92 (0.92–0.93)
Hypertension + diabetes	8414	3065	0.92 (0.92–0.92)	0.90 (0.90–0.90)	0.92 (0.92–0.92)	0.97 (0.97–0.98)
Hypertension + coronary heart disease	2032	871	0.93 (0.92–0.93)	0.90 (0.90–0.91)	0.93 (0.92–0.93)	0.97 (0.97–0.98)
Hypertensive + diabetes + coronary heart disease	909	389	0.96 (0.95–0.96)	0.95 (0.94–0.95)	0.95 (0.94–0.97)	0.99 (0.98–0.99)
Hyperlipidemia	1687	722	0.86 (0.85–0.86)	0.82 (0.81–0.83)	0.85 (0.84–0.86)	0.94 (0.93–0.94)
Coronary heart disease	1298	557	0.90 (0.89–0.92)	0.87 (0.84–0.89)	0.90 (0.88–0.91)	0.96 (0.96–0.97)
Coronary heart disease + diabetes	274	118	0.93 (0.93–0.94)	0.91 (0.88–0.95)	0.94 (0.92–0.95)	0.98 (0.97–1.00)
Rhinallergosis	152	66	0.71 (0.64–0.79)	0.80 (0.73–0.86)	0.70 (0.64–0.76)	0.79 (0.73–0.84)
Hypothyroidism	1627	698	0.77 (0.76–0.78)	0.73 (0.71–0.74)	0.76 (0.75–0.76)	0.84 (0.83–0.86)
Hyperthyroidism	751	322	0.72 (0.71–0.72)	0.73 (0.71–0.75)	0.73 (0.71–0.74)	0.79 (0.77–0.81)
Cervical spondylopathy	311	134	0.71 (0.66–0.75)	0.73 (0.64–0.81)	0.71 (0.69–0.73)	0.78 (0.78–0.80)
Rheumatoid arthritis	387	167	0.79 (0.78–0.81)	0.76 (0.71–0.81)	0.78 (0.77–0.79)	0.86 (0.83–0.89)
Chronic rhinitis	313	135	0.61 (0.58–0.64)	0.57 (0.56–0.57)	0.61 (0.58–0.63)	0.66 (0.60–0.72)
Nephropathy	564	242	0.73 (0.72–0.74)	0.81 (0.79–0.84)	0.76 (0.75–0.78)	0.84 (0.82–0.85)
Diabetes	11,545	4949	0.90 (0.90–0.90)	0.90 (0.89–0.90)	0.90 (0.90–0.90)	0.96 (0.96–0.96)
Gout	2095	898	0.88 (0.88–0.88)	0.85 (0.84–0.87)	0.86 (0.83–0.88)	0.94 (0.94–0.94)
Parkinson's syndrome	192	83	0.91 (0.90–0.91)	0.87 (0.79–0.94)	0.90 (0.89–0.91)	0.97 (0.95–0.98)
Stomach trouble	1269	545	0.68 (0.68–0.69)	0.71 (0.70–0.73)	0.70 (0.70–0.70)	0.77 (0.75–0.78)
Chronic pharyngitis	765	329	0.63 (0.62–0.65)	0.66 (0.65–0.66)	0.67 (0.65–0.68)	0.72 (0.69–0.75)
Lumbar disc protrusion	377	162	0.77 (0.72–0.81)	0.70 (0.63–0.77)	0.75 (0.70–0.79)	0.85 (0.82–0.88)
Hepatitis B	691	297	0.73 (0.70–0.77)	0.79 (0.77–0.80)	0.75 (0.72–0.77)	0.83 (0.81–0.85)
Hypertension + other diseases	2360	1012	0.86 (0.85–0.88)	0.82 (0.81–0.83)	0.86 (0.85–0.86)	0.93 (0.93–0.94)
Coronary heart disease + other diseases	98	43	0.88 (0.84–0.92)	0.83 (0.76–0.90)	0.86 (0.83–0.88)	0.94 (0.91–0.97)
Diabetes + other diseases	365	157	0.90 (0.87–0.94)	0.89 (0.84–0.94)	0.90 (0.86–0.94)	0.96 (0.93–0.98)
Bronchial disease	562	241	0.76 (0.70–0.83)	0.80 (0.76–0.84)	0.77 (070–0.83)	0.83 (0.79–0.88)
Other disease conditions	2720	1167	0.68 (0.67–0.70)	0.69 (0.66–0.73)	0.69 (0.67–0.71)	0.75 (0.74–0.77)
Brain diseases	251	108	0.86 (0.81–0.90)	0.83 (0.75–0.90)	0.87 (0.82–0.91)	0.93 (0.91–0.95)
Hepatic adipose infiltration	803	115	0.82 (0.78–0.87)	0.75 (0.67–0.82)	0.82 (0.77–0.87)	0.92 (0.89–0.94)
Asthma	1640	803	0.75 (0.74–0.76)	0.77 (0.69–0.84)	0.75 (0.74–0.76)	0.88 (0.84–0.92)
Other cardiac diseases	336	145	0.79 (0.78–0.81)	0.80 (0.75–0.86)	0.78 (0.76–0.80)	0.88 (0.84–0.92)
Heart disease	224	96	0.89 (0.87–0.90)	0.91 (0.87–0.94)	0.89 (0.86–0.92)	0.94 (0.90–0.99)
Hepatopathy	176	76	0.71 (0.65–0.76)	0.78 (0.68–0.87)	0.73 (0.68–0.78)	0.80 (0.74–0.85)
Pregnant	54	24	0.83 (0.76–0.90)	0.85 (0.79–0.92)	0.82 (0.77–0.86)	0.91 (0.90–0.93)
Normal or non-normal condition	91,028	39,012	0.83 (0.83–0.83)	0.81 (0.81–0.81)	0.84 (0.84–0.84)	0.9 (0.90–0.90)

The number of training sets and valid set samples was obtained after under-sampling and data random splitting. Normal condition or disease was used to classify all types of diseases into disease states, followed by under-sampling with a sample of healthy people and data division.

*ROC* receiver operating characteristic, *AUC* area under the curve.

**Figure 8:**
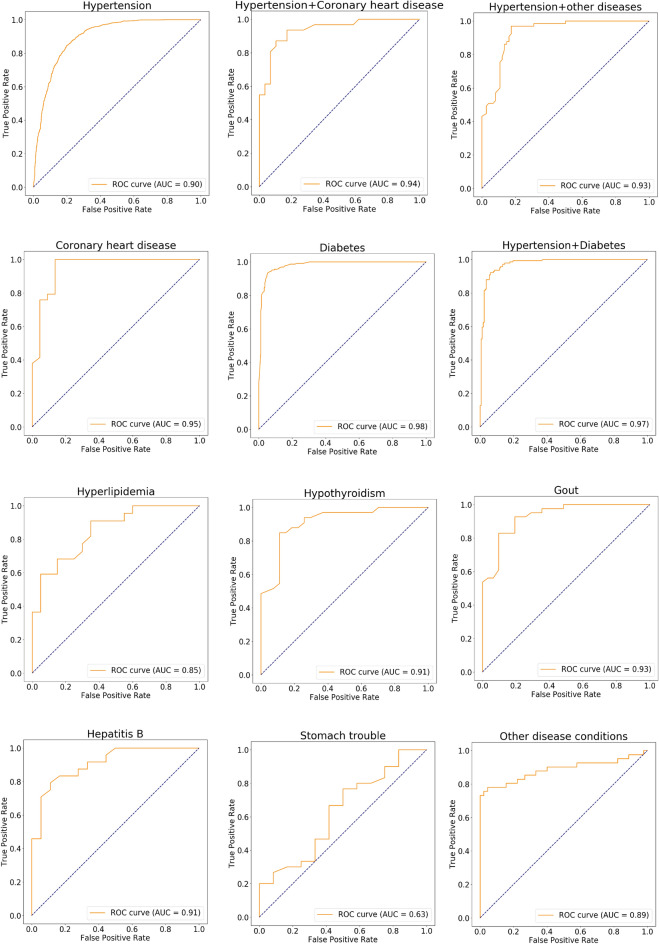
Machine learning replication of the prediction of the 12 physical statuses using the random forest algorithm. The receiver operating characteristic (ROC) curve takes the false-positive rate as the horizontal axis and the true-positive rate as the vertical axis. The horizontal axis represents the proportion of the actual negative instances in the positive class predicted by the classifier to all negative instances. The vertical axis represents the proportion of the actual positive instances in the positive class predicted by the classifier to all positive instances. The AUC is the area under the ROC curve.

To further validate our RF algorithm prediction model, we conducted a replication analysis of 12 diseases in a new dataset. The results are presented in Fig. 8 and Table 3. The ROC of the replication data was 0.63–0.98 (average 0.90) (Fig. 8), suggesting good performance of the prediction effect based on the limited samples. For the remaining diseases, we did not obtain sufficient samples in the new dataset for replication (< 100 samples).

**Table 3:** Predictive validity of models of replication.

	Training set (sample size)	Validation set (sample size)	F1-score (95%CI)	Specificity (95%CI)	Recall (sensitivity) (95%CI)	ROC (AUC) (95%CI)
Hypertension	4270	1813	0.83 (0.83–0.84)	0.88 (0.87–0.88)	0.77 (0.75–0.79)	0.89 (0.88–0.90)
Hypertension + diabetes	709	304	0.92 (0.91–0.94)	0.94 (0.93–0.96)	0.90 (0.89–0.92)	0.98 (0.97–0.99)
Hypertension + coronary heart disease	140	60	0.88 (0.86–0.92)	0.87 (0.81–0.92)	0.92 (0.88–0.94)	0.95 (0.93–0.97)
Hyperlipidemia	98	42	0.77 (0.76–0.79)	0.81 (0.78–0.86)	0.65 (0.56–0.74)	0.78 (0.69–0.85)
Coronary heart disease	117	51	0.88 (0.86–0.92)	0.92 (0.92–0.93)	0.81 (0.76–0.86)	0.92 (0.90–0.95)
Hypothyroidism	140	60	0.76 (0.75–0.86)	0.73 (0.66–0.82)	0.81 (0.74–0.89)	0.83 (0.73–0.91)
Diabetes	1110	476	0.91 (0.88–0.94)	0.92 (0.90–0.94)	0.89 (0.87–0.94)	0.96 (0.95–0.98)
Gout	168	72	0.82 (0.78–0.81)	0.90 (0.89–0.91)	0.71 (0.65–0.81)	0.89 (0.84–0.93)
Stomach trouble	98	42	0.56 (0.54–0.58)	0.43 (0.40–0.45)	0.77 (0.67–0.88)	0.63 (0.55–0.70)
Hepatitis B	98	42	0.79 (0.73–0.82)	0.83 (0.71–0.89)	0.79 (0.64–0.94)	0.85 (0.77–0.91)
Hypertension + other diseases	324	139	0.83 (0.80–0.85)	0.87 (0.83–0.93)	0.77 (0.70–0.85)	0.90 (0.87–0.93)
Other disease conditions	199	86	0.76 (0.70–0.82)	0.70 (0.63–0.78)	0.88 (0.85–0.89)	0.85 (0.82–0.89)

The number of training sets and valid set samples was obtained after under-sampling and data random splitting. Normal condition or disease was used to classify all types of diseases into disease states, followed by under-sampling with a sample of healthy people and data division. This replication is only carried out in diseases with more than 100 patients. *ROC* receiver operating characteristic, *AUC* area under the curve.

In this study, the top 15% or 16% representative PEIs were extracted for RF prediction using feature extraction in each physical status (Table S38), and 66–99% precision in predictions was achieved, depending on the physical state. In total, 161 PEIs were used for the RF prediction of 35 pairs of health statuses. Some PEIs were used more frequently than others, suggesting their important physiological value for the human body. The top 20 PEIs used included monocyte counts (36 health statuses used, the same as below, 100%), anamnesis (33, 92%), age (32, 98%), albumin (31, 86%), estimated glomerular filtration rate (30, 83%), systolic pressure (27, 75%), waistline (27, 75%), red cell distribution width (26, 72%), creatinine (23, 64%), neck size (23, 64%), operation history (23, 64%), red blood cell count (23, 64%), urea (22, 61%), waist–hip ratio (22, 61%), BMI (21, 61%), gender (21, 61%), height (20, 56%), glucose (19, 53%), hemoglobin (19, 53%), and platelet count (19, 53%). Some PEIs were rarely used, suggesting a unique indication of a certain disease. For example, sodium was selected only for cholecystolithiasis prediction, and cholinesterases were only selected for rhinallergosis prediction. Our results provided proof for predicting health conditions using only a set of PEIs.

## Discussion

This study produced correlation maps of 221 routine PEIs using physical examination data obtained from a Chinese population of 811,244 individuals with 35 healthy or unhealthy physical statuses (mainly chronic diseases). We detected a large number of correlations between PEIs in healthy or unhealthy physical states, and these correlations differed according to the 34 unhealthy physical conditions analyzed. Most of the correlations were newly observed in this study. We found a wide range of correlations among PEIs, such as sex, age, BMI, blood lipid, blood pressure, cancer-related indicators, and lifestyle, including drinking, smoking, and exercise habits. Improving our understanding of these PEI interactions can help explain disease mechanisms and pathogenesis. Our results fill the gap in systematic PEI analysis and provide rich information on how PEIs could reflect underlying health conditions, thus improving healthcare research and clinical practice.

One of the unexpected findings from our analysis was that patients with hypertension showed more correlations between hepatitis B virus DNA and hepatitis C virus RNA with other PEIs than a healthy cohort. We also found a strong correlation between the hepatitis C virus and other PEIs in diabetes, suggesting that patients infected with hepatitis C could be more susceptible to diabetes. This finding implies a phenomenon in which viral infections can make an individual more susceptible to developing chronic diseases. For these people, antiviral therapy may be taken into consideration when treating hypertension and diabetes.

Biomarker discovery and development for clinical research, diagnostics, and therapy monitoring in clinical trials are key areas in medicine and healthcare^14–17^. In this study, we presented many candidate markers for chronic diseases. For example, we found that intraocular pressure indicators, which are considered relatively independent markers for glaucoma^18–20^, are closely associated with hypertension, diabetes, and hypertension with diabetes. These results suggest that intraocular pressure may be affected to some extent by systemic diseases and may be used as a clinical marker for the early diagnosis of these diseases. Our results confirmed that a low high-density lipoprotein level is a risk factor for diabetes, especially in women^21^. This result suggests that improving high-density lipoprotein levels through dietary supplementation may be an effective way to prevent diabetes in patients with low high-density lipoprotein levels. However, based on our results, excessive high-density lipoprotein supplementation is also a risk factor; therefore, high-density lipoprotein supplementation should aim to bring high-density lipoprotein levels within the normal range^22^. We detected a significant increase in Alpha-fetoprotein in hepatopathy compared to a healthy cohort. This confirms that an increase in Alpha-fetoprotein is an increased risk factor for primary liver cancer in hepatopathy^23^. K^+^ has significant effects on hypertension^24^ and Cl^−^, and Mg^2+^ has significant effects on diabetes, suggesting that the modulation of these ions may affect these conditions. Living habits, such as exercise, smoking, and drinking, had a more profound effect on the body than we had expected. For example, exercise, drinking, or smoking history had a strong effect on hyperlipidemia^25^, as evidenced by a comparison to healthy status. This finding suggests that, by adjusting these living habits, hyperlipidemia should improve.

As the current physical examination conclusion is generally based on a relatively independent single or several previous indicators to advise on the results of physical examination, most of the results are ambiguous, and the value of judging the health status of the examinees is limited^26^. Therefore, there is an urgent need for a more accurate index system and method to assess the health status of physical examinees. In the final part of our study, we developed RF machine learning algorithms that could predict diseases in 15–16% of all 221 PEIs with good prediction performance (AUC: 66–99%; average 86%). For each disease, we defined about 30 contributed PEIs using feature extraction. In most of our prediction algorithms, only a few hundred samples were needed to give good prediction performance for many chronic diseases. This finding suggests that machine learning can be used on PEI data to help predict the true condition of the examinees, identify “at-risk” patients, and indicate the most relevant follow-up physical examinations for affected individuals. However, the operation of this method is not as convenient as that of multi-classification. We previously used multi-classification models, but we did not achieve good prediction results. When using multiple binary classification models, the total number of times to run the models is greater, but the time required to run a complete model is short, and the prediction effect using binary classification is better.

In this study, we used MLP, kernelized support vector machine, and RF for multi-label machine learning analysis. The MLP uses the multilayer perceptron in a neural network to predict. In using this model for the multi-classification of health and disease conditions, the final prediction results were all health conditions, with no disease conditions, suggesting poor prediction results. The support vector machine method took a long time to predict. In conducting multi-classification prediction using support vector machine, the F1 value of the cholecystolithiasis group reached the highest (0.70), while most of the F1 values of the other types of diseases were 0.00. In using the RF method for multi classification prediction, the F1 value of health type in the prediction results reached 0.80–0.90, while the F1 value of the other types was 0.00–0.40. These methods did not show good prediction performance for multi-classification prediction. Thus, we used two classification methods in this study. We analyzed the reasons for the poor results of the multi-classification prediction, and they could include the following aspects: (1) The sample balance between multiple types of data was not sufficient; (2) Feature selection was poor; (3) The feature dimension was not sufficient; (4) The feature weight was not good enough; (5) The learning ability of the selected algorithm was weak; and (6) The models were not fitted. To improve the multi-classification results in the future, the following aspects may be included: (1) Choose a better algorithm to predict. Compare multiple algorithms to improve the performance of the model, train them together, and find the one that performs best. For example, try to use recurrent neural networks^27^, labeled latent Dirichlet allocation^28^, and bidirectional encoder representations from transformers model^29^. (2) Tune model parameters. Super parameter tuning is a commonly used tuning method. The parameters that need to be selected before the learning process are called super parameters. For example, the number of trees in the RF affects the learning results. Using publicly available libraries, such as optuna, is recommended to help adjust the super parameters. It may be necessary to optimize one of the recall rates for accuracy. (3) Feature engineering. To create new features, transform the original data into features that better express the essence of the problem. Applying these features to the prediction model can improve the prediction accuracy of the invisible data model. (4) Improve the data sample imbalance between multi-class data and the classification inaccuracy caused by the serious inclination of large- and small-class data. Data enhancement can be used to balance the data distribution (upper and lower sampling, subclass sample data generation, etc.).

This study has some limitations. First, missing data are a common problem in all types of research. Overall, mean imputation may lead to inefficient analyses and commonly produces biased estimates of the association(s) investigated. Second, the data used in this study were taken from a single institution, which could introduce significant bias and limitations in machine learning generalization into real-world applications, especially with discrepancies in electronic medical records (EMRs) and patient populations. Third, some models had small datasets; for example, the Parkinson’s model included only about 250 patients. Small datasets may cause insufficient power for machine learning models. Fourth, a multi-classification model may support a stronger case for artificial intelligence and machine learning to be used as clinical tools to improve decision making compared to multiple binary classification models. However, we did not obtain good prediction results using multi-classification in this study. Fifth, the cross-sectional design of the study has some limitations: (1) Patients in the incubation or remission period are prone to misdiagnosis and bias. (2) Only the prevalence rate can be obtained, not the incidence rate. (3) Generally, it is only applicable to the study of chronic diseases. (4) Diseases and factors exist at the same time, and the exact causal relationship cannot be determined. (5) A group effect may occur in horizontal research. (6) Horizontal research is not applicable to the stability of research and development and the role of early effect.

In summary, we systematically explored the correlation between various PEIs and their relationship with chronic diseases and established machine learning prediction models to predict health status. This study provided abundant information to better understand the physiological and pathological characteristics of the human body as a system. More importantly, we identified modifiable factors and directions for disease prediction, diagnosis, and treatment. Our developed machine learning algorithms can be immediately applied to clinical practice to assist in the assessment of physical examination results.

## Methods

### Study approval

This work is a general retrospective study. The study was approved by the institutional ethics committee of Sichuan Provincial People’s Hospital (No. 2019276) according to the following rules: (1) ethical review of biomedical research involving human beings (Order of the State Health and Family Planning Commission of the People’s Republic of China, 2016), (2) World Medical Association Declaration of Helsinki (2013), and (3) The Council for International Organizations and Medical Sciences International Ethical Guidelines for Biomedical Research Involving Human Subjects. This study extracted only clinical PEI data; no patient identity was involved in this study. As the researchers applied for exemption from informed consent, they did their best to protect the information provided by the patients and prevent breaching of personal privacy.

### Study participants

PEI data were obtained from 811,244 Han Chinese patients visiting the Health Management Center and Physical Examination Center of Sichuan Provincial People’s Hospital between 2013 and 2018. The total cohort captured participants with 35 different reported health conditions, including 711,928 healthy participants and 91,686 unhealthy participants. The unhealthy cohort included 46,981 patients with hypertension, 11,745 with diabetes, and 32,960 with other unhealthy statuses (Table 1).

### Detected PEIs

Only the PEIs recorded by the same methods were included in this study. In total, 229 PEIs were initially collected. Eight PEIs detected in several individuals were excluded, leaving 221 PEIs for further analysis (Table 1). The PEIs included the levels of biochemical indicators and the results of the blood tests. Patients’ lifestyles and disease conditions were also investigated during the physical examination.

### Data processing

The PEIs with string variables were converted to integer variables for data analysis. Categorized variables were digitally coded for further calculation. The mean value imputation method was used for missing data (about 20% of all data). For individuals who participated in more than one physical examination, the average values of each PEI were used for the data analysis.

### Statistical analyses

The Pearson correlation coefficient (PCC) method was used to calculate the correlations between two PEIs (e.g., x and y) in R. This method measured the linear dependence between two variables. The PCC correlation (r) (1) and the *P* values (2) were calculated using the following formulae^30^:

1 $$r=\frac{\mathrm{n}\left(\sum \mathrm{xy}\right)-\left(\sum {\mathrm{x}}\right)(\sum \mathrm{y})}{\sqrt{{[\mathrm{n}\sum {\mathrm{x}}}^{2}-(\sum {{\mathrm{x}})}^{2}][\mathrm{n}\sum {\mathrm{y}}^{2}-(\sum \mathrm{y}{)}^{2}]}}$$

2 $$\begin{aligned} & P = 1 - {\text{F.DIST}}\left( {{{\left( {\left( {{\text{n}} - 2} \right)*{\text{r}}^{2} } \right)} \mathord{\left/ {\vphantom {{\left( {\left( {{\text{n}} - 2} \right)*{\text{r}}^{2} } \right)} {\left( {1 - {\text{r}}^{2} } \right),1,{\text{n}} - 2}}} \right. \kern-\nulldelimiterspace} {\left( {1 - {\text{r}}^{2} } \right),1,{\text{n}} - 2}}} \right) \\ & {\text{df}} = {\text{n}} - 2 \\ & {\text{n}} = {\text{number of x}} - {\text{y data pairs}} \\ \end{aligned}$$

The total sample size was required when the correlation coefficient (r) was used, when two-sided α = 0.05, β = 0.20. If r = 0.05, 3134 samples were required; if r = 0.10, 782 samples were required; if r = 0.25, 123 samples were required; and if r = 0.5, 29 samples were required. The general formula for the correlation sample calculation is as follows (3)^31^:

3 $$\begin{aligned} & r = {\text{expected correlation coefficient}} \\ & C = 0.5 \times \ln \left[ {({\text{l}} + {\text{r}})/\left( {{\text{l}}{-}{\text{r}}} \right)} \right] \\ & N = {\text{total number of subjects required}} \\ & {\text{Then}} \\ & N = [(Za + Zb) \div C]2 + 3. \\ \end{aligned}$$

A linear regression model was used to compare the PEIs between the reported healthy status and the unhealthy status, adjusted for sex and age using the R package^31^. The odds ratio of the high-density lipoprotein level was calculated using generalized linear models and adjusted for age using the R package^32^. A 5% confidence interval can be calculated using the model = fit_glm. The correlation interaction network was conducted using qgraph^33^.

### Machine learning (ML)

Three machine learning models, kernelized support vector machine^30,34^, MLP^30,35–37^, and RF^38^, were tested to determine the prediction performance of the PEIs. Using the MLP algorithm prediction in the neural network to predict health and each of the 34 unhealthy statuses (multi-classification) did not achieve good results. Thus, we attempted to predict healthy statuses from each unhealthy status using the binary classification method. The F1 value of the prediction of each result was close to zero. Using support vector machine algorithm prediction to conduct multi-classification prediction, the highest F1_score was obtained for cholecystolithiasis (0.70), while that of other types of diseases was 0.00. We also tried the binary classification method, but all the results were relatively poor. When an RF algorithm was used for prediction in multi-classification (health and each of the 34 unhealthy statuses), the F1 value of a healthy status was 0.80–0.90, whereas the F1 value of an unhealthy status was 0.00–0.40. We then chose an RF algorithm and optimized it. First, due to the uneven distribution of the sample numbers of healthy and non-healthy statuses and the law of large numbers^38^, we used a down-sampling strategy for the randomly used sample. As the data were characterized by serious category imbalance, a random under-sampling method was adopted that balanced the data by randomly selecting the data subset of the target class. Second, we used the PEI feature extraction strategy to extract the most contributed PEIs for each healthy and unhealthy status. Feature extraction adopts the strategy of univariate statistics in automatic feature selection. Univariate statistics select features with high confidence according to the statistical significance of the relationship between each feature and the target. This process can be achieved by using feature_selection in scikit-learn. Finally, in each healthy and non-healthy status, the top 15% or 16% representative PEIs were extracted for prediction using feature extraction. The advantage of this method is that it is usually very fast and completely independent of the model applied after feature selection. Then, the data were randomly divided so that 30% constituted the test set, and the remaining 70% were randomly divided again, with 70% as the training set for the training model and 30% as the validation set for the evaluation model. In the process of improving the generalization performance of the model by adjusting the parameters, a cross-validation method with a grid search was adopted, which can be implemented by GridSearchCV provided by scikit-learn (Table S37 and Supplementary Code). In our RF model, in the randomforestclassifier() function, criterion = 'entropy', random_ State = 3, and for the RF model of binary data, we mainly adjusted n_estimators, max_depth, and min_samples_leaf. These three parameters enabled the model to achieve better results, while the other parameters were defaults. The evaluation of the model effect was mainly based on sklearn, metrics F1 in_score, and ROC_Curve. The AUC was calculated according to the true positive rate and the false-positive rate, and the ROC curve was drawn accordingly.

### Ethics approval and consent to participate

The study was approved by the institutional ethics committee of Sichuan Provincial People’s Hospital (No. 2019276) according to the following rules: (1) ethical review of biomedical research involving human beings (Order of the State Health and Family Planning Commission of the People’s Republic of China, 2016), (2) WMA Declaration of Helsinki (2013), and (3) CIOMS International Ethical Guidelines for Biomedical Research Involving Human Subjects. This study extracted only the clinical PEI data. No patient identity was involved in this study. As the researchers applied for exemption from informed consent, they did their best to protect the information provided by the patients and prevent breaching of personal privacy.

## Supplementary Information

Supplementary Information.

## Data Availability

All of the data can be found in the supplementary datasets.

## Abbreviations

PEIs: Physical examination indicators
BMI: Body mass index
MLP: Multilayer perceptron
AUC: Area under the curve
RF: Random forest

## Code availability

The R script used in this research is publicly available and can be found in the supplementary data.
