# Supplementary material for: A correlation-based feature analysis of physical examination indicators can help predict the overall underlying health status using machine learning
Source: Sci Rep. 2022 Nov 15;12:19626. doi: 10.1038/s41598-022-20474-3 (PMC9666446; doi:10.1038/s41598-022-20474-3)
Supplement: Supplementary file 1 — Supplementary Information. [file 41598_2022_20474_MOESM1_ESM.pdf]

Supplementary Materials for

Correlation-based feature analysis in physical examination indicators can help predict overall underlying health status using Machine Learning

Supplementary Tables

Supplementary Table 1. PEI Correlations in Healthy status

Supplementary Table 2. PEI Correlations in Cholecystolithiasis

Supplementary Table 3. PEI Correlations in Hypertension

Supplementary Table 4. PEI Correlations in Hypertension+Diabetes

Supplementary Table 5. PEI Correlations in Hypertension+Coronary

Supplementary Table 6. PEI Correlations in Hypertensive+Diabetes+Coronary

Supplementary Table 7. PEI Correlations in Hyperlipidemia

Supplementary Table 8. PEI Correlations in Coronary heart disease

Supplementary Table 9. PEI Correlations in Coronary+Diabetes

Supplementary Table 10. PEI Correlations in Rhinallergosis

Supplementary Table 11. PEI Correlations in Hypothyroidism

Supplementary Table 12. PEI Correlations in Hyperthyroidism

Supplementary Table 13. PEI Correlations in Cervical spondylopathy

Supplementary Table 14. PEI Correlations in Rheumatoid arthritis

Supplementary Table 15. PEI Correlations in Chronic rhinitis

Supplementary Table 16. PEI Correlations in Nephropathy

Supplementary Table 17.PEI Correlations in Diabetes

Supplementary Table 18. PEI Correlations in Gout

Supplementary Table 19. PEI Correlations in Parkinson's syndrome

Supplementary Table 20. PEI Correlations in Stomach trouble

Supplementary Table 21. PEI Correlations in Chronic pharyngitis

Supplementary Table 22. PEI Correlations in Lumbar disc protrusion

Supplementary Table 23. PEI Correlations in Hepatitis B

Supplementary Table 24. PEI Correlations in Hypertension+other diseases

Supplementary Table 25. PEI Correlations in Coronary+others

Supplementary Table 26. PEI Correlations in Diabetes+others

Supplementary Table 27. PEI Correlations in Bronchial disease

Supplementary Table 28. PEI Correlations in Other disease conditions

Supplementary Table 29. PEI Correlations in Brain diseases

Supplementary Table 30. PEI Correlations in Hepatic adipose infiltration

Supplementary Table 31. PEI Correlations in Asthma

Supplementary Table 32. PEI Correlations in Other Cardiac diseases

Supplementary Table 33. PEI Correlations in Heart disease

Supplementary Table 34. PEI Correlations in Hepatopathy

Supplementary Table 35. PEI Correlations in Pregnant

Supplementary Table 36. P values of PEIs in healthy physical status vs 34 unhealthy physical status adjusted for age and sex.

Supplementary Table 37. Optimal parameter combination of machine learning

Supplementary Table 38. List of the 15% or 16% representative PEIs were extracted for machine learning prediction by feature extraction

Supplementary codes for machine learning

Supplementary Table 1. PEI Correlations in Healthy status

Row	Column	Row	Column	Cor	*P*
B1	B142	Gender	25-(OH)D	0.006119	9.18E-08
B1	B137	Gender	AFU	-0.03423	0
B1	B24	Gender	αHBDH	0.01309	0
B1	B16	Gender	CD3+T	0.005632	8.79E-07
B1	B15	Gender	CD3-CD19+(B cell)	0.008684	3.42E-14
B1	B23	Gender	CD3-CD16+CD56+(NK)	-0.00619	6.67E-08
B1	B18	Gender	CD3+CD4+helper(inducer)T	0.007416	9.49E-11
B1	B54	Gender	ESR	0.053469	0
B1	B13	Gender	PGI_PGII	-0.00659	8.71E-09
B1	B160	Gender	PH	0.069872	0
B1	B140	Gender	r-GT	-0.22855	0
B1	B143	Gender	β-CTx	-0.0067	4.93E-09
B1	B82	Gender	CEA	-0.00569	6.80E-07
B1	B118	Gender	CEA1	-0.0265	0
B1	B126	Gender	Alb	-0.1868	0
B1	B127	Gender	A-G ratio	-0.17598	0
B1	B161	Gender	LEU	0.170258	0
B1	B40	Gender	WBC	-0.16164	0
B1	B128	Gender	ALT	-0.27333	0
B1	B164	Gender	COM	0.012681	0
B1	B123	Gender	hs-CRP1	-0.01896	0
B1	B111	Gender	TSH	0.053927	0
B1	B124	Gender	ACTH	-0.01648	0
B1	B41	Gender	MON％	-0.0411	0
B1	B165	Gender	bilirubin	-0.00871	2.78E-14
B1	B134	Gender	CHE	-0.07687	0
B1	B166	Gender	Pro	-0.17502	0
B1	B70	Gender	LDL-C	-0.13992	0
B1	B188	Gender	Ca	-0.0283	0
B1	B71	Gender	TG	-0.23139	0
B1	B72	Gender	HDL-C	0.36582	0
B1	B32	Gender	Testo	-0.01778	0
B1	B189	Gender	eGFB	0.224096	0
B1	B141	Gender	AST/ALT	0.304609	0
B1	B146	Gender	CysC	-0.04504	0
B1	B55	Gender	TK	0.015659	0
B1	B56	Gender	IR	-0.05577	0
B1	B57	Gender	RBCAI	-0.02178	0
B1	B43	Gender	MCV	-0.01009	0
B1	B44	Gender	MCH	-0.13679	0
B1	B45	Gender	MCHC	-0.3201	0
B1	B46	Gender	RBC Count	-0.58863	0

B1	B47	Gender	RDW	0.067555	0
B1	B58	Gender	HCT	-0.01435	0
B1	B181	Gender	APTT	0.013743	0
B1	B190	Gender	Cre	-0.65885	0
B1	B25	Gender	CK	-0.01605	0
B1	B26	Gender	CKMB	-0.00901	3.77E-15
B1	B224	Gender	pmh	-0.01316	0
B1	B129	Gender	IBIL	-0.14964	0
B1	B150	Gender	ALP	-0.05854	0
B1	B84	Gender	C-PSA	-0.04495	0
B1	B222	Gender	NS	-0.70683	0
B1	B100	Gender	anti-Ro52	0.006769	3.42E-09
B1	B103	Gender	anti-SSA/Ro	0.008765	1.98E-14
B1	B148	Gender	Anti_TPO	0.061984	0
B1	B149	Gender	TGAb	0.055607	0
B1	B198	Gender	P	0.055717	0
B1	B85	Gender	SCC	-0.02093	0
B1	B194	Gender	Cl	0.020809	0
B1	B199	Gender	Mg	-0.01106	0
B1	B156	Gender	IgE	-0.01045	0
B1	B157	Gender	IgG	0.032658	0
B1	B158	Gender	IgM	0.022932	0
B1	B195	Gender	Na	-0.02171	0
B1	B2	Gender	Age	-0.05802	0
B1	B176	Gender	Ucr	-0.13356	0
B1	B178	Gender	Ig-U	-0.0075	6.00E-11
B1	B191	Gender	Urea	-0.18982	0
B1	B174	Gender	Urea/Cr	0.318269	0
B1	B192	Gender	UA	-0.58161	0
B1	B170	Gender	U-GLU	-0.05063	0
B1	B175	Gender	ALB-U	-0.01348	0
B1	B177	Gender	ACR	0.006481	1.53E-08
B1	B179	Gender	α1-MG	-0.04255	0
B1	B180	Gender	Tf	-0.00562	9.21E-07
B1	B182	Gender	TT	-0.00926	6.66E-16
B1	B125	Gender	Cor	-0.00973	0
B1	B153	Gender	Glu	-0.01674	0
B1	B138	Gender	PAB	-0.09439	0
B1	B130	Gender	GLB	0.12852	0
B1	B59	Gender	WBRV	-0.03039	0
B1	B60	Gender	WBRVI	-0.09873	0
B1	B4	Gender	WB-Ca	0.008595	6.22E-14
B1	B61	Gender	WBHSRV	-0.05458	0
B1	B62	Gender	WBHSRVI	-0.10412	0

B1	B5	Gender	WB-Mg	-0.01124	0
B1	B6	Gender	WB-Fe	-0.01874	0
B1	B8	Gender	WB-Zinc	-0.01371	0
B1	B63	Gender	WBRSR(mPas_200)	-0.01144	0
B1	B64	Gender	WBRSR(mPas_5)	-0.10303	0
B1	B65	Gender	WBRSR(mPas_1)	-0.09742	0
B1	B66	Gender	WBRSR(mPas_100)	-0.10109	0
B1	B67	Gender	WBRSR(mPas_50)	-0.10189	0
B1	B86	Gender	TBhCG	0.0104	0
B1	B27	Gender	LDH	-0.01558	0
B1	B221	Gender	Hei	-0.66881	0
B1	B87	Gender	NSE	-0.01219	0
B1	B187	Gender	NSE	-0.00728	2.04E-10
B1	B200	Gender	OP	-0.02806	0
B1	B227	Gender	OD	-0.00874	2.26E-14
B1	B228	Gender	OS	-0.00648	1.50E-08
B1	B50	Gender	eo%	-0.11116	0
B1	B220	Gender	SBP	-0.27128	0
B1	B219	Gender	OT	0.05683	0
B1	B218	Gender	DBP	-0.27886	0
B1	B88	Gender	CA 242	0.035479	0
B1	B216	Gender	BMI	-0.61822	0
B1	B215	Gender	BMI	-0.35301	0
B1	B131	Gender	AST	-0.15686	0
B1	B3	Gender	Hcy	-0.12242	0
B1	B172	Gender	KB	0.016762	0
B1	B210	Gender	Hip	-0.3691	0
B1	B10	Gender	PG I	-0.04914	0
B1	B12	Gender	PG II	-0.01606	0
B1	B14	Gender	G-17	0.008401	2.22E-13
B1	B209	Gender	Smoking	-0.3766	0
B1	B90	Gender	CYFRA 21-1	-0.03144	0
B1	B139	Gender	ADA	0.02563	0
B1	B34	Gender	TK	0.010848	0
B1	B196	Gender	TCO2	-0.00938	2.22E-16
B1	B51	Gender	Hb	-0.68896	0
B1	B185	Gender	PT	0.007786	1.06E-11
B1	B69	Gender	PV	0.006374	2.62E-08
B1	B52	Gender	PLT/BPC	0.095249	0
B1	B173	Gender	NTT	0.066106	0
B1	B207	Gender	WHR	-0.06406	0
B1	B206	Gender	Waist	-0.54132	0
B1	B205	Gender	KDA	0.052062	0
B1	B78	Gender	HBcAb	0.01236	0

B1	B197	Gender	AG	-0.01879	0
B1	B204	Gender	Drinking	-0.40366	0
B1	B203	Gender	DH	-0.01991	0
B1	B80	Gender	anti-HP IgG	-0.00762	2.96E-11
B1	B112	Gender	FT4	-0.02146	0
B1	B113	Gender	FT3	-0.09289	0
B1	B211	Gender	IOP-R	-0.01424	0
B1	B201	Gender	e-habits	-0.04823	0
B1	B73	Gender	ApoA	0.023537	0
B1	B74	Gender	ApoA1	0.103329	0
B1	B75	Gender	ApoA1/B	0.054433	0
B1	B76	Gender	ApoB	-0.05962	0
B1	B132	Gender	Dbil	-0.10135	0
B1	B53	Gender	GR%	0.010359	0
B1	B77	Gender	TC	-0.05594	0
B1	B133	Gender	TBIL	-0.14723	0
B1	B135	Gender	TBA	-0.0177	0
B1	B91	Gender	TP	-0.00627	4.41E-08
B1	B114	Gender	TSH TT3 TT4(TT4)	0.025425	0
B1	B115	Gender	TSH TT3 TT4(TT3)	-0.02896	0
B1	B212	Gender	IOP-L	-0.01604	0
B10	B137	PG I	AFU	0.010186	0
B10	B24	PG I	αHBDH	0.017946	0
B10	B15	PG I	CD3-CD19+(B cell)	-0.05491	0
B10	B18	PG I	CD3+CD4+helper(inducer)T	0.010841	0
B10	B19	PG I	CD3+CD4+/CD3+CD8+	0.018875	0
B10	B20	PG I	CD3+CD4+CD8+	-0.00592	2.32E-07
B10	B21	PG I	CD3+CD4-CD8-	0.006365	2.74E-08
B10	B22	PG I	CD3+CD8+(T)	-0.01218	0
B10	B92	PG I	ALDH2-Glu504Glu	-0.0134	0
B10	B93	PG I	ALDH2-Glu504Lys	0.010022	0
B10	B160	PG I	PH	-0.02943	0
B10	B94	PG I	ALDH2-Lys504Lys	0.010844	0
B10	B118	PG I	CEA1	0.005876	2.90E-07
B10	B126	PG I	Alb	-0.01971	0
B10	B40	PG I	WBC	0.019164	0
B10	B128	PG I	ALT	0.009124	1.78E-15
B10	B225	PG I	hPBG	-0.00719	3.45E-10
B10	B123	PG I	hs-CRP1	0.006447	1.82E-08
B10	B124	PG I	ACTH	0.015006	0
B10	B70	PG I	LDL-C	0.031616	0
B10	B71	PG I	TG	0.010351	0
B10	B189	PG I	eGFB	-0.08535	0
B10	B141	PG I	AST/ALT	-0.00774	1.36E-11

B10	B146	PG I	CysC	0.014413	0
B10	B57	PG I	RBCAI	0.011771	0
B10	B43	PG I	MCV	0.012397	0
B10	B44	PG I	MCH	0.018991	0
B10	B46	PG I	RBC Count	0.011124	0
B10	B47	PG I	RDW	0.035438	0
B10	B181	PG I	APTT	-0.02577	0
B10	B190	PG I	Cre	0.063659	0
B10	B193	PG I	K	0.006141	8.26E-08
B10	B129	PG I	IBIL	-0.0197	0
B10	B150	PG I	ALP	0.021992	0
B10	B84	PG I	C-PSA	0.008417	2.01E-13
B10	B222	PG I	NS	0.022326	0
B10	B100	PG I	anti-Ro52	-0.00932	4.44E-16
B10	B151	PG I	Anti-CCP	0.007438	8.39E-11
B10	B148	PG I	Anti_TPO	-0.00902	3.55E-15
B10	B149	PG I	TGAb	-0.00754	4.61E-11
B10	B194	PG I	Cl	-0.00593	2.24E-07
B10	B199	PG I	Mg	0.012483	0
B10	B155	PG I	IgA	-0.00851	1.10E-13
B10	B195	PG I	Na	0.020531	0
B10	B176	PG I	Ucr	0.012771	0
B10	B191	PG I	Urea	0.039168	0
B10	B174	PG I	Urea/Cr	-0.01456	0
B10	B192	PG I	UA	0.026381	0
B10	B175	PG I	ALB-U	0.008551	8.30E-14
B10	B177	PG I	ACR	0.008491	1.23E-13
B10	B179	PG I	α1-MG	0.00608	1.11E-07
B10	B125	PG I	Cor	0.02443	0
B10	B138	PG I	PAB	0.02251	0
B10	B130	PG I	GLB	-0.00694	1.36E-09
B10	B59	PG I	WBRV	0.010411	0
B10	B60	PG I	WBRVI	0.012409	0
B10	B64	PG I	WBRSR(mPas_5)	0.009995	0
B10	B65	PG I	WBRSR(mPas_1)	0.01276	0
B10	B27	PG I	LDH	0.012259	0
B10	B221	PG I	Hei	0.014505	0
B10	B87	PG I	NSE	-0.01231	0
B10	B200	PG I	OP	0.021204	0
B10	B50	PG I	eo%	0.012345	0
B10	B220	PG I	SBP	0.022396	0
B10	B219	PG I	OT	0.005827	3.63E-07
B10	B218	PG I	DBP	0.022265	0
B10	B216	PG I	BMI	0.009571	0

B10	B131	PG I	AST	0.01561	0
B10	B12	PG I	PG II	0.628138	0
B10	B14	PG I	G-17	0.085572	0
B10	B209	PG I	Smoking	0.056463	0
B10	B90	PG I	CYFRA 21-1	0.016941	0
B10	B196	PG I	TCO2	0.011114	0
B10	B51	PG I	Hb	0.02663	0
B10	B183	PG I	PTA	-0.03943	0
B10	B185	PG I	PT	-0.00743	8.92E-11
B10	B186	PG I	PFC	0.020994	0
B10	B52	PG I	PLT/BPC	-0.02383	0
B10	B206	PG I	Waist	0.017401	0
B10	B78	PG I	HBcAb	-0.01705	0
B10	B197	PG I	AG	0.007119	5.12E-10
B10	B204	PG I	Drinking	0.027011	0
B10	B80	PG I	anti-HP IgG	0.091035	0
B10	B112	PG I	FT4	0.008042	2.20E-12
B10	B113	PG I	FT3	0.014429	0
B10	B201	PG I	e-habits	0.005969	1.88E-07
B10	B74	PG I	ApoA1	0.022909	0
B10	B76	PG I	ApoB	0.011415	0
B10	B132	PG I	Dbil	-0.00683	2.47E-09
B10	B81	PG I	TSGF	-0.01297	0
B10	B77	PG I	TC	0.024963	0
B10	B133	PG I	TBIL	-0.01767	0
B10	B135	PG I	TBA	0.006542	1.12E-08
B10	B91	PG I	TP	-0.01812	0
B10	B114	PG I	TSH TT3 TT4(TT4)	0.007412	9.74E-11
B100	B137	anti-Ro52	AFU	0.010159	0
B100	B126	anti-Ro52	Alb	-0.00581	3.85E-07
B100	B121	anti-Ro52	C3	-0.01934	0
B100	B122	anti-Ro52	C4	-0.01554	0
B100	B124	anti-Ro52	ACTH	-0.00644	1.89E-08
B100	B181	anti-Ro52	APTT	0.006038	1.35E-07
B100	B105	anti-Ro52	anti-U1RNP	0.068691	0
B100	B101	anti-Ro52	anti-Sc70	0.016829	0
B100	B102	anti-Ro52	anti-Sm	0.081385	0
B100	B103	anti-Ro52	anti-SSA/Ro	0.304624	0
B100	B104	anti-Ro52	anti-SSB	0.187733	0
B100	B106	anti-Ro52	anti-P	0.051897	0
B100	B107	anti-Ro52	AnuA	0.062463	0
B100	B151	anti-Ro52	Anti-CCP	0.040999	0
B100	B149	anti-Ro52	TGAb	0.006771	3.40E-09
B100	B108	anti-Ro52	Anti-Cyclin	0.008594	6.26E-14

B100	B109	anti-Ro52	AMA-M2	0.068363	0
B100	B110	anti-Ro52	AHA	0.013377	0
B100	B154	anti-Ro52	RF	0.021169	0
B100	B194	anti-Ro52	Cl	0.006234	5.26E-08
B100	B155	anti-Ro52	IgA	0.032978	0
B100	B157	anti-Ro52	IgG	0.063538	0
B100	B158	anti-Ro52	IgM	0.01349	0
B100	B125	anti-Ro52	Cor	-0.00772	1.54E-11
B100	B138	anti-Ro52	PAB	-0.02044	0
B100	B130	anti-Ro52	GLB	0.01169	0
B100	B139	anti-Ro52	ADA	0.028859	0
B100	B183	anti-Ro52	PTA	0.007659	2.29E-11
B100	B114	anti-Ro52	TSH TT3 TT4(TT4)	0.011082	0
B100	B115	anti-Ro52	TSH TT3 TT4(TT3)	0.005664	7.62E-07
B101	B193	anti-Sc70	K	0.007466	7.12E-11
B101	B105	anti-Sc70	anti-U1RNP	0.066968	0
B101	B103	anti-Sc70	anti-SSA/Ro	0.023016	0
B101	B104	anti-Sc70	anti-SSB	0.061081	0
B101	B108	anti-Sc70	Anti-Cyclin	0.037171	0
B101	B109	anti-Sc70	AMA-M2	0.020519	0
B101	B110	anti-Sc70	AHA	-0.00718	3.67E-10
B101	B156	anti-Sc70	IgE	-0.00629	3.96E-08
B101	B157	anti-Sc70	IgG	0.01654	0
B101	B179	anti-Sc70	α1-MG	-0.00657	9.71E-09
B101	B138	anti-Sc70	PAB	-0.00849	1.23E-13
B102	B137	anti-Sm	AFU	-0.01489	0
B102	B127	anti-Sm	A-G ratio	0.015283	0
B102	B121	anti-Sm	C3	-0.01543	0
B102	B105	anti-Sm	anti-U1RNP	0.286285	0
B102	B103	anti-Sm	anti-SSA/Ro	0.072992	0
B102	B104	anti-Sm	anti-SSB	0.066566	0
B102	B106	anti-Sm	anti-P	0.041932	0
B102	B109	anti-Sm	AMA-M2	0.019808	0
B102	B110	anti-Sm	AHA	-0.00717	3.80E-10
B102	B194	anti-Sm	Cl	-0.006	1.61E-07
B102	B199	anti-Sm	Mg	0.007209	3.09E-10
B102	B176	anti-Sm	Ucr	0.008071	1.83E-12
B102	B125	anti-Sm	Cor	0.018239	0
B102	B139	anti-Sm	ADA	0.010846	0
B102	B186	anti-Sm	PFC	0.00748	6.55E-11
B103	B126	anti-SSA/Ro	Alb	-0.00704	8.06E-10
B103	B127	anti-SSA/Ro	A-G ratio	-0.00704	8.02E-10
B103	B121	anti-SSA/Ro	C3	-0.01517	0
B103	B122	anti-SSA/Ro	C4	-0.01289	0

B103	B124	anti-SSA/Ro	ACTH	-0.00751	5.45E-11
B103	B134	anti-SSA/Ro	CHE	-0.00998	0
B103	B181	anti-SSA/Ro	APTT	0.012879	0
B103	B222	anti-SSA/Ro	NS	-0.00681	2.73E-09
B103	B105	anti-SSA/Ro	anti-U1RNP	0.057651	0
B103	B104	anti-SSA/Ro	anti-SSB	0.22971	0
B103	B106	anti-SSA/Ro	anti-P	0.091291	0
B103	B107	anti-SSA/Ro	AnuA	0.090977	0
B103	B151	anti-SSA/Ro	Anti-CCP	0.050766	0
B103	B149	anti-SSA/Ro	TGAb	0.00767	2.14E-11
B103	B109	anti-SSA/Ro	AMA-M2	0.040678	0
B103	B110	anti-SSA/Ro	AHA	0.007172	3.81E-10
B103	B154	anti-SSA/Ro	RF	0.011708	0
B103	B199	anti-SSA/Ro	Mg	-0.00687	2.03E-09
B103	B155	anti-SSA/Ro	IgA	0.037388	0
B103	B157	anti-SSA/Ro	IgG	0.080767	0
B103	B158	anti-SSA/Ro	IgM	0.014193	0
B103	B195	anti-SSA/Ro	Na	-0.01097	0
B103	B176	anti-SSA/Ro	Ucr	-0.01038	0
B103	B192	anti-SSA/Ro	UA	-0.00584	3.36E-07
B103	B138	anti-SSA/Ro	PAB	-0.01967	0
B103	B130	anti-SSA/Ro	GLB	0.014644	0
B103	B221	anti-SSA/Ro	Hei	-0.00663	7.15E-09
B103	B200	anti-SSA/Ro	OP	-0.01133	0
B103	B139	anti-SSA/Ro	ADA	0.019352	0
B103	B185	anti-SSA/Ro	PT	0.00852	1.02E-13
B103	B197	anti-SSA/Ro	AG	-0.01103	0
B103	B211	anti-SSA/Ro	IOP-R	-0.00641	2.21E-08
B103	B114	anti-SSA/Ro	TSH TT3 TT4(TT4)	0.00721	3.08E-10
B103	B212	anti-SSA/Ro	IOP-L	-0.00669	5.26E-09
B104	B121	anti-SSB	C3	-0.02096	0
B104	B122	anti-SSB	C4	-0.01066	0
B104	B124	anti-SSB	ACTH	-0.00668	5.57E-09
B104	B181	anti-SSB	APTT	0.011252	0
B104	B105	anti-SSB	anti-U1RNP	0.073403	0
B104	B106	anti-SSB	anti-P	0.028919	0
B104	B107	anti-SSB	AnuA	0.045562	0
B104	B151	anti-SSB	Anti-CCP	-0.00788	5.89E-12
B104	B108	anti-SSB	Anti-Cyclin	-0.00587	2.98E-07
B104	B109	anti-SSB	AMA-M2	0.056055	0
B104	B155	anti-SSB	IgA	0.018072	0
B104	B156	anti-SSB	IgE	0.009132	1.55E-15
B104	B157	anti-SSB	IgG	0.039856	0
B104	B179	anti-SSB	α1-MG	-0.00642	2.10E-08

B104	B182	anti-SSB	TT	0.009376	2.22E-16
B104	B125	anti-SSB	Cor	-0.01473	0
B104	B138	anti-SSB	PAB	-0.00711	5.39E-10
B104	B130	anti-SSB	GLB	0.006943	1.35E-09
B104	B200	anti-SSB	OP	-0.00738	1.20E-10
B104	B139	anti-SSB	ADA	0.00685	2.22E-09
B104	B196	anti-SSB	TCO2	-0.00599	1.67E-07
B105	B137	anti-U1RNP	AFU	-0.00825	5.95E-13
B105	B127	anti-U1RNP	A-G ratio	0.006534	1.16E-08
B105	B121	anti-U1RNP	C3	-0.01244	0
B105	B122	anti-U1RNP	C4	-0.00959	0
B105	B111	anti-U1RNP	TSH	0.01013	0
B105	B193	anti-U1RNP	K	0.005837	3.46E-07
B105	B106	anti-U1RNP	anti-P	0.082975	0
B105	B107	anti-U1RNP	AnuA	0.118194	0
B105	B108	anti-U1RNP	Anti-Cyclin	-0.00767	2.13E-11
B105	B109	anti-U1RNP	AMA-M2	0.027642	0
B105	B110	anti-U1RNP	AHA	0.024614	0
B105	B155	anti-U1RNP	IgA	0.012617	0
B105	B156	anti-U1RNP	IgE	-0.00645	1.75E-08
B105	B157	anti-U1RNP	IgG	0.026022	0
B105	B182	anti-U1RNP	TT	0.006256	4.72E-08
B105	B139	anti-U1RNP	ADA	0.010525	0
B105	B196	anti-U1RNP	TCO2	-0.006	1.64E-07
B105	B113	anti-U1RNP	FT3	0.006363	2.77E-08
B106	B121	anti-P	C3	-0.0133	0
B106	B122	anti-P	C4	-0.01544	0
B106	B107	anti-P	AnuA	0.219492	0
B106	B108	anti-P	Anti-Cyclin	-0.00581	4.00E-07
B106	B109	anti-P	AMA-M2	-0.00594	2.17E-07
B106	B110	anti-P	AHA	0.015638	0
B106	B198	anti-P	P	-0.006	1.59E-07
B106	B194	anti-P	Cl	-0.00838	2.61E-13
B106	B155	anti-P	IgA	0.006514	1.29E-08
B106	B156	anti-P	IgE	0.011679	0
B106	B157	anti-P	IgG	0.044119	0
B106	B195	anti-P	Na	-0.00811	1.44E-12
B106	B182	anti-P	TT	-0.01512	0
B106	B125	anti-P	Cor	-0.0111	0
B106	B200	anti-P	OP	-0.00578	4.45E-07
B106	B196	anti-P	TCO2	0.008402	2.22E-13
B106	B186	anti-P	PFC	0.007512	5.43E-11
B106	B197	anti-P	AG	-0.00592	2.37E-07
B107	B127	AnuA	A-G ratio	-0.00624	5.04E-08

B107	B121	AnuA	C3	-0.02772	0
B107	B122	AnuA	C4	-0.03198	0
B107	B151	AnuA	Anti-CCP	0.013995	0
B107	B149	AnuA	TGAb	0.007849	7.25E-12
B107	B109	AnuA	AMA-M2	0.019017	0
B107	B110	AnuA	AHA	0.078715	0
B107	B157	AnuA	IgG	0.072514	0
B107	B130	AnuA	GLB	0.009929	0
B108	B181	Anti-Cyclin	APTT	0.006197	6.30E-08
B108	B151	Anti-Cyclin	Anti-CCP	0.01202	0
B108	B109	Anti-Cyclin	AMA-M2	0.056451	0
B108	B110	Anti-Cyclin	AHA	0.015779	0
B108	B198	Anti-Cyclin	P	-0.00684	2.40E-09
B108	B195	Anti-Cyclin	Na	-0.00588	2.88E-07
B108	B179	Anti-Cyclin	α1-MG	-0.00695	1.30E-09
B108	B125	Anti-Cyclin	Cor	0.010466	0
B108	B228	Anti-Cyclin	OS	0.005784	4.42E-07
B108	B139	Anti-Cyclin	ADA	0.017155	0
B109	B225	AMA-M2	hPBG	0.00827	5.20E-13
B109	B123	AMA-M2	hs-CRP1	0.018146	0
B109	B181	AMA-M2	APTT	-0.00573	5.61E-07
B109	B194	AMA-M2	Cl	-0.00857	7.11E-14
B109	B199	AMA-M2	Mg	-0.00817	9.80E-13
B109	B157	AMA-M2	IgG	0.021261	0
B109	B158	AMA-M2	IgM	0.00561	9.69E-07
B109	B195	AMA-M2	Na	-0.01041	0
B109	B179	AMA-M2	α1-MG	-0.00591	2.47E-07
B109	B182	AMA-M2	TT	-0.02179	0
B109	B125	AMA-M2	Cor	0.023829	0
B109	B186	AMA-M2	PFC	0.018456	0
B11	B12	PGI/PGII	PG II	-0.01714	0
B11	B14	PGI/PGII	G-17	-0.0057	6.50E-07
B110	B121	AHA	C3	0.007298	1.87E-10
B110	B155	AHA	IgA	0.019731	0
B110	B157	AHA	IgG	0.023016	0
B110	B158	AHA	IgM	0.010773	0
B110	B195	AHA	Na	-0.00861	5.73E-14
B110	B159	AHA	β2-MG	0.006176	6.96E-08
B110	B176	AHA	Ucr	-0.01037	0
B110	B179	AHA	α1-MG	-0.0172	0
B110	B182	AHA	TT	-0.00655	1.09E-08
B110	B138	AHA	PAB	-0.01122	0
B110	B200	AHA	OP	-0.00619	6.59E-08
B110	B196	AHA	TCO2	-0.01051	0

B110	B183	AHA	PTA	0.011047	0
B110	B112	AHA	FT4	0.006071	1.16E-07
B111	B137	TSH	AFU	-0.00726	2.38E-10
B111	B160	TSH	PH	-0.00795	4.00E-12
B111	B140	TSH	r-GT	-0.00744	8.24E-11
B111	B127	TSH	A-G ratio	-0.02812	0
B111	B161	TSH	LEU	0.014382	0
B111	B134	TSH	CHE	-0.01449	0
B111	B166	TSH	Pro	-0.01495	0
B111	B189	TSH	eGFB	-0.03173	0
B111	B141	TSH	AST/ALT	0.017824	0
B111	B190	TSH	Cre	-0.00868	3.60E-14
B111	B193	TSH	K	-0.00668	5.43E-09
B111	B129	TSH	IBIL	-0.018	0
B111	B222	TSH	NS	-0.02494	0
B111	B148	TSH	Anti_TPO	0.162953	0
B111	B149	TSH	TGAb	0.129817	0
B111	B198	TSH	P	0.014221	0
B111	B157	TSH	IgG	0.012499	0
B111	B176	TSH	Ucr	-0.02336	0
B111	B174	TSH	Urea/Cr	0.009609	0
B111	B192	TSH	UA	-0.02139	0
B111	B177	TSH	ACR	0.006254	4.75E-08
B111	B179	TSH	α1-MG	-0.00658	9.10E-09
B111	B125	TSH	Cor	0.007793	1.02E-11
B111	B130	TSH	GLB	0.037154	0
B111	B221	TSH	Hei	-0.04766	0
B111	B219	TSH	OT	0.009276	4.44E-16
B111	B216	TSH	BMI	-0.01884	0
B111	B215	TSH	BMI	0.009579	0
B111	B131	TSH	AST	0.0116	0
B111	B172	TSH	KB	-0.00932	4.44E-16
B111	B209	TSH	Smoking	-0.03073	0
B111	B139	TSH	ADA	0.008606	5.77E-14
B111	B173	TSH	NTT	0.01005	0
B111	B204	TSH	Drinking	-0.02292	0
B111	B112	TSH	FT4	-0.19912	0
B111	B113	TSH	FT3	-0.08867	0
B111	B132	TSH	Dbil	-0.01877	0
B111	B133	TSH	TBIL	-0.01944	0
B111	B135	TSH	TBA	0.005614	9.51E-07
B111	B114	TSH	TSH TT3 TT4(TT4)	-0.10074	0
B111	B115	TSH	TSH TT3 TT4(TT3)	-0.02769	0
B112	B140	FT4	r-GT	-0.01611	0

B112	B143	FT4	β-CTx	0.014658	0
B112	B126	FT4	Alb	0.05703	0
B112	B127	FT4	A-G ratio	0.020847	0
B112	B128	FT4	ALT	0.00599	1.70E-07
B112	B123	FT4	hs-CRP1	0.01995	0
B112	B124	FT4	ACTH	0.006072	1.15E-07
B112	B134	FT4	CHE	0.005762	4.89E-07
B112	B166	FT4	Pro	0.024457	0
B112	B188	FT4	Ca	0.012081	0
B112	B189	FT4	eGFB	0.032014	0
B112	B141	FT4	AST/ALT	0.009844	0
B112	B145	FT4	BGP	0.013181	0
B112	B193	FT4	K	-0.00699	1.05E-09
B112	B129	FT4	IBIL	0.05785	0
B112	B150	FT4	ALP	0.012841	0
B112	B222	FT4	NS	-0.01936	0
B112	B151	FT4	Anti-CCP	0.009364	4.44E-16
B112	B198	FT4	P	0.010652	0
B112	B194	FT4	Cl	-0.01474	0
B112	B199	FT4	Mg	-0.01066	0
B112	B155	FT4	IgA	0.006083	1.09E-07
B112	B157	FT4	IgG	0.0109	0
B112	B195	FT4	Na	-0.01303	0
B112	B176	FT4	Ucr	0.021399	0
B112	B191	FT4	Urea	-0.01024	0
B112	B170	FT4	U-GLU	0.009164	1.33E-15
B112	B182	FT4	TT	-0.01947	0
B112	B125	FT4	Cor	0.019372	0
B112	B138	FT4	PAB	-0.02082	0
B112	B221	FT4	Hei	0.026648	0
B112	B200	FT4	OP	-0.01135	0
B112	B220	FT4	SBP	0.013427	0
B112	B218	FT4	DBP	0.019777	0
B112	B216	FT4	BMI	-0.03137	0
B112	B215	FT4	BMI	-0.06166	0
B112	B131	FT4	AST	0.011017	0
B112	B172	FT4	KB	0.027179	0
B112	B210	FT4	Hip	-0.03318	0
B112	B139	FT4	ADA	0.012418	0
B112	B196	FT4	TCO2	0.007455	7.56E-11
B112	B183	FT4	PTA	0.006364	2.75E-08
B112	B185	FT4	PT	0.007847	7.34E-12
B112	B186	FT4	PFC	0.019034	0
B112	B206	FT4	Waist	-0.04204	0

B112	B204	FT4	Drinking	-0.0097	0
B112	B113	FT4	FT3	0.459532	0
B112	B211	FT4	IOP-R	0.023889	0
B112	B201	FT4	e-habits	-0.00885	1.09E-14
B112	B132	FT4	Dbil	0.049252	0
B112	B117	FT4	TT4	0.013294	0
B112	B133	FT4	TBIL	0.059506	0
B112	B114	FT4	TSH TT3 TT4(TT4)	0.452302	0
B112	B115	FT4	TSH TT3 TT4(TT3)	0.145866	0
B112	B144	FT4	Total-PINP	0.01184	0
B112	B212	FT4	IOP-L	0.023905	0
B113	B137	FT3	AFU	0.025027	0
B113	B160	FT3	PH	-0.03034	0
B113	B140	FT3	r-GT	0.045041	0
B113	B116	FT3	TT3	0.00798	3.23E-12
B113	B143	FT3	β-CTx	0.016132	0
B113	B126	FT3	Alb	0.060354	0
B113	B127	FT3	A-G ratio	0.021817	0
B113	B161	FT3	LEU	-0.0113	0
B113	B128	FT3	ALT	0.067008	0
B113	B123	FT3	hs-CRP1	-0.00864	4.66E-14
B113	B124	FT3	ACTH	0.009186	1.11E-15
B113	B134	FT3	CHE	0.039874	0
B113	B166	FT3	Pro	0.024891	0
B113	B188	FT3	Ca	0.021535	0
B113	B189	FT3	eGFB	0.036524	0
B113	B141	FT3	AST/ALT	-0.06346	0
B113	B145	FT3	BGP	0.018556	0
B113	B146	FT3	CysC	0.011272	0
B113	B181	FT3	APTT	-0.00717	3.80E-10
B113	B190	FT3	Cre	0.024214	0
B113	B129	FT3	IBIL	-0.00789	5.58E-12
B113	B150	FT3	ALP	0.034982	0
B113	B222	FT3	NS	0.067587	0
B113	B148	FT3	Anti_TPO	0.025762	0
B113	B149	FT3	TGAb	0.016269	0
B113	B198	FT3	P	0.00605	1.28E-07
B113	B194	FT3	Cl	-0.01603	0
B113	B195	FT3	Na	0.015977	0
B113	B176	FT3	Ucr	0.029288	0
B113	B191	FT3	Urea	0.013373	0
B113	B192	FT3	UA	0.06916	0
B113	B138	FT3	PAB	0.015225	0
B113	B221	FT3	Hei	0.061932	0

B113	B200	FT3	OP	0.013381	0
B113	B220	FT3	SBP	0.049233	0
B113	B219	FT3	OT	-0.00877	1.84E-14
B113	B218	FT3	DBP	0.049017	0
B113	B216	FT3	BMI	0.060971	0
B113	B215	FT3	BMI	0.036898	0
B113	B131	FT3	AST	0.039435	0
B113	B210	FT3	Hip	0.042137	0
B113	B209	FT3	Smoking	0.036192	0
B113	B139	FT3	ADA	0.010447	0
B113	B183	FT3	PTA	-0.00987	0
B113	B186	FT3	PFC	0.009477	0
B113	B173	FT3	NTT	-0.00843	1.81E-13
B113	B207	FT3	WHR	0.006201	6.17E-08
B113	B206	FT3	Waist	0.055377	0
B113	B205	FT3	KDA	-0.00656	1.03E-08
B113	B197	FT3	AG	0.015931	0
B113	B204	FT3	Drinking	0.037958	0
B113	B203	FT3	DH	0.006819	2.63E-09
B113	B132	FT3	Dbil	0.010858	0
B113	B117	FT3	TT4	0.005668	7.47E-07
B113	B114	FT3	TSH TT3 TT4(TT4)	0.284554	0
B113	B115	FT3	TSH TT3 TT4(TT3)	0.289797	0
B113	B144	FT3	Total-PINP	0.016922	0
B114	B136	TSH TT3 TT4(TT4)	5'NT	0.006893	1.76E-09
B114	B137	TSH TT3 TT4(TT4)	AFU	0.005711	6.16E-07
B114	B160	TSH TT3 TT4(TT4)	PH	-0.00732	1.65E-10
B114	B140	TSH TT3 TT4(TT4)	r-GT	-0.01465	0
B114	B143	TSH TT3 TT4(TT4)	β-CTx	0.005616	9.45E-07
B114	B127	TSH TT3 TT4(TT4)	A-G ratio	-0.01758	0
B114	B161	TSH TT3 TT4(TT4)	LEU	0.010046	0
B114	B128	TSH TT3 TT4(TT4)	ALT	0.009947	0
B114	B119	TSH TT3 TT4(TT4)	Hcv-rna	0.007783	1.08E-11
B114	B123	TSH TT3 TT4(TT4)	hs-CRP1	0.033665	0
B114	B189	TSH TT3 TT4(TT4)	eGFB	0.013817	0
B114	B141	TSH TT3 TT4(TT4)	AST/ALT	0.006367	2.72E-08
B114	B145	TSH TT3 TT4(TT4)	BGP	0.006877	1.92E-09
B114	B181	TSH TT3 TT4(TT4)	APTT	0.006647	6.52E-09
B114	B190	TSH TT3 TT4(TT4)	Cre	-0.02782	0
B114	B150	TSH TT3 TT4(TT4)	ALP	0.027691	0
B114	B222	TSH TT3 TT4(TT4)	NS	-0.03431	0
B114	B151	TSH TT3 TT4(TT4)	Anti-CCP	0.007749	1.33E-11
B114	B148	TSH TT3 TT4(TT4)	Anti_TPO	0.015698	0
B114	B198	TSH TT3 TT4(TT4)	P	0.017899	0

B114	B194	TSH TT3 TT4(TT4)	Cl	-0.01198	0
B114	B199	TSH TT3 TT4(TT4)	Mg	-0.01019	0
B114	B157	TSH TT3 TT4(TT4)	IgG	0.010137	0
B114	B195	TSH TT3 TT4(TT4)	Na	0.013731	0
B114	B191	TSH TT3 TT4(TT4)	Urea	-0.01407	0
B114	B174	TSH TT3 TT4(TT4)	Urea/Cr	0.01307	0
B114	B192	TSH TT3 TT4(TT4)	UA	-0.0267	0
B114	B170	TSH TT3 TT4(TT4)	U-GLU	0.006274	4.30E-08
B114	B182	TSH TT3 TT4(TT4)	TT	-0.00733	1.55E-10
B114	B125	TSH TT3 TT4(TT4)	Cor	0.01055	0
B114	B138	TSH TT3 TT4(TT4)	PAB	-0.06434	0
B114	B130	TSH TT3 TT4(TT4)	GLB	0.025453	0
B114	B221	TSH TT3 TT4(TT4)	Hei	-0.02368	0
B114	B200	TSH TT3 TT4(TT4)	OP	0.006326	3.34E-08
B114	B216	TSH TT3 TT4(TT4)	BMI	-0.03647	0
B114	B215	TSH TT3 TT4(TT4)	BMI	-0.03163	0
B114	B131	TSH TT3 TT4(TT4)	AST	0.016094	0
B114	B172	TSH TT3 TT4(TT4)	KB	0.012024	0
B114	B210	TSH TT3 TT4(TT4)	Hip	-0.02286	0
B114	B139	TSH TT3 TT4(TT4)	ADA	0.048708	0
B114	B185	TSH TT3 TT4(TT4)	PT	0.012444	0
B114	B186	TSH TT3 TT4(TT4)	PFC	0.041179	0
B114	B206	TSH TT3 TT4(TT4)	Waist	-0.03002	0
B114	B197	TSH TT3 TT4(TT4)	AG	0.012921	0
B114	B204	TSH TT3 TT4(TT4)	Drinking	-0.03478	0
B114	B211	TSH TT3 TT4(TT4)	IOP-R	-0.00708	6.44E-10
B114	B201	TSH TT3 TT4(TT4)	e-habits	-0.00691	1.60E-09
B114	B132	TSH TT3 TT4(TT4)	Dbil	0.014309	0
B114	B135	TSH TT3 TT4(TT4)	TBA	0.007335	1.52E-10
B114	B115	TSH TT3 TT4(TT4)	TSH TT3 TT4(TT3)	0.287072	0
B114	B212	TSH TT3 TT4(TT4)	IOP-L	-0.00669	5.17E-09
B115	B137	TSH TT3 TT4(TT3)	AFU	0.023587	0
B115	B160	TSH TT3 TT4(TT3)	PH	-0.00972	0
B115	B140	TSH TT3 TT4(TT3)	r-GT	0.013206	0
B115	B126	TSH TT3 TT4(TT3)	Alb	0.019396	0
B115	B128	TSH TT3 TT4(TT3)	ALT	0.028135	0
B115	B124	TSH TT3 TT4(TT3)	ACTH	0.006834	2.42E-09
B115	B134	TSH TT3 TT4(TT3)	CHE	0.036208	0
B115	B166	TSH TT3 TT4(TT3)	Pro	0.007134	4.70E-10
B115	B188	TSH TT3 TT4(TT3)	Ca	0.009319	4.44E-16
B115	B189	TSH TT3 TT4(TT3)	eGFB	0.01181	0
B115	B141	TSH TT3 TT4(TT3)	AST/ALT	-0.02305	0
B115	B146	TSH TT3 TT4(TT3)	CysC	0.007701	1.78E-11
B115	B129	TSH TT3 TT4(TT3)	IBIL	-0.00601	1.55E-07

B115	B150	TSH TT3 TT4(TT3)	ALP	0.026335	0
B115	B222	TSH TT3 TT4(TT3)	NS	0.021737	0
B115	B148	TSH TT3 TT4(TT3)	Anti_TPO	0.008556	8.04E-14
B115	B194	TSH TT3 TT4(TT3)	Cl	-0.00692	1.50E-09
B115	B195	TSH TT3 TT4(TT3)	Na	0.012707	0
B115	B192	TSH TT3 TT4(TT3)	UA	0.019707	0
B115	B182	TSH TT3 TT4(TT3)	TT	-0.00616	7.45E-08
B115	B221	TSH TT3 TT4(TT3)	Hei	0.015576	0
B115	B200	TSH TT3 TT4(TT3)	OP	0.011125	0
B115	B220	TSH TT3 TT4(TT3)	SBP	0.017474	0
B115	B218	TSH TT3 TT4(TT3)	DBP	0.017796	0
B115	B216	TSH TT3 TT4(TT3)	BMI	0.021331	0
B115	B215	TSH TT3 TT4(TT3)	BMI	0.017463	0
B115	B131	TSH TT3 TT4(TT3)	AST	0.018775	0
B115	B210	TSH TT3 TT4(TT3)	Hip	0.01743	0
B115	B209	TSH TT3 TT4(TT3)	Smoking	0.015248	0
B115	B139	TSH TT3 TT4(TT3)	ADA	0.01792	0
B115	B183	TSH TT3 TT4(TT3)	PTA	-0.00697	1.14E-09
B115	B186	TSH TT3 TT4(TT3)	PFC	0.011657	0
B115	B206	TSH TT3 TT4(TT3)	Waist	0.024623	0
B115	B197	TSH TT3 TT4(TT3)	AG	0.010033	0
B115	B204	TSH TT3 TT4(TT3)	Drinking	0.00704	7.92E-10
B116	B189	TT3	eGFB	0.006853	2.19E-09
B116	B170	TT3	U-GLU	0.012643	0
B116	B117	TT3	TT4	0.773598	0
B117	B137	TT4	AFU	-0.00623	5.42E-08
B117	B190	TT4	Cre	-0.00566	7.71E-07
B117	B170	TT4	U-GLU	0.009446	0
B118	B140	CEA1	r-GT	0.018073	0
B118	B126	CEA1	Alb	-0.01669	0
B118	B127	CEA1	A-G ratio	-0.00607	1.18E-07
B118	B128	CEA1	ALT	0.005881	2.83E-07
B118	B123	CEA1	hs-CRP1	0.030245	0
B118	B166	CEA1	Pro	0.00688	1.90E-09
B118	B189	CEA1	eGFB	-0.03024	0
B118	B146	CEA1	CysC	0.00633	3.26E-08
B118	B190	CEA1	Cre	0.022024	0
B118	B150	CEA1	ALP	0.009823	0
B118	B222	CEA1	NS	0.01557	0
B118	B176	CEA1	Ucr	0.00616	7.52E-08
B118	B178	CEA1	Ig-U	0.005874	2.92E-07
B118	B191	CEA1	Urea	0.011301	0
B118	B174	CEA1	Urea/Cr	-0.00668	5.49E-09
B118	B192	CEA1	UA	0.015489	0

B118	B170	CEA1	U-GLU	0.010053	0
B118	B179	CEA1	α1-MG	0.01058	0
B118	B220	CEA1	SBP	0.02435	0
B118	B218	CEA1	DBP	0.014015	0
B118	B216	CEA1	BMI	0.007085	6.20E-10
B118	B215	CEA1	BMI	0.005894	2.67E-07
B118	B131	CEA1	AST	0.013164	0
B118	B209	CEA1	Smoking	0.039733	0
B118	B173	CEA1	NTT	0.011011	0
B118	B206	CEA1	Waist	0.01713	0
B118	B204	CEA1	Drinking	0.017926	0
B118	B132	CEA1	Dbil	0.007835	7.90E-12
B119	B120	Hcv-rna	HCV-cAg	0.249861	0
B119	B124	Hcv-rna	ACTH	0.011413	0
B119	B125	Hcv-rna	Cor	0.018851	0
B119	B138	Hcv-rna	PAB	-0.00665	6.49E-09
B119	B139	Hcv-rna	ADA	0.0063	3.79E-08
B119	B196	Hcv-rna	TCO2	0.00573	5.66E-07
B119	B135	Hcv-rna	TBA	0.007173	3.78E-10
B12	B137	PG II	AFU	0.012343	0
B12	B24	PG II	αHBDH	0.01839	0
B12	B15	PG II	CD3-CD19+(B cell)	-0.02806	0
B12	B23	PG II	CD3-CD16+CD56+(NK)	-0.00619	6.40E-08
B12	B18	PG II	CD3+CD4+helper(inducer)T	0.012764	0
B12	B19	PG II	CD3+CD4+/CD3+CD8+	0.017442	0
B12	B22	PG II	CD3+CD8+(T)	-0.01069	0
B12	B92	PG II	ALDH2-Glu504Glu	-0.00967	0
B12	B93	PG II	ALDH2-Glu504Lys	0.00749	6.19E-11
B12	B160	PG II	PH	-0.02213	0
B12	B94	PG II	ALDH2-Lys504Lys	0.007276	2.12E-10
B12	B126	PG II	Alb	-0.03523	0
B12	B127	PG II	A-G ratio	-0.0222	0
B12	B40	PG II	WBC	0.015499	0
B12	B124	PG II	ACTH	0.010602	0
B12	B70	PG II	LDL-C	0.027713	0
B12	B72	PG II	HDL-C	0.009807	0
B12	B189	PG II	eGFB	-0.07519	0
B12	B141	PG II	AST/ALT	0.00632	3.43E-08
B12	B146	PG II	CysC	0.012098	0
B12	B43	PG II	MCV	0.010421	0
B12	B44	PG II	MCH	0.010982	0
B12	B45	PG II	MCHC	-0.01339	0
B12	B46	PG II	RBC Count	-0.0079	5.38E-12
B12	B47	PG II	RDW	0.037949	0

B12	B181	PG II	APTT	-0.0157	0
B12	B190	PG II	Cre	0.032402	0
B12	B193	PG II	K	0.008434	1.79E-13
B12	B129	PG II	IBIL	-0.02508	0
B12	B150	PG II	ALP	0.017194	0
B12	B84	PG II	C-PSA	0.007528	4.96E-11
B12	B109	PG II	AMA-M2	0.010273	0
B12	B199	PG II	Mg	0.006445	1.83E-08
B12	B195	PG II	Na	0.013478	0
B12	B191	PG II	Urea	0.028705	0
B12	B125	PG II	Cor	0.012155	0
B12	B130	PG II	GLB	0.014081	0
B12	B27	PG II	LDH	0.013505	0
B12	B221	PG II	Hei	-0.00704	7.98E-10
B12	B200	PG II	OP	0.013043	0
B12	B220	PG II	SBP	0.024386	0
B12	B219	PG II	OT	0.009089	2.22E-15
B12	B218	PG II	DBP	0.018258	0
B12	B131	PG II	AST	0.016569	0
B12	B210	PG II	Hip	-0.00694	1.34E-09
B12	B14	PG II	G-17	0.265469	0
B12	B209	PG II	Smoking	0.033967	0
B12	B90	PG II	CYFRA 21-1	0.022581	0
B12	B139	PG II	ADA	0.010214	0
B12	B196	PG II	TCO2	0.006708	4.74E-09
B12	B183	PG II	PTA	-0.02402	0
B12	B185	PG II	PT	-0.00876	2.07E-14
B12	B186	PG II	PFC	0.012034	0
B12	B52	PG II	PLT/BPC	-0.01915	0
B12	B173	PG II	NTT	0.00574	5.41E-07
B12	B206	PG II	Waist	0.007856	6.96E-12
B12	B205	PG II	KDA	0.005832	3.55E-07
B12	B78	PG II	HBcAb	-0.02834	0
B12	B204	PG II	Drinking	0.012233	0
B12	B80	PG II	anti-HP IgG	0.154105	0
B12	B112	PG II	FT4	0.005975	1.82E-07
B12	B113	PG II	FT3	0.013427	0
B12	B211	PG II	IOP-R	-0.00752	5.16E-11
B12	B201	PG II	e-habits	0.013568	0
B12	B74	PG II	ApoA1	0.020425	0
B12	B76	PG II	ApoB	0.007776	1.13E-11
B12	B132	PG II	Dbil	-0.0114	0
B12	B77	PG II	TC	0.023133	0
B12	B133	PG II	TBIL	-0.02321	0

B12	B135	PG II	TBA	0.006083	1.09E-07
B12	B91	PG II	TP	-0.00982	0
B12	B114	PG II	TSH TT3 TT4(TT4)	0.008243	6.16E-13
B12	B212	PG II	IOP-L	-0.00601	1.53E-07
B120	B140	HCV-cAg	r-GT	0.007076	6.49E-10
B120	B128	HCV-cAg	ALT	0.006804	2.84E-09
B120	B225	HCV-cAg	hPBG	0.008109	1.45E-12
B120	B194	HCV-cAg	Cl	-0.00583	3.60E-07
B120	B130	HCV-cAg	GLB	0.006159	7.57E-08
B120	B131	HCV-cAg	AST	0.007539	4.65E-11
B120	B139	HCV-cAg	ADA	0.006177	6.95E-08
B121	B137	C3	AFU	-0.01337	0
B121	B122	C3	C4	0.68854	0
B121	B124	C3	ACTH	-0.00811	1.43E-12
B121	B134	C3	CHE	0.005955	2.00E-07
B121	B150	C3	ALP	0.006218	5.69E-08
B121	B194	C3	Cl	-0.00808	1.78E-12
B121	B155	C3	IgA	0.014171	0
B121	B157	C3	IgG	-0.006	1.63E-07
B121	B195	C3	Na	-0.01125	0
B121	B125	C3	Cor	-0.00621	6.04E-08
B121	B130	C3	GLB	0.007293	1.93E-10
B121	B200	C3	OP	-0.00849	1.23E-13
B121	B215	C3	BMI	0.006331	3.26E-08
B121	B196	C3	TCO2	-0.00869	3.29E-14
B121	B186	C3	PFC	0.006443	1.85E-08
B121	B206	C3	Waist	0.006079	1.11E-07
B121	B212	C3	IOP-L	0.005631	8.83E-07
B122	B137	C4	AFU	-0.0128	0
B122	B134	C4	CHE	0.006546	1.10E-08
B122	B193	C4	K	-0.00574	5.44E-07
B122	B194	C4	Cl	-0.00688	1.93E-09
B122	B155	C4	IgA	0.015937	0
B122	B156	C4	IgE	-0.00675	3.71E-09
B122	B157	C4	IgG	-0.00693	1.43E-09
B122	B195	C4	Na	-0.00842	2.01E-13
B122	B138	C4	PAB	0.006754	3.72E-09
B122	B200	C4	OP	-0.00782	8.86E-12
B123	B160	hs-CRP1	PH	-0.01379	0
B123	B140	hs-CRP1	r-GT	0.051523	0
B123	B126	hs-CRP1	Alb	-0.04619	0
B123	B127	hs-CRP1	A-G ratio	-0.04504	0
B123	B128	hs-CRP1	ALT	0.022421	0
B123	B124	hs-CRP1	ACTH	0.008831	1.24E-14

B123	B165	hs-CRP1	bilirubin	0.011869	0
B123	B166	hs-CRP1	Pro	0.013669	0
B123	B189	hs-CRP1	eGFB	-0.0115	0
B123	B141	hs-CRP1	AST/ALT	-0.00895	5.77E-15
B123	B146	hs-CRP1	CysC	0.006959	1.23E-09
B123	B181	hs-CRP1	APTT	0.013436	0
B123	B190	hs-CRP1	Cre	0.014177	0
B123	B129	hs-CRP1	IBIL	-0.00936	4.44E-16
B123	B150	hs-CRP1	ALP	0.032254	0
B123	B222	hs-CRP1	NS	0.02482	0
B123	B151	hs-CRP1	Anti-CCP	0.03879	0
B123	B154	hs-CRP1	RF	0.022476	0
B123	B194	hs-CRP1	Cl	-0.0153	0
B123	B155	hs-CRP1	IgA	0.020773	0
B123	B156	hs-CRP1	IgE	0.010865	0
B123	B157	hs-CRP1	IgG	0.011621	0
B123	B176	hs-CRP1	Ucr	0.024929	0
B123	B178	hs-CRP1	Ig-U	0.053154	0
B123	B174	hs-CRP1	Urea/Cr	-0.00909	2.22E-15
B123	B192	hs-CRP1	UA	0.023347	0
B123	B170	hs-CRP1	U-GLU	0.019548	0
B123	B175	hs-CRP1	ALB-U	0.033953	0
B123	B177	hs-CRP1	ACR	0.036719	0
B123	B179	hs-CRP1	α1-MG	0.076544	0
B123	B180	hs-CRP1	Tf	0.018177	0
B123	B182	hs-CRP1	TT	-0.0217	0
B123	B125	hs-CRP1	Cor	0.010643	0
B123	B138	hs-CRP1	PAB	-0.03025	0
B123	B130	hs-CRP1	GLB	0.04401	0
B123	B220	hs-CRP1	SBP	0.01975	0
B123	B218	hs-CRP1	DBP	0.017932	0
B123	B216	hs-CRP1	BMI	0.025322	0
B123	B215	hs-CRP1	BMI	0.031161	0
B123	B131	hs-CRP1	AST	0.02339	0
B123	B172	hs-CRP1	KB	0.01162	0
B123	B210	hs-CRP1	Hip	0.022239	0
B123	B209	hs-CRP1	Smoking	0.02213	0
B123	B139	hs-CRP1	ADA	0.034717	0
B123	B183	hs-CRP1	PTA	-0.01017	0
B123	B185	hs-CRP1	PT	0.009813	0
B123	B186	hs-CRP1	PFC	0.099981	0
B123	B173	hs-CRP1	NTT	0.007088	6.10E-10
B123	B206	hs-CRP1	Waist	0.036601	0
B123	B197	hs-CRP1	AG	0.011258	0

B123	B204	hs-CRP1	Drinking	0.013449	0
B123	B211	hs-CRP1	IOP-R	-0.00647	1.61E-08
B123	B132	hs-CRP1	Dbil	0.007214	3.01E-10
B123	B133	hs-CRP1	TBIL	-0.00564	8.49E-07
B123	B212	hs-CRP1	IOP-L	-0.00655	1.05E-08
B124	B137	ACTH	AFU	0.017003	0
B124	B160	ACTH	PH	-0.00573	5.64E-07
B124	B140	ACTH	r-GT	0.009224	8.88E-16
B124	B126	ACTH	Alb	0.006196	6.31E-08
B124	B128	ACTH	ALT	0.006318	3.47E-08
B124	B225	ACTH	hPBG	-0.00585	3.26E-07
B124	B134	ACTH	CHE	0.025506	0
B124	B146	ACTH	CysC	0.007282	2.05E-10
B124	B181	ACTH	APTT	-0.03804	0
B124	B190	ACTH	Cre	0.014427	0
B124	B129	ACTH	IBIL	0.007012	9.27E-10
B124	B150	ACTH	ALP	0.013387	0
B124	B222	ACTH	NS	0.013112	0
B124	B151	ACTH	Anti-CCP	0.008806	1.51E-14
B124	B194	ACTH	Cl	-0.01824	0
B124	B155	ACTH	IgA	0.014279	0
B124	B156	ACTH	IgE	0.014203	0
B124	B195	ACTH	Na	0.010973	0
B124	B159	ACTH	β2-MG	-0.01434	0
B124	B176	ACTH	Ucr	0.006027	1.43E-07
B124	B174	ACTH	Urea/Cr	-0.0117	0
B124	B192	ACTH	UA	0.01299	0
B124	B179	ACTH	α1-MG	0.013657	0
B124	B182	ACTH	TT	-0.01072	0
B124	B125	ACTH	Cor	0.374521	0
B124	B138	ACTH	PAB	0.041309	0
B124	B221	ACTH	Hei	0.010561	0
B124	B200	ACTH	OP	0.01459	0
B124	B220	ACTH	SBP	0.010108	0
B124	B218	ACTH	DBP	0.010611	0
B124	B216	ACTH	BMI	0.011081	0
B124	B215	ACTH	BMI	0.006982	1.09E-09
B124	B131	ACTH	AST	0.006621	7.43E-09
B124	B210	ACTH	Hip	0.007361	1.31E-10
B124	B209	ACTH	Smoking	0.006541	1.12E-08
B124	B184	ACTH	INR	0.013469	0
B124	B186	ACTH	PFC	0.020591	0
B124	B206	ACTH	Waist	0.00998	0
B124	B197	ACTH	AG	0.013681	0

B124	B204	ACTH	Drinking	0.009504	0
B124	B133	ACTH	TBIL	0.006238	5.15E-08
B124	B135	ACTH	TBA	0.017439	0
B125	B140	Cor	r-GT	0.006722	4.40E-09
B125	B126	Cor	Alb	0.009166	1.33E-15
B125	B127	Cor	A-G ratio	0.005707	6.28E-07
B125	B225	Cor	hPBG	-0.01225	0
B125	B134	Cor	CHE	0.010343	0
B125	B181	Cor	APTT	-0.04929	0
B125	B190	Cor	Cre	0.012807	0
B125	B193	Cor	K	-0.01684	0
B125	B129	Cor	IBIL	0.006404	2.26E-08
B125	B150	Cor	ALP	0.00693	1.45E-09
B125	B151	Cor	Anti-CCP	0.01151	0
B125	B198	Cor	P	0.007489	6.22E-11
B125	B194	Cor	Cl	-0.03926	0
B125	B156	Cor	IgE	0.015964	0
B125	B157	Cor	IgG	0.012758	0
B125	B158	Cor	IgM	-0.00614	8.23E-08
B125	B195	Cor	Na	-0.02948	0
B125	B176	Cor	Ucr	0.007507	5.59E-11
B125	B174	Cor	Urea/Cr	-0.01311	0
B125	B179	Cor	α1-MG	0.019224	0
B125	B182	Cor	TT	-0.05586	0
B125	B138	Cor	PAB	0.033026	0
B125	B221	Cor	Hei	0.008272	5.12E-13
B125	B200	Cor	OP	-0.02224	0
B125	B218	Cor	DBP	0.006602	8.22E-09
B125	B215	Cor	BMI	-0.00611	9.61E-08
B125	B139	Cor	ADA	-0.0093	4.44E-16
B125	B196	Cor	TCO2	0.011283	0
B125	B183	Cor	PTA	-0.01186	0
B125	B185	Cor	PT	0.015761	0
B125	B186	Cor	PFC	0.030921	0
B125	B211	Cor	IOP-R	0.006131	8.67E-08
B125	B135	Cor	TBA	0.029553	0
B126	B160	Alb	PH	-0.03543	0
B126	B140	Alb	r-GT	0.044529	0
B126	B127	Alb	A-G ratio	0.421267	0
B126	B161	Alb	LEU	-0.03806	0
B126	B128	Alb	ALT	0.102731	0
B126	B165	Alb	bilirubin	-0.00597	1.87E-07
B126	B134	Alb	CHE	0.0596	0
B126	B166	Alb	Pro	0.05246	0

B126	B188	Alb	Ca	0.109588	0
B126	B189	Alb	eGFB	0.170044	0
B126	B141	Alb	AST/ALT	-0.09975	0
B126	B146	Alb	CysC	-0.00731	1.73E-10
B126	B190	Alb	Cre	0.108723	0
B126	B224	Alb	pmh	-0.0062	6.21E-08
B126	B193	Alb	K	0.016481	0
B126	B129	Alb	IBIL	0.142249	0
B126	B150	Alb	ALP	0.00816	1.05E-12
B126	B213	Alb	CVA-R	-0.00786	6.77E-12
B126	B214	Alb	CVA-L	-0.0073	1.86E-10
B126	B222	Alb	NS	0.104667	0
B126	B151	Alb	Anti-CCP	-0.00588	2.86E-07
B126	B148	Alb	Anti_TPO	-0.01375	0
B126	B149	Alb	TGAb	-0.01091	0
B126	B154	Alb	RF	-0.01162	0
B126	B194	Alb	Cl	-0.05381	0
B126	B199	Alb	Mg	0.026255	0
B126	B155	Alb	IgA	-0.01149	0
B126	B157	Alb	IgG	-0.0135	0
B126	B176	Alb	Ucr	0.037605	0
B126	B191	Alb	Urea	0.017946	0
B126	B174	Alb	Urea/Cr	-0.06306	0
B126	B192	Alb	UA	0.180228	0
B126	B175	Alb	ALB-U	-0.01337	0
B126	B177	Alb	ACR	-0.01653	0
B126	B180	Alb	Tf	-0.00603	1.39E-07
B126	B138	Alb	PAB	0.076175	0
B126	B130	Alb	GLB	-0.10615	0
B126	B221	Alb	Hei	0.203513	0
B126	B187	Alb	NSE	0.006291	3.97E-08
B126	B200	Alb	OP	0.008653	4.20E-14
B126	B227	Alb	OD	-0.01228	0
B126	B228	Alb	OS	-0.01074	0
B126	B220	Alb	SBP	0.031853	0
B126	B219	Alb	OT	-0.05745	0
B126	B218	Alb	DBP	0.085428	0
B126	B216	Alb	BMI	0.087028	0
B126	B215	Alb	BMI	-0.03157	0
B126	B131	Alb	AST	0.03819	0
B126	B172	Alb	KB	0.031786	0
B126	B210	Alb	Hip	0.027914	0
B126	B209	Alb	Smoking	-0.03691	0
B126	B139	Alb	ADA	-0.01979	0

B126	B196	Alb	TCO2	0.006095	1.03E-07
B126	B186	Alb	PFC	-0.00852	1.01E-13
B126	B173	Alb	NTT	-0.03523	0
B126	B206	Alb	Waist	-0.0078	9.66E-12
B126	B205	Alb	KDA	-0.02335	0
B126	B197	Alb	AG	0.032192	0
B126	B204	Alb	Drinking	0.055872	0
B126	B203	Alb	DH	0.051259	0
B126	B211	Alb	IOP-R	0.0448	0
B126	B132	Alb	Dbil	0.102279	0
B126	B133	Alb	TBIL	0.14143	0
B126	B144	Alb	Total-PINP	-0.00579	4.37E-07
B126	B212	Alb	IOP-L	0.038469	0
B127	B137	A-G ratio	AFU	-0.00842	1.99E-13
B127	B160	A-G ratio	PH	0.013108	0
B127	B140	A-G ratio	r-GT	-0.02387	0
B127	B161	A-G ratio	LEU	-0.04541	0
B127	B128	A-G ratio	ALT	0.006905	1.66E-09
B127	B225	A-G ratio	hPBG	-0.00843	1.85E-13
B127	B166	A-G ratio	Pro	0.037186	0
B127	B188	A-G ratio	Ca	0.041843	0
B127	B189	A-G ratio	eGFB	0.061216	0
B127	B146	A-G ratio	CysC	-0.0079	5.43E-12
B127	B190	A-G ratio	Cre	0.108312	0
B127	B193	A-G ratio	K	-0.01163	0
B127	B129	A-G ratio	IBIL	0.126698	0
B127	B150	A-G ratio	ALP	-0.02053	0
B127	B222	A-G ratio	NS	0.079501	0
B127	B148	A-G ratio	Anti_TPO	-0.03134	0
B127	B149	A-G ratio	TGAb	-0.02857	0
B127	B154	A-G ratio	RF	-0.01001	0
B127	B198	A-G ratio	P	-0.00978	0
B127	B194	A-G ratio	Cl	0.009647	0
B127	B199	A-G ratio	Mg	0.006884	1.86E-09
B127	B155	A-G ratio	IgA	-0.06381	0
B127	B156	A-G ratio	IgE	-0.01008	0
B127	B157	A-G ratio	IgG	-0.09351	0
B127	B158	A-G ratio	IgM	-0.0148	0
B127	B195	A-G ratio	Na	0.014324	0
B127	B159	A-G ratio	β2-MG	-0.01241	0
B127	B176	A-G ratio	Ucr	0.031929	0
B127	B178	A-G ratio	Ig-U	-0.01067	0
B127	B191	A-G ratio	Urea	0.02214	0
B127	B174	A-G ratio	Urea/Cr	-0.061	0

B127	B192	A-G ratio	UA	0.055539	0
B127	B170	A-G ratio	U-GLU	-0.0138	0
B127	B175	A-G ratio	ALB-U	-0.01229	0
B127	B177	A-G ratio	ACR	-0.01589	0
B127	B180	A-G ratio	Tf	-0.00683	2.49E-09
B127	B182	A-G ratio	TT	-0.00634	3.08E-08
B127	B153	A-G ratio	Glu	-0.00916	1.33E-15
B127	B138	A-G ratio	PAB	0.036416	0
B127	B130	A-G ratio	GLB	-0.82057	0
B127	B221	A-G ratio	Hei	0.166903	0
B127	B200	A-G ratio	OP	0.011948	0
B127	B227	A-G ratio	OD	-0.00638	2.50E-08
B127	B220	A-G ratio	SBP	-0.0479	0
B127	B219	A-G ratio	OT	-0.03425	0
B127	B218	A-G ratio	DBP	-0.02345	0
B127	B216	A-G ratio	BMI	0.052086	0
B127	B215	A-G ratio	BMI	-0.04869	0
B127	B131	A-G ratio	AST	-0.03805	0
B127	B172	A-G ratio	KB	0.019526	0
B127	B209	A-G ratio	Smoking	0.092975	0
B127	B139	A-G ratio	ADA	-0.05136	0
B127	B196	A-G ratio	TCO2	0.005715	6.04E-07
B127	B185	A-G ratio	PT	-0.00618	6.73E-08
B127	B186	A-G ratio	PFC	-0.01467	0
B127	B173	A-G ratio	NTT	-0.03182	0
B127	B206	A-G ratio	Waist	-0.01028	0
B127	B205	A-G ratio	KDA	-0.01693	0
B127	B204	A-G ratio	Drinking	0.086184	0
B127	B203	A-G ratio	DH	0.01433	0
B127	B211	A-G ratio	IOP-R	0.016402	0
B127	B132	A-G ratio	Dbil	0.124045	0
B127	B133	A-G ratio	TBIL	0.133679	0
B127	B212	A-G ratio	IOP-L	0.016316	0
B128	B136	ALT	5'NT	0.010901	0
B128	B137	ALT	AFU	0.045256	0
B128	B160	ALT	PH	-0.05802	0
B128	B140	ALT	r-GT	0.403771	0
B128	B161	ALT	LEU	-0.04439	0
B128	B165	ALT	bilirubin	0.068247	0
B128	B134	ALT	CHE	0.050038	0
B128	B166	ALT	Pro	0.069432	0
B128	B188	ALT	Ca	0.021208	0
B128	B189	ALT	eGFB	-0.02295	0
B128	B141	ALT	AST/ALT	-0.41492	0

B128	B146	ALT	CysC	0.015532	0
B128	B181	ALT	APTT	-0.00619	6.65E-08
B128	B190	ALT	Cre	0.1729	0
B128	B129	ALT	IBIL	0.047954	0
B128	B150	ALT	ALP	0.070974	0
B128	B222	ALT	NS	0.302839	0
B128	B148	ALT	Anti_TPO	-0.017	0
B128	B149	ALT	TGAb	-0.01728	0
B128	B198	ALT	P	-0.01343	0
B128	B194	ALT	Cl	-0.00681	2.78E-09
B128	B155	ALT	IgA	0.006064	1.20E-07
B128	B157	ALT	IgG	-0.00738	1.14E-10
B128	B176	ALT	Ucr	0.047489	0
B128	B191	ALT	Urea	0.052947	0
B128	B174	ALT	Urea/Cr	-0.07812	0
B128	B192	ALT	UA	0.288708	0
B128	B170	ALT	U-GLU	0.0347	0
B128	B175	ALT	ALB-U	0.008114	1.40E-12
B128	B179	ALT	α1-MG	0.019834	0
B128	B153	ALT	Glu	0.016857	0
B128	B138	ALT	PAB	0.027712	0
B128	B130	ALT	GLB	0.040043	0
B128	B221	ALT	Hei	0.189621	0
B128	B200	ALT	OP	0.008465	1.47E-13
B128	B220	ALT	SBP	0.132324	0
B128	B219	ALT	OT	-0.01103	0
B128	B218	ALT	DBP	0.146832	0
B128	B216	ALT	BMI	0.338011	0
B128	B215	ALT	BMI	0.309396	0
B128	B131	ALT	AST	0.788494	0
B128	B172	ALT	KB	-0.01448	0
B128	B210	ALT	Hip	0.252878	0
B128	B209	ALT	Smoking	0.086672	0
B128	B139	ALT	ADA	0.043261	0
B128	B173	ALT	NTT	-0.01925	0
B128	B207	ALT	WHR	0.034004	0
B128	B206	ALT	Waist	0.322838	0
B128	B205	ALT	KDA	-0.01606	0
B128	B197	ALT	AG	0.007127	4.91E-10
B128	B204	ALT	Drinking	0.120024	0
B128	B203	ALT	DH	0.019637	0
B128	B211	ALT	IOP-R	0.016359	0
B128	B201	ALT	e-habits	-0.0166	0
B128	B132	ALT	Dbil	0.10045	0

B128	B133	ALT	TBIL	0.065192	0
B128	B135	ALT	TBA	0.034998	0
B128	B144	ALT	Total-PINP	0.00635	2.96E-08
B128	B212	ALT	IOP-L	0.015706	0
B129	B160	IBIL	PH	0.051233	0
B129	B140	IBIL	r-GT	0.030824	0
B129	B161	IBIL	LEU	-0.03448	0
B129	B225	IBIL	hPBG	0.006686	5.31E-09
B129	B165	IBIL	bilirubin	0.020857	0
B129	B166	IBIL	Pro	0.062183	0
B129	B188	IBIL	Ca	0.015112	0
B129	B189	IBIL	eGFB	-0.07786	0
B129	B141	IBIL	AST/ALT	-0.00769	1.87E-11
B129	B181	IBIL	APTT	0.006109	9.65E-08
B129	B190	IBIL	Cre	0.133212	0
B129	B193	IBIL	K	-0.02122	0
B129	B150	IBIL	ALP	-0.00946	0
B129	B222	IBIL	NS	0.081814	0
B129	B151	IBIL	Anti-CCP	-0.00681	2.83E-09
B129	B148	IBIL	Anti_TPO	-0.00894	5.77E-15
B129	B149	IBIL	TGAb	-0.00791	4.94E-12
B129	B198	IBIL	P	-0.02684	0
B129	B194	IBIL	Cl	-0.00836	2.99E-13
B129	B195	IBIL	Na	-0.00819	8.70E-13
B129	B176	IBIL	Ucr	0.039589	0
B129	B191	IBIL	Urea	-0.01121	0
B129	B174	IBIL	Urea/Cr	-0.10202	0
B129	B192	IBIL	UA	0.108605	0
B129	B170	IBIL	U-GLU	0.008552	8.26E-14
B129	B179	IBIL	α1-MG	0.011436	0
B129	B180	IBIL	Tf	0.005883	2.81E-07
B129	B182	IBIL	TT	-0.00579	4.26E-07
B129	B138	IBIL	PAB	0.017346	0
B129	B130	IBIL	GLB	-0.09676	0
B129	B221	IBIL	Hei	0.113724	0
B129	B200	IBIL	OP	-0.00813	1.26E-12
B129	B220	IBIL	SBP	0.054186	0
B129	B218	IBIL	DBP	0.052176	0
B129	B216	IBIL	BMI	0.058772	0
B129	B131	IBIL	AST	0.0704	0
B129	B172	IBIL	KB	0.029041	0
B129	B210	IBIL	Hip	0.010654	0
B129	B209	IBIL	Smoking	-0.02844	0
B129	B196	IBIL	TCO2	0.008696	3.15E-14

B129	B183	IBIL	PTA	0.02915	0
B129	B185	IBIL	PT	0.013682	0
B129	B186	IBIL	PFC	-0.01496	0
B129	B173	IBIL	NTT	-0.01453	0
B129	B207	IBIL	WHR	0.005799	4.13E-07
B129	B206	IBIL	Waist	0.028439	0
B129	B205	IBIL	KDA	-0.01547	0
B129	B197	IBIL	AG	-0.00615	7.75E-08
B129	B204	IBIL	Drinking	0.047248	0
B129	B203	IBIL	DH	-0.02709	0
B129	B211	IBIL	IOP-R	0.020043	0
B129	B132	IBIL	Dbil	0.664802	0
B129	B133	IBIL	TBIL	0.97992	0
B129	B212	IBIL	IOP-L	0.020037	0
B130	B137	GLB	AFU	0.013476	0
B130	B160	GLB	PH	-0.03737	0
B130	B140	GLB	r-GT	0.056979	0
B130	B143	GLB	β-CTx	-0.00611	9.51E-08
B130	B161	GLB	LEU	0.039482	0
B130	B225	GLB	hPBG	0.011356	0
B130	B134	GLB	CHE	0.021598	0
B130	B166	GLB	Pro	-0.01855	0
B130	B188	GLB	Ca	0.027329	0
B130	B189	GLB	eGFB	-0.01114	0
B130	B141	GLB	AST/ALT	0.01312	0
B130	B146	GLB	CysC	0.013201	0
B130	B190	GLB	Cre	-0.08038	0
B130	B193	GLB	K	0.022528	0
B130	B150	GLB	ALP	0.035468	0
B130	B222	GLB	NS	-0.04728	0
B130	B151	GLB	Anti-CCP	0.005779	4.53E-07
B130	B148	GLB	Anti_TPO	0.033603	0
B130	B149	GLB	TGAb	0.031525	0
B130	B154	GLB	RF	0.008777	1.82E-14
B130	B198	GLB	P	0.01117	0
B130	B194	GLB	Cl	-0.03576	0
B130	B155	GLB	IgA	0.076371	0
B130	B156	GLB	IgE	0.010498	0
B130	B157	GLB	IgG	0.114451	0
B130	B158	GLB	IgM	0.019315	0
B130	B195	GLB	Na	-0.01643	0
B130	B176	GLB	Ucr	-0.02393	0
B130	B178	GLB	Ig-U	0.012704	0
B130	B191	GLB	Urea	-0.01586	0

B130	B174	GLB	Urea/Cr	0.046853	0
B130	B192	GLB	UA	0.014458	0
B130	B170	GLB	U-GLU	0.021983	0
B130	B175	GLB	ALB-U	0.012537	0
B130	B177	GLB	ACR	0.015425	0
B130	B180	GLB	Tf	0.006545	1.10E-08
B130	B182	GLB	TT	0.008607	5.68E-14
B130	B153	GLB	Glu	0.01467	0
B130	B138	GLB	PAB	-0.01364	0
B130	B221	GLB	Hei	-0.1159	0
B130	B187	GLB	NSE	0.007339	1.48E-10
B130	B200	GLB	OP	-0.0113	0
B130	B220	GLB	SBP	0.080538	0
B130	B219	GLB	OT	0.018481	0
B130	B218	GLB	DBP	0.070546	0
B130	B216	GLB	BMI	-0.02408	0
B130	B215	GLB	BMI	0.049003	0
B130	B131	GLB	AST	0.070442	0
B130	B172	GLB	KB	-0.00751	5.41E-11
B130	B210	GLB	Hip	0.01748	0
B130	B209	GLB	Smoking	-0.1221	0
B130	B139	GLB	ADA	0.058957	0
B130	B152	GLB	ACA-Ig(G-A-M)	0.008081	1.73E-12
B130	B185	GLB	PT	0.007247	2.49E-10
B130	B186	GLB	PFC	0.015718	0
B130	B173	GLB	NTT	0.025781	0
B130	B206	GLB	Waist	0.014334	0
B130	B205	GLB	KDA	0.011307	0
B130	B197	GLB	AG	0.01796	0
B130	B204	GLB	Drinking	-0.07799	0
B130	B132	GLB	Dbil	-0.09701	0
B130	B133	GLB	TBIL	-0.10351	0
B131	B136	AST	5'NT	0.009387	2.22E-16
B131	B137	AST	AFU	0.044275	0
B131	B160	AST	PH	-0.0366	0
B131	B140	AST	r-GT	0.357884	0
B131	B161	AST	LEU	-0.0206	0
B131	B165	AST	bilirubin	0.104042	0
B131	B134	AST	CHE	0.026954	0
B131	B166	AST	Pro	0.044375	0
B131	B188	AST	Ca	0.013735	0
B131	B189	AST	eGFB	-0.05863	0
B131	B141	AST	AST/ALT	-0.11653	0
B131	B146	AST	CysC	0.015066	0

B131	B190	AST	Cre	0.093817	0
B131	B224	AST	pmh	0.006039	1.35E-07
B131	B150	AST	ALP	0.063964	0
B131	B222	AST	NS	0.168209	0
B131	B148	AST	Anti_TPO	-0.00772	1.57E-11
B131	B149	AST	TGAb	-0.00838	2.53E-13
B131	B198	AST	P	-0.00815	1.12E-12
B131	B155	AST	IgA	0.009449	0
B131	B157	AST	IgG	0.008003	2.80E-12
B131	B176	AST	Ucr	0.025619	0
B131	B191	AST	Urea	0.032396	0
B131	B174	AST	Urea/Cr	-0.03974	0
B131	B192	AST	UA	0.179085	0
B131	B170	AST	U-GLU	0.018832	0
B131	B175	AST	ALB-U	0.007334	1.53E-10
B131	B179	AST	α1-MG	0.026263	0
B131	B153	AST	Glu	0.011646	0
B131	B138	AST	PAB	0.009596	0
B131	B221	AST	Hei	0.071748	0
B131	B220	AST	SBP	0.119283	0
B131	B219	AST	OT	0.006146	8.05E-08
B131	B218	AST	DBP	0.11835	0
B131	B216	AST	BMI	0.174074	0
B131	B215	AST	BMI	0.178589	0
B131	B210	AST	Hip	0.129439	0
B131	B209	AST	Smoking	0.06461	0
B131	B139	AST	ADA	0.060173	0
B131	B207	AST	WHR	0.022351	0
B131	B206	AST	Waist	0.190595	0
B131	B205	AST	KDA	-0.00751	5.43E-11
B131	B204	AST	Drinking	0.099106	0
B131	B211	AST	IOP-R	0.008878	9.10E-15
B131	B132	AST	Dbil	0.136886	0
B131	B133	AST	TBIL	0.092784	0
B131	B135	AST	TBA	0.040404	0
B131	B144	AST	Total-PINP	0.00676	3.60E-09
B131	B212	AST	IOP-L	0.010308	0
B132	B142	Dbil	25-(OH)D	0.005812	3.89E-07
B132	B160	Dbil	PH	0.0441	0
B132	B140	Dbil	r-GT	0.078165	0
B132	B161	Dbil	LEU	-0.02185	0
B132	B225	Dbil	hPBG	0.008258	5.60E-13
B132	B165	Dbil	bilirubin	0.163582	0
B132	B134	Dbil	CHE	-0.02466	0

B132	B166	Dbil	Pro	0.048541	0
B132	B188	Dbil	Ca	0.009162	1.33E-15
B132	B189	Dbil	eGFB	-0.03356	0
B132	B141	Dbil	AST/ALT	-0.00667	5.66E-09
B132	B181	Dbil	APTT	0.01019	0
B132	B190	Dbil	Cre	0.086911	0
B132	B193	Dbil	K	-0.0216	0
B132	B150	Dbil	ALP	0.019648	0
B132	B222	Dbil	NS	0.017748	0
B132	B148	Dbil	Anti_TPO	-0.00616	7.41E-08
B132	B198	Dbil	P	-0.0249	0
B132	B194	Dbil	Cl	-0.01587	0
B132	B155	Dbil	IgA	0.006187	6.62E-08
B132	B176	Dbil	Ucr	0.020863	0
B132	B191	Dbil	Urea	-0.01709	0
B132	B174	Dbil	Urea/Cr	-0.07553	0
B132	B192	Dbil	UA	0.041594	0
B132	B170	Dbil	U-GLU	0.009846	0
B132	B179	Dbil	α1-MG	0.013585	0
B132	B182	Dbil	TT	0.006222	5.57E-08
B132	B153	Dbil	Glu	-0.00661	7.73E-09
B132	B221	Dbil	Hei	0.081533	0
B132	B220	Dbil	SBP	0.02017	0
B132	B219	Dbil	OT	-0.00989	0
B132	B216	Dbil	BMI	-0.01003	0
B132	B215	Dbil	BMI	-0.07053	0
B132	B172	Dbil	KB	0.045898	0
B132	B210	Dbil	Hip	-0.03615	0
B132	B209	Dbil	Smoking	-0.03141	0
B132	B139	Dbil	ADA	0.02171	0
B132	B196	Dbil	TCO2	0.006779	3.25E-09
B132	B185	Dbil	PT	0.016105	0
B132	B186	Dbil	PFC	-0.01276	0
B132	B173	Dbil	NTT	-0.00763	2.74E-11
B132	B206	Dbil	Waist	-0.03988	0
B132	B205	Dbil	KDA	-0.00912	1.78E-15
B132	B197	Dbil	AG	0.007783	1.09E-11
B132	B204	Dbil	Drinking	0.041136	0
B132	B203	Dbil	DH	-0.00867	3.64E-14
B132	B201	Dbil	e-habits	0.02369	0
B132	B133	Dbil	TBIL	0.797354	0
B132	B135	Dbil	TBA	0.049985	0
B132	B212	Dbil	IOP-L	-0.00572	6.04E-07
B133	B160	TBIL	PH	0.0529	0

B133	B140	TBIL	r-GT	0.045513	0
B133	B161	TBIL	LEU	-0.03353	0
B133	B225	TBIL	hPBG	0.007568	3.92E-11
B133	B165	TBIL	bilirubin	0.059846	0
B133	B134	TBIL	CHE	-0.0063	3.86E-08
B133	B166	TBIL	Pro	0.062817	0
B133	B188	TBIL	Ca	0.013987	0
B133	B189	TBIL	eGFB	-0.07151	0
B133	B141	TBIL	AST/ALT	-0.00813	1.27E-12
B133	B181	TBIL	APTT	0.007618	2.92E-11
B133	B190	TBIL	Cre	0.130135	0
B133	B193	TBIL	K	-0.02267	0
B133	B222	TBIL	NS	0.070614	0
B133	B151	TBIL	Anti-CCP	-0.00672	4.47E-09
B133	B148	TBIL	Anti_TPO	-0.00881	1.40E-14
B133	B149	TBIL	TGAb	-0.00766	2.33E-11
B133	B198	TBIL	P	-0.02821	0
B133	B194	TBIL	Cl	-0.01093	0
B133	B176	TBIL	Ucr	0.037389	0
B133	B191	TBIL	Urea	-0.01354	0
B133	B174	TBIL	Urea/Cr	-0.10204	0
B133	B192	TBIL	UA	0.098431	0
B133	B170	TBIL	U-GLU	0.009488	0
B133	B179	TBIL	α1-MG	0.012806	0
B133	B180	TBIL	Tf	0.005762	4.89E-07
B133	B138	TBIL	PAB	0.013142	0
B133	B221	TBIL	Hei	0.113077	0
B133	B200	TBIL	OP	-0.00619	6.64E-08
B133	B220	TBIL	SBP	0.049086	0
B133	B219	TBIL	OT	-0.00664	6.72E-09
B133	B218	TBIL	DBP	0.043096	0
B133	B216	TBIL	BMI	0.044803	0
B133	B215	TBIL	BMI	-0.01987	0
B133	B172	TBIL	KB	0.035454	0
B133	B209	TBIL	Smoking	-0.03117	0
B133	B139	TBIL	ADA	0.007849	7.23E-12
B133	B196	TBIL	TCO2	0.008829	1.29E-14
B133	B183	TBIL	PTA	0.024842	0
B133	B185	TBIL	PT	0.015273	0
B133	B186	TBIL	PFC	-0.0154	0
B133	B173	TBIL	NTT	-0.0137	0
B133	B206	TBIL	Waist	0.012514	0
B133	B205	TBIL	KDA	-0.01497	0
B133	B204	TBIL	Drinking	0.048859	0

B133	B203	TBIL	DH	-0.02411	0
B133	B211	TBIL	IOP-R	0.014794	0
B133	B201	TBIL	e-habits	0.010167	0
B133	B135	TBIL	TBA	0.015337	0
B133	B212	TBIL	IOP-L	0.014662	0
B134	B136	CHE	5'NT	0.02695	0
B134	B137	CHE	AFU	0.149811	0
B134	B160	CHE	PH	-0.02021	0
B134	B140	CHE	r-GT	0.058452	0
B134	B161	CHE	LEU	-0.00767	2.18E-11
B134	B166	CHE	Pro	0.01154	0
B134	B188	CHE	Ca	0.012583	0
B134	B189	CHE	eGFB	-0.01496	0
B134	B141	CHE	AST/ALT	-0.05272	0
B134	B146	CHE	CysC	0.020437	0
B134	B181	CHE	APTT	-0.05503	0
B134	B190	CHE	Cre	0.046635	0
B134	B193	CHE	K	0.029672	0
B134	B150	CHE	ALP	0.144197	0
B134	B222	CHE	NS	0.0845	0
B134	B148	CHE	Anti_TPO	-0.00939	2.22E-16
B134	B149	CHE	TGAb	-0.01321	0
B134	B198	CHE	P	-0.04383	0
B134	B194	CHE	Cl	-0.09625	0
B134	B199	CHE	Mg	0.067279	0
B134	B155	CHE	IgA	0.014363	0
B134	B157	CHE	IgG	-0.02899	0
B134	B158	CHE	IgM	-0.01191	0
B134	B195	CHE	Na	0.072364	0
B134	B176	CHE	Ucr	0.017039	0
B134	B178	CHE	Ig-U	0.007737	1.43E-11
B134	B191	CHE	Urea	0.021847	0
B134	B174	CHE	Urea/Cr	-0.01577	0
B134	B192	CHE	UA	0.081926	0
B134	B170	CHE	U-GLU	0.012457	0
B134	B179	CHE	α1-MG	0.015399	0
B134	B180	CHE	Tf	0.011231	0
B134	B182	CHE	TT	0.024748	0
B134	B153	CHE	Glu	0.006437	1.92E-08
B134	B138	CHE	PAB	0.362062	0
B134	B221	CHE	Hei	0.047744	0
B134	B187	CHE	NSE	0.006603	8.19E-09
B134	B200	CHE	OP	0.097999	0
B134	B220	CHE	SBP	0.042008	0

B134	B218	CHE	DBP	0.05273	0
B134	B216	CHE	BMI	0.087652	0
B134	B215	CHE	BMI	0.082421	0
B134	B172	CHE	KB	-0.00706	7.13E-10
B134	B210	CHE	Hip	0.063961	0
B134	B209	CHE	Smoking	0.030482	0
B134	B139	CHE	ADA	-0.0507	0
B134	B196	CHE	TCO2	0.011728	0
B134	B183	CHE	PTA	-0.00712	5.16E-10
B134	B184	CHE	INR	0.006751	3.77E-09
B134	B185	CHE	PT	-0.03595	0
B134	B186	CHE	PFC	0.020694	0
B134	B207	CHE	WHR	0.009607	0
B134	B206	CHE	Waist	0.088144	0
B134	B197	CHE	AG	0.095804	0
B134	B204	CHE	Drinking	0.04913	0
B134	B203	CHE	DH	0.009168	1.33E-15
B134	B211	CHE	IOP-R	0.01937	0
B134	B135	CHE	TBA	0.014631	0
B134	B212	CHE	IOP-L	0.018614	0
B135	B136	TBA	5'NT	0.025828	0
B135	B137	TBA	AFU	0.089927	0
B135	B160	TBA	PH	-0.0072	3.21E-10
B135	B140	TBA	r-GT	0.053419	0
B135	B165	TBA	bilirubin	0.076696	0
B135	B141	TBA	AST/ALT	-0.00787	6.33E-12
B135	B146	TBA	CysC	0.010812	0
B135	B190	TBA	Cre	0.0087	3.06E-14
B135	B193	TBA	K	-0.02572	0
B135	B150	TBA	ALP	0.108109	0
B135	B222	TBA	NS	0.012913	0
B135	B198	TBA	P	0.007491	6.15E-11
B135	B199	TBA	Mg	0.006843	2.31E-09
B135	B155	TBA	IgA	0.009906	0
B135	B157	TBA	IgG	0.00717	3.86E-10
B135	B195	TBA	Na	0.01441	0
B135	B191	TBA	Urea	0.010688	0
B135	B192	TBA	UA	0.006981	1.10E-09
B135	B170	TBA	U-GLU	0.006035	1.38E-07
B135	B182	TBA	TT	0.008163	1.03E-12
B135	B138	TBA	PAB	-0.00612	9.35E-08
B135	B221	TBA	Hei	0.008202	8.03E-13
B135	B200	TBA	OP	0.02406	0
B135	B220	TBA	SBP	0.010554	0

B135	B218	TBA	DBP	0.010479	0
B135	B216	TBA	BMI	0.010388	0
B135	B215	TBA	BMI	0.00833	3.52E-13
B135	B210	TBA	Hip	0.00564	8.50E-07
B135	B209	TBA	Smoking	0.014983	0
B135	B139	TBA	ADA	0.074533	0
B135	B206	TBA	Waist	0.013703	0
B135	B197	TBA	AG	0.007562	4.06E-11
B135	B204	TBA	Drinking	0.011468	0
B136	B137	5'NT	AFU	0.010218	0
B136	B140	5'NT	r-GT	0.031498	0
B136	B141	5'NT	AST/ALT	-0.00642	2.07E-08
B136	B193	5'NT	K	0.012107	0
B136	B150	5'NT	ALP	0.049173	0
B136	B222	5'NT	NS	0.005978	1.80E-07
B136	B198	5'NT	P	0.011103	0
B136	B194	5'NT	Cl	-0.00648	1.54E-08
B136	B155	5'NT	IgA	0.006096	1.02E-07
B136	B157	5'NT	IgG	-0.01507	0
B136	B138	5'NT	PAB	0.03092	0
B136	B216	5'NT	BMI	0.007224	2.85E-10
B136	B215	5'NT	BMI	0.006523	1.24E-08
B136	B210	5'NT	Hip	0.006468	1.63E-08
B136	B139	5'NT	ADA	0.017509	0
B136	B186	5'NT	PFC	0.005963	1.93E-07
B136	B206	5'NT	Waist	0.006954	1.27E-09
B136	B197	5'NT	AG	0.008114	1.40E-12
B136	B203	5'NT	DH	0.008749	2.20E-14
B137	B160	AFU	PH	-0.00732	1.69E-10
B137	B140	AFU	r-GT	0.050797	0
B137	B165	AFU	bilirubin	0.008322	3.71E-13
B137	B166	AFU	Pro	0.006467	1.64E-08
B137	B189	AFU	eGFB	-0.00918	1.11E-15
B137	B141	AFU	AST/ALT	-0.01979	0
B137	B146	AFU	CysC	0.016412	0
B137	B181	AFU	APTT	-0.02492	0
B137	B190	AFU	Cre	0.015452	0
B137	B193	AFU	K	-0.01634	0
B137	B150	AFU	ALP	0.090536	0
B137	B222	AFU	NS	0.034146	0
B137	B154	AFU	RF	0.010763	0
B137	B198	AFU	P	-0.05866	0
B137	B194	AFU	Cl	-0.03172	0
B137	B155	AFU	IgA	0.018274	0

B137	B156	AFU	IgE	0.008977	4.44E-15
B137	B157	AFU	IgG	-0.00788	5.83E-12
B137	B158	AFU	IgM	-0.00797	3.35E-12
B137	B195	AFU	Na	0.014802	0
B137	B176	AFU	Ucr	0.008176	9.45E-13
B137	B178	AFU	Ig-U	0.014501	0
B137	B191	AFU	Urea	0.006913	1.58E-09
B137	B174	AFU	Urea/Cr	-0.00734	1.46E-10
B137	B192	AFU	UA	0.028656	0
B137	B170	AFU	U-GLU	0.016133	0
B137	B179	AFU	α1-MG	0.025094	0
B137	B180	AFU	Tf	0.015975	0
B137	B182	AFU	TT	0.010393	0
B137	B153	AFU	Glu	0.006078	1.12E-07
B137	B138	AFU	PAB	0.133229	0
B137	B221	AFU	Hei	0.015779	0
B137	B200	AFU	OP	0.053294	0
B137	B220	AFU	SBP	0.029306	0
B137	B218	AFU	DBP	0.030718	0
B137	B216	AFU	BMI	0.031735	0
B137	B215	AFU	BMI	0.031007	0
B137	B210	AFU	Hip	0.022506	0
B137	B209	AFU	Smoking	0.025122	0
B137	B139	AFU	ADA	0.145506	0
B137	B196	AFU	TCO2	0.030939	0
B137	B183	AFU	PTA	0.014871	0
B137	B185	AFU	PT	-0.0133	0
B137	B206	AFU	Waist	0.037415	0
B137	B204	AFU	Drinking	0.031454	0
B137	B211	AFU	IOP-R	0.00722	2.91E-10
B137	B201	AFU	e-habits	-0.00586	3.08E-07
B137	B212	AFU	IOP-L	0.008842	1.15E-14
B138	B160	PAB	PH	-0.01787	0
B138	B140	PAB	r-GT	0.069815	0
B138	B161	PAB	LEU	-0.0139	0
B138	B166	PAB	Pro	0.014008	0
B138	B188	PAB	Ca	0.014025	0
B138	B189	PAB	eGFB	-0.01084	0
B138	B141	PAB	AST/ALT	-0.04159	0
B138	B145	PAB	BGP	-0.00723	2.75E-10
B138	B146	PAB	CysC	0.013909	0
B138	B181	PAB	APTT	-0.08202	0
B138	B190	PAB	Cre	0.070309	0
B138	B224	PAB	pmh	-0.00604	1.35E-07

B138	B193	PAB	K	0.019093	0
B138	B150	PAB	ALP	0.016477	0
B138	B222	PAB	NS	0.082934	0
B138	B151	PAB	Anti-CCP	-0.00857	7.13E-14
B138	B148	PAB	Anti_TPO	-0.01752	0
B138	B149	PAB	TGAb	-0.01812	0
B138	B198	PAB	P	-0.09002	0
B138	B194	PAB	Cl	-0.15743	0
B138	B199	PAB	Mg	0.110038	0
B138	B156	PAB	IgE	0.009053	2.66E-15
B138	B157	PAB	IgG	-0.07199	0
B138	B158	PAB	IgM	-0.0257	0
B138	B195	PAB	Na	0.08734	0
B138	B168	PAB	NMP22	-0.00649	1.43E-08
B138	B176	PAB	Ucr	0.031664	0
B138	B191	PAB	Urea	0.026375	0
B138	B174	PAB	Urea/Cr	-0.03228	0
B138	B192	PAB	UA	0.097115	0
B138	B179	PAB	α1-MG	0.022028	0
B138	B182	PAB	TT	0.023182	0
B138	B221	PAB	Hei	0.073079	0
B138	B187	PAB	NSE	0.009311	4.44E-16
B138	B200	PAB	OP	0.117531	0
B138	B220	PAB	SBP	0.027547	0
B138	B219	PAB	OT	-0.01088	0
B138	B218	PAB	DBP	0.048837	0
B138	B216	PAB	BMI	0.075728	0
B138	B215	PAB	BMI	0.048488	0
B138	B172	PAB	KB	-0.00666	6.12E-09
B138	B210	PAB	Hip	0.043556	0
B138	B209	PAB	Smoking	0.045671	0
B138	B139	PAB	ADA	-0.29099	0
B138	B196	PAB	TCO2	0.020621	0
B138	B183	PAB	PTA	-0.01304	0
B138	B185	PAB	PT	-0.06469	0
B138	B186	PAB	PFC	-0.0241	0
B138	B173	PAB	NTT	-0.01031	0
B138	B207	PAB	WHR	0.007558	4.15E-11
B138	B206	PAB	Waist	0.066945	0
B138	B197	PAB	AG	0.137579	0
B138	B204	PAB	Drinking	0.092287	0
B138	B203	PAB	DH	0.007254	2.41E-10
B138	B211	PAB	IOP-R	0.023701	0
B138	B144	PAB	Total-PINP	-0.00859	6.31E-14

B138	B212	PAB	IOP-L	0.022233	0
B139	B140	ADA	r-GT	0.017748	0
B139	B161	ADA	LEU	0.009713	0
B139	B165	ADA	bilirubin	0.008428	1.87E-13
B139	B189	ADA	eGFB	-0.0123	0
B139	B146	ADA	CysC	0.023131	0
B139	B181	ADA	APTT	0.031234	0
B139	B190	ADA	Cre	-0.01636	0
B139	B193	ADA	K	0.025985	0
B139	B150	ADA	ALP	0.164595	0
B139	B222	ADA	NS	-0.02197	0
B139	B148	ADA	Anti_TPO	0.012095	0
B139	B149	ADA	TGAb	0.013202	0
B139	B154	ADA	RF	0.01828	0
B139	B198	ADA	P	0.018544	0
B139	B194	ADA	Cl	-0.03394	0
B139	B199	ADA	Mg	-0.02925	0
B139	B155	ADA	IgA	0.054775	0
B139	B156	ADA	IgE	0.01108	0
B139	B157	ADA	IgG	0.087996	0
B139	B158	ADA	IgM	0.015894	0
B139	B195	ADA	Na	0.020693	0
B139	B176	ADA	Ucr	-0.00917	1.33E-15
B139	B178	ADA	Ig-U	0.023151	0
B139	B174	ADA	Urea/Cr	0.013711	0
B139	B192	ADA	UA	-0.01383	0
B139	B170	ADA	U-GLU	0.025602	0
B139	B175	ADA	ALB-U	0.007032	8.30E-10
B139	B177	ADA	ACR	0.012757	0
B139	B179	ADA	α1-MG	0.027884	0
B139	B180	ADA	Tf	0.027825	0
B139	B182	ADA	TT	0.020287	0
B139	B153	ADA	Glu	0.006449	1.80E-08
B139	B221	ADA	Hei	-0.0304	0
B139	B200	ADA	OP	0.049715	0
B139	B220	ADA	SBP	0.020284	0
B139	B219	ADA	OT	0.006831	2.46E-09
B139	B216	ADA	BMI	-0.01869	0
B139	B210	ADA	Hip	-0.0074	1.03E-10
B139	B209	ADA	Smoking	-0.01716	0
B139	B196	ADA	TCO2	-0.00854	9.02E-14
B139	B183	ADA	PTA	-0.00598	1.82E-07
B139	B185	ADA	PT	0.042457	0
B139	B186	ADA	PFC	0.024961	0

B139	B173	ADA	NTT	0.011944	0
B139	B206	ADA	Waist	-0.00711	5.40E-10
B139	B197	ADA	AG	0.040968	0
B139	B204	ADA	Drinking	-0.03202	0
B139	B211	ADA	IOP-R	-0.01059	0
B139	B201	ADA	e-habits	0.005907	2.51E-07
B139	B144	ADA	Total-PINP	0.006749	3.82E-09
B139	B212	ADA	IOP-L	-0.00988	0
B14	B24	G-17	αHBDH	0.007151	4.30E-10
B14	B19	G-17	CD3+CD4+/CD3+CD8+	0.006522	1.24E-08
B14	B54	G-17	ESR	0.006257	4.70E-08
B14	B126	G-17	Alb	-0.01402	0
B14	B127	G-17	A-G ratio	-0.01433	0
B14	B40	G-17	WBC	0.009536	0
B14	B111	G-17	TSH	0.007176	3.73E-10
B14	B134	G-17	CHE	-0.00581	3.96E-07
B14	B189	G-17	eGFB	-0.01668	0
B14	B45	G-17	MCHC	-0.01208	0
B14	B46	G-17	RBC Count	-0.01296	0
B14	B47	G-17	RDW	0.017796	0
B14	B129	G-17	IBIL	-0.00876	2.09E-14
B14	B150	G-17	ALP	0.005781	4.49E-07
B14	B148	G-17	Anti_TPO	0.023423	0
B14	B149	G-17	TGAb	0.01026	0
B14	B176	G-17	Ucr	-0.00631	3.69E-08
B14	B191	G-17	Urea	0.0081	1.53E-12
B14	B174	G-17	Urea/Cr	0.010122	0
B14	B192	G-17	UA	-0.00913	1.55E-15
B14	B138	G-17	PAB	-0.01073	0
B14	B130	G-17	GLB	0.012606	0
B14	B27	G-17	LDH	0.006635	6.94E-09
B14	B221	G-17	Hei	-0.01257	0
B14	B220	G-17	SBP	0.014123	0
B14	B218	G-17	DBP	0.009722	0
B14	B88	G-17	CA 242	0.006951	1.29E-09
B14	B216	G-17	BMI	-0.00568	7.02E-07
B14	B131	G-17	AST	0.007068	6.81E-10
B14	B90	G-17	CYFRA 21-1	0.009008	3.55E-15
B14	B139	G-17	ADA	0.015703	0
B14	B51	G-17	Hb	-0.0145	0
B14	B78	G-17	HBcAb	-0.00698	1.13E-09
B14	B80	G-17	anti-HP IgG	0.056382	0
B14	B133	G-17	TBIL	-0.00777	1.17E-11
B140	B160	r-GT	PH	-0.07804	0

B140	B161	r-GT	LEU	-0.03105	0
B140	B165	r-GT	bilirubin	0.078428	0
B140	B166	r-GT	Pro	0.067957	0
B140	B188	r-GT	Ca	0.018036	0
B140	B189	r-GT	eGFB	-0.06086	0
B140	B141	r-GT	AST/ALT	-0.2095	0
B140	B146	r-GT	CysC	0.017799	0
B140	B181	r-GT	APTT	-0.01499	0
B140	B190	r-GT	Cre	0.13314	0
B140	B193	r-GT	K	-0.00893	6.22E-15
B140	B150	r-GT	ALP	0.110765	0
B140	B222	r-GT	NS	0.251348	0
B140	B148	r-GT	Anti_TPO	-0.01875	0
B140	B149	r-GT	TGAb	-0.02149	0
B140	B198	r-GT	P	-0.01403	0
B140	B194	r-GT	Cl	-0.01384	0
B140	B155	r-GT	IgA	0.00755	4.35E-11
B140	B156	r-GT	IgE	0.006251	4.83E-08
B140	B157	r-GT	IgG	-0.0148	0
B140	B176	r-GT	Ucr	0.055914	0
B140	B178	r-GT	Ig-U	0.008529	9.59E-14
B140	B191	r-GT	Urea	0.04127	0
B140	B174	r-GT	Urea/Cr	-0.06255	0
B140	B192	r-GT	UA	0.262809	0
B140	B170	r-GT	U-GLU	0.068512	0
B140	B175	r-GT	ALB-U	0.013542	0
B140	B179	r-GT	α1-MG	0.033932	0
B140	B180	r-GT	Tf	0.01017	0
B140	B153	r-GT	Glu	0.027869	0
B140	B221	r-GT	Hei	0.137382	0
B140	B200	r-GT	OP	0.008215	7.38E-13
B140	B220	r-GT	SBP	0.150156	0
B140	B218	r-GT	DBP	0.177007	0
B140	B216	r-GT	BMI	0.253544	0
B140	B215	r-GT	BMI	0.23702	0
B140	B210	r-GT	Hip	0.178681	0
B140	B209	r-GT	Smoking	0.163015	0
B140	B185	r-GT	PT	-0.00984	0
B140	B186	r-GT	PFC	0.005724	5.80E-07
B140	B173	r-GT	NTT	-0.01211	0
B140	B207	r-GT	WHR	0.031271	0
B140	B206	r-GT	Waist	0.273163	0
B140	B205	r-GT	KDA	-0.01168	0
B140	B197	r-GT	AG	0.012518	0

B140	B204	r-GT	Drinking	0.22511	0
B140	B203	r-GT	DH	0.01944	0
B140	B211	r-GT	IOP-R	0.02123	0
B140	B201	r-GT	e-habits	-0.01188	0
B140	B212	r-GT	IOP-L	0.021965	0
B141	B160	AST/ALT	PH	0.048732	0
B141	B161	AST/ALT	LEU	0.056484	0
B141	B166	AST/ALT	Pro	-0.05617	0
B141	B188	AST/ALT	Ca	-0.00799	3.08E-12
B141	B189	AST/ALT	eGFB	0.041857	0
B141	B146	AST/ALT	CysC	-0.01124	0
B141	B181	AST/ALT	APTT	0.009161	1.33E-15
B141	B190	AST/ALT	Cre	-0.20523	0
B141	B150	AST/ALT	ALP	-0.05068	0
B141	B222	AST/ALT	NS	-0.31421	0
B141	B148	AST/ALT	Anti_TPO	0.01992	0
B141	B149	AST/ALT	TGAb	0.022396	0
B141	B198	AST/ALT	P	0.019157	0
B141	B194	AST/ALT	Cl	0.008969	4.88E-15
B141	B157	AST/ALT	IgG	0.023044	0
B141	B158	AST/ALT	IgM	0.007718	1.60E-11
B141	B195	AST/ALT	Na	-0.01214	0
B141	B176	AST/ALT	Ucr	-0.04409	0
B141	B191	AST/ALT	Urea	-0.08091	0
B141	B174	AST/ALT	Urea/Cr	0.078044	0
B141	B192	AST/ALT	UA	-0.2688	0
B141	B170	AST/ALT	U-GLU	-0.0324	0
B141	B177	AST/ALT	ACR	0.006733	4.15E-09
B141	B179	AST/ALT	α1-MG	-0.01145	0
B141	B182	AST/ALT	TT	-0.00591	2.45E-07
B141	B153	AST/ALT	Glu	-0.01586	0
B141	B221	AST/ALT	Hei	-0.22555	0
B141	B200	AST/ALT	OP	-0.01412	0
B141	B220	AST/ALT	SBP	-0.1134	0
B141	B219	AST/ALT	OT	0.009352	4.44E-16
B141	B218	AST/ALT	DBP	-0.12414	0
B141	B216	AST/ALT	BMI	-0.34558	0
B141	B215	AST/ALT	BMI	-0.30114	0
B141	B172	AST/ALT	KB	0.031638	0
B141	B210	AST/ALT	Hip	-0.25943	0
B141	B209	AST/ALT	Smoking	-0.09784	0
B141	B185	AST/ALT	PT	0.008626	5.02E-14
B141	B173	AST/ALT	NTT	0.026302	0
B141	B207	AST/ALT	WHR	-0.03373	0

B141	B206	AST/ALT	Waist	-0.32588	0
B141	B205	AST/ALT	KDA	0.016425	0
B141	B197	AST/ALT	AG	-0.01205	0
B141	B204	AST/ALT	Drinking	-0.11385	0
B141	B203	AST/ALT	DH	-0.01988	0
B141	B211	AST/ALT	IOP-R	-0.01289	0
B141	B212	AST/ALT	IOP-L	-0.01148	0
B142	B143	25-(OH)D	β-CTx	0.014895	0
B142	B189	25-(OH)D	eGFB	-0.01711	0
B142	B145	25-(OH)D	BGP	-0.03482	0
B142	B222	25-(OH)D	NS	-0.00739	1.08E-10
B142	B151	25-(OH)D	Anti-CCP	0.013041	0
B142	B176	25-(OH)D	Ucr	-0.01842	0
B142	B182	25-(OH)D	TT	0.005933	2.22E-07
B142	B221	25-(OH)D	Hei	-0.00591	2.48E-07
B142	B228	25-(OH)D	OS	0.007436	8.48E-11
B142	B219	25-(OH)D	OT	0.006415	2.13E-08
B142	B216	25-(OH)D	BMI	-0.00967	0
B142	B215	25-(OH)D	BMI	-0.00926	6.66E-16
B142	B210	25-(OH)D	Hip	-0.00637	2.64E-08
B142	B183	25-(OH)D	PTA	-0.01259	0
B142	B206	25-(OH)D	Waist	-0.00762	2.83E-11
B142	B197	25-(OH)D	AG	0.005994	1.67E-07
B142	B201	25-(OH)D	e-habits	0.008182	9.11E-13
B143	B145	β-CTx	BGP	0.579579	0
B143	B190	β-CTx	Cre	0.006473	1.59E-08
B143	B150	β-CTx	ALP	0.026408	0
B143	B222	β-CTx	NS	0.006062	1.21E-07
B143	B198	β-CTx	P	0.009819	0
B143	B194	β-CTx	Cl	0.00577	4.71E-07
B143	B195	β-CTx	Na	0.005872	2.95E-07
B143	B168	β-CTx	NMP22	0.006052	1.27E-07
B143	B176	β-CTx	Ucr	0.027272	0
B143	B182	β-CTx	TT	-0.00724	2.63E-10
B143	B209	β-CTx	Smoking	0.008219	7.20E-13
B143	B183	β-CTx	PTA	0.022752	0
B143	B186	β-CTx	PFC	-0.00624	5.21E-08
B143	B144	β-CTx	Total-PINP	0.739751	0
B144	B145	Total-PINP	BGP	0.592844	0
B144	B150	Total-PINP	ALP	0.030371	0
B144	B148	Total-PINP	Anti_TPO	0.006645	6.56E-09
B144	B198	Total-PINP	P	0.008228	6.82E-13
B144	B194	Total-PINP	Cl	0.006717	4.51E-09
B144	B168	Total-PINP	NMP22	0.010044	0

B144	B176	Total-PINP	Ucr	0.010411	0
B144	B183	Total-PINP	PTA	0.014922	0
B145	B181	BGP	APTT	0.005779	4.52E-07
B145	B150	BGP	ALP	0.043605	0
B145	B148	BGP	Anti_TPO	0.006281	4.16E-08
B145	B198	BGP	P	0.00786	6.76E-12
B145	B194	BGP	Cl	0.008489	1.25E-13
B145	B168	BGP	NMP22	0.020256	0
B145	B182	BGP	TT	-0.01108	0
B145	B183	BGP	PTA	0.010919	0
B145	B185	BGP	PT	0.005713	6.12E-07
B146	B160	CysC	PH	-0.01925	0
B146	B189	CysC	eGFB	-0.07324	0
B146	B190	CysC	Cre	0.074655	0
B146	B193	CysC	K	0.006431	1.97E-08
B146	B150	CysC	ALP	0.023331	0
B146	B222	CysC	NS	0.03703	0
B146	B154	CysC	RF	0.008555	8.08E-14
B146	B198	CysC	P	-0.008	2.92E-12
B146	B194	CysC	Cl	0.007688	1.92E-11
B146	B199	CysC	Mg	0.015997	0
B146	B195	CysC	Na	0.024335	0
B146	B191	CysC	Urea	0.036488	0
B146	B174	CysC	Urea/Cr	-0.01965	0
B146	B192	CysC	UA	0.046767	0
B146	B177	CysC	ACR	0.006013	1.53E-07
B146	B179	CysC	α1-MG	0.008719	2.71E-14
B146	B221	CysC	Hei	0.019733	0
B146	B200	CysC	OP	0.030452	0
B146	B220	CysC	SBP	0.031054	0
B146	B219	CysC	OT	0.006756	3.66E-09
B146	B218	CysC	DBP	0.023135	0
B146	B216	CysC	BMI	0.029109	0
B146	B215	CysC	BMI	0.025486	0
B146	B210	CysC	Hip	0.022761	0
B146	B209	CysC	Smoking	0.031166	0
B146	B183	CysC	PTA	-0.00583	3.64E-07
B146	B206	CysC	Waist	0.037689	0
B146	B197	CysC	AG	0.00883	1.27E-14
B146	B204	CysC	Drinking	0.018672	0
B146	B201	CysC	e-habits	0.006621	7.44E-09
B148	B160	Anti_TPO	PH	0.007499	5.86E-11
B148	B161	Anti_TPO	LEU	0.011423	0
B148	B166	Anti_TPO	Pro	-0.01072	0

B148	B189	Anti_TPO	eGFB	0.011652	0
B148	B190	Anti_TPO	Cre	-0.04163	0
B148	B222	Anti_TPO	NS	-0.0401	0
B148	B149	Anti_TPO	TGAb	0.42356	0
B148	B198	Anti_TPO	P	0.006334	3.21E-08
B148	B157	Anti_TPO	IgG	0.013488	0
B148	B176	Anti_TPO	Ucr	-0.02058	0
B148	B191	Anti_TPO	Urea	-0.01075	0
B148	B174	Anti_TPO	Urea/Cr	0.02317	0
B148	B192	Anti_TPO	UA	-0.035	0
B148	B170	Anti_TPO	U-GLU	-0.0058	4.12E-07
B148	B221	Anti_TPO	Hei	-0.04204	0
B148	B220	Anti_TPO	SBP	-0.01193	0
B148	B218	Anti_TPO	DBP	-0.01553	0
B148	B216	Anti_TPO	BMI	-0.03562	0
B148	B215	Anti_TPO	BMI	-0.01727	0
B148	B210	Anti_TPO	Hip	-0.01795	0
B148	B209	Anti_TPO	Smoking	-0.02429	0
B148	B206	Anti_TPO	Waist	-0.02874	0
B148	B205	Anti_TPO	KDA	0.005814	3.86E-07
B148	B197	Anti_TPO	AG	-0.00563	8.71E-07
B148	B204	Anti_TPO	Drinking	-0.03068	0
B149	B161	TGAb	LEU	0.00847	1.42E-13
B149	B166	TGAb	Pro	-0.01034	0
B149	B189	TGAb	eGFB	0.009748	0
B149	B190	TGAb	Cre	-0.03485	0
B149	B222	TGAb	NS	-0.03983	0
B149	B154	TGAb	RF	0.006437	1.91E-08
B149	B198	TGAb	P	0.008183	9.03E-13
B149	B157	TGAb	IgG	0.015802	0
B149	B168	TGAb	NMP22	0.015794	0
B149	B176	TGAb	Ucr	-0.02114	0
B149	B191	TGAb	Urea	-0.011	0
B149	B174	TGAb	Urea/Cr	0.017386	0
B149	B192	TGAb	UA	-0.03396	0
B149	B169	TGAb	U-CRY	0.00561	9.71E-07
B149	B221	TGAb	Hei	-0.03706	0
B149	B220	TGAb	SBP	-0.01307	0
B149	B218	TGAb	DBP	-0.01475	0
B149	B216	TGAb	BMI	-0.03652	0
B149	B215	TGAb	BMI	-0.02243	0
B149	B210	TGAb	Hip	-0.02055	0
B149	B209	TGAb	Smoking	-0.02615	0
B149	B206	TGAb	Waist	-0.03288	0

B149	B204	TGAb	Drinking	-0.03017	0
B15	B142	CD3-CD19+(B cell)	25-(OH)D	0.005791	4.28E-07
B15	B137	CD3-CD19+(B cell)	AFU	0.02553	0
B15	B16	CD3-CD19+(B cell)	CD3+T	-0.07039	0
B15	B23	CD3-CD19+(B cell)	CD3-CD16+CD56+(NK)	-0.16096	0
B15	B17	CD3-CD19+(B cell)	CD3+CD16+CD56+NK	-0.08175	0
B15	B18	CD3-CD19+(B cell)	CD3+CD4+helper(inducer)T	0.093631	0
B15	B19	CD3-CD19+(B cell)	CD3+CD4+/CD3+CD8+	0.183395	0
B15	B20	CD3-CD19+(B cell)	CD3+CD4+CD8+	-0.03289	0
B15	B21	CD3-CD19+(B cell)	CD3+CD4-CD8-	-0.05047	0
B15	B22	CD3-CD19+(B cell)	CD3+CD8+(T)	-0.15853	0
B15	B54	CD3-CD19+(B cell)	ESR	0.021792	0
B15	B92	CD3-CD19+(B cell)	ALDH2-Glu504Glu	0.022409	0
B15	B93	CD3-CD19+(B cell)	ALDH2-Glu504Lys	-0.01587	0
B15	B140	CD3-CD19+(B cell)	r-GT	-0.00725	2.39E-10
B15	B94	CD3-CD19+(B cell)	ALDH2-Lys504Lys	-0.01907	0
B15	B40	CD3-CD19+(B cell)	WBC	0.019797	0
B15	B121	CD3-CD19+(B cell)	C3	-0.01537	0
B15	B122	CD3-CD19+(B cell)	C4	-0.00935	4.44E-16
B15	B225	CD3-CD19+(B cell)	hPBG	0.009512	0
B15	B124	CD3-CD19+(B cell)	ACTH	-0.00836	2.87E-13
B15	B70	CD3-CD19+(B cell)	LDL-C	-0.01334	0
B15	B141	CD3-CD19+(B cell)	AST/ALT	0.005685	6.94E-07
B15	B145	CD3-CD19+(B cell)	BGP	-0.01103	0
B15	B57	CD3-CD19+(B cell)	RBCAI	-0.0221	0
B15	B44	CD3-CD19+(B cell)	MCH	-0.00894	5.77E-15
B15	B47	CD3-CD19+(B cell)	RDW	-0.00725	2.44E-10
B15	B181	CD3-CD19+(B cell)	APTT	0.023854	0
B15	B26	CD3-CD19+(B cell)	CKMB	0.005858	3.14E-07
B15	B193	CD3-CD19+(B cell)	K	0.024172	0
B15	B222	CD3-CD19+(B cell)	NS	-0.01517	0
B15	B95	CD3-CD19+(B cell)	anti-cenpb	-0.01857	0
B15	B96	CD3-CD19+(B cell)	dsDNA (IIF)	0.026538	0
B15	B98	CD3-CD19+(B cell)	anti-Jo-1	-0.01246	0
B15	B100	CD3-CD19+(B cell)	anti-Ro52	0.030506	0
B15	B101	CD3-CD19+(B cell)	anti-Sc70	0.00824	6.31E-13
B15	B103	CD3-CD19+(B cell)	anti-SSA/Ro	0.024465	0
B15	B104	CD3-CD19+(B cell)	anti-SSB	0.017121	0
B15	B106	CD3-CD19+(B cell)	anti-P	0.00906	2.66E-15
B15	B107	CD3-CD19+(B cell)	AnuA	0.018422	0
B15	B151	CD3-CD19+(B cell)	Anti-CCP	-0.0139	0
B15	B149	CD3-CD19+(B cell)	TGAb	0.009658	0
B15	B109	CD3-CD19+(B cell)	AMA-M2	-0.01216	0
B15	B110	CD3-CD19+(B cell)	AHA	0.016209	0

B15	B194	CD3-CD19+(B cell)	Cl	0.007618	2.91E-11
B15	B199	CD3-CD19+(B cell)	Mg	-0.01441	0
B15	B155	CD3-CD19+(B cell)	IgA	0.022458	0
B15	B156	CD3-CD19+(B cell)	IgE	0.013964	0
B15	B157	CD3-CD19+(B cell)	IgG	0.034429	0
B15	B178	CD3-CD19+(B cell)	Ig-U	0.013164	0
B15	B175	CD3-CD19+(B cell)	ALB-U	0.007123	5.02E-10
B15	B177	CD3-CD19+(B cell)	ACR	0.008619	5.28E-14
B15	B179	CD3-CD19+(B cell)	α1-MG	0.012505	0
B15	B180	CD3-CD19+(B cell)	Tf	0.014645	0
B15	B182	CD3-CD19+(B cell)	TT	-0.01595	0
B15	B125	CD3-CD19+(B cell)	Cor	-0.05394	0
B15	B138	CD3-CD19+(B cell)	PAB	-0.00856	7.97E-14
B15	B130	CD3-CD19+(B cell)	GLB	0.006335	3.19E-08
B15	B59	CD3-CD19+(B cell)	WBRV	-0.01324	0
B15	B60	CD3-CD19+(B cell)	WBRVI	-0.01881	0
B15	B64	CD3-CD19+(B cell)	WBRSR(mPas_5)	-0.01173	0
B15	B65	CD3-CD19+(B cell)	WBRSR(mPas_1)	-0.01779	0
B15	B87	CD3-CD19+(B cell)	NSE	0.010713	0
B15	B200	CD3-CD19+(B cell)	OP	0.00737	1.24E-10
B15	B216	CD3-CD19+(B cell)	BMI	-0.00898	4.44E-15
B15	B215	CD3-CD19+(B cell)	BMI	-0.01221	0
B15	B210	CD3-CD19+(B cell)	Hip	-0.00798	3.17E-12
B15	B90	CD3-CD19+(B cell)	CYFRA 21-1	-0.023	0
B15	B139	CD3-CD19+(B cell)	ADA	0.0277	0
B15	B34	CD3-CD19+(B cell)	TK	0.012498	0
B15	B196	CD3-CD19+(B cell)	TCO2	-0.00953	0
B15	B51	CD3-CD19+(B cell)	Hb	-0.00823	6.90E-13
B15	B183	CD3-CD19+(B cell)	PTA	0.116881	0
B15	B185	CD3-CD19+(B cell)	PT	0.029847	0
B15	B186	CD3-CD19+(B cell)	PFC	-0.02223	0
B15	B52	CD3-CD19+(B cell)	PLT/BPC	0.012683	0
B15	B206	CD3-CD19+(B cell)	Waist	-0.01767	0
B15	B79	CD3-CD19+(B cell)	hb V -dna	0.021302	0
B15	B204	CD3-CD19+(B cell)	Drinking	-0.01942	0
B15	B203	CD3-CD19+(B cell)	DH	0.006683	5.40E-09
B15	B113	CD3-CD19+(B cell)	FT3	-0.00796	3.66E-12
B15	B211	CD3-CD19+(B cell)	IOP-R	-0.00827	5.27E-13
B15	B74	CD3-CD19+(B cell)	ApoA1	-0.01226	0
B15	B81	CD3-CD19+(B cell)	TSGF	-0.01197	0
B15	B77	CD3-CD19+(B cell)	TC	-0.00922	8.88E-16
B15	B91	CD3-CD19+(B cell)	TP	0.007267	2.23E-10
B15	B114	CD3-CD19+(B cell)	TSH TT3 TT4(TT4)	0.010081	0
B15	B144	CD3-CD19+(B cell)	Total-PINP	-0.00697	1.17E-09

B15	B212	CD3-CD19+(B cell)	IOP-L	-0.01333	0
B150	B160	ALP	PH	-0.0353	0
B150	B165	ALP	bilirubin	0.038532	0
B150	B166	ALP	Pro	0.012606	0
B150	B188	ALP	Ca	0.006888	1.81E-09
B150	B189	ALP	eGFB	-0.03756	0
B150	B181	ALP	APTT	-0.01092	0
B150	B190	ALP	Cre	0.032866	0
B150	B224	ALP	pmh	0.006605	8.10E-09
B150	B193	ALP	K	0.021819	0
B150	B222	ALP	NS	0.052374	0
B150	B151	ALP	Anti-CCP	0.006612	7.79E-09
B150	B198	ALP	P	-0.0366	0
B150	B194	ALP	Cl	-0.02307	0
B150	B199	ALP	Mg	0.044147	0
B150	B155	ALP	IgA	0.018485	0
B150	B156	ALP	IgE	0.010177	0
B150	B157	ALP	IgG	-0.00623	5.37E-08
B150	B195	ALP	Na	0.074725	0
B150	B168	ALP	NMP22	0.006091	1.05E-07
B150	B176	ALP	Ucr	0.018873	0
B150	B178	ALP	Ig-U	0.007456	7.52E-11
B150	B191	ALP	Urea	0.028209	0
B150	B192	ALP	UA	0.053797	0
B150	B170	ALP	U-GLU	0.023972	0
B150	B175	ALP	ALB-U	0.008128	1.28E-12
B150	B177	ALP	ACR	0.009088	2.22E-15
B150	B179	ALP	α1-MG	0.033185	0
B150	B180	ALP	Tf	0.007155	4.20E-10
B150	B182	ALP	TT	0.014006	0
B150	B153	ALP	Glu	0.007377	1.19E-10
B150	B221	ALP	Hei	0.019255	0
B150	B200	ALP	OP	0.087917	0
B150	B220	ALP	SBP	0.049262	0
B150	B219	ALP	OT	0.012444	0
B150	B218	ALP	DBP	0.041892	0
B150	B216	ALP	BMI	0.047373	0
B150	B215	ALP	BMI	0.050762	0
B150	B172	ALP	KB	-0.00804	2.30E-12
B150	B210	ALP	Hip	0.035404	0
B150	B209	ALP	Smoking	0.04888	0
B150	B183	ALP	PTA	-0.01921	0
B150	B186	ALP	PFC	0.041455	0
B150	B207	ALP	WHR	0.008132	1.25E-12

B150	B206	ALP	Waist	0.06377	0
B150	B197	ALP	AG	0.060893	0
B150	B204	ALP	Drinking	0.021842	0
B150	B203	ALP	DH	0.007775	1.14E-11
B150	B201	ALP	e-habits	0.008366	2.79E-13
B151	B164	Anti-CCP	COM	0.009475	0
B151	B189	Anti-CCP	eGFB	-0.00626	4.68E-08
B151	B181	Anti-CCP	APTT	-0.00565	8.15E-07
B151	B193	Anti-CCP	K	-0.00756	3.98E-11
B151	B222	Anti-CCP	NS	0.005799	4.13E-07
B151	B154	Anti-CCP	RF	0.125766	0
B151	B198	Anti-CCP	P	0.007564	4.00E-11
B151	B155	Anti-CCP	IgA	0.018037	0
B151	B157	Anti-CCP	IgG	0.029625	0
B151	B158	Anti-CCP	IgM	0.006149	7.96E-08
B151	B195	Anti-CCP	Na	-0.00941	2.22E-16
B151	B176	Anti-CCP	Ucr	-0.00715	4.33E-10
B151	B182	Anti-CCP	TT	-0.01462	0
B151	B200	Anti-CCP	OP	-0.00679	3.00E-09
B151	B183	Anti-CCP	PTA	-0.01849	0
B151	B186	Anti-CCP	PFC	0.020491	0
B151	B206	Anti-CCP	Waist	0.006171	7.15E-08
B151	B197	Anti-CCP	AG	-0.00763	2.70E-11
B152	B181	ACA-Ig(G-A-M)	APTT	0.01493	0
B152	B183	ACA-Ig(G-A-M)	PTA	-0.01027	0
B152	B185	ACA-Ig(G-A-M)	PT	0.006968	1.18E-09
B153	B160	Glu	PH	-0.01582	0
B153	B225	Glu	hPBG	0.008426	1.89E-13
B153	B166	Glu	Pro	0.006958	1.24E-09
B153	B189	Glu	eGFB	-0.01706	0
B153	B222	Glu	NS	0.02785	0
B153	B191	Glu	Urea	0.012081	0
B153	B174	Glu	Urea/Cr	0.007863	6.64E-12
B153	B192	Glu	UA	0.009856	0
B153	B170	Glu	U-GLU	0.09198	0
B153	B179	Glu	α1-MG	0.006879	1.91E-09
B153	B200	Glu	OP	0.006002	1.60E-07
B153	B220	Glu	SBP	0.035648	0
B153	B219	Glu	OT	0.00581	3.92E-07
B153	B218	Glu	DBP	0.032365	0
B153	B216	Glu	BMI	0.02837	0
B153	B215	Glu	BMI	0.035951	0
B153	B172	Glu	KB	0.006209	5.93E-08
B153	B210	Glu	Hip	0.021159	0

B153	B209	Glu	Smoking	0.015658	0
B153	B207	Glu	WHR	0.005726	5.77E-07
B153	B206	Glu	Waist	0.037447	0
B153	B204	Glu	Drinking	0.01404	0
B154	B198	RF	P	-0.0058	4.09E-07
B154	B155	RF	IgA	0.016506	0
B154	B157	RF	IgG	0.006442	1.87E-08
B154	B158	RF	IgM	0.012094	0
B154	B179	RF	α1-MG	0.007918	4.76E-12
B155	B193	IgA	K	0.009463	0
B155	B198	IgA	P	-0.01602	0
B155	B194	IgA	Cl	-0.02687	0
B155	B199	IgA	Mg	-0.01319	0
B155	B156	IgA	IgE	0.063389	0
B155	B157	IgA	IgG	0.251375	0
B155	B158	IgA	IgM	0.022736	0
B155	B195	IgA	Na	-0.02413	0
B155	B159	IgA	β2-MG	0.006845	2.29E-09
B155	B178	IgA	Ig-U	0.012076	0
B155	B174	IgA	Urea/Cr	-0.00681	2.81E-09
B155	B192	IgA	UA	0.010352	0
B155	B170	IgA	U-GLU	0.012869	0
B155	B177	IgA	ACR	0.00711	5.39E-10
B155	B180	IgA	Tf	0.011794	0
B155	B182	IgA	TT	0.014065	0
B155	B200	IgA	OP	-0.0116	0
B155	B220	IgA	SBP	0.008346	3.17E-13
B155	B218	IgA	DBP	0.008926	6.66E-15
B155	B215	IgA	BMI	0.007467	7.08E-11
B155	B209	IgA	Smoking	-0.02223	0
B155	B185	IgA	PT	0.023406	0
B155	B206	IgA	Waist	0.005817	3.81E-07
B155	B197	IgA	AG	0.005918	2.39E-07
B156	B181	IgE	APTT	-0.00868	3.60E-14
B156	B190	IgE	Cre	0.006802	2.87E-09
B156	B193	IgE	K	0.012508	0
B156	B222	IgE	NS	0.007884	5.86E-12
B156	B194	IgE	Cl	-0.01952	0
B156	B157	IgE	IgG	0.040147	0
B156	B158	IgE	IgM	-0.00831	4.10E-13
B156	B195	IgE	Na	-0.01249	0
B156	B176	IgE	Ucr	0.016666	0
B156	B174	IgE	Urea/Cr	-0.00615	7.91E-08
B156	B192	IgE	UA	0.010554	0

B156	B179	IgE	α1-MG	0.00782	8.65E-12
B156	B200	IgE	OP	-0.00728	2.03E-10
B156	B220	IgE	SBP	0.007482	6.47E-11
B156	B218	IgE	DBP	0.006636	6.91E-09
B156	B216	IgE	BMI	0.007639	2.57E-11
B156	B215	IgE	BMI	0.007311	1.74E-10
B156	B210	IgE	Hip	0.005911	2.46E-07
B156	B209	IgE	Smoking	0.013895	0
B156	B206	IgE	Waist	0.007917	4.79E-12
B156	B204	IgE	Drinking	0.006697	5.02E-09
B157	B161	IgG	LEU	0.007657	2.31E-11
B157	B166	IgG	Pro	-0.00936	4.44E-16
B157	B181	IgG	APTT	0.037715	0
B157	B190	IgG	Cre	-0.01937	0
B157	B193	IgG	K	0.024055	0
B157	B222	IgG	NS	-0.02366	0
B157	B198	IgG	P	0.010912	0
B157	B194	IgG	Cl	-0.03279	0
B157	B199	IgG	Mg	-0.02117	0
B157	B158	IgG	IgM	0.074957	0
B157	B195	IgG	Na	-0.05446	0
B157	B159	IgG	β2-MG	-0.01168	0
B157	B176	IgG	Ucr	-0.01827	0
B157	B178	IgG	Ig-U	0.007715	1.63E-11
B157	B191	IgG	Urea	-0.00635	2.98E-08
B157	B174	IgG	Urea/Cr	0.007228	2.78E-10
B157	B192	IgG	UA	-0.0233	0
B157	B179	IgG	α1-MG	-0.02382	0
B157	B182	IgG	TT	0.008872	9.55E-15
B157	B221	IgG	Hei	-0.02725	0
B157	B187	IgG	NSE	0.010252	0
B157	B200	IgG	OP	-0.04891	0
B157	B219	IgG	OT	0.007926	4.51E-12
B157	B216	IgG	BMI	-0.01968	0
B157	B215	IgG	BMI	-0.00652	1.25E-08
B157	B210	IgG	Hip	-0.00892	7.11E-15
B157	B209	IgG	Smoking	-0.03954	0
B157	B183	IgG	PTA	0.008681	3.46E-14
B157	B185	IgG	PT	0.046335	0
B157	B186	IgG	PFC	0.012026	0
B157	B206	IgG	Waist	-0.01767	0
B157	B197	IgG	AG	-0.01454	0
B157	B204	IgG	Drinking	-0.03338	0
B157	B211	IgG	IOP-R	-0.01581	0

B157	B212	IgG	IOP-L	-0.0191	0
B158	B189	IgM	eGFB	0.006957	1.25E-09
B158	B181	IgM	APTT	0.024503	0
B158	B190	IgM	Cre	-0.01633	0
B158	B222	IgM	NS	-0.0178	0
B158	B198	IgM	P	0.008892	8.22E-15
B158	B195	IgM	Na	-0.01577	0
B158	B174	IgM	Urea/Cr	0.009133	1.55E-15
B158	B192	IgM	UA	-0.01955	0
B158	B179	IgM	α1-MG	-0.01053	0
B158	B221	IgM	Hei	-0.01809	0
B158	B200	IgM	OP	-0.01387	0
B158	B220	IgM	SBP	-0.00957	0
B158	B218	IgM	DBP	-0.00774	1.41E-11
B158	B216	IgM	BMI	-0.01809	0
B158	B215	IgM	BMI	-0.01121	0
B158	B210	IgM	Hip	-0.01131	0
B158	B209	IgM	Smoking	-0.00775	1.34E-11
B158	B185	IgM	PT	0.006089	1.06E-07
B158	B206	IgM	Waist	-0.01569	0
B158	B204	IgM	Drinking	-0.00707	6.55E-10
B158	B211	IgM	IOP-R	-0.00743	8.83E-11
B158	B212	IgM	IOP-L	-0.00803	2.33E-12
B159	B193	β2-MG	K	-0.00682	2.66E-09
B16	B23	CD3+T	CD3-CD16+CD56+(NK)	-0.59249	0
B16	B17	CD3+T	CD3+CD16+CD56+NK	0.127806	0
B16	B18	CD3+T	CD3+CD4+helper(inducer)T	0.555559	0
B16	B19	CD3+T	CD3+CD4+/CD3+CD8+	-0.02047	0
B16	B20	CD3+T	CD3+CD4+CD8+	0.16268	0
B16	B21	CD3+T	CD3+CD4-CD8-	0.186846	0
B16	B22	CD3+T	CD3+CD8+(T)	0.561987	0
B16	B92	CD3+T	ALDH2-Glu504Glu	-0.00651	1.30E-08
B16	B94	CD3+T	ALDH2-Lys504Lys	0.014421	0
B16	B143	CD3+T	β-CTx	0.00563	8.86E-07
B16	B126	CD3+T	Alb	-0.00633	3.34E-08
B16	B121	CD3+T	C3	0.012266	0
B16	B124	CD3+T	ACTH	-0.02896	0
B16	B134	CD3+T	CHE	-0.00694	1.38E-09
B16	B70	CD3+T	LDL-C	-0.00612	9.17E-08
B16	B145	CD3+T	BGP	0.006302	3.75E-08
B16	B55	CD3+T	TK	0.006621	7.45E-09
B16	B57	CD3+T	RBCAI	-0.01423	0
B16	B46	CD3+T	RBC Count	-0.00571	6.29E-07
B16	B181	CD3+T	APTT	0.011299	0

B16	B193	CD3+T	K	-0.0256	0
B16	B150	CD3+T	ALP	-0.01197	0
B16	B99	CD3+T	anti-PM-Scl	-0.01023	0
B16	B105	CD3+T	anti-U1RNP	-0.01344	0
B16	B96	CD3+T	dsDNA (IIF)	-0.00581	3.86E-07
B16	B98	CD3+T	anti-Jo-1	0.007088	6.10E-10
B16	B100	CD3+T	anti-Ro52	0.008779	1.80E-14
B16	B101	CD3+T	anti-Sc70	-0.01083	0
B16	B102	CD3+T	anti-Sm	-0.00884	1.20E-14
B16	B103	CD3+T	anti-SSA/Ro	0.015106	0
B16	B104	CD3+T	anti-SSB	0.010587	0
B16	B151	CD3+T	Anti-CCP	-0.01572	0
B16	B198	CD3+T	P	0.037015	0
B16	B194	CD3+T	Cl	0.013874	0
B16	B199	CD3+T	Mg	-0.00882	1.33E-14
B16	B155	CD3+T	IgA	-0.02193	0
B16	B156	CD3+T	IgE	-0.01746	0
B16	B157	CD3+T	IgG	-0.02333	0
B16	B195	CD3+T	Na	-0.01373	0
B16	B178	CD3+T	Ig-U	-0.00991	0
B16	B177	CD3+T	ACR	-0.00587	2.94E-07
B16	B179	CD3+T	α1-MG	-0.01991	0
B16	B180	CD3+T	Tf	-0.00581	4.00E-07
B16	B182	CD3+T	TT	-0.02599	0
B16	B138	CD3+T	PAB	-0.00868	3.44E-14
B16	B59	CD3+T	WBRV	-0.01331	0
B16	B60	CD3+T	WBRVI	-0.01678	0
B16	B61	CD3+T	WBHSRV	-0.00599	1.68E-07
B16	B62	CD3+T	WBHSRVI	-0.00868	3.51E-14
B16	B64	CD3+T	WBRSR(mPas_5)	-0.01945	0
B16	B65	CD3+T	WBRSR(mPas_1)	-0.0208	0
B16	B66	CD3+T	WBRSR(mPas_100)	-0.01366	0
B16	B67	CD3+T	WBRSR(mPas_50)	-0.01426	0
B16	B200	CD3+T	OP	-0.0175	0
B16	B220	CD3+T	SBP	-0.00849	1.22E-13
B16	B218	CD3+T	DBP	-0.00736	1.32E-10
B16	B90	CD3+T	CYFRA 21-1	0.007562	4.06E-11
B16	B139	CD3+T	ADA	-0.00921	8.88E-16
B16	B35	CD3+T	β2-MG	0.014317	0
B16	B196	CD3+T	TCO2	-0.00753	4.85E-11
B16	B183	CD3+T	PTA	0.018023	0
B16	B184	CD3+T	INR	-0.00963	0
B16	B186	CD3+T	PFC	-0.01791	0
B16	B69	CD3+T	PV	-0.01047	0

B16	B197	CD3+T	AG	-0.01127	0
B16	B73	CD3+T	ApoA	-0.00598	1.81E-07
B16	B74	CD3+T	ApoA1	-0.01249	0
B16	B75	CD3+T	ApoA1/B	-0.00714	4.62E-10
B16	B76	CD3+T	ApoB	-0.0128	0
B16	B91	CD3+T	TP	-0.00685	2.27E-09
B16	B114	CD3+T	TSH TT3 TT4(TT4)	-0.00606	1.25E-07
B160	B161	PH	LEU	-0.01453	0
B160	B166	PH	Pro	-0.00865	4.17E-14
B160	B188	PH	Ca	-0.00756	4.12E-11
B160	B189	PH	eGFB	0.126966	0
B160	B181	PH	APTT	0.00575	5.17E-07
B160	B190	PH	Cre	-0.09701	0
B160	B224	PH	pmh	-0.01055	0
B160	B193	PH	K	-0.00866	4.04E-14
B160	B222	PH	NS	-0.08135	0
B160	B198	PH	P	-0.0258	0
B160	B167	PH	PC	0.007371	1.23E-10
B160	B194	PH	Cl	-0.01112	0
B160	B195	PH	Na	-0.00805	2.08E-12
B160	B168	PH	NMP22	0.011912	0
B160	B176	PH	Ucr	-0.0486	0
B160	B191	PH	Urea	-0.12436	0
B160	B174	PH	Urea/Cr	-0.03313	0
B160	B192	PH	UA	-0.13123	0
B160	B170	PH	U-GLU	-0.04383	0
B160	B175	PH	ALB-U	-0.00633	3.27E-08
B160	B179	PH	α1-MG	-0.00655	1.08E-08
B160	B221	PH	Hei	-0.01785	0
B160	B200	PH	OP	-0.01841	0
B160	B220	PH	SBP	-0.0347	0
B160	B219	PH	OT	-0.01161	0
B160	B218	PH	DBP	-0.05627	0
B160	B216	PH	BMI	-0.07956	0
B160	B215	PH	BMI	-0.09373	0
B160	B172	PH	KB	-0.02695	0
B160	B210	PH	Hip	-0.06148	0
B160	B209	PH	Smoking	-0.11482	0
B160	B196	PH	TCO2	0.012716	0
B160	B183	PH	PTA	0.010005	0
B160	B186	PH	PFC	-0.00886	1.02E-14
B160	B207	PH	WHR	-0.01404	0
B160	B206	PH	Waist	-0.10629	0
B160	B197	PH	AG	-0.00748	6.59E-11

B160	B204	PH	Drinking	-0.06155	0
B160	B211	PH	IOP-R	0.012408	0
B160	B212	PH	IOP-L	0.012331	0
B161	B166	LEU	Pro	0.021578	0
B161	B189	LEU	eGFB	0.020062	0
B161	B190	LEU	Cre	-0.11034	0
B161	B222	LEU	NS	-0.1134	0
B161	B198	LEU	P	0.011349	0
B161	B168	LEU	NMP22	0.011819	0
B161	B176	LEU	Ucr	-0.01305	0
B161	B191	LEU	Urea	-0.02035	0
B161	B174	LEU	Urea/Cr	0.062878	0
B161	B192	LEU	UA	-0.09161	0
B161	B221	LEU	Hei	-0.1238	0
B161	B220	LEU	SBP	-0.02885	0
B161	B219	LEU	OT	0.015672	0
B161	B218	LEU	DBP	-0.03639	0
B161	B216	LEU	BMI	-0.09446	0
B161	B215	LEU	BMI	-0.03738	0
B161	B172	LEU	KB	0.012867	0
B161	B210	LEU	Hip	-0.05055	0
B161	B209	LEU	Smoking	-0.05776	0
B161	B173	LEU	NTT	0.097968	0
B161	B207	LEU	WHR	-0.00978	0
B161	B206	LEU	Waist	-0.07318	0
B161	B205	LEU	KDA	0.008525	9.86E-14
B161	B204	LEU	Drinking	-0.0646	0
B161	B203	LEU	DH	-0.00594	2.15E-07
B161	B211	LEU	IOP-R	-0.00771	1.69E-11
B161	B201	LEU	e-habits	-0.0129	0
B161	B212	LEU	IOP-L	-0.00639	2.42E-08
B164	B166	COM	Pro	-0.0242	0
B164	B189	COM	eGFB	0.009408	2.22E-16
B164	B190	COM	Cre	-0.01024	0
B164	B222	COM	NS	-0.00878	1.73E-14
B164	B192	COM	UA	-0.00845	1.61E-13
B164	B170	COM	U-GLU	-0.00581	3.87E-07
B164	B221	COM	Hei	-0.0076	3.30E-11
B164	B220	COM	SBP	-0.00675	3.74E-09
B164	B216	COM	BMI	-0.00615	8.06E-08
B164	B209	COM	Smoking	-0.00988	0
B164	B206	COM	Waist	-0.00657	9.78E-09
B165	B166	bilirubin	Pro	0.01089	0
B165	B192	bilirubin	UA	0.005635	8.65E-07

B165	B170	bilirubin	U-GLU	0.008353	3.05E-13
B165	B179	bilirubin	α1-MG	0.007469	6.97E-11
B165	B218	bilirubin	DBP	0.006481	1.53E-08
B165	B172	bilirubin	KB	0.022441	0
B165	B209	bilirubin	Smoking	0.012234	0
B165	B204	bilirubin	Drinking	0.010922	0
B166	B189	Pro	eGFB	-0.02833	0
B166	B190	Pro	Cre	0.134786	0
B166	B193	Pro	K	-0.01123	0
B166	B222	Pro	NS	0.125222	0
B166	B198	Pro	P	-0.00563	8.82E-07
B166	B199	Pro	Mg	0.007945	4.02E-12
B166	B168	Pro	NMP22	0.007338	1.49E-10
B166	B176	Pro	Ucr	0.091618	0
B166	B178	Pro	Ig-U	0.012436	0
B166	B191	Pro	Urea	0.075399	0
B166	B174	Pro	Urea/Cr	-0.03696	0
B166	B192	Pro	UA	0.095187	0
B166	B170	Pro	U-GLU	0.052736	0
B166	B175	Pro	ALB-U	0.022481	0
B166	B177	Pro	ACR	0.013003	0
B166	B179	Pro	α1-MG	0.029629	0
B166	B180	Pro	Tf	0.009748	0
B166	B221	Pro	Hei	0.131942	0
B166	B200	Pro	OP	0.008677	3.55E-14
B166	B220	Pro	SBP	0.049179	0
B166	B219	Pro	OT	-0.01407	0
B166	B218	Pro	DBP	0.056933	0
B166	B216	Pro	BMI	0.114801	0
B166	B215	Pro	BMI	0.059663	0
B166	B172	Pro	KB	0.084113	0
B166	B210	Pro	Hip	0.063535	0
B166	B209	Pro	Smoking	0.092168	0
B166	B173	Pro	NTT	-0.00835	3.12E-13
B166	B207	Pro	WHR	0.010181	0
B166	B206	Pro	Waist	0.091096	0
B166	B205	Pro	KDA	-0.01434	0
B166	B197	Pro	AG	0.006121	9.08E-08
B166	B204	Pro	Drinking	0.08129	0
B166	B203	Pro	DH	0.011832	0
B167	B178	PC	Ig-U	-0.0084	2.24E-13
B167	B180	PC	Tf	-0.01186	0
B167	B171	PC	EC	-0.0494	0
B168	B175	NMP22	ALB-U	0.010238	0

B168	B177	NMP22	ACR	0.011798	0
B168	B219	NMP22	OT	0.006474	1.59E-08
B168	B173	NMP22	NTT	0.018297	0
B169	B225	U-CRY	hPBG	-0.00619	6.43E-08
B17	B137	CD3+CD16+CD56+NK	AFU	-0.01194	0
B17	B23	CD3+CD16+CD56+NK	CD3-CD16+CD56+(NK)	-0.07935	0
B17	B18	CD3+CD16+CD56+NK	CD3+CD4+helper(inducer)T	-0.10188	0
B17	B19	CD3+CD16+CD56+NK	CD3+CD4+/CD3+CD8+	-0.16721	0
B17	B20	CD3+CD16+CD56+NK	CD3+CD4+CD8+	0.173318	0
B17	B21	CD3+CD16+CD56+NK	CD3+CD4-CD8-	0.239841	0
B17	B22	CD3+CD16+CD56+NK	CD3+CD8+(T)	0.198102	0
B17	B145	CD3+CD16+CD56+NK	BGP	0.005616	9.42E-07
B17	B55	CD3+CD16+CD56+NK	TK	0.006541	1.13E-08
B17	B96	CD3+CD16+CD56+NK	dsDNA (IIF)	-0.0154	0
B17	B100	CD3+CD16+CD56+NK	anti-Ro52	-0.01947	0
B17	B103	CD3+CD16+CD56+NK	anti-SSA/Ro	-0.00673	4.27E-09
B17	B110	CD3+CD16+CD56+NK	AHA	0.010131	0
B17	B199	CD3+CD16+CD56+NK	Mg	0.007331	1.55E-10
B17	B155	CD3+CD16+CD56+NK	IgA	-0.00715	4.30E-10
B17	B182	CD3+CD16+CD56+NK	TT	-0.01141	0
B17	B209	CD3+CD16+CD56+NK	Smoking	-0.00575	5.06E-07
B17	B90	CD3+CD16+CD56+NK	CYFRA 21-1	-0.00854	9.15E-14
B17	B186	CD3+CD16+CD56+NK	PFC	0.005679	7.12E-07
B17	B69	CD3+CD16+CD56+NK	PV	-0.01041	0
B17	B114	CD3+CD16+CD56+NK	TSH TT3 TT4(TT4)	-0.00816	1.06E-12
B170	B225	U-GLU	hPBG	0.040796	0
B170	B189	U-GLU	eGFB	-0.00701	9.55E-10
B170	B181	U-GLU	APTT	-0.01051	0
B170	B222	U-GLU	NS	0.055219	0
B170	B198	U-GLU	P	-0.00671	4.61E-09
B170	B194	U-GLU	Cl	-0.02035	0
B170	B199	U-GLU	Mg	-0.00804	2.29E-12
B170	B195	U-GLU	Na	-0.01281	0
B170	B176	U-GLU	Ucr	0.013617	0
B170	B178	U-GLU	Ig-U	0.010876	0
B170	B191	U-GLU	Urea	0.027006	0
B170	B174	U-GLU	Urea/Cr	0.024362	0
B170	B192	U-GLU	UA	-0.01435	0
B170	B175	U-GLU	ALB-U	0.018141	0
B170	B177	U-GLU	ACR	0.01435	0
B170	B179	U-GLU	α1-MG	0.035928	0
B170	B180	U-GLU	Tf	0.013807	0
B170	B221	U-GLU	Hei	0.017612	0
B170	B200	U-GLU	OP	0.018745	0

B170	B220	U-GLU	SBP	0.052173	0
B170	B218	U-GLU	DBP	0.051275	0
B170	B216	U-GLU	BMI	0.044254	0
B170	B215	U-GLU	BMI	0.046543	0
B170	B172	U-GLU	KB	0.076181	0
B170	B210	U-GLU	Hip	0.021164	0
B170	B209	U-GLU	Smoking	0.058272	0
B170	B183	U-GLU	PTA	-0.00654	1.13E-08
B170	B173	U-GLU	NTT	0.012217	0
B170	B207	U-GLU	WHR	0.014667	0
B170	B206	U-GLU	Waist	0.062032	0
B170	B197	U-GLU	AG	0.007164	4.00E-10
B170	B204	U-GLU	Drinking	0.039986	0
B170	B211	U-GLU	IOP-R	0.012923	0
B170	B212	U-GLU	IOP-L	0.012993	0
B172	B225	KB	hPBG	0.006382	2.52E-08
B172	B189	KB	eGFB	0.041775	0
B172	B190	KB	Cre	-0.02788	0
B172	B222	KB	NS	-0.02974	0
B172	B194	KB	Cl	-0.00647	1.64E-08
B172	B176	KB	Ucr	0.018726	0
B172	B191	KB	Urea	0.013297	0
B172	B174	KB	Urea/Cr	0.029041	0
B172	B192	KB	UA	-0.01296	0
B172	B179	KB	α1-MG	0.008447	1.65E-13
B172	B221	KB	Hei	-0.012	0
B172	B220	KB	SBP	-0.01209	0
B172	B219	KB	OT	-0.00917	1.11E-15
B172	B218	KB	DBP	-0.00663	7.25E-09
B172	B216	KB	BMI	-0.03734	0
B172	B215	KB	BMI	-0.04178	0
B172	B210	KB	Hip	-0.03227	0
B172	B206	KB	Waist	-0.04336	0
B172	B204	KB	Drinking	0.005756	5.02E-07
B172	B203	KB	DH	0.006726	4.30E-09
B173	B189	NTT	eGFB	-0.03015	0
B173	B190	NTT	Cre	-0.03352	0
B173	B222	NTT	NS	-0.0432	0
B173	B198	NTT	P	0.006416	2.13E-08
B173	B176	NTT	Ucr	-0.01428	0
B173	B174	NTT	Urea/Cr	0.024008	0
B173	B192	NTT	UA	-0.03545	0
B173	B221	NTT	Hei	-0.05878	0
B173	B227	NTT	OD	0.007186	3.53E-10

B173	B220	NTT	SBP	0.007674	2.09E-11
B173	B219	NTT	OT	0.021772	0
B173	B218	NTT	DBP	-0.00858	6.82E-14
B173	B216	NTT	BMI	-0.0393	0
B173	B215	NTT	BMI	-0.01017	0
B173	B210	NTT	Hip	-0.01794	0
B173	B209	NTT	Smoking	-0.02432	0
B173	B185	NTT	PT	0.010025	0
B173	B206	NTT	Waist	-0.01835	0
B173	B205	NTT	KDA	0.006377	2.58E-08
B173	B204	NTT	Drinking	-0.02593	0
B173	B203	NTT	DH	-0.00827	5.25E-13
B174	B188	Urea/Cr	Ca	-0.01131	0
B174	B189	Urea/Cr	eGFB	0.199106	0
B174	B190	Urea/Cr	Cre	-0.40955	0
B174	B222	Urea/Cr	NS	-0.2253	0
B174	B198	Urea/Cr	P	0.028537	0
B174	B194	Urea/Cr	Cl	0.008021	2.51E-12
B174	B176	Urea/Cr	Ucr	-0.05497	0
B174	B191	Urea/Cr	Urea	0.531287	0
B174	B192	Urea/Cr	UA	-0.25168	0
B174	B175	Urea/Cr	ALB-U	-0.00834	3.37E-13
B174	B179	Urea/Cr	α1-MG	-0.01015	0
B174	B221	Urea/Cr	Hei	-0.28703	0
B174	B200	Urea/Cr	OP	0.026916	0
B174	B220	Urea/Cr	SBP	-0.04791	0
B174	B219	Urea/Cr	OT	0.037463	0
B174	B218	Urea/Cr	DBP	-0.072	0
B174	B216	Urea/Cr	BMI	-0.20907	0
B174	B215	Urea/Cr	BMI	-0.07376	0
B174	B210	Urea/Cr	Hip	-0.12119	0
B174	B209	Urea/Cr	Smoking	-0.10514	0
B174	B196	Urea/Cr	TCO2	-0.00864	4.49E-14
B174	B183	Urea/Cr	PTA	-0.00705	7.64E-10
B174	B207	Urea/Cr	WHR	-0.01304	0
B174	B206	Urea/Cr	Waist	-0.12648	0
B174	B205	Urea/Cr	KDA	0.0091	1.78E-15
B174	B204	Urea/Cr	Drinking	-0.12023	0
B174	B203	Urea/Cr	DH	-0.0121	0
B174	B211	Urea/Cr	IOP-R	-0.01156	0
B174	B201	Urea/Cr	e-habits	-0.01942	0
B174	B212	Urea/Cr	IOP-L	-0.00961	0
B175	B189	ALB-U	eGFB	-0.02063	0
B175	B190	ALB-U	Cre	0.03468	0

B175	B222	ALB-U	NS	0.014193	0
B175	B176	ALB-U	Ucr	0.060978	0
B175	B178	ALB-U	Ig-U	0.050885	0
B175	B191	ALB-U	Urea	0.014406	0
B175	B192	ALB-U	UA	0.01902	0
B175	B177	ALB-U	ACR	0.68259	0
B175	B179	ALB-U	α1-MG	0.028359	0
B175	B180	ALB-U	Tf	0.068871	0
B175	B221	ALB-U	Hei	0.008149	1.12E-12
B175	B200	ALB-U	OP	0.006231	5.34E-08
B175	B220	ALB-U	SBP	0.020497	0
B175	B218	ALB-U	DBP	0.021166	0
B175	B216	ALB-U	BMI	0.011976	0
B175	B215	ALB-U	BMI	0.009864	0
B175	B210	ALB-U	Hip	0.008278	4.92E-13
B175	B209	ALB-U	Smoking	0.006233	5.28E-08
B175	B206	ALB-U	Waist	0.011881	0
B176	B189	Ucr	eGFB	-0.02177	0
B176	B181	Ucr	APTT	-0.00665	6.28E-09
B176	B190	Ucr	Cre	0.105648	0
B176	B193	Ucr	K	-0.00622	5.55E-08
B176	B222	Ucr	NS	0.100495	0
B176	B194	Ucr	Cl	-0.0101	0
B176	B199	Ucr	Mg	0.010394	0
B176	B195	Ucr	Na	0.01016	0
B176	B178	Ucr	Ig-U	0.0411	0
B176	B191	Ucr	Urea	0.031313	0
B176	B192	Ucr	UA	0.08974	0
B176	B177	Ucr	ACR	-0.05089	0
B176	B179	Ucr	α1-MG	0.145167	0
B176	B180	Ucr	Tf	0.017285	0
B176	B221	Ucr	Hei	0.105982	0
B176	B187	Ucr	NSE	0.007256	2.37E-10
B176	B200	Ucr	OP	0.014097	0
B176	B220	Ucr	SBP	0.01172	0
B176	B219	Ucr	OT	-0.01742	0
B176	B218	Ucr	DBP	0.033039	0
B176	B216	Ucr	BMI	0.088477	0
B176	B215	Ucr	BMI	0.041257	0
B176	B210	Ucr	Hip	0.047436	0
B176	B209	Ucr	Smoking	0.068363	0
B176	B183	Ucr	PTA	0.006981	1.09E-09
B176	B207	Ucr	WHR	0.00614	8.31E-08
B176	B206	Ucr	Waist	0.06537	0

B176	B205	Ucr	KDA	-0.0077	1.81E-11
B176	B197	Ucr	AG	0.011435	0
B176	B204	Ucr	Drinking	0.065196	0
B176	B201	Ucr	e-habits	-0.0085	1.15E-13
B177	B189	ACR	eGFB	-0.0175	0
B177	B190	ACR	Cre	0.02286	0
B177	B199	ACR	Mg	-0.00568	7.11E-07
B177	B178	ACR	Ig-U	0.148455	0
B177	B191	ACR	Urea	0.012404	0
B177	B192	ACR	UA	0.007429	8.81E-11
B177	B179	ACR	α1-MG	0.042284	0
B177	B180	ACR	Tf	0.242956	0
B177	B221	ACR	Hei	-0.00734	1.49E-10
B177	B200	ACR	OP	0.008572	7.19E-14
B177	B220	ACR	SBP	0.021184	0
B177	B218	ACR	DBP	0.01686	0
B177	B186	ACR	PFC	0.005857	3.16E-07
B177	B204	ACR	Drinking	-0.00742	9.09E-11
B178	B190	Ig-U	Cre	0.013258	0
B178	B222	Ig-U	NS	0.006285	4.08E-08
B178	B192	Ig-U	UA	0.007948	3.94E-12
B178	B179	Ig-U	α1-MG	0.330879	0
B178	B180	Ig-U	Tf	0.53879	0
B178	B200	Ig-U	OP	0.013078	0
B178	B220	Ig-U	SBP	0.010731	0
B178	B218	Ig-U	DBP	0.008388	2.42E-13
B178	B216	Ig-U	BMI	0.006632	7.02E-09
B178	B215	Ig-U	BMI	0.006287	4.05E-08
B178	B183	Ig-U	PTA	0.007051	7.47E-10
B178	B186	Ig-U	PFC	0.014591	0
B178	B206	Ig-U	Waist	0.006722	4.39E-09
B178	B197	Ig-U	AG	0.007843	7.52E-12
B179	B189	α1-MG	eGFB	-0.00593	2.23E-07
B179	B181	α1-MG	APTT	-0.01503	0
B179	B190	α1-MG	Cre	0.037556	0
B179	B193	α1-MG	K	-0.01793	0
B179	B222	α1-MG	NS	0.028059	0
B179	B198	α1-MG	P	-0.01488	0
B179	B194	α1-MG	Cl	-0.00873	2.53E-14
B179	B199	α1-MG	Mg	0.007182	3.61E-10
B179	B195	α1-MG	Na	0.014559	0
B179	B191	α1-MG	Urea	0.023683	0
B179	B192	α1-MG	UA	0.023851	0
B179	B180	α1-MG	Tf	0.224128	0

B179	B221	α1-MG	Hei	0.025174	0
B179	B187	α1-MG	NSE	0.006498	1.40E-08
B179	B200	α1-MG	OP	0.039158	0
B179	B220	α1-MG	SBP	0.020138	0
B179	B218	α1-MG	DBP	0.019628	0
B179	B216	α1-MG	BMI	0.02086	0
B179	B215	α1-MG	BMI	0.009915	0
B179	B210	α1-MG	Hip	0.009277	4.44E-16
B179	B209	α1-MG	Smoking	0.035807	0
B179	B196	α1-MG	TCO2	-0.00596	1.97E-07
B179	B185	α1-MG	PT	-0.00751	5.45E-11
B179	B186	α1-MG	PFC	0.031008	0
B179	B206	α1-MG	Waist	0.021849	0
B179	B197	α1-MG	AG	0.018984	0
B179	B204	α1-MG	Drinking	0.030057	0
B18	B24	CD3+CD4+helper(inducer)T	αHBDH	-0.00737	1.23E-10
B18	B23	CD3+CD4+helper(inducer)T	CD3-CD16+CD56+(NK)	-0.39092	0
B18	B19	CD3+CD4+helper(inducer)T	CD3+CD4+/CD3+CD8+	0.716139	0
B18	B20	CD3+CD4+helper(inducer)T	CD3+CD4+CD8+	0.099828	0
B18	B21	CD3+CD4+helper(inducer)T	CD3+CD4-CD8-	-0.14759	0
B18	B22	CD3+CD4+helper(inducer)T	CD3+CD8+(T)	-0.27596	0
B18	B92	CD3+CD4+helper(inducer)T	ALDH2-Glu504Glu	-0.00608	1.10E-07
B18	B93	CD3+CD4+helper(inducer)T	ALDH2-Glu504Lys	0.00621	5.90E-08
B18	B126	CD3+CD4+helper(inducer)T	Alb	-0.01204	0
B18	B121	CD3+CD4+helper(inducer)T	C3	0.011777	0
B18	B31	CD3+CD4+helper(inducer)T	FSH	0.006408	2.21E-08
B18	B124	CD3+CD4+helper(inducer)T	ACTH	-0.04536	0
B18	B134	CD3+CD4+helper(inducer)T	CHE	-0.00799	3.10E-12
B18	B55	CD3+CD4+helper(inducer)T	TK	0.007662	2.25E-11
B18	B56	CD3+CD4+helper(inducer)T	IR	-0.00736	1.31E-10
B18	B57	CD3+CD4+helper(inducer)T	RBCAI	-0.00845	1.57E-13
B18	B46	CD3+CD4+helper(inducer)T	RBC Count	-0.00959	0
B18	B181	CD3+CD4+helper(inducer)T	APTT	-0.01699	0
B18	B190	CD3+CD4+helper(inducer)T	Cre	-0.00774	1.37E-11
B18	B25	CD3+CD4+helper(inducer)T	CK	-0.00649	1.45E-08
B18	B193	CD3+CD4+helper(inducer)T	K	-0.02883	0
B18	B129	CD3+CD4+helper(inducer)T	IBIL	-0.00953	0
B18	B105	CD3+CD4+helper(inducer)T	anti-U1RNP	-0.00832	3.75E-13
B18	B95	CD3+CD4+helper(inducer)T	anti-cenpb	0.014093	0
B18	B98	CD3+CD4+helper(inducer)T	anti-Jo-1	-0.03099	0
B18	B100	CD3+CD4+helper(inducer)T	anti-Ro52	0.009072	2.22E-15
B18	B101	CD3+CD4+helper(inducer)T	anti-Sc70	-0.00695	1.28E-09
B18	B102	CD3+CD4+helper(inducer)T	anti-Sm	-0.00977	0
B18	B104	CD3+CD4+helper(inducer)T	anti-SSB	0.008914	7.11E-15

B18	B106	CD3+CD4+helper(inducer)T	anti-P	0.01847	0
B18	B107	CD3+CD4+helper(inducer)T	AnuA	0.005884	2.80E-07
B18	B109	CD3+CD4+helper(inducer)T	AMA-M2	0.009827	0
B18	B198	CD3+CD4+helper(inducer)T	P	0.048553	0
B18	B194	CD3+CD4+helper(inducer)T	Cl	0.020594	0
B18	B199	CD3+CD4+helper(inducer)T	Mg	-0.00868	3.60E-14
B18	B155	CD3+CD4+helper(inducer)T	IgA	-0.02647	0
B18	B156	CD3+CD4+helper(inducer)T	IgE	-0.00918	1.11E-15
B18	B157	CD3+CD4+helper(inducer)T	IgG	-0.05305	0
B18	B179	CD3+CD4+helper(inducer)T	α1-MG	-0.01711	0
B18	B182	CD3+CD4+helper(inducer)T	TT	-0.01241	0
B18	B125	CD3+CD4+helper(inducer)T	Cor	-0.03692	0
B18	B138	CD3+CD4+helper(inducer)T	PAB	-0.01255	0
B18	B130	CD3+CD4+helper(inducer)T	GLB	-0.00638	2.49E-08
B18	B59	CD3+CD4+helper(inducer)T	WBRV	-0.01343	0
B18	B60	CD3+CD4+helper(inducer)T	WBRVI	-0.02067	0
B18	B61	CD3+CD4+helper(inducer)T	WBHSRV	-0.01432	0
B18	B62	CD3+CD4+helper(inducer)T	WBHSRVI	-0.01863	0
B18	B64	CD3+CD4+helper(inducer)T	WBRSR(mPas_5)	-0.02816	0
B18	B65	CD3+CD4+helper(inducer)T	WBRSR(mPas_1)	-0.02673	0
B18	B66	CD3+CD4+helper(inducer)T	WBRSR(mPas_100)	-0.02607	0
B18	B67	CD3+CD4+helper(inducer)T	WBRSR(mPas_50)	-0.02636	0
B18	B27	CD3+CD4+helper(inducer)T	LDH	-0.00586	3.14E-07
B18	B200	CD3+CD4+helper(inducer)T	OP	-0.00842	2.02E-13
B18	B220	CD3+CD4+helper(inducer)T	SBP	-0.00697	1.15E-09
B18	B209	CD3+CD4+helper(inducer)T	Smoking	0.016917	0
B18	B90	CD3+CD4+helper(inducer)T	CYFRA 21-1	0.011613	0
B18	B139	CD3+CD4+helper(inducer)T	ADA	-0.01047	0
B18	B35	CD3+CD4+helper(inducer)T	β2-MG	0.011826	0
B18	B196	CD3+CD4+helper(inducer)T	TCO2	-0.01246	0
B18	B51	CD3+CD4+helper(inducer)T	Hb	-0.0081	1.51E-12
B18	B184	CD3+CD4+helper(inducer)T	INR	-0.00825	5.80E-13
B18	B185	CD3+CD4+helper(inducer)T	PT	-0.01783	0
B18	B69	CD3+CD4+helper(inducer)T	PV	-0.01768	0
B18	B79	CD3+CD4+helper(inducer)T	hb V -dna	-0.01	0
B18	B197	CD3+CD4+helper(inducer)T	AG	-0.00575	5.05E-07
B18	B204	CD3+CD4+helper(inducer)T	Drinking	0.007366	1.27E-10
B18	B74	CD3+CD4+helper(inducer)T	ApoA1	-0.00702	9.02E-10
B18	B75	CD3+CD4+helper(inducer)T	ApoA1/B	-0.00722	2.94E-10
B18	B132	CD3+CD4+helper(inducer)T	Dbil	-0.00757	3.84E-11
B18	B81	CD3+CD4+helper(inducer)T	TSGF	0.016373	0
B18	B133	CD3+CD4+helper(inducer)T	TBIL	-0.00969	0
B18	B91	CD3+CD4+helper(inducer)T	TP	-0.01292	0
B180	B190	Tf	Cre	0.011613	0

B180	B222	Tf	NS	0.007631	2.70E-11
B180	B194	Tf	Cl	-0.0094	2.22E-16
B180	B199	Tf	Mg	-0.0093	4.44E-16
B180	B192	Tf	UA	0.01037	0
B180	B200	Tf	OP	0.012887	0
B180	B220	Tf	SBP	0.010616	0
B180	B218	Tf	DBP	0.009224	8.88E-16
B180	B216	Tf	BMI	0.007453	7.67E-11
B180	B215	Tf	BMI	0.006454	1.75E-08
B180	B210	Tf	Hip	0.006658	6.15E-09
B180	B183	Tf	PTA	0.007558	4.16E-11
B180	B186	Tf	PFC	0.011963	0
B180	B206	Tf	Waist	0.007654	2.36E-11
B180	B197	Tf	AG	0.007173	3.79E-10
B181	B189	APTT	eGFB	0.00718	3.65E-10
B181	B190	APTT	Cre	-0.00816	1.08E-12
B181	B193	APTT	K	0.006432	1.96E-08
B181	B222	APTT	NS	-0.01668	0
B181	B198	APTT	P	0.010186	0
B181	B194	APTT	Cl	0.0167	0
B181	B199	APTT	Mg	-0.01094	0
B181	B191	APTT	Urea	-0.00761	3.11E-11
B181	B192	APTT	UA	-0.01176	0
B181	B182	APTT	TT	0.115298	0
B181	B221	APTT	Hei	-0.00583	3.61E-07
B181	B200	APTT	OP	-0.00796	3.72E-12
B181	B220	APTT	SBP	-0.01241	0
B181	B218	APTT	DBP	-0.01574	0
B181	B216	APTT	BMI	-0.01599	0
B181	B215	APTT	BMI	-0.01755	0
B181	B210	APTT	Hip	-0.01203	0
B181	B209	APTT	Smoking	-0.01109	0
B181	B183	APTT	PTA	0.008565	7.55E-14
B181	B184	APTT	INR	-0.11264	0
B181	B185	APTT	PT	0.426257	0
B181	B186	APTT	PFC	-0.1293	0
B181	B206	APTT	Waist	-0.02021	0
B181	B197	APTT	AG	-0.00873	2.44E-14
B181	B204	APTT	Drinking	-0.01672	0
B181	B211	APTT	IOP-R	-0.01183	0
B181	B212	APTT	IOP-L	-0.01183	0
B182	B225	TT	hPBG	0.009232	8.88E-16
B182	B193	TT	K	0.00944	2.22E-16
B182	B222	TT	NS	0.007962	3.61E-12

B182	B194	TT	Cl	-0.04223	0
B182	B199	TT	Mg	0.017787	0
B182	B195	TT	Na	0.051766	0
B182	B192	TT	UA	0.008455	1.56E-13
B182	B187	TT	NSE	0.006542	1.12E-08
B182	B200	TT	OP	0.044566	0
B182	B218	TT	DBP	0.006183	6.73E-08
B182	B216	TT	BMI	0.006875	1.95E-09
B182	B210	TT	Hip	0.006783	3.19E-09
B182	B196	TT	TCO2	-0.01093	0
B182	B183	TT	PTA	-0.29512	0
B182	B185	TT	PT	0.012079	0
B182	B186	TT	PFC	-0.20472	0
B182	B206	TT	Waist	0.008645	4.44E-14
B182	B197	TT	AG	0.066146	0
B182	B204	TT	Drinking	0.005977	1.81E-07
B183	B225	PTA	hPBG	0.007776	1.13E-11
B183	B190	PTA	Cre	0.006019	1.48E-07
B183	B193	PTA	K	-0.01277	0
B183	B198	PTA	P	-0.00877	1.95E-14
B183	B194	PTA	Cl	0.046794	0
B183	B199	PTA	Mg	-0.02811	0
B183	B195	PTA	Na	-0.01744	0
B183	B200	PTA	OP	-0.00986	0
B183	B210	PTA	Hip	-0.01171	0
B183	B185	PTA	PT	0.022891	0
B183	B186	PTA	PFC	-0.15	0
B183	B206	PTA	Waist	-0.00908	2.22E-15
B183	B197	PTA	AG	-0.0444	0
B183	B204	PTA	Drinking	-0.00605	1.29E-07
B183	B212	PTA	IOP-L	-0.00944	2.22E-16
B184	B199	INR	Mg	-0.00577	4.63E-07
B184	B196	INR	TCO2	0.007651	2.39E-11
B184	B185	INR	PT	0.035657	0
B184	B186	INR	PFC	0.553698	0
B184	B197	INR	AG	-0.00914	1.33E-15
B185	B222	PT	NS	-0.00848	1.31E-13
B185	B199	PT	Mg	-0.01076	0
B185	B195	PT	Na	-0.00583	3.62E-07
B185	B219	PT	OT	0.009165	1.33E-15
B185	B218	PT	DBP	-0.00868	3.42E-14
B185	B216	PT	BMI	-0.00869	3.20E-14
B185	B215	PT	BMI	-0.00841	2.11E-13
B185	B209	PT	Smoking	-0.00959	0

B185	B206	PT	Waist	-0.00888	8.88E-15
B185	B197	PT	AG	-0.00907	2.44E-15
B185	B204	PT	Drinking	-0.01768	0
B185	B211	PT	IOP-R	-0.0064	2.35E-08
B185	B212	PT	IOP-L	-0.00736	1.35E-10
B186	B193	PFC	K	0.026157	0
B186	B198	PFC	P	0.006677	5.56E-09
B186	B194	PFC	Cl	-0.01295	0
B186	B199	PFC	Mg	0.011852	0
B186	B221	PFC	Hei	-0.00661	8.05E-09
B186	B200	PFC	OP	0.006194	6.37E-08
B186	B215	PFC	BMI	0.006312	3.58E-08
B186	B209	PFC	Smoking	0.007221	2.90E-10
B186	B196	PFC	TCO2	0.007351	1.38E-10
B186	B206	PFC	Waist	0.007661	2.26E-11
B187	B189	NSE	eGFB	-0.00671	4.66E-09
B187	B190	NSE	Cre	0.007348	1.41E-10
B187	B193	NSE	K	0.006196	6.34E-08
B187	B222	NSE	NS	0.006182	6.75E-08
B187	B194	NSE	Cl	-0.0102	0
B187	B192	NSE	UA	0.00758	3.64E-11
B187	B220	NSE	SBP	0.007197	3.32E-10
B187	B218	NSE	DBP	0.007734	1.45E-11
B187	B196	NSE	TCO2	0.006773	3.35E-09
B187	B206	NSE	Waist	0.005979	1.79E-07
B188	B189	Ca	eGFB	0.01027	0
B188	B190	Ca	Cre	0.025429	0
B188	B193	Ca	K	0.013861	0
B188	B222	Ca	NS	0.015996	0
B188	B194	Ca	Cl	-0.01254	0
B188	B195	Ca	Na	0.005674	7.27E-07
B188	B191	Ca	Urea	0.008155	1.08E-12
B188	B192	Ca	UA	0.043744	0
B188	B221	Ca	Hei	0.029559	0
B188	B200	Ca	OP	0.006021	1.46E-07
B188	B220	Ca	SBP	0.015698	0
B188	B218	Ca	DBP	0.017632	0
B188	B216	Ca	BMI	0.014775	0
B188	B210	Ca	Hip	0.007986	3.11E-12
B188	B197	Ca	AG	0.008978	4.44E-15
B188	B204	Ca	Drinking	0.010964	0
B188	B203	Ca	DH	0.011198	0
B188	B211	Ca	IOP-R	0.009369	4.44E-16
B188	B212	Ca	IOP-L	0.008669	3.77E-14

B189	B190	eGFB	Cre	-0.58387	0
B189	B224	eGFB	pmh	-0.02193	0
B189	B193	eGFB	K	-0.00873	2.44E-14
B189	B213	eGFB	CVA-R	-0.01819	0
B189	B214	eGFB	CVA-L	-0.01487	0
B189	B222	eGFB	NS	-0.20519	0
B189	B198	eGFB	P	0.019496	0
B189	B194	eGFB	Cl	-0.01706	0
B189	B199	eGFB	Mg	-0.02021	0
B189	B195	eGFB	Na	-0.02348	0
B189	B191	eGFB	Urea	-0.27207	0
B189	B192	eGFB	UA	-0.23116	0
B189	B221	eGFB	Hei	-0.03846	0
B189	B200	eGFB	OP	-0.03049	0
B189	B227	eGFB	OD	-0.02242	0
B189	B228	eGFB	OS	-0.02131	0
B189	B220	eGFB	SBP	-0.25659	0
B189	B219	eGFB	OT	-0.10283	0
B189	B218	eGFB	DBP	-0.18206	0
B189	B216	eGFB	BMI	-0.15785	0
B189	B215	eGFB	BMI	-0.18862	0
B189	B210	eGFB	Hip	-0.11002	0
B189	B209	eGFB	Smoking	-0.1587	0
B189	B196	eGFB	TCO2	-0.00923	8.88E-16
B189	B207	eGFB	WHR	-0.03571	0
B189	B206	eGFB	Waist	-0.24966	0
B189	B205	eGFB	KDA	-0.0262	0
B189	B204	eGFB	Drinking	-0.09394	0
B189	B203	eGFB	DH	0.065439	0
B189	B211	eGFB	IOP-R	0.01849	0
B189	B201	eGFB	e-habits	-0.07927	0
B189	B212	eGFB	IOP-L	0.010427	0
B19	B23	CD3+CD4+/CD3+CD8+	CD3-CD16+CD56+(NK)	0.007839	7.69E-12
B19	B20	CD3+CD4+/CD3+CD8+	CD3+CD4+CD8+	-0.08366	0
B19	B21	CD3+CD4+/CD3+CD8+	CD3+CD4-CD8-	-0.11014	0
B19	B22	CD3+CD4+/CD3+CD8+	CD3+CD8+(T)	-0.73723	0
B19	B92	CD3+CD4+/CD3+CD8+	ALDH2-Glu504Glu	-0.01171	0
B19	B93	CD3+CD4+/CD3+CD8+	ALDH2-Glu504Lys	0.014378	0
B19	B160	CD3+CD4+/CD3+CD8+	PH	-0.00583	3.57E-07
B19	B94	CD3+CD4+/CD3+CD8+	ALDH2-Lys504Lys	-0.00679	3.13E-09
B19	B126	CD3+CD4+/CD3+CD8+	Alb	-0.00681	2.77E-09
B19	B124	CD3+CD4+/CD3+CD8+	ACTH	-0.02373	0
B19	B56	CD3+CD4+/CD3+CD8+	IR	-0.00641	2.17E-08
B19	B46	CD3+CD4+/CD3+CD8+	RBC Count	-0.00629	4.01E-08

B19	B181	CD3+CD4+/CD3+CD8+	APTT	-0.02453	0
B19	B25	CD3+CD4+/CD3+CD8+	CK	-0.00587	3.03E-07
B19	B193	CD3+CD4+/CD3+CD8+	K	-0.00813	1.25E-12
B19	B129	CD3+CD4+/CD3+CD8+	IBIL	-0.00749	6.24E-11
B19	B150	CD3+CD4+/CD3+CD8+	ALP	0.006412	2.16E-08
B19	B99	CD3+CD4+/CD3+CD8+	anti-PM-Scl	0.00783	8.13E-12
B19	B105	CD3+CD4+/CD3+CD8+	anti-U1RNP	-0.00606	1.22E-07
B19	B95	CD3+CD4+/CD3+CD8+	anti-cenpb	0.011873	0
B19	B98	CD3+CD4+/CD3+CD8+	anti-Jo-1	-0.02194	0
B19	B100	CD3+CD4+/CD3+CD8+	anti-Ro52	0.008511	1.08E-13
B19	B106	CD3+CD4+/CD3+CD8+	anti-P	0.026886	0
B19	B151	CD3+CD4+/CD3+CD8+	Anti-CCP	0.025272	0
B19	B108	CD3+CD4+/CD3+CD8+	Anti-Cyclin	0.011813	0
B19	B109	CD3+CD4+/CD3+CD8+	AMA-M2	0.006856	2.16E-09
B19	B110	CD3+CD4+/CD3+CD8+	AHA	-0.00769	1.88E-11
B19	B198	CD3+CD4+/CD3+CD8+	P	0.024423	0
B19	B155	CD3+CD4+/CD3+CD8+	IgA	-0.01117	0
B19	B157	CD3+CD4+/CD3+CD8+	IgG	-0.03205	0
B19	B179	CD3+CD4+/CD3+CD8+	α1-MG	-0.00886	1.02E-14
B19	B180	CD3+CD4+/CD3+CD8+	Tf	0.00724	2.59E-10
B19	B125	CD3+CD4+/CD3+CD8+	Cor	-0.02447	0
B19	B138	CD3+CD4+/CD3+CD8+	PAB	-0.00971	0
B19	B60	CD3+CD4+/CD3+CD8+	WBRVI	-0.00925	6.66E-16
B19	B61	CD3+CD4+/CD3+CD8+	WBHSRV	-0.00883	1.24E-14
B19	B62	CD3+CD4+/CD3+CD8+	WBHSRVI	-0.01328	0
B19	B64	CD3+CD4+/CD3+CD8+	WBRSR(mPas_5)	-0.0133	0
B19	B65	CD3+CD4+/CD3+CD8+	WBRSR(mPas_1)	-0.01116	0
B19	B66	CD3+CD4+/CD3+CD8+	WBRSR(mPas_100)	-0.01523	0
B19	B67	CD3+CD4+/CD3+CD8+	WBRSR(mPas_50)	-0.0152	0
B19	B209	CD3+CD4+/CD3+CD8+	Smoking	0.013342	0
B19	B90	CD3+CD4+/CD3+CD8+	CYFRA 21-1	0.008731	2.49E-14
B19	B34	CD3+CD4+/CD3+CD8+	TK	0.008654	4.17E-14
B19	B196	CD3+CD4+/CD3+CD8+	TCO2	-0.00744	8.19E-11
B19	B183	CD3+CD4+/CD3+CD8+	PTA	-0.02182	0
B19	B184	CD3+CD4+/CD3+CD8+	INR	-0.01012	0
B19	B185	CD3+CD4+/CD3+CD8+	PT	-0.01025	0
B19	B186	CD3+CD4+/CD3+CD8+	PFC	0.011992	0
B19	B69	CD3+CD4+/CD3+CD8+	PV	-0.00612	9.27E-08
B19	B79	CD3+CD4+/CD3+CD8+	hb V -dna	-0.00711	5.31E-10
B19	B81	CD3+CD4+/CD3+CD8+	TSGF	0.011805	0
B19	B133	CD3+CD4+/CD3+CD8+	TBIL	-0.00734	1.47E-10
B19	B91	CD3+CD4+/CD3+CD8+	TP	-0.00698	1.08E-09
B19	B114	CD3+CD4+/CD3+CD8+	TSH TT3 TT4(TT4)	0.010131	0
B190	B224	Cre	pmh	0.014467	0

B190	B222	Cre	NS	0.482262	0
B190	B198	Cre	P	-0.03617	0
B190	B199	Cre	Mg	0.018282	0
B190	B195	Cre	Na	0.022326	0
B190	B191	Cre	Urea	0.291557	0
B190	B192	Cre	UA	0.539978	0
B190	B221	Cre	Hei	0.465767	0
B190	B200	Cre	OP	0.029514	0
B190	B227	Cre	OD	0.009489	0
B190	B228	Cre	OS	0.010538	0
B190	B220	Cre	SBP	0.194704	0
B190	B219	Cre	OT	-0.01953	0
B190	B218	Cre	DBP	0.183969	0
B190	B216	Cre	BMI	0.434313	0
B190	B215	Cre	BMI	0.250718	0
B190	B210	Cre	Hip	0.268206	0
B190	B209	Cre	Smoking	0.222345	0
B190	B196	Cre	TCO2	0.008415	2.03E-13
B190	B207	Cre	WHR	0.042384	0
B190	B206	Cre	Waist	0.362621	0
B190	B205	Cre	KDA	-0.02347	0
B190	B197	Cre	AG	0.007777	1.12E-11
B190	B204	Cre	Drinking	0.241118	0
B190	B201	Cre	e-habits	0.05589	0
B190	B212	Cre	IOP-L	0.005912	2.45E-07
B191	B224	Urea	pmh	0.006897	1.73E-09
B191	B193	Urea	K	0.011089	0
B191	B214	Urea	CVA-L	0.006278	4.22E-08
B191	B222	Urea	NS	0.144281	0
B191	B194	Urea	Cl	0.008039	2.24E-12
B191	B199	Urea	Mg	0.010696	0
B191	B195	Urea	Na	0.014833	0
B191	B192	Urea	UA	0.158119	0
B191	B221	Urea	Hei	0.058377	0
B191	B200	Urea	OP	0.061755	0
B191	B227	Urea	OD	0.006875	1.95E-09
B191	B228	Urea	OS	0.011866	0
B191	B220	Urea	SBP	0.107692	0
B191	B219	Urea	OT	0.028622	0
B191	B218	Urea	DBP	0.068489	0
B191	B216	Urea	BMI	0.119375	0
B191	B215	Urea	BMI	0.121806	0
B191	B210	Urea	Hip	0.080558	0
B191	B209	Urea	Smoking	0.071088	0

B191	B207	Urea	WHR	0.020404	0
B191	B206	Urea	Waist	0.156584	0
B191	B205	Urea	KDA	-0.00922	8.88E-16
B191	B197	Urea	AG	0.005974	1.83E-07
B191	B204	Urea	Drinking	0.069174	0
B191	B203	Urea	DH	-0.01057	0
B191	B211	Urea	IOP-R	-0.01017	0
B191	B201	Urea	e-habits	0.028615	0
B192	B222	UA	NS	0.514218	0
B192	B198	UA	P	-0.02427	0
B192	B194	UA	Cl	-0.01534	0
B192	B199	UA	Mg	0.007933	4.32E-12
B192	B195	UA	Na	0.024927	0
B192	B221	UA	Hei	0.421672	0
B192	B200	UA	OP	0.028294	0
B192	B220	UA	SBP	0.221459	0
B192	B219	UA	OT	-0.02799	0
B192	B218	UA	DBP	0.227819	0
B192	B216	UA	BMI	0.519827	0
B192	B215	UA	BMI	0.391728	0
B192	B210	UA	Hip	0.358818	0
B192	B209	UA	Smoking	0.199914	0
B192	B207	UA	WHR	0.051369	0
B192	B206	UA	Waist	0.468074	0
B192	B205	UA	KDA	-0.02286	0
B192	B197	UA	AG	0.02684	0
B192	B204	UA	Drinking	0.272118	0
B192	B203	UA	DH	0.036963	0
B192	B211	UA	IOP-R	0.023536	0
B192	B201	UA	e-habits	0.027226	0
B192	B212	UA	IOP-L	0.022122	0
B193	B222	K	NS	-0.00656	1.01E-08
B193	B198	K	P	0.063268	0
B193	B194	K	Cl	-0.01764	0
B193	B199	K	Mg	0.073608	0
B193	B195	K	Na	0.035789	0
B193	B200	K	OP	0.050483	0
B193	B220	K	SBP	-0.00685	2.24E-09
B193	B218	K	DBP	-0.0072	3.23E-10
B193	B216	K	BMI	-0.00606	1.21E-07
B193	B215	K	BMI	-0.00591	2.50E-07
B193	B209	K	Smoking	0.00773	1.49E-11
B193	B196	K	TCO2	0.054049	0
B193	B206	K	Waist	-0.00661	7.72E-09

B193	B197	K	AG	-0.01018	0
B193	B203	K	DH	0.006021	1.47E-07
B193	B201	K	e-habits	0.006035	1.37E-07
B194	B222	Cl	NS	-0.01164	0
B194	B198	Cl	P	-0.03185	0
B194	B199	Cl	Mg	-0.02534	0
B194	B195	Cl	Na	0.222963	0
B194	B221	Cl	Hei	-0.01597	0
B194	B200	Cl	OP	0.155251	0
B194	B220	Cl	SBP	-0.01109	0
B194	B218	Cl	DBP	-0.01865	0
B194	B215	Cl	BMI	0.006463	1.68E-08
B194	B209	Cl	Smoking	0.007976	3.31E-12
B194	B196	Cl	TCO2	-0.15189	0
B194	B197	Cl	AG	-0.3976	0
B194	B204	Cl	Drinking	-0.00968	0
B194	B203	Cl	DH	-0.0069	1.69E-09
B194	B211	Cl	IOP-R	-0.009	4.00E-15
B195	B224	Na	pmh	0.00629	3.98E-08
B195	B222	Na	NS	0.013038	0
B195	B199	Na	Mg	0.136216	0
B195	B221	Na	Hei	0.006535	1.16E-08
B195	B200	Na	OP	0.780663	0
B195	B220	Na	SBP	0.021958	0
B195	B218	Na	DBP	0.016509	0
B195	B216	Na	BMI	0.009286	4.44E-16
B195	B215	Na	BMI	0.008044	2.18E-12
B195	B209	Na	Smoking	0.014668	0
B195	B196	Na	TCO2	0.068238	0
B195	B206	Na	Waist	0.015191	0
B195	B197	Na	AG	0.388506	0
B195	B204	Na	Drinking	0.006236	5.20E-08
B195	B201	Na	e-habits	0.007263	2.29E-10
B196	B200	TCO2	OP	0.057895	0
B196	B220	TCO2	SBP	0.010271	0
B196	B218	TCO2	DBP	0.009587	0
B196	B197	TCO2	AG	-0.65001	0
B197	B225	AG	hPBG	0.006625	7.31E-09
B197	B222	AG	NS	0.012765	0
B197	B198	AG	P	0.023818	0
B197	B199	AG	Mg	0.094062	0
B197	B221	AG	Hei	0.009948	0
B197	B200	AG	OP	0.31185	0
B197	B220	AG	SBP	0.012019	0

B197	B218	AG	DBP	0.014168	0
B197	B216	AG	BMI	0.008065	1.91E-12
B197	B206	AG	Waist	0.008075	1.79E-12
B197	B204	AG	Drinking	0.012224	0
B197	B203	AG	DH	0.007168	3.91E-10
B198	B222	P	NS	-0.04019	0
B198	B199	P	Mg	0.010202	0
B198	B221	P	Hei	-0.03138	0
B198	B220	P	SBP	-0.02671	0
B198	B219	P	OT	0.007502	5.76E-11
B198	B218	P	DBP	-0.02905	0
B198	B216	P	BMI	-0.03492	0
B198	B215	P	BMI	-0.0247	0
B198	B210	P	Hip	-0.02179	0
B198	B209	P	Smoking	-0.01392	0
B198	B206	P	Waist	-0.03501	0
B198	B204	P	Drinking	-0.02665	0
B198	B211	P	IOP-R	-0.00595	2.03E-07
B198	B212	P	IOP-L	-0.00673	4.15E-09
B199	B225	Mg	hPBG	0.006407	2.23E-08
B199	B222	Mg	NS	0.006601	8.28E-09
B199	B200	Mg	OP	0.123289	0
B199	B218	Mg	DBP	0.005611	9.64E-07
B199	B209	Mg	Smoking	0.008405	2.17E-13
B199	B206	Mg	Waist	0.006471	1.61E-08
B199	B204	Mg	Drinking	0.009941	0
B2	B142	Age	25-(OH)D	0.013047	0
B2	B137	Age	AFU	0.015063	0
B2	B24	Age	αHBDH	0.05383	0
B2	B15	Age	CD3-CD19+(B cell)	-0.01722	0
B2	B19	Age	CD3+CD4+/CD3+CD8+	0.006912	1.60E-09
B2	B21	Age	CD3+CD4-CD8-	-0.00789	5.67E-12
B2	B54	Age	ESR	0.030422	0
B2	B13	Age	PGI_PGII	-0.00737	1.27E-10
B2	B160	Age	PH	-0.0969	0
B2	B140	Age	r-GT	0.053397	0
B2	B82	Age	CEA	0.008054	2.04E-12
B2	B118	Age	CEA1	0.040375	0
B2	B126	Age	Alb	-0.3222	0
B2	B127	Age	A-G ratio	-0.1507	0
B2	B40	Age	WBC	-0.07639	0
B2	B128	Age	ALT	-0.02705	0
B2	B164	Age	COM	-0.00844	1.73E-13
B2	B123	Age	hs-CRP1	0.017846	0

B2	B28	Age	PRL	-0.00586	3.10E-07
B2	B30	Age	LH	0.015282	0
B2	B111	Age	TSH	0.024201	0
B2	B31	Age	FSH	0.020073	0
B2	B41	Age	MON％	0.018641	0
B2	B166	Age	Pro	-0.02853	0
B2	B70	Age	LDL-C	0.211126	0
B2	B188	Age	Ca	-0.03611	0
B2	B71	Age	TG	0.087963	0
B2	B72	Age	HDL-C	0.025593	0
B2	B189	Age	eGFB	-0.61704	0
B2	B141	Age	AST/ALT	0.014151	0
B2	B146	Age	CysC	0.049244	0
B2	B56	Age	IR	-0.00926	6.66E-16
B2	B43	Age	MCV	0.082436	0
B2	B44	Age	MCH	0.107085	0
B2	B45	Age	MCHC	-0.0606	0
B2	B46	Age	RBC Count	-0.14239	0
B2	B47	Age	RDW	0.275648	0
B2	B181	Age	APTT	-0.00944	2.22E-16
B2	B190	Age	Cre	0.108984	0
B2	B25	Age	CK	-0.0082	8.19E-13
B2	B26	Age	CKMB	0.007249	2.47E-10
B2	B224	Age	pmh	0.038327	0
B2	B193	Age	K	0.008823	1.33E-14
B2	B129	Age	IBIL	0.018501	0
B2	B150	Age	ALP	0.048589	0
B2	B213	Age	CVA-R	0.021956	0
B2	B214	Age	CVA-L	0.01748	0
B2	B84	Age	C-PSA	0.028949	0
B2	B222	Age	NS	0.093939	0
B2	B151	Age	Anti-CCP	0.008692	3.24E-14
B2	B154	Age	RF	0.006366	2.73E-08
B2	B48	Age	LYM%	-0.00742	9.09E-11
B2	B198	Age	P	-0.01737	0
B2	B194	Age	Cl	0.017422	0
B2	B199	Age	Mg	0.014678	0
B2	B157	Age	IgG	0.010403	0
B2	B195	Age	Na	0.025294	0
B2	B176	Age	Ucr	-0.02734	0
B2	B191	Age	Urea	0.193604	0
B2	B174	Age	Urea/Cr	0.075295	0
B2	B192	Age	UA	-0.01089	0
B2	B170	Age	U-GLU	0.04909	0

B2	B179	Age	α1-MG	0.008861	1.02E-14
B2	B153	Age	Glu	0.03657	0
B2	B138	Age	PAB	-0.0189	0
B2	B130	Age	GLB	0.056443	0
B2	B60	Age	WBRVI	-0.01521	0
B2	B61	Age	WBHSRV	-0.00603	1.42E-07
B2	B62	Age	WBHSRVI	-0.015	0
B2	B64	Age	WBRSR(mPas_5)	-0.01225	0
B2	B65	Age	WBRSR(mPas_1)	-0.01131	0
B2	B66	Age	WBRSR(mPas_100)	-0.00985	0
B2	B67	Age	WBRSR(mPas_50)	-0.00998	0
B2	B86	Age	TBhCG	-0.00833	3.49E-13
B2	B27	Age	LDH	0.067283	0
B2	B221	Age	Hei	-0.21056	0
B2	B87	Age	NSE	0.007843	7.51E-12
B2	B200	Age	OP	0.029237	0
B2	B227	Age	OD	0.030788	0
B2	B228	Age	OS	0.028486	0
B2	B50	Age	eo%	0.076035	0
B2	B220	Age	SBP	0.33427	0
B2	B219	Age	OT	0.178928	0
B2	B218	Age	DBP	0.212567	0
B2	B216	Age	BMI	0.019969	0
B2	B215	Age	BMI	0.185226	0
B2	B131	Age	AST	0.059654	0
B2	B3	Age	Hcy	0.022298	0
B2	B172	Age	KB	-0.04448	0
B2	B210	Age	Hip	0.035208	0
B2	B10	Age	PG I	0.062429	0
B2	B12	Age	PG II	0.069004	0
B2	B14	Age	G-17	0.0236	0
B2	B209	Age	Smoking	0.200649	0
B2	B90	Age	CYFRA 21-1	0.051407	0
B2	B139	Age	ADA	0.024948	0
B2	B34	Age	TK	-0.00717	3.90E-10
B2	B196	Age	TCO2	0.007939	4.17E-12
B2	B51	Age	Hb	-0.05999	0
B2	B186	Age	PFC	0.011036	0
B2	B69	Age	PV	0.014237	0
B2	B52	Age	PLT/BPC	-0.19651	0
B2	B173	Age	NTT	0.053396	0
B2	B207	Age	WHR	0.042304	0
B2	B206	Age	Waist	0.255597	0
B2	B205	Age	KDA	0.051087	0

B2	B79	Age	hb V -dna	-0.01185	0
B2	B204	Age	Drinking	0.067285	0
B2	B203	Age	DH	-0.08575	0
B2	B80	Age	anti-HP IgG	0.027881	0
B2	B112	Age	FT4	-0.04668	0
B2	B113	Age	FT3	-0.03091	0
B2	B211	Age	IOP-R	-0.03108	0
B2	B33	Age	Prog	-0.00695	1.28E-09
B2	B201	Age	e-habits	0.092417	0
B2	B73	Age	ApoA	0.015942	0
B2	B74	Age	ApoA1	0.036453	0
B2	B75	Age	ApoA1/B	-0.01153	0
B2	B76	Age	ApoB	0.047252	0
B2	B77	Age	TC	0.223795	0
B2	B133	Age	TBIL	0.013788	0
B2	B135	Age	TBA	0.006842	2.33E-09
B2	B91	Age	TP	-0.15174	0
B2	B212	Age	IOP-L	-0.01739	0
B20	B142	CD3+CD4+CD8+	25-(OH)D	-0.0063	3.79E-08
B20	B137	CD3+CD4+CD8+	AFU	0.012503	0
B20	B23	CD3+CD4+CD8+	CD3-CD16+CD56+(NK)	-0.06029	0
B20	B21	CD3+CD4+CD8+	CD3+CD4-CD8-	-0.08134	0
B20	B22	CD3+CD4+CD8+	CD3+CD8+(T)	0.205694	0
B20	B54	CD3+CD4+CD8+	ESR	0.011212	0
B20	B92	CD3+CD4+CD8+	ALDH2-Glu504Glu	0.018756	0
B20	B93	CD3+CD4+CD8+	ALDH2-Glu504Lys	-0.01603	0
B20	B94	CD3+CD4+CD8+	ALDH2-Lys504Lys	-0.0087	3.15E-14
B20	B143	CD3+CD4+CD8+	β-CTx	-0.00919	8.88E-16
B20	B124	CD3+CD4+CD8+	ACTH	0.006534	1.17E-08
B20	B134	CD3+CD4+CD8+	CHE	-0.00675	3.83E-09
B20	B145	CD3+CD4+CD8+	BGP	-0.00913	1.78E-15
B20	B56	CD3+CD4+CD8+	IR	-0.0085	1.18E-13
B20	B181	CD3+CD4+CD8+	APTT	-0.0108	0
B20	B193	CD3+CD4+CD8+	K	-0.01573	0
B20	B150	CD3+CD4+CD8+	ALP	-0.00587	3.05E-07
B20	B105	CD3+CD4+CD8+	anti-U1RNP	-0.00728	2.07E-10
B20	B95	CD3+CD4+CD8+	anti-cenpb	-0.00587	2.94E-07
B20	B96	CD3+CD4+CD8+	dsDNA (IIF)	-0.00604	1.33E-07
B20	B97	CD3+CD4+CD8+	dsDNA	-0.01424	0
B20	B101	CD3+CD4+CD8+	anti-Sc70	-0.00594	2.15E-07
B20	B104	CD3+CD4+CD8+	anti-SSB	-0.01138	0
B20	B107	CD3+CD4+CD8+	AnuA	-0.00639	2.38E-08
B20	B108	CD3+CD4+CD8+	Anti-Cyclin	-0.00715	4.30E-10
B20	B154	CD3+CD4+CD8+	RF	0.027419	0

B20	B194	CD3+CD4+CD8+	Cl	0.017549	0
B20	B155	CD3+CD4+CD8+	IgA	-0.0086	6.04E-14
B20	B157	CD3+CD4+CD8+	IgG	-0.01012	0
B20	B158	CD3+CD4+CD8+	IgM	0.00968	0
B20	B195	CD3+CD4+CD8+	Na	-0.03264	0
B20	B182	CD3+CD4+CD8+	TT	-0.05778	0
B20	B125	CD3+CD4+CD8+	Cor	0.031011	0
B20	B60	CD3+CD4+CD8+	WBRVI	-0.01198	0
B20	B62	CD3+CD4+CD8+	WBHSRVI	-0.01158	0
B20	B64	CD3+CD4+CD8+	WBRSR(mPas_5)	-0.00852	1.03E-13
B20	B65	CD3+CD4+CD8+	WBRSR(mPas_1)	-0.00888	9.10E-15
B20	B66	CD3+CD4+CD8+	WBRSR(mPas_100)	-0.00772	1.59E-11
B20	B67	CD3+CD4+CD8+	WBRSR(mPas_50)	-0.00825	6.01E-13
B20	B200	CD3+CD4+CD8+	OP	-0.02936	0
B20	B183	CD3+CD4+CD8+	PTA	0.060774	0
B20	B184	CD3+CD4+CD8+	INR	-0.01143	0
B20	B186	CD3+CD4+CD8+	PFC	-0.01331	0
B20	B69	CD3+CD4+CD8+	PV	0.009955	0
B20	B197	CD3+CD4+CD8+	AG	-0.03337	0
B20	B112	CD3+CD4+CD8+	FT4	0.006687	5.27E-09
B20	B81	CD3+CD4+CD8+	TSGF	-0.04496	0
B20	B144	CD3+CD4+CD8+	Total-PINP	-0.01048	0
B200	B225	OP	hPBG	0.007625	2.79E-11
B200	B222	OP	NS	0.020906	0
B200	B221	OP	Hei	0.007614	2.98E-11
B200	B220	OP	SBP	0.027335	0
B200	B218	OP	DBP	0.021643	0
B200	B216	OP	BMI	0.016159	0
B200	B215	OP	BMI	0.01661	0
B200	B210	OP	Hip	0.009388	2.22E-16
B200	B209	OP	Smoking	0.01718	0
B200	B206	OP	Waist	0.024027	0
B200	B204	OP	Drinking	0.008567	7.44E-14
B200	B201	OP	e-habits	0.00648	1.53E-08
B201	B224	e-habits	pmh	0.029061	0
B201	B222	e-habits	NS	0.038569	0
B201	B221	e-habits	Hei	0.030901	0
B201	B220	e-habits	SBP	0.051815	0
B201	B219	e-habits	OT	0.034243	0
B201	B218	e-habits	DBP	0.019519	0
B201	B216	e-habits	BMI	0.025036	0
B201	B215	e-habits	BMI	0.012022	0
B201	B210	e-habits	Hip	0.017568	0
B201	B209	e-habits	Smoking	0.015281	0

B201	B207	e-habits	WHR	0.00654	1.13E-08
B201	B206	e-habits	Waist	0.022283	0
B201	B205	e-habits	KDA	0.024682	0
B201	B204	e-habits	Drinking	0.038322	0
B201	B203	e-habits	DH	0.041673	0
B201	B212	e-habits	IOP-L	0.008497	1.18E-13
B202	B223	e-times	FH	0.039708	0
B203	B224	DH	pmh	0.007099	5.72E-10
B203	B222	DH	NS	0.029124	0
B203	B221	DH	Hei	0.041701	0
B203	B219	DH	OT	-0.00898	4.44E-15
B203	B216	DH	BMI	0.032642	0
B203	B215	DH	BMI	0.013386	0
B203	B210	DH	Hip	0.031638	0
B203	B209	DH	Smoking	0.045301	0
B203	B206	DH	Waist	0.008631	4.88E-14
B203	B205	DH	KDA	0.007112	5.33E-10
B203	B204	DH	Drinking	0.051462	0
B204	B222	Drinking	NS	0.330087	0
B204	B221	Drinking	Hei	0.264183	0
B204	B220	Drinking	SBP	0.136096	0
B204	B219	Drinking	OT	-0.02692	0
B204	B218	Drinking	DBP	0.185393	0
B204	B216	Drinking	BMI	0.290784	0
B204	B215	Drinking	BMI	0.199569	0
B204	B210	Drinking	Hip	0.175366	0
B204	B209	Drinking	Smoking	0.396509	0
B204	B207	Drinking	WHR	0.033783	0
B204	B206	Drinking	Waist	0.283569	0
B204	B205	Drinking	KDA	-0.02106	0
B204	B211	Drinking	IOP-R	0.020188	0
B204	B212	Drinking	IOP-L	0.023211	0
B205	B224	KDA	pmh	0.015565	0
B205	B222	KDA	NS	-0.03031	0
B205	B221	KDA	Hei	-0.02989	0
B205	B219	KDA	OT	0.036333	0
B205	B218	KDA	DBP	-0.00934	4.44E-16
B205	B216	KDA	BMI	-0.02668	0
B205	B215	KDA	BMI	-0.01448	0
B205	B210	KDA	Hip	-0.01402	0
B205	B209	KDA	Smoking	-0.01209	0
B205	B206	KDA	Waist	-0.01379	0
B206	B224	Waist	pmh	0.013056	0
B206	B222	Waist	NS	0.740723	0

B206	B221	Waist	Hei	0.410046	0
B206	B227	Waist	OD	0.006603	8.19E-09
B206	B228	Waist	OS	0.006171	7.14E-08
B206	B220	Waist	SBP	0.363749	0
B206	B219	Waist	OT	0.023081	0
B206	B218	Waist	DBP	0.340036	0
B206	B216	Waist	BMI	0.852527	0
B206	B215	Waist	BMI	0.843667	0
B206	B210	Waist	Hip	0.757443	0
B206	B209	Waist	Smoking	0.256563	0
B206	B207	Waist	WHR	0.106494	0
B206	B211	Waist	IOP-R	0.032725	0
B206	B212	Waist	IOP-L	0.036407	0
B207	B222	WHR	NS	0.099593	0
B207	B221	WHR	Hei	0.032516	0
B207	B220	WHR	SBP	0.038853	0
B207	B219	WHR	OT	0.006154	7.74E-08
B207	B218	WHR	DBP	0.038459	0
B207	B216	WHR	BMI	0.076905	0
B207	B215	WHR	BMI	0.079402	0
B207	B210	WHR	Hip	0.046155	0
B207	B209	WHR	Smoking	0.03473	0
B209	B224	Smoking	pmh	0.009192	8.88E-16
B209	B222	Smoking	NS	0.286309	0
B209	B221	Smoking	Hei	0.214305	0
B209	B220	Smoking	SBP	0.09975	0
B209	B219	Smoking	OT	-0.00792	4.63E-12
B209	B218	Smoking	DBP	0.142455	0
B209	B216	Smoking	BMI	0.225963	0
B209	B215	Smoking	BMI	0.150541	0
B209	B210	Smoking	Hip	0.121901	0
B209	B211	Smoking	IOP-R	0.007627	2.77E-11
B209	B212	Smoking	IOP-L	0.011704	0
B21	B137	CD3+CD4-CD8-	AFU	-0.01045	0
B21	B23	CD3+CD4-CD8-	CD3-CD16+CD56+(NK)	-0.09983	0
B21	B22	CD3+CD4-CD8-	CD3+CD8+(T)	0.041037	0
B21	B54	CD3+CD4-CD8-	ESR	0.005958	1.98E-07
B21	B92	CD3+CD4-CD8-	ALDH2-Glu504Glu	-0.01449	0
B21	B93	CD3+CD4-CD8-	ALDH2-Glu504Lys	0.012923	0
B21	B126	CD3+CD4-CD8-	Alb	0.007522	5.14E-11
B21	B122	CD3+CD4-CD8-	C4	-0.00827	5.33E-13
B21	B189	CD3+CD4-CD8-	eGFB	0.00695	1.30E-09
B21	B193	CD3+CD4-CD8-	K	0.013068	0
B21	B95	CD3+CD4-CD8-	anti-cenpb	-0.01171	0

B21	B96	CD3+CD4-CD8-	dsDNA (IIF)	-0.00915	1.33E-15
B21	B97	CD3+CD4-CD8-	dsDNA	0.008035	2.30E-12
B21	B102	CD3+CD4-CD8-	anti-Sm	0.009223	8.88E-16
B21	B103	CD3+CD4-CD8-	anti-SSA/Ro	0.011031	0
B21	B107	CD3+CD4-CD8-	AnuA	-0.00646	1.72E-08
B21	B108	CD3+CD4-CD8-	Anti-Cyclin	0.020909	0
B21	B109	CD3+CD4-CD8-	AMA-M2	-0.0088	1.55E-14
B21	B198	CD3+CD4-CD8-	P	-0.00663	6.98E-09
B21	B194	CD3+CD4-CD8-	Cl	-0.02203	0
B21	B156	CD3+CD4-CD8-	IgE	-0.01082	0
B21	B157	CD3+CD4-CD8-	IgG	0.020038	0
B21	B158	CD3+CD4-CD8-	IgM	0.009069	2.44E-15
B21	B179	CD3+CD4-CD8-	α1-MG	-0.00614	8.46E-08
B21	B125	CD3+CD4-CD8-	Cor	0.019775	0
B21	B62	CD3+CD4-CD8-	WBHSRVI	-0.00658	9.03E-09
B21	B87	CD3+CD4-CD8-	NSE	-0.00625	4.95E-08
B21	B200	CD3+CD4-CD8-	OP	-0.00876	2.09E-14
B21	B215	CD3+CD4-CD8-	BMI	-0.00622	5.66E-08
B21	B209	CD3+CD4-CD8-	Smoking	-0.00711	5.34E-10
B21	B90	CD3+CD4-CD8-	CYFRA 21-1	-0.00699	1.02E-09
B21	B34	CD3+CD4-CD8-	TK	0.005942	2.13E-07
B21	B183	CD3+CD4-CD8-	PTA	-0.02051	0
B21	B185	CD3+CD4-CD8-	PT	0.011668	0
B21	B186	CD3+CD4-CD8-	PFC	0.012788	0
B21	B69	CD3+CD4-CD8-	PV	0.00922	8.88E-16
B21	B206	CD3+CD4-CD8-	Waist	-0.00657	9.69E-09
B21	B79	CD3+CD4-CD8-	hb V -dna	0.009423	2.22E-16
B21	B197	CD3+CD4-CD8-	AG	0.01394	0
B21	B204	CD3+CD4-CD8-	Drinking	-0.0065	1.41E-08
B21	B33	CD3+CD4-CD8-	Prog	0.008449	1.63E-13
B21	B74	CD3+CD4-CD8-	ApoA1	0.013082	0
B21	B75	CD3+CD4-CD8-	ApoA1/B	0.007037	8.06E-10
B21	B76	CD3+CD4-CD8-	ApoB	-0.0089	7.99E-15
B21	B91	CD3+CD4-CD8-	TP	0.006914	1.57E-09
B21	B114	CD3+CD4-CD8-	TSH TT3 TT4(TT4)	0.007751	1.31E-11
B210	B222	Hip	NS	0.617325	0
B210	B221	Hip	Hei	0.439634	0
B210	B220	Hip	SBP	0.244922	0
B210	B218	Hip	DBP	0.229459	0
B210	B216	Hip	BMI	0.799915	0
B210	B215	Hip	BMI	0.752018	0
B210	B211	Hip	IOP-R	0.026776	0
B210	B212	Hip	IOP-L	0.026189	0
B211	B222	IOP-R	NS	0.032754	0

B211	B221	IOP-R	Hei	0.01198	0
B211	B220	IOP-R	SBP	0.068813	0
B211	B218	IOP-R	DBP	0.068281	0
B211	B216	IOP-R	BMI	0.030509	0
B211	B215	IOP-R	BMI	0.031521	0
B211	B212	IOP-R	IOP-L	0.742305	0
B212	B222	IOP-L	NS	0.034459	0
B212	B221	IOP-L	Hei	0.009887	0
B212	B228	IOP-L	OS	0.007073	6.60E-10
B212	B220	IOP-L	SBP	0.070111	0
B212	B218	IOP-L	DBP	0.067882	0
B212	B216	IOP-L	BMI	0.029974	0
B212	B215	IOP-L	BMI	0.032483	0
B213	B214	CVA-R	CVA-L	0.08962	0
B213	B227	CVA-R	OD	0.012842	0
B213	B220	CVA-R	SBP	0.010456	0
B214	B220	CVA-L	SBP	0.008057	2.01E-12
B215	B222	BMI	NS	0.648673	0
B215	B221	BMI	Hei	0.175979	0
B215	B220	BMI	SBP	0.333062	0
B215	B219	BMI	OT	0.020493	0
B215	B218	BMI	DBP	0.314042	0
B215	B216	BMI	BMI	0.842498	0
B216	B222	BMI	NS	0.779174	0
B216	B221	BMI	Hei	0.667687	0
B216	B220	BMI	SBP	0.309844	0
B216	B219	BMI	OT	-0.02188	0
B216	B218	BMI	DBP	0.312086	0
B218	B222	DBP	NS	0.30803	0
B218	B221	DBP	Hei	0.143842	0
B218	B228	DBP	OS	0.006898	1.72E-09
B218	B220	DBP	SBP	0.737704	0
B218	B219	DBP	OT	0.021716	0
B219	B224	OT	pmh	0.040252	0
B219	B222	OT	NS	-0.02176	0
B219	B221	OT	Hei	-0.06766	0
B219	B227	OT	OD	0.005617	9.37E-07
B219	B228	OT	OS	0.006375	2.61E-08
B219	B220	OT	SBP	0.050604	0
B22	B23	CD3+CD8+(T)	CD3-CD16+CD56+(NK)	-0.26412	0
B22	B92	CD3+CD8+(T)	ALDH2-Glu504Glu	0.012588	0
B22	B93	CD3+CD8+(T)	ALDH2-Glu504Lys	-0.0151	0
B22	B94	CD3+CD8+(T)	ALDH2-Lys504Lys	0.006284	4.11E-08
B22	B124	CD3+CD8+(T)	ACTH	0.010334	0

B22	B57	CD3+CD8+(T)	RBCAI	-0.00764	2.51E-11
B22	B181	CD3+CD8+(T)	APTT	0.027746	0
B22	B25	CD3+CD8+(T)	CK	0.009525	0
B22	B129	CD3+CD8+(T)	IBIL	0.005737	5.49E-07
B22	B150	CD3+CD8+(T)	ALP	-0.00799	3.11E-12
B22	B99	CD3+CD8+(T)	anti-PM-Scl	-0.00849	1.26E-13
B22	B95	CD3+CD8+(T)	anti-cenpb	-0.00562	9.23E-07
B22	B98	CD3+CD8+(T)	anti-Jo-1	0.042048	0
B22	B101	CD3+CD8+(T)	anti-Sc70	-0.00671	4.79E-09
B22	B102	CD3+CD8+(T)	anti-Sm	-0.00583	3.55E-07
B22	B103	CD3+CD8+(T)	anti-SSA/Ro	0.013149	0
B22	B106	CD3+CD8+(T)	anti-P	-0.01434	0
B22	B151	CD3+CD8+(T)	Anti-CCP	-0.01675	0
B22	B108	CD3+CD8+(T)	Anti-Cyclin	-0.01034	0
B22	B157	CD3+CD8+(T)	IgG	0.017454	0
B22	B195	CD3+CD8+(T)	Na	-0.00745	7.79E-11
B22	B178	CD3+CD8+(T)	Ig-U	-0.00577	4.62E-07
B22	B180	CD3+CD8+(T)	Tf	-0.00568	6.99E-07
B22	B182	CD3+CD8+(T)	TT	-0.01054	0
B22	B125	CD3+CD8+(T)	Cor	0.018996	0
B22	B62	CD3+CD8+(T)	WBHSRVI	0.011361	0
B22	B64	CD3+CD8+(T)	WBRSR(mPas_5)	0.007515	5.34E-11
B22	B66	CD3+CD8+(T)	WBRSR(mPas_100)	0.012206	0
B22	B67	CD3+CD8+(T)	WBRSR(mPas_50)	0.01197	0
B22	B209	CD3+CD8+(T)	Smoking	-0.00662	7.42E-09
B22	B34	CD3+CD8+(T)	TK	-0.00715	4.28E-10
B22	B35	CD3+CD8+(T)	β2-MG	0.006625	7.31E-09
B22	B183	CD3+CD8+(T)	PTA	0.030156	0
B22	B184	CD3+CD8+(T)	INR	0.007243	2.56E-10
B22	B185	CD3+CD8+(T)	PT	0.008826	1.31E-14
B22	B186	CD3+CD8+(T)	PFC	-0.01456	0
B22	B197	CD3+CD8+(T)	AG	-0.00995	0
B22	B74	CD3+CD8+(T)	ApoA1	-0.01291	0
B22	B81	CD3+CD8+(T)	TSGF	-0.0075	5.69E-11
B22	B114	CD3+CD8+(T)	TSH TT3 TT4(TT4)	-0.01285	0
B220	B225	SBP	hPBG	0.011394	0
B220	B222	SBP	NS	0.305004	0
B220	B221	SBP	Hei	0.116148	0
B220	B227	SBP	OD	0.014962	0
B220	B228	SBP	OS	0.017161	0
B221	B222	Hei	NS	0.546121	0
B221	B227	Hei	OD	-0.00629	4.07E-08
B222	B224	NS	pmh	0.010065	0
B227	B228	OD	OS	0.12255	0

B23	B24	CD3-CD16+CD56+(NK)	αHBDH	0.008182	9.09E-13
B23	B92	CD3-CD16+CD56+(NK)	ALDH2-Glu504Glu	0.010913	0
B23	B93	CD3-CD16+CD56+(NK)	ALDH2-Glu504Lys	-0.0069	1.67E-09
B23	B94	CD3-CD16+CD56+(NK)	ALDH2-Lys504Lys	-0.0119	0
B23	B143	CD3-CD16+CD56+(NK)	β-CTx	-0.0067	4.92E-09
B23	B126	CD3-CD16+CD56+(NK)	Alb	0.009537	0
B23	B121	CD3-CD16+CD56+(NK)	C3	-0.02015	0
B23	B122	CD3-CD16+CD56+(NK)	C4	0.006095	1.03E-07
B23	B124	CD3-CD16+CD56+(NK)	ACTH	0.048086	0
B23	B71	CD3-CD16+CD56+(NK)	TG	-0.00577	4.67E-07
B23	B55	CD3-CD16+CD56+(NK)	TK	-0.00847	1.45E-13
B23	B57	CD3-CD16+CD56+(NK)	RBCAI	0.012698	0
B23	B46	CD3-CD16+CD56+(NK)	RBC Count	0.008581	6.79E-14
B23	B181	CD3-CD16+CD56+(NK)	APTT	0.007469	7.00E-11
B23	B190	CD3-CD16+CD56+(NK)	Cre	0.005776	4.59E-07
B23	B193	CD3-CD16+CD56+(NK)	K	0.027071	0
B23	B129	CD3-CD16+CD56+(NK)	IBIL	0.006883	1.86E-09
B23	B150	CD3-CD16+CD56+(NK)	ALP	0.007124	5.00E-10
B23	B105	CD3-CD16+CD56+(NK)	anti-U1RNP	0.024392	0
B23	B95	CD3-CD16+CD56+(NK)	anti-cenpb	0.00678	3.23E-09
B23	B96	CD3-CD16+CD56+(NK)	dsDNA (IIF)	-0.00674	3.95E-09
B23	B98	CD3-CD16+CD56+(NK)	anti-Jo-1	0.006135	8.51E-08
B23	B100	CD3-CD16+CD56+(NK)	anti-Ro52	-0.01648	0
B23	B101	CD3-CD16+CD56+(NK)	anti-Sc70	0.020854	0
B23	B102	CD3-CD16+CD56+(NK)	anti-Sm	0.011051	0
B23	B103	CD3-CD16+CD56+(NK)	anti-SSA/Ro	-0.01587	0
B23	B104	CD3-CD16+CD56+(NK)	anti-SSB	-0.01724	0
B23	B107	CD3-CD16+CD56+(NK)	AnuA	-0.00707	6.60E-10
B23	B151	CD3-CD16+CD56+(NK)	Anti-CCP	0.020358	0
B23	B108	CD3-CD16+CD56+(NK)	Anti-Cyclin	0.007512	5.45E-11
B23	B154	CD3-CD16+CD56+(NK)	RF	0.009043	2.89E-15
B23	B198	CD3-CD16+CD56+(NK)	P	-0.05133	0
B23	B194	CD3-CD16+CD56+(NK)	Cl	-0.02324	0
B23	B199	CD3-CD16+CD56+(NK)	Mg	0.010157	0
B23	B155	CD3-CD16+CD56+(NK)	IgA	0.030396	0
B23	B156	CD3-CD16+CD56+(NK)	IgE	0.012411	0
B23	B157	CD3-CD16+CD56+(NK)	IgG	0.030046	0
B23	B195	CD3-CD16+CD56+(NK)	Na	0.01918	0
B23	B168	CD3-CD16+CD56+(NK)	NMP22	0.006306	3.69E-08
B23	B178	CD3-CD16+CD56+(NK)	Ig-U	0.008853	1.09E-14
B23	B179	CD3-CD16+CD56+(NK)	α1-MG	0.010753	0
B23	B180	CD3-CD16+CD56+(NK)	Tf	0.007669	2.15E-11
B23	B182	CD3-CD16+CD56+(NK)	TT	0.030419	0
B23	B125	CD3-CD16+CD56+(NK)	Cor	0.032065	0

B23	B130	CD3-CD16+CD56+(NK)	GLB	0.005762	4.88E-07
B23	B59	CD3-CD16+CD56+(NK)	WBRV	0.010434	0
B23	B60	CD3-CD16+CD56+(NK)	WBRVI	0.014656	0
B23	B62	CD3-CD16+CD56+(NK)	WBHSRVI	0.00798	3.24E-12
B23	B64	CD3-CD16+CD56+(NK)	WBRSR(mPas_5)	0.016708	0
B23	B65	CD3-CD16+CD56+(NK)	WBRSR(mPas_1)	0.018271	0
B23	B66	CD3-CD16+CD56+(NK)	WBRSR(mPas_100)	0.014156	0
B23	B67	CD3-CD16+CD56+(NK)	WBRSR(mPas_50)	0.014456	0
B23	B27	CD3-CD16+CD56+(NK)	LDH	0.006451	1.78E-08
B23	B200	CD3-CD16+CD56+(NK)	OP	0.023482	0
B23	B220	CD3-CD16+CD56+(NK)	SBP	0.009451	0
B23	B218	CD3-CD16+CD56+(NK)	DBP	0.007786	1.06E-11
B23	B209	CD3-CD16+CD56+(NK)	Smoking	-0.01391	0
B23	B90	CD3-CD16+CD56+(NK)	CYFRA 21-1	-0.00665	6.46E-09
B23	B139	CD3-CD16+CD56+(NK)	ADA	0.020083	0
B23	B35	CD3-CD16+CD56+(NK)	β2-MG	-0.01932	0
B23	B196	CD3-CD16+CD56+(NK)	TCO2	0.011944	0
B23	B51	CD3-CD16+CD56+(NK)	Hb	0.005678	7.16E-07
B23	B183	CD3-CD16+CD56+(NK)	PTA	-0.01266	0
B23	B185	CD3-CD16+CD56+(NK)	PT	0.008638	4.62E-14
B23	B186	CD3-CD16+CD56+(NK)	PFC	0.009359	4.44E-16
B23	B69	CD3-CD16+CD56+(NK)	PV	0.011373	0
B23	B197	CD3-CD16+CD56+(NK)	AG	0.016831	0
B23	B33	CD3-CD16+CD56+(NK)	Prog	0.006046	1.30E-07
B23	B73	CD3-CD16+CD56+(NK)	ApoA	0.006087	1.07E-07
B23	B74	CD3-CD16+CD56+(NK)	ApoA1	0.016133	0
B23	B132	CD3-CD16+CD56+(NK)	Dbil	0.008081	1.73E-12
B23	B133	CD3-CD16+CD56+(NK)	TBIL	0.007702	1.76E-11
B23	B91	CD3-CD16+CD56+(NK)	TP	0.010834	0
B24	B137	αHBDH	AFU	0.006143	8.18E-08
B24	B54	αHBDH	ESR	0.062313	0
B24	B160	αHBDH	PH	-0.013	0
B24	B140	αHBDH	r-GT	0.022478	0
B24	B118	αHBDH	CEA1	0.011736	0
B24	B126	αHBDH	Alb	0.010663	0
B24	B127	αHBDH	A-G ratio	-0.0201	0
B24	B161	αHBDH	LEU	0.01085	0
B24	B128	αHBDH	ALT	0.05181	0
B24	B123	αHBDH	hs-CRP1	0.08152	0
B24	B111	αHBDH	TSH	0.017826	0
B24	B124	αHBDH	ACTH	0.009755	0
B24	B42	αHBDH	MON	0.007275	2.14E-10
B24	B165	αHBDH	bilirubin	0.008765	1.98E-14
B24	B70	αHBDH	LDL-C	0.02855	0

B24	B38	αHBDH	BAND%	0.015581	0
B24	B72	αHBDH	HDL-C	0.02477	0
B24	B189	αHBDH	eGFB	-0.03468	0
B24	B141	αHBDH	AST/ALT	0.008785	1.73E-14
B24	B146	αHBDH	CysC	0.012726	0
B24	B43	αHBDH	MCV	0.011396	0
B24	B44	αHBDH	MCH	0.017836	0
B24	B46	αHBDH	RBC Count	-0.01009	0
B24	B47	αHBDH	RDW	0.037417	0
B24	B181	αHBDH	APTT	-0.00702	8.99E-10
B24	B25	αHBDH	CK	0.133739	0
B24	B26	αHBDH	CKMB	0.225009	0
B24	B193	αHBDH	K	0.01701	0
B24	B129	αHBDH	IBIL	0.010874	0
B24	B150	αHBDH	ALP	0.026816	0
B24	B222	αHBDH	NS	-0.0061	1.03E-07
B24	B98	αHBDH	anti-Jo-1	0.008339	3.32E-13
B24	B151	αHBDH	Anti-CCP	0.00668	5.46E-09
B24	B194	αHBDH	Cl	-0.00746	7.40E-11
B24	B199	αHBDH	Mg	0.010895	0
B24	B157	αHBDH	IgG	0.007123	5.00E-10
B24	B195	αHBDH	Na	0.012793	0
B24	B176	αHBDH	Ucr	-0.0124	0
B24	B191	αHBDH	Urea	0.021329	0
B24	B174	αHBDH	Urea/Cr	0.018442	0
B24	B175	αHBDH	ALB-U	0.007957	3.73E-12
B24	B177	αHBDH	ACR	0.012774	0
B24	B179	αHBDH	α1-MG	0.01516	0
B24	B182	αHBDH	TT	0.006498	1.40E-08
B24	B125	αHBDH	Cor	0.015138	0
B24	B138	αHBDH	PAB	-0.0118	0
B24	B130	αHBDH	GLB	0.03306	0
B24	B59	αHBDH	WBRV	0.016313	0
B24	B61	αHBDH	WBHSRV	0.014869	0
B24	B64	αHBDH	WBRSR(mPas_5)	0.018857	0
B24	B65	αHBDH	WBRSR(mPas_1)	0.017375	0
B24	B66	αHBDH	WBRSR(mPas_100)	0.020543	0
B24	B67	αHBDH	WBRSR(mPas_50)	0.021081	0
B24	B27	αHBDH	LDH	0.723643	0
B24	B221	αHBDH	Hei	-0.03526	0
B24	B87	αHBDH	NSE	0.026032	0
B24	B187	αHBDH	NSE	0.011473	0
B24	B200	αHBDH	OP	0.016147	0
B24	B228	αHBDH	OS	0.005862	3.10E-07

B24	B50	αHBDH	eo%	0.008694	3.20E-14
B24	B220	αHBDH	SBP	0.048447	0
B24	B219	αHBDH	OT	0.008453	1.59E-13
B24	B218	αHBDH	DBP	0.031595	0
B24	B215	αHBDH	BMI	0.020568	0
B24	B131	αHBDH	AST	0.096301	0
B24	B210	αHBDH	Hip	0.009378	2.22E-16
B24	B209	αHBDH	Smoking	-0.01718	0
B24	B90	αHBDH	CYFRA 21-1	0.010968	0
B24	B139	αHBDH	ADA	0.056551	0
B24	B183	αHBDH	PTA	-0.02035	0
B24	B185	αHBDH	PT	0.016033	0
B24	B186	αHBDH	PFC	0.016409	0
B24	B69	αHBDH	PV	0.045373	0
B24	B52	αHBDH	PLT/BPC	-0.02835	0
B24	B173	αHBDH	NTT	0.009923	0
B24	B206	αHBDH	Waist	0.015082	0
B24	B197	αHBDH	AG	0.009828	0
B24	B204	αHBDH	Drinking	-0.01652	0
B24	B112	αHBDH	FT4	0.010207	0
B24	B113	αHBDH	FT3	0.007331	1.55E-10
B24	B211	αHBDH	IOP-R	-0.007	1.00E-09
B24	B73	αHBDH	ApoA	0.017158	0
B24	B74	αHBDH	ApoA1	0.064402	0
B24	B75	αHBDH	ApoA1/B	0.009231	8.88E-16
B24	B76	αHBDH	ApoB	0.021186	0
B24	B132	αHBDH	Dbil	0.013025	0
B24	B81	αHBDH	TSGF	-0.01653	0
B24	B77	αHBDH	TC	0.029186	0
B24	B133	αHBDH	TBIL	0.012071	0
B24	B91	αHBDH	TP	0.034851	0
B24	B114	αHBDH	TSH TT3 TT4(TT4)	0.037439	0
B24	B212	αHBDH	IOP-L	-0.00853	9.30E-14
B25	B54	CK	ESR	-0.00845	1.66E-13
B25	B160	CK	PH	0.006079	1.11E-07
B25	B126	CK	Alb	0.008493	1.22E-13
B25	B127	CK	A-G ratio	0.005639	8.50E-07
B25	B128	CK	ALT	0.02272	0
B25	B134	CK	CHE	0.005941	2.13E-07
B25	B70	CK	LDL-C	0.009051	2.66E-15
B25	B141	CK	AST/ALT	0.007586	3.52E-11
B25	B45	CK	MCHC	0.007278	2.10E-10
B25	B46	CK	RBC Count	0.009968	0
B25	B190	CK	Cre	0.01507	0

B25	B26	CK	CKMB	0.358423	0
B25	B129	CK	IBIL	0.006719	4.46E-09
B25	B222	CK	NS	0.013682	0
B25	B176	CK	Ucr	0.006555	1.05E-08
B25	B174	CK	Urea/Cr	-0.007	9.75E-10
B25	B192	CK	UA	0.014845	0
B25	B138	CK	PAB	0.005966	1.91E-07
B25	B60	CK	WBRVI	0.009676	0
B25	B62	CK	WBHSRVI	0.00799	3.04E-12
B25	B64	CK	WBRSR(mPas_5)	0.008592	6.31E-14
B25	B65	CK	WBRSR(mPas_1)	0.008944	5.77E-15
B25	B66	CK	WBRSR(mPas_100)	0.007285	2.01E-10
B25	B67	CK	WBRSR(mPas_50)	0.007587	3.51E-11
B25	B27	CK	LDH	0.232108	0
B25	B221	CK	Hei	0.016422	0
B25	B87	CK	NSE	0.010643	0
B25	B216	CK	BMI	0.01487	0
B25	B215	CK	BMI	0.007291	1.95E-10
B25	B131	CK	AST	0.086066	0
B25	B210	CK	Hip	0.008403	2.20E-13
B25	B51	CK	Hb	0.011686	0
B25	B206	CK	Waist	0.00819	8.67E-13
B25	B204	CK	Drinking	0.0079	5.30E-12
B25	B77	CK	TC	0.006485	1.50E-08
B25	B133	CK	TBIL	0.006056	1.24E-07
B25	B114	CK	TSH TT3 TT4(TT4)	-0.00648	1.56E-08
B26	B54	CKMB	ESR	0.012293	0
B26	B140	CKMB	r-GT	0.006976	1.12E-09
B26	B118	CKMB	CEA1	0.00634	3.10E-08
B26	B127	CKMB	A-G ratio	-0.00898	4.44E-15
B26	B128	CKMB	ALT	0.015306	0
B26	B123	CKMB	hs-CRP1	0.048385	0
B26	B189	CKMB	eGFB	-0.00618	6.70E-08
B26	B55	CKMB	TK	-0.00768	2.03E-11
B26	B56	CKMB	IR	0.009966	0
B26	B46	CKMB	RBC Count	0.012761	0
B26	B193	CKMB	K	0.010617	0
B26	B130	CKMB	GLB	0.011903	0
B26	B59	CKMB	WBRV	0.006739	4.03E-09
B26	B60	CKMB	WBRVI	0.019938	0
B26	B61	CKMB	WBHSRV	0.013575	0
B26	B62	CKMB	WBHSRVI	0.023408	0
B26	B64	CKMB	WBRSR(mPas_5)	0.025962	0
B26	B65	CKMB	WBRSR(mPas_1)	0.023548	0

B26	B66	CKMB	WBRSR(mPas_100)	0.027319	0
B26	B67	CKMB	WBRSR(mPas_50)	0.027183	0
B26	B27	CKMB	LDH	0.219087	0
B26	B87	CKMB	NSE	0.013491	0
B26	B220	CKMB	SBP	0.011211	0
B26	B218	CKMB	DBP	0.008327	3.59E-13
B26	B131	CKMB	AST	0.048743	0
B26	B90	CKMB	CYFRA 21-1	0.011904	0
B26	B139	CKMB	ADA	0.01527	0
B26	B51	CKMB	Hb	0.01547	0
B26	B69	CKMB	PV	0.009481	0
B26	B52	CKMB	PLT/BPC	0.007191	3.42E-10
B26	B197	CKMB	AG	0.005844	3.36E-07
B26	B113	CKMB	FT3	0.005731	5.63E-07
B26	B201	CKMB	e-habits	0.007185	3.55E-10
B26	B135	CKMB	TBA	0.007699	1.80E-11
B26	B91	CKMB	TP	0.007827	8.27E-12
B26	B114	CKMB	TSH TT3 TT4(TT4)	0.006286	4.07E-08
B27	B136	LDH	5'NT	0.00686	2.11E-09
B27	B137	LDH	AFU	0.01497	0
B27	B54	LDH	ESR	0.045281	0
B27	B160	LDH	PH	-0.01449	0
B27	B140	LDH	r-GT	0.061568	0
B27	B118	LDH	CEA1	0.013925	0
B27	B126	LDH	Alb	0.012686	0
B27	B127	LDH	A-G ratio	-0.02401	0
B27	B161	LDH	LEU	0.007051	7.48E-10
B27	B40	LDH	WBC	0.015275	0
B27	B128	LDH	ALT	0.124116	0
B27	B123	LDH	hs-CRP1	0.078681	0
B27	B29	LDH	E2	-0.00567	7.41E-07
B27	B30	LDH	LH	0.007625	2.79E-11
B27	B111	LDH	TSH	0.019706	0
B27	B31	LDH	FSH	0.009738	0
B27	B124	LDH	ACTH	0.011395	0
B27	B165	LDH	bilirubin	0.020175	0
B27	B134	LDH	CHE	0.059152	0
B27	B166	LDH	Pro	0.007653	2.37E-11
B27	B70	LDH	LDL-C	0.045602	0
B27	B71	LDH	TG	0.026049	0
B27	B38	LDH	BAND%	0.009878	0
B27	B72	LDH	HDL-C	0.010474	0
B27	B189	LDH	eGFB	-0.06065	0
B27	B141	LDH	AST/ALT	-0.0078	9.68E-12

B27	B146	LDH	CysC	0.01635	0
B27	B55	LDH	TK	-0.00572	6.03E-07
B27	B56	LDH	IR	0.008184	8.97E-13
B27	B43	LDH	MCV	0.012954	0
B27	B44	LDH	MCH	0.027212	0
B27	B45	LDH	MCHC	0.017473	0
B27	B46	LDH	RBC Count	0.013333	0
B27	B47	LDH	RDW	0.037852	0
B27	B181	LDH	APTT	-0.01013	0
B27	B190	LDH	Cre	0.027953	0
B27	B193	LDH	K	0.022272	0
B27	B129	LDH	IBIL	0.033854	0
B27	B150	LDH	ALP	0.120096	0
B27	B222	LDH	NS	0.028986	0
B27	B99	LDH	anti-PM-Scl	0.007358	1.33E-10
B27	B98	LDH	anti-Jo-1	0.006707	4.76E-09
B27	B104	LDH	anti-SSB	-0.00566	7.69E-07
B27	B108	LDH	Anti-Cyclin	0.007766	1.20E-11
B27	B199	LDH	Mg	0.022977	0
B27	B157	LDH	IgG	0.007337	1.50E-10
B27	B195	LDH	Na	0.024814	0
B27	B191	LDH	Urea	0.028981	0
B27	B174	LDH	Urea/Cr	0.007341	1.46E-10
B27	B192	LDH	UA	0.03495	0
B27	B175	LDH	ALB-U	0.01006	0
B27	B177	LDH	ACR	0.014468	0
B27	B179	LDH	α1-MG	0.014703	0
B27	B125	LDH	Cor	0.016643	0
B27	B130	LDH	GLB	0.039367	0
B27	B59	LDH	WBRV	0.017522	0
B27	B60	LDH	WBRVI	0.01446	0
B27	B61	LDH	WBHSRV	0.02529	0
B27	B62	LDH	WBHSRVI	0.01962	0
B27	B64	LDH	WBRSR(mPas_5)	0.03544	0
B27	B65	LDH	WBRSR(mPas_1)	0.030886	0
B27	B66	LDH	WBRSR(mPas_100)	0.037791	0
B27	B67	LDH	WBRSR(mPas_50)	0.037787	0
B27	B221	LDH	Hei	-0.01792	0
B27	B87	LDH	NSE	0.034266	0
B27	B187	LDH	NSE	0.013552	0
B27	B200	LDH	OP	0.028904	0
B27	B50	LDH	eo%	0.015778	0
B27	B220	LDH	SBP	0.070859	0
B27	B219	LDH	OT	0.013561	0

B27	B218	LDH	DBP	0.054241	0
B27	B216	LDH	BMI	0.03423	0
B27	B215	LDH	BMI	0.059159	0
B27	B131	LDH	AST	0.189361	0
B27	B210	LDH	Hip	0.035449	0
B27	B90	LDH	CYFRA 21-1	0.010114	0
B27	B139	LDH	ADA	0.122438	0
B27	B152	LDH	ACA-Ig(G-A-M)	0.008601	5.95E-14
B27	B196	LDH	TCO2	-0.0068	2.98E-09
B27	B51	LDH	Hb	0.034384	0
B27	B185	LDH	PT	0.010929	0
B27	B186	LDH	PFC	0.015157	0
B27	B69	LDH	PV	0.047329	0
B27	B52	LDH	PLT/BPC	-0.02608	0
B27	B173	LDH	NTT	0.007517	5.29E-11
B27	B207	LDH	WHR	0.006033	1.39E-07
B27	B206	LDH	Waist	0.052743	0
B27	B79	LDH	hb V -dna	0.009578	0
B27	B78	LDH	HBcAb	0.008002	2.82E-12
B27	B197	LDH	AG	0.019048	0
B27	B112	LDH	FT4	0.010972	0
B27	B113	LDH	FT3	0.009722	0
B27	B73	LDH	ApoA	0.014827	0
B27	B74	LDH	ApoA1	0.037812	0
B27	B76	LDH	ApoB	0.046704	0
B27	B132	LDH	Dbil	0.022828	0
B27	B81	LDH	TSGF	-0.0134	0
B27	B77	LDH	TC	0.046114	0
B27	B133	LDH	TBIL	0.032974	0
B27	B135	LDH	TBA	0.028086	0
B27	B91	LDH	TP	0.041433	0
B27	B114	LDH	TSH TT3 TT4(TT4)	0.031	0
B27	B115	LDH	TSH TT3 TT4(TT3)	0.009909	0
B27	B144	LDH	Total-PINP	0.008257	5.63E-13
B28	B29	PRL	E2	0.203584	0
B28	B30	PRL	LH	-0.12281	0
B28	B111	PRL	TSH	0.006666	5.89E-09
B28	B31	PRL	FSH	-0.18198	0
B28	B32	PRL	Testo	-0.03593	0
B28	B150	PRL	ALP	-0.00641	2.23E-08
B28	B105	PRL	anti-U1RNP	0.012887	0
B28	B100	PRL	anti-Ro52	0.005779	4.53E-07
B28	B102	PRL	anti-Sm	0.009847	0
B28	B103	PRL	anti-SSA/Ro	0.018653	0

B28	B195	PRL	Na	-0.00669	5.29E-09
B28	B125	PRL	Cor	0.00562	9.25E-07
B28	B200	PRL	OP	-0.00668	5.48E-09
B28	B33	PRL	Prog	0.114036	0
B29	B126	E2	Alb	-0.0078	9.67E-12
B29	B40	E2	WBC	0.007317	1.68E-10
B29	B30	E2	LH	-0.15742	0
B29	B31	E2	FSH	-0.41511	0
B29	B134	E2	CHE	-0.0077	1.79E-11
B29	B70	E2	LDL-C	-0.00759	3.41E-11
B29	B32	E2	Testo	-0.07899	0
B29	B141	E2	AST/ALT	0.00586	3.12E-07
B29	B150	E2	ALP	-0.01132	0
B29	B101	E2	anti-Sc70	0.010684	0
B29	B109	E2	AMA-M2	0.008358	2.94E-13
B29	B198	E2	P	-0.01532	0
B29	B199	E2	Mg	-0.00638	2.51E-08
B29	B195	E2	Na	-0.01497	0
B29	B191	E2	Urea	-0.0079	5.21E-12
B29	B192	E2	UA	-0.00573	5.69E-07
B29	B200	E2	OP	-0.01535	0
B29	B206	E2	Waist	-0.00562	9.36E-07
B29	B33	E2	Prog	0.250751	0
B29	B76	E2	ApoB	-0.00901	3.77E-15
B29	B77	E2	TC	-0.00615	7.89E-08
B29	B91	E2	TP	-0.00605	1.27E-07
B3	B137	Hcy	AFU	0.016505	0
B3	B24	Hcy	αHBDH	0.035038	0
B3	B21	Hcy	CD3+CD4-CD8-	-0.00777	1.16E-11
B3	B54	Hcy	ESR	-0.01986	0
B3	B160	Hcy	PH	-0.02279	0
B3	B140	Hcy	r-GT	0.04459	0
B3	B143	Hcy	β-CTx	0.00643	1.98E-08
B3	B118	Hcy	CEA1	0.007407	1.00E-10
B3	B126	Hcy	Alb	0.027731	0
B3	B127	Hcy	A-G ratio	0.02291	0
B3	B161	Hcy	LEU	-0.01247	0
B3	B40	Hcy	WBC	0.035031	0
B3	B128	Hcy	ALT	0.031564	0
B3	B123	Hcy	hs-CRP1	0.031213	0
B3	B124	Hcy	ACTH	0.024666	0
B3	B41	Hcy	MON％	0.005886	2.77E-07
B3	B134	Hcy	CHE	0.01948	0
B3	B166	Hcy	Pro	0.028372	0

B3	B70	Hcy	LDL-C	0.022399	0
B3	B188	Hcy	Ca	0.006765	3.50E-09
B3	B71	Hcy	TG	0.028893	0
B3	B72	Hcy	HDL-C	-0.05307	0
B3	B32	Hcy	Testo	0.005746	5.26E-07
B3	B189	Hcy	eGFB	-0.07711	0
B3	B141	Hcy	AST/ALT	-0.02717	0
B3	B145	Hcy	BGP	0.005653	8.00E-07
B3	B146	Hcy	CysC	0.025888	0
B3	B55	Hcy	TK	-0.00569	6.92E-07
B3	B56	Hcy	IR	0.029144	0
B3	B57	Hcy	RBCAI	0.011902	0
B3	B43	Hcy	MCV	0.010792	0
B3	B44	Hcy	MCH	0.035553	0
B3	B45	Hcy	MCHC	0.049852	0
B3	B46	Hcy	RBC Count	0.076112	0
B3	B47	Hcy	RDW	0.018916	0
B3	B58	Hcy	HCT	0.006348	2.99E-08
B3	B181	Hcy	APTT	-0.00676	3.68E-09
B3	B190	Hcy	Cre	0.120071	0
B3	B25	Hcy	CK	0.015207	0
B3	B26	Hcy	CKMB	0.012352	0
B3	B193	Hcy	K	-0.00964	0
B3	B129	Hcy	IBIL	0.022124	0
B3	B150	Hcy	ALP	0.041295	0
B3	B84	Hcy	C-PSA	0.019857	0
B3	B222	Hcy	NS	0.090289	0
B3	B148	Hcy	Anti_TPO	-0.00845	1.59E-13
B3	B149	Hcy	TGAb	-0.01164	0
B3	B198	Hcy	P	-0.02322	0
B3	B85	Hcy	SCC	0.011352	0
B3	B194	Hcy	Cl	-0.00859	6.42E-14
B3	B199	Hcy	Mg	0.010092	0
B3	B157	Hcy	IgG	-0.01291	0
B3	B158	Hcy	IgM	-0.00687	2.02E-09
B3	B195	Hcy	Na	0.02506	0
B3	B176	Hcy	Ucr	0.060897	0
B3	B191	Hcy	Urea	0.037869	0
B3	B174	Hcy	Urea/Cr	-0.05401	0
B3	B192	Hcy	UA	0.099978	0
B3	B175	Hcy	ALB-U	0.013722	0
B3	B177	Hcy	ACR	0.006798	2.94E-09
B3	B179	Hcy	α1-MG	0.01148	0
B3	B182	Hcy	TT	0.018626	0

B3	B125	Hcy	Cor	0.022	0
B3	B138	Hcy	PAB	0.03758	0
B3	B130	Hcy	GLB	-0.01379	0
B3	B59	Hcy	WBRV	0.017776	0
B3	B60	Hcy	WBRVI	0.050513	0
B3	B61	Hcy	WBHSRV	0.028567	0
B3	B62	Hcy	WBHSRVI	0.052256	0
B3	B64	Hcy	WBRSR(mPas_5)	0.051486	0
B3	B65	Hcy	WBRSR(mPas_1)	0.048881	0
B3	B66	Hcy	WBRSR(mPas_100)	0.050854	0
B3	B67	Hcy	WBRSR(mPas_50)	0.051787	0
B3	B86	Hcy	TBhCG	-0.00803	2.38E-12
B3	B27	Hcy	LDH	0.045873	0
B3	B221	Hcy	Hei	0.085687	0
B3	B200	Hcy	OP	0.02482	0
B3	B50	Hcy	eo%	0.01758	0
B3	B220	Hcy	SBP	0.048104	0
B3	B219	Hcy	OT	-0.00746	7.57E-11
B3	B218	Hcy	DBP	0.049028	0
B3	B88	Hcy	CA 242	-0.01	0
B3	B216	Hcy	BMI	0.079837	0
B3	B215	Hcy	BMI	0.045414	0
B3	B131	Hcy	AST	0.024822	0
B3	B210	Hcy	Hip	0.050658	0
B3	B10	Hcy	PG I	0.021548	0
B3	B12	Hcy	PG II	0.017698	0
B3	B14	Hcy	G-17	0.011367	0
B3	B209	Hcy	Smoking	0.076504	0
B3	B90	Hcy	CYFRA 21-1	0.036777	0
B3	B139	Hcy	ADA	0.00689	1.80E-09
B3	B34	Hcy	TK	-0.00835	3.15E-13
B3	B51	Hcy	Hb	0.103389	0
B3	B183	Hcy	PTA	-0.00695	1.29E-09
B3	B52	Hcy	PLT/BPC	-0.01679	0
B3	B173	Hcy	NTT	-0.00618	6.94E-08
B3	B207	Hcy	WHR	0.008245	6.08E-13
B3	B206	Hcy	Waist	0.073933	0
B3	B205	Hcy	KDA	-0.00601	1.55E-07
B3	B197	Hcy	AG	0.021169	0
B3	B204	Hcy	Drinking	0.059746	0
B3	B80	Hcy	anti-HP IgG	0.007352	1.37E-10
B3	B112	Hcy	FT4	0.010549	0
B3	B113	Hcy	FT3	0.016301	0
B3	B73	Hcy	ApoA	-0.00725	2.50E-10

B3	B74	Hcy	ApoA1	-0.03897	0
B3	B75	Hcy	ApoA1/B	-0.02252	0
B3	B76	Hcy	ApoB	0.025345	0
B3	B132	Hcy	Dbil	0.023961	0
B3	B81	Hcy	TSGF	0.006291	3.97E-08
B3	B133	Hcy	TBIL	0.024054	0
B3	B135	Hcy	TBA	0.010138	0
B3	B115	Hcy	TSH TT3 TT4(TT3)	0.00749	6.19E-11
B3	B144	Hcy	Total-PINP	0.006658	6.15E-09
B30	B40	LH	WBC	-0.00784	7.75E-12
B30	B31	LH	FSH	0.80422	0
B30	B134	LH	CHE	0.011506	0
B30	B70	LH	LDL-C	0.007591	3.42E-11
B30	B72	LH	HDL-C	0.008656	4.13E-14
B30	B32	LH	Testo	-0.12477	0
B30	B189	LH	eGFB	-0.01049	0
B30	B145	LH	BGP	0.005632	8.80E-07
B30	B150	LH	ALP	0.012699	0
B30	B100	LH	anti-Ro52	-0.00575	5.13E-07
B30	B101	LH	anti-Sc70	-0.0078	9.90E-12
B30	B102	LH	anti-Sm	-0.00836	2.90E-13
B30	B108	LH	Anti-Cyclin	0.015656	0
B30	B198	LH	P	0.011728	0
B30	B199	LH	Mg	0.008257	5.65E-13
B30	B156	LH	IgE	-0.00666	5.99E-09
B30	B195	LH	Na	0.017205	0
B30	B200	LH	OP	0.014623	0
B30	B139	LH	ADA	0.007147	4.39E-10
B30	B112	LH	FT4	0.005931	2.24E-07
B30	B33	LH	Prog	-0.32409	0
B30	B74	LH	ApoA1	0.012199	0
B30	B77	LH	TC	0.010653	0
B30	B114	LH	TSH TT3 TT4(TT4)	0.006753	3.74E-09
B31	B40	FSH	WBC	-0.01063	0
B31	B134	FSH	CHE	0.013394	0
B31	B70	FSH	LDL-C	0.0097	0
B31	B72	FSH	HDL-C	0.007178	3.68E-10
B31	B32	FSH	Testo	-0.10966	0
B31	B189	FSH	eGFB	-0.01351	0
B31	B145	FSH	BGP	0.006421	2.07E-08
B31	B150	FSH	ALP	0.017181	0
B31	B101	FSH	anti-Sc70	-0.00789	5.52E-12
B31	B103	FSH	anti-SSA/Ro	-0.00626	4.56E-08
B31	B108	FSH	Anti-Cyclin	0.00692	1.53E-09

B31	B198	FSH	P	0.019797	0
B31	B194	FSH	Cl	0.007901	5.27E-12
B31	B199	FSH	Mg	0.009282	4.44E-16
B31	B156	FSH	IgE	-0.0069	1.72E-09
B31	B195	FSH	Na	0.023315	0
B31	B191	FSH	Urea	0.007794	1.02E-11
B31	B200	FSH	OP	0.021141	0
B31	B219	FSH	OT	0.007334	1.53E-10
B31	B139	FSH	ADA	0.008362	2.88E-13
B31	B112	FSH	FT4	0.006307	3.67E-08
B31	B33	FSH	Prog	-0.29017	0
B31	B74	FSH	ApoA1	0.007617	2.92E-11
B31	B76	FSH	ApoB	0.007694	1.85E-11
B31	B77	FSH	TC	0.012598	0
B31	B114	FSH	TSH TT3 TT4(TT4)	0.007536	4.72E-11
B32	B92	Testo	ALDH2-Glu504Glu	-0.00764	2.59E-11
B32	B93	Testo	ALDH2-Glu504Lys	0.008051	2.08E-12
B32	B134	Testo	CHE	0.007504	5.70E-11
B32	B45	Testo	MCHC	0.005918	2.39E-07
B32	B46	Testo	RBC Count	0.011593	0
B32	B190	Testo	Cre	0.011358	0
B32	B222	Testo	NS	0.012226	0
B32	B104	Testo	anti-SSB	0.008184	8.98E-13
B32	B198	Testo	P	-0.00828	4.91E-13
B32	B176	Testo	Ucr	0.00615	7.92E-08
B32	B192	Testo	UA	0.012265	0
B32	B138	Testo	PAB	0.015863	0
B32	B60	Testo	WBRVI	0.008318	3.81E-13
B32	B62	Testo	WBHSRVI	0.006603	8.17E-09
B32	B64	Testo	WBRSR(mPas_5)	0.009697	0
B32	B65	Testo	WBRSR(mPas_1)	0.009836	0
B32	B66	Testo	WBRSR(mPas_100)	0.00873	2.51E-14
B32	B67	Testo	WBRSR(mPas_50)	0.008997	4.00E-15
B32	B221	Testo	Hei	0.01213	0
B32	B200	Testo	OP	0.006087	1.07E-07
B32	B216	Testo	BMI	0.01088	0
B32	B215	Testo	BMI	0.00575	5.17E-07
B32	B209	Testo	Smoking	0.007902	5.23E-12
B32	B51	Testo	Hb	0.013546	0
B32	B186	Testo	PFC	0.006661	6.04E-09
B32	B206	Testo	Waist	0.009594	0
B32	B197	Testo	AG	0.00652	1.26E-08
B32	B204	Testo	Drinking	0.006203	6.10E-08
B32	B33	Testo	Prog	-0.07545	0

B32	B76	Testo	ApoB	0.006806	2.82E-09
B32	B135	Testo	TBA	0.005869	3.00E-07
B33	B40	Prog	WBC	0.006056	1.24E-07
B33	B134	Prog	CHE	-0.00677	3.35E-09
B33	B70	Prog	LDL-C	-0.00801	2.63E-12
B33	B150	Prog	ALP	-0.00954	0
B33	B101	Prog	anti-Sc70	0.044794	0
B33	B102	Prog	anti-Sm	0.00676	3.60E-09
B33	B103	Prog	anti-SSA/Ro	0.012889	0
B33	B104	Prog	anti-SSB	0.006101	1.00E-07
B33	B107	Prog	AnuA	0.006444	1.84E-08
B33	B109	Prog	AMA-M2	0.005933	2.22E-07
B33	B110	Prog	AHA	0.007451	7.78E-11
B33	B156	Prog	IgE	0.006291	3.97E-08
B33	B195	Prog	Na	-0.01514	0
B33	B192	Prog	UA	-0.00665	6.33E-09
B33	B86	Prog	TBhCG	0.014095	0
B33	B200	Prog	OP	-0.01385	0
B33	B215	Prog	BMI	-0.00564	8.46E-07
B33	B196	Prog	TCO2	-0.00826	5.38E-13
B33	B76	Prog	ApoB	-0.00648	1.51E-08
B33	B77	Prog	TC	-0.00718	3.61E-10
B34	B137	TK	AFU	-0.01281	0
B34	B54	TK	ESR	0.007379	1.18E-10
B34	B93	TK	ALDH2-Glu504Lys	0.005921	2.35E-07
B34	B140	TK	r-GT	-0.01214	0
B34	B143	TK	β-CTx	-0.00597	1.87E-07
B34	B128	TK	ALT	-0.00745	7.59E-11
B34	B123	TK	hs-CRP1	0.00811	1.44E-12
B34	B134	TK	CHE	-0.02192	0
B34	B70	TK	LDL-C	-0.00815	1.12E-12
B34	B71	TK	TG	-0.0197	0
B34	B72	TK	HDL-C	0.007167	3.92E-10
B34	B189	TK	eGFB	0.005643	8.38E-07
B34	B141	TK	AST/ALT	0.007717	1.61E-11
B34	B56	TK	IR	-0.00572	5.86E-07
B34	B44	TK	MCH	-0.00568	6.99E-07
B34	B46	TK	RBC Count	-0.00625	4.92E-08
B34	B181	TK	APTT	0.013639	0
B34	B190	TK	Cre	-0.00585	3.28E-07
B34	B222	TK	NS	-0.01479	0
B34	B156	TK	IgE	0.006798	2.94E-09
B34	B157	TK	IgG	0.011607	0
B34	B158	TK	IgM	0.01064	0

B34	B195	TK	Na	-0.00766	2.24E-11
B34	B168	TK	NMP22	0.012893	0
B34	B176	TK	Ucr	-0.00871	2.95E-14
B34	B192	TK	UA	-0.01226	0
B34	B138	TK	PAB	-0.02889	0
B34	B60	TK	WBRVI	-0.00843	1.82E-13
B34	B62	TK	WBHSRVI	-0.0098	0
B34	B64	TK	WBRSR(mPas_5)	-0.0069	1.75E-09
B34	B65	TK	WBRSR(mPas_1)	-0.00647	1.58E-08
B34	B66	TK	WBRSR(mPas_100)	-0.00784	7.44E-12
B34	B67	TK	WBRSR(mPas_50)	-0.00764	2.59E-11
B34	B221	TK	Hei	-0.00591	2.50E-07
B34	B200	TK	OP	-0.00717	3.76E-10
B34	B220	TK	SBP	-0.00644	1.85E-08
B34	B218	TK	DBP	-0.00909	2.22E-15
B34	B216	TK	BMI	-0.01375	0
B34	B215	TK	BMI	-0.01419	0
B34	B210	TK	Hip	-0.00948	0
B34	B209	TK	Smoking	-0.00872	2.66E-14
B34	B90	TK	CYFRA 21-1	-0.00682	2.68E-09
B34	B139	TK	ADA	0.01295	0
B34	B196	TK	TCO2	-0.01115	0
B34	B51	TK	Hb	-0.01072	0
B34	B185	TK	PT	0.00769	1.90E-11
B34	B186	TK	PFC	0.009788	0
B34	B69	TK	PV	0.00563	8.86E-07
B34	B206	TK	Waist	-0.01492	0
B34	B204	TK	Drinking	-0.01436	0
B34	B112	TK	FT4	0.005926	2.29E-07
B34	B75	TK	ApoA1/B	0.007597	3.30E-11
B34	B76	TK	ApoB	-0.0164	0
B34	B81	TK	TSGF	-0.01771	0
B34	B77	TK	TC	-0.01251	0
B34	B144	TK	Total-PINP	-0.00671	4.72E-09
B35	B92	β2-MG	ALDH2-Glu504Glu	0.011064	0
B35	B93	β2-MG	ALDH2-Glu504Lys	-0.01046	0
B35	B124	β2-MG	ACTH	-0.01967	0
B35	B199	β2-MG	Mg	-0.00624	4.98E-08
B35	B159	β2-MG	β2-MG	0.012338	0
B35	B125	β2-MG	Cor	0.015492	0
B35	B138	β2-MG	PAB	0.00683	2.48E-09
B35	B139	β2-MG	ADA	-0.00561	9.58E-07
B36	B44	V-Lym%	MCH	-0.01849	0
B36	B45	V-Lym%	MCHC	-0.03731	0

B36	B47	V-Lym%	RDW	-0.00782	8.84E-12
B37	B40	BAND	WBC	0.182714	0
B37	B42	BAND	MON	0.094158	0
B37	B38	BAND	BAND%	0.460037	0
B37	B47	BAND	RDW	0.009347	4.44E-16
B37	B39	BAND	Other cells	0.042291	0
B38	B40	BAND%	WBC	0.045292	0
B38	B42	BAND%	MON	0.049296	0
B38	B46	BAND%	RBC Count	-0.00677	3.42E-09
B38	B47	BAND%	RDW	0.008552	8.26E-14
B38	B83	BAND%	AFP	-0.00586	3.12E-07
B38	B39	BAND%	Other cells	0.015687	0
B38	B86	BAND%	TBhCG	-0.00844	1.68E-13
B39	B40	Other cells	WBC	0.019282	0
B39	B42	Other cells	MON	0.014243	0
B39	B70	Other cells	LDL-C	-0.01469	0
B39	B72	Other cells	HDL-C	-0.00697	1.18E-09
B39	B43	Other cells	MCV	-0.05352	0
B39	B44	Other cells	MCH	-0.10105	0
B39	B45	Other cells	MCHC	-0.05843	0
B39	B46	Other cells	RBC Count	0.05542	0
B39	B47	Other cells	RDW	-0.04076	0
B39	B129	Other cells	IBIL	0.010478	0
B39	B105	Other cells	anti-U1RNP	0.006288	4.03E-08
B39	B51	Other cells	Hb	-0.04284	0
B39	B52	Other cells	PLT/BPC	0.0186	0
B39	B78	Other cells	HBcAb	-0.04166	0
B39	B203	Other cells	DH	0.00628	4.19E-08
B39	B201	Other cells	e-habits	0.007322	1.64E-10
B39	B132	Other cells	Dbil	0.012957	0
B39	B77	Other cells	TC	-0.01431	0
B39	B133	Other cells	TBIL	0.011857	0
B4	B20	WB-Ca	CD3+CD4+CD8+	0.005671	7.39E-07
B4	B143	WB-Ca	β-CTx	0.007751	1.31E-11
B4	B124	WB-Ca	ACTH	-0.00675	3.72E-09
B4	B32	WB-Ca	Testo	-0.01267	0
B4	B145	WB-Ca	BGP	0.010033	0
B4	B44	WB-Ca	MCH	-0.00607	1.18E-07
B4	B45	WB-Ca	MCHC	-0.01	0
B4	B46	WB-Ca	RBC Count	-0.00841	2.13E-13
B4	B190	WB-Ca	Cre	-0.00783	8.03E-12
B4	B129	WB-Ca	IBIL	-0.00865	4.44E-14
B4	B222	WB-Ca	NS	-0.00702	8.92E-10
B4	B194	WB-Ca	Cl	-0.00719	3.49E-10

B4	B192	WB-Ca	UA	-0.0062	6.11E-08
B4	B125	WB-Ca	Cor	-0.00626	4.72E-08
B4	B5	WB-Ca	WB-Mg	-0.05622	0
B4	B6	WB-Ca	WB-Fe	-0.15851	0
B4	B7	WB-Ca	WB-Cop	0.345601	0
B4	B8	WB-Ca	WB-Zinc	0.087553	0
B4	B64	WB-Ca	WBRSR(mPas_5)	-0.00581	3.97E-07
B4	B66	WB-Ca	WBRSR(mPas_100)	-0.00612	9.34E-08
B4	B67	WB-Ca	WBRSR(mPas_50)	-0.00614	8.17E-08
B4	B221	WB-Ca	Hei	-0.00572	5.82E-07
B4	B51	WB-Ca	Hb	-0.01297	0
B4	B183	WB-Ca	PTA	-0.00833	3.50E-13
B4	B206	WB-Ca	Waist	-0.00564	8.64E-07
B4	B211	WB-Ca	IOP-R	-0.0059	2.64E-07
B4	B133	WB-Ca	TBIL	-0.0075	5.75E-11
B4	B144	WB-Ca	Total-PINP	0.007189	3.46E-10
B40	B137	WBC	AFU	0.011248	0
B40	B54	WBC	ESR	0.012157	0
B40	B160	WBC	PH	-0.08048	0
B40	B140	WBC	r-GT	0.116599	0
B40	B118	WBC	CEA1	0.014182	0
B40	B126	WBC	Alb	0.05108	0
B40	B127	WBC	A-G ratio	-0.05806	0
B40	B161	WBC	LEU	-0.00923	8.88E-16
B40	B128	WBC	ALT	0.107731	0
B40	B123	WBC	hs-CRP1	0.070519	0
B40	B111	WBC	TSH	-0.00692	1.53E-09
B40	B124	WBC	ACTH	0.008739	2.35E-14
B40	B41	WBC	MON％	-0.02742	0
B40	B42	WBC	MON	0.02892	0
B40	B165	WBC	bilirubin	0.006203	6.10E-08
B40	B134	WBC	CHE	0.038935	0
B40	B166	WBC	Pro	0.069601	0
B40	B70	WBC	LDL-C	0.089664	0
B40	B188	WBC	Ca	0.020938	0
B40	B71	WBC	TG	0.174823	0
B40	B72	WBC	HDL-C	-0.17055	0
B40	B189	WBC	eGFB	0.022768	0
B40	B141	WBC	AST/ALT	-0.09593	0
B40	B146	WBC	CysC	0.013946	0
B40	B56	WBC	IR	0.026568	0
B40	B57	WBC	RBCAI	0.007415	9.58E-11
B40	B43	WBC	MCV	-0.02093	0
B40	B44	WBC	MCH	-0.01164	0

B40	B45	WBC	MCHC	0.061404	0
B40	B46	WBC	RBC Count	0.241882	0
B40	B47	WBC	RDW	-0.03174	0
B40	B58	WBC	HCT	0.008889	8.44E-15
B40	B181	WBC	APTT	-0.0072	3.24E-10
B40	B190	WBC	Cre	0.103742	0
B40	B129	WBC	IBIL	-0.0675	0
B40	B150	WBC	ALP	0.040977	0
B40	B84	WBC	C-PSA	0.013553	0
B40	B222	WBC	NS	0.189431	0
B40	B148	WBC	Anti_TPO	-0.01727	0
B40	B149	WBC	TGAb	-0.01671	0
B40	B48	WBC	LYM%	-0.0282	0
B40	B198	WBC	P	0.006201	6.17E-08
B40	B85	WBC	SCC	0.014258	0
B40	B194	WBC	Cl	-0.02323	0
B40	B155	WBC	IgA	0.008955	5.33E-15
B40	B156	WBC	IgE	0.011837	0
B40	B176	WBC	Ucr	0.042107	0
B40	B178	WBC	Ig-U	0.009492	0
B40	B191	WBC	Urea	0.013629	0
B40	B174	WBC	Urea/Cr	-0.06166	0
B40	B192	WBC	UA	0.193542	0
B40	B170	WBC	U-GLU	0.032729	0
B40	B175	WBC	ALB-U	0.015617	0
B40	B177	WBC	ACR	0.012953	0
B40	B179	WBC	α1-MG	0.017899	0
B40	B125	WBC	Cor	0.007041	7.90E-10
B40	B138	WBC	PAB	0.034349	0
B40	B130	WBC	GLB	0.099464	0
B40	B59	WBC	WBRV	0.026457	0
B40	B60	WBC	WBRVI	0.040626	0
B40	B61	WBC	WBHSRV	0.037489	0
B40	B62	WBC	WBHSRVI	0.04414	0
B40	B64	WBC	WBRSR(mPas_5)	0.054933	0
B40	B65	WBC	WBRSR(mPas_1)	0.05045	0
B40	B66	WBC	WBRSR(mPas_100)	0.055794	0
B40	B67	WBC	WBRSR(mPas_50)	0.055989	0
B40	B86	WBC	TBhCG	0.011911	0
B40	B221	WBC	Hei	0.110518	0
B40	B87	WBC	NSE	0.008027	2.42E-12
B40	B49	WBC	baso%	-0.01343	0
B40	B50	WBC	eo%	0.00839	2.39E-13
B40	B220	WBC	SBP	0.093916	0

B40	B219	WBC	OT	-0.02403	0
B40	B218	WBC	DBP	0.116944	0
B40	B216	WBC	BMI	0.201538	0
B40	B215	WBC	BMI	0.188337	0
B40	B131	WBC	AST	0.048973	0
B40	B210	WBC	Hip	0.156867	0
B40	B209	WBC	Smoking	0.191576	0
B40	B90	WBC	CYFRA 21-1	0.014918	0
B40	B139	WBC	ADA	-0.01233	0
B40	B196	WBC	TCO2	-0.0092	8.88E-16
B40	B51	WBC	Hb	0.227264	0
B40	B185	WBC	PT	-0.00731	1.76E-10
B40	B186	WBC	PFC	0.016331	0
B40	B69	WBC	PV	0.026266	0
B40	B52	WBC	PLT/BPC	0.230124	0
B40	B173	WBC	NTT	-0.00846	1.56E-13
B40	B207	WBC	WHR	0.020897	0
B40	B206	WBC	Waist	0.196794	0
B40	B205	WBC	KDA	-0.00612	9.09E-08
B40	B78	WBC	HBcAb	-0.01808	0
B40	B197	WBC	AG	0.020173	0
B40	B204	WBC	Drinking	0.079711	0
B40	B203	WBC	DH	0.054822	0
B40	B80	WBC	anti-HP IgG	0.022348	0
B40	B112	WBC	FT4	0.025709	0
B40	B113	WBC	FT3	0.032676	0
B40	B211	WBC	IOP-R	0.023092	0
B40	B201	WBC	e-habits	-0.01007	0
B40	B74	WBC	ApoA1	-0.0398	0
B40	B75	WBC	ApoA1/B	-0.02509	0
B40	B76	WBC	ApoB	0.044966	0
B40	B132	WBC	Dbil	-0.09713	0
B40	B53	WBC	GR%	0.024264	0
B40	B81	WBC	TSGF	-0.00832	3.67E-13
B40	B77	WBC	TC	0.06102	0
B40	B133	WBC	TBIL	-0.07995	0
B40	B135	WBC	TBA	0.006799	2.93E-09
B40	B91	WBC	TP	0.116429	0
B40	B114	WBC	TSH TT3 TT4(TT4)	0.015348	0
B40	B115	WBC	TSH TT3 TT4(TT3)	0.01437	0
B40	B212	WBC	IOP-L	0.020489	0
B41	B140	MON %	r-GT	0.014742	0
B41	B126	MON %	Alb	-0.01735	0
B41	B127	MON %	A-G ratio	-0.00672	4.47E-09

B41	B161	MON %	LEU	-0.00659	8.59E-09
B41	B128	MON %	ALT	0.020049	0
B41	B123	MON %	hs-CRP1	0.006507	1.34E-08
B41	B166	MON %	Pro	0.012656	0
B41	B72	MON %	HDL-C	-0.00755	4.38E-11
B41	B189	MON %	eGFB	-0.0223	0
B41	B141	MON %	AST/ALT	-0.01102	0
B41	B44	MON %	MCH	0.008693	3.22E-14
B41	B46	MON %	RBC Count	0.013774	0
B41	B47	MON %	RDW	0.010208	0
B41	B190	MON %	Cre	0.030156	0
B41	B129	MON %	IBIL	0.005949	2.06E-07
B41	B222	MON %	NS	0.026169	0
B41	B48	MON %	LYM%	0.01232	0
B41	B176	MON %	Ucr	0.006657	6.18E-09
B41	B191	MON %	Urea	0.011935	0
B41	B174	MON %	Urea/Cr	-0.01235	0
B41	B192	MON %	UA	0.023079	0
B41	B221	MON %	Hei	0.024445	0
B41	B49	MON %	baso%	0.007259	2.34E-10
B41	B50	MON %	eo%	0.075646	0
B41	B220	MON %	SBP	0.0136	0
B41	B218	MON %	DBP	0.008313	3.93E-13
B41	B216	MON %	BMI	0.020068	0
B41	B215	MON %	BMI	0.009733	0
B41	B131	MON %	AST	0.021346	0
B41	B210	MON %	Hip	0.011905	0
B41	B209	MON %	Smoking	0.023039	0
B41	B139	MON %	ADA	0.006288	4.02E-08
B41	B51	MON %	Hb	0.021044	0
B41	B52	MON %	PLT/BPC	-0.00605	1.29E-07
B41	B206	MON %	Waist	0.020187	0
B41	B204	MON %	Drinking	0.021816	0
B41	B112	MON %	FT4	0.006926	1.48E-09
B41	B113	MON %	FT3	0.012547	0
B41	B132	MON %	Dbil	0.010367	0
B41	B53	MON %	GR%	0.012906	0
B41	B133	MON %	TBIL	0.007529	4.93E-11
B41	B91	MON %	TP	-0.00922	8.88E-16
B42	B228	MON	OS	0.006392	2.40E-08
B43	B54	MCV	ESR	-0.00906	2.66E-15
B43	B160	MCV	PH	-0.0118	0
B43	B140	MCV	r-GT	0.021076	0
B43	B126	MCV	Alb	-0.02889	0

B43	B111	MCV	TSH	0.005697	6.57E-07
B43	B70	MCV	LDL-C	0.032962	0
B43	B72	MCV	HDL-C	0.034252	0
B43	B189	MCV	eGFB	-0.05756	0
B43	B146	MCV	CysC	0.00567	7.40E-07
B43	B55	MCV	TK	-0.01128	0
B43	B56	MCV	IR	-0.00584	3.46E-07
B43	B57	MCV	RBCAI	-0.00699	1.04E-09
B43	B44	MCV	MCH	0.373399	0
B43	B45	MCV	MCHC	0.127131	0
B43	B46	MCV	RBC Count	-0.2282	0
B43	B47	MCV	RDW	0.227143	0
B43	B190	MCV	Cre	0.016219	0
B43	B129	MCV	IBIL	-0.00588	2.86E-07
B43	B195	MCV	Na	0.00757	3.87E-11
B43	B191	MCV	Urea	0.023561	0
B43	B174	MCV	Urea/Cr	0.005755	5.06E-07
B43	B192	MCV	UA	-0.01148	0
B43	B130	MCV	GLB	-0.01751	0
B43	B59	MCV	WBRV	-0.0143	0
B43	B61	MCV	WBHSRV	-0.00723	2.71E-10
B43	B221	MCV	Hei	-0.015	0
B43	B200	MCV	OP	0.006245	4.97E-08
B43	B49	MCV	baso%	-0.01472	0
B43	B219	MCV	OT	0.012524	0
B43	B218	MCV	DBP	0.006055	1.25E-07
B43	B216	MCV	BMI	-0.01279	0
B43	B215	MCV	BMI	-0.00582	3.75E-07
B43	B131	MCV	AST	0.017726	0
B43	B210	MCV	Hip	-0.01367	0
B43	B209	MCV	Smoking	0.058974	0
B43	B90	MCV	CYFRA 21-1	0.005882	2.81E-07
B43	B51	MCV	Hb	0.09568	0
B43	B183	MCV	PTA	-0.00591	2.43E-07
B43	B52	MCV	PLT/BPC	-0.09612	0
B43	B206	MCV	Waist	0.006954	1.27E-09
B43	B205	MCV	KDA	0.006338	3.14E-08
B43	B204	MCV	Drinking	0.0408	0
B43	B112	MCV	FT4	-0.02414	0
B43	B113	MCV	FT3	-0.01121	0
B43	B201	MCV	e-habits	0.010841	0
B43	B74	MCV	ApoA1	0.016164	0
B43	B76	MCV	ApoB	0.007983	3.17E-12
B43	B132	MCV	Dbil	0.008793	1.64E-14

B43	B77	MCV	TC	0.04107	0
B43	B91	MCV	TP	-0.03279	0
B43	B114	MCV	TSH TT3 TT4(TT4)	-0.00929	4.44E-16
B44	B137	MCH	AFU	0.012405	0
B44	B54	MCH	ESR	-0.03269	0
B44	B160	MCH	PH	-0.02149	0
B44	B140	MCH	r-GT	0.081685	0
B44	B118	MCH	CEA1	0.011003	0
B44	B127	MCH	A-G ratio	0.052811	0
B44	B161	MCH	LEU	-0.02029	0
B44	B128	MCH	ALT	0.050227	0
B44	B165	MCH	bilirubin	0.008818	1.38E-14
B44	B134	MCH	CHE	0.012462	0
B44	B166	MCH	Pro	0.024072	0
B44	B70	MCH	LDL-C	0.070308	0
B44	B188	MCH	Ca	0.007049	7.54E-10
B44	B71	MCH	TG	0.071172	0
B44	B72	MCH	HDL-C	-0.00742	9.09E-11
B44	B189	MCH	eGFB	-0.10424	0
B44	B141	MCH	AST/ALT	-0.04654	0
B44	B146	MCH	CysC	0.012643	0
B44	B55	MCH	TK	-0.01679	0
B44	B56	MCH	IR	0.010513	0
B44	B57	MCH	RBCAI	-0.01136	0
B44	B45	MCH	MCHC	0.518693	0
B44	B46	MCH	RBC Count	-0.28448	0
B44	B47	MCH	RDW	0.344621	0
B44	B58	MCH	HCT	0.005971	1.86E-07
B44	B181	MCH	APTT	-0.00658	9.30E-09
B44	B190	MCH	Cre	0.109466	0
B44	B224	MCH	pmh	0.007293	1.93E-10
B44	B193	MCH	K	-0.01153	0
B44	B129	MCH	IBIL	0.045167	0
B44	B150	MCH	ALP	0.014512	0
B44	B84	MCH	C-PSA	0.007165	3.96E-10
B44	B222	MCH	NS	0.097755	0
B44	B148	MCH	Anti_TPO	-0.01537	0
B44	B149	MCH	TGAb	-0.01514	0
B44	B198	MCH	P	-0.01397	0
B44	B167	MCH	PC	-0.01201	0
B44	B194	MCH	Cl	-0.006	1.61E-07
B44	B157	MCH	IgG	-0.01304	0
B44	B195	MCH	Na	0.008192	8.57E-13
B44	B176	MCH	Ucr	0.021394	0

B44	B191	MCH	Urea	0.045455	0
B44	B174	MCH	Urea/Cr	-0.04501	0
B44	B192	MCH	UA	0.076167	0
B44	B170	MCH	U-GLU	0.013374	0
B44	B177	MCH	ACR	-0.00611	9.39E-08
B44	B179	MCH	α1-MG	0.010281	0
B44	B182	MCH	TT	0.006844	2.30E-09
B44	B153	MCH	Glu	0.0065	1.39E-08
B44	B138	MCH	PAB	0.025457	0
B44	B130	MCH	GLB	-0.06179	0
B44	B59	MCH	WBRV	-0.01536	0
B44	B60	MCH	WBRVI	0.017757	0
B44	B61	MCH	WBHSRV	0.008423	1.93E-13
B44	B62	MCH	WBHSRVI	0.032939	0
B44	B64	MCH	WBRSR(mPas_5)	0.022069	0
B44	B65	MCH	WBRSR(mPas_1)	0.016358	0
B44	B66	MCH	WBRSR(mPas_100)	0.029397	0
B44	B67	MCH	WBRSR(mPas_50)	0.028404	0
B44	B221	MCH	Hei	0.068535	0
B44	B200	MCH	OP	0.008772	1.89E-14
B44	B49	MCH	baso%	0.329804	0
B44	B50	MCH	eo%	0.015343	0
B44	B220	MCH	SBP	0.041244	0
B44	B219	MCH	OT	0.010906	0
B44	B218	MCH	DBP	0.057045	0
B44	B216	MCH	BMI	0.070717	0
B44	B215	MCH	BMI	0.046346	0
B44	B131	MCH	AST	0.053874	0
B44	B172	MCH	KB	0.006971	1.16E-09
B44	B210	MCH	Hip	0.034118	0
B44	B209	MCH	Smoking	0.139071	0
B44	B90	MCH	CYFRA 21-1	0.012596	0
B44	B51	MCH	Hb	0.341282	0
B44	B183	MCH	PTA	-0.00672	4.33E-09
B44	B52	MCH	PLT/BPC	-0.16242	0
B44	B173	MCH	NTT	-0.00683	2.53E-09
B44	B207	MCH	WHR	0.011721	0
B44	B206	MCH	Waist	0.082011	0
B44	B197	MCH	AG	0.007242	2.57E-10
B44	B204	MCH	Drinking	0.122577	0
B44	B112	MCH	FT4	-0.0248	0
B44	B201	MCH	e-habits	0.02185	0
B44	B74	MCH	ApoA1	0.006056	1.24E-07
B44	B75	MCH	ApoA1/B	-0.00786	6.92E-12

B44	B76	MCH	ApoB	0.024311	0
B44	B132	MCH	Dbil	0.047117	0
B44	B77	MCH	TC	0.077278	0
B44	B133	MCH	TBIL	0.048796	0
B44	B135	MCH	TBA	0.006276	4.26E-08
B44	B91	MCH	TP	-0.05175	0
B44	B114	MCH	TSH TT3 TT4(TT4)	-0.01898	0
B45	B137	MCHC	AFU	0.016985	0
B45	B54	MCHC	ESR	-0.05048	0
B45	B160	MCHC	PH	-0.00706	7.07E-10
B45	B140	MCHC	r-GT	0.125507	0
B45	B118	MCHC	CEA1	0.006781	3.21E-09
B45	B126	MCHC	Alb	0.133935	0
B45	B127	MCHC	A-G ratio	0.116677	0
B45	B161	MCHC	LEU	-0.05848	0
B45	B128	MCHC	ALT	0.137613	0
B45	B121	MCHC	C3	0.006358	2.84E-08
B45	B111	MCHC	TSH	-0.02799	0
B45	B165	MCHC	bilirubin	0.005823	3.71E-07
B45	B134	MCHC	CHE	0.041957	0
B45	B166	MCHC	Pro	0.07412	0
B45	B70	MCHC	LDL-C	0.055744	0
B45	B188	MCHC	Ca	0.018315	0
B45	B71	MCHC	TG	0.166272	0
B45	B72	MCHC	HDL-C	-0.15565	0
B45	B189	MCHC	eGFB	-0.03656	0
B45	B141	MCHC	AST/ALT	-0.15129	0
B45	B146	MCHC	CysC	0.010122	0
B45	B56	MCHC	IR	0.050953	0
B45	B46	MCHC	RBC Count	0.106713	0
B45	B47	MCHC	RDW	-0.21803	0
B45	B58	MCHC	HCT	0.009149	1.33E-15
B45	B181	MCHC	APTT	-0.00801	2.61E-12
B45	B190	MCHC	Cre	0.227742	0
B45	B193	MCHC	K	-0.02653	0
B45	B129	MCHC	IBIL	0.155983	0
B45	B150	MCHC	ALP	0.015895	0
B45	B84	MCHC	C-PSA	0.011693	0
B45	B222	MCHC	NS	0.26076	0
B45	B148	MCHC	Anti_TPO	-0.02431	0
B45	B149	MCHC	TGAb	-0.02315	0
B45	B198	MCHC	P	-0.01908	0
B45	B194	MCHC	Cl	-0.01924	0
B45	B199	MCHC	Mg	-0.00588	2.79E-07

B45	B157	MCHC	IgG	-0.01611	0
B45	B158	MCHC	IgM	-0.00791	5.07E-12
B45	B195	MCHC	Na	-0.00928	4.44E-16
B45	B176	MCHC	Ucr	0.058303	0
B45	B191	MCHC	Urea	0.027176	0
B45	B174	MCHC	Urea/Cr	-0.14262	0
B45	B192	MCHC	UA	0.248848	0
B45	B170	MCHC	U-GLU	0.042804	0
B45	B177	MCHC	ACR	-0.00606	1.24E-07
B45	B179	MCHC	α1-MG	0.01618	0
B45	B153	MCHC	Glu	0.014506	0
B45	B138	MCHC	PAB	0.049442	0
B45	B130	MCHC	GLB	-0.08154	0
B45	B59	MCHC	WBRV	0.016824	0
B45	B60	MCHC	WBRVI	0.060666	0
B45	B61	MCHC	WBHSRV	0.05142	0
B45	B62	MCHC	WBHSRVI	0.078276	0
B45	B63	MCHC	WBRSR(mPas_200)	0.008423	1.92E-13
B45	B64	MCHC	WBRSR(mPas_5)	0.069327	0
B45	B65	MCHC	WBRSR(mPas_1)	0.060529	0
B45	B66	MCHC	WBRSR(mPas_100)	0.075514	0
B45	B67	MCHC	WBRSR(mPas_50)	0.074761	0
B45	B221	MCHC	Hei	0.240264	0
B45	B87	MCHC	NSE	0.012884	0
B45	B49	MCHC	baso%	-0.08102	0
B45	B50	MCHC	eo%	0.015436	0
B45	B220	MCHC	SBP	0.081719	0
B45	B219	MCHC	OT	-0.02476	0
B45	B218	MCHC	DBP	0.120666	0
B45	B88	MCHC	CA 242	-0.01096	0
B45	B216	MCHC	BMI	0.239877	0
B45	B215	MCHC	BMI	0.147854	0
B45	B131	MCHC	AST	0.071171	0
B45	B172	MCHC	KB	0.009945	0
B45	B210	MCHC	Hip	0.145859	0
B45	B209	MCHC	Smoking	0.119145	0
B45	B90	MCHC	CYFRA 21-1	0.008746	2.24E-14
B45	B139	MCHC	ADA	-0.01457	0
B45	B51	MCHC	Hb	0.57014	0
B45	B183	MCHC	PTA	0.006931	1.44E-09
B45	B186	MCHC	PFC	-0.00599	1.68E-07
B45	B52	MCHC	PLT/BPC	-0.03727	0
B45	B173	MCHC	NTT	-0.03233	0
B45	B207	MCHC	WHR	0.020458	0

B45	B206	MCHC	Waist	0.187266	0
B45	B205	MCHC	KDA	-0.01626	0
B45	B197	MCHC	AG	0.007324	1.61E-10
B45	B204	MCHC	Drinking	0.151542	0
B45	B203	MCHC	DH	0.025009	0
B45	B112	MCHC	FT4	0.027355	0
B45	B113	MCHC	FT3	0.034476	0
B45	B211	MCHC	IOP-R	0.016271	0
B45	B201	MCHC	e-habits	0.009953	0
B45	B73	MCHC	ApoA	-0.01328	0
B45	B74	MCHC	ApoA1	-0.05103	0
B45	B75	MCHC	ApoA1/B	-0.02669	0
B45	B76	MCHC	ApoB	0.033665	0
B45	B132	MCHC	Dbil	0.093638	0
B45	B53	MCHC	GR%	-0.0075	5.90E-11
B45	B81	MCHC	TSGF	-0.01852	0
B45	B77	MCHC	TC	0.041651	0
B45	B133	MCHC	TBIL	0.150271	0
B45	B91	MCHC	TP	0.013391	0
B45	B114	MCHC	TSH TT3 TT4(TT4)	-0.01106	0
B45	B115	MCHC	TSH TT3 TT4(TT3)	0.006286	4.07E-08
B45	B212	MCHC	IOP-L	0.013947	0
B46	B137	RBC Count	AFU	0.02665	0
B46	B54	RBC Count	ESR	-0.04958	0
B46	B160	RBC Count	PH	-0.05744	0
B46	B140	RBC Count	r-GT	0.163294	0
B46	B143	RBC Count	β-CTx	0.007371	1.23E-10
B46	B118	RBC Count	CEA1	0.006055	1.25E-07
B46	B126	RBC Count	Alb	0.296364	0
B46	B127	RBC Count	A-G ratio	0.137564	0
B46	B161	RBC Count	LEU	-0.10357	0
B46	B128	RBC Count	ALT	0.245399	0
B46	B123	RBC Count	hs-CRP1	0.007345	1.43E-10
B46	B111	RBC Count	TSH	-0.0519	0
B46	B124	RBC Count	ACTH	0.01248	0
B46	B134	RBC Count	CHE	0.079588	0
B46	B166	RBC Count	Pro	0.130202	0
B46	B70	RBC Count	LDL-C	0.142941	0
B46	B188	RBC Count	Ca	0.047782	0
B46	B71	RBC Count	TG	0.184238	0
B46	B72	RBC Count	HDL-C	-0.28815	0
B46	B189	RBC Count	eGFB	-0.05149	0
B46	B141	RBC Count	AST/ALT	-0.27685	0
B46	B146	RBC Count	CysC	0.020639	0

B46	B55	RBC Count	TK	-0.0147	0
B46	B56	RBC Count	IR	0.077533	0
B46	B57	RBC Count	RBCAI	0.035061	0
B46	B47	RBC Count	RDW	-0.38034	0
B46	B58	RBC Count	HCT	0.018811	0
B46	B181	RBC Count	APTT	-0.00724	2.66E-10
B46	B190	RBC Count	Cre	0.421161	0
B46	B193	RBC Count	K	0.008208	7.71E-13
B46	B129	RBC Count	IBIL	0.139534	0
B46	B150	RBC Count	ALP	0.049339	0
B46	B84	RBC Count	C-PSA	0.022902	0
B46	B222	RBC Count	NS	0.458771	0
B46	B100	RBC Count	anti-Ro52	-0.00609	1.08E-07
B46	B103	RBC Count	anti-SSA/Ro	-0.00829	4.66E-13
B46	B148	RBC Count	Anti_TPO	-0.04057	0
B46	B149	RBC Count	TGAb	-0.03611	0
B46	B198	RBC Count	P	-0.03132	0
B46	B85	RBC Count	SCC	0.015678	0
B46	B194	RBC Count	Cl	-0.0326	0
B46	B199	RBC Count	Mg	0.011756	0
B46	B155	RBC Count	IgA	0.007584	3.56E-11
B46	B157	RBC Count	IgG	-0.01711	0
B46	B158	RBC Count	IgM	-0.01299	0
B46	B195	RBC Count	Na	0.008741	2.31E-14
B46	B176	RBC Count	Ucr	0.100774	0
B46	B178	RBC Count	Ig-U	0.005901	2.58E-07
B46	B191	RBC Count	Urea	0.078449	0
B46	B174	RBC Count	Urea/Cr	-0.2371	0
B46	B192	RBC Count	UA	0.439121	0
B46	B170	RBC Count	U-GLU	0.044493	0
B46	B175	RBC Count	ALB-U	0.012359	0
B46	B179	RBC Count	α1-MG	0.026408	0
B46	B180	RBC Count	Tf	0.009733	0
B46	B182	RBC Count	TT	0.00806	1.97E-12
B46	B125	RBC Count	Cor	0.013248	0
B46	B153	RBC Count	Glu	0.009283	4.44E-16
B46	B138	RBC Count	PAB	0.080242	0
B46	B130	RBC Count	GLB	-0.04154	0
B46	B59	RBC Count	WBRV	0.059226	0
B46	B60	RBC Count	WBRVI	0.134921	0
B46	B61	RBC Count	WBHSRV	0.083171	0
B46	B62	RBC Count	WBHSRVI	0.137139	0
B46	B63	RBC Count	WBRSR(mPas_200)	0.015804	0
B46	B64	RBC Count	WBRSR(mPas_5)	0.146163	0

B46	B65	RBC Count	WBRSR(mPas_1)	0.138743	0
B46	B66	RBC Count	WBRSR(mPas_100)	0.141169	0
B46	B67	RBC Count	WBRSR(mPas_50)	0.14281	0
B46	B86	RBC Count	TBhCG	-0.01305	0
B46	B221	RBC Count	Hei	0.446902	0
B46	B87	RBC Count	NSE	0.019412	0
B46	B187	RBC Count	NSE	0.011751	0
B46	B200	RBC Count	OP	0.012996	0
B46	B49	RBC Count	baso%	0.057184	0
B46	B50	RBC Count	eo%	0.057189	0
B46	B220	RBC Count	SBP	0.1785	0
B46	B219	RBC Count	OT	-0.05354	0
B46	B218	RBC Count	DBP	0.231775	0
B46	B88	RBC Count	CA 242	-0.02139	0
B46	B216	RBC Count	BMI	0.438868	0
B46	B215	RBC Count	BMI	0.268384	0
B46	B131	RBC Count	AST	0.106116	0
B46	B210	RBC Count	Hip	0.286075	0
B46	B209	RBC Count	Smoking	0.167107	0
B46	B90	RBC Count	CYFRA 21-1	0.014758	0
B46	B139	RBC Count	ADA	-0.01568	0
B46	B51	RBC Count	Hb	0.708712	0
B46	B185	RBC Count	PT	-0.00735	1.42E-10
B46	B186	RBC Count	PFC	-0.00577	4.78E-07
B46	B69	RBC Count	PV	0.009172	1.33E-15
B46	B52	RBC Count	PLT/BPC	0.08631	0
B46	B173	RBC Count	NTT	-0.05184	0
B46	B207	RBC Count	WHR	0.039745	0
B46	B206	RBC Count	Waist	0.357086	0
B46	B205	RBC Count	KDA	-0.0376	0
B46	B197	RBC Count	AG	0.022229	0
B46	B204	RBC Count	Drinking	0.208541	0
B46	B203	RBC Count	DH	0.035999	0
B46	B112	RBC Count	FT4	0.06412	0
B46	B113	RBC Count	FT3	0.108877	0
B46	B211	RBC Count	IOP-R	0.025597	0
B46	B201	RBC Count	e-habits	0.009379	2.22E-16
B46	B73	RBC Count	ApoA	-0.01426	0
B46	B74	RBC Count	ApoA1	-0.07646	0
B46	B75	RBC Count	ApoA1/B	-0.04937	0
B46	B76	RBC Count	ApoB	0.066011	0
B46	B132	RBC Count	Dbil	0.06168	0
B46	B53	RBC Count	GR%	-0.00562	9.40E-07
B46	B81	RBC Count	TSGF	-0.01043	0

B46	B77	RBC Count	TC	0.057253	0
B46	B133	RBC Count	TBIL	0.128598	0
B46	B135	RBC Count	TBA	0.007622	2.85E-11
B46	B91	RBC Count	TP	0.148389	0
B46	B115	RBC Count	TSH TT3 TT4(TT3)	0.036598	0
B46	B212	RBC Count	IOP-L	0.023185	0
B47	B54	RDW	ESR	0.016458	0
B47	B160	RDW	PH	-0.04848	0
B47	B140	RDW	r-GT	0.038347	0
B47	B118	RDW	CEA1	0.018694	0
B47	B126	RDW	Alb	-0.17325	0
B47	B127	RDW	A-G ratio	-0.07067	0
B47	B161	RDW	LEU	0.017629	0
B47	B128	RDW	ALT	-0.02207	0
B47	B123	RDW	hs-CRP1	0.009671	0
B47	B111	RDW	TSH	0.042407	0
B47	B165	RDW	bilirubin	0.01602	0
B47	B134	RDW	CHE	-0.02627	0
B47	B166	RDW	Pro	-0.01867	0
B47	B70	RDW	LDL-C	0.020896	0
B47	B188	RDW	Ca	-0.02179	0
B47	B72	RDW	HDL-C	0.098941	0
B47	B189	RDW	eGFB	-0.15226	0
B47	B141	RDW	AST/ALT	0.058055	0
B47	B146	RDW	CysC	0.015339	0
B47	B55	RDW	TK	-0.00655	1.08E-08
B47	B56	RDW	IR	-0.02324	0
B47	B57	RDW	RBCAI	-0.01052	0
B47	B190	RDW	Cre	-0.0384	0
B47	B224	RDW	pmh	0.011113	0
B47	B83	RDW	AFP	0.006048	1.29E-07
B47	B193	RDW	K	0.006898	1.72E-09
B47	B129	RDW	IBIL	-0.06167	0
B47	B150	RDW	ALP	0.023234	0
B47	B213	RDW	CVA-R	0.007651	2.39E-11
B47	B214	RDW	CVA-L	0.008175	9.52E-13
B47	B222	RDW	NS	-0.05816	0
B47	B154	RDW	RF	0.006131	8.69E-08
B47	B48	RDW	LYM%	-0.00669	5.30E-09
B47	B198	RDW	P	-0.0105	0
B47	B194	RDW	Cl	0.018966	0
B47	B199	RDW	Mg	0.006708	4.72E-09
B47	B195	RDW	Na	0.014894	0
B47	B176	RDW	Ucr	-0.02191	0

B47	B191	RDW	Urea	0.053411	0
B47	B174	RDW	Urea/Cr	0.068566	0
B47	B192	RDW	UA	-0.08599	0
B47	B170	RDW	U-GLU	-0.01118	0
B47	B138	RDW	PAB	-0.01938	0
B47	B130	RDW	GLB	0.021714	0
B47	B59	RDW	WBRV	-0.02104	0
B47	B60	RDW	WBRVI	-0.02872	0
B47	B61	RDW	WBHSRV	-0.0261	0
B47	B62	RDW	WBHSRVI	-0.02797	0
B47	B64	RDW	WBRSR(mPas_5)	-0.03215	0
B47	B65	RDW	WBRSR(mPas_1)	-0.03064	0
B47	B66	RDW	WBRSR(mPas_100)	-0.03025	0
B47	B67	RDW	WBRSR(mPas_50)	-0.03058	0
B47	B221	RDW	Hei	-0.12818	0
B47	B87	RDW	NSE	-0.00707	6.82E-10
B47	B200	RDW	OP	0.010244	0
B47	B227	RDW	OD	0.008208	7.73E-13
B47	B228	RDW	OS	0.010504	0
B47	B49	RDW	baso%	0.152752	0
B47	B50	RDW	eo%	0.019948	0
B47	B220	RDW	SBP	0.028335	0
B47	B219	RDW	OT	0.045741	0
B47	B89	RDW	CA 72-4	0.006272	4.35E-08
B47	B216	RDW	BMI	-0.07973	0
B47	B215	RDW	BMI	-0.01221	0
B47	B131	RDW	AST	0.047312	0
B47	B210	RDW	Hip	-0.05113	0
B47	B209	RDW	Smoking	0.150727	0
B47	B90	RDW	CYFRA 21-1	0.011365	0
B47	B139	RDW	ADA	0.016705	0
B47	B51	RDW	Hb	-0.15237	0
B47	B183	RDW	PTA	-0.01327	0
B47	B186	RDW	PFC	0.006371	2.67E-08
B47	B52	RDW	PLT/BPC	-0.20927	0
B47	B173	RDW	NTT	0.023664	0
B47	B205	RDW	KDA	0.016254	0
B47	B78	RDW	HBcAb	0.011318	0
B47	B204	RDW	Drinking	0.050244	0
B47	B203	RDW	DH	-0.01473	0
B47	B112	RDW	FT4	-0.07597	0
B47	B113	RDW	FT3	-0.04452	0
B47	B211	RDW	IOP-R	-0.0256	0
B47	B201	RDW	e-habits	0.021666	0

B47	B74	RDW	ApoA1	0.045592	0
B47	B75	RDW	ApoA1/B	0.018419	0
B47	B76	RDW	ApoB	-0.00885	1.11E-14
B47	B132	RDW	Dbil	0.012914	0
B47	B81	RDW	TSGF	0.013684	0
B47	B77	RDW	TC	0.061672	0
B47	B133	RDW	TBIL	-0.04627	0
B47	B135	RDW	TBA	0.011341	0
B47	B91	RDW	TP	-0.08885	0
B47	B114	RDW	TSH TT3 TT4(TT4)	-0.01651	0
B47	B115	RDW	TSH TT3 TT4(TT3)	-0.01184	0
B47	B212	RDW	IOP-L	-0.0219	0
B48	B123	LYM%	hs-CRP1	-0.00695	1.29E-09
B48	B221	LYM%	Hei	0.006547	1.09E-08
B48	B50	LYM%	eo%	0.033887	0
B48	B209	LYM%	Smoking	-0.00655	1.08E-08
B48	B91	LYM%	TP	0.005613	9.57E-07
B49	B70	baso%	LDL-C	0.007303	1.82E-10
B49	B189	baso%	eGFB	-0.00966	0
B49	B190	baso%	Cre	0.006684	5.37E-09
B49	B167	baso%	PC	-0.02783	0
B49	B50	baso%	eo%	0.051679	0
B49	B51	baso%	Hb	-0.02445	0
B49	B52	baso%	PLT/BPC	0.006076	1.13E-07
B49	B77	baso%	TC	0.007284	2.02E-10
B5	B23	WB-Mg	CD3-CD16+CD56+(NK)	-0.00707	6.59E-10
B5	B21	WB-Mg	CD3+CD4-CD8-	-0.00705	7.56E-10
B5	B128	WB-Mg	ALT	0.007334	1.53E-10
B5	B121	WB-Mg	C3	-0.00699	1.03E-09
B5	B29	WB-Mg	E2	-0.00831	4.10E-13
B5	B134	WB-Mg	CHE	0.007764	1.22E-11
B5	B71	WB-Mg	TG	0.005627	8.99E-07
B5	B72	WB-Mg	HDL-C	-0.00716	4.13E-10
B5	B32	WB-Mg	Testo	0.008932	6.22E-15
B5	B141	WB-Mg	AST/ALT	-0.00718	3.59E-10
B5	B44	WB-Mg	MCH	0.007029	8.44E-10
B5	B45	WB-Mg	MCHC	0.008788	1.69E-14
B5	B46	WB-Mg	RBC Count	0.010355	0
B5	B190	WB-Mg	Cre	0.008	2.86E-12
B5	B222	WB-Mg	NS	0.009447	0
B5	B108	WB-Mg	Anti-Cyclin	-0.00706	7.02E-10
B5	B157	WB-Mg	IgG	-0.00823	6.51E-13
B5	B195	WB-Mg	Na	0.006127	8.84E-08
B5	B192	WB-Mg	UA	0.009279	4.44E-16

B5	B138	WB-Mg	PAB	0.008277	4.97E-13
B5	B6	WB-Mg	WB-Fe	0.723819	0
B5	B7	WB-Mg	WB-Cop	0.056887	0
B5	B8	WB-Mg	WB-Zinc	0.43236	0
B5	B221	WB-Mg	Hei	0.006693	5.11E-09
B5	B200	WB-Mg	OP	0.005814	3.85E-07
B5	B88	WB-Mg	CA 242	0.008691	3.24E-14
B5	B216	WB-Mg	BMI	0.009643	0
B5	B215	WB-Mg	BMI	0.008224	6.96E-13
B5	B210	WB-Mg	Hip	0.006337	3.15E-08
B5	B51	WB-Mg	Hb	0.01568	0
B5	B206	WB-Mg	Waist	0.009485	0
B5	B204	WB-Mg	Drinking	0.006231	5.32E-08
B5	B76	WB-Mg	ApoB	0.008921	6.66E-15
B50	B137	eo%	AFU	0.007594	3.36E-11
B50	B160	eo%	PH	-0.03242	0
B50	B140	eo%	r-GT	0.030693	0
B50	B118	eo%	CEA1	0.009263	6.66E-16
B50	B126	eo%	Alb	-0.04573	0
B50	B161	eo%	LEU	-0.01743	0
B50	B128	eo%	ALT	0.029238	0
B50	B123	eo%	hs-CRP1	-0.00566	7.69E-07
B50	B166	eo%	Pro	0.014053	0
B50	B70	eo%	LDL-C	0.037368	0
B50	B71	eo%	TG	0.021696	0
B50	B72	eo%	HDL-C	-0.03393	0
B50	B189	eo%	eGFB	-0.08348	0
B50	B141	eo%	AST/ALT	-0.03426	0
B50	B146	eo%	CysC	0.013231	0
B50	B190	eo%	Cre	0.101063	0
B50	B193	eo%	K	0.005944	2.11E-07
B50	B129	eo%	IBIL	-0.01838	0
B50	B150	eo%	ALP	0.010741	0
B50	B222	eo%	NS	0.077173	0
B50	B85	eo%	SCC	0.014847	0
B50	B156	eo%	IgE	0.026791	0
B50	B176	eo%	Ucr	0.013611	0
B50	B191	eo%	Urea	0.041334	0
B50	B174	eo%	Urea/Cr	-0.03721	0
B50	B192	eo%	UA	0.071782	0
B50	B130	eo%	GLB	-0.01122	0
B50	B62	eo%	WBHSRVI	0.005826	3.64E-07
B50	B221	eo%	Hei	0.071713	0
B50	B220	eo%	SBP	0.030904	0

B50	B218	eo%	DBP	0.026646	0
B50	B216	eo%	BMI	0.066337	0
B50	B215	eo%	BMI	0.03911	0
B50	B131	eo%	AST	0.022935	0
B50	B172	eo%	KB	-0.01757	0
B50	B210	eo%	Hip	0.041519	0
B50	B209	eo%	Smoking	0.095944	0
B50	B90	eo%	CYFRA 21-1	0.014205	0
B50	B51	eo%	Hb	0.069618	0
B50	B173	eo%	NTT	-0.00565	8.01E-07
B50	B207	eo%	WHR	0.008236	6.46E-13
B50	B206	eo%	Waist	0.07011	0
B50	B205	eo%	KDA	0.005884	2.79E-07
B50	B204	eo%	Drinking	0.058835	0
B50	B113	eo%	FT3	0.008556	8.02E-14
B50	B211	eo%	IOP-R	-0.009	4.00E-15
B50	B201	eo%	e-habits	0.011885	0
B50	B74	eo%	ApoA1	-0.00949	0
B50	B75	eo%	ApoA1/B	-0.00914	1.55E-15
B50	B76	eo%	ApoB	0.009238	8.88E-16
B50	B132	eo%	Dbil	-0.0242	0
B50	B53	eo%	GR%	0.02333	0
B50	B77	eo%	TC	0.025358	0
B50	B133	eo%	TBIL	-0.02121	0
B50	B91	eo%	TP	-0.038	0
B50	B212	eo%	IOP-L	-0.00792	4.62E-12
B51	B137	Hb	AFU	0.036285	0
B51	B54	Hb	ESR	-0.07548	0
B51	B160	Hb	PH	-0.07245	0
B51	B140	Hb	r-GT	0.22511	0
B51	B143	Hb	β-CTx	0.007978	3.29E-12
B51	B118	Hb	CEA1	0.014492	0
B51	B126	Hb	Alb	0.294475	0
B51	B127	Hb	A-G ratio	0.175962	0
B51	B161	Hb	LEU	-0.11915	0
B51	B128	Hb	ALT	0.283834	0
B51	B164	Hb	COM	-0.00677	3.35E-09
B51	B123	Hb	hs-CRP1	0.006078	1.12E-07
B51	B111	Hb	TSH	-0.0532	0
B51	B124	Hb	ACTH	0.01516	0
B51	B134	Hb	CHE	0.089699	0
B51	B166	Hb	Pro	0.147743	0
B51	B70	Hb	LDL-C	0.202292	0
B51	B188	Hb	Ca	0.053089	0

B51	B71	Hb	TG	0.239243	0
B51	B72	Hb	HDL-C	-0.29033	0
B51	B189	Hb	eGFB	-0.12971	0
B51	B141	Hb	AST/ALT	-0.31128	0
B51	B146	Hb	CysC	0.029721	0
B51	B55	Hb	TK	-0.02911	0
B51	B56	Hb	IR	0.085089	0
B51	B57	Hb	RBCAI	0.023204	0
B51	B58	Hb	HCT	0.023831	0
B51	B181	Hb	APTT	-0.01224	0
B51	B190	Hb	Cre	0.502252	0
B51	B224	Hb	pmh	0.007871	6.32E-12
B51	B129	Hb	IBIL	0.169845	0
B51	B150	Hb	ALP	0.060886	0
B51	B84	Hb	C-PSA	0.027993	0
B51	B222	Hb	NS	0.532854	0
B51	B100	Hb	anti-Ro52	-0.00638	2.52E-08
B51	B103	Hb	anti-SSA/Ro	-0.00931	4.44E-16
B51	B148	Hb	Anti_TPO	-0.0517	0
B51	B149	Hb	TGAb	-0.04743	0
B51	B198	Hb	P	-0.04232	0
B51	B85	Hb	SCC	0.017093	0
B51	B194	Hb	Cl	-0.03746	0
B51	B199	Hb	Mg	0.012575	0
B51	B156	Hb	IgE	0.008164	1.02E-12
B51	B157	Hb	IgG	-0.02736	0
B51	B158	Hb	IgM	-0.01634	0
B51	B195	Hb	Na	0.014727	0
B51	B176	Hb	Ucr	0.116492	0
B51	B178	Hb	Ig-U	0.006389	2.44E-08
B51	B191	Hb	Urea	0.111983	0
B51	B174	Hb	Urea/Cr	-0.27033	0
B51	B192	Hb	UA	0.491369	0
B51	B170	Hb	U-GLU	0.055079	0
B51	B175	Hb	ALB-U	0.011217	0
B51	B177	Hb	ACR	-0.00655	1.08E-08
B51	B179	Hb	α1-MG	0.034433	0
B51	B180	Hb	Tf	0.00968	0
B51	B182	Hb	TT	0.013835	0
B51	B125	Hb	Cor	0.015983	0
B51	B153	Hb	Glu	0.014654	0
B51	B138	Hb	PAB	0.100009	0
B51	B130	Hb	GLB	-0.0878	0
B51	B59	Hb	WBRV	0.044083	0

B51	B60	Hb	WBRVI	0.147109	0
B51	B61	Hb	WBHSRV	0.08909	0
B51	B62	Hb	WBHSRVI	0.163495	0
B51	B63	Hb	WBRSR(mPas_200)	0.018274	0
B51	B64	Hb	WBRSR(mPas_5)	0.162584	0
B51	B65	Hb	WBRSR(mPas_1)	0.149934	0
B51	B66	Hb	WBRSR(mPas_100)	0.16454	0
B51	B67	Hb	WBRSR(mPas_50)	0.165173	0
B51	B86	Hb	TBhCG	-0.01156	0
B51	B221	Hb	Hei	0.498251	0
B51	B87	Hb	NSE	0.020628	0
B51	B187	Hb	NSE	0.014108	0
B51	B200	Hb	OP	0.019422	0
B51	B220	Hb	SBP	0.207602	0
B51	B219	Hb	OT	-0.04564	0
B51	B218	Hb	DBP	0.273879	0
B51	B88	Hb	CA 242	-0.02284	0
B51	B216	Hb	BMI	0.493654	0
B51	B215	Hb	BMI	0.305373	0
B51	B131	Hb	AST	0.147225	0
B51	B210	Hb	Hip	0.313404	0
B51	B209	Hb	Smoking	0.274759	0
B51	B90	Hb	CYFRA 21-1	0.024156	0
B51	B139	Hb	ADA	-0.01934	0
B51	B196	Hb	TCO2	0.006322	3.41E-08
B51	B185	Hb	PT	-0.01029	0
B51	B52	Hb	PLT/BPC	-0.04153	0
B51	B173	Hb	NTT	-0.05676	0
B51	B207	Hb	WHR	0.048574	0
B51	B206	Hb	Waist	0.420168	0
B51	B205	Hb	KDA	-0.03399	0
B51	B197	Hb	AG	0.027878	0
B51	B204	Hb	Drinking	0.303026	0
B51	B203	Hb	DH	0.039617	0
B51	B112	Hb	FT4	0.043181	0
B51	B113	Hb	FT3	0.105316	0
B51	B211	Hb	IOP-R	0.02449	0
B51	B201	Hb	e-habits	0.024775	0
B51	B73	Hb	ApoA	-0.01541	0
B51	B74	Hb	ApoA1	-0.07259	0
B51	B75	Hb	ApoA1/B	-0.05641	0
B51	B76	Hb	ApoB	0.086624	0
B51	B132	Hb	Dbil	0.091654	0
B51	B53	Hb	GR%	-0.00816	1.07E-12

B51	B81	Hb	TSGF	-0.0116	0
B51	B77	Hb	TC	0.121989	0
B51	B133	Hb	TBIL	0.160915	0
B51	B135	Hb	TBA	0.012009	0
B51	B91	Hb	TP	0.107758	0
B51	B114	Hb	TSH TT3 TT4(TT4)	-0.00952	0
B51	B115	Hb	TSH TT3 TT4(TT3)	0.033734	0
B51	B212	Hb	IOP-L	0.022583	0
B52	B54	PLT/BPC	ESR	0.017593	0
B52	B160	PLT/BPC	PH	0.030061	0
B52	B118	PLT/BPC	CEA1	-0.00953	0
B52	B126	PLT/BPC	Alb	0.007513	5.42E-11
B52	B127	PLT/BPC	A-G ratio	-0.0868	0
B52	B161	PLT/BPC	LEU	0.018724	0
B52	B128	PLT/BPC	ALT	-0.05388	0
B52	B123	PLT/BPC	hs-CRP1	0.017242	0
B52	B111	PLT/BPC	TSH	-0.02181	0
B52	B134	PLT/BPC	CHE	0.012541	0
B52	B166	PLT/BPC	Pro	0.006366	2.74E-08
B52	B70	PLT/BPC	LDL-C	0.074683	0
B52	B188	PLT/BPC	Ca	0.013505	0
B52	B71	PLT/BPC	TG	-0.04154	0
B52	B72	PLT/BPC	HDL-C	0.01162	0
B52	B189	PLT/BPC	eGFB	0.159328	0
B52	B141	PLT/BPC	AST/ALT	0.053415	0
B52	B146	PLT/BPC	CysC	-0.01885	0
B52	B190	PLT/BPC	Cre	-0.09429	0
B52	B224	PLT/BPC	pmh	-0.00984	0
B52	B193	PLT/BPC	K	0.025644	0
B52	B129	PLT/BPC	IBIL	-0.08672	0
B52	B150	PLT/BPC	ALP	-0.01897	0
B52	B214	PLT/BPC	CVA-L	-0.00594	2.10E-07
B52	B222	PLT/BPC	NS	-0.04059	0
B52	B148	PLT/BPC	Anti_TPO	0.009656	0
B52	B149	PLT/BPC	TGAb	0.007344	1.44E-10
B52	B198	PLT/BPC	P	0.031992	0
B52	B194	PLT/BPC	Cl	-0.01308	0
B52	B155	PLT/BPC	IgA	0.008	2.86E-12
B52	B156	PLT/BPC	IgE	0.005981	1.78E-07
B52	B157	PLT/BPC	IgG	0.008686	3.38E-14
B52	B195	PLT/BPC	Na	-0.01883	0
B52	B176	PLT/BPC	Ucr	0.006476	1.57E-08
B52	B191	PLT/BPC	Urea	-0.12398	0
B52	B174	PLT/BPC	Urea/Cr	-0.03316	0

B52	B192	PLT/BPC	UA	-0.02731	0
B52	B170	PLT/BPC	U-GLU	-0.02274	0
B52	B153	PLT/BPC	Glu	-0.00743	8.95E-11
B52	B138	PLT/BPC	PAB	0.009739	0
B52	B130	PLT/BPC	GLB	0.1062	0
B52	B59	PLT/BPC	WBRV	0.010684	0
B52	B61	PLT/BPC	WBHSRV	0.011403	0
B52	B64	PLT/BPC	WBRSR(mPas_5)	0.009677	0
B52	B65	PLT/BPC	WBRSR(mPas_1)	0.008361	2.88E-13
B52	B66	PLT/BPC	WBRSR(mPas_100)	0.010364	0
B52	B67	PLT/BPC	WBRSR(mPas_50)	0.010379	0
B52	B87	PLT/BPC	NSE	0.009365	4.44E-16
B52	B187	PLT/BPC	NSE	0.005761	4.92E-07
B52	B200	PLT/BPC	OP	-0.02153	0
B52	B220	PLT/BPC	SBP	-0.03882	0
B52	B219	PLT/BPC	OT	-0.02318	0
B52	B218	PLT/BPC	DBP	-0.02134	0
B52	B88	PLT/BPC	CA 242	0.006905	1.65E-09
B52	B89	PLT/BPC	CA 72-4	-0.00733	1.60E-10
B52	B215	PLT/BPC	BMI	-0.01265	0
B52	B131	PLT/BPC	AST	-0.06791	0
B52	B172	PLT/BPC	KB	0.006961	1.22E-09
B52	B210	PLT/BPC	Hip	0.009666	0
B52	B209	PLT/BPC	Smoking	-0.07134	0
B52	B139	PLT/BPC	ADA	-0.02212	0
B52	B183	PLT/BPC	PTA	0.011467	0
B52	B69	PLT/BPC	PV	0.014197	0
B52	B207	PLT/BPC	WHR	-0.00771	1.70E-11
B52	B206	PLT/BPC	Waist	-0.04948	0
B52	B205	PLT/BPC	KDA	0.009325	4.44E-16
B52	B78	PLT/BPC	HBcAb	-0.02139	0
B52	B204	PLT/BPC	Drinking	-0.05432	0
B52	B203	PLT/BPC	DH	0.025226	0
B52	B80	PLT/BPC	anti-HP IgG	0.005747	5.24E-07
B52	B112	PLT/BPC	FT4	0.045683	0
B52	B211	PLT/BPC	IOP-R	0.019225	0
B52	B201	PLT/BPC	e-habits	-0.02088	0
B52	B73	PLT/BPC	ApoA	0.00679	3.07E-09
B52	B74	PLT/BPC	ApoA1	-0.01919	0
B52	B75	PLT/BPC	ApoA1/B	-0.01905	0
B52	B76	PLT/BPC	ApoB	0.03053	0
B52	B132	PLT/BPC	Dbil	-0.1335	0
B52	B77	PLT/BPC	TC	0.035261	0
B52	B133	PLT/BPC	TBIL	-0.10504	0

B52	B135	PLT/BPC	TBA	-0.00786	6.61E-12
B52	B91	PLT/BPC	TP	0.095242	0
B52	B114	PLT/BPC	TSH TT3 TT4(TT4)	0.016661	0
B52	B212	PLT/BPC	IOP-L	0.015626	0
B53	B70	GR%	LDL-C	-0.00676	3.62E-09
B53	B190	GR%	Cre	-0.00892	6.66E-15
B53	B222	GR%	NS	-0.00563	8.94E-07
B53	B192	GR%	UA	-0.00612	9.17E-08
B53	B221	GR%	Hei	-0.00966	0
B53	B216	GR%	BMI	-0.00563	8.76E-07
B53	B77	GR%	TC	-0.00576	4.83E-07
B54	B160	ESR	PH	-0.00966	0
B54	B126	ESR	Alb	-0.0415	0
B54	B127	ESR	A-G ratio	-0.06539	0
B54	B161	ESR	LEU	0.024328	0
B54	B128	ESR	ALT	-0.01122	0
B54	B123	ESR	hs-CRP1	0.187069	0
B54	B111	ESR	TSH	0.01569	0
B54	B124	ESR	ACTH	-0.00967	0
B54	B134	ESR	CHE	-0.0095	0
B54	B166	ESR	Pro	-0.00643	1.99E-08
B54	B70	ESR	LDL-C	0.014131	0
B54	B71	ESR	TG	-0.00795	3.94E-12
B54	B72	ESR	HDL-C	0.017707	0
B54	B141	ESR	AST/ALT	0.023805	0
B54	B55	ESR	TK	0.037163	0
B54	B56	ESR	IR	-0.16488	0
B54	B57	ESR	RBCAI	-0.05474	0
B54	B58	ESR	HCT	-0.04086	0
B54	B181	ESR	APTT	0.006208	5.96E-08
B54	B190	ESR	Cre	-0.03407	0
B54	B193	ESR	K	0.011998	0
B54	B129	ESR	IBIL	-0.0295	0
B54	B150	ESR	ALP	0.013957	0
B54	B222	ESR	NS	-0.03538	0
B54	B105	ESR	anti-U1RNP	0.00661	7.90E-09
B54	B96	ESR	dsDNA (IIF)	0.01028	0
B54	B98	ESR	anti-Jo-1	-0.00743	8.83E-11
B54	B100	ESR	anti-Ro52	0.013715	0
B54	B101	ESR	anti-Sc70	0.012708	0
B54	B102	ESR	anti-Sm	0.007135	4.68E-10
B54	B103	ESR	anti-SSA/Ro	0.030426	0
B54	B106	ESR	anti-P	0.007521	5.17E-11
B54	B107	ESR	AnuA	0.018133	0

B54	B151	ESR	Anti-CCP	0.0299	0
B54	B148	ESR	Anti_TPO	0.014474	0
B54	B149	ESR	TGAb	0.015391	0
B54	B110	ESR	AHA	0.005961	1.95E-07
B54	B154	ESR	RF	0.013551	0
B54	B198	ESR	P	0.024557	0
B54	B155	ESR	IgA	0.054268	0
B54	B156	ESR	IgE	0.011768	0
B54	B157	ESR	IgG	0.077813	0
B54	B158	ESR	IgM	0.023271	0
B54	B195	ESR	Na	-0.01939	0
B54	B176	ESR	Ucr	-0.01876	0
B54	B178	ESR	Ig-U	0.006435	1.93E-08
B54	B174	ESR	Urea/Cr	0.02693	0
B54	B192	ESR	UA	-0.02738	0
B54	B175	ESR	ALB-U	0.022258	0
B54	B177	ESR	ACR	0.028607	0
B54	B182	ESR	TT	-0.03893	0
B54	B125	ESR	Cor	-0.00622	5.56E-08
B54	B138	ESR	PAB	-0.04933	0
B54	B130	ESR	GLB	0.078945	0
B54	B59	ESR	WBRV	0.027191	0
B54	B60	ESR	WBRVI	-0.27842	0
B54	B61	ESR	WBHSRV	-0.0464	0
B54	B62	ESR	WBHSRVI	-0.29596	0
B54	B63	ESR	WBRSR(mPas_200)	-0.01987	0
B54	B64	ESR	WBRSR(mPas_5)	-0.16797	0
B54	B65	ESR	WBRSR(mPas_1)	-0.16428	0
B54	B66	ESR	WBRSR(mPas_100)	-0.15624	0
B54	B67	ESR	WBRSR(mPas_50)	-0.1602	0
B54	B221	ESR	Hei	-0.05114	0
B54	B200	ESR	OP	-0.01117	0
B54	B220	ESR	SBP	0.011958	0
B54	B219	ESR	OT	0.007445	8.06E-11
B54	B89	ESR	CA 72-4	-0.0066	8.32E-09
B54	B216	ESR	BMI	-0.03067	0
B54	B131	ESR	AST	0.005725	5.79E-07
B54	B210	ESR	Hip	-0.01119	0
B54	B209	ESR	Smoking	-0.02153	0
B54	B139	ESR	ADA	0.047935	0
B54	B196	ESR	TCO2	-0.00906	2.44E-15
B54	B183	ESR	PTA	0.007874	6.21E-12
B54	B185	ESR	PT	0.02255	0
B54	B186	ESR	PFC	0.105067	0

B54	B69	ESR	PV	0.350583	0
B54	B173	ESR	NTT	0.017116	0
B54	B206	ESR	Waist	-0.01405	0
B54	B197	ESR	AG	-0.00724	2.60E-10
B54	B204	ESR	Drinking	-0.02964	0
B54	B112	ESR	FT4	0.018041	0
B54	B113	ESR	FT3	-0.00738	1.17E-10
B54	B211	ESR	IOP-R	-0.01557	0
B54	B73	ESR	ApoA	0.033934	0
B54	B74	ESR	ApoA1	0.041023	0
B54	B76	ESR	ApoB	0.027026	0
B54	B132	ESR	Dbil	-0.02549	0
B54	B77	ESR	TC	0.015769	0
B54	B133	ESR	TBIL	-0.03023	0
B54	B135	ESR	TBA	-0.00705	7.35E-10
B54	B91	ESR	TP	0.041896	0
B54	B114	ESR	TSH TT3 TT4(TT4)	0.053628	0
B54	B212	ESR	IOP-L	-0.01663	0
B55	B137	TK	AFU	-0.01159	0
B55	B126	TK	Alb	-0.00967	0
B55	B124	TK	ACTH	-0.01029	0
B55	B134	TK	CHE	-0.00766	2.32E-11
B55	B70	TK	LDL-C	-0.0089	7.55E-15
B55	B56	TK	IR	0.692617	0
B55	B57	TK	RBCAI	0.110263	0
B55	B58	TK	HCT	-0.09445	0
B55	B181	TK	APTT	-0.00712	4.98E-10
B55	B190	TK	Cre	-0.01397	0
B55	B193	TK	K	-0.01144	0
B55	B129	TK	IBIL	-0.00915	1.33E-15
B55	B222	TK	NS	-0.00735	1.42E-10
B55	B107	TK	AnuA	-0.00602	1.46E-07
B55	B155	TK	IgA	0.011947	0
B55	B158	TK	IgM	0.006012	1.53E-07
B55	B176	TK	Ucr	-0.00848	1.36E-13
B55	B174	TK	Urea/Cr	0.008217	7.30E-13
B55	B192	TK	UA	-0.00828	4.98E-13
B55	B125	TK	Cor	0.012372	0
B55	B138	TK	PAB	-0.00667	5.84E-09
B55	B59	TK	WBRV	0.524444	0
B55	B60	TK	WBRVI	0.071851	0
B55	B61	TK	WBHSRV	0.652153	0
B55	B62	TK	WBHSRVI	0.159897	0
B55	B64	TK	WBRSR(mPas_5)	0.030805	0

B55	B66	TK	WBRSR(mPas_100)	0.079978	0
B55	B67	TK	WBRSR(mPas_50)	0.07384	0
B55	B221	TK	Hei	-0.01181	0
B55	B209	TK	Smoking	-0.01075	0
B55	B183	TK	PTA	-0.02015	0
B55	B69	TK	PV	-0.19268	0
B55	B78	TK	HBcAb	0.007489	6.23E-11
B55	B204	TK	Drinking	-0.00748	6.65E-11
B55	B74	TK	ApoA1	-0.01123	0
B55	B76	TK	ApoB	-0.00924	8.88E-16
B55	B77	TK	TC	-0.00571	6.21E-07
B55	B133	TK	TBIL	-0.00734	1.49E-10
B55	B91	TK	TP	-0.00652	1.23E-08
B56	B137	IR	AFU	0.016021	0
B56	B160	IR	PH	-0.00839	2.33E-13
B56	B140	IR	r-GT	0.031289	0
B56	B126	IR	Alb	0.015421	0
B56	B127	IR	A-G ratio	0.018033	0
B56	B161	IR	LEU	-0.01008	0
B56	B128	IR	ALT	0.025373	0
B56	B121	IR	C3	0.007462	7.27E-11
B56	B111	IR	TSH	-0.01306	0
B56	B124	IR	ACTH	0.014594	0
B56	B134	IR	CHE	0.038374	0
B56	B166	IR	Pro	0.012799	0
B56	B70	IR	LDL-C	0.012004	0
B56	B71	IR	TG	0.024878	0
B56	B72	IR	HDL-C	-0.03063	0
B56	B141	IR	AST/ALT	-0.02763	0
B56	B57	IR	RBCAI	-0.07951	0
B56	B58	IR	HCT	-0.01457	0
B56	B181	IR	APTT	-0.02043	0
B56	B190	IR	Cre	0.03468	0
B56	B193	IR	K	-0.00582	3.80E-07
B56	B129	IR	IBIL	0.013126	0
B56	B150	IR	ALP	0.018889	0
B56	B222	IR	NS	0.051298	0
B56	B96	IR	dsDNA (IIF)	-0.00576	4.94E-07
B56	B98	IR	anti-Jo-1	0.007096	5.82E-10
B56	B100	IR	anti-Ro52	-0.00729	1.94E-10
B56	B103	IR	anti-SSA/Ro	-0.0172	0
B56	B107	IR	AnuA	-0.01125	0
B56	B148	IR	Anti_TPO	-0.01032	0
B56	B149	IR	TGAb	-0.0099	0

B56	B110	IR	AHA	-0.00881	1.49E-14
B56	B198	IR	P	-0.02018	0
B56	B194	IR	Cl	-0.03314	0
B56	B199	IR	Mg	0.006916	1.56E-09
B56	B155	IR	IgA	0.009784	0
B56	B157	IR	IgG	-0.02632	0
B56	B158	IR	IgM	-0.00994	0
B56	B176	IR	Ucr	0.023007	0
B56	B174	IR	Urea/Cr	-0.02388	0
B56	B192	IR	UA	0.043865	0
B56	B170	IR	U-GLU	0.01144	0
B56	B177	IR	ACR	-0.0064	2.25E-08
B56	B179	IR	α1-MG	0.019526	0
B56	B182	IR	TT	0.019082	0
B56	B125	IR	Cor	0.023453	0
B56	B138	IR	PAB	0.058108	0
B56	B130	IR	GLB	-0.0134	0
B56	B59	IR	WBRV	0.616791	0
B56	B60	IR	WBRVI	0.588248	0
B56	B61	IR	WBHSRV	0.937299	0
B56	B62	IR	WBHSRVI	0.772861	0
B56	B63	IR	WBRSR(mPas_200)	0.064732	0
B56	B64	IR	WBRSR(mPas_5)	0.577484	0
B56	B65	IR	WBRSR(mPas_1)	0.496499	0
B56	B66	IR	WBRSR(mPas_100)	0.659466	0
B56	B67	IR	WBRSR(mPas_50)	0.652239	0
B56	B221	IR	Hei	0.041068	0
B56	B187	IR	NSE	0.007116	5.20E-10
B56	B200	IR	OP	0.010247	0
B56	B220	IR	SBP	0.013658	0
B56	B218	IR	DBP	0.022354	0
B56	B216	IR	BMI	0.04585	0
B56	B215	IR	BMI	0.031273	0
B56	B131	IR	AST	0.009604	0
B56	B210	IR	Hip	0.031274	0
B56	B209	IR	Smoking	0.026892	0
B56	B90	IR	CYFRA 21-1	0.006046	1.31E-07
B56	B139	IR	ADA	-0.00605	1.29E-07
B56	B183	IR	PTA	-0.02766	0
B56	B185	IR	PT	-0.01046	0
B56	B186	IR	PFC	-0.00873	2.44E-14
B56	B69	IR	PV	-0.24745	0
B56	B173	IR	NTT	-0.00832	3.87E-13
B56	B206	IR	Waist	0.043409	0

B56	B78	IR	HBcAb	0.008689	3.31E-14
B56	B197	IR	AG	0.019582	0
B56	B204	IR	Drinking	0.028372	0
B56	B113	IR	FT3	0.01978	0
B56	B211	IR	IOP-R	0.011658	0
B56	B73	IR	ApoA	-0.01303	0
B56	B74	IR	ApoA1	-0.07043	0
B56	B75	IR	ApoA1/B	-0.03871	0
B56	B76	IR	ApoB	0.048421	0
B56	B132	IR	Dbil	0.014359	0
B56	B77	IR	TC	0.006292	3.96E-08
B56	B133	IR	TBIL	0.014329	0
B56	B114	IR	TSH TT3 TT4(TT4)	-0.01478	0
B56	B115	IR	TSH TT3 TT4(TT3)	0.010913	0
B56	B212	IR	IOP-L	0.010168	0
B57	B92	RBCAI	ALDH2-Glu504Glu	-0.00807	1.82E-12
B57	B93	RBCAI	ALDH2-Glu504Lys	0.007726	1.52E-11
B57	B126	RBCAI	Alb	0.013638	0
B57	B127	RBCAI	A-G ratio	0.018305	0
B57	B161	RBCAI	LEU	-0.00685	2.24E-09
B57	B123	RBCAI	hs-CRP1	-0.01716	0
B57	B111	RBCAI	TSH	-0.00785	7.36E-12
B57	B124	RBCAI	ACTH	0.011525	0
B57	B134	RBCAI	CHE	0.006856	2.16E-09
B57	B71	RBCAI	TG	-0.00735	1.38E-10
B57	B72	RBCAI	HDL-C	0.008919	6.88E-15
B57	B58	RBCAI	HCT	0.012272	0
B57	B181	RBCAI	APTT	-0.01504	0
B57	B190	RBCAI	Cre	0.014577	0
B57	B193	RBCAI	K	0.00797	3.44E-12
B57	B129	RBCAI	IBIL	0.00601	1.55E-07
B57	B222	RBCAI	NS	0.013231	0
B57	B96	RBCAI	dsDNA (IIF)	0.006567	9.87E-09
B57	B100	RBCAI	anti-Ro52	-0.00727	2.19E-10
B57	B103	RBCAI	anti-SSA/Ro	-0.00627	4.29E-08
B57	B104	RBCAI	anti-SSB	-0.00788	5.88E-12
B57	B107	RBCAI	AnuA	0.005955	2.00E-07
B57	B151	RBCAI	Anti-CCP	0.016623	0
B57	B109	RBCAI	AMA-M2	-0.00651	1.31E-08
B57	B194	RBCAI	Cl	0.015345	0
B57	B158	RBCAI	IgM	-0.00702	8.75E-10
B57	B176	RBCAI	Ucr	0.01157	0
B57	B174	RBCAI	Urea/Cr	-0.01259	0
B57	B192	RBCAI	UA	0.009341	4.44E-16

B57	B125	RBCAI	Cor	0.025294	0
B57	B138	RBCAI	PAB	0.009412	2.22E-16
B57	B130	RBCAI	GLB	-0.01903	0
B57	B59	RBCAI	WBRV	0.46308	0
B57	B60	RBCAI	WBRVI	0.453115	0
B57	B61	RBCAI	WBHSRV	-0.07802	0
B57	B62	RBCAI	WBHSRVI	-0.03042	0
B57	B64	RBCAI	WBRSR(mPas_5)	0.293588	0
B57	B65	RBCAI	WBRSR(mPas_1)	0.448014	0
B57	B67	RBCAI	WBRSR(mPas_50)	0.051256	0
B57	B86	RBCAI	TBhCG	-0.00686	2.09E-09
B57	B221	RBCAI	Hei	0.015363	0
B57	B216	RBCAI	BMI	0.008185	8.96E-13
B57	B209	RBCAI	Smoking	0.013965	0
B57	B90	RBCAI	CYFRA 21-1	0.006445	1.84E-08
B57	B139	RBCAI	ADA	-0.02017	0
B57	B183	RBCAI	PTA	-0.03768	0
B57	B184	RBCAI	INR	0.0166	0
B57	B186	RBCAI	PFC	0.00831	4.02E-13
B57	B69	RBCAI	PV	0.011574	0
B57	B206	RBCAI	Waist	0.00565	8.09E-07
B57	B78	RBCAI	HBcAb	-0.01921	0
B57	B204	RBCAI	Drinking	0.013301	0
B57	B112	RBCAI	FT4	0.01791	0
B57	B113	RBCAI	FT3	0.006878	1.91E-09
B57	B211	RBCAI	IOP-R	0.010078	0
B57	B73	RBCAI	ApoA	-0.00588	2.84E-07
B57	B74	RBCAI	ApoA1	0.03585	0
B57	B75	RBCAI	ApoA1/B	0.016023	0
B57	B76	RBCAI	ApoB	-0.01928	0
B57	B81	RBCAI	TSGF	-0.04164	0
B57	B91	RBCAI	TP	-0.00782	8.42E-12
B57	B212	RBCAI	IOP-L	0.012163	0
B58	B140	HCT	r-GT	0.006346	3.01E-08
B58	B126	HCT	Alb	0.005902	2.57E-07
B58	B128	HCT	ALT	0.006803	2.85E-09
B58	B72	HCT	HDL-C	-0.00663	6.97E-09
B58	B141	HCT	AST/ALT	-0.00603	1.40E-07
B58	B190	HCT	Cre	0.009654	0
B58	B222	HCT	NS	0.012207	0
B58	B194	HCT	Cl	-0.0084	2.27E-13
B58	B157	HCT	IgG	-0.00849	1.28E-13
B58	B174	HCT	Urea/Cr	-0.006	1.61E-07
B58	B192	HCT	UA	0.010366	0

B58	B138	HCT	PAB	0.011106	0
B58	B59	HCT	WBRV	-0.03671	0
B58	B60	HCT	WBRVI	0.084846	0
B58	B61	HCT	WBHSRV	-0.01501	0
B58	B62	HCT	WBHSRVI	0.094695	0
B58	B63	HCT	WBRSR(mPas_200)	0.010419	0
B58	B64	HCT	WBRSR(mPas_5)	0.094997	0
B58	B65	HCT	WBRSR(mPas_1)	0.08734	0
B58	B66	HCT	WBRSR(mPas_100)	0.097165	0
B58	B67	HCT	WBRSR(mPas_50)	0.097515	0
B58	B221	HCT	Hei	0.009857	0
B58	B218	HCT	DBP	0.006593	8.60E-09
B58	B216	HCT	BMI	0.010476	0
B58	B215	HCT	BMI	0.00711	5.38E-10
B58	B210	HCT	Hip	0.007777	1.12E-11
B58	B209	HCT	Smoking	0.010598	0
B58	B206	HCT	Waist	0.010086	0
B58	B204	HCT	Drinking	0.006414	2.15E-08
B58	B74	HCT	ApoA1	-0.0104	0
B58	B75	HCT	ApoA1/B	-0.00724	2.59E-10
B58	B76	HCT	ApoB	0.009337	4.44E-16
B59	B137	WBRV	AFU	0.006855	2.16E-09
B59	B160	WBRV	PH	-0.00908	2.22E-15
B59	B140	WBRV	r-GT	0.020313	0
B59	B126	WBRV	Alb	0.02348	0
B59	B128	WBRV	ALT	0.013684	0
B59	B121	WBRV	C3	0.007129	4.85E-10
B59	B123	WBRV	hs-CRP1	0.023684	0
B59	B111	WBRV	TSH	-0.00643	1.97E-08
B59	B124	WBRV	ACTH	0.014672	0
B59	B134	WBRV	CHE	0.028496	0
B59	B166	WBRV	Pro	0.008776	1.82E-14
B59	B70	WBRV	LDL-C	0.014758	0
B59	B71	WBRV	TG	0.015289	0
B59	B141	WBRV	AST/ALT	-0.00744	8.13E-11
B59	B181	WBRV	APTT	-0.02417	0
B59	B190	WBRV	Cre	0.020222	0
B59	B193	WBRV	K	0.009804	0
B59	B129	WBRV	IBIL	0.009836	0
B59	B150	WBRV	ALP	0.016363	0
B59	B222	WBRV	NS	0.027082	0
B59	B99	WBRV	anti-PM-Scl	0.00712	5.10E-10
B59	B104	WBRV	anti-SSB	-0.00947	0
B59	B151	WBRV	Anti-CCP	0.015524	0

B59	B198	WBRV	P	-0.00924	8.88E-16
B59	B194	WBRV	Cl	-0.01838	0
B59	B155	WBRV	IgA	0.020994	0
B59	B156	WBRV	IgE	0.009207	8.88E-16
B59	B157	WBRV	IgG	0.022984	0
B59	B195	WBRV	Na	-0.00922	8.88E-16
B59	B176	WBRV	Ucr	0.014442	0
B59	B174	WBRV	Urea/Cr	-0.01533	0
B59	B192	WBRV	UA	0.025777	0
B59	B170	WBRV	U-GLU	0.009921	0
B59	B179	WBRV	α1-MG	0.013709	0
B59	B182	WBRV	TT	-0.00645	1.83E-08
B59	B125	WBRV	Cor	0.038132	0
B59	B138	WBRV	PAB	0.031083	0
B59	B130	WBRV	GLB	0.010883	0
B59	B60	WBRV	WBRVI	0.639131	0
B59	B61	WBRV	WBHSRV	0.701324	0
B59	B62	WBRV	WBHSRVI	0.355169	0
B59	B63	WBRV	WBRSR(mPas_200)	0.044243	0
B59	B64	WBRV	WBRSR(mPas_5)	0.615759	0
B59	B65	WBRV	WBRSR(mPas_1)	0.679647	0
B59	B66	WBRV	WBRSR(mPas_100)	0.437669	0
B59	B67	WBRV	WBRSR(mPas_50)	0.469037	0
B59	B221	WBRV	Hei	0.019678	0
B59	B220	WBRV	SBP	0.008061	1.96E-12
B59	B218	WBRV	DBP	0.017004	0
B59	B216	WBRV	BMI	0.022065	0
B59	B215	WBRV	BMI	0.015087	0
B59	B131	WBRV	AST	0.011704	0
B59	B210	WBRV	Hip	0.01725	0
B59	B209	WBRV	Smoking	0.014934	0
B59	B90	WBRV	CYFRA 21-1	0.005852	3.23E-07
B59	B183	WBRV	PTA	-0.02934	0
B59	B184	WBRV	INR	0.018696	0
B59	B186	WBRV	PFC	0.030527	0
B59	B69	WBRV	PV	0.148166	0
B59	B206	WBRV	Waist	0.023277	0
B59	B78	WBRV	HBcAb	-0.01004	0
B59	B197	WBRV	AG	0.00734	1.47E-10
B59	B204	WBRV	Drinking	0.015424	0
B59	B112	WBRV	FT4	0.027051	0
B59	B113	WBRV	FT3	0.013634	0
B59	B211	WBRV	IOP-R	0.014035	0
B59	B75	WBRV	ApoA1/B	-0.00635	2.96E-08

B59	B76	WBRV	ApoB	0.021793	0
B59	B81	WBRV	TSGF	-0.0415	0
B59	B77	WBRV	TC	0.008702	3.02E-14
B59	B133	WBRV	TBIL	0.008564	7.59E-14
B59	B91	WBRV	TP	0.023977	0
B59	B114	WBRV	TSH TT3 TT4(TT4)	0.008511	1.08E-13
B59	B115	WBRV	TSH TT3 TT4(TT3)	0.007399	1.05E-10
B59	B212	WBRV	IOP-L	0.01332	0
B6	B137	WB-Fe	AFU	0.00565	8.11E-07
B6	B140	WB-Fe	r-GT	0.005998	1.64E-07
B6	B126	WB-Fe	Alb	0.006532	1.18E-08
B6	B40	WB-Fe	WBC	0.006331	3.25E-08
B6	B128	WB-Fe	ALT	0.008575	7.08E-14
B6	B29	WB-Fe	E2	-0.00562	9.33E-07
B6	B134	WB-Fe	CHE	0.008973	4.88E-15
B6	B70	WB-Fe	LDL-C	0.006048	1.29E-07
B6	B72	WB-Fe	HDL-C	-0.00726	2.34E-10
B6	B32	WB-Fe	Testo	0.014461	0
B6	B189	WB-Fe	eGFB	-0.00612	9.28E-08
B6	B141	WB-Fe	AST/ALT	-0.00957	0
B6	B145	WB-Fe	BGP	-0.00677	3.40E-09
B6	B44	WB-Fe	MCH	0.012148	0
B6	B45	WB-Fe	MCHC	0.017503	0
B6	B46	WB-Fe	RBC Count	0.020118	0
B6	B190	WB-Fe	Cre	0.014423	0
B6	B129	WB-Fe	IBIL	0.006605	8.09E-09
B6	B222	WB-Fe	NS	0.014153	0
B6	B195	WB-Fe	Na	0.005676	7.23E-07
B6	B176	WB-Fe	Ucr	0.006342	3.08E-08
B6	B174	WB-Fe	Urea/Cr	-0.00762	2.94E-11
B6	B192	WB-Fe	UA	0.014128	0
B6	B138	WB-Fe	PAB	0.012725	0
B6	B60	WB-Fe	WBRVI	0.008325	3.65E-13
B6	B62	WB-Fe	WBHSRVI	0.009671	0
B6	B7	WB-Fe	WB-Cop	-0.0158	0
B6	B8	WB-Fe	WB-Zinc	0.567008	0
B6	B64	WB-Fe	WBRSR(mPas_5)	0.008891	8.44E-15
B6	B65	WB-Fe	WBRSR(mPas_1)	0.008085	1.68E-12
B6	B66	WB-Fe	WBRSR(mPas_100)	0.009065	2.44E-15
B6	B67	WB-Fe	WBRSR(mPas_50)	0.009036	3.11E-15
B6	B27	WB-Fe	LDH	0.006113	9.47E-08
B6	B221	WB-Fe	Hei	0.013266	0
B6	B200	WB-Fe	OP	0.00671	4.68E-09
B6	B220	WB-Fe	SBP	0.007201	3.24E-10

B6	B218	WB-Fe	DBP	0.007711	1.67E-11
B6	B216	WB-Fe	BMI	0.013835	0
B6	B215	WB-Fe	BMI	0.008937	6.22E-15
B6	B210	WB-Fe	Hip	0.008656	4.13E-14
B6	B209	WB-Fe	Smoking	0.006995	1.02E-09
B6	B51	WB-Fe	Hb	0.029163	0
B6	B206	WB-Fe	Waist	0.011707	0
B6	B204	WB-Fe	Drinking	0.007805	9.50E-12
B6	B113	WB-Fe	FT3	0.008991	4.00E-15
B6	B76	WB-Fe	ApoB	0.007388	1.12E-10
B6	B133	WB-Fe	TBIL	0.006179	6.86E-08
B60	B137	WBRVI	AFU	0.028921	0
B60	B92	WBRVI	ALDH2-Glu504Glu	-0.00861	5.51E-14
B60	B93	WBRVI	ALDH2-Glu504Lys	0.007373	1.22E-10
B60	B160	WBRVI	PH	-0.00954	0
B60	B140	WBRVI	r-GT	0.042959	0
B60	B126	WBRVI	Alb	0.036866	0
B60	B127	WBRVI	A-G ratio	0.034292	0
B60	B161	WBRVI	LEU	-0.02063	0
B60	B128	WBRVI	ALT	0.037697	0
B60	B111	WBRVI	TSH	-0.02411	0
B60	B124	WBRVI	ACTH	0.034631	0
B60	B134	WBRVI	CHE	0.058938	0
B60	B166	WBRVI	Pro	0.021104	0
B60	B70	WBRVI	LDL-C	0.025391	0
B60	B188	WBRVI	Ca	0.006478	1.55E-08
B60	B71	WBRVI	TG	0.025983	0
B60	B72	WBRVI	HDL-C	-0.03416	0
B60	B189	WBRVI	eGFB	-0.00638	2.59E-08
B60	B141	WBRVI	AST/ALT	-0.0389	0
B60	B146	WBRVI	CysC	0.007931	4.38E-12
B60	B181	WBRVI	APTT	-0.02834	0
B60	B190	WBRVI	Cre	0.066281	0
B60	B193	WBRVI	K	0.006016	1.50E-07
B60	B129	WBRVI	IBIL	0.029247	0
B60	B150	WBRVI	ALP	0.028572	0
B60	B84	WBRVI	C-PSA	0.007706	1.72E-11
B60	B222	WBRVI	NS	0.080105	0
B60	B99	WBRVI	anti-PM-Scl	0.005801	4.09E-07
B60	B105	WBRVI	anti-U1RNP	-0.01158	0
B60	B95	WBRVI	anti-cenpb	-0.00661	7.70E-09
B60	B98	WBRVI	anti-Jo-1	0.005614	9.51E-07
B60	B100	WBRVI	anti-Ro52	-0.01454	0
B60	B101	WBRVI	anti-Sc70	-0.00818	9.14E-13

B60	B103	WBRVI	anti-SSA/Ro	-0.02398	0
B60	B104	WBRVI	anti-SSB	-0.00696	1.23E-09
B60	B107	WBRVI	AnuA	-0.00704	7.91E-10
B60	B148	WBRVI	Anti_TPO	-0.01761	0
B60	B149	WBRVI	TGAb	-0.01665	0
B60	B109	WBRVI	AMA-M2	-0.01041	0
B60	B110	WBRVI	AHA	-0.01124	0
B60	B198	WBRVI	P	-0.034	0
B60	B194	WBRVI	Cl	-0.02887	0
B60	B199	WBRVI	Mg	0.010432	0
B60	B156	WBRVI	IgE	0.006334	3.21E-08
B60	B157	WBRVI	IgG	-0.03951	0
B60	B158	WBRVI	IgM	-0.02255	0
B60	B195	WBRVI	Na	0.009325	4.44E-16
B60	B176	WBRVI	Ucr	0.04342	0
B60	B191	WBRVI	Urea	0.008277	4.95E-13
B60	B174	WBRVI	Urea/Cr	-0.0456	0
B60	B192	WBRVI	UA	0.069495	0
B60	B170	WBRVI	U-GLU	0.013816	0
B60	B179	WBRVI	α1-MG	0.032337	0
B60	B182	WBRVI	TT	0.025805	0
B60	B125	WBRVI	Cor	0.036431	0
B60	B138	WBRVI	PAB	0.084846	0
B60	B130	WBRVI	GLB	-0.02913	0
B60	B61	WBRVI	WBHSRV	0.54836	0
B60	B62	WBRVI	WBHSRVI	0.812154	0
B60	B63	WBRVI	WBRSR(mPas_200)	0.079658	0
B60	B64	WBRVI	WBRSR(mPas_5)	0.900017	0
B60	B65	WBRVI	WBRSR(mPas_1)	0.927042	0
B60	B66	WBRVI	WBRSR(mPas_100)	0.759581	0
B60	B67	WBRVI	WBRSR(mPas_50)	0.788976	0
B60	B86	WBRVI	TBhCG	-0.00729	1.98E-10
B60	B221	WBRVI	Hei	0.072385	0
B60	B87	WBRVI	NSE	0.007398	1.05E-10
B60	B187	WBRVI	NSE	0.007042	7.85E-10
B60	B200	WBRVI	OP	0.018703	0
B60	B228	WBRVI	OS	0.005861	3.11E-07
B60	B220	WBRVI	SBP	0.01503	0
B60	B219	WBRVI	OT	-0.01025	0
B60	B218	WBRVI	DBP	0.034684	0
B60	B88	WBRVI	CA 242	-0.00715	4.32E-10
B60	B216	WBRVI	BMI	0.068076	0
B60	B215	WBRVI	BMI	0.038192	0
B60	B131	WBRVI	AST	0.019098	0

B60	B210	WBRVI	Hip	0.042245	0
B60	B209	WBRVI	Smoking	0.053253	0
B60	B90	WBRVI	CYFRA 21-1	0.012188	0
B60	B139	WBRVI	ADA	-0.02137	0
B60	B183	WBRVI	PTA	-0.0443	0
B60	B184	WBRVI	INR	0.019578	0
B60	B185	WBRVI	PT	-0.01533	0
B60	B186	WBRVI	PFC	-0.0098	0
B60	B69	WBRVI	PV	-0.15533	0
B60	B173	WBRVI	NTT	-0.01236	0
B60	B207	WBRVI	WHR	0.006334	3.21E-08
B60	B206	WBRVI	Waist	0.059731	0
B60	B78	WBRVI	HBcAb	-0.00926	6.66E-16
B60	B197	WBRVI	AG	0.025977	0
B60	B204	WBRVI	Drinking	0.051587	0
B60	B112	WBRVI	FT4	0.013312	0
B60	B113	WBRVI	FT3	0.033214	0
B60	B211	WBRVI	IOP-R	0.021535	0
B60	B73	WBRVI	ApoA	-0.01808	0
B60	B74	WBRVI	ApoA1	-0.05382	0
B60	B75	WBRVI	ApoA1/B	-0.03767	0
B60	B76	WBRVI	ApoB	0.05363	0
B60	B132	WBRVI	Dbil	0.01837	0
B60	B81	WBRVI	TSGF	-0.02518	0
B60	B77	WBRVI	TC	0.010246	0
B60	B133	WBRVI	TBIL	0.028358	0
B60	B135	WBRVI	TBA	0.007356	1.34E-10
B60	B114	WBRVI	TSH TT3 TT4(TT4)	-0.01944	0
B60	B115	WBRVI	TSH TT3 TT4(TT3)	0.017597	0
B60	B212	WBRVI	IOP-L	0.023447	0
B61	B137	WBHSRV	AFU	0.020753	0
B61	B160	WBHSRV	PH	-0.01359	0
B61	B140	WBHSRV	r-GT	0.038829	0
B61	B126	WBHSRV	Alb	0.025219	0
B61	B161	WBHSRV	LEU	-0.00841	2.14E-13
B61	B128	WBHSRV	ALT	0.029542	0
B61	B121	WBHSRV	C3	0.009539	0
B61	B123	WBHSRV	hs-CRP1	0.039522	0
B61	B111	WBHSRV	TSH	-0.01098	0
B61	B124	WBHSRV	ACTH	0.022417	0
B61	B134	WBHSRV	CHE	0.051755	0
B61	B166	WBHSRV	Pro	0.013999	0
B61	B70	WBHSRV	LDL-C	0.02239	0
B61	B188	WBHSRV	Ca	0.006328	3.31E-08

B61	B71	WBHSRV	TG	0.035729	0
B61	B72	WBHSRV	HDL-C	-0.03028	0
B61	B141	WBHSRV	AST/ALT	-0.02702	0
B61	B181	WBHSRV	APTT	-0.02502	0
B61	B190	WBHSRV	Cre	0.035814	0
B61	B129	WBHSRV	IBIL	0.015665	0
B61	B150	WBHSRV	ALP	0.027958	0
B61	B222	WBHSRV	NS	0.051892	0
B61	B105	WBHSRV	anti-U1RNP	-0.00586	3.19E-07
B61	B98	WBHSRV	anti-Jo-1	0.008335	3.40E-13
B61	B103	WBHSRV	anti-SSA/Ro	-0.00811	1.45E-12
B61	B107	WBHSRV	AnuA	-0.00999	0
B61	B148	WBHSRV	Anti_TPO	-0.00781	9.05E-12
B61	B149	WBHSRV	TGAb	-0.00741	9.89E-11
B61	B108	WBHSRV	Anti-Cyclin	-0.00575	5.06E-07
B61	B198	WBHSRV	P	-0.01801	0
B61	B194	WBHSRV	Cl	-0.04476	0
B61	B199	WBHSRV	Mg	0.009468	0
B61	B155	WBHSRV	IgA	0.030972	0
B61	B156	WBHSRV	IgE	0.007946	4.01E-12
B61	B157	WBHSRV	IgG	0.008793	1.64E-14
B61	B195	WBHSRV	Na	-0.00689	1.82E-09
B61	B176	WBHSRV	Ucr	0.024229	0
B61	B178	WBHSRV	Ig-U	0.006334	3.20E-08
B61	B174	WBHSRV	Urea/Cr	-0.02387	0
B61	B192	WBHSRV	UA	0.048657	0
B61	B170	WBHSRV	U-GLU	0.016031	0
B61	B179	WBHSRV	α1-MG	0.024499	0
B61	B182	WBHSRV	TT	0.008588	6.48E-14
B61	B125	WBHSRV	Cor	0.03944	0
B61	B138	WBHSRV	PAB	0.062107	0
B61	B130	WBHSRV	GLB	0.018838	0
B61	B62	WBHSRV	WBHSRVI	0.721489	0
B61	B63	WBHSRV	WBRSR(mPas_200)	0.07399	0
B61	B64	WBHSRV	WBRSR(mPas_5)	0.664765	0
B61	B65	WBHSRV	WBRSR(mPas_1)	0.574121	0
B61	B66	WBHSRV	WBRSR(mPas_100)	0.742621	0
B61	B67	WBHSRV	WBRSR(mPas_50)	0.734691	0
B61	B221	WBHSRV	Hei	0.038117	0
B61	B87	WBHSRV	NSE	0.006905	1.65E-09
B61	B187	WBHSRV	NSE	0.0059	2.59E-07
B61	B220	WBHSRV	SBP	0.020445	0
B61	B218	WBHSRV	DBP	0.029571	0
B61	B216	WBHSRV	BMI	0.046847	0

B61	B215	WBHSRV	BMI	0.034752	0
B61	B131	WBHSRV	AST	0.016511	0
B61	B210	WBHSRV	Hip	0.033196	0
B61	B209	WBHSRV	Smoking	0.024406	0
B61	B183	WBHSRV	PTA	-0.01247	0
B61	B184	WBHSRV	INR	0.006966	1.19E-09
B61	B185	WBHSRV	PT	-0.00717	3.82E-10
B61	B186	WBHSRV	PFC	0.023364	0
B61	B69	WBHSRV	PV	0.051181	0
B61	B173	WBHSRV	NTT	-0.00709	6.05E-10
B61	B206	WBHSRV	Waist	0.046464	0
B61	B78	WBHSRV	HBcAb	0.007553	4.27E-11
B61	B197	WBHSRV	AG	0.020864	0
B61	B204	WBHSRV	Drinking	0.025803	0
B61	B112	WBHSRV	FT4	0.01595	0
B61	B113	WBHSRV	FT3	0.021941	0
B61	B211	WBHSRV	IOP-R	0.013762	0
B61	B74	WBHSRV	ApoA1	-0.06033	0
B61	B75	WBHSRV	ApoA1/B	-0.04257	0
B61	B76	WBHSRV	ApoB	0.071348	0
B61	B132	WBHSRV	Dbil	0.010967	0
B61	B81	WBHSRV	TSGF	-0.00891	7.55E-15
B61	B77	WBHSRV	TC	0.017326	0
B61	B133	WBHSRV	TBIL	0.015523	0
B61	B91	WBHSRV	TP	0.031907	0
B61	B115	WBHSRV	TSH TT3 TT4(TT3)	0.013947	0
B61	B212	WBHSRV	IOP-L	0.010468	0
B62	B137	WBHSRVI	AFU	0.03578	0
B62	B160	WBHSRVI	PH	-0.01218	0
B62	B140	WBHSRVI	r-GT	0.051505	0
B62	B126	WBHSRVI	Alb	0.033307	0
B62	B127	WBHSRVI	A-G ratio	0.025907	0
B62	B161	WBHSRVI	LEU	-0.02026	0
B62	B128	WBHSRVI	ALT	0.045093	0
B62	B121	WBHSRVI	C3	0.006815	2.68E-09
B62	B123	WBHSRVI	hs-CRP1	0.008028	2.40E-12
B62	B111	WBHSRVI	TSH	-0.02424	0
B62	B124	WBHSRVI	ACTH	0.035326	0
B62	B134	WBHSRVI	CHE	0.069042	0
B62	B166	WBHSRVI	Pro	0.022421	0
B62	B70	WBHSRVI	LDL-C	0.028079	0
B62	B188	WBHSRVI	Ca	0.007892	5.58E-12
B62	B71	WBHSRVI	TG	0.037735	0
B62	B72	WBHSRVI	HDL-C	-0.04922	0

B62	B189	WBHSRVI	eGFB	-0.00962	0
B62	B141	WBHSRVI	AST/ALT	-0.04858	0
B62	B146	WBHSRVI	CysC	0.006931	1.44E-09
B62	B181	WBHSRVI	APTT	-0.02509	0
B62	B190	WBHSRVI	Cre	0.069288	0
B62	B129	WBHSRVI	IBIL	0.030192	0
B62	B150	WBHSRVI	ALP	0.033746	0
B62	B84	WBHSRVI	C-PSA	0.0096	0
B62	B222	WBHSRVI	NS	0.088696	0
B62	B105	WBHSRVI	anti-U1RNP	-0.0107	0
B62	B95	WBHSRVI	anti-cenpb	-0.00784	7.66E-12
B62	B98	WBHSRVI	anti-Jo-1	0.009509	0
B62	B100	WBHSRVI	anti-Ro52	-0.01204	0
B62	B101	WBHSRVI	anti-Sc70	-0.00781	9.33E-12
B62	B103	WBHSRVI	anti-SSA/Ro	-0.02442	0
B62	B107	WBHSRVI	AnuA	-0.01193	0
B62	B151	WBHSRVI	Anti-CCP	-0.00962	0
B62	B148	WBHSRVI	Anti_TPO	-0.01838	0
B62	B149	WBHSRVI	TGAb	-0.01702	0
B62	B109	WBHSRVI	AMA-M2	-0.00682	2.59E-09
B62	B110	WBHSRVI	AHA	-0.01277	0
B62	B198	WBHSRVI	P	-0.03615	0
B62	B194	WBHSRVI	Cl	-0.04633	0
B62	B199	WBHSRVI	Mg	0.014951	0
B62	B155	WBHSRVI	IgA	0.006797	2.95E-09
B62	B157	WBHSRVI	IgG	-0.04388	0
B62	B158	WBHSRVI	IgM	-0.02278	0
B62	B195	WBHSRVI	Na	0.009242	6.66E-16
B62	B176	WBHSRVI	Ucr	0.045665	0
B62	B178	WBHSRVI	Ig-U	0.005819	3.77E-07
B62	B191	WBHSRVI	Urea	0.010202	0
B62	B174	WBHSRVI	Urea/Cr	-0.04592	0
B62	B192	WBHSRVI	UA	0.077661	0
B62	B170	WBHSRVI	U-GLU	0.016868	0
B62	B177	WBHSRVI	ACR	-0.00599	1.72E-07
B62	B179	WBHSRVI	α1-MG	0.035744	0
B62	B180	WBHSRVI	Tf	0.006234	5.24E-08
B62	B182	WBHSRVI	TT	0.035255	0
B62	B125	WBHSRVI	Cor	0.033762	0
B62	B138	WBHSRVI	PAB	0.097056	0
B62	B130	WBHSRVI	GLB	-0.01903	0
B62	B63	WBHSRVI	WBRSR(mPas_200)	0.093264	0
B62	B64	WBHSRVI	WBRSR(mPas_5)	0.834503	0
B62	B65	WBHSRVI	WBRSR(mPas_1)	0.739122	0

B62	B66	WBHSRVI	WBRSR(mPas_100)	0.907817	0
B62	B67	WBHSRVI	WBRSR(mPas_50)	0.903004	0
B62	B221	WBHSRVI	Hei	0.077001	0
B62	B87	WBHSRVI	NSE	0.010134	0
B62	B187	WBHSRVI	NSE	0.010071	0
B62	B200	WBHSRVI	OP	0.020139	0
B62	B220	WBHSRVI	SBP	0.022064	0
B62	B219	WBHSRVI	OT	-0.01082	0
B62	B218	WBHSRVI	DBP	0.039489	0
B62	B88	WBHSRVI	CA 242	-0.00752	5.33E-11
B62	B216	WBHSRVI	BMI	0.078295	0
B62	B215	WBHSRVI	BMI	0.048405	0
B62	B131	WBHSRVI	AST	0.020376	0
B62	B210	WBHSRVI	Hip	0.049138	0
B62	B209	WBHSRVI	Smoking	0.053638	0
B62	B90	WBHSRVI	CYFRA 21-1	0.009225	8.88E-16
B62	B139	WBHSRVI	ADA	-0.01207	0
B62	B183	WBHSRVI	PTA	-0.02575	0
B62	B184	WBHSRVI	INR	0.007307	1.78E-10
B62	B185	WBHSRVI	PT	-0.01625	0
B62	B186	WBHSRVI	PFC	-0.01428	0
B62	B69	WBHSRVI	PV	-0.19438	0
B62	B173	WBHSRVI	NTT	-0.01508	0
B62	B207	WBHSRVI	WHR	0.007381	1.17E-10
B62	B206	WBHSRVI	Waist	0.069807	0
B62	B78	WBHSRVI	HBcAb	0.005861	3.10E-07
B62	B197	WBHSRVI	AG	0.033224	0
B62	B204	WBHSRVI	Drinking	0.052742	0
B62	B113	WBHSRVI	FT3	0.035293	0
B62	B211	WBHSRVI	IOP-R	0.017523	0
B62	B73	WBHSRVI	ApoA	-0.0161	0
B62	B74	WBHSRVI	ApoA1	-0.09626	0
B62	B75	WBHSRVI	ApoA1/B	-0.06082	0
B62	B76	WBHSRVI	ApoB	0.085183	0
B62	B132	WBHSRVI	Dbil	0.02261	0
B62	B77	WBHSRVI	TC	0.015431	0
B62	B133	WBHSRVI	TBIL	0.030226	0
B62	B135	WBHSRVI	TBA	0.008658	4.09E-14
B62	B114	WBHSRVI	TSH TT3 TT4(TT4)	-0.02338	0
B62	B115	WBHSRVI	TSH TT3 TT4(TT3)	0.020391	0
B62	B212	WBHSRVI	IOP-L	0.017718	0
B63	B140	WBRSR(mPas_200)	r-GT	0.006179	6.88E-08
B63	B134	WBRSR(mPas_200)	CHE	0.007841	7.62E-12
B63	B190	WBRSR(mPas_200)	Cre	0.008113	1.41E-12

B63	B222	WBRSR(mPas_200)	NS	0.008996	4.00E-15
B63	B194	WBRSR(mPas_200)	Cl	-0.00599	1.67E-07
B63	B192	WBRSR(mPas_200)	UA	0.008946	5.77E-15
B63	B125	WBRSR(mPas_200)	Cor	0.005931	2.25E-07
B63	B138	WBRSR(mPas_200)	PAB	0.01032	0
B63	B64	WBRSR(mPas_200)	WBRSR(mPas_5)	0.096483	0
B63	B65	WBRSR(mPas_200)	WBRSR(mPas_1)	0.086244	0
B63	B66	WBRSR(mPas_200)	WBRSR(mPas_100)	0.101358	0
B63	B67	WBRSR(mPas_200)	WBRSR(mPas_50)	0.100746	0
B63	B221	WBRSR(mPas_200)	Hei	0.008157	1.07E-12
B63	B218	WBRSR(mPas_200)	DBP	0.005718	5.98E-07
B63	B216	WBRSR(mPas_200)	BMI	0.008285	4.72E-13
B63	B69	WBRSR(mPas_200)	PV	0.022775	0
B63	B206	WBRSR(mPas_200)	Waist	0.007647	2.46E-11
B63	B74	WBRSR(mPas_200)	ApoA1	-0.00875	2.18E-14
B63	B75	WBRSR(mPas_200)	ApoA1/B	-0.00657	9.89E-09
B63	B76	WBRSR(mPas_200)	ApoB	0.011183	0
B64	B136	WBRSR(mPas_5)	5'NT	0.005959	1.97E-07
B64	B137	WBRSR(mPas_5)	AFU	0.037182	0
B64	B160	WBRSR(mPas_5)	PH	-0.01654	0
B64	B140	WBRSR(mPas_5)	r-GT	0.055793	0
B64	B126	WBRSR(mPas_5)	Alb	0.047542	0
B64	B127	WBRSR(mPas_5)	A-G ratio	0.011442	0
B64	B161	WBRSR(mPas_5)	LEU	-0.01938	0
B64	B128	WBRSR(mPas_5)	ALT	0.045941	0
B64	B121	WBRSR(mPas_5)	C3	0.008862	1.02E-14
B64	B123	WBRSR(mPas_5)	hs-CRP1	0.03626	0
B64	B111	WBRSR(mPas_5)	TSH	-0.02258	0
B64	B124	WBRSR(mPas_5)	ACTH	0.044739	0
B64	B134	WBRSR(mPas_5)	CHE	0.080016	0
B64	B166	WBRSR(mPas_5)	Pro	0.023421	0
B64	B70	WBRSR(mPas_5)	LDL-C	0.038884	0
B64	B188	WBRSR(mPas_5)	Ca	0.010408	0
B64	B71	WBRSR(mPas_5)	TG	0.042391	0
B64	B72	WBRSR(mPas_5)	HDL-C	-0.03989	0
B64	B189	WBRSR(mPas_5)	eGFB	-0.00933	4.44E-16
B64	B141	WBRSR(mPas_5)	AST/ALT	-0.04293	0
B64	B146	WBRSR(mPas_5)	CysC	0.007481	6.51E-11
B64	B181	WBRSR(mPas_5)	APTT	-0.03365	0
B64	B190	WBRSR(mPas_5)	Cre	0.070868	0
B64	B193	WBRSR(mPas_5)	K	0.018128	0
B64	B129	WBRSR(mPas_5)	IBIL	0.033682	0
B64	B150	WBRSR(mPas_5)	ALP	0.041297	0
B64	B84	WBRSR(mPas_5)	C-PSA	0.008411	2.08E-13

B64	B222	WBRSR(mPas_5)	NS	0.08642	0
B64	B105	WBRSR(mPas_5)	anti-U1RNP	-0.01266	0
B64	B95	WBRSR(mPas_5)	anti-cenpb	-0.00702	8.80E-10
B64	B98	WBRSR(mPas_5)	anti-Jo-1	0.008214	7.44E-13
B64	B100	WBRSR(mPas_5)	anti-Ro52	-0.0108	0
B64	B101	WBRSR(mPas_5)	anti-Sc70	-0.00828	4.89E-13
B64	B103	WBRSR(mPas_5)	anti-SSA/Ro	-0.01562	0
B64	B104	WBRSR(mPas_5)	anti-SSB	-0.00788	5.97E-12
B64	B107	WBRSR(mPas_5)	AnuA	-0.00746	7.46E-11
B64	B148	WBRSR(mPas_5)	Anti_TPO	-0.01585	0
B64	B149	WBRSR(mPas_5)	TGAb	-0.01484	0
B64	B109	WBRSR(mPas_5)	AMA-M2	-0.00669	5.13E-09
B64	B110	WBRSR(mPas_5)	AHA	-0.00659	8.79E-09
B64	B198	WBRSR(mPas_5)	P	-0.03385	0
B64	B194	WBRSR(mPas_5)	Cl	-0.04922	0
B64	B199	WBRSR(mPas_5)	Mg	0.015757	0
B64	B155	WBRSR(mPas_5)	IgA	0.023766	0
B64	B156	WBRSR(mPas_5)	IgE	0.014269	0
B64	B158	WBRSR(mPas_5)	IgM	-0.01535	0
B64	B176	WBRSR(mPas_5)	Ucr	0.046864	0
B64	B178	WBRSR(mPas_5)	Ig-U	0.010458	0
B64	B191	WBRSR(mPas_5)	Urea	0.010002	0
B64	B174	WBRSR(mPas_5)	Urea/Cr	-0.04719	0
B64	B192	WBRSR(mPas_5)	UA	0.079731	0
B64	B170	WBRSR(mPas_5)	U-GLU	0.020365	0
B64	B179	WBRSR(mPas_5)	α1-MG	0.040275	0
B64	B180	WBRSR(mPas_5)	Tf	0.00915	1.33E-15
B64	B182	WBRSR(mPas_5)	TT	0.019447	0
B64	B125	WBRSR(mPas_5)	Cor	0.054948	0
B64	B138	WBRSR(mPas_5)	PAB	0.096229	0
B64	B130	WBRSR(mPas_5)	GLB	0.007478	6.63E-11
B64	B65	WBRSR(mPas_5)	WBRSR(mPas_1)	0.973614	0
B64	B66	WBRSR(mPas_5)	WBRSR(mPas_100)	0.916792	0
B64	B67	WBRSR(mPas_5)	WBRSR(mPas_50)	0.934203	0
B64	B86	WBRSR(mPas_5)	TBhCG	-0.00628	4.29E-08
B64	B221	WBRSR(mPas_5)	Hei	0.07358	0
B64	B87	WBRSR(mPas_5)	NSE	0.009823	0
B64	B187	WBRSR(mPas_5)	NSE	0.006979	1.11E-09
B64	B200	WBRSR(mPas_5)	OP	0.013933	0
B64	B228	WBRSR(mPas_5)	OS	0.006378	2.57E-08
B64	B220	WBRSR(mPas_5)	SBP	0.024874	0
B64	B219	WBRSR(mPas_5)	OT	-0.01007	0
B64	B218	WBRSR(mPas_5)	DBP	0.045256	0
B64	B88	WBRSR(mPas_5)	CA 242	-0.00674	3.92E-09

B64	B216	WBRSR(mPas_5)	BMI	0.074961	0
B64	B215	WBRSR(mPas_5)	BMI	0.04648	0
B64	B131	WBRSR(mPas_5)	AST	0.027768	0
B64	B210	WBRSR(mPas_5)	Hip	0.047812	0
B64	B209	WBRSR(mPas_5)	Smoking	0.052846	0
B64	B90	WBRSR(mPas_5)	CYFRA 21-1	0.009109	1.78E-15
B64	B139	WBRSR(mPas_5)	ADA	-0.00667	5.77E-09
B64	B183	WBRSR(mPas_5)	PTA	-0.02254	0
B64	B184	WBRSR(mPas_5)	INR	0.020663	0
B64	B185	WBRSR(mPas_5)	PT	-0.01287	0
B64	B186	WBRSR(mPas_5)	PFC	0.023642	0
B64	B69	WBRSR(mPas_5)	PV	0.17087	0
B64	B173	WBRSR(mPas_5)	NTT	-0.01267	0
B64	B207	WBRSR(mPas_5)	WHR	0.00714	4.57E-10
B64	B206	WBRSR(mPas_5)	Waist	0.068259	0
B64	B197	WBRSR(mPas_5)	AG	0.030891	0
B64	B204	WBRSR(mPas_5)	Drinking	0.050944	0
B64	B112	WBRSR(mPas_5)	FT4	0.023595	0
B64	B113	WBRSR(mPas_5)	FT3	0.037038	0
B64	B211	WBRSR(mPas_5)	IOP-R	0.022612	0
B64	B73	WBRSR(mPas_5)	ApoA	-0.0078	1.00E-11
B64	B74	WBRSR(mPas_5)	ApoA1	-0.05925	0
B64	B75	WBRSR(mPas_5)	ApoA1/B	-0.0514	0
B64	B76	WBRSR(mPas_5)	ApoB	0.09233	0
B64	B132	WBRSR(mPas_5)	Dbil	0.016638	0
B64	B81	WBRSR(mPas_5)	TSGF	-0.03057	0
B64	B77	WBRSR(mPas_5)	TC	0.024593	0
B64	B133	WBRSR(mPas_5)	TBIL	0.031511	0
B64	B135	WBRSR(mPas_5)	TBA	0.00806	1.96E-12
B64	B91	WBRSR(mPas_5)	TP	0.035925	0
B64	B114	WBRSR(mPas_5)	TSH TT3 TT4(TT4)	-0.00658	9.25E-09
B64	B115	WBRSR(mPas_5)	TSH TT3 TT4(TT3)	0.022762	0
B64	B212	WBRSR(mPas_5)	IOP-L	0.022229	0
B65	B137	WBRSR(mPas_1)	AFU	0.032851	0
B65	B92	WBRSR(mPas_1)	ALDH2-Glu504Glu	-0.00714	4.44E-10
B65	B93	WBRSR(mPas_1)	ALDH2-Glu504Lys	0.006051	1.27E-07
B65	B160	WBRSR(mPas_1)	PH	-0.015	0
B65	B140	WBRSR(mPas_1)	r-GT	0.049856	0
B65	B126	WBRSR(mPas_1)	Alb	0.045686	0
B65	B127	WBRSR(mPas_1)	A-G ratio	0.015479	0
B65	B161	WBRSR(mPas_1)	LEU	-0.01872	0
B65	B128	WBRSR(mPas_1)	ALT	0.041172	0
B65	B121	WBRSR(mPas_1)	C3	0.008024	2.46E-12
B65	B123	WBRSR(mPas_1)	hs-CRP1	0.027025	0

B65	B111	WBRSR(mPas_1)	TSH	-0.0215	0
B65	B124	WBRSR(mPas_1)	ACTH	0.042875	0
B65	B134	WBRSR(mPas_1)	CHE	0.07271	0
B65	B166	WBRSR(mPas_1)	Pro	0.021736	0
B65	B70	WBRSR(mPas_1)	LDL-C	0.036091	0
B65	B188	WBRSR(mPas_1)	Ca	0.009181	1.11E-15
B65	B71	WBRSR(mPas_1)	TG	0.035869	0
B65	B72	WBRSR(mPas_1)	HDL-C	-0.03308	0
B65	B189	WBRSR(mPas_1)	eGFB	-0.00786	6.61E-12
B65	B141	WBRSR(mPas_1)	AST/ALT	-0.03799	0
B65	B146	WBRSR(mPas_1)	CysC	0.007606	3.13E-11
B65	B181	WBRSR(mPas_1)	APTT	-0.03382	0
B65	B190	WBRSR(mPas_1)	Cre	0.066731	0
B65	B193	WBRSR(mPas_1)	K	0.018175	0
B65	B129	WBRSR(mPas_1)	IBIL	0.031165	0
B65	B150	WBRSR(mPas_1)	ALP	0.037442	0
B65	B84	WBRSR(mPas_1)	C-PSA	0.007593	3.39E-11
B65	B222	WBRSR(mPas_1)	NS	0.080264	0
B65	B99	WBRSR(mPas_1)	anti-PM-Scl	0.006005	1.58E-07
B65	B105	WBRSR(mPas_1)	anti-U1RNP	-0.01216	0
B65	B95	WBRSR(mPas_1)	anti-cenpb	-0.00661	7.85E-09
B65	B98	WBRSR(mPas_1)	anti-Jo-1	0.006351	2.94E-08
B65	B100	WBRSR(mPas_1)	anti-Ro52	-0.01152	0
B65	B101	WBRSR(mPas_1)	anti-Sc70	-0.00812	1.33E-12
B65	B103	WBRSR(mPas_1)	anti-SSA/Ro	-0.01574	0
B65	B104	WBRSR(mPas_1)	anti-SSB	-0.00924	8.88E-16
B65	B151	WBRSR(mPas_1)	Anti-CCP	0.007659	2.28E-11
B65	B148	WBRSR(mPas_1)	Anti_TPO	-0.0152	0
B65	B149	WBRSR(mPas_1)	TGAb	-0.01432	0
B65	B109	WBRSR(mPas_1)	AMA-M2	-0.00777	1.16E-11
B65	B110	WBRSR(mPas_1)	AHA	-0.0066	8.11E-09
B65	B198	WBRSR(mPas_1)	P	-0.03173	0
B65	B194	WBRSR(mPas_1)	Cl	-0.04093	0
B65	B199	WBRSR(mPas_1)	Mg	0.012408	0
B65	B155	WBRSR(mPas_1)	IgA	0.018506	0
B65	B156	WBRSR(mPas_1)	IgE	0.01353	0
B65	B158	WBRSR(mPas_1)	IgM	-0.01543	0
B65	B176	WBRSR(mPas_1)	Ucr	0.043736	0
B65	B178	WBRSR(mPas_1)	Ig-U	0.009242	6.66E-16
B65	B191	WBRSR(mPas_1)	Urea	0.009186	1.11E-15
B65	B174	WBRSR(mPas_1)	Urea/Cr	-0.04485	0
B65	B192	WBRSR(mPas_1)	UA	0.073249	0
B65	B170	WBRSR(mPas_1)	U-GLU	0.018293	0
B65	B179	WBRSR(mPas_1)	α1-MG	0.037103	0

B65	B180	WBRSR(mPas_1)	Tf	0.007774	1.14E-11
B65	B182	WBRSR(mPas_1)	TT	0.017173	0
B65	B125	WBRSR(mPas_1)	Cor	0.052751	0
B65	B138	WBRSR(mPas_1)	PAB	0.087897	0
B65	B66	WBRSR(mPas_1)	WBRSR(mPas_100)	0.820515	0
B65	B67	WBRSR(mPas_1)	WBRSR(mPas_50)	0.848598	0
B65	B86	WBRSR(mPas_1)	TBhCG	-0.00709	6.18E-10
B65	B221	WBRSR(mPas_1)	Hei	0.069111	0
B65	B87	WBRSR(mPas_1)	NSE	0.008195	8.40E-13
B65	B187	WBRSR(mPas_1)	NSE	0.005722	5.87E-07
B65	B200	WBRSR(mPas_1)	OP	0.013399	0
B65	B228	WBRSR(mPas_1)	OS	0.006479	1.54E-08
B65	B220	WBRSR(mPas_1)	SBP	0.021115	0
B65	B219	WBRSR(mPas_1)	OT	-0.00934	4.44E-16
B65	B218	WBRSR(mPas_1)	DBP	0.041347	0
B65	B88	WBRSR(mPas_1)	CA 242	-0.00625	4.77E-08
B65	B216	WBRSR(mPas_1)	BMI	0.068519	0
B65	B215	WBRSR(mPas_1)	BMI	0.041179	0
B65	B131	WBRSR(mPas_1)	AST	0.025649	0
B65	B210	WBRSR(mPas_1)	Hip	0.043492	0
B65	B209	WBRSR(mPas_1)	Smoking	0.051007	0
B65	B90	WBRSR(mPas_1)	CYFRA 21-1	0.009764	0
B65	B139	WBRSR(mPas_1)	ADA	-0.01037	0
B65	B183	WBRSR(mPas_1)	PTA	-0.03007	0
B65	B184	WBRSR(mPas_1)	INR	0.024471	0
B65	B185	WBRSR(mPas_1)	PT	-0.01248	0
B65	B186	WBRSR(mPas_1)	PFC	0.022678	0
B65	B69	WBRSR(mPas_1)	PV	0.153411	0
B65	B173	WBRSR(mPas_1)	NTT	-0.01103	0
B65	B207	WBRSR(mPas_1)	WHR	0.006524	1.23E-08
B65	B206	WBRSR(mPas_1)	Waist	0.062136	0
B65	B78	WBRSR(mPas_1)	HBcAb	-0.00964	0
B65	B197	WBRSR(mPas_1)	AG	0.027575	0
B65	B204	WBRSR(mPas_1)	Drinking	0.048958	0
B65	B112	WBRSR(mPas_1)	FT4	0.024672	0
B65	B113	WBRSR(mPas_1)	FT3	0.035315	0
B65	B211	WBRSR(mPas_1)	IOP-R	0.022321	0
B65	B73	WBRSR(mPas_1)	ApoA	-0.00812	1.36E-12
B65	B74	WBRSR(mPas_1)	ApoA1	-0.0428	0
B65	B75	WBRSR(mPas_1)	ApoA1/B	-0.04163	0
B65	B76	WBRSR(mPas_1)	ApoB	0.077727	0
B65	B132	WBRSR(mPas_1)	Dbil	0.014358	0
B65	B81	WBRSR(mPas_1)	TSGF	-0.03783	0
B65	B77	WBRSR(mPas_1)	TC	0.021417	0

B65	B133	WBRSR(mPas_1)	TBIL	0.028883	0
B65	B135	WBRSR(mPas_1)	TBA	0.007207	3.13E-10
B65	B91	WBRSR(mPas_1)	TP	0.029112	0
B65	B114	WBRSR(mPas_1)	TSH TT3 TT4(TT4)	-0.00578	4.47E-07
B65	B115	WBRSR(mPas_1)	TSH TT3 TT4(TT3)	0.02104	0
B65	B212	WBRSR(mPas_1)	IOP-L	0.022744	0
B66	B136	WBRSR(mPas_100)	5'NT	0.006625	7.31E-09
B66	B137	WBRSR(mPas_100)	AFU	0.04083	0
B66	B160	WBRSR(mPas_100)	PH	-0.01741	0
B66	B140	WBRSR(mPas_100)	r-GT	0.059224	0
B66	B126	WBRSR(mPas_100)	Alb	0.04402	0
B66	B161	WBRSR(mPas_100)	LEU	-0.01836	0
B66	B128	WBRSR(mPas_100)	ALT	0.048667	0
B66	B121	WBRSR(mPas_100)	C3	0.006945	1.33E-09
B66	B123	WBRSR(mPas_100)	hs-CRP1	0.046471	0
B66	B111	WBRSR(mPas_100)	TSH	-0.02125	0
B66	B124	WBRSR(mPas_100)	ACTH	0.041105	0
B66	B134	WBRSR(mPas_100)	CHE	0.080342	0
B66	B166	WBRSR(mPas_100)	Pro	0.023129	0
B66	B70	WBRSR(mPas_100)	LDL-C	0.039802	0
B66	B188	WBRSR(mPas_100)	Ca	0.011293	0
B66	B71	WBRSR(mPas_100)	TG	0.049005	0
B66	B72	WBRSR(mPas_100)	HDL-C	-0.04752	0
B66	B189	WBRSR(mPas_100)	eGFB	-0.01186	0
B66	B141	WBRSR(mPas_100)	AST/ALT	-0.04645	0
B66	B146	WBRSR(mPas_100)	CysC	0.006717	4.50E-09
B66	B181	WBRSR(mPas_100)	APTT	-0.02735	0
B66	B190	WBRSR(mPas_100)	Cre	0.069334	0
B66	B193	WBRSR(mPas_100)	K	0.012216	0
B66	B129	WBRSR(mPas_100)	IBIL	0.032719	0
B66	B150	WBRSR(mPas_100)	ALP	0.042493	0
B66	B84	WBRSR(mPas_100)	C-PSA	0.009589	0
B66	B222	WBRSR(mPas_100)	NS	0.088589	0
B66	B105	WBRSR(mPas_100)	anti-U1RNP	-0.01138	0
B66	B95	WBRSR(mPas_100)	anti-cenpb	-0.00733	1.57E-10
B66	B98	WBRSR(mPas_100)	anti-Jo-1	0.011125	0
B66	B100	WBRSR(mPas_100)	anti-Ro52	-0.00623	5.38E-08
B66	B101	WBRSR(mPas_100)	anti-Sc70	-0.00685	2.23E-09
B66	B103	WBRSR(mPas_100)	anti-SSA/Ro	-0.01275	0
B66	B107	WBRSR(mPas_100)	AnuA	-0.00979	0
B66	B148	WBRSR(mPas_100)	Anti_TPO	-0.01457	0
B66	B149	WBRSR(mPas_100)	TGAb	-0.01364	0
B66	B109	WBRSR(mPas_100)	AMA-M2	-0.00657	9.88E-09
B66	B110	WBRSR(mPas_100)	AHA	-0.00899	4.44E-15

B66	B154	WBRSR(mPas_100)	RF	0.006366	2.73E-08
B66	B198	WBRSR(mPas_100)	P	-0.03364	0
B66	B194	WBRSR(mPas_100)	Cl	-0.05712	0
B66	B199	WBRSR(mPas_100)	Mg	0.017739	0
B66	B155	WBRSR(mPas_100)	IgA	0.030033	0
B66	B156	WBRSR(mPas_100)	IgE	0.01184	0
B66	B157	WBRSR(mPas_100)	IgG	-0.00731	1.80E-10
B66	B158	WBRSR(mPas_100)	IgM	-0.01417	0
B66	B176	WBRSR(mPas_100)	Ucr	0.04672	0
B66	B178	WBRSR(mPas_100)	Ig-U	0.010539	0
B66	B191	WBRSR(mPas_100)	Urea	0.010656	0
B66	B174	WBRSR(mPas_100)	Urea/Cr	-0.04509	0
B66	B192	WBRSR(mPas_100)	UA	0.081483	0
B66	B170	WBRSR(mPas_100)	U-GLU	0.021988	0
B66	B179	WBRSR(mPas_100)	α1-MG	0.039665	0
B66	B180	WBRSR(mPas_100)	Tf	0.010095	0
B66	B182	WBRSR(mPas_100)	TT	0.021359	0
B66	B125	WBRSR(mPas_100)	Cor	0.035863	0
B66	B138	WBRSR(mPas_100)	PAB	0.098544	0
B66	B130	WBRSR(mPas_100)	GLB	0.018211	0
B66	B67	WBRSR(mPas_100)	WBRSR(mPas_50)	0.995076	0
B66	B221	WBRSR(mPas_100)	Hei	0.072041	0
B66	B87	WBRSR(mPas_100)	NSE	0.01169	0
B66	B187	WBRSR(mPas_100)	NSE	0.008555	8.06E-14
B66	B200	WBRSR(mPas_100)	OP	0.015827	0
B66	B220	WBRSR(mPas_100)	SBP	0.029859	0
B66	B219	WBRSR(mPas_100)	OT	-0.00985	0
B66	B218	WBRSR(mPas_100)	DBP	0.047247	0
B66	B88	WBRSR(mPas_100)	CA 242	-0.00688	1.87E-09
B66	B216	WBRSR(mPas_100)	BMI	0.077846	0
B66	B215	WBRSR(mPas_100)	BMI	0.051439	0
B66	B131	WBRSR(mPas_100)	AST	0.027969	0
B66	B210	WBRSR(mPas_100)	Hip	0.051031	0
B66	B209	WBRSR(mPas_100)	Smoking	0.04995	0
B66	B90	WBRSR(mPas_100)	CYFRA 21-1	0.007157	4.14E-10
B66	B185	WBRSR(mPas_100)	PT	-0.0134	0
B66	B186	WBRSR(mPas_100)	PFC	0.013827	0
B66	B69	WBRSR(mPas_100)	PV	0.146808	0
B66	B173	WBRSR(mPas_100)	NTT	-0.01321	0
B66	B207	WBRSR(mPas_100)	WHR	0.007633	2.66E-11
B66	B206	WBRSR(mPas_100)	Waist	0.072342	0
B66	B197	WBRSR(mPas_100)	AG	0.034032	0
B66	B204	WBRSR(mPas_100)	Drinking	0.049401	0
B66	B112	WBRSR(mPas_100)	FT4	0.01674	0

B66	B113	WBRSR(mPas_100)	FT3	0.036482	0
B66	B211	WBRSR(mPas_100)	IOP-R	0.020073	0
B66	B73	WBRSR(mPas_100)	ApoA	-0.00568	6.98E-07
B66	B74	WBRSR(mPas_100)	ApoA1	-0.0806	0
B66	B75	WBRSR(mPas_100)	ApoA1/B	-0.06348	0
B66	B76	WBRSR(mPas_100)	ApoB	0.109661	0
B66	B132	WBRSR(mPas_100)	Dbil	0.01839	0
B66	B81	WBRSR(mPas_100)	TSGF	-0.00995	0
B66	B77	WBRSR(mPas_100)	TC	0.02811	0
B66	B133	WBRSR(mPas_100)	TBIL	0.031198	0
B66	B135	WBRSR(mPas_100)	TBA	0.008386	2.46E-13
B66	B91	WBRSR(mPas_100)	TP	0.042931	0
B66	B114	WBRSR(mPas_100)	TSH TT3 TT4(TT4)	-0.0066	8.31E-09
B66	B115	WBRSR(mPas_100)	TSH TT3 TT4(TT3)	0.023694	0
B66	B212	WBRSR(mPas_100)	IOP-L	0.018306	0
B67	B136	WBRSR(mPas_50)	5'NT	0.006529	1.20E-08
B67	B137	WBRSR(mPas_50)	AFU	0.040761	0
B67	B160	WBRSR(mPas_50)	PH	-0.0173	0
B67	B140	WBRSR(mPas_50)	r-GT	0.058994	0
B67	B126	WBRSR(mPas_50)	Alb	0.044997	0
B67	B161	WBRSR(mPas_50)	LEU	-0.01876	0
B67	B128	WBRSR(mPas_50)	ALT	0.048412	0
B67	B121	WBRSR(mPas_50)	C3	0.006855	2.17E-09
B67	B123	WBRSR(mPas_50)	hs-CRP1	0.044888	0
B67	B111	WBRSR(mPas_50)	TSH	-0.02147	0
B67	B124	WBRSR(mPas_50)	ACTH	0.041684	0
B67	B134	WBRSR(mPas_50)	CHE	0.080124	0
B67	B166	WBRSR(mPas_50)	Pro	0.023434	0
B67	B70	WBRSR(mPas_50)	LDL-C	0.040125	0
B67	B188	WBRSR(mPas_50)	Ca	0.011307	0
B67	B71	WBRSR(mPas_50)	TG	0.048274	0
B67	B72	WBRSR(mPas_50)	HDL-C	-0.04675	0
B67	B189	WBRSR(mPas_50)	eGFB	-0.01169	0
B67	B141	WBRSR(mPas_50)	AST/ALT	-0.04613	0
B67	B146	WBRSR(mPas_50)	CysC	0.006848	2.25E-09
B67	B181	WBRSR(mPas_50)	APTT	-0.02765	0
B67	B190	WBRSR(mPas_50)	Cre	0.069945	0
B67	B193	WBRSR(mPas_50)	K	0.012483	0
B67	B129	WBRSR(mPas_50)	IBIL	0.033116	0
B67	B150	WBRSR(mPas_50)	ALP	0.04256	0
B67	B84	WBRSR(mPas_50)	C-PSA	0.009475	0
B67	B222	WBRSR(mPas_50)	NS	0.088923	0
B67	B105	WBRSR(mPas_50)	anti-U1RNP	-0.01162	0
B67	B95	WBRSR(mPas_50)	anti-cenpb	-0.00723	2.76E-10

B67	B98	WBRSR(mPas_50)	anti-Jo-1	0.010941	0
B67	B100	WBRSR(mPas_50)	anti-Ro52	-0.00649	1.43E-08
B67	B101	WBRSR(mPas_50)	anti-Sc70	-0.00696	1.21E-09
B67	B103	WBRSR(mPas_50)	anti-SSA/Ro	-0.01297	0
B67	B107	WBRSR(mPas_50)	AnuA	-0.00944	0
B67	B148	WBRSR(mPas_50)	Anti_TPO	-0.01466	0
B67	B149	WBRSR(mPas_50)	TGAb	-0.01384	0
B67	B109	WBRSR(mPas_50)	AMA-M2	-0.00704	7.87E-10
B67	B110	WBRSR(mPas_50)	AHA	-0.00888	8.88E-15
B67	B154	WBRSR(mPas_50)	RF	0.006243	5.02E-08
B67	B198	WBRSR(mPas_50)	P	-0.03377	0
B67	B194	WBRSR(mPas_50)	Cl	-0.0557	0
B67	B199	WBRSR(mPas_50)	Mg	0.017632	0
B67	B155	WBRSR(mPas_50)	IgA	0.030083	0
B67	B156	WBRSR(mPas_50)	IgE	0.011971	0
B67	B157	WBRSR(mPas_50)	IgG	-0.00791	5.12E-12
B67	B158	WBRSR(mPas_50)	IgM	-0.0147	0
B67	B176	WBRSR(mPas_50)	Ucr	0.047219	0
B67	B178	WBRSR(mPas_50)	Ig-U	0.010514	0
B67	B191	WBRSR(mPas_50)	Urea	0.010449	0
B67	B174	WBRSR(mPas_50)	Urea/Cr	-0.04576	0
B67	B192	WBRSR(mPas_50)	UA	0.08168	0
B67	B170	WBRSR(mPas_50)	U-GLU	0.021992	0
B67	B179	WBRSR(mPas_50)	α1-MG	0.039651	0
B67	B180	WBRSR(mPas_50)	Tf	0.009981	0
B67	B182	WBRSR(mPas_50)	TT	0.021286	0
B67	B125	WBRSR(mPas_50)	Cor	0.037367	0
B67	B138	WBRSR(mPas_50)	PAB	0.098774	0
B67	B130	WBRSR(mPas_50)	GLB	0.016803	0
B67	B221	WBRSR(mPas_50)	Hei	0.072566	0
B67	B87	WBRSR(mPas_50)	NSE	0.011523	0
B67	B187	WBRSR(mPas_50)	NSE	0.008363	2.86E-13
B67	B200	WBRSR(mPas_50)	OP	0.016273	0
B67	B220	WBRSR(mPas_50)	SBP	0.029494	0
B67	B219	WBRSR(mPas_50)	OT	-0.00986	0
B67	B218	WBRSR(mPas_50)	DBP	0.047434	0
B67	B88	WBRSR(mPas_50)	CA 242	-0.00692	1.54E-09
B67	B216	WBRSR(mPas_50)	BMI	0.077873	0
B67	B215	WBRSR(mPas_50)	BMI	0.051112	0
B67	B131	WBRSR(mPas_50)	AST	0.027969	0
B67	B210	WBRSR(mPas_50)	Hip	0.051053	0
B67	B209	WBRSR(mPas_50)	Smoking	0.050672	0
B67	B90	WBRSR(mPas_50)	CYFRA 21-1	0.007251	2.44E-10
B67	B183	WBRSR(mPas_50)	PTA	-0.00722	2.94E-10

B67	B185	WBRSR(mPas_50)	PT	-0.01391	0
B67	B186	WBRSR(mPas_50)	PFC	0.011528	0
B67	B69	WBRSR(mPas_50)	PV	0.145414	0
B67	B173	WBRSR(mPas_50)	NTT	-0.01322	0
B67	B207	WBRSR(mPas_50)	WHR	0.007628	2.75E-11
B67	B206	WBRSR(mPas_50)	Waist	0.072288	0
B67	B197	WBRSR(mPas_50)	AG	0.034039	0
B67	B204	WBRSR(mPas_50)	Drinking	0.049951	0
B67	B112	WBRSR(mPas_50)	FT4	0.017761	0
B67	B113	WBRSR(mPas_50)	FT3	0.036806	0
B67	B211	WBRSR(mPas_50)	IOP-R	0.020957	0
B67	B73	WBRSR(mPas_50)	ApoA	-0.00613	8.72E-08
B67	B74	WBRSR(mPas_50)	ApoA1	-0.07714	0
B67	B75	WBRSR(mPas_50)	ApoA1/B	-0.06176	0
B67	B76	WBRSR(mPas_50)	ApoB	0.107737	0
B67	B132	WBRSR(mPas_50)	Dbil	0.018353	0
B67	B81	WBRSR(mPas_50)	TSGF	-0.01239	0
B67	B77	WBRSR(mPas_50)	TC	0.02784	0
B67	B133	WBRSR(mPas_50)	TBIL	0.031508	0
B67	B135	WBRSR(mPas_50)	TBA	0.008389	2.40E-13
B67	B91	WBRSR(mPas_50)	TP	0.042331	0
B67	B114	WBRSR(mPas_50)	TSH TT3 TT4(TT4)	-0.00657	9.46E-09
B67	B115	WBRSR(mPas_50)	TSH TT3 TT4(TT3)	0.023618	0
B67	B212	WBRSR(mPas_50)	IOP-L	0.01909	0
B69	B137	PV	AFU	0.009355	4.44E-16
B69	B160	PV	PH	-0.01453	0
B69	B140	PV	r-GT	0.019253	0
B69	B126	PV	Alb	0.024067	0
B69	B127	PV	A-G ratio	-0.05445	0
B69	B161	PV	LEU	0.005876	2.90E-07
B69	B128	PV	ALT	0.0086	6.00E-14
B69	B121	PV	C3	0.012645	0
B69	B122	PV	C4	0.012188	0
B69	B123	PV	hs-CRP1	0.101694	0
B69	B111	PV	TSH	0.008035	2.29E-12
B69	B124	PV	ACTH	0.019867	0
B69	B134	PV	CHE	0.04015	0
B69	B70	PV	LDL-C	0.029419	0
B69	B188	PV	Ca	0.007664	2.21E-11
B69	B71	PV	TG	0.026669	0
B69	B189	PV	eGFB	-0.00584	3.45E-07
B69	B181	PV	APTT	-0.01487	0
B69	B193	PV	K	0.030719	0
B69	B150	PV	ALP	0.023857	0

B69	B102	PV	anti-Sm	0.009064	2.66E-15
B69	B103	PV	anti-SSA/Ro	0.026871	0
B69	B107	PV	AnuA	0.005704	6.36E-07
B69	B151	PV	Anti-CCP	0.022775	0
B69	B148	PV	Anti_TPO	0.007209	3.09E-10
B69	B149	PV	TGAb	0.00835	3.09E-13
B69	B109	PV	AMA-M2	0.006359	2.83E-08
B69	B110	PV	AHA	0.013166	0
B69	B154	PV	RF	0.006536	1.15E-08
B69	B198	PV	P	0.006445	1.83E-08
B69	B194	PV	Cl	-0.02943	0
B69	B199	PV	Mg	0.008601	5.95E-14
B69	B155	PV	IgA	0.054288	0
B69	B156	PV	IgE	0.018811	0
B69	B157	PV	IgG	0.098155	0
B69	B158	PV	IgM	0.021485	0
B69	B195	PV	Na	-0.02285	0
B69	B178	PV	Ig-U	0.011914	0
B69	B192	PV	UA	0.008648	4.35E-14
B69	B170	PV	U-GLU	0.011282	0
B69	B175	PV	ALB-U	0.009763	0
B69	B177	PV	ACR	0.012657	0
B69	B179	PV	α1-MG	0.010281	0
B69	B180	PV	Tf	0.008351	3.08E-13
B69	B182	PV	TT	-0.02803	0
B69	B125	PV	Cor	0.045723	0
B69	B138	PV	PAB	0.008497	1.18E-13
B69	B130	PV	GLB	0.087785	0
B69	B221	PV	Hei	-0.01278	0
B69	B200	PV	OP	-0.01499	0
B69	B220	PV	SBP	0.017314	0
B69	B218	PV	DBP	0.018503	0
B69	B215	PV	BMI	0.009074	2.22E-15
B69	B131	PV	AST	0.017833	0
B69	B209	PV	Smoking	-0.00587	2.99E-07
B69	B139	PV	ADA	0.028199	0
B69	B183	PV	PTA	0.040337	0
B69	B184	PV	INR	0.008817	1.38E-14
B69	B185	PV	PT	0.006881	1.88E-09
B69	B186	PV	PFC	0.088952	0
B69	B206	PV	Waist	0.007343	1.45E-10
B69	B204	PV	Drinking	-0.0071	5.65E-10
B69	B112	PV	FT4	0.03458	0
B69	B73	PV	ApoA	0.027433	0

B69	B74	PV	ApoA1	0.032591	0
B69	B75	PV	ApoA1/B	-0.00978	0
B69	B76	PV	ApoB	0.062832	0
B69	B132	PV	Dbil	-0.01076	0
B69	B81	PV	TSGF	-0.03746	0
B69	B77	PV	TC	0.031198	0
B69	B91	PV	TP	0.089977	0
B69	B114	PV	TSH TT3 TT4(TT4)	0.042099	0
B69	B115	PV	TSH TT3 TT4(TT3)	0.008632	4.84E-14
B7	B17	WB-Cop	CD3+CD16+CD56+NK	0.005648	8.19E-07
B7	B21	WB-Cop	CD3+CD4-CD8-	0.00728	2.07E-10
B7	B92	WB-Cop	ALDH2-Glu504Glu	0.007169	3.88E-10
B7	B29	WB-Cop	E2	-0.00961	0
B7	B145	WB-Cop	BGP	0.005741	5.38E-07
B7	B100	WB-Cop	anti-Ro52	0.006655	6.25E-09
B7	B194	WB-Cop	Cl	-0.00624	5.04E-08
B7	B8	WB-Cop	WB-Zinc	0.12801	0
B7	B139	WB-Cop	ADA	0.006056	1.24E-07
B7	B183	WB-Cop	PTA	-0.00804	2.29E-12
B70	B137	LDL-C	AFU	0.011305	0
B70	B160	LDL-C	PH	-0.06247	0
B70	B140	LDL-C	r-GT	0.112318	0
B70	B118	LDL-C	CEA1	0.009693	0
B70	B126	LDL-C	Alb	0.049628	0
B70	B127	LDL-C	A-G ratio	-0.04588	0
B70	B161	LDL-C	LEU	-0.01366	0
B70	B128	LDL-C	ALT	0.109537	0
B70	B111	LDL-C	TSH	0.025447	0
B70	B134	LDL-C	CHE	0.061736	0
B70	B166	LDL-C	Pro	0.018909	0
B70	B188	LDL-C	Ca	0.026635	0
B70	B71	LDL-C	TG	0.054724	0
B70	B72	LDL-C	HDL-C	-0.01584	0
B70	B189	LDL-C	eGFB	-0.18963	0
B70	B141	LDL-C	AST/ALT	-0.12327	0
B70	B146	LDL-C	CysC	0.011112	0
B70	B181	LDL-C	APTT	-0.01575	0
B70	B190	LDL-C	Cre	0.133332	0
B70	B224	LDL-C	pmh	0.008792	1.64E-14
B70	B193	LDL-C	K	0.021605	0
B70	B129	LDL-C	IBIL	-0.01132	0
B70	B150	LDL-C	ALP	0.042074	0
B70	B84	LDL-C	C-PSA	0.007996	2.93E-12
B70	B222	LDL-C	NS	0.17903	0

B70	B148	LDL-C	Anti_TPO	-0.00721	3.15E-10
B70	B149	LDL-C	TGAb	-0.01223	0
B70	B194	LDL-C	Cl	-0.01945	0
B70	B199	LDL-C	Mg	0.022106	0
B70	B155	LDL-C	IgA	-0.00874	2.26E-14
B70	B157	LDL-C	IgG	-0.01311	0
B70	B158	LDL-C	IgM	-0.00662	7.44E-09
B70	B195	LDL-C	Na	0.016551	0
B70	B176	LDL-C	Ucr	0.01846	0
B70	B191	LDL-C	Urea	0.096648	0
B70	B174	LDL-C	Urea/Cr	-0.01725	0
B70	B192	LDL-C	UA	0.154107	0
B70	B170	LDL-C	U-GLU	0.020398	0
B70	B182	LDL-C	TT	0.008865	9.99E-15
B70	B153	LDL-C	Glu	0.020301	0
B70	B138	LDL-C	PAB	0.0379	0
B70	B130	LDL-C	GLB	0.091565	0
B70	B86	LDL-C	TBhCG	-0.00571	6.33E-07
B70	B221	LDL-C	Hei	0.032399	0
B70	B187	LDL-C	NSE	0.007472	6.87E-11
B70	B200	LDL-C	OP	0.018967	0
B70	B220	LDL-C	SBP	0.152743	0
B70	B219	LDL-C	OT	0.040882	0
B70	B218	LDL-C	DBP	0.149865	0
B70	B216	LDL-C	BMI	0.181468	0
B70	B215	LDL-C	BMI	0.220734	0
B70	B131	LDL-C	AST	0.056807	0
B70	B172	LDL-C	KB	-0.01811	0
B70	B210	LDL-C	Hip	0.155064	0
B70	B209	LDL-C	Smoking	0.095043	0
B70	B196	LDL-C	TCO2	0.007556	4.19E-11
B70	B183	LDL-C	PTA	-0.0095	0
B70	B185	LDL-C	PT	-0.01274	0
B70	B186	LDL-C	PFC	0.009659	0
B70	B207	LDL-C	WHR	0.029713	0
B70	B206	LDL-C	Waist	0.235114	0
B70	B205	LDL-C	KDA	0.012208	0
B70	B78	LDL-C	HBcAb	0.009177	1.11E-15
B70	B197	LDL-C	AG	0.016327	0
B70	B204	LDL-C	Drinking	0.065971	0
B70	B80	LDL-C	anti-HP IgG	0.016841	0
B70	B112	LDL-C	FT4	-0.03144	0
B70	B113	LDL-C	FT3	0.009328	4.44E-16
B70	B211	LDL-C	IOP-R	0.010382	0

B70	B201	LDL-C	e-habits	0.02633	0
B70	B73	LDL-C	ApoA	0.065895	0
B70	B74	LDL-C	ApoA1	0.031286	0
B70	B75	LDL-C	ApoA1/B	-0.12256	0
B70	B76	LDL-C	ApoB	0.29875	0
B70	B132	LDL-C	Dbil	-0.17898	0
B70	B77	LDL-C	TC	0.702373	0
B70	B133	LDL-C	TBIL	-0.05625	0
B70	B91	LDL-C	TP	0.108741	0
B70	B212	LDL-C	IOP-L	0.012175	0
B71	B136	TG	5'NT	0.008487	1.27E-13
B71	B137	TG	AFU	0.026811	0
B71	B160	TG	PH	-0.10558	0
B71	B140	TG	r-GT	0.280684	0
B71	B82	TG	CEA	0.007695	1.84E-11
B71	B118	TG	CEA1	0.010273	0
B71	B126	TG	Alb	0.088561	0
B71	B127	TG	A-G ratio	-0.11386	0
B71	B161	TG	LEU	-0.03477	0
B71	B128	TG	ALT	0.203419	0
B71	B120	TG	HCV-cAg	0.012953	0
B71	B121	TG	C3	0.010166	0
B71	B122	TG	C4	0.008321	3.73E-13
B71	B225	TG	hPBG	0.012083	0
B71	B164	TG	COM	-0.00731	1.78E-10
B71	B123	TG	hs-CRP1	0.010398	0
B71	B111	TG	TSH	0.012425	0
B71	B124	TG	ACTH	0.00923	8.88E-16
B71	B134	TG	CHE	0.084892	0
B71	B166	TG	Pro	0.049322	0
B71	B188	TG	Ca	0.020504	0
B71	B72	TG	HDL-C	-0.38296	0
B71	B189	TG	eGFB	-0.10316	0
B71	B141	TG	AST/ALT	-0.17909	0
B71	B146	TG	CysC	0.037524	0
B71	B181	TG	APTT	-0.01561	0
B71	B190	TG	Cre	0.155513	0
B71	B193	TG	K	-0.01137	0
B71	B129	TG	IBIL	-0.03346	0
B71	B150	TG	ALP	0.052395	0
B71	B84	TG	C-PSA	0.009058	2.66E-15
B71	B222	TG	NS	0.286972	0
B71	B148	TG	Anti_TPO	-0.01557	0
B71	B149	TG	TGAb	-0.01816	0

B71	B198	TG	P	-0.01873	0
B71	B194	TG	Cl	-0.02835	0
B71	B199	TG	Mg	0.011226	0
B71	B155	TG	IgA	0.020054	0
B71	B157	TG	IgG	-0.01533	0
B71	B195	TG	Na	-0.0077	1.74E-11
B71	B176	TG	Ucr	0.034027	0
B71	B191	TG	Urea	0.05374	0
B71	B174	TG	Urea/Cr	-0.06696	0
B71	B192	TG	UA	0.30307	0
B71	B170	TG	U-GLU	0.104891	0
B71	B175	TG	ALB-U	0.015604	0
B71	B177	TG	ACR	0.008013	2.63E-12
B71	B179	TG	α1-MG	0.018284	0
B71	B153	TG	Glu	0.039806	0
B71	B138	TG	PAB	0.073812	0
B71	B130	TG	GLB	0.216693	0
B71	B221	TG	Hei	0.13065	0
B71	B87	TG	NSE	0.010395	0
B71	B187	TG	NSE	0.008429	1.86E-13
B71	B200	TG	OP	0.006891	1.79E-09
B71	B220	TG	SBP	0.173343	0
B71	B219	TG	OT	0.011927	0
B71	B218	TG	DBP	0.203935	0
B71	B88	TG	CA 242	-0.00853	9.66E-14
B71	B216	TG	BMI	0.29716	0
B71	B215	TG	BMI	0.300896	0
B71	B131	TG	AST	0.134622	0
B71	B172	TG	KB	-0.01196	0
B71	B210	TG	Hip	0.210564	0
B71	B209	TG	Smoking	0.174543	0
B71	B90	TG	CYFRA 21-1	0.008586	6.57E-14
B71	B139	TG	ADA	-0.02604	0
B71	B196	TG	TCO2	-0.00572	5.92E-07
B71	B185	TG	PT	-0.01324	0
B71	B173	TG	NTT	-0.01005	0
B71	B207	TG	WHR	0.036362	0
B71	B206	TG	Waist	0.322684	0
B71	B197	TG	AG	0.018407	0
B71	B204	TG	Drinking	0.170423	0
B71	B203	TG	DH	0.025036	0
B71	B80	TG	anti-HP IgG	0.006074	1.14E-07
B71	B112	TG	FT4	-0.04249	0
B71	B113	TG	FT3	0.02873	0

B71	B211	TG	IOP-R	0.024473	0
B71	B73	TG	ApoA	-0.02545	0
B71	B74	TG	ApoA1	-0.071	0
B71	B75	TG	ApoA1/B	-0.01134	0
B71	B76	TG	ApoB	0.052268	0
B71	B132	TG	Dbil	-0.09963	0
B71	B77	TG	TC	0.277804	0
B71	B133	TG	TBIL	-0.05322	0
B71	B135	TG	TBA	0.015314	0
B71	B91	TG	TP	0.239175	0
B71	B114	TG	TSH TT3 TT4(TT4)	-0.0326	0
B71	B115	TG	TSH TT3 TT4(TT3)	0.006213	5.83E-08
B71	B212	TG	IOP-L	0.025153	0
B72	B142	HDL-C	25-(OH)D	0.008602	5.91E-14
B72	B137	HDL-C	AFU	-0.01326	0
B72	B160	HDL-C	PH	0.076333	0
B72	B140	HDL-C	r-GT	-0.1392	0
B72	B118	HDL-C	CEA1	-0.00861	5.51E-14
B72	B126	HDL-C	Alb	-0.0065	1.36E-08
B72	B127	HDL-C	A-G ratio	0.083458	0
B72	B161	HDL-C	LEU	0.05353	0
B72	B128	HDL-C	ALT	-0.20615	0
B72	B225	HDL-C	hPBG	-0.00976	0
B72	B164	HDL-C	COM	0.00601	1.55E-07
B72	B123	HDL-C	hs-CRP1	-0.03048	0
B72	B111	HDL-C	TSH	0.016563	0
B72	B124	HDL-C	ACTH	-0.00629	3.89E-08
B72	B134	HDL-C	CHE	-0.06157	0
B72	B166	HDL-C	Pro	-0.08246	0
B72	B189	HDL-C	eGFB	0.057565	0
B72	B141	HDL-C	AST/ALT	0.259041	0
B72	B146	HDL-C	CysC	-0.02819	0
B72	B190	HDL-C	Cre	-0.25863	0
B72	B193	HDL-C	K	0.01109	0
B72	B129	HDL-C	IBIL	0.02371	0
B72	B150	HDL-C	ALP	-0.04288	0
B72	B84	HDL-C	C-PSA	-0.01331	0
B72	B222	HDL-C	NS	-0.38355	0
B72	B148	HDL-C	Anti_TPO	0.018597	0
B72	B149	HDL-C	TGAb	0.020066	0
B72	B198	HDL-C	P	0.026812	0
B72	B85	HDL-C	SCC	-0.006	1.62E-07
B72	B194	HDL-C	Cl	-0.00659	8.57E-09
B72	B155	HDL-C	IgA	-0.01415	0

B72	B157	HDL-C	IgG	0.012986	0
B72	B158	HDL-C	IgM	0.008263	5.43E-13
B72	B195	HDL-C	Na	-0.01533	0
B72	B176	HDL-C	Ucr	-0.06213	0
B72	B178	HDL-C	Ig-U	-0.00733	1.57E-10
B72	B191	HDL-C	Urea	-0.04755	0
B72	B174	HDL-C	Urea/Cr	0.142797	0
B72	B192	HDL-C	UA	-0.37684	0
B72	B170	HDL-C	U-GLU	-0.04628	0
B72	B175	HDL-C	ALB-U	-0.01001	0
B72	B179	HDL-C	α1-MG	-0.0213	0
B72	B180	HDL-C	Tf	-0.00603	1.38E-07
B72	B182	HDL-C	TT	-0.00903	3.11E-15
B72	B125	HDL-C	Cor	0.008015	2.60E-12
B72	B153	HDL-C	Glu	-0.01956	0
B72	B138	HDL-C	PAB	-0.03913	0
B72	B130	HDL-C	GLB	-0.05751	0
B72	B86	HDL-C	TBhCG	0.009394	2.22E-16
B72	B221	HDL-C	Hei	-0.26528	0
B72	B200	HDL-C	OP	-0.01835	0
B72	B220	HDL-C	SBP	-0.11651	0
B72	B219	HDL-C	OT	0.020387	0
B72	B218	HDL-C	DBP	-0.12187	0
B72	B88	HDL-C	CA 242	0.016927	0
B72	B216	HDL-C	BMI	-0.40889	0
B72	B215	HDL-C	BMI	-0.35792	0
B72	B131	HDL-C	AST	-0.07013	0
B72	B172	HDL-C	KB	0.024672	0
B72	B210	HDL-C	Hip	-0.29564	0
B72	B209	HDL-C	Smoking	-0.16393	0
B72	B90	HDL-C	CYFRA 21-1	-0.00819	8.77E-13
B72	B196	HDL-C	TCO2	0.008552	8.24E-14
B72	B173	HDL-C	NTT	0.022222	0
B72	B207	HDL-C	WHR	-0.04233	0
B72	B206	HDL-C	Waist	-0.39315	0
B72	B205	HDL-C	KDA	0.016929	0
B72	B78	HDL-C	HBcAb	0.008097	1.56E-12
B72	B197	HDL-C	AG	-0.01151	0
B72	B204	HDL-C	Drinking	-0.12023	0
B72	B203	HDL-C	DH	-0.03689	0
B72	B80	HDL-C	anti-HP IgG	-0.01167	0
B72	B112	HDL-C	FT4	0.03819	0
B72	B113	HDL-C	FT3	-0.05243	0
B72	B73	HDL-C	ApoA	0.02899	0

B72	B74	HDL-C	ApoA1	0.252805	0
B72	B75	HDL-C	ApoA1/B	0.086103	0
B72	B76	HDL-C	ApoB	-0.03811	0
B72	B132	HDL-C	Dbil	0.068316	0
B72	B81	HDL-C	TSGF	-0.02987	0
B72	B77	HDL-C	TC	0.122275	0
B72	B133	HDL-C	TBIL	0.036277	0
B72	B135	HDL-C	TBA	-0.00824	6.13E-13
B72	B91	HDL-C	TP	-0.05218	0
B72	B114	HDL-C	TSH TT3 TT4(TT4)	0.019044	0
B72	B115	HDL-C	TSH TT3 TT4(TT3)	-0.02401	0
B73	B136	ApoA	5'NT	-0.0064	2.30E-08
B73	B137	ApoA	AFU	-0.02361	0
B73	B140	ApoA	r-GT	-0.02287	0
B73	B126	ApoA	Alb	-0.01813	0
B73	B127	ApoA	A-G ratio	-0.0166	0
B73	B128	ApoA	ALT	-0.02009	0
B73	B123	ApoA	hs-CRP1	0.014101	0
B73	B124	ApoA	ACTH	-0.0064	2.29E-08
B73	B134	ApoA	CHE	-0.00759	3.39E-11
B73	B166	ApoA	Pro	-0.00642	2.05E-08
B73	B189	ApoA	eGFB	-0.00875	2.20E-14
B73	B141	ApoA	AST/ALT	0.012548	0
B73	B190	ApoA	Cre	-0.00912	1.78E-15
B73	B193	ApoA	K	0.022317	0
B73	B222	ApoA	NS	-0.0229	0
B73	B154	ApoA	RF	0.008034	2.32E-12
B73	B198	ApoA	P	0.014025	0
B73	B199	ApoA	Mg	0.006078	1.12E-07
B73	B159	ApoA	β2-MG	-0.00605	1.25E-07
B73	B176	ApoA	Ucr	-0.0069	1.67E-09
B73	B174	ApoA	Urea/Cr	0.00622	5.61E-08
B73	B192	ApoA	UA	-0.01918	0
B73	B170	ApoA	U-GLU	-0.00773	1.46E-11
B73	B179	ApoA	α1-MG	-0.00564	8.65E-07
B73	B182	ApoA	TT	-0.00737	1.26E-10
B73	B138	ApoA	PAB	-0.02642	0
B73	B130	ApoA	GLB	0.011915	0
B73	B221	ApoA	Hei	-0.02417	0
B73	B200	ApoA	OP	-0.00636	2.83E-08
B73	B218	ApoA	DBP	-0.00699	1.06E-09
B73	B216	ApoA	BMI	-0.0248	0
B73	B215	ApoA	BMI	-0.01493	0
B73	B131	ApoA	AST	-0.01493	0

B73	B210	ApoA	Hip	-0.01603	0
B73	B209	ApoA	Smoking	-0.01125	0
B73	B139	ApoA	ADA	0.007752	1.31E-11
B73	B186	ApoA	PFC	0.018223	0
B73	B206	ApoA	Waist	-0.01855	0
B73	B204	ApoA	Drinking	-0.01438	0
B73	B113	ApoA	FT3	-0.01222	0
B73	B74	ApoA	ApoA1	0.063271	0
B73	B75	ApoA	ApoA1/B	-0.08203	0
B73	B76	ApoA	ApoB	0.220151	0
B73	B132	ApoA	Dbil	-0.015	0
B73	B77	ApoA	TC	0.045832	0
B73	B135	ApoA	TBA	-0.02263	0
B73	B114	ApoA	TSH TT3 TT4(TT4)	0.005966	1.90E-07
B74	B137	ApoA1	AFU	-0.00935	4.44E-16
B74	B92	ApoA1	ALDH2-Glu504Glu	0.014405	0
B74	B93	ApoA1	ALDH2-Glu504Lys	-0.01241	0
B74	B140	ApoA1	r-GT	-0.01811	0
B74	B94	ApoA1	ALDH2-Lys504Lys	-0.00643	1.95E-08
B74	B143	ApoA1	β-CTx	-0.01269	0
B74	B126	ApoA1	Alb	0.043187	0
B74	B127	ApoA1	A-G ratio	0.008912	7.11E-15
B74	B161	ApoA1	LEU	0.019324	0
B74	B128	ApoA1	ALT	-0.04725	0
B74	B121	ApoA1	C3	-0.00728	2.07E-10
B74	B123	ApoA1	hs-CRP1	-0.05786	0
B74	B111	ApoA1	TSH	0.019688	0
B74	B124	ApoA1	ACTH	-0.0102	0
B74	B165	ApoA1	bilirubin	-0.00809	1.65E-12
B74	B134	ApoA1	CHE	-0.06623	0
B74	B166	ApoA1	Pro	-0.02553	0
B74	B188	ApoA1	Ca	0.007503	5.74E-11
B74	B141	ApoA1	AST/ALT	0.053786	0
B74	B146	ApoA1	CysC	-0.00967	0
B74	B181	ApoA1	APTT	-0.0215	0
B74	B190	ApoA1	Cre	-0.07246	0
B74	B193	ApoA1	K	0.040426	0
B74	B129	ApoA1	IBIL	-0.01964	0
B74	B150	ApoA1	ALP	-0.02016	0
B74	B84	ApoA1	C-PSA	-0.00749	6.01E-11
B74	B222	ApoA1	NS	-0.10818	0
B74	B95	ApoA1	anti-cenpb	0.007713	1.65E-11
B74	B148	ApoA1	Anti_TPO	0.007453	7.69E-11
B74	B149	ApoA1	TGAb	0.007208	3.11E-10

B74	B154	ApoA1	RF	-0.00704	7.81E-10
B74	B198	ApoA1	P	0.063598	0
B74	B194	ApoA1	Cl	-0.05405	0
B74	B199	ApoA1	Mg	0.037822	0
B74	B155	ApoA1	IgA	-0.01476	0
B74	B157	ApoA1	IgG	0.012839	0
B74	B176	ApoA1	Ucr	-0.04821	0
B74	B178	ApoA1	Ig-U	-0.00804	2.28E-12
B74	B174	ApoA1	Urea/Cr	0.05692	0
B74	B192	ApoA1	UA	-0.09383	0
B74	B170	ApoA1	U-GLU	-0.01231	0
B74	B177	ApoA1	ACR	0.006985	1.07E-09
B74	B179	ApoA1	α1-MG	-0.03048	0
B74	B180	ApoA1	Tf	-0.00813	1.28E-12
B74	B125	ApoA1	Cor	0.01652	0
B74	B138	ApoA1	PAB	-0.03162	0
B74	B221	ApoA1	Hei	-0.09676	0
B74	B87	ApoA1	NSE	-0.00649	1.46E-08
B74	B219	ApoA1	OT	0.008687	3.33E-14
B74	B218	ApoA1	DBP	-0.00764	2.55E-11
B74	B88	ApoA1	CA 242	0.007821	8.57E-12
B74	B216	ApoA1	BMI	-0.11399	0
B74	B215	ApoA1	BMI	-0.083	0
B74	B131	ApoA1	AST	-0.00939	2.22E-16
B74	B210	ApoA1	Hip	-0.07465	0
B74	B209	ApoA1	Smoking	-0.04632	0
B74	B139	ApoA1	ADA	0.006078	1.12E-07
B74	B196	ApoA1	TCO2	0.024544	0
B74	B183	ApoA1	PTA	-0.02356	0
B74	B185	ApoA1	PT	-0.01232	0
B74	B173	ApoA1	NTT	0.009705	0
B74	B207	ApoA1	WHR	-0.01105	0
B74	B206	ApoA1	Waist	-0.10181	0
B74	B78	ApoA1	HBcAb	0.022863	0
B74	B197	ApoA1	AG	0.014146	0
B74	B204	ApoA1	Drinking	-0.02259	0
B74	B80	ApoA1	anti-HP IgG	-0.01038	0
B74	B112	ApoA1	FT4	0.008353	3.04E-13
B74	B201	ApoA1	e-habits	-0.0062	6.30E-08
B74	B75	ApoA1	ApoA1/B	0.295874	0
B74	B76	ApoA1	ApoB	-0.04483	0
B74	B81	ApoA1	TSGF	-0.02724	0
B74	B77	ApoA1	TC	0.078541	0
B74	B133	ApoA1	TBIL	-0.01627	0

B74	B135	ApoA1	TBA	-0.0082	8.34E-13
B74	B91	ApoA1	TP	0.030414	0
B74	B114	ApoA1	TSH TT3 TT4(TT4)	0.01959	0
B74	B115	ApoA1	TSH TT3 TT4(TT3)	-0.00732	1.68E-10
B74	B212	ApoA1	IOP-L	-0.00776	1.25E-11
B75	B136	ApoA1/B	5'NT	-0.00585	3.27E-07
B75	B137	ApoA1/B	AFU	-0.02775	0
B75	B160	ApoA1/B	PH	0.006687	5.29E-09
B75	B140	ApoA1/B	r-GT	-0.02822	0
B75	B127	ApoA1/B	A-G ratio	0.041383	0
B75	B128	ApoA1/B	ALT	-0.02816	0
B75	B123	ApoA1/B	hs-CRP1	-0.02291	0
B75	B124	ApoA1/B	ACTH	-0.00718	3.71E-10
B75	B134	ApoA1/B	CHE	-0.07473	0
B75	B166	ApoA1/B	Pro	-0.01136	0
B75	B188	ApoA1/B	Ca	-0.00579	4.26E-07
B75	B189	ApoA1/B	eGFB	0.018843	0
B75	B141	ApoA1/B	AST/ALT	0.062072	0
B75	B181	ApoA1/B	APTT	0.011803	0
B75	B190	ApoA1/B	Cre	-0.04017	0
B75	B129	ApoA1/B	IBIL	-0.00794	4.05E-12
B75	B150	ApoA1/B	ALP	-0.02559	0
B75	B84	ApoA1/B	C-PSA	-0.00576	4.84E-07
B75	B222	ApoA1/B	NS	-0.06247	0
B75	B149	ApoA1/B	TGAb	0.006115	9.34E-08
B75	B198	ApoA1/B	P	0.023786	0
B75	B194	ApoA1/B	Cl	0.008464	1.47E-13
B75	B199	ApoA1/B	Mg	-0.0179	0
B75	B157	ApoA1/B	IgG	0.013556	0
B75	B158	ApoA1/B	IgM	0.008193	8.51E-13
B75	B195	ApoA1/B	Na	-0.02085	0
B75	B176	ApoA1/B	Ucr	-0.02496	0
B75	B178	ApoA1/B	Ig-U	-0.0059	2.61E-07
B75	B191	ApoA1/B	Urea	-0.01302	0
B75	B174	ApoA1/B	Urea/Cr	0.019231	0
B75	B192	ApoA1/B	UA	-0.05618	0
B75	B170	ApoA1/B	U-GLU	-0.01023	0
B75	B179	ApoA1/B	α1-MG	-0.01788	0
B75	B138	ApoA1/B	PAB	-0.07058	0
B75	B221	ApoA1/B	Hei	-0.03557	0
B75	B200	ApoA1/B	OP	-0.02697	0
B75	B220	ApoA1/B	SBP	-0.0266	0
B75	B218	ApoA1/B	DBP	-0.02963	0
B75	B216	ApoA1/B	BMI	-0.06218	0

B75	B215	ApoA1/B	BMI	-0.0584	0
B75	B131	ApoA1/B	AST	-0.0102	0
B75	B172	ApoA1/B	KB	0.00858	6.84E-14
B75	B210	ApoA1/B	Hip	-0.0457	0
B75	B209	ApoA1/B	Smoking	-0.02778	0
B75	B196	ApoA1/B	TCO2	-0.00754	4.57E-11
B75	B185	ApoA1/B	PT	0.007724	1.55E-11
B75	B186	ApoA1/B	PFC	-0.00838	2.61E-13
B75	B207	ApoA1/B	WHR	-0.00745	7.61E-11
B75	B206	ApoA1/B	Waist	-0.06746	0
B75	B78	ApoA1/B	HBcAb	0.012844	0
B75	B197	ApoA1/B	AG	-0.01183	0
B75	B204	ApoA1/B	Drinking	-0.02216	0
B75	B80	ApoA1/B	anti-HP IgG	-0.00567	7.30E-07
B75	B112	ApoA1/B	FT4	0.018393	0
B75	B211	ApoA1/B	IOP-R	-0.01258	0
B75	B76	ApoA1/B	ApoB	-0.45204	0
B75	B132	ApoA1/B	Dbil	0.02571	0
B75	B77	ApoA1/B	TC	-0.06904	0
B75	B114	ApoA1/B	TSH TT3 TT4(TT4)	0.014433	0
B75	B212	ApoA1/B	IOP-L	-0.01466	0
B76	B136	ApoB	5'NT	0.012015	0
B76	B137	ApoB	AFU	0.03498	0
B76	B160	ApoB	PH	-0.02505	0
B76	B140	ApoB	r-GT	0.067104	0
B76	B118	ApoB	CEA1	0.006242	5.04E-08
B76	B126	ApoB	Alb	0.031667	0
B76	B127	ApoB	A-G ratio	-0.01967	0
B76	B128	ApoB	ALT	0.039652	0
B76	B123	ApoB	hs-CRP1	0.018175	0
B76	B111	ApoB	TSH	0.020482	0
B76	B124	ApoB	ACTH	0.010691	0
B76	B134	ApoB	CHE	0.148628	0
B76	B166	ApoB	Pro	0.010341	0
B76	B188	ApoB	Ca	0.015756	0
B76	B189	ApoB	eGFB	-0.04337	0
B76	B141	ApoB	AST/ALT	-0.03774	0
B76	B146	ApoB	CysC	0.006304	3.72E-08
B76	B181	ApoB	APTT	-0.03808	0
B76	B190	ApoB	Cre	0.046402	0
B76	B193	ApoB	K	0.043747	0
B76	B129	ApoB	IBIL	0.010727	0
B76	B150	ApoB	ALP	0.050289	0
B76	B84	ApoB	C-PSA	0.005799	4.14E-07

B76	B222	ApoB	NS	0.074734	0
B76	B97	ApoB	dsDNA	-0.00785	7.33E-12
B76	B100	ApoB	anti-Ro52	-0.00851	1.08E-13
B76	B103	ApoB	anti-SSA/Ro	-0.0101	0
B76	B104	ApoB	anti-SSB	-0.0087	3.06E-14
B76	B198	ApoB	P	-0.01343	0
B76	B194	ApoB	Cl	-0.05611	0
B76	B199	ApoB	Mg	0.069022	0
B76	B156	ApoB	IgE	0.006927	1.47E-09
B76	B157	ApoB	IgG	-0.0233	0
B76	B158	ApoB	IgM	-0.00996	0
B76	B195	ApoB	Na	0.040871	0
B76	B176	ApoB	Ucr	0.025898	0
B76	B178	ApoB	Ig-U	0.008585	6.64E-14
B76	B191	ApoB	Urea	0.032004	0
B76	B174	ApoB	Urea/Cr	-0.00871	2.82E-14
B76	B192	ApoB	UA	0.073097	0
B76	B170	ApoB	U-GLU	0.024138	0
B76	B179	ApoB	α1-MG	0.023902	0
B76	B182	ApoB	TT	0.014453	0
B76	B125	ApoB	Cor	0.010109	0
B76	B153	ApoB	Glu	0.009657	0
B76	B138	ApoB	PAB	0.147966	0
B76	B130	ApoB	GLB	0.036582	0
B76	B221	ApoB	Hei	0.021267	0
B76	B187	ApoB	NSE	0.008919	6.88E-15
B76	B200	ApoB	OP	0.05806	0
B76	B220	ApoB	SBP	0.058823	0
B76	B219	ApoB	OT	0.006033	1.38E-07
B76	B218	ApoB	DBP	0.063913	0
B76	B216	ApoB	BMI	0.0689	0
B76	B215	ApoB	BMI	0.078114	0
B76	B131	ApoB	AST	0.022718	0
B76	B172	ApoB	KB	-0.00565	8.06E-07
B76	B210	ApoB	Hip	0.053373	0
B76	B209	ApoB	Smoking	0.041066	0
B76	B90	ApoB	CYFRA 21-1	0.008557	7.95E-14
B76	B196	ApoB	TCO2	0.013702	0
B76	B183	ApoB	PTA	-0.01507	0
B76	B185	ApoB	PT	-0.02602	0
B76	B186	ApoB	PFC	0.026238	0
B76	B207	ApoB	WHR	0.010228	0
B76	B206	ApoB	Waist	0.088554	0
B76	B78	ApoB	HBcAb	-0.01376	0

B76	B197	ApoB	AG	0.049732	0
B76	B204	ApoB	Drinking	0.036986	0
B76	B112	ApoB	FT4	-0.03061	0
B76	B211	ApoB	IOP-R	0.024828	0
B76	B132	ApoB	Dbil	-0.05236	0
B76	B81	ApoB	TSGF	0.006561	1.02E-08
B76	B77	ApoB	TC	0.231797	0
B76	B91	ApoB	TP	0.050663	0
B76	B114	ApoB	TSH TT3 TT4(TT4)	-0.01662	0
B76	B212	ApoB	IOP-L	0.024532	0
B77	B137	TC	AFU	0.016095	0
B77	B160	TC	PH	-0.05727	0
B77	B140	TC	r-GT	0.149644	0
B77	B118	TC	CEA1	0.010913	0
B77	B126	TC	Alb	0.054009	0
B77	B127	TC	A-G ratio	-0.08872	0
B77	B128	TC	ALT	0.084454	0
B77	B111	TC	TSH	0.032561	0
B77	B134	TC	CHE	0.057358	0
B77	B188	TC	Ca	0.028306	0
B77	B189	TC	eGFB	-0.18161	0
B77	B141	TC	AST/ALT	-0.07349	0
B77	B146	TC	CysC	0.013297	0
B77	B181	TC	APTT	-0.01666	0
B77	B190	TC	Cre	0.068126	0
B77	B224	TC	pmh	0.006469	1.63E-08
B77	B193	TC	K	0.015774	0
B77	B129	TC	IBIL	-0.01851	0
B77	B150	TC	ALP	0.033567	0
B77	B84	TC	C-PSA	0.005864	3.07E-07
B77	B222	TC	NS	0.102148	0
B77	B149	TC	TGAb	-0.00756	4.13E-11
B77	B85	TC	SCC	-0.00638	2.56E-08
B77	B194	TC	Cl	-0.02296	0
B77	B199	TC	Mg	0.020862	0
B77	B157	TC	IgG	-0.0115	0
B77	B191	TC	Urea	0.084538	0
B77	B174	TC	Urea/Cr	0.017992	0
B77	B192	TC	UA	0.095406	0
B77	B170	TC	U-GLU	0.044415	0
B77	B175	TC	ALB-U	0.00681	2.76E-09
B77	B153	TC	Glu	0.025517	0
B77	B138	TC	PAB	0.042361	0
B77	B130	TC	GLB	0.134853	0

B77	B221	TC	Hei	-0.02776	0
B77	B87	TC	NSE	0.008264	5.40E-13
B77	B187	TC	NSE	0.007111	5.34E-10
B77	B200	TC	OP	0.009802	0
B77	B220	TC	SBP	0.149067	0
B77	B219	TC	OT	0.047469	0
B77	B218	TC	DBP	0.15024	0
B77	B216	TC	BMI	0.096645	0
B77	B215	TC	BMI	0.148328	0
B77	B131	TC	AST	0.070057	0
B77	B172	TC	KB	-0.00873	2.53E-14
B77	B210	TC	Hip	0.085225	0
B77	B209	TC	Smoking	0.081294	0
B77	B90	TC	CYFRA 21-1	0.009656	0
B77	B139	TC	ADA	-0.00856	7.82E-14
B77	B196	TC	TCO2	0.005811	3.91E-07
B77	B185	TC	PT	-0.01289	0
B77	B186	TC	PFC	0.007733	1.47E-11
B77	B173	TC	NTT	0.009907	0
B77	B207	TC	WHR	0.021008	0
B77	B206	TC	Waist	0.157925	0
B77	B205	TC	KDA	0.0145	0
B77	B197	TC	AG	0.012012	0
B77	B204	TC	Drinking	0.072091	0
B77	B80	TC	anti-HP IgG	0.011782	0
B77	B112	TC	FT4	-0.03317	0
B77	B113	TC	FT3	-0.00725	2.42E-10
B77	B211	TC	IOP-R	0.016944	0
B77	B201	TC	e-habits	0.021419	0
B77	B132	TC	Dbil	-0.15695	0
B77	B133	TC	TBIL	-0.05626	0
B77	B91	TC	TP	0.148378	0
B77	B114	TC	TSH TT3 TT4(TT4)	-0.01304	0
B77	B212	TC	IOP-L	0.019196	0
B78	B142	HBcAb	25-(OH)D	-0.0087	3.06E-14
B78	B160	HBcAb	PH	-0.01911	0
B78	B126	HBcAb	Alb	0.015803	0
B78	B127	HBcAb	A-G ratio	0.007068	6.81E-10
B78	B161	HBcAb	LEU	0.006581	9.15E-09
B78	B128	HBcAb	ALT	0.01278	0
B78	B111	HBcAb	TSH	0.010068	0
B78	B189	HBcAb	eGFB	0.008232	6.61E-13
B78	B190	HBcAb	Cre	-0.02051	0
B78	B224	HBcAb	pmh	-0.00563	8.73E-07

B78	B151	HBcAb	Anti-CCP	0.008485	1.28E-13
B78	B110	HBcAb	AHA	0.008394	2.33E-13
B78	B157	HBcAb	IgG	0.020005	0
B78	B195	HBcAb	Na	-0.0063	3.77E-08
B78	B176	HBcAb	Ucr	-0.0088	1.60E-14
B78	B178	HBcAb	Ig-U	-0.0104	0
B78	B191	HBcAb	Urea	0.015086	0
B78	B174	HBcAb	Urea/Cr	0.026904	0
B78	B192	HBcAb	UA	-0.01921	0
B78	B175	HBcAb	ALB-U	-0.00764	2.58E-11
B78	B177	HBcAb	ACR	-0.0089	7.99E-15
B78	B179	HBcAb	α1-MG	-0.01783	0
B78	B180	HBcAb	Tf	-0.0096	0
B78	B221	HBcAb	Hei	-0.02391	0
B78	B220	HBcAb	SBP	-0.01515	0
B78	B216	HBcAb	BMI	-0.00688	1.90E-09
B78	B215	HBcAb	BMI	0.008615	5.44E-14
B78	B131	HBcAb	AST	0.013822	0
B78	B172	HBcAb	KB	0.006066	1.18E-07
B78	B205	HBcAb	KDA	-0.00689	1.84E-09
B78	B79	HBcAb	hbＶ-dna	0.010568	0
B78	B204	HBcAb	Drinking	0.007379	1.17E-10
B78	B203	HBcAb	DH	-0.02271	0
B78	B80	HBcAb	anti-HP IgG	-0.17537	0
B78	B112	HBcAb	FT4	-0.01793	0
B78	B113	HBcAb	FT3	0.00607	1.16E-07
B78	B201	HBcAb	e-habits	-0.03047	0
B78	B81	HBcAb	TSGF	-0.02779	0
B78	B91	HBcAb	TP	0.010079	0
B78	B212	HBcAb	IOP-L	-0.0066	8.51E-09
B79	B137	hbＶ-dna	AFU	0.012384	0
B79	B92	hbＶ-dna	ALDH2-Glu504Glu	0.007985	3.13E-12
B79	B93	hbＶ-dna	ALDH2-Glu504Lys	-0.00701	9.39E-10
B79	B126	hbＶ-dna	Alb	-0.00974	0
B79	B128	hbＶ-dna	ALT	0.032258	0
B79	B165	hbＶ-dna	bilirubin	0.00576	4.94E-07
B79	B134	hbＶ-dna	CHE	-0.00954	0
B79	B189	hbＶ-dna	eGFB	0.005931	2.24E-07
B79	B141	hbＶ-dna	AST/ALT	-0.00694	1.39E-09
B79	B181	hbＶ-dna	APTT	0.007989	3.06E-12
B79	B194	hbＶ-dna	Cl	0.011523	0
B79	B199	hbＶ-dna	Mg	-0.00598	1.82E-07
B79	B157	hbＶ-dna	IgG	0.011982	0
B79	B195	hbＶ-dna	Na	-0.00642	2.08E-08

B79	B138	hb V -dna	PAB	-0.02168	0
B79	B200	hb V -dna	OP	-0.00626	4.65E-08
B79	B218	hb V -dna	DBP	-0.00567	7.33E-07
B79	B131	hb V -dna	AST	0.034175	0
B79	B139	hb V -dna	ADA	0.019321	0
B79	B183	hb V -dna	PTA	0.018016	0
B79	B185	hb V -dna	PT	0.01159	0
B79	B186	hb V -dna	PFC	-0.00573	5.72E-07
B79	B197	hb V -dna	AG	-0.00827	5.03E-13
B79	B81	hb V -dna	TSGF	-0.01305	0
B79	B135	hb V -dna	TBA	0.012257	0
B79	B91	hb V -dna	TP	-0.00588	2.90E-07
B8	B92	WB-Zinc	ALDH2-Glu504Glu	-0.00588	2.82E-07
B8	B93	WB-Zinc	ALDH2-Glu504Lys	0.008628	4.97E-14
B8	B94	WB-Zinc	ALDH2-Lys504Lys	-0.00732	1.65E-10
B8	B126	WB-Zinc	Alb	0.006971	1.15E-09
B8	B121	WB-Zinc	C3	-0.00606	1.22E-07
B8	B122	WB-Zinc	C4	-0.00907	2.22E-15
B8	B134	WB-Zinc	CHE	0.007705	1.73E-11
B8	B72	WB-Zinc	HDL-C	-0.00571	6.11E-07
B8	B189	WB-Zinc	eGFB	-0.00654	1.11E-08
B8	B141	WB-Zinc	AST/ALT	-0.00706	6.97E-10
B8	B45	WB-Zinc	MCHC	0.005951	2.04E-07
B8	B46	WB-Zinc	RBC Count	0.016817	0
B8	B190	WB-Zinc	Cre	0.01131	0
B8	B222	WB-Zinc	NS	0.010093	0
B8	B199	WB-Zinc	Mg	0.006089	1.06E-07
B8	B195	WB-Zinc	Na	0.009673	0
B8	B192	WB-Zinc	UA	0.010571	0
B8	B138	WB-Zinc	PAB	0.013398	0
B8	B60	WB-Zinc	WBRVI	0.007907	5.07E-12
B8	B62	WB-Zinc	WBHSRVI	0.007446	7.99E-11
B8	B64	WB-Zinc	WBRSR(mPas_5)	0.00729	1.96E-10
B8	B65	WB-Zinc	WBRSR(mPas_1)	0.007222	2.88E-10
B8	B66	WB-Zinc	WBRSR(mPas_100)	0.006855	2.17E-09
B8	B67	WB-Zinc	WBRSR(mPas_50)	0.007001	9.80E-10
B8	B221	WB-Zinc	Hei	0.010543	0
B8	B200	WB-Zinc	OP	0.009367	4.44E-16
B8	B216	WB-Zinc	BMI	0.010381	0
B8	B215	WB-Zinc	BMI	0.006501	1.38E-08
B8	B210	WB-Zinc	Hip	0.006671	5.73E-09
B8	B51	WB-Zinc	Hb	0.017016	0
B8	B186	WB-Zinc	PFC	-0.00642	2.10E-08
B8	B206	WB-Zinc	Waist	0.008994	4.00E-15

B8	B197	WB-Zinc	AG	0.00736	1.31E-10
B8	B135	WB-Zinc	TBA	0.008482	1.31E-13
B80	B160	anti-HP IgG	PH	-0.01611	0
B80	B140	anti-HP IgG	r-GT	0.006878	1.92E-09
B80	B126	anti-HP IgG	Alb	-0.03284	0
B80	B127	anti-HP IgG	A-G ratio	-0.02156	0
B80	B161	anti-HP IgG	LEU	-0.00897	4.88E-15
B80	B166	anti-HP IgG	Pro	-0.02176	0
B80	B189	anti-HP IgG	eGFB	-0.02562	0
B80	B141	anti-HP IgG	AST/ALT	-0.00749	6.24E-11
B80	B190	anti-HP IgG	Cre	0.01416	0
B80	B129	anti-HP IgG	IBIL	-0.01256	0
B80	B222	anti-HP IgG	NS	0.016048	0
B80	B174	anti-HP IgG	Urea/Cr	-0.01101	0
B80	B192	anti-HP IgG	UA	0.006675	5.63E-09
B80	B130	anti-HP IgG	GLB	0.01159	0
B80	B221	anti-HP IgG	Hei	0.010826	0
B80	B220	anti-HP IgG	SBP	0.006017	1.50E-07
B80	B216	anti-HP IgG	BMI	0.018739	0
B80	B215	anti-HP IgG	BMI	0.016486	0
B80	B172	anti-HP IgG	KB	-0.00712	5.06E-10
B80	B210	anti-HP IgG	Hip	0.016135	0
B80	B209	anti-HP IgG	Smoking	0.012143	0
B80	B206	anti-HP IgG	Waist	0.020605	0
B80	B203	anti-HP IgG	DH	0.010744	0
B80	B201	anti-HP IgG	e-habits	0.010701	0
B80	B132	anti-HP IgG	Dbil	-0.01541	0
B80	B133	anti-HP IgG	TBIL	-0.01419	0
B80	B91	anti-HP IgG	TP	-0.01049	0
B81	B137	TSGF	AFU	0.026353	0
B81	B92	TSGF	ALDH2-Glu504Glu	-0.01276	0
B81	B93	TSGF	ALDH2-Glu504Lys	0.013373	0
B81	B160	TSGF	PH	0.012419	0
B81	B126	TSGF	Alb	-0.01495	0
B81	B127	TSGF	A-G ratio	-0.02943	0
B81	B124	TSGF	ACTH	-0.04503	0
B81	B134	TSGF	CHE	0.015693	0
B81	B189	TSGF	eGFB	0.00984	0
B81	B141	TSGF	AST/ALT	-0.02069	0
B81	B181	TSGF	APTT	0.025896	0
B81	B129	TSGF	IBIL	-0.01592	0
B81	B150	TSGF	ALP	0.013103	0
B81	B100	TSGF	anti-Ro52	0.007374	1.21E-10
B81	B104	TSGF	anti-SSB	0.010724	0

B81	B151	TSGF	Anti-CCP	-0.02794	0
B81	B108	TSGF	Anti-Cyclin	-0.00727	2.20E-10
B81	B198	TSGF	P	-0.0077	1.74E-11
B81	B194	TSGF	Cl	-0.01902	0
B81	B199	TSGF	Mg	0.012926	0
B81	B155	TSGF	IgA	0.007658	2.29E-11
B81	B156	TSGF	IgE	-0.01537	0
B81	B157	TSGF	IgG	-0.07881	0
B81	B158	TSGF	IgM	-0.00606	1.22E-07
B81	B195	TSGF	Na	0.059431	0
B81	B178	TSGF	Ig-U	-0.00587	3.00E-07
B81	B192	TSGF	UA	0.010552	0
B81	B179	TSGF	α1-MG	-0.00759	3.44E-11
B81	B180	TSGF	Tf	-0.00653	1.18E-08
B81	B182	TSGF	TT	0.130388	0
B81	B125	TSGF	Cor	-0.11148	0
B81	B138	TSGF	PAB	0.012995	0
B81	B130	TSGF	GLB	0.026816	0
B81	B187	TSGF	NSE	0.005641	8.45E-07
B81	B200	TSGF	OP	0.04553	0
B81	B220	TSGF	SBP	0.006292	3.95E-08
B81	B218	TSGF	DBP	-0.00593	2.28E-07
B81	B131	TSGF	AST	-0.00662	7.43E-09
B81	B90	TSGF	CYFRA 21-1	0.017851	0
B81	B139	TSGF	ADA	0.026465	0
B81	B196	TSGF	TCO2	-0.01596	0
B81	B183	TSGF	PTA	-0.06212	0
B81	B184	TSGF	INR	-0.00743	8.79E-11
B81	B185	TSGF	PT	-0.01859	0
B81	B186	TSGF	PFC	-0.00648	1.56E-08
B81	B197	TSGF	AG	0.059765	0
B81	B112	TSGF	FT4	-0.01918	0
B81	B113	TSGF	FT3	0.006417	2.12E-08
B81	B132	TSGF	Dbil	0.010915	0
B81	B133	TSGF	TBIL	-0.0099	0
B81	B135	TSGF	TBA	-0.00639	2.45E-08
B81	B91	TSGF	TP	0.013661	0
B81	B212	TSGF	IOP-L	0.006088	1.07E-07
B82	B189	CEA	eGFB	-0.00762	2.94E-11
B82	B83	CEA	AFP	0.007914	4.87E-12
B82	B84	CEA	C-PSA	0.014847	0
B82	B85	CEA	SCC	0.03199	0
B82	B86	CEA	TBhCG	0.008162	1.04E-12
B82	B87	CEA	NSE	0.016547	0

B82	B88	CEA	CA 242	0.030864	0
B82	B89	CEA	CA 72-4	0.043844	0
B82	B209	CEA	Smoking	0.008022	2.50E-12
B82	B90	CEA	CYFRA 21-1	0.014698	0
B83	B137	AFP	AFU	0.027567	0
B83	B140	AFP	r-GT	0.006417	2.12E-08
B83	B126	AFP	Alb	-0.00826	5.68E-13
B83	B193	AFP	K	0.008847	1.13E-14
B83	B150	AFP	ALP	0.011178	0
B83	B84	AFP	C-PSA	0.006031	1.40E-07
B83	B85	AFP	SCC	0.006899	1.71E-09
B83	B194	AFP	Cl	-0.00922	8.88E-16
B83	B155	AFP	IgA	-0.00597	1.91E-07
B83	B138	AFP	PAB	-0.00863	4.75E-14
B83	B86	AFP	TBhCG	0.184804	0
B83	B88	AFP	CA 242	0.039676	0
B83	B89	AFP	CA 72-4	0.017582	0
B83	B131	AFP	AST	0.007121	5.05E-10
B83	B90	AFP	CYFRA 21-1	0.023591	0
B83	B139	AFP	ADA	0.013703	0
B83	B114	AFP	TSH TT3 TT4(TT4)	0.005627	8.99E-07
B84	B137	C-PSA	AFU	0.010664	0
B84	B140	C-PSA	r-GT	0.011086	0
B84	B128	C-PSA	ALT	0.00689	1.79E-09
B84	B134	C-PSA	CHE	0.006295	3.88E-08
B84	B166	C-PSA	Pro	0.008197	8.28E-13
B84	B189	C-PSA	eGFB	-0.03205	0
B84	B141	C-PSA	AST/ALT	-0.00721	3.03E-10
B84	B146	C-PSA	CysC	0.008725	2.58E-14
B84	B190	C-PSA	Cre	0.034592	0
B84	B129	C-PSA	IBIL	0.008308	4.08E-13
B84	B222	C-PSA	NS	0.029918	0
B84	B198	C-PSA	P	-0.01734	0
B84	B85	C-PSA	SCC	0.060655	0
B84	B157	C-PSA	IgG	-0.00566	7.82E-07
B84	B195	C-PSA	Na	0.009848	0
B84	B191	C-PSA	Urea	0.013341	0
B84	B174	C-PSA	Urea/Cr	-0.01415	0
B84	B192	C-PSA	UA	0.02494	0
B84	B138	C-PSA	PAB	0.013905	0
B84	B130	C-PSA	GLB	-0.00567	7.57E-07
B84	B86	C-PSA	TBhCG	-0.01028	0
B84	B221	C-PSA	Hei	0.021118	0
B84	B87	C-PSA	NSE	0.065083	0

B84	B200	C-PSA	OP	0.012017	0
B84	B220	C-PSA	SBP	0.022236	0
B84	B218	C-PSA	DBP	0.017188	0
B84	B88	C-PSA	CA 242	0.006693	5.11E-09
B84	B89	C-PSA	CA 72-4	0.028595	0
B84	B216	C-PSA	BMI	0.020573	0
B84	B215	C-PSA	BMI	0.013133	0
B84	B131	C-PSA	AST	0.00696	1.23E-09
B84	B210	C-PSA	Hip	0.009997	0
B84	B209	C-PSA	Smoking	0.019676	0
B84	B90	C-PSA	CYFRA 21-1	0.093019	0
B84	B206	C-PSA	Waist	0.023414	0
B84	B204	C-PSA	Drinking	0.018548	0
B84	B113	C-PSA	FT3	0.006091	1.05E-07
B84	B133	C-PSA	TBIL	0.007819	8.69E-12
B84	B91	C-PSA	TP	-0.00635	2.91E-08
B85	B166	SCC	Pro	0.006194	6.40E-08
B85	B189	SCC	eGFB	-0.0163	0
B85	B146	SCC	CysC	0.00645	1.79E-08
B85	B190	SCC	Cre	0.028664	0
B85	B222	SCC	NS	0.010874	0
B85	B156	SCC	IgE	0.008599	6.00E-14
B85	B195	SCC	Na	0.008672	3.71E-14
B85	B191	SCC	Urea	0.007632	2.68E-11
B85	B174	SCC	Urea/Cr	-0.01351	0
B85	B192	SCC	UA	0.016167	0
B85	B138	SCC	PAB	0.007245	2.53E-10
B85	B130	SCC	GLB	-0.00707	6.68E-10
B85	B86	SCC	TBhCG	-0.01726	0
B85	B221	SCC	Hei	0.014669	0
B85	B87	SCC	NSE	0.162894	0
B85	B200	SCC	OP	0.008372	2.68E-13
B85	B88	SCC	CA 242	0.050806	0
B85	B216	SCC	BMI	0.008434	1.79E-13
B85	B209	SCC	Smoking	0.010487	0
B85	B90	SCC	CYFRA 21-1	0.066678	0
B85	B197	SCC	AG	0.006871	1.99E-09
B85	B204	SCC	Drinking	0.007156	4.18E-10
B85	B91	SCC	TP	-0.00808	1.70E-12
B86	B137	TBhCG	AFU	0.009367	4.44E-16
B86	B160	TBhCG	PH	0.006558	1.03E-08
B86	B126	TBhCG	Alb	-0.01536	0
B86	B127	TBhCG	A-G ratio	-0.00644	1.84E-08
B86	B134	TBhCG	CHE	-0.00886	9.99E-15

B86	B189	TBhCG	eGFB	0.015692	0
B86	B190	TBhCG	Cre	-0.01335	0
B86	B129	TBhCG	IBIL	-0.00715	4.20E-10
B86	B191	TBhCG	Urea	-0.01211	0
B86	B192	TBhCG	UA	-0.0123	0
B86	B138	TBhCG	PAB	-0.00669	5.20E-09
B86	B87	TBhCG	NSE	-0.01372	0
B86	B200	TBhCG	OP	-0.00651	1.33E-08
B86	B220	TBhCG	SBP	-0.00655	1.09E-08
B86	B218	TBhCG	DBP	-0.00853	9.81E-14
B86	B88	TBhCG	CA 242	0.008172	9.69E-13
B86	B89	TBhCG	CA 72-4	0.025218	0
B86	B186	TBhCG	PFC	0.006211	5.86E-08
B86	B204	TBhCG	Drinking	-0.00567	7.32E-07
B86	B133	TBhCG	TBIL	-0.00702	8.87E-10
B86	B91	TBhCG	TP	-0.00892	6.66E-15
B86	B114	TBhCG	TSH TT3 TT4(TT4)	0.013783	0
B87	B137	NSE	AFU	0.013029	0
B87	B160	NSE	PH	0.011408	0
B87	B140	NSE	r-GT	0.007016	9.06E-10
B87	B128	NSE	ALT	0.005931	2.24E-07
B87	B134	NSE	CHE	0.01025	0
B87	B189	NSE	eGFB	-0.01279	0
B87	B190	NSE	Cre	0.013376	0
B87	B129	NSE	IBIL	0.028079	0
B87	B150	NSE	ALP	-0.00695	1.27E-09
B87	B222	NSE	NS	0.009292	4.44E-16
B87	B154	NSE	RF	0.005946	2.09E-07
B87	B198	NSE	P	0.005917	2.40E-07
B87	B195	NSE	Na	-0.01278	0
B87	B191	NSE	Urea	0.006419	2.09E-08
B87	B192	NSE	UA	0.008832	1.24E-14
B87	B182	NSE	TT	-0.00925	6.66E-16
B87	B138	NSE	PAB	0.00575	5.16E-07
B87	B200	NSE	OP	-0.00998	0
B87	B220	NSE	SBP	0.018983	0
B87	B218	NSE	DBP	0.013515	0
B87	B88	NSE	CA 242	0.105575	0
B87	B216	NSE	BMI	0.010012	0
B87	B215	NSE	BMI	0.010097	0
B87	B131	NSE	AST	0.01198	0
B87	B209	NSE	Smoking	0.005612	9.59E-07
B87	B90	NSE	CYFRA 21-1	0.09353	0
B87	B183	NSE	PTA	0.015408	0

B87	B206	NSE	Waist	0.009991	0
B87	B197	NSE	AG	-0.01326	0
B87	B204	NSE	Drinking	0.005972	1.85E-07
B87	B112	NSE	FT4	0.021714	0
B87	B211	NSE	IOP-R	0.007611	3.03E-11
B87	B133	NSE	TBIL	0.022522	0
B87	B114	NSE	TSH TT3 TT4(TT4)	-0.00575	5.17E-07
B87	B212	NSE	IOP-L	0.007074	6.58E-10
B88	B140	CA 242	r-GT	-0.01064	0
B88	B126	CA 242	Alb	-0.00712	4.98E-10
B88	B127	CA 242	A-G ratio	-0.01104	0
B88	B161	CA 242	LEU	0.00819	8.63E-13
B88	B128	CA 242	ALT	-0.01301	0
B88	B189	CA 242	eGFB	0.008233	6.59E-13
B88	B141	CA 242	AST/ALT	0.017977	0
B88	B190	CA 242	Cre	-0.02436	0
B88	B129	CA 242	IBIL	-0.00592	2.33E-07
B88	B150	CA 242	ALP	-0.00745	7.91E-11
B88	B222	CA 242	NS	-0.02957	0
B88	B198	CA 242	P	0.006346	3.02E-08
B88	B156	CA 242	IgE	-0.00601	1.54E-07
B88	B191	CA 242	Urea	-0.00792	4.64E-12
B88	B174	CA 242	Urea/Cr	0.01112	0
B88	B192	CA 242	UA	-0.0272	0
B88	B170	CA 242	U-GLU	0.007355	1.35E-10
B88	B138	CA 242	PAB	-0.01203	0
B88	B130	CA 242	GLB	0.010771	0
B88	B221	CA 242	Hei	-0.02717	0
B88	B218	CA 242	DBP	-0.00692	1.55E-09
B88	B89	CA 242	CA 72-4	-0.02408	0
B88	B216	CA 242	BMI	-0.0292	0
B88	B215	CA 242	BMI	-0.01995	0
B88	B210	CA 242	Hip	-0.02021	0
B88	B209	CA 242	Smoking	-0.01302	0
B88	B90	CA 242	CYFRA 21-1	0.028851	0
B88	B139	CA 242	ADA	0.006077	1.12E-07
B88	B206	CA 242	Waist	-0.02614	0
B88	B204	CA 242	Drinking	-0.01754	0
B88	B113	CA 242	FT3	-0.00704	7.80E-10
B89	B129	CA 72-4	IBIL	-0.01618	0
B89	B221	CA 72-4	Hei	-0.00569	6.89E-07
B89	B215	CA 72-4	BMI	0.00846	1.51E-13
B89	B210	CA 72-4	Hip	0.005749	5.18E-07
B89	B90	CA 72-4	CYFRA 21-1	0.023236	0

B89	B196	CA 72-4	TCO2	-0.00562	9.23E-07
B89	B183	CA 72-4	PTA	-0.00955	0
B89	B206	CA 72-4	Waist	0.007569	3.88E-11
B89	B112	CA 72-4	FT4	-0.00651	1.30E-08
B89	B132	CA 72-4	Dbil	-0.00967	0
B89	B133	CA 72-4	TBIL	-0.01553	0
B89	B135	CA 72-4	TBA	0.005729	5.69E-07
B9	B17	RH	CD3+CD16+CD56+NK	-0.00578	4.43E-07
B9	B116	RH	TT3	0.018573	0
B9	B46	RH	RBC Count	-0.00625	4.75E-08
B9	B102	RH	anti-Sm	0.007495	6.02E-11
B9	B152	RH	ACA-Ig(G-A-M)	0.007909	5.02E-12
B9	B51	RH	Hb	-0.00806	1.91E-12
B9	B69	RH	PV	0.009731	0
B9	B203	RH	DH	-0.00761	3.03E-11
B9	B81	RH	TSGF	-0.01173	0
B90	B137	CYFRA 21-1	AFU	0.012962	0
B90	B92	CYFRA 21-1	ALDH2-Glu504Glu	-0.00725	2.39E-10
B90	B160	CYFRA 21-1	PH	-0.01224	0
B90	B140	CYFRA 21-1	r-GT	0.006955	1.27E-09
B90	B94	CYFRA 21-1	ALDH2-Lys504Lys	0.01121	0
B90	B126	CYFRA 21-1	Alb	-0.02435	0
B90	B127	CYFRA 21-1	A-G ratio	-0.00815	1.09E-12
B90	B128	CYFRA 21-1	ALT	0.00655	1.08E-08
B90	B111	CYFRA 21-1	TSH	0.007458	7.43E-11
B90	B166	CYFRA 21-1	Pro	0.010981	0
B90	B189	CYFRA 21-1	eGFB	-0.05998	0
B90	B146	CYFRA 21-1	CysC	0.019842	0
B90	B181	CYFRA 21-1	APTT	-0.01474	0
B90	B190	CYFRA 21-1	Cre	0.043186	0
B90	B150	CYFRA 21-1	ALP	0.012525	0
B90	B222	CYFRA 21-1	NS	0.020133	0
B90	B198	CYFRA 21-1	P	0.005995	1.66E-07
B90	B194	CYFRA 21-1	Cl	-0.00749	6.36E-11
B90	B199	CYFRA 21-1	Mg	0.006694	5.09E-09
B90	B195	CYFRA 21-1	Na	0.021619	0
B90	B191	CYFRA 21-1	Urea	0.027488	0
B90	B174	CYFRA 21-1	Urea/Cr	-0.00672	4.43E-09
B90	B192	CYFRA 21-1	UA	0.022808	0
B90	B170	CYFRA 21-1	U-GLU	0.012828	0
B90	B182	CYFRA 21-1	TT	0.02864	0
B90	B138	CYFRA 21-1	PAB	0.005876	2.89E-07
B90	B221	CYFRA 21-1	Hei	0.011906	0
B90	B187	CYFRA 21-1	NSE	0.01929	0

B90	B200	CYFRA 21-1	OP	0.024833	0
B90	B220	CYFRA 21-1	SBP	0.032423	0
B90	B219	CYFRA 21-1	OT	0.011971	0
B90	B218	CYFRA 21-1	DBP	0.020761	0
B90	B216	CYFRA 21-1	BMI	0.008105	1.48E-12
B90	B131	CYFRA 21-1	AST	0.01325	0
B90	B209	CYFRA 21-1	Smoking	0.029513	0
B90	B139	CYFRA 21-1	ADA	0.024387	0
B90	B196	CYFRA 21-1	TCO2	0.009371	4.44E-16
B90	B183	CYFRA 21-1	PTA	-0.04368	0
B90	B185	CYFRA 21-1	PT	-0.01068	0
B90	B186	CYFRA 21-1	PFC	0.010505	0
B90	B206	CYFRA 21-1	Waist	0.019578	0
B90	B197	CYFRA 21-1	AG	0.010117	0
B90	B204	CYFRA 21-1	Drinking	0.007612	3.02E-11
B90	B113	CYFRA 21-1	FT3	0.005984	1.75E-07
B90	B201	CYFRA 21-1	e-habits	0.006201	6.16E-08
B90	B135	CYFRA 21-1	TBA	0.018988	0
B90	B91	CYFRA 21-1	TP	-0.01241	0
B91	B137	TP	AFU	0.014859	0
B91	B160	TP	PH	-0.05393	0
B91	B140	TP	r-GT	0.076129	0
B91	B143	TP	β-CTx	-0.00565	7.96E-07
B91	B118	TP	CEA1	-0.00818	9.19E-13
B91	B126	TP	Alb	0.529233	0
B91	B127	TP	A-G ratio	-0.43892	0
B91	B161	TP	LEU	0.010063	0
B91	B128	TP	ALT	0.097752	0
B91	B120	TP	HCV-cAg	0.006235	5.23E-08
B91	B121	TP	C3	0.008095	1.58E-12
B91	B122	TP	C4	0.006302	3.76E-08
B91	B225	TP	hPBG	0.010314	0
B91	B123	TP	hs-CRP1	0.008879	9.10E-15
B91	B111	TP	TSH	0.032563	0
B91	B134	TP	CHE	0.055407	0
B91	B166	TP	Pro	0.016826	0
B91	B188	TP	Ca	0.092438	0
B91	B189	TP	eGFB	0.095952	0
B91	B141	TP	AST/ALT	-0.0492	0
B91	B146	TP	CysC	0.006793	3.01E-09
B91	B193	TP	K	0.029375	0
B91	B150	TP	ALP	0.035282	0
B91	B222	TP	NS	0.0246	0
B91	B100	TP	anti-Ro52	0.006262	4.57E-08

B91	B103	TP	anti-SSA/Ro	0.008406	2.16E-13
B91	B107	TP	AnuA	0.00588	2.84E-07
B91	B148	TP	Anti_TPO	0.020093	0
B91	B149	TP	TGAb	0.020108	0
B91	B198	TP	P	0.008966	4.88E-15
B91	B194	TP	Cl	-0.06391	0
B91	B199	TP	Mg	0.020097	0
B91	B155	TP	IgA	0.058089	0
B91	B156	TP	IgE	0.007408	9.97E-11
B91	B157	TP	IgG	0.089226	0
B91	B158	TP	IgM	0.015142	0
B91	B195	TP	Na	-0.01168	0
B91	B178	TP	Ig-U	0.00743	8.78E-11
B91	B192	TP	UA	0.124138	0
B91	B170	TP	U-GLU	0.015279	0
B91	B182	TP	TT	0.00715	4.31E-10
B91	B153	TP	Glu	0.015733	0
B91	B138	TP	PAB	0.035649	0
B91	B130	TP	GLB	0.786316	0
B91	B221	TP	Hei	0.027403	0
B91	B187	TP	NSE	0.010213	0
B91	B220	TP	SBP	0.088378	0
B91	B219	TP	OT	-0.01985	0
B91	B218	TP	DBP	0.11309	0
B91	B216	TP	BMI	0.033451	0
B91	B215	TP	BMI	0.022208	0
B91	B131	TP	AST	0.083612	0
B91	B172	TP	KB	0.013317	0
B91	B210	TP	Hip	0.032208	0
B91	B209	TP	Smoking	-0.12697	0
B91	B139	TP	ADA	0.037977	0
B91	B152	TP	ACA-Ig(G-A-M)	0.009007	3.77E-15
B91	B183	TP	PTA	-0.00755	4.37E-11
B91	B186	TP	PFC	0.008122	1.33E-12
B91	B206	TP	Waist	0.007401	1.03E-10
B91	B197	TP	AG	0.035331	0
B91	B204	TP	Drinking	-0.03181	0
B91	B203	TP	DH	0.03649	0
B91	B112	TP	FT4	0.036694	0
B91	B113	TP	FT3	0.036929	0
B91	B211	TP	IOP-R	0.026051	0
B91	B132	TP	Dbil	-0.01917	0
B91	B114	TP	TSH TT3 TT4(TT4)	0.024084	0
B91	B115	TP	TSH TT3 TT4(TT3)	0.014753	0

B91	B144	TP	Total-PINP	-0.00772	1.61E-11
B91	B212	TP	IOP-L	0.020379	0
B92	B137	ALDH2-Glu504Glu	AFU	-0.00828	4.83E-13
B92	B93	ALDH2-Glu504Glu	ALDH2-Glu504Lys	-0.93834	0
B92	B140	ALDH2-Glu504Glu	r-GT	0.011946	0
B92	B94	ALDH2-Glu504Glu	ALDH2-Lys504Lys	-0.2403	0
B92	B128	ALDH2-Glu504Glu	ALT	0.006264	4.54E-08
B92	B121	ALDH2-Glu504Glu	C3	0.006753	3.73E-09
B92	B122	ALDH2-Glu504Glu	C4	-0.01556	0
B92	B225	ALDH2-Glu504Glu	hPBG	0.007462	7.27E-11
B92	B134	ALDH2-Glu504Glu	CHE	-0.01334	0
B92	B181	ALDH2-Glu504Glu	APTT	-0.00984	0
B92	B150	ALDH2-Glu504Glu	ALP	-0.00743	8.78E-11
B92	B95	ALDH2-Glu504Glu	anti-cenpb	-0.00631	3.52E-08
B92	B97	ALDH2-Glu504Glu	dsDNA	-0.00819	8.69E-13
B92	B98	ALDH2-Glu504Glu	anti-Jo-1	0.01036	0
B92	B103	ALDH2-Glu504Glu	anti-SSA/Ro	0.018127	0
B92	B104	ALDH2-Glu504Glu	anti-SSB	0.008987	4.44E-15
B92	B106	ALDH2-Glu504Glu	anti-P	0.007281	2.06E-10
B92	B199	ALDH2-Glu504Glu	Mg	-0.00648	1.54E-08
B92	B155	ALDH2-Glu504Glu	IgA	0.0118	0
B92	B156	ALDH2-Glu504Glu	IgE	-0.00604	1.34E-07
B92	B157	ALDH2-Glu504Glu	IgG	0.009776	0
B92	B158	ALDH2-Glu504Glu	IgM	0.010695	0
B92	B195	ALDH2-Glu504Glu	Na	-0.01545	0
B92	B176	ALDH2-Glu504Glu	Ucr	-0.0059	2.54E-07
B92	B178	ALDH2-Glu504Glu	Ig-U	-0.00966	0
B92	B180	ALDH2-Glu504Glu	Tf	-0.01261	0
B92	B182	ALDH2-Glu504Glu	TT	-0.00809	1.67E-12
B92	B125	ALDH2-Glu504Glu	Cor	-0.00908	2.22E-15
B92	B138	ALDH2-Glu504Glu	PAB	0.017929	0
B92	B200	ALDH2-Glu504Glu	OP	-0.01385	0
B92	B131	ALDH2-Glu504Glu	AST	0.006705	4.80E-09
B92	B139	ALDH2-Glu504Glu	ADA	0.011189	0
B92	B183	ALDH2-Glu504Glu	PTA	0.020885	0
B92	B186	ALDH2-Glu504Glu	PFC	-0.03249	0
B92	B204	ALDH2-Glu504Glu	Drinking	0.020353	0
B92	B112	ALDH2-Glu504Glu	FT4	-0.00756	4.02E-11
B92	B114	ALDH2-Glu504Glu	TSH TT3 TT4(TT4)	-0.01562	0
B93	B137	ALDH2-Glu504Lys	AFU	0.009021	3.55E-15
B93	B140	ALDH2-Glu504Lys	r-GT	-0.01092	0
B93	B94	ALDH2-Glu504Lys	ALDH2-Lys504Lys	-0.10688	0
B93	B128	ALDH2-Glu504Lys	ALT	-0.00583	3.51E-07
B93	B121	ALDH2-Glu504Lys	C3	-0.006	1.60E-07

B93	B122	ALDH2-Glu504Lys	C4	0.013816	0
B93	B225	ALDH2-Glu504Lys	hPBG	-0.00647	1.63E-08
B93	B124	ALDH2-Glu504Lys	ACTH	-0.00621	5.98E-08
B93	B134	ALDH2-Glu504Lys	CHE	0.011947	0
B93	B181	ALDH2-Glu504Lys	APTT	0.010921	0
B93	B150	ALDH2-Glu504Lys	ALP	0.006742	3.95E-09
B93	B95	ALDH2-Glu504Lys	anti-cenpb	0.008522	1.00E-13
B93	B97	ALDH2-Glu504Lys	dsDNA	0.010535	0
B93	B98	ALDH2-Glu504Lys	anti-Jo-1	-0.00922	8.88E-16
B93	B103	ALDH2-Glu504Lys	anti-SSA/Ro	-0.01546	0
B93	B104	ALDH2-Glu504Lys	anti-SSB	-0.00807	1.90E-12
B93	B106	ALDH2-Glu504Lys	anti-P	-0.00664	6.80E-09
B93	B199	ALDH2-Glu504Lys	Mg	0.006322	3.39E-08
B93	B155	ALDH2-Glu504Lys	IgA	-0.01043	0
B93	B157	ALDH2-Glu504Lys	IgG	-0.01046	0
B93	B158	ALDH2-Glu504Lys	IgM	-0.00976	0
B93	B195	ALDH2-Glu504Lys	Na	0.016295	0
B93	B176	ALDH2-Glu504Lys	Ucr	0.006575	9.44E-09
B93	B178	ALDH2-Glu504Lys	Ig-U	0.011195	0
B93	B180	ALDH2-Glu504Lys	Tf	0.014059	0
B93	B182	ALDH2-Glu504Lys	TT	0.005969	1.87E-07
B93	B125	ALDH2-Glu504Lys	Cor	0.007738	1.42E-11
B93	B138	ALDH2-Glu504Lys	PAB	-0.0157	0
B93	B200	ALDH2-Glu504Lys	OP	0.01502	0
B93	B131	ALDH2-Glu504Lys	AST	-0.00597	1.85E-07
B93	B139	ALDH2-Glu504Lys	ADA	-0.00956	0
B93	B196	ALDH2-Glu504Lys	TCO2	0.006468	1.63E-08
B93	B183	ALDH2-Glu504Lys	PTA	-0.0172	0
B93	B186	ALDH2-Glu504Lys	PFC	0.027449	0
B93	B204	ALDH2-Glu504Lys	Drinking	-0.01737	0
B93	B112	ALDH2-Glu504Lys	FT4	0.007532	4.85E-11
B93	B114	ALDH2-Glu504Lys	TSH TT3 TT4(TT4)	0.015977	0
B94	B122	ALDH2-Lys504Lys	C4	0.005923	2.33E-07
B94	B124	ALDH2-Lys504Lys	ACTH	0.016307	0
B94	B105	ALDH2-Lys504Lys	anti-U1RNP	-0.00739	1.09E-10
B94	B95	ALDH2-Lys504Lys	anti-cenpb	-0.00577	4.73E-07
B94	B97	ALDH2-Lys504Lys	dsDNA	-0.00605	1.26E-07
B94	B100	ALDH2-Lys504Lys	anti-Ro52	-0.00721	3.06E-10
B94	B102	ALDH2-Lys504Lys	anti-Sm	-0.00578	4.44E-07
B94	B103	ALDH2-Lys504Lys	anti-SSA/Ro	-0.00858	6.88E-14
B94	B108	ALDH2-Lys504Lys	Anti-Cyclin	-0.00588	2.85E-07
B94	B109	ALDH2-Lys504Lys	AMA-M2	-0.0058	4.06E-07
B94	B110	ALDH2-Lys504Lys	AHA	0.016206	0
B94	B198	ALDH2-Lys504Lys	P	0.007055	7.29E-10

B94	B194	ALDH2-Lys504Lys	Cl	0.011486	0
B94	B156	ALDH2-Lys504Lys	IgE	0.018842	0
B94	B179	ALDH2-Lys504Lys	α1-MG	-0.01165	0
B94	B182	ALDH2-Lys504Lys	TT	0.006328	3.30E-08
B94	B138	ALDH2-Lys504Lys	PAB	-0.00628	4.22E-08
B94	B187	ALDH2-Lys504Lys	NSE	0.008087	1.66E-12
B94	B139	ALDH2-Lys504Lys	ADA	-0.00587	3.00E-07
B94	B196	ALDH2-Lys504Lys	TCO2	-0.00723	2.79E-10
B94	B183	ALDH2-Lys504Lys	PTA	-0.01172	0
B94	B185	ALDH2-Lys504Lys	PT	0.006959	1.24E-09
B94	B186	ALDH2-Lys504Lys	PFC	0.015994	0
B94	B204	ALDH2-Lys504Lys	Drinking	-0.00974	0
B95	B193	anti-cenpb	K	0.008497	1.19E-13
B95	B99	anti-cenpb	anti-PM-Scl	0.012844	0
B95	B97	anti-cenpb	dsDNA	0.012748	0
B95	B98	anti-cenpb	anti-Jo-1	-0.00599	1.69E-07
B95	B100	anti-cenpb	anti-Ro52	0.039815	0
B95	B101	anti-cenpb	anti-Sc70	-0.0059	2.57E-07
B95	B103	anti-cenpb	anti-SSA/Ro	0.027409	0
B95	B104	anti-cenpb	anti-SSB	0.017915	0
B95	B106	anti-cenpb	anti-P	-0.00639	2.43E-08
B95	B151	anti-cenpb	Anti-CCP	-0.00625	4.91E-08
B95	B109	anti-cenpb	AMA-M2	0.079647	0
B95	B199	anti-cenpb	Mg	-0.00701	9.37E-10
B95	B157	anti-cenpb	IgG	0.013932	0
B95	B195	anti-cenpb	Na	0.006494	1.43E-08
B95	B125	anti-cenpb	Cor	-0.01914	0
B95	B138	anti-cenpb	PAB	-0.01204	0
B95	B200	anti-cenpb	OP	0.006341	3.09E-08
B95	B139	anti-cenpb	ADA	0.010962	0
B96	B127	dsDNA (IIF)	A-G ratio	-0.00618	6.96E-08
B96	B105	dsDNA (IIF)	anti-U1RNP	0.086568	0
B96	B97	dsDNA (IIF)	dsDNA	0.152215	0
B96	B100	dsDNA (IIF)	anti-Ro52	0.103872	0
B96	B102	dsDNA (IIF)	anti-Sm	-0.00693	1.43E-09
B96	B103	dsDNA (IIF)	anti-SSA/Ro	0.097177	0
B96	B104	dsDNA (IIF)	anti-SSB	0.174663	0
B96	B106	dsDNA (IIF)	anti-P	0.121867	0
B96	B107	dsDNA (IIF)	AnuA	0.602683	0
B96	B151	dsDNA (IIF)	Anti-CCP	0.009058	2.66E-15
B96	B109	dsDNA (IIF)	AMA-M2	0.110505	0
B96	B110	dsDNA (IIF)	AHA	0.068124	0
B96	B155	dsDNA (IIF)	IgA	0.006931	1.44E-09
B96	B156	dsDNA (IIF)	IgE	0.00693	1.44E-09

B96	B157	dsDNA (IIF)	IgG	0.073274	0
B96	B179	dsDNA (IIF)	α1-MG	0.008467	1.44E-13
B96	B130	dsDNA (IIF)	GLB	0.009769	0
B96	B152	dsDNA (IIF)	ACA-Ig(G-A-M)	-0.01188	0
B96	B183	dsDNA (IIF)	PTA	0.005672	7.36E-07
B97	B121	dsDNA	C3	-0.01542	0
B97	B122	dsDNA	C4	-0.01784	0
B97	B105	dsDNA	anti-U1RNP	0.065061	0
B97	B100	dsDNA	anti-Ro52	0.030207	0
B97	B102	dsDNA	anti-Sm	0.048145	0
B97	B103	dsDNA	anti-SSA/Ro	0.060723	0
B97	B104	dsDNA	anti-SSB	0.04287	0
B97	B106	dsDNA	anti-P	0.050724	0
B97	B107	dsDNA	AnuA	0.177627	0
B97	B108	dsDNA	Anti-Cyclin	-0.00691	1.57E-09
B97	B109	dsDNA	AMA-M2	0.038138	0
B97	B110	dsDNA	AHA	0.0414	0
B97	B199	dsDNA	Mg	0.01165	0
B97	B155	dsDNA	IgA	0.006467	1.65E-08
B97	B157	dsDNA	IgG	0.016796	0
B97	B195	dsDNA	Na	0.010331	0
B97	B179	dsDNA	α1-MG	-0.00649	1.48E-08
B97	B182	dsDNA	TT	0.007031	8.34E-10
B97	B125	dsDNA	Cor	-0.00643	1.95E-08
B97	B197	dsDNA	AG	0.007758	1.26E-11
B98	B122	anti-Jo-1	C4	0.028752	0
B98	B124	anti-Jo-1	ACTH	0.007464	7.18E-11
B98	B100	anti-Jo-1	anti-Ro52	-0.00585	3.27E-07
B98	B103	anti-Jo-1	anti-SSA/Ro	0.048558	0
B98	B109	anti-Jo-1	AMA-M2	-0.00779	1.01E-11
B98	B110	anti-Jo-1	AHA	-0.00731	1.72E-10
B98	B194	anti-Jo-1	Cl	-0.00602	1.48E-07
B98	B155	anti-Jo-1	IgA	0.008218	7.26E-13
B98	B156	anti-Jo-1	IgE	-0.00771	1.72E-11
B98	B157	anti-Jo-1	IgG	0.014698	0
B98	B139	anti-Jo-1	ADA	0.007483	6.44E-11
B98	B186	anti-Jo-1	PFC	-0.01013	0
B99	B137	anti-PM-Scl	AFU	-0.00962	0
B99	B124	anti-PM-Scl	ACTH	0.015796	0
B99	B150	anti-PM-Scl	ALP	0.006979	1.11E-09
B99	B100	anti-PM-Scl	anti-Ro52	0.045148	0
B99	B103	anti-PM-Scl	anti-SSA/Ro	0.011285	0
B99	B104	anti-PM-Scl	anti-SSB	0.042925	0
B99	B109	anti-PM-Scl	AMA-M2	-0.00584	3.36E-07

B99	B199	anti-PM-Scl	Mg	0.007926	4.51E-12
B99	B179	anti-PM-Scl	α1-MG	-0.00703	8.31E-10
B99	B125	anti-PM-Scl	Cor	0.016071	0
B99	B138	anti-PM-Scl	PAB	-0.00626	4.54E-08
B99	B196	anti-PM-Scl	TCO2	-0.00612	9.15E-08
B99	B183	anti-PM-Scl	PTA	-0.00686	2.05E-09
B99	B186	anti-PM-Scl	PFC	0.007068	6.81E-10
B99	B212	anti-PM-Scl	IOP-L	0.006889	1.81E-09

Code	PEI Name	PEI Name abbreviation	Unit
B1	Gender	Gender	-
B2	Age	Age	years
B3	Homocysteine	Hcy	umol/L
B4	Whole Blood Calcium	WB-Ca	mmol/L
B5	Whole Blood Magnesium	WB-Mg	mmol/L
B6	Iron Of Whole Blood	WB-Fe	mmol/L
B7	Whole Blood Copper	WB-Cop	umol/L
B8	Zinc In Whole Blood	WB-Zinc	umol/L
B9	Rh Blood Group	RH	-
B10	Pepsinogen I	PGI	ug/L
B11	PgI/PgII	PGI/PGII	-
B12	Pepsinogen II	PGII	ug/L
B14	Gastrin-17	G-17	pmol/l
B15	Cd3-Cd19+(B Cell)	CD3-CD19+(B cell)	-
B16	Cd3+ Total T Lymphocyte	CD3+T	-
B17	Cd3+Cd16+Cd56+Nk	CD3+CD16+CD56+NK	-
B18	Cd3+Cd4+Helper(Inducer)T Cells	CD3+CD4+helper(inducer)T	-
B19	Cd3+Cd4+/Cd3+Cd8+	CD3+CD4+/CD3+CD8+	-
B20	Cd3+Cd4+Cd8+	CD3+CD4+CD8+	-
B21	Cd3+Cd4-Cd8-	CD3+CD4-CD8-	-
B22	Cd3+Cd8+(T)	CD3+CD8+(T)	-
B23	Cd3-Cd16+Cd56+(Nk)	CD3-CD16+CD56+(NK)	-
B24	α Hydroxybutyrate Dehydrogenas	αHBDH	U/L
B25	Creatine Kinase	CK	U/L
B26	Creatine Kinase Isoenzyme	CKMB	U/L
B27	Lactic Dehydrogenase	LDH	U/L
B28	Pituitary Prolactin	PRL	mIU/L
B29	Estradiol E2	E2	pmol/L
B30	Luteinizing Hormone	LH	mIU/ml

B31	Follicle Stimulating Hormone	FSH	mIU/ml
B32	Testosterone	Testo	nmol/L
B33	Progesterone	Prog	nmol/L
B34	Thymidine Kinase	TK	PM/L
B35	Blood Beta-Microglobulin	β2-MG	-
B36	Variant Lymphocyte Rate	V-Lym%	-
B37	Band Cell	BAND	10E-9/L
B38	Band Cell%	BAND%	-
B39	Other Cell Types	Other cells	-
B40	Leukocyte Count	WBC	10E-9/L
B41	Monocytes	MON	-
B42	Monocytes %	MON %	10E-9/L
B43	Mean Corpuscular Volume	MCV	fl
B44	Mean Corpuscular Hemoglobin	MCH	pg
B45	Mean Corpuscular Hemoglobin Concentration	MCHC	g/l
B46	Red Blood Cell Count	RBC Count	10E-12/L
B47	Red Cell Distribution Width	RDW	fl
B48	Lymphocyte Rate	LYM%	-
B49	Basophilic Granulocyte Rate	baso%	-
B50	Eosinophil Rate	eo%	-
B51	Hemoglobin	Hb	g/L
B52	Platelet Count	PLT/BPC	10E-9/L
B53	Granulocyte Rate	GR%	-
B54	Erythrocyte Sedimentation Rate	ESR	mm/60min
B55	Erythrocyte Deformation Index	TK	-
B56	The Index Of Rigidity Of Erythrocyte	IR	-
B57	Erythrocyte Aggregation Indices	RBCAI	-
B58	Hematocrit	HCT	-
B59	Whole Blood Reduced Viscosity	WBRV	-
B60	Whole Blood Reduced Viscosity Index	WBRVI	-
B61	Whole Blood High Shear Reduced Viscosity	WBHSRV	-
B62	Whole Blood High Shear Reduced Viscosity Index	WBHSRVI	-
B63	Shear Rate Of Whole Blood Viscosity_Mpas_1 (200)	WBRSR(mPas_200)	-
B64	Shear Rate Of Whole Blood Viscosity_Mpas_3 (5)	WBRSR(mPas_5)	-
B65	Shear Rate Of Whole Blood Viscosity_Mpas_4 (1)	WBRSR(mPas_1)	-
B66	Shear Rate Of Whole Blood Viscosity_Mpas (100)	WBRSR(mPas_100)	-
B67	Shear Rate Of Whole Blood Viscosity_Mpas (50)	WBRSR(mPas_50)	-
B69	Plasma Viscosity	PV	-
B70	Low-Density Lipoprotein	LDL-C	mmol/L
B71	Triglyceride	TG	mmol/L
B72	High-Density Lipoprotein	HDL-C	mmol/L
B73	Apolipoprotein A	ApoA	mg/L
B74	Apolipoprotein A1	ApoA1	g/L
B75	Apolipoprotein A1/B	ApoA1/B	g/L

B76	Apolipoprotein B	ApoB	g/L
B77	Total Cholesterol	TC	mmol/L
B78	Hepatitis B Virus Core Im Antibod	HBcAb	-
B79	Quantitative Detection Of Hepatitis B Virus Dna	hbV-dna	IU/ml
B80	Helicobacter Pylori Antibody-Igg	anti-HP IgG	-
B81	Tumor Supplied Group Of Factors	TSGF	U/ML
B82	Carcinoembryonic Antigen	CEA	ng/ml
B83	Alpha-Fetoprotein	AFP	ng/ml
B84	Complexed Prostate Special Antigen	C-PSA	ng/ml
B85	Squamous Cell Carcinoma Antigen	SCC	ng/ml
B86	Chorionic Gonadotropin	TBhCG	mIU/ml
B87	Neuron-Specific Enolase	NSE	ng/ml
B88	Carbohydrate Antigen， Ca242	CA 242	U/ml
B89	Carbohydrate Antigen， Ca72_4	CA 72-4	U/ml
B90	Cytokeratin-19-Fragment Cyfra21-1	CYFRA 21-1	ng/ml
B91	Total Protein	TP	g/L
B92	Aldh2-Glu504Glu	ALDH2-Glu504Glu	-
B93	Aldh2-Glu504Lys	ALDH2-Glu504Lys	-
B94	Aldh2-Lys504Lys	ALDH2-Lys504Lys	-
B95	Anti-Cenpb	anti-cenpb	-
B96	Dsdna (Iif)	dsDNA (IIF)	-
B97	Anti-Dsdna Antibody	dsDNA	-
B98	Jo-1 Antibody	anti-Jo-1	-
B99	Anti-Pm-Scl	anti-PM-Scl	-
B100	Anti-Ro52	anti-Ro52	-
B101	Anti-Sc70	anti-Sc70	-
B102	Anti Sm Antibody	anti-Sm	-
B103	Anti-Ssa/Ro Antibody	anti-SSA/Ro	-
B104	Anti Ssb Antibody	anti-SSB	-
B105	Anti-U1Rnp Antibody	anti-U1RNP	-
B106	Anti-Ribosomal P Protein Antibody	anti-P	-
B107	Anti-Nucleosome Antibody Systemic	AnuA	-
B108	Anti-Cyclin Antibody	Anti-Cyclin	-
B109	Antimitochondrial Antibody M2	AMA-M2	-
B110	Anti-Histone Antibody	AHA	-
B111	Thyroid-Stimulating Hormon	TSH	mIU/L
B112	Free Thyroxine	FT4	pmol/L
B113	Free Triiodothyronine	FT3	pmol/L
B114	Total Thyroxine	TSH TT3 TT4(TT4)	nmol/L
B115	Total Triiodothyronine	TSH TT3 TT4(TT3)	nmol/L
B116	Total Triiodothyronine	TT3	nmol/L
B117	Total Thyroxine	TT4	nmol/L
B118	Carcinoembryonic Antigen 1	CEA1	ng/ml
B119	Quantitative Detection Of Hepatitis C Virus Rna	Hcv-rna	-

B120	Hcv Core Antigen	HCV-cAg	-
B121	Complement C3	C3	-
B122	Complement C4	C4	-
B123	High Sensitivity C-Reactive Protein 1	hs-CRP1	mg/L
B124	Adrenocorticotropic Hormone	ACTH	pg/ml
B125	Cortisol	Cor	ug/dl
B126	Albumin	Alb	g/L
B127	Albumin-Globulin	A-G ratio	-
B128	Alanine Aminotransferase	ALT	U/L
B129	Indirect Bilirubin	IBIL	umol/L
B130	Globulin	GLB	g/L
B131	Aspartate Transaminase	AST	U/L
B132	Direct Bilirubin	Dbil	umol/L
B133	Total Bilirubin	TBIL	umol/L
B134	Cholinesterases	CHE	KU/L
B135	Total Bile Acid	TBA	umol/L
B136	5-Nucleotidase	5'NT	U/L
B137	Alpha-L-Fucosidase	AFU	U/L
B138	Prealbumin	PAB	mg/L
B139	Adenosine Deaminase	ADA	U/L
B140	R-Glutamyl Transpeptidase	r-GT	U/L
B141	Ast/Alt	AST/ALT	-
B142	25-Hydroxyvitamin D	25-(OH)D	ng/mL
B143	β-Crosslaps	β-CTx	pg/mL
B144	Total-Pinp	Total-PINP	ng/mL
B145	Osteocalcin	BGP	ng/mL
B146	Cystatin C	CysC	mg/L
B148	Antithyroperoxidase Antibody	Anti_TPO	IU/ml
B149	Anti-Thyroglobulin Antibodies	TGAb	IU/ml
B150	Alkaline Phosphatase	ALP	U/L
B151	Anti-Cyclic Peptide Containing Citrulline	Anti-CCP	Ru/ml
B152	Anti Cardiolipin Antibodyig(G-A-M)	ACA-Ig(G-A-M)	u/ml
B153	Glucose	Glu	mmol/L
B154	Rheumatoid Factor	RF	IU/ml
B155	Immunoglobulin A	IgA	g/l
B156	Immunoglobulin E	IgE	IU/ml
B157	Immunoglobulin G	IgG	g/l
B158	Immunoglobulin M	IgM	g/l
B159	Urine Beta-Microglobulin	β2-MG	-
B160	Ph	PH	-
B161	Leucocyte	LEU	/ul
B163	Specific Gravity	SG	-
B164	Calcium Oxalate Crystals	COM	HPF
B165	Bilirubin	bilirubin	-

B166	Protein	Pro	-
B167	Phosphate Crystals	PC	-
B168	Urinary Nuclear Matrix Protein 22	NMP22	-
B169	Urate Crystal	U-CRY	-
B170	Urine Sugar	U-GLU	-
B171	Epithelial Cell	EC	HPF
B172	Ketone Body	KB	-
B173	Nitrous Acid	NTT	-
B174	Urea Creatinine	Urea/Cr	-
B175	Microalbuminuria	ALB-U	mg/L
B176	Urine Creatinine	Ucr	umol/L
B177	Ratio Of Urinary Microalbumin To Creatinin	ACR	ug/mg
B178	Urinary Immunoglobulin G	Ig-U	mg/L
B179	Urine Alpha-Microglobulin	α1-MG	mg/L
B180	Urine Transferrin	Tf	mg/L
B181	Activated Partial Thromboplastin Time	APTT	s
B182	Thrombin Time	TT	s
B183	Percentage Activity Of Prothrombin In Plasma	PTA	-
B184	International Normalized Ratio	INR	-
B185	Prothrombin Time	PT	s
B186	Plasma Fibrinogen Concentration	PFC	g/l
B187	Neuron-Specific Enolase	NSE	ng/ml
B188	Calcium	Ca	mmol/L
B189	Estimated Glomerular Filtration Rate	eGFB	ml/min
B190	Creatinine	Cre	umol/L
B191	Urea	Urea	mmol/L
B192	Uric Acid	UA	umol/L
B193	Kalium	K	mmol/L
B194	Chlorine	Cl	mmol/L
B195	Sodium	Na	mmol/L
B196	Blood Carbon Dioxide	TCO2	mmol/L
B197	Anion Gap	AG	mmol/L
B198	Phosphorus	P	mmol/L
B199	Magnesium	Mg	mmol/L
B200	Osmotic Pressure	OP	mOsm/L
B201	Exercise Habits	e-habits	-
B202	Frequency Of Exercises	e-times	-
B203	Dietary Habits 1	DH	-
B204	History Of Alcohol Intake	Drinking	-
B205	Known Of Drug Allergy	KDA	-
B206	Waistline	Waist	cm
B207	Waist-Hip Ratio	WHR	-
B209	Smoking History	Smoking	-
B210	Hipline	Hip	cm

B211	Intra-Ocular Tension Of The Right Eye	IOP-R	mmHg
B212	Intra-Ocular Tension Of The Left Eye	IOP-L	mmHg
B213	Corrected Visual Acuity The Right Eye	CVA-R	-
B214	Corrected Visual Acuity The Left Eye	CVA-L	-
B215	Body Mass Index	BMI	-
B216	Body Mass	BMI	kg
B218	Diastolic Pressure	DBP	mmHg
B219	Operation History	OT	-
B220	Systolic Pressure	SBP	mmHg
B221	Height	Hei	cm
B222	Neck Size	NS	cm
B223	Family History	FH	-
B224	Anamnesis	pmh	-
B225	Hourly Postprandial Blood Sugar	hPBG	mmol/L
B227	Vision Of The Right Eye	OD	-
B228	Vision Of The Left Eye	OS	-

Supplementary Table 2. PEI Correlations in Cholecystolithiasis

Row	Column	Row	Column	Cor	*P*
B1	B44	Gender	MCH	-0.18202	9.05E-09
B1	B45	Gender	MCHC	-0.31897	0
B1	B46	Gender	RBC Count	-0.50021	0
B1	B51	Gender	Hb	-0.64675	0
B1	B71	Gender	TG	-0.18502	5.09E-09
B1	B72	Gender	HDL-C	0.304637	0
B1	B126	Gender	Alb	-0.17113	6.72E-08
B1	B127	Gender	A-G ratio	-0.17028	7.82E-08
B1	B128	Gender	ALT	-0.28035	0
B1	B131	Gender	AST	-0.15719	7.31E-07
B1	B140	Gender	r-GT	-0.252	8.88E-16
B1	B141	Gender	AST/ALT	0.252548	8.88E-16
B1	B161	Gender	LEU	0.16185	3.37E-07
B1	B163	Gender	SG	-0.37088	0
B1	B166	Gender	Pro	-0.17468	3.55E-08
B1	B174	Gender	Urea/Cr	0.3788	0
B1	B190	Gender	Cre	-0.51593	0
B1	B192	Gender	UA	-0.562	0
B1	B204	Gender	Drinking	-0.36613	0
B1	B206	Gender	Waist	-0.50716	0
B1	B209	Gender	Smoking	-0.39753	0
B1	B210	Gender	Hip	-0.31913	0
B1	B215	Gender	BMI	-0.29241	0
B1	B216	Gender	BMI	-0.60175	0
B1	B218	Gender	DBP	-0.19742	4.29E-10
B1	B221	Gender	Hei	-0.67814	0
B1	B222	Gender	NS	-0.72003	0
B10	B12	PG I	PG II	0.502872	0
B10	B189	PG I	eGFB	-0.23336	1.27E-13
B10	B190	PG I	Cre	0.499029	0
B10	B191	PG I	Urea	0.37099	0
B100	B101	anti-Ro52	anti-Sc70	-0.34578	0
B100	B102	anti-Ro52	anti-Sm	-0.34578	0
B100	B103	anti-Ro52	anti-SSA/Ro	0.97918	0
B100	B104	anti-Ro52	anti-SSB	0.98189	0
B100	B105	anti-Ro52	anti-U1RNP	-0.34578	0
B100	B106	anti-Ro52	anti-P	-0.34578	0
B100	B107	anti-Ro52	AnuA	-0.34578	0
B100	B108	anti-Ro52	Anti-Cyclin	-0.34578	0
B100	B109	anti-Ro52	AMA-M2	-0.34578	0
B100	B110	anti-Ro52	AHA	-0.34578	0
B100	B157	anti-Ro52	IgG	0.516221	0

B101	B102	anti-Sc70	anti-Sm	1	0
B101	B103	anti-Sc70	anti-SSA/Ro	-0.27591	0
B101	B104	anti-Sc70	anti-SSB	-0.32367	0
B101	B105	anti-Sc70	anti-U1RNP	1	0
B101	B107	anti-Sc70	AnuA	1	0
B101	B108	anti-Sc70	Anti-Cyclin	1	0
B101	B109	anti-Sc70	AMA-M2	1	0
B101	B134	anti-Sc70	CHE	-0.29695	0
B101	B151	anti-Sc70	Anti-CCP	-0.23405	1.07E-13
B101	B155	anti-Sc70	IgA	-0.17398	4.03E-08
B101	B156	anti-Sc70	IgE	-0.42651	0
B101	B157	anti-Sc70	IgG	-0.21397	1.21E-11
B101	B175	anti-Sc70	ALB-U	0.22405	1.19E-12
B101	B176	anti-Sc70	Ucr	0.200525	2.24E-10
B101	B178	anti-Sc70	Ig-U	0.771362	0
B101	B179	anti-Sc70	α1-MG	0.719949	0
B101	B180	anti-Sc70	Tf	0.779882	0
B101	B181	anti-Sc70	APTT	0.331973	0
B101	B182	anti-Sc70	TT	0.330901	0
B101	B183	anti-Sc70	PTA	0.331949	0
B101	B184	anti-Sc70	INR	0.331973	0
B101	B185	anti-Sc70	PT	0.331264	0
B101	B186	anti-Sc70	PFC	-0.33197	0
B101	B194	anti-Sc70	Cl	-0.33683	0
B101	B197	anti-Sc70	AG	0.254711	4.44E-16
B102	B103	anti-Sm	anti-SSA/Ro	-0.27591	0
B102	B104	anti-Sm	anti-SSB	-0.32367	0
B102	B107	anti-Sm	AnuA	1	0
B102	B134	anti-Sm	CHE	-0.29695	0
B102	B151	anti-Sm	Anti-CCP	-0.23405	1.07E-13
B102	B155	anti-Sm	IgA	-0.17398	4.03E-08
B102	B156	anti-Sm	IgE	-0.42651	0
B102	B157	anti-Sm	IgG	-0.21397	1.21E-11
B102	B175	anti-Sm	ALB-U	0.22405	1.19E-12
B102	B176	anti-Sm	Ucr	0.200525	2.24E-10
B102	B178	anti-Sm	Ig-U	0.771362	0
B102	B179	anti-Sm	α1-MG	0.719949	0
B102	B180	anti-Sm	Tf	0.779882	0
B102	B181	anti-Sm	APTT	0.331973	0
B102	B182	anti-Sm	TT	0.330901	0
B102	B183	anti-Sm	PTA	0.331949	0
B102	B184	anti-Sm	INR	0.331973	0
B102	B185	anti-Sm	PT	0.331264	0
B102	B186	anti-Sm	PFC	-0.33197	0

B102	B194	anti-Sm	Cl	-0.33683	0
B102	B197	anti-Sm	AG	0.254711	4.44E-16
B103	B104	anti-SSA/Ro	anti-SSB	0.998747	0
B103	B105	anti-SSA/Ro	anti-U1RNP	-0.27591	0
B103	B106	anti-SSA/Ro	anti-P	-0.27591	0
B103	B107	anti-SSA/Ro	AnuA	-0.27591	0
B103	B108	anti-SSA/Ro	Anti-Cyclin	-0.27591	0
B103	B109	anti-SSA/Ro	AMA-M2	-0.27591	0
B103	B110	anti-SSA/Ro	AHA	-0.27591	0
B103	B157	anti-SSA/Ro	IgG	0.524665	0
B104	B105	anti-SSB	anti-U1RNP	-0.32367	0
B104	B106	anti-SSB	anti-P	-0.32367	0
B104	B107	anti-SSB	AnuA	-0.32367	0
B104	B108	anti-SSB	Anti-Cyclin	-0.32367	0
B104	B109	anti-SSB	AMA-M2	-0.32367	0
B104	B110	anti-SSB	AHA	-0.32367	0
B104	B157	anti-SSB	IgG	0.527611	0
B105	B106	anti-U1RNP	anti-P	1	0
B105	B107	anti-U1RNP	AnuA	1	0
B105	B109	anti-U1RNP	AMA-M2	1	0
B105	B134	anti-U1RNP	CHE	-0.29695	0
B105	B151	anti-U1RNP	Anti-CCP	-0.23405	1.07E-13
B105	B155	anti-U1RNP	IgA	-0.17398	4.03E-08
B105	B156	anti-U1RNP	IgE	-0.42651	0
B105	B157	anti-U1RNP	IgG	-0.21397	1.21E-11
B105	B175	anti-U1RNP	ALB-U	0.22405	1.19E-12
B105	B176	anti-U1RNP	Ucr	0.200525	2.24E-10
B105	B178	anti-U1RNP	Ig-U	0.771362	0
B105	B179	anti-U1RNP	α1-MG	0.719949	0
B105	B180	anti-U1RNP	Tf	0.779882	0
B105	B181	anti-U1RNP	APTT	0.331973	0
B105	B182	anti-U1RNP	TT	0.330901	0
B105	B183	anti-U1RNP	PTA	0.331949	0
B105	B184	anti-U1RNP	INR	0.331973	0
B105	B185	anti-U1RNP	PT	0.331264	0
B105	B186	anti-U1RNP	PFC	-0.33197	0
B105	B194	anti-U1RNP	Cl	-0.33683	0
B105	B197	anti-U1RNP	AG	0.254711	4.44E-16
B106	B107	anti-P	AnuA	1	0
B106	B108	anti-P	Anti-Cyclin	1	0
B106	B109	anti-P	AMA-M2	1	0
B106	B110	anti-P	AHA	1	0
B106	B134	anti-P	CHE	-0.29695	0
B106	B151	anti-P	Anti-CCP	-0.23405	1.07E-13

B106	B155	anti-P	IgA	-0.17398	4.03E-08
B106	B156	anti-P	IgE	-0.42651	0
B106	B157	anti-P	IgG	-0.21397	1.21E-11
B106	B175	anti-P	ALB-U	0.22405	1.19E-12
B106	B176	anti-P	Ucr	0.200525	2.24E-10
B106	B178	anti-P	Ig-U	0.771362	0
B106	B179	anti-P	α1-MG	0.719949	0
B106	B180	anti-P	Tf	0.779882	0
B106	B181	anti-P	APTT	0.331973	0
B106	B182	anti-P	TT	0.330901	0
B106	B183	anti-P	PTA	0.331949	0
B106	B184	anti-P	INR	0.331973	0
B106	B185	anti-P	PT	0.331264	0
B106	B186	anti-P	PFC	-0.33197	0
B106	B194	anti-P	Cl	-0.33683	0
B106	B197	anti-P	AG	0.254711	4.44E-16
B107	B108	AnuA	Anti-Cyclin	1	0
B107	B109	AnuA	AMA-M2	1	0
B107	B110	AnuA	AHA	1	0
B107	B134	AnuA	CHE	-0.29695	0
B107	B151	AnuA	Anti-CCP	-0.23405	1.07E-13
B107	B155	AnuA	IgA	-0.17398	4.03E-08
B107	B156	AnuA	IgE	-0.42651	0
B107	B157	AnuA	IgG	-0.21397	1.21E-11
B107	B175	AnuA	ALB-U	0.224049	1.19E-12
B107	B176	AnuA	Ucr	0.200525	2.25E-10
B107	B178	AnuA	Ig-U	0.771362	0
B107	B179	AnuA	α1-MG	0.719949	0
B107	B180	AnuA	Tf	0.779882	0
B107	B181	AnuA	APTT	0.331973	0
B107	B182	AnuA	TT	0.330901	0
B107	B183	AnuA	PTA	0.331949	0
B107	B184	AnuA	INR	0.331973	0
B107	B185	AnuA	PT	0.331264	0
B107	B186	AnuA	PFC	-0.33197	0
B107	B194	AnuA	Cl	-0.33683	0
B107	B197	AnuA	AG	0.254711	4.44E-16
B108	B110	Anti-Cyclin	AHA	1	0
B108	B134	Anti-Cyclin	CHE	-0.29695	0
B108	B151	Anti-Cyclin	Anti-CCP	-0.23405	1.07E-13
B108	B155	Anti-Cyclin	IgA	-0.17398	4.03E-08
B108	B156	Anti-Cyclin	IgE	-0.42651	0
B108	B157	Anti-Cyclin	IgG	-0.21397	1.21E-11
B108	B175	Anti-Cyclin	ALB-U	0.22405	1.19E-12

B108	B176	Anti-Cyclin	Ucr	0.200525	2.24E-10
B108	B178	Anti-Cyclin	Ig-U	0.771362	0
B108	B179	Anti-Cyclin	α1-MG	0.719949	0
B108	B180	Anti-Cyclin	Tf	0.779882	0
B108	B181	Anti-Cyclin	APTT	0.331973	0
B108	B182	Anti-Cyclin	TT	0.330901	0
B108	B183	Anti-Cyclin	PTA	0.331949	0
B108	B184	Anti-Cyclin	INR	0.331973	0
B108	B185	Anti-Cyclin	PT	0.331264	0
B108	B186	Anti-Cyclin	PFC	-0.33197	0
B108	B194	Anti-Cyclin	Cl	-0.33683	0
B108	B197	Anti-Cyclin	AG	0.254711	4.44E-16
B109	B110	AMA-M2	AHA	1	0
B109	B134	AMA-M2	CHE	-0.29695	0
B109	B151	AMA-M2	Anti-CCP	-0.23405	1.07E-13
B109	B155	AMA-M2	IgA	-0.17398	4.03E-08
B109	B156	AMA-M2	IgE	-0.42651	0
B109	B157	AMA-M2	IgG	-0.21397	1.21E-11
B109	B175	AMA-M2	ALB-U	0.22405	1.19E-12
B109	B176	AMA-M2	Ucr	0.200525	2.24E-10
B109	B178	AMA-M2	Ig-U	0.771362	0
B109	B179	AMA-M2	α1-MG	0.719949	0
B109	B180	AMA-M2	Tf	0.779882	0
B109	B181	AMA-M2	APTT	0.331973	0
B109	B182	AMA-M2	TT	0.330901	0
B109	B183	AMA-M2	PTA	0.331949	0
B109	B184	AMA-M2	INR	0.331973	0
B109	B185	AMA-M2	PT	0.331264	0
B109	B186	AMA-M2	PFC	-0.33197	0
B109	B194	AMA-M2	Cl	-0.33683	0
B109	B197	AMA-M2	AG	0.254711	4.44E-16
B11	B12	PGI/PGII	PG II	-0.41922	0
B11	B14	PGI/PGII	G-17	-0.1779	1.96E-08
B11	B95	PGI/PGII	anti-cenpb	-0.28758	0
B11	B97	PGI/PGII	dsDNA	-0.28758	0
B11	B98	PGI/PGII	anti-Jo-1	-0.28758	0
B11	B101	PGI/PGII	anti-Sc70	-0.28758	0
B11	B102	PGI/PGII	anti-Sm	-0.28758	0
B11	B105	PGI/PGII	anti-U1RNP	-0.28758	0
B11	B106	PGI/PGII	anti-P	-0.28758	0
B11	B107	PGI/PGII	AnuA	-0.28758	0
B11	B108	PGI/PGII	Anti-Cyclin	-0.28758	0
B11	B109	PGI/PGII	AMA-M2	-0.28758	0
B11	B110	PGI/PGII	AHA	-0.28758	0

B110	B134	AHA	CHE	-0.29695	0
B110	B151	AHA	Anti-CCP	-0.23405	1.07E-13
B110	B155	AHA	IgA	-0.17398	4.03E-08
B110	B156	AHA	IgE	-0.42651	0
B110	B157	AHA	IgG	-0.21397	1.21E-11
B110	B175	AHA	ALB-U	0.22405	1.19E-12
B110	B176	AHA	Ucr	0.200525	2.24E-10
B110	B178	AHA	Ig-U	0.771362	0
B110	B179	AHA	α1-MG	0.719949	0
B110	B180	AHA	Tf	0.779882	0
B110	B181	AHA	APTT	0.331973	0
B110	B182	AHA	TT	0.330901	0
B110	B183	AHA	PTA	0.331949	0
B110	B184	AHA	INR	0.331973	0
B110	B185	AHA	PT	0.331264	0
B110	B186	AHA	PFC	-0.33197	0
B110	B194	AHA	Cl	-0.33683	0
B110	B197	AHA	AG	0.254711	4.44E-16
B111	B134	TSH	CHE	0.195932	5.82E-10
B111	B148	TSH	Anti_TPO	0.248254	2.89E-15
B111	B149	TSH	TGAb	0.182178	8.78E-09
B112	B114	FT4	TSH TT3 TT4(TT4)	0.375623	0
B112	B154	FT4	RF	0.160852	3.98E-07
B113	B115	FT3	TSH TT3 TT4(TT3)	0.303551	0
B113	B148	FT3	Anti_TPO	-0.15709	7.43E-07
B114	B115	TSH TT3 TT4(TT4)	TSH TT3 TT4(TT3)	0.334011	0
B115	B124	TSH TT3 TT4(TT3)	ACTH	0.166034	1.65E-07
B115	B125	TSH TT3 TT4(TT3)	Cor	0.166034	1.65E-07
B116	B167	TT3	PC	0.465184	0
B116	B202	TT3	e-times	0.708765	0
B117	B167	TT4	PC	0.576681	0
B117	B202	TT4	e-times	0.843804	0
B118	B176	CEA1	Ucr	-0.16162	3.50E-07
B118	B214	CEA1	CVA-L	0.168556	1.06E-07
B118	B220	CEA1	SBP	0.163352	2.61E-07
B118	B228	CEA1	OS	0.232578	1.53E-13
B119	B120	Hcv-rna	HCV-cAg	0.56139	0
B119	B121	Hcv-rna	C3	-0.88101	0
B119	B122	Hcv-rna	C4	-0.88101	0
B119	B159	Hcv-rna	β2-MG	-0.88101	0
B119	B169	Hcv-rna	U-CRY	-0.88101	0
B119	B195	Hcv-rna	Na	0.242012	1.44E-14
B119	B200	Hcv-rna	OP	0.182567	8.15E-09
B12	B14	PG II	G-17	0.22303	1.52E-12

B12	B80	PG II	anti-HP IgG	0.194788	7.35E-10
B120	B121	HCV-cAg	C3	-0.49459	0
B120	B122	HCV-cAg	C4	-0.49459	0
B120	B154	HCV-cAg	RF	-0.1817	9.62E-09
B120	B157	HCV-cAg	IgG	-0.19536	6.54E-10
B120	B159	HCV-cAg	β2-MG	-0.49459	0
B120	B169	HCV-cAg	U-CRY	-0.49459	0
B121	B122	C3	C4	1	0
B121	B159	C3	β2-MG	1	0
B121	B169	C3	U-CRY	0.999999	0
B121	B195	C3	Na	-0.19632	5.38E-10
B122	B195	C4	Na	-0.19632	5.38E-10
B123	B138	hs-CRP1	PAB	-0.30676	0
B123	B139	hs-CRP1	ADA	0.283631	0
B123	B146	hs-CRP1	CysC	-0.15627	8.50E-07
B123	B154	hs-CRP1	RF	0.293498	0
B123	B168	hs-CRP1	NMP22	-0.23009	2.82E-13
B123	B193	hs-CRP1	K	-0.21884	4.02E-12
B123	B195	hs-CRP1	Na	-0.27462	0
B123	B199	hs-CRP1	Mg	-0.2122	1.80E-11
B123	B211	hs-CRP1	IOP-R	0.174237	3.84E-08
B123	B212	hs-CRP1	IOP-L	0.159912	4.66E-07
B124	B125	ACTH	Cor	1	0
B126	B127	Alb	A-G ratio	0.519628	0
B126	B129	Alb	IBIL	0.191077	1.55E-09
B126	B130	Alb	GLB	-0.20895	3.69E-11
B126	B133	Alb	TBIL	0.17225	5.50E-08
B126	B188	Alb	Ca	0.34146	0
B126	B189	Alb	eGFB	0.194454	7.86E-10
B126	B192	Alb	UA	0.157443	7.01E-07
B126	B221	Alb	Hei	0.218326	4.51E-12
B127	B129	A-G ratio	IBIL	0.21057	2.58E-11
B127	B130	A-G ratio	GLB	-0.90294	0
B127	B133	A-G ratio	TBIL	0.194415	7.92E-10
B127	B221	A-G ratio	Hei	0.217709	5.20E-12
B128	B131	ALT	AST	0.777816	0
B128	B140	ALT	r-GT	0.469661	0
B128	B141	ALT	AST/ALT	-0.56915	0
B128	B163	ALT	SG	0.186083	4.15E-09
B128	B192	ALT	UA	0.315074	0
B128	B206	ALT	Waist	0.34859	0
B128	B210	ALT	Hip	0.254	6.66E-16
B128	B215	ALT	BMI	0.344584	0
B128	B216	ALT	BMI	0.381513	0

B128	B221	ALT	Hei	0.206616	6.13E-11
B128	B222	ALT	NS	0.351156	0
B129	B130	IBIL	GLB	-0.173	4.81E-08
B129	B132	IBIL	Dbil	0.677229	0
B129	B133	IBIL	TBIL	0.98049	0
B130	B133	GLB	TBIL	-0.16015	4.48E-07
B130	B221	GLB	Hei	-0.15737	7.10E-07
B131	B140	AST	r-GT	0.288175	0
B131	B141	AST	AST/ALT	-0.16313	2.71E-07
B131	B192	AST	UA	0.219507	3.44E-12
B131	B206	AST	Waist	0.20965	3.17E-11
B131	B215	AST	BMI	0.200723	2.15E-10
B131	B216	AST	BMI	0.181482	1.00E-08
B131	B222	AST	NS	0.185155	4.96E-09
B132	B133	Dbil	TBIL	0.808538	0
B132	B139	Dbil	ADA	0.157393	7.07E-07
B134	B151	CHE	Anti-CCP	0.474144	0
B134	B155	CHE	IgA	0.525154	0
B134	B178	CHE	Ig-U	-0.31521	0
B134	B179	CHE	α1-MG	-0.1735	4.39E-08
B134	B180	CHE	Tf	-0.34073	0
B134	B193	CHE	K	-0.25664	4.44E-16
B134	B197	CHE	AG	-0.26748	0
B135	B137	TBA	AFU	0.224115	1.17E-12
B135	B139	TBA	ADA	0.303365	0
B135	B148	TBA	Anti_TPO	0.164122	2.29E-07
B135	B153	TBA	Glu	0.180074	1.31E-08
B135	B194	TBA	Cl	-0.23849	3.51E-14
B135	B197	TBA	AG	0.301568	0
B135	B198	TBA	P	0.160832	4.00E-07
B135	B199	TBA	Mg	0.178777	1.67E-08
B135	B200	TBA	OP	0.233061	1.36E-13
B136	B140	5'NT	r-GT	0.267343	0
B136	B150	5'NT	ALP	0.253871	6.66E-16
B136	B196	5'NT	TCO2	-0.17394	4.06E-08
B137	B139	AFU	ADA	0.362117	0
B137	B146	AFU	CysC	0.216388	7.03E-12
B137	B150	AFU	ALP	0.176001	2.78E-08
B137	B153	AFU	Glu	0.273862	0
B137	B194	AFU	Cl	-0.19824	3.61E-10
B137	B200	AFU	OP	0.380681	0
B137	B205	AFU	KDA	0.171724	6.04E-08
B138	B139	PAB	ADA	-0.39446	0
B138	B150	PAB	ALP	-0.15919	5.26E-07

B138	B156	PAB	IgE	-0.25107	1.33E-15
B138	B175	PAB	ALB-U	0.242752	1.20E-14
B138	B178	PAB	Ig-U	0.165432	1.83E-07
B138	B179	PAB	α1-MG	0.161145	3.79E-07
B138	B180	PAB	Tf	0.165287	1.87E-07
B138	B194	PAB	Cl	-0.18783	2.95E-09
B138	B199	PAB	Mg	0.23943	2.78E-14
B139	B150	ADA	ALP	0.299514	0
B139	B153	ADA	Glu	0.420627	0
B139	B170	ADA	U-GLU	0.199851	2.59E-10
B139	B194	ADA	Cl	-0.29477	0
B139	B195	ADA	Na	-0.16638	1.55E-07
B139	B200	ADA	OP	0.514541	0
B139	B205	ADA	KDA	0.177994	1.93E-08
B14	B129	G-17	IBIL	0.166838	1.43E-07
B14	B133	G-17	TBIL	0.163868	2.39E-07
B140	B141	r-GT	AST/ALT	-0.34942	0
B140	B192	r-GT	UA	0.275665	0
B140	B204	r-GT	Drinking	0.172709	5.06E-08
B140	B206	r-GT	Waist	0.305629	0
B140	B210	r-GT	Hip	0.241205	1.78E-14
B140	B215	r-GT	BMI	0.283028	0
B140	B216	r-GT	BMI	0.321796	0
B140	B218	r-GT	DBP	0.161083	3.83E-07
B140	B221	r-GT	Hei	0.17885	1.64E-08
B140	B222	r-GT	NS	0.323443	0
B141	B163	AST/ALT	SG	-0.18847	2.60E-09
B141	B192	AST/ALT	UA	-0.23555	7.37E-14
B141	B206	AST/ALT	Waist	-0.26774	0
B141	B210	AST/ALT	Hip	-0.18236	8.48E-09
B141	B215	AST/ALT	BMI	-0.2619	0
B141	B216	AST/ALT	BMI	-0.33123	0
B141	B221	AST/ALT	Hei	-0.23435	9.90E-14
B141	B222	AST/ALT	NS	-0.29951	0
B142	B143	25-(OH)D	β-CTx	-1	0
B142	B175	25-(OH)D	ALB-U	-0.17475	3.50E-08
B143	B144	β-CTx	Total-PINP	0.999997	0
B143	B145	β-CTx	BGP	0.999997	0
B143	B175	β-CTx	ALB-U	0.174778	3.48E-08
B144	B175	Total-PINP	ALB-U	0.174746	3.50E-08
B145	B175	BGP	ALB-U	0.174746	3.50E-08
B146	B181	CysC	APTT	-0.17173	6.04E-08
B146	B182	CysC	TT	-0.17121	6.63E-08
B146	B183	CysC	PTA	-0.17172	6.05E-08

B146	B184	CysC	INR	-0.17173	6.04E-08
B146	B185	CysC	PT	-0.17135	6.46E-08
B146	B186	CysC	PFC	0.171732	6.04E-08
B146	B189	CysC	eGFB	-0.18361	6.68E-09
B146	B192	CysC	UA	0.174623	3.58E-08
B146	B195	CysC	Na	0.1554	9.79E-07
B146	B199	CysC	Mg	0.182408	8.40E-09
B146	B200	CysC	OP	0.226437	6.77E-13
B146	B206	CysC	Waist	0.187368	3.23E-09
B148	B149	Anti_TPO	TGAb	0.231204	2.14E-13
B149	B228	TGAb	OS	0.495633	0
B15	B16	CD3-CD19+(B cell)	CD3+T	-0.68148	0
B15	B17	CD3-CD19+(B cell)	CD3+CD16+CD56+NK	-0.26043	0
B15	B20	CD3-CD19+(B cell)	CD3+CD4+CD8+	0.270452	0
B15	B22	CD3-CD19+(B cell)	CD3+CD8+(T)	0.177102	2.27E-08
B15	B23	CD3-CD19+(B cell)	CD3-CD16+CD56+(NK)	0.314987	0
B15	B79	CD3-CD19+(B cell)	hb V -dna	0.184882	5.23E-09
B15	B99	CD3-CD19+(B cell)	anti-PM-Scl	0.271389	0
B15	B120	CD3-CD19+(B cell)	HCV-cAg	0.268668	0
B15	B138	CD3-CD19+(B cell)	PAB	0.17187	5.89E-08
B15	B156	CD3-CD19+(B cell)	IgE	-0.40673	0
B15	B157	CD3-CD19+(B cell)	IgG	-0.31507	0
B15	B158	CD3-CD19+(B cell)	IgM	-0.18491	5.21E-09
B15	B178	CD3-CD19+(B cell)	Ig-U	0.236666	5.57E-14
B15	B179	CD3-CD19+(B cell)	α1-MG	0.218243	4.60E-12
B15	B180	CD3-CD19+(B cell)	Tf	0.224059	1.19E-12
B15	B181	CD3-CD19+(B cell)	APTT	-0.26043	0
B15	B182	CD3-CD19+(B cell)	TT	-0.25859	2.22E-16
B15	B183	CD3-CD19+(B cell)	PTA	-0.26044	0
B15	B184	CD3-CD19+(B cell)	INR	-0.26043	0
B15	B185	CD3-CD19+(B cell)	PT	-0.26033	0
B15	B186	CD3-CD19+(B cell)	PFC	0.260432	0
B15	B199	CD3-CD19+(B cell)	Mg	0.156325	8.42E-07
B150	B194	ALP	Cl	-0.16289	2.83E-07
B150	B200	ALP	OP	0.198711	3.28E-10
B151	B155	Anti-CCP	IgA	0.204536	9.60E-11
B151	B156	Anti-CCP	IgE	0.237949	4.04E-14
B151	B178	Anti-CCP	Ig-U	-0.4731	0
B151	B179	Anti-CCP	α1-MG	-0.36783	0
B151	B180	Anti-CCP	Tf	-0.4481	0
B151	B181	Anti-CCP	APTT	0.494682	0
B151	B182	Anti-CCP	TT	0.491607	0
B151	B183	Anti-CCP	PTA	0.49469	0
B151	B184	Anti-CCP	INR	0.494682	0

B151	B185	Anti-CCP	PT	0.49429	0
B151	B186	Anti-CCP	PFC	-0.49468	0
B151	B193	Anti-CCP	K	0.183491	6.84E-09
B151	B196	Anti-CCP	TCO2	0.206152	6.78E-11
B151	B197	Anti-CCP	AG	-0.39127	0
B151	B198	Anti-CCP	P	-0.17917	1.55E-08
B152	B177	ACA-Ig(G-A-M)	ACR	0.224473	1.08E-12
B152	B178	ACA-Ig(G-A-M)	Ig-U	-0.21827	4.57E-12
B152	B179	ACA-Ig(G-A-M)	α1-MG	-0.35067	0
B152	B180	ACA-Ig(G-A-M)	Tf	-0.23983	2.51E-14
B152	B195	ACA-Ig(G-A-M)	Na	-0.17134	6.47E-08
B152	B196	ACA-Ig(G-A-M)	TCO2	0.189847	1.98E-09
B152	B197	ACA-Ig(G-A-M)	AG	-0.17957	1.43E-08
B152	B200	ACA-Ig(G-A-M)	OP	-0.17289	4.90E-08
B152	B211	ACA-Ig(G-A-M)	IOP-R	-0.2388	3.24E-14
B152	B212	ACA-Ig(G-A-M)	IOP-L	-0.19711	4.57E-10
B153	B170	Glu	U-GLU	0.575713	0
B153	B200	Glu	OP	0.327728	0
B153	B206	Glu	Waist	0.21512	9.36E-12
B153	B213	Glu	CVA-R	0.221537	2.15E-12
B153	B215	Glu	BMI	0.159915	4.66E-07
B153	B220	Glu	SBP	0.231718	1.89E-13
B153	B222	Glu	NS	0.183985	6.22E-09
B154	B155	RF	IgA	0.430572	0
B154	B157	RF	IgG	0.572648	0
B154	B158	RF	IgM	0.184784	5.33E-09
B154	B161	RF	LEU	0.177772	2.01E-08
B155	B157	IgA	IgG	0.360284	0
B155	B158	IgA	IgM	0.168654	1.04E-07
B155	B178	IgA	Ig-U	-0.17746	2.13E-08
B155	B180	IgA	Tf	-0.19472	7.45E-10
B156	B158	IgE	IgM	-0.27827	0
B156	B175	IgE	ALB-U	-0.15735	7.13E-07
B156	B176	IgE	Ucr	-0.1792	1.54E-08
B156	B178	IgE	Ig-U	-0.49302	0
B156	B179	IgE	α1-MG	-0.45623	0
B156	B180	IgE	Tf	-0.45119	0
B156	B194	IgE	Cl	0.316797	0
B156	B197	IgE	AG	-0.27929	0
B157	B158	IgG	IgM	0.33764	0
B157	B181	IgG	APTT	0.161402	3.63E-07
B157	B182	IgG	TT	0.15879	5.62E-07
B157	B183	IgG	PTA	0.161451	3.60E-07
B157	B184	IgG	INR	0.161402	3.63E-07

B157	B185	IgG	PT	0.161995	3.28E-07
B157	B186	IgG	PFC	-0.1614	3.63E-07
B158	B178	IgM	Ig-U	0.15856	5.83E-07
B158	B181	IgM	APTT	0.23611	6.39E-14
B158	B182	IgM	TT	0.229355	3.36E-13
B158	B183	IgM	PTA	0.236266	6.17E-14
B158	B184	IgM	INR	0.23611	6.39E-14
B158	B185	IgM	PT	0.238295	3.71E-14
B158	B186	IgM	PFC	-0.23611	6.39E-14
B159	B169	β2-MG	U-CRY	1	0
B159	B195	β2-MG	Na	-0.19632	5.38E-10
B16	B17	CD3+T	CD3+CD16+CD56+NK	0.868628	0
B16	B18	CD3+T	CD3+CD4+helper(inducer)T	-0.46022	0
B16	B19	CD3+T	CD3+CD4+/CD3+CD8+	-0.42269	0
B16	B20	CD3+T	CD3+CD4+CD8+	0.27631	0
B16	B21	CD3+T	CD3+CD4-CD8-	0.402159	0
B16	B22	CD3+T	CD3+CD8+(T)	0.456233	0
B16	B23	CD3+T	CD3-CD16+CD56+(NK)	-0.18875	2.46E-09
B16	B24	CD3+T	αHBDH	0.177788	2.00E-08
B16	B56	CD3+T	IR	-0.15844	5.95E-07
B16	B95	CD3+T	anti-cenpb	0.237656	4.35E-14
B16	B97	CD3+T	dsDNA	0.237656	4.35E-14
B16	B98	CD3+T	anti-Jo-1	0.237656	4.35E-14
B16	B99	CD3+T	anti-PM-Scl	-0.87028	0
B16	B101	CD3+T	anti-Sc70	0.237656	4.35E-14
B16	B102	CD3+T	anti-Sm	0.237656	4.35E-14
B16	B105	CD3+T	anti-U1RNP	0.237656	4.35E-14
B16	B106	CD3+T	anti-P	0.237656	4.35E-14
B16	B107	CD3+T	AnuA	0.237656	4.35E-14
B16	B108	CD3+T	Anti-Cyclin	0.237656	4.35E-14
B16	B109	CD3+T	AMA-M2	0.237656	4.35E-14
B16	B110	CD3+T	AHA	0.237656	4.35E-14
B16	B151	CD3+T	Anti-CCP	0.428917	0
B16	B157	CD3+T	IgG	0.275495	0
B16	B158	CD3+T	IgM	0.318008	0
B16	B181	CD3+T	APTT	0.868628	0
B16	B182	CD3+T	TT	0.863656	0
B16	B183	CD3+T	PTA	0.86863	0
B16	B184	CD3+T	INR	0.868628	0
B16	B185	CD3+T	PT	0.867747	0
B16	B186	CD3+T	PFC	-0.86863	0
B16	B193	CD3+T	K	0.251283	1.33E-15
B16	B198	CD3+T	P	-0.30216	0
B16	B199	CD3+T	Mg	-0.25297	8.88E-16

B16	B204	CD3+T	Drinking	-0.18172	9.58E-09
B160	B192	PH	UA	-0.15825	6.14E-07
B161	B173	LEU	NTT	0.316902	0
B163	B166	SG	Pro	0.380664	0
B163	B176	SG	Ucr	0.275922	0
B163	B190	SG	Cre	0.195126	6.86E-10
B163	B191	SG	Urea	0.23807	3.91E-14
B163	B192	SG	UA	0.249288	2.22E-15
B163	B206	SG	Waist	0.229982	2.89E-13
B163	B209	SG	Smoking	0.192291	1.22E-09
B163	B210	SG	Hip	0.184912	5.20E-09
B163	B215	SG	BMI	0.161663	3.47E-07
B163	B216	SG	BMI	0.293824	0
B163	B221	SG	Hei	0.302459	0
B163	B222	SG	NS	0.321061	0
B168	B175	NMP22	ALB-U	0.202793	1.39E-10
B169	B195	U-CRY	Na	-0.19632	5.38E-10
B17	B18	CD3+CD16+CD56+NK	CD3+CD4+helper(inducer)T	-0.43712	0
B17	B19	CD3+CD16+CD56+NK	CD3+CD4+/CD3+CD8+	-0.48831	0
B17	B20	CD3+CD16+CD56+NK	CD3+CD4+CD8+	0.427657	0
B17	B21	CD3+CD16+CD56+NK	CD3+CD4-CD8-	0.568357	0
B17	B22	CD3+CD16+CD56+NK	CD3+CD8+(T)	0.582584	0
B17	B24	CD3+CD16+CD56+NK	αHBDH	0.2041	1.05E-10
B17	B56	CD3+CD16+CD56+NK	IR	-0.18264	8.04E-09
B17	B95	CD3+CD16+CD56+NK	anti-cenpb	0.331973	0
B17	B97	CD3+CD16+CD56+NK	dsDNA	0.331973	0
B17	B98	CD3+CD16+CD56+NK	anti-Jo-1	0.331973	0
B17	B99	CD3+CD16+CD56+NK	anti-PM-Scl	-0.9983	0
B17	B101	CD3+CD16+CD56+NK	anti-Sc70	0.331973	0
B17	B102	CD3+CD16+CD56+NK	anti-Sm	0.331973	0
B17	B105	CD3+CD16+CD56+NK	anti-U1RNP	0.331973	0
B17	B106	CD3+CD16+CD56+NK	anti-P	0.331973	0
B17	B107	CD3+CD16+CD56+NK	AnuA	0.331973	0
B17	B108	CD3+CD16+CD56+NK	Anti-Cyclin	0.331973	0
B17	B109	CD3+CD16+CD56+NK	AMA-M2	0.331973	0
B17	B110	CD3+CD16+CD56+NK	AHA	0.331973	0
B17	B146	CD3+CD16+CD56+NK	CysC	-0.17173	6.04E-08
B17	B151	CD3+CD16+CD56+NK	Anti-CCP	0.494682	0
B17	B157	CD3+CD16+CD56+NK	IgG	0.161402	3.63E-07
B17	B158	CD3+CD16+CD56+NK	IgM	0.23611	6.39E-14
B17	B181	CD3+CD16+CD56+NK	APTT	1	0
B17	B182	CD3+CD16+CD56+NK	TT	0.994483	0
B17	B183	CD3+CD16+CD56+NK	PTA	0.999995	0
B17	B185	CD3+CD16+CD56+NK	PT	0.998892	0

B17	B186	CD3+CD16+CD56+NK	PFC	-1	0
B17	B193	CD3+CD16+CD56+NK	K	0.283561	0
B17	B194	CD3+CD16+CD56+NK	Cl	-0.2028	1.39E-10
B17	B198	CD3+CD16+CD56+NK	P	-0.36229	0
B17	B199	CD3+CD16+CD56+NK	Mg	-0.26397	0
B17	B204	CD3+CD16+CD56+NK	Drinking	-0.1983	3.57E-10
B170	B172	U-GLU	KB	0.186013	4.20E-09
B170	B213	U-GLU	CVA-R	0.253468	6.66E-16
B173	B227	NTT	OD	0.194413	7.93E-10
B174	B190	Urea/Cr	Cre	-0.38659	0
B174	B191	Urea/Cr	Urea	0.568913	0
B174	B192	Urea/Cr	UA	-0.26117	0
B174	B206	Urea/Cr	Waist	-0.16993	8.33E-08
B174	B216	Urea/Cr	BMI	-0.26229	0
B174	B221	Urea/Cr	Hei	-0.33826	0
B174	B222	Urea/Cr	NS	-0.25307	8.88E-16
B175	B176	ALB-U	Ucr	0.355126	0
B175	B177	ALB-U	ACR	0.375797	0
B175	B178	ALB-U	Ig-U	0.219825	3.20E-12
B175	B179	ALB-U	α1-MG	0.215949	7.76E-12
B175	B180	ALB-U	Tf	0.218864	3.99E-12
B175	B194	ALB-U	Cl	-0.23602	6.55E-14
B176	B177	Ucr	ACR	-0.18201	9.07E-09
B176	B178	Ucr	Ig-U	0.228055	4.60E-13
B176	B179	Ucr	α1-MG	0.241709	1.55E-14
B176	B180	Ucr	Tf	0.222601	1.68E-12
B178	B179	Ig-U	α1-MG	0.965659	0
B178	B180	Ig-U	Tf	0.98973	0
B178	B194	Ig-U	Cl	-0.29488	0
B178	B197	Ig-U	AG	0.326446	0
B179	B180	α1-MG	Tf	0.973876	0
B179	B194	α1-MG	Cl	-0.2766	0
B179	B197	α1-MG	AG	0.322681	0
B18	B19	CD3+CD4+helper(inducer)T	CD3+CD4+/CD3+CD8+	0.98491	0
B18	B20	CD3+CD4+helper(inducer)T	CD3+CD4+CD8+	-0.70753	0
B18	B21	CD3+CD4+helper(inducer)T	CD3+CD4-CD8-	-0.67214	0
B18	B22	CD3+CD4+helper(inducer)T	CD3+CD8+(T)	-0.92668	0
B18	B23	CD3+CD4+helper(inducer)T	CD3-CD16+CD56+(NK)	0.242448	1.29E-14
B18	B79	CD3+CD4+helper(inducer)T	hb V -dna	-0.25866	2.22E-16
B18	B95	CD3+CD4+helper(inducer)T	anti-cenpb	-0.40527	0
B18	B97	CD3+CD4+helper(inducer)T	dsDNA	-0.40527	0
B18	B98	CD3+CD4+helper(inducer)T	anti-Jo-1	-0.40527	0
B18	B99	CD3+CD4+helper(inducer)T	anti-PM-Scl	0.420306	0
B18	B101	CD3+CD4+helper(inducer)T	anti-Sc70	-0.40527	0

B18	B102	CD3+CD4+helper(inducer)T	anti-Sm	-0.40527	0
B18	B105	CD3+CD4+helper(inducer)T	anti-U1RNP	-0.40527	0
B18	B106	CD3+CD4+helper(inducer)T	anti-P	-0.40527	0
B18	B107	CD3+CD4+helper(inducer)T	AnuA	-0.40527	0
B18	B108	CD3+CD4+helper(inducer)T	Anti-Cyclin	-0.40527	0
B18	B109	CD3+CD4+helper(inducer)T	AMA-M2	-0.40527	0
B18	B110	CD3+CD4+helper(inducer)T	AHA	-0.40527	0
B18	B138	CD3+CD4+helper(inducer)T	PAB	-0.21157	2.07E-11
B18	B151	CD3+CD4+helper(inducer)T	Anti-CCP	-0.21679	6.41E-12
B18	B156	CD3+CD4+helper(inducer)T	IgE	0.746105	0
B18	B158	CD3+CD4+helper(inducer)T	IgM	-0.4022	0
B18	B175	CD3+CD4+helper(inducer)T	ALB-U	-0.19897	3.10E-10
B18	B176	CD3+CD4+helper(inducer)T	Ucr	-0.1609	3.95E-07
B18	B178	CD3+CD4+helper(inducer)T	Ig-U	-0.33027	0
B18	B179	CD3+CD4+helper(inducer)T	α1-MG	-0.30473	0
B18	B180	CD3+CD4+helper(inducer)T	Tf	-0.31279	0
B18	B181	CD3+CD4+helper(inducer)T	APTT	-0.43712	0
B18	B182	CD3+CD4+helper(inducer)T	TT	-0.43483	0
B18	B183	CD3+CD4+helper(inducer)T	PTA	-0.43711	0
B18	B184	CD3+CD4+helper(inducer)T	INR	-0.43712	0
B18	B185	CD3+CD4+helper(inducer)T	PT	-0.43657	0
B18	B186	CD3+CD4+helper(inducer)T	PFC	0.437115	0
B18	B194	CD3+CD4+helper(inducer)T	Cl	0.241115	1.82E-14
B18	B198	CD3+CD4+helper(inducer)T	P	0.213027	1.50E-11
B180	B194	Tf	Cl	-0.28563	0
B180	B197	Tf	AG	0.352806	0
B181	B182	APTT	TT	0.994483	0
B181	B183	APTT	PTA	0.999995	0
B181	B184	APTT	INR	1	0
B181	B185	APTT	PT	0.998892	0
B181	B186	APTT	PFC	-1	0
B181	B193	APTT	K	0.283561	0
B181	B194	APTT	Cl	-0.2028	1.39E-10
B181	B198	APTT	P	-0.36229	0
B181	B199	APTT	Mg	-0.26397	0
B181	B204	APTT	Drinking	-0.1983	3.57E-10
B182	B183	TT	PTA	0.994163	0
B182	B184	TT	INR	0.994483	0
B182	B185	TT	PT	0.988446	0
B182	B186	TT	PFC	-0.99448	0
B182	B193	TT	K	0.274866	0
B182	B194	TT	Cl	-0.21348	1.35E-11
B182	B198	TT	P	-0.36067	0
B182	B199	TT	Mg	-0.26388	0

B182	B204	TT	Drinking	-0.19498	7.06E-10
B183	B184	PTA	INR	0.999995	0
B183	B185	PTA	PT	0.999029	0
B183	B186	PTA	PFC	-1	0
B183	B193	PTA	K	0.283764	0
B183	B194	PTA	Cl	-0.20246	1.49E-10
B183	B198	PTA	P	-0.36228	0
B183	B199	PTA	Mg	-0.26393	0
B183	B204	PTA	Drinking	-0.19836	3.52E-10
B184	B185	INR	PT	0.998892	0
B184	B186	INR	PFC	-1	0
B184	B193	INR	K	0.283561	0
B184	B194	INR	Cl	-0.2028	1.39E-10
B184	B198	INR	P	-0.36229	0
B184	B199	INR	Mg	-0.26397	0
B184	B204	INR	Drinking	-0.1983	3.57E-10
B185	B186	PT	PFC	-0.99889	0
B185	B193	PT	K	0.286445	0
B185	B194	PT	Cl	-0.19728	4.41E-10
B185	B198	PT	P	-0.36172	0
B185	B199	PT	Mg	-0.26306	0
B185	B204	PT	Drinking	-0.19908	3.04E-10
B186	B193	PFC	K	-0.28356	0
B186	B194	PFC	Cl	0.202798	1.39E-10
B186	B198	PFC	P	0.362289	0
B186	B199	PFC	Mg	0.263966	0
B186	B204	PFC	Drinking	0.198299	3.57E-10
B188	B196	Ca	TCO2	0.170335	7.74E-08
B189	B190	eGFB	Cre	-0.51868	0
B189	B191	eGFB	Urea	-0.37192	0
B189	B192	eGFB	UA	-0.19602	5.71E-10
B189	B220	eGFB	SBP	-0.33073	0
B19	B20	CD3+CD4+/CD3+CD8+	CD3+CD4+CD8+	-0.75088	0
B19	B21	CD3+CD4+/CD3+CD8+	CD3+CD4-CD8-	-0.72853	0
B19	B22	CD3+CD4+/CD3+CD8+	CD3+CD8+(T)	-0.96288	0
B19	B23	CD3+CD4+/CD3+CD8+	CD3-CD16+CD56+(NK)	0.182827	7.76E-09
B19	B79	CD3+CD4+/CD3+CD8+	hb V -dna	-0.27184	0
B19	B95	CD3+CD4+/CD3+CD8+	anti-cenpb	-0.43567	0
B19	B97	CD3+CD4+/CD3+CD8+	dsDNA	-0.43567	0
B19	B98	CD3+CD4+/CD3+CD8+	anti-Jo-1	-0.43567	0
B19	B99	CD3+CD4+/CD3+CD8+	anti-PM-Scl	0.470588	0
B19	B101	CD3+CD4+/CD3+CD8+	anti-Sc70	-0.43567	0
B19	B102	CD3+CD4+/CD3+CD8+	anti-Sm	-0.43567	0
B19	B105	CD3+CD4+/CD3+CD8+	anti-U1RNP	-0.43567	0

B19	B106	CD3+CD4+/CD3+CD8+	anti-P	-0.43567	0
B19	B107	CD3+CD4+/CD3+CD8+	AnuA	-0.43567	0
B19	B108	CD3+CD4+/CD3+CD8+	Anti-Cyclin	-0.43567	0
B19	B109	CD3+CD4+/CD3+CD8+	AMA-M2	-0.43567	0
B19	B110	CD3+CD4+/CD3+CD8+	AHA	-0.43567	0
B19	B138	CD3+CD4+/CD3+CD8+	PAB	-0.22136	2.24E-12
B19	B151	CD3+CD4+/CD3+CD8+	Anti-CCP	-0.24236	1.33E-14
B19	B156	CD3+CD4+/CD3+CD8+	IgE	0.770562	0
B19	B158	CD3+CD4+/CD3+CD8+	IgM	-0.37318	0
B19	B175	CD3+CD4+/CD3+CD8+	ALB-U	-0.21233	1.75E-11
B19	B176	CD3+CD4+/CD3+CD8+	Ucr	-0.17153	6.26E-08
B19	B178	CD3+CD4+/CD3+CD8+	Ig-U	-0.34733	0
B19	B179	CD3+CD4+/CD3+CD8+	α1-MG	-0.32043	0
B19	B180	CD3+CD4+/CD3+CD8+	Tf	-0.32892	0
B19	B181	CD3+CD4+/CD3+CD8+	APTT	-0.48831	0
B19	B182	CD3+CD4+/CD3+CD8+	TT	-0.48581	0
B19	B183	CD3+CD4+/CD3+CD8+	PTA	-0.48831	0
B19	B184	CD3+CD4+/CD3+CD8+	INR	-0.48831	0
B19	B185	CD3+CD4+/CD3+CD8+	PT	-0.48769	0
B19	B186	CD3+CD4+/CD3+CD8+	PFC	0.488313	0
B19	B194	CD3+CD4+/CD3+CD8+	Cl	0.261796	0
B19	B198	CD3+CD4+/CD3+CD8+	P	0.235604	7.26E-14
B190	B191	Cre	Urea	0.50424	0
B190	B192	Cre	UA	0.471329	0
B190	B204	Cre	Drinking	0.18294	7.59E-09
B190	B206	Cre	Waist	0.287713	0
B190	B209	Cre	Smoking	0.181742	9.54E-09
B190	B210	Cre	Hip	0.164916	2.00E-07
B190	B216	Cre	BMI	0.289705	0
B190	B221	Cre	Hei	0.341231	0
B190	B222	Cre	NS	0.380308	0
B191	B192	Urea	UA	0.202779	1.40E-10
B191	B220	Urea	SBP	0.163352	2.61E-07
B192	B204	UA	Drinking	0.235879	6.79E-14
B192	B206	UA	Waist	0.450732	0
B192	B209	UA	Smoking	0.16365	2.48E-07
B192	B210	UA	Hip	0.333237	0
B192	B215	UA	BMI	0.315932	0
B192	B216	UA	BMI	0.454795	0
B192	B218	UA	DBP	0.203104	1.30E-10
B192	B220	UA	SBP	0.159735	4.80E-07
B192	B221	UA	Hei	0.386722	0
B192	B222	UA	NS	0.504643	0
B194	B195	Cl	Na	0.406988	0

B194	B196	Cl	TCO2	-0.24427	7.99E-15
B194	B197	Cl	AG	-0.26825	0
B194	B228	Cl	OS	0.162854	2.84E-07
B195	B196	Na	TCO2	0.167158	1.35E-07
B195	B200	Na	OP	0.599878	0
B196	B197	TCO2	AG	-0.69588	0
B196	B198	TCO2	P	0.164909	2.00E-07
B196	B200	TCO2	OP	0.243745	9.33E-15
B198	B228	P	OS	-0.15563	9.44E-07
B2	B43	Age	MCV	0.159579	4.93E-07
B2	B46	Age	RBC Count	-0.18175	9.53E-09
B2	B47	Age	RDW	0.281922	0
B2	B52	Age	PLT/BPC	-0.18233	8.52E-09
B2	B77	Age	TC	0.158194	6.20E-07
B2	B118	Age	CEA1	0.192097	1.27E-09
B2	B126	Age	Alb	-0.3248	0
B2	B127	Age	A-G ratio	-0.19608	5.65E-10
B2	B141	Age	AST/ALT	0.164101	2.30E-07
B2	B153	Age	Glu	0.219247	3.65E-12
B2	B176	Age	Ucr	-0.18007	1.31E-08
B2	B189	Age	eGFB	-0.63196	0
B2	B190	Age	Cre	0.194015	8.59E-10
B2	B191	Age	Urea	0.256261	4.44E-16
B2	B201	Age	e-habits	0.195611	6.21E-10
B2	B206	Age	Waist	0.201243	1.93E-10
B2	B220	Age	SBP	0.511494	0
B2	B221	Age	Hei	-0.22789	4.79E-13
B20	B21	CD3+CD4+CD8+	CD3+CD4-CD8-	0.213121	1.47E-11
B20	B22	CD3+CD4+CD8+	CD3+CD8+(T)	0.88645	0
B20	B23	CD3+CD4+CD8+	CD3-CD16+CD56+(NK)	0.508941	0
B20	B79	CD3+CD4+CD8+	hb V -dna	0.184696	5.42E-09
B20	B95	CD3+CD4+CD8+	anti-cenpb	0.328412	0
B20	B97	CD3+CD4+CD8+	dsDNA	0.328412	0
B20	B98	CD3+CD4+CD8+	anti-Jo-1	0.328412	0
B20	B99	CD3+CD4+CD8+	anti-PM-Scl	-0.41542	0
B20	B101	CD3+CD4+CD8+	anti-Sc70	0.328412	0
B20	B102	CD3+CD4+CD8+	anti-Sm	0.328412	0
B20	B105	CD3+CD4+CD8+	anti-U1RNP	0.328412	0
B20	B106	CD3+CD4+CD8+	anti-P	0.328412	0
B20	B107	CD3+CD4+CD8+	AnuA	0.328412	0
B20	B108	CD3+CD4+CD8+	Anti-Cyclin	0.328412	0
B20	B109	CD3+CD4+CD8+	AMA-M2	0.328412	0
B20	B110	CD3+CD4+CD8+	AHA	0.328412	0
B20	B120	CD3+CD4+CD8+	HCV-cAg	0.23463	9.24E-14

B20	B138	CD3+CD4+CD8+	PAB	0.16432	2.21E-07
B20	B151	CD3+CD4+CD8+	Anti-CCP	0.213107	1.47E-11
B20	B156	CD3+CD4+CD8+	IgE	-0.51864	0
B20	B158	CD3+CD4+CD8+	IgM	0.220625	2.66E-12
B20	B178	CD3+CD4+CD8+	Ig-U	0.23702	5.11E-14
B20	B179	CD3+CD4+CD8+	α1-MG	0.218445	4.39E-12
B20	B180	CD3+CD4+CD8+	Tf	0.224313	1.12E-12
B20	B181	CD3+CD4+CD8+	APTT	0.427657	0
B20	B182	CD3+CD4+CD8+	TT	0.425658	0
B20	B183	CD3+CD4+CD8+	PTA	0.427644	0
B20	B184	CD3+CD4+CD8+	INR	0.427657	0
B20	B185	CD3+CD4+CD8+	PT	0.427021	0
B20	B186	CD3+CD4+CD8+	PFC	-0.42766	0
B204	B206	Drinking	Waist	0.276212	0
B204	B209	Drinking	Smoking	0.365854	0
B204	B210	Drinking	Hip	0.189203	2.25E-09
B204	B215	Drinking	BMI	0.185739	4.43E-09
B204	B216	Drinking	BMI	0.276259	0
B204	B221	Drinking	Hei	0.241138	1.80E-14
B204	B222	Drinking	NS	0.298335	0
B206	B209	Waist	Smoking	0.230872	2.33E-13
B206	B210	Waist	Hip	0.730068	0
B206	B215	Waist	BMI	0.82773	0
B206	B216	Waist	BMI	0.840225	0
B206	B218	Waist	DBP	0.246816	4.00E-15
B206	B220	Waist	SBP	0.276708	0
B206	B221	Waist	Hei	0.392198	0
B206	B222	Waist	NS	0.778052	0
B209	B216	Smoking	BMI	0.229599	3.17E-13
B209	B221	Smoking	Hei	0.249846	1.78E-15
B209	B222	Smoking	NS	0.289161	0
B21	B22	CD3+CD4-CD8-	CD3+CD8+(T)	0.618194	0
B21	B23	CD3+CD4-CD8-	CD3-CD16+CD56+(NK)	-0.59632	0
B21	B79	CD3+CD4-CD8-	hb V -dna	0.205615	7.61E-11
B21	B95	CD3+CD4-CD8-	anti-cenpb	0.396064	0
B21	B97	CD3+CD4-CD8-	dsDNA	0.396064	0
B21	B98	CD3+CD4-CD8-	anti-Jo-1	0.396064	0
B21	B99	CD3+CD4-CD8-	anti-PM-Scl	-0.55458	0
B21	B101	CD3+CD4-CD8-	anti-Sc70	0.396064	0
B21	B102	CD3+CD4-CD8-	anti-Sm	0.396064	0
B21	B105	CD3+CD4-CD8-	anti-U1RNP	0.396064	0
B21	B106	CD3+CD4-CD8-	anti-P	0.396064	0
B21	B107	CD3+CD4-CD8-	AnuA	0.396064	0
B21	B108	CD3+CD4-CD8-	Anti-Cyclin	0.396064	0

B21	B109	CD3+CD4-CD8-	AMA-M2	0.396064	0
B21	B110	CD3+CD4-CD8-	AHA	0.396064	0
B21	B151	CD3+CD4-CD8-	Anti-CCP	0.281253	0
B21	B156	CD3+CD4-CD8-	IgE	-0.57335	0
B21	B158	CD3+CD4-CD8-	IgM	0.264964	0
B21	B175	CD3+CD4-CD8-	ALB-U	0.188523	2.57E-09
B21	B178	CD3+CD4-CD8-	Ig-U	0.262465	0
B21	B179	CD3+CD4-CD8-	α1-MG	0.242188	1.38E-14
B21	B180	CD3+CD4-CD8-	Tf	0.248585	2.66E-15
B21	B181	CD3+CD4-CD8-	APTT	0.568357	0
B21	B182	CD3+CD4-CD8-	TT	0.565244	0
B21	B183	CD3+CD4-CD8-	PTA	0.568354	0
B21	B184	CD3+CD4-CD8-	INR	0.568357	0
B21	B185	CD3+CD4-CD8-	PT	0.567718	0
B21	B186	CD3+CD4-CD8-	PFC	-0.56836	0
B21	B193	CD3+CD4-CD8-	K	0.174499	3.66E-08
B21	B194	CD3+CD4-CD8-	Cl	-0.39671	0
B21	B197	CD3+CD4-CD8-	AG	0.206597	6.16E-11
B21	B198	CD3+CD4-CD8-	P	-0.32945	0
B210	B215	Hip	BMI	0.756217	0
B210	B216	Hip	BMI	0.768833	0
B210	B221	Hip	Hei	0.367737	0
B210	B222	Hip	NS	0.630005	0
B211	B212	IOP-R	IOP-L	0.826274	0
B214	B219	CVA-L	OT	0.166802	1.44E-07
B215	B216	BMI	BMI	0.82088	0
B215	B218	BMI	DBP	0.25985	0
B215	B220	BMI	SBP	0.254899	4.44E-16
B215	B222	BMI	NS	0.675684	0
B216	B218	BMI	DBP	0.24693	4.00E-15
B216	B221	BMI	Hei	0.675255	0
B216	B222	BMI	NS	0.820925	0
B218	B220	DBP	SBP	0.591999	0
B218	B222	DBP	NS	0.251425	1.33E-15
B22	B79	CD3+CD8+(T)	hb V -dna	0.242056	1.42E-14
B22	B95	CD3+CD8+(T)	anti-cenpb	0.437554	0
B22	B97	CD3+CD8+(T)	dsDNA	0.437554	0
B22	B98	CD3+CD8+(T)	anti-Jo-1	0.437554	0
B22	B99	CD3+CD8+(T)	anti-PM-Scl	-0.56652	0
B22	B101	CD3+CD8+(T)	anti-Sc70	0.437554	0
B22	B102	CD3+CD8+(T)	anti-Sm	0.437554	0
B22	B105	CD3+CD8+(T)	anti-U1RNP	0.437554	0
B22	B106	CD3+CD8+(T)	anti-P	0.437554	0
B22	B107	CD3+CD8+(T)	AnuA	0.437553	0

B22	B108	CD3+CD8+(T)	Anti-Cyclin	0.437554	0
B22	B109	CD3+CD8+(T)	AMA-M2	0.437554	0
B22	B110	CD3+CD8+(T)	AHA	0.437554	0
B22	B120	CD3+CD8+(T)	HCV-cAg	0.165354	1.85E-07
B22	B138	CD3+CD8+(T)	PAB	0.198081	3.74E-10
B22	B151	CD3+CD8+(T)	Anti-CCP	0.289299	0
B22	B156	CD3+CD8+(T)	IgE	-0.68871	0
B22	B158	CD3+CD8+(T)	IgM	0.337854	0
B22	B175	CD3+CD8+(T)	ALB-U	0.207555	5.00E-11
B22	B176	CD3+CD8+(T)	Ucr	0.166895	1.42E-07
B22	B178	CD3+CD8+(T)	Ig-U	0.309797	0
B22	B179	CD3+CD8+(T)	α1-MG	0.285693	0
B22	B180	CD3+CD8+(T)	Tf	0.293302	0
B22	B181	CD3+CD8+(T)	APTT	0.582584	0
B22	B182	CD3+CD8+(T)	TT	0.579627	0
B22	B183	CD3+CD8+(T)	PTA	0.582574	0
B22	B184	CD3+CD8+(T)	INR	0.582584	0
B22	B185	CD3+CD8+(T)	PT	0.581824	0
B22	B186	CD3+CD8+(T)	PFC	-0.58258	0
B22	B194	CD3+CD8+(T)	Cl	-0.20885	3.78E-11
B22	B198	CD3+CD8+(T)	P	-0.23615	6.35E-14
B220	B222	SBP	NS	0.200963	2.05E-10
B221	B222	Hei	NS	0.559134	0
B23	B75	CD3-CD16+CD56+(NK)	ApoA1/B	0.191126	1.54E-09
B23	B76	CD3-CD16+CD56+(NK)	ApoB	-0.19713	4.55E-10
B23	B120	CD3-CD16+CD56+(NK)	HCV-cAg	0.210065	2.89E-11
B23	B137	CD3-CD16+CD56+(NK)	AFU	-0.17825	1.84E-08
B23	B156	CD3-CD16+CD56+(NK)	IgE	0.207111	5.51E-11
B23	B158	CD3-CD16+CD56+(NK)	IgM	-0.17704	2.30E-08
B23	B194	CD3-CD16+CD56+(NK)	Cl	0.269282	0
B23	B197	CD3-CD16+CD56+(NK)	AG	-0.22991	2.94E-13
B24	B26	αHBDH	CKMB	0.499784	0
B24	B27	αHBDH	LDH	0.307643	0
B24	B55	αHBDH	TK	-0.2905	0
B24	B58	αHBDH	HCT	0.291852	0
B24	B61	αHBDH	WBHSRV	0.165554	1.79E-07
B24	B63	αHBDH	WBRSR(mPas_200)	0.310512	0
B24	B64	αHBDH	WBRSR(mPas_5)	0.291219	0
B24	B65	αHBDH	WBRSR(mPas_1)	0.265227	0
B24	B66	αHBDH	WBRSR(mPas_100)	0.176597	2.49E-08
B24	B67	αHBDH	WBRSR(mPas_50)	0.182331	8.53E-09
B24	B69	αHBDH	PV	0.2073	5.29E-11
B24	B75	αHBDH	ApoA1/B	0.164022	2.33E-07
B24	B99	αHBDH	anti-PM-Scl	-0.20087	2.09E-10

B24	B154	αHBDH	RF	0.408502	0
B24	B155	αHBDH	IgA	0.218012	4.85E-12
B24	B157	αHBDH	IgG	0.262655	0
B24	B181	αHBDH	APTT	0.2041	1.05E-10
B24	B182	αHBDH	TT	0.203193	1.28E-10
B24	B183	αHBDH	PTA	0.204092	1.06E-10
B24	B184	αHBDH	INR	0.2041	1.05E-10
B24	B185	αHBDH	PT	0.203775	1.13E-10
B24	B186	αHBDH	PFC	-0.2041	1.05E-10
B25	B26	CK	CKMB	0.39795	0
B25	B59	CK	WBRV	-0.17286	4.93E-08
B25	B123	CK	hs-CRP1	-0.19367	9.21E-10
B25	B124	CK	ACTH	0.325781	0
B25	B125	CK	Cor	0.325781	0
B25	B138	CK	PAB	0.165038	1.96E-07
B25	B154	CK	RF	-0.17054	7.47E-08
B25	B168	CK	NMP22	0.174902	3.40E-08
B26	B27	CKMB	LDH	0.176326	2.62E-08
B26	B55	CKMB	TK	-0.25657	4.44E-16
B26	B58	CKMB	HCT	0.247271	3.55E-15
B26	B61	CKMB	WBHSRV	0.216193	7.34E-12
B26	B63	CKMB	WBRSR(mPas_200)	0.335252	0
B26	B64	CKMB	WBRSR(mPas_5)	0.247472	3.55E-15
B26	B65	CKMB	WBRSR(mPas_1)	0.189014	2.34E-09
B26	B69	CKMB	PV	0.276487	0
B26	B78	CKMB	HBcAb	-0.18975	2.02E-09
B26	B95	CKMB	anti-cenpb	0.155429	9.74E-07
B26	B97	CKMB	dsDNA	0.155429	9.74E-07
B26	B98	CKMB	anti-Jo-1	0.155429	9.74E-07
B26	B101	CKMB	anti-Sc70	0.155429	9.74E-07
B26	B102	CKMB	anti-Sm	0.155429	9.74E-07
B26	B105	CKMB	anti-U1RNP	0.155429	9.74E-07
B26	B106	CKMB	anti-P	0.155429	9.74E-07
B26	B107	CKMB	AnuA	0.155429	9.74E-07
B26	B108	CKMB	Anti-Cyclin	0.155429	9.74E-07
B26	B109	CKMB	AMA-M2	0.155429	9.74E-07
B26	B110	CKMB	AHA	0.155429	9.74E-07
B26	B134	CKMB	CHE	-0.21222	1.79E-11
B26	B151	CKMB	Anti-CCP	-0.21549	8.62E-12
B26	B168	CKMB	NMP22	0.189556	2.10E-09
B27	B38	LDH	BAND%	0.617147	0
B27	B47	LDH	RDW	0.184635	5.49E-09
B27	B148	LDH	Anti_TPO	0.218283	4.56E-12
B28	B30	PRL	LH	-0.7792	0

B28	B31	PRL	FSH	-0.48213	0
B28	B32	PRL	Testo	-0.75579	0
B28	B33	PRL	Prog	-0.52949	0
B29	B30	E2	LH	-0.20972	3.12E-11
B29	B31	E2	FSH	0.322966	0
B29	B33	E2	Prog	0.235749	7.02E-14
B29	B137	E2	AFU	0.188885	2.40E-09
B29	B150	E2	ALP	0.160191	4.45E-07
B29	B198	E2	P	-0.22932	3.39E-13
B3	B58	Hcy	HCT	0.216049	7.59E-12
B3	B60	Hcy	WBRVI	0.175931	2.82E-08
B3	B62	Hcy	WBHSRVI	0.185579	4.57E-09
B3	B64	Hcy	WBRSR(mPas_5)	0.160181	4.46E-07
B3	B65	Hcy	WBRSR(mPas_1)	0.159096	5.34E-07
B3	B66	Hcy	WBRSR(mPas_100)	0.180963	1.11E-08
B3	B67	Hcy	WBRSR(mPas_50)	0.18364	6.64E-09
B3	B114	Hcy	TSH TT3 TT4(TT4)	0.155589	9.50E-07
B30	B31	LH	FSH	0.820986	0
B30	B32	LH	Testo	0.945916	0
B30	B33	LH	Prog	0.869631	0
B30	B95	LH	anti-cenpb	0.176145	2.71E-08
B30	B97	LH	dsDNA	0.176145	2.71E-08
B30	B98	LH	anti-Jo-1	0.176145	2.71E-08
B30	B101	LH	anti-Sc70	0.176145	2.71E-08
B30	B102	LH	anti-Sm	0.176145	2.71E-08
B30	B105	LH	anti-U1RNP	0.176145	2.71E-08
B30	B106	LH	anti-P	0.176145	2.71E-08
B30	B107	LH	AnuA	0.176145	2.71E-08
B30	B108	LH	Anti-Cyclin	0.176145	2.71E-08
B30	B109	LH	AMA-M2	0.176145	2.71E-08
B30	B110	LH	AHA	0.176145	2.71E-08
B30	B178	LH	Ig-U	0.225766	7.95E-13
B30	B179	LH	α1-MG	0.208132	4.41E-11
B30	B180	LH	Tf	0.213701	1.29E-11
B31	B32	FSH	Testo	0.934657	0
B31	B33	FSH	Prog	0.99557	0
B31	B95	FSH	anti-cenpb	0.198669	3.31E-10
B31	B97	FSH	dsDNA	0.198669	3.31E-10
B31	B98	FSH	anti-Jo-1	0.198669	3.31E-10
B31	B101	FSH	anti-Sc70	0.198669	3.31E-10
B31	B102	FSH	anti-Sm	0.198669	3.31E-10
B31	B105	FSH	anti-U1RNP	0.198669	3.31E-10
B31	B106	FSH	anti-P	0.198669	3.31E-10
B31	B107	FSH	AnuA	0.198669	3.31E-10

B31	B108	FSH	Anti-Cyclin	0.198669	3.31E-10
B31	B109	FSH	AMA-M2	0.198669	3.31E-10
B31	B110	FSH	AHA	0.198669	3.31E-10
B31	B178	FSH	Ig-U	0.254841	4.44E-16
B31	B179	FSH	α1-MG	0.234893	8.66E-14
B31	B180	FSH	Tf	0.241193	1.78E-14
B32	B33	Testo	Prog	0.955554	0
B32	B178	Testo	Ig-U	0.190223	1.84E-09
B32	B179	Testo	α1-MG	0.17526	3.19E-08
B32	B180	Testo	Tf	0.179988	1.33E-08
B33	B95	Prog	anti-cenpb	0.205529	7.75E-11
B33	B97	Prog	dsDNA	0.205529	7.75E-11
B33	B98	Prog	anti-Jo-1	0.205529	7.75E-11
B33	B101	Prog	anti-Sc70	0.205529	7.75E-11
B33	B102	Prog	anti-Sm	0.205529	7.75E-11
B33	B105	Prog	anti-U1RNP	0.205529	7.75E-11
B33	B106	Prog	anti-P	0.205529	7.75E-11
B33	B107	Prog	AnuA	0.205529	7.76E-11
B33	B108	Prog	Anti-Cyclin	0.205529	7.75E-11
B33	B109	Prog	AMA-M2	0.205529	7.75E-11
B33	B110	Prog	AHA	0.205529	7.75E-11
B33	B178	Prog	Ig-U	0.263554	0
B33	B179	Prog	α1-MG	0.242942	1.13E-14
B33	B180	Prog	Tf	0.249452	2.22E-15
B35	B92	β2-MG	ALDH2-Glu504Glu	0.730988	0
B35	B94	β2-MG	ALDH2-Lys504Lys	0.613636	0
B35	B96	β2-MG	dsDNA (IIF)	0.940127	0
B35	B116	β2-MG	TT3	0.837161	0
B35	B167	β2-MG	PC	0.46127	0
B37	B38	BAND	BAND%	0.63316	0
B38	B148	BAND%	Anti_TPO	0.168657	1.04E-07
B4	B7	WB-Ca	WB-Cop	1	0
B4	B28	WB-Ca	PRL	0.991516	0
B4	B30	WB-Ca	LH	-0.84269	0
B4	B31	WB-Ca	FSH	-0.59145	0
B4	B32	WB-Ca	Testo	-0.83448	0
B4	B33	WB-Ca	Prog	-0.63526	0
B40	B42	WBC	MON	0.45174	0
B40	B46	WBC	RBC Count	0.263793	0
B40	B48	WBC	LYM%	-0.22809	4.56E-13
B40	B51	WBC	Hb	0.161295	3.70E-07
B40	B52	WBC	PLT/BPC	0.272471	0
B40	B53	WBC	GR%	0.215081	9.44E-12
B40	B72	WBC	HDL-C	-0.19452	7.75E-10

B40	B192	WBC	UA	0.16159	3.52E-07
B40	B206	WBC	Waist	0.198571	3.38E-10
B40	B209	WBC	Smoking	0.198138	3.69E-10
B40	B215	WBC	BMI	0.18421	5.96E-09
B41	B42	MON %	MON	0.808279	0
B41	B53	MON %	GR%	-0.29607	0
B42	B192	MON	UA	0.181996	9.09E-09
B43	B44	MCV	MCH	0.929391	0
B43	B45	MCV	MCHC	0.333526	0
B43	B46	MCV	RBC Count	-0.53634	0
B43	B47	MCV	RDW	0.593605	0
B43	B51	MCV	Hb	0.235625	7.24E-14
B43	B52	MCV	PLT/BPC	-0.19096	1.59E-09
B44	B45	MCH	MCHC	0.65614	0
B44	B46	MCH	RBC Count	-0.41454	0
B44	B47	MCH	RDW	0.425203	0
B44	B51	MCH	Hb	0.40871	0
B44	B52	MCH	PLT/BPC	-0.17344	4.44E-08
B45	B51	MCHC	Hb	0.568577	0
B45	B71	MCHC	TG	0.181519	9.95E-09
B45	B126	MCHC	Alb	0.161066	3.84E-07
B45	B153	MCHC	Glu	0.156185	8.62E-07
B45	B192	MCHC	UA	0.181305	1.04E-08
B45	B206	MCHC	Waist	0.185377	4.75E-09
B45	B210	MCHC	Hip	0.158053	6.34E-07
B45	B216	MCHC	BMI	0.260427	0
B45	B221	MCHC	Hei	0.257321	2.22E-16
B45	B222	MCHC	NS	0.285474	0
B46	B47	RBC Count	RDW	-0.44193	0
B46	B51	RBC Count	Hb	0.652328	0
B46	B71	RBC Count	TG	0.164329	2.21E-07
B46	B72	RBC Count	HDL-C	-0.25094	1.33E-15
B46	B113	RBC Count	FT3	0.174439	3.70E-08
B46	B126	RBC Count	Alb	0.266646	0
B46	B127	RBC Count	A-G ratio	0.164363	2.20E-07
B46	B128	RBC Count	ALT	0.260605	0
B46	B140	RBC Count	r-GT	0.217416	5.56E-12
B46	B141	RBC Count	AST/ALT	-0.30043	0
B46	B163	RBC Count	SG	0.244014	8.44E-15
B46	B174	RBC Count	Urea/Cr	-0.25206	8.88E-16
B46	B190	RBC Count	Cre	0.218232	4.61E-12
B46	B192	RBC Count	UA	0.373959	0
B46	B206	RBC Count	Waist	0.335862	0
B46	B209	RBC Count	Smoking	0.189765	2.01E-09

B46	B210	RBC Count	Hip	0.231666	1.92E-13
B46	B215	RBC Count	BMI	0.248633	2.66E-15
B46	B216	RBC Count	BMI	0.39548	0
B46	B218	RBC Count	DBP	0.217319	5.68E-12
B46	B221	RBC Count	Hei	0.373874	0
B46	B222	RBC Count	NS	0.41626	0
B47	B52	RDW	PLT/BPC	-0.29523	0
B47	B126	RDW	Alb	-0.19528	6.64E-10
B47	B189	RDW	eGFB	-0.25436	4.44E-16
B48	B53	LYM%	GR%	-0.91991	0
B49	B50	baso%	eo%	0.269582	0
B49	B53	baso%	GR%	-0.17335	4.51E-08
B5	B6	WB-Mg	WB-Fe	-1	0
B5	B7	WB-Mg	WB-Cop	-1	0
B5	B28	WB-Mg	PRL	-0.99152	0
B5	B30	WB-Mg	LH	0.842692	0
B5	B31	WB-Mg	FSH	0.591453	0
B5	B32	WB-Mg	Testo	0.834483	0
B5	B33	WB-Mg	Prog	0.635264	0
B50	B53	eo%	GR%	-0.28746	0
B50	B209	eo%	Smoking	0.167596	1.25E-07
B51	B58	Hb	HCT	0.173433	4.44E-08
B51	B64	Hb	WBRSR(mPas_5)	0.167636	1.25E-07
B51	B65	Hb	WBRSR(mPas_1)	0.16824	1.12E-07
B51	B71	Hb	TG	0.234713	9.06E-14
B51	B72	Hb	HDL-C	-0.20101	2.03E-10
B51	B113	Hb	FT3	0.214672	1.04E-11
B51	B126	Hb	Alb	0.281614	0
B51	B127	Hb	A-G ratio	0.210046	2.90E-11
B51	B128	Hb	ALT	0.305759	0
B51	B138	Hb	PAB	0.155302	9.95E-07
B51	B140	Hb	r-GT	0.270003	0
B51	B141	Hb	AST/ALT	-0.28736	0
B51	B163	Hb	SG	0.288435	0
B51	B174	Hb	Urea/Cr	-0.31558	0
B51	B190	Hb	Cre	0.327012	0
B51	B192	Hb	UA	0.42431	0
B51	B204	Hb	Drinking	0.239895	2.49E-14
B51	B206	Hb	Waist	0.388224	0
B51	B209	Hb	Smoking	0.286431	0
B51	B210	Hb	Hip	0.260215	0
B51	B215	Hb	BMI	0.266715	0
B51	B216	Hb	BMI	0.476943	0
B51	B218	Hb	DBP	0.251293	1.33E-15

B51	B221	Hb	Hei	0.487677	0
B51	B222	Hb	NS	0.514167	0
B52	B129	PLT/BPC	IBIL	-0.20037	2.32E-10
B52	B132	PLT/BPC	Dbil	-0.21673	6.50E-12
B52	B133	PLT/BPC	TBIL	-0.21783	5.06E-12
B52	B189	PLT/BPC	eGFB	0.165376	1.84E-07
B52	B190	PLT/BPC	Cre	-0.16758	1.26E-07
B52	B191	PLT/BPC	Urea	-0.17518	3.24E-08
B54	B56	ESR	IR	-0.48872	0
B54	B57	ESR	RBCAI	-0.24705	4.00E-15
B54	B58	ESR	HCT	-0.53734	0
B54	B59	ESR	WBRV	-0.26347	0
B54	B60	ESR	WBRVI	-0.53597	0
B54	B61	ESR	WBHSRV	-0.45296	0
B54	B62	ESR	WBHSRVI	-0.57851	0
B54	B63	ESR	WBRSR(mPas_200)	-0.43781	0
B54	B64	ESR	WBRSR(mPas_5)	-0.48454	0
B54	B65	ESR	WBRSR(mPas_1)	-0.48085	0
B54	B66	ESR	WBRSR(mPas_100)	-0.48492	0
B54	B67	ESR	WBRSR(mPas_50)	-0.49435	0
B54	B73	ESR	ApoA	-0.19776	3.99E-10
B54	B78	ESR	HBcAb	0.180248	1.26E-08
B54	B123	ESR	hs-CRP1	0.257635	2.22E-16
B54	B138	ESR	PAB	-0.31623	0
B54	B153	ESR	Glu	0.174341	3.77E-08
B54	B155	ESR	IgA	0.193093	1.04E-09
B54	B200	ESR	OP	0.156531	8.14E-07
B55	B56	TK	IR	0.638554	0
B55	B58	TK	HCT	-0.41436	0
B55	B59	TK	WBRV	-0.24051	2.11E-14
B55	B62	TK	WBHSRVI	0.191153	1.53E-09
B55	B63	TK	WBRSR(mPas_200)	-0.48505	0
B55	B64	TK	WBRSR(mPas_5)	-0.49097	0
B55	B65	TK	WBRSR(mPas_1)	-0.46513	0
B55	B66	TK	WBRSR(mPas_100)	-0.18335	7.02E-09
B55	B67	TK	WBRSR(mPas_50)	-0.18682	3.59E-09
B55	B69	TK	PV	-0.68421	0
B55	B99	TK	anti-PM-Scl	0.159517	4.98E-07
B55	B134	TK	CHE	-0.23741	4.62E-14
B55	B151	TK	Anti-CCP	-0.28809	0
B55	B195	TK	Na	-0.15653	8.14E-07
B55	B200	TK	OP	-0.16914	9.57E-08
B56	B58	IR	HCT	0.426213	0
B56	B60	IR	WBRVI	0.675721	0

B56	B61	IR	WBHSRV	0.488544	0
B56	B62	IR	WBHSRVI	0.87503	0
B56	B63	IR	WBRSR(mPas_200)	0.188419	2.63E-09
B56	B64	IR	WBRSR(mPas_5)	0.202937	1.35E-10
B56	B65	IR	WBRSR(mPas_1)	0.196917	4.75E-10
B56	B66	IR	WBRSR(mPas_100)	0.549277	0
B56	B67	IR	WBRSR(mPas_50)	0.554074	0
B56	B69	IR	PV	-0.67794	0
B56	B99	IR	anti-PM-Scl	0.19261	1.14E-09
B56	B134	IR	CHE	-0.21816	4.69E-12
B56	B139	IR	ADA	0.179355	1.50E-08
B56	B151	IR	Anti-CCP	-0.39603	0
B56	B155	IR	IgA	-0.16833	1.10E-07
B56	B178	IR	Ig-U	0.194854	7.25E-10
B56	B180	IR	Tf	0.187183	3.35E-09
B56	B181	IR	APTT	-0.18264	8.04E-09
B56	B182	IR	TT	-0.18154	9.91E-09
B56	B183	IR	PTA	-0.18264	8.04E-09
B56	B184	IR	INR	-0.18264	8.04E-09
B56	B185	IR	PT	-0.18248	8.30E-09
B56	B186	IR	PFC	0.182639	8.04E-09
B56	B197	IR	AG	0.185423	4.71E-09
B56	B228	IR	OS	0.179299	1.51E-08
B57	B58	RBCAI	HCT	0.205959	7.07E-11
B57	B59	RBCAI	WBRV	0.703405	0
B57	B60	RBCAI	WBRVI	0.67358	0
B57	B61	RBCAI	WBHSRV	-0.17025	7.86E-08
B57	B62	RBCAI	WBHSRVI	0.182694	7.96E-09
B57	B64	RBCAI	WBRSR(mPas_5)	0.383675	0
B57	B65	RBCAI	WBRSR(mPas_1)	0.575485	0
B57	B66	RBCAI	WBRSR(mPas_100)	0.162186	3.18E-07
B57	B67	RBCAI	WBRSR(mPas_50)	0.222449	1.74E-12
B57	B95	RBCAI	anti-cenpb	-0.17305	4.77E-08
B57	B97	RBCAI	dsDNA	-0.17305	4.77E-08
B57	B98	RBCAI	anti-Jo-1	-0.17305	4.77E-08
B57	B101	RBCAI	anti-Sc70	-0.17305	4.77E-08
B57	B102	RBCAI	anti-Sm	-0.17305	4.77E-08
B57	B105	RBCAI	anti-U1RNP	-0.17305	4.77E-08
B57	B106	RBCAI	anti-P	-0.17304	4.77E-08
B57	B107	RBCAI	AnuA	-0.17304	4.77E-08
B57	B108	RBCAI	Anti-Cyclin	-0.17305	4.77E-08
B57	B109	RBCAI	AMA-M2	-0.17305	4.77E-08
B57	B110	RBCAI	AHA	-0.17305	4.77E-08
B57	B134	RBCAI	CHE	0.162487	3.02E-07

B57	B151	RBCAI	Anti-CCP	0.480729	0
B57	B156	RBCAI	IgE	0.163671	2.47E-07
B57	B165	RBCAI	bilirubin	0.467975	0
B57	B178	RBCAI	Ig-U	-0.25663	4.44E-16
B57	B179	RBCAI	α1-MG	-0.20839	4.17E-11
B57	B180	RBCAI	Tf	-0.23629	6.13E-14
B57	B194	RBCAI	Cl	0.238751	3.29E-14
B57	B197	RBCAI	AG	-0.17288	4.92E-08
B57	B198	RBCAI	P	-0.18639	3.91E-09
B57	B228	RBCAI	OS	0.358405	0
B58	B59	HCT	WBRV	0.162842	2.85E-07
B58	B60	HCT	WBRVI	0.718378	0
B58	B61	HCT	WBHSRV	0.510952	0
B58	B62	HCT	WBHSRVI	0.803503	0
B58	B63	HCT	WBRSR(mPas_200)	0.751168	0
B58	B64	HCT	WBRSR(mPas_5)	0.77024	0
B58	B65	HCT	WBRSR(mPas_1)	0.737146	0
B58	B66	HCT	WBRSR(mPas_100)	0.840656	0
B58	B67	HCT	WBRSR(mPas_50)	0.850548	0
B58	B78	HCT	HBcAb	-0.22632	6.97E-13
B58	B123	HCT	hs-CRP1	-0.25229	8.88E-16
B58	B137	HCT	AFU	0.202362	1.52E-10
B58	B138	HCT	PAB	0.321857	0
B58	B146	HCT	CysC	0.165261	1.88E-07
B58	B165	HCT	bilirubin	0.176585	2.50E-08
B58	B175	HCT	ALB-U	0.155482	9.66E-07
B58	B195	HCT	Na	0.183866	6.36E-09
B58	B200	HCT	OP	0.274134	0
B58	B228	HCT	OS	0.282227	0
B59	B60	WBRV	WBRVI	0.447326	0
B59	B61	WBRV	WBHSRV	0.387169	0
B59	B63	WBRV	WBRSR(mPas_200)	0.443899	0
B59	B64	WBRV	WBRSR(mPas_5)	0.699172	0
B59	B65	WBRV	WBRSR(mPas_1)	0.777997	0
B59	B66	WBRV	WBRSR(mPas_100)	0.333559	0
B59	B67	WBRV	WBRSR(mPas_50)	0.3732	0
B59	B69	WBRV	PV	0.430991	0
B59	B124	WBRV	ACTH	-0.15816	6.23E-07
B59	B125	WBRV	Cor	-0.15816	6.23E-07
B59	B139	WBRV	ADA	0.222563	1.69E-12
B59	B151	WBRV	Anti-CCP	0.38262	0
B59	B165	WBRV	bilirubin	0.34056	0
B59	B178	WBRV	Ig-U	-0.18237	8.46E-09
B59	B180	WBRV	Tf	-0.16377	2.43E-07

B59	B198	WBRV	P	-0.19208	1.27E-09
B59	B228	WBRV	OS	0.272052	0
B6	B7	WB-Fe	WB-Cop	1	0
B6	B28	WB-Fe	PRL	0.991516	0
B6	B30	WB-Fe	LH	-0.84269	0
B6	B31	WB-Fe	FSH	-0.59145	0
B6	B32	WB-Fe	Testo	-0.83448	0
B6	B33	WB-Fe	Prog	-0.63526	0
B60	B61	WBRVI	WBHSRV	0.348606	0
B60	B62	WBRVI	WBHSRVI	0.845692	0
B60	B63	WBRVI	WBRSR(mPas_200)	0.386234	0
B60	B64	WBRVI	WBRSR(mPas_5)	0.636648	0
B60	B65	WBRVI	WBRSR(mPas_1)	0.728849	0
B60	B66	WBRVI	WBRSR(mPas_100)	0.690821	0
B60	B67	WBRVI	WBRSR(mPas_50)	0.7332	0
B60	B69	WBRVI	PV	-0.41041	0
B60	B73	WBRVI	ApoA	0.172278	5.47E-08
B60	B123	WBRVI	hs-CRP1	-0.16887	1.00E-07
B60	B138	WBRVI	PAB	0.180128	1.29E-08
B60	B139	WBRVI	ADA	0.160697	4.09E-07
B60	B146	WBRVI	CysC	0.171467	6.33E-08
B60	B165	WBRVI	bilirubin	0.352208	0
B60	B200	WBRVI	OP	0.158151	6.24E-07
B60	B228	WBRVI	OS	0.452061	0
B61	B62	WBHSRV	WBHSRVI	0.595976	0
B61	B63	WBHSRV	WBRSR(mPas_200)	0.857028	0
B61	B64	WBHSRV	WBRSR(mPas_5)	0.725553	0
B61	B65	WBHSRV	WBRSR(mPas_1)	0.598066	0
B61	B66	WBHSRV	WBRSR(mPas_100)	0.754845	0
B61	B67	WBHSRV	WBRSR(mPas_50)	0.739909	0
B61	B69	WBHSRV	PV	0.288437	0
B61	B134	WBHSRV	CHE	-0.16228	3.13E-07
B61	B137	WBHSRV	AFU	0.23665	5.60E-14
B61	B139	WBHSRV	ADA	0.413968	0
B61	B151	WBHSRV	Anti-CCP	-0.27868	0
B61	B153	WBHSRV	Glu	0.198539	3.40E-10
B61	B178	WBHSRV	Ig-U	0.164957	1.98E-07
B61	B180	WBHSRV	Tf	0.157748	6.67E-07
B61	B194	WBHSRV	Cl	-0.25538	4.44E-16
B61	B200	WBHSRV	OP	0.215959	7.74E-12
B62	B63	WBHSRVI	WBRSR(mPas_200)	0.527692	0
B62	B64	WBHSRVI	WBRSR(mPas_5)	0.56514	0
B62	B65	WBHSRVI	WBRSR(mPas_1)	0.549805	0
B62	B66	WBHSRVI	WBRSR(mPas_100)	0.817151	0

B62	B67	WBHSRVI	WBRSR(mPas_50)	0.827887	0
B62	B69	WBHSRVI	PV	-0.45789	0
B62	B78	WBHSRVI	HBcAb	-0.19575	6.04E-10
B62	B123	WBHSRVI	hs-CRP1	-0.15896	5.46E-07
B62	B137	WBHSRVI	AFU	0.196296	5.40E-10
B62	B138	WBHSRVI	PAB	0.234479	9.59E-14
B62	B139	WBHSRVI	ADA	0.230163	2.76E-13
B62	B146	WBHSRVI	CysC	0.178623	1.71E-08
B62	B151	WBHSRVI	Anti-CCP	-0.31497	0
B62	B178	WBHSRVI	Ig-U	0.155462	9.69E-07
B62	B197	WBHSRVI	AG	0.183952	6.26E-09
B62	B200	WBHSRVI	OP	0.235132	8.17E-14
B62	B228	WBHSRVI	OS	0.303547	0
B63	B64	WBRSR(mPas_200)	WBRSR(mPas_5)	0.911195	0
B63	B65	WBRSR(mPas_200)	WBRSR(mPas_1)	0.799695	0
B63	B66	WBRSR(mPas_200)	WBRSR(mPas_100)	0.803022	0
B63	B67	WBRSR(mPas_200)	WBRSR(mPas_50)	0.796622	0
B63	B69	WBRSR(mPas_200)	PV	0.498608	0
B63	B78	WBRSR(mPas_200)	HBcAb	-0.17259	5.17E-08
B63	B137	WBRSR(mPas_200)	AFU	0.245758	5.33E-15
B63	B138	WBRSR(mPas_200)	PAB	0.18324	7.17E-09
B63	B139	WBRSR(mPas_200)	ADA	0.328778	0
B63	B153	WBRSR(mPas_200)	Glu	0.163718	2.45E-07
B63	B194	WBRSR(mPas_200)	Cl	-0.21689	6.26E-12
B63	B200	WBRSR(mPas_200)	OP	0.270855	0
B63	B228	WBRSR(mPas_200)	OS	0.163511	2.54E-07
B64	B65	WBRSR(mPas_5)	WBRSR(mPas_1)	0.975674	0
B64	B66	WBRSR(mPas_5)	WBRSR(mPas_100)	0.805302	0
B64	B67	WBRSR(mPas_5)	WBRSR(mPas_50)	0.825014	0
B64	B69	WBRSR(mPas_5)	PV	0.402682	0
B64	B137	WBRSR(mPas_5)	AFU	0.204298	1.01E-10
B64	B138	WBRSR(mPas_5)	PAB	0.16638	1.55E-07
B64	B139	WBRSR(mPas_5)	ADA	0.309133	0
B64	B165	WBRSR(mPas_5)	bilirubin	0.260312	0
B64	B200	WBRSR(mPas_5)	OP	0.245156	6.44E-15
B64	B228	WBRSR(mPas_5)	OS	0.316202	0
B65	B66	WBRSR(mPas_1)	WBRSR(mPas_100)	0.75314	0
B65	B67	WBRSR(mPas_1)	WBRSR(mPas_50)	0.785242	0
B65	B69	WBRSR(mPas_1)	PV	0.315035	0
B65	B137	WBRSR(mPas_1)	AFU	0.162276	3.13E-07
B65	B139	WBRSR(mPas_1)	ADA	0.266234	0
B65	B151	WBRSR(mPas_1)	Anti-CCP	0.157704	6.72E-07
B65	B165	WBRSR(mPas_1)	bilirubin	0.344616	0
B65	B200	WBRSR(mPas_1)	OP	0.207309	5.28E-11

B65	B228	WBRSR(mPas_1)	OS	0.375248	0
B66	B67	WBRSR(mPas_100)	WBRSR(mPas_50)	0.997475	0
B66	B78	WBRSR(mPas_100)	HBcAb	-0.18953	2.11E-09
B66	B137	WBRSR(mPas_100)	AFU	0.311448	0
B66	B138	WBRSR(mPas_100)	PAB	0.206292	6.58E-11
B66	B139	WBRSR(mPas_100)	ADA	0.431549	0
B66	B153	WBRSR(mPas_100)	Glu	0.220261	2.89E-12
B66	B194	WBRSR(mPas_100)	Cl	-0.20713	5.48E-11
B66	B200	WBRSR(mPas_100)	OP	0.351182	0
B66	B228	WBRSR(mPas_100)	OS	0.238814	3.24E-14
B67	B78	WBRSR(mPas_50)	HBcAb	-0.18109	1.08E-08
B67	B137	WBRSR(mPas_50)	AFU	0.301093	0
B67	B138	WBRSR(mPas_50)	PAB	0.203402	1.22E-10
B67	B139	WBRSR(mPas_50)	ADA	0.425038	0
B67	B153	WBRSR(mPas_50)	Glu	0.211123	2.29E-11
B67	B165	WBRSR(mPas_50)	bilirubin	0.164538	2.13E-07
B67	B194	WBRSR(mPas_50)	Cl	-0.19282	1.09E-09
B67	B200	WBRSR(mPas_50)	OP	0.34289	0
B67	B228	WBRSR(mPas_50)	OS	0.267575	0
B69	B115	PV	TSH TT3 TT4(TT3)	0.165203	1.90E-07
B69	B151	PV	Anti-CCP	0.177477	2.12E-08
B7	B8	WB-Cop	WB-Zinc	1	0
B7	B28	WB-Cop	PRL	0.991516	0
B7	B30	WB-Cop	LH	-0.84269	0
B7	B31	WB-Cop	FSH	-0.59145	0
B7	B32	WB-Cop	Testo	-0.83448	0
B7	B33	WB-Cop	Prog	-0.63526	0
B70	B76	LDL-C	ApoB	0.185226	4.90E-09
B70	B77	LDL-C	TC	0.858077	0
B70	B132	LDL-C	Dbil	-0.21757	5.37E-12
B71	B72	TG	HDL-C	-0.41343	0
B71	B77	TG	TC	0.255079	4.44E-16
B71	B91	TG	TP	0.289772	0
B71	B128	TG	ALT	0.186157	4.09E-09
B71	B130	TG	GLB	0.213522	1.34E-11
B71	B140	TG	r-GT	0.20896	3.68E-11
B71	B141	TG	AST/ALT	-0.17797	1.94E-08
B71	B153	TG	Glu	0.162277	3.13E-07
B71	B192	TG	UA	0.231908	1.81E-13
B71	B206	TG	Waist	0.262616	0
B71	B210	TG	Hip	0.159067	5.36E-07
B71	B215	TG	BMI	0.239496	2.73E-14
B71	B216	TG	BMI	0.255438	4.44E-16
B71	B218	TG	DBP	0.192698	1.12E-09

B71	B222	TG	NS	0.23114	2.18E-13
B72	B74	HDL-C	ApoA1	0.206127	6.82E-11
B72	B77	HDL-C	TC	0.243012	1.11E-14
B72	B128	HDL-C	ALT	-0.2498	1.78E-15
B72	B140	HDL-C	r-GT	-0.21741	5.57E-12
B72	B141	HDL-C	AST/ALT	0.292891	0
B72	B163	HDL-C	SG	-0.16849	1.07E-07
B72	B190	HDL-C	Cre	-0.17455	3.63E-08
B72	B192	HDL-C	UA	-0.34774	0
B72	B206	HDL-C	Waist	-0.32867	0
B72	B209	HDL-C	Smoking	-0.1704	7.65E-08
B72	B210	HDL-C	Hip	-0.24359	9.55E-15
B72	B215	HDL-C	BMI	-0.31149	0
B72	B216	HDL-C	BMI	-0.35137	0
B72	B221	HDL-C	Hei	-0.21381	1.26E-11
B72	B222	HDL-C	NS	-0.33561	0
B73	B76	ApoA	ApoB	0.183858	6.37E-09
B73	B124	ApoA	ACTH	0.27259	0
B73	B125	ApoA	Cor	0.27259	0
B73	B138	ApoA	PAB	0.169019	9.77E-08
B73	B180	ApoA	Tf	0.155423	9.75E-07
B73	B196	ApoA	TCO2	0.184564	5.56E-09
B74	B75	ApoA1	ApoA1/B	0.589474	0
B74	B78	ApoA1	HBcAb	-0.16355	2.52E-07
B74	B123	ApoA1	hs-CRP1	-0.18111	1.07E-08
B74	B124	ApoA1	ACTH	-0.16503	1.96E-07
B74	B125	ApoA1	Cor	-0.16503	1.96E-07
B74	B136	ApoA1	5'NT	-0.15615	8.67E-07
B74	B139	ApoA1	ADA	-0.18833	2.67E-09
B74	B195	ApoA1	Na	0.208147	4.40E-11
B75	B76	ApoA1/B	ApoB	-0.74071	0
B75	B124	ApoA1/B	ACTH	-0.16468	2.08E-07
B75	B125	ApoA1/B	Cor	-0.16468	2.08E-07
B75	B138	ApoA1/B	PAB	-0.2266	6.51E-13
B76	B77	ApoB	TC	0.213138	1.46E-11
B76	B138	ApoB	PAB	0.404497	0
B76	B139	ApoB	ADA	-0.15664	8.00E-07
B77	B132	TC	Dbil	-0.19753	4.19E-10
B77	B218	TC	DBP	0.159975	4.61E-07
B78	B123	HBcAb	hs-CRP1	0.169737	8.61E-08
B78	B134	HBcAb	CHE	0.238824	3.24E-14
B78	B138	HBcAb	PAB	-0.18571	4.46E-09
B78	B175	HBcAb	ALB-U	-0.2974	0
B78	B193	HBcAb	K	-0.19911	3.02E-10

B79	B95	hb V -dna	anti-cenpb	0.215035	9.54E-12
B79	B97	hb V -dna	dsDNA	0.215035	9.54E-12
B79	B98	hb V -dna	anti-Jo-1	0.215035	9.54E-12
B79	B101	hb V -dna	anti-Sc70	0.215035	9.54E-12
B79	B102	hb V -dna	anti-Sm	0.215035	9.54E-12
B79	B105	hb V -dna	anti-U1RNP	0.215035	9.54E-12
B79	B106	hb V -dna	anti-P	0.215035	9.54E-12
B79	B107	hb V -dna	AnuA	0.215035	9.54E-12
B79	B108	hb V -dna	Anti-Cyclin	0.215035	9.54E-12
B79	B109	hb V -dna	AMA-M2	0.215035	9.54E-12
B79	B110	hb V -dna	AHA	0.215035	9.54E-12
B79	B114	hb V -dna	TSH TT3 TT4(TT4)	0.172125	5.63E-08
B79	B115	hb V -dna	TSH TT3 TT4(TT3)	0.155324	9.91E-07
B79	B119	hb V -dna	Hcv-rna	0.21504	9.53E-12
B79	B120	hb V -dna	HCV-cAg	0.240362	2.20E-14
B79	B156	hb V -dna	IgE	-0.29079	0
B79	B178	hb V -dna	Ig-U	0.226782	6.24E-13
B79	B179	hb V -dna	α1-MG	0.263076	0
B79	B180	hb V -dna	Tf	0.258	2.22E-16
B8	B28	WB-Zinc	PRL	0.991516	0
B8	B30	WB-Zinc	LH	-0.84269	0
B8	B31	WB-Zinc	FSH	-0.59145	0
B8	B32	WB-Zinc	Testo	-0.83448	0
B8	B33	WB-Zinc	Prog	-0.63526	0
B82	B83	CEA	AFP	0.373548	0
B82	B87	CEA	NSE	0.317378	0
B82	B88	CEA	CA 242	0.389121	0
B82	B89	CEA	CA 72-4	0.221963	1.95E-12
B83	B84	AFP	C-PSA	0.3166	0
B83	B87	AFP	NSE	0.382715	0
B83	B88	AFP	CA 242	0.197765	3.99E-10
B83	B89	AFP	CA 72-4	0.423413	0
B83	B103	AFP	anti-SSA/Ro	-0.1565	8.19E-07
B83	B104	AFP	anti-SSB	-0.15939	5.09E-07
B83	B152	AFP	ACA-Ig(G-A-M)	0.306304	0
B84	B85	C-PSA	SCC	0.474324	0
B84	B89	C-PSA	CA 72-4	0.166948	1.40E-07
B84	B90	C-PSA	CYFRA 21-1	0.24454	7.55E-15
B84	B112	C-PSA	FT4	0.156484	8.21E-07
B85	B87	SCC	NSE	0.192159	1.25E-09
B85	B88	SCC	CA 242	0.606969	0
B86	B90	TBhCG	CYFRA 21-1	0.459366	0
B87	B88	NSE	CA 242	0.366829	0
B87	B89	NSE	CA 72-4	0.308775	0

B87	B138	NSE	PAB	-0.15756	6.88E-07
B87	B139	NSE	ADA	0.194811	7.31E-10
B87	B150	NSE	ALP	0.208125	4.42E-11
B87	B196	NSE	TCO2	0.232135	1.71E-13
B89	B193	CA 72-4	K	0.164057	2.31E-07
B89	B199	CA 72-4	Mg	-0.20145	1.85E-10
B9	B223	RH	FH	-0.40109	0
B91	B126	TP	Alb	0.378881	0
B91	B127	TP	A-G ratio	-0.55483	0
B91	B130	TP	GLB	0.82584	0
B91	B188	TP	Ca	0.225773	7.94E-13
B92	B94	ALDH2-Glu504Glu	ALDH2-Lys504Lys	0.536323	0
B92	B96	ALDH2-Glu504Glu	dsDNA (IIF)	-0.27468	0
B92	B116	ALDH2-Glu504Glu	TT3	0.633866	0
B92	B202	ALDH2-Glu504Glu	e-times	0.938612	0
B93	B94	ALDH2-Glu504Lys	ALDH2-Lys504Lys	0.938466	0
B93	B116	ALDH2-Glu504Lys	TT3	0.525765	0
B93	B167	ALDH2-Glu504Lys	PC	0.786527	0
B94	B96	ALDH2-Lys504Lys	dsDNA (IIF)	0.745386	0
B94	B167	ALDH2-Lys504Lys	PC	0.762338	0
B95	B97	anti-cenpb	dsDNA	1	0
B95	B99	anti-cenpb	anti-PM-Scl	-0.27646	0
B95	B100	anti-cenpb	anti-Ro52	-0.34578	0
B95	B103	anti-cenpb	anti-SSA/Ro	-0.27591	0
B95	B104	anti-cenpb	anti-SSB	-0.32367	0
B95	B105	anti-cenpb	anti-U1RNP	1	0
B95	B107	anti-cenpb	AnuA	1	0
B95	B108	anti-cenpb	Anti-Cyclin	1	0
B95	B109	anti-cenpb	AMA-M2	1	0
B95	B110	anti-cenpb	AHA	1	0
B95	B134	anti-cenpb	CHE	-0.29695	0
B95	B151	anti-cenpb	Anti-CCP	-0.23405	1.07E-13
B95	B155	anti-cenpb	IgA	-0.17398	4.03E-08
B95	B156	anti-cenpb	IgE	-0.42651	0
B95	B157	anti-cenpb	IgG	-0.21397	1.21E-11
B95	B175	anti-cenpb	ALB-U	0.22405	1.19E-12
B95	B176	anti-cenpb	Ucr	0.200525	2.24E-10
B95	B178	anti-cenpb	Ig-U	0.771362	0
B95	B179	anti-cenpb	α1-MG	0.719949	0
B95	B180	anti-cenpb	Tf	0.779882	0
B95	B181	anti-cenpb	APTT	0.331973	0
B95	B182	anti-cenpb	TT	0.330901	0
B95	B183	anti-cenpb	PTA	0.331949	0
B95	B184	anti-cenpb	INR	0.331973	0

B95	B185	anti-cenpb	PT	0.331264	0
B95	B186	anti-cenpb	PFC	-0.33197	0
B95	B194	anti-cenpb	CI	-0.33683	0
B95	B197	anti-cenpb	AG	0.254711	4.44E-16
B96	B116	dsDNA (IIF)	TT3	0.97619	0
B97	B98	dsDNA	anti-Jo-1	1	0
B97	B99	dsDNA	anti-PM-Scl	-0.27646	0
B97	B100	dsDNA	anti-Ro52	-0.34578	0
B97	B102	dsDNA	anti-Sm	1	0
B97	B103	dsDNA	anti-SSA/Ro	-0.27591	0
B97	B104	dsDNA	anti-SSB	-0.32367	0
B97	B105	dsDNA	anti-U1RNP	1	0
B97	B106	dsDNA	anti-P	1	0
B97	B107	dsDNA	AnuA	1	0
B97	B108	dsDNA	Anti-Cyclin	1	0
B97	B109	dsDNA	AMA-M2	1	0
B97	B110	dsDNA	AHA	1	0
B97	B134	dsDNA	CHE	-0.29695	0
B97	B151	dsDNA	Anti-CCP	-0.23405	1.07E-13
B97	B155	dsDNA	IgA	-0.17398	4.03E-08
B97	B156	dsDNA	IgE	-0.42651	0
B97	B157	dsDNA	IgG	-0.21397	1.21E-11
B97	B175	dsDNA	ALB-U	0.22405	1.19E-12
B97	B176	dsDNA	Ucr	0.200525	2.24E-10
B97	B178	dsDNA	Ig-U	0.771362	0
B97	B179	dsDNA	α1-MG	0.719949	0
B97	B180	dsDNA	Tf	0.779882	0
B97	B181	dsDNA	APTT	0.331973	0
B97	B182	dsDNA	TT	0.330901	0
B97	B183	dsDNA	PTA	0.331949	0
B97	B184	dsDNA	INR	0.331973	0
B97	B185	dsDNA	PT	0.331264	0
B97	B186	dsDNA	PFC	-0.33197	0
B97	B194	dsDNA	CI	-0.33683	0
B97	B197	dsDNA	AG	0.254711	4.44E-16
B98	B99	anti-Jo-1	anti-PM-Scl	-0.27646	0
B98	B100	anti-Jo-1	anti-Ro52	-0.34578	0
B98	B101	anti-Jo-1	anti-Sc70	1	0
B98	B102	anti-Jo-1	anti-Sm	1	0
B98	B103	anti-Jo-1	anti-SSA/Ro	-0.27591	0
B98	B104	anti-Jo-1	anti-SSB	-0.32367	0
B98	B107	anti-Jo-1	AnuA	1	0
B98	B108	anti-Jo-1	Anti-Cyclin	1	0
B98	B134	anti-Jo-1	CHE	-0.29695	0

B98	B151	anti-Jo-1	Anti-CCP	-0.23405	1.07E-13
B98	B155	anti-Jo-1	IgA	-0.17398	4.03E-08
B98	B156	anti-Jo-1	IgE	-0.42651	0
B98	B157	anti-Jo-1	IgG	-0.21397	1.21E-11
B98	B175	anti-Jo-1	ALB-U	0.22405	1.19E-12
B98	B176	anti-Jo-1	Ucr	0.200525	2.24E-10
B98	B178	anti-Jo-1	Ig-U	0.771362	0
B98	B179	anti-Jo-1	α1-MG	0.719949	0
B98	B180	anti-Jo-1	Tf	0.779882	0
B98	B181	anti-Jo-1	APTT	0.331973	0
B98	B182	anti-Jo-1	TT	0.330901	0
B98	B183	anti-Jo-1	PTA	0.331949	0
B98	B184	anti-Jo-1	INR	0.331973	0
B98	B185	anti-Jo-1	PT	0.331264	0
B98	B186	anti-Jo-1	PFC	-0.33197	0
B98	B194	anti-Jo-1	Cl	-0.33683	0
B98	B197	anti-Jo-1	AG	0.254711	4.44E-16
B99	B101	anti-PM-Scl	anti-Sc70	-0.27646	0
B99	B102	anti-PM-Scl	anti-Sm	-0.27646	0
B99	B105	anti-PM-Scl	anti-U1RNP	-0.27646	0
B99	B106	anti-PM-Scl	anti-P	-0.27646	0
B99	B107	anti-PM-Scl	AnuA	-0.27646	0
B99	B108	anti-PM-Scl	Anti-Cyclin	-0.27646	0
B99	B109	anti-PM-Scl	AMA-M2	-0.27646	0
B99	B110	anti-PM-Scl	AHA	-0.27646	0
B99	B146	anti-PM-Scl	CysC	0.174585	3.61E-08
B99	B151	anti-PM-Scl	Anti-CCP	-0.51844	0
B99	B157	anti-PM-Scl	IgG	-0.17765	2.05E-08
B99	B158	anti-PM-Scl	IgM	-0.23263	1.51E-13
B99	B181	anti-PM-Scl	APTT	-0.9983	0
B99	B182	anti-PM-Scl	TT	-0.99275	0
B99	B183	anti-PM-Scl	PTA	-0.9983	0
B99	B184	anti-PM-Scl	INR	-0.9983	0
B99	B185	anti-PM-Scl	PT	-0.99722	0
B99	B186	anti-PM-Scl	PFC	0.998302	0
B99	B193	anti-PM-Scl	K	-0.2897	0
B99	B194	anti-PM-Scl	Cl	0.185809	4.37E-09
B99	B198	anti-PM-Scl	P	0.364908	0
B99	B199	anti-PM-Scl	Mg	0.264999	0
B99	B204	anti-PM-Scl	Drinking	0.200102	2.45E-10

Supplementary Table 3. PEI Correlations in Hypertension

Row	Column	Row	Column	Cor	*P*
B1	B111	Gender	TSH	0.071717	0
B1	B113	Gender	FT3	-0.0512	0
B1	B114	Gender	TSH TT3 TT4(TT4)	0.052299	0
B1	B126	Gender	Alb	-0.07375	0
B1	B127	Gender	A-G ratio	-0.1529	0
B1	B128	Gender	ALT	-0.15361	0
B1	B129	Gender	IBIL	-0.13299	0
B1	B130	Gender	GLB	0.138351	0
B1	B131	Gender	AST	-0.04977	0
B1	B132	Gender	Dbil	-0.1529	0
B1	B133	Gender	TBIL	-0.14844	0
B1	B138	Gender	PAB	-0.08218	0
B1	B139	Gender	ADA	0.03961	0
B1	B140	Gender	r-GT	-0.14838	0
B1	B141	Gender	AST/ALT	0.192708	0
B1	B146	Gender	CysC	-0.04363	0
B1	B148	Gender	Anti_TPO	0.065245	0
B1	B149	Gender	TGAb	0.056628	0
B1	B153	Gender	Glu	-0.04835	0
B1	B160	Gender	PH	0.065417	0
B1	B161	Gender	LEU	0.206085	0
B1	B163	Gender	SG	-0.38918	0
B1	B166	Gender	Pro	-0.1573	0
B1	B170	Gender	U-GLU	-0.05839	0
B1	B173	Gender	NTT	0.118155	0
B1	B174	Gender	Urea/Cr	0.411039	0
B1	B176	Gender	Ucr	-0.14529	0
B1	B179	Gender	α1-MG	-0.04096	0
B1	B188	Gender	Ca	0.063179	0
B1	B190	Gender	Cre	-0.32238	0
B1	B191	Gender	Urea	-0.06407	0
B1	B192	Gender	UA	-0.40615	0
B1	B198	Gender	P	0.084699	0
B1	B2	Gender	Age	0.124492	0
B1	B203	Gender	DH	-0.04512	0
B1	B204	Gender	Drinking	-0.32752	0
B1	B205	Gender	KDA	0.071334	0
B1	B206	Gender	Waist	-0.3719	0
B1	B209	Gender	Smoking	-0.33436	0
B1	B210	Gender	Hip	-0.24997	0
B1	B215	Gender	BMI	-0.16036	0

B1	B216	Gender	BMI	-0.52624	0
B1	B218	Gender	DBP	-0.16885	0
B1	B219	Gender	OT	0.139931	0
B1	B220	Gender	SBP	0.058115	0
B1	B221	Gender	Hei	-0.6861	0
B1	B222	Gender	NS	-0.6465	0
B1	B24	Gender	αHBDH	0.051592	0
B1	B25	Gender	CK	-0.04166	0
B1	B27	Gender	LDH	0.046952	0
B1	B3	Gender	Hcy	-0.11243	0
B1	B40	Gender	WBC	-0.14967	0
B1	B42	Gender	MON	-0.24501	0
B1	B44	Gender	MCH	-0.1555	0
B1	B45	Gender	MCHC	-0.28992	0
B1	B46	Gender	RBC Count	-0.41655	0
B1	B48	Gender	LYM%	0.123838	0
B1	B50	Gender	eo%	-0.11995	0
B1	B51	Gender	Hb	-0.54237	0
B1	B52	Gender	PLT/BPC	0.100778	0
B1	B53	Gender	GR%	-0.04932	0
B1	B54	Gender	ESR	0.07864	0
B1	B56	Gender	IR	-0.06884	0
B1	B58	Gender	HCT	-0.06309	0
B1	B60	Gender	WBRVI	-0.10287	0
B1	B61	Gender	WBHSRV	-0.0603	0
B1	B62	Gender	WBHSRVI	-0.10874	0
B1	B63	Gender	WBRSR(mPas_200)	-0.09821	0
B1	B64	Gender	WBRSR(mPas_5)	-0.09895	0
B1	B65	Gender	WBRSR(mPas_1)	-0.09482	0
B1	B66	Gender	WBRSR(mPas_100)	-0.09738	0
B1	B67	Gender	WBRSR(mPas_50)	-0.09846	0
B1	B70	Gender	LDL-C	0.071795	0
B1	B71	Gender	TG	-0.08957	0
B1	B72	Gender	HDL-C	0.313334	0
B1	B74	Gender	ApoA1	0.123934	0
B1	B75	Gender	ApoA1/B	0.051883	0
B1	B77	Gender	TC	0.149732	0
B1	B84	Gender	C-PSA	-0.04196	0
B1	B86	Gender	TBhCG	0.051205	0
B1	B91	Gender	TP	0.081199	0
B1	B157	Gender	IgG	0.037115	8.88E-16
B1	B134	Gender	CHE	-0.03668	1.78E-15
B1	B49	Gender	baso%	-0.0366	1.78E-15
B1	B59	Gender	WBRV	-0.03546	1.38E-14

B1	B207	Gender	WHR	-0.03466	5.24E-14
B1	B201	Gender	e-habits	-0.03144	8.79E-12
B1	B137	Gender	AFU	-0.0314	9.29E-12
B1	B85	Gender	SCC	-0.0314	9.34E-12
B1	B144	Gender	Total-PINP	0.030891	1.99E-11
B1	B142	Gender	25-(OH)D	0.030451	3.82E-11
B1	B73	Gender	ApoA	0.030437	3.90E-11
B1	B193	Gender	K	0.030064	6.71E-11
B1	B57	Gender	RBCAI	-0.02981	9.74E-11
B1	B189	Gender	eGFB	0.029768	1.03E-10
B1	B43	Gender	MCV	-0.0296	1.31E-10
B1	B31	Gender	FSH	0.029463	1.59E-10
B1	B33	Gender	Prog	-0.02905	2.87E-10
B1	B143	Gender	β-CTx	0.027973	1.26E-09
B1	B69	Gender	PV	0.02788	1.43E-09
B1	B150	Gender	ALP	0.027667	1.90E-09
B1	B82	Gender	CEA	-0.02543	3.40E-08
B1	B158	Gender	IgM	0.025113	4.99E-08
B1	B30	Gender	LH	0.024373	1.22E-07
B1	B29	Gender	E2	-0.02293	6.43E-07
B1	B172	Gender	KB	-0.02257	9.60E-07
B10	B107	PG I	AnuA	-0.06445	0
B10	B11	PG I	PGI/PGII	0.095873	0
B10	B119	PG I	Hcv-rna	-0.05147	0
B10	B12	PG I	PG II	0.554769	0
B10	B126	PG I	Alb	-0.03961	0
B10	B14	PG I	G-17	0.05082	0
B10	B146	PG I	CysC	0.07041	0
B10	B15	PG I	CD3-CD19+(B cell)	-0.04705	0
B10	B159	PG I	β2-MG	0.038497	0
B10	B175	PG I	ALB-U	0.057422	0
B10	B177	PG I	ACR	0.077714	0
B10	B181	PG I	APTT	-0.04071	0
B10	B189	PG I	eGFB	-0.09737	0
B10	B190	PG I	Cre	0.093365	0
B10	B191	PG I	Urea	0.085856	0
B10	B35	PG I	β2-MG	0.062654	0
B10	B79	PG I	hb V -dna	-0.06371	0
B10	B80	PG I	anti-HP IgG	0.052653	0
B10	B183	PG I	PTA	-0.03757	4.44E-16
B10	B186	PG I	PFC	0.037075	8.88E-16
B10	B184	PG I	INR	-0.03128	1.12E-11
B10	B192	PG I	UA	0.028985	3.12E-10
B10	B47	PG I	RDW	0.028891	3.56E-10

B10	B215	PG I	BMI	-0.02878	4.14E-10
B10	B200	PG I	OP	0.027843	1.50E-09
B10	B210	PG I	Hip	-0.02655	8.27E-09
B10	B133	PG I	TBIL	-0.02554	2.94E-08
B10	B129	PG I	IBIL	-0.02492	6.32E-08
B10	B198	PG I	P	0.02301	5.88E-07
B100	B103	anti-Ro52	anti-SSA/Ro	0.387877	0
B100	B104	anti-Ro52	anti-SSB	0.329561	0
B100	B105	anti-Ro52	anti-U1RNP	0.07212	0
B100	B109	anti-Ro52	AMA-M2	0.062129	0
B100	B157	anti-Ro52	IgG	0.052587	0
B100	B155	anti-Ro52	IgA	0.035914	6.22E-15
B100	B139	anti-Ro52	ADA	0.032244	2.55E-12
B100	B138	anti-Ro52	PAB	-0.02619	1.30E-08
B100	B195	anti-Ro52	Na	-0.02473	7.94E-08
B100	B114	anti-Ro52	TSH TT3 TT4(TT4)	0.024251	1.41E-07
B100	B182	anti-Ro52	TT	0.023591	3.03E-07
B100	B199	anti-Ro52	Mg	-0.02331	4.19E-07
B100	B119	anti-Ro52	Hcv-rna	0.0228	7.44E-07
B101	B103	anti-Sc70	anti-SSA/Ro	0.241719	0
B101	B108	anti-Sc70	Anti-Cyclin	0.034201	1.12E-13
B102	B105	anti-Sm	anti-U1RNP	0.340392	0
B102	B109	anti-Sm	AMA-M2	0.238616	0
B102	B182	anti-Sm	TT	-0.0466	0
B102	B186	anti-Sm	PFC	0.051299	0
B102	B199	anti-Sm	Mg	-0.0379	2.22E-16
B102	B197	anti-Sm	AG	-0.03514	2.35E-14
B102	B107	anti-Sm	AnuA	-0.03508	2.60E-14
B102	B183	anti-Sm	PTA	-0.02779	1.61E-09
B102	B134	anti-Sm	CHE	-0.02434	1.27E-07
B103	B104	anti-SSA/Ro	anti-SSB	0.327209	0
B103	B105	anti-SSA/Ro	anti-U1RNP	0.08675	0
B103	B109	anti-SSA/Ro	AMA-M2	0.074334	0
B103	B110	anti-SSA/Ro	AHA	0.12884	0
B103	B155	anti-SSA/Ro	IgA	0.041149	0
B103	B157	anti-SSA/Ro	IgG	0.075154	0
B103	B138	anti-SSA/Ro	PAB	-0.03122	1.21E-11
B103	B139	anti-SSA/Ro	ADA	0.03038	4.24E-11
B103	B119	anti-SSA/Ro	Hcv-rna	0.026591	7.80E-09
B103	B158	anti-SSA/Ro	IgM	0.02415	1.58E-07
B103	B107	anti-SSA/Ro	AnuA	-0.02383	2.30E-07
B103	B127	anti-SSA/Ro	A-G ratio	-0.02347	3.49E-07
B103	B130	anti-SSA/Ro	GLB	0.023443	3.60E-07
B104	B109	anti-SSB	AMA-M2	0.038708	0

B104	B155	anti-SSB	IgA	0.047448	0
B104	B157	anti-SSB	IgG	0.11359	0
B104	B158	anti-SSB	IgM	0.044152	0
B104	B107	anti-SSB	AnuA	-0.0374	4.44E-16
B104	B195	anti-SSB	Na	-0.03349	3.56E-13
B104	B136	anti-SSB	5'NT	0.028851	3.77E-10
B105	B107	anti-U1RNP	AnuA	-0.06863	0
B105	B109	anti-U1RNP	AMA-M2	0.056055	0
B105	B110	anti-U1RNP	AHA	0.073356	0
B105	B151	anti-U1RNP	Anti-CCP	0.138415	0
B105	B182	anti-U1RNP	TT	-0.03901	0
B105	B186	anti-U1RNP	PFC	0.038954	0
B105	B199	anti-U1RNP	Mg	-0.03869	0
B105	B135	anti-U1RNP	TBA	0.035904	6.44E-15
B105	B125	anti-U1RNP	Cor	-0.02288	6.78E-07
B106	B110	anti-P	AHA	0.034711	4.84E-14
B106	B212	anti-P	IOP-L	0.02912	2.59E-10
B106	B170	anti-P	U-GLU	0.027627	2.00E-09
B106	B179	anti-P	α1-MG	0.024371	1.22E-07
B106	B193	anti-P	K	-0.02363	2.91E-07
B106	B178	anti-P	Ig-U	0.022658	8.72E-07
B107	B119	AnuA	Hcv-rna	0.139827	0
B107	B125	AnuA	Cor	-0.03926	0
B107	B134	AnuA	CHE	-0.05036	0
B107	B138	AnuA	PAB	-0.06145	0
B107	B150	AnuA	ALP	-0.0381	0
B107	B151	AnuA	Anti-CCP	-0.05379	0
B107	B155	AnuA	IgA	-0.05318	0
B107	B158	AnuA	IgM	0.039338	0
B107	B178	AnuA	Ig-U	-0.03961	0
B107	B181	AnuA	APTT	0.106004	0
B107	B183	AnuA	PTA	0.115774	0
B107	B184	AnuA	INR	0.130397	0
B107	B185	AnuA	PT	0.044087	0
B107	B186	AnuA	PFC	-0.13112	0
B107	B189	AnuA	eGFB	-0.06885	0
B107	B193	AnuA	K	0.125729	0
B107	B194	AnuA	Cl	0.05476	0
B107	B196	AnuA	TCO2	-0.04576	0
B107	B199	AnuA	Mg	-0.04411	0
B107	B152	AnuA	ACA-Ig(G-A-M)	-0.03781	2.22E-16
B107	B179	AnuA	α1-MG	-0.03691	1.11E-15
B107	B123	AnuA	hs-CRP1	-0.03361	2.95E-13
B107	B157	AnuA	IgG	-0.03245	1.85E-12

B107	B218	AnuA	DBP	-0.0303	4.77E-11
B107	B212	AnuA	IOP-L	-0.02998	7.56E-11
B107	B195	AnuA	Na	-0.0295	1.52E-10
B107	B122	AnuA	C4	-0.02714	3.81E-09
B107	B108	AnuA	Anti-Cyclin	0.026552	8.21E-09
B107	B204	AnuA	Drinking	-0.02569	2.45E-08
B107	B201	AnuA	e-habits	0.02462	9.06E-08
B107	B198	AnuA	P	0.023786	2.42E-07
B107	B210	AnuA	Hip	-0.02279	7.50E-07
B107	B190	AnuA	Cre	0.022691	8.40E-07
B107	B174	AnuA	Urea/Cr	-0.02263	9.01E-07
B108	B123	Anti-Cyclin	hs-CRP1	0.075708	0
B108	B176	Anti-Cyclin	Ucr	0.045386	0
B108	B181	Anti-Cyclin	APTT	0.039287	0
B108	B135	Anti-Cyclin	TBA	0.031622	6.63E-12
B108	B179	Anti-Cyclin	α1-MG	-0.0256	2.72E-08
B109	B158	AMA-M2	IgM	0.056779	0
B109	B199	AMA-M2	Mg	-0.04527	0
B109	B180	AMA-M2	Tf	0.037918	2.22E-16
B109	B135	AMA-M2	TBA	0.035508	1.27E-14
B109	B182	AMA-M2	TT	-0.03492	3.42E-14
B109	B155	AMA-M2	IgA	0.024878	6.64E-08
B109	B195	AMA-M2	Na	-0.02437	1.22E-07
B11	B12	PGI/PGII	PG II	-0.43774	0
B11	B14	PGI/PGII	G-17	-0.18533	0
B11	B175	PGI/PGII	ALB-U	0.054278	0
B11	B177	PGI/PGII	ACR	0.058894	0
B11	B79	PGI/PGII	hb V -dna	0.056905	0
B11	B80	PGI/PGII	anti-HP IgG	-0.10986	0
B11	B139	PGI/PGII	ADA	-0.03308	6.91E-13
B11	B127	PGI/PGII	A-G ratio	0.030782	2.34E-11
B11	B107	PGI/PGII	AnuA	0.030388	4.19E-11
B11	B47	PGI/PGII	RDW	-0.02995	7.87E-11
B11	B146	PGI/PGII	CysC	-0.02778	1.64E-09
B11	B153	PGI/PGII	Glu	0.026888	5.31E-09
B11	B183	PGI/PGII	PTA	0.026882	5.36E-09
B11	B166	PGI/PGII	Pro	0.026219	1.26E-08
B11	B78	PGI/PGII	HBcAb	0.025332	3.81E-08
B11	B137	PGI/PGII	AFU	-0.02502	5.61E-08
B11	B24	PGI/PGII	αHBDH	-0.02392	2.07E-07
B11	B130	PGI/PGII	GLB	-0.02355	3.17E-07
B11	B201	PGI/PGII	e-habits	-0.02342	3.70E-07
B11	B189	PGI/PGII	eGFB	0.023366	3.93E-07
B11	B126	PGI/PGII	Alb	0.023174	4.89E-07

B11	B182	PGI/PGII	TT	-0.02271	8.27E-07
B110	B122	AHA	C4	0.056661	0
B110	B138	AHA	PAB	-0.03524	2.00E-14
B110	B182	AHA	TT	-0.03475	4.55E-14
B110	B139	AHA	ADA	0.026786	6.06E-09
B110	B155	AHA	IgA	0.025445	3.32E-08
B110	B137	AHA	AFU	-0.02455	9.81E-08
B111	B112	TSH	FT4	-0.21359	0
B111	B113	TSH	FT3	-0.09914	0
B111	B114	TSH	TSH TT3 TT4(TT4)	-0.10954	0
B111	B115	TSH	TSH TT3 TT4(TT3)	-0.04984	0
B111	B127	TSH	A-G ratio	-0.04218	0
B111	B130	TSH	GLB	0.051328	0
B111	B148	TSH	Anti_TPO	0.205116	0
B111	B149	TSH	TGAb	0.186666	0
B111	B163	TSH	SG	-0.05667	0
B111	B204	TSH	Drinking	-0.0383	0
B111	B209	TSH	Smoking	-0.04892	0
B111	B221	TSH	Hei	-0.05542	0
B111	B176	TSH	Ucr	-0.03703	8.88E-16
B111	B189	TSH	eGFB	-0.03648	2.22E-15
B111	B222	TSH	NS	-0.03588	6.66E-15
B111	B216	TSH	BMI	-0.03027	4.95E-11
B111	B220	TSH	SBP	0.029313	1.97E-10
B111	B121	TSH	C3	0.029305	1.99E-10
B111	B157	TSH	IgG	0.025477	3.19E-08
B111	B219	TSH	OT	0.023544	3.20E-07
B112	B113	FT4	FT3	0.273486	0
B112	B114	FT4	TSH TT3 TT4(TT4)	0.487362	0
B112	B115	FT4	TSH TT3 TT4(TT3)	0.173978	0
B112	B125	FT4	Cor	0.049188	0
B112	B126	FT4	Alb	0.044009	0
B112	B129	FT4	IBIL	0.045532	0
B112	B133	FT4	TBIL	0.044092	0
B112	B206	FT4	Waist	-0.04742	0
B112	B210	FT4	Hip	-0.04218	0
B112	B215	FT4	BMI	-0.05695	0
B112	B216	FT4	BMI	-0.05216	0
B112	B222	FT4	NS	-0.03918	0
B112	B204	FT4	Drinking	-0.03541	1.49E-14
B112	B192	FT4	UA	-0.0343	9.46E-14
B112	B189	FT4	eGFB	0.034107	1.31E-13
B112	B187	FT4	NSE	0.029288	2.04E-10
B112	B138	FT4	PAB	-0.02867	4.84E-10

B112	B132	FT4	Dbil	0.027395	2.73E-09
B112	B139	FT4	ADA	0.027216	3.45E-09
B112	B178	FT4	Ig-U	0.026888	5.31E-09
B112	B149	FT4	TGAb	-0.02645	9.38E-09
B112	B153	FT4	Glu	0.026426	9.65E-09
B112	B194	FT4	Cl	-0.02603	1.60E-08
B112	B188	FT4	Ca	0.026005	1.65E-08
B112	B141	FT4	AST/ALT	0.025541	2.95E-08
B112	B150	FT4	ALP	0.025357	3.70E-08
B112	B140	FT4	r-GT	-0.02469	8.32E-08
B112	B180	FT4	Tf	0.024077	1.73E-07
B112	B181	FT4	APTT	-0.0237	2.68E-07
B112	B182	FT4	TT	-0.02259	9.36E-07
B113	B114	FT3	TSH TT3 TT4(TT4)	0.175276	0
B113	B115	FT3	TSH TT3 TT4(TT3)	0.402342	0
B113	B123	FT3	hs-CRP1	-0.03959	0
B113	B126	FT3	Alb	0.072542	0
B113	B128	FT3	ALT	0.060341	0
B113	B134	FT3	CHE	0.069704	0
B113	B141	FT3	AST/ALT	-0.05917	0
B113	B163	FT3	SG	0.042578	0
B113	B189	FT3	eGFB	0.072851	0
B113	B216	FT3	BMI	0.049145	0
B113	B218	FT3	DBP	0.048315	0
B113	B221	FT3	Hei	0.038869	0
B113	B222	FT3	NS	0.048072	0
B113	B215	FT3	BMI	0.036223	3.55E-15
B113	B206	FT3	Waist	0.036194	4.00E-15
B113	B188	FT3	Ca	0.035556	1.15E-14
B113	B131	FT3	AST	0.034691	5.00E-14
B113	B176	FT3	Ucr	0.03428	9.86E-14
B113	B210	FT3	Hip	0.033914	1.79E-13
B113	B119	FT3	Hcv-rna	-0.0319	4.31E-12
B113	B137	FT3	AFU	0.031692	5.96E-12
B113	B127	FT3	A-G ratio	0.031539	7.53E-12
B113	B209	FT3	Smoking	0.030219	5.36E-11
B113	B191	FT3	Urea	-0.02909	2.68E-10
B113	B190	FT3	Cre	-0.0288	4.04E-10
B113	B219	FT3	OT	-0.02868	4.76E-10
B113	B197	FT3	AG	0.028251	8.62E-10
B113	B140	FT3	r-GT	0.027917	1.36E-09
B113	B125	FT3	Cor	0.024964	5.98E-08
B113	B138	FT3	PAB	0.023829	2.30E-07
B113	B187	FT3	NSE	0.02339	3.82E-07

B113	B194	FT3	Cl	-0.02298	6.08E-07
B114	B115	TSH TT3 TT4(TT4)	TSH TT3 TT4(TT3)	0.425803	0
B114	B138	TSH TT3 TT4(TT4)	PAB	-0.07801	0
B114	B139	TSH TT3 TT4(TT4)	ADA	0.071701	0
B114	B150	TSH TT3 TT4(TT4)	ALP	0.040207	0
B114	B187	TSH TT3 TT4(TT4)	NSE	0.039982	0
B114	B192	TSH TT3 TT4(TT4)	UA	-0.04623	0
B114	B194	TSH TT3 TT4(TT4)	Cl	-0.04058	0
B114	B204	TSH TT3 TT4(TT4)	Drinking	-0.0482	0
B114	B216	TSH TT3 TT4(TT4)	BMI	-0.04677	0
B114	B221	TSH TT3 TT4(TT4)	Hei	-0.04003	0
B114	B222	TSH TT3 TT4(TT4)	NS	-0.04992	0
B114	B198	TSH TT3 TT4(TT4)	P	0.035952	5.77E-15
B114	B186	TSH TT3 TT4(TT4)	PFC	0.035648	9.99E-15
B114	B206	TSH TT3 TT4(TT4)	Waist	-0.03558	1.11E-14
B114	B215	TSH TT3 TT4(TT4)	BMI	-0.03072	2.58E-11
B114	B149	TSH TT3 TT4(TT4)	TGAb	-0.02951	1.48E-10
B114	B178	TSH TT3 TT4(TT4)	Ig-U	0.026276	1.17E-08
B114	B130	TSH TT3 TT4(TT4)	GLB	0.026204	1.28E-08
B114	B140	TSH TT3 TT4(TT4)	r-GT	-0.02616	1.36E-08
B114	B188	TSH TT3 TT4(TT4)	Ca	0.025904	1.87E-08
B114	B163	TSH TT3 TT4(TT4)	SG	-0.02574	2.29E-08
B114	B197	TSH TT3 TT4(TT4)	AG	0.025573	2.83E-08
B114	B180	TSH TT3 TT4(TT4)	Tf	0.025213	4.42E-08
B115	B124	TSH TT3 TT4(TT3)	ACTH	0.041197	0
B115	B126	TSH TT3 TT4(TT3)	Alb	0.055101	0
B115	B128	TSH TT3 TT4(TT3)	ALT	0.054999	0
B115	B134	TSH TT3 TT4(TT3)	CHE	0.097409	0
B115	B137	TSH TT3 TT4(TT3)	AFU	0.052451	0
B115	B141	TSH TT3 TT4(TT3)	AST/ALT	-0.04713	0
B115	B188	TSH TT3 TT4(TT3)	Ca	0.043938	0
B115	B189	TSH TT3 TT4(TT3)	eGFB	0.041813	0
B115	B215	TSH TT3 TT4(TT3)	BMI	0.036228	3.55E-15
B115	B216	TSH TT3 TT4(TT3)	BMI	0.035081	2.60E-14
B115	B125	TSH TT3 TT4(TT3)	Cor	0.034855	3.80E-14
B115	B131	TSH TT3 TT4(TT3)	AST	0.032543	1.60E-12
B115	B149	TSH TT3 TT4(TT3)	TGAb	-0.03242	1.94E-12
B115	B210	TSH TT3 TT4(TT3)	Hip	0.031705	5.85E-12
B115	B206	TSH TT3 TT4(TT3)	Waist	0.031623	6.62E-12
B115	B187	TSH TT3 TT4(TT3)	NSE	0.031457	8.52E-12
B115	B218	TSH TT3 TT4(TT3)	DBP	0.029601	1.31E-10
B115	B139	TSH TT3 TT4(TT3)	ADA	0.02915	2.48E-10
B115	B222	TSH TT3 TT4(TT3)	NS	0.028447	6.59E-10
B115	B181	TSH TT3 TT4(TT3)	APTT	-0.0265	8.75E-09

B115	B212	TSH TT3 TT4(TT3)	IOP-L	0.026433	9.57E-09
B115	B132	TSH TT3 TT4(TT3)	Dbil	-0.02519	4.54E-08
B115	B211	TSH TT3 TT4(TT3)	IOP-R	0.02513	4.89E-08
B115	B191	TSH TT3 TT4(TT3)	Urea	-0.02444	1.12E-07
B115	B150	TSH TT3 TT4(TT3)	ALP	0.024282	1.36E-07
B115	B133	TSH TT3 TT4(TT3)	TBIL	-0.02421	1.47E-07
B115	B190	TSH TT3 TT4(TT3)	Cre	-0.02337	3.91E-07
B115	B178	TSH TT3 TT4(TT3)	Ig-U	0.023132	5.12E-07
B116	B117	TT3	TT4	0.482667	0
B119	B121	Hcv-rna	C3	-0.11422	0
B119	B122	Hcv-rna	C4	-0.07205	0
B119	B138	Hcv-rna	PAB	-0.0687	0
B119	B150	Hcv-rna	ALP	-0.03879	0
B119	B181	Hcv-rna	APTT	0.066776	0
B119	B183	Hcv-rna	PTA	0.080739	0
B119	B184	Hcv-rna	INR	0.071933	0
B119	B185	Hcv-rna	PT	0.039204	0
B119	B186	Hcv-rna	PFC	-0.06474	0
B119	B189	Hcv-rna	eGFB	-0.04576	0
B119	B193	Hcv-rna	K	0.060676	0
B119	B194	Hcv-rna	Cl	0.081126	0
B119	B197	Hcv-rna	AG	-0.06115	0
B119	B199	Hcv-rna	Mg	-0.05295	0
B119	B222	Hcv-rna	NS	-0.03932	0
B119	B134	Hcv-rna	CHE	-0.03599	5.55E-15
B119	B125	Hcv-rna	Cor	-0.03217	2.87E-12
B119	B218	Hcv-rna	DBP	-0.03211	3.12E-12
B119	B158	Hcv-rna	IgM	0.03017	5.76E-11
B119	B204	Hcv-rna	Drinking	-0.0274	2.71E-09
B119	B163	Hcv-rna	SG	-0.02651	8.69E-09
B119	B216	Hcv-rna	BMI	-0.02497	5.96E-08
B119	B221	Hcv-rna	Hei	-0.02414	1.61E-07
B119	B210	Hcv-rna	Hip	-0.02358	3.09E-07
B119	B146	Hcv-rna	CysC	-0.02322	4.65E-07
B119	B212	Hcv-rna	IOP-L	-0.02301	5.91E-07
B119	B198	Hcv-rna	P	0.022751	7.86E-07
B12	B107	PG II	AnuA	-0.0651	0
B12	B126	PG II	Alb	-0.04298	0
B12	B14	PG II	G-17	0.172843	0
B12	B146	PG II	CysC	0.05754	0
B12	B15	PG II	CD3-CD19+(B cell)	-0.05886	0
B12	B183	PG II	PTA	-0.04685	0
B12	B186	PG II	PFC	0.068336	0
B12	B189	PG II	eGFB	-0.0805	0

B12	B190	PG II	Cre	0.054095	0
B12	B191	PG II	Urea	0.055962	0
B12	B79	PG II	hb V -dna	-0.07345	0
B12	B80	PG II	anti-HP IgG	0.108533	0
B12	B93	PG II	ALDH2-Glu504Lys	0.038985	0
B12	B99	PG II	anti-PM-Scl	0.040831	0
B12	B92	PG II	ALDH2-Glu504Glu	-0.03682	1.33E-15
B12	B47	PG II	RDW	0.036209	3.77E-15
B12	B35	PG II	β2-MG	0.036192	4.00E-15
B12	B127	PG II	A-G ratio	-0.03572	8.88E-15
B12	B199	PG II	Mg	0.033993	1.58E-13
B12	B184	PG II	INR	-0.03086	2.09E-11
B12	B216	PG II	BMI	-0.03046	3.77E-11
B12	B119	PG II	Hcv-rna	-0.02933	1.91E-10
B12	B90	PG II	CYFRA 21-1	0.029148	2.48E-10
B12	B215	PG II	BMI	-0.02895	3.26E-10
B12	B197	PG II	AG	0.027634	1.98E-09
B12	B78	PG II	HBcAb	-0.02755	2.22E-09
B12	B129	PG II	IBIL	-0.02717	3.67E-09
B12	B195	PG II	Na	0.025842	2.02E-08
B12	B200	PG II	OP	0.025181	4.59E-08
B12	B210	PG II	Hip	-0.02477	7.55E-08
B12	B133	PG II	TBIL	-0.02469	8.35E-08
B12	B130	PG II	GLB	0.024575	9.56E-08
B12	B139	PG II	ADA	0.023653	2.83E-07
B12	B182	PG II	TT	-0.02263	8.95E-07
B120	B158	HCV-cAg	IgM	0.045093	0
B120	B182	HCV-cAg	TT	0.042333	0
B120	B186	HCV-cAg	PFC	0.048963	0
B121	B122	C3	C4	0.509212	0
B121	B156	C3	IgE	0.053691	0
B121	B195	C3	Na	-0.04185	0
B121	B155	C3	IgA	0.037132	8.88E-16
B121	B157	C3	IgG	0.03173	5.62E-12
B121	B196	C3	TCO2	-0.03102	1.65E-11
B121	B194	C3	Cl	-0.02968	1.17E-10
B121	B200	C3	OP	-0.02826	8.54E-10
B121	B141	C3	AST/ALT	0.027188	3.59E-09
B121	B197	C3	AG	0.024282	1.36E-07
B122	B156	C4	IgE	0.047871	0
B123	B126	hs-CRP1	Alb	-0.07371	0
B123	B127	hs-CRP1	A-G ratio	-0.06077	0
B123	B130	hs-CRP1	GLB	0.04455	0
B123	B138	hs-CRP1	PAB	-0.04328	0

B123	B176	hs-CRP1	Ucr	0.046302	0
B123	B179	hs-CRP1	α1-MG	0.045318	0
B123	B186	hs-CRP1	PFC	0.076071	0
B123	B190	hs-CRP1	Cre	0.037079	8.88E-16
B123	B206	hs-CRP1	Waist	0.036952	8.88E-16
B123	B215	hs-CRP1	BMI	0.035773	7.99E-15
B123	B175	hs-CRP1	ALB-U	0.033074	6.94E-13
B123	B177	hs-CRP1	ACR	0.030648	2.86E-11
B123	B150	hs-CRP1	ALP	0.029626	1.26E-10
B123	B192	hs-CRP1	UA	0.029548	1.41E-10
B123	B140	hs-CRP1	r-GT	0.029345	1.88E-10
B123	B191	hs-CRP1	Urea	0.027293	3.12E-09
B123	B216	hs-CRP1	BMI	0.025886	1.92E-08
B123	B210	hs-CRP1	Hip	0.024025	1.83E-07
B123	B193	hs-CRP1	K	-0.02376	2.51E-07
B123	B188	hs-CRP1	Ca	-0.02343	3.66E-07
B124	B125	ACTH	Cor	0.537347	0
B124	B134	ACTH	CHE	0.044663	0
B124	B135	ACTH	TBA	0.041874	0
B124	B157	ACTH	IgG	0.0342	1.13E-13
B124	B183	ACTH	PTA	-0.02977	1.03E-10
B124	B195	ACTH	Na	-0.02632	1.11E-08
B125	B134	Cor	CHE	0.044108	0
B125	B157	Cor	IgG	0.038996	0
B125	B181	Cor	APTT	-0.05354	0
B125	B183	Cor	PTA	-0.05927	0
B125	B194	Cor	Cl	-0.05055	0
B125	B195	Cor	Na	-0.05959	0
B125	B156	Cor	IgE	-0.03506	2.71E-14
B125	B184	Cor	INR	-0.03389	1.86E-13
B125	B126	Cor	Alb	0.033369	4.33E-13
B125	B135	Cor	TBA	0.03092	1.91E-11
B125	B182	Cor	TT	-0.03036	4.39E-11
B125	B138	Cor	PAB	0.03023	5.28E-11
B125	B200	Cor	OP	-0.02964	1.24E-10
B125	B186	Cor	PFC	0.028299	8.07E-10
B125	B137	Cor	AFU	0.024912	6.37E-08
B126	B127	Alb	A-G ratio	0.492015	0
B126	B128	Alb	ALT	0.143325	0
B126	B129	Alb	IBIL	0.114229	0
B126	B130	Alb	GLB	-0.16	0
B126	B131	Alb	AST	0.054105	0
B126	B132	Alb	Dbil	0.048942	0
B126	B133	Alb	TBIL	0.105256	0

B126	B134	Alb	CHE	0.091459	0
B126	B138	Alb	PAB	0.097417	0
B126	B141	Alb	AST/ALT	-0.16403	0
B126	B146	Alb	CysC	-0.0803	0
B126	B153	Alb	Glu	0.068232	0
B126	B161	Alb	LEU	-0.0438	0
B126	B163	Alb	SG	0.049303	0
B126	B175	Alb	ALB-U	-0.03942	0
B126	B176	Alb	Ucr	0.040923	0
B126	B177	Alb	ACR	-0.05689	0
B126	B188	Alb	Ca	0.388354	0
B126	B189	Alb	eGFB	0.245767	0
B126	B190	Alb	Cre	-0.08983	0
B126	B191	Alb	Urea	-0.11203	0
B126	B192	Alb	UA	0.043903	0
B126	B194	Alb	Cl	-0.05728	0
B126	B197	Alb	AG	0.038349	0
B126	B199	Alb	Mg	0.039633	0
B126	B203	Alb	DH	0.039503	0
B126	B204	Alb	Drinking	0.045174	0
B126	B211	Alb	IOP-R	0.053882	0
B126	B212	Alb	IOP-L	0.052193	0
B126	B216	Alb	BMI	0.097359	0
B126	B218	Alb	DBP	0.143532	0
B126	B219	Alb	OT	-0.03991	0
B126	B221	Alb	Hei	0.117444	0
B126	B222	Alb	NS	0.089696	0
B126	B140	Alb	r-GT	0.036731	1.55E-15
B126	B173	Alb	NTT	-0.03626	3.55E-15
B126	B215	Alb	BMI	0.033894	1.86E-13
B126	B210	Alb	Hip	0.032721	1.21E-12
B126	B172	Alb	KB	0.031547	7.44E-12
B126	B155	Alb	IgA	-0.03118	1.30E-11
B126	B139	Alb	ADA	-0.03001	7.28E-11
B126	B174	Alb	Urea/Cr	-0.02897	3.18E-10
B126	B228	Alb	OS	-0.02796	1.28E-09
B126	B205	Alb	KDA	-0.02405	1.78E-07
B126	B157	Alb	IgG	-0.02388	2.18E-07
B127	B129	A-G ratio	IBIL	0.129484	0
B127	B130	A-G ratio	GLB	-0.90162	0
B127	B131	A-G ratio	AST	-0.06477	0
B127	B132	A-G ratio	Dbil	0.10118	0
B127	B133	A-G ratio	TBIL	0.131652	0
B127	B138	A-G ratio	PAB	0.058497	0

B127	B139	A-G ratio	ADA	-0.06683	0
B127	B141	A-G ratio	AST/ALT	-0.11966	0
B127	B146	A-G ratio	CysC	-0.06115	0
B127	B150	A-G ratio	ALP	-0.04996	0
B127	B153	A-G ratio	Glu	-0.06167	0
B127	B155	A-G ratio	IgA	-0.08781	0
B127	B157	A-G ratio	IgG	-0.11739	0
B127	B161	A-G ratio	LEU	-0.06186	0
B127	B163	A-G ratio	SG	0.1003	0
B127	B173	A-G ratio	NTT	-0.04711	0
B127	B174	A-G ratio	Urea/Cr	-0.04501	0
B127	B176	A-G ratio	Ucr	0.043462	0
B127	B177	A-G ratio	ACR	-0.03953	0
B127	B188	A-G ratio	Ca	0.041598	0
B127	B189	A-G ratio	eGFB	0.198697	0
B127	B190	A-G ratio	Cre	-0.04187	0
B127	B191	A-G ratio	Urea	-0.07555	0
B127	B192	A-G ratio	UA	-0.0444	0
B127	B204	A-G ratio	Drinking	0.098074	0
B127	B209	A-G ratio	Smoking	0.129065	0
B127	B216	A-G ratio	BMI	0.102209	0
B127	B218	A-G ratio	DBP	0.063646	0
B127	B220	A-G ratio	SBP	-0.10344	0
B127	B221	A-G ratio	Hei	0.176335	0
B127	B222	A-G ratio	NS	0.100607	0
B127	B128	A-G ratio	ALT	0.035172	2.22E-14
B127	B205	A-G ratio	KDA	-0.03383	2.07E-13
B127	B158	A-G ratio	IgM	-0.03363	2.84E-13
B127	B219	A-G ratio	OT	-0.03349	3.57E-13
B127	B149	A-G ratio	TGAb	-0.03203	3.56E-12
B127	B136	A-G ratio	5'NT	-0.03166	6.30E-12
B127	B175	A-G ratio	ALB-U	-0.02874	4.38E-10
B127	B148	A-G ratio	Anti_TPO	-0.02639	1.01E-08
B127	B212	A-G ratio	IOP-L	0.026111	1.44E-08
B127	B140	A-G ratio	r-GT	-0.02459	9.40E-08
B127	B211	A-G ratio	IOP-R	0.023349	4.01E-07
B127	B186	A-G ratio	PFC	-0.02285	7.07E-07
B127	B134	A-G ratio	CHE	0.02273	8.05E-07
B128	B129	ALT	IBIL	0.051427	0
B128	B131	ALT	AST	0.742183	0
B128	B132	ALT	Dbil	0.100488	0
B128	B133	ALT	TBIL	0.068559	0
B128	B134	ALT	CHE	0.057641	0
B128	B136	ALT	5'NT	0.072309	0

B128	B137	ALT	AFU	0.05439	0
B128	B139	ALT	ADA	0.040679	0
B128	B140	ALT	r-GT	0.358489	0
B128	B141	ALT	AST/ALT	-0.49844	0
B128	B150	ALT	ALP	0.04454	0
B128	B153	ALT	Glu	0.098031	0
B128	B160	ALT	PH	-0.04255	0
B128	B163	ALT	SG	0.114729	0
B128	B166	ALT	Pro	0.045979	0
B128	B170	ALT	U-GLU	0.041984	0
B128	B174	ALT	Urea/Cr	-0.06206	0
B128	B176	ALT	Ucr	0.067298	0
B128	B188	ALT	Ca	0.059837	0
B128	B189	ALT	eGFB	0.140244	0
B128	B191	ALT	Urea	-0.05036	0
B128	B192	ALT	UA	0.175498	0
B128	B203	ALT	DH	0.041517	0
B128	B204	ALT	Drinking	0.105922	0
B128	B206	ALT	Waist	0.213473	0
B128	B209	ALT	Smoking	0.074441	0
B128	B210	ALT	Hip	0.181444	0
B128	B215	ALT	BMI	0.230384	0
B128	B216	ALT	BMI	0.266859	0
B128	B218	ALT	DBP	0.124008	0
B128	B219	ALT	OT	-0.03882	0
B128	B220	ALT	SBP	-0.04622	0
B128	B221	ALT	Hei	0.161585	0
B128	B222	ALT	NS	0.223495	0
B128	B173	ALT	NTT	-0.03162	6.70E-12
B128	B212	ALT	IOP-L	0.03155	7.40E-12
B128	B161	ALT	LEU	-0.03123	1.20E-11
B128	B135	ALT	TBA	0.03063	2.93E-11
B128	B211	ALT	IOP-R	0.029837	9.32E-11
B128	B201	ALT	e-habits	-0.0297	1.14E-10
B128	B138	ALT	PAB	0.029046	2.87E-10
B128	B130	ALT	GLB	0.026745	6.40E-09
B128	B198	ALT	P	-0.02488	6.62E-08
B128	B193	ALT	K	-0.02461	9.17E-08
B128	B205	ALT	KDA	-0.02435	1.25E-07
B128	B149	ALT	TGAb	-0.02406	1.76E-07
B128	B148	ALT	Anti_TPO	-0.02404	1.80E-07
B128	B165	ALT	bilirubin	0.023928	2.05E-07
B129	B130	IBIL	GLB	-0.1045	0
B129	B131	IBIL	AST	0.083968	0

B129	B132	IBIL	Dbil	0.641139	0
B129	B133	IBIL	TBIL	0.978225	0
B129	B140	IBIL	r-GT	0.041231	0
B129	B160	IBIL	PH	0.07786	0
B129	B163	IBIL	SG	0.06853	0
B129	B166	IBIL	Pro	0.041236	0
B129	B174	IBIL	Urea/Cr	-0.09968	0
B129	B191	IBIL	Urea	-0.06626	0
B129	B192	IBIL	UA	0.053292	0
B129	B198	IBIL	P	-0.03941	0
B129	B209	IBIL	Smoking	-0.04949	0
B129	B216	IBIL	BMI	0.066142	0
B129	B221	IBIL	Hei	0.100567	0
B129	B222	IBIL	NS	0.077336	0
B129	B176	IBIL	Ucr	0.037182	6.66E-16
B129	B189	IBIL	eGFB	-0.03715	8.88E-16
B129	B188	IBIL	Ca	0.036157	4.00E-15
B129	B183	IBIL	PTA	0.035687	9.33E-15
B129	B204	IBIL	Drinking	0.033949	1.70E-13
B129	B161	IBIL	LEU	-0.03358	3.10E-13
B129	B218	IBIL	DBP	0.031434	8.82E-12
B129	B150	IBIL	ALP	-0.03054	3.34E-11
B129	B146	IBIL	CysC	-0.02854	5.77E-10
B129	B165	IBIL	bilirubin	0.025037	5.47E-08
B129	B193	IBIL	K	-0.0244	1.17E-07
B129	B205	IBIL	KDA	-0.02434	1.27E-07
B129	B206	IBIL	Waist	0.023996	1.90E-07
B129	B173	IBIL	NTT	-0.02269	8.39E-07
B130	B131	GLB	AST	0.111932	0
B130	B132	GLB	Dbil	-0.08071	0
B130	B133	GLB	TBIL	-0.10599	0
B130	B136	GLB	5'NT	0.044965	0
B130	B139	GLB	ADA	0.065882	0
B130	B140	GLB	r-GT	0.065015	0
B130	B141	GLB	AST/ALT	0.074911	0
B130	B146	GLB	CysC	0.049696	0
B130	B150	GLB	ALP	0.0536	0
B130	B153	GLB	Glu	0.104081	0
B130	B155	GLB	IgA	0.091155	0
B130	B157	GLB	IgG	0.127641	0
B130	B161	GLB	LEU	0.054145	0
B130	B163	GLB	SG	-0.09084	0
B130	B173	GLB	NTT	0.042834	0
B130	B188	GLB	Ca	0.107795	0

B130	B189	GLB	eGFB	-0.13282	0
B130	B191	GLB	Urea	0.046945	0
B130	B192	GLB	UA	0.076451	0
B130	B204	GLB	Drinking	-0.08907	0
B130	B209	GLB	Smoking	-0.13743	0
B130	B216	GLB	BMI	-0.07934	0
B130	B220	GLB	SBP	0.110137	0
B130	B221	GLB	Hei	-0.15331	0
B130	B222	GLB	NS	-0.07793	0
B130	B174	GLB	Urea/Cr	0.037165	6.66E-16
B130	B158	GLB	IgM	0.036821	1.33E-15
B130	B194	GLB	Cl	-0.03613	4.44E-15
B130	B149	GLB	TGAb	0.034107	1.31E-13
B130	B138	GLB	PAB	-0.03135	1.00E-11
B130	B176	GLB	Ucr	-0.02994	8.00E-11
B130	B148	GLB	Anti_TPO	0.029114	2.61E-10
B130	B205	GLB	KDA	0.027047	4.32E-09
B130	B166	GLB	Pro	0.02606	1.54E-08
B130	B193	GLB	K	0.025328	3.83E-08
B130	B170	GLB	U-GLU	0.024973	5.92E-08
B130	B219	GLB	OT	0.022702	8.30E-07
B131	B132	AST	Dbil	0.169738	0
B131	B133	AST	TBIL	0.113394	0
B131	B135	AST	TBA	0.048742	0
B131	B136	AST	5'NT	0.080567	0
B131	B137	AST	AFU	0.060139	0
B131	B139	AST	ADA	0.065919	0
B131	B140	AST	r-GT	0.39731	0
B131	B141	AST	AST/ALT	-0.06955	0
B131	B150	AST	ALP	0.049596	0
B131	B153	AST	Glu	0.063458	0
B131	B165	AST	bilirubin	0.066323	0
B131	B166	AST	Pro	0.038043	0
B131	B188	AST	Ca	0.051209	0
B131	B192	AST	UA	0.125298	0
B131	B204	AST	Drinking	0.089523	0
B131	B206	AST	Waist	0.088841	0
B131	B209	AST	Smoking	0.038413	0
B131	B210	AST	Hip	0.052198	0
B131	B215	AST	BMI	0.091469	0
B131	B216	AST	BMI	0.076293	0
B131	B218	AST	DBP	0.042024	0
B131	B222	AST	NS	0.07755	0
B131	B189	AST	eGFB	0.037548	4.44E-16

B131	B225	AST	hPBG	0.036275	3.33E-15
B131	B163	AST	SG	0.035269	1.91E-14
B131	B191	AST	Urea	-0.0322	2.74E-12
B131	B176	AST	Ucr	0.028485	6.25E-10
B131	B179	AST	α1-MG	0.026553	8.19E-09
B131	B174	AST	Urea/Cr	-0.02633	1.10E-08
B132	B133	Dbil	TBIL	0.786212	0
B132	B140	Dbil	r-GT	0.15977	0
B132	B160	Dbil	PH	0.047042	0
B132	B163	Dbil	SG	0.051732	0
B132	B165	Dbil	bilirubin	0.069013	0
B132	B166	Dbil	Pro	0.042883	0
B132	B174	Dbil	Urea/Cr	-0.093	0
B132	B189	Dbil	eGFB	-0.07582	0
B132	B192	Dbil	UA	0.04697	0
B132	B198	Dbil	P	-0.04455	0
B132	B204	Dbil	Drinking	0.06815	0
B132	B216	Dbil	BMI	0.040725	0
B132	B221	Dbil	Hei	0.093605	0
B132	B222	Dbil	NS	0.069878	0
B132	B193	Dbil	K	-0.03659	1.78E-15
B132	B161	Dbil	LEU	-0.03411	1.31E-13
B132	B139	Dbil	ADA	0.033979	1.61E-13
B132	B191	Dbil	Urea	-0.03214	2.97E-12
B132	B134	Dbil	CHE	-0.03126	1.15E-11
B132	B190	Dbil	Cre	0.028026	1.17E-09
B132	B172	Dbil	KB	0.024326	1.29E-07
B132	B176	Dbil	Ucr	0.023647	2.85E-07
B132	B136	Dbil	5'NT	0.022699	8.33E-07
B133	B140	TBIL	r-GT	0.076369	0
B133	B160	TBIL	PH	0.075491	0
B133	B163	TBIL	SG	0.069083	0
B133	B165	TBIL	bilirubin	0.038854	0
B133	B166	TBIL	Pro	0.04482	0
B133	B174	TBIL	Urea/Cr	-0.10537	0
B133	B189	TBIL	eGFB	-0.05037	0
B133	B191	TBIL	Urea	-0.06202	0
B133	B192	TBIL	UA	0.055625	0
B133	B198	TBIL	P	-0.04365	0
B133	B204	TBIL	Drinking	0.045719	0
B133	B209	TBIL	Smoking	-0.0457	0
B133	B216	TBIL	BMI	0.064313	0
B133	B221	TBIL	Hei	0.10633	0
B133	B222	TBIL	NS	0.081183	0

B133	B161	TBIL	LEU	-0.0363	3.11E-15
B133	B176	TBIL	Ucr	0.036332	3.11E-15
B133	B183	TBIL	PTA	0.032428	1.92E-12
B133	B188	TBIL	Ca	0.030583	3.14E-11
B133	B193	TBIL	K	-0.02963	1.25E-10
B133	B206	TBIL	Waist	0.025291	4.01E-08
B133	B218	TBIL	DBP	0.024783	7.44E-08
B133	B150	TBIL	ALP	-0.02401	1.88E-07
B134	B136	CHE	5'NT	0.132961	0
B134	B137	CHE	AFU	0.157904	0
B134	B138	CHE	PAB	0.348863	0
B134	B139	CHE	ADA	-0.10819	0
B134	B140	CHE	r-GT	0.040754	0
B134	B141	CHE	AST/ALT	-0.06235	0
B134	B146	CHE	CysC	-0.03974	0
B134	B150	CHE	ALP	0.044697	0
B134	B157	CHE	IgG	-0.06114	0
B134	B181	CHE	APTT	-0.06066	0
B134	B184	CHE	INR	-0.05021	0
B134	B188	CHE	Ca	0.062607	0
B134	B189	CHE	eGFB	0.042173	0
B134	B192	CHE	UA	0.042482	0
B134	B193	CHE	K	-0.04424	0
B134	B194	CHE	Cl	-0.1414	0
B134	B195	CHE	Na	0.05485	0
B134	B197	CHE	AG	0.140927	0
B134	B199	CHE	Mg	0.044271	0
B134	B200	CHE	OP	0.040075	0
B134	B204	CHE	Drinking	0.045299	0
B134	B206	CHE	Waist	0.061004	0
B134	B210	CHE	Hip	0.050886	0
B134	B215	CHE	BMI	0.068069	0
B134	B216	CHE	BMI	0.076328	0
B134	B218	CHE	DBP	0.039099	0
B134	B221	CHE	Hei	0.043716	0
B134	B222	CHE	NS	0.062816	0
B134	B185	CHE	PT	-0.03793	2.22E-16
B134	B186	CHE	PFC	0.036215	3.77E-15
B134	B163	CHE	SG	0.035944	6.00E-15
B134	B153	CHE	Glu	0.033335	4.57E-13
B134	B156	CHE	IgE	-0.03319	5.77E-13
B134	B212	CHE	IOP-L	0.032917	8.89E-13
B134	B209	CHE	Smoking	0.027375	2.80E-09
B134	B211	CHE	IOP-R	0.023777	2.45E-07

B135	B136	TBA	5'NT	0.092569	0
B135	B137	TBA	AFU	0.091105	0
B135	B139	TBA	ADA	0.101901	0
B135	B140	TBA	r-GT	0.053213	0
B135	B150	TBA	ALP	0.08296	0
B135	B182	TBA	TT	0.040025	0
B135	B193	TBA	K	-0.07819	0
B135	B200	TBA	OP	0.034894	3.55E-14
B135	B179	TBA	α1-MG	0.034864	3.73E-14
B135	B198	TBA	P	-0.03316	6.08E-13
B135	B195	TBA	Na	0.029378	1.80E-10
B135	B194	TBA	Cl	0.026331	1.09E-08
B135	B181	TBA	APTT	0.025157	4.73E-08
B135	B165	TBA	bilirubin	0.024574	9.57E-08
B135	B156	TBA	IgE	-0.02353	3.25E-07
B136	B137	5'NT	AFU	0.074189	0
B136	B138	5'NT	PAB	0.073439	0
B136	B139	5'NT	ADA	0.17969	0
B136	B140	5'NT	r-GT	0.19426	0
B136	B150	5'NT	ALP	0.2108	0
B136	B155	5'NT	IgA	0.047615	0
B136	B157	5'NT	IgG	0.055904	0
B136	B158	5'NT	IgM	0.04148	0
B136	B179	5'NT	α1-MG	0.090308	0
B136	B181	5'NT	APTT	0.041144	0
B136	B182	5'NT	TT	0.06403	0
B136	B194	5'NT	Cl	-0.04344	0
B136	B197	5'NT	AG	0.061889	0
B136	B186	5'NT	PFC	0.031025	1.63E-11
B136	B200	5'NT	OP	0.028216	9.04E-10
B136	B193	5'NT	K	-0.02741	2.68E-09
B136	B141	5'NT	AST/ALT	-0.02548	3.18E-08
B136	B196	5'NT	TCO2	-0.02535	3.71E-08
B137	B138	AFU	PAB	0.093297	0
B137	B139	AFU	ADA	0.183255	0
B137	B140	AFU	r-GT	0.068504	0
B137	B150	AFU	ALP	0.06927	0
B137	B156	AFU	IgE	-0.05784	0
B137	B179	AFU	α1-MG	0.038602	0
B137	B193	AFU	K	-0.04339	0
B137	B194	AFU	Cl	-0.04279	0
B137	B197	AFU	AG	0.038073	0
B137	B198	AFU	P	-0.05524	0
B137	B200	AFU	OP	0.067118	0

B137	B222	AFU	NS	0.035984	5.55E-15
B137	B206	AFU	Waist	0.035525	1.22E-14
B137	B216	AFU	BMI	0.032944	8.52E-13
B137	B153	AFU	Glu	0.031918	4.22E-12
B137	B204	AFU	Drinking	0.026258	1.20E-08
B137	B215	AFU	BMI	0.026052	1.55E-08
B137	B195	AFU	Na	0.024925	6.27E-08
B137	B221	AFU	Hei	0.02264	8.89E-07
B138	B139	PAB	ADA	-0.32591	0
B138	B140	PAB	r-GT	0.064877	0
B138	B141	PAB	AST/ALT	-0.04211	0
B138	B150	PAB	ALP	-0.0571	0
B138	B157	PAB	IgG	-0.09821	0
B138	B163	PAB	SG	0.043528	0
B138	B179	PAB	α1-MG	0.038586	0
B138	B181	PAB	APTT	-0.07859	0
B138	B184	PAB	INR	-0.05571	0
B138	B185	PAB	PT	-0.04488	0
B138	B188	PAB	Ca	0.056489	0
B138	B190	PAB	Cre	0.039408	0
B138	B192	PAB	UA	0.087583	0
B138	B194	PAB	Cl	-0.14683	0
B138	B197	PAB	AG	0.117761	0
B138	B198	PAB	P	-0.04249	0
B138	B199	PAB	Mg	0.042032	0
B138	B204	PAB	Drinking	0.09791	0
B138	B212	PAB	IOP-L	0.038376	0
B138	B216	PAB	BMI	0.063435	0
B138	B221	PAB	Hei	0.08231	0
B138	B222	PAB	NS	0.075804	0
B138	B176	PAB	Ucr	0.037906	2.22E-16
B138	B174	PAB	Urea/Cr	-0.03728	4.44E-16
B138	B218	PAB	DBP	0.036974	8.88E-16
B138	B206	PAB	Waist	0.036889	1.11E-15
B138	B211	PAB	IOP-R	0.034605	5.77E-14
B138	B209	PAB	Smoking	0.033975	1.63E-13
B138	B200	PAB	OP	0.032213	2.68E-12
B138	B186	PAB	PFC	-0.02974	1.07E-10
B138	B210	PAB	Hip	0.026443	9.44E-09
B138	B161	PAB	LEU	-0.02559	2.78E-08
B138	B219	PAB	OT	-0.02417	1.55E-07
B139	B140	ADA	r-GT	0.041458	0
B139	B146	ADA	CysC	0.07773	0
B139	B150	ADA	ALP	0.182014	0

B139	B153	ADA	Glu	0.042726	0
B139	B155	ADA	IgA	0.084211	0
B139	B157	ADA	IgG	0.085627	0
B139	B179	ADA	α1-MG	0.03823	0
B139	B186	ADA	PFC	0.038024	0
B139	B193	ADA	K	-0.05724	0
B139	B194	ADA	Cl	-0.04626	0
B139	B195	ADA	Na	0.09312	0
B139	B197	ADA	AG	0.082966	0
B139	B200	ADA	OP	0.115009	0
B139	B221	ADA	Hei	-0.04791	0
B139	B220	ADA	SBP	0.036531	2.22E-15
B139	B174	ADA	Urea/Cr	0.035575	1.13E-14
B139	B222	ADA	NS	-0.03078	2.36E-11
B139	B181	ADA	APTT	0.029247	2.16E-10
B139	B182	ADA	TT	0.028477	6.32E-10
B139	B216	ADA	BMI	-0.02787	1.44E-09
B139	B204	ADA	Drinking	-0.02743	2.60E-09
B139	B184	ADA	INR	0.02641	9.85E-09
B139	B163	ADA	SG	-0.02638	1.03E-08
B139	B185	ADA	PT	0.025443	3.33E-08
B139	B154	ADA	RF	0.024979	5.87E-08
B139	B209	ADA	Smoking	-0.02256	9.67E-07
B14	B80	G-17	anti-HP IgG	0.038216	0
B14	B139	G-17	ADA	0.033056	7.14E-13
B140	B141	r-GT	AST/ALT	-0.14319	0
B140	B150	r-GT	ALP	0.123542	0
B140	B153	r-GT	Glu	0.107362	0
B140	B160	r-GT	PH	-0.05079	0
B140	B163	r-GT	SG	0.097937	0
B140	B165	r-GT	bilirubin	0.060442	0
B140	B166	r-GT	Pro	0.053349	0
B140	B170	r-GT	U-GLU	0.059085	0
B140	B174	r-GT	Urea/Cr	-0.0802	0
B140	B176	r-GT	Ucr	0.06463	0
B140	B179	r-GT	α1-MG	0.079367	0
B140	B188	r-GT	Ca	0.041977	0
B140	B189	r-GT	eGFB	0.081265	0
B140	B191	r-GT	Urea	-0.04725	0
B140	B192	r-GT	UA	0.175731	0
B140	B203	r-GT	DH	0.043248	0
B140	B204	r-GT	Drinking	0.209676	0
B140	B206	r-GT	Waist	0.134856	0
B140	B209	r-GT	Smoking	0.140665	0

B140	B210	r-GT	Hip	0.090138	0
B140	B215	r-GT	BMI	0.105103	0
B140	B216	r-GT	BMI	0.150006	0
B140	B218	r-GT	DBP	0.09648	0
B140	B221	r-GT	Hei	0.121214	0
B140	B222	r-GT	NS	0.160643	0
B140	B193	r-GT	K	-0.03528	1.84E-14
B140	B201	r-GT	e-habits	-0.02663	7.40E-09
B140	B219	r-GT	OT	-0.02645	9.35E-09
B140	B211	r-GT	IOP-R	0.025775	2.20E-08
B140	B212	r-GT	IOP-L	0.025746	2.28E-08
B140	B182	r-GT	TT	0.023878	2.18E-07
B140	B198	r-GT	P	-0.02349	3.39E-07
B141	B146	AST/ALT	CysC	0.041776	0
B141	B153	AST/ALT	Glu	-0.0798	0
B141	B161	AST/ALT	LEU	0.053495	0
B141	B163	AST/ALT	SG	-0.1362	0
B141	B170	AST/ALT	U-GLU	-0.04113	0
B141	B173	AST/ALT	NTT	0.0472	0
B141	B174	AST/ALT	Urea/Cr	0.068322	0
B141	B176	AST/ALT	Ucr	-0.06852	0
B141	B189	AST/ALT	eGFB	-0.15748	0
B141	B191	AST/ALT	Urea	0.054503	0
B141	B192	AST/ALT	UA	-0.14668	0
B141	B203	AST/ALT	DH	-0.04182	0
B141	B204	AST/ALT	Drinking	-0.08152	0
B141	B205	AST/ALT	KDA	0.040631	0
B141	B206	AST/ALT	Waist	-0.24087	0
B141	B209	AST/ALT	Smoking	-0.07697	0
B141	B210	AST/ALT	Hip	-0.22459	0
B141	B211	AST/ALT	IOP-R	-0.03939	0
B141	B215	AST/ALT	BMI	-0.26512	0
B141	B216	AST/ALT	BMI	-0.33233	0
B141	B218	AST/ALT	DBP	-0.14312	0
B141	B219	AST/ALT	OT	0.048723	0
B141	B220	AST/ALT	SBP	0.089517	0
B141	B221	AST/ALT	Hei	-0.23736	0
B141	B222	AST/ALT	NS	-0.2639	0
B141	B157	AST/ALT	IgG	0.037204	6.66E-16
B141	B212	AST/ALT	IOP-L	-0.0347	4.93E-14
B141	B188	AST/ALT	Ca	-0.03463	5.57E-14
B141	B160	AST/ALT	PH	0.032853	9.83E-13
B141	B166	AST/ALT	Pro	-0.02981	9.64E-11
B141	B224	AST/ALT	pmh	0.029321	1.95E-10

B141	B228	AST/ALT	OS	0.028921	3.41E-10
B141	B148	AST/ALT	Anti_TPO	0.024483	1.07E-07
B141	B149	AST/ALT	TGAb	0.023916	2.08E-07
B142	B144	25-(OH)D	Total-PINP	-0.04594	0
B142	B145	25-(OH)D	BGP	-0.05649	0
B142	B176	25-(OH)D	Ucr	-0.04358	0
B142	B189	25-(OH)D	eGFB	-0.03343	3.92E-13
B142	B216	25-(OH)D	BMI	-0.03208	3.30E-12
B142	B222	25-(OH)D	NS	-0.0296	1.30E-10
B142	B218	25-(OH)D	DBP	-0.028	1.21E-09
B142	B221	25-(OH)D	Hei	-0.02501	5.69E-08
B142	B215	25-(OH)D	BMI	-0.02451	1.03E-07
B142	B210	25-(OH)D	Hip	-0.02301	5.85E-07
B143	B144	β-CTx	Total-PINP	0.790642	0
B143	B145	β-CTx	BGP	0.573647	0
B143	B150	β-CTx	ALP	0.051779	0
B143	B176	β-CTx	Ucr	0.026696	6.81E-09
B143	B192	β-CTx	UA	-0.02513	4.91E-08
B143	B183	β-CTx	PTA	0.023165	4.94E-07
B144	B145	Total-PINP	BGP	0.57821	0
B144	B150	Total-PINP	ALP	0.067005	0
B144	B177	Total-PINP	ACR	-0.0277	1.82E-09
B145	B150	BGP	ALP	0.065247	0
B145	B201	BGP	e-habits	-0.02391	2.10E-07
B146	B174	CysC	Urea/Cr	-0.0557	0
B146	B189	CysC	eGFB	-0.19826	0
B146	B190	CysC	Cre	0.274403	0
B146	B191	CysC	Urea	0.179065	0
B146	B192	CysC	UA	0.095235	0
B146	B195	CysC	Na	0.050335	0
B146	B200	CysC	OP	0.072231	0
B146	B220	CysC	SBP	0.039374	0
B146	B194	CysC	Cl	0.036657	1.78E-15
B146	B160	CysC	PH	-0.03557	1.15E-14
B146	B177	CysC	ACR	0.032497	1.72E-12
B146	B218	CysC	DBP	-0.03176	5.36E-12
B146	B150	CysC	ALP	0.027193	3.56E-09
B146	B228	CysC	OS	0.024869	6.71E-08
B146	B175	CysC	ALB-U	0.02348	3.45E-07
B146	B186	CysC	PFC	0.022735	8.00E-07
B148	B149	Anti_TPO	TGAb	0.480417	0
B148	B221	Anti_TPO	Hei	-0.04687	0
B148	B222	Anti_TPO	NS	-0.03963	0
B148	B204	Anti_TPO	Drinking	-0.03386	1.95E-13

B148	B216	Anti_TPO	BMI	-0.03298	8.07E-13
B148	B192	Anti_TPO	UA	-0.02692	5.06E-09
B148	B206	Anti_TPO	Waist	-0.02619	1.30E-08
B148	B163	Anti_TPO	SG	-0.02349	3.41E-07
B149	B216	TGAb	BMI	-0.04098	0
B149	B221	TGAb	Hei	-0.0455	0
B149	B222	TGAb	NS	-0.03972	0
B149	B204	TGAb	Drinking	-0.03274	1.17E-12
B149	B192	TGAb	UA	-0.02825	8.57E-10
B149	B206	TGAb	Waist	-0.02753	2.27E-09
B149	B209	TGAb	Smoking	-0.02705	4.31E-09
B149	B163	TGAb	SG	-0.02513	4.88E-08
B149	B174	TGAb	Urea/Cr	0.022605	9.24E-07
B15	B100	CD3-CD19+(B cell)	anti-Ro52	0.0658	0
B15	B103	CD3-CD19+(B cell)	anti-SSA/Ro	0.054423	0
B15	B106	CD3-CD19+(B cell)	anti-P	0.039246	0
B15	B107	CD3-CD19+(B cell)	AnuA	0.439816	0
B15	B119	CD3-CD19+(B cell)	Hcv-rna	0.117881	0
B15	B122	CD3-CD19+(B cell)	C4	0.051542	0
B15	B125	CD3-CD19+(B cell)	Cor	-0.0483	0
B15	B136	CD3-CD19+(B cell)	5'NT	0.047995	0
B15	B138	CD3-CD19+(B cell)	PAB	-0.09995	0
B15	B157	CD3-CD19+(B cell)	IgG	0.045303	0
B15	B158	CD3-CD19+(B cell)	IgM	0.044003	0
B15	B16	CD3-CD19+(B cell)	CD3+T	-0.09467	0
B15	B17	CD3-CD19+(B cell)	CD3+CD16+CD56+NK	-0.13696	0
B15	B18	CD3-CD19+(B cell)	CD3+CD4+helper(inducer)T	0.114633	0
B15	B181	CD3-CD19+(B cell)	APTT	0.099941	0
B15	B182	CD3-CD19+(B cell)	TT	0.056101	0
B15	B183	CD3-CD19+(B cell)	PTA	0.097817	0
B15	B184	CD3-CD19+(B cell)	INR	0.115354	0
B15	B185	CD3-CD19+(B cell)	PT	0.047916	0
B15	B186	CD3-CD19+(B cell)	PFC	-0.12454	0
B15	B19	CD3-CD19+(B cell)	CD3+CD4+/CD3+CD8+	0.197557	0
B15	B193	CD3-CD19+(B cell)	K	0.087969	0
B15	B194	CD3-CD19+(B cell)	Cl	0.074453	0
B15	B196	CD3-CD19+(B cell)	TCO2	-0.04079	0
B15	B198	CD3-CD19+(B cell)	P	0.045033	0
B15	B199	CD3-CD19+(B cell)	Mg	-0.05346	0
B15	B20	CD3-CD19+(B cell)	CD3+CD4+CD8+	-0.09839	0
B15	B21	CD3-CD19+(B cell)	CD3+CD4-CD8-	0.070818	0
B15	B22	CD3-CD19+(B cell)	CD3+CD8+(T)	-0.25419	0
B15	B23	CD3-CD19+(B cell)	CD3-CD16+CD56+(NK)	-0.23244	0
B15	B32	CD3-CD19+(B cell)	Testo	-0.05265	0

B15	B33	CD3-CD19+(B cell)	Prog	0.051788	0
B15	B79	CD3-CD19+(B cell)	hb V -dna	0.20053	0
B15	B92	CD3-CD19+(B cell)	ALDH2-Glu504Glu	-0.06633	0
B15	B93	CD3-CD19+(B cell)	ALDH2-Glu504Lys	0.050768	0
B15	B94	CD3-CD19+(B cell)	ALDH2-Lys504Lys	0.091063	0
B15	B96	CD3-CD19+(B cell)	dsDNA (IIF)	0.085257	0
B15	B99	CD3-CD19+(B cell)	anti-PM-Scl	-0.05178	0
B15	B212	CD3-CD19+(B cell)	IOP-L	-0.03678	1.33E-15
B15	B29	CD3-CD19+(B cell)	E2	0.036604	1.78E-15
B15	B189	CD3-CD19+(B cell)	eGFB	-0.03604	5.11E-15
B15	B64	CD3-CD19+(B cell)	WBRSR(mPas_5)	-0.03529	1.82E-14
B15	B40	CD3-CD19+(B cell)	WBC	0.034293	9.64E-14
B15	B98	CD3-CD19+(B cell)	anti-Jo-1	0.033773	2.26E-13
B15	B134	CD3-CD19+(B cell)	CHE	-0.03205	3.45E-12
B15	B69	CD3-CD19+(B cell)	PV	-0.03188	4.48E-12
B15	B65	CD3-CD19+(B cell)	WBRSR(mPas_1)	-0.03167	6.15E-12
B15	B42	CD3-CD19+(B cell)	MON	0.030648	2.86E-11
B15	B139	CD3-CD19+(B cell)	ADA	0.029525	1.46E-10
B15	B211	CD3-CD19+(B cell)	IOP-R	-0.02951	1.48E-10
B15	B86	CD3-CD19+(B cell)	TBhCG	0.029504	1.50E-10
B15	B76	CD3-CD19+(B cell)	ApoB	-0.02906	2.81E-10
B15	B222	CD3-CD19+(B cell)	NS	-0.02854	5.79E-10
B15	B45	CD3-CD19+(B cell)	MCHC	-0.02789	1.40E-09
B15	B104	CD3-CD19+(B cell)	anti-SSB	0.0262	1.29E-08
B15	B51	CD3-CD19+(B cell)	Hb	-0.02615	1.38E-08
B15	B204	CD3-CD19+(B cell)	Drinking	-0.02612	1.43E-08
B15	B197	CD3-CD19+(B cell)	AG	-0.02496	6.04E-08
B15	B90	CD3-CD19+(B cell)	CYFRA 21-1	-0.02481	7.19E-08
B15	B97	CD3-CD19+(B cell)	dsDNA	0.024754	7.71E-08
B15	B81	CD3-CD19+(B cell)	TSGF	-0.02456	9.78E-08
B15	B108	CD3-CD19+(B cell)	Anti-Cyclin	-0.0238	2.37E-07
B15	B60	CD3-CD19+(B cell)	WBRVI	-0.02299	5.99E-07
B150	B179	ALP	α1-MG	0.055586	0
B150	B186	ALP	PFC	0.041669	0
B150	B193	ALP	K	-0.04172	0
B150	B195	ALP	Na	0.076378	0
B150	B197	ALP	AG	0.05977	0
B150	B199	ALP	Mg	0.054464	0
B150	B200	ALP	OP	0.065499	0
B150	B221	ALP	Hei	-0.03081	2.26E-11
B150	B170	ALP	U-GLU	0.029376	1.80E-10
B150	B174	ALP	Urea/Cr	0.027238	3.36E-09
B150	B198	ALP	P	-0.02673	6.51E-09
B150	B155	ALP	IgA	0.026715	6.65E-09

B150	B216	ALP	BMI	-0.02626	1.20E-08
B151	B157	Anti-CCP	IgG	0.031934	4.12E-12
B152	B181	ACA-Ig(G-A-M)	APTT	-0.04662	0
B152	B182	ACA-Ig(G-A-M)	TT	-0.04497	0
B152	B183	ACA-Ig(G-A-M)	PTA	0.079601	0
B152	B156	ACA-Ig(G-A-M)	IgE	0.029088	2.70E-10
B152	B155	ACA-Ig(G-A-M)	IgA	0.026325	1.10E-08
B152	B194	ACA-Ig(G-A-M)	Cl	0.026038	1.58E-08
B152	B186	ACA-Ig(G-A-M)	PFC	0.022976	6.11E-07
B153	B160	Glu	PH	-0.07298	0
B153	B163	Glu	SG	0.107225	0
B153	B166	Glu	Pro	0.079095	0
B153	B170	Glu	U-GLU	0.513936	0
B153	B172	Glu	KB	0.07406	0
B153	B174	Glu	Urea/Cr	0.055293	0
B153	B180	Glu	Tf	0.040778	0
B153	B188	Glu	Ca	0.069559	0
B153	B194	Glu	Cl	-0.04444	0
B153	B206	Glu	Waist	0.138325	0
B153	B210	Glu	Hip	0.080769	0
B153	B215	Glu	BMI	0.118914	0
B153	B216	Glu	BMI	0.098025	0
B153	B220	Glu	SBP	0.096938	0
B153	B222	Glu	NS	0.09918	0
B153	B225	Glu	hPBG	0.061668	0
B153	B212	Glu	IOP-L	0.037197	6.66E-16
B153	B204	Glu	Drinking	0.035429	1.44E-14
B153	B178	Glu	Ig-U	0.034137	1.25E-13
B153	B211	Glu	IOP-R	0.033757	2.32E-13
B153	B179	Glu	α1-MG	0.033606	2.96E-13
B153	B177	Glu	ACR	0.033302	4.82E-13
B153	B191	Glu	Urea	0.030962	1.79E-11
B153	B218	Glu	DBP	0.030631	2.93E-11
B153	B195	Glu	Na	-0.02946	1.60E-10
B153	B175	Glu	ALB-U	0.027157	3.74E-09
B153	B200	Glu	OP	0.025471	3.21E-08
B154	B155	RF	IgA	0.023975	1.94E-07
B155	B157	IgA	IgG	0.283739	0
B155	B158	IgA	IgM	-0.0476	0
B155	B186	IgA	PFC	0.037988	0
B155	B156	IgA	IgE	0.033538	3.30E-13
B155	B178	IgA	Ig-U	0.023955	1.99E-07
B155	B209	IgA	Smoking	-0.02386	2.23E-07
B155	B194	IgA	Cl	-0.02333	4.08E-07

B156	B157	IgE	IgG	0.040895	0
B156	B193	IgE	K	0.046638	0
B156	B172	IgE	KB	0.025038	5.47E-08
B157	B193	IgG	K	0.046034	0
B157	B195	IgG	Na	-0.06284	0
B157	B200	IgG	OP	-0.03956	0
B157	B209	IgG	Smoking	-0.04333	0
B157	B221	IgG	Hei	-0.03735	4.44E-16
B157	B222	IgG	NS	-0.0375	4.44E-16
B157	B198	IgG	P	0.035355	1.64E-14
B157	B184	IgG	INR	0.03527	1.89E-14
B157	B204	IgG	Drinking	-0.03509	2.58E-14
B157	B197	IgG	AG	-0.03444	7.53E-14
B157	B216	IgG	BMI	-0.03124	1.18E-11
B157	B179	IgG	α1-MG	-0.03123	1.20E-11
B157	B185	IgG	PT	0.02478	7.47E-08
B157	B194	IgG	Cl	-0.02455	9.87E-08
B157	B183	IgG	PTA	0.024154	1.58E-07
B159	B186	β2-MG	PFC	0.034419	7.86E-14
B159	B182	β2-MG	TT	-0.03112	1.42E-11
B159	B183	β2-MG	PTA	-0.02425	1.40E-07
B16	B108	CD3+T	Anti-Cyclin	0.057579	0
B16	B138	CD3+T	PAB	0.044985	0
B16	B139	CD3+T	ADA	-0.04762	0
B16	B155	CD3+T	IgA	-0.03943	0
B16	B157	CD3+T	IgG	-0.09188	0
B16	B17	CD3+T	CD3+CD16+CD56+NK	0.162667	0
B16	B18	CD3+T	CD3+CD4+helper(inducer)T	0.456512	0
B16	B19	CD3+T	CD3+CD4+/CD3+CD8+	-0.17303	0
B16	B20	CD3+T	CD3+CD4+CD8+	0.143701	0
B16	B21	CD3+T	CD3+CD4-CD8-	0.169731	0
B16	B22	CD3+T	CD3+CD8+(T)	0.605682	0
B16	B23	CD3+T	CD3-CD16+CD56+(NK)	-0.61812	0
B16	B30	CD3+T	LH	0.040437	0
B16	B32	CD3+T	Testo	-0.08731	0
B16	B74	CD3+T	ApoA1	-0.04827	0
B16	B92	CD3+T	ALDH2-Glu504Glu	0.068408	0
B16	B93	CD3+T	ALDH2-Glu504Lys	-0.07013	0
B16	B104	CD3+T	anti-SSB	-0.03449	6.99E-14
B16	B54	CD3+T	ESR	-0.0328	1.07E-12
B16	B105	CD3+T	anti-U1RNP	-0.03188	4.49E-12
B16	B184	CD3+T	INR	-0.03125	1.17E-11
B16	B81	CD3+T	TSGF	0.027415	2.66E-09
B16	B96	CD3+T	dsDNA (IIF)	-0.02721	3.49E-09

B16	B185	CD3+T	PT	-0.02678	6.11E-09
B16	B58	CD3+T	HCT	-0.02555	2.92E-08
B16	B125	CD3+T	Cor	-0.02421	1.47E-07
B16	B31	CD3+T	FSH	0.024116	1.65E-07
B16	B28	CD3+T	PRL	0.023638	2.88E-07
B16	B176	CD3+T	Ucr	0.023451	3.57E-07
B16	B119	CD3+T	Hcv-rna	-0.02295	6.33E-07
B16	B100	CD3+T	anti-Ro52	-0.02276	7.77E-07
B160	B163	PH	SG	-0.19168	0
B160	B170	PH	U-GLU	-0.04764	0
B160	B174	PH	Urea/Cr	-0.04605	0
B160	B176	PH	Ucr	-0.08041	0
B160	B188	PH	Ca	-0.04522	0
B160	B189	PH	eGFB	0.062311	0
B160	B190	PH	Cre	-0.08959	0
B160	B191	PH	Urea	-0.17333	0
B160	B192	PH	UA	-0.16695	0
B160	B206	PH	Waist	-0.08462	0
B160	B209	PH	Smoking	-0.07087	0
B160	B210	PH	Hip	-0.06277	0
B160	B215	PH	BMI	-0.06142	0
B160	B216	PH	BMI	-0.0676	0
B160	B218	PH	DBP	0.055755	0
B160	B220	PH	SBP	0.064866	0
B160	B221	PH	Hei	-0.04052	0
B160	B222	PH	NS	-0.07102	0
B160	B204	PH	Drinking	-0.0373	4.44E-16
B160	B198	PH	P	-0.03547	1.35E-14
B160	B166	PH	Pro	-0.03405	1.44E-13
B160	B193	PH	K	-0.0339	1.83E-13
B160	B194	PH	Cl	-0.03133	1.04E-11
B160	B196	PH	TCO2	0.030083	6.53E-11
B160	B168	PH	NMP22	0.02525	4.22E-08
B161	B163	LEU	SG	-0.04887	0
B161	B173	LEU	NTT	0.170839	0
B161	B174	LEU	Urea/Cr	0.099057	0
B161	B190	LEU	Cre	-0.04438	0
B161	B192	LEU	UA	-0.06959	0
B161	B204	LEU	Drinking	-0.0628	0
B161	B206	LEU	Waist	-0.04403	0
B161	B209	LEU	Smoking	-0.06035	0
B161	B216	LEU	BMI	-0.09582	0
B161	B218	LEU	DBP	-0.0425	0
B161	B221	LEU	Hei	-0.15606	0

B161	B222	LEU	NS	-0.1216	0
B161	B219	LEU	OT	0.036818	1.33E-15
B161	B166	LEU	Pro	0.031343	1.01E-11
B161	B210	LEU	Hip	-0.02821	9.14E-10
B161	B189	LEU	eGFB	-0.0275	2.38E-09
B161	B201	LEU	e-habits	-0.02378	2.44E-07
B163	B166	SG	Pro	0.321912	0
B163	B170	SG	U-GLU	0.119404	0
B163	B172	SG	KB	0.075096	0
B163	B173	SG	NTT	-0.0539	0
B163	B176	SG	Ucr	0.285825	0
B163	B179	SG	α1-MG	0.060563	0
B163	B188	SG	Ca	-0.04734	0
B163	B189	SG	eGFB	0.05877	0
B163	B190	SG	Cre	0.095175	0
B163	B191	SG	Urea	0.174321	0
B163	B192	SG	UA	0.159515	0
B163	B204	SG	Drinking	0.163182	0
B163	B205	SG	KDA	-0.04552	0
B163	B206	SG	Waist	0.217039	0
B163	B209	SG	Smoking	0.18082	0
B163	B210	SG	Hip	0.159358	0
B163	B215	SG	BMI	0.151395	0
B163	B216	SG	BMI	0.2864	0
B163	B218	SG	DBP	0.095031	0
B163	B219	SG	OT	-0.07261	0
B163	B220	SG	SBP	-0.08025	0
B163	B221	SG	Hei	0.295616	0
B163	B222	SG	NS	0.311398	0
B163	B201	SG	e-habits	-0.0319	4.34E-12
B163	B165	SG	bilirubin	0.03108	1.50E-11
B163	B203	SG	DH	0.028864	3.70E-10
B163	B198	SG	P	-0.023	5.93E-07
B163	B178	SG	Ig-U	0.022821	7.27E-07
B165	B172	bilirubin	KB	0.039154	0
B166	B170	Pro	U-GLU	0.076825	0
B166	B172	Pro	KB	0.05263	0
B166	B174	Pro	Urea/Cr	-0.04696	0
B166	B175	Pro	ALB-U	0.047301	0
B166	B176	Pro	Ucr	0.128767	0
B166	B179	Pro	α1-MG	0.039013	0
B166	B189	Pro	eGFB	-0.0618	0
B166	B190	Pro	Cre	0.099287	0
B166	B191	Pro	Urea	0.080083	0

B166	B192	Pro	UA	0.08385	0
B166	B204	Pro	Drinking	0.055173	0
B166	B206	Pro	Waist	0.093623	0
B166	B209	Pro	Smoking	0.086151	0
B166	B210	Pro	Hip	0.055265	0
B166	B215	Pro	BMI	0.062949	0
B166	B216	Pro	BMI	0.104501	0
B166	B218	Pro	DBP	0.054078	0
B166	B221	Pro	Hei	0.097571	0
B166	B222	Pro	NS	0.121801	0
B166	B177	Pro	ACR	0.036813	1.33E-15
B166	B188	Pro	Ca	-0.0348	4.20E-14
B166	B220	Pro	SBP	0.032763	1.13E-12
B166	B178	Pro	Ig-U	0.028269	8.41E-10
B166	B203	Pro	DH	0.024102	1.68E-07
B168	B183	NMP22	PTA	0.037927	2.22E-16
B168	B173	NMP22	NTT	0.031065	1.54E-11
B168	B193	NMP22	K	-0.02877	4.23E-10
B169	B196	U-CRY	TCO2	0.023381	3.86E-07
B17	B136	CD3+CD16+CD56+NK	5'NT	-0.04081	0
B17	B18	CD3+CD16+CD56+NK	CD3+CD4+helper(inducer)T	-0.16747	0
B17	B182	CD3+CD16+CD56+NK	TT	-0.08322	0
B17	B183	CD3+CD16+CD56+NK	PTA	0.061179	0
B17	B19	CD3+CD16+CD56+NK	CD3+CD4+/CD3+CD8+	-0.24224	0
B17	B20	CD3+CD16+CD56+NK	CD3+CD4+CD8+	0.072881	0
B17	B21	CD3+CD16+CD56+NK	CD3+CD4-CD8-	0.259926	0
B17	B22	CD3+CD16+CD56+NK	CD3+CD8+(T)	0.269105	0
B17	B23	CD3+CD16+CD56+NK	CD3-CD16+CD56+(NK)	-0.06483	0
B17	B95	CD3+CD16+CD56+NK	anti-cenpb	-0.0426	0
B17	B99	CD3+CD16+CD56+NK	anti-PM-Scl	-0.06513	0
B17	B137	CD3+CD16+CD56+NK	AFU	-0.03236	2.12E-12
B17	B186	CD3+CD16+CD56+NK	PFC	0.032341	2.19E-12
B17	B125	CD3+CD16+CD56+NK	Cor	0.032013	3.65E-12
B17	B181	CD3+CD16+CD56+NK	APTT	0.031655	6.31E-12
B17	B76	CD3+CD16+CD56+NK	ApoB	0.027619	2.02E-09
B17	B105	CD3+CD16+CD56+NK	anti-U1RNP	0.026864	5.48E-09
B17	B139	CD3+CD16+CD56+NK	ADA	-0.02578	2.19E-08
B17	B135	CD3+CD16+CD56+NK	TBA	-0.02324	4.55E-07
B170	B172	U-GLU	KB	0.091254	0
B170	B180	U-GLU	Tf	0.040283	0
B170	B192	U-GLU	UA	-0.03971	0
B170	B204	U-GLU	Drinking	0.037976	0
B170	B206	U-GLU	Waist	0.055683	0
B170	B209	U-GLU	Smoking	0.046635	0

B170	B216	U-GLU	BMI	0.049388	0
B170	B221	U-GLU	Hei	0.03985	0
B170	B222	U-GLU	NS	0.059792	0
B170	B225	U-GLU	hPBG	0.037665	4.44E-16
B170	B215	U-GLU	BMI	0.035249	1.95E-14
B170	B194	U-GLU	Cl	-0.03512	2.42E-14
B170	B178	U-GLU	Ig-U	0.03473	4.69E-14
B170	B218	U-GLU	DBP	0.034012	1.53E-13
B170	B220	U-GLU	SBP	0.031496	8.03E-12
B170	B179	U-GLU	α1-MG	0.030623	2.97E-11
B170	B189	U-GLU	eGFB	0.02934	1.89E-10
B170	B177	U-GLU	ACR	0.028679	4.78E-10
B170	B191	U-GLU	Urea	0.0263	1.13E-08
B170	B174	U-GLU	Urea/Cr	0.025653	2.56E-08
B170	B175	U-GLU	ALB-U	0.025039	5.46E-08
B172	B204	KB	Drinking	0.030439	3.89E-11
B172	B188	KB	Ca	0.028183	9.45E-10
B172	B176	KB	Ucr	0.027342	2.92E-09
B172	B189	KB	eGFB	0.026845	5.62E-09
B173	B174	NTT	Urea/Cr	0.054511	0
B173	B189	NTT	eGFB	-0.05707	0
B173	B204	NTT	Drinking	-0.04258	0
B173	B209	NTT	Smoking	-0.04148	0
B173	B216	NTT	BMI	-0.07475	0
B173	B221	NTT	Hei	-0.09571	0
B173	B222	NTT	NS	-0.07996	0
B173	B218	NTT	DBP	-0.03622	3.55E-15
B173	B192	NTT	UA	-0.03245	1.84E-12
B173	B206	NTT	Waist	-0.03241	1.96E-12
B173	B191	NTT	Urea	0.030391	4.17E-11
B173	B176	NTT	Ucr	-0.03036	4.39E-11
B173	B210	NTT	Hip	-0.03031	4.67E-11
B173	B220	NTT	SBP	0.025759	2.25E-08
B173	B215	NTT	BMI	-0.02517	4.67E-08
B173	B219	NTT	OT	0.023957	1.99E-07
B174	B176	Urea/Cr	Ucr	-0.06917	0
B174	B189	Urea/Cr	eGFB	0.18244	0
B174	B190	Urea/Cr	Cre	-0.32958	0
B174	B191	Urea/Cr	Urea	0.492792	0
B174	B192	Urea/Cr	UA	-0.25096	0
B174	B198	Urea/Cr	P	0.042676	0
B174	B200	Urea/Cr	OP	0.046807	0
B174	B204	Urea/Cr	Drinking	-0.13302	0
B174	B206	Urea/Cr	Waist	-0.12498	0

B174	B209	Urea/Cr	Smoking	-0.13893	0
B174	B210	Urea/Cr	Hip	-0.10316	0
B174	B215	Urea/Cr	BMI	-0.04082	0
B174	B216	Urea/Cr	BMI	-0.23203	0
B174	B218	Urea/Cr	DBP	-0.07828	0
B174	B219	Urea/Cr	OT	0.056075	0
B174	B220	Urea/Cr	SBP	0.041228	0
B174	B221	Urea/Cr	Hei	-0.33345	0
B174	B222	Urea/Cr	NS	-0.27261	0
B174	B188	Urea/Cr	Ca	0.022577	9.54E-07
B175	B176	ALB-U	Ucr	0.084365	0
B175	B177	ALB-U	ACR	0.792501	0
B175	B178	ALB-U	Ig-U	0.118585	0
B175	B179	ALB-U	α1-MG	0.057124	0
B175	B180	ALB-U	Tf	0.110939	0
B175	B189	ALB-U	eGFB	-0.06568	0
B175	B190	ALB-U	Cre	0.114583	0
B175	B191	ALB-U	Urea	0.073086	0
B175	B192	ALB-U	UA	0.039827	0
B175	B218	ALB-U	DBP	0.044999	0
B175	B220	ALB-U	SBP	0.040491	0
B175	B187	ALB-U	NSE	0.033168	5.97E-13
B175	B198	ALB-U	P	0.029456	1.61E-10
B175	B216	ALB-U	BMI	0.028949	3.28E-10
B175	B206	ALB-U	Waist	0.027592	2.10E-09
B175	B222	ALB-U	NS	0.026164	1.35E-08
B175	B215	ALB-U	BMI	0.025756	2.25E-08
B176	B177	Ucr	ACR	-0.059	0
B176	B178	Ucr	Ig-U	0.073756	0
B176	B179	Ucr	α1-MG	0.162156	0
B176	B190	Ucr	Cre	0.05094	0
B176	B192	Ucr	UA	0.08102	0
B176	B204	Ucr	Drinking	0.058543	0
B176	B206	Ucr	Waist	0.078324	0
B176	B209	Ucr	Smoking	0.072424	0
B176	B210	Ucr	Hip	0.06406	0
B176	B215	Ucr	BMI	0.058082	0
B176	B216	Ucr	BMI	0.119513	0
B176	B218	Ucr	DBP	0.040657	0
B176	B220	Ucr	SBP	-0.04897	0
B176	B221	Ucr	Hei	0.12591	0
B176	B222	Ucr	NS	0.128483	0
B176	B201	Ucr	e-habits	-0.03783	2.22E-16
B176	B219	Ucr	OT	-0.0345	6.82E-14

B176	B193	Ucr	K	-0.03398	1.60E-13
B176	B180	Ucr	Tf	0.030644	2.87E-11
B176	B198	Ucr	P	-0.0236	3.02E-07
B177	B178	ACR	Ig-U	0.217913	0
B177	B179	ACR	α1-MG	0.074194	0
B177	B180	ACR	Tf	0.257501	0
B177	B189	ACR	eGFB	-0.06192	0
B177	B190	ACR	Cre	0.108365	0
B177	B191	ACR	Urea	0.081464	0
B177	B198	ACR	P	0.038441	0
B177	B220	ACR	SBP	0.048348	0
B177	B218	ACR	DBP	0.031197	1.26E-11
B177	B192	ACR	UA	0.025754	2.26E-08
B178	B179	Ig-U	α1-MG	0.423824	0
B178	B180	Ig-U	Tf	0.881563	0
B178	B187	Ig-U	NSE	0.048409	0
B178	B193	Ig-U	K	-0.03247	1.78E-12
B178	B186	Ig-U	PFC	0.028039	1.15E-09
B178	B194	Ig-U	Cl	-0.02365	2.84E-07
B179	B180	α1-MG	Tf	0.351159	0
B179	B182	α1-MG	TT	0.058	0
B179	B193	α1-MG	K	-0.0693	0
B179	B198	α1-MG	P	-0.03821	0
B179	B225	α1-MG	hPBG	0.04598	0
B179	B181	α1-MG	APTT	0.03675	1.33E-15
B179	B183	α1-MG	PTA	-0.03612	4.44E-15
B179	B209	α1-MG	Smoking	0.029393	1.76E-10
B179	B221	α1-MG	Hei	0.026793	6.01E-09
B179	B190	α1-MG	Cre	0.025798	2.14E-08
B179	B200	α1-MG	OP	0.025006	5.68E-08
B179	B191	α1-MG	Urea	0.022613	9.16E-07
B18	B104	CD3+CD4+helper(inducer)T	anti-SSB	-0.05025	0
B18	B107	CD3+CD4+helper(inducer)T	AnuA	-0.04646	0
B18	B108	CD3+CD4+helper(inducer)T	Anti-Cyclin	0.04815	0
B18	B110	CD3+CD4+helper(inducer)T	AHA	0.046148	0
B18	B122	CD3+CD4+helper(inducer)T	C4	0.052849	0
B18	B125	CD3+CD4+helper(inducer)T	Cor	-0.05464	0
B18	B136	CD3+CD4+helper(inducer)T	5'NT	0.041816	0
B18	B157	CD3+CD4+helper(inducer)T	IgG	-0.06273	0
B18	B183	CD3+CD4+helper(inducer)T	PTA	-0.04519	0
B18	B186	CD3+CD4+helper(inducer)T	PFC	0.046977	0
B18	B19	CD3+CD4+helper(inducer)T	CD3+CD4+/CD3+CD8+	0.657733	0
B18	B193	CD3+CD4+helper(inducer)T	K	-0.04124	0
B18	B20	CD3+CD4+helper(inducer)T	CD3+CD4+CD8+	0.109364	0

B18	B21	CD3+CD4+helper(inducer)T	CD3+CD4-CD8-	-0.12315	0
B18	B22	CD3+CD4+helper(inducer)T	CD3+CD8+(T)	-0.33889	0
B18	B23	CD3+CD4+helper(inducer)T	CD3-CD16+CD56+(NK)	-0.34513	0
B18	B32	CD3+CD4+helper(inducer)T	Testo	-0.05826	0
B18	B79	CD3+CD4+helper(inducer)T	hb V -dna	-0.05031	0
B18	B93	CD3+CD4+helper(inducer)T	ALDH2-Glu504Lys	-0.03976	0
B18	B98	CD3+CD4+helper(inducer)T	anti-Jo-1	-0.04621	0
B18	B99	CD3+CD4+helper(inducer)T	anti-PM-Scl	0.039911	0
B18	B92	CD3+CD4+helper(inducer)T	ALDH2-Glu504Glu	0.037955	2.22E-16
B18	B198	CD3+CD4+helper(inducer)T	P	0.036425	2.66E-15
B18	B156	CD3+CD4+helper(inducer)T	IgE	0.036155	4.00E-15
B18	B195	CD3+CD4+helper(inducer)T	Na	0.036074	4.88E-15
B18	B194	CD3+CD4+helper(inducer)T	Cl	0.03501	2.93E-14
B18	B97	CD3+CD4+helper(inducer)T	dsDNA	-0.03482	4.02E-14
B18	B138	CD3+CD4+helper(inducer)T	PAB	-0.03307	6.99E-13
B18	B196	CD3+CD4+helper(inducer)T	TCO2	-0.03207	3.35E-12
B18	B74	CD3+CD4+helper(inducer)T	ApoA1	-0.03191	4.29E-12
B18	B95	CD3+CD4+helper(inducer)T	anti-cenpb	0.0317	5.89E-12
B18	B30	CD3+CD4+helper(inducer)T	LH	0.026895	5.26E-09
B18	B197	CD3+CD4+helper(inducer)T	AG	0.024818	7.14E-08
B18	B123	CD3+CD4+helper(inducer)T	hs-CRP1	0.024013	1.86E-07
B18	B184	CD3+CD4+helper(inducer)T	INR	-0.02387	2.20E-07
B18	B28	CD3+CD4+helper(inducer)T	PRL	0.0229	6.66E-07
B18	B124	CD3+CD4+helper(inducer)T	ACTH	-0.02279	7.55E-07
B18	B75	CD3+CD4+helper(inducer)T	ApoA1/B	-0.02269	8.45E-07
B180	B187	Tf	NSE	0.053879	0
B180	B186	Tf	PFC	0.032851	9.87E-13
B181	B182	APTT	TT	0.099627	0
B181	B183	APTT	PTA	0.069529	0
B181	B184	APTT	INR	0.501398	0
B181	B185	APTT	PT	0.462767	0
B181	B186	APTT	PFC	-0.04954	0
B181	B194	APTT	Cl	0.033494	3.54E-13
B181	B197	APTT	AG	-0.03329	4.92E-13
B181	B193	APTT	K	0.030528	3.41E-11
B182	B183	TT	PTA	-0.20101	0
B182	B184	TT	INR	-0.04572	0
B182	B186	TT	PFC	-0.21609	0
B182	B194	TT	Cl	-0.04799	0
B182	B195	TT	Na	0.060301	0
B182	B197	TT	AG	0.071067	0
B182	B200	TT	OP	0.037293	4.44E-16
B182	B199	TT	Mg	0.027241	3.34E-09
B182	B198	TT	P	-0.02371	2.66E-07

B183	B184	PTA	INR	0.115539	0
B183	B186	PTA	PFC	-0.17544	0
B183	B194	PTA	Cl	0.045934	0
B183	B197	PTA	AG	-0.07086	0
B183	B198	PTA	P	-0.03137	9.78E-12
B183	B196	PTA	TCO2	0.028905	3.49E-10
B183	B195	PTA	Na	-0.02372	2.60E-07
B183	B200	PTA	OP	-0.02261	9.19E-07
B184	B185	INR	PT	0.945881	0
B184	B186	INR	PFC	-0.06959	0
B184	B193	INR	K	0.040335	0
B184	B195	INR	Na	-0.04417	0
B184	B197	INR	AG	-0.03753	4.44E-16
B184	B189	INR	eGFB	-0.03644	2.66E-15
B184	B200	INR	OP	-0.03575	8.44E-15
B184	B199	INR	Mg	-0.02734	2.92E-09
B184	B194	INR	Cl	0.022674	8.56E-07
B185	B186	PT	PFC	0.047293	0
B185	B195	PT	Na	-0.02355	3.19E-07
B185	B189	PT	eGFB	-0.0233	4.23E-07
B186	B193	PFC	K	-0.04717	0
B186	B194	PFC	Cl	-0.06659	0
B186	B198	PFC	P	0.047584	0
B186	B199	PFC	Mg	0.03997	0
B186	B197	PFC	AG	0.036318	3.11E-15
B187	B225	NSE	hPBG	-0.03264	1.37E-12
B187	B194	NSE	Cl	-0.03247	1.80E-12
B187	B193	NSE	K	-0.02666	7.15E-09
B188	B190	Ca	Cre	-0.03868	0
B188	B192	Ca	UA	0.085267	0
B188	B193	Ca	K	0.060831	0
B188	B194	Ca	Cl	-0.0447	0
B188	B211	Ca	IOP-R	0.037671	4.44E-16
B188	B206	Ca	Waist	-0.03498	3.06E-14
B188	B212	Ca	IOP-L	0.033515	3.43E-13
B188	B222	Ca	NS	-0.03264	1.37E-12
B188	B197	Ca	AG	0.031638	6.47E-12
B188	B216	Ca	BMI	-0.03106	1.54E-11
B188	B218	Ca	DBP	0.030295	4.80E-11
B188	B198	Ca	P	0.027273	3.21E-09
B188	B201	Ca	e-habits	0.027161	3.71E-09
B188	B215	Ca	BMI	-0.02569	2.45E-08
B188	B221	Ca	Hei	-0.02435	1.24E-07
B189	B190	eGFB	Cre	-0.50678	0

B189	B191	eGFB	Urea	-0.42399	0
B189	B192	eGFB	UA	-0.23658	0
B189	B193	eGFB	K	-0.04264	0
B189	B203	eGFB	DH	0.039829	0
B189	B204	eGFB	Drinking	0.104047	0
B189	B209	eGFB	Smoking	0.068922	0
B189	B210	eGFB	Hip	0.10232	0
B189	B212	eGFB	IOP-L	0.039446	0
B189	B215	eGFB	BMI	0.098161	0
B189	B216	eGFB	BMI	0.136396	0
B189	B218	eGFB	DBP	0.187327	0
B189	B219	eGFB	OT	-0.05556	0
B189	B220	eGFB	SBP	-0.13501	0
B189	B221	eGFB	Hei	0.107677	0
B189	B222	eGFB	NS	0.05857	0
B189	B211	eGFB	IOP-R	0.037641	4.44E-16
B189	B227	eGFB	OD	-0.03551	1.27E-14
B189	B228	eGFB	OS	-0.03262	1.42E-12
B189	B201	eGFB	e-habits	-0.03179	5.13E-12
B189	B194	eGFB	Cl	-0.02831	7.89E-10
B189	B205	eGFB	KDA	-0.0257	2.42E-08
B189	B224	eGFB	pmh	0.025693	2.44E-08
B19	B102	CD3+CD4+/CD3+CD8+	anti-Sm	0.051509	0
B19	B105	CD3+CD4+/CD3+CD8+	anti-U1RNP	0.039565	0
B19	B110	CD3+CD4+/CD3+CD8+	AHA	0.077427	0
B19	B122	CD3+CD4+/CD3+CD8+	C4	0.058347	0
B19	B136	CD3+CD4+/CD3+CD8+	5'NT	0.041193	0
B19	B138	CD3+CD4+/CD3+CD8+	PAB	-0.06562	0
B19	B139	CD3+CD4+/CD3+CD8+	ADA	0.041654	0
B19	B156	CD3+CD4+/CD3+CD8+	IgE	0.049758	0
B19	B183	CD3+CD4+/CD3+CD8+	PTA	-0.04008	0
B19	B20	CD3+CD4+/CD3+CD8+	CD3+CD4+CD8+	-0.08073	0
B19	B21	CD3+CD4+/CD3+CD8+	CD3+CD4-CD8-	-0.05772	0
B19	B22	CD3+CD4+/CD3+CD8+	CD3+CD8+(T)	-0.77321	0
B19	B23	CD3+CD4+/CD3+CD8+	CD3-CD16+CD56+(NK)	0.105484	0
B19	B58	CD3+CD4+/CD3+CD8+	HCT	0.058853	0
B19	B79	CD3+CD4+/CD3+CD8+	hb V -dna	-0.03965	0
B19	B98	CD3+CD4+/CD3+CD8+	anti-Jo-1	-0.04966	0
B19	B104	CD3+CD4+/CD3+CD8+	anti-SSB	-0.03431	9.41E-14
B19	B81	CD3+CD4+/CD3+CD8+	TSGF	-0.03423	1.07E-13
B19	B54	CD3+CD4+/CD3+CD8+	ESR	0.032468	1.80E-12
B19	B195	CD3+CD4+/CD3+CD8+	Na	0.031509	7.87E-12
B19	B134	CD3+CD4+/CD3+CD8+	CHE	-0.03098	1.76E-11
B19	B186	CD3+CD4+/CD3+CD8+	PFC	0.030434	3.92E-11

B19	B107	CD3+CD4+/CD3+CD8+	AnuA	-0.02983	9.40E-11
B19	B95	CD3+CD4+/CD3+CD8+	anti-cenpb	0.028554	5.68E-10
B19	B56	CD3+CD4+/CD3+CD8+	IR	-0.02843	6.71E-10
B19	B155	CD3+CD4+/CD3+CD8+	IgA	0.027126	3.89E-09
B19	B32	CD3+CD4+/CD3+CD8+	Testo	-0.02711	3.99E-09
B19	B62	CD3+CD4+/CD3+CD8+	WBHSRVI	-0.0265	8.82E-09
B19	B198	CD3+CD4+/CD3+CD8+	P	0.025792	2.15E-08
B19	B137	CD3+CD4+/CD3+CD8+	AFU	-0.02503	5.53E-08
B19	B61	CD3+CD4+/CD3+CD8+	WBHSRV	-0.02429	1.34E-07
B19	B60	CD3+CD4+/CD3+CD8+	WBRVI	-0.02345	3.57E-07
B19	B99	CD3+CD4+/CD3+CD8+	anti-PM-Scl	0.02336	3.96E-07
B19	B55	CD3+CD4+/CD3+CD8+	TK	-0.02328	4.33E-07
B19	B119	CD3+CD4+/CD3+CD8+	Hcv-rna	-0.02315	5.05E-07
B19	B69	CD3+CD4+/CD3+CD8+	PV	0.022953	6.27E-07
B190	B191	Cre	Urea	0.524005	0
B190	B192	Cre	UA	0.34191	0
B190	B204	Cre	Drinking	0.052938	0
B190	B206	Cre	Waist	0.106204	0
B190	B209	Cre	Smoking	0.073678	0
B190	B210	Cre	Hip	0.052268	0
B190	B216	Cre	BMI	0.135614	0
B190	B221	Cre	Hei	0.196191	0
B190	B222	Cre	NS	0.191996	0
B190	B219	Cre	OT	-0.03332	4.66E-13
B190	B224	Cre	pmh	0.025841	2.03E-08
B190	B215	Cre	BMI	0.024165	1.56E-07
B191	B192	Urea	UA	0.210353	0
B191	B200	Urea	OP	0.058142	0
B191	B204	Urea	Drinking	-0.04011	0
B191	B218	Urea	DBP	-0.07389	0
B191	B220	Urea	SBP	0.038126	0
B191	B221	Urea	Hei	-0.03963	0
B191	B216	Urea	BMI	-0.0314	9.23E-12
B191	B211	Urea	IOP-R	-0.02825	8.65E-10
B191	B206	Urea	Waist	0.028169	9.64E-10
B191	B209	Urea	Smoking	-0.02685	5.57E-09
B191	B212	Urea	IOP-L	-0.02368	2.75E-07
B192	B203	UA	DH	0.046149	0
B192	B204	UA	Drinking	0.179036	0
B192	B206	UA	Waist	0.306236	0
B192	B209	UA	Smoking	0.137573	0
B192	B210	UA	Hip	0.218911	0
B192	B215	UA	BMI	0.237918	0
B192	B216	UA	BMI	0.350447	0

B192	B218	UA	DBP	0.072648	0
B192	B219	UA	OT	-0.05602	0
B192	B221	UA	Hei	0.298414	0
B192	B222	UA	NS	0.366099	0
B192	B220	UA	SBP	-0.02685	5.56E-09
B192	B207	UA	WHR	0.025659	2.54E-08
B193	B194	K	Cl	0.082736	0
B193	B195	K	Na	-0.10703	0
B193	B196	K	TCO2	-0.042	0
B193	B197	K	AG	-0.08886	0
B193	B198	K	P	0.14843	0
B193	B200	K	OP	-0.05786	0
B193	B204	K	Drinking	-0.03826	0
B193	B216	K	BMI	-0.03868	0
B193	B222	K	NS	-0.03788	2.22E-16
B193	B221	K	Hei	-0.03075	2.47E-11
B193	B206	K	Waist	-0.02847	6.34E-10
B193	B215	K	BMI	-0.0273	3.09E-09
B193	B218	K	DBP	-0.0271	4.01E-09
B194	B195	Cl	Na	0.311991	0
B194	B196	Cl	TCO2	-0.18314	0
B194	B197	Cl	AG	-0.36172	0
B194	B198	Cl	P	-0.03983	0
B194	B200	Cl	OP	0.160852	0
B195	B196	Na	TCO2	0.075184	0
B195	B197	Na	AG	0.33613	0
B195	B199	Na	Mg	0.129475	0
B195	B200	Na	OP	0.579329	0
B195	B198	Na	P	-0.03484	3.91E-14
B195	B207	Na	WHR	0.023698	2.68E-07
B196	B197	TCO2	AG	-0.64905	0
B196	B200	TCO2	OP	0.058942	0
B197	B199	AG	Mg	0.085774	0
B197	B200	AG	OP	0.196241	0
B198	B216	P	BMI	-0.03931	0
B198	B221	P	Hei	-0.0539	0
B198	B222	P	NS	-0.05108	0
B198	B204	P	Drinking	-0.03591	6.22E-15
B198	B206	P	Waist	-0.03118	1.30E-11
B198	B200	P	OP	-0.02832	7.84E-10
B199	B200	Mg	OP	0.077148	0
B2	B107	Age	AnuA	0.062409	0
B2	B113	Age	FT3	-0.06627	0
B2	B115	Age	TSH TT3 TT4(TT3)	-0.04231	0

B2	B119	Age	Hcv-rna	0.044276	0
B2	B12	Age	PG II	0.042263	0
B2	B126	Age	Alb	-0.28205	0
B2	B127	Age	A-G ratio	-0.20823	0
B2	B128	Age	ALT	-0.26433	0
B2	B130	Age	GLB	0.128224	0
B2	B131	Age	AST	-0.04948	0
B2	B132	Age	Dbil	0.082916	0
B2	B134	Age	CHE	-0.08009	0
B2	B138	Age	PAB	-0.07638	0
B2	B139	Age	ADA	0.049542	0
B2	B140	Age	r-GT	-0.15359	0
B2	B141	Age	AST/ALT	0.337023	0
B2	B146	Age	CysC	0.121262	0
B2	B153	Age	Glu	0.044062	0
B2	B161	Age	LEU	0.06433	0
B2	B163	Age	SG	-0.14265	0
B2	B173	Age	NTT	0.082897	0
B2	B174	Age	Urea/Cr	0.081079	0
B2	B176	Age	Ucr	-0.0908	0
B2	B188	Age	Ca	-0.07707	0
B2	B189	Age	eGFB	-0.52421	0
B2	B190	Age	Cre	0.078873	0
B2	B191	Age	Urea	0.199046	0
B2	B192	Age	UA	-0.07875	0
B2	B193	Age	K	0.040464	0
B2	B201	Age	e-habits	0.061812	0
B2	B203	Age	DH	-0.08528	0
B2	B204	Age	Drinking	-0.1954	0
B2	B205	Age	KDA	0.063246	0
B2	B206	Age	Waist	-0.10544	0
B2	B209	Age	Smoking	-0.15181	0
B2	B210	Age	Hip	-0.20832	0
B2	B211	Age	IOP-R	-0.07278	0
B2	B212	Age	IOP-L	-0.06948	0
B2	B215	Age	BMI	-0.22875	0
B2	B216	Age	BMI	-0.37465	0
B2	B218	Age	DBP	-0.3729	0
B2	B219	Age	OT	0.124826	0
B2	B220	Age	SBP	0.210202	0
B2	B221	Age	Hei	-0.33981	0
B2	B222	Age	NS	-0.23789	0
B2	B227	Age	OD	0.053492	0
B2	B228	Age	OS	0.068843	0

B2	B24	Age	αHBDH	0.056877	0
B2	B27	Age	LDH	0.06258	0
B2	B40	Age	WBC	-0.05389	0
B2	B42	Age	MON	0.077687	0
B2	B43	Age	MCV	0.150143	0
B2	B44	Age	MCH	0.050639	0
B2	B45	Age	MCHC	-0.16853	0
B2	B46	Age	RBC Count	-0.30167	0
B2	B47	Age	RDW	0.23361	0
B2	B50	Age	eo%	0.052806	0
B2	B51	Age	Hb	-0.28533	0
B2	B52	Age	PLT/BPC	-0.11319	0
B2	B62	Age	WBHSRVI	-0.04805	0
B2	B63	Age	WBRSR(mPas_200)	-0.0402	0
B2	B64	Age	WBRSR(mPas_5)	-0.03864	0
B2	B66	Age	WBRSR(mPas_100)	-0.04295	0
B2	B67	Age	WBRSR(mPas_50)	-0.04257	0
B2	B71	Age	TG	-0.20307	0
B2	B72	Age	HDL-C	0.197179	0
B2	B74	Age	ApoA1	0.064409	0
B2	B75	Age	ApoA1/B	0.051887	0
B2	B79	Age	hb V -dna	0.064807	0
B2	B86	Age	TBhCG	0.056099	0
B2	B90	Age	CYFRA 21-1	0.052121	0
B2	B91	Age	TP	-0.05193	0
B2	B15	Age	CD3-CD19+(B cell)	0.036713	1.55E-15
B2	B60	Age	WBRVI	-0.03628	3.33E-15
B2	B56	Age	IR	-0.03562	1.02E-14
B2	B224	Age	pmh	0.035453	1.38E-14
B2	B3	Age	Hcy	0.035403	1.51E-14
B2	B184	Age	INR	0.035306	1.78E-14
B2	B54	Age	ESR	0.034989	3.04E-14
B2	B111	Age	TSH	0.03405	1.44E-13
B2	B70	Age	LDL-C	-0.03385	1.99E-13
B2	B65	Age	WBRSR(mPas_1)	-0.03373	2.41E-13
B2	B142	Age	25-(OH)D	0.033441	3.85E-13
B2	B61	Age	WBHSRV	-0.03309	6.72E-13
B2	B133	Age	TBIL	0.032162	2.90E-12
B2	B77	Age	TC	-0.03201	3.68E-12
B2	B149	Age	TGAb	0.031367	9.77E-12
B2	B49	Age	baso%	-0.03115	1.35E-11
B2	B157	Age	IgG	0.030777	2.36E-11
B2	B118	Age	CEA1	0.02965	1.22E-10
B2	B10	Age	PG I	0.028164	9.70E-10

B2	B84	Age	C-PSA	0.02571	2.39E-08
B2	B76	Age	ApoB	-0.02562	2.65E-08
B2	B53	Age	GR%	-0.02539	3.55E-08
B2	B172	Age	KB	-0.02517	4.67E-08
B2	B48	Age	LYM%	-0.02389	2.15E-07
B2	B144	Age	Total-PINP	-0.02363	2.90E-07
B2	B185	Age	PT	0.023501	3.37E-07
B2	B73	Age	ApoA	0.023318	4.15E-07
B20	B107	CD3+CD4+CD8+	AnuA	-0.06114	0
B20	B21	CD3+CD4+CD8+	CD3+CD4-CD8-	-0.06728	0
B20	B22	CD3+CD4+CD8+	CD3+CD8+(T)	0.219818	0
B20	B23	CD3+CD4+CD8+	CD3-CD16+CD56+(NK)	-0.082	0
B20	B79	CD3+CD4+CD8+	hb V -dna	-0.04477	0
B20	B138	CD3+CD4+CD8+	PAB	0.033118	6.46E-13
B20	B199	CD3+CD4+CD8+	Mg	0.031367	9.76E-12
B20	B187	CD3+CD4+CD8+	NSE	0.028957	3.25E-10
B20	B136	CD3+CD4+CD8+	5'NT	-0.02874	4.39E-10
B20	B148	CD3+CD4+CD8+	Anti_TPO	0.028457	6.50E-10
B201	B204	e-habits	Drinking	0.075874	0
B201	B205	e-habits	KDA	0.045297	0
B201	B218	e-habits	DBP	-0.04229	0
B201	B224	e-habits	pmh	0.062608	0
B201	B215	e-habits	BMI	-0.0363	3.11E-15
B201	B210	e-habits	Hip	-0.02255	9.81E-07
B203	B204	DH	Drinking	0.077104	0
B203	B206	DH	Waist	0.051822	0
B203	B209	DH	Smoking	0.096666	0
B203	B210	DH	Hip	0.057514	0
B203	B215	DH	BMI	0.06011	0
B203	B216	DH	BMI	0.073412	0
B203	B218	DH	DBP	0.049893	0
B203	B221	DH	Hei	0.049409	0
B203	B222	DH	NS	0.066148	0
B204	B206	Drinking	Waist	0.177858	0
B204	B209	Drinking	Smoking	0.396459	0
B204	B210	Drinking	Hip	0.125408	0
B204	B215	Drinking	BMI	0.115221	0
B204	B216	Drinking	BMI	0.247754	0
B204	B218	Drinking	DBP	0.126336	0
B204	B219	Drinking	OT	-0.07009	0
B204	B220	Drinking	SBP	-0.0445	0
B204	B221	Drinking	Hei	0.269908	0
B204	B222	Drinking	NS	0.271878	0
B204	B212	Drinking	IOP-L	0.037104	8.88E-16

B204	B211	Drinking	IOP-R	0.035053	2.73E-14
B204	B205	Drinking	KDA	-0.02981	9.71E-11
B205	B216	KDA	BMI	-0.04792	0
B205	B218	KDA	DBP	-0.0665	0
B205	B219	KDA	OT	0.039138	0
B205	B221	KDA	Hei	-0.04303	0
B205	B222	KDA	NS	-0.0473	0
B205	B206	KDA	Waist	-0.03424	1.05E-13
B205	B215	KDA	BMI	-0.03161	6.76E-12
B205	B209	KDA	Smoking	-0.03067	2.76E-11
B205	B210	KDA	Hip	-0.02635	1.06E-08
B205	B220	KDA	SBP	-0.02393	2.06E-07
B206	B207	Waist	WHR	0.073367	0
B206	B209	Waist	Smoking	0.152873	0
B206	B210	Waist	Hip	0.766366	0
B206	B215	Waist	BMI	0.797093	0
B206	B216	Waist	BMI	0.798864	0
B206	B218	Waist	DBP	0.127938	0
B206	B219	Waist	OT	-0.0478	0
B206	B221	Waist	Hei	0.37801	0
B206	B222	Waist	NS	0.676928	0
B206	B212	Waist	IOP-L	0.03155	7.40E-12
B206	B211	Waist	IOP-R	0.02517	4.65E-08
B207	B215	WHR	BMI	0.041216	0
B207	B216	WHR	BMI	0.04334	0
B207	B222	WHR	NS	0.078304	0
B207	B209	WHR	Smoking	0.026258	1.20E-08
B207	B221	WHR	Hei	0.023454	3.55E-07
B209	B210	Smoking	Hip	0.091049	0
B209	B215	Smoking	BMI	0.061397	0
B209	B216	Smoking	BMI	0.209098	0
B209	B218	Smoking	DBP	0.07241	0
B209	B219	Smoking	OT	-0.07171	0
B209	B220	Smoking	SBP	-0.07704	0
B209	B221	Smoking	Hei	0.272708	0
B209	B222	Smoking	NS	0.244606	0
B21	B107	CD3+CD4-CD8-	AnuA	0.072979	0
B21	B23	CD3+CD4-CD8-	CD3-CD16+CD56+(NK)	-0.12461	0
B21	B81	CD3+CD4-CD8-	TSGF	-0.03958	0
B21	B98	CD3+CD4-CD8-	anti-Jo-1	0.078102	0
B21	B125	CD3+CD4-CD8-	Cor	0.031368	9.74E-12
B21	B183	CD3+CD4-CD8-	PTA	0.031202	1.25E-11
B21	B198	CD3+CD4-CD8-	P	-0.03037	4.31E-11
B21	B63	CD3+CD4-CD8-	WBRSR(mPas_200)	0.029096	2.67E-10

B21	B69	CD3+CD4-CD8-	PV	0.028026	1.17E-09
B21	B96	CD3+CD4-CD8-	dsDNA (IIF)	0.027482	2.43E-09
B21	B66	CD3+CD4-CD8-	WBRSR(mPas_100)	0.026179	1.32E-08
B21	B22	CD3+CD4-CD8-	CD3+CD8+(T)	0.025927	1.82E-08
B21	B95	CD3+CD4-CD8-	anti-cenpb	-0.02585	2.00E-08
B21	B108	CD3+CD4-CD8-	Anti-Cyclin	-0.02585	2.01E-08
B21	B61	CD3+CD4-CD8-	WBHSRV	0.02559	2.77E-08
B21	B67	CD3+CD4-CD8-	WBRSR(mPas_50)	0.025548	2.92E-08
B21	B64	CD3+CD4-CD8-	WBRSR(mPas_5)	0.024744	7.81E-08
B21	B124	CD3+CD4-CD8-	ACTH	0.02471	8.14E-08
B21	B193	CD3+CD4-CD8-	K	0.023407	3.75E-07
B21	B105	CD3+CD4-CD8-	anti-U1RNP	0.023282	4.33E-07
B21	B136	CD3+CD4-CD8-	5'NT	-0.02323	4.61E-07
B21	B181	CD3+CD4-CD8-	APTT	-0.02321	4.69E-07
B21	B94	CD3+CD4-CD8-	ALDH2-Lys504Lys	-0.02295	6.29E-07
B210	B215	Hip	BMI	0.760949	0
B210	B216	Hip	BMI	0.783482	0
B210	B218	Hip	DBP	0.142364	0
B210	B219	Hip	OT	-0.03864	0
B210	B221	Hip	Hei	0.397218	0
B210	B222	Hip	NS	0.592621	0
B210	B220	Hip	SBP	-0.0357	9.10E-15
B210	B212	Hip	IOP-L	0.024724	8.00E-08
B211	B212	IOP-R	IOP-L	0.73689	0
B211	B215	IOP-R	BMI	0.041315	0
B211	B218	IOP-R	DBP	0.072378	0
B211	B220	IOP-R	SBP	0.05348	0
B211	B222	IOP-R	NS	0.0314	9.29E-12
B211	B216	IOP-R	BMI	0.030142	6.00E-11
B212	B215	IOP-L	BMI	0.044938	0
B212	B218	IOP-L	DBP	0.072236	0
B212	B220	IOP-L	SBP	0.059402	0
B212	B222	IOP-L	NS	0.03076	2.42E-11
B212	B216	IOP-L	BMI	0.029562	1.38E-10
B212	B228	IOP-L	OS	0.02382	2.33E-07
B213	B214	CVA-R	CVA-L	0.046984	0
B215	B216	BMI	BMI	0.792149	0
B215	B218	BMI	DBP	0.165822	0
B215	B221	BMI	Hei	0.134591	0
B215	B222	BMI	NS	0.574264	0
B215	B219	BMI	OT	-0.03688	1.11E-15
B216	B218	BMI	DBP	0.233824	0
B216	B219	BMI	OT	-0.0903	0
B216	B220	BMI	SBP	-0.08465	0

B216	B221	BMI	Hei	0.704188	0
B216	B222	BMI	NS	0.759673	0
B216	B228	BMI	OS	-0.02959	1.33E-10
B218	B219	DBP	OT	-0.09227	0
B218	B220	DBP	SBP	0.467863	0
B218	B221	DBP	Hei	0.187156	0
B218	B222	DBP	NS	0.194134	0
B218	B224	DBP	pmh	-0.0249	6.46E-08
B219	B221	OT	Hei	-0.10605	0
B219	B222	OT	NS	-0.09648	0
B219	B224	OT	pmh	0.039477	0
B22	B110	CD3+CD8+(T)	AHA	-0.04894	0
B22	B122	CD3+CD8+(T)	C4	-0.05449	0
B22	B138	CD3+CD8+(T)	PAB	0.071457	0
B22	B139	CD3+CD8+(T)	ADA	-0.04517	0
B22	B155	CD3+CD8+(T)	IgA	-0.04342	0
B22	B157	CD3+CD8+(T)	IgG	-0.03846	0
B22	B23	CD3+CD8+(T)	CD3-CD16+CD56+(NK)	-0.30728	0
B22	B32	CD3+CD8+(T)	Testo	-0.04787	0
B22	B58	CD3+CD8+(T)	HCT	-0.04081	0
B22	B81	CD3+CD8+(T)	TSGF	0.042499	0
B22	B102	CD3+CD8+(T)	anti-Sm	-0.03686	1.33E-15
B22	B107	CD3+CD8+(T)	AnuA	-0.03599	5.55E-15
B22	B105	CD3+CD8+(T)	anti-U1RNP	-0.03585	7.11E-15
B22	B54	CD3+CD8+(T)	ESR	-0.03581	7.55E-15
B22	B96	CD3+CD8+(T)	dsDNA (IIF)	-0.03575	8.44E-15
B22	B156	CD3+CD8+(T)	IgE	-0.03419	1.14E-13
B22	B195	CD3+CD8+(T)	Na	-0.0341	1.32E-13
B22	B103	CD3+CD8+(T)	anti-SSA/Ro	-0.03285	9.95E-13
B22	B134	CD3+CD8+(T)	CHE	0.031407	9.20E-12
B22	B95	CD3+CD8+(T)	anti-cenpb	-0.02863	5.14E-10
B22	B194	CD3+CD8+(T)	Cl	-0.02831	7.95E-10
B22	B93	CD3+CD8+(T)	ALDH2-Glu504Lys	-0.02713	3.86E-09
B22	B92	CD3+CD8+(T)	ALDH2-Glu504Glu	0.02597	1.72E-08
B22	B97	CD3+CD8+(T)	dsDNA	0.02538	3.60E-08
B22	B98	CD3+CD8+(T)	anti-Jo-1	0.025268	4.13E-08
B22	B185	CD3+CD8+(T)	PT	-0.02459	9.36E-08
B22	B184	CD3+CD8+(T)	INR	-0.02323	4.57E-07
B22	B183	CD3+CD8+(T)	PTA	0.023028	5.77E-07
B22	B99	CD3+CD8+(T)	anti-PM-Scl	-0.02282	7.27E-07
B220	B221	SBP	Hei	-0.15257	0
B220	B222	SBP	NS	-0.04402	0
B220	B228	SBP	OS	0.0263	1.13E-08
B220	B224	SBP	pmh	-0.02559	2.79E-08

B221	B222	Hei	NS	0.573975	0
B221	B228	Hei	OS	-0.0337	2.55E-13
B227	B228	OD	OS	0.098378	0
B23	B108	CD3-CD16+CD56+(NK)	Anti-Cyclin	-0.04184	0
B23	B125	CD3-CD16+CD56+(NK)	Cor	0.053553	0
B23	B136	CD3-CD16+CD56+(NK)	5'NT	-0.04151	0
B23	B157	CD3-CD16+CD56+(NK)	IgG	0.053737	0
B23	B32	CD3-CD16+CD56+(NK)	Testo	-0.05219	0
B23	B74	CD3-CD16+CD56+(NK)	ApoA1	0.055786	0
B23	B122	CD3-CD16+CD56+(NK)	C4	-0.03696	8.88E-16
B23	B33	CD3-CD16+CD56+(NK)	Prog	0.033704	2.52E-13
B23	B139	CD3-CD16+CD56+(NK)	ADA	0.033434	3.90E-13
B23	B98	CD3-CD16+CD56+(NK)	anti-Jo-1	-0.03253	1.63E-12
B23	B156	CD3-CD16+CD56+(NK)	IgE	0.028707	4.60E-10
B23	B93	CD3-CD16+CD56+(NK)	ALDH2-Glu504Lys	0.028576	5.51E-10
B23	B92	CD3-CD16+CD56+(NK)	ALDH2-Glu504Glu	-0.02678	6.11E-09
B23	B184	CD3-CD16+CD56+(NK)	INR	0.026008	1.64E-08
B23	B155	CD3-CD16+CD56+(NK)	IgA	0.02585	2.00E-08
B23	B134	CD3-CD16+CD56+(NK)	CHE	-0.02406	1.76E-07
B23	B99	CD3-CD16+CD56+(NK)	anti-PM-Scl	0.023665	2.79E-07
B23	B198	CD3-CD16+CD56+(NK)	P	-0.02353	3.25E-07
B23	B96	CD3-CD16+CD56+(NK)	dsDNA (IIF)	0.023486	3.42E-07
B23	B76	CD3-CD16+CD56+(NK)	ApoB	0.022584	9.46E-07
B24	B114	αHBDH	TSH TT3 TT4(TT4)	0.053841	0
B24	B123	αHBDH	hs-CRP1	0.058144	0
B24	B127	αHBDH	A-G ratio	-0.03916	0
B24	B130	αHBDH	GLB	0.044517	0
B24	B131	αHBDH	AST	0.071963	0
B24	B139	αHBDH	ADA	0.06393	0
B24	B146	αHBDH	CysC	0.040932	0
B24	B150	αHBDH	ALP	0.038184	0
B24	B174	αHBDH	Urea/Cr	0.04713	0
B24	B216	αHBDH	BMI	-0.0389	0
B24	B220	αHBDH	SBP	0.06354	0
B24	B221	αHBDH	Hei	-0.07026	0
B24	B222	αHBDH	NS	-0.04773	0
B24	B25	αHBDH	CK	0.133829	0
B24	B26	αHBDH	CKMB	0.273672	0
B24	B27	αHBDH	LDH	0.68828	0
B24	B43	αHBDH	MCV	0.047812	0
B24	B44	αHBDH	MCH	0.038565	0
B24	B46	αHBDH	RBC Count	-0.04363	0
B24	B47	αHBDH	RDW	0.076446	0
B24	B52	αHBDH	PLT/BPC	-0.04219	0

B24	B54	αHBDH	ESR	0.097883	0
B24	B69	αHBDH	PV	0.085441	0
B24	B72	αHBDH	HDL-C	0.059566	0
B24	B74	αHBDH	ApoA1	0.095002	0
B24	B91	αHBDH	TP	0.037783	2.22E-16
B24	B177	αHBDH	ACR	0.037544	4.44E-16
B24	B209	αHBDH	Smoking	-0.03656	2.00E-15
B24	B176	αHBDH	Ucr	-0.03626	3.55E-15
B24	B138	αHBDH	PAB	-0.03574	8.44E-15
B24	B163	αHBDH	SG	-0.03522	2.09E-14
B24	B141	αHBDH	AST/ALT	0.03509	2.58E-14
B24	B186	αHBDH	PFC	0.033983	1.60E-13
B24	B204	αHBDH	Drinking	-0.03379	2.21E-13
B24	B76	αHBDH	ApoB	0.033179	5.87E-13
B24	B77	αHBDH	TC	0.031841	4.75E-12
B24	B75	αHBDH	ApoA1/B	0.030834	2.17E-11
B24	B191	αHBDH	Urea	0.03039	4.17E-11
B24	B70	αHBDH	LDL-C	0.029993	7.44E-11
B24	B110	αHBDH	AHA	0.02784	1.50E-09
B24	B183	αHBDH	PTA	-0.02727	3.24E-09
B24	B107	αHBDH	AnuA	-0.02638	1.02E-08
B24	B128	αHBDH	ALT	0.025952	1.76E-08
B24	B192	αHBDH	UA	-0.02579	2.17E-08
B24	B115	αHBDH	TSH TT3 TT4(TT3)	0.025409	3.47E-08
B24	B73	αHBDH	ApoA	0.025114	4.99E-08
B24	B175	αHBDH	ALB-U	0.024882	6.60E-08
B24	B134	αHBDH	CHE	-0.02396	1.98E-07
B24	B71	αHBDH	TG	-0.02297	6.15E-07
B24	B40	αHBDH	WBC	-0.02271	8.23E-07
B25	B128	CK	ALT	0.03955	0
B25	B131	CK	AST	0.056486	0
B25	B175	CK	ALB-U	0.047233	0
B25	B177	CK	ACR	0.057008	0
B25	B190	CK	Cre	0.050177	0
B25	B216	CK	BMI	0.042996	0
B25	B222	CK	NS	0.042678	0
B25	B26	CK	CKMB	0.22824	0
B25	B27	CK	LDH	0.14616	0
B25	B192	CK	UA	0.035971	5.77E-15
B25	B174	CK	Urea/Cr	-0.0353	1.80E-14
B25	B221	CK	Hei	0.033368	4.33E-13
B25	B215	CK	BMI	0.030323	4.61E-11
B25	B206	CK	Waist	0.029688	1.15E-10
B25	B204	CK	Drinking	0.025144	4.80E-08

B25	B210	CK	Hip	0.025134	4.87E-08
B25	B138	CK	PAB	0.023935	2.04E-07
B26	B131	CKMB	AST	0.040018	0
B26	B27	CKMB	LDH	0.218679	0
B26	B62	CKMB	WBHSRVI	0.045341	0
B26	B63	CKMB	WBRSR(mPas_200)	0.058721	0
B26	B64	CKMB	WBRSR(mPas_5)	0.051472	0
B26	B65	CKMB	WBRSR(mPas_1)	0.043826	0
B26	B66	CKMB	WBRSR(mPas_100)	0.059859	0
B26	B67	CKMB	WBRSR(mPas_50)	0.059052	0
B26	B61	CKMB	WBHSRV	0.035128	2.40E-14
B26	B90	CKMB	CYFRA 21-1	0.033111	6.54E-13
B26	B60	CKMB	WBRVI	0.032582	1.51E-12
B26	B51	CKMB	Hb	0.031206	1.24E-11
B26	B69	CKMB	PV	0.031115	1.43E-11
B26	B139	CKMB	ADA	0.030601	3.06E-11
B26	B130	CKMB	GLB	0.030071	6.64E-11
B26	B177	CKMB	ACR	0.028668	4.85E-10
B26	B127	CKMB	A-G ratio	-0.02857	5.54E-10
B26	B54	CKMB	ESR	0.027384	2.77E-09
B26	B220	CKMB	SBP	0.026253	1.20E-08
B26	B46	CKMB	RBC Count	0.024835	7.00E-08
B26	B58	CKMB	HCT	0.024703	8.20E-08
B26	B87	CKMB	NSE	0.022794	7.49E-07
B27	B114	LDH	TSH TT3 TT4(TT4)	0.050433	0
B27	B118	LDH	CEA1	0.057966	0
B27	B123	LDH	hs-CRP1	0.052881	0
B27	B127	LDH	A-G ratio	-0.04969	0
B27	B128	LDH	ALT	0.080138	0
B27	B130	LDH	GLB	0.055008	0
B27	B131	LDH	AST	0.149293	0
B27	B136	LDH	5'NT	0.03805	0
B27	B138	LDH	PAB	-0.05853	0
B27	B139	LDH	ADA	0.140226	0
B27	B140	LDH	r-GT	0.038378	0
B27	B145	LDH	BGP	-0.03975	0
B27	B146	LDH	CysC	0.041484	0
B27	B150	LDH	ALP	0.130333	0
B27	B163	LDH	SG	-0.03819	0
B27	B176	LDH	Ucr	-0.05749	0
B27	B177	LDH	ACR	0.055198	0
B27	B220	LDH	SBP	0.060418	0
B27	B221	LDH	Hei	-0.06349	0
B27	B43	LDH	MCV	0.046474	0

B27	B44	LDH	MCH	0.041046	0
B27	B47	LDH	RDW	0.067166	0
B27	B54	LDH	ESR	0.065439	0
B27	B69	LDH	PV	0.074583	0
B27	B72	LDH	HDL-C	0.053515	0
B27	B74	LDH	ApoA1	0.071928	0
B27	B76	LDH	ApoB	0.045261	0
B27	B91	LDH	TP	0.045326	0
B27	B186	LDH	PFC	0.037511	4.44E-16
B27	B77	LDH	TC	0.037235	6.66E-16
B27	B174	LDH	Urea/Cr	0.037106	8.88E-16
B27	B175	LDH	ALB-U	0.03708	8.88E-16
B27	B46	LDH	RBC Count	-0.03596	5.77E-15
B27	B66	LDH	WBRSR(mPas_100)	0.03559	1.11E-14
B27	B67	LDH	WBRSR(mPas_50)	0.035018	2.89E-14
B27	B209	LDH	Smoking	-0.03424	1.06E-13
B27	B87	LDH	NSE	0.034225	1.08E-13
B27	B70	LDH	LDL-C	0.033584	3.06E-13
B27	B52	LDH	PLT/BPC	-0.03284	1.00E-12
B27	B64	LDH	WBRSR(mPas_5)	0.032795	1.08E-12
B27	B129	LDH	IBIL	0.032125	3.07E-12
B27	B63	LDH	WBRSR(mPas_200)	0.031866	4.57E-12
B27	B191	LDH	Urea	0.031512	7.84E-12
B27	B133	LDH	TBIL	0.03148	8.23E-12
B27	B204	LDH	Drinking	-0.03086	2.09E-11
B27	B65	LDH	WBRSR(mPas_1)	0.028913	3.45E-10
B27	B197	LDH	AG	0.028819	3.94E-10
B27	B189	LDH	eGFB	-0.02869	4.72E-10
B27	B200	LDH	OP	0.028562	5.62E-10
B27	B222	LDH	NS	-0.02638	1.02E-08
B27	B135	LDH	TBA	0.025805	2.12E-08
B27	B141	LDH	AST/ALT	0.025356	3.70E-08
B27	B112	LDH	FT4	0.025018	5.60E-08
B27	B195	LDH	Na	0.02494	6.16E-08
B27	B61	LDH	WBHSRV	0.024414	1.16E-07
B27	B215	LDH	BMI	0.02417	1.55E-07
B27	B111	LDH	TSH	0.023907	2.10E-07
B27	B109	LDH	AMA-M2	0.022884	6.78E-07
B28	B108	PRL	Anti-Cyclin	0.041547	0
B28	B29	PRL	E2	0.166889	0
B28	B30	PRL	LH	-0.13841	0
B28	B31	PRL	FSH	-0.24829	0
B28	B32	PRL	Testo	-0.06296	0
B28	B33	PRL	Prog	0.214543	0

B29	B108	E2	Anti-Cyclin	-0.06542	0
B29	B110	E2	AHA	0.041219	0
B29	B181	E2	APTT	0.038284	0
B29	B198	E2	P	-0.03951	0
B29	B30	E2	LH	-0.40478	0
B29	B31	E2	FSH	-0.64645	0
B29	B32	E2	Testo	-0.13263	0
B29	B33	E2	Prog	0.551986	0
B29	B121	E2	C3	-0.03558	1.11E-14
B29	B139	E2	ADA	-0.03226	2.50E-12
B29	B195	E2	Na	-0.03047	3.71E-11
B29	B114	E2	TSH TT3 TT4(TT4)	-0.02895	3.29E-10
B29	B107	E2	AnuA	0.027881	1.42E-09
B29	B199	E2	Mg	-0.02593	1.81E-08
B29	B196	E2	TCO2	-0.02559	2.76E-08
B29	B79	E2	hb V -dna	0.025005	5.69E-08
B3	B123	Hcy	hs-CRP1	0.04207	0
B3	B132	Hcy	Dbil	0.042754	0
B3	B138	Hcy	PAB	0.038723	0
B3	B140	Hcy	r-GT	0.055992	0
B3	B146	Hcy	CysC	0.071722	0
B3	B163	Hcy	SG	0.047466	0
B3	B174	Hcy	Urea/Cr	-0.06693	0
B3	B175	Hcy	ALB-U	0.063701	0
B3	B176	Hcy	Ucr	0.058348	0
B3	B177	Hcy	ACR	0.052669	0
B3	B189	Hcy	eGFB	-0.12635	0
B3	B190	Hcy	Cre	0.109901	0
B3	B191	Hcy	Urea	0.076924	0
B3	B192	Hcy	UA	0.108538	0
B3	B204	Hcy	Drinking	0.052307	0
B3	B206	Hcy	Waist	0.065077	0
B3	B209	Hcy	Smoking	0.056217	0
B3	B216	Hcy	BMI	0.063781	0
B3	B221	Hcy	Hei	0.06574	0
B3	B222	Hcy	NS	0.073265	0
B3	B27	Hcy	LDH	0.040838	0
B3	B42	Hcy	MON	0.045142	0
B3	B44	Hcy	MCH	0.043172	0
B3	B47	Hcy	RDW	0.062723	0
B3	B48	Hcy	LYM%	-0.0478	0
B3	B51	Hcy	Hb	0.060994	0
B3	B72	Hcy	HDL-C	-0.04834	0
B3	B74	Hcy	ApoA1	-0.05874	0

B3	B79	Hcy	hb V -dna	-0.04466	0
B3	B86	Hcy	TBhCG	-0.04479	0
B3	B75	Hcy	ApoA1/B	-0.03754	4.44E-16
B3	B43	Hcy	MCV	0.033264	5.12E-13
B3	B10	Hcy	PG I	0.033214	5.55E-13
B3	B210	Hcy	Hip	0.03293	8.71E-13
B3	B45	Hcy	MCHC	0.032881	9.41E-13
B3	B215	Hcy	BMI	0.032223	2.64E-12
B3	B133	Hcy	TBIL	0.032054	3.42E-12
B3	B46	Hcy	RBC Count	0.031705	5.84E-12
B3	B150	Hcy	ALP	0.030207	5.45E-11
B3	B53	Hcy	GR%	0.029832	9.39E-11
B3	B193	Hcy	K	-0.0298	9.87E-11
B3	B62	Hcy	WBHSRVI	0.029208	2.28E-10
B3	B24	Hcy	αHBDH	0.028837	3.84E-10
B3	B126	Hcy	Alb	-0.02859	5.37E-10
B3	B66	Hcy	WBRSR(mPas_100)	0.027897	1.39E-09
B3	B52	Hcy	PLT/BPC	-0.02765	1.94E-09
B3	B67	Hcy	WBRSR(mPas_50)	0.027478	2.44E-09
B3	B90	Hcy	CYFRA 21-1	0.026753	6.33E-09
B3	B63	Hcy	WBRSR(mPas_200)	0.026326	1.10E-08
B3	B137	Hcy	AFU	0.026001	1.66E-08
B3	B50	Hcy	eo%	0.025885	1.92E-08
B3	B220	Hcy	SBP	0.025709	2.39E-08
B3	B129	Hcy	IBIL	0.025462	3.25E-08
B3	B198	Hcy	P	-0.02541	3.45E-08
B3	B166	Hcy	Pro	0.025326	3.84E-08
B3	B184	Hcy	INR	-0.02496	6.01E-08
B3	B119	Hcy	Hcv-rna	-0.02384	2.28E-07
B3	B179	Hcy	α1-MG	0.0238	2.38E-07
B3	B12	Hcy	PG II	0.023772	2.46E-07
B3	B64	Hcy	WBRSR(mPas_5)	0.02331	4.19E-07
B3	B131	Hcy	AST	0.022876	6.84E-07
B30	B108	LH	Anti-Cyclin	-0.04793	0
B30	B121	LH	C3	0.055612	0
B30	B198	LH	P	0.040657	0
B30	B31	LH	FSH	0.839542	0
B30	B32	LH	Testo	-0.33544	0
B30	B33	LH	Prog	-0.43777	0
B30	B36	LH	V-Lym%	-0.05235	0
B30	B115	LH	TSH TT3 TT4(TT3)	0.028893	3.55E-10
B30	B122	LH	C4	0.026358	1.05E-08
B31	B108	FSH	Anti-Cyclin	-0.04018	0
B31	B198	FSH	P	0.04625	0

B31	B32	FSH	Testo	-0.23757	0
B31	B33	FSH	Prog	-0.53324	0
B31	B36	FSH	V-Lym%	-0.04712	0
B31	B115	FSH	TSH TT3 TT4(TT3)	0.03656	2.22E-15
B31	B114	FSH	TSH TT3 TT4(TT4)	0.031682	6.05E-12
B31	B121	FSH	C3	0.030482	3.65E-11
B31	B195	FSH	Na	0.02868	4.77E-10
B31	B138	FSH	PAB	-0.02668	6.92E-09
B32	B108	Testo	Anti-Cyclin	0.245022	0
B32	B123	Testo	hs-CRP1	0.041894	0
B32	B33	Testo	Prog	-0.16514	0
B32	B212	Testo	IOP-L	0.029421	1.69E-10
B32	B211	Testo	IOP-R	0.025055	5.35E-08
B33	B103	Prog	anti-SSA/Ro	-0.04961	0
B33	B108	Prog	Anti-Cyclin	-0.06411	0
B33	B110	Prog	AHA	-0.05026	0
B33	B125	Prog	Cor	0.045047	0
B33	B181	Prog	APTT	0.050426	0
B33	B138	Prog	PAB	0.036088	4.66E-15
B33	B107	Prog	AnuA	0.034839	3.91E-14
B33	B198	Prog	P	-0.03431	9.44E-14
B33	B193	Prog	K	0.029453	1.61E-10
B33	B199	Prog	Mg	-0.02867	4.85E-10
B33	B93	Prog	ALDH2-Glu504Lys	0.024733	7.91E-08
B33	B92	Prog	ALDH2-Glu504Glu	-0.02467	8.52E-08
B33	B137	Prog	AFU	0.023969	1.96E-07
B33	B184	Prog	INR	0.022756	7.82E-07
B34	B138	TK	PAB	-0.03893	0
B34	B86	TK	TBhCG	0.032383	2.06E-12
B34	B71	TK	TG	-0.02938	1.80E-10
B34	B79	TK	hb V -dna	0.028231	8.86E-10
B34	B193	TK	K	0.027234	3.37E-09
B34	B125	TK	Cor	0.026512	8.64E-09
B34	B137	TK	AFU	-0.02614	1.38E-08
B34	B135	TK	TBA	0.025398	3.52E-08
B34	B134	TK	CHE	-0.0247	8.23E-08
B34	B198	TK	P	0.024498	1.05E-07
B34	B81	TK	TSGF	-0.02326	4.45E-07
B35	B125	β2-MG	Cor	-0.05468	0
B35	B159	β2-MG	β2-MG	0.601897	0
B35	B181	β2-MG	APTT	0.039646	0
B35	B182	β2-MG	TT	-0.06565	0
B35	B184	β2-MG	INR	0.044951	0
B35	B185	β2-MG	PT	0.063408	0

B35	B186	β2-MG	PFC	0.060118	0
B35	B124	β2-MG	ACTH	0.025539	2.95E-08
B37	B38	BAND	BAND%	0.892053	0
B37	B39	BAND	Other cells	0.354909	0
B37	B40	BAND	WBC	0.108625	0
B37	B42	BAND	MON	0.117027	0
B37	B47	BAND	RDW	0.085334	0
B37	B53	BAND	GR%	-0.02852	6.00E-10
B37	B141	BAND	AST/ALT	0.025993	1.67E-08
B38	B39	BAND%	Other cells	0.387787	0
B38	B40	BAND%	WBC	0.082307	0
B38	B42	BAND%	MON	0.100535	0
B38	B47	BAND%	RDW	0.079057	0
B38	B53	BAND%	GR%	-0.03537	1.60E-14
B38	B141	BAND%	AST/ALT	0.025358	3.69E-08
B39	B40	Other cells	WBC	0.052925	0
B39	B42	Other cells	MON	0.052823	0
B39	B43	Other cells	MCV	-0.07255	0
B39	B44	Other cells	MCH	-0.07916	0
B39	B45	Other cells	MCHC	-0.04989	0
B39	B46	Other cells	RBC Count	0.036655	1.78E-15
B39	B51	Other cells	Hb	-0.03512	2.44E-14
B4	B5	WB-Ca	WB-Mg	0.181293	0
B4	B6	WB-Ca	WB-Fe	0.070394	0
B4	B7	WB-Ca	WB-Cop	0.131195	0
B4	B8	WB-Ca	WB-Zinc	-0.10782	0
B4	B100	WB-Ca	anti-Ro52	-0.03491	3.46E-14
B40	B123	WBC	hs-CRP1	0.086941	0
B40	B127	WBC	A-G ratio	-0.10491	0
B40	B128	WBC	ALT	0.061564	0
B40	B129	WBC	IBIL	-0.07256	0
B40	B130	WBC	GLB	0.131906	0
B40	B132	WBC	Dbil	-0.0868	0
B40	B133	WBC	TBIL	-0.08189	0
B40	B140	WBC	r-GT	0.074642	0
B40	B141	WBC	AST/ALT	-0.06969	0
B40	B153	WBC	Glu	0.066361	0
B40	B160	WBC	PH	-0.0825	0
B40	B163	WBC	SG	0.093591	0
B40	B166	WBC	Pro	0.082814	0
B40	B170	WBC	U-GLU	0.04585	0
B40	B174	WBC	Urea/Cr	-0.08639	0
B40	B176	WBC	Ucr	0.047914	0
B40	B188	WBC	Ca	0.058318	0

B40	B190	WBC	Cre	0.081981	0
B40	B192	WBC	UA	0.16591	0
B40	B203	WBC	DH	0.043355	0
B40	B204	WBC	Drinking	0.038285	0
B40	B206	WBC	Waist	0.163751	0
B40	B209	WBC	Smoking	0.176442	0
B40	B210	WBC	Hip	0.11635	0
B40	B215	WBC	BMI	0.1375	0
B40	B216	WBC	BMI	0.153572	0
B40	B221	WBC	Hei	0.093359	0
B40	B222	WBC	NS	0.171844	0
B40	B42	WBC	MON	0.665533	0
B40	B43	WBC	MCV	-0.05869	0
B40	B44	WBC	MCH	-0.04238	0
B40	B46	WBC	RBC Count	0.218918	0
B40	B48	WBC	LYM%	-0.19354	0
B40	B49	WBC	baso%	-0.0763	0
B40	B51	WBC	Hb	0.199471	0
B40	B52	WBC	PLT/BPC	0.252248	0
B40	B53	WBC	GR%	0.210673	0
B40	B60	WBC	WBRVI	0.039165	0
B40	B62	WBC	WBHSRVI	0.038053	0
B40	B63	WBC	WBRSR(mPas_200)	0.054885	0
B40	B64	WBC	WBRSR(mPas_5)	0.053543	0
B40	B65	WBC	WBRSR(mPas_1)	0.050719	0
B40	B66	WBC	WBRSR(mPas_100)	0.054876	0
B40	B67	WBC	WBRSR(mPas_50)	0.055465	0
B40	B70	WBC	LDL-C	0.045462	0
B40	B71	WBC	TG	0.146738	0
B40	B72	WBC	HDL-C	-0.15952	0
B40	B77	WBC	TC	0.040307	0
B40	B91	WBC	TP	0.128677	0
B40	B61	WBC	WBHSRV	0.037941	2.22E-16
B40	B146	WBC	CysC	0.037369	4.44E-16
B40	B75	WBC	ApoA1/B	-0.03615	4.22E-15
B40	B76	WBC	ApoB	0.036133	4.44E-15
B40	B74	WBC	ApoA1	-0.03487	3.71E-14
B40	B134	WBC	CHE	0.034822	4.02E-14
B40	B59	WBC	WBRV	0.034524	6.59E-14
B40	B69	WBC	PV	0.032804	1.06E-12
B40	B107	WBC	AnuA	0.029733	1.08E-10
B40	B218	WBC	DBP	0.02926	2.12E-10
B40	B194	WBC	Cl	-0.02782	1.55E-09
B40	B150	WBC	ALP	0.027805	1.58E-09

B40	B191	WBC	Urea	0.026406	9.90E-09
B40	B56	WBC	IR	0.023031	5.74E-07
B40	B112	WBC	FT4	0.022999	5.96E-07
B40	B83	WBC	AFP	0.022911	6.57E-07
B40	B58	WBC	HCT	0.022811	7.35E-07
B41	B42	MON %	MON	0.026957	4.85E-09
B42	B123	MON	hs-CRP1	0.081788	0
B42	B126	MON	Alb	-0.08972	0
B42	B127	MON	A-G ratio	-0.12921	0
B42	B128	MON	ALT	0.074387	0
B42	B130	MON	GLB	0.118621	0
B42	B131	MON	AST	0.078164	0
B42	B140	MON	r-GT	0.090861	0
B42	B146	MON	CysC	0.063924	0
B42	B160	MON	PH	-0.06547	0
B42	B163	MON	SG	0.126502	0
B42	B166	MON	Pro	0.084389	0
B42	B174	MON	Urea/Cr	-0.11081	0
B42	B176	MON	Ucr	0.051024	0
B42	B189	MON	eGFB	-0.0922	0
B42	B190	MON	Cre	0.12557	0
B42	B191	MON	Urea	0.067013	0
B42	B192	MON	UA	0.188808	0
B42	B204	MON	Drinking	0.077806	0
B42	B206	MON	Waist	0.174541	0
B42	B209	MON	Smoking	0.185912	0
B42	B210	MON	Hip	0.108602	0
B42	B215	MON	BMI	0.104645	0
B42	B216	MON	BMI	0.149288	0
B42	B221	MON	Hei	0.125438	0
B42	B222	MON	NS	0.199522	0
B42	B46	MON	RBC Count	0.152986	0
B42	B47	MON	RDW	0.098107	0
B42	B48	MON	LYM%	-0.15616	0
B42	B50	MON	eo%	0.047674	0
B42	B51	MON	Hb	0.185042	0
B42	B52	MON	PLT/BPC	0.163112	0
B42	B62	MON	WBHSRVI	0.039587	0
B42	B63	MON	WBRSR(mPas_200)	0.048941	0
B42	B64	MON	WBRSR(mPas_5)	0.044422	0
B42	B65	MON	WBRSR(mPas_1)	0.041353	0
B42	B66	MON	WBRSR(mPas_100)	0.048047	0
B42	B67	MON	WBRSR(mPas_50)	0.047985	0
B42	B71	MON	TG	0.041155	0

B42	B72	MON	HDL-C	-0.11724	0
B42	B91	MON	TP	0.053891	0
B42	B44	MON	MCH	0.037931	2.22E-16
B42	B107	MON	AnuA	0.037608	4.44E-16
B42	B60	MON	WBRVI	0.036706	1.55E-15
B42	B77	MON	TC	-0.03449	6.99E-14
B42	B90	MON	CYFRA 21-1	0.034164	1.19E-13
B42	B43	MON	MCV	0.033453	3.79E-13
B42	B74	MON	ApoA1	-0.03337	4.31E-13
B42	B113	MON	FT3	0.032316	2.28E-12
B42	B129	MON	IBIL	-0.03177	5.32E-12
B42	B170	MON	U-GLU	0.030791	2.31E-11
B42	B161	MON	LEU	-0.02896	3.23E-10
B42	B61	MON	WBHSRV	0.027563	2.18E-09
B42	B203	MON	DH	0.026803	5.93E-09
B42	B153	MON	Glu	0.02628	1.16E-08
B42	B58	MON	HCT	0.025429	3.39E-08
B42	B118	MON	CEA1	0.024677	8.46E-08
B42	B141	MON	AST/ALT	-0.02466	8.63E-08
B42	B194	MON	Cl	-0.02455	9.85E-08
B42	B133	MON	TBIL	-0.0245	1.05E-07
B42	B112	MON	FT4	0.023316	4.16E-07
B42	B75	MON	ApoA1/B	-0.02278	7.62E-07
B43	B112	MCV	FT4	-0.04219	0
B43	B126	MCV	Alb	-0.05689	0
B43	B131	MCV	AST	0.068195	0
B43	B132	MCV	Dbil	0.070929	0
B43	B140	MCV	r-GT	0.063713	0
B43	B141	MCV	AST/ALT	0.074362	0
B43	B189	MCV	eGFB	-0.09978	0
B43	B191	MCV	Urea	0.048248	0
B43	B204	MCV	Drinking	0.079735	0
B43	B209	MCV	Smoking	0.085334	0
B43	B215	MCV	BMI	-0.05282	0
B43	B216	MCV	BMI	-0.05002	0
B43	B218	MCV	DBP	-0.0558	0
B43	B44	MCV	MCH	0.901898	0
B43	B45	MCV	MCHC	0.177171	0
B43	B46	MCV	RBC Count	-0.53735	0
B43	B47	MCV	RDW	0.585043	0
B43	B51	MCV	Hb	0.116625	0
B43	B52	MCV	PLT/BPC	-0.15828	0
B43	B53	MCV	GR%	-0.05872	0
B43	B59	MCV	WBRV	-0.04091	0

B43	B72	MCV	HDL-C	0.090648	0
B43	B91	MCV	TP	-0.05757	0
B43	B74	MCV	ApoA1	0.036531	2.22E-15
B43	B55	MCV	TK	-0.0343	9.55E-14
B43	B160	MCV	PH	-0.03359	3.03E-13
B43	B153	MCV	Glu	-0.03187	4.53E-12
B43	B48	MCV	LYM%	0.03166	6.26E-12
B43	B77	MCV	TC	0.030979	1.75E-11
B43	B190	MCV	Cre	0.029586	1.34E-10
B43	B57	MCV	RBCAI	-0.02948	1.56E-10
B43	B210	MCV	Hip	-0.0291	2.64E-10
B43	B201	MCV	e-habits	0.028693	4.69E-10
B43	B71	MCV	TG	-0.02708	4.13E-09
B43	B130	MCV	GLB	-0.02631	1.12E-08
B43	B134	MCV	CHE	-0.02545	3.31E-08
B43	B146	MCV	CysC	0.023232	4.58E-07
B44	B127	MCH	A-G ratio	0.060051	0
B44	B128	MCH	ALT	0.047466	0
B44	B129	MCH	IBIL	0.080058	0
B44	B130	MCH	GLB	-0.0604	0
B44	B131	MCH	AST	0.087678	0
B44	B132	MCH	Dbil	0.114684	0
B44	B133	MCH	TBIL	0.095367	0
B44	B140	MCH	r-GT	0.103767	0
B44	B163	MCH	SG	0.063518	0
B44	B174	MCH	Urea/Cr	-0.06762	0
B44	B190	MCH	Cre	0.038087	0
B44	B192	MCH	UA	0.055787	0
B44	B204	MCH	Drinking	0.1373	0
B44	B206	MCH	Waist	0.05226	0
B44	B209	MCH	Smoking	0.12945	0
B44	B216	MCH	BMI	0.059231	0
B44	B221	MCH	Hei	0.096803	0
B44	B222	MCH	NS	0.092207	0
B44	B45	MCH	MCHC	0.582185	0
B44	B46	MCH	RBC Count	-0.41693	0
B44	B47	MCH	RDW	0.399959	0
B44	B51	MCH	Hb	0.312533	0
B44	B52	MCH	PLT/BPC	-0.1368	0
B44	B53	MCH	GR%	-0.05722	0
B44	B54	MCH	ESR	-0.04097	0
B44	B62	MCH	WBHSRVI	0.040239	0
B44	B71	MCH	TG	0.053753	0
B44	B91	MCH	TP	-0.04895	0

B44	B63	MCH	WBRSR(mPas_200)	0.037168	6.66E-16
B44	B66	MCH	WBRSR(mPas_100)	0.034801	4.17E-14
B44	B48	MCH	LYM%	0.033554	3.22E-13
B44	B67	MCH	WBRSR(mPas_50)	0.032698	1.26E-12
B44	B176	MCH	Ucr	0.032058	3.40E-12
B44	B112	MCH	FT4	-0.03116	1.33E-11
B44	B189	MCH	eGFB	-0.03103	1.63E-11
B44	B114	MCH	TSH TT3 TT4(TT4)	-0.02976	1.04E-10
B44	B58	MCH	HCT	0.029716	1.11E-10
B44	B198	MCH	P	-0.0294	1.75E-10
B44	B138	MCH	PAB	0.029026	2.95E-10
B44	B219	MCH	OT	-0.02867	4.85E-10
B44	B57	MCH	RBCAI	-0.02806	1.11E-09
B44	B210	MCH	Hip	0.027905	1.38E-09
B44	B203	MCH	DH	0.02611	1.44E-08
B44	B166	MCH	Pro	0.02492	6.31E-08
B44	B161	MCH	LEU	-0.02475	7.71E-08
B44	B172	MCH	KB	0.023201	4.74E-07
B44	B201	MCH	e-habits	0.022592	9.37E-07
B45	B126	MCHC	Alb	0.130591	0
B45	B127	MCHC	A-G ratio	0.125934	0
B45	B128	MCHC	ALT	0.13378	0
B45	B129	MCHC	IBIL	0.173084	0
B45	B130	MCHC	GLB	-0.08613	0
B45	B131	MCHC	AST	0.064723	0
B45	B132	MCHC	Dbil	0.117655	0
B45	B133	MCHC	TBIL	0.171122	0
B45	B134	MCHC	CHE	0.04179	0
B45	B138	MCHC	PAB	0.052618	0
B45	B140	MCHC	r-GT	0.107674	0
B45	B141	MCHC	AST/ALT	-0.14611	0
B45	B153	MCHC	Glu	0.108664	0
B45	B161	MCHC	LEU	-0.06743	0
B45	B163	MCHC	SG	0.135622	0
B45	B166	MCHC	Pro	0.05405	0
B45	B170	MCHC	U-GLU	0.065476	0
B45	B173	MCHC	NTT	-0.05233	0
B45	B174	MCHC	Urea/Cr	-0.15215	0
B45	B176	MCHC	Ucr	0.070823	0
B45	B189	MCHC	eGFB	0.116434	0
B45	B191	MCHC	Urea	-0.0796	0
B45	B192	MCHC	UA	0.142377	0
B45	B193	MCHC	K	-0.05607	0
B45	B203	MCHC	DH	0.039856	0

B45	B204	MCHC	Drinking	0.153135	0
B45	B206	MCHC	Waist	0.136006	0
B45	B209	MCHC	Smoking	0.124822	0
B45	B210	MCHC	Hip	0.117365	0
B45	B215	MCHC	BMI	0.102642	0
B45	B216	MCHC	BMI	0.226577	0
B45	B218	MCHC	DBP	0.119369	0
B45	B219	MCHC	OT	-0.06485	0
B45	B220	MCHC	SBP	-0.04977	0
B45	B221	MCHC	Hei	0.249038	0
B45	B222	MCHC	NS	0.242206	0
B45	B46	MCHC	RBC Count	0.059778	0
B45	B47	MCHC	RDW	-0.19955	0
B45	B51	MCHC	Hb	0.497198	0
B45	B54	MCHC	ESR	-0.05902	0
B45	B56	MCHC	IR	0.073772	0
B45	B60	MCHC	WBRVI	0.063578	0
B45	B61	MCHC	WBHSRV	0.074713	0
B45	B62	MCHC	WBHSRVI	0.086838	0
B45	B63	MCHC	WBRSR(mPas_200)	0.084546	0
B45	B64	MCHC	WBRSR(mPas_5)	0.073205	0
B45	B65	MCHC	WBRSR(mPas_1)	0.063251	0
B45	B66	MCHC	WBRSR(mPas_100)	0.081371	0
B45	B67	MCHC	WBRSR(mPas_50)	0.080178	0
B45	B71	MCHC	TG	0.17231	0
B45	B72	MCHC	HDL-C	-0.1491	0
B45	B74	MCHC	ApoA1	-0.06268	0
B45	B58	MCHC	HCT	0.037161	6.66E-16
B45	B146	MCHC	CysC	-0.03349	3.57E-13
B45	B194	MCHC	Cl	-0.03218	2.82E-12
B45	B111	MCHC	TSH	-0.03208	3.31E-12
B45	B75	MCHC	ApoA1/B	-0.03136	9.93E-12
B45	B160	MCHC	PH	0.030791	2.31E-11
B45	B190	MCHC	Cre	0.030241	5.19E-11
B45	B59	MCHC	WBRV	0.03022	5.36E-11
B45	B172	MCHC	KB	0.02822	8.99E-10
B45	B198	MCHC	P	-0.02765	1.93E-09
B45	B114	MCHC	TSH TT3 TT4(TT4)	-0.02683	5.74E-09
B45	B149	MCHC	TGAb	-0.02551	3.05E-08
B45	B73	MCHC	ApoA	-0.02481	7.23E-08
B45	B148	MCHC	Anti_TPO	-0.02413	1.63E-07
B45	B55	MCHC	TK	0.023568	3.12E-07
B45	B195	MCHC	Na	-0.02255	9.82E-07
B46	B111	RBC Count	TSH	-0.05217	0

B46	B113	RBC Count	FT3	0.093337	0
B46	B115	RBC Count	TSH TT3 TT4(TT3)	0.061776	0
B46	B126	RBC Count	Alb	0.240363	0
B46	B127	RBC Count	A-G ratio	0.128421	0
B46	B128	RBC Count	ALT	0.192564	0
B46	B129	RBC Count	IBIL	0.1317	0
B46	B130	RBC Count	GLB	-0.05352	0
B46	B132	RBC Count	Dbil	0.055655	0
B46	B133	RBC Count	TBIL	0.121127	0
B46	B134	RBC Count	CHE	0.078155	0
B46	B138	RBC Count	PAB	0.062093	0
B46	B140	RBC Count	r-GT	0.085724	0
B46	B141	RBC Count	AST/ALT	-0.27009	0
B46	B146	RBC Count	CysC	-0.04644	0
B46	B153	RBC Count	Glu	0.059763	0
B46	B161	RBC Count	LEU	-0.09926	0
B46	B163	RBC Count	SG	0.237486	0
B46	B166	RBC Count	Pro	0.087731	0
B46	B170	RBC Count	U-GLU	0.053828	0
B46	B173	RBC Count	NTT	-0.06821	0
B46	B174	RBC Count	Urea/Cr	-0.22316	0
B46	B176	RBC Count	Ucr	0.103055	0
B46	B188	RBC Count	Ca	0.090683	0
B46	B189	RBC Count	eGFB	0.185915	0
B46	B191	RBC Count	Urea	-0.13342	0
B46	B192	RBC Count	UA	0.205474	0
B46	B194	RBC Count	Cl	-0.04581	0
B46	B198	RBC Count	P	-0.04127	0
B46	B203	RBC Count	DH	0.038967	0
B46	B204	RBC Count	Drinking	0.144403	0
B46	B205	RBC Count	KDA	-0.04952	0
B46	B206	RBC Count	Waist	0.270529	0
B46	B209	RBC Count	Smoking	0.14454	0
B46	B210	RBC Count	Hip	0.231995	0
B46	B215	RBC Count	BMI	0.229537	0
B46	B216	RBC Count	BMI	0.377675	0
B46	B218	RBC Count	DBP	0.248801	0
B46	B219	RBC Count	OT	-0.08225	0
B46	B221	RBC Count	Hei	0.351894	0
B46	B222	RBC Count	NS	0.370386	0
B46	B47	RBC Count	RDW	-0.3989	0
B46	B48	RBC Count	LYM%	-0.04809	0
B46	B49	RBC Count	baso%	0.066265	0
B46	B51	RBC Count	Hb	0.724535	0

B46	B52	RBC Count	PLT/BPC	0.092356	0
B46	B53	RBC Count	GR%	0.047564	0
B46	B54	RBC Count	ESR	-0.06172	0
B46	B56	RBC Count	IR	0.104259	0
B46	B57	RBC Count	RBCAI	0.054886	0
B46	B58	RBC Count	HCT	0.092467	0
B46	B59	RBC Count	WBRV	0.085424	0
B46	B60	RBC Count	WBRVI	0.165232	0
B46	B61	RBC Count	WBHSRV	0.110139	0
B46	B62	RBC Count	WBHSRVI	0.167287	0
B46	B63	RBC Count	WBRSR(mPas_200)	0.169111	0
B46	B64	RBC Count	WBRSR(mPas_5)	0.175563	0
B46	B65	RBC Count	WBRSR(mPas_1)	0.168015	0
B46	B66	RBC Count	WBRSR(mPas_100)	0.171198	0
B46	B67	RBC Count	WBRSR(mPas_50)	0.173329	0
B46	B70	RBC Count	LDL-C	0.079357	0
B46	B71	RBC Count	TG	0.119677	0
B46	B72	RBC Count	HDL-C	-0.22577	0
B46	B74	RBC Count	ApoA1	-0.08107	0
B46	B75	RBC Count	ApoA1/B	-0.06913	0
B46	B76	RBC Count	ApoB	0.043581	0
B46	B79	RBC Count	hb V -dna	-0.03976	0
B46	B91	RBC Count	TP	0.094642	0
B46	B148	RBC Count	Anti_TPO	-0.03134	1.02E-11
B46	B149	RBC Count	TGAb	-0.02866	4.92E-10
B46	B197	RBC Count	AG	0.028416	6.88E-10
B46	B119	RBC Count	Hcv-rna	-0.02837	7.30E-10
B46	B142	RBC Count	25-(OH)D	-0.02731	3.07E-09
B46	B137	RBC Count	AFU	0.026927	5.05E-09
B46	B220	RBC Count	SBP	-0.02616	1.36E-08
B46	B112	RBC Count	FT4	0.026146	1.38E-08
B46	B55	RBC Count	TK	-0.02556	2.87E-08
B46	B187	RBC Count	NSE	0.024664	8.59E-08
B46	B228	RBC Count	OS	-0.02405	1.78E-07
B46	B73	RBC Count	ApoA	-0.02389	2.14E-07
B46	B139	RBC Count	ADA	-0.02375	2.52E-07
B46	B131	RBC Count	AST	0.023398	3.79E-07
B46	B190	RBC Count	Cre	0.022946	6.32E-07
B47	B111	RDW	TSH	0.038918	0
B47	B112	RDW	FT4	-0.04554	0
B47	B126	RDW	Alb	-0.16833	0
B47	B127	RDW	A-G ratio	-0.09883	0
B47	B130	RDW	GLB	0.054134	0
B47	B131	RDW	AST	0.092004	0

B47	B132	RDW	Dbil	0.08373	0
B47	B134	RDW	CHE	-0.05501	0
B47	B140	RDW	r-GT	0.070781	0
B47	B141	RDW	AST/ALT	0.125786	0
B47	B146	RDW	CysC	0.058354	0
B47	B153	RDW	Glu	-0.08009	0
B47	B160	RDW	PH	-0.04866	0
B47	B188	RDW	Ca	-0.05898	0
B47	B189	RDW	eGFB	-0.17463	0
B47	B190	RDW	Cre	0.07201	0
B47	B191	RDW	Urea	0.107915	0
B47	B204	RDW	Drinking	0.038502	0
B47	B209	RDW	Smoking	0.103969	0
B47	B210	RDW	Hip	-0.05137	0
B47	B215	RDW	BMI	-0.06474	0
B47	B216	RDW	BMI	-0.09744	0
B47	B218	RDW	DBP	-0.07167	0
B47	B220	RDW	SBP	0.055261	0
B47	B221	RDW	Hei	-0.07996	0
B47	B222	RDW	NS	-0.05504	0
B47	B50	RDW	eo%	0.046395	0
B47	B51	RDW	Hb	-0.124	0
B47	B52	RDW	PLT/BPC	-0.19477	0
B47	B71	RDW	TG	-0.05725	0
B47	B72	RDW	HDL-C	0.119633	0
B47	B74	RDW	ApoA1	0.047867	0
B47	B75	RDW	ApoA1/B	0.040134	0
B47	B91	RDW	TP	-0.05114	0
B47	B170	RDW	U-GLU	-0.03536	1.62E-14
B47	B113	RDW	FT3	-0.0335	3.49E-13
B47	B211	RDW	IOP-R	-0.03296	8.25E-13
B47	B129	RDW	IBIL	-0.03278	1.10E-12
B47	B212	RDW	IOP-L	-0.03277	1.13E-12
B47	B128	RDW	ALT	-0.03258	1.51E-12
B47	B138	RDW	PAB	-0.03161	6.76E-12
B47	B61	RDW	WBHSRV	-0.02982	9.51E-11
B47	B194	RDW	Cl	0.02949	1.53E-10
B47	B48	RDW	LYM%	-0.02913	2.55E-10
B47	B118	RDW	CEA1	0.028645	5.02E-10
B47	B56	RDW	IR	-0.02825	8.61E-10
B47	B150	RDW	ALP	0.026611	7.60E-09
B47	B115	RDW	TSH TT3 TT4(TT3)	-0.0265	8.74E-09
B47	B139	RDW	ADA	0.025452	3.29E-08
B47	B228	RDW	OS	0.025204	4.46E-08

B47	B70	RDW	LDL-C	-0.02515	4.76E-08
B47	B55	RDW	TK	-0.02495	6.11E-08
B47	B174	RDW	Urea/Cr	0.024325	1.29E-07
B47	B59	RDW	WBRV	-0.02366	2.79E-07
B47	B219	RDW	OT	0.023085	5.41E-07
B47	B173	RDW	NTT	0.022808	7.38E-07
B48	B123	LYM%	hs-CRP1	-0.07244	0
B48	B126	LYM%	Alb	0.057145	0
B48	B130	LYM%	GLB	0.060846	0
B48	B131	LYM%	AST	0.039981	0
B48	B132	LYM%	Dbil	-0.05999	0
B48	B146	LYM%	CysC	-0.04438	0
B48	B163	LYM%	SG	-0.05837	0
B48	B166	LYM%	Pro	-0.05933	0
B48	B174	LYM%	Urea/Cr	0.051211	0
B48	B188	LYM%	Ca	0.050221	0
B48	B189	LYM%	eGFB	0.087141	0
B48	B190	LYM%	Cre	-0.1072	0
B48	B191	LYM%	Urea	-0.07087	0
B48	B192	LYM%	UA	-0.06864	0
B48	B206	LYM%	Waist	-0.05255	0
B48	B209	LYM%	Smoking	-0.07121	0
B48	B221	LYM%	Hei	-0.04053	0
B48	B222	LYM%	NS	-0.06772	0
B48	B49	LYM%	baso%	0.049793	0
B48	B52	LYM%	PLT/BPC	0.039893	0
B48	B53	LYM%	GR%	-0.93767	0
B48	B70	LYM%	LDL-C	0.066832	0
B48	B72	LYM%	HDL-C	0.080074	0
B48	B77	LYM%	TC	0.09312	0
B48	B91	LYM%	TP	0.088987	0
B48	B127	LYM%	A-G ratio	-0.03675	1.33E-15
B48	B133	LYM%	TBIL	-0.03511	2.46E-14
B48	B153	LYM%	Glu	-0.03486	3.73E-14
B48	B111	LYM%	TSH	0.033818	2.10E-13
B48	B74	LYM%	ApoA1	0.032208	2.70E-12
B48	B175	LYM%	ALB-U	-0.03081	2.26E-11
B48	B149	LYM%	TGAb	0.030121	6.18E-11
B48	B201	LYM%	e-habits	0.030119	6.20E-11
B48	B51	LYM%	Hb	-0.02718	3.61E-09
B48	B179	LYM%	α1-MG	-0.02709	4.05E-09
B48	B86	LYM%	TBhCG	0.026692	6.85E-09
B48	B198	LYM%	P	0.026671	7.04E-09
B48	B177	LYM%	ACR	-0.02641	9.80E-09

B48	B128	LYM%	ALT	0.0261	1.46E-08
B48	B170	LYM%	U-GLU	-0.02604	1.58E-08
B48	B176	LYM%	Ucr	-0.02567	2.52E-08
B48	B148	LYM%	Anti_TPO	0.024669	8.54E-08
B48	B220	LYM%	SBP	-0.02461	9.18E-08
B48	B204	LYM%	Drinking	-0.02402	1.85E-07
B48	B140	LYM%	r-GT	-0.02372	2.62E-07
B48	B129	LYM%	IBIL	-0.02349	3.42E-07
B49	B140	baso%	r-GT	0.044011	0
B49	B209	baso%	Smoking	0.042246	0
B49	B221	baso%	Hei	0.046981	0
B49	B50	baso%	eo%	0.271707	0
B49	B51	baso%	Hb	0.06827	0
B49	B52	baso%	PLT/BPC	0.061358	0
B49	B53	baso%	GR%	-0.1712	0
B49	B70	baso%	LDL-C	0.038375	0
B49	B77	baso%	TC	0.04191	0
B49	B204	baso%	Drinking	0.037725	2.22E-16
B49	B216	baso%	BMI	0.035093	2.55E-14
B49	B128	baso%	ALT	0.030514	3.48E-11
B49	B62	baso%	WBHSRVI	0.02987	8.88E-11
B49	B174	baso%	Urea/Cr	-0.02968	1.17E-10
B49	B220	baso%	SBP	-0.02899	3.10E-10
B49	B191	baso%	Urea	-0.02898	3.15E-10
B49	B141	baso%	AST/ALT	-0.02781	1.58E-09
B49	B123	baso%	hs-CRP1	-0.02746	2.51E-09
B49	B192	baso%	UA	0.026823	5.78E-09
B49	B66	baso%	WBRSR(mPas_100)	0.02578	2.19E-08
B49	B67	baso%	WBRSR(mPas_50)	0.025595	2.75E-08
B49	B129	baso%	IBIL	-0.02558	2.81E-08
B49	B222	baso%	NS	0.02457	9.62E-08
B49	B131	baso%	AST	0.024335	1.27E-07
B49	B153	baso%	Glu	-0.02389	2.15E-07
B49	B60	baso%	WBRVI	0.023427	3.66E-07
B49	B54	baso%	ESR	-0.02292	6.48E-07
B49	B63	baso%	WBRSR(mPas_200)	0.022842	7.10E-07
B5	B6	WB-Mg	WB-Fe	0.697108	0
B5	B7	WB-Mg	WB-Cop	0.469229	0
B5	B8	WB-Mg	WB-Zinc	0.56987	0
B5	B107	WB-Mg	AnuA	-0.03099	1.72E-11
B5	B74	WB-Mg	ApoA1	-0.02291	6.58E-07
B5	B15	WB-Mg	CD3-CD19+(B cell)	-0.02257	9.57E-07
B50	B126	eo%	Alb	-0.07138	0
B50	B146	eo%	CysC	0.04181	0

B50	B174	eo%	Urea/Cr	-0.07158	0
B50	B189	eo%	eGFB	-0.082	0
B50	B190	eo%	Cre	0.089597	0
B50	B191	eo%	Urea	0.048001	0
B50	B192	eo%	UA	0.072681	0
B50	B204	eo%	Drinking	0.04504	0
B50	B206	eo%	Waist	0.047376	0
B50	B209	eo%	Smoking	0.086929	0
B50	B216	eo%	BMI	0.051107	0
B50	B221	eo%	Hei	0.070233	0
B50	B222	eo%	NS	0.063682	0
B50	B53	eo%	GR%	-0.24678	0
B50	B91	eo%	TP	-0.05461	0
B50	B153	eo%	Glu	-0.03586	6.88E-15
B50	B129	eo%	IBIL	-0.03502	2.89E-14
B50	B133	eo%	TBIL	-0.03355	3.24E-13
B50	B210	eo%	Hip	0.032014	3.64E-12
B50	B77	eo%	TC	-0.03083	2.19E-11
B50	B156	eo%	IgE	0.030229	5.28E-11
B50	B72	eo%	HDL-C	-0.02997	7.67E-11
B50	B188	eo%	Ca	-0.02865	4.98E-10
B50	B52	eo%	PLT/BPC	-0.02851	6.00E-10
B50	B160	eo%	PH	-0.02777	1.65E-09
B50	B51	eo%	Hb	0.023674	2.76E-07
B50	B211	eo%	IOP-R	-0.023	5.97E-07
B50	B220	eo%	SBP	-0.023	5.98E-07
B51	B111	Hb	TSH	-0.05688	0
B51	B113	Hb	FT3	0.093228	0
B51	B115	Hb	TSH TT3 TT4(TT3)	0.051632	0
B51	B126	Hb	Alb	0.259903	0
B51	B127	Hb	A-G ratio	0.176466	0
B51	B128	Hb	ALT	0.2346	0
B51	B129	Hb	IBIL	0.191184	0
B51	B130	Hb	GLB	-0.09754	0
B51	B131	Hb	AST	0.082942	0
B51	B132	Hb	Dbil	0.130706	0
B51	B133	Hb	TBIL	0.189252	0
B51	B134	Hb	CHE	0.081531	0
B51	B137	Hb	AFU	0.04246	0
B51	B138	Hb	PAB	0.0861	0
B51	B140	Hb	r-GT	0.159068	0
B51	B141	Hb	AST/ALT	-0.2863	0
B51	B146	Hb	CysC	-0.04701	0
B51	B148	Hb	Anti_TPO	-0.0426	0

B51	B149	Hb	TGAb	-0.03961	0
B51	B153	Hb	Glu	0.078938	0
B51	B161	Hb	LEU	-0.1193	0
B51	B163	Hb	SG	0.293968	0
B51	B166	Hb	Pro	0.109703	0
B51	B170	Hb	U-GLU	0.070397	0
B51	B173	Hb	NTT	-0.08266	0
B51	B174	Hb	Urea/Cr	-0.28019	0
B51	B176	Hb	Ucr	0.131565	0
B51	B188	Hb	Ca	0.098183	0
B51	B189	Hb	eGFB	0.175889	0
B51	B190	Hb	Cre	0.049393	0
B51	B191	Hb	Urea	-0.13771	0
B51	B192	Hb	UA	0.251602	0
B51	B194	Hb	Cl	-0.04991	0
B51	B198	Hb	P	-0.06509	0
B51	B203	Hb	DH	0.06009	0
B51	B204	Hb	Drinking	0.247168	0
B51	B205	Hb	KDA	-0.04716	0
B51	B206	Hb	Waist	0.321566	0
B51	B209	Hb	Smoking	0.241912	0
B51	B210	Hb	Hip	0.265893	0
B51	B215	Hb	BMI	0.243738	0
B51	B216	Hb	BMI	0.439802	0
B51	B218	Hb	DBP	0.267471	0
B51	B219	Hb	OT	-0.10647	0
B51	B220	Hb	SBP	-0.04305	0
B51	B221	Hb	Hei	0.437633	0
B51	B222	Hb	NS	0.454253	0
B51	B54	Hb	ESR	-0.09398	0
B51	B55	Hb	TK	-0.04205	0
B51	B56	Hb	IR	0.123996	0
B51	B58	Hb	HCT	0.119468	0
B51	B59	Hb	WBRV	0.071045	0
B51	B60	Hb	WBRVI	0.182968	0
B51	B61	Hb	WBHSRV	0.128851	0
B51	B62	Hb	WBHSRVI	0.205735	0
B51	B63	Hb	WBRSR(mPas_200)	0.205492	0
B51	B64	Hb	WBRSR(mPas_5)	0.199835	0
B51	B65	Hb	WBRSR(mPas_1)	0.184509	0
B51	B66	Hb	WBRSR(mPas_100)	0.205898	0
B51	B67	Hb	WBRSR(mPas_50)	0.206341	0
B51	B70	Hb	LDL-C	0.090872	0
B51	B71	Hb	TG	0.16395	0

B51	B72	Hb	HDL-C	-0.22883	0
B51	B74	Hb	ApoA1	-0.08345	0
B51	B75	Hb	ApoA1/B	-0.07328	0
B51	B76	Hb	ApoB	0.055222	0
B51	B77	Hb	TC	0.04171	0
B51	B79	Hb	hb V -dna	-0.04073	0
B51	B91	Hb	TP	0.066506	0
B51	B172	Hb	KB	0.036271	3.55E-15
B51	B57	Hb	RBCAI	0.032907	9.04E-13
B51	B179	Hb	α1-MG	0.032406	1.98E-12
B51	B193	Hb	K	-0.03143	8.93E-12
B51	B197	Hb	AG	0.029954	7.87E-11
B51	B86	Hb	TBhCG	-0.02957	1.36E-10
B51	B119	Hb	Hcv-rna	-0.02922	2.26E-10
B51	B114	Hb	TSH TT3 TT4(TT4)	-0.02759	2.11E-09
B51	B73	Hb	ApoA	-0.02745	2.52E-09
B51	B139	Hb	ADA	-0.0262	1.29E-08
B51	B228	Hb	OS	-0.02617	1.33E-08
B51	B187	Hb	NSE	0.025624	2.66E-08
B51	B142	Hb	25-(OH)D	-0.02526	4.18E-08
B51	B157	Hb	IgG	-0.02507	5.25E-08
B51	B160	Hb	PH	-0.02274	7.92E-07
B52	B112	PLT/BPC	FT4	0.038456	0
B52	B127	PLT/BPC	A-G ratio	-0.0916	0
B52	B128	PLT/BPC	ALT	-0.06799	0
B52	B129	PLT/BPC	IBIL	-0.11232	0
B52	B130	PLT/BPC	GLB	0.100141	0
B52	B131	PLT/BPC	AST	-0.08779	0
B52	B132	PLT/BPC	Dbil	-0.17739	0
B52	B133	PLT/BPC	TBIL	-0.1384	0
B52	B146	PLT/BPC	CysC	-0.04042	0
B52	B160	PLT/BPC	PH	0.043275	0
B52	B188	PLT/BPC	Ca	0.065669	0
B52	B189	PLT/BPC	eGFB	0.097005	0
B52	B190	PLT/BPC	Cre	-0.07461	0
B52	B191	PLT/BPC	Urea	-0.10792	0
B52	B192	PLT/BPC	UA	-0.05843	0
B52	B198	PLT/BPC	P	0.043334	0
B52	B220	PLT/BPC	SBP	-0.04244	0
B52	B70	PLT/BPC	LDL-C	0.134981	0
B52	B77	PLT/BPC	TC	0.122747	0
B52	B91	PLT/BPC	TP	0.093164	0
B52	B134	PLT/BPC	CHE	0.037605	4.44E-16
B52	B153	PLT/BPC	Glu	-0.03612	4.44E-15

B52	B139	PLT/BPC	ADA	-0.03601	5.33E-15
B52	B76	PLT/BPC	ApoB	0.033663	2.70E-13
B52	B222	PLT/BPC	NS	-0.0318	5.08E-12
B52	B141	PLT/BPC	AST/ALT	0.030629	2.94E-11
B52	B206	PLT/BPC	Waist	-0.03046	3.79E-11
B52	B193	PLT/BPC	K	0.028477	6.32E-10
B52	B201	PLT/BPC	e-habits	-0.02465	8.77E-08
B52	B161	PLT/BPC	LEU	0.023839	2.28E-07
B52	B195	PLT/BPC	Na	-0.02342	3.71E-07
B52	B194	PLT/BPC	Cl	-0.02277	7.71E-07
B52	B53	PLT/BPC	GR%	-0.02255	9.81E-07
B53	B123	GR%	hs-CRP1	0.066752	0
B53	B127	GR%	A-G ratio	0.05445	0
B53	B130	GR%	GLB	-0.06088	0
B53	B131	GR%	AST	-0.05996	0
B53	B132	GR%	Dbil	0.040137	0
B53	B153	GR%	Glu	0.047207	0
B53	B166	GR%	Pro	0.046387	0
B53	B190	GR%	Cre	0.060129	0
B53	B191	GR%	Urea	0.040244	0
B53	B70	GR%	LDL-C	-0.04986	0
B53	B72	GR%	HDL-C	-0.06959	0
B53	B77	GR%	TC	-0.06391	0
B53	B91	GR%	TP	-0.05693	0
B53	B163	GR%	SG	0.035985	5.55E-15
B53	B128	GR%	ALT	-0.03404	1.45E-13
B53	B111	GR%	TSH	-0.03367	2.68E-13
B53	B220	GR%	SBP	0.032696	1.26E-12
B53	B218	GR%	DBP	0.032083	3.27E-12
B53	B189	GR%	eGFB	-0.03144	8.69E-12
B53	B133	GR%	TBIL	0.031112	1.43E-11
B53	B201	GR%	e-habits	-0.03056	3.25E-11
B53	B209	GR%	Smoking	0.029871	8.87E-11
B53	B222	GR%	NS	0.029738	1.08E-10
B53	B175	GR%	ALB-U	0.028979	3.15E-10
B53	B170	GR%	U-GLU	0.028802	4.03E-10
B53	B192	GR%	UA	0.02636	1.05E-08
B53	B74	GR%	ApoA1	-0.02634	1.08E-08
B53	B188	GR%	Ca	-0.02593	1.80E-08
B53	B149	GR%	TGAb	-0.02568	2.49E-08
B53	B129	GR%	IBIL	0.025199	4.49E-08
B53	B179	GR%	α1-MG	0.024867	6.73E-08
B53	B206	GR%	Waist	0.024421	1.15E-07
B53	B86	GR%	TBhCG	-0.02389	2.14E-07

B53	B177	GR%	ACR	0.023637	2.88E-07
B53	B198	GR%	P	-0.02352	3.28E-07
B53	B112	GR%	FT4	0.022745	7.92E-07
B54	B107	ESR	AnuA	-0.05372	0
B54	B114	ESR	TSH TT3 TT4(TT4)	0.054765	0
B54	B121	ESR	C3	0.04571	0
B54	B123	ESR	hs-CRP1	0.149412	0
B54	B126	ESR	Alb	-0.05478	0
B54	B127	ESR	A-G ratio	-0.10239	0
B54	B129	ESR	IBIL	-0.05183	0
B54	B130	ESR	GLB	0.102625	0
B54	B132	ESR	Dbil	-0.04268	0
B54	B133	ESR	TBIL	-0.05342	0
B54	B136	ESR	5'NT	0.042129	0
B54	B138	ESR	PAB	-0.05854	0
B54	B139	ESR	ADA	0.04872	0
B54	B141	ESR	AST/ALT	0.04452	0
B54	B155	ESR	IgA	0.059265	0
B54	B157	ESR	IgG	0.085224	0
B54	B174	ESR	Urea/Cr	0.048926	0
B54	B186	ESR	PFC	0.106369	0
B54	B198	ESR	P	0.051977	0
B54	B216	ESR	BMI	-0.04426	0
B54	B221	ESR	Hei	-0.07797	0
B54	B222	ESR	NS	-0.05313	0
B54	B55	ESR	TK	-0.04341	0
B54	B56	ESR	IR	-0.24523	0
B54	B58	ESR	HCT	-0.1416	0
B54	B59	ESR	WBRV	0.054587	0
B54	B60	ESR	WBRVI	-0.27233	0
B54	B61	ESR	WBHSRV	-0.08489	0
B54	B62	ESR	WBHSRVI	-0.32815	0
B54	B63	ESR	WBRSR(mPas_200)	-0.16293	0
B54	B64	ESR	WBRSR(mPas_5)	-0.14181	0
B54	B65	ESR	WBRSR(mPas_1)	-0.12822	0
B54	B66	ESR	WBRSR(mPas_100)	-0.14984	0
B54	B67	ESR	WBRSR(mPas_50)	-0.14885	0
B54	B69	ESR	PV	0.45622	0
B54	B70	ESR	LDL-C	0.04978	0
B54	B73	ESR	ApoA	0.081539	0
B54	B74	ESR	ApoA1	0.087368	0
B54	B76	ESR	ApoB	0.090925	0
B54	B77	ESR	TC	0.046984	0
B54	B91	ESR	TP	0.060266	0

B54	B101	ESR	anti-Sc70	0.037378	4.44E-16
B54	B161	ESR	LEU	0.035968	5.77E-15
B54	B111	ESR	TSH	0.034384	8.30E-14
B54	B104	ESR	anti-SSB	0.034269	1.00E-13
B54	B156	ESR	IgE	0.034155	1.21E-13
B54	B163	ESR	SG	-0.03328	5.00E-13
B54	B204	ESR	Drinking	-0.03148	8.27E-12
B54	B72	ESR	HDL-C	0.031211	1.23E-11
B54	B192	ESR	UA	-0.0302	5.53E-11
B54	B134	ESR	CHE	-0.03012	6.17E-11
B54	B122	ESR	C4	0.029986	7.51E-11
B54	B97	ESR	dsDNA	0.028597	5.35E-10
B54	B177	ESR	ACR	0.027515	2.32E-09
B54	B75	ESR	ApoA1/B	-0.02656	8.16E-09
B54	B209	ESR	Smoking	-0.02627	1.17E-08
B54	B148	ESR	Anti_TPO	0.026241	1.22E-08
B54	B176	ESR	Ucr	-0.02458	9.53E-08
B54	B103	ESR	anti-SSA/Ro	0.023952	2.00E-07
B54	B191	ESR	Urea	0.023926	2.06E-07
B54	B137	ESR	AFU	-0.02375	2.52E-07
B54	B92	ESR	ALDH2-Glu504Glu	-0.02373	2.60E-07
B54	B93	ESR	ALDH2-Glu504Lys	0.023647	2.85E-07
B54	B158	ESR	IgM	0.023318	4.15E-07
B55	B121	TK	C3	-0.06814	0
B55	B122	TK	C4	-0.05776	0
B55	B56	TK	IR	0.724677	0
B55	B57	TK	RBCAI	-0.16718	0
B55	B58	TK	HCT	-0.33158	0
B55	B59	TK	WBRV	0.384377	0
B55	B60	TK	WBRVI	0.181103	0
B55	B61	TK	WBHSRV	0.642358	0
B55	B62	TK	WBHSRVI	0.332604	0
B55	B63	TK	WBRSR(mPas_200)	0.217128	0
B55	B64	TK	WBRSR(mPas_5)	0.141433	0
B55	B65	TK	WBRSR(mPas_1)	0.091863	0
B55	B66	TK	WBRSR(mPas_100)	0.208069	0
B55	B67	TK	WBRSR(mPas_50)	0.197747	0
B55	B69	TK	PV	-0.30643	0
B55	B74	TK	ApoA1	-0.04166	0
B55	B78	TK	HBcAb	0.039546	0
B55	B107	TK	AnuA	0.031246	1.17E-11
B55	B183	TK	PTA	-0.03054	3.35E-11
B55	B199	TK	Mg	-0.02985	9.17E-11
B55	B120	TK	HCV-cAg	0.029611	1.29E-10

B55	B198	TK	P	0.02934	1.89E-10
B55	B73	TK	ApoA	-0.02499	5.79E-08
B55	B84	TK	C-PSA	-0.02437	1.23E-07
B55	B100	TK	anti-Ro52	-0.02393	2.05E-07
B55	B123	TK	hs-CRP1	-0.02309	5.35E-07
B55	B81	TK	TSGF	0.02266	8.69E-07
B55	B115	TK	TSH TT3 TT4(TT3)	-0.02259	9.35E-07
B56	B107	IR	AnuA	0.038004	0
B56	B121	IR	C3	-0.06009	0
B56	B122	IR	C4	-0.04841	0
B56	B129	IR	IBIL	0.038663	0
B56	B133	IR	TBIL	0.039896	0
B56	B134	IR	CHE	0.058681	0
B56	B138	IR	PAB	0.054626	0
B56	B163	IR	SG	0.038207	0
B56	B174	IR	Urea/Cr	-0.04785	0
B56	B192	IR	UA	0.041612	0
B56	B194	IR	Cl	-0.03811	0
B56	B206	IR	Waist	0.048595	0
B56	B210	IR	Hip	0.043317	0
B56	B215	IR	BMI	0.039249	0
B56	B216	IR	BMI	0.063912	0
B56	B221	IR	Hei	0.056905	0
B56	B222	IR	NS	0.062755	0
B56	B57	IR	RBCAI	-0.05919	0
B56	B58	IR	HCT	0.104578	0
B56	B59	IR	WBRV	0.540399	0
B56	B60	IR	WBRVI	0.683507	0
B56	B61	IR	WBHSRV	0.9229	0
B56	B62	IR	WBHSRVI	0.882109	0
B56	B63	IR	WBRSR(mPas_200)	0.767481	0
B56	B64	IR	WBRSR(mPas_5)	0.678806	0
B56	B65	IR	WBRSR(mPas_1)	0.59463	0
B56	B66	IR	WBRSR(mPas_100)	0.755023	0
B56	B67	IR	WBRSR(mPas_50)	0.747348	0
B56	B69	IR	PV	-0.29974	0
B56	B73	IR	ApoA	-0.04144	0
B56	B74	IR	ApoA1	-0.0908	0
B56	B75	IR	ApoA1/B	-0.05795	0
B56	B72	IR	HDL-C	-0.03736	4.44E-16
B56	B100	IR	anti-Ro52	-0.03693	1.11E-15
B56	B141	IR	AST/ALT	-0.0353	1.82E-14
B56	B157	IR	IgG	-0.03424	1.06E-13
B56	B132	IR	Dbil	0.033201	5.67E-13

B56	B187	IR	NSE	0.032203	2.72E-12
B56	B115	IR	TSH TT3 TT4(TT3)	0.031836	4.79E-12
B56	B176	IR	Ucr	0.031369	9.73E-12
B56	B78	IR	HBcAb	0.029584	1.34E-10
B56	B120	IR	HCV-cAg	0.029284	2.05E-10
B56	B218	IR	DBP	0.028832	3.86E-10
B56	B128	IR	ALT	0.028802	4.03E-10
B56	B197	IR	AG	0.027877	1.43E-09
B56	B71	IR	TG	0.027832	1.52E-09
B56	B76	IR	ApoB	0.027123	3.90E-09
B56	B127	IR	A-G ratio	0.02657	8.02E-09
B56	B183	IR	PTA	-0.02547	3.21E-08
B56	B113	IR	FT3	0.025407	3.48E-08
B56	B140	IR	r-GT	0.024811	7.20E-08
B56	B104	IR	anti-SSB	-0.02323	4.59E-07
B56	B126	IR	Alb	0.023188	4.81E-07
B56	B207	IR	WHR	0.022585	9.45E-07
B57	B121	RBCAI	C3	-0.06254	0
B57	B122	RBCAI	C4	-0.04609	0
B57	B58	RBCAI	HCT	0.059074	0
B57	B59	RBCAI	WBRV	0.668907	0
B57	B60	RBCAI	WBRVI	0.570792	0
B57	B61	RBCAI	WBHSRV	-0.05768	0
B57	B64	RBCAI	WBRSR(mPas_5)	0.392466	0
B57	B65	RBCAI	WBRSR(mPas_1)	0.563471	0
B57	B66	RBCAI	WBRSR(mPas_100)	0.064714	0
B57	B67	RBCAI	WBRSR(mPas_50)	0.123325	0
B57	B76	RBCAI	ApoB	-0.04103	0
B57	B81	RBCAI	TSGF	-0.05233	0
B57	B75	RBCAI	ApoA1/B	0.035903	6.44E-15
B57	B135	RBCAI	TBA	0.032947	8.48E-13
B57	B92	RBCAI	ALDH2-Glu504Glu	0.032662	1.33E-12
B57	B93	RBCAI	ALDH2-Glu504Lys	-0.03249	1.73E-12
B57	B102	RBCAI	anti-Sm	0.031577	7.10E-12
B57	B105	RBCAI	anti-U1RNP	0.03062	2.98E-11
B57	B189	RBCAI	eGFB	0.027472	2.46E-09
B57	B127	RBCAI	A-G ratio	0.027409	2.68E-09
B57	B112	RBCAI	FT4	0.026954	4.87E-09
B57	B78	RBCAI	HBcAb	-0.02614	1.38E-08
B57	B130	RBCAI	GLB	-0.02601	1.64E-08
B57	B63	RBCAI	WBRSR(mPas_200)	0.025169	4.66E-08
B57	B74	RBCAI	ApoA1	0.024914	6.36E-08
B57	B183	RBCAI	PTA	-0.02479	7.42E-08
B57	B111	RBCAI	TSH	-0.02477	7.61E-08

B57	B186	RBCAI	PFC	-0.02434	1.26E-07
B57	B107	RBCAI	AnuA	-0.02425	1.40E-07
B57	B209	RBCAI	Smoking	0.02407	1.74E-07
B57	B72	RBCAI	HDL-C	0.023283	4.32E-07
B58	B125	HCT	Cor	0.082765	0
B58	B134	HCT	CHE	0.04948	0
B58	B137	HCT	AFU	0.048203	0
B58	B138	HCT	PAB	0.044427	0
B58	B192	HCT	UA	0.0384	0
B58	B216	HCT	BMI	0.043763	0
B58	B221	HCT	Hei	0.045092	0
B58	B222	HCT	NS	0.050237	0
B58	B60	HCT	WBRVI	0.323094	0
B58	B61	HCT	WBHSRV	0.098564	0
B58	B62	HCT	WBHSRVI	0.361107	0
B58	B63	HCT	WBRSR(mPas_200)	0.377236	0
B58	B64	HCT	WBRSR(mPas_5)	0.359795	0
B58	B65	HCT	WBRSR(mPas_1)	0.330705	0
B58	B66	HCT	WBRSR(mPas_100)	0.368967	0
B58	B67	HCT	WBRSR(mPas_50)	0.370445	0
B58	B74	HCT	ApoA1	-0.03828	0
B58	B92	HCT	ALDH2-Glu504Glu	-0.06165	0
B58	B93	HCT	ALDH2-Glu504Lys	0.066113	0
B58	B163	HCT	SG	0.037085	8.88E-16
B58	B206	HCT	Waist	0.035834	7.11E-15
B58	B69	HCT	PV	0.035241	2.00E-14
B58	B176	HCT	Ucr	0.034974	3.11E-14
B58	B141	HCT	AST/ALT	-0.03471	4.84E-14
B58	B75	HCT	ApoA1/B	-0.03361	2.95E-13
B58	B174	HCT	Urea/Cr	-0.03197	3.87E-12
B58	B128	HCT	ALT	0.02719	3.57E-09
B58	B72	HCT	HDL-C	-0.02665	7.21E-09
B58	B129	HCT	IBIL	0.025778	2.19E-08
B58	B133	HCT	TBIL	0.024961	6.01E-08
B58	B204	HCT	Drinking	0.024621	9.05E-08
B58	B199	HCT	Mg	0.024273	1.37E-07
B58	B209	HCT	Smoking	0.023267	4.40E-07
B58	B76	HCT	ApoB	0.023125	5.17E-07
B58	B210	HCT	Hip	0.023016	5.84E-07
B58	B113	HCT	FT3	0.022847	7.06E-07
B59	B112	WBRV	FT4	0.043356	0
B59	B121	WBRV	C3	-0.06956	0
B59	B122	WBRV	C4	-0.0546	0
B59	B134	WBRV	CHE	0.039312	0

B59	B60	WBRV	WBRVI	0.769684	0
B59	B61	WBRV	WBHSRV	0.64807	0
B59	B62	WBRV	WBHSRVI	0.464133	0
B59	B63	WBRV	WBRSR(mPas_200)	0.548887	0
B59	B64	WBRV	WBRSR(mPas_5)	0.760027	0
B59	B65	WBRV	WBRSR(mPas_1)	0.82402	0
B59	B66	WBRV	WBRSR(mPas_100)	0.568793	0
B59	B67	WBRV	WBRSR(mPas_50)	0.605986	0
B59	B69	WBRV	PV	0.186082	0
B59	B91	WBRV	TP	0.037321	4.44E-16
B59	B115	WBRV	TSH TT3 TT4(TT3)	0.033932	1.75E-13
B59	B81	WBRV	TSGF	-0.0339	1.85E-13
B59	B125	WBRV	Cor	0.033854	1.98E-13
B59	B194	WBRV	Cl	-0.0321	3.18E-12
B59	B189	WBRV	eGFB	0.031924	4.18E-12
B59	B126	WBRV	Alb	0.031858	4.62E-12
B59	B135	WBRV	TBA	0.031434	8.83E-12
B59	B120	WBRV	HCV-cAg	0.031088	1.49E-11
B59	B222	WBRV	NS	0.029152	2.47E-10
B59	B123	WBRV	hs-CRP1	0.027931	1.33E-09
B59	B163	WBRV	SG	0.027852	1.48E-09
B59	B107	WBRV	AnuA	-0.02757	2.16E-09
B59	B210	WBRV	Hip	0.027474	2.45E-09
B59	B216	WBRV	BMI	0.026799	5.96E-09
B59	B206	WBRV	Waist	0.025957	1.75E-08
B59	B113	WBRV	FT3	0.025483	3.17E-08
B59	B93	WBRV	ALDH2-Glu504Lys	-0.02544	3.32E-08
B59	B114	WBRV	TSH TT3 TT4(TT4)	0.025198	4.49E-08
B59	B92	WBRV	ALDH2-Glu504Glu	0.025102	5.06E-08
B59	B97	WBRV	dsDNA	0.025026	5.55E-08
B59	B129	WBRV	IBIL	0.024349	1.25E-07
B59	B138	WBRV	PAB	0.024052	1.78E-07
B59	B221	WBRV	Hei	0.023396	3.80E-07
B59	B70	WBRV	LDL-C	0.02328	4.33E-07
B59	B140	WBRV	r-GT	0.023001	5.94E-07
B59	B183	WBRV	PTA	-0.02297	6.13E-07
B59	B124	WBRV	ACTH	0.022543	9.90E-07
B6	B7	WB-Fe	WB-Cop	0.330578	0
B6	B8	WB-Fe	WB-Zinc	0.643656	0
B6	B113	WB-Fe	FT3	-0.02996	7.81E-11
B6	B29	WB-Fe	E2	0.023378	3.88E-07
B6	B33	WB-Fe	Prog	0.02272	8.14E-07
B6	B74	WB-Fe	ApoA1	-0.02254	9.90E-07
B60	B113	WBRVI	FT3	0.048256	0

B60	B115	WBRVI	TSH TT3 TT4(TT3)	0.052176	0
B60	B121	WBRVI	C3	-0.06376	0
B60	B122	WBRVI	C4	-0.04884	0
B60	B127	WBRVI	A-G ratio	0.041825	0
B60	B128	WBRVI	ALT	0.038504	0
B60	B129	WBRVI	IBIL	0.046607	0
B60	B133	WBRVI	TBIL	0.045026	0
B60	B134	WBRVI	CHE	0.06543	0
B60	B138	WBRVI	PAB	0.064358	0
B60	B141	WBRVI	AST/ALT	-0.04165	0
B60	B163	WBRVI	SG	0.064981	0
B60	B174	WBRVI	Urea/Cr	-0.05655	0
B60	B176	WBRVI	Ucr	0.050442	0
B60	B192	WBRVI	UA	0.053938	0
B60	B204	WBRVI	Drinking	0.039644	0
B60	B206	WBRVI	Waist	0.06037	0
B60	B209	WBRVI	Smoking	0.040517	0
B60	B210	WBRVI	Hip	0.049517	0
B60	B216	WBRVI	BMI	0.075365	0
B60	B218	WBRVI	DBP	0.03876	0
B60	B221	WBRVI	Hei	0.077753	0
B60	B222	WBRVI	NS	0.083412	0
B60	B61	WBRVI	WBHSRV	0.642513	0
B60	B62	WBRVI	WBHSRVI	0.822324	0
B60	B63	WBRVI	WBRSR(mPas_200)	0.753455	0
B60	B64	WBRVI	WBRSR(mPas_5)	0.902817	0
B60	B65	WBRVI	WBRSR(mPas_1)	0.929779	0
B60	B66	WBRVI	WBRSR(mPas_100)	0.768266	0
B60	B67	WBRVI	WBRSR(mPas_50)	0.798028	0
B60	B69	WBRVI	PV	-0.17913	0
B60	B73	WBRVI	ApoA	-0.0419	0
B60	B74	WBRVI	ApoA1	-0.06563	0
B60	B75	WBRVI	ApoA1/B	-0.03775	2.22E-16
B60	B157	WBRVI	IgG	-0.03759	4.44E-16
B60	B215	WBRVI	BMI	0.037292	4.44E-16
B60	B140	WBRVI	r-GT	0.037051	8.88E-16
B60	B111	WBRVI	TSH	-0.03626	3.55E-15
B60	B126	WBRVI	Alb	0.035748	8.44E-15
B60	B130	WBRVI	GLB	-0.03344	3.87E-13
B60	B198	WBRVI	P	-0.03129	1.10E-11
B60	B194	WBRVI	Cl	-0.0306	3.09E-11
B60	B125	WBRVI	Cor	0.029382	1.79E-10
B60	B100	WBRVI	anti-Ro52	-0.02912	2.57E-10
B60	B135	WBRVI	TBA	0.028419	6.85E-10

B60	B132	WBRVI	Dbil	0.028222	8.97E-10
B60	B148	WBRVI	Anti_TPO	-0.02807	1.11E-09
B60	B124	WBRVI	ACTH	0.02759	2.10E-09
B60	B72	WBRVI	HDL-C	-0.02705	4.29E-09
B60	B149	WBRVI	TGAb	-0.02678	6.09E-09
B60	B161	WBRVI	LEU	-0.02591	1.85E-08
B60	B79	WBRVI	hb V -dna	-0.02503	5.51E-08
B60	B104	WBRVI	anti-SSB	-0.0246	9.23E-08
B60	B189	WBRVI	eGFB	0.024296	1.33E-07
B60	B139	WBRVI	ADA	-0.02425	1.40E-07
B60	B183	WBRVI	PTA	-0.0235	3.36E-07
B60	B92	WBRVI	ALDH2-Glu504Glu	0.023504	3.36E-07
B60	B187	WBRVI	NSE	0.023457	3.54E-07
B61	B115	WBHSRV	TSH TT3 TT4(TT3)	0.051519	0
B61	B120	WBHSRV	HCV-cAg	0.047139	0
B61	B121	WBHSRV	C3	-0.05693	0
B61	B122	WBHSRV	C4	-0.04158	0
B61	B134	WBHSRV	CHE	0.075461	0
B61	B138	WBHSRV	PAB	0.049545	0
B61	B174	WBHSRV	Urea/Cr	-0.04272	0
B61	B192	WBHSRV	UA	0.044204	0
B61	B194	WBHSRV	Cl	-0.05968	0
B61	B206	WBHSRV	Waist	0.049541	0
B61	B210	WBHSRV	Hip	0.0439	0
B61	B215	WBHSRV	BMI	0.041511	0
B61	B216	WBHSRV	BMI	0.060095	0
B61	B221	WBHSRV	Hei	0.048223	0
B61	B222	WBHSRV	NS	0.056294	0
B61	B62	WBHSRV	WBHSRVI	0.826278	0
B61	B63	WBHSRV	WBRSR(mPas_200)	0.870694	0
B61	B64	WBHSRV	WBRSR(mPas_5)	0.779342	0
B61	B65	WBHSRV	WBRSR(mPas_1)	0.684366	0
B61	B66	WBHSRV	WBRSR(mPas_100)	0.857048	0
B61	B67	WBHSRV	WBRSR(mPas_50)	0.848976	0
B61	B69	WBHSRV	PV	0.074632	0
B61	B71	WBHSRV	TG	0.0465	0
B61	B74	WBHSRV	ApoA1	-0.07042	0
B61	B75	WBHSRV	ApoA1/B	-0.07439	0
B61	B76	WBHSRV	ApoB	0.072708	0
B61	B91	WBHSRV	TP	0.047119	0
B61	B126	WBHSRV	Alb	0.037248	4.44E-16
B61	B163	WBHSRV	SG	0.037581	4.44E-16
B61	B129	WBHSRV	IBIL	0.037195	6.66E-16
B61	B133	WBHSRV	TBIL	0.036478	2.22E-15

B61	B140	WBHSRV	r-GT	0.035153	2.31E-14
B61	B186	WBHSRV	PFC	0.034588	5.93E-14
B61	B125	WBHSRV	Cor	0.034263	1.01E-13
B61	B218	WBHSRV	DBP	0.033778	2.24E-13
B61	B72	WBHSRV	HDL-C	-0.03282	1.04E-12
B61	B123	WBHSRV	hs-CRP1	0.03224	2.57E-12
B61	B113	WBHSRV	FT3	0.032231	2.60E-12
B61	B100	WBHSRV	anti-Ro52	-0.03206	3.40E-12
B61	B187	WBHSRV	NSE	0.030883	2.02E-11
B61	B176	WBHSRV	Ucr	0.030793	2.30E-11
B61	B128	WBHSRV	ALT	0.030396	4.14E-11
B61	B136	WBHSRV	5'NT	0.029432	1.66E-10
B61	B188	WBHSRV	Ca	0.029218	2.25E-10
B61	B153	WBHSRV	Glu	0.028438	6.67E-10
B61	B141	WBHSRV	AST/ALT	-0.02811	1.05E-09
B61	B197	WBHSRV	AG	0.027895	1.40E-09
B61	B130	WBHSRV	GLB	0.027613	2.04E-09
B61	B124	WBHSRV	ACTH	0.025077	5.21E-08
B61	B132	WBHSRV	Dbil	0.025033	5.50E-08
B61	B78	WBHSRV	HBcAb	0.024854	6.84E-08
B61	B73	WBHSRV	ApoA	-0.02479	7.38E-08
B61	B207	WBHSRV	WHR	0.023616	2.95E-07
B61	B170	WBHSRV	U-GLU	0.023577	3.09E-07
B61	B77	WBHSRV	TC	0.0235	3.37E-07
B61	B200	WBHSRV	OP	-0.02316	4.96E-07
B62	B113	WBHSRVI	FT3	0.049634	0
B62	B115	WBHSRVI	TSH TT3 TT4(TT3)	0.061179	0
B62	B121	WBHSRVI	C3	-0.0498	0
B62	B128	WBHSRVI	ALT	0.045958	0
B62	B129	WBHSRVI	IBIL	0.052546	0
B62	B132	WBHSRVI	Dbil	0.040108	0
B62	B133	WBHSRVI	TBIL	0.053074	0
B62	B134	WBHSRVI	CHE	0.083916	0
B62	B138	WBHSRVI	PAB	0.074372	0
B62	B140	WBHSRVI	r-GT	0.041742	0
B62	B141	WBHSRVI	AST/ALT	-0.05685	0
B62	B157	WBHSRVI	IgG	-0.04256	0
B62	B163	WBHSRVI	SG	0.064706	0
B62	B174	WBHSRVI	Urea/Cr	-0.06415	0
B62	B176	WBHSRVI	Ucr	0.053181	0
B62	B192	WBHSRVI	UA	0.063557	0
B62	B194	WBHSRVI	Cl	-0.0488	0
B62	B204	WBHSRVI	Drinking	0.038508	0
B62	B206	WBHSRVI	Waist	0.070531	0

B62	B210	WBHSRVI	Hip	0.055909	0
B62	B215	WBHSRVI	BMI	0.05095	0
B62	B216	WBHSRVI	BMI	0.090127	0
B62	B218	WBHSRVI	DBP	0.043438	0
B62	B221	WBHSRVI	Hei	0.085913	0
B62	B222	WBHSRVI	NS	0.093531	0
B62	B63	WBHSRVI	WBRSR(mPas_200)	0.912713	0
B62	B64	WBHSRVI	WBRSR(mPas_5)	0.840143	0
B62	B65	WBHSRVI	WBRSR(mPas_1)	0.754901	0
B62	B66	WBHSRVI	WBRSR(mPas_100)	0.902629	0
B62	B67	WBHSRVI	WBRSR(mPas_50)	0.898357	0
B62	B69	WBHSRVI	PV	-0.22649	0
B62	B72	WBHSRVI	HDL-C	-0.05056	0
B62	B73	WBHSRVI	ApoA	-0.0427	0
B62	B74	WBHSRVI	ApoA1	-0.10202	0
B62	B75	WBHSRVI	ApoA1/B	-0.07662	0
B62	B76	WBHSRVI	ApoB	0.04825	0
B62	B100	WBHSRVI	anti-Ro52	-0.03778	2.22E-16
B62	B126	WBHSRVI	Alb	0.035816	7.55E-15
B62	B187	WBHSRVI	NSE	0.035795	7.77E-15
B62	B122	WBHSRVI	C4	-0.03502	2.86E-14
B62	B209	WBHSRVI	Smoking	0.034583	5.97E-14
B62	B107	WBHSRVI	AnuA	0.034316	9.28E-14
B62	B197	WBHSRVI	AG	0.033267	5.10E-13
B62	B127	WBHSRVI	A-G ratio	0.032687	1.28E-12
B62	B161	WBHSRVI	LEU	-0.02947	1.57E-10
B62	B125	WBHSRVI	Cor	0.029424	1.68E-10
B62	B111	WBHSRVI	TSH	-0.02924	2.20E-10
B62	B198	WBHSRVI	P	-0.02908	2.73E-10
B62	B137	WBHSRVI	AFU	0.028718	4.53E-10
B62	B71	WBHSRVI	TG	0.02857	5.56E-10
B62	B148	WBHSRVI	Anti_TPO	-0.02837	7.29E-10
B62	B79	WBHSRVI	hb V -dna	-0.02827	8.38E-10
B62	B124	WBHSRVI	ACTH	0.02759	2.10E-09
B62	B207	WBHSRVI	WHR	0.02631	1.12E-08
B62	B149	WBHSRVI	TGAb	-0.02417	1.54E-07
B62	B104	WBHSRVI	anti-SSB	-0.02413	1.62E-07
B62	B101	WBHSRVI	anti-Sc70	-0.02412	1.65E-07
B62	B130	WBHSRVI	GLB	-0.02326	4.43E-07
B62	B188	WBHSRVI	Ca	0.023256	4.46E-07
B63	B113	WBRSR(mPas_200)	FT3	0.052978	0
B63	B115	WBRSR(mPas_200)	TSH TT3 TT4(TT3)	0.078079	0
B63	B121	WBRSR(mPas_200)	C3	-0.04495	0
B63	B123	WBRSR(mPas_200)	hs-CRP1	0.045571	0

B63	B125	WBRSR(mPas_200)	Cor	0.04916	0
B63	B126	WBRSR(mPas_200)	Alb	0.04778	0
B63	B128	WBRSR(mPas_200)	ALT	0.045886	0
B63	B129	WBRSR(mPas_200)	IBIL	0.052816	0
B63	B133	WBRSR(mPas_200)	TBIL	0.050873	0
B63	B134	WBRSR(mPas_200)	CHE	0.095918	0
B63	B138	WBRSR(mPas_200)	PAB	0.06557	0
B63	B140	WBRSR(mPas_200)	r-GT	0.05149	0
B63	B141	WBRSR(mPas_200)	AST/ALT	-0.04688	0
B63	B163	WBRSR(mPas_200)	SG	0.062746	0
B63	B174	WBRSR(mPas_200)	Urea/Cr	-0.05981	0
B63	B176	WBRSR(mPas_200)	Ucr	0.051716	0
B63	B188	WBRSR(mPas_200)	Ca	0.045218	0
B63	B192	WBRSR(mPas_200)	UA	0.065719	0
B63	B194	WBRSR(mPas_200)	Cl	-0.0678	0
B63	B206	WBRSR(mPas_200)	Waist	0.068424	0
B63	B210	WBRSR(mPas_200)	Hip	0.052874	0
B63	B215	WBRSR(mPas_200)	BMI	0.049818	0
B63	B216	WBRSR(mPas_200)	BMI	0.082537	0
B63	B218	WBRSR(mPas_200)	DBP	0.045252	0
B63	B221	WBRSR(mPas_200)	Hei	0.075091	0
B63	B222	WBRSR(mPas_200)	NS	0.083695	0
B63	B64	WBRSR(mPas_200)	WBRSR(mPas_5)	0.919882	0
B63	B65	WBRSR(mPas_200)	WBRSR(mPas_1)	0.827608	0
B63	B66	WBRSR(mPas_200)	WBRSR(mPas_100)	0.984721	0
B63	B67	WBRSR(mPas_200)	WBRSR(mPas_50)	0.979905	0
B63	B69	WBRSR(mPas_200)	PV	0.172005	0
B63	B71	WBRSR(mPas_200)	TG	0.046139	0
B63	B72	WBRSR(mPas_200)	HDL-C	-0.04461	0
B63	B74	WBRSR(mPas_200)	ApoA1	-0.07945	0
B63	B75	WBRSR(mPas_200)	ApoA1/B	-0.09066	0
B63	B76	WBRSR(mPas_200)	ApoB	0.092255	0
B63	B91	WBRSR(mPas_200)	TP	0.05392	0
B63	B137	WBRSR(mPas_200)	AFU	0.034305	9.46E-14
B63	B204	WBRSR(mPas_200)	Drinking	0.03272	1.21E-12
B63	B187	WBRSR(mPas_200)	NSE	0.032519	1.66E-12
B63	B124	WBRSR(mPas_200)	ACTH	0.032404	1.99E-12
B63	B120	WBRSR(mPas_200)	HCV-cAg	0.031862	4.60E-12
B63	B132	WBRSR(mPas_200)	Dbil	0.031255	1.16E-11
B63	B197	WBRSR(mPas_200)	AG	0.03115	1.35E-11
B63	B136	WBRSR(mPas_200)	5'NT	0.03109	1.48E-11
B63	B70	WBRSR(mPas_200)	LDL-C	0.031078	1.51E-11
B63	B100	WBRSR(mPas_200)	anti-Ro52	-0.03033	4.54E-11
B63	B209	WBRSR(mPas_200)	Smoking	0.029703	1.13E-10

B63	B130	WBRSR(mPas_200)	GLB	0.028191	9.35E-10
B63	B153	WBRSR(mPas_200)	Glu	0.028006	1.20E-09
B63	B77	WBRSR(mPas_200)	TC	0.027264	3.24E-09
B63	B186	WBRSR(mPas_200)	PFC	0.027027	4.43E-09
B63	B170	WBRSR(mPas_200)	U-GLU	0.026994	4.62E-09
B63	B122	WBRSR(mPas_200)	C4	-0.02682	5.82E-09
B63	B179	WBRSR(mPas_200)	α1-MG	0.026387	1.01E-08
B63	B207	WBRSR(mPas_200)	WHR	0.025816	2.09E-08
B63	B148	WBRSR(mPas_200)	Anti_TPO	-0.0252	4.49E-08
B63	B166	WBRSR(mPas_200)	Pro	0.024857	6.81E-08
B63	B200	WBRSR(mPas_200)	OP	-0.02473	7.96E-08
B63	B112	WBRSR(mPas_200)	FT4	0.024637	8.88E-08
B63	B198	WBRSR(mPas_200)	P	-0.02448	1.07E-07
B63	B73	WBRSR(mPas_200)	ApoA	-0.02332	4.13E-07
B63	B161	WBRSR(mPas_200)	LEU	-0.02312	5.18E-07
B63	B131	WBRSR(mPas_200)	AST	0.023096	5.34E-07
B64	B113	WBRSR(mPas_5)	FT3	0.056368	0
B64	B115	WBRSR(mPas_5)	TSH TT3 TT4(TT3)	0.076297	0
B64	B121	WBRSR(mPas_5)	C3	-0.05717	0
B64	B123	WBRSR(mPas_5)	hs-CRP1	0.04101	0
B64	B125	WBRSR(mPas_5)	Cor	0.050069	0
B64	B126	WBRSR(mPas_5)	Alb	0.050453	0
B64	B128	WBRSR(mPas_5)	ALT	0.043667	0
B64	B129	WBRSR(mPas_5)	IBIL	0.048805	0
B64	B133	WBRSR(mPas_5)	TBIL	0.04599	0
B64	B134	WBRSR(mPas_5)	CHE	0.09055	0
B64	B138	WBRSR(mPas_5)	PAB	0.063768	0
B64	B140	WBRSR(mPas_5)	r-GT	0.04965	0
B64	B141	WBRSR(mPas_5)	AST/ALT	-0.04113	0
B64	B163	WBRSR(mPas_5)	SG	0.065161	0
B64	B174	WBRSR(mPas_5)	Urea/Cr	-0.05526	0
B64	B176	WBRSR(mPas_5)	Ucr	0.05106	0
B64	B188	WBRSR(mPas_5)	Ca	0.041777	0
B64	B192	WBRSR(mPas_5)	UA	0.060626	0
B64	B194	WBRSR(mPas_5)	Cl	-0.05975	0
B64	B206	WBRSR(mPas_5)	Waist	0.066278	0
B64	B210	WBRSR(mPas_5)	Hip	0.053736	0
B64	B215	WBRSR(mPas_5)	BMI	0.045232	0
B64	B216	WBRSR(mPas_5)	BMI	0.078549	0
B64	B218	WBRSR(mPas_5)	DBP	0.045073	0
B64	B221	WBRSR(mPas_5)	Hei	0.073911	0
B64	B222	WBRSR(mPas_5)	NS	0.082499	0
B64	B65	WBRSR(mPas_5)	WBRSR(mPas_1)	0.976519	0
B64	B66	WBRSR(mPas_5)	WBRSR(mPas_100)	0.923723	0

B64	B67	WBRSR(mPas_5)	WBRSR(mPas_50)	0.941551	0
B64	B69	WBRSR(mPas_5)	PV	0.166934	0
B64	B74	WBRSR(mPas_5)	ApoA1	-0.05878	0
B64	B75	WBRSR(mPas_5)	ApoA1/B	-0.06881	0
B64	B76	WBRSR(mPas_5)	ApoB	0.070256	0
B64	B91	WBRSR(mPas_5)	TP	0.044145	0
B64	B122	WBRSR(mPas_5)	C4	-0.03785	2.22E-16
B64	B204	WBRSR(mPas_5)	Drinking	0.035522	1.24E-14
B64	B71	WBRSR(mPas_5)	TG	0.035104	2.49E-14
B64	B209	WBRSR(mPas_5)	Smoking	0.034841	3.91E-14
B64	B124	WBRSR(mPas_5)	ACTH	0.034382	8.35E-14
B64	B137	WBRSR(mPas_5)	AFU	0.033197	5.70E-13
B64	B112	WBRSR(mPas_5)	FT4	0.031769	5.30E-12
B64	B70	WBRSR(mPas_5)	LDL-C	0.031533	7.59E-12
B64	B72	WBRSR(mPas_5)	HDL-C	-0.03127	1.14E-11
B64	B198	WBRSR(mPas_5)	P	-0.0298	9.87E-11
B64	B135	WBRSR(mPas_5)	TBA	0.028384	7.19E-10
B64	B100	WBRSR(mPas_5)	anti-Ro52	-0.02753	2.27E-09
B64	B187	WBRSR(mPas_5)	NSE	0.026597	7.75E-09
B64	B148	WBRSR(mPas_5)	Anti_TPO	-0.02658	7.96E-09
B64	B73	WBRSR(mPas_5)	ApoA	-0.02642	9.72E-09
B64	B197	WBRSR(mPas_5)	AG	0.025996	1.67E-08
B64	B79	WBRSR(mPas_5)	hb V -dna	-0.0257	2.42E-08
B64	B189	WBRSR(mPas_5)	eGFB	0.025533	2.97E-08
B64	B120	WBRSR(mPas_5)	HCV-cAg	0.02552	3.02E-08
B64	B179	WBRSR(mPas_5)	α1-MG	0.025399	3.51E-08
B64	B132	WBRSR(mPas_5)	Dbil	0.025248	4.23E-08
B64	B170	WBRSR(mPas_5)	U-GLU	0.025051	5.38E-08
B64	B149	WBRSR(mPas_5)	TGAb	-0.0248	7.25E-08
B64	B131	WBRSR(mPas_5)	AST	0.024691	8.32E-08
B64	B153	WBRSR(mPas_5)	Glu	0.023709	2.65E-07
B64	B166	WBRSR(mPas_5)	Pro	0.023694	2.70E-07
B64	B111	WBRSR(mPas_5)	TSH	-0.02343	3.65E-07
B65	B113	WBRSR(mPas_1)	FT3	0.052903	0
B65	B115	WBRSR(mPas_1)	TSH TT3 TT4(TT3)	0.068335	0
B65	B121	WBRSR(mPas_1)	C3	-0.06067	0
B65	B122	WBRSR(mPas_1)	C4	-0.04203	0
B65	B125	WBRSR(mPas_1)	Cor	0.046892	0
B65	B126	WBRSR(mPas_1)	Alb	0.047416	0
B65	B128	WBRSR(mPas_1)	ALT	0.039509	0
B65	B129	WBRSR(mPas_1)	IBIL	0.045272	0
B65	B133	WBRSR(mPas_1)	TBIL	0.042214	0
B65	B134	WBRSR(mPas_1)	CHE	0.078956	0
B65	B138	WBRSR(mPas_1)	PAB	0.058028	0

B65	B140	WBRSR(mPas_1)	r-GT	0.046461	0
B65	B163	WBRSR(mPas_1)	SG	0.063406	0
B65	B174	WBRSR(mPas_1)	Urea/Cr	-0.05092	0
B65	B176	WBRSR(mPas_1)	Ucr	0.048437	0
B65	B192	WBRSR(mPas_1)	UA	0.055621	0
B65	B194	WBRSR(mPas_1)	Cl	-0.0502	0
B65	B206	WBRSR(mPas_1)	Waist	0.060982	0
B65	B210	WBRSR(mPas_1)	Hip	0.050749	0
B65	B215	WBRSR(mPas_1)	BMI	0.039817	0
B65	B216	WBRSR(mPas_1)	BMI	0.072022	0
B65	B218	WBRSR(mPas_1)	DBP	0.041465	0
B65	B221	WBRSR(mPas_1)	Hei	0.069656	0
B65	B222	WBRSR(mPas_1)	NS	0.077329	0
B65	B66	WBRSR(mPas_1)	WBRSR(mPas_100)	0.839557	0
B65	B67	WBRSR(mPas_1)	WBRSR(mPas_50)	0.867929	0
B65	B69	WBRSR(mPas_1)	PV	0.14849	0
B65	B74	WBRSR(mPas_1)	ApoA1	-0.04734	0
B65	B75	WBRSR(mPas_1)	ApoA1/B	-0.05251	0
B65	B76	WBRSR(mPas_1)	ApoB	0.053361	0
B65	B188	WBRSR(mPas_1)	Ca	0.037542	4.44E-16
B65	B209	WBRSR(mPas_1)	Smoking	0.036026	5.33E-15
B65	B141	WBRSR(mPas_1)	AST/ALT	-0.03544	1.42E-14
B65	B91	WBRSR(mPas_1)	TP	0.03516	2.26E-14
B65	B123	WBRSR(mPas_1)	hs-CRP1	0.035141	2.35E-14
B65	B204	WBRSR(mPas_1)	Drinking	0.034473	7.19E-14
B65	B112	WBRSR(mPas_1)	FT4	0.033078	6.89E-13
B65	B135	WBRSR(mPas_1)	TBA	0.032539	1.61E-12
B65	B124	WBRSR(mPas_1)	ACTH	0.032408	1.98E-12
B65	B70	WBRSR(mPas_1)	LDL-C	0.02999	7.47E-11
B65	B198	WBRSR(mPas_1)	P	-0.0299	8.51E-11
B65	B189	WBRSR(mPas_1)	eGFB	0.029038	2.90E-10
B65	B137	WBRSR(mPas_1)	AFU	0.027212	3.47E-09
B65	B81	WBRSR(mPas_1)	TSGF	-0.02676	6.27E-09
B65	B71	WBRSR(mPas_1)	TG	0.026303	1.13E-08
B65	B111	WBRSR(mPas_1)	TSH	-0.02582	2.07E-08
B65	B148	WBRSR(mPas_1)	Anti_TPO	-0.02567	2.50E-08
B65	B79	WBRSR(mPas_1)	hb V -dna	-0.02538	3.59E-08
B65	B73	WBRSR(mPas_1)	ApoA	-0.02518	4.62E-08
B65	B149	WBRSR(mPas_1)	TGAb	-0.02466	8.61E-08
B65	B92	WBRSR(mPas_1)	ALDH2-Glu504Glu	0.024135	1.61E-07
B65	B131	WBRSR(mPas_1)	AST	0.024117	1.65E-07
B65	B100	WBRSR(mPas_1)	anti-Ro52	-0.0237	2.68E-07
B65	B170	WBRSR(mPas_1)	U-GLU	0.023418	3.70E-07
B65	B72	WBRSR(mPas_1)	HDL-C	-0.02296	6.23E-07

B65	B93	WBRSR(mPas_1)	ALDH2-Glu504Lys	-0.02273	8.01E-07
B66	B113	WBRSR(mPas_100)	FT3	0.055542	0
B66	B115	WBRSR(mPas_100)	TSH TT3 TT4(TT3)	0.079475	0
B66	B121	WBRSR(mPas_100)	C3	-0.04616	0
B66	B123	WBRSR(mPas_100)	hs-CRP1	0.048567	0
B66	B126	WBRSR(mPas_100)	Alb	0.048198	0
B66	B128	WBRSR(mPas_100)	ALT	0.045715	0
B66	B129	WBRSR(mPas_100)	IBIL	0.049926	0
B66	B133	WBRSR(mPas_100)	TBIL	0.048438	0
B66	B134	WBRSR(mPas_100)	CHE	0.093379	0
B66	B138	WBRSR(mPas_100)	PAB	0.063924	0
B66	B140	WBRSR(mPas_100)	r-GT	0.050725	0
B66	B141	WBRSR(mPas_100)	AST/ALT	-0.04591	0
B66	B163	WBRSR(mPas_100)	SG	0.061768	0
B66	B174	WBRSR(mPas_100)	Urea/Cr	-0.05755	0
B66	B176	WBRSR(mPas_100)	Ucr	0.052232	0
B66	B188	WBRSR(mPas_100)	Ca	0.045042	0
B66	B192	WBRSR(mPas_100)	UA	0.064495	0
B66	B194	WBRSR(mPas_100)	Cl	-0.06489	0
B66	B206	WBRSR(mPas_100)	Waist	0.069277	0
B66	B210	WBRSR(mPas_100)	Hip	0.054859	0
B66	B215	WBRSR(mPas_100)	BMI	0.051056	0
B66	B216	WBRSR(mPas_100)	BMI	0.082359	0
B66	B218	WBRSR(mPas_100)	DBP	0.048316	0
B66	B221	WBRSR(mPas_100)	Hei	0.073435	0
B66	B222	WBRSR(mPas_100)	NS	0.085024	0
B66	B67	WBRSR(mPas_100)	WBRSR(mPas_50)	0.997581	0
B66	B69	WBRSR(mPas_100)	PV	0.167861	0
B66	B71	WBRSR(mPas_100)	TG	0.044246	0
B66	B72	WBRSR(mPas_100)	HDL-C	-0.04351	0
B66	B74	WBRSR(mPas_100)	ApoA1	-0.07806	0
B66	B75	WBRSR(mPas_100)	ApoA1/B	-0.09123	0
B66	B76	WBRSR(mPas_100)	ApoB	0.09386	0
B66	B91	WBRSR(mPas_100)	TP	0.054815	0
B66	B137	WBRSR(mPas_100)	AFU	0.034199	1.13E-13
B66	B125	WBRSR(mPas_100)	Cor	0.033913	1.80E-13
B66	B187	WBRSR(mPas_100)	NSE	0.033794	2.18E-13
B66	B204	WBRSR(mPas_100)	Drinking	0.032465	1.81E-12
B66	B120	WBRSR(mPas_100)	HCV-cAg	0.032331	2.23E-12
B66	B70	WBRSR(mPas_100)	LDL-C	0.03233	2.23E-12
B66	B136	WBRSR(mPas_100)	5'NT	0.031865	4.58E-12
B66	B186	WBRSR(mPas_100)	PFC	0.031802	5.04E-12
B66	B79	WBRSR(mPas_100)	hb V -dna	-0.03166	6.24E-12
B66	B197	WBRSR(mPas_100)	AG	0.031429	8.90E-12

B66	B132	WBRSR(mPas_100)	Dbil	0.030869	2.06E-11
B66	B124	WBRSR(mPas_100)	ACTH	0.030428	3.95E-11
B66	B198	WBRSR(mPas_100)	P	-0.02981	9.70E-11
B66	B209	WBRSR(mPas_100)	Smoking	0.029513	1.48E-10
B66	B122	WBRSR(mPas_100)	C4	-0.02933	1.92E-10
B66	B100	WBRSR(mPas_100)	anti-Ro52	-0.02922	2.25E-10
B66	B130	WBRSR(mPas_100)	GLB	0.028902	3.51E-10
B66	B179	WBRSR(mPas_100)	α1-MG	0.028239	8.76E-10
B66	B170	WBRSR(mPas_100)	U-GLU	0.027646	1.95E-09
B66	B207	WBRSR(mPas_100)	WHR	0.026832	5.72E-09
B66	B153	WBRSR(mPas_100)	Glu	0.026758	6.29E-09
B66	B77	WBRSR(mPas_100)	TC	0.026739	6.44E-09
B66	B148	WBRSR(mPas_100)	Anti_TPO	-0.02544	3.35E-08
B66	B166	WBRSR(mPas_100)	Pro	0.024352	1.25E-07
B66	B73	WBRSR(mPas_100)	ApoA	-0.02422	1.46E-07
B66	B131	WBRSR(mPas_100)	AST	0.023475	3.47E-07
B66	B200	WBRSR(mPas_100)	OP	-0.02308	5.42E-07
B67	B113	WBRSR(mPas_50)	FT3	0.056433	0
B67	B115	WBRSR(mPas_50)	TSH TT3 TT4(TT3)	0.079669	0
B67	B121	WBRSR(mPas_50)	C3	-0.04896	0
B67	B123	WBRSR(mPas_50)	hs-CRP1	0.048033	0
B67	B126	WBRSR(mPas_50)	Alb	0.048916	0
B67	B128	WBRSR(mPas_50)	ALT	0.045623	0
B67	B129	WBRSR(mPas_50)	IBIL	0.049766	0
B67	B133	WBRSR(mPas_50)	TBIL	0.048153	0
B67	B134	WBRSR(mPas_50)	CHE	0.092771	0
B67	B138	WBRSR(mPas_50)	PAB	0.06413	0
B67	B140	WBRSR(mPas_50)	r-GT	0.050686	0
B67	B141	WBRSR(mPas_50)	AST/ALT	-0.04514	0
B67	B163	WBRSR(mPas_50)	SG	0.062689	0
B67	B174	WBRSR(mPas_50)	Urea/Cr	-0.05774	0
B67	B176	WBRSR(mPas_50)	Ucr	0.052598	0
B67	B188	WBRSR(mPas_50)	Ca	0.044926	0
B67	B192	WBRSR(mPas_50)	UA	0.064208	0
B67	B194	WBRSR(mPas_50)	Cl	-0.06366	0
B67	B206	WBRSR(mPas_50)	Waist	0.069321	0
B67	B210	WBRSR(mPas_50)	Hip	0.05503	0
B67	B215	WBRSR(mPas_50)	BMI	0.050357	0
B67	B216	WBRSR(mPas_50)	BMI	0.082229	0
B67	B218	WBRSR(mPas_50)	DBP	0.04837	0
B67	B221	WBRSR(mPas_50)	Hei	0.074038	0
B67	B222	WBRSR(mPas_50)	NS	0.085304	0
B67	B69	WBRSR(mPas_50)	PV	0.167044	0
B67	B71	WBRSR(mPas_50)	TG	0.042534	0

B67	B72	WBRSR(mPas_50)	HDL-C	-0.04175	0
B67	B74	WBRSR(mPas_50)	ApoA1	-0.07585	0
B67	B75	WBRSR(mPas_50)	ApoA1/B	-0.08866	0
B67	B76	WBRSR(mPas_50)	ApoB	0.090897	0
B67	B91	WBRSR(mPas_50)	TP	0.053772	0
B67	B125	WBRSR(mPas_50)	Cor	0.034612	5.68E-14
B67	B137	WBRSR(mPas_50)	AFU	0.033648	2.76E-13
B67	B204	WBRSR(mPas_50)	Drinking	0.03309	6.75E-13
B67	B187	WBRSR(mPas_50)	NSE	0.033029	7.45E-13
B67	B70	WBRSR(mPas_50)	LDL-C	0.032633	1.39E-12
B67	B79	WBRSR(mPas_50)	hb V -dna	-0.03207	3.36E-12
B67	B122	WBRSR(mPas_50)	C4	-0.03158	7.05E-12
B67	B120	WBRSR(mPas_50)	HCV-cAg	0.031449	8.62E-12
B67	B124	WBRSR(mPas_50)	ACTH	0.030974	1.76E-11
B67	B197	WBRSR(mPas_50)	AG	0.030885	2.01E-11
B67	B136	WBRSR(mPas_50)	5'NT	0.030777	2.36E-11
B67	B209	WBRSR(mPas_50)	Smoking	0.03068	2.72E-11
B67	B198	WBRSR(mPas_50)	P	-0.03059	3.12E-11
B67	B132	WBRSR(mPas_50)	Dbil	0.030313	4.67E-11
B67	B186	WBRSR(mPas_50)	PFC	0.029844	9.23E-11
B67	B100	WBRSR(mPas_50)	anti-Ro52	-0.02873	4.43E-10
B67	B179	WBRSR(mPas_50)	α1-MG	0.028011	1.19E-09
B67	B170	WBRSR(mPas_50)	U-GLU	0.027737	1.73E-09
B67	B130	WBRSR(mPas_50)	GLB	0.027278	3.19E-09
B67	B153	WBRSR(mPas_50)	Glu	0.026421	9.71E-09
B67	B207	WBRSR(mPas_50)	WHR	0.026403	9.93E-09
B67	B77	WBRSR(mPas_50)	TC	0.025874	1.94E-08
B67	B148	WBRSR(mPas_50)	Anti_TPO	-0.02572	2.35E-08
B67	B73	WBRSR(mPas_50)	ApoA	-0.02486	6.80E-08
B67	B166	WBRSR(mPas_50)	Pro	0.024832	7.02E-08
B67	B131	WBRSR(mPas_50)	AST	0.023894	2.14E-07
B67	B112	WBRSR(mPas_50)	FT4	0.023217	4.66E-07
B67	B149	WBRSR(mPas_50)	TGAb	-0.023	5.92E-07
B67	B200	WBRSR(mPas_50)	OP	-0.02267	8.57E-07
B69	B107	PV	AnuA	-0.10305	0
B69	B109	PV	AMA-M2	0.038571	0
B69	B112	PV	FT4	0.042755	0
B69	B114	PV	TSH TT3 TT4(TT4)	0.067339	0
B69	B115	PV	TSH TT3 TT4(TT3)	0.047586	0
B69	B123	PV	hs-CRP1	0.113282	0
B69	B125	PV	Cor	0.053793	0
B69	B127	PV	A-G ratio	-0.09019	0
B69	B130	PV	GLB	0.124137	0
B69	B134	PV	CHE	0.039872	0

B69	B136	PV	5'NT	0.058947	0
B69	B155	PV	IgA	0.072741	0
B69	B157	PV	IgG	0.105354	0
B69	B186	PV	PFC	0.089862	0
B69	B188	PV	Ca	0.051833	0
B69	B194	PV	Cl	-0.04841	0
B69	B70	PV	LDL-C	0.06493	0
B69	B71	PV	TG	0.042896	0
B69	B73	PV	ApoA	0.049306	0
B69	B74	PV	ApoA1	0.065902	0
B69	B75	PV	ApoA1/B	-0.03842	0
B69	B76	PV	ApoB	0.118015	0
B69	B77	PV	TC	0.073771	0
B69	B91	PV	TP	0.132609	0
B69	B139	PV	ADA	0.037466	4.44E-16
B69	B97	PV	dsDNA	0.036814	1.33E-15
B69	B153	PV	Glu	0.034203	1.12E-13
B69	B126	PV	Alb	0.034169	1.18E-13
B69	B158	PV	IgM	0.033556	3.21E-13
B69	B111	PV	TSH	0.033195	5.72E-13
B69	B183	PV	PTA	0.031594	6.92E-12
B69	B193	PV	K	0.030433	3.92E-11
B69	B221	PV	Hei	-0.02933	1.91E-10
B69	B95	PV	anti-cenpb	0.028662	4.90E-10
B69	B150	PV	ALP	0.027944	1.31E-09
B69	B120	PV	HCV-cAg	0.027629	2.00E-09
B69	B195	PV	Na	-0.02722	3.46E-09
B69	B199	PV	Mg	0.026725	6.56E-09
B69	B140	PV	r-GT	0.025129	4.89E-08
B69	B81	PV	TSGF	-0.02423	1.45E-07
B69	B131	PV	AST	0.023625	2.92E-07
B69	B196	PV	TCO2	0.023452	3.56E-07
B7	B113	WB-Cop	FT3	0.053273	0
B7	B8	WB-Cop	WB-Zinc	0.296204	0
B7	B23	WB-Cop	CD3-CD16+CD56+(NK)	-0.03533	1.73E-14
B7	B100	WB-Cop	anti-Ro52	-0.03517	2.24E-14
B7	B21	WB-Cop	CD3+CD4-CD8-	-0.02696	4.84E-09
B7	B107	WB-Cop	AnuA	-0.02617	1.33E-08
B7	B115	WB-Cop	TSH TT3 TT4(TT3)	0.024463	1.09E-07
B7	B17	WB-Cop	CD3+CD16+CD56+NK	0.023483	3.44E-07
B7	B195	WB-Cop	Na	-0.02283	7.24E-07
B70	B126	LDL-C	Alb	0.073653	0
B70	B127	LDL-C	A-G ratio	-0.08118	0
B70	B129	LDL-C	IBIL	-0.03881	0

B70	B130	LDL-C	GLB	0.108905	0
B70	B132	LDL-C	Dbil	-0.20475	0
B70	B133	LDL-C	TBIL	-0.08651	0
B70	B134	LDL-C	CHE	0.048435	0
B70	B188	LDL-C	Ca	0.105638	0
B70	B189	LDL-C	eGFB	0.043021	0
B70	B218	LDL-C	DBP	0.071081	0
B70	B220	LDL-C	SBP	0.067659	0
B70	B221	LDL-C	Hei	-0.06535	0
B70	B71	LDL-C	TG	-0.06821	0
B70	B72	LDL-C	HDL-C	0.123035	0
B70	B73	LDL-C	ApoA	0.068285	0
B70	B74	LDL-C	ApoA1	0.055146	0
B70	B75	LDL-C	ApoA1/B	-0.20894	0
B70	B76	LDL-C	ApoB	0.327584	0
B70	B77	LDL-C	TC	0.832036	0
B70	B91	LDL-C	TP	0.142424	0
B70	B111	LDL-C	TSH	0.03542	1.47E-14
B70	B194	LDL-C	Cl	-0.0351	2.51E-14
B70	B174	LDL-C	Urea/Cr	0.03456	6.22E-14
B70	B222	LDL-C	NS	-0.03233	2.22E-12
B70	B131	LDL-C	AST	-0.0306	3.08E-11
B70	B136	LDL-C	5'NT	0.029966	7.74E-11
B70	B153	LDL-C	Glu	0.029007	3.03E-10
B70	B161	LDL-C	LEU	0.028429	6.75E-10
B70	B190	LDL-C	Cre	-0.02814	1.00E-09
B70	B215	LDL-C	BMI	0.027761	1.67E-09
B70	B119	LDL-C	Hcv-rna	-0.0267	6.79E-09
B70	B150	LDL-C	ALP	0.025372	3.63E-08
B70	B146	LDL-C	CysC	-0.02393	2.05E-07
B70	B86	LDL-C	TBhCG	0.023693	2.70E-07
B70	B181	LDL-C	APTT	-0.02362	2.93E-07
B71	B126	TG	Alb	0.150119	0
B71	B127	TG	A-G ratio	-0.10828	0
B71	B128	TG	ALT	0.155299	0
B71	B129	TG	IBIL	-0.05472	0
B71	B130	TG	GLB	0.213029	0
B71	B131	TG	AST	0.094286	0
B71	B132	TG	Dbil	-0.11575	0
B71	B133	TG	TBIL	-0.07534	0
B71	B134	TG	CHE	0.079428	0
B71	B136	TG	5'NT	0.038953	0
B71	B138	TG	PAB	0.068414	0
B71	B140	TG	r-GT	0.206844	0

B71	B141	TG	AST/ALT	-0.14344	0
B71	B153	TG	Glu	0.189924	0
B71	B160	TG	PH	-0.06649	0
B71	B163	TG	SG	0.07648	0
B71	B166	TG	Pro	0.049499	0
B71	B170	TG	U-GLU	0.1043	0
B71	B174	TG	Urea/Cr	-0.05522	0
B71	B188	TG	Ca	0.089575	0
B71	B189	TG	eGFB	0.080301	0
B71	B192	TG	UA	0.213864	0
B71	B203	TG	DH	0.066315	0
B71	B204	TG	Drinking	0.112054	0
B71	B206	TG	Waist	0.156569	0
B71	B209	TG	Smoking	0.125514	0
B71	B210	TG	Hip	0.108123	0
B71	B211	TG	IOP-R	0.040032	0
B71	B212	TG	IOP-L	0.04086	0
B71	B215	TG	BMI	0.161435	0
B71	B216	TG	BMI	0.18333	0
B71	B218	TG	DBP	0.118913	0
B71	B221	TG	Hei	0.107883	0
B71	B222	TG	NS	0.162612	0
B71	B72	TG	HDL-C	-0.38096	0
B71	B73	TG	ApoA	-0.04001	0
B71	B74	TG	ApoA1	-0.05905	0
B71	B77	TG	TC	0.278171	0
B71	B91	TG	TP	0.282048	0
B71	B176	TG	Ucr	0.034685	5.04E-14
B71	B219	TG	OT	-0.03433	9.08E-14
B71	B194	TG	Cl	-0.03381	2.14E-13
B71	B191	TG	Urea	-0.03153	7.68E-12
B71	B112	TG	FT4	-0.03078	2.33E-11
B71	B114	TG	TSH TT3 TT4(TT4)	-0.03072	2.57E-11
B71	B109	TG	AMA-M2	0.030415	4.03E-11
B71	B76	TG	ApoB	0.028084	1.08E-09
B71	B150	TG	ALP	0.027901	1.39E-09
B71	B86	TG	TBhCG	-0.02636	1.05E-08
B71	B201	TG	e-habits	-0.02625	1.21E-08
B71	B135	TG	TBA	0.025571	2.84E-08
B71	B157	TG	IgG	-0.0253	3.97E-08
B71	B137	TG	AFU	0.024111	1.66E-07
B71	B193	TG	K	-0.02388	2.17E-07
B72	B112	HDL-C	FT4	0.061734	0
B72	B127	HDL-C	A-G ratio	0.05612	0

B72	B128	HDL-C	ALT	-0.16935	0
B72	B130	HDL-C	GLB	-0.06923	0
B72	B132	HDL-C	Dbil	0.061629	0
B72	B133	HDL-C	TBIL	0.040983	0
B72	B134	HDL-C	CHE	-0.05291	0
B72	B140	HDL-C	r-GT	-0.06139	0
B72	B141	HDL-C	AST/ALT	0.266028	0
B72	B153	HDL-C	Glu	-0.11058	0
B72	B160	HDL-C	PH	0.069072	0
B72	B161	HDL-C	LEU	0.055718	0
B72	B163	HDL-C	SG	-0.17009	0
B72	B166	HDL-C	Pro	-0.07222	0
B72	B170	HDL-C	U-GLU	-0.05759	0
B72	B174	HDL-C	Urea/Cr	0.166279	0
B72	B176	HDL-C	Ucr	-0.07209	0
B72	B188	HDL-C	Ca	0.069442	0
B72	B190	HDL-C	Cre	-0.12094	0
B72	B192	HDL-C	UA	-0.29621	0
B72	B203	HDL-C	DH	-0.04514	0
B72	B204	HDL-C	Drinking	-0.06794	0
B72	B206	HDL-C	Waist	-0.32944	0
B72	B209	HDL-C	Smoking	-0.14148	0
B72	B210	HDL-C	Hip	-0.2478	0
B72	B215	HDL-C	BMI	-0.29403	0
B72	B216	HDL-C	BMI	-0.38448	0
B72	B218	HDL-C	DBP	-0.08424	0
B72	B219	HDL-C	OT	0.048585	0
B72	B220	HDL-C	SBP	0.071989	0
B72	B221	HDL-C	Hei	-0.2918	0
B72	B222	HDL-C	NS	-0.34696	0
B72	B73	HDL-C	ApoA	0.047937	0
B72	B74	HDL-C	ApoA1	0.295675	0
B72	B75	HDL-C	ApoA1/B	0.139488	0
B72	B77	HDL-C	TC	0.253632	0
B72	B91	HDL-C	TP	-0.05465	0
B72	B113	HDL-C	FT3	-0.0353	1.82E-14
B72	B81	HDL-C	TSGF	-0.03486	3.75E-14
B72	B115	HDL-C	TSH TT3 TT4(TT3)	-0.03454	6.39E-14
B72	B173	HDL-C	NTT	0.032614	1.43E-12
B72	B129	HDL-C	IBIL	0.030291	4.82E-11
B72	B205	HDL-C	KDA	0.029048	2.86E-10
B72	B193	HDL-C	K	0.028376	7.26E-10
B72	B198	HDL-C	P	0.028283	8.25E-10
B72	B114	HDL-C	TSH TT3 TT4(TT4)	0.027966	1.27E-09

B72	B189	HDL-C	eGFB	0.027176	3.64E-09
B72	B149	HDL-C	TGAb	0.026844	5.63E-09
B72	B123	HDL-C	hs-CRP1	-0.02524	4.30E-08
B72	B179	HDL-C	α1-MG	-0.02305	5.59E-07
B72	B224	HDL-C	pmh	0.022967	6.18E-07
B73	B123	ApoA	hs-CRP1	0.064069	0
B73	B137	ApoA	AFU	-0.04156	0
B73	B193	ApoA	K	0.047775	0
B73	B74	ApoA	ApoA1	0.092623	0
B73	B75	ApoA	ApoA1/B	-0.10362	0
B73	B76	ApoA	ApoB	0.208747	0
B73	B77	ApoA	TC	0.054293	0
B73	B222	ApoA	NS	-0.03775	2.22E-16
B73	B216	ApoA	BMI	-0.03519	2.15E-14
B73	B206	ApoA	Waist	-0.02793	1.34E-09
B73	B127	ApoA	A-G ratio	-0.02758	2.13E-09
B73	B128	ApoA	ALT	-0.02715	3.75E-09
B73	B140	ApoA	r-GT	-0.02661	7.66E-09
B73	B126	ApoA	Alb	-0.02624	1.22E-08
B73	B215	ApoA	BMI	-0.02618	1.32E-08
B73	B204	ApoA	Drinking	-0.02548	3.17E-08
B73	B163	ApoA	SG	-0.02533	3.82E-08
B73	B135	ApoA	TBA	-0.02522	4.38E-08
B73	B221	ApoA	Hei	-0.0248	7.26E-08
B73	B194	ApoA	Cl	0.023694	2.70E-07
B73	B131	ApoA	AST	-0.02355	3.18E-07
B73	B198	ApoA	P	0.023486	3.43E-07
B74	B123	ApoA1	hs-CRP1	-0.05125	0
B74	B126	ApoA1	Alb	0.046823	0
B74	B128	ApoA1	ALT	-0.04487	0
B74	B134	ApoA1	CHE	-0.05716	0
B74	B141	ApoA1	AST/ALT	0.076663	0
B74	B163	ApoA1	SG	-0.0609	0
B74	B174	ApoA1	Urea/Cr	0.075867	0
B74	B176	ApoA1	Ucr	-0.06838	0
B74	B188	ApoA1	Ca	0.051087	0
B74	B190	ApoA1	Cre	-0.04225	0
B74	B192	ApoA1	UA	-0.07795	0
B74	B193	ApoA1	K	0.06652	0
B74	B194	ApoA1	Cl	-0.06757	0
B74	B198	ApoA1	P	0.096406	0
B74	B199	ApoA1	Mg	0.046666	0
B74	B206	ApoA1	Waist	-0.10394	0
B74	B209	ApoA1	Smoking	-0.04832	0

B74	B210	ApoA1	Hip	-0.08062	0
B74	B215	ApoA1	BMI	-0.08402	0
B74	B216	ApoA1	BMI	-0.12861	0
B74	B220	ApoA1	SBP	0.050341	0
B74	B221	ApoA1	Hei	-0.11156	0
B74	B222	ApoA1	NS	-0.12788	0
B74	B75	ApoA1	ApoA1/B	0.437847	0
B74	B77	ApoA1	TC	0.115457	0
B74	B86	ApoA1	TBhCG	0.062068	0
B74	B91	ApoA1	TP	0.033537	3.30E-13
B74	B138	ApoA1	PAB	0.033471	3.68E-13
B74	B114	ApoA1	TSH TT3 TT4(TT4)	0.033203	5.65E-13
B74	B181	ApoA1	APTT	-0.03242	1.94E-12
B74	B76	ApoA1	ApoB	0.030574	3.19E-11
B74	B81	ApoA1	TSGF	-0.02734	2.94E-09
B74	B161	ApoA1	LEU	0.027196	3.55E-09
B74	B156	ApoA1	IgE	0.0266	7.72E-09
B74	B197	ApoA1	AG	0.024398	1.18E-07
B74	B179	ApoA1	α1-MG	-0.02423	1.44E-07
B74	B121	ApoA1	C3	0.023857	2.23E-07
B74	B204	ApoA1	Drinking	-0.02344	3.59E-07
B74	B92	ApoA1	ALDH2-Glu504Glu	0.023147	5.04E-07
B75	B123	ApoA1/B	hs-CRP1	-0.05699	0
B75	B132	ApoA1/B	Dbil	0.050082	0
B75	B134	ApoA1/B	CHE	-0.13173	0
B75	B136	ApoA1/B	5'NT	-0.06172	0
B75	B137	ApoA1/B	AFU	-0.03965	0
B75	B138	ApoA1/B	PAB	-0.07594	0
B75	B141	ApoA1/B	AST/ALT	0.053494	0
B75	B192	ApoA1/B	UA	-0.05347	0
B75	B195	ApoA1/B	Na	-0.04498	0
B75	B200	ApoA1/B	OP	-0.04001	0
B75	B206	ApoA1/B	Waist	-0.06738	0
B75	B210	ApoA1/B	Hip	-0.05018	0
B75	B215	ApoA1/B	BMI	-0.06328	0
B75	B216	ApoA1/B	BMI	-0.07063	0
B75	B221	ApoA1/B	Hei	-0.04213	0
B75	B222	ApoA1/B	NS	-0.06589	0
B75	B76	ApoA1/B	ApoB	-0.70084	0
B75	B77	ApoA1/B	TC	-0.1457	0
B75	B218	ApoA1/B	DBP	-0.03583	7.33E-15
B75	B119	ApoA1/B	Hcv-rna	0.035441	1.42E-14
B75	B79	ApoA1/B	hb V -dna	0.033157	6.08E-13
B75	B163	ApoA1/B	SG	-0.0328	1.07E-12

B75	B176	ApoA1/B	Ucr	-0.03274	1.18E-12
B75	B153	ApoA1/B	Glu	-0.03128	1.11E-11
B75	B193	ApoA1/B	K	0.030829	2.18E-11
B75	B150	ApoA1/B	ALP	-0.03007	6.64E-11
B75	B128	ApoA1/B	ALT	-0.02976	1.04E-10
B75	B197	ApoA1/B	AG	-0.02949	1.54E-10
B75	B107	ApoA1/B	AnuA	0.027854	1.48E-09
B75	B139	ApoA1/B	ADA	-0.02743	2.60E-09
B75	B86	ApoA1/B	TBhCG	0.026261	1.19E-08
B75	B224	ApoA1/B	pmh	0.0256	2.74E-08
B75	B178	ApoA1/B	Ig-U	-0.02545	3.30E-08
B75	B209	ApoA1/B	Smoking	-0.02436	1.24E-07
B75	B180	ApoA1/B	Tf	-0.02335	3.99E-07
B75	B186	ApoA1/B	PFC	-0.0228	7.44E-07
B75	B135	ApoA1/B	TBA	0.022654	8.76E-07
B76	B119	ApoB	Hcv-rna	-0.04339	0
B76	B123	ApoB	hs-CRP1	0.048295	0
B76	B130	ApoB	GLB	0.055502	0
B76	B132	ApoB	Dbil	-0.06514	0
B76	B134	ApoB	CHE	0.169802	0
B76	B136	ApoB	5'NT	0.105269	0
B76	B138	ApoB	PAB	0.107726	0
B76	B153	ApoB	Glu	0.044272	0
B76	B181	ApoB	APTT	-0.04798	0
B76	B186	ApoB	PFC	0.044096	0
B76	B188	ApoB	Ca	0.063994	0
B76	B194	ApoB	Cl	-0.09376	0
B76	B196	ApoB	TCO2	0.039075	0
B76	B197	ApoB	AG	0.051458	0
B76	B199	ApoB	Mg	0.042456	0
B76	B200	ApoB	OP	0.040994	0
B76	B218	ApoB	DBP	0.040375	0
B76	B77	ApoB	TC	0.304537	0
B76	B91	ApoB	TP	0.072128	0
B76	B127	ApoB	A-G ratio	-0.03773	2.22E-16
B76	B180	ApoB	Tf	0.037323	4.44E-16
B76	B126	ApoB	Alb	0.036771	1.33E-15
B76	B140	ApoB	r-GT	0.036426	2.66E-15
B76	B150	ApoB	ALP	0.036201	4.00E-15
B76	B192	ApoB	UA	0.033015	7.62E-13
B76	B184	ApoB	INR	-0.03297	8.16E-13
B76	B215	ApoB	BMI	0.032322	2.26E-12
B76	B79	ApoB	hbV-dna	-0.03098	1.73E-11
B76	B220	ApoB	SBP	0.030641	2.89E-11

B76	B178	ApoB	Ig-U	0.030317	4.65E-11
B76	B206	ApoB	Waist	0.029617	1.28E-10
B76	B170	ApoB	U-GLU	0.029004	3.04E-10
B76	B195	ApoB	Na	0.0278	1.59E-09
B76	B107	ApoB	AnuA	-0.02747	2.48E-09
B76	B135	ApoB	TBA	-0.02609	1.48E-08
B76	B141	ApoB	AST/ALT	-0.02578	2.18E-08
B76	B137	ApoB	AFU	0.025776	2.20E-08
B76	B111	ApoB	TSH	0.023468	3.50E-07
B76	B179	ApoB	α1-MG	0.023151	5.02E-07
B76	B185	ApoB	PT	-0.02294	6.35E-07
B77	B111	TC	TSH	0.046918	0
B77	B126	TC	Alb	0.115746	0
B77	B127	TC	A-G ratio	-0.12217	0
B77	B129	TC	IBIL	-0.05399	0
B77	B130	TC	GLB	0.181722	0
B77	B132	TC	Dbil	-0.22625	0
B77	B133	TC	TBIL	-0.10457	0
B77	B134	TC	CHE	0.060787	0
B77	B136	TC	5'NT	0.04395	0
B77	B138	TC	PAB	0.039462	0
B77	B140	TC	r-GT	0.080469	0
B77	B153	TC	Glu	0.081489	0
B77	B161	TC	LEU	0.039898	0
B77	B163	TC	SG	-0.04834	0
B77	B174	TC	Urea/Cr	0.075207	0
B77	B188	TC	Ca	0.156336	0
B77	B190	TC	Cre	-0.05005	0
B77	B194	TC	Cl	-0.04449	0
B77	B206	TC	Waist	-0.05576	0
B77	B210	TC	Hip	-0.04882	0
B77	B216	TC	BMI	-0.0919	0
B77	B218	TC	DBP	0.079758	0
B77	B220	TC	SBP	0.099327	0
B77	B221	TC	Hei	-0.12497	0
B77	B222	TC	NS	-0.0959	0
B77	B91	TC	TP	0.233301	0
B77	B212	TC	IOP-L	0.036527	2.22E-15
B77	B189	TC	eGFB	0.034415	7.90E-14
B77	B211	TC	IOP-R	0.033107	6.58E-13
B77	B170	TC	U-GLU	0.032359	2.13E-12
B77	B150	TC	ALP	0.02919	2.34E-10
B77	B177	TC	ACR	0.026502	8.75E-09
B77	B181	TC	APTT	-0.0262	1.29E-08

B77	B215	TC	BMI	-0.02448	1.07E-07
B77	B184	TC	INR	-0.02394	2.03E-07
B77	B185	TC	PT	-0.02306	5.55E-07
B78	B80	HBcAb	anti-HP IgG	-0.16027	0
B78	B180	HBcAb	Tf	0.024423	1.15E-07
B79	B100	hb V -dna	anti-Ro52	0.047519	0
B79	B103	hb V -dna	anti-SSA/Ro	0.041094	0
B79	B107	hb V -dna	AnuA	0.286921	0
B79	B119	hb V -dna	Hcv-rna	0.348262	0
B79	B134	hb V -dna	CHE	-0.05967	0
B79	B138	hb V -dna	PAB	-0.06002	0
B79	B150	hb V -dna	ALP	-0.042	0
B79	B157	hb V -dna	IgG	0.038244	0
B79	B176	hb V -dna	Ucr	-0.04365	0
B79	B179	hb V -dna	α1-MG	-0.0508	0
B79	B181	hb V -dna	APTT	0.076941	0
B79	B182	hb V -dna	TT	-0.07047	0
B79	B183	hb V -dna	PTA	0.193192	0
B79	B184	hb V -dna	INR	0.142832	0
B79	B185	hb V -dna	PT	0.048411	0
B79	B186	hb V -dna	PFC	-0.14102	0
B79	B187	hb V -dna	NSE	-0.0387	0
B79	B189	hb V -dna	eGFB	-0.04727	0
B79	B193	hb V -dna	K	0.106669	0
B79	B194	hb V -dna	Cl	0.073341	0
B79	B195	hb V -dna	Na	-0.04419	0
B79	B197	hb V -dna	AG	-0.07344	0
B79	B199	hb V -dna	Mg	-0.04866	0
B79	B204	hb V -dna	Drinking	-0.0383	0
B79	B81	hb V -dna	TSGF	-0.12091	0
B79	B90	hb V -dna	CYFRA 21-1	-0.03818	0
B79	B92	hb V -dna	ALDH2-Glu504Glu	-0.04943	0
B79	B94	hb V -dna	ALDH2-Lys504Lys	0.104349	0
B79	B96	hb V -dna	dsDNA (IIF)	0.073205	0
B79	B98	hb V -dna	anti-Jo-1	0.05121	0
B79	B218	hb V -dna	DBP	-0.03718	6.66E-16
B79	B200	hb V -dna	OP	-0.03695	8.88E-16
B79	B222	hb V -dna	NS	-0.03713	8.88E-16
B79	B137	hb V -dna	AFU	-0.03526	1.91E-14
B79	B216	hb V -dna	BMI	-0.03449	6.93E-14
B79	B109	hb V -dna	AMA-M2	0.031588	6.99E-12
B79	B93	hb V -dna	ALDH2-Glu504Lys	0.031182	1.29E-11
B79	B135	hb V -dna	TBA	0.030896	1.98E-11
B79	B110	hb V -dna	AHA	0.030515	3.47E-11

B79	B206	hb V -dna	Waist	-0.02991	8.44E-11
B79	B97	hb V -dna	dsDNA	0.029202	2.30E-10
B79	B141	hb V -dna	AST/ALT	0.028994	3.08E-10
B79	B113	hb V -dna	FT3	-0.02817	9.60E-10
B79	B105	hb V -dna	anti-U1RNP	-0.02804	1.15E-09
B79	B210	hb V -dna	Hip	-0.02791	1.37E-09
B79	B163	hb V -dna	SG	-0.02721	3.49E-09
B79	B215	hb V -dna	BMI	-0.027	4.60E-09
B79	B128	hb V -dna	ALT	-0.02632	1.10E-08
B79	B95	hb V -dna	anti-cenpb	0.025735	2.31E-08
B79	B99	hb V -dna	anti-PM-Scl	-0.02537	3.64E-08
B79	B198	hb V -dna	P	0.025237	4.29E-08
B79	B178	hb V -dna	Ig-U	-0.02483	7.06E-08
B79	B221	hb V -dna	Hei	-0.02473	7.92E-08
B79	B102	hb V -dna	anti-Sm	-0.02322	4.63E-07
B79	B115	hb V -dna	TSH TT3 TT4(TT3)	-0.02271	8.24E-07
B8	B113	WB-Zinc	FT3	-0.03535	1.64E-14
B8	B23	WB-Zinc	CD3-CD16+CD56+(NK)	-0.03284	1.01E-12
B8	B29	WB-Zinc	E2	-0.03072	2.58E-11
B8	B33	WB-Zinc	Prog	-0.02942	1.68E-10
B8	B18	WB-Zinc	CD3+CD4+helper(inducer)T	0.029036	2.91E-10
B8	B31	WB-Zinc	FSH	0.028594	5.38E-10
B80	B166	anti-HP IgG	Pro	-0.02348	3.46E-07
B81	B124	TSGF	ACTH	-0.04478	0
B81	B125	TSGF	Cor	-0.09872	0
B81	B136	TSGF	5'NT	0.047723	0
B81	B151	TSGF	Anti-CCP	-0.04446	0
B81	B157	TSGF	IgG	-0.05984	0
B81	B181	TSGF	APTT	0.055504	0
B81	B182	TSGF	TT	0.158345	0
B81	B183	TSGF	PTA	-0.0584	0
B81	B184	TSGF	INR	-0.05235	0
B81	B194	TSGF	Cl	-0.03834	0
B81	B195	TSGF	Na	0.050085	0
B81	B197	TSGF	AG	0.058949	0
B81	B200	TSGF	OP	0.055709	0
B81	B92	TSGF	ALDH2-Glu504Glu	0.040004	0
B81	B127	TSGF	A-G ratio	-0.03676	1.33E-15
B81	B93	TSGF	ALDH2-Glu504Lys	-0.03534	1.69E-14
B81	B139	TSGF	ADA	0.035059	2.71E-14
B81	B137	TSGF	AFU	0.034232	1.07E-13
B81	B193	TSGF	K	-0.03399	1.60E-13
B81	B179	TSGF	α1-MG	0.031899	4.34E-12
B81	B108	TSGF	Anti-Cyclin	0.03096	1.80E-11

B81	B189	TSGF	eGFB	-0.03053	3.39E-11
B81	B130	TSGF	GLB	0.029515	1.48E-10
B81	B150	TSGF	ALP	0.029268	2.10E-10
B81	B94	TSGF	ALDH2-Lys504Lys	-0.02901	3.01E-10
B81	B198	TSGF	P	-0.02636	1.05E-08
B81	B112	TSGF	FT4	-0.02614	1.40E-08
B81	B224	TSGF	pmh	-0.02591	1.85E-08
B81	B132	TSGF	Dbil	0.024364	1.23E-07
B81	B134	TSGF	CHE	0.023786	2.42E-07
B81	B95	TSGF	anti-cenpb	-0.02373	2.59E-07
B81	B141	TSGF	AST/ALT	-0.02353	3.26E-07
B81	B110	TSGF	AHA	-0.02255	9.85E-07
B82	B83	CEA	AFP	0.119676	0
B82	B86	CEA	TBhCG	-0.13499	0
B82	B87	CEA	NSE	0.092738	0
B82	B88	CEA	CA 242	0.235054	0
B82	B89	CEA	CA 72-4	0.115828	0
B82	B85	CEA	SCC	0.037765	2.22E-16
B82	B209	CEA	Smoking	0.033378	4.27E-13
B82	B84	CEA	C-PSA	0.030492	3.60E-11
B83	B85	AFP	SCC	0.103167	0
B83	B86	AFP	TBhCG	0.097319	0
B83	B87	AFP	NSE	0.12049	0
B83	B88	AFP	CA 242	0.164298	0
B83	B90	AFP	CYFRA 21-1	0.065555	0
B83	B138	AFP	PAB	0.036445	2.66E-15
B83	B136	AFP	5'NT	0.034126	1.27E-13
B83	B194	AFP	Cl	-0.02465	8.73E-08
B83	B140	AFP	r-GT	0.023599	3.01E-07
B84	B85	C-PSA	SCC	0.051642	0
B84	B86	C-PSA	TBhCG	-0.12859	0
B84	B88	C-PSA	CA 242	0.033652	2.74E-13
B84	B90	C-PSA	CYFRA 21-1	0.024293	1.34E-07
B84	B189	C-PSA	eGFB	-0.02358	3.08E-07
B84	B101	C-PSA	anti-Sc70	0.023571	3.11E-07
B84	B87	C-PSA	NSE	0.022563	9.68E-07
B85	B86	SCC	TBhCG	-0.04038	0
B85	B87	SCC	NSE	0.172992	0
B85	B88	SCC	CA 242	0.058478	0
B85	B90	SCC	CYFRA 21-1	0.055212	0
B85	B189	SCC	eGFB	-0.03058	3.14E-11
B85	B190	SCC	Cre	0.029057	2.82E-10
B85	B191	SCC	Urea	0.023616	2.95E-07
B86	B119	TBhCG	Hcv-rna	0.044909	0

B86	B129	TBhCG	IBIL	-0.04708	0
B86	B133	TBhCG	TBIL	-0.04294	0
B86	B134	TBhCG	CHE	-0.04065	0
B86	B137	TBhCG	AFU	-0.03968	0
B86	B193	TBhCG	K	0.084202	0
B86	B198	TBhCG	P	0.062711	0
B86	B204	TBhCG	Drinking	-0.04929	0
B86	B216	TBhCG	BMI	-0.04562	0
B86	B221	TBhCG	Hei	-0.04295	0
B86	B222	TBhCG	NS	-0.03987	0
B86	B87	TBhCG	NSE	-0.0948	0
B86	B90	TBhCG	CYFRA 21-1	0.202399	0
B86	B183	TBhCG	PTA	-0.03601	5.33E-15
B86	B146	TBhCG	CysC	-0.02876	4.28E-10
B86	B160	TBhCG	PH	-0.02856	5.63E-10
B86	B136	TBhCG	5'NT	0.027922	1.35E-09
B86	B218	TBhCG	DBP	-0.02731	3.06E-09
B86	B192	TBhCG	UA	-0.02689	5.28E-09
B86	B215	TBhCG	BMI	-0.02675	6.33E-09
B86	B107	TBhCG	AnuA	0.026372	1.03E-08
B86	B141	TBhCG	AST/ALT	0.025558	2.89E-08
B86	B88	TBhCG	CA 242	-0.02513	4.86E-08
B86	B128	TBhCG	ALT	-0.02477	7.60E-08
B86	B163	TBhCG	SG	-0.0244	1.17E-07
B86	B158	TBhCG	IgM	0.023634	2.89E-07
B86	B131	TBhCG	AST	-0.02359	3.04E-07
B86	B174	TBhCG	Urea/Cr	0.023488	3.42E-07
B86	B206	TBhCG	Waist	-0.02294	6.35E-07
B86	B116	TBhCG	TT3	0.022733	8.02E-07
B87	B110	NSE	AHA	0.046956	0
B87	B88	NSE	CA 242	0.147555	0
B87	B89	NSE	CA 72-4	-0.04286	0
B87	B90	NSE	CYFRA 21-1	0.062139	0
B87	B129	NSE	IBIL	0.034702	4.91E-14
B87	B189	NSE	eGFB	-0.02795	1.29E-09
B87	B133	NSE	TBIL	0.026745	6.39E-09
B87	B119	NSE	Hcv-rna	0.025621	2.67E-08
B87	B183	NSE	PTA	0.024566	9.67E-08
B88	B138	CA 242	PAB	-0.03059	3.10E-11
B90	B126	CYFRA 21-1	Alb	-0.04174	0
B90	B146	CYFRA 21-1	CysC	0.041166	0
B90	B187	CYFRA 21-1	NSE	0.038551	0
B90	B189	CYFRA 21-1	eGFB	-0.06843	0
B90	B190	CYFRA 21-1	Cre	0.036128	4.44E-15

B90	B191	CYFRA 21-1	Urea	0.03326	5.16E-13
B90	B183	CYFRA 21-1	PTA	-0.03189	4.43E-12
B90	B186	CYFRA 21-1	PFC	0.030374	4.27E-11
B90	B108	CYFRA 21-1	Anti-Cyclin	0.029725	1.09E-10
B90	B127	CYFRA 21-1	A-G ratio	-0.02693	5.02E-09
B90	B107	CYFRA 21-1	AnuA	-0.02677	6.17E-09
B90	B153	CYFRA 21-1	Glu	0.026081	1.50E-08
B90	B200	CYFRA 21-1	OP	0.023379	3.87E-07
B91	B111	TP	TSH	0.049474	0
B91	B126	TP	Alb	0.450521	0
B91	B127	TP	A-G ratio	-0.52256	0
B91	B128	TP	ALT	0.10949	0
B91	B130	TP	GLB	0.809169	0
B91	B131	TP	AST	0.133401	0
B91	B132	TP	Dbil	-0.04387	0
B91	B134	TP	CHE	0.061968	0
B91	B136	TP	5'NT	0.048189	0
B91	B139	TP	ADA	0.041762	0
B91	B140	TP	r-GT	0.080693	0
B91	B150	TP	ALP	0.04017	0
B91	B153	TP	Glu	0.134765	0
B91	B155	TP	IgA	0.063907	0
B91	B157	TP	IgG	0.101205	0
B91	B163	TP	SG	-0.05284	0
B91	B188	TP	Ca	0.32867	0
B91	B190	TP	Cre	-0.03847	0
B91	B192	TP	UA	0.095304	0
B91	B194	TP	Cl	-0.06678	0
B91	B204	TP	Drinking	-0.05361	0
B91	B209	TP	Smoking	-0.12507	0
B91	B218	TP	DBP	0.071995	0
B91	B220	TP	SBP	0.088851	0
B91	B221	TP	Hei	-0.0687	0
B91	B197	TP	AG	0.037859	2.22E-16
B91	B215	TP	BMI	0.037691	2.22E-16
B91	B115	TP	TSH TT3 TT4(TT3)	0.036782	1.33E-15
B91	B113	TP	FT3	0.035887	6.66E-15
B91	B133	TP	TBIL	-0.0334	4.10E-13
B91	B114	TP	TSH TT3 TT4(TT4)	0.032605	1.45E-12
B91	B193	TP	K	0.032124	3.07E-12
B91	B199	TP	Mg	0.03114	1.37E-11
B91	B158	TP	IgM	0.030902	1.96E-11
B91	B141	TP	AST/ALT	-0.02993	8.18E-11
B91	B138	TP	PAB	0.029661	1.20E-10

B91	B166	TP	Pro	0.029315	1.96E-10
B91	B149	TP	TGAb	0.028738	4.41E-10
B91	B112	TP	FT4	0.026947	4.91E-09
B91	B129	TP	IBIL	-0.02676	6.31E-09
B91	B170	TP	U-GLU	0.026692	6.85E-09
B91	B189	TP	eGFB	0.026213	1.27E-08
B91	B211	TP	IOP-R	0.025026	5.55E-08
B91	B137	TP	AFU	0.025021	5.58E-08
B91	B191	TP	Urea	-0.02422	1.45E-07
B91	B160	TP	PH	-0.02421	1.47E-07
B91	B148	TP	Anti_TPO	0.024181	1.53E-07
B91	B161	TP	LEU	0.022919	6.52E-07
B91	B203	TP	DH	0.022828	7.22E-07
B92	B138	ALDH2-Glu504Glu	PAB	0.073598	0
B92	B157	ALDH2-Glu504Glu	IgG	-0.04169	0
B92	B193	ALDH2-Glu504Glu	K	-0.03917	0
B92	B93	ALDH2-Glu504Glu	ALDH2-Glu504Lys	-0.98319	0
B92	B94	ALDH2-Glu504Glu	ALDH2-Lys504Lys	-0.17577	0
B92	B97	ALDH2-Glu504Glu	dsDNA	-0.05659	0
B92	B108	ALDH2-Glu504Glu	Anti-Cyclin	-0.03355	3.26E-13
B92	B124	ALDH2-Glu504Glu	ACTH	-0.03311	6.51E-13
B92	B125	ALDH2-Glu504Glu	Cor	-0.03224	2.58E-12
B92	B176	ALDH2-Glu504Glu	Ucr	0.031003	1.69E-11
B92	B99	ALDH2-Glu504Glu	anti-PM-Scl	0.030797	2.29E-11
B92	B96	ALDH2-Glu504Glu	dsDNA (IIF)	-0.03012	6.17E-11
B92	B204	ALDH2-Glu504Glu	Drinking	0.029884	8.70E-11
B92	B198	ALDH2-Glu504Glu	P	-0.02891	3.49E-10
B92	B135	ALDH2-Glu504Glu	TBA	0.026621	7.51E-09
B92	B107	ALDH2-Glu504Glu	AnuA	-0.02648	8.95E-09
B92	B181	ALDH2-Glu504Glu	APTT	-0.02648	9.04E-09
B92	B179	ALDH2-Glu504Glu	α1-MG	0.024268	1.38E-07
B92	B140	ALDH2-Glu504Glu	r-GT	0.024063	1.75E-07
B93	B138	ALDH2-Glu504Lys	PAB	-0.06705	0
B93	B157	ALDH2-Glu504Lys	IgG	0.042141	0
B93	B193	ALDH2-Glu504Lys	K	0.038785	0
B93	B97	ALDH2-Glu504Lys	dsDNA	0.058233	0
B93	B108	ALDH2-Glu504Lys	Anti-Cyclin	0.037079	8.88E-16
B93	B124	ALDH2-Glu504Lys	ACTH	0.035234	2.00E-14
B93	B125	ALDH2-Glu504Lys	Cor	0.034073	1.39E-13
B93	B99	ALDH2-Glu504Lys	anti-PM-Scl	-0.02996	7.83E-11
B93	B179	ALDH2-Glu504Lys	α1-MG	-0.02955	1.41E-10
B93	B176	ALDH2-Glu504Lys	Ucr	-0.02879	4.09E-10
B93	B198	ALDH2-Glu504Lys	P	0.028208	9.13E-10
B93	B135	ALDH2-Glu504Lys	TBA	-0.02658	7.92E-09

B93	B204	ALDH2-Glu504Lys	Drinking	-0.02617	1.34E-08
B94	B107	ALDH2-Lys504Lys	AnuA	0.066196	0
B94	B119	ALDH2-Lys504Lys	Hcv-rna	0.060135	0
B94	B138	ALDH2-Lys504Lys	PAB	-0.04216	0
B94	B183	ALDH2-Lys504Lys	PTA	0.062508	0
B94	B184	ALDH2-Lys504Lys	INR	0.052287	0
B94	B96	ALDH2-Lys504Lys	dsDNA (IIF)	0.046009	0
B94	B181	ALDH2-Lys504Lys	APTT	0.036986	8.88E-16
B94	B186	ALDH2-Lys504Lys	PFC	-0.0371	8.88E-16
B94	B179	ALDH2-Lys504Lys	α1-MG	0.025958	1.75E-08
B94	B180	ALDH2-Lys504Lys	Tf	0.02377	2.47E-07
B94	B204	ALDH2-Lys504Lys	Drinking	-0.02291	6.59E-07
B95	B107	anti-cenpb	AnuA	0.043126	0
B95	B183	anti-cenpb	PTA	0.057131	0
B95	B134	anti-cenpb	CHE	0.029598	1.31E-10
B96	B105	dsDNA (IIF)	anti-U1RNP	-0.05386	0
B96	B107	dsDNA (IIF)	AnuA	0.190139	0
B96	B119	dsDNA (IIF)	Hcv-rna	0.080446	0
B96	B122	dsDNA (IIF)	C4	-0.10754	0
B96	B124	dsDNA (IIF)	ACTH	-0.05241	0
B96	B138	dsDNA (IIF)	PAB	-0.05069	0
B96	B157	dsDNA (IIF)	IgG	0.045133	0
B96	B183	dsDNA (IIF)	PTA	0.054809	0
B96	B186	dsDNA (IIF)	PFC	-0.04484	0
B96	B97	dsDNA (IIF)	dsDNA	-0.37414	0
B96	B100	dsDNA (IIF)	anti-Ro52	0.035719	8.88E-15
B96	B137	dsDNA (IIF)	AFU	0.034709	4.84E-14
B96	B98	dsDNA (IIF)	anti-Jo-1	0.033599	2.99E-13
B96	B211	dsDNA (IIF)	IOP-R	-0.03099	1.72E-11
B96	B114	dsDNA (IIF)	TSH TT3 TT4(TT4)	0.02812	1.03E-09
B96	B212	dsDNA (IIF)	IOP-L	-0.02713	3.86E-09
B96	B109	dsDNA (IIF)	AMA-M2	-0.02585	2.01E-08
B96	B184	dsDNA (IIF)	INR	0.02555	2.91E-08
B96	B199	dsDNA (IIF)	Mg	-0.02393	2.06E-07
B97	B122	dsDNA	C4	0.090067	0
B97	B156	dsDNA	IgE	0.059585	0
B97	B109	dsDNA	AMA-M2	0.034444	7.53E-14
B97	B158	dsDNA	IgM	0.030331	4.55E-11
B97	B157	dsDNA	IgG	0.02628	1.16E-08
B97	B211	dsDNA	IOP-R	0.02357	3.11E-07
B98	B104	anti-Jo-1	anti-SSB	0.424772	0
B98	B107	anti-Jo-1	AnuA	0.107359	0
B98	B136	anti-Jo-1	5'NT	0.07154	0
B98	B157	anti-Jo-1	IgG	0.042911	0

B98	B193	anti-Jo-1	K	0.048868	0
B98	B197	anti-Jo-1	AG	-0.04687	0
B98	B138	anti-Jo-1	PAB	-0.03397	1.65E-13
B98	B195	anti-Jo-1	Na	-0.03366	2.73E-13
B98	B196	anti-Jo-1	TCO2	0.029696	1.14E-10
B98	B200	anti-Jo-1	OP	-0.02941	1.72E-10
B98	B115	anti-Jo-1	TSH TT3 TT4(TT3)	-0.02641	9.84E-09
B98	B119	anti-Jo-1	Hcv-rna	0.025229	4.33E-08
B98	B186	anti-Jo-1	PFC	-0.02349	3.41E-07
B98	B184	anti-Jo-1	INR	0.023308	4.20E-07
B98	B135	anti-Jo-1	TBA	-0.02308	5.41E-07
B99	B181	anti-PM-Scl	APTT	-0.05231	0
B99	B138	anti-PM-Scl	PAB	-0.03019	5.59E-11
B99	B134	anti-PM-Scl	CHE	-0.02956	1.39E-10
B99	B196	anti-PM-Scl	TCO2	-0.02613	1.41E-08
B99	B183	anti-PM-Scl	PTA	-0.02608	1.49E-08

Supplementary Table 4. PEI Correlations in Hypertension+Diabetes

Row	Column	Row	Column	Cor	*P*
B1	B127	Gender	A-G ratio	-0.12851	0
B1	B128	Gender	ALT	-0.10755	0
B1	B129	Gender	IBIL	-0.1385	0
B1	B130	Gender	GLB	0.134163	0
B1	B132	Gender	Dbil	-0.14545	0
B1	B133	Gender	TBIL	-0.15168	0
B1	B140	Gender	r-GT	-0.0897	0
B1	B141	Gender	AST/ALT	0.153424	0
B1	B161	Gender	LEU	0.231079	0
B1	B163	Gender	SG	-0.3312	0
B1	B166	Gender	Pro	-0.11354	0
B1	B170	Gender	U-GLU	-0.13798	0
B1	B173	Gender	NTT	0.161704	0
B1	B174	Gender	Urea/Cr	0.383518	0
B1	B176	Gender	Ucr	-0.09721	0
B1	B190	Gender	Cre	-0.21401	0
B1	B192	Gender	UA	-0.21452	0
B1	B2	Gender	Age	0.153036	0
B1	B204	Gender	Drinking	-0.26344	0
B1	B205	Gender	KDA	0.091752	0
B1	B206	Gender	Waist	-0.27292	0
B1	B207	Gender	WHR	-0.19793	0
B1	B209	Gender	Smoking	-0.30681	0
B1	B210	Gender	Hip	-0.20304	0
B1	B216	Gender	BMI	-0.49449	0
B1	B218	Gender	DBP	-0.16018	0
B1	B219	Gender	OT	0.117138	0
B1	B220	Gender	SBP	0.09064	0
B1	B221	Gender	Hei	-0.70189	0
B1	B222	Gender	NS	-0.40299	0
B1	B3	Gender	Hcy	-0.09383	0
B1	B41	Gender	MON％	-0.18094	0
B1	B42	Gender	MON	-0.18464	0
B1	B44	Gender	MCH	-0.15223	0
B1	B45	Gender	MCHC	-0.26604	0
B1	B46	Gender	RBC Count	-0.33072	0
B1	B48	Gender	LYM%	0.1453	0
B1	B50	Gender	eo%	-0.11451	0
B1	B51	Gender	Hb	-0.44565	0
B1	B52	Gender	PLT/BPC	0.131197	0
B1	B72	Gender	HDL-C	0.258304	0

B1	B74	Gender	ApoA1	0.120705	0
B1	B77	Gender	TC	0.154559	0
B1	B91	Gender	TP	0.089766	0
B1	B70	Gender	LDL-C	0.087694	4.44E-16
B1	B215	Gender	BMI	-0.08593	1.55E-15
B1	B62	Gender	WBHSRVI	-0.07919	2.00E-13
B1	B60	Gender	WBRVI	-0.07645	1.31E-12
B1	B149	Gender	TGAb	0.075437	2.58E-12
B1	B64	Gender	WBRSR(mPas_5)	-0.07081	5.07E-11
B1	B65	Gender	WBRSR(mPas_1)	-0.06971	1.00E-10
B1	B49	Gender	baso%	-0.06812	2.64E-10
B1	B53	Gender	GR%	-0.06736	4.16E-10
B1	B67	Gender	WBRSR(mPas_50)	-0.06692	5.41E-10
B1	B66	Gender	WBRSR(mPas_100)	-0.06601	9.23E-10
B1	B63	Gender	WBRSR(mPas_200)	-0.06529	1.40E-09
B1	B114	Gender	TSH TT3 TT4(TT4)	0.064045	2.86E-09
B1	B148	Gender	Anti_TPO	0.060982	1.56E-08
B1	B40	Gender	WBC	-0.0603	2.25E-08
B1	B201	Gender	e-habits	-0.05701	1.25E-07
B1	B86	Gender	TBhCG	0.056362	1.73E-07
B1	B172	Gender	KB	-0.05545	2.73E-07
B1	B56	Gender	IR	-0.05418	5.09E-07
B1	B54	Gender	ESR	0.053542	6.90E-07
B10	B11	PG I	PGI/PGII	0.137877	0
B10	B12	PG I	PG II	0.489321	0
B10	B189	PG I	eGFB	-0.1358	0
B10	B190	PG I	Cre	0.174876	0
B10	B191	PG I	Urea	0.148788	0
B10	B46	PG I	RBC Count	-0.08805	4.44E-16
B10	B145	PG I	BGP	-0.08574	1.78E-15
B10	B51	PG I	Hb	-0.07726	7.55E-13
B10	B126	PG I	Alb	-0.05962	3.22E-08
B10	B80	PG I	anti-HP IgG	0.055506	2.65E-07
B10	B144	PG I	Total-PINP	-0.05335	7.58E-07
B100	B101	anti-Ro52	anti-Sc70	0.163075	0
B100	B104	anti-Ro52	anti-SSB	0.163075	0
B100	B107	anti-Ro52	AnuA	0.161154	0
B100	B109	anti-Ro52	AMA-M2	0.94335	0
B100	B136	anti-Ro52	5'NT	0.197629	0
B100	B138	anti-Ro52	PAB	-0.08191	2.93E-14
B100	B139	anti-Ro52	ADA	0.074712	4.16E-12
B100	B184	anti-Ro52	INR	0.072702	1.54E-11
B100	B198	anti-Ro52	P	0.068092	2.69E-10
B100	B183	anti-Ro52	PTA	0.065635	1.15E-09

B100	B119	anti-Ro52	Hcv-rna	0.065257	1.43E-09
B100	B150	anti-Ro52	ALP	0.053938	5.71E-07
B100	B140	anti-Ro52	r-GT	0.053858	5.93E-07
B100	B161	anti-Ro52	LEU	0.053185	8.18E-07
B101	B107	anti-Sc70	AnuA	0.99526	0
B101	B109	anti-Sc70	AMA-M2	0.285121	0
B101	B115	anti-Sc70	TSH TT3 TT4(TT3)	0.097149	0
B101	B119	anti-Sc70	Hcv-rna	0.22665	0
B101	B124	anti-Sc70	ACTH	-0.1739	0
B101	B138	anti-Sc70	PAB	-0.12155	0
B101	B155	anti-Sc70	IgA	-0.11956	0
B101	B157	anti-Sc70	IgG	0.102519	0
B101	B158	anti-Sc70	IgM	0.152403	0
B101	B181	anti-Sc70	APTT	0.174055	0
B101	B182	anti-Sc70	TT	0.095817	0
B101	B184	anti-Sc70	INR	0.270459	0
B101	B185	anti-Sc70	PT	0.1744	0
B101	B186	anti-Sc70	PFC	-0.11045	0
B101	B194	anti-Sc70	Cl	0.185271	0
B101	B195	anti-Sc70	Na	0.134201	0
B101	B196	anti-Sc70	TCO2	0.119685	0
B101	B197	anti-Sc70	AG	-0.14837	0
B101	B199	anti-Sc70	Mg	0.168027	0
B101	B200	anti-Sc70	OP	-0.11588	0
B101	B179	anti-Sc70	α1-MG	-0.08204	2.66E-14
B101	B136	anti-Sc70	5'NT	-0.08078	6.57E-14
B101	B178	anti-Sc70	Ig-U	-0.07759	6.05E-13
B101	B139	anti-Sc70	ADA	-0.07705	8.74E-13
B101	B180	anti-Sc70	Tf	-0.0763	1.45E-12
B101	B125	anti-Sc70	Cor	-0.0744	5.11E-12
B101	B189	anti-Sc70	eGFB	-0.07423	5.72E-12
B101	B121	anti-Sc70	C3	-0.07191	2.54E-11
B101	B212	anti-Sc70	IOP-L	-0.06297	5.22E-09
B101	B102	anti-Sc70	anti-Sm	-0.06065	1.86E-08
B101	B103	anti-Sc70	anti-SSA/Ro	0.056307	1.78E-07
B101	B211	anti-Sc70	IOP-R	-0.05567	2.45E-07
B102	B103	anti-Sm	anti-SSA/Ro	0.776383	0
B102	B105	anti-Sm	anti-U1RNP	0.997977	0
B102	B151	anti-Sm	Anti-CCP	0.077406	6.86E-13
B102	B107	anti-Sm	AnuA	-0.06128	1.33E-08
B102	B104	anti-Sm	anti-SSB	-0.06065	1.86E-08
B103	B105	anti-SSA/Ro	anti-U1RNP	0.781397	0
B103	B124	anti-SSA/Ro	ACTH	0.207708	0
B103	B182	anti-SSA/Ro	TT	-0.14512	0

B103	B151	anti-SSA/Ro	Anti-CCP	0.084699	3.77E-15
B103	B185	anti-SSA/Ro	PT	-0.08089	6.06E-14
B103	B104	anti-SSA/Ro	anti-SSB	0.056307	1.78E-07
B103	B107	anti-SSA/Ro	AnuA	0.054593	4.16E-07
B104	B107	anti-SSB	AnuA	0.995262	0
B104	B109	anti-SSB	AMA-M2	0.285121	0
B104	B115	anti-SSB	TSH TT3 TT4(TT3)	0.097149	0
B104	B119	anti-SSB	Hcv-rna	0.22665	0
B104	B124	anti-SSB	ACTH	-0.1739	0
B104	B138	anti-SSB	PAB	-0.12155	0
B104	B155	anti-SSB	IgA	-0.11956	0
B104	B157	anti-SSB	IgG	0.102519	0
B104	B158	anti-SSB	IgM	0.152403	0
B104	B181	anti-SSB	APTT	0.174055	0
B104	B182	anti-SSB	TT	0.095817	0
B104	B184	anti-SSB	INR	0.270459	0
B104	B185	anti-SSB	PT	0.1744	0
B104	B186	anti-SSB	PFC	-0.11045	0
B104	B194	anti-SSB	Cl	0.185271	0
B104	B195	anti-SSB	Na	0.134201	0
B104	B196	anti-SSB	TCO2	0.119685	0
B104	B197	anti-SSB	AG	-0.14837	0
B104	B199	anti-SSB	Mg	0.168027	0
B104	B200	anti-SSB	OP	-0.11588	0
B104	B179	anti-SSB	α1-MG	-0.08204	2.66E-14
B104	B136	anti-SSB	5'NT	-0.08078	6.57E-14
B104	B178	anti-SSB	Ig-U	-0.07759	6.05E-13
B104	B139	anti-SSB	ADA	-0.07705	8.74E-13
B104	B180	anti-SSB	Tf	-0.0763	1.45E-12
B104	B125	anti-SSB	Cor	-0.0744	5.11E-12
B104	B189	anti-SSB	eGFB	-0.07423	5.72E-12
B104	B121	anti-SSB	C3	-0.07191	2.54E-11
B104	B212	anti-SSB	IOP-L	-0.06297	5.22E-09
B104	B211	anti-SSB	IOP-R	-0.05567	2.45E-07
B105	B151	anti-U1RNP	Anti-CCP	0.078292	3.74E-13
B106	B194	anti-P	Cl	0.085267	2.44E-15
B106	B197	anti-P	AG	-0.06377	3.34E-09
B107	B109	AnuA	AMA-M2	0.28289	0
B107	B115	AnuA	TSH TT3 TT4(TT3)	0.099071	0
B107	B119	AnuA	Hcv-rna	0.227775	0
B107	B124	AnuA	ACTH	-0.17474	0
B107	B138	AnuA	PAB	-0.12213	0
B107	B155	AnuA	IgA	-0.12015	0
B107	B157	AnuA	IgG	0.103022	0

B107	B158	AnuA	IgM	0.153164	0
B107	B181	AnuA	APTT	0.174909	0
B107	B182	AnuA	TT	0.09628	0
B107	B184	AnuA	INR	0.27178	0
B107	B185	AnuA	PT	0.175248	0
B107	B186	AnuA	PFC	-0.11101	0
B107	B194	AnuA	Cl	0.186205	0
B107	B195	AnuA	Na	0.13484	0
B107	B196	AnuA	TCO2	0.120255	0
B107	B197	AnuA	AG	-0.14912	0
B107	B199	AnuA	Mg	0.16892	0
B107	B200	AnuA	OP	-0.11655	0
B107	B179	AnuA	α1-MG	-0.08247	1.95E-14
B107	B136	AnuA	5'NT	-0.08117	4.97E-14
B107	B178	AnuA	Ig-U	-0.07798	4.63E-13
B107	B139	AnuA	ADA	-0.07751	6.40E-13
B107	B180	AnuA	Tf	-0.07668	1.12E-12
B107	B125	AnuA	Cor	-0.07477	4.01E-12
B107	B189	AnuA	eGFB	-0.07365	8.35E-12
B107	B121	AnuA	C3	-0.07225	2.04E-11
B107	B212	AnuA	IOP-L	-0.06206	8.66E-09
B107	B211	AnuA	IOP-R	-0.0552	3.09E-07
B108	B151	Anti-Cyclin	Anti-CCP	-0.10356	0
B108	B185	Anti-Cyclin	PT	-0.09646	0
B108	B199	Anti-Cyclin	Mg	-0.12652	0
B108	B145	Anti-Cyclin	BGP	-0.0676	3.60E-10
B108	B195	Anti-Cyclin	Na	-0.06676	5.95E-10
B108	B125	Anti-Cyclin	Cor	-0.06471	1.95E-09
B108	B137	Anti-Cyclin	AFU	0.055259	3.00E-07
B108	B176	Anti-Cyclin	Ucr	-0.05487	3.63E-07
B108	B144	Anti-Cyclin	Total-PINP	-0.0548	3.76E-07
B108	B182	Anti-Cyclin	TT	-0.05429	4.81E-07
B109	B136	AMA-M2	5'NT	0.201726	0
B109	B184	AMA-M2	INR	0.102764	0
B109	B138	AMA-M2	PAB	-0.08797	4.44E-16
B109	B119	AMA-M2	Hcv-rna	0.086293	1.11E-15
B109	B185	AMA-M2	PT	0.066219	8.16E-10
B109	B181	AMA-M2	APTT	0.06616	8.45E-10
B109	B124	AMA-M2	ACTH	-0.06593	9.65E-10
B109	B198	AMA-M2	P	0.065495	1.24E-09
B109	B200	AMA-M2	OP	-0.06375	3.37E-09
B109	B150	AMA-M2	ALP	0.05941	3.61E-08
B109	B158	AMA-M2	IgM	0.05807	7.26E-08
B109	B139	AMA-M2	ADA	0.055115	3.22E-07

B109	B161	AMA-M2	LEU	0.054432	4.50E-07
B109	B140	AMA-M2	r-GT	0.05296	9.11E-07
B11	B12	PGI/PGII	PG II	-0.38597	0
B11	B14	PGI/PGII	G-17	-0.257	0
B11	B80	PGI/PGII	anti-HP IgG	-0.12218	0
B11	B145	PGI/PGII	BGP	-0.08747	4.44E-16
B11	B144	PGI/PGII	Total-PINP	-0.06283	5.65E-09
B11	B149	PGI/PGII	TGAb	-0.05882	4.92E-08
B11	B143	PGI/PGII	β-CTx	-0.0581	7.14E-08
B110	B119	AHA	Hcv-rna	-0.1391	0
B110	B150	AHA	ALP	0.095019	0
B110	B194	AHA	Cl	-0.1925	0
B110	B195	AHA	Na	-0.1366	0
B110	B200	AHA	OP	-0.09559	0
B110	B228	AHA	OS	0.125856	0
B111	B112	TSH	FT4	-0.21582	0
B111	B113	TSH	FT3	-0.16747	0
B111	B114	TSH	TSH TT3 TT4(TT4)	-0.10289	0
B111	B139	TSH	ADA	0.110945	0
B111	B148	TSH	Anti_TPO	0.167898	0
B111	B149	TSH	TGAb	0.181257	0
B111	B115	TSH	TSH TT3 TT4(TT3)	-0.08865	2.22E-16
B111	B131	TSH	AST	0.058975	4.54E-08
B112	B113	FT4	FT3	0.182664	0
B112	B114	FT4	TSH TT3 TT4(TT4)	0.522105	0
B112	B115	FT4	TSH TT3 TT4(TT3)	0.19654	0
B112	B189	FT4	eGFB	0.064772	1.89E-09
B112	B199	FT4	Mg	-0.05551	2.65E-07
B113	B114	FT3	TSH TT3 TT4(TT4)	0.249349	0
B113	B115	FT3	TSH TT3 TT4(TT3)	0.518979	0
B113	B126	FT3	Alb	0.097472	0
B113	B145	FT3	BGP	0.104127	0
B113	B189	FT3	eGFB	0.093612	0
B113	B191	FT3	Urea	-0.08825	2.22E-16
B113	B123	FT3	hs-CRP1	-0.07409	6.26E-12
B113	B190	FT3	Cre	-0.07354	8.92E-12
B113	B176	FT3	Ucr	0.070766	5.22E-11
B113	B146	FT3	CysC	-0.06563	1.15E-09
B113	B134	FT3	CHE	0.061747	1.03E-08
B113	B144	FT3	Total-PINP	0.058829	4.90E-08
B113	B127	FT3	A-G ratio	0.056024	2.05E-07
B113	B195	FT3	Na	0.054276	4.85E-07
B114	B115	TSH TT3 TT4(TT4)	TSH TT3 TT4(TT3)	0.436263	0
B114	B199	TSH TT3 TT4(TT4)	Mg	-0.06472	1.94E-09

B114	B136	TSH TT3 TT4(TT4)	5'NT	0.064671	2.00E-09
B114	B225	TSH TT3 TT4(TT4)	hPBG	0.059038	4.39E-08
B114	B156	TSH TT3 TT4(TT4)	IgE	0.057742	8.60E-08
B114	B149	TSH TT3 TT4(TT4)	TGAb	0.053852	5.95E-07
B115	B123	TSH TT3 TT4(TT3)	hs-CRP1	-0.09596	0
B115	B200	TSH TT3 TT4(TT3)	OP	-0.09334	0
B115	B191	TSH TT3 TT4(TT3)	Urea	-0.08672	8.88E-16
B115	B190	TSH TT3 TT4(TT3)	Cre	-0.0831	1.24E-14
B115	B146	TSH TT3 TT4(TT3)	CysC	-0.08143	4.13E-14
B115	B119	TSH TT3 TT4(TT3)	Hcv-rna	0.079472	1.65E-13
B115	B196	TSH TT3 TT4(TT3)	TCO2	0.078929	2.41E-13
B115	B136	TSH TT3 TT4(TT3)	5'NT	0.070333	6.84E-11
B115	B199	TSH TT3 TT4(TT3)	Mg	0.062089	8.52E-09
B115	B186	TSH TT3 TT4(TT3)	PFC	-0.06178	1.01E-08
B115	B189	TSH TT3 TT4(TT3)	eGFB	0.060903	1.63E-08
B115	B179	TSH TT3 TT4(TT3)	α1-MG	-0.05746	9.92E-08
B115	B134	TSH TT3 TT4(TT3)	CHE	0.054191	5.05E-07
B115	B126	TSH TT3 TT4(TT3)	Alb	0.053753	6.24E-07
B116	B117	TT3	TT4	-0.42507	0
B118	B189	CEA1	eGFB	-0.07469	4.21E-12
B118	B126	CEA1	Alb	-0.06618	8.33E-10
B119	B120	Hcv-rna	HCV-cAg	-0.15138	0
B119	B124	Hcv-rna	ACTH	-0.14992	0
B119	B157	Hcv-rna	IgG	0.096414	0
B119	B183	Hcv-rna	PTA	0.11187	0
B119	B184	Hcv-rna	INR	0.120911	0
B119	B194	Hcv-rna	Cl	0.16201	0
B119	B197	Hcv-rna	AG	-0.11004	0
B119	B186	Hcv-rna	PFC	-0.08131	4.51E-14
B119	B181	Hcv-rna	APTT	0.077338	7.18E-13
B119	B179	Hcv-rna	α1-MG	-0.07723	7.74E-13
B119	B200	Hcv-rna	OP	-0.07118	4.02E-11
B119	B193	Hcv-rna	K	0.066158	8.46E-10
B119	B121	Hcv-rna	C3	-0.06546	1.27E-09
B119	B182	Hcv-rna	TT	0.06529	1.40E-09
B119	B185	Hcv-rna	PT	0.064618	2.06E-09
B119	B139	Hcv-rna	ADA	-0.06335	4.23E-09
B119	B211	Hcv-rna	IOP-R	-0.06318	4.65E-09
B119	B212	Hcv-rna	IOP-L	-0.06305	4.99E-09
B119	B151	Hcv-rna	Anti-CCP	-0.059	4.47E-08
B119	B134	Hcv-rna	CHE	0.058918	4.67E-08
B119	B189	Hcv-rna	eGFB	-0.05404	5.42E-07
B12	B14	PG II	G-17	0.478576	0
B12	B189	PG II	eGFB	-0.10346	0

B12	B190	PG II	Cre	0.093293	0
B12	B80	PG II	anti-HP IgG	0.126273	0
B12	B191	PG II	Urea	0.078561	3.10E-13
B120	B155	HCV-cAg	IgA	0.151734	0
B120	B158	HCV-cAg	IgM	0.354185	0
B120	B181	HCV-cAg	APTT	0.106332	0
B120	B184	HCV-cAg	INR	-0.18451	0
B120	B185	HCV-cAg	PT	-0.1221	0
B120	B186	HCV-cAg	PFC	0.235194	0
B120	B195	HCV-cAg	Na	-0.09503	0
B120	B198	HCV-cAg	P	0.14511	0
B120	B199	HCV-cAg	Mg	0.097477	0
B120	B194	HCV-cAg	Cl	-0.07945	1.67E-13
B120	B134	HCV-cAg	CHE	0.072499	1.75E-11
B120	B182	HCV-cAg	TT	0.068573	2.01E-10
B120	B124	HCV-cAg	ACTH	-0.06568	1.12E-09
B120	B196	HCV-cAg	TCO2	-0.06161	1.11E-08
B120	B183	HCV-cAg	PTA	-0.05564	2.48E-07
B121	B122	C3	C4	0.888077	0
B121	B134	C3	CHE	0.103215	0
B121	B194	C3	Cl	-0.09202	0
B121	B155	C3	IgA	0.056258	1.83E-07
B122	B134	C4	CHE	0.096094	0
B122	B194	C4	Cl	-0.07536	2.71E-12
B122	B158	C4	IgM	0.062493	6.81E-09
B122	B155	C4	IgA	0.058841	4.86E-08
B122	B156	C4	IgE	-0.05324	7.98E-07
B123	B125	hs-CRP1	Cor	0.142839	0
B123	B179	hs-CRP1	α1-MG	0.197096	0
B123	B186	hs-CRP1	PFC	0.200928	0
B123	B126	hs-CRP1	Alb	-0.08027	9.39E-14
B123	B127	hs-CRP1	A-G ratio	-0.07014	7.72E-11
B123	B176	hs-CRP1	Ucr	0.066032	9.11E-10
B123	B124	hs-CRP1	ACTH	0.061317	1.30E-08
B123	B157	hs-CRP1	IgG	-0.05821	6.74E-08
B123	B194	hs-CRP1	Cl	-0.05778	8.44E-08
B123	B150	hs-CRP1	ALP	0.056859	1.35E-07
B123	B130	hs-CRP1	GLB	0.056832	1.37E-07
B123	B153	hs-CRP1	Glu	0.056702	1.46E-07
B123	B175	hs-CRP1	ALB-U	0.054019	5.49E-07
B124	B125	ACTH	Cor	0.337736	0
B124	B182	ACTH	TT	-0.1231	0
B124	B183	ACTH	PTA	-0.12986	0
B124	B184	ACTH	INR	-0.10328	0

B124	B186	ACTH	PFC	0.158316	0
B124	B181	ACTH	APTT	-0.0658	1.04E-09
B124	B194	ACTH	Cl	-0.06212	8.38E-09
B124	B212	ACTH	IOP-L	0.059668	3.15E-08
B124	B155	ACTH	IgA	-0.05661	1.53E-07
B124	B151	ACTH	Anti-CCP	0.056213	1.87E-07
B124	B137	ACTH	AFU	-0.05372	6.34E-07
B125	B159	Cor	β2-MG	-0.21125	0
B125	B181	Cor	APTT	-0.16551	0
B125	B184	Cor	INR	-0.15857	0
B125	B186	Cor	PFC	0.18127	0
B125	B225	Cor	hPBG	0.164001	0
B125	B183	Cor	PTA	-0.08522	2.66E-15
B125	B156	Cor	IgE	-0.06822	2.48E-10
B125	B151	Cor	Anti-CCP	0.061685	1.06E-08
B125	B157	Cor	IgG	-0.06168	1.06E-08
B125	B155	Cor	IgA	-0.05852	5.76E-08
B126	B127	Alb	A-G ratio	0.523887	0
B126	B128	Alb	ALT	0.123123	0
B126	B129	Alb	IBIL	0.158081	0
B126	B130	Alb	GLB	-0.20006	0
B126	B133	Alb	TBIL	0.150828	0
B126	B138	Alb	PAB	0.103262	0
B126	B141	Alb	AST/ALT	-0.13606	0
B126	B146	Alb	CysC	-0.13226	0
B126	B175	Alb	ALB-U	-0.11669	0
B126	B177	Alb	ACR	-0.13885	0
B126	B188	Alb	Ca	0.35527	0
B126	B189	Alb	eGFB	0.273514	0
B126	B190	Alb	Cre	-0.16744	0
B126	B191	Alb	Urea	-0.20664	0
B126	B218	Alb	DBP	0.143708	0
B126	B221	Alb	Hei	0.111628	0
B126	B132	Alb	Dbil	0.088526	2.22E-16
B126	B178	Alb	Ig-U	-0.08629	1.11E-15
B126	B204	Alb	Drinking	0.083697	7.99E-15
B126	B216	Alb	BMI	0.081147	5.06E-14
B126	B180	Alb	Tf	-0.08051	7.93E-14
B126	B166	Alb	Pro	-0.07869	2.83E-13
B126	B134	Alb	CHE	0.062631	6.31E-09
B126	B173	Alb	NTT	-0.0601	2.50E-08
B126	B131	Alb	AST	0.055088	3.26E-07
B126	B139	Alb	ADA	-0.05504	3.34E-07
B126	B179	Alb	α1-MG	-0.05308	8.59E-07

B127	B129	A-G ratio	IBIL	0.161975	0
B127	B130	A-G ratio	GLB	-0.88609	0
B127	B132	A-G ratio	Dbil	0.13161	0
B127	B133	A-G ratio	TBIL	0.166505	0
B127	B141	A-G ratio	AST/ALT	-0.12129	0
B127	B153	A-G ratio	Glu	-0.11349	0
B127	B157	A-G ratio	IgG	-0.09943	0
B127	B189	A-G ratio	eGFB	0.229236	0
B127	B190	A-G ratio	Cre	-0.11513	0
B127	B191	A-G ratio	Urea	-0.12758	0
B127	B204	A-G ratio	Drinking	0.111134	0
B127	B209	A-G ratio	Smoking	0.108884	0
B127	B216	A-G ratio	BMI	0.090908	0
B127	B220	A-G ratio	SBP	-0.12114	0
B127	B221	A-G ratio	Hei	0.174514	0
B127	B146	A-G ratio	CysC	-0.08795	4.44E-16
B127	B155	A-G ratio	IgA	-0.08317	1.18E-14
B127	B192	A-G ratio	UA	-0.07993	1.19E-13
B127	B163	A-G ratio	SG	0.077515	6.37E-13
B127	B150	A-G ratio	ALP	-0.07728	7.49E-13
B127	B139	A-G ratio	ADA	-0.0756	2.31E-12
B127	B138	A-G ratio	PAB	0.074009	6.60E-12
B127	B166	A-G ratio	Pro	-0.06993	8.78E-11
B127	B140	A-G ratio	r-GT	-0.06757	3.67E-10
B127	B177	A-G ratio	ACR	-0.06107	1.48E-08
B127	B161	A-G ratio	LEU	-0.05934	3.75E-08
B127	B188	A-G ratio	Ca	0.05742	1.01E-07
B127	B207	A-G ratio	WHR	-0.0562	1.88E-07
B127	B131	A-G ratio	AST	-0.05573	2.38E-07
B127	B222	A-G ratio	NS	0.053047	8.74E-07
B128	B131	ALT	AST	0.78266	0
B128	B132	ALT	Dbil	0.155954	0
B128	B133	ALT	TBIL	0.110425	0
B128	B140	ALT	r-GT	0.347904	0
B128	B141	ALT	AST/ALT	-0.49505	0
B128	B163	ALT	SG	0.093193	0
B128	B189	ALT	eGFB	0.147465	0
B128	B191	ALT	Urea	-0.10772	0
B128	B206	ALT	Waist	0.112597	0
B128	B215	ALT	BMI	0.109935	0
B128	B216	ALT	BMI	0.152465	0
B128	B218	ALT	DBP	0.105302	0
B128	B221	ALT	Hei	0.115145	0
B128	B210	ALT	Hip	0.082547	1.84E-14

B128	B129	ALT	IBIL	0.082491	1.91E-14
B128	B136	ALT	5'NT	0.082324	2.18E-14
B128	B207	ALT	WHR	0.079105	2.13E-13
B128	B222	ALT	NS	0.079017	2.26E-13
B128	B204	ALT	Drinking	0.078563	3.10E-13
B128	B190	ALT	Cre	-0.06806	2.74E-10
B128	B137	ALT	AFU	0.066885	5.52E-10
B128	B176	ALT	Ucr	0.063426	4.05E-09
B128	B139	ALT	ADA	0.058049	7.34E-08
B128	B188	ALT	Ca	0.057114	1.19E-07
B128	B192	ALT	UA	0.056377	1.72E-07
B129	B130	IBIL	GLB	-0.12929	0
B129	B131	IBIL	AST	0.11458	0
B129	B132	IBIL	Dbil	0.62861	0
B129	B133	IBIL	TBIL	0.974239	0
B129	B163	IBIL	SG	0.091817	0
B129	B191	IBIL	Urea	-0.12879	0
B129	B221	IBIL	Hei	0.128133	0
B129	B174	IBIL	Urea/Cr	-0.0856	1.78E-15
B129	B190	IBIL	Cre	-0.07919	2.01E-13
B129	B218	IBIL	DBP	0.071893	2.57E-11
B129	B216	IBIL	BMI	0.067074	4.93E-10
B129	B160	IBIL	PH	0.064828	1.83E-09
B129	B144	IBIL	Total-PINP	-0.06166	1.07E-08
B129	B145	IBIL	BGP	-0.05867	5.31E-08
B129	B143	IBIL	β-CTx	-0.05317	8.25E-07
B130	B131	GLB	AST	0.095717	0
B130	B132	GLB	Dbil	-0.10963	0
B130	B133	GLB	TBIL	-0.13422	0
B130	B153	GLB	Glu	0.131622	0
B130	B155	GLB	IgA	0.099175	0
B130	B157	GLB	IgG	0.123394	0
B130	B189	GLB	eGFB	-0.16273	0
B130	B192	GLB	UA	0.107441	0
B130	B204	GLB	Drinking	-0.10191	0
B130	B209	GLB	Smoking	-0.11527	0
B130	B220	GLB	SBP	0.129724	0
B130	B221	GLB	Hei	-0.16547	0
B130	B141	GLB	AST/ALT	0.086842	8.88E-16
B130	B163	GLB	SG	-0.08404	6.22E-15
B130	B216	GLB	BMI	-0.08307	1.27E-14
B130	B140	GLB	r-GT	0.082697	1.64E-14
B130	B150	GLB	ALP	0.081325	4.44E-14
B130	B188	GLB	Ca	0.076089	1.67E-12

B130	B191	GLB	Urea	0.072085	2.28E-11
B130	B190	GLB	Cre	0.071282	3.78E-11
B130	B139	GLB	ADA	0.066078	8.86E-10
B130	B207	GLB	WHR	0.060884	1.64E-08
B130	B158	GLB	IgM	0.058247	6.63E-08
B130	B161	GLB	LEU	0.056633	1.51E-07
B130	B136	GLB	5'NT	0.055338	2.88E-07
B130	B194	GLB	Cl	-0.05519	3.11E-07
B130	B166	GLB	Pro	0.052955	9.13E-07
B131	B132	AST	Dbil	0.206544	0
B131	B133	AST	TBIL	0.150596	0
B131	B136	AST	5'NT	0.11215	0
B131	B139	AST	ADA	0.090851	0
B131	B140	AST	r-GT	0.42592	0
B131	B141	AST	AST/ALT	-0.10245	0
B131	B188	AST	Ca	0.080932	5.91E-14
B131	B191	AST	Urea	-0.07934	1.81E-13
B131	B137	AST	AFU	0.078955	2.36E-13
B131	B192	AST	UA	0.075053	3.32E-12
B131	B204	AST	Drinking	0.070389	6.60E-11
B131	B215	AST	BMI	0.066383	7.41E-10
B131	B206	AST	Waist	0.06339	4.13E-09
B131	B189	AST	eGFB	0.060431	2.10E-08
B131	B190	AST	Cre	-0.05837	6.22E-08
B131	B216	AST	BMI	0.05782	8.26E-08
B132	B133	Dbil	TBIL	0.787705	0
B132	B140	Dbil	r-GT	0.144084	0
B132	B191	Dbil	Urea	-0.10764	0
B132	B221	Dbil	Hei	0.116554	0
B132	B174	Dbil	Urea/Cr	-0.08106	5.37E-14
B132	B152	Dbil	ACA-Ig(G-A-M)	0.068314	2.35E-10
B132	B204	Dbil	Drinking	0.067252	4.44E-10
B132	B160	Dbil	PH	0.061508	1.17E-08
B132	B141	Dbil	AST/ALT	-0.05809	7.19E-08
B132	B190	Dbil	Cre	-0.05563	2.49E-07
B132	B135	Dbil	TBA	0.054602	4.14E-07
B133	B174	TBIL	Urea/Cr	-0.09132	0
B133	B191	TBIL	Urea	-0.1333	0
B133	B221	TBIL	Hei	0.135157	0
B133	B163	TBIL	SG	0.087178	4.44E-16
B133	B190	TBIL	Cre	-0.07888	2.50E-13
B133	B218	TBIL	DBP	0.069555	1.10E-10
B133	B140	TBIL	r-GT	0.069375	1.23E-10
B133	B160	TBIL	PH	0.069055	1.50E-10

B133	B216	TBIL	BMI	0.066936	5.35E-10
B133	B152	TBIL	ACA-Ig(G-A-M)	0.060925	1.61E-08
B133	B145	TBIL	BGP	-0.05377	6.20E-07
B134	B135	CHE	TBA	-0.10557	0
B134	B136	CHE	5'NT	0.167477	0
B134	B137	CHE	AFU	0.232748	0
B134	B138	CHE	PAB	0.250666	0
B134	B146	CHE	CysC	-0.11392	0
B134	B184	CHE	INR	-0.0898	0
B134	B194	CHE	Cl	-0.18043	0
B134	B197	CHE	AG	0.101758	0
B134	B198	CHE	P	-0.12829	0
B134	B151	CHE	Anti-CCP	-0.08121	4.84E-14
B134	B216	CHE	BMI	0.0804	8.59E-14
B134	B185	CHE	PT	-0.07931	1.85E-13
B134	B150	CHE	ALP	0.077365	7.05E-13
B134	B215	CHE	BMI	0.071693	2.92E-11
B134	B140	CHE	r-GT	0.071686	2.93E-11
B134	B186	CHE	PFC	0.065731	1.09E-09
B134	B195	CHE	Na	-0.06561	1.16E-09
B134	B153	CHE	Glu	0.064508	2.19E-09
B134	B190	CHE	Cre	-0.06344	4.02E-09
B134	B193	CHE	K	-0.06181	9.95E-09
B134	B218	CHE	DBP	0.060132	2.46E-08
B134	B199	CHE	Mg	-0.0587	5.25E-08
B134	B141	CHE	AST/ALT	-0.05826	6.59E-08
B134	B206	CHE	Waist	0.055866	2.22E-07
B134	B170	CHE	U-GLU	0.054775	3.80E-07
B135	B137	TBA	AFU	0.122446	0
B135	B138	TBA	PAB	-0.16254	0
B135	B139	TBA	ADA	0.157055	0
B135	B150	TBA	ALP	0.221218	0
B135	B157	TBA	IgG	0.134816	0
B135	B194	TBA	Cl	0.055092	3.26E-07
B135	B198	TBA	P	0.053514	7.00E-07
B136	B137	5'NT	AFU	0.182733	0
B136	B139	5'NT	ADA	0.184754	0
B136	B140	5'NT	r-GT	0.310373	0
B136	B150	5'NT	ALP	0.249265	0
B136	B155	5'NT	IgA	0.105224	0
B136	B157	5'NT	IgG	0.096301	0
B136	B182	5'NT	TT	0.109363	0
B136	B184	5'NT	INR	-0.12153	0
B136	B193	5'NT	K	-0.15961	0

B136	B194	5'NT	Cl	-0.18477	0
B136	B197	5'NT	AG	0.137573	0
B136	B198	5'NT	P	-0.13305	0
B136	B181	5'NT	APTT	-0.08546	2.22E-15
B136	B185	5'NT	PT	-0.06968	1.02E-10
B136	B199	5'NT	Mg	-0.06864	1.93E-10
B136	B195	5'NT	Na	-0.0677	3.39E-10
B136	B196	5'NT	TCO2	-0.05465	4.04E-07
B137	B139	AFU	ADA	0.322652	0
B137	B140	AFU	r-GT	0.130002	0
B137	B150	AFU	ALP	0.165956	0
B137	B180	AFU	Tf	0.100361	0
B137	B194	AFU	Cl	-0.17934	0
B137	B199	AFU	Mg	-0.15935	0
B137	B200	AFU	OP	0.150628	0
B137	B155	AFU	IgA	0.08749	4.44E-16
B137	B178	AFU	Ig-U	0.088119	4.44E-16
B137	B204	AFU	Drinking	0.077102	8.43E-13
B137	B175	AFU	ALB-U	0.069408	1.21E-10
B137	B198	AFU	P	-0.0683	2.38E-10
B137	B193	AFU	K	-0.06655	6.72E-10
B137	B157	AFU	IgG	0.062453	6.96E-09
B137	B170	AFU	U-GLU	0.061713	1.05E-08
B137	B153	AFU	Glu	0.061076	1.48E-08
B137	B179	AFU	α1-MG	0.059522	3.40E-08
B137	B197	AFU	AG	0.058108	7.12E-08
B137	B163	AFU	SG	0.054735	3.88E-07
B137	B225	AFU	hPBG	0.053875	5.88E-07
B137	B195	AFU	Na	-0.05377	6.20E-07
B138	B139	PAB	ADA	-0.3112	0
B138	B150	PAB	ALP	-0.15324	0
B138	B157	PAB	IgG	-0.11377	0
B138	B194	PAB	Cl	-0.14952	0
B138	B197	PAB	AG	0.129872	0
B138	B198	PAB	P	-0.11826	0
B138	B185	PAB	PT	-0.08273	1.62E-14
B138	B184	PAB	INR	-0.07725	7.64E-13
B138	B155	PAB	IgA	-0.07678	1.05E-12
B138	B212	PAB	IOP-L	0.073373	9.97E-12
B138	B204	PAB	Drinking	0.072738	1.50E-11
B138	B146	PAB	CysC	-0.06913	1.43E-10
B138	B181	PAB	APTT	-0.06436	2.39E-09
B138	B211	PAB	IOP-R	0.059634	3.21E-08
B138	B188	PAB	Ca	0.057983	7.60E-08

B138	B180	PAB	Tf	0.054446	4.46E-07
B139	B140	ADA	r-GT	0.101992	0
B139	B146	ADA	CysC	0.119859	0
B139	B150	ADA	ALP	0.259728	0
B139	B153	ADA	Glu	0.090296	0
B139	B155	ADA	IgA	0.098713	0
B139	B181	ADA	APTT	-0.10043	0
B139	B186	ADA	PFC	0.091788	0
B139	B194	ADA	Cl	-0.22164	0
B139	B197	ADA	AG	0.097127	0
B139	B199	ADA	Mg	-0.21036	0
B139	B200	ADA	OP	0.323721	0
B139	B193	ADA	K	-0.07496	3.54E-12
B139	B178	ADA	Ig-U	0.074858	3.78E-12
B139	B182	ADA	TT	0.068709	1.85E-10
B139	B170	ADA	U-GLU	0.068621	1.95E-10
B139	B148	ADA	Anti_TPO	0.068292	2.38E-10
B139	B180	ADA	Tf	0.066436	7.19E-10
B139	B177	ADA	ACR	0.065871	1.00E-09
B139	B195	ADA	Na	-0.06296	5.25E-09
B139	B152	ADA	ACA-Ig(G-A-M)	0.060544	1.97E-08
B139	B184	ADA	INR	-0.05885	4.84E-08
B14	B149	G-17	TGAb	0.139228	0
B14	B148	G-17	Anti_TPO	0.072761	1.48E-11
B140	B141	r-GT	AST/ALT	-0.10962	0
B140	B150	r-GT	ALP	0.138885	0
B140	B194	r-GT	Cl	-0.09249	0
B140	B204	r-GT	Drinking	0.131537	0
B140	B206	r-GT	Waist	0.090611	0
B140	B209	r-GT	Smoking	0.095859	0
B140	B216	r-GT	BMI	0.098027	0
B140	B153	r-GT	Glu	0.088684	2.22E-16
B140	B218	r-GT	DBP	0.086411	8.88E-16
B140	B189	r-GT	eGFB	0.0832	1.15E-14
B140	B192	r-GT	UA	0.082293	2.22E-14
B140	B163	r-GT	SG	0.080989	5.66E-14
B140	B207	r-GT	WHR	0.080854	6.22E-14
B140	B170	r-GT	U-GLU	0.080341	8.97E-14
B140	B215	r-GT	BMI	0.076983	9.14E-13
B140	B193	r-GT	K	-0.07688	9.80E-13
B140	B221	r-GT	Hei	0.064992	1.66E-09
B140	B222	r-GT	NS	0.061405	1.24E-08
B140	B176	r-GT	Ucr	0.05717	1.15E-07
B140	B199	r-GT	Mg	-0.05705	1.22E-07

B140	B197	r-GT	AG	0.056368	1.73E-07
B141	B153	AST/ALT	Glu	-0.11327	0
B141	B163	AST/ALT	SG	-0.11846	0
B141	B189	AST/ALT	eGFB	-0.15906	0
B141	B191	AST/ALT	Urea	0.103493	0
B141	B206	AST/ALT	Waist	-0.14821	0
B141	B207	AST/ALT	WHR	-0.10625	0
B141	B210	AST/ALT	Hip	-0.11328	0
B141	B215	AST/ALT	BMI	-0.1385	0
B141	B216	AST/ALT	BMI	-0.2135	0
B141	B218	AST/ALT	DBP	-0.13248	0
B141	B221	AST/ALT	Hei	-0.18679	0
B141	B222	AST/ALT	NS	-0.12504	0
B141	B209	AST/ALT	Smoking	-0.08424	5.33E-15
B141	B170	AST/ALT	U-GLU	-0.07501	3.43E-12
B141	B204	AST/ALT	Drinking	-0.06936	1.24E-10
B141	B176	AST/ALT	Ucr	-0.05393	5.73E-07
B141	B161	AST/ALT	LEU	0.053676	6.47E-07
B142	B143	25-(OH)D	β-CTx	-0.15428	0
B142	B144	25-(OH)D	Total-PINP	-0.12927	0
B142	B151	25-(OH)D	Anti-CCP	-0.13191	0
B142	B176	25-(OH)D	Ucr	-0.07351	9.14E-12
B143	B144	β-CTx	Total-PINP	0.696225	0
B143	B145	β-CTx	BGP	0.62655	0
B143	B151	β-CTx	Anti-CCP	0.275707	0
B143	B150	β-CTx	ALP	0.088187	2.22E-16
B143	B201	β-CTx	e-habits	-0.06803	2.79E-10
B144	B145	Total-PINP	BGP	0.63851	0
B144	B151	Total-PINP	Anti-CCP	0.246128	0
B144	B201	Total-PINP	e-habits	-0.08176	3.26E-14
B144	B150	Total-PINP	ALP	0.078993	2.30E-13
B144	B190	Total-PINP	Cre	0.061521	1.16E-08
B144	B176	Total-PINP	Ucr	0.053811	6.07E-07
B145	B201	BGP	e-habits	-0.10991	0
B145	B176	BGP	Ucr	0.076614	1.17E-12
B145	B150	BGP	ALP	0.075	3.44E-12
B145	B183	BGP	PTA	-0.06326	4.44E-09
B145	B203	BGP	DH	0.058455	5.95E-08
B145	B207	BGP	WHR	-0.05528	2.97E-07
B145	B175	BGP	ALB-U	-0.05329	7.78E-07
B146	B189	CysC	eGFB	-0.2364	0
B146	B190	CysC	Cre	0.360512	0
B146	B191	CysC	Urea	0.271955	0
B146	B192	CysC	UA	0.106225	0

B146	B198	CysC	P	0.163459	0
B146	B200	CysC	OP	0.192858	0
B146	B174	CysC	Urea/Cr	-0.08355	8.88E-15
B146	B196	CysC	TCO2	-0.08133	4.44E-14
B146	B188	CysC	Ca	-0.06751	3.81E-10
B146	B218	CysC	DBP	-0.05997	2.68E-08
B148	B149	Anti_TPO	TGAb	0.520305	0
B149	B221	TGAb	Hei	-0.07261	1.63E-11
B15	B100	CD3-CD19+(B cell)	anti-Ro52	0.106232	0
B15	B101	CD3-CD19+(B cell)	anti-Sc70	0.46939	0
B15	B103	CD3-CD19+(B cell)	anti-SSA/Ro	0.128249	0
B15	B104	CD3-CD19+(B cell)	anti-SSB	0.46939	0
B15	B107	CD3-CD19+(B cell)	AnuA	0.471646	0
B15	B109	CD3-CD19+(B cell)	AMA-M2	0.177977	0
B15	B110	CD3-CD19+(B cell)	AHA	-0.10072	0
B15	B115	CD3-CD19+(B cell)	TSH TT3 TT4(TT3)	0.09607	0
B15	B124	CD3-CD19+(B cell)	ACTH	-0.11149	0
B15	B136	CD3-CD19+(B cell)	5'NT	-0.10737	0
B15	B151	CD3-CD19+(B cell)	Anti-CCP	0.09029	0
B15	B158	CD3-CD19+(B cell)	IgM	0.117072	0
B15	B16	CD3-CD19+(B cell)	CD3+T	0.130458	0
B15	B18	CD3-CD19+(B cell)	CD3+CD4+helper(inducer)T	0.25555	0
B15	B181	CD3-CD19+(B cell)	APTT	0.12944	0
B15	B184	CD3-CD19+(B cell)	INR	0.111378	0
B15	B186	CD3-CD19+(B cell)	PFC	-0.13881	0
B15	B19	CD3-CD19+(B cell)	CD3+CD4+/CD3+CD8+	0.123482	0
B15	B194	CD3-CD19+(B cell)	Cl	0.139788	0
B15	B195	CD3-CD19+(B cell)	Na	0.090821	0
B15	B199	CD3-CD19+(B cell)	Mg	0.168775	0
B15	B20	CD3-CD19+(B cell)	CD3+CD4+CD8+	-0.1182	0
B15	B200	CD3-CD19+(B cell)	OP	-0.09499	0
B15	B21	CD3-CD19+(B cell)	CD3+CD4-CD8-	0.203413	0
B15	B22	CD3-CD19+(B cell)	CD3+CD8+(T)	-0.21084	0
B15	B225	CD3-CD19+(B cell)	hPBG	-0.09839	0
B15	B23	CD3-CD19+(B cell)	CD3-CD16+CD56+(NK)	-0.43942	0
B15	B28	CD3-CD19+(B cell)	PRL	0.136065	0
B15	B29	CD3-CD19+(B cell)	E2	0.106428	0
B15	B30	CD3-CD19+(B cell)	LH	-0.20844	0
B15	B31	CD3-CD19+(B cell)	FSH	-0.16554	0
B15	B33	CD3-CD19+(B cell)	Prog	0.15222	0
B15	B79	CD3-CD19+(B cell)	hb V -dna	0.176109	0
B15	B92	CD3-CD19+(B cell)	ALDH2-Glu504Glu	-0.18591	0
B15	B93	CD3-CD19+(B cell)	ALDH2-Glu504Lys	0.178613	0
B15	B94	CD3-CD19+(B cell)	ALDH2-Lys504Lys	0.208787	0

B15	B95	CD3-CD19+(B cell)	anti-cenpb	-0.15416	0
B15	B96	CD3-CD19+(B cell)	dsDNA (IIF)	0.146863	0
B15	B97	CD3-CD19+(B cell)	dsDNA	-0.1361	0
B15	B98	CD3-CD19+(B cell)	anti-Jo-1	-0.13175	0
B15	B99	CD3-CD19+(B cell)	anti-PM-Scl	0.46939	0
B15	B197	CD3-CD19+(B cell)	AG	-0.0829	1.42E-14
B15	B119	CD3-CD19+(B cell)	Hcv-rna	0.079959	1.17E-13
B15	B123	CD3-CD19+(B cell)	hs-CRP1	-0.07991	1.21E-13
B15	B139	CD3-CD19+(B cell)	ADA	-0.07843	3.39E-13
B15	B17	CD3-CD19+(B cell)	CD3+CD16+CD56+NK	0.078146	4.13E-13
B15	B125	CD3-CD19+(B cell)	Cor	-0.06098	1.56E-08
B15	B157	CD3-CD19+(B cell)	IgG	0.060603	1.91E-08
B15	B113	CD3-CD19+(B cell)	FT3	0.059844	2.87E-08
B15	B122	CD3-CD19+(B cell)	C4	0.058826	4.90E-08
B15	B156	CD3-CD19+(B cell)	IgE	-0.05727	1.10E-07
B15	B179	CD3-CD19+(B cell)	α1-MG	-0.05573	2.37E-07
B15	B177	CD3-CD19+(B cell)	ACR	-0.05566	2.46E-07
B15	B178	CD3-CD19+(B cell)	Ig-U	-0.05456	4.22E-07
B15	B134	CD3-CD19+(B cell)	CHE	-0.05438	4.61E-07
B150	B186	ALP	PFC	0.105022	0
B150	B200	ALP	OP	0.110871	0
B150	B153	ALP	Glu	0.080426	8.44E-14
B150	B155	ALP	IgA	0.080022	1.12E-13
B150	B194	ALP	Cl	-0.06153	1.16E-08
B150	B182	ALP	TT	0.0536	6.71E-07
B150	B199	ALP	Mg	-0.05346	7.18E-07
B151	B152	Anti-CCP	ACA-Ig(G-A-M)	-0.13705	0
B151	B183	Anti-CCP	PTA	-0.08597	1.33E-15
B151	B185	Anti-CCP	PT	0.069497	1.14E-10
B152	B183	ACA-Ig(G-A-M)	PTA	0.060701	1.81E-08
B152	B182	ACA-Ig(G-A-M)	TT	-0.05356	6.86E-07
B153	B160	Glu	PH	-0.08957	0
B153	B163	Glu	SG	0.218745	0
B153	B166	Glu	Pro	0.108253	0
B153	B170	Glu	U-GLU	0.538424	0
B153	B172	Glu	KB	0.131358	0
B153	B174	Glu	Urea/Cr	0.120708	0
B153	B194	Glu	Cl	-0.09555	0
B153	B195	Glu	Na	-0.0936	0
B153	B206	Glu	Waist	0.104389	0
B153	B207	Glu	WHR	0.097139	0
B153	B218	Glu	DBP	0.101749	0
B153	B220	Glu	SBP	0.108417	0
B153	B192	Glu	UA	-0.08673	8.88E-16

B153	B216	Glu	BMI	0.084777	3.55E-15
B153	B215	Glu	BMI	0.079311	1.85E-13
B153	B225	Glu	hPBG	0.073701	8.06E-12
B153	B188	Glu	Ca	0.069563	1.10E-10
B153	B204	Glu	Drinking	0.064981	1.67E-09
B153	B200	Glu	OP	0.058725	5.17E-08
B153	B210	Glu	Hip	0.058171	6.89E-08
B154	B183	RF	PTA	0.061697	1.06E-08
B155	B157	IgA	IgG	0.416146	0
B155	B182	IgA	TT	0.09156	0
B155	B184	IgA	INR	-0.10921	0
B155	B194	IgA	Cl	-0.08531	2.44E-15
B155	B195	IgA	Na	-0.07843	3.39E-13
B155	B185	IgA	PT	-0.07775	5.44E-13
B155	B186	IgA	PFC	0.06453	2.17E-09
B155	B197	IgA	AG	0.05484	3.68E-07
B156	B199	IgE	Mg	-0.07509	3.23E-12
B156	B183	IgE	PTA	0.069132	1.43E-10
B156	B185	IgE	PT	0.066567	6.65E-10
B156	B184	IgE	INR	0.055061	3.31E-07
B157	B200	IgG	OP	-0.12423	0
B157	B195	IgG	Na	-0.08065	7.19E-14
B157	B199	IgG	Mg	0.069631	1.05E-10
B157	B158	IgG	IgM	0.068603	1.97E-10
B157	B180	IgG	Tf	-0.06559	1.17E-09
B157	B178	IgG	Ig-U	-0.05891	4.69E-08
B157	B212	IgG	IOP-L	-0.05672	1.45E-07
B157	B184	IgG	INR	0.055664	2.46E-07
B157	B204	IgG	Drinking	-0.05442	4.52E-07
B158	B198	IgM	P	0.104742	0
B158	B199	IgM	Mg	0.136894	0
B158	B200	IgM	OP	-0.09107	0
B158	B181	IgM	APTT	0.075363	2.71E-12
B158	B186	IgM	PFC	0.073672	8.21E-12
B159	B181	β2-MG	APTT	0.148057	0
B159	B184	β2-MG	INR	0.153074	0
B159	B186	β2-MG	PFC	-0.09161	0
B159	B185	β2-MG	PT	0.08688	8.88E-16
B159	B183	β2-MG	PTA	0.056872	1.34E-07
B16	B101	CD3+T	anti-Sc70	0.113431	0
B16	B104	CD3+T	anti-SSB	0.113431	0
B16	B107	CD3+T	AnuA	0.113983	0
B16	B110	CD3+T	AHA	-0.26064	0
B16	B122	CD3+T	C4	0.125872	0

B16	B137	CD3+T	AFU	-0.09521	0
B16	B139	CD3+T	ADA	-0.09932	0
B16	B17	CD3+T	CD3+CD16+CD56+NK	0.229289	0
B16	B18	CD3+T	CD3+CD4+helper(inducer)T	0.463219	0
B16	B194	CD3+T	Cl	0.092997	0
B16	B21	CD3+T	CD3+CD4-CD8-	0.162433	0
B16	B22	CD3+T	CD3+CD8+(T)	0.429998	0
B16	B23	CD3+T	CD3-CD16+CD56+(NK)	-0.8729	0
B16	B28	CD3+T	PRL	0.091472	0
B16	B96	CD3+T	dsDNA (IIF)	0.126874	0
B16	B97	CD3+T	dsDNA	-0.2841	0
B16	B98	CD3+T	anti-Jo-1	0.14744	0
B16	B99	CD3+T	anti-PM-Scl	0.113431	0
B16	B180	CD3+T	Tf	-0.08797	4.44E-16
B16	B158	CD3+T	IgM	0.085217	2.66E-15
B16	B33	CD3+T	Prog	0.084639	4.00E-15
B16	B183	CD3+T	PTA	-0.08389	6.88E-15
B16	B195	CD3+T	Na	0.076762	1.06E-12
B16	B54	CD3+T	ESR	-0.0765	1.27E-12
B16	B199	CD3+T	Mg	0.074087	6.27E-12
B16	B200	CD3+T	OP	-0.0697	1.01E-10
B16	B175	CD3+T	ALB-U	-0.06909	1.47E-10
B16	B69	CD3+T	PV	-0.06843	2.19E-10
B16	B30	CD3+T	LH	-0.06748	3.88E-10
B16	B31	CD3+T	FSH	-0.0668	5.81E-10
B16	B178	CD3+T	Ig-U	-0.06525	1.44E-09
B16	B121	CD3+T	C3	0.061514	1.17E-08
B16	B29	CD3+T	E2	0.060463	2.06E-08
B16	B119	CD3+T	Hcv-rna	0.05895	4.59E-08
B16	B186	CD3+T	PFC	-0.05649	1.63E-07
B16	B136	CD3+T	5'NT	0.052897	9.38E-07
B160	B163	PH	SG	-0.16413	0
B160	B191	PH	Urea	-0.1198	0
B160	B192	PH	UA	-0.13197	0
B160	B176	PH	Ucr	-0.07849	3.27E-13
B160	B220	PH	SBP	0.068505	2.09E-10
B160	B174	PH	Urea/Cr	-0.06208	8.57E-09
B160	B218	PH	DBP	0.059893	2.79E-08
B161	B173	LEU	NTT	0.213174	0
B161	B174	LEU	Urea/Cr	0.099319	0
B161	B216	LEU	BMI	-0.09033	0
B161	B221	LEU	Hei	-0.17237	0
B161	B222	LEU	NS	-0.08426	5.33E-15
B161	B209	LEU	Smoking	-0.06893	1.62E-10

B161	B204	LEU	Drinking	-0.05952	3.40E-08
B161	B218	LEU	DBP	-0.05585	2.23E-07
B163	B166	SG	Pro	0.254502	0
B163	B170	SG	U-GLU	0.282109	0
B163	B172	SG	KB	0.143978	0
B163	B174	SG	Urea/Cr	0.090974	0
B163	B176	SG	Ucr	0.288752	0
B163	B189	SG	eGFB	0.111127	0
B163	B191	SG	Urea	0.089572	0
B163	B204	SG	Drinking	0.128654	0
B163	B206	SG	Waist	0.163896	0
B163	B207	SG	WHR	0.109238	0
B163	B209	SG	Smoking	0.176425	0
B163	B210	SG	Hip	0.128076	0
B163	B215	SG	BMI	0.121051	0
B163	B216	SG	BMI	0.252181	0
B163	B218	SG	DBP	0.10627	0
B163	B221	SG	Hei	0.266263	0
B163	B222	SG	NS	0.183947	0
B163	B220	SG	SBP	-0.07786	5.03E-13
B163	B173	SG	NTT	-0.06804	2.77E-10
B163	B179	SG	α1-MG	0.061701	1.05E-08
B163	B188	SG	Ca	-0.0577	8.80E-08
B163	B201	SG	e-habits	-0.05683	1.37E-07
B164	B178	COM	Ig-U	0.078247	3.86E-13
B166	B170	Pro	U-GLU	0.149345	0
B166	B172	Pro	KB	0.103611	0
B166	B176	Pro	Ucr	0.111	0
B166	B189	Pro	eGFB	-0.10901	0
B166	B190	Pro	Cre	0.123254	0
B166	B191	Pro	Urea	0.116275	0
B166	B207	Pro	WHR	0.072996	1.27E-11
B166	B206	Pro	Waist	0.06661	6.49E-10
B166	B175	Pro	ALB-U	0.059715	3.07E-08
B166	B177	Pro	ACR	0.059607	3.25E-08
B166	B216	Pro	BMI	0.057565	9.42E-08
B166	B188	Pro	Ca	-0.05592	2.16E-07
B166	B221	Pro	Hei	0.054663	4.02E-07
B166	B222	Pro	NS	0.053221	8.04E-07
B169	B203	U-CRY	DH	0.065392	1.32E-09
B17	B101	CD3+CD16+CD56+NK	anti-Sc70	0.208531	0
B17	B104	CD3+CD16+CD56+NK	anti-SSB	0.208531	0
B17	B107	CD3+CD16+CD56+NK	AnuA	0.209532	0
B17	B108	CD3+CD16+CD56+NK	Anti-Cyclin	-0.15999	0

B17	B137	CD3+CD16+CD56+NK	AFU	-0.11764	0
B17	B145	CD3+CD16+CD56+NK	BGP	0.102583	0
B17	B151	CD3+CD16+CD56+NK	Anti-CCP	0.15066	0
B17	B175	CD3+CD16+CD56+NK	ALB-U	-0.11732	0
B17	B178	CD3+CD16+CD56+NK	Ig-U	-0.10714	0
B17	B180	CD3+CD16+CD56+NK	Tf	-0.14121	0
B17	B183	CD3+CD16+CD56+NK	PTA	-0.20922	0
B17	B185	CD3+CD16+CD56+NK	PT	0.092702	0
B17	B19	CD3+CD16+CD56+NK	CD3+CD4+/CD3+CD8+	-0.16098	0
B17	B21	CD3+CD16+CD56+NK	CD3+CD4-CD8-	0.352404	0
B17	B22	CD3+CD16+CD56+NK	CD3+CD8+(T)	0.173597	0
B17	B23	CD3+CD16+CD56+NK	CD3-CD16+CD56+(NK)	-0.18501	0
B17	B96	CD3+CD16+CD56+NK	dsDNA (IIF)	0.126016	0
B17	B99	CD3+CD16+CD56+NK	anti-PM-Scl	0.208531	0
B17	B195	CD3+CD16+CD56+NK	Na	0.079426	1.70E-13
B17	B109	CD3+CD16+CD56+NK	AMA-M2	0.079056	2.20E-13
B17	B20	CD3+CD16+CD56+NK	CD3+CD4+CD8+	0.077495	6.45E-13
B17	B138	CD3+CD16+CD56+NK	PAB	-0.07598	1.80E-12
B17	B184	CD3+CD16+CD56+NK	INR	0.074858	3.78E-12
B17	B97	CD3+CD16+CD56+NK	dsDNA	-0.06948	1.16E-10
B17	B158	CD3+CD16+CD56+NK	IgM	0.067803	3.20E-10
B17	B199	CD3+CD16+CD56+NK	Mg	0.06744	3.97E-10
B17	B194	CD3+CD16+CD56+NK	Cl	0.066375	7.45E-10
B17	B34	CD3+CD16+CD56+NK	TK	0.065704	1.10E-09
B17	B182	CD3+CD16+CD56+NK	TT	0.063789	3.30E-09
B17	B144	CD3+CD16+CD56+NK	Total-PINP	0.063721	3.43E-09
B17	B136	CD3+CD16+CD56+NK	5'NT	0.058202	6.78E-08
B17	B69	CD3+CD16+CD56+NK	PV	-0.05796	7.68E-08
B17	B57	CD3+CD16+CD56+NK	RBCAI	0.057363	1.04E-07
B170	B172	U-GLU	KB	0.147134	0
B170	B192	U-GLU	UA	-0.11749	0
B170	B204	U-GLU	Drinking	0.109117	0
B170	B209	U-GLU	Smoking	0.097708	0
B170	B216	U-GLU	BMI	0.090597	0
B170	B218	U-GLU	DBP	0.102135	0
B170	B221	U-GLU	Hei	0.111835	0
B170	B194	U-GLU	Cl	-0.08103	5.51E-14
B170	B207	U-GLU	WHR	0.080547	7.75E-14
B170	B206	U-GLU	Waist	0.076262	1.49E-12
B170	B222	U-GLU	NS	0.073803	7.54E-12
B170	B225	U-GLU	hPBG	0.068629	1.94E-10
B170	B200	U-GLU	OP	0.067096	4.87E-10
B170	B220	U-GLU	SBP	0.06175	1.03E-08
B170	B176	U-GLU	Ucr	0.059835	2.88E-08

B170	B174	U-GLU	Urea/Cr	0.057637	9.07E-08
B170	B189	U-GLU	eGFB	0.053327	7.65E-07
B170	B195	U-GLU	Na	-0.05282	9.73E-07
B173	B221	NTT	Hei	-0.12945	0
B173	B216	NTT	BMI	-0.07445	4.93E-12
B173	B174	NTT	Urea/Cr	0.066804	5.79E-10
B173	B222	NTT	NS	-0.05832	6.39E-08
B173	B204	NTT	Drinking	-0.05462	4.11E-07
B174	B189	Urea/Cr	eGFB	0.224326	0
B174	B190	Urea/Cr	Cre	-0.33012	0
B174	B191	Urea/Cr	Urea	0.323161	0
B174	B192	Urea/Cr	UA	-0.18102	0
B174	B204	Urea/Cr	Drinking	-0.09799	0
B174	B209	Urea/Cr	Smoking	-0.11222	0
B174	B216	Urea/Cr	BMI	-0.18585	0
B174	B221	Urea/Cr	Hei	-0.28103	0
B174	B222	Urea/Cr	NS	-0.15311	0
B174	B206	Urea/Cr	Waist	-0.08563	1.78E-15
B174	B200	Urea/Cr	OP	0.076389	1.37E-12
B174	B218	Urea/Cr	DBP	-0.07629	1.46E-12
B174	B210	Urea/Cr	Hip	-0.07009	7.94E-11
B174	B207	Urea/Cr	WHR	-0.05826	6.59E-08
B175	B177	ALB-U	ACR	0.848692	0
B175	B178	ALB-U	Ig-U	0.633189	0
B175	B179	ALB-U	α1-MG	0.298483	0
B175	B180	ALB-U	Tf	0.714224	0
B175	B189	ALB-U	eGFB	-0.06798	2.88E-10
B175	B190	ALB-U	Cre	0.057694	8.81E-08
B176	B177	Ucr	ACR	-0.09286	0
B176	B179	Ucr	α1-MG	0.143693	0
B176	B221	Ucr	Hei	0.092519	0
B176	B216	Ucr	BMI	0.088104	4.44E-16
B176	B209	Ucr	Smoking	0.066087	8.82E-10
B176	B222	Ucr	NS	0.062748	5.91E-09
B176	B201	Ucr	e-habits	-0.05361	6.69E-07
B177	B178	ACR	Ig-U	0.534878	0
B177	B179	ACR	α1-MG	0.284178	0
B177	B180	ACR	Tf	0.558802	0
B177	B220	ACR	SBP	0.080181	1.00E-13
B177	B186	ACR	PFC	0.075358	2.72E-12
B177	B189	ACR	eGFB	-0.07317	1.14E-11
B177	B190	ACR	Cre	0.069542	1.11E-10
B177	B191	ACR	Urea	0.060842	1.68E-08
B178	B179	Ig-U	α1-MG	0.53678	0

B178	B180	Ig-U	Tf	0.970606	0
B178	B200	Ig-U	OP	0.081967	2.80E-14
B178	B186	Ig-U	PFC	0.076338	1.41E-12
B178	B184	Ig-U	INR	-0.05516	3.16E-07
B179	B180	α1-MG	Tf	0.478549	0
B179	B200	α1-MG	OP	0.072641	1.60E-11
B179	B186	α1-MG	PFC	0.062342	7.41E-09
B18	B110	CD3+CD4+helper(inducer)T	AHA	-0.10595	0
B18	B122	CD3+CD4+helper(inducer)T	C4	0.148445	0
B18	B124	CD3+CD4+helper(inducer)T	ACTH	0.140133	0
B18	B139	CD3+CD4+helper(inducer)T	ADA	-0.0922	0
B18	B156	CD3+CD4+helper(inducer)T	IgE	-0.13859	0
B18	B19	CD3+CD4+helper(inducer)T	CD3+CD4+/CD3+CD8+	0.733839	0
B18	B22	CD3+CD4+helper(inducer)T	CD3+CD8+(T)	-0.51924	0
B18	B23	CD3+CD4+helper(inducer)T	CD3-CD16+CD56+(NK)	-0.50781	0
B18	B28	CD3+CD4+helper(inducer)T	PRL	0.253586	0
B18	B29	CD3+CD4+helper(inducer)T	E2	0.192524	0
B18	B30	CD3+CD4+helper(inducer)T	LH	-0.35772	0
B18	B31	CD3+CD4+helper(inducer)T	FSH	-0.29106	0
B18	B33	CD3+CD4+helper(inducer)T	Prog	0.277885	0
B18	B92	CD3+CD4+helper(inducer)T	ALDH2-Glu504Glu	0.129659	0
B18	B93	CD3+CD4+helper(inducer)T	ALDH2-Glu504Lys	-0.12926	0
B18	B138	CD3+CD4+helper(inducer)T	PAB	0.086837	8.88E-16
B18	B97	CD3+CD4+helper(inducer)T	dsDNA	-0.0831	1.24E-14
B18	B158	CD3+CD4+helper(inducer)T	IgM	0.081977	2.80E-14
B18	B136	CD3+CD4+helper(inducer)T	5'NT	-0.08154	3.82E-14
B18	B125	CD3+CD4+helper(inducer)T	Cor	0.078598	3.03E-13
B18	B96	CD3+CD4+helper(inducer)T	dsDNA (IIF)	0.077981	4.63E-13
B18	B21	CD3+CD4+helper(inducer)T	CD3+CD4-CD8-	-0.07692	9.54E-13
B18	B94	CD3+CD4+helper(inducer)T	ALDH2-Lys504Lys	-0.07567	2.21E-12
B18	B200	CD3+CD4+helper(inducer)T	OP	-0.07463	4.40E-12
B18	B176	CD3+CD4+helper(inducer)T	Ucr	0.074609	4.45E-12
B18	B197	CD3+CD4+helper(inducer)T	AG	-0.07403	6.49E-12
B18	B157	CD3+CD4+helper(inducer)T	IgG	-0.06592	9.72E-10
B18	B24	CD3+CD4+helper(inducer)T	αHBDH	-0.06296	5.25E-09
B18	B186	CD3+CD4+helper(inducer)T	PFC	-0.06036	2.18E-08
B18	B181	CD3+CD4+helper(inducer)T	APTT	0.056567	1.56E-07
B18	B26	CD3+CD4+helper(inducer)T	CKMB	-0.05632	1.77E-07
B18	B49	CD3+CD4+helper(inducer)T	baso%	-0.0563	1.79E-07
B18	B178	CD3+CD4+helper(inducer)T	Ig-U	-0.05458	4.18E-07
B180	B200	Tf	OP	0.071903	2.55E-11
B180	B186	Tf	PFC	0.06364	3.59E-09
B181	B184	APTT	INR	0.532547	0
B181	B185	APTT	PT	0.467061	0

B181	B194	APTT	Cl	0.102522	0
B181	B199	APTT	Mg	0.108974	0
B181	B200	APTT	OP	-0.09177	0
B181	B197	APTT	AG	-0.0793	1.85E-13
B181	B212	APTT	IOP-L	-0.07306	1.22E-11
B181	B211	APTT	IOP-R	-0.06101	1.53E-08
B182	B183	TT	PTA	-0.16684	0
B182	B184	TT	INR	-0.15454	0
B182	B195	TT	Na	0.055757	2.34E-07
B182	B200	TT	OP	0.053177	8.22E-07
B183	B184	PTA	INR	0.157789	0
B183	B186	PTA	PFC	-0.1174	0
B183	B197	PTA	AG	-0.08912	0
B183	B195	PTA	Na	-0.05808	7.23E-08
B183	B200	PTA	OP	-0.05552	2.64E-07
B184	B185	INR	PT	0.81983	0
B184	B186	INR	PFC	-0.25083	0
B184	B194	INR	Cl	0.118704	0
B184	B196	INR	TCO2	0.096339	0
B184	B197	INR	AG	-0.14608	0
B184	B199	INR	Mg	0.098272	0
B184	B200	INR	OP	-0.09873	0
B184	B193	INR	K	0.076738	1.08E-12
B185	B193	PT	K	0.091699	0
B185	B197	PT	AG	-0.09731	0
B185	B199	PT	Mg	0.077453	6.64E-13
B185	B196	PT	TCO2	0.073127	1.17E-11
B185	B194	PT	Cl	0.070112	7.84E-11
B185	B212	PT	IOP-L	-0.06101	1.54E-08
B185	B200	PT	OP	-0.05685	1.36E-07
B186	B225	PFC	hPBG	0.078761	2.70E-13
B186	B194	PFC	Cl	-0.07223	2.08E-11
B186	B197	PFC	AG	0.064421	2.31E-09
B186	B200	PFC	OP	0.062238	7.85E-09
B188	B192	Ca	UA	0.102715	0
B188	B190	Ca	Cre	-0.08703	6.66E-16
B188	B193	Ca	K	0.055522	2.63E-07
B188	B191	Ca	Urea	-0.05363	6.63E-07
B189	B190	eGFB	Cre	-0.57109	0
B189	B191	eGFB	Urea	-0.54279	0
B189	B192	eGFB	UA	-0.28922	0
B189	B204	eGFB	Drinking	0.113189	0
B189	B209	eGFB	Smoking	0.093262	0
B189	B210	eGFB	Hip	0.091579	0

B189	B216	eGFB	BMI	0.138969	0
B189	B218	eGFB	DBP	0.19613	0
B189	B220	eGFB	SBP	-0.14107	0
B189	B221	eGFB	Hei	0.125996	0
B189	B215	eGFB	BMI	0.081324	4.44E-14
B189	B194	eGFB	Cl	-0.05901	4.46E-08
B189	B212	eGFB	IOP-L	0.056907	1.32E-07
B189	B207	eGFB	WHR	-0.05672	1.45E-07
B19	B101	CD3+CD4+/CD3+CD8+	anti-Sc70	-0.16701	0
B19	B104	CD3+CD4+/CD3+CD8+	anti-SSB	-0.16701	0
B19	B107	CD3+CD4+/CD3+CD8+	AnuA	-0.1678	0
B19	B122	CD3+CD4+/CD3+CD8+	C4	0.113103	0
B19	B124	CD3+CD4+/CD3+CD8+	ACTH	0.153856	0
B19	B125	CD3+CD4+/CD3+CD8+	Cor	0.102847	0
B19	B136	CD3+CD4+/CD3+CD8+	5'NT	-0.10729	0
B19	B156	CD3+CD4+/CD3+CD8+	IgE	-0.12966	0
B19	B157	CD3+CD4+/CD3+CD8+	IgG	-0.11142	0
B19	B195	CD3+CD4+/CD3+CD8+	Na	-0.09127	0
B19	B20	CD3+CD4+/CD3+CD8+	CD3+CD4+CD8+	-0.14085	0
B19	B22	CD3+CD4+/CD3+CD8+	CD3+CD8+(T)	-0.77694	0
B19	B23	CD3+CD4+/CD3+CD8+	CD3-CD16+CD56+(NK)	-0.11653	0
B19	B28	CD3+CD4+/CD3+CD8+	PRL	0.134673	0
B19	B29	CD3+CD4+/CD3+CD8+	E2	0.103039	0
B19	B30	CD3+CD4+/CD3+CD8+	LH	-0.19517	0
B19	B31	CD3+CD4+/CD3+CD8+	FSH	-0.15856	0
B19	B33	CD3+CD4+/CD3+CD8+	Prog	0.149439	0
B19	B92	CD3+CD4+/CD3+CD8+	ALDH2-Glu504Glu	0.132475	0
B19	B93	CD3+CD4+/CD3+CD8+	ALDH2-Glu504Lys	-0.13088	0
B19	B94	CD3+CD4+/CD3+CD8+	ALDH2-Lys504Lys	-0.09499	0
B19	B97	CD3+CD4+/CD3+CD8+	dsDNA	0.107598	0
B19	B99	CD3+CD4+/CD3+CD8+	anti-PM-Scl	-0.16701	0
B19	B158	CD3+CD4+/CD3+CD8+	IgM	0.082	2.75E-14
B19	B21	CD3+CD4+/CD3+CD8+	CD3+CD4-CD8-	-0.0801	1.06E-13
B19	B197	CD3+CD4+/CD3+CD8+	AG	-0.07827	3.81E-13
B19	B183	CD3+CD4+/CD3+CD8+	PTA	0.077332	7.21E-13
B19	B73	CD3+CD4+/CD3+CD8+	ApoA	0.073079	1.20E-11
B19	B185	CD3+CD4+/CD3+CD8+	PT	-0.06846	2.15E-10
B19	B109	CD3+CD4+/CD3+CD8+	AMA-M2	-0.06319	4.62E-09
B19	B138	CD3+CD4+/CD3+CD8+	PAB	0.054276	4.85E-07
B19	B24	CD3+CD4+/CD3+CD8+	αHBDH	-0.05349	7.09E-07
B190	B191	Cre	Urea	0.646286	0
B190	B192	Cre	UA	0.26022	0
B190	B198	Cre	P	0.097843	0
B190	B221	Cre	Hei	0.110656	0

B190	B222	Cre	NS	0.077279	7.47E-13
B190	B200	Cre	OP	0.070613	5.74E-11
B190	B196	Cre	TCO2	-0.06469	1.97E-09
B190	B207	Cre	WHR	0.064554	2.14E-09
B190	B216	Cre	BMI	0.062055	8.68E-09
B190	B206	Cre	Waist	0.053887	5.85E-07
B191	B192	Urea	UA	0.270509	0
B191	B200	Urea	OP	0.10083	0
B191	B218	Urea	DBP	-0.12461	0
B191	B198	Urea	P	0.075653	2.23E-12
B191	B204	Urea	Drinking	-0.06806	2.73E-10
B191	B220	Urea	SBP	0.063843	3.20E-09
B191	B209	Urea	Smoking	-0.053	8.96E-07
B192	B206	UA	Waist	0.181067	0
B192	B207	UA	WHR	0.147958	0
B192	B210	UA	Hip	0.11949	0
B192	B215	UA	BMI	0.136529	0
B192	B216	UA	BMI	0.195821	0
B192	B221	UA	Hei	0.156525	0
B192	B222	UA	NS	0.130143	0
B192	B204	UA	Drinking	0.065596	1.17E-09
B193	B195	K	Na	-0.13299	0
B193	B197	K	AG	-0.12331	0
B193	B199	K	Mg	0.1526	0
B193	B198	K	P	0.070292	7.01E-11
B193	B200	K	OP	-0.06913	1.44E-10
B194	B195	Cl	Na	0.356668	0
B194	B196	Cl	TCO2	-0.12645	0
B194	B197	Cl	AG	-0.40326	0
B194	B199	Cl	Mg	0.17413	0
B195	B196	Na	TCO2	0.143094	0
B195	B197	Na	AG	0.269914	0
B195	B200	Na	OP	0.551638	0
B195	B199	Na	Mg	0.0876	4.44E-16
B196	B197	TCO2	AG	-0.63687	0
B197	B199	AG	Mg	-0.09042	0
B197	B200	AG	OP	0.343288	0
B199	B200	Mg	OP	-0.2002	0
B199	B216	Mg	BMI	-0.06336	4.20E-09
B199	B204	Mg	Drinking	-0.06252	6.70E-09
B199	B221	Mg	Hei	-0.05802	7.45E-08
B199	B225	Mg	hPBG	-0.05283	9.67E-07
B2	B126	Age	Alb	-0.25925	0
B2	B127	Age	A-G ratio	-0.22298	0

B2	B128	Age	ALT	-0.21767	0
B2	B130	Age	GLB	0.158168	0
B2	B134	Age	CHE	-0.10058	0
B2	B140	Age	r-GT	-0.13694	0
B2	B141	Age	AST/ALT	0.275056	0
B2	B146	Age	CysC	0.097924	0
B2	B163	Age	SG	-0.16844	0
B2	B170	Age	U-GLU	-0.09517	0
B2	B173	Age	NTT	0.091594	0
B2	B176	Age	Ucr	-0.09728	0
B2	B189	Age	eGFB	-0.46944	0
B2	B190	Age	Cre	0.100894	0
B2	B191	Age	Urea	0.197271	0
B2	B199	Age	Mg	0.089962	0
B2	B203	Age	DH	-0.09278	0
B2	B204	Age	Drinking	-0.23178	0
B2	B206	Age	Waist	-0.12803	0
B2	B209	Age	Smoking	-0.22728	0
B2	B210	Age	Hip	-0.16866	0
B2	B211	Age	IOP-R	-0.09398	0
B2	B212	Age	IOP-L	-0.09965	0
B2	B215	Age	BMI	-0.23185	0
B2	B216	Age	BMI	-0.38829	0
B2	B218	Age	DBP	-0.39829	0
B2	B219	Age	OT	0.114069	0
B2	B220	Age	SBP	0.18955	0
B2	B221	Age	Hei	-0.34285	0
B2	B222	Age	NS	-0.17063	0
B2	B41	Age	MON%	0.155147	0
B2	B42	Age	MON	0.101007	0
B2	B43	Age	MCV	0.13543	0
B2	B45	Age	MCHC	-0.18903	0
B2	B46	Age	RBC Count	-0.31174	0
B2	B47	Age	RDW	0.247659	0
B2	B51	Age	Hb	-0.31442	0
B2	B71	Age	TG	-0.24335	0
B2	B72	Age	HDL-C	0.165495	0
B2	B74	Age	ApoA1	0.089655	0
B2	B101	Age	anti-Sc70	0.084269	5.33E-15
B2	B104	Age	anti-SSB	0.084269	5.33E-15
B2	B99	Age	anti-PM-Scl	0.084269	5.33E-15
B2	B107	Age	AnuA	0.083771	7.55E-15
B2	B27	Age	LDH	0.083061	1.27E-14
B2	B188	Age	Ca	-0.0827	1.64E-14

B2	B48	Age	LYM%	-0.08084	6.31E-14
B2	B138	Age	PAB	-0.07416	5.99E-12
B2	B113	Age	FT3	-0.07243	1.83E-11
B2	B24	Age	αHBDH	0.070473	6.26E-11
B2	B77	Age	TC	-0.07015	7.63E-11
B2	B94	Age	ALDH2-Lys504Lys	0.069855	9.18E-11
B2	B62	Age	WBHSRVI	-0.068	2.84E-10
B2	B227	Age	OD	0.067815	3.17E-10
B2	B118	Age	CEA1	0.067565	3.68E-10
B2	B184	Age	INR	0.067126	4.78E-10
B2	B66	Age	WBRSR(mPas_100)	-0.06291	5.40E-09
B2	B56	Age	IR	-0.06275	5.92E-09
B2	B67	Age	WBRSR(mPas_50)	-0.06227	7.69E-09
B2	B61	Age	WBHSRV	-0.06038	2.15E-08
B2	B205	Age	KDA	0.058969	4.55E-08
B2	B63	Age	WBRSR(mPas_200)	-0.05882	4.92E-08
B2	B131	Age	AST	-0.05801	7.48E-08
B2	B64	Age	WBRSR(mPas_5)	-0.05749	9.80E-08
B2	B79	Age	hb V -dna	0.057309	1.07E-07
B2	B153	Age	Glu	-0.05722	1.12E-07
B2	B185	Age	PT	0.057104	1.19E-07
B2	B161	Age	LEU	0.055945	2.13E-07
B2	B112	Age	FT4	-0.05491	3.56E-07
B2	B65	Age	WBRSR(mPas_1)	-0.0529	9.39E-07
B20	B151	CD3+CD4+CD8+	Anti-CCP	-0.11858	0
B20	B181	CD3+CD4+CD8+	APTT	0.133183	0
B20	B21	CD3+CD4+CD8+	CD3+CD4-CD8-	-0.14315	0
B20	B22	CD3+CD4+CD8+	CD3+CD8+(T)	0.160975	0
B20	B79	CD3+CD4+CD8+	hb V -dna	-0.17488	0
B20	B81	CD3+CD4+CD8+	TSGF	0.094201	0
B20	B196	CD3+CD4+CD8+	TCO2	-0.08851	2.22E-16
B20	B194	CD3+CD4+CD8+	Cl	0.08322	1.13E-14
B20	B74	CD3+CD4+CD8+	ApoA1	-0.07881	2.62E-13
B20	B119	CD3+CD4+CD8+	Hcv-rna	-0.07796	4.70E-13
B20	B137	CD3+CD4+CD8+	AFU	0.070056	8.11E-11
B20	B150	CD3+CD4+CD8+	ALP	0.068918	1.63E-10
B20	B138	CD3+CD4+CD8+	PAB	-0.06838	2.26E-10
B20	B107	CD3+CD4+CD8+	AnuA	-0.06729	4.34E-10
B20	B101	CD3+CD4+CD8+	anti-Sc70	-0.06697	5.24E-10
B20	B104	CD3+CD4+CD8+	anti-SSB	-0.06697	5.24E-10
B20	B99	CD3+CD4+CD8+	anti-PM-Scl	-0.06697	5.24E-10
B20	B75	CD3+CD4+CD8+	ApoA1/B	-0.0666	6.52E-10
B20	B200	CD3+CD4+CD8+	OP	0.065558	1.20E-09
B20	B182	CD3+CD4+CD8+	TT	-0.06463	2.05E-09

B20	B69	CD3+CD4+CD8+	PV	-0.05975	3.02E-08
B20	B55	CD3+CD4+CD8+	TK	0.058464	5.92E-08
B20	B56	CD3+CD4+CD8+	IR	0.056404	1.70E-07
B20	B94	CD3+CD4+CD8+	ALDH2-Lys504Lys	0.055681	2.43E-07
B201	B204	e-habits	Drinking	0.075318	2.79E-12
B202	B223	e-times	FH	-0.51925	0
B203	B209	DH	Smoking	0.079871	1.25E-13
B203	B216	DH	BMI	0.076689	1.11E-12
B203	B215	DH	BMI	0.06347	3.95E-09
B203	B204	DH	Drinking	0.058336	6.33E-08
B203	B210	DH	Hip	0.057032	1.24E-07
B203	B206	DH	Waist	0.055791	2.30E-07
B204	B206	Drinking	Waist	0.124616	0
B204	B209	Drinking	Smoking	0.368555	0
B204	B210	Drinking	Hip	0.097167	0
B204	B216	Drinking	BMI	0.212415	0
B204	B218	Drinking	DBP	0.118769	0
B204	B221	Drinking	Hei	0.235947	0
B204	B222	Drinking	NS	0.153695	0
B204	B215	Drinking	BMI	0.086909	6.66E-16
B204	B207	Drinking	WHR	0.085294	2.44E-15
B204	B220	Drinking	SBP	-0.08456	4.22E-15
B204	B219	Drinking	OT	-0.07302	1.25E-11
B205	B221	KDA	Hei	-0.07142	3.47E-11
B206	B207	Waist	WHR	0.716404	0
B206	B209	Waist	Smoking	0.097959	0
B206	B210	Waist	Hip	0.740675	0
B206	B215	Waist	BMI	0.791569	0
B206	B216	Waist	BMI	0.779129	0
B206	B218	Waist	DBP	0.11334	0
B206	B221	Waist	Hei	0.33044	0
B206	B222	Waist	NS	0.442615	0
B206	B219	Waist	OT	-0.06199	9.01E-09
B207	B215	WHR	BMI	0.401022	0
B207	B216	WHR	BMI	0.370225	0
B207	B221	WHR	Hei	0.126724	0
B207	B222	WHR	NS	0.250995	0
B207	B210	WHR	Hip	0.08536	2.22E-15
B207	B209	WHR	Smoking	0.083718	7.99E-15
B209	B216	Smoking	BMI	0.185295	0
B209	B220	Smoking	SBP	-0.12904	0
B209	B221	Smoking	Hei	0.262865	0
B209	B222	Smoking	NS	0.145435	0
B209	B219	Smoking	OT	-0.07308	1.20E-11

B209	B218	Smoking	DBP	0.063781	3.32E-09
B209	B210	Smoking	Hip	0.060131	2.46E-08
B21	B101	CD3+CD4-CD8-	anti-Sc70	0.125962	0
B21	B104	CD3+CD4-CD8-	anti-SSB	0.125962	0
B21	B107	CD3+CD4-CD8-	AnuA	0.126569	0
B21	B138	CD3+CD4-CD8-	PAB	-0.13664	0
B21	B151	CD3+CD4-CD8-	Anti-CCP	0.123623	0
B21	B183	CD3+CD4-CD8-	PTA	-0.11587	0
B21	B23	CD3+CD4-CD8-	CD3-CD16+CD56+(NK)	-0.22222	0
B21	B99	CD3+CD4-CD8-	anti-PM-Scl	0.125962	0
B21	B158	CD3+CD4-CD8-	IgM	0.082547	1.84E-14
B21	B180	CD3+CD4-CD8-	Tf	-0.07897	2.33E-13
B21	B30	CD3+CD4-CD8-	LH	-0.07614	1.61E-12
B21	B187	CD3+CD4-CD8-	NSE	0.068278	2.40E-10
B21	B22	CD3+CD4-CD8-	CD3+CD8+(T)	-0.06512	1.54E-09
B21	B94	CD3+CD4-CD8-	ALDH2-Lys504Lys	0.06376	3.35E-09
B21	B81	CD3+CD4-CD8-	TSGF	-0.06339	4.12E-09
B21	B31	CD3+CD4-CD8-	FSH	-0.06209	8.53E-09
B21	B178	CD3+CD4-CD8-	Ig-U	-0.06166	1.07E-08
B21	B175	CD3+CD4-CD8-	ALB-U	-0.06089	1.64E-08
B21	B194	CD3+CD4-CD8-	Cl	0.060262	2.30E-08
B21	B156	CD3+CD4-CD8-	IgE	-0.05747	9.88E-08
B21	B92	CD3+CD4-CD8-	ALDH2-Glu504Glu	-0.05456	4.23E-07
B21	B139	CD3+CD4-CD8-	ADA	-0.05419	5.06E-07
B210	B215	Hip	BMI	0.739314	0
B210	B216	Hip	BMI	0.751748	0
B210	B218	Hip	DBP	0.125106	0
B210	B221	Hip	Hei	0.350276	0
B210	B222	Hip	NS	0.400558	0
B211	B212	IOP-R	IOP-L	0.777675	0
B211	B218	IOP-R	DBP	0.074779	3.98E-12
B211	B215	IOP-R	BMI	0.053903	5.80E-07
B212	B218	IOP-L	DBP	0.077124	8.31E-13
B212	B215	IOP-L	BMI	0.058915	4.68E-08
B215	B216	BMI	BMI	0.776218	0
B215	B218	BMI	DBP	0.145497	0
B215	B221	BMI	Hei	0.095216	0
B215	B222	BMI	NS	0.381745	0
B215	B219	BMI	OT	-0.05588	2.20E-07
B216	B218	BMI	DBP	0.243321	0
B216	B219	BMI	OT	-0.09589	0
B216	B221	BMI	Hei	0.69376	0
B216	B222	BMI	NS	0.510631	0
B216	B220	BMI	SBP	-0.07711	8.42E-13

B218	B220	DBP	SBP	0.411964	0
B218	B221	DBP	Hei	0.216004	0
B218	B222	DBP	NS	0.118144	0
B218	B219	DBP	OT	-0.0767	1.11E-12
B219	B221	OT	Hei	-0.08451	4.44E-15
B219	B222	OT	NS	-0.0689	1.65E-10
B22	B101	CD3+CD8+(T)	anti-Sc70	0.098107	0
B22	B104	CD3+CD8+(T)	anti-SSB	0.098107	0
B22	B107	CD3+CD8+(T)	AnuA	0.098568	0
B22	B110	CD3+CD8+(T)	AHA	-0.11753	0
B22	B125	CD3+CD8+(T)	Cor	-0.09271	0
B22	B136	CD3+CD8+(T)	5'NT	0.146308	0
B22	B156	CD3+CD8+(T)	IgE	0.171964	0
B22	B183	CD3+CD8+(T)	PTA	-0.10051	0
B22	B195	CD3+CD8+(T)	Na	0.111958	0
B22	B23	CD3+CD8+(T)	CD3-CD16+CD56+(NK)	-0.22958	0
B22	B28	CD3+CD8+(T)	PRL	-0.1609	0
B22	B29	CD3+CD8+(T)	E2	-0.13224	0
B22	B30	CD3+CD8+(T)	LH	0.293783	0
B22	B31	CD3+CD8+(T)	FSH	0.226987	0
B22	B33	CD3+CD8+(T)	Prog	-0.19351	0
B22	B97	CD3+CD8+(T)	dsDNA	-0.15601	0
B22	B99	CD3+CD8+(T)	anti-PM-Scl	0.098107	0
B22	B93	CD3+CD8+(T)	ALDH2-Glu504Lys	0.086141	1.33E-15
B22	B92	CD3+CD8+(T)	ALDH2-Glu504Glu	-0.08236	2.13E-14
B22	B193	CD3+CD8+(T)	K	-0.08073	6.79E-14
B22	B122	CD3+CD8+(T)	C4	-0.07901	2.27E-13
B22	B185	CD3+CD8+(T)	PT	0.075433	2.58E-12
B22	B73	CD3+CD8+(T)	ApoA	-0.06667	6.26E-10
B22	B69	CD3+CD8+(T)	PV	-0.06594	9.59E-10
B22	B124	CD3+CD8+(T)	ACTH	-0.06342	4.06E-09
B22	B24	CD3+CD8+(T)	αHBDH	0.063367	4.19E-09
B22	B194	CD3+CD8+(T)	Cl	0.060035	2.59E-08
B22	B54	CD3+CD8+(T)	ESR	-0.05953	3.39E-08
B22	B197	CD3+CD8+(T)	AG	0.053551	6.87E-07
B22	B138	CD3+CD8+(T)	PAB	-0.05346	7.19E-07
B220	B221	SBP	Hei	-0.13631	0
B221	B222	Hei	NS	0.376237	0
B227	B228	OD	OS	0.072631	1.61E-11
B23	B101	CD3-CD16+CD56+(NK)	anti-Sc70	-0.10311	0
B23	B104	CD3-CD16+CD56+(NK)	anti-SSB	-0.10311	0
B23	B107	CD3-CD16+CD56+(NK)	AnuA	-0.10362	0
B23	B110	CD3-CD16+CD56+(NK)	AHA	0.306932	0
B23	B122	CD3-CD16+CD56+(NK)	C4	-0.13445	0

B23	B139	CD3-CD16+CD56+(NK)	ADA	0.100519	0
B23	B158	CD3-CD16+CD56+(NK)	IgM	-0.09022	0
B23	B183	CD3-CD16+CD56+(NK)	PTA	0.119259	0
B23	B194	CD3-CD16+CD56+(NK)	Cl	-0.11377	0
B23	B199	CD3-CD16+CD56+(NK)	Mg	-0.10709	0
B23	B28	CD3-CD16+CD56+(NK)	PRL	-0.11636	0
B23	B30	CD3-CD16+CD56+(NK)	LH	0.122704	0
B23	B31	CD3-CD16+CD56+(NK)	FSH	0.107466	0
B23	B33	CD3-CD16+CD56+(NK)	Prog	-0.11648	0
B23	B94	CD3-CD16+CD56+(NK)	ALDH2-Lys504Lys	0.089768	0
B23	B96	CD3-CD16+CD56+(NK)	dsDNA (IIF)	-0.09927	0
B23	B97	CD3-CD16+CD56+(NK)	dsDNA	0.327534	0
B23	B98	CD3-CD16+CD56+(NK)	anti-Jo-1	-0.09138	0
B23	B99	CD3-CD16+CD56+(NK)	anti-PM-Scl	-0.10311	0
B23	B195	CD3-CD16+CD56+(NK)	Na	-0.08246	1.95E-14
B23	B29	CD3-CD16+CD56+(NK)	E2	-0.08198	2.78E-14
B23	B186	CD3-CD16+CD56+(NK)	PFC	0.078652	2.92E-13
B23	B95	CD3-CD16+CD56+(NK)	anti-cenpb	0.075641	2.25E-12
B23	B180	CD3-CD16+CD56+(NK)	Tf	0.074723	4.13E-12
B23	B54	CD3-CD16+CD56+(NK)	ESR	0.073136	1.16E-11
B23	B137	CD3-CD16+CD56+(NK)	AFU	0.072534	1.71E-11
B23	B200	CD3-CD16+CD56+(NK)	OP	0.072159	2.17E-11
B23	B178	CD3-CD16+CD56+(NK)	Ig-U	0.06005	2.57E-08
B23	B69	CD3-CD16+CD56+(NK)	PV	0.058126	7.05E-08
B23	B175	CD3-CD16+CD56+(NK)	ALB-U	0.057388	1.03E-07
B23	B121	CD3-CD16+CD56+(NK)	C3	-0.0545	4.35E-07
B23	B184	CD3-CD16+CD56+(NK)	INR	0.053325	7.66E-07
B24	B111	αHBDH	TSH	0.191223	0
B24	B146	αHBDH	CysC	0.114008	0
B24	B177	αHBDH	ACR	0.151699	0
B24	B179	αHBDH	α1-MG	0.093425	0
B24	B25	αHBDH	CK	0.291389	0
B24	B26	αHBDH	CKMB	0.412911	0
B24	B27	αHBDH	LDH	0.649514	0
B24	B37	αHBDH	BAND	-0.13662	0
B24	B38	αHBDH	BAND%	-0.19124	0
B24	B47	αHBDH	RDW	0.097283	0
B24	B50	αHBDH	eo%	0.09623	0
B24	B74	αHBDH	ApoA1	0.105328	0
B24	B69	αHBDH	PV	0.086438	8.88E-16
B24	B139	αHBDH	ADA	0.083338	1.04E-14
B24	B175	αHBDH	ALB-U	0.077123	8.31E-13
B24	B131	αHBDH	AST	0.076123	1.63E-12
B24	B72	αHBDH	HDL-C	0.075362	2.71E-12

B24	B178	αHBDH	Ig-U	0.068671	1.89E-10
B24	B54	αHBDH	ESR	0.066926	5.38E-10
B24	B127	αHBDH	A-G ratio	-0.0667	6.16E-10
B24	B199	αHBDH	Mg	0.065132	1.53E-09
B24	B123	αHBDH	hs-CRP1	0.058296	6.46E-08
B24	B220	αHBDH	SBP	0.056718	1.45E-07
B24	B130	αHBDH	GLB	0.055408	2.79E-07
B24	B124	αHBDH	ACTH	0.054479	4.39E-07
B24	B221	αHBDH	Hei	-0.05311	8.49E-07
B24	B126	αHBDH	Alb	-0.05289	9.41E-07
B25	B111	CK	TSH	0.290853	0
B25	B114	CK	TSH TT3 TT4(TT4)	-0.10133	0
B25	B177	CK	ACR	0.089616	0
B25	B26	CK	CKMB	0.427238	0
B25	B27	CK	LDH	0.242266	0
B25	B131	CK	AST	0.086275	1.11E-15
B25	B112	CK	FT4	-0.08161	3.64E-14
B25	B178	CK	Ig-U	0.071328	3.67E-11
B25	B87	CK	NSE	0.059428	3.57E-08
B25	B180	CK	Tf	0.058465	5.92E-08
B25	B193	CK	K	-0.0564	1.70E-07
B25	B115	CK	TSH TT3 TT4(TT3)	-0.05524	3.03E-07
B26	B111	CKMB	TSH	0.190536	0
B26	B27	CKMB	LDH	0.31559	0
B26	B66	CKMB	WBRSR(mPas_100)	0.080055	1.10E-13
B26	B67	CKMB	WBRSR(mPas_50)	0.076473	1.29E-12
B26	B177	CKMB	ACR	0.075196	3.02E-12
B26	B63	CKMB	WBRSR(mPas_200)	0.075111	3.20E-12
B26	B62	CKMB	WBHSRVI	0.074307	5.43E-12
B26	B64	CKMB	WBRSR(mPas_5)	0.072571	1.67E-11
B26	B131	CKMB	AST	0.066554	6.71E-10
B26	B139	CKMB	ADA	0.06642	7.26E-10
B26	B81	CKMB	TSGF	0.064364	2.38E-09
B26	B87	CKMB	NSE	0.06402	2.90E-09
B26	B65	CKMB	WBRSR(mPas_1)	0.061934	9.27E-09
B26	B60	CKMB	WBRVI	0.0578	8.35E-08
B26	B114	CKMB	TSH TT3 TT4(TT4)	-0.05567	2.45E-07
B27	B111	LDH	TSH	0.133829	0
B27	B118	LDH	CEA1	0.10943	0
B27	B131	LDH	AST	0.132312	0
B27	B139	LDH	ADA	0.145368	0
B27	B146	LDH	CysC	0.124898	0
B27	B175	LDH	ALB-U	0.090258	0
B27	B177	LDH	ACR	0.139935	0

B27	B37	LDH	BAND	-0.08915	0
B27	B38	LDH	BAND%	-0.12691	0
B27	B47	LDH	RDW	0.085563	2.00E-15
B27	B72	LDH	HDL-C	0.080957	5.77E-14
B27	B127	LDH	A-G ratio	-0.07964	1.47E-13
B27	B50	LDH	eo%	0.079275	1.89E-13
B27	B74	LDH	ApoA1	0.075062	3.30E-12
B27	B179	LDH	α1-MG	0.073092	1.19E-11
B27	B150	LDH	ALP	0.072969	1.29E-11
B27	B69	LDH	PV	0.067918	2.98E-10
B27	B126	LDH	Alb	-0.06546	1.27E-09
B27	B30	LDH	LH	-0.06461	2.07E-09
B27	B128	LDH	ALT	0.064138	2.71E-09
B27	B123	LDH	hs-CRP1	0.063609	3.65E-09
B27	B33	LDH	Prog	0.061945	9.22E-09
B27	B134	LDH	CHE	-0.06014	2.45E-08
B27	B130	LDH	GLB	0.059366	3.69E-08
B27	B220	LDH	SBP	0.05909	4.27E-08
B27	B178	LDH	Ig-U	0.056687	1.47E-07
B27	B190	LDH	Cre	0.056318	1.77E-07
B27	B113	LDH	FT3	-0.05431	4.77E-07
B27	B28	LDH	PRL	0.053804	6.09E-07
B27	B191	LDH	Urea	0.053707	6.38E-07
B28	B121	PRL	C3	0.109718	0
B28	B122	PRL	C4	0.127688	0
B28	B156	PRL	IgE	-0.1629	0
B28	B29	PRL	E2	0.597137	0
B28	B30	PRL	LH	-0.70238	0
B28	B31	PRL	FSH	-0.80939	0
B28	B32	PRL	Testo	-0.26458	0
B28	B33	PRL	Prog	0.802833	0
B28	B49	PRL	baso%	-0.09392	0
B28	B50	PRL	eo%	-0.09579	0
B28	B150	PRL	ALP	0.075785	2.04E-12
B28	B113	PRL	FT3	0.066672	6.26E-10
B28	B176	PRL	Ucr	0.064426	2.30E-09
B28	B119	PRL	Hcv-rna	0.063362	4.20E-09
B28	B79	PRL	hb V -dna	0.053902	5.81E-07
B29	B122	E2	C4	0.089603	0
B29	B156	E2	IgE	-0.12808	0
B29	B30	E2	LH	-0.40288	0
B29	B31	E2	FSH	-0.65218	0
B29	B33	E2	Prog	0.121265	0
B29	B32	E2	Testo	-0.07931	1.85E-13

B29	B121	E2	C3	0.0725	1.75E-11
B29	B125	E2	Cor	-0.05434	4.71E-07
B3	B189	Hcy	eGFB	-0.16068	0
B3	B190	Hcy	Cre	0.096202	0
B3	B191	Hcy	Urea	0.11488	0
B3	B192	Hcy	UA	0.092261	0
B3	B74	Hcy	ApoA1	-0.08943	0
B3	B86	Hcy	TBhCG	-0.09846	0
B3	B146	Hcy	CysC	0.087134	4.44E-16
B3	B120	Hcy	HCV-cAg	-0.0731	1.19E-11
B3	B115	Hcy	TSH TT3 TT4(TT3)	-0.06292	5.37E-09
B3	B84	Hcy	C-PSA	0.061712	1.05E-08
B3	B199	Hcy	Mg	-0.0557	2.41E-07
B3	B48	Hcy	LYM%	-0.05443	4.49E-07
B30	B121	LH	C3	-0.12954	0
B30	B122	LH	C4	-0.16301	0
B30	B150	LH	ALP	-0.09064	0
B30	B156	LH	IgE	0.243842	0
B30	B31	LH	FSH	0.885853	0
B30	B32	LH	Testo	-0.26107	0
B30	B33	LH	Prog	-0.60418	0
B30	B49	LH	baso%	0.114471	0
B30	B50	LH	eo%	0.115242	0
B30	B176	LH	Ucr	-0.06544	1.28E-09
B30	B113	LH	FT3	-0.06425	2.55E-09
B30	B199	LH	Mg	-0.05965	3.18E-08
B30	B203	LH	DH	0.057867	8.06E-08
B30	B158	LH	IgM	-0.05739	1.03E-07
B30	B193	LH	K	-0.05415	5.16E-07
B30	B155	LH	IgA	-0.05351	7.00E-07
B31	B121	FSH	C3	-0.0968	0
B31	B122	FSH	C4	-0.1286	0
B31	B156	FSH	IgE	0.203052	0
B31	B32	FSH	Testo	-0.20413	0
B31	B33	FSH	Prog	-0.57595	0
B31	B49	FSH	baso%	0.091654	0
B31	B50	FSH	eo%	0.091419	0
B31	B150	FSH	ALP	-0.07727	7.51E-13
B31	B113	FSH	FT3	-0.05623	1.86E-07
B32	B178	Testo	Ig-U	0.141301	0
B32	B33	Testo	Prog	-0.21045	0
B32	B63	Testo	WBRSR(mPas_200)	0.121214	0
B32	B64	Testo	WBRSR(mPas_5)	0.091205	0
B32	B66	Testo	WBRSR(mPas_100)	0.122549	0

B32	B67	Testo	WBRSR(mPas_50)	0.118366	0
B32	B62	Testo	WBHSRVI	0.087113	6.66E-16
B32	B61	Testo	WBHSRV	0.079539	1.57E-13
B32	B180	Testo	Tf	0.076296	1.45E-12
B32	B69	Testo	PV	0.074351	5.28E-12
B32	B65	Testo	WBRSR(mPas_1)	0.069286	1.30E-10
B32	B200	Testo	OP	0.066186	8.32E-10
B32	B177	Testo	ACR	0.06038	2.15E-08
B33	B121	Prog	C3	0.104763	0
B33	B122	Prog	C4	0.129493	0
B33	B156	Prog	IgE	-0.18581	0
B33	B49	Prog	baso%	-0.09493	0
B33	B50	Prog	eo%	-0.10006	0
B33	B176	Prog	Ucr	0.074584	4.53E-12
B33	B150	Prog	ALP	0.071793	2.74E-11
B33	B113	Prog	FT3	0.061781	1.01E-08
B34	B73	TK	ApoA	0.123663	0
B34	B81	TK	TSGF	-0.08875	2.22E-16
B34	B193	TK	K	-0.08707	6.66E-16
B34	B79	TK	hb V -dna	0.077125	8.30E-13
B34	B184	TK	INR	0.07399	6.68E-12
B34	B185	TK	PT	0.0667	6.15E-10
B34	B107	TK	AnuA	0.059446	3.54E-08
B34	B101	TK	anti-Sc70	0.059155	4.13E-08
B34	B104	TK	anti-SSB	0.059155	4.13E-08
B34	B99	TK	anti-PM-Scl	0.059155	4.13E-08
B35	B125	β2-MG	Cor	-0.21088	0
B35	B159	β2-MG	β2-MG	0.99982	0
B35	B181	β2-MG	APTT	0.149658	0
B35	B184	β2-MG	INR	0.155095	0
B35	B186	β2-MG	PFC	-0.09282	0
B35	B185	β2-MG	PT	0.088531	2.22E-16
B35	B74	β2-MG	ApoA1	-0.07883	2.57E-13
B35	B183	β2-MG	PTA	0.058419	6.06E-08
B36	B164	V-Lym%	COM	-0.16995	0
B36	B40	V-Lym%	WBC	-0.11107	0
B36	B48	V-Lym%	LYM%	-0.07098	4.57E-11
B36	B53	V-Lym%	GR%	0.065337	1.36E-09
B37	B38	BAND	BAND%	0.955417	0
B37	B49	BAND	baso%	-0.22818	0
B37	B50	BAND	eo%	-0.12212	0
B37	B41	BAND	MON %	0.066526	6.82E-10
B37	B78	BAND	HBcAb	-0.05477	3.82E-07
B37	B146	BAND	CysC	-0.05471	3.93E-07

B38	B49	BAND%	baso%	-0.20542	0
B38	B50	BAND%	eo%	-0.14433	0
B38	B146	BAND%	CysC	-0.07555	2.39E-12
B38	B41	BAND%	MON %	0.071911	2.54E-11
B38	B40	BAND%	WBC	-0.06405	2.85E-09
B39	B44	Other cells	MCH	-0.09796	0
B39	B43	Other cells	MCV	-0.08655	8.88E-16
B39	B45	Other cells	MCHC	-0.06611	8.69E-10
B4	B108	WB-Ca	Anti-Cyclin	-0.17346	0
B4	B17	WB-Ca	CD3+CD16+CD56+NK	0.0987	0
B4	B5	WB-Ca	WB-Mg	0.612206	0
B4	B7	WB-Ca	WB-Cop	0.734551	0
B4	B8	WB-Ca	WB-Zinc	0.101182	0
B4	B185	WB-Ca	PT	0.083019	1.31E-14
B4	B54	WB-Ca	ESR	0.072426	1.83E-11
B4	B184	WB-Ca	INR	0.05928	3.86E-08
B40	B127	WBC	A-G ratio	-0.129	0
B40	B129	WBC	IBIL	-0.10871	0
B40	B130	WBC	GLB	0.144757	0
B40	B132	WBC	Dbil	-0.13456	0
B40	B133	WBC	TBIL	-0.12511	0
B40	B192	WBC	UA	0.091153	0
B40	B206	WBC	Waist	0.103582	0
B40	B207	WBC	WHR	0.096126	0
B40	B209	WBC	Smoking	0.132646	0
B40	B41	WBC	MON %	-0.16593	0
B40	B42	WBC	MON	0.653613	0
B40	B46	WBC	RBC Count	0.145648	0
B40	B48	WBC	LYM%	-0.1829	0
B40	B51	WBC	Hb	0.105616	0
B40	B52	WBC	PLT/BPC	0.278309	0
B40	B53	WBC	GR%	0.203	0
B40	B72	WBC	HDL-C	-0.11698	0
B40	B91	WBC	TP	0.113365	0
B40	B166	WBC	Pro	0.084243	5.33E-15
B40	B153	WBC	Glu	0.076395	1.36E-12
B40	B44	WBC	MCH	-0.07533	2.77E-12
B40	B43	WBC	MCV	-0.07378	7.68E-12
B40	B160	WBC	PH	-0.07083	5.02E-11
B40	B49	WBC	baso%	-0.06933	1.27E-10
B40	B215	WBC	BMI	0.068189	2.53E-10
B40	B216	WBC	BMI	0.067555	3.71E-10
B40	B71	WBC	TG	0.066661	6.29E-10
B40	B188	WBC	Ca	0.062125	8.35E-09

B40	B222	WBC	NS	0.060479	2.04E-08
B40	B163	WBC	SG	0.05893	4.64E-08
B40	B210	WBC	Hip	0.055986	2.09E-07
B40	B170	WBC	U-GLU	0.053318	7.68E-07
B41	B126	MON %	Alb	-0.12774	0
B41	B131	MON %	AST	0.091132	0
B41	B132	MON %	Dbil	0.118358	0
B41	B189	MON %	eGFB	-0.1314	0
B41	B190	MON %	Cre	0.098661	0
B41	B42	MON %	MON	0.595941	0
B41	B43	MON %	MCV	0.125608	0
B41	B44	MON %	MCH	0.106274	0
B41	B47	MON %	RDW	0.129092	0
B41	B53	MON %	GR%	-0.19323	0
B41	B71	MON %	TG	-0.10718	0
B41	B221	MON %	Hei	0.086763	8.88E-16
B41	B77	MON %	TC	-0.08664	8.88E-16
B41	B191	MON %	Urea	0.084483	4.44E-15
B41	B49	MON %	baso%	0.078031	4.47E-13
B41	B218	MON %	DBP	-0.07764	5.84E-13
B41	B192	MON %	UA	0.0724	1.86E-11
B41	B127	MON %	A-G ratio	-0.06936	1.24E-10
B41	B146	MON %	CysC	0.067913	2.99E-10
B41	B212	MON %	IOP-L	-0.06741	4.05E-10
B41	B133	MON %	TBIL	0.06596	9.49E-10
B41	B174	MON %	Urea/Cr	-0.06254	6.63E-09
B41	B211	MON %	IOP-R	-0.06042	2.11E-08
B41	B46	MON %	RBC Count	-0.05988	2.81E-08
B41	B91	MON %	TP	-0.05336	7.54E-07
B42	B126	MON	Alb	-0.11289	0
B42	B127	MON	A-G ratio	-0.15774	0
B42	B130	MON	GLB	0.142905	0
B42	B189	MON	eGFB	-0.10363	0
B42	B190	MON	Cre	0.10257	0
B42	B191	MON	Urea	0.089329	0
B42	B192	MON	UA	0.121954	0
B42	B206	MON	Waist	0.110888	0
B42	B207	MON	WHR	0.111857	0
B42	B209	MON	Smoking	0.132578	0
B42	B221	MON	Hei	0.090755	0
B42	B48	MON	LYM%	-0.17626	0
B42	B51	MON	Hb	0.093665	0
B42	B52	MON	PLT/BPC	0.197003	0
B42	B163	MON	SG	0.087053	6.66E-16

B42	B166	MON	Pro	0.086494	8.88E-16
B42	B46	MON	RBC Count	0.083679	7.99E-15
B42	B222	MON	NS	0.082285	2.22E-14
B42	B216	MON	BMI	0.081001	5.60E-14
B42	B174	MON	Urea/Cr	-0.07958	1.53E-13
B42	B72	MON	HDL-C	-0.06856	2.02E-10
B42	B123	MON	hs-CRP1	0.062285	7.64E-09
B42	B146	MON	CysC	0.061467	1.20E-08
B42	B91	MON	TP	0.059864	2.84E-08
B42	B210	MON	Hip	0.053353	7.55E-07
B42	B129	MON	IBIL	-0.05327	7.85E-07
B43	B153	MCV	Glu	-0.11351	0
B43	B189	MCV	eGFB	-0.09732	0
B43	B44	MCV	MCH	0.883656	0
B43	B45	MCV	MCHC	0.114511	0
B43	B46	MCV	RBC Count	-0.5334	0
B43	B47	MCV	RDW	0.581018	0
B43	B52	MCV	PLT/BPC	-0.13205	0
B43	B53	MCV	GR%	-0.10591	0
B43	B72	MCV	HDL-C	0.139975	0
B43	B141	MCV	AST/ALT	0.077907	4.87E-13
B43	B71	MCV	TG	-0.07784	5.09E-13
B43	B48	MCV	LYM%	0.074329	5.35E-12
B43	B218	MCV	DBP	-0.07137	3.57E-11
B43	B204	MCV	Drinking	0.068058	2.74E-10
B43	B209	MCV	Smoking	0.066034	9.09E-10
B43	B190	MCV	Cre	0.064276	2.50E-09
B43	B132	MCV	Dbil	0.06321	4.57E-09
B43	B191	MCV	Urea	0.063098	4.87E-09
B43	B112	MCV	FT4	-0.05783	8.21E-08
B43	B170	MCV	U-GLU	-0.05608	2.00E-07
B43	B135	MCV	TBA	-0.05504	3.34E-07
B43	B59	MCV	WBRV	-0.05333	7.64E-07
B44	B132	MCH	Dbil	0.116382	0
B44	B133	MCH	TBIL	0.098961	0
B44	B204	MCH	Drinking	0.139269	0
B44	B209	MCH	Smoking	0.125557	0
B44	B45	MCH	MCHC	0.564308	0
B44	B46	MCH	RBC Count	-0.39453	0
B44	B47	MCH	RDW	0.353494	0
B44	B51	MCH	Hb	0.268762	0
B44	B52	MCH	PLT/BPC	-0.11414	0
B44	B53	MCH	GR%	-0.10736	0
B44	B221	MCH	Hei	0.08494	3.11E-15

B44	B129	MCH	IBIL	0.082299	2.22E-14
B44	B48	MCH	LYM%	0.081524	3.86E-14
B44	B174	MCH	Urea/Cr	-0.06893	1.62E-10
B44	B72	MCH	HDL-C	0.068001	2.84E-10
B44	B140	MCH	r-GT	0.061745	1.03E-08
B44	B131	MCH	AST	0.058498	5.82E-08
B44	B220	MCH	SBP	-0.05745	9.99E-08
B44	B135	MCH	TBA	-0.05576	2.35E-07
B44	B150	MCH	ALP	-0.05378	6.16E-07
B45	B126	MCHC	Alb	0.131928	0
B45	B127	MCHC	A-G ratio	0.11621	0
B45	B128	MCHC	ALT	0.106588	0
B45	B129	MCHC	IBIL	0.189126	0
B45	B132	MCHC	Dbil	0.127479	0
B45	B133	MCHC	TBIL	0.18678	0
B45	B141	MCHC	AST/ALT	-0.11864	0
B45	B153	MCHC	Glu	0.13771	0
B45	B163	MCHC	SG	0.125556	0
B45	B170	MCHC	U-GLU	0.102889	0
B45	B174	MCHC	Urea/Cr	-0.10554	0
B45	B189	MCHC	eGFB	0.159608	0
B45	B191	MCHC	Urea	-0.14654	0
B45	B204	MCHC	Drinking	0.16775	0
B45	B209	MCHC	Smoking	0.142795	0
B45	B216	MCHC	BMI	0.172594	0
B45	B218	MCHC	DBP	0.142266	0
B45	B221	MCHC	Hei	0.234085	0
B45	B222	MCHC	NS	0.12465	0
B45	B46	MCHC	RBC Count	0.104396	0
B45	B47	MCHC	RDW	-0.28016	0
B45	B51	MCHC	Hb	0.490069	0
B45	B71	MCHC	TG	0.169519	0
B45	B72	MCHC	HDL-C	-0.1025	0
B45	B130	MCHC	GLB	-0.08108	5.33E-14
B45	B190	MCHC	Cre	-0.07656	1.22E-12
B45	B161	MCHC	LEU	-0.07556	2.38E-12
B45	B140	MCHC	r-GT	0.075504	2.47E-12
B45	B62	MCHC	WBHSRVI	0.07405	6.42E-12
B45	B194	MCHC	Cl	-0.07248	1.77E-11
B45	B146	MCHC	CysC	-0.07209	2.27E-11
B45	B66	MCHC	WBRSR(mPas_100)	0.071617	3.06E-11
B45	B63	MCHC	WBRSR(mPas_200)	0.070458	6.33E-11
B45	B67	MCHC	WBRSR(mPas_50)	0.06899	1.56E-10
B45	B206	MCHC	Waist	0.065881	9.94E-10

B45	B219	MCHC	OT	-0.06495	1.71E-09
B45	B64	MCHC	WBRSR(mPas_5)	0.064298	2.47E-09
B45	B94	MCHC	ALDH2-Lys504Lys	-0.06194	9.22E-09
B45	B120	MCHC	HCV-cAg	0.061839	9.77E-09
B45	B56	MCHC	IR	0.060974	1.56E-08
B45	B61	MCHC	WBHSRV	0.060143	2.44E-08
B45	B131	MCHC	AST	0.058663	5.34E-08
B45	B193	MCHC	K	-0.05795	7.73E-08
B45	B65	MCHC	WBRSR(mPas_1)	0.057022	1.24E-07
B45	B60	MCHC	WBRVI	0.054571	4.20E-07
B45	B138	MCHC	PAB	0.054516	4.31E-07
B45	B54	MCHC	ESR	-0.05447	4.41E-07
B45	B145	MCHC	BGP	-0.05438	4.60E-07
B45	B220	MCHC	SBP	-0.0536	6.71E-07
B46	B113	RBC Count	FT3	0.096377	0
B46	B126	RBC Count	Alb	0.269637	0
B46	B127	RBC Count	A-G ratio	0.153565	0
B46	B128	RBC Count	ALT	0.166313	0
B46	B129	RBC Count	IBIL	0.186386	0
B46	B133	RBC Count	TBIL	0.169879	0
B46	B141	RBC Count	AST/ALT	-0.23779	0
B46	B146	RBC Count	CysC	-0.10594	0
B46	B153	RBC Count	Glu	0.144489	0
B46	B161	RBC Count	LEU	-0.08949	0
B46	B163	RBC Count	SG	0.237652	0
B46	B170	RBC Count	U-GLU	0.138157	0
B46	B173	RBC Count	NTT	-0.09229	0
B46	B174	RBC Count	Urea/Cr	-0.11671	0
B46	B188	RBC Count	Ca	0.089184	0
B46	B189	RBC Count	eGFB	0.296256	0
B46	B190	RBC Count	Cre	-0.16355	0
B46	B191	RBC Count	Urea	-0.26147	0
B46	B204	RBC Count	Drinking	0.112901	0
B46	B206	RBC Count	Waist	0.1701	0
B46	B207	RBC Count	WHR	0.104117	0
B46	B209	RBC Count	Smoking	0.131196	0
B46	B210	RBC Count	Hip	0.143247	0
B46	B215	RBC Count	BMI	0.131154	0
B46	B216	RBC Count	BMI	0.300431	0
B46	B218	RBC Count	DBP	0.273798	0
B46	B221	RBC Count	Hei	0.330475	0
B46	B222	RBC Count	NS	0.195568	0
B46	B47	RBC Count	RDW	-0.45902	0
B46	B51	RBC Count	Hb	0.771649	0

B46	B56	RBC Count	IR	0.091864	0
B46	B60	RBC Count	WBRVI	0.143157	0
B46	B61	RBC Count	WBHSRV	0.096883	0
B46	B62	RBC Count	WBHSRVI	0.147846	0
B46	B63	RBC Count	WBRSR(mPas_200)	0.149688	0
B46	B64	RBC Count	WBRSR(mPas_5)	0.15759	0
B46	B65	RBC Count	WBRSR(mPas_1)	0.151643	0
B46	B66	RBC Count	WBRSR(mPas_100)	0.151204	0
B46	B67	RBC Count	WBRSR(mPas_50)	0.153761	0
B46	B71	RBC Count	TG	0.101587	0
B46	B72	RBC Count	HDL-C	-0.17157	0
B46	B91	RBC Count	TP	0.096509	0
B46	B176	RBC Count	Ucr	0.088504	2.22E-16
B46	B59	RBC Count	WBRV	0.081322	4.46E-14
B46	B130	RBC Count	GLB	-0.07948	1.64E-13
B46	B132	RBC Count	Dbil	0.077029	8.86E-13
B46	B49	RBC Count	baso%	0.076532	1.24E-12
B46	B70	RBC Count	LDL-C	0.074655	4.32E-12
B46	B52	RBC Count	PLT/BPC	0.070358	6.73E-11
B46	B54	RBC Count	ESR	-0.06202	8.83E-09
B46	B115	RBC Count	TSH TT3 TT4(TT3)	0.061996	8.96E-09
B46	B74	RBC Count	ApoA1	-0.0592	4.04E-08
B46	B219	RBC Count	OT	-0.0591	4.26E-08
B46	B172	RBC Count	KB	0.058109	7.12E-08
B46	B57	RBC Count	RBCAI	0.055221	3.06E-07
B47	B126	RDW	Alb	-0.14538	0
B47	B127	RDW	A-G ratio	-0.08983	0
B47	B141	RDW	AST/ALT	0.135393	0
B47	B146	RDW	CysC	0.090543	0
B47	B153	RDW	Glu	-0.16978	0
B47	B170	RDW	U-GLU	-0.09271	0
B47	B189	RDW	eGFB	-0.2091	0
B47	B190	RDW	Cre	0.142228	0
B47	B191	RDW	Urea	0.163624	0
B47	B218	RDW	DBP	-0.11137	0
B47	B221	RDW	Hei	-0.08972	0
B47	B51	RDW	Hb	-0.24174	0
B47	B52	RDW	PLT/BPC	-0.19784	0
B47	B71	RDW	TG	-0.09953	0
B47	B72	RDW	HDL-C	0.121169	0
B47	B192	RDW	UA	0.085078	2.89E-15
B47	B132	RDW	Dbil	0.084627	4.00E-15
B47	B216	RDW	BMI	-0.08005	1.10E-13
B47	B163	RDW	SG	-0.06846	2.15E-10

B47	B112	RDW	FT4	-0.06189	9.52E-09
B47	B128	RDW	ALT	-0.05737	1.04E-07
B48	B189	LYM%	eGFB	0.151675	0
B48	B190	LYM%	Cre	-0.14748	0
B48	B191	LYM%	Urea	-0.14239	0
B48	B53	LYM%	GR%	-0.93334	0
B48	B72	LYM%	HDL-C	0.115498	0
B48	B77	LYM%	TC	0.097699	0
B48	B91	LYM%	TP	0.088635	2.22E-16
B48	B126	LYM%	Alb	0.087672	4.44E-16
B48	B166	LYM%	Pro	-0.08596	1.33E-15
B48	B192	LYM%	UA	-0.08485	3.55E-15
B48	B123	LYM%	hs-CRP1	-0.08364	8.44E-15
B48	B153	LYM%	Glu	-0.08134	4.42E-14
B48	B146	LYM%	CysC	-0.07126	3.83E-11
B48	B131	LYM%	AST	0.070372	6.67E-11
B48	B207	LYM%	WHR	-0.06469	1.98E-09
B48	B70	LYM%	LDL-C	0.064664	2.01E-09
B48	B118	LYM%	CEA1	-0.05929	3.84E-08
B48	B163	LYM%	SG	-0.05578	2.31E-07
B48	B50	LYM%	eo%	-0.05487	3.63E-07
B48	B51	LYM%	Hb	0.053051	8.72E-07
B49	B50	baso%	eo%	0.259722	0
B49	B53	baso%	GR%	-0.16402	0
B49	B52	baso%	PLT/BPC	0.083498	9.33E-15
B49	B51	baso%	Hb	0.083214	1.13E-14
B49	B221	baso%	Hei	0.067408	4.05E-10
B49	B156	baso%	IgE	0.057553	9.48E-08
B49	B216	baso%	BMI	0.057234	1.11E-07
B5	B108	WB-Mg	Anti-Cyclin	-0.33692	0
B5	B17	WB-Mg	CD3+CD16+CD56+NK	0.191982	0
B5	B21	WB-Mg	CD3+CD4-CD8-	0.09406	0
B5	B6	WB-Mg	WB-Fe	0.521552	0
B5	B7	WB-Mg	WB-Cop	0.553771	0
B5	B8	WB-Mg	WB-Zinc	0.577559	0
B5	B145	WB-Mg	BGP	0.073212	1.11E-11
B5	B15	WB-Mg	CD3-CD19+(B cell)	0.072641	1.60E-11
B5	B54	WB-Mg	ESR	0.061376	1.26E-08
B5	B144	WB-Mg	Total-PINP	0.059169	4.10E-08
B5	B107	WB-Mg	AnuA	0.058458	5.94E-08
B5	B101	WB-Mg	anti-Sc70	0.058195	6.81E-08
B5	B104	WB-Mg	anti-SSB	0.058195	6.81E-08
B5	B99	WB-Mg	anti-PM-Scl	0.058195	6.81E-08
B5	B195	WB-Mg	Na	0.056516	1.60E-07

B5	B143	WB-Mg	β-CTx	0.053932	5.72E-07
B50	B156	eo%	IgE	0.101561	0
B50	B189	eo%	eGFB	-0.09253	0
B50	B190	eo%	Cre	0.121959	0
B50	B191	eo%	Urea	0.107642	0
B50	B192	eo%	UA	0.096102	0
B50	B53	eo%	GR%	-0.23096	0
B50	B146	eo%	CysC	0.079059	2.20E-13
B50	B126	eo%	Alb	-0.06685	5.63E-10
B50	B133	eo%	TBIL	-0.06496	1.69E-09
B50	B222	eo%	NS	0.063061	4.97E-09
B50	B129	eo%	IBIL	-0.06211	8.44E-09
B50	B174	eo%	Urea/Cr	-0.06035	2.19E-08
B50	B209	eo%	Smoking	0.05863	5.43E-08
B50	B220	eo%	SBP	-0.05535	2.87E-07
B50	B132	eo%	Dbil	-0.05448	4.38E-07
B51	B113	Hb	FT3	0.113284	0
B51	B126	Hb	Alb	0.296711	0
B51	B127	Hb	A-G ratio	0.187167	0
B51	B128	Hb	ALT	0.18081	0
B51	B129	Hb	IBIL	0.246651	0
B51	B130	Hb	GLB	-0.11083	0
B51	B132	Hb	Dbil	0.148133	0
B51	B133	Hb	TBIL	0.238241	0
B51	B140	Hb	r-GT	0.089298	0
B51	B141	Hb	AST/ALT	-0.24406	0
B51	B146	Hb	CysC	-0.11172	0
B51	B153	Hb	Glu	0.133765	0
B51	B161	Hb	LEU	-0.11004	0
B51	B163	Hb	SG	0.276625	0
B51	B170	Hb	U-GLU	0.146628	0
B51	B173	Hb	NTT	-0.11137	0
B51	B174	Hb	Urea/Cr	-0.16721	0
B51	B176	Hb	Ucr	0.095592	0
B51	B188	Hb	Ca	0.096458	0
B51	B189	Hb	eGFB	0.31089	0
B51	B190	Hb	Cre	-0.16229	0
B51	B191	Hb	Urea	-0.28798	0
B51	B204	Hb	Drinking	0.212042	0
B51	B206	Hb	Waist	0.190465	0
B51	B207	Hb	WHR	0.114336	0
B51	B209	Hb	Smoking	0.221467	0
B51	B210	Hb	Hip	0.163541	0
B51	B215	Hb	BMI	0.127981	0

B51	B216	Hb	BMI	0.343618	0
B51	B218	Hb	DBP	0.293901	0
B51	B221	Hb	Hei	0.401919	0
B51	B222	Hb	NS	0.228783	0
B51	B56	Hb	IR	0.093542	0
B51	B60	Hb	WBRVI	0.147445	0
B51	B61	Hb	WBHSRV	0.095494	0
B51	B62	Hb	WBHSRVI	0.165827	0
B51	B63	Hb	WBRSR(mPas_200)	0.163153	0
B51	B64	Hb	WBRSR(mPas_5)	0.164259	0
B51	B65	Hb	WBRSR(mPas_1)	0.15358	0
B51	B66	Hb	WBRSR(mPas_100)	0.163963	0
B51	B67	Hb	WBRSR(mPas_50)	0.164171	0
B51	B70	Hb	LDL-C	0.095641	0
B51	B71	Hb	TG	0.114917	0
B51	B72	Hb	HDL-C	-0.13167	0
B51	B91	Hb	TP	0.084874	3.33E-15
B51	B219	Hb	OT	-0.08324	1.11E-14
B51	B54	Hb	ESR	-0.08234	2.13E-14
B51	B172	Hb	KB	0.067764	3.27E-10
B51	B194	Hb	Cl	-0.06721	4.56E-10
B51	B77	Hb	TC	0.063468	3.96E-09
B51	B134	Hb	CHE	0.062304	7.57E-09
B51	B149	Hb	TGAb	-0.06187	9.62E-09
B51	B59	Hb	WBRV	0.060458	2.07E-08
B51	B131	Hb	AST	0.060038	2.59E-08
B51	B115	Hb	TSH TT3 TT4(TT3)	0.059647	3.18E-08
B51	B220	Hb	SBP	-0.05919	4.05E-08
B51	B74	Hb	ApoA1	-0.05671	1.46E-07
B51	B94	Hb	ALDH2-Lys504Lys	-0.05465	4.05E-07
B51	B138	Hb	PAB	0.053838	5.99E-07
B52	B128	PLT/BPC	ALT	-0.11509	0
B52	B129	PLT/BPC	IBIL	-0.11235	0
B52	B130	PLT/BPC	GLB	0.091876	0
B52	B131	PLT/BPC	AST	-0.09909	0
B52	B132	PLT/BPC	Dbil	-0.17379	0
B52	B133	PLT/BPC	TBIL	-0.13921	0
B52	B191	PLT/BPC	Urea	-0.09191	0
B52	B70	PLT/BPC	LDL-C	0.122838	0
B52	B77	PLT/BPC	TC	0.088412	2.22E-16
B52	B141	PLT/BPC	AST/ALT	0.087308	4.44E-16
B52	B127	PLT/BPC	A-G ratio	-0.08136	4.33E-14
B52	B188	PLT/BPC	Ca	0.079158	2.05E-13
B52	B91	PLT/BPC	TP	0.078394	3.49E-13

B52	B189	PLT/BPC	eGFB	0.077838	5.10E-13
B52	B192	PLT/BPC	UA	-0.06814	2.61E-10
B52	B190	PLT/BPC	Cre	-0.06706	4.97E-10
B52	B112	PLT/BPC	FT4	0.059773	2.98E-08
B52	B221	PLT/BPC	Hei	-0.05577	2.33E-07
B53	B189	GR%	eGFB	-0.09097	0
B53	B72	GR%	HDL-C	-0.10177	0
B53	B191	GR%	Urea	0.088457	2.22E-16
B53	B153	GR%	Glu	0.087147	4.44E-16
B53	B190	GR%	Cre	0.086134	1.33E-15
B53	B131	GR%	AST	-0.08367	7.99E-15
B53	B123	GR%	hs-CRP1	0.079446	1.68E-13
B53	B166	GR%	Pro	0.069786	9.58E-11
B53	B77	GR%	TC	-0.06765	3.51E-10
B53	B91	GR%	TP	-0.06538	1.33E-09
B54	B101	ESR	anti-Sc70	-0.09588	0
B54	B104	ESR	anti-SSB	-0.09588	0
B54	B107	ESR	AnuA	-0.09639	0
B54	B123	ESR	hs-CRP1	0.116567	0
B54	B127	ESR	A-G ratio	-0.09455	0
B54	B130	ESR	GLB	0.108206	0
B54	B56	ESR	IR	-0.20063	0
B54	B57	ESR	RBCAI	-0.23157	0
B54	B58	ESR	HCT	-0.10093	0
B54	B60	ESR	WBRVI	-0.37798	0
B54	B62	ESR	WBHSRVI	-0.34325	0
B54	B63	ESR	WBRSR(mPas_200)	-0.16769	0
B54	B64	ESR	WBRSR(mPas_5)	-0.21453	0
B54	B65	ESR	WBRSR(mPas_1)	-0.2358	0
B54	B66	ESR	WBRSR(mPas_100)	-0.14693	0
B54	B67	ESR	WBRSR(mPas_50)	-0.16062	0
B54	B69	ESR	PV	0.458887	0
B54	B76	ESR	ApoB	0.11205	0
B54	B81	ESR	TSGF	0.089054	0
B54	B99	ESR	anti-PM-Scl	-0.09588	0
B54	B156	ESR	IgE	0.085721	1.78E-15
B54	B179	ESR	α1-MG	0.078737	2.75E-13
B54	B91	ESR	TP	0.073425	9.63E-12
B54	B115	ESR	TSH TT3 TT4(TT3)	-0.07289	1.36E-11
B54	B155	ESR	IgA	0.069282	1.31E-10
B54	B199	ESR	Mg	-0.06391	3.08E-09
B54	B77	ESR	TC	0.062518	6.72E-09
B54	B221	ESR	Hei	-0.06062	1.90E-08
B54	B225	ESR	hPBG	0.060286	2.27E-08

B54	B157	ESR	IgG	0.05986	2.84E-08
B54	B75	ESR	ApoA1/B	-0.05647	1.64E-07
B54	B195	ESR	Na	-0.05586	2.23E-07
B54	B186	ESR	PFC	0.053457	7.19E-07
B55	B156	TK	IgE	-0.09852	0
B55	B56	TK	IR	0.769071	0
B55	B57	TK	RBCAI	-0.1484	0
B55	B58	TK	HCT	-0.56624	0
B55	B59	TK	WBRV	0.557922	0
B55	B60	TK	WBRVI	0.226532	0
B55	B61	TK	WBHSRV	0.713446	0
B55	B62	TK	WBHSRVI	0.380229	0
B55	B63	TK	WBRSR(mPas_200)	0.284647	0
B55	B64	TK	WBRSR(mPas_5)	0.212026	0
B55	B65	TK	WBRSR(mPas_1)	0.173944	0
B55	B66	TK	WBRSR(mPas_100)	0.284059	0
B55	B67	TK	WBRSR(mPas_50)	0.276498	0
B55	B69	TK	PV	-0.23762	0
B55	B79	TK	hb V -dna	-0.08436	4.88E-15
B55	B94	TK	ALDH2-Lys504Lys	-0.08243	2.00E-14
B55	B103	TK	anti-SSA/Ro	0.078915	2.43E-13
B55	B92	TK	ALDH2-Glu504Glu	0.071426	3.45E-11
B55	B107	TK	AnuA	-0.0701	7.90E-11
B55	B101	TK	anti-Sc70	-0.06976	9.75E-11
B55	B104	TK	anti-SSB	-0.06976	9.75E-11
B55	B99	TK	anti-PM-Scl	-0.06976	9.75E-11
B55	B93	TK	ALDH2-Glu504Lys	-0.06847	2.14E-10
B55	B196	TK	TCO2	-0.06563	1.15E-09
B55	B111	TK	TSH	0.059531	3.38E-08
B55	B184	TK	INR	-0.05952	3.41E-08
B55	B119	TK	Hcv-rna	-0.0583	6.44E-08
B55	B186	TK	PFC	0.056253	1.83E-07
B55	B75	TK	ApoA1/B	0.053213	8.07E-07
B56	B156	IR	IgE	-0.0912	0
B56	B58	IR	HCT	-0.23905	0
B56	B59	IR	WBRV	0.6836	0
B56	B60	IR	WBRVI	0.693749	0
B56	B61	IR	WBHSRV	0.932728	0
B56	B62	IR	WBHSRVI	0.8776	0
B56	B63	IR	WBRSR(mPas_200)	0.775448	0
B56	B64	IR	WBRSR(mPas_5)	0.713913	0
B56	B65	IR	WBRSR(mPas_1)	0.653555	0
B56	B66	IR	WBRSR(mPas_100)	0.765679	0
B56	B67	IR	WBRSR(mPas_50)	0.763972	0

B56	B69	IR	PV	-0.23576	0
B56	B94	IR	ALDH2-Lys504Lys	-0.0903	0
B56	B79	IR	hb V -dna	-0.0855	2.22E-15
B56	B74	IR	ApoA1	-0.08205	2.66E-14
B56	B89	IR	CA 72-4	0.08112	5.15E-14
B56	B92	IR	ALDH2-Glu504Glu	0.078982	2.32E-13
B56	B134	IR	CHE	0.078347	3.60E-13
B56	B184	IR	INR	-0.07824	3.87E-13
B56	B93	IR	ALDH2-Glu504Lys	-0.07577	2.06E-12
B56	B108	IR	Anti-Cyclin	-0.07379	7.59E-12
B56	B197	IR	AG	0.072965	1.30E-11
B56	B119	IR	Hcv-rna	-0.07174	2.83E-11
B56	B120	IR	HCV-cAg	0.071625	3.05E-11
B56	B103	IR	anti-SSA/Ro	0.069953	8.65E-11
B56	B125	IR	Cor	0.065594	1.17E-09
B56	B123	IR	hs-CRP1	0.063685	3.50E-09
B56	B138	IR	PAB	0.056844	1.36E-07
B57	B58	RBCAI	HCT	0.13755	0
B57	B59	RBCAI	WBRV	0.577785	0
B57	B60	RBCAI	WBRVI	0.633696	0
B57	B62	RBCAI	WBHSRVI	0.126823	0
B57	B64	RBCAI	WBRSR(mPas_5)	0.40013	0
B57	B65	RBCAI	WBRSR(mPas_1)	0.549716	0
B57	B67	RBCAI	WBRSR(mPas_50)	0.129731	0
B57	B69	RBCAI	PV	-0.19118	0
B57	B81	RBCAI	TSGF	-0.09249	0
B57	B76	RBCAI	ApoB	-0.07688	9.82E-13
B57	B122	RBCAI	C4	-0.07502	3.40E-12
B57	B183	RBCAI	PTA	-0.07389	7.15E-12
B57	B75	RBCAI	ApoA1/B	0.065895	9.86E-10
B57	B186	RBCAI	PFC	-0.06545	1.28E-09
B57	B66	RBCAI	WBRSR(mPas_100)	0.064675	2.00E-09
B57	B121	RBCAI	C3	-0.06156	1.14E-08
B57	B125	RBCAI	Cor	0.059704	3.09E-08
B57	B111	RBCAI	TSH	-0.05952	3.41E-08
B57	B178	RBCAI	Ig-U	-0.05798	7.61E-08
B57	B61	RBCAI	WBHSRV	-0.05621	1.87E-07
B57	B156	RBCAI	IgE	-0.05462	4.10E-07
B57	B136	RBCAI	5'NT	-0.05387	5.89E-07
B58	B59	HCT	WBRV	-0.26615	0
B58	B61	HCT	WBHSRV	-0.23616	0
B58	B64	HCT	WBRSR(mPas_5)	0.104823	0
B58	B65	HCT	WBRSR(mPas_1)	0.121595	0
B58	B69	HCT	PV	0.130756	0

B58	B60	HCT	WBRVI	0.075908	1.88E-12
B58	B67	HCT	WBRSR(mPas_50)	0.074213	5.78E-12
B58	B66	HCT	WBRSR(mPas_100)	0.067488	3.86E-10
B58	B63	HCT	WBRSR(mPas_200)	0.063436	4.03E-09
B59	B103	WBRV	anti-SSA/Ro	0.098417	0
B59	B123	WBRV	hs-CRP1	0.08999	0
B59	B60	WBRV	WBRVI	0.764572	0
B59	B61	WBRV	WBHSRV	0.730593	0
B59	B62	WBRV	WBHSRVI	0.56759	0
B59	B63	WBRV	WBRSR(mPas_200)	0.598411	0
B59	B64	WBRV	WBRSR(mPas_5)	0.760547	0
B59	B65	WBRV	WBRSR(mPas_1)	0.80159	0
B59	B66	WBRV	WBRSR(mPas_100)	0.601513	0
B59	B67	WBRV	WBRSR(mPas_50)	0.639835	0
B59	B125	WBRV	Cor	0.087907	4.44E-16
B59	B156	WBRV	IgE	-0.08283	1.51E-14
B59	B89	WBRV	CA 72-4	0.076259	1.49E-12
B59	B107	WBRV	AnuA	-0.07506	3.30E-12
B59	B101	WBRV	anti-Sc70	-0.07471	4.18E-12
B59	B104	WBRV	anti-SSB	-0.07471	4.18E-12
B59	B99	WBRV	anti-PM-Scl	-0.07471	4.18E-12
B59	B75	WBRV	ApoA1/B	0.069484	1.15E-10
B59	B194	WBRV	Cl	-0.06765	3.51E-10
B59	B185	WBRV	PT	-0.06542	1.30E-09
B59	B69	WBRV	PV	0.064509	2.19E-09
B59	B79	WBRV	hb V -dna	-0.06353	3.82E-09
B59	B124	WBRV	ACTH	0.063507	3.87E-09
B59	B119	WBRV	Hcv-rna	-0.0626	6.43E-09
B59	B184	WBRV	INR	-0.06099	1.55E-08
B59	B94	WBRV	ALDH2-Lys504Lys	-0.06022	2.34E-08
B59	B120	WBRV	HCV-cAg	0.055124	3.20E-07
B6	B108	WB-Fe	Anti-Cyclin	-0.48381	0
B6	B145	WB-Fe	BGP	0.103477	0
B6	B15	WB-Fe	CD3-CD19+(B cell)	0.102909	0
B6	B17	WB-Fe	CD3+CD16+CD56+NK	0.275108	0
B6	B21	WB-Fe	CD3+CD4-CD8-	0.134671	0
B6	B60	WB-Fe	WBRVI	0.10327	0
B6	B62	WB-Fe	WBHSRVI	0.103488	0
B6	B63	WB-Fe	WBRSR(mPas_200)	0.091245	0
B6	B64	WB-Fe	WBRSR(mPas_5)	0.0979	0
B6	B65	WB-Fe	WBRSR(mPas_1)	0.098396	0
B6	B66	WB-Fe	WBRSR(mPas_100)	0.090728	0
B6	B67	WB-Fe	WBRSR(mPas_50)	0.092421	0
B6	B7	WB-Fe	WB-Cop	-0.40295	0

B6	B8	WB-Fe	WB-Zinc	0.80767	0
B6	B144	WB-Fe	Total-PINP	0.083286	1.07E-14
B6	B56	WB-Fe	IR	0.08176	3.26E-14
B6	B107	WB-Fe	AnuA	0.081666	3.49E-14
B6	B101	WB-Fe	anti-Sc70	0.081278	4.62E-14
B6	B104	WB-Fe	anti-SSB	0.081278	4.62E-14
B6	B99	WB-Fe	anti-PM-Scl	0.081278	4.62E-14
B6	B195	WB-Fe	Na	0.080878	6.13E-14
B6	B143	WB-Fe	β-CTx	0.075857	1.95E-12
B6	B151	WB-Fe	Anti-CCP	0.072743	1.50E-11
B6	B199	WB-Fe	Mg	0.071129	4.16E-11
B6	B61	WB-Fe	WBHSRV	0.07021	7.37E-11
B6	B18	WB-Fe	CD3+CD4+helper(inducer)T	-0.06739	4.10E-10
B6	B57	WB-Fe	RBCAI	0.061066	1.49E-08
B6	B59	WB-Fe	WBRV	0.060367	2.17E-08
B60	B108	WBRVI	Anti-Cyclin	-0.09926	0
B60	B125	WBRVI	Cor	0.096663	0
B60	B61	WBRVI	WBHSRV	0.607817	0
B60	B62	WBRVI	WBHSRVI	0.842493	0
B60	B63	WBRVI	WBRSR(mPas_200)	0.736307	0
B60	B64	WBRVI	WBRSR(mPas_5)	0.898168	0
B60	B65	WBRVI	WBRSR(mPas_1)	0.934267	0
B60	B66	WBRVI	WBRSR(mPas_100)	0.73146	0
B60	B67	WBRVI	WBRSR(mPas_50)	0.768998	0
B60	B69	WBRVI	PV	-0.24943	0
B60	B156	WBRVI	IgE	-0.07948	1.64E-13
B60	B185	WBRVI	PT	-0.07145	3.40E-11
B60	B123	WBRVI	hs-CRP1	0.070124	7.78E-11
B60	B183	WBRVI	PTA	-0.0685	2.10E-10
B60	B134	WBRVI	CHE	0.066712	6.11E-10
B60	B221	WBRVI	Hei	0.064843	1.81E-09
B60	B103	WBRVI	anti-SSA/Ro	0.062054	8.68E-09
B60	B119	WBRVI	Hcv-rna	-0.06167	1.07E-08
B60	B89	WBRVI	CA 72-4	0.06115	1.42E-08
B60	B79	WBRVI	hb V -dna	-0.06095	1.59E-08
B60	B94	WBRVI	ALDH2-Lys504Lys	-0.05882	4.92E-08
B60	B129	WBRVI	IBIL	0.056885	1.33E-07
B60	B181	WBRVI	APTT	-0.05566	2.46E-07
B60	B218	WBRVI	DBP	0.053103	8.51E-07
B61	B120	WBHSRV	HCV-cAg	0.097737	0
B61	B123	WBHSRV	hs-CRP1	0.107367	0
B61	B134	WBHSRV	CHE	0.097792	0
B61	B184	WBHSRV	INR	-0.09348	0
B61	B62	WBHSRV	WBHSRVI	0.814665	0

B61	B63	WBHSRV	WBRSR(mPas_200)	0.862779	0
B61	B64	WBHSRV	WBRSR(mPas_5)	0.771802	0
B61	B65	WBHSRV	WBRSR(mPas_1)	0.68934	0
B61	B66	WBHSRV	WBRSR(mPas_100)	0.853111	0
B61	B67	WBHSRV	WBRSR(mPas_50)	0.847544	0
B61	B69	WBHSRV	PV	0.117545	0
B61	B89	WBHSRV	CA 72-4	0.085422	2.22E-15
B61	B194	WBHSRV	Cl	-0.08489	3.33E-15
B61	B74	WBHSRV	ApoA1	-0.08128	4.57E-14
B61	B197	WBHSRV	AG	0.080674	7.06E-14
B61	B94	WBHSRV	ALDH2-Lys504Lys	-0.07887	2.50E-13
B61	B71	WBHSRV	TG	0.077863	5.02E-13
B61	B92	WBHSRV	ALDH2-Glu504Glu	0.077181	7.99E-13
B61	B93	WBHSRV	ALDH2-Glu504Lys	-0.07467	4.27E-12
B61	B136	WBHSRV	5'NT	0.074358	5.25E-12
B61	B186	WBHSRV	PFC	0.073356	1.01E-11
B61	B103	WBHSRV	anti-SSA/Ro	0.070363	6.71E-11
B61	B125	WBHSRV	Cor	0.069892	8.98E-11
B61	B138	WBHSRV	PAB	0.068275	2.41E-10
B61	B79	WBHSRV	hb V -dna	-0.06661	6.50E-10
B61	B156	WBHSRV	IgE	-0.06272	6.01E-09
B61	B91	WBHSRV	TP	0.059765	2.99E-08
B61	B185	WBHSRV	PT	-0.05772	8.72E-08
B61	B121	WBHSRV	C3	0.057513	9.67E-08
B61	B119	WBHSRV	Hcv-rna	-0.05746	9.92E-08
B61	B108	WBHSRV	Anti-Cyclin	-0.05544	2.75E-07
B61	B140	WBHSRV	r-GT	0.054347	4.68E-07
B62	B108	WBHSRVI	Anti-Cyclin	-0.10395	0
B62	B134	WBHSRVI	CHE	0.111292	0
B62	B63	WBHSRVI	WBRSR(mPas_200)	0.913386	0
B62	B64	WBHSRVI	WBRSR(mPas_5)	0.875431	0
B62	B65	WBHSRVI	WBRSR(mPas_1)	0.814485	0
B62	B66	WBHSRVI	WBRSR(mPas_100)	0.899059	0
B62	B67	WBHSRVI	WBRSR(mPas_50)	0.901577	0
B62	B69	WBHSRVI	PV	-0.19438	0
B62	B123	WBHSRVI	hs-CRP1	0.084241	5.33E-15
B62	B74	WBHSRVI	ApoA1	-0.07959	1.51E-13
B62	B125	WBHSRVI	Cor	0.075447	2.56E-12
B62	B221	WBHSRVI	Hei	0.074457	4.92E-12
B62	B197	WBHSRVI	AG	0.071736	2.84E-11
B62	B120	WBHSRVI	HCV-cAg	0.070266	7.12E-11
B62	B184	WBHSRVI	INR	-0.06964	1.05E-10
B62	B138	WBHSRVI	PAB	0.06812	2.64E-10
B62	B89	WBHSRVI	CA 72-4	0.067081	4.91E-10

B62	B94	WBHSRVI	ALDH2-Lys504Lys	-0.06681	5.78E-10
B62	B156	WBHSRVI	IgE	-0.06447	2.24E-09
B62	B216	WBHSRVI	BMI	0.06101	1.53E-08
B62	B185	WBHSRVI	PT	-0.06059	1.92E-08
B62	B218	WBHSRVI	DBP	0.06002	2.61E-08
B62	B92	WBHSRVI	ALDH2-Glu504Glu	0.059025	4.42E-08
B62	B129	WBHSRVI	IBIL	0.05847	5.90E-08
B62	B163	WBHSRVI	SG	0.058156	6.95E-08
B62	B79	WBHSRVI	hb V -dna	-0.05716	1.16E-07
B62	B93	WBHSRVI	ALDH2-Glu504Lys	-0.05667	1.48E-07
B62	B121	WBHSRVI	C3	0.054336	4.71E-07
B62	B133	WBHSRVI	TBIL	0.053537	6.92E-07
B62	B204	WBHSRVI	Drinking	0.053141	8.36E-07
B63	B120	WBRSR(mPas_200)	HCV-cAg	0.096194	0
B63	B123	WBRSR(mPas_200)	hs-CRP1	0.130946	0
B63	B134	WBRSR(mPas_200)	CHE	0.129242	0
B63	B64	WBRSR(mPas_200)	WBRSR(mPas_5)	0.929679	0
B63	B65	WBRSR(mPas_200)	WBRSR(mPas_1)	0.846247	0
B63	B66	WBRSR(mPas_200)	WBRSR(mPas_100)	0.982956	0
B63	B67	WBRSR(mPas_200)	WBRSR(mPas_50)	0.981738	0
B63	B69	WBRSR(mPas_200)	PV	0.206035	0
B63	B194	WBRSR(mPas_200)	Cl	-0.08875	2.22E-16
B63	B108	WBRSR(mPas_200)	Anti-Cyclin	-0.08382	7.33E-15
B63	B138	WBRSR(mPas_200)	PAB	0.077667	5.74E-13
B63	B125	WBRSR(mPas_200)	Cor	0.076836	1.01E-12
B63	B184	WBRSR(mPas_200)	INR	-0.07625	1.50E-12
B63	B197	WBRSR(mPas_200)	AG	0.073743	7.84E-12
B63	B74	WBRSR(mPas_200)	ApoA1	-0.07333	1.03E-11
B63	B89	WBRSR(mPas_200)	CA 72-4	0.070852	4.95E-11
B63	B121	WBRSR(mPas_200)	C3	0.069877	9.06E-11
B63	B71	WBRSR(mPas_200)	TG	0.068413	2.21E-10
B63	B136	WBRSR(mPas_200)	5'NT	0.066292	7.82E-10
B63	B129	WBRSR(mPas_200)	IBIL	0.063762	3.35E-09
B63	B91	WBRSR(mPas_200)	TP	0.061968	9.10E-09
B63	B218	WBRSR(mPas_200)	DBP	0.061864	9.64E-09
B63	B122	WBRSR(mPas_200)	C4	0.059269	3.89E-08
B63	B221	WBRSR(mPas_200)	Hei	0.057851	8.13E-08
B63	B185	WBRSR(mPas_200)	PT	-0.05779	8.39E-08
B63	B133	WBRSR(mPas_200)	TBIL	0.056043	2.03E-07
B63	B140	WBRSR(mPas_200)	r-GT	0.055895	2.19E-07
B63	B216	WBRSR(mPas_200)	BMI	0.054575	4.19E-07
B64	B123	WBRSR(mPas_5)	hs-CRP1	0.12714	0
B64	B125	WBRSR(mPas_5)	Cor	0.09959	0
B64	B134	WBRSR(mPas_5)	CHE	0.098605	0

B64	B65	WBRSR(mPas_5)	WBRSR(mPas_1)	0.977885	0
B64	B66	WBRSR(mPas_5)	WBRSR(mPas_100)	0.924186	0
B64	B67	WBRSR(mPas_5)	WBRSR(mPas_50)	0.942781	0
B64	B69	WBRSR(mPas_5)	PV	0.132518	0
B64	B108	WBRSR(mPas_5)	Anti-Cyclin	-0.088	4.44E-16
B64	B194	WBRSR(mPas_5)	Cl	-0.08302	1.31E-14
B64	B120	WBRSR(mPas_5)	HCV-cAg	0.078338	3.62E-13
B64	B184	WBRSR(mPas_5)	INR	-0.07411	6.19E-12
B64	B185	WBRSR(mPas_5)	PT	-0.07257	1.67E-11
B64	B89	WBRSR(mPas_5)	CA 72-4	0.071265	3.82E-11
B64	B129	WBRSR(mPas_5)	IBIL	0.06548	1.25E-09
B64	B218	WBRSR(mPas_5)	DBP	0.064447	2.27E-09
B64	B138	WBRSR(mPas_5)	PAB	0.064414	2.32E-09
B64	B197	WBRSR(mPas_5)	AG	0.064342	2.41E-09
B64	B74	WBRSR(mPas_5)	ApoA1	-0.0599	2.78E-08
B64	B103	WBRSR(mPas_5)	anti-SSA/Ro	0.058724	5.17E-08
B64	B221	WBRSR(mPas_5)	Hei	0.058273	6.54E-08
B64	B133	WBRSR(mPas_5)	TBIL	0.05801	7.49E-08
B64	B163	WBRSR(mPas_5)	SG	0.052924	9.26E-07
B65	B123	WBRSR(mPas_1)	hs-CRP1	0.117892	0
B65	B125	WBRSR(mPas_1)	Cor	0.106675	0
B65	B66	WBRSR(mPas_1)	WBRSR(mPas_100)	0.842672	0
B65	B67	WBRSR(mPas_1)	WBRSR(mPas_50)	0.8722	0
B65	B108	WBRSR(mPas_1)	Anti-Cyclin	-0.0875	4.44E-16
B65	B134	WBRSR(mPas_1)	CHE	0.085842	1.78E-15
B65	B69	WBRSR(mPas_1)	PV	0.078482	3.28E-13
B65	B194	WBRSR(mPas_1)	Cl	-0.0773	7.37E-13
B65	B185	WBRSR(mPas_1)	PT	-0.07541	2.63E-12
B65	B89	WBRSR(mPas_1)	CA 72-4	0.068241	2.46E-10
B65	B103	WBRSR(mPas_1)	anti-SSA/Ro	0.066106	8.72E-10
B65	B184	WBRSR(mPas_1)	INR	-0.06522	1.46E-09
B65	B120	WBRSR(mPas_1)	HCV-cAg	0.063634	3.60E-09
B65	B138	WBRSR(mPas_1)	PAB	0.0635	3.88E-09
B65	B129	WBRSR(mPas_1)	IBIL	0.062564	6.55E-09
B65	B218	WBRSR(mPas_1)	DBP	0.060959	1.58E-08
B65	B197	WBRSR(mPas_1)	AG	0.060829	1.69E-08
B65	B133	WBRSR(mPas_1)	TBIL	0.055626	2.50E-07
B65	B221	WBRSR(mPas_1)	Hei	0.054686	3.97E-07
B65	B181	WBRSR(mPas_1)	APTT	-0.0539	5.81E-07
B65	B156	WBRSR(mPas_1)	IgE	-0.05352	6.97E-07
B66	B120	WBRSR(mPas_100)	HCV-cAg	0.098271	0
B66	B123	WBRSR(mPas_100)	hs-CRP1	0.136816	0
B66	B134	WBRSR(mPas_100)	CHE	0.125301	0
B66	B67	WBRSR(mPas_100)	WBRSR(mPas_50)	0.994633	0

B66	B69	WBRSR(mPas_100)	PV	0.208638	0
B66	B194	WBRSR(mPas_100)	CI	-0.08653	8.88E-16
B66	B184	WBRSR(mPas_100)	INR	-0.08486	3.33E-15
B66	B108	WBRSR(mPas_100)	Anti-Cyclin	-0.08417	5.77E-15
B66	B197	WBRSR(mPas_100)	AG	0.079904	1.22E-13
B66	B138	WBRSR(mPas_100)	PAB	0.075709	2.15E-12
B66	B74	WBRSR(mPas_100)	ApoA1	-0.07558	2.35E-12
B66	B89	WBRSR(mPas_100)	CA 72-4	0.071894	2.57E-11
B66	B185	WBRSR(mPas_100)	PT	-0.0669	5.48E-10
B66	B71	WBRSR(mPas_100)	TG	0.066122	8.64E-10
B66	B218	WBRSR(mPas_100)	DBP	0.065769	1.06E-09
B66	B136	WBRSR(mPas_100)	5'NT	0.065002	1.65E-09
B66	B91	WBRSR(mPas_100)	TP	0.063452	3.99E-09
B66	B129	WBRSR(mPas_100)	IBIL	0.063062	4.97E-09
B66	B92	WBRSR(mPas_100)	ALDH2-Glu504Glu	0.058545	5.68E-08
B66	B221	WBRSR(mPas_100)	Hei	0.057849	8.14E-08
B66	B140	WBRSR(mPas_100)	r-GT	0.057078	1.21E-07
B66	B93	WBRSR(mPas_100)	ALDH2-Glu504Lys	-0.05688	1.33E-07
B66	B163	WBRSR(mPas_100)	SG	0.056342	1.75E-07
B66	B94	WBRSR(mPas_100)	ALDH2-Lys504Lys	-0.05623	1.85E-07
B66	B133	WBRSR(mPas_100)	TBIL	0.056114	1.96E-07
B66	B216	WBRSR(mPas_100)	BMI	0.055541	2.61E-07
B66	B186	WBRSR(mPas_100)	PFC	0.05485	3.67E-07
B67	B120	WBRSR(mPas_50)	HCV-cAg	0.09574	0
B67	B123	WBRSR(mPas_50)	hs-CRP1	0.137365	0
B67	B134	WBRSR(mPas_50)	CHE	0.123178	0
B67	B69	WBRSR(mPas_50)	PV	0.199283	0
B67	B108	WBRSR(mPas_50)	Anti-Cyclin	-0.08551	2.22E-15
B67	B194	WBRSR(mPas_50)	CI	-0.08526	2.66E-15
B67	B184	WBRSR(mPas_50)	INR	-0.0843	5.11E-15
B67	B197	WBRSR(mPas_50)	AG	0.07886	2.52E-13
B67	B74	WBRSR(mPas_50)	ApoA1	-0.07745	6.64E-13
B67	B138	WBRSR(mPas_50)	PAB	0.073606	8.57E-12
B67	B89	WBRSR(mPas_50)	CA 72-4	0.07261	1.63E-11
B67	B185	WBRSR(mPas_50)	PT	-0.06962	1.06E-10
B67	B218	WBRSR(mPas_50)	DBP	0.06543	1.29E-09
B67	B129	WBRSR(mPas_50)	IBIL	0.064651	2.02E-09
B67	B71	WBRSR(mPas_50)	TG	0.063358	4.21E-09
B67	B136	WBRSR(mPas_50)	5'NT	0.061505	1.17E-08
B67	B91	WBRSR(mPas_50)	TP	0.059258	3.91E-08
B67	B221	WBRSR(mPas_50)	Hei	0.058652	5.37E-08
B67	B92	WBRSR(mPas_50)	ALDH2-Glu504Glu	0.058323	6.37E-08
B67	B133	WBRSR(mPas_50)	TBIL	0.057578	9.35E-08
B67	B94	WBRSR(mPas_50)	ALDH2-Lys504Lys	-0.05737	1.04E-07

B67	B93	WBRSR(mPas_50)	ALDH2-Glu504Lys	-0.05658	1.55E-07
B67	B216	WBRSR(mPas_50)	BMI	0.056439	1.67E-07
B67	B163	WBRSR(mPas_50)	SG	0.055233	3.04E-07
B67	B140	WBRSR(mPas_50)	r-GT	0.054004	5.53E-07
B69	B123	PV	hs-CRP1	0.109091	0
B69	B127	PV	A-G ratio	-0.09493	0
B69	B130	PV	GLB	0.13039	0
B69	B136	PV	5'NT	0.090784	0
B69	B155	PV	IgA	0.120961	0
B69	B156	PV	IgE	0.095404	0
B69	B157	PV	IgG	0.092743	0
B69	B194	PV	Cl	-0.11974	0
B69	B91	PV	TP	0.127391	0
B69	B114	PV	TSH TT3 TT4(TT4)	0.087743	4.44E-16
B69	B195	PV	Na	-0.08467	4.00E-15
B69	B71	PV	TG	0.08079	6.53E-14
B69	B77	PV	TC	0.078931	2.40E-13
B69	B180	PV	Tf	0.078793	2.65E-13
B69	B76	PV	ApoB	0.075008	3.42E-12
B69	B175	PV	ALB-U	0.072351	1.92E-11
B69	B179	PV	α1-MG	0.072	2.40E-11
B69	B137	PV	AFU	0.068374	2.27E-10
B69	B120	PV	HCV-cAg	0.062964	5.24E-09
B69	B186	PV	PFC	0.061503	1.17E-08
B69	B183	PV	PTA	0.05888	4.77E-08
B69	B178	PV	Ig-U	0.058423	6.05E-08
B69	B112	PV	FT4	0.057113	1.19E-07
B69	B122	PV	C4	0.057017	1.25E-07
B69	B134	PV	CHE	0.054747	3.85E-07
B69	B139	PV	ADA	0.054262	4.88E-07
B69	B108	PV	Anti-Cyclin	0.052944	9.17E-07
B7	B8	WB-Cop	WB-Zinc	-0.22448	0
B7	B62	WB-Cop	WBHSRVI	-0.08656	8.88E-16
B7	B56	WB-Cop	IR	-0.08556	2.00E-15
B7	B54	WB-Cop	ESR	0.077908	4.87E-13
B7	B60	WB-Cop	WBRVI	-0.07207	2.30E-11
B7	B61	WB-Cop	WBHSRV	-0.06837	2.27E-10
B7	B63	WB-Cop	WBRSR(mPas_200)	-0.06765	3.50E-10
B7	B67	WB-Cop	WBRSR(mPas_50)	-0.06486	1.80E-09
B7	B66	WB-Cop	WBRSR(mPas_100)	-0.0647	1.97E-09
B7	B64	WB-Cop	WBRSR(mPas_5)	-0.06281	5.70E-09
B7	B55	WB-Cop	TK	-0.06259	6.46E-09
B7	B108	WB-Cop	Anti-Cyclin	0.060669	1.84E-08
B7	B69	WB-Cop	PV	0.059004	4.47E-08

B7	B65	WB-Cop	WBRSR(mPas_1)	-0.05882	4.92E-08
B70	B127	LDL-C	A-G ratio	-0.13669	0
B70	B130	LDL-C	GLB	0.135952	0
B70	B132	LDL-C	Dbil	-0.22177	0
B70	B133	LDL-C	TBIL	-0.0934	0
B70	B220	LDL-C	SBP	0.099338	0
B70	B72	LDL-C	HDL-C	0.169174	0
B70	B75	LDL-C	ApoA1/B	-0.19075	0
B70	B76	LDL-C	ApoB	0.285936	0
B70	B77	LDL-C	TC	0.813208	0
B70	B91	LDL-C	TP	0.120936	0
B70	B221	LDL-C	Hei	-0.08556	2.00E-15
B70	B218	LDL-C	DBP	0.074103	6.21E-12
B70	B74	LDL-C	ApoA1	0.073976	6.74E-12
B70	B188	LDL-C	Ca	0.068358	2.29E-10
B70	B128	LDL-C	ALT	-0.06518	1.49E-09
B70	B141	LDL-C	AST/ALT	0.061967	9.11E-09
B70	B71	LDL-C	TG	-0.05808	7.23E-08
B70	B153	LDL-C	Glu	0.053963	5.64E-07
B70	B131	LDL-C	AST	-0.05383	6.02E-07
B71	B126	TG	Alb	0.127529	0
B71	B127	TG	A-G ratio	-0.11896	0
B71	B128	TG	ALT	0.106461	0
B71	B130	TG	GLB	0.211958	0
B71	B132	TG	Dbil	-0.10872	0
B71	B134	TG	CHE	0.126546	0
B71	B136	TG	5'NT	0.101085	0
B71	B140	TG	r-GT	0.162746	0
B71	B153	TG	Glu	0.240642	0
B71	B170	TG	U-GLU	0.152219	0
B71	B189	TG	eGFB	0.089521	0
B71	B192	TG	UA	0.131907	0
B71	B194	TG	Cl	-0.10271	0
B71	B204	TG	Drinking	0.104858	0
B71	B206	TG	Waist	0.136312	0
B71	B207	TG	WHR	0.128333	0
B71	B209	TG	Smoking	0.1193	0
B71	B215	TG	BMI	0.157026	0
B71	B216	TG	BMI	0.150521	0
B71	B218	TG	DBP	0.139098	0
B71	B222	TG	NS	0.090726	0
B71	B72	TG	HDL-C	-0.34672	0
B71	B77	TG	TC	0.387895	0
B71	B91	TG	TP	0.273816	0

B71	B120	TG	HCV-cAg	0.088107	4.44E-16
B71	B188	TG	Ca	0.087295	4.44E-16
B71	B195	TG	Na	-0.08659	8.88E-16
B71	B141	TG	AST/ALT	-0.08618	1.33E-15
B71	B133	TG	TBIL	-0.07921	1.98E-13
B71	B131	TG	AST	0.075802	2.02E-12
B71	B163	TG	SG	0.07553	2.42E-12
B71	B210	TG	Hip	0.072812	1.43E-11
B71	B74	TG	ApoA1	-0.07094	4.69E-11
B71	B172	TG	KB	0.069369	1.24E-10
B71	B155	TG	IgA	0.066961	5.27E-10
B71	B137	TG	AFU	0.065522	1.22E-09
B71	B138	TG	PAB	0.062954	5.27E-09
B71	B184	TG	INR	-0.06161	1.11E-08
B71	B81	TG	TSGF	0.060771	1.75E-08
B71	B129	TG	IBIL	-0.06024	2.32E-08
B71	B221	TG	Hei	0.05449	4.37E-07
B72	B128	HDL-C	ALT	-0.09761	0
B72	B141	HDL-C	AST/ALT	0.184369	0
B72	B153	HDL-C	Glu	-0.15163	0
B72	B163	HDL-C	SG	-0.13851	0
B72	B174	HDL-C	Urea/Cr	0.102069	0
B72	B192	HDL-C	UA	-0.20787	0
B72	B206	HDL-C	Waist	-0.25577	0
B72	B207	HDL-C	WHR	-0.20694	0
B72	B209	HDL-C	Smoking	-0.1468	0
B72	B210	HDL-C	Hip	-0.17191	0
B72	B215	HDL-C	BMI	-0.22394	0
B72	B216	HDL-C	BMI	-0.3107	0
B72	B221	HDL-C	Hei	-0.24022	0
B72	B222	HDL-C	NS	-0.20628	0
B72	B74	HDL-C	ApoA1	0.241322	0
B72	B77	HDL-C	TC	0.234569	0
B72	B170	HDL-C	U-GLU	-0.08469	4.00E-15
B72	B75	HDL-C	ApoA1/B	0.083077	1.24E-14
B72	B190	HDL-C	Cre	-0.08182	3.11E-14
B72	B130	HDL-C	GLB	-0.08063	7.31E-14
B72	B160	HDL-C	PH	0.079449	1.67E-13
B72	B218	HDL-C	DBP	-0.07844	3.38E-13
B72	B220	HDL-C	SBP	0.065783	1.05E-09
B72	B91	HDL-C	TP	-0.06518	1.49E-09
B72	B189	HDL-C	eGFB	0.060569	1.95E-08
B72	B127	HDL-C	A-G ratio	0.060502	2.02E-08
B72	B176	HDL-C	Ucr	-0.05493	3.52E-07

B72	B188	HDL-C	Ca	0.052881	9.45E-07
B73	B137	ApoA	AFU	-0.13593	0
B73	B74	ApoA	ApoA1	0.092379	0
B73	B76	ApoA	ApoB	0.138609	0
B73	B75	ApoA	ApoA1/B	-0.07176	2.79E-11
B73	B199	ApoA	Mg	0.071688	2.93E-11
B73	B96	ApoA	dsDNA (IIF)	-0.06729	4.33E-10
B73	B122	ApoA	C4	0.065783	1.05E-09
B73	B194	ApoA	Cl	0.065073	1.59E-09
B73	B138	ApoA	PAB	-0.05482	3.72E-07
B74	B110	ApoA1	AHA	0.092582	0
B74	B195	ApoA1	Na	0.116184	0
B74	B196	ApoA1	TCO2	0.104174	0
B74	B199	ApoA1	Mg	0.143379	0
B74	B216	ApoA1	BMI	-0.11385	0
B74	B221	ApoA1	Hei	-0.1083	0
B74	B75	ApoA1	ApoA1/B	0.334482	0
B74	B76	ApoA1	ApoB	0.139879	0
B74	B77	ApoA1	TC	0.093033	0
B74	B176	ApoA1	Ucr	-0.08818	2.22E-16
B74	B86	ApoA1	TBhCG	0.085716	1.78E-15
B74	B159	ApoA1	β2-MG	-0.07867	2.88E-13
B74	B123	ApoA1	hs-CRP1	-0.07592	1.87E-12
B74	B95	ApoA1	anti-cenpb	0.07592	1.87E-12
B74	B206	ApoA1	Waist	-0.07569	2.17E-12
B74	B97	ApoA1	dsDNA	0.075469	2.52E-12
B74	B120	ApoA1	HCV-cAg	-0.07378	7.66E-12
B74	B222	ApoA1	NS	-0.07359	8.67E-12
B74	B141	ApoA1	AST/ALT	0.069223	1.35E-10
B74	B163	ApoA1	SG	-0.06899	1.56E-10
B74	B81	ApoA1	TSGF	-0.0679	3.01E-10
B74	B146	ApoA1	CysC	-0.06727	4.40E-10
B74	B188	ApoA1	Ca	0.066482	6.99E-10
B74	B181	ApoA1	APTT	-0.06315	4.74E-09
B74	B207	ApoA1	WHR	-0.06248	6.86E-09
B74	B215	ApoA1	BMI	-0.06213	8.34E-09
B74	B107	ApoA1	AnuA	0.061968	9.10E-09
B74	B101	ApoA1	anti-Sc70	0.061603	1.11E-08
B74	B104	ApoA1	anti-SSB	0.061603	1.11E-08
B74	B99	ApoA1	anti-PM-Scl	0.061603	1.11E-08
B74	B209	ApoA1	Smoking	-0.05896	4.57E-08
B74	B151	ApoA1	Anti-CCP	0.057065	1.22E-07
B74	B125	ApoA1	Cor	0.056824	1.37E-07
B74	B180	ApoA1	Tf	0.056417	1.69E-07

B74	B134	ApoA1	CHE	-0.05626	1.83E-07
B74	B115	ApoA1	TSH TT3 TT4(TT3)	0.053932	5.72E-07
B74	B109	ApoA1	AMA-M2	0.053391	7.42E-07
B75	B134	ApoA1/B	CHE	-0.09454	0
B75	B136	ApoA1/B	5'NT	0.100135	0
B75	B155	ApoA1/B	IgA	0.10361	0
B75	B157	ApoA1/B	IgG	0.123422	0
B75	B76	ApoA1/B	ApoB	-0.72783	0
B75	B77	ApoA1/B	TC	-0.11978	0
B75	B182	ApoA1/B	TT	0.077833	5.12E-13
B75	B139	ApoA1/B	ADA	-0.0729	1.35E-11
B75	B132	ApoA1/B	Dbil	0.071892	2.57E-11
B75	B113	ApoA1/B	FT3	0.069754	9.78E-11
B75	B125	ApoA1/B	Cor	-0.06398	2.96E-09
B75	B193	ApoA1/B	K	-0.06153	1.16E-08
B75	B179	ApoA1/B	α1-MG	-0.05669	1.47E-07
B75	B106	ApoA1/B	anti-P	0.055598	2.54E-07
B76	B134	ApoB	CHE	0.152328	0
B76	B139	ApoB	ADA	0.089248	0
B76	B77	ApoB	TC	0.253275	0
B76	B125	ApoB	Cor	0.083408	9.77E-15
B76	B132	ApoB	Dbil	-0.08319	1.15E-14
B76	B193	ApoB	K	0.07967	1.43E-13
B76	B135	ApoB	TBA	-0.07825	3.84E-13
B76	B122	ApoB	C4	0.069023	1.53E-10
B76	B91	ApoB	TP	0.065414	1.30E-09
B76	B150	ApoB	ALP	0.06265	6.25E-09
B76	B121	ApoB	C3	0.061943	9.23E-09
B76	B137	ApoB	AFU	0.061588	1.12E-08
B76	B130	ApoB	GLB	0.059139	4.16E-08
B76	B106	ApoB	anti-P	-0.05874	5.13E-08
B76	B148	ApoB	Anti_TPO	0.058427	6.04E-08
B76	B157	ApoB	IgG	-0.0579	7.91E-08
B76	B196	ApoB	TCO2	0.057186	1.14E-07
B76	B181	ApoB	APTT	-0.05584	2.25E-07
B76	B153	ApoB	Glu	0.054293	4.81E-07
B76	B111	ApoB	TSH	0.054281	4.84E-07
B77	B127	TC	A-G ratio	-0.18313	0
B77	B130	TC	GLB	0.217545	0
B77	B132	TC	Dbil	-0.23659	0
B77	B133	TC	TBIL	-0.11404	0
B77	B153	TC	Glu	0.140766	0
B77	B188	TC	Ca	0.11624	0
B77	B218	TC	DBP	0.096675	0

B77	B220	TC	SBP	0.128036	0
B77	B221	TC	Hei	-0.13515	0
B77	B91	TC	TP	0.223438	0
B77	B174	TC	Urea/Cr	0.082511	1.91E-14
B77	B134	TC	CHE	0.08201	2.71E-14
B77	B136	TC	5'NT	0.081299	4.53E-14
B77	B170	TC	U-GLU	0.079813	1.30E-13
B77	B140	TC	r-GT	0.079764	1.35E-13
B77	B216	TC	BMI	-0.07655	1.23E-12
B77	B141	TC	AST/ALT	0.068973	1.58E-10
B77	B194	TC	Cl	-0.06234	7.43E-09
B77	B175	TC	ALB-U	0.060521	2.00E-08
B77	B177	TC	ACR	0.060118	2.48E-08
B77	B150	TC	ALP	0.05765	9.02E-08
B77	B129	TC	IBIL	-0.05744	1.00E-07
B77	B222	TC	NS	-0.05398	5.61E-07
B77	B180	TC	Tf	0.053855	5.94E-07
B78	B102	HBcAb	anti-Sm	0.116822	0
B78	B103	HBcAb	anti-SSA/Ro	0.098037	0
B78	B105	HBcAb	anti-U1RNP	0.118621	0
B78	B80	HBcAb	anti-HP IgG	-0.10733	0
B78	B186	HBcAb	PFC	-0.05974	3.03E-08
B78	B98	HBcAb	anti-Jo-1	-0.05402	5.50E-07
B79	B101	hbV-dna	anti-Sc70	0.246321	0
B79	B104	hbV-dna	anti-SSB	0.246321	0
B79	B107	hbV-dna	AnuA	0.247549	0
B79	B109	hbV-dna	AMA-M2	0.093831	0
B79	B119	hbV-dna	Hcv-rna	0.327036	0
B79	B120	hbV-dna	HCV-cAg	-0.13367	0
B79	B124	hbV-dna	ACTH	-0.09908	0
B79	B139	hbV-dna	ADA	-0.12936	0
B79	B157	hbV-dna	IgG	0.129405	0
B79	B181	hbV-dna	APTT	0.111708	0
B79	B183	hbV-dna	PTA	0.158517	0
B79	B184	hbV-dna	INR	0.283293	0
B79	B185	hbV-dna	PT	0.161344	0
B79	B186	hbV-dna	PFC	-0.14812	0
B79	B194	hbV-dna	Cl	0.097753	0
B79	B196	hbV-dna	TCO2	0.130083	0
B79	B197	hbV-dna	AG	-0.17039	0
B79	B200	hbV-dna	OP	-0.13932	0
B79	B81	hbV-dna	TSGF	-0.17262	0
B79	B92	hbV-dna	ALDH2-Glu504Glu	-0.20247	0
B79	B93	hbV-dna	ALDH2-Glu504Lys	0.189629	0

B79	B94	hb V -dna	ALDH2-Lys504Lys	0.30032	0
B79	B96	hb V -dna	dsDNA (IIF)	0.102139	0
B79	B99	hb V -dna	anti-PM-Scl	0.246321	0
B79	B179	hb V -dna	α1-MG	-0.08563	1.78E-15
B79	B198	hb V -dna	P	-0.07687	9.85E-13
B79	B193	hb V -dna	K	0.074385	5.16E-12
B79	B115	hb V -dna	TSH TT3 TT4(TT3)	0.073213	1.11E-11
B79	B100	hb V -dna	anti-Ro52	0.070984	4.56E-11
B79	B134	hb V -dna	CHE	-0.06556	1.20E-09
B79	B125	hb V -dna	Cor	0.061691	1.06E-08
B79	B177	hb V -dna	ACR	-0.0606	1.92E-08
B79	B123	hb V -dna	hs-CRP1	-0.05968	3.12E-08
B79	B178	hb V -dna	Ig-U	-0.05762	9.16E-08
B79	B170	hb V -dna	U-GLU	-0.05582	2.27E-07
B8	B108	WB-Zinc	Anti-Cyclin	-0.28458	0
B8	B17	WB-Zinc	CD3+CD16+CD56+NK	0.161873	0
B8	B185	WB-Zinc	PT	-0.08658	8.88E-16
B8	B21	WB-Zinc	CD3+CD4-CD8-	0.079251	1.92E-13
B8	B184	WB-Zinc	INR	-0.06199	9.00E-09
B8	B145	WB-Zinc	BGP	0.061025	1.52E-08
B8	B15	WB-Zinc	CD3-CD19+(B cell)	0.060667	1.85E-08
B81	B125	TSGF	Cor	-0.14959	0
B81	B139	TSGF	ADA	0.104218	0
B81	B151	TSGF	Anti-CCP	-0.15147	0
B81	B183	TSGF	PTA	-0.09181	0
B81	B184	TSGF	INR	-0.19047	0
B81	B185	TSGF	PT	-0.1652	0
B81	B186	TSGF	PFC	0.13555	0
B81	B197	TSGF	AG	0.122987	0
B81	B200	TSGF	OP	0.092473	0
B81	B92	TSGF	ALDH2-Glu504Glu	0.137371	0
B81	B93	TSGF	ALDH2-Glu504Lys	-0.13212	0
B81	B94	TSGF	ALDH2-Lys504Lys	-0.15214	0
B81	B182	TSGF	TT	0.087808	4.44E-16
B81	B134	TSGF	CHE	0.08556	2.00E-15
B81	B155	TSGF	IgA	0.081875	3.00E-14
B81	B196	TSGF	TCO2	-0.07777	5.34E-13
B81	B108	TSGF	Anti-Cyclin	0.076871	9.86E-13
B81	B98	TSGF	anti-Jo-1	-0.07485	3.81E-12
B81	B194	TSGF	Cl	-0.07075	5.28E-11
B81	B199	TSGF	Mg	-0.06621	8.18E-10
B81	B107	TSGF	AnuA	-0.06472	1.94E-09
B81	B101	TSGF	anti-Sc70	-0.06439	2.34E-09
B81	B104	TSGF	anti-SSB	-0.06439	2.34E-09

B81	B99	TSGF	anti-PM-Scl	-0.06439	2.34E-09
B81	B178	TSGF	Ig-U	0.062113	8.40E-09
B81	B177	TSGF	ACR	0.056452	1.66E-07
B82	B83	CEA	AFP	0.148396	0
B82	B84	CEA	C-PSA	0.154253	0
B82	B85	CEA	SCC	0.217115	0
B82	B86	CEA	TBhCG	-0.25405	0
B82	B87	CEA	NSE	0.141703	0
B82	B88	CEA	CA 242	0.481277	0
B82	B90	CEA	CYFRA 21-1	0.346823	0
B82	B89	CEA	CA 72-4	0.086718	8.88E-16
B82	B139	CEA	ADA	0.053106	8.50E-07
B83	B106	AFP	anti-P	0.144115	0
B83	B136	AFP	5'NT	0.12838	0
B83	B139	AFP	ADA	0.094992	0
B83	B85	AFP	SCC	0.317113	0
B83	B86	AFP	TBhCG	0.163733	0
B83	B87	AFP	NSE	0.250257	0
B83	B88	AFP	CA 242	0.178464	0
B83	B89	AFP	CA 72-4	0.079937	1.19E-13
B83	B150	AFP	ALP	0.076886	9.76E-13
B83	B140	AFP	r-GT	0.07601	1.76E-12
B83	B137	AFP	AFU	0.075179	3.06E-12
B84	B228	C-PSA	OS	0.106655	0
B84	B85	C-PSA	SCC	0.18802	0
B84	B86	C-PSA	TBhCG	-0.21528	0
B84	B87	C-PSA	NSE	0.116465	0
B84	B88	C-PSA	CA 242	0.117916	0
B84	B116	C-PSA	TT3	-0.0784	3.47E-13
B84	B90	C-PSA	CYFRA 21-1	0.062164	8.17E-09
B84	B117	C-PSA	TT4	0.05939	3.65E-08
B85	B87	SCC	NSE	0.28637	0
B85	B88	SCC	CA 242	0.240651	0
B85	B90	SCC	CYFRA 21-1	0.119799	0
B85	B191	SCC	Urea	0.05607	2.01E-07
B85	B135	SCC	TBA	0.054326	4.73E-07
B85	B168	SCC	NMP22	0.053281	7.82E-07
B85	B189	SCC	eGFB	-0.05278	9.89E-07
B86	B139	TBhCG	ADA	-0.11661	0
B86	B199	TBhCG	Mg	0.118443	0
B86	B200	TBhCG	OP	-0.0894	0
B86	B88	TBhCG	CA 242	-0.11668	0
B86	B137	TBhCG	AFU	-0.08813	4.44E-16
B86	B194	TBhCG	Cl	0.067502	3.82E-10

B86	B115	TBhCG	TSH TT3 TT4(TT3)	0.056846	1.36E-07
B86	B89	TBhCG	CA 72-4	-0.05504	3.35E-07
B86	B129	TBhCG	IBIL	-0.05387	5.89E-07
B87	B88	NSE	CA 242	0.223606	0
B87	B90	NSE	CYFRA 21-1	0.083343	1.02E-14
B87	B198	NSE	P	0.071673	2.95E-11
B88	B90	CA 242	CYFRA 21-1	0.191616	0
B9	B101	RH	anti-Sc70	-0.09394	0
B9	B104	RH	anti-SSB	-0.09394	0
B9	B107	RH	AnuA	-0.09438	0
B9	B125	RH	Cor	0.137001	0
B9	B131	RH	AST	0.093637	0
B9	B136	RH	5'NT	0.411662	0
B9	B139	RH	ADA	0.090407	0
B9	B140	RH	r-GT	0.110302	0
B9	B15	RH	CD3-CD19+(B cell)	-0.1409	0
B9	B18	RH	CD3+CD4+helper(inducer)T	-0.22267	0
B9	B181	RH	APTT	-0.15857	0
B9	B184	RH	INR	-0.16639	0
B9	B185	RH	PT	-0.10297	0
B9	B19	RH	CD3+CD4+/CD3+CD8+	-0.13906	0
B9	B193	RH	K	-0.08982	0
B9	B22	RH	CD3+CD8+(T)	0.20984	0
B9	B99	RH	anti-PM-Scl	-0.09394	0
B9	B23	RH	CD3-CD16+CD56+(NK)	0.065935	9.63E-10
B9	B150	RH	ALP	0.064009	2.91E-09
B9	B76	RH	ApoB	0.062375	7.27E-09
B9	B200	RH	OP	0.057444	1.00E-07
B9	B27	RH	LDH	0.057076	1.21E-07
B9	B183	RH	PTA	-0.05681	1.38E-07
B9	B198	RH	P	-0.05564	2.48E-07
B90	B190	CYFRA 21-1	Cre	0.105923	0
B90	B189	CYFRA 21-1	eGFB	-0.08444	4.66E-15
B90	B191	CYFRA 21-1	Urea	0.075234	2.95E-12
B90	B139	CYFRA 21-1	ADA	0.072876	1.37E-11
B90	B199	CYFRA 21-1	Mg	-0.06245	6.98E-09
B90	B200	CYFRA 21-1	OP	0.06128	1.33E-08
B90	B146	CYFRA 21-1	CysC	0.061256	1.34E-08
B91	B126	TP	Alb	0.44473	0
B91	B127	TP	A-G ratio	-0.48117	0
B91	B128	TP	ALT	0.091397	0
B91	B130	TP	GLB	0.788563	0
B91	B131	TP	AST	0.122031	0
B91	B153	TP	Glu	0.109582	0

B91	B157	TP	IgG	0.111989	0
B91	B188	TP	Ca	0.292489	0
B91	B192	TP	UA	0.104777	0
B91	B209	TP	Smoking	-0.09538	0
B91	B218	TP	DBP	0.093728	0
B91	B220	TP	SBP	0.098682	0
B91	B155	TP	IgA	0.085224	2.66E-15
B91	B221	TP	Hei	-0.08132	4.49E-14
B91	B194	TP	Cl	-0.0812	4.88E-14
B91	B177	TP	ACR	-0.07947	1.66E-13
B91	B175	TP	ALB-U	-0.07269	1.55E-11
B91	B158	TP	IgM	0.068913	1.64E-10
B91	B136	TP	5'NT	0.06618	8.35E-10
B91	B134	TP	CHE	0.06427	2.51E-09
B91	B178	TP	Ig-U	-0.06386	3.17E-09
B91	B191	TP	Urea	-0.06379	3.29E-09
B91	B140	TP	r-GT	0.062766	5.86E-09
B91	B180	TP	Tf	-0.06252	6.73E-09
B91	B163	TP	SG	-0.05908	4.29E-08
B91	B150	TP	ALP	0.058389	6.16E-08
B91	B120	TP	HCV-cAg	0.057837	8.19E-08
B91	B111	TP	TSH	0.052912	9.31E-07
B92	B101	ALDH2-Glu504Glu	anti-Sc70	-0.27314	0
B92	B104	ALDH2-Glu504Glu	anti-SSB	-0.27314	0
B92	B107	ALDH2-Glu504Glu	AnuA	-0.26218	0
B92	B109	ALDH2-Glu504Glu	AMA-M2	-0.10357	0
B92	B119	ALDH2-Glu504Glu	Hcv-rna	-0.19616	0
B92	B124	ALDH2-Glu504Glu	ACTH	0.204391	0
B92	B138	ALDH2-Glu504Glu	PAB	0.101473	0
B92	B179	ALDH2-Glu504Glu	α1-MG	0.092321	0
B92	B181	ALDH2-Glu504Glu	APTT	-0.12422	0
B92	B183	ALDH2-Glu504Glu	PTA	-0.10756	0
B92	B184	ALDH2-Glu504Glu	INR	-0.20983	0
B92	B185	ALDH2-Glu504Glu	PT	-0.13279	0
B92	B186	ALDH2-Glu504Glu	PFC	0.11306	0
B92	B194	ALDH2-Glu504Glu	Cl	-0.12676	0
B92	B197	ALDH2-Glu504Glu	AG	0.117697	0
B92	B93	ALDH2-Glu504Glu	ALDH2-Glu504Lys	-0.99846	0
B92	B94	ALDH2-Glu504Glu	ALDH2-Lys504Lys	-0.56054	0
B92	B96	ALDH2-Glu504Glu	dsDNA (IIF)	0.155462	0
B92	B99	ALDH2-Glu504Glu	anti-PM-Scl	-0.27314	0
B92	B122	ALDH2-Glu504Glu	C4	-0.08021	9.81E-14
B92	B100	ALDH2-Glu504Glu	anti-Ro52	-0.07809	4.28E-13
B92	B134	ALDH2-Glu504Glu	CHE	0.077516	6.36E-13

B92	B125	ALDH2-Glu504Glu	Cor	0.074842	3.82E-12
B92	B212	ALDH2-Glu504Glu	IOP-L	0.067527	3.77E-10
B92	B121	ALDH2-Glu504Glu	C3	-0.05895	4.60E-08
B92	B155	ALDH2-Glu504Glu	IgA	0.054521	4.31E-07
B92	B200	ALDH2-Glu504Glu	OP	0.053737	6.29E-07
B92	B178	ALDH2-Glu504Glu	Ig-U	0.053002	8.93E-07
B93	B101	ALDH2-Glu504Lys	anti-Sc70	0.255563	0
B93	B104	ALDH2-Glu504Lys	anti-SSB	0.255563	0
B93	B107	ALDH2-Glu504Lys	AnuA	0.245052	0
B93	B109	ALDH2-Glu504Lys	AMA-M2	0.096901	0
B93	B119	ALDH2-Glu504Lys	Hcv-rna	0.182874	0
B93	B124	ALDH2-Glu504Lys	ACTH	-0.19661	0
B93	B138	ALDH2-Glu504Lys	PAB	-0.098	0
B93	B179	ALDH2-Glu504Lys	α1-MG	-0.08907	0
B93	B181	ALDH2-Glu504Lys	APTT	0.116933	0
B93	B183	ALDH2-Glu504Lys	PTA	0.101137	0
B93	B184	ALDH2-Glu504Lys	INR	0.199474	0
B93	B185	ALDH2-Glu504Lys	PT	0.128631	0
B93	B186	ALDH2-Glu504Lys	PFC	-0.109	0
B93	B194	ALDH2-Glu504Lys	Cl	0.122512	0
B93	B197	ALDH2-Glu504Lys	AG	-0.11391	0
B93	B94	ALDH2-Glu504Lys	ALDH2-Lys504Lys	0.513678	0
B93	B96	ALDH2-Glu504Lys	dsDNA (IIF)	-0.17217	0
B93	B99	ALDH2-Glu504Lys	anti-PM-Scl	0.255563	0
B93	B125	ALDH2-Glu504Lys	Cor	-0.07753	6.28E-13
B93	B122	ALDH2-Glu504Lys	C4	0.076864	9.91E-13
B93	B134	ALDH2-Glu504Lys	CHE	-0.07424	5.67E-12
B93	B100	ALDH2-Glu504Lys	anti-Ro52	0.073059	1.22E-11
B93	B212	ALDH2-Glu504Lys	IOP-L	-0.06228	7.66E-09
B93	B121	ALDH2-Glu504Lys	C3	0.056639	1.51E-07
B93	B155	ALDH2-Glu504Lys	IgA	-0.05333	7.64E-07
B94	B100	ALDH2-Lys504Lys	anti-Ro52	0.117053	0
B94	B101	ALDH2-Lys504Lys	anti-Sc70	0.408833	0
B94	B104	ALDH2-Lys504Lys	anti-SSB	0.408833	0
B94	B107	ALDH2-Lys504Lys	AnuA	0.396363	0
B94	B109	ALDH2-Lys504Lys	AMA-M2	0.155153	0
B94	B119	ALDH2-Lys504Lys	Hcv-rna	0.30355	0
B94	B122	ALDH2-Lys504Lys	C4	0.093056	0
B94	B124	ALDH2-Lys504Lys	ACTH	-0.22589	0
B94	B134	ALDH2-Lys504Lys	CHE	-0.09048	0
B94	B138	ALDH2-Lys504Lys	PAB	-0.10628	0
B94	B157	ALDH2-Lys504Lys	IgG	0.107041	0
B94	B179	ALDH2-Lys504Lys	α1-MG	-0.09814	0
B94	B181	ALDH2-Lys504Lys	APTT	0.175443	0

B94	B183	ALDH2-Lys504Lys	PTA	0.153612	0
B94	B184	ALDH2-Lys504Lys	INR	0.267246	0
B94	B185	ALDH2-Lys504Lys	PT	0.133437	0
B94	B186	ALDH2-Lys504Lys	PFC	-0.12124	0
B94	B194	ALDH2-Lys504Lys	Cl	0.13139	0
B94	B197	ALDH2-Lys504Lys	AG	-0.11978	0
B94	B212	ALDH2-Lys504Lys	IOP-L	-0.11453	0
B94	B96	ALDH2-Lys504Lys	dsDNA (IIF)	0.165605	0
B94	B99	ALDH2-Lys504Lys	anti-PM-Scl	0.408833	0
B94	B211	ALDH2-Lys504Lys	IOP-R	-0.08718	4.44E-16
B94	B193	ALDH2-Lys504Lys	K	0.087093	6.66E-16
B94	B200	ALDH2-Lys504Lys	OP	-0.06994	8.71E-11
B94	B121	ALDH2-Lys504Lys	C3	0.066094	8.78E-10
B94	B196	ALDH2-Lys504Lys	TCO2	0.062828	5.66E-09
B94	B108	ALDH2-Lys504Lys	Anti-Cyclin	0.059977	2.67E-08
B94	B178	ALDH2-Lys504Lys	Ig-U	-0.0597	3.10E-08
B94	B216	ALDH2-Lys504Lys	BMI	-0.05849	5.85E-08
B94	B199	ALDH2-Lys504Lys	Mg	0.058167	6.91E-08
B94	B221	ALDH2-Lys504Lys	Hei	-0.05745	9.99E-08
B94	B115	ALDH2-Lys504Lys	TSH TT3 TT4(TT3)	0.056054	2.02E-07
B94	B204	ALDH2-Lys504Lys	Drinking	-0.05566	2.46E-07
B94	B189	ALDH2-Lys504Lys	eGFB	-0.05522	3.05E-07
B94	B95	ALDH2-Lys504Lys	anti-cenpb	0.054383	4.60E-07
B95	B195	anti-cenpb	Na	0.077948	4.73E-13
B95	B193	anti-cenpb	K	0.063178	4.65E-09
B95	B134	anti-cenpb	CHE	-0.05634	1.75E-07
B96	B101	dsDNA (IIF)	anti-Sc70	0.25428	0
B96	B104	dsDNA (IIF)	anti-SSB	0.254279	0
B96	B107	dsDNA (IIF)	AnuA	0.255492	0
B96	B109	dsDNA (IIF)	AMA-M2	0.096322	0
B96	B110	dsDNA (IIF)	AHA	-0.35554	0
B96	B119	dsDNA (IIF)	Hcv-rna	0.180161	0
B96	B182	dsDNA (IIF)	TT	0.121242	0
B96	B194	dsDNA (IIF)	Cl	0.162303	0
B96	B197	dsDNA (IIF)	AG	-0.10253	0
B96	B97	dsDNA (IIF)	dsDNA	-0.52548	0
B96	B99	dsDNA (IIF)	anti-PM-Scl	0.25428	0
B96	B193	dsDNA (IIF)	K	0.063446	4.01E-09
B96	B184	dsDNA (IIF)	INR	0.063048	5.00E-09
B96	B156	dsDNA (IIF)	IgE	-0.06273	5.97E-09
B96	B186	dsDNA (IIF)	PFC	-0.05522	3.06E-07
B96	B181	dsDNA (IIF)	APTT	0.054337	4.71E-07
B96	B195	dsDNA (IIF)	Na	0.053571	6.81E-07
B96	B103	dsDNA (IIF)	anti-SSA/Ro	0.05352	6.98E-07

B97	B101	dsDNA	anti-Sc70	-0.09071	0
B97	B104	dsDNA	anti-SSB	-0.09071	0
B97	B107	dsDNA	AnuA	-0.09158	0
B97	B110	dsDNA	AHA	0.939101	0
B97	B119	dsDNA	Hcv-rna	-0.14071	0
B97	B150	dsDNA	ALP	0.091821	0
B97	B194	dsDNA	Cl	-0.20482	0
B97	B195	dsDNA	Na	-0.12632	0
B97	B228	dsDNA	OS	0.118216	0
B97	B99	dsDNA	anti-PM-Scl	-0.09071	0
B97	B197	dsDNA	AG	0.06615	8.50E-10
B97	B200	dsDNA	OP	-0.0649	1.75E-09
B98	B124	anti-Jo-1	ACTH	-0.10257	0
B98	B125	anti-Jo-1	Cor	0.337758	0
B98	B181	anti-Jo-1	APTT	-0.16743	0
B98	B184	anti-Jo-1	INR	-0.16255	0
B98	B185	anti-Jo-1	PT	-0.10038	0
B98	B195	anti-Jo-1	Na	-0.10928	0
B98	B225	anti-Jo-1	hPBG	0.175819	0
B98	B199	anti-Jo-1	Mg	-0.07898	2.32E-13
B98	B194	anti-Jo-1	Cl	-0.07863	2.96E-13
B98	B172	anti-Jo-1	KB	0.075533	2.42E-12
B98	B151	anti-Jo-1	Anti-CCP	0.066462	7.08E-10
B98	B107	anti-Jo-1	AnuA	-0.0653	1.39E-09
B98	B101	anti-Jo-1	anti-Sc70	-0.0647	1.97E-09
B98	B104	anti-Jo-1	anti-SSB	-0.0647	1.97E-09
B98	B99	anti-Jo-1	anti-PM-Scl	-0.0647	1.97E-09
B98	B138	anti-Jo-1	PAB	0.06305	5.00E-09
B98	B183	anti-Jo-1	PTA	-0.05731	1.07E-07
B99	B100	anti-PM-Scl	anti-Ro52	0.163075	0
B99	B104	anti-PM-Scl	anti-SSB	1	0
B99	B107	anti-PM-Scl	AnuA	0.995262	0
B99	B109	anti-PM-Scl	AMA-M2	0.285121	0
B99	B115	anti-PM-Scl	TSH TT3 TT4(TT3)	0.097149	0
B99	B119	anti-PM-Scl	Hcv-rna	0.22665	0
B99	B124	anti-PM-Scl	ACTH	-0.1739	0
B99	B138	anti-PM-Scl	PAB	-0.12155	0
B99	B155	anti-PM-Scl	IgA	-0.11956	0
B99	B157	anti-PM-Scl	IgG	0.102519	0
B99	B158	anti-PM-Scl	IgM	0.152403	0
B99	B181	anti-PM-Scl	APTT	0.174055	0
B99	B182	anti-PM-Scl	TT	0.095817	0
B99	B184	anti-PM-Scl	INR	0.270459	0
B99	B185	anti-PM-Scl	PT	0.1744	0

B99	B186	anti-PM-Scl	PFC	-0.11045	0
B99	B194	anti-PM-Scl	Cl	0.185271	0
B99	B195	anti-PM-Scl	Na	0.134201	0
B99	B196	anti-PM-Scl	TCO2	0.119685	0
B99	B197	anti-PM-Scl	AG	-0.14837	0
B99	B199	anti-PM-Scl	Mg	0.168027	0
B99	B200	anti-PM-Scl	OP	-0.11588	0
B99	B179	anti-PM-Scl	α1-MG	-0.08204	2.66E-14
B99	B136	anti-PM-Scl	5'NT	-0.08078	6.57E-14
B99	B178	anti-PM-Scl	Ig-U	-0.07759	6.05E-13
B99	B139	anti-PM-Scl	ADA	-0.07705	8.74E-13
B99	B180	anti-PM-Scl	Tf	-0.0763	1.45E-12
B99	B125	anti-PM-Scl	Cor	-0.0744	5.11E-12
B99	B189	anti-PM-Scl	eGFB	-0.07423	5.72E-12
B99	B121	anti-PM-Scl	C3	-0.07191	2.54E-11
B99	B212	anti-PM-Scl	IOP-L	-0.06297	5.22E-09
B99	B102	anti-PM-Scl	anti-Sm	-0.06065	1.86E-08
B99	B103	anti-PM-Scl	anti-SSA/Ro	0.056307	1.78E-07
B99	B211	anti-PM-Scl	IOP-R	-0.05567	2.45E-07

Supplementary Table 5. PEI Correlations in Hypertension+Coronary

Row	Column	Row	Column	Cor	*P*
B1	B132	Gender	Dbil	-0.18785	0
B1	B161	Gender	LEU	0.205613	0
B1	B163	Gender	SG	-0.26225	0
B1	B174	Gender	Urea/Cr	0.367654	0
B1	B190	Gender	Cre	-0.41531	0
B1	B192	Gender	UA	-0.34775	0
B1	B204	Gender	Drinking	-0.21708	0
B1	B206	Gender	Waist	-0.29726	0
B1	B207	Gender	WHR	-0.22	0
B1	B209	Gender	Smoking	-0.22765	0
B1	B210	Gender	Hip	-0.18635	0
B1	B216	Gender	BMI	-0.49505	0
B1	B221	Gender	Hei	-0.7245	0
B1	B222	Gender	NS	-0.63871	0
B1	B42	Gender	MON	-0.23091	0
B1	B45	Gender	MCHC	-0.2022	0
B1	B46	Gender	RBC Count	-0.37808	0
B1	B51	Gender	Hb	-0.46703	0
B1	B72	Gender	HDL-C	0.294963	0
B1	B77	Gender	TC	0.226815	0
B1	B40	Gender	WBC	-0.17467	1.11E-15
B1	B173	Gender	NTT	0.165963	2.82E-14
B1	B133	Gender	TBIL	-0.16089	1.69E-13
B1	B141	Gender	AST/ALT	0.156217	8.43E-13
B1	B129	Gender	IBIL	-0.13756	3.16E-10
B1	B41	Gender	MON％	-0.1328	1.27E-09
B1	B219	Gender	OT	-0.12045	3.75E-08
B1	B50	Gender	eo%	-0.11904	5.41E-08
B1	B2	Gender	Age	0.118513	6.20E-08
B1	B130	Gender	GLB	0.117726	7.58E-08
B1	B127	Gender	A-G ratio	-0.11674	9.75E-08
B1	B52	Gender	PLT/BPC	0.113118	2.40E-07
B1	B191	Gender	Urea	-0.11259	2.74E-07
B1	B58	Gender	HCT	-0.11187	3.26E-07
B1	B201	Gender	e-habits	-0.11181	3.31E-07
B1	B3	Gender	Hcy	-0.11145	3.61E-07
B1	B70	Gender	LDL-C	0.110486	4.56E-07
B1	B205	Gender	KDA	0.108006	8.21E-07
B10	B11	PGⅠ	PGI/PGII	0.20813	0
B10	B12	PGⅠ	PG II	0.453391	0
B10	B145	PGⅠ	BGP	-0.13924	1.91E-10

B10	B144	PG I	Total-PINP	-0.1187	5.90E-08
B10	B189	PG I	eGFB	-0.10822	7.82E-07
B10	B143	PG I	β-CTx	-0.10761	9.02E-07
B100	B102	anti-Ro52	anti-Sm	-0.27973	0
B100	B105	anti-Ro52	anti-U1RNP	-0.33755	0
B100	B107	anti-Ro52	AnuA	0.999998	0
B100	B108	anti-Ro52	Anti-Cyclin	-0.22672	0
B100	B109	anti-Ro52	AMA-M2	-0.26181	0
B100	B125	anti-Ro52	Cor	0.234516	0
B100	B154	anti-Ro52	RF	0.204755	0
B100	B183	anti-Ro52	PTA	0.273181	0
B100	B155	anti-Ro52	IgA	-0.16404	5.60E-14
B100	B156	anti-Ro52	IgE	-0.14475	3.52E-11
B100	B181	anti-Ro52	APTT	0.13878	2.19E-10
B100	B195	anti-Ro52	Na	0.137023	3.70E-10
B100	B103	anti-Ro52	anti-SSA/Ro	-0.13644	4.40E-10
B100	B137	anti-Ro52	AFU	-0.12881	3.93E-09
B100	B198	anti-Ro52	P	-0.12517	1.07E-08
B100	B139	anti-Ro52	ADA	0.118712	5.89E-08
B100	B138	anti-Ro52	PAB	0.113683	2.09E-07
B100	B185	anti-Ro52	PT	-0.10972	5.48E-07
B101	B102	anti-Sc70	anti-Sm	-0.27973	0
B101	B104	anti-Sc70	anti-SSB	1	0
B101	B105	anti-Sc70	anti-U1RNP	-0.33755	0
B101	B107	anti-Sc70	AnuA	0.999999	0
B101	B108	anti-Sc70	Anti-Cyclin	-0.22672	0
B101	B109	anti-Sc70	AMA-M2	-0.26181	0
B101	B125	anti-Sc70	Cor	0.234516	0
B101	B154	anti-Sc70	RF	0.204755	0
B101	B183	anti-Sc70	PTA	0.273181	0
B101	B155	anti-Sc70	IgA	-0.16404	5.60E-14
B101	B156	anti-Sc70	IgE	-0.14475	3.52E-11
B101	B181	anti-Sc70	APTT	0.13878	2.19E-10
B101	B195	anti-Sc70	Na	0.137023	3.70E-10
B101	B103	anti-Sc70	anti-SSA/Ro	-0.13644	4.40E-10
B101	B137	anti-Sc70	AFU	-0.12881	3.93E-09
B101	B198	anti-Sc70	P	-0.12517	1.07E-08
B101	B139	anti-Sc70	ADA	0.118712	5.89E-08
B101	B138	anti-Sc70	PAB	0.113683	2.09E-07
B101	B185	anti-Sc70	PT	-0.10972	5.48E-07
B102	B104	anti-Sm	anti-SSB	-0.27973	0
B102	B105	anti-Sm	anti-U1RNP	0.970103	0
B102	B106	anti-Sm	anti-P	-0.27973	0
B102	B107	anti-Sm	AnuA	-0.27973	0

B102	B110	anti-Sm	AHA	-0.27973	0
B102	B124	anti-Sm	ACTH	-0.36402	0
B102	B125	anti-Sm	Cor	-0.44287	0
B102	B138	anti-Sm	PAB	-0.30825	0
B102	B156	anti-Sm	IgE	0.282049	0
B102	B185	anti-Sm	PT	0.306909	0
B102	B228	anti-Sm	OS	0.179948	0
B102	B120	anti-Sm	HCV-cAg	-0.17165	3.55E-15
B102	B186	anti-Sm	PFC	0.150587	5.46E-12
B102	B183	anti-Sm	PTA	-0.14799	1.26E-11
B102	B179	anti-Sm	α1-MG	0.14166	9.16E-11
B102	B134	anti-Sm	CHE	-0.13424	8.38E-10
B102	B181	anti-Sm	APTT	0.125827	8.94E-09
B102	B182	anti-Sm	TT	-0.11359	2.14E-07
B103	B158	anti-SSA/Ro	IgM	0.386089	0
B103	B178	anti-SSA/Ro	Ig-U	0.185639	0
B103	B179	anti-SSA/Ro	α1-MG	0.256249	0
B103	B180	anti-SSA/Ro	Tf	0.329367	0
B103	B104	anti-SSA/Ro	anti-SSB	-0.13644	4.40E-10
B103	B106	anti-SSA/Ro	anti-P	-0.13644	4.40E-10
B103	B107	anti-SSA/Ro	AnuA	-0.13644	4.40E-10
B103	B110	anti-SSA/Ro	AHA	-0.13644	4.40E-10
B103	B151	anti-SSA/Ro	Anti-CCP	0.124293	1.35E-08
B103	B177	anti-SSA/Ro	ACR	0.110605	4.43E-07
B104	B105	anti-SSB	anti-U1RNP	-0.33755	0
B104	B106	anti-SSB	anti-P	1	0
B104	B107	anti-SSB	AnuA	0.999998	0
B104	B108	anti-SSB	Anti-Cyclin	-0.22672	0
B104	B109	anti-SSB	AMA-M2	-0.26181	0
B104	B110	anti-SSB	AHA	1	0
B104	B125	anti-SSB	Cor	0.234516	0
B104	B154	anti-SSB	RF	0.204755	0
B104	B183	anti-SSB	PTA	0.273181	0
B104	B155	anti-SSB	IgA	-0.16404	5.60E-14
B104	B156	anti-SSB	IgE	-0.14475	3.52E-11
B104	B181	anti-SSB	APTT	0.13878	2.19E-10
B104	B195	anti-SSB	Na	0.137023	3.70E-10
B104	B137	anti-SSB	AFU	-0.12881	3.93E-09
B104	B198	anti-SSB	P	-0.12517	1.07E-08
B104	B139	anti-SSB	ADA	0.118712	5.89E-08
B104	B138	anti-SSB	PAB	0.113683	2.09E-07
B104	B185	anti-SSB	PT	-0.10972	5.48E-07
B105	B106	anti-U1RNP	anti-P	-0.33755	0
B105	B107	anti-U1RNP	AnuA	-0.33755	0

B105	B108	anti-U1RNP	Anti-Cyclin	0.213195	0
B105	B110	anti-U1RNP	AHA	-0.33755	0
B105	B124	anti-U1RNP	ACTH	-0.27546	0
B105	B125	anti-U1RNP	Cor	-0.43629	0
B105	B138	anti-U1RNP	PAB	-0.27426	0
B105	B156	anti-U1RNP	IgE	0.273801	0
B105	B185	anti-U1RNP	PT	0.282689	0
B105	B183	anti-U1RNP	PTA	-0.17855	2.22E-16
B105	B228	anti-U1RNP	OS	0.174314	1.33E-15
B105	B120	anti-U1RNP	HCV-cAg	-0.16572	3.06E-14
B105	B179	anti-U1RNP	α1-MG	0.137064	3.66E-10
B105	B134	anti-U1RNP	CHE	-0.13633	4.55E-10
B105	B186	anti-U1RNP	PFC	0.130221	2.65E-09
B105	B195	anti-U1RNP	Na	0.111044	3.98E-07
B106	B107	anti-P	AnuA	0.999999	0
B106	B108	anti-P	Anti-Cyclin	-0.22672	0
B106	B109	anti-P	AMA-M2	-0.26181	0
B106	B125	anti-P	Cor	0.234516	0
B106	B154	anti-P	RF	0.204755	0
B106	B183	anti-P	PTA	0.273181	0
B106	B155	anti-P	IgA	-0.16404	5.60E-14
B106	B156	anti-P	IgE	-0.14475	3.52E-11
B106	B181	anti-P	APTT	0.13878	2.19E-10
B106	B195	anti-P	Na	0.137023	3.70E-10
B106	B137	anti-P	AFU	-0.12881	3.93E-09
B106	B198	anti-P	P	-0.12517	1.07E-08
B106	B139	anti-P	ADA	0.118712	5.89E-08
B106	B138	anti-P	PAB	0.113683	2.09E-07
B106	B185	anti-P	PT	-0.10972	5.48E-07
B107	B108	AnuA	Anti-Cyclin	-0.22671	0
B107	B109	AnuA	AMA-M2	-0.26181	0
B107	B110	AnuA	AHA	0.999998	0
B107	B125	AnuA	Cor	0.234516	0
B107	B154	AnuA	RF	0.204754	0
B107	B183	AnuA	PTA	0.273181	0
B107	B155	AnuA	IgA	-0.16404	5.60E-14
B107	B156	AnuA	IgE	-0.14475	3.52E-11
B107	B181	AnuA	APTT	0.13878	2.19E-10
B107	B195	AnuA	Na	0.137023	3.70E-10
B107	B137	AnuA	AFU	-0.12881	3.93E-09
B107	B198	AnuA	P	-0.12517	1.07E-08
B107	B139	AnuA	ADA	0.118712	5.89E-08
B107	B138	AnuA	PAB	0.113683	2.09E-07
B107	B185	AnuA	PT	-0.10972	5.48E-07

B108	B110	Anti-Cyclin	AHA	-0.22672	0
B108	B124	Anti-Cyclin	ACTH	0.333032	0
B108	B135	Anti-Cyclin	TBA	0.348353	0
B108	B181	Anti-Cyclin	APTT	-0.31556	0
B108	B198	Anti-Cyclin	P	0.266175	0
B108	B184	Anti-Cyclin	INR	-0.17385	1.55E-15
B108	B151	Anti-Cyclin	Anti-CCP	-0.16694	2.00E-14
B108	B195	Anti-Cyclin	Na	0.15744	5.57E-13
B108	B196	Anti-Cyclin	TCO2	0.141027	1.11E-10
B108	B183	Anti-Cyclin	PTA	-0.13104	2.10E-09
B108	B137	Anti-Cyclin	AFU	0.12105	3.21E-08
B108	B182	Anti-Cyclin	TT	0.119197	5.19E-08
B108	B138	Anti-Cyclin	PAB	0.113653	2.11E-07
B108	B139	Anti-Cyclin	ADA	-0.11059	4.44E-07
B109	B110	AMA-M2	AHA	-0.26181	0
B109	B151	AMA-M2	Anti-CCP	0.220051	0
B109	B198	AMA-M2	P	0.246809	0
B109	B120	AMA-M2	HCV-cAg	-0.17068	5.11E-15
B109	B196	AMA-M2	TCO2	-0.16256	9.46E-14
B109	B199	AMA-M2	Mg	-0.15659	7.43E-13
B109	B197	AMA-M2	AG	0.149153	8.69E-12
B109	B137	AMA-M2	AFU	0.133206	1.13E-09
B109	B179	AMA-M2	α1-MG	-0.11733	8.37E-08
B109	B195	AMA-M2	Na	-0.11019	4.89E-07
B11	B12	PGI/PGII	PG II	-0.51051	0
B11	B14	PGI/PGII	G-17	-0.22661	0
B11	B80	PGI/PGII	anti-HP IgG	-0.22687	0
B110	B125	AHA	Cor	0.234516	0
B110	B154	AHA	RF	0.204755	0
B110	B183	AHA	PTA	0.273181	0
B110	B155	AHA	IgA	-0.16404	5.60E-14
B110	B156	AHA	IgE	-0.14475	3.52E-11
B110	B181	AHA	APTT	0.13878	2.19E-10
B110	B195	AHA	Na	0.137023	3.70E-10
B110	B137	AHA	AFU	-0.12881	3.93E-09
B110	B198	AHA	P	-0.12517	1.07E-08
B110	B139	AHA	ADA	0.118712	5.89E-08
B110	B138	AHA	PAB	0.113683	2.09E-07
B110	B185	AHA	PT	-0.10972	5.48E-07
B111	B112	TSH	FT4	-0.18086	0
B112	B114	FT4	TSH TT3 TT4(TT4)	0.417322	0
B112	B125	FT4	Cor	0.115093	1.47E-07
B113	B114	FT3	TSH TT3 TT4(TT4)	0.218855	0
B113	B115	FT3	TSH TT3 TT4(TT3)	0.402109	0

B113	B126	FT3	Alb	0.155879	9.45E-13
B113	B176	FT3	Ucr	0.137811	2.93E-10
B114	B115	TSH TT3 TT4(TT4)	TSH TT3 TT4(TT3)	0.425703	0
B114	B125	TSH TT3 TT4(TT4)	Cor	0.127745	5.28E-09
B115	B134	TSH TT3 TT4(TT3)	CHE	0.17635	4.44E-16
B115	B196	TSH TT3 TT4(TT3)	TCO2	0.1215	2.85E-08
B115	B135	TSH TT3 TT4(TT3)	TBA	-0.11678	9.65E-08
B116	B117	TT3	TT4	0.542235	0
B119	B120	Hcv-rna	HCV-cAg	0.386606	0
B119	B125	Hcv-rna	Cor	0.232793	0
B119	B135	Hcv-rna	TBA	-0.26329	0
B119	B137	Hcv-rna	AFU	-0.22152	0
B119	B199	Hcv-rna	Mg	0.165722	3.06E-14
B119	B194	Hcv-rna	Cl	-0.13295	1.22E-09
B119	B124	Hcv-rna	ACTH	0.130491	2.45E-09
B119	B134	Hcv-rna	CHE	0.109902	5.24E-07
B12	B84	PG II	C-PSA	0.18918	0
B12	B80	PG II	anti-HP IgG	0.151688	3.81E-12
B120	B121	HCV-cAg	C3	-0.38661	0
B120	B122	HCV-cAg	C4	-0.38661	0
B120	B152	HCV-cAg	ACA-Ig(G-A-M)	-0.38672	0
B120	B169	HCV-cAg	U-CRY	-0.38661	0
B120	B125	HCV-cAg	Cor	0.166303	2.49E-14
B120	B136	HCV-cAg	5'NT	-0.15559	1.04E-12
B120	B146	HCV-cAg	CysC	-0.12751	5.63E-09
B120	B134	HCV-cAg	CHE	0.124535	1.27E-08
B120	B199	HCV-cAg	Mg	0.118731	5.86E-08
B120	B124	HCV-cAg	ACTH	0.113911	1.98E-07
B120	B137	HCV-cAg	AFU	-0.10911	6.32E-07
B121	B125	C3	Cor	-0.23279	0
B121	B135	C3	TBA	0.263295	0
B121	B137	C3	AFU	0.221518	0
B121	B199	C3	Mg	-0.16572	3.06E-14
B121	B194	C3	Cl	0.132954	1.22E-09
B121	B124	C3	ACTH	-0.13049	2.45E-09
B121	B134	C3	CHE	-0.1099	5.24E-07
B122	B125	C4	Cor	-0.23279	0
B122	B135	C4	TBA	0.263296	0
B122	B137	C4	AFU	0.221519	0
B122	B169	C4	U-CRY	0.999992	0
B122	B199	C4	Mg	-0.16572	3.06E-14
B122	B194	C4	Cl	0.132955	1.21E-09
B122	B124	C4	ACTH	-0.13049	2.45E-09
B122	B134	C4	CHE	-0.1099	5.24E-07

B123	B138	hs-CRP1	PAB	-0.19699	0
B123	B139	hs-CRP1	ADA	0.272404	0
B123	B193	hs-CRP1	K	-0.27	0
B123	B194	hs-CRP1	Cl	-0.17772	4.44E-16
B123	B224	hs-CRP1	pmh	0.115258	1.41E-07
B123	B196	hs-CRP1	TCO2	0.113578	2.15E-07
B124	B125	ACTH	Cor	0.608604	0
B124	B135	ACTH	TBA	0.215726	0
B124	B181	ACTH	APTT	-0.30335	0
B124	B186	ACTH	PFC	0.178117	4.44E-16
B124	B199	ACTH	Mg	0.143514	5.17E-11
B124	B152	ACTH	ACA-Ig(G-A-M)	-0.13053	2.43E-09
B124	B169	ACTH	U-CRY	-0.13049	2.45E-09
B124	B136	ACTH	5'NT	-0.13028	2.60E-09
B124	B140	ACTH	r-GT	-0.12026	3.95E-08
B124	B156	ACTH	IgE	-0.11478	1.59E-07
B125	B152	Cor	ACA-Ig(G-A-M)	-0.23286	0
B125	B169	Cor	U-CRY	-0.2328	0
B125	B181	Cor	APTT	-0.28438	0
B125	B185	Cor	PT	-0.17457	1.33E-15
B125	B138	Cor	PAB	0.164916	4.09E-14
B125	B182	Cor	TT	-0.15128	4.36E-12
B125	B199	Cor	Mg	0.147547	1.45E-11
B125	B194	Cor	Cl	-0.13967	1.68E-10
B126	B127	Alb	A-G ratio	0.49192	0
B126	B188	Alb	Ca	0.278822	0
B126	B189	Alb	eGFB	0.252026	0
B126	B130	Alb	GLB	-0.16719	1.82E-14
B126	B190	Alb	Cre	-0.16576	3.02E-14
B126	B191	Alb	Urea	-0.1619	1.19E-13
B126	B141	Alb	AST/ALT	-0.15635	8.06E-13
B126	B216	Alb	BMI	0.128323	4.50E-09
B126	B221	Alb	Hei	0.123588	1.64E-08
B126	B134	Alb	CHE	0.109603	5.63E-07
B127	B130	A-G ratio	GLB	-0.90608	0
B127	B221	A-G ratio	Hei	0.174007	1.33E-15
B127	B189	A-G ratio	eGFB	0.161193	1.53E-13
B127	B132	A-G ratio	Dbil	0.144548	3.75E-11
B127	B209	A-G ratio	Smoking	0.131251	1.98E-09
B127	B133	A-G ratio	TBIL	0.126218	8.03E-09
B127	B163	A-G ratio	SG	0.119485	4.82E-08
B127	B216	A-G ratio	BMI	0.11941	4.92E-08
B127	B129	A-G ratio	IBIL	0.108413	7.46E-07
B128	B131	ALT	AST	0.750504	0

B128	B140	ALT	r-GT	0.199166	0
B128	B141	ALT	AST/ALT	-0.36467	0
B128	B216	ALT	BMI	0.112636	2.71E-07
B129	B132	IBIL	Dbil	0.666556	0
B129	B133	IBIL	TBIL	0.979071	0
B130	B132	GLB	Dbil	-0.14872	9.99E-12
B130	B221	GLB	Hei	-0.14855	1.06E-11
B130	B209	GLB	Smoking	-0.13743	3.29E-10
B130	B133	GLB	TBIL	-0.12201	2.49E-08
B130	B220	GLB	SBP	0.117971	7.12E-08
B130	B192	GLB	UA	0.11675	9.72E-08
B130	B163	GLB	SG	-0.11011	4.99E-07
B131	B140	AST	r-GT	0.266777	0
B132	B133	Dbil	TBIL	0.80421	0
B132	B221	Dbil	Hei	0.127175	6.18E-09
B133	B221	TBIL	Hei	0.117291	8.47E-08
B134	B136	CHE	5'NT	-0.21374	0
B134	B138	CHE	PAB	0.386548	0
B134	B159	CHE	β2-MG	-0.19401	0
B134	B194	CHE	Cl	-0.15947	2.78E-13
B134	B139	CHE	ADA	-0.15669	7.17E-13
B134	B185	CHE	PT	-0.15028	6.03E-12
B134	B196	CHE	TCO2	0.14751	1.47E-11
B134	B137	CHE	AFU	0.126977	6.53E-09
B134	B152	CHE	ACA-Ig(G-A-M)	-0.10993	5.20E-07
B134	B169	CHE	U-CRY	-0.1099	5.24E-07
B135	B137	TBA	AFU	0.19707	0
B135	B152	TBA	ACA-Ig(G-A-M)	0.263366	0
B135	B169	TBA	U-CRY	0.263298	0
B135	B195	TBA	Na	0.24905	0
B135	B200	TBA	OP	0.218785	0
B135	B139	TBA	ADA	0.172302	2.66E-15
B135	B159	TBA	β2-MG	-0.15388	1.85E-12
B135	B196	TBA	TCO2	0.145552	2.74E-11
B135	B138	TBA	PAB	-0.12824	4.60E-09
B135	B181	TBA	APTT	-0.10724	9.84E-07
B136	B198	5'NT	P	0.246082	0
B136	B140	5'NT	r-GT	0.170233	6.00E-15
B136	B194	5'NT	Cl	-0.12484	1.17E-08
B136	B200	5'NT	OP	0.121488	2.86E-08
B136	B197	5'NT	AG	0.114527	1.70E-07
B136	B199	5'NT	Mg	-0.10882	6.79E-07
B137	B152	AFU	ACA-Ig(G-A-M)	0.221578	0
B137	B169	AFU	U-CRY	0.221521	0

B137	B196	AFU	TCO2	0.265338	0
B137	B185	AFU	PT	-0.17593	6.66E-16
B137	B194	AFU	Cl	-0.16059	1.88E-13
B137	B199	AFU	Mg	-0.15805	4.53E-13
B137	B159	AFU	β2-MG	-0.1502	6.19E-12
B137	B180	AFU	Tf	0.143721	4.85E-11
B137	B178	AFU	Ig-U	0.139119	1.98E-10
B138	B139	PAB	ADA	-0.43427	0
B138	B178	PAB	Ig-U	-0.24877	0
B138	B180	PAB	Tf	-0.22637	0
B138	B185	PAB	PT	-0.27079	0
B138	B184	PAB	INR	-0.16021	2.15E-13
B138	B179	PAB	α1-MG	-0.1534	2.17E-12
B138	B182	PAB	TT	0.13944	1.80E-10
B138	B159	PAB	β2-MG	-0.13797	2.80E-10
B138	B181	PAB	APTT	-0.12009	4.12E-08
B138	B193	PAB	K	0.119469	4.84E-08
B138	B186	PAB	PFC	-0.10778	8.67E-07
B139	B178	ADA	Ig-U	0.191453	0
B139	B200	ADA	OP	0.193705	0
B139	B180	ADA	Tf	0.177265	4.44E-16
B139	B182	ADA	TT	-0.17637	4.44E-16
B139	B193	ADA	K	-0.17701	4.44E-16
B139	B197	ADA	AG	0.162695	9.02E-14
B139	B195	ADA	Na	0.162431	9.90E-14
B139	B199	ADA	Mg	0.142688	6.68E-11
B139	B186	ADA	PFC	0.112399	2.87E-07
B14	B22	G-17	CD3+CD8+(T)	-0.17994	0
B14	B19	G-17	CD3+CD4+/CD3+CD8+	0.151762	3.72E-12
B14	B25	G-17	CK	0.116216	1.11E-07
B140	B150	r-GT	ALP	0.160021	2.29E-13
B140	B141	r-GT	AST/ALT	-0.1559	9.38E-13
B140	B216	r-GT	BMI	0.141286	1.03E-10
B140	B184	r-GT	INR	-0.13861	2.31E-10
B140	B206	r-GT	Waist	0.120067	4.15E-08
B140	B215	r-GT	BMI	0.113729	2.07E-07
B140	B192	r-GT	UA	0.110706	4.32E-07
B141	B206	AST/ALT	Waist	-0.19087	0
B141	B215	AST/ALT	BMI	-0.18206	0
B141	B216	AST/ALT	BMI	-0.24878	0
B141	B221	AST/ALT	Hei	-0.19006	0
B141	B222	AST/ALT	NS	-0.20112	0
B141	B207	AST/ALT	WHR	-0.13487	6.98E-10
B141	B210	AST/ALT	Hip	-0.13485	7.02E-10

B142	B151	25-(OH)D	Anti-CCP	-0.18964	0
B142	B168	25-(OH)D	NMP22	0.40179	0
B142	B177	25-(OH)D	ACR	0.172301	2.66E-15
B142	B175	25-(OH)D	ALB-U	0.171105	4.44E-15
B143	B144	β-CTx	Total-PINP	0.833381	0
B143	B145	β-CTx	BGP	0.455304	0
B143	B151	β-CTx	Anti-CCP	-0.15148	4.08E-12
B144	B145	Total-PINP	BGP	0.499681	0
B144	B177	Total-PINP	ACR	0.187788	0
B144	B175	Total-PINP	ALB-U	0.171765	3.33E-15
B144	B151	Total-PINP	Anti-CCP	-0.11607	1.15E-07
B146	B190	CysC	Cre	0.17976	0
B146	B200	CysC	OP	0.163683	6.35E-14
B146	B194	CysC	Cl	0.146078	2.32E-11
B146	B189	CysC	eGFB	-0.13956	1.73E-10
B146	B191	CysC	Urea	0.137033	3.69E-10
B146	B192	CysC	UA	0.134515	7.74E-10
B146	B179	CysC	α1-MG	0.134207	8.46E-10
B148	B149	Anti_TPO	TGAb	0.489007	0
B15	B100	CD3-CD19+(B cell)	anti-Ro52	0.434097	0
B15	B101	CD3-CD19+(B cell)	anti-Sc70	0.434097	0
B15	B102	CD3-CD19+(B cell)	anti-Sm	-0.74637	0
B15	B104	CD3-CD19+(B cell)	anti-SSB	0.434097	0
B15	B105	CD3-CD19+(B cell)	anti-U1RNP	-0.77701	0
B15	B106	CD3-CD19+(B cell)	anti-P	0.434097	0
B15	B107	CD3-CD19+(B cell)	AnuA	0.434096	0
B15	B108	CD3-CD19+(B cell)	Anti-Cyclin	-0.18818	0
B15	B109	CD3-CD19+(B cell)	AMA-M2	-0.27735	0
B15	B110	CD3-CD19+(B cell)	AHA	0.434097	0
B15	B120	CD3-CD19+(B cell)	HCV-cAg	0.227238	0
B15	B124	CD3-CD19+(B cell)	ACTH	0.253964	0
B15	B125	CD3-CD19+(B cell)	Cor	0.417769	0
B15	B136	CD3-CD19+(B cell)	5'NT	-0.23975	0
B15	B138	CD3-CD19+(B cell)	PAB	0.266068	0
B15	B16	CD3-CD19+(B cell)	CD3+T	0.502518	0
B15	B18	CD3-CD19+(B cell)	CD3+CD4+helper(inducer)T	0.302353	0
B15	B181	CD3-CD19+(B cell)	APTT	-0.18581	0
B15	B185	CD3-CD19+(B cell)	PT	-0.31048	0
B15	B186	CD3-CD19+(B cell)	PFC	-0.1991	0
B15	B19	CD3-CD19+(B cell)	CD3+CD4+/CD3+CD8+	0.256275	0
B15	B198	CD3-CD19+(B cell)	P	-0.25879	0
B15	B20	CD3-CD19+(B cell)	CD3+CD4+CD8+	0.300325	0
B15	B21	CD3-CD19+(B cell)	CD3+CD4-CD8-	0.199171	0
B15	B23	CD3-CD19+(B cell)	CD3-CD16+CD56+(NK)	-0.66868	0

B15	B55	CD3-CD19+(B cell)	TK	0.237744	0
B15	B56	CD3-CD19+(B cell)	IR	0.204065	0
B15	B60	CD3-CD19+(B cell)	WBRVI	0.210333	0
B15	B62	CD3-CD19+(B cell)	WBHSRVI	0.196642	0
B15	B69	CD3-CD19+(B cell)	PV	-0.47332	0
B15	B79	CD3-CD19+(B cell)	hb V -dna	0.206411	0
B15	B92	CD3-CD19+(B cell)	ALDH2-Glu504Glu	0.40486	0
B15	B93	CD3-CD19+(B cell)	ALDH2-Glu504Lys	-0.41027	0
B15	B94	CD3-CD19+(B cell)	ALDH2-Lys504Lys	0.335158	0
B15	B95	CD3-CD19+(B cell)	anti-cenpb	0.434097	0
B15	B97	CD3-CD19+(B cell)	dsDNA	0.434097	0
B15	B98	CD3-CD19+(B cell)	anti-Jo-1	0.434097	0
B15	B99	CD3-CD19+(B cell)	anti-PM-Scl	0.434097	0
B15	B183	CD3-CD19+(B cell)	PTA	0.174829	1.11E-15
B15	B156	CD3-CD19+(B cell)	IgE	-0.17426	1.33E-15
B15	B182	CD3-CD19+(B cell)	TT	0.174149	1.33E-15
B15	B54	CD3-CD19+(B cell)	ESR	-0.15993	2.37E-13
B15	B197	CD3-CD19+(B cell)	AG	-0.15328	2.25E-12
B15	B134	CD3-CD19+(B cell)	CHE	0.149955	6.70E-12
B15	B199	CD3-CD19+(B cell)	Mg	0.137353	3.36E-10
B15	B228	CD3-CD19+(B cell)	OS	-0.13287	1.25E-09
B15	B193	CD3-CD19+(B cell)	K	-0.12812	4.75E-09
B15	B151	CD3-CD19+(B cell)	Anti-CCP	-0.12577	9.08E-09
B15	B158	CD3-CD19+(B cell)	IgM	0.122945	1.94E-08
B15	B57	CD3-CD19+(B cell)	RBCAI	0.112862	2.56E-07
B15	B25	CD3-CD19+(B cell)	CK	0.108953	6.57E-07
B15	B34	CD3-CD19+(B cell)	TK	-0.10749	9.26E-07
B150	B176	ALP	Ucr	-0.12326	1.79E-08
B150	B195	ALP	Na	0.117826	7.39E-08
B150	B197	ALP	AG	0.109306	6.04E-07
B151	B168	Anti-CCP	NMP22	-0.26762	0
B151	B181	Anti-CCP	APTT	0.19287	0
B151	B156	Anti-CCP	IgE	0.118772	5.80E-08
B152	B199	ACA-Ig(G-A-M)	Mg	-0.16577	3.02E-14
B152	B194	ACA-Ig(G-A-M)	Cl	0.132989	1.20E-09
B153	B170	Glu	U-GLU	0.422119	0
B153	B206	Glu	Waist	0.179042	2.22E-16
B153	B215	Glu	BMI	0.170683	4.88E-15
B153	B210	Glu	Hip	0.152525	2.89E-12
B153	B222	Glu	NS	0.137939	2.82E-10
B153	B216	Glu	BMI	0.131767	1.71E-09
B153	B178	Glu	Ig-U	0.111007	4.02E-07
B153	B160	Glu	PH	-0.10855	7.24E-07
B155	B157	IgA	IgG	0.237382	0

B155	B156	IgA	IgE	0.173605	1.78E-15
B155	B195	IgA	Na	-0.12189	2.57E-08
B156	B157	IgE	IgG	0.187215	0
B156	B195	IgE	Na	-0.17055	5.33E-15
B156	B194	IgE	Cl	-0.15	6.60E-12
B156	B159	IgE	β2-MG	0.127301	5.97E-09
B157	B158	IgG	IgM	-0.22341	0
B157	B182	IgG	TT	-0.16781	1.44E-14
B157	B195	IgG	Na	-0.16702	1.91E-14
B157	B194	IgG	Cl	-0.13545	5.89E-10
B157	B159	IgG	β2-MG	0.11899	5.48E-08
B158	B159	IgM	β2-MG	-0.48856	0
B158	B180	IgM	Tf	0.126432	7.58E-09
B159	B199	β2-MG	Mg	0.137218	3.50E-10
B16	B100	CD3+T	anti-Ro52	0.292769	0
B16	B101	CD3+T	anti-Sc70	0.292769	0
B16	B102	CD3+T	anti-Sm	-0.82773	0
B16	B104	CD3+T	anti-SSB	0.292769	0
B16	B105	CD3+T	anti-U1RNP	-0.75466	0
B16	B106	CD3+T	anti-P	0.292769	0
B16	B107	CD3+T	AnuA	0.292769	0
B16	B108	CD3+T	Anti-Cyclin	0.228855	0
B16	B110	CD3+T	AHA	0.292769	0
B16	B124	CD3+T	ACTH	0.409083	0
B16	B125	CD3+T	Cor	0.324952	0
B16	B136	CD3+T	5'NT	0.21856	0
B16	B138	CD3+T	PAB	0.270377	0
B16	B156	CD3+T	IgE	-0.22273	0
B16	B18	CD3+T	CD3+CD4+helper(inducer)T	0.80126	0
B16	B185	CD3+T	PT	-0.26139	0
B16	B19	CD3+T	CD3+CD4+/CD3+CD8+	0.308089	0
B16	B198	CD3+T	P	0.184234	0
B16	B20	CD3+T	CD3+CD4+CD8+	-0.23734	0
B16	B22	CD3+T	CD3+CD8+(T)	0.311183	0
B16	B23	CD3+T	CD3-CD16+CD56+(NK)	-0.95899	0
B16	B55	CD3+T	TK	0.294705	0
B16	B56	CD3+T	IR	0.271599	0
B16	B62	CD3+T	WBHSRVI	0.262177	0
B16	B69	CD3+T	PV	-0.47251	0
B16	B95	CD3+T	anti-cenpb	0.292769	0
B16	B97	CD3+T	dsDNA	0.292769	0
B16	B98	CD3+T	anti-Jo-1	0.292769	0
B16	B99	CD3+T	anti-PM-Scl	0.292769	0
B16	B181	CD3+T	APTT	-0.17941	2.22E-16

B16	B81	CD3+T	TSGF	0.176896	4.44E-16
B16	B183	CD3+T	PTA	0.172372	2.66E-15
B16	B109	CD3+T	AMA-M2	0.168666	1.07E-14
B16	B21	CD3+T	CD3+CD4-CD8-	-0.16279	8.70E-14
B16	B228	CD3+T	OS	-0.1494	8.02E-12
B16	B61	CD3+T	WBHSRV	0.14576	2.56E-11
B16	B60	CD3+T	WBRVI	0.129529	3.21E-09
B16	B179	CD3+T	α1-MG	-0.12401	1.46E-08
B16	B54	CD3+T	ESR	-0.10965	5.56E-07
B16	B182	CD3+T	TT	0.107778	8.67E-07
B160	B163	PH	SG	-0.22288	0
B160	B191	PH	Urea	-0.22761	0
B160	B190	PH	Cre	-0.17357	1.78E-15
B160	B192	PH	UA	-0.17223	2.89E-15
B161	B173	LEU	NTT	0.205024	0
B161	B221	LEU	Hei	-0.12644	7.56E-09
B163	B166	SG	Pro	0.320612	0
B163	B176	SG	Ucr	0.359705	0
B163	B191	SG	Urea	0.18978	0
B163	B216	SG	BMI	0.218358	0
B163	B221	SG	Hei	0.209711	0
B163	B222	SG	NS	0.199666	0
B163	B174	SG	Urea/Cr	0.130439	2.49E-09
B163	B210	SG	Hip	0.127655	5.41E-09
B163	B206	SG	Waist	0.126034	8.45E-09
B163	B215	SG	BMI	0.122943	1.94E-08
B166	B176	Pro	Ucr	0.17232	2.66E-15
B166	B190	Pro	Cre	0.130772	2.26E-09
B166	B191	Pro	Urea	0.116553	1.02E-07
B167	B223	PC	FH	0.502639	0
B168	B175	NMP22	ALB-U	0.300219	0
B168	B177	NMP22	ACR	0.280838	0
B168	B173	NMP22	NTT	0.1106	4.43E-07
B169	B199	U-CRY	Mg	-0.16572	3.06E-14
B169	B194	U-CRY	Cl	0.132956	1.21E-09
B17	B100	CD3+CD16+CD56+NK	anti-Ro52	0.300783	0
B17	B101	CD3+CD16+CD56+NK	anti-Sc70	0.300783	0
B17	B104	CD3+CD16+CD56+NK	anti-SSB	0.300783	0
B17	B106	CD3+CD16+CD56+NK	anti-P	0.300783	0
B17	B107	CD3+CD16+CD56+NK	AnuA	0.300783	0
B17	B110	CD3+CD16+CD56+NK	AHA	0.300783	0
B17	B137	CD3+CD16+CD56+NK	AFU	-0.22074	0
B17	B151	CD3+CD16+CD56+NK	Anti-CCP	0.197844	0
B17	B181	CD3+CD16+CD56+NK	APTT	0.416309	0

B17	B19	CD3+CD16+CD56+NK	CD3+CD4+/CD3+CD8+	-0.26215	0
B17	B20	CD3+CD16+CD56+NK	CD3+CD4+CD8+	0.390063	0
B17	B21	CD3+CD16+CD56+NK	CD3+CD4-CD8-	0.384703	0
B17	B22	CD3+CD16+CD56+NK	CD3+CD8+(T)	0.302299	0
B17	B25	CD3+CD16+CD56+NK	CK	-0.18775	0
B17	B34	CD3+CD16+CD56+NK	TK	0.309032	0
B17	B95	CD3+CD16+CD56+NK	anti-cenpb	0.300783	0
B17	B97	CD3+CD16+CD56+NK	dsDNA	0.300783	0
B17	B98	CD3+CD16+CD56+NK	anti-Jo-1	0.300783	0
B17	B99	CD3+CD16+CD56+NK	anti-PM-Scl	0.300783	0
B17	B185	CD3+CD16+CD56+NK	PT	0.176668	4.44E-16
B17	B18	CD3+CD16+CD56+NK	CD3+CD4+helper(inducer)T	-0.17167	3.55E-15
B17	B182	CD3+CD16+CD56+NK	TT	0.153944	1.81E-12
B17	B183	CD3+CD16+CD56+NK	PTA	0.150694	5.27E-12
B17	B199	CD3+CD16+CD56+NK	Mg	-0.13721	3.50E-10
B17	B134	CD3+CD16+CD56+NK	CHE	-0.13496	6.80E-10
B17	B158	CD3+CD16+CD56+NK	IgM	0.126931	6.61E-09
B17	B60	CD3+CD16+CD56+NK	WBRVI	-0.12613	8.22E-09
B17	B138	CD3+CD16+CD56+NK	PAB	-0.12032	3.88E-08
B17	B176	CD3+CD16+CD56+NK	Ucr	-0.11532	1.39E-07
B170	B228	U-GLU	OS	0.119773	4.47E-08
B170	B190	U-GLU	Cre	0.113328	2.28E-07
B173	B222	NTT	NS	-0.11574	1.25E-07
B174	B189	Urea/Cr	eGFB	0.188401	0
B174	B190	Urea/Cr	Cre	-0.37159	0
B174	B191	Urea/Cr	Urea	0.471356	0
B174	B216	Urea/Cr	BMI	-0.19134	0
B174	B221	Urea/Cr	Hei	-0.28253	0
B174	B222	Urea/Cr	NS	-0.24473	0
B174	B192	Urea/Cr	UA	-0.16102	1.62E-13
B174	B206	Urea/Cr	Waist	-0.11423	1.83E-07
B175	B177	ALB-U	ACR	0.799262	0
B176	B214	Ucr	CVA-L	0.175488	8.88E-16
B176	B177	Ucr	ACR	-0.16658	2.26E-14
B178	B179	Ig-U	α1-MG	0.508778	0
B178	B180	Ig-U	Tf	0.977038	0
B178	B197	Ig-U	AG	-0.18247	0
B178	B196	Ig-U	TCO2	0.132886	1.24E-09
B178	B200	Ig-U	OP	0.111103	3.93E-07
B178	B198	Ig-U	P	0.10786	8.50E-07
B179	B180	α1-MG	Tf	0.45688	0
B179	B194	α1-MG	Cl	0.187441	0
B179	B197	α1-MG	AG	-0.20122	0
B179	B186	α1-MG	PFC	0.161292	1.47E-13

B179	B200	α1-MG	OP	0.15014	6.31E-12
B18	B102	CD3+CD4+helper(inducer)T	anti-Sm	-0.73759	0
B18	B105	CD3+CD4+helper(inducer)T	anti-U1RNP	-0.62695	0
B18	B108	CD3+CD4+helper(inducer)T	Anti-Cyclin	0.379543	0
B18	B109	CD3+CD4+helper(inducer)T	AMA-M2	0.405803	0
B18	B124	CD3+CD4+helper(inducer)T	ACTH	0.392819	0
B18	B125	CD3+CD4+helper(inducer)T	Cor	0.335056	0
B18	B136	CD3+CD4+helper(inducer)T	5'NT	0.322609	0
B18	B138	CD3+CD4+helper(inducer)T	PAB	0.261504	0
B18	B156	CD3+CD4+helper(inducer)T	IgE	-0.24024	0
B18	B181	CD3+CD4+helper(inducer)T	APTT	-0.30098	0
B18	B185	CD3+CD4+helper(inducer)T	PT	-0.29482	0
B18	B19	CD3+CD4+helper(inducer)T	CD3+CD4+/CD3+CD8+	0.773566	0
B18	B198	CD3+CD4+helper(inducer)T	P	0.275816	0
B18	B20	CD3+CD4+helper(inducer)T	CD3+CD4+CD8+	-0.47658	0
B18	B21	CD3+CD4+helper(inducer)T	CD3+CD4-CD8-	-0.32139	0
B18	B22	CD3+CD4+helper(inducer)T	CD3+CD8+(T)	-0.28261	0
B18	B23	CD3+CD4+helper(inducer)T	CD3-CD16+CD56+(NK)	-0.80182	0
B18	B55	CD3+CD4+helper(inducer)T	TK	0.331785	0
B18	B56	CD3+CD4+helper(inducer)T	IR	0.273179	0
B18	B60	CD3+CD4+helper(inducer)T	WBRVI	0.245043	0
B18	B62	CD3+CD4+helper(inducer)T	WBHSRVI	0.224677	0
B18	B69	CD3+CD4+helper(inducer)T	PV	-0.46907	0
B18	B186	CD3+CD4+helper(inducer)T	PFC	-0.17273	2.22E-15
B18	B81	CD3+CD4+helper(inducer)T	TSGF	0.167197	1.82E-14
B18	B93	CD3+CD4+helper(inducer)T	ALDH2-Glu504Lys	0.158588	3.76E-13
B18	B92	CD3+CD4+helper(inducer)T	ALDH2-Glu504Glu	-0.15649	7.68E-13
B18	B179	CD3+CD4+helper(inducer)T	α1-MG	-0.15639	7.95E-13
B18	B54	CD3+CD4+helper(inducer)T	ESR	-0.15537	1.12E-12
B18	B184	CD3+CD4+helper(inducer)T	INR	-0.15274	2.70E-12
B18	B61	CD3+CD4+helper(inducer)T	WBHSRV	0.135248	6.25E-10
B18	B57	CD3+CD4+helper(inducer)T	RBCAI	0.135177	6.38E-10
B18	B228	CD3+CD4+helper(inducer)T	OS	-0.13492	6.87E-10
B18	B94	CD3+CD4+helper(inducer)T	ALDH2-Lys504Lys	-0.12963	3.12E-09
B18	B59	CD3+CD4+helper(inducer)T	WBRV	0.123764	1.56E-08
B18	B182	CD3+CD4+helper(inducer)T	TT	0.122388	2.25E-08
B18	B137	CD3+CD4+helper(inducer)T	AFU	0.12149	2.86E-08
B18	B139	CD3+CD4+helper(inducer)T	ADA	-0.12038	3.82E-08
B180	B197	Tf	AG	-0.17106	4.44E-15
B180	B196	Tf	TCO2	0.131447	1.87E-09
B180	B200	Tf	OP	0.107379	9.52E-07
B181	B184	APTT	INR	0.413204	0
B181	B185	APTT	PT	0.34529	0
B181	B182	APTT	TT	-0.13181	1.69E-09

B182	B183	TT	PTA	-0.2832	0
B182	B184	TT	INR	-0.21404	0
B182	B185	TT	PT	-0.33012	0
B182	B187	TT	NSE	-0.15901	3.26E-13
B182	B195	TT	Na	0.107256	9.79E-07
B183	B184	PTA	INR	0.201747	0
B183	B186	PTA	PFC	0.241168	0
B184	B185	INR	PT	0.857259	0
B184	B186	INR	PFC	-0.15126	4.38E-12
B186	B193	PFC	K	0.110832	4.19E-07
B189	B190	eGFB	Cre	-0.60751	0
B189	B191	eGFB	Urea	-0.41723	0
B189	B192	eGFB	UA	-0.27087	0
B189	B218	eGFB	DBP	0.169673	7.33E-15
B189	B220	eGFB	SBP	-0.115	1.51E-07
B189	B210	eGFB	Hip	0.111256	3.79E-07
B189	B216	eGFB	BMI	0.109236	6.14E-07
B19	B100	CD3+CD4+/CD3+CD8+	anti-Ro52	-0.2983	0
B19	B101	CD3+CD4+/CD3+CD8+	anti-Sc70	-0.2983	0
B19	B102	CD3+CD4+/CD3+CD8+	anti-Sm	-0.49415	0
B19	B104	CD3+CD4+/CD3+CD8+	anti-SSB	-0.2983	0
B19	B105	CD3+CD4+/CD3+CD8+	anti-U1RNP	-0.43202	0
B19	B106	CD3+CD4+/CD3+CD8+	anti-P	-0.2983	0
B19	B107	CD3+CD4+/CD3+CD8+	AnuA	-0.2983	0
B19	B108	CD3+CD4+/CD3+CD8+	Anti-Cyclin	0.192917	0
B19	B109	CD3+CD4+/CD3+CD8+	AMA-M2	0.403614	0
B19	B110	CD3+CD4+/CD3+CD8+	AHA	-0.2983	0
B19	B124	CD3+CD4+/CD3+CD8+	ACTH	0.217394	0
B19	B125	CD3+CD4+/CD3+CD8+	Cor	0.28324	0
B19	B136	CD3+CD4+/CD3+CD8+	5'NT	0.214756	0
B19	B139	CD3+CD4+/CD3+CD8+	ADA	-0.21317	0
B19	B156	CD3+CD4+/CD3+CD8+	IgE	-0.22308	0
B19	B181	CD3+CD4+/CD3+CD8+	APTT	-0.28451	0
B19	B185	CD3+CD4+/CD3+CD8+	PT	-0.23856	0
B19	B186	CD3+CD4+/CD3+CD8+	PFC	-0.26885	0
B19	B20	CD3+CD4+/CD3+CD8+	CD3+CD4+CD8+	-0.36734	0
B19	B22	CD3+CD4+/CD3+CD8+	CD3+CD8+(T)	-0.8021	0
B19	B23	CD3+CD4+/CD3+CD8+	CD3-CD16+CD56+(NK)	-0.42518	0
B19	B25	CD3+CD4+/CD3+CD8+	CK	0.285997	0
B19	B34	CD3+CD4+/CD3+CD8+	TK	-0.22837	0
B19	B54	CD3+CD4+/CD3+CD8+	ESR	-0.17986	0
B19	B55	CD3+CD4+/CD3+CD8+	TK	0.312015	0
B19	B56	CD3+CD4+/CD3+CD8+	IR	0.222585	0
B19	B57	CD3+CD4+/CD3+CD8+	RBCAI	0.322942	0

B19	B59	CD3+CD4+/CD3+CD8+	WBRV	0.248465	0
B19	B60	CD3+CD4+/CD3+CD8+	WBRVI	0.309957	0
B19	B69	CD3+CD4+/CD3+CD8+	PV	-0.37563	0
B19	B95	CD3+CD4+/CD3+CD8+	anti-cenpb	-0.2983	0
B19	B97	CD3+CD4+/CD3+CD8+	dsDNA	-0.2983	0
B19	B98	CD3+CD4+/CD3+CD8+	anti-Jo-1	-0.2983	0
B19	B99	CD3+CD4+/CD3+CD8+	anti-PM-Scl	-0.2983	0
B19	B21	CD3+CD4+/CD3+CD8+	CD3+CD4-CD8-	-0.17394	1.33E-15
B19	B65	CD3+CD4+/CD3+CD8+	WBRSR(mPas_1)	0.166521	2.31E-14
B19	B157	CD3+CD4+/CD3+CD8+	IgG	-0.16115	1.55E-13
B19	B93	CD3+CD4+/CD3+CD8+	ALDH2-Glu504Lys	0.158139	4.38E-13
B19	B182	CD3+CD4+/CD3+CD8+	TT	0.15614	8.65E-13
B19	B92	CD3+CD4+/CD3+CD8+	ALDH2-Glu504Glu	-0.15607	8.86E-13
B19	B138	CD3+CD4+/CD3+CD8+	PAB	0.149245	8.43E-12
B19	B137	CD3+CD4+/CD3+CD8+	AFU	0.146634	1.94E-11
B19	B193	CD3+CD4+/CD3+CD8+	K	-0.14466	3.62E-11
B19	B76	CD3+CD4+/CD3+CD8+	ApoB	-0.14019	1.43E-10
B19	B62	CD3+CD4+/CD3+CD8+	WBHSRVI	0.139214	1.93E-10
B19	B179	CD3+CD4+/CD3+CD8+	α1-MG	-0.13842	2.44E-10
B19	B194	CD3+CD4+/CD3+CD8+	Cl	-0.13513	6.46E-10
B19	B94	CD3+CD4+/CD3+CD8+	ALDH2-Lys504Lys	-0.12882	3.91E-09
B19	B71	CD3+CD4+/CD3+CD8+	TG	0.115445	1.35E-07
B19	B184	CD3+CD4+/CD3+CD8+	INR	-0.11472	1.62E-07
B19	B198	CD3+CD4+/CD3+CD8+	P	0.111437	3.62E-07
B19	B75	CD3+CD4+/CD3+CD8+	ApoA1/B	0.109286	6.07E-07
B19	B197	CD3+CD4+/CD3+CD8+	AG	0.108418	7.46E-07
B19	B58	CD3+CD4+/CD3+CD8+	HCT	-0.10813	7.98E-07
B190	B191	Cre	Urea	0.597297	0
B190	B192	Cre	UA	0.432332	0
B190	B221	Cre	Hei	0.26698	0
B190	B222	Cre	NS	0.274139	0
B190	B216	Cre	BMI	0.163971	5.73E-14
B190	B206	Cre	Waist	0.135017	6.68E-10
B190	B207	Cre	WHR	0.134831	7.05E-10
B191	B192	Urea	UA	0.308584	0
B192	B206	UA	Waist	0.264919	0
B192	B207	UA	WHR	0.180472	0
B192	B210	UA	Hip	0.207145	0
B192	B215	UA	BMI	0.20391	0
B192	B216	UA	BMI	0.311538	0
B192	B221	UA	Hei	0.268713	0
B192	B222	UA	NS	0.325184	0
B193	B194	K	Cl	0.36024	0
B193	B200	K	OP	0.297414	0

B193	B195	K	Na	0.152412	3.00E-12
B193	B199	K	Mg	0.125035	1.11E-08
B194	B195	Cl	Na	0.386468	0
B194	B196	Cl	TCO2	-0.25842	0
B194	B197	Cl	AG	-0.33921	0
B194	B200	Cl	OP	0.463706	0
B195	B197	Na	AG	0.26149	0
B195	B198	Na	P	-0.22239	0
B195	B200	Na	OP	0.871635	0
B195	B196	Na	TCO2	0.138143	2.65E-10
B196	B197	TCO2	AG	-0.58307	0
B196	B199	TCO2	Mg	-0.17162	3.55E-15
B196	B200	TCO2	OP	0.13748	3.23E-10
B196	B198	TCO2	P	-0.13341	1.06E-09
B197	B199	AG	Mg	0.139858	1.59E-10
B198	B200	P	OP	-0.20584	0
B198	B199	P	Mg	-0.12112	3.15E-08
B2	B126	Age	Alb	-0.25659	0
B2	B127	Age	A-G ratio	-0.18385	0
B2	B141	Age	AST/ALT	0.296104	0
B2	B163	Age	SG	-0.19333	0
B2	B189	Age	eGFB	-0.40789	0
B2	B191	Age	Urea	0.209161	0
B2	B210	Age	Hip	-0.2054	0
B2	B215	Age	BMI	-0.23398	0
B2	B216	Age	BMI	-0.36524	0
B2	B218	Age	DBP	-0.29872	0
B2	B220	Age	SBP	0.231753	0
B2	B221	Age	Hei	-0.30725	0
B2	B222	Age	NS	-0.21856	0
B2	B46	Age	RBC Count	-0.2634	0
B2	B47	Age	RDW	0.208905	0
B2	B51	Age	Hb	-0.30456	0
B2	B72	Age	HDL-C	0.229562	0
B2	B71	Age	TG	-0.17959	2.22E-16
B2	B176	Age	Ucr	-0.17626	6.66E-16
B2	B128	Age	ALT	-0.17289	2.22E-15
B2	B190	Age	Cre	0.163174	7.62E-14
B2	B206	Age	Waist	-0.14649	2.03E-11
B2	B74	Age	ApoA1	0.141699	9.05E-11
B2	B41	Age	MON％	0.138176	2.63E-10
B2	B209	Age	Smoking	-0.13675	4.02E-10
B2	B94	Age	ALDH2-Lys504Lys	0.128759	3.99E-09
B2	B130	Age	GLB	0.110347	4.71E-07

B2	B79	Age	hb V -dna	0.108004	8.22E-07
B20	B100	CD3+CD4+CD8+	anti-Ro52	0.236801	0
B20	B101	CD3+CD4+CD8+	anti-Sc70	0.236801	0
B20	B104	CD3+CD4+CD8+	anti-SSB	0.236801	0
B20	B106	CD3+CD4+CD8+	anti-P	0.236801	0
B20	B107	CD3+CD4+CD8+	AnuA	0.2368	0
B20	B108	CD3+CD4+CD8+	Anti-Cyclin	-0.2822	0
B20	B110	CD3+CD4+CD8+	AHA	0.236801	0
B20	B136	CD3+CD4+CD8+	5'NT	-0.81738	0
B20	B181	CD3+CD4+CD8+	APTT	0.244057	0
B20	B198	CD3+CD4+CD8+	P	-0.28224	0
B20	B200	CD3+CD4+CD8+	OP	-0.18423	0
B20	B21	CD3+CD4+CD8+	CD3+CD4-CD8-	0.31993	0
B20	B22	CD3+CD4+CD8+	CD3+CD8+(T)	0.296521	0
B20	B23	CD3+CD4+CD8+	CD3-CD16+CD56+(NK)	0.246043	0
B20	B74	CD3+CD4+CD8+	ApoA1	0.200603	0
B20	B92	CD3+CD4+CD8+	ALDH2-Glu504Glu	0.215469	0
B20	B93	CD3+CD4+CD8+	ALDH2-Glu504Lys	-0.21833	0
B20	B95	CD3+CD4+CD8+	anti-cenpb	0.236801	0
B20	B97	CD3+CD4+CD8+	dsDNA	0.236801	0
B20	B98	CD3+CD4+CD8+	anti-Jo-1	0.236801	0
B20	B99	CD3+CD4+CD8+	anti-PM-Scl	0.236801	0
B20	B94	CD3+CD4+CD8+	ALDH2-Lys504Lys	0.177902	4.44E-16
B20	B134	CD3+CD4+CD8+	CHE	0.161645	1.30E-13
B20	B193	CD3+CD4+CD8+	K	-0.15156	3.98E-12
B20	B120	CD3+CD4+CD8+	HCV-cAg	0.147342	1.55E-11
B20	B151	CD3+CD4+CD8+	Anti-CCP	0.143083	5.91E-11
B20	B183	CD3+CD4+CD8+	PTA	0.118507	6.21E-08
B20	B34	CD3+CD4+CD8+	TK	0.117674	7.68E-08
B20	B25	CD3+CD4+CD8+	CK	-0.11368	2.09E-07
B20	B137	CD3+CD4+CD8+	AFU	-0.11081	4.21E-07
B20	B81	CD3+CD4+CD8+	TSGF	-0.10981	5.36E-07
B20	B79	CD3+CD4+CD8+	hb V -dna	0.108765	6.87E-07
B201	B204	e-habits	Drinking	0.208545	0
B202	B223	e-times	FH	0.6484	0
B204	B209	Drinking	Smoking	0.33833	0
B204	B221	Drinking	Hei	0.169848	6.88E-15
B204	B222	Drinking	NS	0.16542	3.42E-14
B204	B216	Drinking	BMI	0.14155	9.48E-11
B205	B222	KDA	NS	-0.10857	7.18E-07
B206	B207	Waist	WHR	0.621222	0
B206	B210	Waist	Hip	0.754244	0
B206	B215	Waist	BMI	0.790754	0
B206	B216	Waist	BMI	0.795572	0

B206	B221	Waist	Hei	0.360138	0
B206	B222	Waist	NS	0.667941	0
B207	B215	WHR	BMI	0.336904	0
B207	B216	WHR	BMI	0.341698	0
B207	B222	WHR	NS	0.344144	0
B207	B221	WHR	Hei	0.157906	4.75E-13
B207	B210	WHR	Hip	0.126665	7.11E-09
B209	B221	Smoking	Hei	0.20381	0
B209	B222	Smoking	NS	0.175385	8.88E-16
B209	B216	Smoking	BMI	0.155178	1.20E-12
B21	B100	CD3+CD4-CD8-	anti-Ro52	0.468546	0
B21	B101	CD3+CD4-CD8-	anti-Sc70	0.468546	0
B21	B104	CD3+CD4-CD8-	anti-SSB	0.468546	0
B21	B105	CD3+CD4-CD8-	anti-U1RNP	-0.19489	0
B21	B106	CD3+CD4-CD8-	anti-P	0.468546	0
B21	B107	CD3+CD4-CD8-	AnuA	0.468545	0
B21	B108	CD3+CD4-CD8-	Anti-Cyclin	-0.83772	0
B21	B110	CD3+CD4-CD8-	AHA	0.468546	0
B21	B124	CD3+CD4-CD8-	ACTH	-0.25657	0
B21	B135	CD3+CD4-CD8-	TBA	-0.28536	0
B21	B151	CD3+CD4-CD8-	Anti-CCP	0.272545	0
B21	B181	CD3+CD4-CD8-	APTT	0.399105	0
B21	B183	CD3+CD4-CD8-	PTA	0.234255	0
B21	B184	CD3+CD4-CD8-	INR	0.179706	0
B21	B198	CD3+CD4-CD8-	P	-0.24088	0
B21	B25	CD3+CD4-CD8-	CK	-0.18928	0
B21	B79	CD3+CD4-CD8-	hb V -dna	0.323628	0
B21	B81	CD3+CD4-CD8-	TSGF	-0.31898	0
B21	B92	CD3+CD4-CD8-	ALDH2-Glu504Glu	0.631142	0
B21	B93	CD3+CD4-CD8-	ALDH2-Glu504Lys	-0.63961	0
B21	B94	CD3+CD4-CD8-	ALDH2-Lys504Lys	0.523283	0
B21	B95	CD3+CD4-CD8-	anti-cenpb	0.468546	0
B21	B97	CD3+CD4-CD8-	dsDNA	0.468546	0
B21	B98	CD3+CD4-CD8-	anti-Jo-1	0.468546	0
B21	B99	CD3+CD4-CD8-	anti-PM-Scl	0.468546	0
B21	B137	CD3+CD4-CD8-	AFU	-0.16943	7.99E-15
B21	B74	CD3+CD4-CD8-	ApoA1	0.158204	4.29E-13
B21	B23	CD3+CD4-CD8-	CD3-CD16+CD56+(NK)	0.143438	5.30E-11
B21	B60	CD3+CD4-CD8-	WBRVI	-0.12783	5.15E-09
B21	B155	CD3+CD4-CD8-	IgA	-0.12504	1.11E-08
B21	B158	CD3+CD4-CD8-	IgM	0.122086	2.44E-08
B21	B136	CD3+CD4-CD8-	5'NT	0.121436	2.90E-08
B21	B125	CD3+CD4-CD8-	Cor	0.120866	3.36E-08
B21	B34	CD3+CD4-CD8-	TK	0.11238	2.88E-07

B21	B54	CD3+CD4-CD8-	ESR	0.109162	6.25E-07
B210	B215	Hip	BMI	0.769689	0
B210	B216	Hip	BMI	0.779982	0
B210	B221	Hip	Hei	0.364468	0
B210	B222	Hip	NS	0.588266	0
B211	B212	IOP-R	IOP-L	0.767812	0
B215	B216	BMI	BMI	0.778045	0
B215	B222	BMI	NS	0.542868	0
B216	B221	BMI	Hei	0.695406	0
B216	B222	BMI	NS	0.765443	0
B216	B218	BMI	DBP	0.135172	6.39E-10
B216	B220	BMI	SBP	-0.12272	2.06E-08
B218	B220	DBP	SBP	0.397651	0
B218	B221	DBP	Hei	0.158296	4.16E-13
B219	B222	OT	NS	0.121727	2.68E-08
B22	B100	CD3+CD8+(T)	anti-Ro52	0.466933	0
B22	B101	CD3+CD8+(T)	anti-Sc70	0.466933	0
B22	B104	CD3+CD8+(T)	anti-SSB	0.466933	0
B22	B106	CD3+CD8+(T)	anti-P	0.466933	0
B22	B107	CD3+CD8+(T)	AnuA	0.466932	0
B22	B109	CD3+CD8+(T)	AMA-M2	-0.29678	0
B22	B110	CD3+CD8+(T)	AHA	0.466933	0
B22	B139	CD3+CD8+(T)	ADA	0.189924	0
B22	B157	CD3+CD8+(T)	IgG	0.188819	0
B22	B181	CD3+CD8+(T)	APTT	0.182325	0
B22	B183	CD3+CD8+(T)	PTA	0.186857	0
B22	B186	CD3+CD8+(T)	PFC	0.219535	0
B22	B25	CD3+CD8+(T)	CK	-0.32355	0
B22	B34	CD3+CD8+(T)	TK	0.268891	0
B22	B57	CD3+CD8+(T)	RBCAI	-0.3575	0
B22	B59	CD3+CD8+(T)	WBRV	-0.24882	0
B22	B60	CD3+CD8+(T)	WBRVI	-0.20792	0
B22	B76	CD3+CD8+(T)	ApoB	0.188575	0
B22	B95	CD3+CD8+(T)	anti-cenpb	0.466933	0
B22	B97	CD3+CD8+(T)	dsDNA	0.466933	0
B22	B98	CD3+CD8+(T)	anti-Jo-1	0.466933	0
B22	B99	CD3+CD8+(T)	anti-PM-Scl	0.466933	0
B22	B23	CD3+CD8+(T)	CD3-CD16+CD56+(NK)	-0.17097	4.44E-15
B22	B136	CD3+CD8+(T)	5'NT	-0.16475	4.35E-14
B22	B75	CD3+CD8+(T)	ApoA1/B	-0.16235	1.02E-13
B22	B65	CD3+CD8+(T)	WBRSR(mPas_1)	-0.16183	1.22E-13
B22	B58	CD3+CD8+(T)	HCT	0.158728	3.58E-13
B22	B137	CD3+CD8+(T)	AFU	-0.15004	6.52E-12
B22	B193	CD3+CD8+(T)	K	0.149957	6.70E-12

B22	B197	CD3+CD8+(T)	AG	-0.14703	1.71E-11
B22	B93	CD3+CD8+(T)	ALDH2-Glu504Lys	-0.14216	7.86E-11
B22	B92	CD3+CD8+(T)	ALDH2-Glu504Glu	0.140316	1.38E-10
B22	B194	CD3+CD8+(T)	CI	0.136767	4.00E-10
B22	B71	CD3+CD8+(T)	TG	-0.12231	2.30E-08
B22	B94	CD3+CD8+(T)	ALDH2-Lys504Lys	0.115412	1.36E-07
B22	B55	CD3+CD8+(T)	TK	-0.10991	5.23E-07
B220	B221	SBP	Hei	-0.1405	1.31E-10
B221	B222	Hei	NS	0.5974	0
B23	B100	CD3-CD16+CD56+(NK)	anti-Ro52	-0.2727	0
B23	B101	CD3-CD16+CD56+(NK)	anti-Sc70	-0.2727	0
B23	B102	CD3-CD16+CD56+(NK)	anti-Sm	0.868509	0
B23	B104	CD3-CD16+CD56+(NK)	anti-SSB	-0.2727	0
B23	B105	CD3-CD16+CD56+(NK)	anti-U1RNP	0.807192	0
B23	B106	CD3-CD16+CD56+(NK)	anti-P	-0.2727	0
B23	B107	CD3-CD16+CD56+(NK)	AnuA	-0.2727	0
B23	B110	CD3-CD16+CD56+(NK)	AHA	-0.2727	0
B23	B124	CD3-CD16+CD56+(NK)	ACTH	-0.41314	0
B23	B125	CD3-CD16+CD56+(NK)	Cor	-0.36293	0
B23	B136	CD3-CD16+CD56+(NK)	5'NT	-0.20635	0
B23	B138	CD3-CD16+CD56+(NK)	PAB	-0.27362	0
B23	B156	CD3-CD16+CD56+(NK)	IgE	0.243486	0
B23	B181	CD3-CD16+CD56+(NK)	APTT	0.262009	0
B23	B185	CD3-CD16+CD56+(NK)	PT	0.305845	0
B23	B55	CD3-CD16+CD56+(NK)	TK	-0.32081	0
B23	B56	CD3-CD16+CD56+(NK)	IR	-0.29233	0
B23	B60	CD3-CD16+CD56+(NK)	WBRVI	-0.18626	0
B23	B62	CD3-CD16+CD56+(NK)	WBHSRVI	-0.27789	0
B23	B69	CD3-CD16+CD56+(NK)	PV	0.524321	0
B23	B95	CD3-CD16+CD56+(NK)	anti-cenpb	-0.2727	0
B23	B97	CD3-CD16+CD56+(NK)	dsDNA	-0.2727	0
B23	B98	CD3-CD16+CD56+(NK)	anti-Jo-1	-0.2727	0
B23	B99	CD3-CD16+CD56+(NK)	anti-PM-Scl	-0.2727	0
B23	B108	CD3-CD16+CD56+(NK)	Anti-Cyclin	-0.17466	1.11E-15
B23	B228	CD3-CD16+CD56+(NK)	OS	0.156942	6.59E-13
B23	B81	CD3-CD16+CD56+(NK)	TSGF	-0.15577	9.80E-13
B23	B54	CD3-CD16+CD56+(NK)	ESR	0.153824	1.88E-12
B23	B151	CD3-CD16+CD56+(NK)	Anti-CCP	0.153482	2.11E-12
B23	B182	CD3-CD16+CD56+(NK)	TT	-0.15255	2.87E-12
B23	B186	CD3-CD16+CD56+(NK)	PFC	0.14801	1.25E-11
B23	B183	CD3-CD16+CD56+(NK)	PTA	-0.14719	1.63E-11
B23	B61	CD3-CD16+CD56+(NK)	WBHSRV	-0.14426	4.10E-11
B23	B179	CD3-CD16+CD56+(NK)	α1-MG	0.124335	1.34E-08
B23	B93	CD3-CD16+CD56+(NK)	ALDH2-Glu504Lys	0.109277	6.08E-07

B23	B92	CD3-CD16+CD56+(NK)	ALDH2-Glu504Glu	-0.10784	8.54E-07
B24	B25	αHBDH	CK	0.185369	0
B24	B27	αHBDH	LDH	0.508159	0
B24	B54	αHBDH	ESR	0.251309	0
B24	B58	αHBDH	HCT	-0.26642	0
B24	B84	αHBDH	C-PSA	0.170548	5.33E-15
B24	B75	αHBDH	ApoA1/B	0.15526	1.16E-12
B24	B62	αHBDH	WBHSRVI	-0.14692	1.77E-11
B24	B60	αHBDH	WBRVI	-0.14216	7.86E-11
B24	B69	αHBDH	PV	0.133111	1.16E-09
B24	B74	αHBDH	ApoA1	0.118134	6.83E-08
B24	B26	αHBDH	CKMB	0.114087	1.89E-07
B24	B139	αHBDH	ADA	0.113896	1.98E-07
B25	B100	CK	anti-Ro52	-0.20217	0
B25	B101	CK	anti-Sc70	-0.20217	0
B25	B104	CK	anti-SSB	-0.20217	0
B25	B106	CK	anti-P	-0.20217	0
B25	B107	CK	AnuA	-0.20217	0
B25	B110	CK	AHA	-0.20217	0
B25	B181	CK	APTT	-0.20816	0
B25	B95	CK	anti-cenpb	-0.20217	0
B25	B97	CK	dsDNA	-0.20217	0
B25	B98	CK	anti-Jo-1	-0.20217	0
B25	B99	CK	anti-PM-Scl	-0.20217	0
B25	B26	CK	CKMB	0.167193	1.82E-14
B25	B57	CK	RBCAI	0.16399	5.71E-14
B25	B139	CK	ADA	-0.15731	5.82E-13
B25	B151	CK	Anti-CCP	-0.15188	3.58E-12
B25	B34	CK	TK	-0.14803	1.25E-11
B25	B76	CK	ApoB	-0.14587	2.48E-11
B25	B27	CK	LDH	0.144975	3.28E-11
B25	B54	CK	ESR	-0.14492	3.33E-11
B25	B75	CK	ApoA1/B	0.138462	2.41E-10
B25	B186	CK	PFC	-0.13413	8.65E-10
B25	B108	CK	Anti-Cyclin	0.127529	5.61E-09
B25	B135	CK	TBA	0.108335	7.60E-07
B26	B123	CKMB	hs-CRP1	0.251045	0
B26	B131	CKMB	AST	0.118825	5.72E-08
B26	B27	CKMB	LDH	0.111658	3.43E-07
B27	B150	LDH	ALP	0.283976	0
B27	B176	LDH	Ucr	-0.21387	0
B27	B37	LDH	BAND	-0.29853	0
B27	B38	LDH	BAND%	-0.32609	0
B27	B54	LDH	ESR	0.147114	1.67E-11

B27	B139	LDH	ADA	0.128313	4.51E-09
B27	B84	LDH	C-PSA	0.12776	5.26E-09
B27	B58	LDH	HCT	-0.12321	1.81E-08
B27	B163	LDH	SG	-0.12298	1.93E-08
B27	B138	LDH	PAB	-0.11308	2.43E-07
B27	B69	LDH	PV	0.109415	5.89E-07
B27	B131	LDH	AST	0.109219	6.17E-07
B27	B199	LDH	Mg	0.108342	7.59E-07
B28	B29	PRL	E2	0.817691	0
B28	B30	PRL	LH	0.495176	0
B28	B31	PRL	FSH	-0.29949	0
B28	B32	PRL	Testo	0.982135	0
B28	B33	PRL	Prog	0.95868	0
B28	B75	PRL	ApoA1/B	-0.16648	2.35E-14
B28	B76	PRL	ApoB	0.134703	7.32E-10
B28	B174	PRL	Urea/Cr	-0.12324	1.79E-08
B28	B156	PRL	IgE	0.11327	2.32E-07
B29	B30	E2	LH	0.373514	0
B29	B31	E2	FSH	-0.6248	0
B29	B32	E2	Testo	0.705601	0
B29	B33	E2	Prog	0.621095	0
B3	B190	Hcy	Cre	0.14549	2.79E-11
B3	B189	Hcy	eGFB	-0.13267	1.32E-09
B30	B31	LH	FSH	0.485133	0
B30	B32	LH	Testo	0.424409	0
B30	B33	LH	Prog	0.463477	0
B30	B200	LH	OP	-0.13886	2.14E-10
B30	B195	LH	Na	-0.13271	1.30E-09
B31	B32	FSH	Testo	-0.23893	0
B31	B33	FSH	Prog	-0.12165	2.74E-08
B32	B33	Testo	Prog	0.991969	0
B32	B75	Testo	ApoA1/B	-0.19873	0
B32	B76	Testo	ApoB	0.157876	4.80E-13
B32	B156	Testo	IgE	0.133407	1.07E-09
B32	B174	Testo	Urea/Cr	-0.11892	5.57E-08
B32	B155	Testo	IgA	0.118918	5.58E-08
B32	B199	Testo	Mg	0.114013	1.93E-07
B32	B137	Testo	AFU	0.111668	3.43E-07
B33	B75	Prog	ApoA1/B	-0.21827	0
B33	B76	Prog	ApoB	0.174325	1.33E-15
B33	B156	Prog	IgE	0.147029	1.71E-11
B33	B155	Prog	IgA	0.130706	2.31E-09
B33	B199	Prog	Mg	0.126173	8.13E-09
B33	B137	Prog	AFU	0.123677	1.60E-08

B33	B174	Prog	Urea/Cr	-0.11963	4.64E-08
B34	B181	TK	APTT	0.208739	0
B34	B182	TK	TT	-0.19984	0
B34	B81	TK	TSGF	-0.19421	0
B34	B94	TK	ALDH2-Lys504Lys	0.175401	8.88E-16
B34	B186	TK	PFC	0.171147	4.22E-15
B34	B100	TK	anti-Ro52	0.170217	6.00E-15
B34	B101	TK	anti-Sc70	0.170217	6.00E-15
B34	B104	TK	anti-SSB	0.170217	6.00E-15
B34	B106	TK	anti-P	0.170217	6.00E-15
B34	B107	TK	AnuA	0.170216	6.00E-15
B34	B110	TK	AHA	0.170217	6.00E-15
B34	B95	TK	anti-cenpb	0.170217	6.00E-15
B34	B97	TK	dsDNA	0.170217	6.00E-15
B34	B98	TK	anti-Jo-1	0.170217	6.00E-15
B34	B99	TK	anti-PM-Scl	0.170217	6.00E-15
B34	B138	TK	PAB	-0.14965	7.39E-12
B34	B183	TK	PTA	0.147684	1.39E-11
B34	B79	TK	hb V -dna	0.143124	5.84E-11
B34	B185	TK	PT	0.128982	3.74E-09
B34	B187	TK	NSE	0.120886	3.35E-08
B34	B194	TK	Cl	-0.11922	5.16E-08
B34	B157	TK	IgG	0.116683	9.88E-08
B34	B92	TK	ALDH2-Glu504Glu	-0.11594	1.19E-07
B34	B151	TK	Anti-CCP	0.111425	3.63E-07
B35	B202	β2-MG	e-times	0.575696	0
B35	B223	β2-MG	FH	0.99355	0
B37	B38	BAND	BAND%	0.856733	0
B37	B227	BAND	OD	-0.11694	9.25E-08
B4	B5	WB-Ca	WB-Mg	0.869365	0
B4	B6	WB-Ca	WB-Fe	0.486503	0
B4	B7	WB-Ca	WB-Cop	0.49016	0
B4	B8	WB-Ca	WB-Zinc	0.290579	0
B4	B197	WB-Ca	AG	-0.13429	8.26E-10
B40	B192	WBC	UA	0.220884	0
B40	B222	WBC	NS	0.214832	0
B40	B42	WBC	MON	0.674285	0
B40	B46	WBC	RBC Count	0.2594	0
B40	B51	WBC	Hb	0.223165	0
B40	B52	WBC	PLT/BPC	0.223021	0
B40	B72	WBC	HDL-C	-0.1638	6.08E-14
B40	B216	WBC	BMI	0.158992	3.27E-13
B40	B53	WBC	GR%	0.156558	7.51E-13
B40	B206	WBC	Waist	0.153801	1.90E-12

B40	B41	WBC	MON %	-0.15136	4.24E-12
B40	B190	WBC	Cre	0.148555	1.05E-11
B40	B48	WBC	LYM%	-0.13719	3.53E-10
B40	B210	WBC	Hip	0.128293	4.54E-09
B40	B215	WBC	BMI	0.122333	2.29E-08
B40	B221	WBC	Hei	0.121619	2.76E-08
B40	B130	WBC	GLB	0.119188	5.21E-08
B40	B43	WBC	MCV	-0.11385	2.01E-07
B40	B91	WBC	TP	0.109876	5.27E-07
B41	B42	MON %	MON	0.599858	0
B41	B53	MON %	GR%	-0.1587	3.61E-13
B41	B132	MON %	Dbil	0.157136	6.17E-13
B41	B126	MON %	Alb	-0.13976	1.63E-10
B41	B77	MON %	TC	-0.12761	5.49E-09
B41	B71	MON %	TG	-0.11746	8.11E-08
B41	B189	MON %	eGFB	-0.11072	4.31E-07
B41	B70	MON %	LDL-C	-0.11045	4.60E-07
B42	B192	MON	UA	0.231015	0
B42	B222	MON	NS	0.201537	0
B42	B51	MON	Hb	0.181779	0
B42	B190	MON	Cre	0.172264	2.66E-15
B42	B46	MON	RBC Count	0.170619	5.11E-15
B42	B52	MON	PLT/BPC	0.16392	5.86E-14
B42	B48	MON	LYM%	-0.15723	5.98E-13
B42	B77	MON	TC	-0.14756	1.45E-11
B42	B221	MON	Hei	0.138786	2.19E-10
B42	B72	MON	HDL-C	-0.13804	2.74E-10
B42	B216	MON	BMI	0.12687	6.72E-09
B42	B206	MON	Waist	0.117762	7.51E-08
B42	B174	MON	Urea/Cr	-0.112	3.16E-07
B43	B44	MCV	MCH	0.88805	0
B43	B46	MCV	RBC Count	-0.571	0
B43	B47	MCV	RDW	0.570257	0
B44	B45	MCH	MCHC	0.538201	0
B44	B46	MCH	RBC Count	-0.5004	0
B44	B47	MCH	RDW	0.373901	0
B44	B51	MCH	Hb	0.186364	0
B45	B47	MCHC	RDW	-0.22893	0
B45	B51	MCHC	Hb	0.335748	0
B45	B221	MCHC	Hei	0.169715	7.11E-15
B45	B129	MCHC	IBIL	0.161473	1.39E-13
B45	B222	MCHC	NS	0.156506	7.64E-13
B45	B133	MCHC	TBIL	0.155474	1.08E-12
B45	B141	MCHC	AST/ALT	-0.14139	9.96E-11

B45	B216	MCHC	BMI	0.138405	2.45E-10
B45	B189	MCHC	eGFB	0.130857	2.21E-09
B45	B126	MCHC	Alb	0.123238	1.80E-08
B45	B204	MCHC	Drinking	0.113982	1.94E-07
B46	B126	RBC Count	Alb	0.225578	0
B46	B163	RBC Count	SG	0.195147	0
B46	B174	RBC Count	Urea/Cr	-0.19127	0
B46	B189	RBC Count	eGFB	0.212803	0
B46	B206	RBC Count	Waist	0.214556	0
B46	B210	RBC Count	Hip	0.183985	0
B46	B216	RBC Count	BMI	0.320202	0
B46	B218	RBC Count	DBP	0.180117	0
B46	B221	RBC Count	Hei	0.332525	0
B46	B222	RBC Count	NS	0.333469	0
B46	B47	RBC Count	RDW	-0.43402	0
B46	B51	RBC Count	Hb	0.747719	0
B46	B72	RBC Count	HDL-C	-0.20137	0
B46	B141	RBC Count	AST/ALT	-0.17868	2.22E-16
B46	B191	RBC Count	Urea	-0.16303	8.02E-14
B46	B215	RBC Count	BMI	0.15867	3.65E-13
B46	B192	RBC Count	UA	0.15401	1.77E-12
B46	B113	RBC Count	FT3	0.13859	2.32E-10
B46	B207	RBC Count	WHR	0.131557	1.81E-09
B46	B129	RBC Count	IBIL	0.11875	5.83E-08
B46	B209	RBC Count	Smoking	0.113137	2.39E-07
B46	B58	RBC Count	HCT	0.111199	3.84E-07
B46	B133	RBC Count	TBIL	0.111134	3.90E-07
B46	B91	RBC Count	TP	0.110367	4.69E-07
B47	B126	RDW	Alb	-0.18679	0
B47	B189	RDW	eGFB	-0.20696	0
B47	B51	RDW	Hb	-0.20636	0
B47	B52	RDW	PLT/BPC	-0.1741	1.33E-15
B47	B190	RDW	Cre	0.152442	2.97E-12
B47	B191	RDW	Urea	0.136881	3.86E-10
B47	B127	RDW	A-G ratio	-0.11972	4.53E-08
B47	B216	RDW	BMI	-0.11117	3.86E-07
B48	B190	LYM%	Cre	-0.1833	0
B48	B53	LYM%	GR%	-0.93078	0
B48	B189	LYM%	eGFB	0.162895	8.39E-14
B48	B191	LYM%	Urea	-0.15015	6.29E-12
B48	B192	LYM%	UA	-0.11768	7.67E-08
B48	B72	LYM%	HDL-C	0.110876	4.15E-07
B49	B50	baso%	eo%	0.283038	0
B49	B53	baso%	GR%	-0.13412	8.67E-10

B49	B52	baso%	PLT/BPC	0.120076	4.14E-08
B5	B6	WB-Mg	WB-Fe	0.723597	0
B5	B8	WB-Mg	WB-Zinc	0.22807	0
B5	B120	WB-Mg	HCV-cAg	0.133361	1.08E-09
B5	B123	WB-Mg	hs-CRP1	-0.11834	6.47E-08
B50	B53	eo%	GR%	-0.23932	0
B50	B190	eo%	Cre	0.134352	8.11E-10
B51	B126	Hb	Alb	0.262755	0
B51	B141	Hb	AST/ALT	-0.24592	0
B51	B163	Hb	SG	0.211422	0
B51	B174	Hb	Urea/Cr	-0.25847	0
B51	B189	Hb	eGFB	0.257581	0
B51	B191	Hb	Urea	-0.23004	0
B51	B206	Hb	Waist	0.247425	0
B51	B210	Hb	Hip	0.224961	0
B51	B216	Hb	BMI	0.393776	0
B51	B218	Hb	DBP	0.235244	0
B51	B221	Hb	Hei	0.429786	0
B51	B222	Hb	NS	0.402375	0
B51	B72	Hb	HDL-C	-0.21169	0
B51	B215	Hb	BMI	0.176317	4.44E-16
B51	B129	Hb	IBIL	0.175848	8.88E-16
B51	B133	Hb	TBIL	0.175568	8.88E-16
B51	B209	Hb	Smoking	0.164189	5.33E-14
B51	B192	Hb	UA	0.149003	9.12E-12
B51	B127	Hb	A-G ratio	0.139497	1.77E-10
B51	B207	Hb	WHR	0.137093	3.63E-10
B51	B58	Hb	HCT	0.134728	7.27E-10
B51	B132	Hb	Dbil	0.128628	4.13E-09
B51	B204	Hb	Drinking	0.127812	5.18E-09
B51	B113	Hb	FT3	0.126148	8.19E-09
B51	B173	Hb	NTT	-0.11624	1.10E-07
B51	B188	Hb	Ca	0.112424	2.85E-07
B52	B132	PLT/BPC	Dbil	-0.20031	0
B52	B133	PLT/BPC	TBIL	-0.14799	1.26E-11
B52	B70	PLT/BPC	LDL-C	0.135978	5.04E-10
B52	B129	PLT/BPC	IBIL	-0.11737	8.30E-08
B52	B190	PLT/BPC	Cre	-0.11068	4.35E-07
B53	B190	GR%	Cre	0.11915	5.26E-08
B53	B191	GR%	Urea	0.1138	2.03E-07
B53	B189	GR%	eGFB	-0.11338	2.26E-07
B53	B153	GR%	Glu	0.109031	6.45E-07
B54	B102	ESR	anti-Sm	0.20821	0
B54	B103	ESR	anti-SSA/Ro	0.208423	0

B54	B105	ESR	anti-U1RNP	0.187345	0
B54	B57	ESR	RBCAI	-0.19769	0
B54	B58	ESR	HCT	-0.44649	0
B54	B60	ESR	WBRVI	-0.37464	0
B54	B62	ESR	WBHSRVI	-0.3423	0
B54	B69	ESR	PV	0.518071	0
B54	B156	ESR	IgE	0.167278	1.75E-14
B54	B56	ESR	IR	-0.15554	1.06E-12
B54	B74	ESR	ApoA1	0.154913	1.31E-12
B54	B65	ESR	WBRSR(mPas_1)	-0.14159	9.37E-11
B54	B94	ESR	ALDH2-Lys504Lys	0.141131	1.08E-10
B54	B73	ESR	ApoA	0.138992	2.06E-10
B54	B93	ESR	ALDH2-Glu504Lys	-0.11076	4.27E-07
B55	B102	TK	anti-Sm	-0.36132	0
B55	B105	TK	anti-U1RNP	-0.37649	0
B55	B109	TK	AMA-M2	0.236557	0
B55	B156	TK	IgE	-0.18281	0
B55	B56	TK	IR	0.874343	0
B55	B58	TK	HCT	-0.32265	0
B55	B59	TK	WBRV	0.611619	0
B55	B60	TK	WBRVI	0.377932	0
B55	B61	TK	WBHSRV	0.802596	0
B55	B62	TK	WBHSRVI	0.55019	0
B55	B63	TK	WBRSR(mPas_200)	0.4281	0
B55	B64	TK	WBRSR(mPas_5)	0.369617	0
B55	B65	TK	WBRSR(mPas_1)	0.304004	0
B55	B66	TK	WBRSR(mPas_100)	0.479927	0
B55	B67	TK	WBRSR(mPas_50)	0.471573	0
B55	B69	TK	PV	-0.29537	0
B55	B75	TK	ApoA1/B	0.181461	0
B55	B125	TK	Cor	0.164409	4.93E-14
B55	B124	TK	ACTH	0.145895	2.46E-11
B55	B76	TK	ApoB	-0.13434	8.14E-10
B56	B102	IR	anti-Sm	-0.32749	0
B56	B105	IR	anti-U1RNP	-0.33102	0
B56	B59	IR	WBRV	0.657759	0
B56	B60	IR	WBRVI	0.664707	0
B56	B61	IR	WBHSRV	0.910284	0
B56	B62	IR	WBHSRVI	0.877751	0
B56	B63	IR	WBRSR(mPas_200)	0.739452	0
B56	B64	IR	WBRSR(mPas_5)	0.678443	0
B56	B65	IR	WBRSR(mPas_1)	0.58907	0
B56	B66	IR	WBRSR(mPas_100)	0.772737	0
B56	B67	IR	WBRSR(mPas_50)	0.768218	0

B56	B69	IR	PV	-0.31188	0
B56	B58	IR	HCT	0.151207	4.46E-12
B56	B75	IR	ApoA1/B	0.142089	8.03E-11
B56	B76	IR	ApoB	-0.13832	2.52E-10
B56	B124	IR	ACTH	0.129402	3.33E-09
B56	B73	IR	ApoA	0.120491	3.71E-08
B56	B125	IR	Cor	0.117044	9.02E-08
B56	B109	IR	AMA-M2	0.11316	2.38E-07
B57	B100	RBCAI	anti-Ro52	-0.19079	0
B57	B101	RBCAI	anti-Sc70	-0.19079	0
B57	B104	RBCAI	anti-SSB	-0.19079	0
B57	B106	RBCAI	anti-P	-0.19079	0
B57	B107	RBCAI	AnuA	-0.19079	0
B57	B110	RBCAI	AHA	-0.19079	0
B57	B59	RBCAI	WBRV	0.555059	0
B57	B60	RBCAI	WBRVI	0.650941	0
B57	B64	RBCAI	WBRSR(mPas_5)	0.390919	0
B57	B65	RBCAI	WBRSR(mPas_1)	0.595561	0
B57	B69	RBCAI	PV	-0.20198	0
B57	B95	RBCAI	anti-cenpb	-0.19079	0
B57	B97	RBCAI	dsDNA	-0.19079	0
B57	B98	RBCAI	anti-Jo-1	-0.19079	0
B57	B99	RBCAI	anti-PM-Scl	-0.19079	0
B57	B157	RBCAI	IgG	-0.16615	2.64E-14
B57	B184	RBCAI	INR	-0.1642	5.28E-14
B57	B58	RBCAI	HCT	0.162359	1.01E-13
B57	B94	RBCAI	ALDH2-Lys504Lys	-0.15736	5.72E-13
B57	B93	RBCAI	ALDH2-Glu504Lys	0.138098	2.69E-10
B57	B92	RBCAI	ALDH2-Glu504Glu	-0.13435	8.11E-10
B57	B102	RBCAI	anti-Sm	-0.13409	8.75E-10
B57	B108	RBCAI	Anti-Cyclin	0.129992	2.82E-09
B57	B156	RBCAI	IgE	-0.12624	7.98E-09
B57	B109	RBCAI	AMA-M2	-0.11948	4.83E-08
B57	B183	RBCAI	PTA	-0.11742	8.20E-08
B57	B185	RBCAI	PT	-0.11273	2.64E-07
B58	B109	HCT	AMA-M2	-0.23809	0
B58	B60	HCT	WBRVI	0.553331	0
B58	B62	HCT	WBHSRVI	0.59494	0
B58	B63	HCT	WBRSR(mPas_200)	0.524321	0
B58	B64	HCT	WBRSR(mPas_5)	0.538985	0
B58	B65	HCT	WBRSR(mPas_1)	0.514321	0
B58	B66	HCT	WBRSR(mPas_100)	0.468438	0
B58	B67	HCT	WBRSR(mPas_50)	0.477662	0
B58	B96	HCT	dsDNA (IIF)	0.170963	4.44E-15

B58	B156	HCT	IgE	0.1666	2.24E-14
B58	B155	HCT	IgA	0.132438	1.41E-09
B58	B69	HCT	PV	-0.12887	3.87E-09
B58	B75	HCT	ApoA1/B	-0.12555	9.63E-09
B58	B197	HCT	AG	-0.11675	9.72E-08
B58	B196	HCT	TCO2	0.113572	2.15E-07
B58	B114	HCT	TSH TT3 TT4(TT4)	-0.11023	4.84E-07
B59	B60	WBRV	WBRVI	0.717387	0
B59	B61	WBRV	WBHSRV	0.755449	0
B59	B62	WBRV	WBHSRVI	0.497084	0
B59	B63	WBRV	WBRSR(mPas_200)	0.609618	0
B59	B64	WBRV	WBRSR(mPas_5)	0.812095	0
B59	B65	WBRV	WBRSR(mPas_1)	0.84867	0
B59	B66	WBRV	WBRSR(mPas_100)	0.640775	0
B59	B67	WBRV	WBRSR(mPas_50)	0.674756	0
B59	B75	WBRV	ApoA1/B	0.180326	0
B59	B100	WBRV	anti-Ro52	-0.15982	2.46E-13
B59	B101	WBRV	anti-Sc70	-0.15982	2.46E-13
B59	B104	WBRV	anti-SSB	-0.15982	2.46E-13
B59	B106	WBRV	anti-P	-0.15982	2.46E-13
B59	B107	WBRV	AnuA	-0.15982	2.46E-13
B59	B110	WBRV	AHA	-0.15982	2.46E-13
B59	B95	WBRV	anti-cenpb	-0.15982	2.46E-13
B59	B97	WBRV	dsDNA	-0.15982	2.46E-13
B59	B98	WBRV	anti-Jo-1	-0.15982	2.46E-13
B59	B99	WBRV	anti-PM-Scl	-0.15982	2.46E-13
B59	B76	WBRV	ApoB	-0.15074	5.20E-12
B59	B69	WBRV	PV	0.115186	1.44E-07
B59	B73	WBRV	ApoA	0.112528	2.78E-07
B6	B7	WB-Fe	WB-Cop	-0.23476	0
B6	B8	WB-Fe	WB-Zinc	0.590005	0
B6	B123	WB-Fe	hs-CRP1	-0.15593	9.27E-13
B6	B85	WB-Fe	SCC	-0.15103	4.73E-12
B6	B86	WB-Fe	TBhCG	0.135194	6.35E-10
B60	B102	WBRVI	anti-Sm	-0.30319	0
B60	B105	WBRVI	anti-U1RNP	-0.27361	0
B60	B61	WBRVI	WBHSRV	0.530508	0
B60	B62	WBRVI	WBHSRVI	0.796344	0
B60	B63	WBRVI	WBRSR(mPas_200)	0.603617	0
B60	B64	WBRVI	WBRSR(mPas_5)	0.836374	0
B60	B65	WBRVI	WBRSR(mPas_1)	0.895158	0
B60	B66	WBRVI	WBRSR(mPas_100)	0.641076	0
B60	B67	WBRVI	WBRSR(mPas_50)	0.680527	0
B60	B69	WBRVI	PV	-0.37162	0

B60	B93	WBRVI	ALDH2-Glu504Lys	0.173717	1.78E-15
B60	B92	WBRVI	ALDH2-Glu504Glu	-0.1709	4.66E-15
B60	B94	WBRVI	ALDH2-Lys504Lys	-0.15401	1.77E-12
B60	B76	WBRVI	ApoB	-0.14009	1.48E-10
B60	B100	WBRVI	anti-Ro52	-0.12681	6.84E-09
B60	B101	WBRVI	anti-Sc70	-0.12681	6.84E-09
B60	B104	WBRVI	anti-SSB	-0.12681	6.84E-09
B60	B106	WBRVI	anti-P	-0.12681	6.84E-09
B60	B107	WBRVI	AnuA	-0.12681	6.84E-09
B60	B110	WBRVI	AHA	-0.12681	6.84E-09
B60	B95	WBRVI	anti-cenpb	-0.12681	6.84E-09
B60	B97	WBRVI	dsDNA	-0.12681	6.84E-09
B60	B98	WBRVI	anti-Jo-1	-0.12681	6.84E-09
B60	B99	WBRVI	anti-PM-Scl	-0.12681	6.84E-09
B60	B125	WBRVI	Cor	0.126267	7.93E-09
B61	B62	WBHSRV	WBHSRVI	0.752583	0
B61	B63	WBHSRV	WBRSR(mPas_200)	0.860319	0
B61	B64	WBHSRV	WBRSR(mPas_5)	0.764671	0
B61	B65	WBHSRV	WBRSR(mPas_1)	0.646642	0
B61	B66	WBHSRV	WBRSR(mPas_100)	0.858801	0
B61	B67	WBHSRV	WBRSR(mPas_50)	0.850463	0
B61	B105	WBHSRV	anti-U1RNP	-0.15511	1.22E-12
B61	B73	WBHSRV	ApoA	0.151748	3.73E-12
B61	B102	WBHSRV	anti-Sm	-0.14516	3.10E-11
B61	B75	WBHSRV	ApoA1/B	0.135952	5.08E-10
B61	B109	WBHSRV	AMA-M2	0.128237	4.61E-09
B61	B96	WBHSRV	dsDNA (IIF)	0.120998	3.25E-08
B61	B76	WBHSRV	ApoB	-0.10821	7.82E-07
B62	B102	WBHSRVI	anti-Sm	-0.31218	0
B62	B105	WBHSRVI	anti-U1RNP	-0.30357	0
B62	B63	WBHSRVI	WBRSR(mPas_200)	0.82684	0
B62	B64	WBHSRVI	WBRSR(mPas_5)	0.780748	0
B62	B65	WBHSRVI	WBRSR(mPas_1)	0.695639	0
B62	B66	WBHSRVI	WBRSR(mPas_100)	0.830438	0
B62	B67	WBHSRVI	WBRSR(mPas_50)	0.830203	0
B62	B69	WBHSRVI	PV	-0.34745	0
B62	B96	WBHSRVI	dsDNA (IIF)	0.167919	1.38E-14
B62	B93	WBHSRVI	ALDH2-Glu504Lys	0.120301	3.90E-08
B62	B92	WBHSRVI	ALDH2-Glu504Glu	-0.11958	4.70E-08
B62	B124	WBHSRVI	ACTH	0.118056	6.97E-08
B62	B195	WBHSRVI	Na	-0.11756	7.91E-08
B62	B155	WBHSRVI	IgA	0.116073	1.15E-07
B63	B64	WBRSR(mPas_200)	WBRSR(mPas_5)	0.896778	0
B63	B65	WBRSR(mPas_200)	WBRSR(mPas_1)	0.768705	0

B63	B66	WBRSR(mPas_200)	WBRSR(mPas_100)	0.953376	0
B63	B67	WBRSR(mPas_200)	WBRSR(mPas_50)	0.946992	0
B63	B69	WBRSR(mPas_200)	PV	0.224471	0
B63	B96	WBRSR(mPas_200)	dsDNA (IIF)	0.198301	0
B63	B182	WBRSR(mPas_200)	TT	-0.15973	2.54E-13
B63	B157	WBRSR(mPas_200)	IgG	0.153057	2.43E-12
B63	B156	WBRSR(mPas_200)	IgE	0.14025	1.41E-10
B63	B155	WBRSR(mPas_200)	IgA	0.140135	1.46E-10
B63	B185	WBRSR(mPas_200)	PT	0.128025	4.89E-09
B63	B195	WBRSR(mPas_200)	Na	-0.11785	7.34E-08
B63	B73	WBRSR(mPas_200)	ApoA	0.115192	1.44E-07
B63	B123	WBRSR(mPas_200)	hs-CRP1	0.112521	2.78E-07
B64	B65	WBRSR(mPas_5)	WBRSR(mPas_1)	0.971511	0
B64	B66	WBRSR(mPas_5)	WBRSR(mPas_100)	0.881656	0
B64	B67	WBRSR(mPas_5)	WBRSR(mPas_50)	0.9046	0
B64	B96	WBRSR(mPas_5)	dsDNA (IIF)	0.150826	5.05E-12
B64	B123	WBRSR(mPas_5)	hs-CRP1	0.138812	2.17E-10
B64	B69	WBRSR(mPas_5)	PV	0.13498	6.75E-10
B64	B93	WBRSR(mPas_5)	ALDH2-Glu504Lys	0.126762	6.92E-09
B64	B92	WBRSR(mPas_5)	ALDH2-Glu504Glu	-0.12642	7.60E-09
B64	B155	WBRSR(mPas_5)	IgA	0.121544	2.82E-08
B64	B100	WBRSR(mPas_5)	anti-Ro52	-0.11736	8.32E-08
B64	B101	WBRSR(mPas_5)	anti-Sc70	-0.11736	8.32E-08
B64	B104	WBRSR(mPas_5)	anti-SSB	-0.11736	8.32E-08
B64	B106	WBRSR(mPas_5)	anti-P	-0.11736	8.32E-08
B64	B107	WBRSR(mPas_5)	AnuA	-0.11736	8.32E-08
B64	B110	WBRSR(mPas_5)	AHA	-0.11736	8.32E-08
B64	B95	WBRSR(mPas_5)	anti-cenpb	-0.11736	8.32E-08
B64	B97	WBRSR(mPas_5)	dsDNA	-0.11736	8.32E-08
B64	B98	WBRSR(mPas_5)	anti-Jo-1	-0.11736	8.32E-08
B64	B99	WBRSR(mPas_5)	anti-PM-Scl	-0.11736	8.32E-08
B64	B182	WBRSR(mPas_5)	TT	-0.10952	5.74E-07
B64	B195	WBRSR(mPas_5)	Na	-0.10862	7.11E-07
B65	B66	WBRSR(mPas_1)	WBRSR(mPas_100)	0.768386	0
B65	B67	WBRSR(mPas_1)	WBRSR(mPas_50)	0.804901	0
B65	B100	WBRSR(mPas_1)	anti-Ro52	-0.14979	7.07E-12
B65	B101	WBRSR(mPas_1)	anti-Sc70	-0.14979	7.07E-12
B65	B104	WBRSR(mPas_1)	anti-SSB	-0.14979	7.07E-12
B65	B106	WBRSR(mPas_1)	anti-P	-0.14979	7.07E-12
B65	B107	WBRSR(mPas_1)	AnuA	-0.14979	7.07E-12
B65	B110	WBRSR(mPas_1)	AHA	-0.14979	7.07E-12
B65	B95	WBRSR(mPas_1)	anti-cenpb	-0.14979	7.07E-12
B65	B97	WBRSR(mPas_1)	dsDNA	-0.14979	7.07E-12
B65	B98	WBRSR(mPas_1)	anti-Jo-1	-0.14979	7.07E-12

B65	B99	WBRSR(mPas_1)	anti-PM-Scl	-0.14979	7.07E-12
B65	B93	WBRSR(mPas_1)	ALDH2-Glu504Lys	0.143796	4.74E-11
B65	B92	WBRSR(mPas_1)	ALDH2-Glu504Glu	-0.1426	6.87E-11
B65	B123	WBRSR(mPas_1)	hs-CRP1	0.139492	1.77E-10
B65	B96	WBRSR(mPas_1)	dsDNA (IIF)	0.116376	1.07E-07
B65	B76	WBRSR(mPas_1)	ApoB	-0.10936	5.97E-07
B66	B67	WBRSR(mPas_100)	WBRSR(mPas_50)	0.997582	0
B66	B96	WBRSR(mPas_100)	dsDNA (IIF)	0.195885	0
B66	B155	WBRSR(mPas_100)	IgA	0.157931	4.71E-13
B66	B157	WBRSR(mPas_100)	IgG	0.157818	4.89E-13
B66	B69	WBRSR(mPas_100)	PV	0.155539	1.06E-12
B66	B182	WBRSR(mPas_100)	TT	-0.15032	5.95E-12
B66	B156	WBRSR(mPas_100)	IgE	0.129713	3.05E-09
B66	B73	WBRSR(mPas_100)	ApoA	0.126354	7.74E-09
B66	B195	WBRSR(mPas_100)	Na	-0.12412	1.42E-08
B66	B123	WBRSR(mPas_100)	hs-CRP1	0.12057	3.64E-08
B66	B185	WBRSR(mPas_100)	PT	0.120047	4.17E-08
B67	B96	WBRSR(mPas_50)	dsDNA (IIF)	0.189785	0
B67	B155	WBRSR(mPas_50)	IgA	0.15587	9.48E-13
B67	B69	WBRSR(mPas_50)	PV	0.146308	2.16E-11
B67	B157	WBRSR(mPas_50)	IgG	0.145326	2.94E-11
B67	B182	WBRSR(mPas_50)	TT	-0.14039	1.35E-10
B67	B123	WBRSR(mPas_50)	hs-CRP1	0.126393	7.66E-09
B67	B156	WBRSR(mPas_50)	IgE	0.126216	8.04E-09
B67	B73	WBRSR(mPas_50)	ApoA	0.125507	9.75E-09
B67	B195	WBRSR(mPas_50)	Na	-0.12287	1.98E-08
B67	B185	WBRSR(mPas_50)	PT	0.110386	4.67E-07
B67	B194	WBRSR(mPas_50)	Cl	-0.10977	5.41E-07
B69	B102	PV	anti-Sm	0.627639	0
B69	B105	PV	anti-U1RNP	0.604828	0
B69	B125	PV	Cor	-0.2453	0
B69	B138	PV	PAB	-0.22453	0
B69	B156	PV	IgE	0.230995	0
B69	B185	PV	PT	0.2679	0
B69	B124	PV	ACTH	-0.17937	2.22E-16
B69	B186	PV	PFC	0.167264	1.78E-14
B69	B181	PV	APTT	0.155367	1.12E-12
B69	B184	PV	INR	0.137843	2.90E-10
B69	B139	PV	ADA	0.132056	1.57E-09
B69	B120	PV	HCV-cAg	-0.11966	4.61E-08
B69	B100	PV	anti-Ro52	-0.11922	5.17E-08
B69	B101	PV	anti-Sc70	-0.11922	5.17E-08
B69	B104	PV	anti-SSB	-0.11922	5.17E-08
B69	B106	PV	anti-P	-0.11922	5.17E-08

B69	B107	PV	AnuA	-0.11922	5.17E-08
B69	B110	PV	AHA	-0.11922	5.17E-08
B69	B95	PV	anti-cenpb	-0.11922	5.17E-08
B69	B97	PV	dsDNA	-0.11922	5.17E-08
B69	B98	PV	anti-Jo-1	-0.11922	5.17E-08
B69	B99	PV	anti-PM-Scl	-0.11922	5.17E-08
B69	B182	PV	TT	-0.11496	1.52E-07
B69	B228	PV	OS	0.111954	3.20E-07
B7	B197	WB-Cop	AG	-0.18106	0
B7	B8	WB-Cop	WB-Zinc	0.401874	0
B7	B196	WB-Cop	TCO2	0.127191	6.15E-09
B70	B132	LDL-C	Dbil	-0.30711	0
B70	B76	LDL-C	ApoB	0.232088	0
B70	B77	LDL-C	TC	0.894083	0
B70	B75	LDL-C	ApoA1/B	-0.17666	4.44E-16
B70	B220	LDL-C	SBP	0.167237	1.78E-14
B70	B91	LDL-C	TP	0.163778	6.15E-14
B70	B130	LDL-C	GLB	0.160707	1.81E-13
B70	B133	LDL-C	TBIL	-0.15675	7.03E-13
B70	B218	LDL-C	DBP	0.150072	6.45E-12
B70	B127	LDL-C	A-G ratio	-0.13201	1.59E-09
B70	B219	LDL-C	OT	-0.10818	7.88E-07
B71	B132	TG	Dbil	-0.18427	0
B71	B153	TG	Glu	0.204701	0
B71	B206	TG	Waist	0.186522	0
B71	B215	TG	BMI	0.225808	0
B71	B216	TG	BMI	0.19205	0
B71	B72	TG	HDL-C	-0.36876	0
B71	B77	TG	TC	0.281525	0
B71	B91	TG	TP	0.206669	0
B71	B192	TG	UA	0.162318	1.03E-13
B71	B210	TG	Hip	0.161448	1.40E-13
B71	B130	TG	GLB	0.142316	7.49E-11
B71	B126	TG	Alb	0.129953	2.85E-09
B71	B141	TG	AST/ALT	-0.1285	4.28E-09
B71	B222	TG	NS	0.124097	1.43E-08
B71	B140	TG	r-GT	0.118755	5.82E-08
B72	B141	HDL-C	AST/ALT	0.239425	0
B72	B192	HDL-C	UA	-0.33507	0
B72	B206	HDL-C	Waist	-0.34279	0
B72	B207	HDL-C	WHR	-0.21362	0
B72	B210	HDL-C	Hip	-0.26256	0
B72	B215	HDL-C	BMI	-0.32139	0
B72	B216	HDL-C	BMI	-0.41338	0

B72	B221	HDL-C	Hei	-0.29805	0
B72	B222	HDL-C	NS	-0.35151	0
B72	B74	HDL-C	ApoA1	0.248652	0
B72	B77	HDL-C	TC	0.288421	0
B72	B174	HDL-C	Urea/Cr	0.147612	1.42E-11
B72	B112	HDL-C	FT4	0.142874	6.31E-11
B72	B163	HDL-C	SG	-0.14282	6.41E-11
B72	B190	HDL-C	Cre	-0.14118	1.06E-10
B72	B75	HDL-C	ApoA1/B	0.131193	2.01E-09
B72	B128	HDL-C	ALT	-0.11846	6.29E-08
B72	B153	HDL-C	Glu	-0.11623	1.11E-07
B72	B140	HDL-C	r-GT	-0.11562	1.29E-07
B73	B200	ApoA	OP	0.12417	1.40E-08
B73	B196	ApoA	TCO2	0.114981	1.52E-07
B73	B74	ApoA	ApoA1	0.10857	7.19E-07
B74	B75	ApoA1	ApoA1/B	0.462701	0
B74	B156	ApoA1	IgE	0.165703	3.11E-14
B74	B195	ApoA1	Na	-0.16265	9.15E-14
B74	B216	ApoA1	BMI	-0.15862	3.72E-13
B74	B200	ApoA1	OP	-0.1532	2.31E-12
B74	B151	ApoA1	Anti-CCP	0.137985	2.78E-10
B74	B221	ApoA1	Hei	-0.1347	7.34E-10
B74	B94	ApoA1	ALDH2-Lys504Lys	0.134621	7.50E-10
B74	B181	ApoA1	APTT	0.134583	7.58E-10
B74	B206	ApoA1	Waist	-0.12092	3.32E-08
B74	B136	ApoA1	5'NT	-0.12007	4.14E-08
B74	B141	ApoA1	AST/ALT	0.115233	1.42E-07
B74	B108	ApoA1	Anti-Cyclin	-0.11516	1.45E-07
B74	B81	ApoA1	TSGF	-0.11022	4.86E-07
B74	B222	ApoA1	NS	-0.10936	5.97E-07
B75	B76	ApoA1/B	ApoB	-0.76249	0
B75	B197	ApoA1/B	AG	-0.14704	1.71E-11
B75	B135	ApoA1/B	TBA	0.128806	3.93E-09
B75	B77	ApoA1/B	TC	-0.12513	1.08E-08
B75	B199	ApoA1/B	Mg	-0.12158	2.79E-08
B75	B139	ApoA1/B	ADA	-0.11971	4.55E-08
B75	B90	ApoA1/B	CYFRA 21-1	0.116256	1.10E-07
B75	B134	ApoA1/B	CHE	-0.11099	4.04E-07
B76	B197	ApoB	AG	0.198419	0
B76	B77	ApoB	TC	0.215737	0
B76	B100	ApoB	anti-Ro52	0.165585	3.24E-14
B76	B101	ApoB	anti-Sc70	0.165585	3.24E-14
B76	B104	ApoB	anti-SSB	0.165585	3.24E-14
B76	B106	ApoB	anti-P	0.165585	3.24E-14

B76	B107	ApoB	AnuA	0.165585	3.24E-14
B76	B110	ApoB	AHA	0.165585	3.24E-14
B76	B95	ApoB	anti-cenpb	0.165585	3.24E-14
B76	B97	ApoB	dsDNA	0.165585	3.24E-14
B76	B98	ApoB	anti-Jo-1	0.165585	3.24E-14
B76	B99	ApoB	anti-PM-Scl	0.165585	3.24E-14
B76	B134	ApoB	CHE	0.147647	1.41E-11
B76	B193	ApoB	K	0.135304	6.15E-10
B76	B194	ApoB	Cl	-0.12598	8.58E-09
B76	B135	ApoB	TBA	-0.11692	9.30E-08
B76	B159	ApoB	β2-MG	0.11375	2.06E-07
B76	B181	ApoB	APTT	0.109559	5.69E-07
B77	B132	TC	Dbil	-0.31191	0
B77	B220	TC	SBP	0.192433	0
B77	B221	TC	Hei	-0.19209	0
B77	B91	TC	TP	0.174294	1.33E-15
B77	B130	TC	GLB	0.159681	2.58E-13
B77	B133	TC	TBIL	-0.15509	1.23E-12
B77	B222	TC	NS	-0.13906	2.01E-10
B77	B218	TC	DBP	0.13478	7.16E-10
B77	B219	TC	OT	-0.13141	1.89E-09
B77	B127	TC	A-G ratio	-0.12371	1.58E-08
B77	B216	TC	BMI	-0.12168	2.72E-08
B77	B174	TC	Urea/Cr	0.109743	5.44E-07
B77	B190	TC	Cre	-0.10791	8.40E-07
B78	B96	HBcAb	dsDNA (IIF)	-0.23949	0
B78	B80	HBcAb	anti-HP IgG	-0.14201	8.22E-11
B79	B100	hb V -dna	anti-Ro52	0.246533	0
B79	B101	hb V -dna	anti-Sc70	0.246533	0
B79	B104	hb V -dna	anti-SSB	0.246533	0
B79	B106	hb V -dna	anti-P	0.246533	0
B79	B107	hb V -dna	AnuA	0.246533	0
B79	B108	hb V -dna	Anti-Cyclin	-0.26259	0
B79	B110	hb V -dna	AHA	0.246533	0
B79	B119	hb V -dna	Hcv-rna	0.369822	0
B79	B121	hb V -dna	C3	-0.36982	0
B79	B122	hb V -dna	C4	-0.36982	0
B79	B125	hb V -dna	Cor	0.370172	0
B79	B135	hb V -dna	TBA	-0.21863	0
B79	B152	hb V -dna	ACA-Ig(G-A-M)	-0.36992	0
B79	B169	hb V -dna	U-CRY	-0.36983	0
B79	B182	hb V -dna	TT	-0.20476	0
B79	B183	hb V -dna	PTA	0.250114	0
B79	B81	hb V -dna	TSGF	-0.33585	0

B79	B92	hb V -dna	ALDH2-Glu504Glu	0.306911	0
B79	B93	hb V -dna	ALDH2-Glu504Lys	-0.32643	0
B79	B94	hb V -dna	ALDH2-Lys504Lys	0.618974	0
B79	B95	hb V -dna	anti-cenpb	0.246533	0
B79	B97	hb V -dna	dsDNA	0.246533	0
B79	B98	hb V -dna	anti-Jo-1	0.246533	0
B79	B99	hb V -dna	anti-PM-Scl	0.246533	0
B79	B124	hb V -dna	ACTH	0.15953	2.72E-13
B79	B186	hb V -dna	PFC	0.142622	6.82E-11
B79	B120	hb V -dna	HCV-cAg	0.136368	4.50E-10
B79	B137	hb V -dna	AFU	-0.13135	1.92E-09
B79	B195	hb V -dna	Na	-0.12616	8.15E-09
B79	B187	hb V -dna	NSE	0.116356	1.07E-07
B79	B138	hb V -dna	PAB	-0.11598	1.18E-07
B81	B108	TSGF	Anti-Cyclin	0.286663	0
B81	B182	TSGF	TT	0.2381	0
B81	B94	TSGF	ALDH2-Lys504Lys	-0.45344	0
B81	B138	TSGF	PAB	0.176402	4.44E-16
B81	B157	TSGF	IgG	-0.17373	1.78E-15
B81	B93	TSGF	ALDH2-Glu504Lys	0.163201	7.55E-14
B81	B125	TSGF	Cor	-0.15	6.60E-12
B81	B92	TSGF	ALDH2-Glu504Glu	-0.14722	1.61E-11
B81	B184	TSGF	INR	-0.13964	1.69E-10
B81	B183	TSGF	PTA	-0.13541	5.96E-10
B81	B181	TSGF	APTT	-0.1228	2.02E-08
B81	B198	TSGF	P	0.117888	7.27E-08
B81	B102	TSGF	anti-Sm	-0.11631	1.08E-07
B81	B100	TSGF	anti-Ro52	-0.11339	2.25E-07
B81	B101	TSGF	anti-Sc70	-0.11339	2.25E-07
B81	B104	TSGF	anti-SSB	-0.11339	2.25E-07
B81	B106	TSGF	anti-P	-0.11339	2.25E-07
B81	B107	TSGF	AnuA	-0.11339	2.25E-07
B81	B110	TSGF	AHA	-0.11339	2.25E-07
B81	B95	TSGF	anti-cenpb	-0.11339	2.25E-07
B81	B97	TSGF	dsDNA	-0.11339	2.25E-07
B81	B98	TSGF	anti-Jo-1	-0.11339	2.25E-07
B81	B99	TSGF	anti-PM-Scl	-0.11339	2.25E-07
B82	B116	CEA	TT3	-0.20162	0
B82	B83	CEA	AFP	0.202669	0
B82	B86	CEA	TBhCG	-0.41291	0
B82	B88	CEA	CA 242	0.422788	0
B82	B85	CEA	SCC	0.151277	4.36E-12
B82	B87	CEA	NSE	0.126604	7.23E-09
B82	B117	CEA	TT4	-0.10987	5.28E-07

B83	B84	AFP	C-PSA	0.279306	0
B83	B85	AFP	SCC	0.251752	0
B83	B87	AFP	NSE	0.178779	2.22E-16
B83	B115	AFP	TSH TT3 TT4(TT3)	-0.13693	3.81E-10
B84	B115	C-PSA	TSH TT3 TT4(TT3)	-0.23336	0
B84	B86	C-PSA	TBhCG	-0.202	0
B84	B199	C-PSA	Mg	0.175304	8.88E-16
B84	B227	C-PSA	OD	0.138389	2.47E-10
B84	B90	C-PSA	CYFRA 21-1	0.135793	5.32E-10
B84	B200	C-PSA	OP	0.131914	1.64E-09
B84	B87	C-PSA	NSE	0.121875	2.58E-08
B84	B114	C-PSA	TSH TT3 TT4(TT4)	-0.10763	8.98E-07
B85	B87	SCC	NSE	0.264954	0
B85	B90	SCC	CYFRA 21-1	0.203145	0
B85	B117	SCC	TT4	-0.15783	4.87E-13
B85	B116	SCC	TT3	-0.1528	2.64E-12
B86	B87	TBhCG	NSE	-0.32069	0
B86	B88	TBhCG	CA 242	-0.2504	0
B86	B89	TBhCG	CA 72-4	0.250829	0
B86	B200	TBhCG	OP	-0.16667	2.18E-14
B86	B195	TBhCG	Na	-0.15102	4.73E-12
B86	B199	TBhCG	Mg	-0.14759	1.43E-11
B86	B129	TBhCG	IBIL	-0.13575	5.39E-10
B86	B133	TBhCG	TBIL	-0.12828	4.56E-09
B86	B194	TBhCG	Cl	-0.11851	6.20E-08
B86	B196	TBhCG	TCO2	0.117592	7.84E-08
B86	B197	TBhCG	AG	-0.11292	2.52E-07
B87	B89	NSE	CA 72-4	-0.17615	6.66E-16
B87	B88	NSE	CA 242	0.175175	8.88E-16
B87	B129	NSE	IBIL	0.120344	3.86E-08
B88	B186	CA 242	PFC	0.151794	3.68E-12
B88	B90	CA 242	CYFRA 21-1	0.115404	1.36E-07
B9	B100	RH	anti-Ro52	0.300783	0
B9	B101	RH	anti-Sc70	0.300783	0
B9	B104	RH	anti-SSB	0.300783	0
B9	B106	RH	anti-P	0.300783	0
B9	B107	RH	AnuA	0.300783	0
B9	B110	RH	AHA	0.300783	0
B9	B125	RH	Cor	0.309099	0
B9	B15	RH	CD3-CD19+(B cell)	0.250125	0
B9	B151	RH	Anti-CCP	0.197844	0
B9	B185	RH	PT	-0.22196	0
B9	B19	RH	CD3+CD4+/CD3+CD8+	0.235304	0
B9	B21	RH	CD3+CD4-CD8-	0.313829	0

B9	B22	RH	CD3+CD8+(T)	-0.34507	0
B9	B57	RH	RBCAI	0.198628	0
B9	B62	RH	WBHSRVI	-0.1838	0
B9	B79	RH	hb V -dna	0.276546	0
B9	B81	RH	TSGF	-0.22435	0
B9	B92	RH	ALDH2-Glu504Glu	0.538708	0
B9	B93	RH	ALDH2-Glu504Lys	-0.54594	0
B9	B94	RH	ALDH2-Lys504Lys	0.446782	0
B9	B95	RH	anti-cenpb	0.300783	0
B9	B97	RH	dsDNA	0.300783	0
B9	B98	RH	anti-Jo-1	0.300783	0
B9	B99	RH	anti-PM-Scl	0.300783	0
B9	B196	RH	TCO2	0.163517	6.75E-14
B9	B138	RH	PAB	0.16234	1.02E-13
B9	B56	RH	IR	-0.15821	4.28E-13
B9	B195	RH	Na	0.156877	6.74E-13
B9	B66	RH	WBRSR(mPas_100)	-0.15685	6.81E-13
B9	B183	RH	PTA	0.151496	4.06E-12
B9	B63	RH	WBRSR(mPas_200)	-0.15148	4.08E-12
B9	B74	RH	ApoA1	0.150469	5.67E-12
B9	B67	RH	WBRSR(mPas_50)	-0.14034	1.37E-10
B9	B61	RH	WBHSRV	-0.13672	4.05E-10
B9	B155	RH	IgA	-0.12849	4.30E-09
B9	B182	RH	TT	0.127238	6.07E-09
B9	B16	RH	CD3+T	-0.12323	1.80E-08
B9	B156	RH	IgE	0.123162	1.83E-08
B9	B200	RH	OP	0.118647	5.99E-08
B9	B186	RH	PFC	-0.11427	1.81E-07
B9	B181	RH	APTT	-0.11378	2.04E-07
B90	B157	CYFRA 21-1	IgG	0.1155	1.33E-07
B91	B126	TP	Alb	0.442776	0
B91	B127	TP	A-G ratio	-0.53138	0
B91	B130	TP	GLB	0.809972	0
B91	B188	TP	Ca	0.225093	0
B91	B209	TP	Smoking	-0.13261	1.34E-09
B91	B153	TP	Glu	0.11681	9.57E-08
B91	B131	TP	AST	0.115072	1.48E-07
B91	B192	TP	UA	0.110114	4.98E-07
B91	B220	TP	SBP	0.108919	6.62E-07
B92	B100	ALDH2-Glu504Glu	anti-Ro52	0.487165	0
B92	B101	ALDH2-Glu504Glu	anti-Sc70	0.487165	0
B92	B104	ALDH2-Glu504Glu	anti-SSB	0.487165	0
B92	B106	ALDH2-Glu504Glu	anti-P	0.487165	0
B92	B107	ALDH2-Glu504Glu	AnuA	0.487164	0

B92	B108	ALDH2-Glu504Glu	Anti-Cyclin	-0.51096	0
B92	B110	ALDH2-Glu504Glu	AHA	0.487165	0
B92	B135	ALDH2-Glu504Glu	TBA	-0.19776	0
B92	B182	ALDH2-Glu504Glu	TT	0.288465	0
B92	B185	ALDH2-Glu504Glu	PT	-0.19664	0
B92	B93	ALDH2-Glu504Glu	ALDH2-Glu504Lys	-0.99933	0
B92	B94	ALDH2-Glu504Glu	ALDH2-Lys504Lys	0.495838	0
B92	B95	ALDH2-Glu504Glu	anti-cenpb	0.487165	0
B92	B97	ALDH2-Glu504Glu	dsDNA	0.487165	0
B92	B98	ALDH2-Glu504Glu	anti-Jo-1	0.487165	0
B92	B99	ALDH2-Glu504Glu	anti-PM-Scl	0.487165	0
B92	B124	ALDH2-Glu504Glu	ACTH	-0.17683	4.44E-16
B92	B183	ALDH2-Glu504Glu	PTA	0.167879	1.42E-14
B92	B198	ALDH2-Glu504Glu	P	-0.15761	5.26E-13
B92	B138	ALDH2-Glu504Glu	PAB	0.129778	3.00E-09
B92	B194	ALDH2-Glu504Glu	Cl	0.125513	9.74E-09
B92	B155	ALDH2-Glu504Glu	IgA	-0.1205	3.70E-08
B92	B105	ALDH2-Glu504Glu	anti-U1RNP	-0.11636	1.07E-07
B93	B100	ALDH2-Glu504Lys	anti-Ro52	-0.49364	0
B93	B101	ALDH2-Glu504Lys	anti-Sc70	-0.49364	0
B93	B104	ALDH2-Glu504Lys	anti-SSB	-0.49364	0
B93	B106	ALDH2-Glu504Lys	anti-P	-0.49364	0
B93	B107	ALDH2-Glu504Lys	AnuA	-0.49364	0
B93	B108	ALDH2-Glu504Lys	Anti-Cyclin	0.517823	0
B93	B110	ALDH2-Glu504Lys	AHA	-0.49364	0
B93	B135	ALDH2-Glu504Lys	TBA	0.197295	0
B93	B182	ALDH2-Glu504Lys	TT	-0.26748	0
B93	B185	ALDH2-Glu504Lys	PT	0.191832	0
B93	B94	ALDH2-Glu504Lys	ALDH2-Lys504Lys	-0.52738	0
B93	B95	ALDH2-Glu504Lys	anti-cenpb	-0.49364	0
B93	B97	ALDH2-Glu504Lys	dsDNA	-0.49364	0
B93	B98	ALDH2-Glu504Lys	anti-Jo-1	-0.49364	0
B93	B99	ALDH2-Glu504Lys	anti-PM-Scl	-0.49364	0
B93	B183	ALDH2-Glu504Lys	PTA	-0.17845	2.22E-16
B93	B124	ALDH2-Glu504Lys	ACTH	0.167659	1.53E-14
B93	B198	ALDH2-Glu504Lys	P	0.1635	6.79E-14
B93	B138	ALDH2-Glu504Lys	PAB	-0.1239	1.50E-08
B93	B194	ALDH2-Glu504Lys	Cl	-0.12215	2.40E-08
B93	B155	ALDH2-Glu504Lys	IgA	0.119302	5.06E-08
B93	B105	ALDH2-Glu504Lys	anti-U1RNP	0.117893	7.26E-08
B94	B100	ALDH2-Lys504Lys	anti-Ro52	0.402497	0
B94	B101	ALDH2-Lys504Lys	anti-Sc70	0.402497	0
B94	B104	ALDH2-Lys504Lys	anti-SSB	0.402497	0
B94	B106	ALDH2-Lys504Lys	anti-P	0.402497	0

B94	B107	ALDH2-Lys504Lys	AnuA	0.402496	0
B94	B108	ALDH2-Lys504Lys	Anti-Cyclin	-0.42389	0
B94	B110	ALDH2-Lys504Lys	AHA	0.402497	0
B94	B125	ALDH2-Lys504Lys	Cor	0.363801	0
B94	B139	ALDH2-Lys504Lys	ADA	0.214157	0
B94	B182	ALDH2-Lys504Lys	TT	-0.34882	0
B94	B183	ALDH2-Lys504Lys	PTA	0.336162	0
B94	B184	ALDH2-Lys504Lys	INR	0.222288	0
B94	B198	ALDH2-Lys504Lys	P	-0.22006	0
B94	B95	ALDH2-Lys504Lys	anti-cenpb	0.402497	0
B94	B97	ALDH2-Lys504Lys	dsDNA	0.402497	0
B94	B98	ALDH2-Lys504Lys	anti-Jo-1	0.402497	0
B94	B99	ALDH2-Lys504Lys	anti-PM-Scl	0.402497	0
B94	B157	ALDH2-Lys504Lys	IgG	0.155732	9.93E-13
B94	B158	ALDH2-Lys504Lys	IgM	0.146643	1.94E-11
B94	B187	ALDH2-Lys504Lys	NSE	0.141212	1.05E-10
B94	B124	ALDH2-Lys504Lys	ACTH	0.126515	7.41E-09
B94	B186	ALDH2-Lys504Lys	PFC	0.125412	1.00E-08
B94	B156	ALDH2-Lys504Lys	IgE	0.125122	1.08E-08
B94	B154	ALDH2-Lys504Lys	RF	0.123335	1.75E-08
B94	B140	ALDH2-Lys504Lys	r-GT	-0.1184	6.39E-08
B95	B100	anti-cenpb	anti-Ro52	1	0
B95	B102	anti-cenpb	anti-Sm	-0.27973	0
B95	B104	anti-cenpb	anti-SSB	1	0
B95	B105	anti-cenpb	anti-U1RNP	-0.33755	0
B95	B107	anti-cenpb	AnuA	0.999998	0
B95	B108	anti-cenpb	Anti-Cyclin	-0.22672	0
B95	B109	anti-cenpb	AMA-M2	-0.26181	0
B95	B110	anti-cenpb	AHA	1	0
B95	B125	anti-cenpb	Cor	0.234516	0
B95	B154	anti-cenpb	RF	0.204755	0
B95	B183	anti-cenpb	PTA	0.273181	0
B95	B96	anti-cenpb	dsDNA (IIF)	0.300783	0
B95	B97	anti-cenpb	dsDNA	1	0
B95	B155	anti-cenpb	IgA	-0.16404	5.60E-14
B95	B156	anti-cenpb	IgE	-0.14475	3.52E-11
B95	B181	anti-cenpb	APTT	0.13878	2.19E-10
B95	B195	anti-cenpb	Na	0.137023	3.70E-10
B95	B103	anti-cenpb	anti-SSA/Ro	-0.13644	4.40E-10
B95	B137	anti-cenpb	AFU	-0.12881	3.93E-09
B95	B198	anti-cenpb	P	-0.12517	1.07E-08
B95	B139	anti-cenpb	ADA	0.118712	5.89E-08
B95	B138	anti-cenpb	PAB	0.113683	2.09E-07
B95	B185	anti-cenpb	PT	-0.10972	5.48E-07

B96	B100	dsDNA (IIF)	anti-Ro52	0.300783	0
B96	B101	dsDNA (IIF)	anti-Sc70	0.300783	0
B96	B104	dsDNA (IIF)	anti-SSB	0.300783	0
B96	B106	dsDNA (IIF)	anti-P	0.300783	0
B96	B107	dsDNA (IIF)	AnuA	0.300783	0
B96	B110	dsDNA (IIF)	AHA	0.300783	0
B96	B97	dsDNA (IIF)	dsDNA	0.300783	0
B96	B98	dsDNA (IIF)	anti-Jo-1	0.300783	0
B96	B99	dsDNA (IIF)	anti-PM-Scl	0.300783	0
B96	B155	dsDNA (IIF)	IgA	0.167849	1.42E-14
B96	B157	dsDNA (IIF)	IgG	0.153947	1.81E-12
B96	B151	dsDNA (IIF)	Anti-CCP	-0.11584	1.22E-07
B96	B158	dsDNA (IIF)	IgM	0.107858	8.51E-07
B97	B101	dsDNA	anti-Sc70	1	0
B97	B102	dsDNA	anti-Sm	-0.27973	0
B97	B104	dsDNA	anti-SSB	1	0
B97	B105	dsDNA	anti-U1RNP	-0.33755	0
B97	B106	dsDNA	anti-P	1	0
B97	B107	dsDNA	AnuA	0.999998	0
B97	B108	dsDNA	Anti-Cyclin	-0.22672	0
B97	B109	dsDNA	AMA-M2	-0.26181	0
B97	B110	dsDNA	AHA	1	0
B97	B125	dsDNA	Cor	0.234516	0
B97	B154	dsDNA	RF	0.204755	0
B97	B183	dsDNA	PTA	0.273181	0
B97	B99	dsDNA	anti-PM-Scl	1	0
B97	B155	dsDNA	IgA	-0.16404	5.60E-14
B97	B156	dsDNA	IgE	-0.14475	3.52E-11
B97	B181	dsDNA	APTT	0.13878	2.19E-10
B97	B195	dsDNA	Na	0.137023	3.70E-10
B97	B103	dsDNA	anti-SSA/Ro	-0.13644	4.40E-10
B97	B137	dsDNA	AFU	-0.12881	3.93E-09
B97	B198	dsDNA	P	-0.12517	1.07E-08
B97	B139	dsDNA	ADA	0.118712	5.89E-08
B97	B138	dsDNA	PAB	0.113683	2.09E-07
B97	B185	dsDNA	PT	-0.10972	5.48E-07
B98	B102	anti-Jo-1	anti-Sm	-0.27973	0
B98	B105	anti-Jo-1	anti-U1RNP	-0.33755	0
B98	B107	anti-Jo-1	AnuA	0.999998	0
B98	B108	anti-Jo-1	Anti-Cyclin	-0.22672	0
B98	B109	anti-Jo-1	AMA-M2	-0.26181	0
B98	B125	anti-Jo-1	Cor	0.234516	0
B98	B154	anti-Jo-1	RF	0.204755	0
B98	B183	anti-Jo-1	PTA	0.273181	0

B98	B155	anti-Jo-1	IgA	-0.16404	5.60E-14
B98	B156	anti-Jo-1	IgE	-0.14475	3.52E-11
B98	B181	anti-Jo-1	APTT	0.13878	2.19E-10
B98	B195	anti-Jo-1	Na	0.137023	3.70E-10
B98	B103	anti-Jo-1	anti-SSA/Ro	-0.13644	4.40E-10
B98	B137	anti-Jo-1	AFU	-0.12881	3.93E-09
B98	B198	anti-Jo-1	P	-0.12517	1.07E-08
B98	B139	anti-Jo-1	ADA	0.118712	5.89E-08
B98	B138	anti-Jo-1	PAB	0.113683	2.09E-07
B98	B185	anti-Jo-1	PT	-0.10972	5.48E-07
B99	B101	anti-PM-Scl	anti-Sc70	1	0
B99	B102	anti-PM-Scl	anti-Sm	-0.27973	0
B99	B104	anti-PM-Scl	anti-SSB	1	0
B99	B105	anti-PM-Scl	anti-U1RNP	-0.33755	0
B99	B107	anti-PM-Scl	AnuA	0.999998	0
B99	B108	anti-PM-Scl	Anti-Cyclin	-0.22672	0
B99	B109	anti-PM-Scl	AMA-M2	-0.26181	0
B99	B110	anti-PM-Scl	AHA	1	0
B99	B125	anti-PM-Scl	Cor	0.234516	0
B99	B154	anti-PM-Scl	RF	0.204755	0
B99	B183	anti-PM-Scl	PTA	0.273181	0
B99	B155	anti-PM-Scl	IgA	-0.16404	5.60E-14
B99	B156	anti-PM-Scl	IgE	-0.14475	3.52E-11
B99	B181	anti-PM-Scl	APTT	0.13878	2.19E-10
B99	B195	anti-PM-Scl	Na	0.137023	3.70E-10
B99	B103	anti-PM-Scl	anti-SSA/Ro	-0.13644	4.40E-10
B99	B137	anti-PM-Scl	AFU	-0.12881	3.93E-09
B99	B198	anti-PM-Scl	P	-0.12517	1.07E-08
B99	B139	anti-PM-Scl	ADA	0.118712	5.89E-08
B99	B138	anti-PM-Scl	PAB	0.113683	2.09E-07
B99	B185	anti-PM-Scl	PT	-0.10972	5.48E-07

Supplementary Table 6. PEI Correlations in Hypertensive+Diabetes+Coronary

Row	Column	Row	Column	Cor	*P*
B1	B161	Gender	LEU	0.311715	0
B1	B174	Gender	Urea/Cr	0.377869	0
B1	B216	Gender	BMI	-0.46849	0
B1	B221	Gender	Hei	-0.69222	0
B1	B222	Gender	NS	-0.55315	0
B1	B46	Gender	RBC Count	-0.32883	0
B1	B51	Gender	Hb	-0.39553	0
B1	B173	Gender	NTT	0.260691	6.66E-16
B1	B45	Gender	MCHC	-0.25945	8.88E-16
B1	B209	Gender	Smoking	-0.25534	2.66E-15
B1	B72	Gender	HDL-C	0.22396	5.16E-12
B1	B205	Gender	KDA	0.201732	5.61E-10
B1	B163	Gender	SG	-0.20051	7.16E-10
B1	B190	Gender	Cre	-0.18301	1.97E-08
B1	B210	Gender	Hip	-0.17261	1.22E-07
B1	B2	Gender	Age	0.172135	1.32E-07
B1	B204	Gender	Drinking	-0.16505	4.29E-07
B1	B42	Gender	MON	-0.1637	5.33E-07
B1	B50	Gender	eo%	-0.16063	8.71E-07
B10	B12	PG I	PG II	0.621901	0
B10	B144	PG I	Total-PINP	0.289629	0
B10	B145	PG I	BGP	0.281342	0
B10	B175	PG I	ALB-U	0.271446	0
B10	B191	PG I	Urea	0.27824	0
B10	B11	PG I	PGI/PGII	0.252651	5.55E-15
B10	B177	PG I	ACR	0.235389	3.78E-13
B10	B190	PG I	Cre	0.219859	1.27E-11
B10	B189	PG I	eGFB	-0.19314	3.00E-09
B100	B103	anti-Ro52	anti-SSA/Ro	1	0
B100	B104	anti-Ro52	anti-SSB	1	0
B100	B106	anti-Ro52	anti-P	1	0
B100	B107	anti-Ro52	AnuA	0.925318	0
B100	B109	anti-Ro52	AMA-M2	1	0
B100	B110	anti-Ro52	AHA	0.925318	0
B100	B119	anti-Ro52	Hcv-rna	0.433836	0
B100	B120	anti-Ro52	HCV-cAg	0.352785	0
B100	B121	anti-Ro52	C3	-0.43384	0
B100	B122	anti-Ro52	C4	-0.43384	0
B100	B124	anti-Ro52	ACTH	-0.37674	0
B100	B125	anti-Ro52	Cor	-0.37674	0
B100	B139	anti-Ro52	ADA	-0.45548	0

B100	B152	anti-Ro52	ACA-Ig(G-A-M)	-0.43381	0
B100	B157	anti-Ro52	IgG	-0.33455	0
B100	B159	anti-Ro52	β2-MG	-0.43384	0
B100	B169	anti-Ro52	U-CRY	-0.43383	0
B100	B180	anti-Ro52	Tf	0.440213	0
B100	B193	anti-Ro52	K	0.3357	0
B100	B194	anti-Ro52	Cl	0.354015	0
B100	B155	anti-Ro52	IgA	-0.24381	5.02E-14
B100	B137	anti-Ro52	AFU	-0.24337	5.60E-14
B100	B154	anti-Ro52	RF	0.242501	6.93E-14
B100	B195	anti-Ro52	Na	0.219271	1.45E-11
B100	B184	anti-Ro52	INR	-0.20636	2.21E-10
B100	B183	anti-Ro52	PTA	0.202624	4.70E-10
B100	B178	anti-Ro52	Ig-U	0.196769	1.49E-09
B100	B135	anti-Ro52	TBA	-0.18726	9.04E-09
B100	B196	anti-Ro52	TCO2	-0.17771	5.04E-08
B101	B102	anti-Sc70	anti-Sm	1	0
B101	B104	anti-Sc70	anti-SSB	1	0
B101	B105	anti-Sc70	anti-U1RNP	1	0
B101	B107	anti-Sc70	AnuA	0.925317	0
B101	B108	anti-Sc70	Anti-Cyclin	1	0
B101	B109	anti-Sc70	AMA-M2	1	0
B101	B110	anti-Sc70	AHA	0.925318	0
B101	B119	anti-Sc70	Hcv-rna	0.433836	0
B101	B120	anti-Sc70	HCV-cAg	0.352785	0
B101	B121	anti-Sc70	C3	-0.43384	0
B101	B122	anti-Sc70	C4	-0.43384	0
B101	B124	anti-Sc70	ACTH	-0.37674	0
B101	B125	anti-Sc70	Cor	-0.37674	0
B101	B139	anti-Sc70	ADA	-0.45548	0
B101	B152	anti-Sc70	ACA-Ig(G-A-M)	-0.43381	0
B101	B157	anti-Sc70	IgG	-0.33455	0
B101	B159	anti-Sc70	β2-MG	-0.43384	0
B101	B169	anti-Sc70	U-CRY	-0.43383	0
B101	B180	anti-Sc70	Tf	0.440213	0
B101	B193	anti-Sc70	K	0.3357	0
B101	B194	anti-Sc70	Cl	0.354015	0
B101	B155	anti-Sc70	IgA	-0.24381	5.02E-14
B101	B137	anti-Sc70	AFU	-0.24337	5.60E-14
B101	B154	anti-Sc70	RF	0.242501	6.93E-14
B101	B195	anti-Sc70	Na	0.219271	1.45E-11
B101	B184	anti-Sc70	INR	-0.20636	2.21E-10
B101	B183	anti-Sc70	PTA	0.202624	4.70E-10
B101	B178	anti-Sc70	Ig-U	0.196769	1.49E-09

B101	B135	anti-Sc70	TBA	-0.18726	9.04E-09
B101	B196	anti-Sc70	TCO2	-0.17771	5.04E-08
B102	B107	anti-Sm	AnuA	0.925318	0
B102	B110	anti-Sm	AHA	0.925318	0
B102	B119	anti-Sm	Hcv-rna	0.433836	0
B102	B120	anti-Sm	HCV-cAg	0.352785	0
B102	B121	anti-Sm	C3	-0.43384	0
B102	B122	anti-Sm	C4	-0.43384	0
B102	B124	anti-Sm	ACTH	-0.37674	0
B102	B125	anti-Sm	Cor	-0.37674	0
B102	B139	anti-Sm	ADA	-0.45548	0
B102	B152	anti-Sm	ACA-Ig(G-A-M)	-0.43381	0
B102	B157	anti-Sm	IgG	-0.33455	0
B102	B159	anti-Sm	β2-MG	-0.43384	0
B102	B169	anti-Sm	U-CRY	-0.43383	0
B102	B180	anti-Sm	Tf	0.440213	0
B102	B193	anti-Sm	K	0.3357	0
B102	B194	anti-Sm	Cl	0.354015	0
B102	B155	anti-Sm	IgA	-0.24381	5.02E-14
B102	B137	anti-Sm	AFU	-0.24337	5.60E-14
B102	B154	anti-Sm	RF	0.242501	6.93E-14
B102	B195	anti-Sm	Na	0.219271	1.45E-11
B102	B184	anti-Sm	INR	-0.20636	2.21E-10
B102	B183	anti-Sm	PTA	0.202624	4.70E-10
B102	B178	anti-Sm	Ig-U	0.196769	1.49E-09
B102	B135	anti-Sm	TBA	-0.18726	9.04E-09
B102	B196	anti-Sm	TCO2	-0.17771	5.04E-08
B103	B104	anti-SSA/Ro	anti-SSB	1	0
B103	B106	anti-SSA/Ro	anti-P	1	0
B103	B107	anti-SSA/Ro	AnuA	0.925318	0
B103	B109	anti-SSA/Ro	AMA-M2	1	0
B103	B110	anti-SSA/Ro	AHA	0.925318	0
B103	B119	anti-SSA/Ro	Hcv-rna	0.433836	0
B103	B120	anti-SSA/Ro	HCV-cAg	0.352785	0
B103	B121	anti-SSA/Ro	C3	-0.43384	0
B103	B122	anti-SSA/Ro	C4	-0.43384	0
B103	B124	anti-SSA/Ro	ACTH	-0.37674	0
B103	B125	anti-SSA/Ro	Cor	-0.37674	0
B103	B139	anti-SSA/Ro	ADA	-0.45548	0
B103	B152	anti-SSA/Ro	ACA-Ig(G-A-M)	-0.43381	0
B103	B157	anti-SSA/Ro	IgG	-0.33455	0
B103	B159	anti-SSA/Ro	β2-MG	-0.43384	0
B103	B169	anti-SSA/Ro	U-CRY	-0.43383	0
B103	B180	anti-SSA/Ro	Tf	0.440213	0

B103	B193	anti-SSA/Ro	K	0.3357	0
B103	B194	anti-SSA/Ro	Cl	0.354015	0
B103	B155	anti-SSA/Ro	IgA	-0.24381	5.02E-14
B103	B137	anti-SSA/Ro	AFU	-0.24337	5.60E-14
B103	B154	anti-SSA/Ro	RF	0.242501	6.93E-14
B103	B195	anti-SSA/Ro	Na	0.219271	1.45E-11
B103	B184	anti-SSA/Ro	INR	-0.20636	2.21E-10
B103	B183	anti-SSA/Ro	PTA	0.202624	4.70E-10
B103	B178	anti-SSA/Ro	Ig-U	0.196769	1.49E-09
B103	B135	anti-SSA/Ro	TBA	-0.18726	9.04E-09
B103	B196	anti-SSA/Ro	TCO2	-0.17771	5.04E-08
B104	B105	anti-SSB	anti-U1RNP	1	0
B104	B106	anti-SSB	anti-P	1	0
B104	B107	anti-SSB	AnuA	0.925318	0
B104	B108	anti-SSB	Anti-Cyclin	1	0
B104	B109	anti-SSB	AMA-M2	1	0
B104	B110	anti-SSB	AHA	0.925318	0
B104	B119	anti-SSB	Hcv-rna	0.433836	0
B104	B120	anti-SSB	HCV-cAg	0.352785	0
B104	B121	anti-SSB	C3	-0.43384	0
B104	B122	anti-SSB	C4	-0.43384	0
B104	B124	anti-SSB	ACTH	-0.37674	0
B104	B125	anti-SSB	Cor	-0.37674	0
B104	B139	anti-SSB	ADA	-0.45548	0
B104	B152	anti-SSB	ACA-Ig(G-A-M)	-0.43381	0
B104	B157	anti-SSB	IgG	-0.33455	0
B104	B159	anti-SSB	β2-MG	-0.43384	0
B104	B169	anti-SSB	U-CRY	-0.43383	0
B104	B180	anti-SSB	Tf	0.440213	0
B104	B193	anti-SSB	K	0.3357	0
B104	B194	anti-SSB	Cl	0.354015	0
B104	B155	anti-SSB	IgA	-0.24381	5.02E-14
B104	B137	anti-SSB	AFU	-0.24337	5.60E-14
B104	B154	anti-SSB	RF	0.242501	6.93E-14
B104	B195	anti-SSB	Na	0.219271	1.45E-11
B104	B184	anti-SSB	INR	-0.20636	2.21E-10
B104	B183	anti-SSB	PTA	0.202624	4.70E-10
B104	B178	anti-SSB	Ig-U	0.196769	1.49E-09
B104	B135	anti-SSB	TBA	-0.18726	9.04E-09
B104	B196	anti-SSB	TCO2	-0.17771	5.04E-08
B105	B106	anti-U1RNP	anti-P	1	0
B105	B107	anti-U1RNP	AnuA	0.925318	0
B105	B109	anti-U1RNP	AMA-M2	1	0
B105	B110	anti-U1RNP	AHA	0.925318	0

B105	B119	anti-U1RNP	Hcv-rna	0.433836	0
B105	B120	anti-U1RNP	HCV-cAg	0.352785	0
B105	B121	anti-U1RNP	C3	-0.43384	0
B105	B122	anti-U1RNP	C4	-0.43384	0
B105	B124	anti-U1RNP	ACTH	-0.37674	0
B105	B125	anti-U1RNP	Cor	-0.37674	0
B105	B139	anti-U1RNP	ADA	-0.45548	0
B105	B152	anti-U1RNP	ACA-Ig(G-A-M)	-0.43381	0
B105	B157	anti-U1RNP	IgG	-0.33455	0
B105	B159	anti-U1RNP	β2-MG	-0.43384	0
B105	B169	anti-U1RNP	U-CRY	-0.43383	0
B105	B180	anti-U1RNP	Tf	0.440213	0
B105	B193	anti-U1RNP	K	0.3357	0
B105	B194	anti-U1RNP	Cl	0.354015	0
B105	B155	anti-U1RNP	IgA	-0.24381	5.02E-14
B105	B137	anti-U1RNP	AFU	-0.24337	5.60E-14
B105	B154	anti-U1RNP	RF	0.242501	6.93E-14
B105	B195	anti-U1RNP	Na	0.219271	1.45E-11
B105	B184	anti-U1RNP	INR	-0.20636	2.21E-10
B105	B183	anti-U1RNP	PTA	0.202624	4.70E-10
B105	B178	anti-U1RNP	Ig-U	0.196769	1.49E-09
B105	B135	anti-U1RNP	TBA	-0.18726	9.04E-09
B105	B196	anti-U1RNP	TCO2	-0.17771	5.04E-08
B106	B107	anti-P	AnuA	0.925318	0
B106	B108	anti-P	Anti-Cyclin	1	0
B106	B109	anti-P	AMA-M2	1	0
B106	B110	anti-P	AHA	0.925318	0
B106	B119	anti-P	Hcv-rna	0.433836	0
B106	B120	anti-P	HCV-cAg	0.352785	0
B106	B121	anti-P	C3	-0.43384	0
B106	B122	anti-P	C4	-0.43384	0
B106	B124	anti-P	ACTH	-0.37674	0
B106	B125	anti-P	Cor	-0.37674	0
B106	B139	anti-P	ADA	-0.45548	0
B106	B152	anti-P	ACA-Ig(G-A-M)	-0.43381	0
B106	B157	anti-P	IgG	-0.33455	0
B106	B159	anti-P	β2-MG	-0.43384	0
B106	B169	anti-P	U-CRY	-0.43383	0
B106	B180	anti-P	Tf	0.440213	0
B106	B193	anti-P	K	0.3357	0
B106	B194	anti-P	Cl	0.354015	0
B106	B155	anti-P	IgA	-0.24381	5.02E-14
B106	B137	anti-P	AFU	-0.24337	5.60E-14
B106	B154	anti-P	RF	0.242501	6.93E-14

B106	B195	anti-P	Na	0.219271	1.45E-11
B106	B184	anti-P	INR	-0.20636	2.21E-10
B106	B183	anti-P	PTA	0.202624	4.70E-10
B106	B178	anti-P	Ig-U	0.196769	1.49E-09
B106	B135	anti-P	TBA	-0.18726	9.04E-09
B106	B196	anti-P	TCO2	-0.17771	5.04E-08
B107	B108	AnuA	Anti-Cyclin	0.925318	0
B107	B109	AnuA	AMA-M2	0.925318	0
B107	B110	AnuA	AHA	1	0
B107	B119	AnuA	Hcv-rna	0.469108	0
B107	B120	AnuA	HCV-cAg	0.382247	0
B107	B121	AnuA	C3	-0.46911	0
B107	B122	AnuA	C4	-0.46911	0
B107	B124	AnuA	ACTH	-0.40715	0
B107	B125	AnuA	Cor	-0.40715	0
B107	B139	AnuA	ADA	-0.41313	0
B107	B152	AnuA	ACA-Ig(G-A-M)	-0.46907	0
B107	B155	AnuA	IgA	-0.28136	0
B107	B159	AnuA	β2-MG	-0.46911	0
B107	B169	AnuA	U-CRY	-0.4691	0
B107	B180	AnuA	Tf	0.475926	0
B107	B194	AnuA	Cl	0.416382	0
B107	B195	AnuA	Na	0.287963	0
B107	B137	AnuA	AFU	-0.25172	7.11E-15
B107	B184	AnuA	INR	-0.22365	5.52E-12
B107	B183	AnuA	PTA	0.218414	1.74E-11
B107	B154	AnuA	RF	0.214323	4.20E-11
B107	B178	AnuA	Ig-U	0.212351	6.37E-11
B107	B193	AnuA	K	0.211512	7.59E-11
B107	B157	AnuA	IgG	-0.20289	4.45E-10
B107	B135	AnuA	TBA	-0.18054	3.06E-08
B107	B181	AnuA	APTT	-0.16572	3.84E-07
B108	B110	Anti-Cyclin	AHA	0.925318	0
B108	B119	Anti-Cyclin	Hcv-rna	0.433836	0
B108	B120	Anti-Cyclin	HCV-cAg	0.352785	0
B108	B121	Anti-Cyclin	C3	-0.43384	0
B108	B122	Anti-Cyclin	C4	-0.43384	0
B108	B124	Anti-Cyclin	ACTH	-0.37674	0
B108	B125	Anti-Cyclin	Cor	-0.37674	0
B108	B139	Anti-Cyclin	ADA	-0.45548	0
B108	B152	Anti-Cyclin	ACA-Ig(G-A-M)	-0.43381	0
B108	B157	Anti-Cyclin	IgG	-0.33455	0
B108	B159	Anti-Cyclin	β2-MG	-0.43384	0
B108	B169	Anti-Cyclin	U-CRY	-0.43383	0

B108	B180	Anti-Cyclin	Tf	0.440213	0
B108	B193	Anti-Cyclin	K	0.3357	0
B108	B194	Anti-Cyclin	Cl	0.354015	0
B108	B155	Anti-Cyclin	IgA	-0.24381	5.02E-14
B108	B137	Anti-Cyclin	AFU	-0.24337	5.60E-14
B108	B154	Anti-Cyclin	RF	0.242501	6.93E-14
B108	B195	Anti-Cyclin	Na	0.219271	1.45E-11
B108	B184	Anti-Cyclin	INR	-0.20636	2.21E-10
B108	B183	Anti-Cyclin	PTA	0.202624	4.70E-10
B108	B178	Anti-Cyclin	Ig-U	0.196769	1.49E-09
B108	B135	Anti-Cyclin	TBA	-0.18726	9.04E-09
B108	B196	Anti-Cyclin	TCO2	-0.17771	5.04E-08
B109	B110	AMA-M2	AHA	0.925318	0
B109	B119	AMA-M2	Hcv-rna	0.433836	0
B109	B120	AMA-M2	HCV-cAg	0.352785	0
B109	B121	AMA-M2	C3	-0.43384	0
B109	B122	AMA-M2	C4	-0.43384	0
B109	B124	AMA-M2	ACTH	-0.37674	0
B109	B125	AMA-M2	Cor	-0.37674	0
B109	B139	AMA-M2	ADA	-0.45548	0
B109	B152	AMA-M2	ACA-Ig(G-A-M)	-0.43381	0
B109	B157	AMA-M2	IgG	-0.33455	0
B109	B159	AMA-M2	β2-MG	-0.43384	0
B109	B169	AMA-M2	U-CRY	-0.43383	0
B109	B180	AMA-M2	Tf	0.440213	0
B109	B193	AMA-M2	K	0.3357	0
B109	B194	AMA-M2	Cl	0.354015	0
B109	B155	AMA-M2	IgA	-0.24381	5.02E-14
B109	B137	AMA-M2	AFU	-0.24337	5.60E-14
B109	B154	AMA-M2	RF	0.242501	6.93E-14
B109	B195	AMA-M2	Na	0.219271	1.45E-11
B109	B184	AMA-M2	INR	-0.20636	2.21E-10
B109	B183	AMA-M2	PTA	0.202624	4.70E-10
B109	B178	AMA-M2	Ig-U	0.196769	1.49E-09
B109	B135	AMA-M2	TBA	-0.18726	9.04E-09
B109	B196	AMA-M2	TCO2	-0.17771	5.04E-08
B11	B12	PGI/PGII	PG II	-0.40811	0
B11	B14	PGI/PGII	G-17	-0.25011	1.07E-14
B11	B132	PGI/PGII	Dbil	-0.17949	3.68E-08
B110	B119	AHA	Hcv-rna	0.469108	0
B110	B120	AHA	HCV-cAg	0.382248	0
B110	B121	AHA	C3	-0.46911	0
B110	B122	AHA	C4	-0.46911	0
B110	B124	AHA	ACTH	-0.40715	0

B110	B125	AHA	Cor	-0.40715	0
B110	B139	AHA	ADA	-0.41313	0
B110	B152	AHA	ACA-Ig(G-A-M)	-0.46908	0
B110	B155	AHA	IgA	-0.28136	0
B110	B159	AHA	β2-MG	-0.46911	0
B110	B169	AHA	U-CRY	-0.46911	0
B110	B180	AHA	Tf	0.475926	0
B110	B194	AHA	Cl	0.416382	0
B110	B195	AHA	Na	0.287963	0
B110	B137	AHA	AFU	-0.25172	7.11E-15
B110	B184	AHA	INR	-0.22365	5.52E-12
B110	B183	AHA	PTA	0.218414	1.74E-11
B110	B154	AHA	RF	0.214323	4.20E-11
B110	B178	AHA	Ig-U	0.212352	6.37E-11
B110	B193	AHA	K	0.211513	7.59E-11
B110	B157	AHA	IgG	-0.20289	4.45E-10
B110	B135	AHA	TBA	-0.18054	3.06E-08
B110	B181	AHA	APTT	-0.16572	3.84E-07
B111	B228	TSH	OS	0.170241	1.82E-07
B112	B114	FT4	TSH TT3 TT4(TT4)	0.49946	0
B112	B181	FT4	APTT	-0.1621	6.89E-07
B113	B115	FT3	TSH TT3 TT4(TT3)	0.413715	0
B113	B185	FT3	PT	-0.21094	8.57E-11
B113	B184	FT3	INR	-0.2066	2.10E-10
B113	B181	FT3	APTT	-0.19625	1.65E-09
B113	B176	FT3	Ucr	0.194952	2.12E-09
B113	B183	FT3	PTA	-0.18813	7.70E-09
B113	B177	FT3	ACR	-0.17385	9.84E-08
B114	B171	TSH TT3 TT4(TT4)	EC	-0.31416	0
B114	B124	TSH TT3 TT4(TT4)	ACTH	0.244607	4.13E-14
B114	B125	TSH TT3 TT4(TT4)	Cor	0.244607	4.13E-14
B114	B135	TSH TT3 TT4(TT4)	TBA	0.219373	1.42E-11
B114	B151	TSH TT3 TT4(TT4)	Anti-CCP	0.183933	1.66E-08
B114	B180	TSH TT3 TT4(TT4)	Tf	-0.1621	6.89E-07
B115	B134	TSH TT3 TT4(TT3)	CHE	0.199864	8.13E-10
B116	B117	TT3	TT4	-0.96562	0
B118	B126	CEA1	Alb	-0.19643	1.59E-09
B118	B140	CEA1	r-GT	0.172776	1.18E-07
B118	B132	CEA1	Dbil	0.170568	1.72E-07
B119	B120	Hcv-rna	HCV-cAg	0.545646	0
B119	B122	Hcv-rna	C4	-1	0
B119	B124	Hcv-rna	ACTH	-0.57673	0
B119	B125	Hcv-rna	Cor	-0.57673	0
B119	B139	Hcv-rna	ADA	-0.43647	0

B119	B151	Hcv-rna	Anti-CCP	-0.31503	0
B119	B152	Hcv-rna	ACA-Ig(G-A-M)	-0.99994	0
B119	B155	Hcv-rna	IgA	-0.34006	0
B119	B159	Hcv-rna	β2-MG	-1	0
B119	B169	Hcv-rna	U-CRY	-1	0
B119	B135	Hcv-rna	TBA	-0.25213	6.22E-15
B119	B184	Hcv-rna	INR	-0.25025	1.02E-14
B119	B193	Hcv-rna	K	0.249729	1.15E-14
B119	B138	Hcv-rna	PAB	0.239223	1.52E-13
B119	B157	Hcv-rna	IgG	-0.21452	4.03E-11
B119	B180	Hcv-rna	Tf	0.193367	2.87E-09
B119	B194	Hcv-rna	Cl	0.188594	7.06E-09
B119	B195	Hcv-rna	Na	0.183484	1.80E-08
B119	B136	Hcv-rna	5'NT	-0.18293	1.99E-08
B119	B185	Hcv-rna	PT	-0.16585	3.76E-07
B12	B144	PG II	Total-PINP	0.352405	0
B12	B145	PG II	BGP	0.381873	0
B12	B175	PG II	ALB-U	0.36334	0
B12	B177	PG II	ACR	0.324465	0
B12	B190	PG II	Cre	0.306566	0
B12	B191	PG II	Urea	0.404415	0
B12	B80	PG II	anti-HP IgG	0.20969	1.11E-10
B12	B189	PG II	eGFB	-0.20533	2.72E-10
B12	B17	PG II	CD3+CD16+CD56+NK	-0.18178	2.45E-08
B12	B143	PG II	β-CTx	0.172767	1.19E-07
B120	B121	HCV-cAg	C3	-0.54565	0
B120	B122	HCV-cAg	C4	-0.54565	0
B120	B124	HCV-cAg	ACTH	-0.31469	0
B120	B125	HCV-cAg	Cor	-0.31469	0
B120	B152	HCV-cAg	ACA-Ig(G-A-M)	-0.54562	0
B120	B155	HCV-cAg	IgA	-0.33734	0
B120	B159	HCV-cAg	β2-MG	-0.54565	0
B120	B169	HCV-cAg	U-CRY	-0.54565	0
B120	B139	HCV-cAg	ADA	-0.21722	2.26E-11
B120	B135	HCV-cAg	TBA	-0.21442	4.12E-11
B120	B156	HCV-cAg	IgE	0.203616	3.85E-10
B120	B157	HCV-cAg	IgG	-0.1912	4.33E-09
B120	B193	HCV-cAg	K	0.187032	9.43E-09
B120	B180	HCV-cAg	Tf	0.178888	4.10E-08
B120	B151	HCV-cAg	Anti-CCP	-0.17864	4.29E-08
B121	B122	C3	C4	1	0
B121	B124	C3	ACTH	0.576727	0
B121	B125	C3	Cor	0.576727	0
B121	B139	C3	ADA	0.436469	0

B121	B151	C3	Anti-CCP	0.315028	0
B121	B152	C3	ACA-Ig(G-A-M)	0.99995	0
B121	B155	C3	IgA	0.340064	0
B121	B159	C3	β2-MG	1	0
B121	B169	C3	U-CRY	0.999998	0
B121	B135	C3	TBA	0.252131	6.22E-15
B121	B184	C3	INR	0.250246	1.02E-14
B121	B193	C3	K	-0.24973	1.15E-14
B121	B138	C3	PAB	-0.23922	1.52E-13
B121	B157	C3	IgG	0.214518	4.03E-11
B121	B180	C3	Tf	-0.19337	2.87E-09
B121	B194	C3	Cl	-0.18859	7.06E-09
B121	B195	C3	Na	-0.18348	1.80E-08
B121	B136	C3	5'NT	0.182935	1.99E-08
B121	B185	C3	PT	0.165846	3.76E-07
B122	B124	C4	ACTH	0.576727	0
B122	B125	C4	Cor	0.576727	0
B122	B139	C4	ADA	0.436468	0
B122	B151	C4	Anti-CCP	0.315028	0
B122	B152	C4	ACA-Ig(G-A-M)	0.999949	0
B122	B155	C4	IgA	0.340064	0
B122	B135	C4	TBA	0.252131	6.22E-15
B122	B184	C4	INR	0.250245	1.02E-14
B122	B193	C4	K	-0.24973	1.15E-14
B122	B138	C4	PAB	-0.23922	1.52E-13
B122	B157	C4	IgG	0.214518	4.03E-11
B122	B180	C4	Tf	-0.19337	2.87E-09
B122	B194	C4	Cl	-0.18859	7.06E-09
B122	B195	C4	Na	-0.18348	1.80E-08
B122	B136	C4	5'NT	0.182934	1.99E-08
B122	B185	C4	PT	0.165846	3.76E-07
B123	B172	hs-CRP1	KB	0.234466	4.69E-13
B124	B135	ACTH	TBA	0.59701	0
B124	B136	ACTH	5'NT	0.280012	0
B124	B138	ACTH	PAB	-0.37484	0
B124	B139	ACTH	ADA	0.572505	0
B124	B151	ACTH	Anti-CCP	0.540198	0
B124	B152	ACTH	ACA-Ig(G-A-M)	0.576694	0
B124	B157	ACTH	IgG	0.346398	0
B124	B158	ACTH	IgM	0.326704	0
B124	B159	ACTH	β2-MG	0.576727	0
B124	B169	ACTH	U-CRY	0.576724	0
B124	B180	ACTH	Tf	-0.33798	0
B124	B184	ACTH	INR	0.437275	0

B124	B185	ACTH	PT	0.305403	0
B124	B193	ACTH	K	-0.32623	0
B124	B194	ACTH	Cl	-0.44099	0
B124	B197	ACTH	AG	0.277445	0
B124	B199	ACTH	Mg	0.27051	0
B124	B183	ACTH	PTA	-0.26493	2.22E-16
B124	B155	ACTH	IgA	0.238695	1.73E-13
B124	B195	ACTH	Na	-0.18283	2.03E-08
B124	B198	ACTH	P	0.163147	5.83E-07
B125	B135	Cor	TBA	0.59701	0
B125	B136	Cor	5'NT	0.280012	0
B125	B138	Cor	PAB	-0.37484	0
B125	B139	Cor	ADA	0.572505	0
B125	B151	Cor	Anti-CCP	0.540198	0
B125	B152	Cor	ACA-Ig(G-A-M)	0.576694	0
B125	B157	Cor	IgG	0.346398	0
B125	B158	Cor	IgM	0.326704	0
B125	B159	Cor	β2-MG	0.576727	0
B125	B169	Cor	U-CRY	0.576724	0
B125	B180	Cor	Tf	-0.33798	0
B125	B184	Cor	INR	0.437275	0
B125	B185	Cor	PT	0.305403	0
B125	B193	Cor	K	-0.32623	0
B125	B194	Cor	Cl	-0.44099	0
B125	B197	Cor	AG	0.277445	0
B125	B199	Cor	Mg	0.27051	0
B125	B183	Cor	PTA	-0.26493	2.22E-16
B125	B155	Cor	IgA	0.238695	1.73E-13
B125	B195	Cor	Na	-0.18283	2.03E-08
B125	B198	Cor	P	0.163147	5.83E-07
B126	B127	Alb	A-G ratio	0.537404	0
B126	B188	Alb	Ca	0.345114	0
B126	B189	Alb	eGFB	0.308957	0
B126	B191	Alb	Urea	-0.26648	0
B126	B190	Alb	Cre	-0.23542	3.75E-13
B126	B130	Alb	GLB	-0.19366	2.72E-09
B127	B130	A-G ratio	GLB	-0.901	0
B127	B189	A-G ratio	eGFB	0.231861	8.59E-13
B127	B129	A-G ratio	IBIL	0.168286	2.52E-07
B127	B220	A-G ratio	SBP	-0.16383	5.22E-07
B128	B131	ALT	AST	0.740293	0
B128	B132	ALT	Dbil	0.349139	0
B128	B140	ALT	r-GT	0.434533	0
B128	B141	ALT	AST/ALT	-0.54309	0

B128	B133	ALT	TBIL	0.255834	2.44E-15
B128	B216	ALT	BMI	0.195355	1.96E-09
B128	B136	ALT	5'NT	0.166803	3.22E-07
B129	B132	IBIL	Dbil	0.325892	0
B129	B133	IBIL	TBIL	0.823504	0
B129	B130	IBIL	GLB	-0.16241	6.56E-07
B130	B153	GLB	Glu	0.166722	3.26E-07
B131	B132	AST	Dbil	0.462077	0
B131	B133	AST	TBIL	0.349927	0
B131	B140	AST	r-GT	0.527614	0
B132	B133	Dbil	TBIL	0.80468	0
B132	B140	Dbil	r-GT	0.885761	0
B133	B140	TBIL	r-GT	0.596874	0
B134	B138	CHE	PAB	0.561042	0
B134	B139	CHE	ADA	-0.23891	1.64E-13
B134	B157	CHE	IgG	-0.23567	3.54E-13
B134	B186	CHE	PFC	-0.23307	6.50E-13
B134	B171	CHE	EC	-0.21139	7.79E-11
B134	B150	CHE	ALP	-0.20191	5.42E-10
B134	B136	CHE	5'NT	-0.17349	1.05E-07
B134	B135	CHE	TBA	-0.17247	1.25E-07
B134	B182	CHE	TT	0.165503	3.98E-07
B135	B138	TBA	PAB	-0.29783	0
B135	B139	TBA	ADA	0.403014	0
B135	B151	TBA	Anti-CCP	0.347199	0
B135	B157	TBA	IgG	0.281903	0
B135	B193	TBA	K	-0.38757	0
B135	B194	TBA	Cl	-0.26644	0
B135	B197	TBA	AG	0.287397	0
B135	B198	TBA	P	0.453291	0
B135	B152	TBA	ACA-Ig(G-A-M)	0.252117	6.22E-15
B135	B159	TBA	β2-MG	0.252131	6.22E-15
B135	B169	TBA	U-CRY	0.25213	6.22E-15
B135	B184	TBA	INR	0.249488	1.24E-14
B135	B200	TBA	OP	0.224905	4.18E-12
B135	B180	TBA	Tf	-0.19797	1.18E-09
B135	B185	TBA	PT	0.187019	9.46E-09
B135	B137	TBA	AFU	-0.16133	7.79E-07
B136	B137	5'NT	AFU	0.286331	0
B136	B150	5'NT	ALP	0.291357	0
B136	B193	5'NT	K	0.386247	0
B136	B196	5'NT	TCO2	-0.33329	0
B136	B197	5'NT	AG	0.289579	0
B136	B199	5'NT	Mg	0.41058	0

B136	B159	5'NT	β2-MG	0.182935	1.99E-08
B136	B169	5'NT	U-CRY	0.182934	1.99E-08
B136	B152	5'NT	ACA-Ig(G-A-M)	0.182924	2.00E-08
B136	B151	5'NT	Anti-CCP	0.174557	8.72E-08
B136	B158	5'NT	IgM	0.17024	1.82E-07
B136	B186	5'NT	PFC	-0.17017	1.84E-07
B137	B139	AFU	ADA	0.420277	0
B137	B193	AFU	K	0.484625	0
B137	B194	AFU	Cl	-0.41315	0
B137	B195	AFU	Na	-0.5994	0
B137	B200	AFU	OP	-0.28686	0
B137	B150	AFU	ALP	0.264603	2.22E-16
B137	B138	AFU	PAB	-0.20337	4.04E-10
B137	B180	AFU	Tf	-0.1701	1.86E-07
B138	B139	PAB	ADA	-0.55876	0
B138	B157	PAB	IgG	-0.26832	0
B138	B151	PAB	Anti-CCP	-0.25944	8.88E-16
B138	B194	PAB	Cl	0.257126	1.78E-15
B138	B159	PAB	β2-MG	-0.23922	1.52E-13
B138	B169	PAB	U-CRY	-0.23922	1.52E-13
B138	B152	PAB	ACA-Ig(G-A-M)	-0.23921	1.53E-13
B138	B195	PAB	Na	0.230646	1.14E-12
B138	B184	PAB	INR	-0.22658	2.87E-12
B138	B185	PAB	PT	-0.20314	4.23E-10
B138	B182	PAB	TT	0.197383	1.32E-09
B138	B155	PAB	IgA	-0.16648	3.39E-07
B139	B151	ADA	Anti-CCP	0.30815	0
B139	B152	ADA	ACA-Ig(G-A-M)	0.436444	0
B139	B155	ADA	IgA	0.269165	0
B139	B157	ADA	IgG	0.469032	0
B139	B159	ADA	β2-MG	0.436469	0
B139	B169	ADA	U-CRY	0.436467	0
B139	B180	ADA	Tf	-0.2679	0
B139	B184	ADA	INR	0.305602	0
B139	B193	ADA	K	-0.27232	0
B139	B194	ADA	Cl	-0.52179	0
B139	B195	ADA	Na	-0.38191	0
B139	B198	ADA	P	0.238974	1.62E-13
B139	B185	ADA	PT	0.22976	1.39E-12
B139	B154	ADA	RF	-0.21187	7.04E-11
B139	B196	ADA	TCO2	0.193911	2.59E-09
B139	B146	ADA	CysC	0.167969	2.65E-07
B14	B114	G-17	TSH TT3 TT4(TT4)	0.177105	5.61E-08
B141	B216	AST/ALT	BMI	-0.1861	1.12E-08

B141	B222	AST/ALT	NS	-0.1616	7.47E-07
B142	B143	25-(OH)D	β-CTx	-0.33868	0
B142	B144	25-(OH)D	Total-PINP	-0.31317	0
B142	B151	25-(OH)D	Anti-CCP	-0.42731	0
B142	B168	25-(OH)D	NMP22	-0.25766	1.55E-15
B142	B145	25-(OH)D	BGP	-0.20523	2.78E-10
B142	B175	25-(OH)D	ALB-U	-0.16265	6.31E-07
B143	B144	β-CTx	Total-PINP	0.799358	0
B143	B145	β-CTx	BGP	0.772205	0
B143	B151	β-CTx	Anti-CCP	0.278567	0
B143	B175	β-CTx	ALB-U	0.267389	0
B143	B191	β-CTx	Urea	0.212662	5.96E-11
B143	B177	β-CTx	ACR	0.19387	2.61E-09
B143	B190	β-CTx	Cre	0.177596	5.15E-08
B144	B145	Total-PINP	BGP	0.862777	0
B144	B175	Total-PINP	ALB-U	0.529697	0
B144	B177	Total-PINP	ACR	0.444951	0
B144	B190	Total-PINP	Cre	0.304735	0
B144	B191	Total-PINP	Urea	0.337366	0
B144	B151	Total-PINP	Anti-CCP	0.243696	5.17E-14
B145	B175	BGP	ALB-U	0.622157	0
B145	B177	BGP	ACR	0.545746	0
B145	B190	BGP	Cre	0.344116	0
B145	B191	BGP	Urea	0.364225	0
B146	B214	CysC	CVA-L	0.237184	2.47E-13
B146	B173	CysC	NTT	0.219377	1.41E-11
B146	B199	CysC	Mg	-0.16989	1.93E-07
B148	B149	Anti_TPO	TGAb	0.204687	3.10E-10
B15	B100	CD3-CD19+(B cell)	anti-Ro52	0.443462	0
B15	B101	CD3-CD19+(B cell)	anti-Sc70	0.443462	0
B15	B102	CD3-CD19+(B cell)	anti-Sm	0.443462	0
B15	B103	CD3-CD19+(B cell)	anti-SSA/Ro	0.443462	0
B15	B104	CD3-CD19+(B cell)	anti-SSB	0.443462	0
B15	B105	CD3-CD19+(B cell)	anti-U1RNP	0.443462	0
B15	B106	CD3-CD19+(B cell)	anti-P	0.443462	0
B15	B107	CD3-CD19+(B cell)	AnuA	0.299599	0
B15	B108	CD3-CD19+(B cell)	Anti-Cyclin	0.443462	0
B15	B109	CD3-CD19+(B cell)	AMA-M2	0.443462	0
B15	B110	CD3-CD19+(B cell)	AHA	0.299599	0
B15	B139	CD3-CD19+(B cell)	ADA	-0.48431	0
B15	B157	CD3-CD19+(B cell)	IgG	-0.41878	0
B15	B17	CD3-CD19+(B cell)	CD3+CD16+CD56+NK	0.508556	0
B15	B18	CD3-CD19+(B cell)	CD3+CD4+helper(inducer) T	-0.36469	0

B15	B19	CD3-CD19+(B cell)	CD3+CD4+/CD3+CD8+	-0.59228	0
B15	B193	CD3-CD19+(B cell)	K	0.307483	0
B15	B20	CD3-CD19+(B cell)	CD3+CD4+CD8+	-0.61919	0
B15	B21	CD3-CD19+(B cell)	CD3+CD4-CD8-	0.602925	0
B15	B22	CD3-CD19+(B cell)	CD3+CD8+(T)	0.602957	0
B15	B23	CD3-CD19+(B cell)	CD3-CD16+CD56+(NK)	-0.40702	0
B15	B95	CD3-CD19+(B cell)	anti-cenpb	-0.42794	0
B15	B97	CD3-CD19+(B cell)	dsDNA	0.443462	0
B15	B98	CD3-CD19+(B cell)	anti-Jo-1	0.443462	0
B15	B99	CD3-CD19+(B cell)	anti-PM-Scl	0.443462	0
B15	B135	CD3-CD19+(B cell)	TBA	-0.264	4.44E-16
B15	B16	CD3-CD19+(B cell)	CD3+T	0.256072	2.22E-15
B15	B124	CD3-CD19+(B cell)	ACTH	-0.23421	4.98E-13
B15	B125	CD3-CD19+(B cell)	Cor	-0.23421	4.98E-13
B15	B180	CD3-CD19+(B cell)	Tf	0.21566	3.16E-11
B15	B138	CD3-CD19+(B cell)	PAB	0.188963	6.59E-09
B15	B74	CD3-CD19+(B cell)	ApoA1	0.173054	1.13E-07
B15	B92	CD3-CD19+(B cell)	ALDH2-Glu504Glu	-0.16403	5.05E-07
B15	B93	CD3-CD19+(B cell)	ALDH2-Glu504Lys	0.164034	5.05E-07
B15	B94	CD3-CD19+(B cell)	ALDH2-Lys504Lys	0.164034	5.05E-07
B15	B154	CD3-CD19+(B cell)	RF	0.159765	9.99E-07
B150	B195	ALP	Na	-0.26468	2.22E-16
B150	B193	ALP	K	0.218778	1.61E-11
B150	B198	ALP	P	-0.20699	1.94E-10
B150	B200	ALP	OP	-0.20336	4.05E-10
B150	B181	ALP	APTT	-0.19764	1.26E-09
B151	B152	Anti-CCP	ACA-Ig(G-A-M)	0.31501	0
B151	B159	Anti-CCP	β2-MG	0.315028	0
B151	B168	Anti-CCP	NMP22	0.426315	0
B151	B169	Anti-CCP	U-CRY	0.315027	0
B151	B194	Anti-CCP	Cl	-0.22356	5.63E-12
B151	B184	Anti-CCP	INR	0.209939	1.05E-10
B151	B158	Anti-CCP	IgM	0.195062	2.07E-09
B151	B180	Anti-CCP	Tf	-0.18634	1.07E-08
B151	B157	Anti-CCP	IgG	0.173131	1.11E-07
B151	B154	Anti-CCP	RF	-0.17032	1.79E-07
B151	B185	Anti-CCP	PT	0.163092	5.88E-07
B152	B155	ACA-Ig(G-A-M)	IgA	0.340045	0
B152	B159	ACA-Ig(G-A-M)	β2-MG	0.999955	0
B152	B169	ACA-Ig(G-A-M)	U-CRY	0.999985	0
B152	B184	ACA-Ig(G-A-M)	INR	0.250231	1.02E-14
B152	B193	ACA-Ig(G-A-M)	K	-0.24972	1.15E-14
B152	B157	ACA-Ig(G-A-M)	IgG	0.214506	4.04E-11
B152	B180	ACA-Ig(G-A-M)	Tf	-0.19336	2.88E-09

B152	B194	ACA-Ig(G-A-M)	Cl	-0.18858	7.08E-09
B152	B195	ACA-Ig(G-A-M)	Na	-0.18347	1.81E-08
B152	B185	ACA-Ig(G-A-M)	PT	0.165837	3.77E-07
B153	B170	Glu	U-GLU	0.520439	0
B153	B163	Glu	SG	0.18395	1.66E-08
B153	B165	Glu	bilirubin	-0.18369	1.74E-08
B154	B194	RF	Cl	0.17494	8.17E-08
B155	B157	IgA	IgG	0.377399	0
B155	B158	IgA	IgM	-0.35052	0
B155	B159	IgA	β2-MG	0.340064	0
B155	B169	IgA	U-CRY	0.340062	0
B155	B193	IgA	K	-0.1968	1.48E-09
B155	B194	IgA	Cl	-0.18272	2.07E-08
B156	B196	IgE	TCO2	-0.22162	8.65E-12
B157	B193	IgG	K	-0.2992	0
B157	B159	IgG	β2-MG	0.214518	4.03E-11
B157	B169	IgG	U-CRY	0.214517	4.03E-11
B157	B184	IgG	INR	0.178413	4.46E-08
B158	B199	IgM	Mg	0.184385	1.53E-08
B158	B186	IgM	PFC	-0.1662	3.55E-07
B159	B184	β2-MG	INR	0.250246	1.02E-14
B159	B193	β2-MG	K	-0.24973	1.15E-14
B159	B180	β2-MG	Tf	-0.19337	2.87E-09
B159	B194	β2-MG	Cl	-0.18859	7.06E-09
B159	B195	β2-MG	Na	-0.18348	1.80E-08
B159	B185	β2-MG	PT	0.165846	3.76E-07
B16	B100	CD3+T	anti-Ro52	0.385844	0
B16	B101	CD3+T	anti-Sc70	0.385844	0
B16	B102	CD3+T	anti-Sm	0.385844	0
B16	B103	CD3+T	anti-SSA/Ro	0.385844	0
B16	B104	CD3+T	anti-SSB	0.385844	0
B16	B105	CD3+T	anti-U1RNP	0.385844	0
B16	B106	CD3+T	anti-P	0.385844	0
B16	B107	CD3+T	AnuA	0.469506	0
B16	B108	CD3+T	Anti-Cyclin	0.385844	0
B16	B109	CD3+T	AMA-M2	0.385844	0
B16	B110	CD3+T	AHA	0.469506	0
B16	B119	CD3+T	Hcv-rna	0.617272	0
B16	B120	CD3+T	HCV-cAg	0.336041	0
B16	B121	CD3+T	C3	-0.61727	0
B16	B122	CD3+T	C4	-0.61727	0
B16	B124	CD3+T	ACTH	-0.89126	0
B16	B125	CD3+T	Cor	-0.89126	0
B16	B135	CD3+T	TBA	-0.53188	0

B16	B138	CD3+T	PAB	0.294026	0
B16	B139	CD3+T	ADA	-0.66852	0
B16	B151	CD3+T	Anti-CCP	-0.42758	0
B16	B152	CD3+T	ACA-Ig(G-A-M)	-0.61724	0
B16	B155	CD3+T	IgA	-0.30085	0
B16	B157	CD3+T	IgG	-0.39327	0
B16	B159	CD3+T	β2-MG	-0.61727	0
B16	B169	CD3+T	U-CRY	-0.61727	0
B16	B18	CD3+T	CD3+CD4+helper(inducer) T	0.664175	0
B16	B183	CD3+T	PTA	0.293286	0
B16	B184	CD3+T	INR	-0.41453	0
B16	B185	CD3+T	PT	-0.27597	0
B16	B19	CD3+T	CD3+CD4+/CD3+CD8+	-0.38672	0
B16	B193	CD3+T	K	0.31108	0
B16	B194	CD3+T	Cl	0.418845	0
B16	B22	CD3+T	CD3+CD8+(T)	0.74405	0
B16	B23	CD3+T	CD3-CD16+CD56+(NK)	-0.96923	0
B16	B24	CD3+T	αHBDH	-0.27985	0
B16	B63	CD3+T	WBRSR(mPas_200)	-0.28509	0
B16	B64	CD3+T	WBRSR(mPas_5)	-0.2955	0
B16	B66	CD3+T	WBRSR(mPas_100)	-0.32463	0
B16	B67	CD3+T	WBRSR(mPas_50)	-0.32731	0
B16	B79	CD3+T	hb V -dna	0.444097	0
B16	B92	CD3+T	ALDH2-Glu504Glu	-0.62949	0
B16	B93	CD3+T	ALDH2-Glu504Lys	0.629494	0
B16	B94	CD3+T	ALDH2-Lys504Lys	0.629494	0
B16	B97	CD3+T	dsDNA	0.385844	0
B16	B98	CD3+T	anti-Jo-1	0.385844	0
B16	B99	CD3+T	anti-PM-Scl	0.385844	0
B16	B180	CD3+T	Tf	0.264551	2.22E-16
B16	B197	CD3+T	AG	-0.26186	4.44E-16
B16	B61	CD3+T	WBHSRV	-0.26192	4.44E-16
B16	B65	CD3+T	WBRSR(mPas_1)	-0.25243	5.77E-15
B16	B199	CD3+T	Mg	-0.24198	7.84E-14
B16	B195	CD3+T	Na	0.24074	1.06E-13
B16	B62	CD3+T	WBHSRVI	-0.21168	7.34E-11
B16	B59	CD3+T	WBRV	-0.20924	1.22E-10
B16	B198	CD3+T	P	-0.19583	1.79E-09
B16	B158	CD3+T	IgM	-0.19347	2.82E-09
B16	B54	CD3+T	ESR	-0.19187	3.82E-09
B16	B136	CD3+T	5'NT	-0.17772	5.04E-08
B16	B60	CD3+T	WBRVI	-0.17476	8.42E-08
B16	B56	CD3+T	IR	-0.17125	1.53E-07

B16	B182	CD3+T	TT	-0.16038	9.06E-07
B160	B163	PH	SG	-0.16868	2.36E-07
B161	B173	LEU	NTT	0.312406	0
B161	B221	LEU	Hei	-0.21485	3.75E-11
B163	B166	SG	Pro	0.289655	0
B163	B176	SG	Ucr	0.287433	0
B163	B216	SG	BMI	0.292263	0
B163	B222	SG	NS	0.26524	2.22E-16
B163	B170	SG	U-GLU	0.245423	3.38E-14
B163	B221	SG	Hei	0.218254	1.81E-11
B163	B210	SG	Hip	0.213886	4.61E-11
B163	B206	SG	Waist	0.196693	1.51E-09
B163	B215	SG	BMI	0.196569	1.55E-09
B163	B218	SG	DBP	0.162605	6.36E-07
B163	B172	SG	KB	0.160254	9.25E-07
B163	B189	SG	eGFB	0.160005	9.62E-07
B166	B170	Pro	U-GLU	0.19956	8.63E-10
B169	B184	U-CRY	INR	0.250244	1.02E-14
B169	B193	U-CRY	K	-0.24973	1.15E-14
B169	B180	U-CRY	Tf	-0.19337	2.87E-09
B169	B194	U-CRY	Cl	-0.18859	7.06E-09
B169	B195	U-CRY	Na	-0.18348	1.80E-08
B169	B185	U-CRY	PT	0.165845	3.76E-07
B17	B100	CD3+CD16+CD56+NK	anti-Ro52	0.506404	0
B17	B101	CD3+CD16+CD56+NK	anti-Sc70	0.506404	0
B17	B102	CD3+CD16+CD56+NK	anti-Sm	0.506404	0
B17	B103	CD3+CD16+CD56+NK	anti-SSA/Ro	0.506404	0
B17	B104	CD3+CD16+CD56+NK	anti-SSB	0.506404	0
B17	B105	CD3+CD16+CD56+NK	anti-U1RNP	0.506404	0
B17	B106	CD3+CD16+CD56+NK	anti-P	0.506404	0
B17	B107	CD3+CD16+CD56+NK	AnuA	0.547413	0
B17	B108	CD3+CD16+CD56+NK	Anti-Cyclin	0.506404	0
B17	B109	CD3+CD16+CD56+NK	AMA-M2	0.506404	0
B17	B110	CD3+CD16+CD56+NK	AHA	0.547413	0
B17	B18	CD3+CD16+CD56+NK	CD3+CD4+helper(inducer) T	-0.38716	0
B17	B181	CD3+CD16+CD56+NK	APTT	-0.27186	0
B17	B20	CD3+CD16+CD56+NK	CD3+CD4+CD8+	-0.34185	0
B17	B21	CD3+CD16+CD56+NK	CD3+CD4-CD8-	0.977995	0
B17	B22	CD3+CD16+CD56+NK	CD3+CD8+(T)	0.269626	0
B17	B34	CD3+CD16+CD56+NK	TK	0.27812	0
B17	B97	CD3+CD16+CD56+NK	dsDNA	0.506404	0
B17	B98	CD3+CD16+CD56+NK	anti-Jo-1	0.506404	0
B17	B99	CD3+CD16+CD56+NK	anti-PM-Scl	0.506404	0

B17	B19	CD3+CD16+CD56+NK	CD3+CD4+/CD3+CD8+	-0.24112	9.68E-14
B17	B156	CD3+CD16+CD56+NK	IgE	-0.20062	7.00E-10
B17	B180	CD3+CD16+CD56+NK	Tf	0.182719	2.07E-08
B17	B183	CD3+CD16+CD56+NK	PTA	0.179299	3.81E-08
B17	B196	CD3+CD16+CD56+NK	TCO2	0.173565	1.03E-07
B17	B73	CD3+CD16+CD56+NK	ApoA	-0.17159	1.45E-07
B17	B151	CD3+CD16+CD56+NK	Anti-CCP	0.170202	1.83E-07
B17	B23	CD3+CD16+CD56+NK	CD3-CD16+CD56+(NK)	-0.16127	7.87E-07
B172	B200	KB	OP	0.252669	5.55E-15
B173	B221	NTT	Hei	-0.17758	5.16E-08
B174	B190	Urea/Cr	Cre	-0.30404	0
B174	B191	Urea/Cr	Urea	0.304336	0
B174	B221	Urea/Cr	Hei	-0.26461	2.22E-16
B174	B222	Urea/Cr	NS	-0.18251	2.15E-08
B174	B189	Urea/Cr	eGFB	0.1653	4.11E-07
B175	B177	ALB-U	ACR	0.929329	0
B175	B190	ALB-U	Cre	0.359039	0
B175	B191	ALB-U	Urea	0.389708	0
B176	B204	Ucr	Drinking	-0.21962	1.34E-11
B176	B177	Ucr	ACR	-0.18957	5.89E-09
B176	B178	Ucr	Ig-U	0.182815	2.04E-08
B176	B179	Ucr	α1-MG	0.175852	6.97E-08
B176	B209	Ucr	Smoking	-0.17462	8.63E-08
B177	B190	ACR	Cre	0.328991	0
B177	B191	ACR	Urea	0.349128	0
B178	B179	Ig-U	α1-MG	0.85617	0
B178	B180	Ig-U	Tf	0.786737	0
B179	B180	α1-MG	Tf	0.425214	0
B18	B119	CD3+CD4+helper(inducer)T	Hcv-rna	0.27889	0
B18	B121	CD3+CD4+helper(inducer)T	C3	-0.27889	0
B18	B122	CD3+CD4+helper(inducer)T	C4	-0.27889	0
B18	B124	CD3+CD4+helper(inducer)T	ACTH	-0.46313	0
B18	B125	CD3+CD4+helper(inducer)T	Cor	-0.46313	0
B18	B139	CD3+CD4+helper(inducer)T	ADA	-0.30785	0
B18	B152	CD3+CD4+helper(inducer)T	ACA-Ig(G-A-M)	-0.27887	0
B18	B159	CD3+CD4+helper(inducer)T	β2-MG	-0.27889	0
B18	B169	CD3+CD4+helper(inducer)T	U-CRY	-0.27889	0
B18	B19	CD3+CD4+helper(inducer)T	CD3+CD4+/CD3+CD8+	0.352986	0
B18	B194	CD3+CD4+helper(inducer)T	Cl	0.271142	0
B18	B20	CD3+CD4+helper(inducer)T	CD3+CD4+CD8+	0.356158	0
B18	B21	CD3+CD4+helper(inducer)T	CD3+CD4-CD8-	-0.46846	0
B18	B23	CD3+CD4+helper(inducer)T	CD3-CD16+CD56+(NK)	-0.57493	0
B18	B92	CD3+CD4+helper(inducer)T	ALDH2-Glu504Glu	-0.32797	0
B18	B93	CD3+CD4+helper(inducer)T	ALDH2-Glu504Lys	0.32797	0

B18	B94	CD3+CD4+helper(inducer)T	ALDH2-Lys504Lys	0.32797	0
B18	B95	CD3+CD4+helper(inducer)T	anti-cenpb	0.547557	0
B18	B199	CD3+CD4+helper(inducer)T	Mg	-0.26511	2.22E-16
B18	B135	CD3+CD4+helper(inducer)T	TBA	-0.25813	1.33E-15
B18	B79	CD3+CD4+helper(inducer)T	hbV-dna	0.232597	7.25E-13
B18	B151	CD3+CD4+helper(inducer)T	Anti-CCP	-0.23224	7.87E-13
B18	B184	CD3+CD4+helper(inducer)T	INR	-0.21107	8.34E-11
B18	B198	CD3+CD4+helper(inducer)T	P	-0.2061	2.32E-10
B18	B100	CD3+CD4+helper(inducer)T	anti-Ro52	-0.1955	1.91E-09
B18	B101	CD3+CD4+helper(inducer)T	anti-Sc70	-0.1955	1.91E-09
B18	B102	CD3+CD4+helper(inducer)T	anti-Sm	-0.1955	1.91E-09
B18	B103	CD3+CD4+helper(inducer)T	anti-SSA/Ro	-0.1955	1.91E-09
B18	B104	CD3+CD4+helper(inducer)T	anti-SSB	-0.1955	1.91E-09
B18	B105	CD3+CD4+helper(inducer)T	anti-U1RNP	-0.1955	1.91E-09
B18	B106	CD3+CD4+helper(inducer)T	anti-P	-0.1955	1.91E-09
B18	B108	CD3+CD4+helper(inducer)T	Anti-Cyclin	-0.1955	1.91E-09
B18	B109	CD3+CD4+helper(inducer)T	AMA-M2	-0.1955	1.91E-09
B18	B97	CD3+CD4+helper(inducer)T	dsDNA	-0.1955	1.91E-09
B18	B98	CD3+CD4+helper(inducer)T	anti-Jo-1	-0.1955	1.91E-09
B18	B99	CD3+CD4+helper(inducer)T	anti-PM-Scl	-0.1955	1.91E-09
B18	B67	CD3+CD4+helper(inducer)T	WBRSR(mPas_50)	-0.17456	8.72E-08
B18	B64	CD3+CD4+helper(inducer)T	WBRSR(mPas_5)	-0.17372	1.01E-07
B18	B195	CD3+CD4+helper(inducer)T	Na	0.172109	1.33E-07
B18	B211	CD3+CD4+helper(inducer)T	IOP-R	-0.17061	1.71E-07
B18	B66	CD3+CD4+helper(inducer)T	WBRSR(mPas_100)	-0.16933	2.12E-07
B18	B197	CD3+CD4+helper(inducer)T	AG	-0.16502	4.31E-07
B18	B155	CD3+CD4+helper(inducer)T	IgA	-0.16308	5.89E-07
B18	B65	CD3+CD4+helper(inducer)T	WBRSR(mPas_1)	-0.1612	7.96E-07
B180	B194	Tf	Cl	0.304139	0
B180	B195	Tf	Na	0.187413	8.79E-09
B181	B182	APTT	TT	0.638126	0
B181	B183	APTT	PTA	0.524274	0
B181	B184	APTT	INR	0.822094	0
B181	B185	APTT	PT	0.78087	0
B182	B184	TT	INR	0.386807	0
B182	B186	TT	PFC	-0.75055	0
B182	B185	TT	PT	0.241921	7.95E-14
B182	B197	TT	AG	0.160609	8.74E-07
B183	B184	PTA	INR	0.586316	0
B183	B185	PTA	PT	0.619629	0
B183	B197	PTA	AG	-0.20533	2.72E-10
B183	B186	PTA	PFC	0.191225	4.31E-09
B184	B185	INR	PT	0.956719	0
B184	B194	INR	Cl	-0.23652	2.90E-13

B184	B193	INR	K	-0.21381	4.68E-11
B184	B195	INR	Na	-0.16261	6.35E-07
B185	B186	PT	PFC	0.280722	0
B185	B194	PT	Cl	-0.17552	7.39E-08
B185	B193	PT	K	-0.16928	2.13E-07
B186	B195	PFC	Na	-0.17553	7.37E-08
B186	B200	PFC	OP	-0.1639	5.16E-07
B189	B190	eGFB	Cre	-0.55214	0
B189	B191	eGFB	Urea	-0.57439	0
B189	B192	eGFB	UA	-0.32807	0
B189	B218	eGFB	DBP	0.196044	1.72E-09
B189	B203	eGFB	DH	0.171545	1.46E-07
B19	B100	CD3+CD4+/CD3+CD8+	anti-Ro52	-0.58695	0
B19	B101	CD3+CD4+/CD3+CD8+	anti-Sc70	-0.58695	0
B19	B102	CD3+CD4+/CD3+CD8+	anti-Sm	-0.58695	0
B19	B103	CD3+CD4+/CD3+CD8+	anti-SSA/Ro	-0.58695	0
B19	B104	CD3+CD4+/CD3+CD8+	anti-SSB	-0.58695	0
B19	B105	CD3+CD4+/CD3+CD8+	anti-U1RNP	-0.58695	0
B19	B106	CD3+CD4+/CD3+CD8+	anti-P	-0.58695	0
B19	B107	CD3+CD4+/CD3+CD8+	AnuA	-0.37269	0
B19	B108	CD3+CD4+/CD3+CD8+	Anti-Cyclin	-0.58695	0
B19	B109	CD3+CD4+/CD3+CD8+	AMA-M2	-0.58695	0
B19	B110	CD3+CD4+/CD3+CD8+	AHA	-0.37269	0
B19	B114	CD3+CD4+/CD3+CD8+	TSH TT3 TT4(TT4)	0.274132	0
B19	B119	CD3+CD4+/CD3+CD8+	Hcv-rna	-0.37435	0
B19	B121	CD3+CD4+/CD3+CD8+	C3	0.37435	0
B19	B122	CD3+CD4+/CD3+CD8+	C4	0.37435	0
B19	B124	CD3+CD4+/CD3+CD8+	ACTH	0.63744	0
B19	B125	CD3+CD4+/CD3+CD8+	Cor	0.63744	0
B19	B135	CD3+CD4+/CD3+CD8+	TBA	0.410616	0
B19	B138	CD3+CD4+/CD3+CD8+	PAB	-0.35872	0
B19	B139	CD3+CD4+/CD3+CD8+	ADA	0.410459	0
B19	B151	CD3+CD4+/CD3+CD8+	Anti-CCP	0.355992	0
B19	B152	CD3+CD4+/CD3+CD8+	ACA-Ig(G-A-M)	0.374329	0
B19	B157	CD3+CD4+/CD3+CD8+	IgG	0.378775	0
B19	B158	CD3+CD4+/CD3+CD8+	IgM	0.289804	0
B19	B159	CD3+CD4+/CD3+CD8+	β2-MG	0.37435	0
B19	B169	CD3+CD4+/CD3+CD8+	U-CRY	0.374348	0
B19	B180	CD3+CD4+/CD3+CD8+	Tf	-0.29745	0
B19	B184	CD3+CD4+/CD3+CD8+	INR	0.273091	0
B19	B193	CD3+CD4+/CD3+CD8+	K	-0.45401	0
B19	B21	CD3+CD4+/CD3+CD8+	CD3+CD4-CD8-	-0.29308	0
B19	B22	CD3+CD4+/CD3+CD8+	CD3+CD8+(T)	-0.89384	0
B19	B23	CD3+CD4+/CD3+CD8+	CD3-CD16+CD56+(NK)	0.456717	0

B19	B79	CD3+CD4+/CD3+CD8+	hb V -dna	-0.31675	0
B19	B92	CD3+CD4+/CD3+CD8+	ALDH2-Glu504Glu	0.449808	0
B19	B93	CD3+CD4+/CD3+CD8+	ALDH2-Glu504Lys	-0.44981	0
B19	B94	CD3+CD4+/CD3+CD8+	ALDH2-Lys504Lys	-0.44981	0
B19	B95	CD3+CD4+/CD3+CD8+	anti-cenpb	0.625369	0
B19	B97	CD3+CD4+/CD3+CD8+	dsDNA	-0.58695	0
B19	B98	CD3+CD4+/CD3+CD8+	anti-Jo-1	-0.58695	0
B19	B99	CD3+CD4+/CD3+CD8+	anti-PM-Scl	-0.58695	0
B19	B20	CD3+CD4+/CD3+CD8+	CD3+CD4+CD8+	0.254105	4.00E-15
B19	B156	CD3+CD4+/CD3+CD8+	IgE	-0.22696	2.63E-12
B19	B194	CD3+CD4+/CD3+CD8+	Cl	-0.21328	5.23E-11
B19	B66	CD3+CD4+/CD3+CD8+	WBRSR(mPas_100)	0.203065	4.30E-10
B19	B120	CD3+CD4+/CD3+CD8+	HCV-cAg	-0.20213	5.18E-10
B19	B67	CD3+CD4+/CD3+CD8+	WBRSR(mPas_50)	0.198093	1.15E-09
B19	B24	CD3+CD4+/CD3+CD8+	αHBDH	0.195601	1.87E-09
B19	B185	CD3+CD4+/CD3+CD8+	PT	0.191902	3.80E-09
B19	B63	CD3+CD4+/CD3+CD8+	WBRSR(mPas_200)	0.190859	4.62E-09
B19	B62	CD3+CD4+/CD3+CD8+	WBHSRVI	0.169139	2.18E-07
B19	B61	CD3+CD4+/CD3+CD8+	WBHSRV	0.164573	4.63E-07
B19	B136	CD3+CD4+/CD3+CD8+	5'NT	0.164345	4.80E-07
B19	B183	CD3+CD4+/CD3+CD8+	PTA	-0.16073	8.58E-07
B190	B191	Cre	Urea	0.619123	0
B190	B207	Cre	WHR	0.434298	0
B190	B192	Cre	UA	0.215575	3.21E-11
B191	B192	Urea	UA	0.281736	0
B193	B198	K	P	-0.19924	9.20E-10
B193	B195	K	Na	-0.17746	5.27E-08
B193	B196	K	TCO2	-0.16654	3.36E-07
B193	B197	K	AG	0.16062	8.73E-07
B194	B195	Cl	Na	0.497107	0
B194	B196	Cl	TCO2	-0.49512	0
B194	B197	Cl	AG	-0.2978	0
B194	B198	Cl	P	-0.16329	5.70E-07
B194	B200	Cl	OP	0.161571	7.50E-07
B195	B197	Na	AG	0.269477	0
B195	B200	Na	OP	0.673743	0
B195	B198	Na	P	0.198834	9.96E-10
B196	B197	TCO2	AG	-0.43419	0
B196	B198	TCO2	P	-0.18118	2.73E-08
B197	B198	AG	P	0.535825	0
B197	B200	AG	OP	0.467884	0
B198	B200	P	OP	0.285742	0
B198	B199	P	Mg	-0.18502	1.36E-08
B199	B200	Mg	OP	-0.34061	0

B2	B163	Age	SG	-0.29075	0
B2	B189	Age	eGFB	-0.42783	0
B2	B216	Age	BMI	-0.39055	0
B2	B218	Age	DBP	-0.30821	0
B2	B221	Age	Hei	-0.3635	0
B2	B222	Age	NS	-0.27285	0
B2	B46	Age	RBC Count	-0.31889	0
B2	B51	Age	Hb	-0.31657	0
B2	B141	Age	AST/ALT	0.254601	3.55E-15
B2	B126	Age	Alb	-0.25378	4.22E-15
B2	B128	Age	ALT	-0.23108	1.03E-12
B2	B47	Age	RDW	0.209134	1.25E-10
B2	B127	Age	A-G ratio	-0.19663	1.53E-09
B2	B215	Age	BMI	-0.19165	3.98E-09
B2	B191	Age	Urea	0.179213	3.87E-08
B2	B176	Age	Ucr	-0.17267	1.21E-07
B2	B203	Age	DH	-0.16639	3.44E-07
B2	B210	Age	Hip	-0.16619	3.55E-07
B2	B71	Age	TG	-0.16608	3.62E-07
B2	B209	Age	Smoking	-0.16416	4.95E-07
B2	B220	Age	SBP	0.16034	9.12E-07
B20	B21	CD3+CD4+CD8+	CD3+CD4-CD8-	-0.46032	0
B20	B87	CD3+CD4+CD8+	NSE	0.288252	0
B20	B22	CD3+CD4+CD8+	CD3+CD8+(T)	-0.24732	2.13E-14
B20	B199	CD3+CD4+CD8+	Mg	-0.19316	2.99E-09
B20	B74	CD3+CD4+CD8+	ApoA1	-0.1605	8.89E-07
B201	B204	e-habits	Drinking	0.204584	3.16E-10
B204	B209	Drinking	Smoking	0.517493	0
B205	B221	KDA	Hei	-0.17776	5.00E-08
B206	B207	Waist	WHR	0.513301	0
B206	B210	Waist	Hip	0.736295	0
B206	B215	Waist	BMI	0.794754	0
B206	B216	Waist	BMI	0.75418	0
B206	B222	Waist	NS	0.635494	0
B206	B221	Waist	Hei	0.257134	1.78E-15
B207	B215	WHR	BMI	0.30677	0
B207	B216	WHR	BMI	0.251805	6.88E-15
B207	B222	WHR	NS	0.222942	6.47E-12
B209	B221	Smoking	Hei	0.224612	4.46E-12
B209	B222	Smoking	NS	0.177199	5.52E-08
B21	B100	CD3+CD4-CD8-	anti-Ro52	0.478849	0
B21	B101	CD3+CD4-CD8-	anti-Sc70	0.478849	0
B21	B102	CD3+CD4-CD8-	anti-Sm	0.478849	0
B21	B103	CD3+CD4-CD8-	anti-SSA/Ro	0.478849	0

B21	B104	CD3+CD4-CD8-	anti-SSB	0.478849	0
B21	B105	CD3+CD4-CD8-	anti-U1RNP	0.478849	0
B21	B106	CD3+CD4-CD8-	anti-P	0.478849	0
B21	B107	CD3+CD4-CD8-	AnuA	0.517672	0
B21	B108	CD3+CD4-CD8-	Anti-Cyclin	0.478849	0
B21	B109	CD3+CD4-CD8-	AMA-M2	0.478849	0
B21	B110	CD3+CD4-CD8-	AHA	0.517672	0
B21	B22	CD3+CD4-CD8-	CD3+CD8+(T)	0.28714	0
B21	B97	CD3+CD4-CD8-	dsDNA	0.478849	0
B21	B98	CD3+CD4-CD8-	anti-Jo-1	0.478849	0
B21	B99	CD3+CD4-CD8-	anti-PM-Scl	0.478849	0
B21	B34	CD3+CD4-CD8-	TK	0.208689	1.37E-10
B21	B180	CD3+CD4-CD8-	Tf	0.208521	1.41E-10
B21	B181	CD3+CD4-CD8-	APTT	-0.20403	3.54E-10
B210	B215	Hip	BMI	0.70934	0
B210	B216	Hip	BMI	0.731883	0
B210	B221	Hip	Hei	0.325612	0
B210	B222	Hip	NS	0.632276	0
B211	B212	IOP-R	IOP-L	0.742208	0
B213	B214	CVA-R	CVA-L	-0.17209	1.33E-07
B215	B216	BMI	BMI	0.739076	0
B215	B222	BMI	NS	0.549001	0
B216	B221	BMI	Hei	0.683234	0
B216	B222	BMI	NS	0.752759	0
B216	B218	BMI	DBP	0.209781	1.09E-10
B218	B220	DBP	SBP	0.408534	0
B218	B221	DBP	Hei	0.191096	4.42E-09
B22	B100	CD3+CD8+(T)	anti-Ro52	0.645824	0
B22	B101	CD3+CD8+(T)	anti-Sc70	0.645824	0
B22	B102	CD3+CD8+(T)	anti-Sm	0.645824	0
B22	B103	CD3+CD8+(T)	anti-SSA/Ro	0.645824	0
B22	B104	CD3+CD8+(T)	anti-SSB	0.645824	0
B22	B105	CD3+CD8+(T)	anti-U1RNP	0.645824	0
B22	B106	CD3+CD8+(T)	anti-P	0.645824	0
B22	B107	CD3+CD8+(T)	AnuA	0.528359	0
B22	B108	CD3+CD8+(T)	Anti-Cyclin	0.645824	0
B22	B109	CD3+CD8+(T)	AMA-M2	0.645824	0
B22	B110	CD3+CD8+(T)	AHA	0.528359	0
B22	B119	CD3+CD8+(T)	Hcv-rna	0.578055	0
B22	B120	CD3+CD8+(T)	HCV-cAg	0.313095	0
B22	B121	CD3+CD8+(T)	C3	-0.57806	0
B22	B122	CD3+CD8+(T)	C4	-0.57806	0
B22	B124	CD3+CD8+(T)	ACTH	-0.83621	0
B22	B125	CD3+CD8+(T)	Cor	-0.83621	0

B22	B135	CD3+CD8+(T)	TBA	-0.52147	0
B22	B138	CD3+CD8+(T)	PAB	0.38841	0
B22	B139	CD3+CD8+(T)	ADA	-0.62706	0
B22	B151	CD3+CD8+(T)	Anti-CCP	-0.42439	0
B22	B152	CD3+CD8+(T)	ACA-Ig(G-A-M)	-0.57802	0
B22	B157	CD3+CD8+(T)	IgG	-0.46782	0
B22	B158	CD3+CD8+(T)	IgM	-0.2665	0
B22	B159	CD3+CD8+(T)	β2-MG	-0.57806	0
B22	B169	CD3+CD8+(T)	U-CRY	-0.57805	0
B22	B180	CD3+CD8+(T)	Tf	0.330112	0
B22	B184	CD3+CD8+(T)	INR	-0.37799	0
B22	B193	CD3+CD8+(T)	K	0.488858	0
B22	B194	CD3+CD8+(T)	Cl	0.330778	0
B22	B23	CD3+CD8+(T)	CD3-CD16+CD56+(NK)	-0.77592	0
B22	B66	CD3+CD8+(T)	WBRSR(mPas_100)	-0.29045	0
B22	B67	CD3+CD8+(T)	WBRSR(mPas_50)	-0.28876	0
B22	B79	CD3+CD8+(T)	hb V -dna	0.41559	0
B22	B92	CD3+CD8+(T)	ALDH2-Glu504Glu	-0.5901	0
B22	B93	CD3+CD8+(T)	ALDH2-Glu504Lys	0.590103	0
B22	B94	CD3+CD8+(T)	ALDH2-Lys504Lys	0.590103	0
B22	B95	CD3+CD8+(T)	anti-cenpb	-0.39561	0
B22	B97	CD3+CD8+(T)	dsDNA	0.645824	0
B22	B98	CD3+CD8+(T)	anti-Jo-1	0.645824	0
B22	B99	CD3+CD8+(T)	anti-PM-Scl	0.645824	0
B22	B63	CD3+CD8+(T)	WBRSR(mPas_200)	-0.26266	4.44E-16
B22	B24	CD3+CD8+(T)	αHBDH	-0.25883	1.11E-15
B22	B185	CD3+CD8+(T)	PT	-0.25537	2.66E-15
B22	B183	CD3+CD8+(T)	PTA	0.254205	3.77E-15
B22	B64	CD3+CD8+(T)	WBRSR(mPas_5)	-0.24495	3.80E-14
B22	B155	CD3+CD8+(T)	IgA	-0.24277	6.48E-14
B22	B61	CD3+CD8+(T)	WBHSRV	-0.23476	4.37E-13
B22	B114	CD3+CD8+(T)	TSH TT3 TT4(TT4)	-0.2223	7.46E-12
B22	B62	CD3+CD8+(T)	WBHSRVI	-0.2136	4.89E-11
B22	B197	CD3+CD8+(T)	AG	-0.19806	1.16E-09
B22	B65	CD3+CD8+(T)	WBRSR(mPas_1)	-0.19661	1.54E-09
B22	B136	CD3+CD8+(T)	5'NT	-0.17126	1.53E-07
B22	B156	CD3+CD8+(T)	IgE	0.170248	1.81E-07
B22	B154	CD3+CD8+(T)	RF	0.169713	1.98E-07
B22	B56	CD3+CD8+(T)	IR	-0.16896	2.25E-07
B220	B221	SBP	Hei	-0.17961	3.61E-08
B221	B222	Hei	NS	0.530881	0
B23	B100	CD3-CD16+CD56+(NK)	anti-Ro52	-0.36042	0
B23	B101	CD3-CD16+CD56+(NK)	anti-Sc70	-0.36042	0
B23	B102	CD3-CD16+CD56+(NK)	anti-Sm	-0.36042	0

B23	B103	CD3-CD16+CD56+(NK)	anti-SSA/Ro	-0.36042	0
B23	B104	CD3-CD16+CD56+(NK)	anti-SSB	-0.36042	0
B23	B105	CD3-CD16+CD56+(NK)	anti-U1RNP	-0.36042	0
B23	B106	CD3-CD16+CD56+(NK)	anti-P	-0.36042	0
B23	B107	CD3-CD16+CD56+(NK)	AnuA	-0.43092	0
B23	B108	CD3-CD16+CD56+(NK)	Anti-Cyclin	-0.36042	0
B23	B109	CD3-CD16+CD56+(NK)	AMA-M2	-0.36042	0
B23	B110	CD3-CD16+CD56+(NK)	AHA	-0.43092	0
B23	B119	CD3-CD16+CD56+(NK)	Hcv-rna	-0.5182	0
B23	B120	CD3-CD16+CD56+(NK)	HCV-cAg	-0.28136	0
B23	B121	CD3-CD16+CD56+(NK)	C3	0.5182	0
B23	B122	CD3-CD16+CD56+(NK)	C4	0.518199	0
B23	B124	CD3-CD16+CD56+(NK)	ACTH	0.899773	0
B23	B125	CD3-CD16+CD56+(NK)	Cor	0.899773	0
B23	B135	CD3-CD16+CD56+(NK)	TBA	0.57149	0
B23	B138	CD3-CD16+CD56+(NK)	PAB	-0.29828	0
B23	B139	CD3-CD16+CD56+(NK)	ADA	0.677416	0
B23	B151	CD3-CD16+CD56+(NK)	Anti-CCP	0.441254	0
B23	B152	CD3-CD16+CD56+(NK)	ACA-Ig(G-A-M)	0.51817	0
B23	B157	CD3-CD16+CD56+(NK)	IgG	0.414368	0
B23	B159	CD3-CD16+CD56+(NK)	β2-MG	0.5182	0
B23	B169	CD3-CD16+CD56+(NK)	U-CRY	0.518197	0
B23	B180	CD3-CD16+CD56+(NK)	Tf	-0.29804	0
B23	B183	CD3-CD16+CD56+(NK)	PTA	-0.28655	0
B23	B184	CD3-CD16+CD56+(NK)	INR	0.414032	0
B23	B185	CD3-CD16+CD56+(NK)	PT	0.278718	0
B23	B193	CD3-CD16+CD56+(NK)	K	-0.30012	0
B23	B194	CD3-CD16+CD56+(NK)	Cl	-0.42043	0
B23	B24	CD3-CD16+CD56+(NK)	αHBDH	0.281761	0
B23	B63	CD3-CD16+CD56+(NK)	WBRSR(mPas_200)	0.285976	0
B23	B64	CD3-CD16+CD56+(NK)	WBRSR(mPas_5)	0.301042	0
B23	B66	CD3-CD16+CD56+(NK)	WBRSR(mPas_100)	0.326208	0
B23	B67	CD3-CD16+CD56+(NK)	WBRSR(mPas_50)	0.329607	0
B23	B79	CD3-CD16+CD56+(NK)	hb V -dna	-0.44822	0
B23	B92	CD3-CD16+CD56+(NK)	ALDH2-Glu504Glu	0.635449	0
B23	B93	CD3-CD16+CD56+(NK)	ALDH2-Glu504Lys	-0.63545	0
B23	B94	CD3-CD16+CD56+(NK)	ALDH2-Lys504Lys	-0.63545	0
B23	B97	CD3-CD16+CD56+(NK)	dsDNA	-0.36042	0
B23	B98	CD3-CD16+CD56+(NK)	anti-Jo-1	-0.36042	0
B23	B99	CD3-CD16+CD56+(NK)	anti-PM-Scl	-0.36042	0
B23	B61	CD3-CD16+CD56+(NK)	WBHSRV	0.263106	4.44E-16
B23	B195	CD3-CD16+CD56+(NK)	Na	-0.25958	8.88E-16
B23	B65	CD3-CD16+CD56+(NK)	WBRSR(mPas_1)	0.259751	8.88E-16
B23	B197	CD3-CD16+CD56+(NK)	AG	0.252951	5.33E-15

B23	B155	CD3-CD16+CD56+(NK)	IgA	0.239068	1.58E-13
B23	B198	CD3-CD16+CD56+(NK)	P	0.236039	3.24E-13
B23	B158	CD3-CD16+CD56+(NK)	IgM	0.227709	2.22E-12
B23	B199	CD3-CD16+CD56+(NK)	Mg	0.221937	8.07E-12
B23	B59	CD3-CD16+CD56+(NK)	WBRV	0.219652	1.33E-11
B23	B62	CD3-CD16+CD56+(NK)	WBHSRVI	0.20749	1.75E-10
B23	B54	CD3-CD16+CD56+(NK)	ESR	0.203202	4.18E-10
B23	B136	CD3-CD16+CD56+(NK)	5'NT	0.182778	2.05E-08
B23	B60	CD3-CD16+CD56+(NK)	WBRVI	0.177296	5.42E-08
B23	B27	CD3-CD16+CD56+(NK)	LDH	0.169679	2.00E-07
B23	B56	CD3-CD16+CD56+(NK)	IR	0.168527	2.42E-07
B24	B124	αHBDH	ACTH	0.313291	0
B24	B125	αHBDH	Cor	0.313291	0
B24	B26	αHBDH	CKMB	0.36185	0
B24	B27	αHBDH	LDH	0.48692	0
B24	B135	αHBDH	TBA	0.241072	9.77E-14
B24	B138	αHBDH	PAB	-0.23804	2.02E-13
B24	B139	αHBDH	ADA	0.191549	4.06E-09
B24	B151	αHBDH	Anti-CCP	0.177468	5.26E-08
B24	B119	αHBDH	Hcv-rna	-0.17596	6.85E-08
B24	B121	αHBDH	C3	0.175957	6.85E-08
B24	B122	αHBDH	C4	0.175957	6.85E-08
B24	B159	αHBDH	β2-MG	0.175957	6.85E-08
B24	B169	αHBDH	U-CRY	0.175957	6.85E-08
B24	B152	αHBDH	ACA-Ig(G-A-M)	0.175947	6.86E-08
B24	B67	αHBDH	WBRSR(mPas_50)	0.168005	2.64E-07
B24	B92	αHBDH	ALDH2-Glu504Glu	0.167863	2.70E-07
B24	B93	αHBDH	ALDH2-Glu504Lys	-0.16786	2.70E-07
B24	B94	αHBDH	ALDH2-Lys504Lys	-0.16786	2.70E-07
B24	B64	αHBDH	WBRSR(mPas_5)	0.167796	2.73E-07
B24	B66	αHBDH	WBRSR(mPas_100)	0.164226	4.90E-07
B24	B157	αHBDH	IgG	0.163937	5.13E-07
B25	B87	CK	NSE	0.382323	0
B25	B82	CK	CEA	0.212205	6.57E-11
B25	B86	CK	TBhCG	-0.20853	1.41E-10
B25	B26	CK	CKMB	0.206905	1.97E-10
B25	B89	CK	CA 72-4	0.173526	1.04E-07
B26	B89	CKMB	CA 72-4	0.320188	0
B26	B87	CKMB	NSE	0.252218	6.22E-15
B26	B155	CKMB	IgA	0.213051	5.50E-11
B26	B27	CKMB	LDH	0.211688	7.32E-11
B26	B82	CKMB	CEA	0.188941	6.62E-09
B26	B156	CKMB	IgE	-0.18192	2.39E-08
B26	B124	CKMB	ACTH	0.179604	3.61E-08

B26	B125	CKMB	Cor	0.179604	3.61E-08
B26	B163	CKMB	SG	0.173039	1.13E-07
B26	B138	CKMB	PAB	-0.1695	2.06E-07
B26	B170	CKMB	U-GLU	0.160381	9.06E-07
B27	B138	LDH	PAB	-0.22528	3.84E-12
B27	B136	LDH	5'NT	0.214471	4.07E-11
B27	B139	LDH	ADA	0.19657	1.55E-09
B27	B137	LDH	AFU	0.182792	2.04E-08
B27	B124	LDH	ACTH	0.178635	4.29E-08
B27	B125	LDH	Cor	0.178635	4.29E-08
B28	B202	PRL	e-times	0.823087	0
B28	B35	PRL	β2-MG	0.54386	0
B28	B167	PRL	PC	0.229018	1.65E-12
B29	B202	E2	e-times	0.617052	0
B29	B31	E2	FSH	0.627628	0
B29	B32	E2	Testo	0.502928	0
B3	B124	Hcy	ACTH	0.195264	2.00E-09
B3	B125	Hcy	Cor	0.195264	2.00E-09
B3	B84	Hcy	C-PSA	0.178085	4.72E-08
B3	B23	Hcy	CD3-CD16+CD56+(NK)	0.172196	1.31E-07
B3	B16	Hcy	CD3+T	-0.17097	1.61E-07
B30	B33	LH	Prog	-0.23652	2.89E-13
B31	B167	FSH	PC	0.705999	0
B31	B35	FSH	β2-MG	0.652926	0
B31	B32	FSH	Testo	0.240048	1.25E-13
B32	B202	Testo	e-times	0.796437	0
B33	B36	Prog	V-Lym%	-0.65133	0
B33	B38	Prog	BAND%	-0.59611	0
B33	B223	Prog	FH	-0.22193	8.08E-12
B34	B181	TK	APTT	-0.1673	2.96E-07
B34	B107	TK	AnuA	0.166439	3.41E-07
B34	B110	TK	AHA	0.166439	3.41E-07
B35	B167	β2-MG	PC	0.464399	0
B35	B37	β2-MG	BAND	0.911291	0
B36	B223	V-Lym%	FH	0.319553	0
B37	B202	BAND	e-times	0.490137	0
B38	B223	BAND%	FH	-0.75074	0
B39	B78	Other cells	HBcAb	0.522491	0
B39	B225	Other cells	hPBG	-0.22102	9.87E-12
B4	B137	WB-Ca	AFU	-0.31359	0
B4	B139	WB-Ca	ADA	-0.44985	0
B4	B15	WB-Ca	CD3-CD19+(B cell)	0.444867	0
B4	B157	WB-Ca	IgG	-0.37859	0
B4	B16	WB-Ca	CD3+T	0.298987	0

B4	B18	WB-Ca	CD3+CD4+helper(inducer) T	0.385709	0
B4	B23	WB-Ca	CD3-CD16+CD56+(NK)	-0.33846	0
B4	B6	WB-Ca	WB-Fe	-1	0
B4	B19	WB-Ca	CD3+CD4+/CD3+CD8+	0.228677	1.78E-12
B4	B54	WB-Ca	ESR	-0.2235	5.71E-12
B4	B59	WB-Ca	WBRV	-0.21355	4.94E-11
B4	B198	WB-Ca	P	-0.20696	1.95E-10
B4	B85	WB-Ca	SCC	0.196611	1.54E-09
B4	B69	WB-Ca	PV	-0.17633	6.42E-08
B4	B57	WB-Ca	RBCAI	-0.16653	3.36E-07
B40	B42	WBC	MON	0.681892	0
B40	B130	WBC	GLB	0.257887	1.33E-15
B40	B52	WBC	PLT/BPC	0.22867	1.79E-12
B40	B127	WBC	A-G ratio	-0.22438	4.69E-12
B40	B91	WBC	TP	0.200133	7.71E-10
B40	B72	WBC	HDL-C	-0.17722	5.49E-08
B40	B192	WBC	UA	0.176175	6.59E-08
B40	B71	WBC	TG	0.16418	4.93E-07
B41	B42	MON％	MON	0.603712	0
B41	B118	MON％	CEA1	0.167424	2.90E-07
B42	B127	MON	A-G ratio	-0.2144	4.13E-11
B42	B192	MON	UA	0.204855	3.00E-10
B42	B48	MON	LYM%	-0.20005	7.83E-10
B42	B130	MON	GLB	0.19437	2.37E-09
B42	B52	MON	PLT/BPC	0.183399	1.83E-08
B42	B123	MON	hs-CRP1	0.168929	2.26E-07
B43	B44	MCV	MCH	0.855515	0
B43	B46	MCV	RBC Count	-0.43928	0
B43	B47	MCV	RDW	0.521707	0
B43	B52	MCV	PLT/BPC	-0.16061	8.74E-07
B44	B45	MCH	MCHC	0.56285	0
B44	B46	MCH	RBC Count	-0.28999	0
B44	B47	MCH	RDW	0.270455	0
B44	B51	MCH	Hb	0.274286	0
B44	B133	MCH	TBIL	0.179231	3.86E-08
B45	B47	MCHC	RDW	-0.31683	0
B45	B51	MCHC	Hb	0.467885	0
B45	B222	MCHC	NS	0.206077	2.34E-10
B45	B221	MCHC	Hei	0.190497	4.95E-09
B45	B129	MCHC	IBIL	0.179708	3.55E-08
B46	B126	RBC Count	Alb	0.268896	0
B46	B189	RBC Count	eGFB	0.345415	0
B46	B191	RBC Count	Urea	-0.28976	0

B46	B216	RBC Count	BMI	0.303708	0
B46	B218	RBC Count	DBP	0.27977	0
B46	B221	RBC Count	Hei	0.322046	0
B46	B47	RBC Count	RDW	-0.44008	0
B46	B51	RBC Count	Hb	0.836771	0
B46	B222	RBC Count	NS	0.260232	8.88E-16
B46	B163	RBC Count	SG	0.257412	1.78E-15
B46	B141	RBC Count	AST/ALT	-0.23897	1.62E-13
B46	B129	RBC Count	IBIL	0.218175	1.84E-11
B46	B190	RBC Count	Cre	-0.20855	1.41E-10
B46	B170	RBC Count	U-GLU	0.207082	1.90E-10
B46	B173	RBC Count	NTT	-0.1886	7.06E-09
B46	B128	RBC Count	ALT	0.160174	9.37E-07
B47	B51	RDW	Hb	-0.28797	0
B47	B111	RDW	TSH	0.215616	3.19E-11
B47	B126	RDW	Alb	-0.21213	6.67E-11
B47	B191	RDW	Urea	0.174288	9.13E-08
B47	B127	RDW	A-G ratio	-0.16734	2.95E-07
B47	B52	RDW	PLT/BPC	-0.16675	3.24E-07
B47	B170	RDW	U-GLU	-0.16206	6.94E-07
B48	B53	LYM%	GR%	-0.92051	0
B48	B190	LYM%	Cre	-0.18857	7.09E-09
B48	B189	LYM%	eGFB	0.184265	1.57E-08
B48	B72	LYM%	HDL-C	0.179033	4.00E-08
B49	B50	baso%	eo%	0.253322	4.66E-15
B5	B137	WB-Mg	AFU	-0.31359	0
B5	B139	WB-Mg	ADA	-0.44985	0
B5	B15	WB-Mg	CD3-CD19+(B cell)	0.444867	0
B5	B157	WB-Mg	IgG	-0.37859	0
B5	B16	WB-Mg	CD3+T	0.298987	0
B5	B18	WB-Mg	CD3+CD4+helper(inducer) T	0.385709	0
B5	B23	WB-Mg	CD3-CD16+CD56+(NK)	-0.33846	0
B5	B19	WB-Mg	CD3+CD4+/CD3+CD8+	0.228677	1.78E-12
B5	B54	WB-Mg	ESR	-0.2235	5.71E-12
B5	B59	WB-Mg	WBRV	-0.21355	4.94E-11
B5	B198	WB-Mg	P	-0.20696	1.95E-10
B5	B85	WB-Mg	SCC	0.196611	1.54E-09
B5	B69	WB-Mg	PV	-0.17633	6.42E-08
B5	B57	WB-Mg	RBCAI	-0.16653	3.36E-07
B50	B132	eo%	Dbil	0.560793	0
B50	B133	eo%	TBIL	0.343238	0
B50	B140	eo%	r-GT	0.611773	0
B50	B131	eo%	AST	0.264732	2.22E-16

B50	B53	eo%	GR%	-0.261	6.66E-16
B50	B128	eo%	ALT	0.221886	8.17E-12
B50	B190	eo%	Cre	0.180207	3.25E-08
B51	B126	Hb	Alb	0.27008	0
B51	B129	Hb	IBIL	0.30224	0
B51	B163	Hb	SG	0.26875	0
B51	B189	Hb	eGFB	0.355955	0
B51	B191	Hb	Urea	-0.30892	0
B51	B216	Hb	BMI	0.334989	0
B51	B221	Hb	Hei	0.342946	0
B51	B222	Hb	NS	0.305232	0
B51	B218	Hb	DBP	0.265743	2.22E-16
B51	B133	Hb	TBIL	0.226019	3.25E-12
B51	B141	Hb	AST/ALT	-0.22355	5.65E-12
B51	B209	Hb	Smoking	0.197105	1.40E-09
B51	B170	Hb	U-GLU	0.195129	2.05E-09
B51	B173	Hb	NTT	-0.19135	4.21E-09
B51	B190	Hb	Cre	-0.18418	1.59E-08
B51	B128	Hb	ALT	0.183591	1.77E-08
B51	B127	Hb	A-G ratio	0.183146	1.92E-08
B51	B206	Hb	Waist	0.161049	8.15E-07
B51	B210	Hb	Hip	0.159903	9.78E-07
B52	B129	PLT/BPC	IBIL	-0.24075	1.05E-13
B52	B133	PLT/BPC	TBIL	-0.23583	3.40E-13
B53	B140	GR%	r-GT	-0.17998	3.38E-08
B54	B58	ESR	HCT	-0.47296	0
B54	B59	ESR	WBRV	0.319805	0
B54	B69	ESR	PV	0.366541	0
B54	B62	ESR	WBHSRVI	-0.24238	7.13E-14
B54	B139	ESR	ADA	0.241139	9.61E-14
B54	B138	ESR	PAB	-0.18855	7.12E-09
B54	B55	ESR	TK	0.18139	2.63E-08
B54	B172	ESR	KB	0.176953	5.76E-08
B54	B195	ESR	Na	-0.17465	8.59E-08
B54	B123	ESR	hs-CRP1	0.169378	2.10E-07
B55	B56	TK	IR	0.894206	0
B55	B57	TK	RBCAI	-0.26997	0
B55	B58	TK	HCT	-0.46133	0
B55	B59	TK	WBRV	0.380495	0
B55	B61	TK	WBHSRV	0.804455	0
B55	B62	TK	WBHSRVI	0.570656	0
B55	B63	TK	WBRSR(mPas_200)	0.314666	0
B55	B66	TK	WBRSR(mPas_100)	0.29943	0
B55	B67	TK	WBRSR(mPas_50)	0.274019	0

B55	B69	TK	PV	-0.57917	0
B55	B60	TK	WBRVI	0.242988	6.13E-14
B56	B59	IR	WBRV	0.43152	0
B56	B60	IR	WBRVI	0.502272	0
B56	B61	IR	WBHSRV	0.937139	0
B56	B62	IR	WBHSRVI	0.875193	0
B56	B63	IR	WBRSR(mPas_200)	0.662446	0
B56	B64	IR	WBRSR(mPas_5)	0.459886	0
B56	B65	IR	WBRSR(mPas_1)	0.334621	0
B56	B66	IR	WBRSR(mPas_100)	0.642572	0
B56	B67	IR	WBRSR(mPas_50)	0.620565	0
B56	B69	IR	PV	-0.56965	0
B56	B57	IR	RBCAI	-0.24282	6.39E-14
B56	B183	IR	PTA	-0.21675	2.50E-11
B56	B182	IR	TT	0.199663	8.46E-10
B56	B124	IR	ACTH	0.18862	7.03E-09
B56	B125	IR	Cor	0.18862	7.03E-09
B56	B135	IR	TBA	0.180605	3.02E-08
B56	B186	IR	PFC	-0.17743	5.30E-08
B57	B195	RBCAI	Na	0.329545	0
B57	B59	RBCAI	WBRV	0.67815	0
B57	B60	RBCAI	WBRVI	0.630381	0
B57	B61	RBCAI	WBHSRV	-0.2691	0
B57	B64	RBCAI	WBRSR(mPas_5)	0.427094	0
B57	B65	RBCAI	WBRSR(mPas_1)	0.648145	0
B57	B200	RBCAI	OP	0.224312	4.77E-12
B57	B138	RBCAI	PAB	0.196139	1.69E-09
B57	B58	RBCAI	HCT	0.180666	2.99E-08
B57	B194	RBCAI	Cl	0.173868	9.82E-08
B58	B60	HCT	WBRVI	0.49517	0
B58	B62	HCT	WBHSRVI	0.455675	0
B58	B63	HCT	WBRSR(mPas_200)	0.585226	0
B58	B64	HCT	WBRSR(mPas_5)	0.618184	0
B58	B65	HCT	WBRSR(mPas_1)	0.544419	0
B58	B66	HCT	WBRSR(mPas_100)	0.576972	0
B58	B67	HCT	WBRSR(mPas_50)	0.595268	0
B58	B137	HCT	AFU	0.16285	6.11E-07
B59	B60	WBRV	WBRVI	0.808762	0
B59	B61	WBRV	WBHSRV	0.491196	0
B59	B62	WBRV	WBHSRVI	0.389416	0
B59	B63	WBRV	WBRSR(mPas_200)	0.416068	0
B59	B64	WBRV	WBRSR(mPas_5)	0.752807	0
B59	B65	WBRV	WBRSR(mPas_1)	0.842291	0
B59	B66	WBRV	WBRSR(mPas_100)	0.452953	0

B59	B67	WBRV	WBRSR(mPas_50)	0.514036	0
B59	B195	WBRV	Na	0.226093	3.20E-12
B59	B200	WBRV	OP	0.217211	2.26E-11
B59	B186	WBRV	PFC	-0.20799	1.58E-10
B59	B183	WBRV	PTA	-0.19786	1.21E-09
B59	B182	WBRV	TT	0.193051	3.05E-09
B59	B171	WBRV	EC	0.183523	1.79E-08
B59	B197	WBRV	AG	0.176614	6.11E-08
B59	B124	WBRV	ACTH	0.165568	3.94E-07
B59	B125	WBRV	Cor	0.165568	3.94E-07
B6	B137	WB-Fe	AFU	0.313586	0
B6	B139	WB-Fe	ADA	0.449853	0
B6	B15	WB-Fe	CD3-CD19+(B cell)	-0.44487	0
B6	B157	WB-Fe	IgG	0.378593	0
B6	B16	WB-Fe	CD3+T	-0.29899	0
B6	B18	WB-Fe	CD3+CD4+helper(inducer) T	-0.38571	0
B6	B23	WB-Fe	CD3-CD16+CD56+(NK)	0.338457	0
B6	B7	WB-Fe	WB-Cop	-1	0
B6	B8	WB-Fe	WB-Zinc	1	0
B6	B19	WB-Fe	CD3+CD4+/CD3+CD8+	-0.22868	1.78E-12
B6	B54	WB-Fe	ESR	0.223503	5.71E-12
B6	B59	WB-Fe	WBRV	0.213551	4.94E-11
B6	B198	WB-Fe	P	0.206965	1.95E-10
B6	B85	WB-Fe	SCC	-0.19661	1.54E-09
B6	B69	WB-Fe	PV	0.176326	6.42E-08
B6	B57	WB-Fe	RBCAI	0.166529	3.36E-07
B60	B61	WBRVI	WBHSRV	0.465817	0
B60	B62	WBRVI	WBHSRVI	0.678396	0
B60	B63	WBRVI	WBRSR(mPas_200)	0.594514	0
B60	B64	WBRVI	WBRSR(mPas_5)	0.872737	0
B60	B65	WBRVI	WBRSR(mPas_1)	0.917133	0
B60	B66	WBRVI	WBRSR(mPas_100)	0.61004	0
B60	B67	WBRVI	WBRSR(mPas_50)	0.666928	0
B60	B69	WBRVI	PV	-0.34589	0
B60	B183	WBRVI	PTA	-0.23338	6.04E-13
B60	B171	WBRVI	EC	0.229996	1.32E-12
B60	B182	WBRVI	TT	0.228257	1.96E-12
B60	B186	WBRVI	PFC	-0.21788	1.96E-11
B60	B200	WBRVI	OP	0.213278	5.24E-11
B60	B197	WBRVI	AG	0.208734	1.35E-10
B60	B195	WBRVI	Na	0.194516	2.30E-09
B60	B124	WBRVI	ACTH	0.163421	5.58E-07
B60	B125	WBRVI	Cor	0.163421	5.58E-07

B61	B124	WBHSRV	ACTH	0.268099	0
B61	B125	WBHSRV	Cor	0.268099	0
B61	B62	WBHSRV	WBHSRVI	0.851605	0
B61	B63	WBHSRV	WBRSR(mPas_200)	0.805666	0
B61	B64	WBHSRV	WBRSR(mPas_5)	0.588068	0
B61	B65	WBHSRV	WBRSR(mPas_1)	0.431316	0
B61	B66	WBHSRV	WBRSR(mPas_100)	0.799626	0
B61	B67	WBHSRV	WBRSR(mPas_50)	0.776897	0
B61	B69	WBHSRV	PV	-0.25547	2.66E-15
B61	B183	WBHSRV	PTA	-0.23949	1.43E-13
B61	B135	WBHSRV	TBA	0.221432	9.03E-12
B61	B182	WBHSRV	TT	0.212379	6.33E-11
B61	B186	WBHSRV	PFC	-0.19619	1.67E-09
B61	B194	WBHSRV	CI	-0.19584	1.78E-09
B61	B92	WBHSRV	ALDH2-Glu504Glu	0.190896	4.59E-09
B61	B93	WBHSRV	ALDH2-Glu504Lys	-0.1909	4.59E-09
B61	B94	WBHSRV	ALDH2-Lys504Lys	-0.1909	4.59E-09
B62	B63	WBHSRVI	WBRSR(mPas_200)	0.875575	0
B62	B64	WBHSRVI	WBRSR(mPas_5)	0.70655	0
B62	B65	WBHSRVI	WBRSR(mPas_1)	0.554739	0
B62	B66	WBHSRVI	WBRSR(mPas_100)	0.856188	0
B62	B67	WBHSRVI	WBRSR(mPas_50)	0.844693	0
B62	B69	WBHSRVI	PV	-0.44835	0
B62	B183	WBHSRVI	PTA	-0.25511	3.11E-15
B62	B124	WBHSRVI	ACTH	0.237892	2.09E-13
B62	B125	WBHSRVI	Cor	0.237892	2.09E-13
B62	B182	WBHSRVI	TT	0.232931	6.71E-13
B62	B186	WBHSRVI	PFC	-0.20088	6.65E-10
B62	B194	WBHSRVI	CI	-0.18176	2.46E-08
B62	B135	WBHSRVI	TBA	0.180606	3.02E-08
B62	B171	WBHSRVI	EC	0.176573	6.15E-08
B62	B92	WBHSRVI	ALDH2-Glu504Glu	0.170646	1.70E-07
B62	B93	WBHSRVI	ALDH2-Glu504Lys	-0.17065	1.70E-07
B62	B94	WBHSRVI	ALDH2-Lys504Lys	-0.17065	1.70E-07
B62	B197	WBHSRVI	AG	0.163177	5.80E-07
B63	B124	WBRSR(mPas_200)	ACTH	0.300675	0
B63	B125	WBRSR(mPas_200)	Cor	0.300675	0
B63	B64	WBRSR(mPas_200)	WBRSR(mPas_5)	0.839448	0
B63	B65	WBRSR(mPas_200)	WBRSR(mPas_1)	0.662658	0
B63	B66	WBRSR(mPas_200)	WBRSR(mPas_100)	0.994329	0
B63	B67	WBRSR(mPas_200)	WBRSR(mPas_50)	0.985617	0
B63	B194	WBRSR(mPas_200)	CI	-0.26242	4.44E-16
B63	B183	WBRSR(mPas_200)	PTA	-0.25655	2.00E-15
B63	B182	WBRSR(mPas_200)	TT	0.227746	2.20E-12

B63	B92	WBRSR(mPas_200)	ALDH2-Glu504Glu	0.21472	3.86E-11
B63	B93	WBRSR(mPas_200)	ALDH2-Glu504Lys	-0.21472	3.86E-11
B63	B94	WBRSR(mPas_200)	ALDH2-Lys504Lys	-0.21472	3.86E-11
B63	B186	WBRSR(mPas_200)	PFC	-0.21155	7.53E-11
B63	B135	WBRSR(mPas_200)	TBA	0.202269	5.04E-10
B63	B137	WBRSR(mPas_200)	AFU	0.185975	1.15E-08
B63	B139	WBRSR(mPas_200)	ADA	0.184818	1.42E-08
B63	B195	WBRSR(mPas_200)	Na	-0.1775	5.23E-08
B64	B124	WBRSR(mPas_5)	ACTH	0.279773	0
B64	B125	WBRSR(mPas_5)	Cor	0.279773	0
B64	B183	WBRSR(mPas_5)	PTA	-0.26715	0
B64	B65	WBRSR(mPas_5)	WBRSR(mPas_1)	0.959934	0
B64	B66	WBRSR(mPas_5)	WBRSR(mPas_100)	0.864088	0
B64	B67	WBRSR(mPas_5)	WBRSR(mPas_50)	0.905204	0
B64	B182	WBRSR(mPas_5)	TT	0.245363	3.44E-14
B64	B186	WBRSR(mPas_5)	PFC	-0.23599	3.28E-13
B64	B171	WBRSR(mPas_5)	EC	0.210717	8.97E-11
B64	B92	WBRSR(mPas_5)	ALDH2-Glu504Glu	0.199972	7.96E-10
B64	B93	WBRSR(mPas_5)	ALDH2-Glu504Lys	-0.19997	7.96E-10
B64	B94	WBRSR(mPas_5)	ALDH2-Lys504Lys	-0.19997	7.96E-10
B64	B197	WBRSR(mPas_5)	AG	0.198004	1.17E-09
B64	B139	WBRSR(mPas_5)	ADA	0.183668	1.74E-08
B64	B135	WBRSR(mPas_5)	TBA	0.18241	2.19E-08
B64	B158	WBRSR(mPas_5)	IgM	0.168146	2.58E-07
B64	B194	WBRSR(mPas_5)	Cl	-0.16106	8.14E-07
B65	B66	WBRSR(mPas_1)	WBRSR(mPas_100)	0.696918	0
B65	B67	WBRSR(mPas_1)	WBRSR(mPas_50)	0.757138	0
B65	B183	WBRSR(mPas_1)	PTA	-0.23898	1.61E-13
B65	B182	WBRSR(mPas_1)	TT	0.226011	3.26E-12
B65	B186	WBRSR(mPas_1)	PFC	-0.22542	3.72E-12
B65	B124	WBRSR(mPas_1)	ACTH	0.224958	4.13E-12
B65	B125	WBRSR(mPas_1)	Cor	0.224958	4.13E-12
B65	B171	WBRSR(mPas_1)	EC	0.217537	2.11E-11
B65	B197	WBRSR(mPas_1)	AG	0.200031	7.87E-10
B65	B200	WBRSR(mPas_1)	OP	0.180409	3.13E-08
B65	B92	WBRSR(mPas_1)	ALDH2-Glu504Glu	0.161093	8.10E-07
B65	B93	WBRSR(mPas_1)	ALDH2-Glu504Lys	-0.16109	8.10E-07
B65	B94	WBRSR(mPas_1)	ALDH2-Lys504Lys	-0.16109	8.10E-07
B66	B124	WBRSR(mPas_100)	ACTH	0.331523	0
B66	B125	WBRSR(mPas_100)	Cor	0.331523	0
B66	B183	WBRSR(mPas_100)	PTA	-0.27237	0
B66	B194	WBRSR(mPas_100)	Cl	-0.28857	0
B66	B67	WBRSR(mPas_100)	WBRSR(mPas_50)	0.995929	0
B66	B182	WBRSR(mPas_100)	TT	0.238002	2.04E-13

B66	B92	WBRSR(mPas_100)	ALDH2-Glu504Glu	0.236138	3.17E-13
B66	B93	WBRSR(mPas_100)	ALDH2-Glu504Lys	-0.23614	3.17E-13
B66	B94	WBRSR(mPas_100)	ALDH2-Lys504Lys	-0.23614	3.17E-13
B66	B139	WBRSR(mPas_100)	ADA	0.219261	1.45E-11
B66	B186	WBRSR(mPas_100)	PFC	-0.2151	3.56E-11
B66	B135	WBRSR(mPas_100)	TBA	0.214794	3.80E-11
B66	B137	WBRSR(mPas_100)	AFU	0.213345	5.16E-11
B66	B195	WBRSR(mPas_100)	Na	-0.19773	1.24E-09
B66	B107	WBRSR(mPas_100)	AnuA	-0.17391	9.74E-08
B66	B110	WBRSR(mPas_100)	AHA	-0.17391	9.74E-08
B66	B79	WBRSR(mPas_100)	hb V -dna	-0.16941	2.09E-07
B66	B100	WBRSR(mPas_100)	anti-Ro52	-0.16195	7.06E-07
B66	B101	WBRSR(mPas_100)	anti-Sc70	-0.16195	7.06E-07
B66	B102	WBRSR(mPas_100)	anti-Sm	-0.16195	7.06E-07
B66	B103	WBRSR(mPas_100)	anti-SSA/Ro	-0.16195	7.06E-07
B66	B104	WBRSR(mPas_100)	anti-SSB	-0.16195	7.06E-07
B66	B105	WBRSR(mPas_100)	anti-U1RNP	-0.16195	7.06E-07
B66	B106	WBRSR(mPas_100)	anti-P	-0.16195	7.06E-07
B66	B108	WBRSR(mPas_100)	Anti-Cyclin	-0.16195	7.06E-07
B66	B109	WBRSR(mPas_100)	AMA-M2	-0.16195	7.06E-07
B66	B97	WBRSR(mPas_100)	dsDNA	-0.16195	7.06E-07
B66	B98	WBRSR(mPas_100)	anti-Jo-1	-0.16195	7.06E-07
B66	B99	WBRSR(mPas_100)	anti-PM-Scl	-0.16195	7.06E-07
B66	B171	WBRSR(mPas_100)	EC	0.161569	7.51E-07
B67	B124	WBRSR(mPas_50)	ACTH	0.32942	0
B67	B125	WBRSR(mPas_50)	Cor	0.32942	0
B67	B183	WBRSR(mPas_50)	PTA	-0.27743	0
B67	B194	WBRSR(mPas_50)	Cl	-0.2746	0
B67	B182	WBRSR(mPas_50)	TT	0.244308	4.44E-14
B67	B92	WBRSR(mPas_50)	ALDH2-Glu504Glu	0.234699	4.44E-13
B67	B93	WBRSR(mPas_50)	ALDH2-Glu504Lys	-0.2347	4.44E-13
B67	B94	WBRSR(mPas_50)	ALDH2-Lys504Lys	-0.2347	4.44E-13
B67	B186	WBRSR(mPas_50)	PFC	-0.22244	7.22E-12
B67	B139	WBRSR(mPas_50)	ADA	0.219542	1.37E-11
B67	B135	WBRSR(mPas_50)	TBA	0.212166	6.62E-11
B67	B137	WBRSR(mPas_50)	AFU	0.208874	1.32E-10
B67	B171	WBRSR(mPas_50)	EC	0.174182	9.30E-08
B67	B107	WBRSR(mPas_50)	AnuA	-0.17231	1.28E-07
B67	B110	WBRSR(mPas_50)	AHA	-0.17231	1.28E-07
B67	B195	WBRSR(mPas_50)	Na	-0.16918	2.17E-07
B67	B79	WBRSR(mPas_50)	hb V -dna	-0.16846	2.45E-07
B67	B197	WBRSR(mPas_50)	AG	0.167923	2.67E-07
B67	B100	WBRSR(mPas_50)	anti-Ro52	-0.1605	8.90E-07
B67	B101	WBRSR(mPas_50)	anti-Sc70	-0.1605	8.90E-07

B67	B102	WBRSR(mPas_50)	anti-Sm	-0.1605	8.90E-07
B67	B103	WBRSR(mPas_50)	anti-SSA/Ro	-0.1605	8.90E-07
B67	B104	WBRSR(mPas_50)	anti-SSB	-0.1605	8.90E-07
B67	B105	WBRSR(mPas_50)	anti-U1RNP	-0.1605	8.90E-07
B67	B106	WBRSR(mPas_50)	anti-P	-0.1605	8.90E-07
B67	B108	WBRSR(mPas_50)	Anti-Cyclin	-0.1605	8.90E-07
B67	B109	WBRSR(mPas_50)	AMA-M2	-0.1605	8.90E-07
B67	B97	WBRSR(mPas_50)	dsDNA	-0.1605	8.90E-07
B67	B98	WBRSR(mPas_50)	anti-Jo-1	-0.1605	8.90E-07
B67	B99	WBRSR(mPas_50)	anti-PM-Scl	-0.1605	8.90E-07
B69	B137	PV	AFU	0.243401	5.55E-14
B69	B195	PV	Na	-0.20385	3.67E-10
B69	B139	PV	ADA	0.188479	7.21E-09
B69	B138	PV	PAB	-0.18451	1.50E-08
B69	B141	PV	AST/ALT	0.162561	6.41E-07
B7	B137	WB-Cop	AFU	-0.31359	0
B7	B139	WB-Cop	ADA	-0.44985	0
B7	B15	WB-Cop	CD3-CD19+(B cell)	0.444867	0
B7	B157	WB-Cop	IgG	-0.37859	0
B7	B16	WB-Cop	CD3+T	0.298987	0
B7	B18	WB-Cop	CD3+CD4+helper(inducer) T	0.385709	0
B7	B23	WB-Cop	CD3-CD16+CD56+(NK)	-0.33846	0
B7	B8	WB-Cop	WB-Zinc	-1	0
B7	B19	WB-Cop	CD3+CD4+/CD3+CD8+	0.228677	1.78E-12
B7	B54	WB-Cop	ESR	-0.2235	5.71E-12
B7	B59	WB-Cop	WBRV	-0.21355	4.94E-11
B7	B198	WB-Cop	P	-0.20696	1.95E-10
B7	B85	WB-Cop	SCC	0.196611	1.54E-09
B7	B69	WB-Cop	PV	-0.17633	6.42E-08
B7	B57	WB-Cop	RBCAI	-0.16653	3.36E-07
B70	B77	LDL-C	TC	0.900823	0
B70	B76	LDL-C	ApoB	0.190481	4.96E-09
B70	B127	LDL-C	A-G ratio	-0.16998	1.90E-07
B71	B153	TG	Glu	0.32208	0
B71	B181	TG	APTT	0.308366	0
B71	B185	TG	PT	0.271857	0
B71	B72	TG	HDL-C	-0.36076	0
B71	B77	TG	TC	0.284601	0
B71	B91	TG	TP	0.288373	0
B71	B184	TG	INR	0.265547	2.22E-16
B71	B130	TG	GLB	0.25178	7.11E-15
B71	B182	TG	TT	0.21153	7.57E-11
B71	B183	TG	PTA	0.193574	2.76E-09

B71	B127	TG	A-G ratio	-0.17168	1.43E-07
B71	B207	TG	WHR	0.163305	5.68E-07
B71	B192	TG	UA	0.161238	7.91E-07
B72	B216	HDL-C	BMI	-0.27778	0
B72	B222	HDL-C	NS	-0.27838	0
B72	B77	HDL-C	TC	0.295111	0
B72	B206	HDL-C	Waist	-0.2228	6.68E-12
B72	B192	HDL-C	UA	-0.22272	6.79E-12
B72	B74	HDL-C	ApoA1	0.209125	1.25E-10
B72	B221	HDL-C	Hei	-0.20119	6.25E-10
B72	B215	HDL-C	BMI	-0.19764	1.26E-09
B72	B153	HDL-C	Glu	-0.19337	2.87E-09
B72	B207	HDL-C	WHR	-0.1795	3.68E-08
B72	B163	HDL-C	SG	-0.16262	6.35E-07
B72	B141	HDL-C	AST/ALT	0.1604	9.04E-07
B73	B178	ApoA	Ig-U	0.253552	4.44E-15
B73	B179	ApoA	α1-MG	0.234896	4.24E-13
B73	B76	ApoA	ApoB	-0.18249	2.16E-08
B74	B136	ApoA1	5'NT	0.360936	0
B74	B139	ApoA1	ADA	-0.35519	0
B74	B75	ApoA1	ApoA1/B	0.401407	0
B74	B76	ApoA1	ApoB	0.284241	0
B74	B199	ApoA1	Mg	0.235101	4.04E-13
B74	B186	ApoA1	PFC	-0.2128	5.80E-11
B74	B88	ApoA1	CA 242	0.207493	1.75E-10
B74	B182	ApoA1	TT	0.177851	4.92E-08
B74	B195	ApoA1	Na	0.177694	5.06E-08
B74	B193	ApoA1	K	0.160466	8.94E-07
B75	B136	ApoA1/B	5'NT	0.357197	0
B75	B76	ApoA1/B	ApoB	-0.70347	0
B75	B198	ApoA1/B	P	-0.26545	2.22E-16
B75	B134	ApoA1/B	CHE	-0.25022	1.02E-14
B75	B138	ApoA1/B	PAB	-0.22496	4.13E-12
B75	B137	ApoA1/B	AFU	0.201134	6.32E-10
B75	B135	ApoA1/B	TBA	-0.19351	2.80E-09
B75	B172	ApoA1/B	KB	-0.16914	2.18E-07
B75	B193	ApoA1/B	K	0.167982	2.65E-07
B75	B96	ApoA1/B	dsDNA (IIF)	0.162104	6.89E-07
B76	B138	ApoB	PAB	0.349614	0
B76	B139	ApoB	ADA	-0.31816	0
B76	B96	ApoB	dsDNA (IIF)	-0.27953	0
B76	B134	ApoB	CHE	0.229739	1.40E-12
B76	B77	ApoB	TC	0.211754	7.22E-11
B76	B172	ApoB	KB	0.181503	2.58E-08

B76	B137	ApoB	AFU	-0.1791	3.95E-08
B76	B92	ApoB	ALDH2-Glu504Glu	0.177725	5.03E-08
B76	B93	ApoB	ALDH2-Glu504Lys	-0.17773	5.03E-08
B76	B94	ApoB	ALDH2-Lys504Lys	-0.17773	5.03E-08
B76	B86	ApoB	TBhCG	0.176758	5.96E-08
B77	B127	TC	A-G ratio	-0.18553	1.24E-08
B77	B130	TC	GLB	0.183532	1.79E-08
B77	B140	TC	r-GT	0.182566	2.13E-08
B79	B100	hb V -dna	anti-Ro52	0.374482	0
B79	B101	hb V -dna	anti-Sc70	0.374482	0
B79	B102	hb V -dna	anti-Sm	0.374482	0
B79	B103	hb V -dna	anti-SSA/Ro	0.374482	0
B79	B104	hb V -dna	anti-SSB	0.374482	0
B79	B105	hb V -dna	anti-U1RNP	0.374482	0
B79	B106	hb V -dna	anti-P	0.374482	0
B79	B107	hb V -dna	AnuA	0.405152	0
B79	B108	hb V -dna	Anti-Cyclin	0.374482	0
B79	B109	hb V -dna	AMA-M2	0.374482	0
B79	B110	hb V -dna	AHA	0.405152	0
B79	B119	hb V -dna	Hcv-rna	0.286021	0
B79	B121	hb V -dna	C3	-0.28602	0
B79	B122	hb V -dna	C4	-0.28602	0
B79	B124	hb V -dna	ACTH	-0.49919	0
B79	B125	hb V -dna	Cor	-0.49919	0
B79	B139	hb V -dna	ADA	-0.42128	0
B79	B151	hb V -dna	Anti-CCP	-0.27421	0
B79	B152	hb V -dna	ACA-Ig(G-A-M)	-0.286	0
B79	B159	hb V -dna	β2-MG	-0.28602	0
B79	B169	hb V -dna	U-CRY	-0.28602	0
B79	B180	hb V -dna	Tf	0.32558	0
B79	B194	hb V -dna	Cl	0.397401	0
B79	B92	hb V -dna	ALDH2-Glu504Glu	-0.35164	0
B79	B93	hb V -dna	ALDH2-Glu504Lys	0.351642	0
B79	B94	hb V -dna	ALDH2-Lys504Lys	0.351642	0
B79	B97	hb V -dna	dsDNA	0.374482	0
B79	B98	hb V -dna	anti-Jo-1	0.374482	0
B79	B99	hb V -dna	anti-PM-Scl	0.374482	0
B79	B135	hb V -dna	TBA	-0.25305	4.88E-15
B79	B195	hb V -dna	Na	0.249779	1.15E-14
B79	B184	hb V -dna	INR	-0.21576	3.09E-11
B79	B199	hb V -dna	Mg	-0.2006	7.03E-10
B79	B137	hb V -dna	AFU	-0.19993	8.03E-10
B79	B157	hb V -dna	IgG	-0.17002	1.89E-07
B79	B158	hb V -dna	IgM	-0.16938	2.10E-07

B79	B196	hb V -dna	TCO2	-0.16257	6.40E-07
B8	B137	WB-Zinc	AFU	0.313586	0
B8	B139	WB-Zinc	ADA	0.449853	0
B8	B15	WB-Zinc	CD3-CD19+(B cell)	-0.44487	0
B8	B157	WB-Zinc	IgG	0.378593	0
B8	B16	WB-Zinc	CD3+T	-0.29899	0
B8	B18	WB-Zinc	CD3+CD4+helper(inducer) T	-0.38571	0
B8	B23	WB-Zinc	CD3-CD16+CD56+(NK)	0.338457	0
B8	B19	WB-Zinc	CD3+CD4+/CD3+CD8+	-0.22868	1.78E-12
B8	B54	WB-Zinc	ESR	0.223503	5.71E-12
B8	B59	WB-Zinc	WBRV	0.213551	4.94E-11
B8	B198	WB-Zinc	P	0.206965	1.95E-10
B8	B85	WB-Zinc	SCC	-0.19661	1.54E-09
B8	B69	WB-Zinc	PV	0.176326	6.42E-08
B8	B57	WB-Zinc	RBCAI	0.166529	3.36E-07
B82	B85	CEA	SCC	0.289879	0
B82	B86	CEA	TBhCG	-0.47109	0
B82	B87	CEA	NSE	0.394631	0
B82	B88	CEA	CA 242	0.243003	6.13E-14
B82	B89	CEA	CA 72-4	0.234519	4.63E-13
B82	B84	CEA	C-PSA	0.210717	8.97E-11
B82	B83	CEA	AFP	0.191743	3.91E-09
B83	B85	AFP	SCC	0.282203	0
B83	B89	AFP	CA 72-4	0.263046	4.44E-16
B84	B85	C-PSA	SCC	0.42895	0
B84	B86	C-PSA	TBhCG	-0.23501	4.13E-13
B84	B117	C-PSA	TT4	-0.20277	4.56E-10
B84	B116	C-PSA	TT3	0.18219	2.28E-08
B85	B117	SCC	TT4	-0.1881	7.74E-09
B85	B87	SCC	NSE	0.185814	1.18E-08
B85	B116	SCC	TT3	0.178047	4.75E-08
B86	B87	TBhCG	NSE	-0.27091	0
B86	B139	TBhCG	ADA	-0.21724	2.25E-11
B86	B198	TBhCG	P	-0.20754	1.73E-10
B86	B199	TBhCG	Mg	0.172344	1.27E-07
B86	B200	TBhCG	OP	-0.16445	4.72E-07
B86	B88	TBhCG	CA 242	-0.16249	6.47E-07
B87	B88	NSE	CA 242	0.202657	4.66E-10
B87	B89	NSE	CA 72-4	0.18747	8.70E-09
B88	B197	CA 242	AG	0.283448	0
B88	B198	CA 242	P	0.393063	0
B88	B200	CA 242	OP	0.283328	0
B88	B195	CA 242	Na	0.226365	3.01E-12

B88	B135	CA 242	TBA	0.205991	2.38E-10
B90	B154	CYFRA 21-1	RF	-0.1775	5.24E-08
B90	B197	CYFRA 21-1	AG	0.16327	5.72E-07
B91	B126	TP	Alb	0.474847	0
B91	B127	TP	A-G ratio	-0.45969	0
B91	B130	TP	GLB	0.771438	0
B91	B188	TP	Ca	0.319268	0
B92	B100	ALDH2-Glu504Glu	anti-Ro52	-0.53308	0
B92	B101	ALDH2-Glu504Glu	anti-Sc70	-0.53308	0
B92	B102	ALDH2-Glu504Glu	anti-Sm	-0.53308	0
B92	B103	ALDH2-Glu504Glu	anti-SSA/Ro	-0.53308	0
B92	B104	ALDH2-Glu504Glu	anti-SSB	-0.53308	0
B92	B105	ALDH2-Glu504Glu	anti-U1RNP	-0.53308	0
B92	B106	ALDH2-Glu504Glu	anti-P	-0.53308	0
B92	B107	ALDH2-Glu504Glu	AnuA	-0.5761	0
B92	B108	ALDH2-Glu504Glu	Anti-Cyclin	-0.53308	0
B92	B109	ALDH2-Glu504Glu	AMA-M2	-0.53308	0
B92	B110	ALDH2-Glu504Glu	AHA	-0.5761	0
B92	B119	ALDH2-Glu504Glu	Hcv-rna	-0.4067	0
B92	B120	ALDH2-Glu504Glu	HCV-cAg	-0.44528	0
B92	B121	ALDH2-Glu504Glu	C3	0.406705	0
B92	B122	ALDH2-Glu504Glu	C4	0.406704	0
B92	B124	ALDH2-Glu504Glu	ACTH	0.706725	0
B92	B125	ALDH2-Glu504Glu	Cor	0.706725	0
B92	B135	ALDH2-Glu504Glu	TBA	0.49474	0
B92	B139	ALDH2-Glu504Glu	ADA	0.371758	0
B92	B151	ALDH2-Glu504Glu	Anti-CCP	0.383911	0
B92	B152	ALDH2-Glu504Glu	ACA-Ig(G-A-M)	0.406681	0
B92	B157	ALDH2-Glu504Glu	IgG	0.28515	0
B92	B159	ALDH2-Glu504Glu	β2-MG	0.406704	0
B92	B169	ALDH2-Glu504Glu	U-CRY	0.406703	0
B92	B180	ALDH2-Glu504Glu	Tf	-0.40795	0
B92	B184	ALDH2-Glu504Glu	INR	0.307844	0
B92	B193	ALDH2-Glu504Glu	K	-0.34169	0
B92	B194	ALDH2-Glu504Glu	Cl	-0.43843	0
B92	B198	ALDH2-Glu504Glu	P	0.2696	0
B92	B93	ALDH2-Glu504Glu	ALDH2-Glu504Lys	-1	0
B92	B94	ALDH2-Glu504Glu	ALDH2-Lys504Lys	-1	0
B92	B96	ALDH2-Glu504Glu	dsDNA (IIF)	-0.70673	0
B92	B97	ALDH2-Glu504Glu	dsDNA	-0.53308	0
B92	B98	ALDH2-Glu504Glu	anti-Jo-1	-0.53308	0
B92	B99	ALDH2-Glu504Glu	anti-PM-Scl	-0.53308	0
B92	B156	ALDH2-Glu504Glu	IgE	-0.23924	1.52E-13
B92	B196	ALDH2-Glu504Glu	TCO2	0.229601	1.44E-12

B92	B114	ALDH2-Glu504Glu	TSH TT3 TT4(TT4)	0.228984	1.66E-12
B92	B155	ALDH2-Glu504Glu	IgA	0.226636	2.83E-12
B92	B185	ALDH2-Glu504Glu	PT	0.214486	4.05E-11
B92	B195	ALDH2-Glu504Glu	Na	-0.20422	3.41E-10
B92	B183	ALDH2-Glu504Glu	PTA	-0.18799	7.89E-09
B92	B154	ALDH2-Glu504Glu	RF	-0.16625	3.52E-07
B93	B100	ALDH2-Glu504Lys	anti-Ro52	0.533077	0
B93	B101	ALDH2-Glu504Lys	anti-Sc70	0.533077	0
B93	B102	ALDH2-Glu504Lys	anti-Sm	0.533077	0
B93	B103	ALDH2-Glu504Lys	anti-SSA/Ro	0.533077	0
B93	B104	ALDH2-Glu504Lys	anti-SSB	0.533077	0
B93	B105	ALDH2-Glu504Lys	anti-U1RNP	0.533077	0
B93	B106	ALDH2-Glu504Lys	anti-P	0.533077	0
B93	B107	ALDH2-Glu504Lys	AnuA	0.576102	0
B93	B108	ALDH2-Glu504Lys	Anti-Cyclin	0.533077	0
B93	B109	ALDH2-Glu504Lys	AMA-M2	0.533077	0
B93	B110	ALDH2-Glu504Lys	AHA	0.576102	0
B93	B119	ALDH2-Glu504Lys	Hcv-rna	0.406704	0
B93	B120	ALDH2-Glu504Lys	HCV-cAg	0.445278	0
B93	B121	ALDH2-Glu504Lys	C3	-0.4067	0
B93	B122	ALDH2-Glu504Lys	C4	-0.4067	0
B93	B124	ALDH2-Glu504Lys	ACTH	-0.70673	0
B93	B125	ALDH2-Glu504Lys	Cor	-0.70673	0
B93	B135	ALDH2-Glu504Lys	TBA	-0.49474	0
B93	B139	ALDH2-Glu504Lys	ADA	-0.37176	0
B93	B151	ALDH2-Glu504Lys	Anti-CCP	-0.38391	0
B93	B152	ALDH2-Glu504Lys	ACA-Ig(G-A-M)	-0.40668	0
B93	B157	ALDH2-Glu504Lys	IgG	-0.28515	0
B93	B159	ALDH2-Glu504Lys	β2-MG	-0.4067	0
B93	B169	ALDH2-Glu504Lys	U-CRY	-0.4067	0
B93	B180	ALDH2-Glu504Lys	Tf	0.407948	0
B93	B184	ALDH2-Glu504Lys	INR	-0.30784	0
B93	B193	ALDH2-Glu504Lys	K	0.341692	0
B93	B194	ALDH2-Glu504Lys	Cl	0.438426	0
B93	B198	ALDH2-Glu504Lys	P	-0.2696	0
B93	B94	ALDH2-Glu504Lys	ALDH2-Lys504Lys	1	0
B93	B96	ALDH2-Glu504Lys	dsDNA (IIF)	0.706725	0
B93	B97	ALDH2-Glu504Lys	dsDNA	0.533077	0
B93	B98	ALDH2-Glu504Lys	anti-Jo-1	0.533077	0
B93	B99	ALDH2-Glu504Lys	anti-PM-Scl	0.533077	0
B93	B156	ALDH2-Glu504Lys	IgE	0.23924	1.52E-13
B93	B196	ALDH2-Glu504Lys	TCO2	-0.2296	1.44E-12
B93	B114	ALDH2-Glu504Lys	TSH TT3 TT4(TT4)	-0.22898	1.66E-12
B93	B155	ALDH2-Glu504Lys	IgA	-0.22664	2.83E-12

B93	B185	ALDH2-Glu504Lys	PT	-0.21449	4.05E-11
B93	B195	ALDH2-Glu504Lys	Na	0.204215	3.41E-10
B93	B183	ALDH2-Glu504Lys	PTA	0.187995	7.89E-09
B93	B154	ALDH2-Glu504Lys	RF	0.16625	3.52E-07
B94	B100	ALDH2-Lys504Lys	anti-Ro52	0.533077	0
B94	B101	ALDH2-Lys504Lys	anti-Sc70	0.533077	0
B94	B102	ALDH2-Lys504Lys	anti-Sm	0.533077	0
B94	B103	ALDH2-Lys504Lys	anti-SSA/Ro	0.533077	0
B94	B104	ALDH2-Lys504Lys	anti-SSB	0.533077	0
B94	B105	ALDH2-Lys504Lys	anti-U1RNP	0.533077	0
B94	B106	ALDH2-Lys504Lys	anti-P	0.533077	0
B94	B107	ALDH2-Lys504Lys	AnuA	0.576102	0
B94	B108	ALDH2-Lys504Lys	Anti-Cyclin	0.533077	0
B94	B109	ALDH2-Lys504Lys	AMA-M2	0.533077	0
B94	B110	ALDH2-Lys504Lys	AHA	0.576102	0
B94	B119	ALDH2-Lys504Lys	Hcv-rna	0.406704	0
B94	B120	ALDH2-Lys504Lys	HCV-cAg	0.445278	0
B94	B121	ALDH2-Lys504Lys	C3	-0.4067	0
B94	B122	ALDH2-Lys504Lys	C4	-0.4067	0
B94	B124	ALDH2-Lys504Lys	ACTH	-0.70673	0
B94	B125	ALDH2-Lys504Lys	Cor	-0.70673	0
B94	B135	ALDH2-Lys504Lys	TBA	-0.49474	0
B94	B139	ALDH2-Lys504Lys	ADA	-0.37176	0
B94	B151	ALDH2-Lys504Lys	Anti-CCP	-0.38391	0
B94	B152	ALDH2-Lys504Lys	ACA-Ig(G-A-M)	-0.40668	0
B94	B157	ALDH2-Lys504Lys	IgG	-0.28515	0
B94	B159	ALDH2-Lys504Lys	β2-MG	-0.4067	0
B94	B169	ALDH2-Lys504Lys	U-CRY	-0.4067	0
B94	B180	ALDH2-Lys504Lys	Tf	0.407948	0
B94	B184	ALDH2-Lys504Lys	INR	-0.30784	0
B94	B193	ALDH2-Lys504Lys	K	0.341692	0
B94	B194	ALDH2-Lys504Lys	Cl	0.438426	0
B94	B198	ALDH2-Lys504Lys	P	-0.2696	0
B94	B96	ALDH2-Lys504Lys	dsDNA (IIF)	0.706725	0
B94	B97	ALDH2-Lys504Lys	dsDNA	0.533077	0
B94	B98	ALDH2-Lys504Lys	anti-Jo-1	0.533077	0
B94	B99	ALDH2-Lys504Lys	anti-PM-Scl	0.533077	0
B94	B156	ALDH2-Lys504Lys	IgE	0.23924	1.52E-13
B94	B196	ALDH2-Lys504Lys	TCO2	-0.2296	1.44E-12
B94	B114	ALDH2-Lys504Lys	TSH TT3 TT4(TT4)	-0.22898	1.66E-12
B94	B155	ALDH2-Lys504Lys	IgA	-0.22664	2.83E-12
B94	B185	ALDH2-Lys504Lys	PT	-0.21449	4.05E-11
B94	B195	ALDH2-Lys504Lys	Na	0.204215	3.41E-10
B94	B183	ALDH2-Lys504Lys	PTA	0.187995	7.89E-09

B94	B154	ALDH2-Lys504Lys	RF	0.16625	3.52E-07
B95	B100	anti-cenpb	anti-Ro52	-0.34746	0
B95	B101	anti-cenpb	anti-Sc70	-0.34746	0
B95	B102	anti-cenpb	anti-Sm	-0.34746	0
B95	B103	anti-cenpb	anti-SSA/Ro	-0.34746	0
B95	B104	anti-cenpb	anti-SSB	-0.34746	0
B95	B105	anti-cenpb	anti-U1RNP	-0.34746	0
B95	B106	anti-cenpb	anti-P	-0.34746	0
B95	B108	anti-cenpb	Anti-Cyclin	-0.34746	0
B95	B109	anti-cenpb	AMA-M2	-0.34746	0
B95	B157	anti-cenpb	IgG	0.380048	0
B95	B193	anti-cenpb	K	-0.36175	0
B95	B97	anti-cenpb	dsDNA	-0.34746	0
B95	B98	anti-cenpb	anti-Jo-1	-0.34746	0
B95	B99	anti-cenpb	anti-PM-Scl	-0.34746	0
B95	B156	anti-cenpb	IgE	-0.2204	1.13E-11
B95	B114	anti-cenpb	TSH TT3 TT4(TT4)	0.209411	1.18E-10
B95	B199	anti-cenpb	Mg	-0.19517	2.03E-09
B95	B139	anti-cenpb	ADA	0.178886	4.10E-08
B95	B115	anti-cenpb	TSH TT3 TT4(TT3)	0.16711	3.06E-07
B96	B100	dsDNA (IIF)	anti-Ro52	0.376739	0
B96	B101	dsDNA (IIF)	anti-Sc70	0.376739	0
B96	B102	dsDNA (IIF)	anti-Sm	0.376739	0
B96	B103	dsDNA (IIF)	anti-SSA/Ro	0.376739	0
B96	B104	dsDNA (IIF)	anti-SSB	0.376739	0
B96	B105	dsDNA (IIF)	anti-U1RNP	0.376739	0
B96	B106	dsDNA (IIF)	anti-P	0.376739	0
B96	B107	dsDNA (IIF)	AnuA	0.407146	0
B96	B108	dsDNA (IIF)	Anti-Cyclin	0.376739	0
B96	B109	dsDNA (IIF)	AMA-M2	0.376739	0
B96	B110	dsDNA (IIF)	AHA	0.407146	0
B96	B120	dsDNA (IIF)	HCV-cAg	0.314689	0
B96	B138	dsDNA (IIF)	PAB	-0.33578	0
B96	B97	dsDNA (IIF)	dsDNA	0.376739	0
B96	B98	dsDNA (IIF)	anti-Jo-1	0.376739	0
B96	B99	dsDNA (IIF)	anti-PM-Scl	0.376739	0
B96	B180	dsDNA (IIF)	Tf	0.238636	1.75E-13
B96	B196	dsDNA (IIF)	TCO2	-0.22539	3.75E-12
B96	B156	dsDNA (IIF)	IgE	0.221822	8.28E-12
B96	B198	dsDNA (IIF)	P	-0.21792	1.94E-11
B96	B137	dsDNA (IIF)	AFU	-0.18171	2.48E-08
B96	B194	dsDNA (IIF)	Cl	0.178708	4.23E-08
B97	B100	dsDNA	anti-Ro52	1	0
B97	B102	dsDNA	anti-Sm	1	0

B97	B103	dsDNA	anti-SSA/Ro	1	0
B97	B104	dsDNA	anti-SSB	1	0
B97	B105	dsDNA	anti-U1RNP	1	0
B97	B106	dsDNA	anti-P	1	0
B97	B107	dsDNA	AnuA	0.925318	0
B97	B108	dsDNA	Anti-Cyclin	1	0
B97	B109	dsDNA	AMA-M2	1	0
B97	B110	dsDNA	AHA	0.925318	0
B97	B119	dsDNA	Hcv-rna	0.433836	0
B97	B120	dsDNA	HCV-cAg	0.352785	0
B97	B121	dsDNA	C3	-0.43384	0
B97	B122	dsDNA	C4	-0.43384	0
B97	B124	dsDNA	ACTH	-0.37674	0
B97	B125	dsDNA	Cor	-0.37674	0
B97	B139	dsDNA	ADA	-0.45548	0
B97	B152	dsDNA	ACA-Ig(G-A-M)	-0.43381	0
B97	B157	dsDNA	IgG	-0.33455	0
B97	B159	dsDNA	β2-MG	-0.43384	0
B97	B169	dsDNA	U-CRY	-0.43383	0
B97	B180	dsDNA	Tf	0.440213	0
B97	B193	dsDNA	K	0.3357	0
B97	B194	dsDNA	Cl	0.354015	0
B97	B99	dsDNA	anti-PM-Scl	1	0
B97	B155	dsDNA	IgA	-0.24381	5.02E-14
B97	B137	dsDNA	AFU	-0.24337	5.60E-14
B97	B154	dsDNA	RF	0.242501	6.93E-14
B97	B195	dsDNA	Na	0.219271	1.45E-11
B97	B184	dsDNA	INR	-0.20636	2.21E-10
B97	B183	dsDNA	PTA	0.202624	4.70E-10
B97	B178	dsDNA	Ig-U	0.196769	1.49E-09
B97	B135	dsDNA	TBA	-0.18726	9.04E-09
B97	B196	dsDNA	TCO2	-0.17771	5.04E-08
B98	B101	anti-Jo-1	anti-Sc70	1	0
B98	B102	anti-Jo-1	anti-Sm	1	0
B98	B107	anti-Jo-1	AnuA	0.925317	0
B98	B108	anti-Jo-1	Anti-Cyclin	1	0
B98	B110	anti-Jo-1	AHA	0.925318	0
B98	B119	anti-Jo-1	Hcv-rna	0.433836	0
B98	B120	anti-Jo-1	HCV-cAg	0.352785	0
B98	B121	anti-Jo-1	C3	-0.43384	0
B98	B122	anti-Jo-1	C4	-0.43384	0
B98	B124	anti-Jo-1	ACTH	-0.37674	0
B98	B125	anti-Jo-1	Cor	-0.37674	0
B98	B139	anti-Jo-1	ADA	-0.45548	0

B98	B152	anti-Jo-1	ACA-Ig(G-A-M)	-0.43381	0
B98	B157	anti-Jo-1	IgG	-0.33455	0
B98	B159	anti-Jo-1	β2-MG	-0.43384	0
B98	B169	anti-Jo-1	U-CRY	-0.43383	0
B98	B180	anti-Jo-1	Tf	0.440213	0
B98	B193	anti-Jo-1	K	0.3357	0
B98	B194	anti-Jo-1	Cl	0.354015	0
B98	B99	anti-Jo-1	anti-PM-Scl	1	0
B98	B155	anti-Jo-1	IgA	-0.24381	5.02E-14
B98	B137	anti-Jo-1	AFU	-0.24337	5.60E-14
B98	B154	anti-Jo-1	RF	0.242501	6.93E-14
B98	B195	anti-Jo-1	Na	0.219271	1.45E-11
B98	B184	anti-Jo-1	INR	-0.20636	2.21E-10
B98	B183	anti-Jo-1	PTA	0.202624	4.70E-10
B98	B178	anti-Jo-1	Ig-U	0.196769	1.49E-09
B98	B135	anti-Jo-1	TBA	-0.18726	9.04E-09
B98	B196	anti-Jo-1	TCO2	-0.17771	5.04E-08
B99	B101	anti-PM-Scl	anti-Sc70	1	0
B99	B104	anti-PM-Scl	anti-SSB	1	0
B99	B106	anti-PM-Scl	anti-P	1	0
B99	B107	anti-PM-Scl	AnuA	0.925318	0
B99	B110	anti-PM-Scl	AHA	0.925318	0
B99	B119	anti-PM-Scl	Hcv-rna	0.433836	0
B99	B120	anti-PM-Scl	HCV-cAg	0.352785	0
B99	B121	anti-PM-Scl	C3	-0.43384	0
B99	B122	anti-PM-Scl	C4	-0.43384	0
B99	B124	anti-PM-Scl	ACTH	-0.37674	0
B99	B125	anti-PM-Scl	Cor	-0.37674	0
B99	B139	anti-PM-Scl	ADA	-0.45548	0
B99	B152	anti-PM-Scl	ACA-Ig(G-A-M)	-0.43381	0
B99	B157	anti-PM-Scl	IgG	-0.33455	0
B99	B159	anti-PM-Scl	β2-MG	-0.43384	0
B99	B169	anti-PM-Scl	U-CRY	-0.43383	0
B99	B180	anti-PM-Scl	Tf	0.440213	0
B99	B193	anti-PM-Scl	K	0.3357	0
B99	B194	anti-PM-Scl	Cl	0.354015	0
B99	B155	anti-PM-Scl	IgA	-0.24381	5.02E-14
B99	B137	anti-PM-Scl	AFU	-0.24337	5.60E-14
B99	B154	anti-PM-Scl	RF	0.242501	6.93E-14
B99	B195	anti-PM-Scl	Na	0.219271	1.45E-11
B99	B184	anti-PM-Scl	INR	-0.20636	2.21E-10
B99	B183	anti-PM-Scl	PTA	0.202624	4.70E-10
B99	B178	anti-PM-Scl	Ig-U	0.196769	1.49E-09
B99	B135	anti-PM-Scl	TBA	-0.18726	9.04E-09

B99	B196	anti-PM-Scl	TCO2	-0.17771	5.04E-08

Supplementary Table 7. PEI Correlations in Hyperlipidemia

Row	Column	Row	Column	Cor	*P*
B1	B161	Gender	LEU	0.241648	0
B1	B163	Gender	SG	-0.29208	0
B1	B174	Gender	Urea/Cr	0.405881	0
B1	B190	Gender	Cre	-0.48747	0
B1	B192	Gender	UA	-0.45552	0
B1	B204	Gender	Drinking	-0.35298	0
B1	B206	Gender	Waist	-0.44721	0
B1	B207	Gender	WHR	-0.35161	0
B1	B209	Gender	Smoking	-0.36934	0
B1	B210	Gender	Hip	-0.28234	0
B1	B215	Gender	BMI	-0.2678	0
B1	B216	Gender	BMI	-0.57511	0
B1	B221	Gender	Hei	-0.6864	0
B1	B222	Gender	NS	-0.70613	0
B1	B40	Gender	WBC	-0.19843	0
B1	B42	Gender	MON	-0.28678	0
B1	B45	Gender	MCHC	-0.28185	0
B1	B46	Gender	RBC Count	-0.39348	0
B1	B51	Gender	Hb	-0.51389	0
B1	B72	Gender	HDL-C	0.383801	0
B1	B41	Gender	MON％	-0.19516	4.44E-16
B1	B50	Gender	eo%	-0.15026	3.69E-10
B1	B44	Gender	MCH	-0.14784	7.08E-10
B1	B3	Gender	Hcy	-0.14415	1.87E-09
B1	B141	Gender	AST/ALT	0.140733	4.49E-09
B1	B132	Gender	Dbil	-0.13882	7.28E-09
B1	B77	Gender	TC	0.137485	1.01E-08
B1	B128	Gender	ALT	-0.13675	1.22E-08
B1	B201	Gender	e-habits	-0.13149	4.34E-08
B1	B133	Gender	TBIL	-0.13009	6.05E-08
B1	B118	Gender	CEA1	-0.12936	7.17E-08
B1	B173	Gender	NTT	0.122837	3.17E-07
B1	B48	Gender	LYM%	0.119136	7.13E-07
B10	B12	PG I	PG II	0.563497	0
B10	B11	PG I	PGI/PGII	0.12008	5.81E-07
B100	B157	anti-Ro52	IgG	0.206843	0
B100	B151	anti-Ro52	Anti-CCP	0.193813	4.44E-16
B100	B178	anti-Ro52	Ig-U	-0.13784	9.29E-09
B101	B104	anti-Sc70	anti-SSB	1	0
B101	B107	anti-Sc70	AnuA	0.999999	0
B101	B119	anti-Sc70	Hcv-rna	0.384926	0
B101	B120	anti-Sc70	HCV-cAg	0.349051	0

B101	B121	anti-Sc70	C3	-0.27501	0
B101	B122	anti-Sc70	C4	0.242862	0
B101	B125	anti-Sc70	Cor	0.298639	0
B101	B134	anti-Sc70	CHE	-0.23432	0
B101	B151	anti-Sc70	Anti-CCP	0.245648	0
B101	B152	anti-Sc70	ACA-Ig(G-A-M)	-0.38501	0
B101	B156	anti-Sc70	IgE	0.234142	0
B101	B157	anti-Sc70	IgG	0.328845	0
B101	B159	anti-Sc70	β2-MG	-0.38492	0
B101	B138	anti-Sc70	PAB	-0.18058	4.35E-14
B101	B154	anti-Sc70	RF	0.158265	3.99E-11
B101	B178	anti-Sc70	Ig-U	0.151079	2.96E-10
B101	B193	anti-Sc70	K	0.125601	1.70E-07
B101	B182	anti-Sc70	TT	0.124542	2.17E-07
B101	B183	anti-Sc70	PTA	-0.11862	7.97E-07
B102	B103	anti-Sm	anti-SSA/Ro	1	0
B102	B104	anti-Sm	anti-SSB	1	0
B102	B105	anti-Sm	anti-U1RNP	1	0
B102	B106	anti-Sm	anti-P	1	0
B102	B107	anti-Sm	AnuA	0.999999	0
B102	B108	anti-Sm	Anti-Cyclin	1	0
B102	B109	anti-Sm	AMA-M2	1	0
B102	B110	anti-Sm	AHA	1	0
B102	B119	anti-Sm	Hcv-rna	0.384926	0
B102	B120	anti-Sm	HCV-cAg	0.349052	0
B102	B121	anti-Sm	C3	-0.27501	0
B102	B122	anti-Sm	C4	0.242862	0
B102	B125	anti-Sm	Cor	0.298639	0
B102	B134	anti-Sm	CHE	-0.23432	0
B102	B151	anti-Sm	Anti-CCP	0.245648	0
B102	B152	anti-Sm	ACA-Ig(G-A-M)	-0.385	0
B102	B156	anti-Sm	IgE	0.234142	0
B102	B157	anti-Sm	IgG	0.328845	0
B102	B159	anti-Sm	β2-MG	-0.38492	0
B102	B138	anti-Sm	PAB	-0.18058	4.35E-14
B102	B154	anti-Sm	RF	0.158265	3.99E-11
B102	B178	anti-Sm	Ig-U	0.151079	2.96E-10
B102	B193	anti-Sm	K	0.125601	1.70E-07
B102	B182	anti-Sm	TT	0.124542	2.17E-07
B102	B183	anti-Sm	PTA	-0.11862	7.97E-07
B103	B107	anti-SSA/Ro	AnuA	0.999999	0
B103	B119	anti-SSA/Ro	Hcv-rna	0.384926	0
B103	B120	anti-SSA/Ro	HCV-cAg	0.349051	0
B103	B121	anti-SSA/Ro	C3	-0.27501	0

B103	B122	anti-SSA/Ro	C4	0.242862	0
B103	B125	anti-SSA/Ro	Cor	0.298639	0
B103	B134	anti-SSA/Ro	CHE	-0.23432	0
B103	B151	anti-SSA/Ro	Anti-CCP	0.245648	0
B103	B152	anti-SSA/Ro	ACA-Ig(G-A-M)	-0.385	0
B103	B156	anti-SSA/Ro	IgE	0.234142	0
B103	B157	anti-SSA/Ro	IgG	0.328845	0
B103	B159	anti-SSA/Ro	β2-MG	-0.38492	0
B103	B138	anti-SSA/Ro	PAB	-0.18058	4.35E-14
B103	B154	anti-SSA/Ro	RF	0.158265	3.99E-11
B103	B178	anti-SSA/Ro	Ig-U	0.151079	2.96E-10
B103	B193	anti-SSA/Ro	K	0.125601	1.70E-07
B103	B182	anti-SSA/Ro	TT	0.124542	2.17E-07
B103	B183	anti-SSA/Ro	PTA	-0.11862	7.97E-07
B104	B106	anti-SSB	anti-P	1	0
B104	B107	anti-SSB	AnuA	0.999999	0
B104	B108	anti-SSB	Anti-Cyclin	1	0
B104	B110	anti-SSB	AHA	1	0
B104	B119	anti-SSB	Hcv-rna	0.384926	0
B104	B120	anti-SSB	HCV-cAg	0.349051	0
B104	B121	anti-SSB	C3	-0.27501	0
B104	B122	anti-SSB	C4	0.242862	0
B104	B125	anti-SSB	Cor	0.298639	0
B104	B134	anti-SSB	CHE	-0.23432	0
B104	B151	anti-SSB	Anti-CCP	0.245648	0
B104	B152	anti-SSB	ACA-Ig(G-A-M)	-0.385	0
B104	B156	anti-SSB	IgE	0.234142	0
B104	B157	anti-SSB	IgG	0.328845	0
B104	B159	anti-SSB	β2-MG	-0.38492	0
B104	B138	anti-SSB	PAB	-0.18058	4.35E-14
B104	B154	anti-SSB	RF	0.158265	3.99E-11
B104	B178	anti-SSB	Ig-U	0.151079	2.96E-10
B104	B193	anti-SSB	K	0.125601	1.70E-07
B104	B182	anti-SSB	TT	0.124542	2.17E-07
B104	B183	anti-SSB	PTA	-0.11862	7.97E-07
B105	B107	anti-U1RNP	AnuA	0.999999	0
B105	B108	anti-U1RNP	Anti-Cyclin	1	0
B105	B119	anti-U1RNP	Hcv-rna	0.384926	0
B105	B120	anti-U1RNP	HCV-cAg	0.349051	0
B105	B121	anti-U1RNP	C3	-0.27501	0
B105	B122	anti-U1RNP	C4	0.242862	0
B105	B125	anti-U1RNP	Cor	0.298639	0
B105	B134	anti-U1RNP	CHE	-0.23432	0
B105	B151	anti-U1RNP	Anti-CCP	0.245648	0

B105	B152	anti-U1RNP	ACA-Ig(G-A-M)	-0.385	0
B105	B156	anti-U1RNP	IgE	0.234142	0
B105	B157	anti-U1RNP	IgG	0.328845	0
B105	B159	anti-U1RNP	β2-MG	-0.38492	0
B105	B138	anti-U1RNP	PAB	-0.18058	4.35E-14
B105	B154	anti-U1RNP	RF	0.158265	3.99E-11
B105	B178	anti-U1RNP	Ig-U	0.151079	2.96E-10
B105	B193	anti-U1RNP	K	0.125601	1.70E-07
B105	B182	anti-U1RNP	TT	0.124542	2.17E-07
B105	B183	anti-U1RNP	PTA	-0.11862	7.97E-07
B106	B107	anti-P	AnuA	0.999999	0
B106	B119	anti-P	Hcv-rna	0.384926	0
B106	B120	anti-P	HCV-cAg	0.349051	0
B106	B121	anti-P	C3	-0.27501	0
B106	B122	anti-P	C4	0.242862	0
B106	B125	anti-P	Cor	0.298639	0
B106	B134	anti-P	CHE	-0.23432	0
B106	B151	anti-P	Anti-CCP	0.245648	0
B106	B152	anti-P	ACA-Ig(G-A-M)	-0.38501	0
B106	B156	anti-P	IgE	0.234142	0
B106	B157	anti-P	IgG	0.328845	0
B106	B159	anti-P	β2-MG	-0.38492	0
B106	B138	anti-P	PAB	-0.18058	4.35E-14
B106	B154	anti-P	RF	0.158265	3.99E-11
B106	B178	anti-P	Ig-U	0.151079	2.96E-10
B106	B193	anti-P	K	0.125601	1.70E-07
B106	B182	anti-P	TT	0.124542	2.17E-07
B106	B183	anti-P	PTA	-0.11862	7.97E-07
B107	B108	AnuA	Anti-Cyclin	0.999999	0
B107	B109	AnuA	AMA-M2	0.999999	0
B107	B110	AnuA	AHA	0.999999	0
B107	B119	AnuA	Hcv-rna	0.384925	0
B107	B120	AnuA	HCV-cAg	0.349053	0
B107	B121	AnuA	C3	-0.27501	0
B107	B122	AnuA	C4	0.242862	0
B107	B125	AnuA	Cor	0.298638	0
B107	B134	AnuA	CHE	-0.23432	0
B107	B151	AnuA	Anti-CCP	0.245647	0
B107	B152	AnuA	ACA-Ig(G-A-M)	-0.38501	0
B107	B156	AnuA	IgE	0.234142	0
B107	B157	AnuA	IgG	0.328845	0
B107	B159	AnuA	β2-MG	-0.38492	0
B107	B138	AnuA	PAB	-0.18058	4.35E-14
B107	B154	AnuA	RF	0.158265	3.99E-11

B107	B178	AnuA	Ig-U	0.151079	2.96E-10
B107	B193	AnuA	K	0.125601	1.70E-07
B107	B182	AnuA	TT	0.124542	2.17E-07
B107	B183	AnuA	PTA	-0.11862	7.97E-07
B108	B119	Anti-Cyclin	Hcv-rna	0.384926	0
B108	B120	Anti-Cyclin	HCV-cAg	0.349051	0
B108	B121	Anti-Cyclin	C3	-0.27501	0
B108	B122	Anti-Cyclin	C4	0.242862	0
B108	B125	Anti-Cyclin	Cor	0.298639	0
B108	B134	Anti-Cyclin	CHE	-0.23432	0
B108	B151	Anti-Cyclin	Anti-CCP	0.245648	0
B108	B152	Anti-Cyclin	ACA-Ig(G-A-M)	-0.38501	0
B108	B156	Anti-Cyclin	IgE	0.234142	0
B108	B157	Anti-Cyclin	IgG	0.328845	0
B108	B159	Anti-Cyclin	β2-MG	-0.38492	0
B108	B138	Anti-Cyclin	PAB	-0.18058	4.35E-14
B108	B154	Anti-Cyclin	RF	0.158265	3.99E-11
B108	B178	Anti-Cyclin	Ig-U	0.151079	2.96E-10
B108	B193	Anti-Cyclin	K	0.125601	1.70E-07
B108	B182	Anti-Cyclin	TT	0.124542	2.17E-07
B108	B183	Anti-Cyclin	PTA	-0.11862	7.97E-07
B109	B119	AMA-M2	Hcv-rna	0.384926	0
B109	B120	AMA-M2	HCV-cAg	0.349051	0
B109	B121	AMA-M2	C3	-0.27501	0
B109	B122	AMA-M2	C4	0.242862	0
B109	B125	AMA-M2	Cor	0.298639	0
B109	B134	AMA-M2	CHE	-0.23432	0
B109	B151	AMA-M2	Anti-CCP	0.245648	0
B109	B152	AMA-M2	ACA-Ig(G-A-M)	-0.385	0
B109	B156	AMA-M2	IgE	0.234142	0
B109	B157	AMA-M2	IgG	0.328845	0
B109	B159	AMA-M2	β2-MG	-0.38492	0
B109	B138	AMA-M2	PAB	-0.18058	4.35E-14
B109	B154	AMA-M2	RF	0.158265	3.99E-11
B109	B178	AMA-M2	Ig-U	0.151079	2.96E-10
B109	B193	AMA-M2	K	0.125601	1.70E-07
B109	B182	AMA-M2	TT	0.124542	2.17E-07
B109	B183	AMA-M2	PTA	-0.11862	7.97E-07
B11	B12	PGI/PGII	PG II	-0.49833	0
B11	B14	PGI/PGII	G-17	-0.2062	0
B11	B21	PGI/PGII	CD3+CD4-CD8-	0.124866	2.01E-07
B110	B119	AHA	Hcv-rna	0.384926	0
B110	B120	AHA	HCV-cAg	0.349051	0
B110	B121	AHA	C3	-0.27501	0

B110	B122	AHA	C4	0.242862	0
B110	B125	AHA	Cor	0.298639	0
B110	B134	AHA	CHE	-0.23432	0
B110	B151	AHA	Anti-CCP	0.245648	0
B110	B152	AHA	ACA-Ig(G-A-M)	-0.385	0
B110	B156	AHA	IgE	0.234142	0
B110	B157	AHA	IgG	0.328845	0
B110	B159	AHA	β2-MG	-0.38492	0
B110	B138	AHA	PAB	-0.18058	4.35E-14
B110	B154	AHA	RF	0.158265	3.99E-11
B110	B178	AHA	Ig-U	0.151079	2.96E-10
B110	B193	AHA	K	0.125601	1.70E-07
B110	B182	AHA	TT	0.124542	2.17E-07
B110	B183	AHA	PTA	-0.11862	7.97E-07
B111	B112	TSH	FT4	-0.20619	0
B111	B148	TSH	Anti_TPO	0.289146	0
B111	B179	TSH	α1-MG	-0.15679	6.07E-11
B111	B114	TSH	TSH TT3 TT4(TT4)	-0.12447	2.20E-07
B112	B113	FT4	FT3	0.207854	0
B112	B114	FT4	TSH TT3 TT4(TT4)	0.314788	0
B112	B204	FT4	Drinking	-0.17419	3.37E-13
B112	B150	FT4	ALP	0.121745	4.04E-07
B113	B115	FT3	TSH TT3 TT4(TT3)	0.29357	0
B113	B189	FT3	eGFB	0.242024	0
B113	B176	FT3	Ucr	0.195416	4.44E-16
B113	B144	FT3	Total-PINP	0.172988	4.91E-13
B113	B191	FT3	Urea	-0.16866	1.86E-12
B113	B190	FT3	Cre	-0.14454	1.69E-09
B113	B126	FT3	Alb	0.130022	6.14E-08
B113	B128	FT3	ALT	0.124977	1.96E-07
B114	B115	TSH TT3 TT4(TT4)	TSH TT3 TT4(TT3)	0.366559	0
B114	B134	TSH TT3 TT4(TT4)	CHE	-0.26418	0
B114	B138	TSH TT3 TT4(TT4)	PAB	-0.24535	0
B114	B139	TSH TT3 TT4(TT4)	ADA	0.198875	0
B114	B124	TSH TT3 TT4(TT4)	ACTH	0.196371	2.22E-16
B114	B125	TSH TT3 TT4(TT4)	Cor	0.174904	2.69E-13
B114	B181	TSH TT3 TT4(TT4)	APTT	0.149107	5.04E-10
B114	B158	TSH TT3 TT4(TT4)	IgM	-0.13878	7.34E-09
B114	B197	TSH TT3 TT4(TT4)	AG	0.129399	7.11E-08
B115	B119	TSH TT3 TT4(TT3)	Hcv-rna	-0.26976	0
B115	B125	TSH TT3 TT4(TT3)	Cor	-0.24097	0
B115	B152	TSH TT3 TT4(TT3)	ACA-Ig(G-A-M)	0.269812	0
B115	B159	TSH TT3 TT4(TT3)	β2-MG	0.269755	0
B115	B158	TSH TT3 TT4(TT3)	IgM	0.185286	9.10E-15

B115	B139	TSH TT3 TT4(TT3)	ADA	0.14744	7.87E-10
B115	B186	TSH TT3 TT4(TT3)	PFC	0.135541	1.64E-08
B115	B197	TSH TT3 TT4(TT3)	AG	0.127959	9.93E-08
B115	B183	TSH TT3 TT4(TT3)	PTA	-0.12041	5.41E-07
B117	B202	TT4	e-times	0.954475	0
B117	B223	TT4	FH	0.580007	0
B118	B209	CEA1	Smoking	0.18963	2.22E-15
B118	B189	CEA1	eGFB	-0.18487	1.07E-14
B118	B153	CEA1	Glu	0.177394	1.22E-13
B118	B190	CEA1	Cre	0.162684	1.11E-11
B118	B204	CEA1	Drinking	0.14767	7.40E-10
B118	B220	CEA1	SBP	0.128863	8.05E-08
B118	B206	CEA1	Waist	0.126111	1.52E-07
B118	B191	CEA1	Urea	0.124253	2.31E-07
B118	B222	CEA1	NS	0.122242	3.62E-07
B118	B170	CEA1	U-GLU	0.119715	6.29E-07
B119	B120	Hcv-rna	HCV-cAg	0.456823	0
B119	B124	Hcv-rna	ACTH	0.396008	0
B119	B125	Hcv-rna	Cor	0.612351	0
B119	B134	Hcv-rna	CHE	-0.26306	0
B119	B158	Hcv-rna	IgM	-0.49345	0
B119	B159	Hcv-rna	β2-MG	-0.99999	0
B119	B179	Hcv-rna	α1-MG	-0.23378	0
B119	B156	Hcv-rna	IgE	0.182861	2.04E-14
B119	B203	Hcv-rna	DH	-0.1777	1.10E-13
B119	B155	Hcv-rna	IgA	0.165035	5.55E-12
B119	B151	Hcv-rna	Anti-CCP	0.155937	7.71E-11
B119	B138	Hcv-rna	PAB	-0.1451	1.46E-09
B119	B184	Hcv-rna	INR	0.144909	1.53E-09
B119	B181	Hcv-rna	APTT	0.132409	3.49E-08
B119	B186	Hcv-rna	PFC	-0.132	3.85E-08
B119	B196	Hcv-rna	TCO2	-0.11885	7.59E-07
B12	B200	PG II	OP	-0.1234	2.80E-07
B120	B121	HCV-cAg	C3	-0.24949	0
B120	B122	HCV-cAg	C4	0.211315	0
B120	B125	HCV-cAg	Cor	0.278836	0
B120	B134	HCV-cAg	CHE	-0.21023	0
B120	B152	HCV-cAg	ACA-Ig(G-A-M)	-0.45693	0
B120	B159	HCV-cAg	β2-MG	-0.45682	0
B120	B155	HCV-cAg	IgA	0.18479	1.07E-14
B120	B124	HCV-cAg	ACTH	0.179863	5.48E-14
B120	B158	HCV-cAg	IgM	-0.17099	9.12E-13
B120	B138	HCV-cAg	PAB	-0.14604	1.14E-09
B120	B157	HCV-cAg	IgG	0.142019	3.24E-09

B120	B203	HCV-cAg	DH	-0.13713	1.11E-08
B120	B199	HCV-cAg	Mg	-0.13084	5.06E-08
B120	B195	HCV-cAg	Na	-0.11851	8.15E-07
B121	B122	C3	C4	-0.99649	0
B121	B134	C3	CHE	0.328621	0
B121	B156	C3	IgE	0.421915	0
B121	B198	C3	P	-0.21683	0
B121	B193	C3	K	0.174542	3.02E-13
B121	B224	C3	pmh	0.130934	4.96E-08
B121	B138	C3	PAB	0.129366	7.16E-08
B121	B194	C3	Cl	-0.12125	4.50E-07
B122	B134	C4	CHE	-0.3067	0
B122	B156	C4	IgE	-0.43738	0
B122	B198	C4	P	0.214051	0
B122	B193	C4	K	-0.17296	4.94E-13
B122	B224	C4	pmh	-0.13185	3.99E-08
B122	B194	C4	Cl	0.127949	9.95E-08
B123	B186	hs-CRP1	PFC	0.189738	2.00E-15
B124	B125	ACTH	Cor	0.544605	0
B124	B152	ACTH	ACA-Ig(G-A-M)	-0.39609	0
B124	B155	ACTH	IgA	0.273508	0
B124	B158	ACTH	IgM	-0.49415	0
B124	B159	ACTH	β2-MG	-0.396	0
B124	B179	ACTH	α1-MG	-0.28788	0
B124	B182	ACTH	TT	0.287552	0
B124	B178	ACTH	Ig-U	-0.15307	1.71E-10
B124	B134	ACTH	CHE	-0.12844	8.88E-08
B124	B183	ACTH	PTA	0.125098	1.91E-07
B124	B203	ACTH	DH	-0.12088	4.88E-07
B124	B193	ACTH	K	-0.11843	8.30E-07
B125	B134	Cor	CHE	-0.26509	0
B125	B152	Cor	ACA-Ig(G-A-M)	-0.61247	0
B125	B155	Cor	IgA	0.220856	0
B125	B156	Cor	IgE	0.217728	0
B125	B158	Cor	IgM	-0.61831	0
B125	B159	Cor	β2-MG	-0.61234	0
B125	B179	Cor	α1-MG	-0.29925	0
B125	B182	Cor	TT	0.256708	0
B125	B199	Cor	Mg	-0.19846	0
B125	B203	Cor	DH	-0.1872	4.88E-15
B125	B184	Cor	INR	0.184915	1.02E-14
B125	B151	Cor	Anti-CCP	0.177814	1.06E-13
B125	B186	Cor	PFC	-0.17673	1.51E-13
B125	B181	Cor	APTT	0.172666	5.43E-13

B125	B196	Cor	TCO2	-0.1312	4.65E-08
B125	B178	Cor	Ig-U	-0.12803	9.76E-08
B125	B157	Cor	IgG	0.121293	4.46E-07
B126	B127	Alb	A-G ratio	0.472143	0
B126	B188	Alb	Ca	0.265379	0
B126	B189	Alb	eGFB	0.289856	0
B126	B128	Alb	ALT	0.188705	2.89E-15
B126	B141	Alb	AST/ALT	-0.16131	1.66E-11
B126	B176	Alb	Ucr	0.158069	4.22E-11
B126	B163	Alb	SG	0.155075	9.82E-11
B126	B191	Alb	Urea	-0.13778	9.42E-09
B126	B129	Alb	IBIL	0.129585	6.80E-08
B126	B218	Alb	DBP	0.121862	3.93E-07
B126	B190	Alb	Cre	-0.11944	6.68E-07
B127	B130	A-G ratio	GLB	-0.88154	0
B127	B189	A-G ratio	eGFB	0.19365	4.44E-16
B127	B221	A-G ratio	Hei	0.164594	6.33E-12
B127	B220	A-G ratio	SBP	-0.1468	9.33E-10
B127	B139	A-G ratio	ADA	-0.12677	1.31E-07
B127	B138	A-G ratio	PAB	0.126143	1.51E-07
B128	B131	ALT	AST	0.658829	0
B128	B140	ALT	r-GT	0.515257	0
B128	B141	ALT	AST/ALT	-0.47902	0
B128	B215	ALT	BMI	0.22648	0
B128	B216	ALT	BMI	0.246858	0
B128	B222	ALT	NS	0.214803	0
B128	B171	ALT	EC	0.189853	1.78E-15
B128	B206	ALT	Waist	0.18928	2.22E-15
B128	B192	ALT	UA	0.174112	3.45E-13
B128	B163	ALT	SG	0.156351	6.87E-11
B128	B210	ALT	Hip	0.152881	1.81E-10
B128	B133	ALT	TBIL	0.149074	5.09E-10
B128	B129	ALT	IBIL	0.136237	1.38E-08
B128	B132	ALT	Dbil	0.134948	1.89E-08
B128	B221	ALT	Hei	0.134647	2.04E-08
B128	B189	ALT	eGFB	0.131612	4.22E-08
B128	B176	ALT	Ucr	0.129687	6.64E-08
B129	B132	IBIL	Dbil	0.556623	0
B129	B133	IBIL	TBIL	0.971653	0
B130	B220	GLB	SBP	0.129281	7.31E-08
B130	B221	GLB	Hei	-0.1205	5.30E-07
B131	B140	AST	r-GT	0.472169	0
B131	B171	AST	EC	0.525899	0
B131	B132	AST	Dbil	0.145512	1.31E-09

B131	B170	AST	U-GLU	0.133082	2.97E-08
B131	B133	AST	TBIL	0.122571	3.36E-07
B132	B133	Dbil	TBIL	0.737123	0
B132	B140	Dbil	r-GT	0.12513	1.90E-07
B134	B138	CHE	PAB	0.464754	0
B134	B152	CHE	ACA-Ig(G-A-M)	0.263108	0
B134	B159	CHE	β2-MG	0.263053	0
B134	B199	CHE	Mg	0.20485	0
B134	B137	CHE	AFU	0.163224	9.48E-12
B134	B139	CHE	ADA	-0.15955	2.76E-11
B134	B135	CHE	TBA	-0.15607	7.43E-11
B134	B158	CHE	IgM	0.139672	5.87E-09
B134	B146	CHE	CysC	-0.132	3.85E-08
B134	B179	CHE	α1-MG	0.128877	8.03E-08
B134	B180	CHE	Tf	0.127605	1.08E-07
B134	B194	CHE	Cl	-0.12561	1.70E-07
B134	B196	CHE	TCO2	0.119206	7.02E-07
B135	B137	TBA	AFU	0.228946	0
B135	B193	TBA	K	-0.16966	1.38E-12
B135	B178	TBA	Ig-U	-0.15801	4.29E-11
B135	B199	TBA	Mg	-0.15718	5.43E-11
B135	B136	TBA	5'NT	-0.15428	1.23E-10
B135	B138	TBA	PAB	-0.13906	6.85E-09
B135	B180	TBA	Tf	-0.1385	7.88E-09
B136	B137	5'NT	AFU	-0.20896	0
B136	B155	5'NT	IgA	-0.20216	0
B136	B196	5'NT	TCO2	-0.21339	0
B136	B197	5'NT	AG	0.294158	0
B136	B157	5'NT	IgG	-0.19322	4.44E-16
B136	B139	5'NT	ADA	0.187917	3.77E-15
B136	B183	5'NT	PTA	-0.17739	1.22E-13
B136	B181	5'NT	APTT	-0.17108	8.86E-13
B136	B194	5'NT	Cl	-0.14401	1.94E-09
B136	B199	5'NT	Mg	-0.14082	4.40E-09
B136	B158	5'NT	IgM	-0.13309	2.97E-08
B136	B198	5'NT	P	0.132076	3.78E-08
B136	B156	5'NT	IgE	-0.12443	2.22E-07
B137	B138	AFU	PAB	0.298379	0
B137	B193	AFU	K	-0.19264	8.88E-16
B137	B139	AFU	ADA	-0.18902	2.66E-15
B137	B199	AFU	Mg	0.136712	1.23E-08
B138	B139	PAB	ADA	-0.52179	0
B138	B155	PAB	IgA	-0.20036	0
B138	B156	PAB	IgE	-0.23202	0

B138	B199	PAB	Mg	0.197986	0
B138	B179	PAB	α1-MG	0.171527	7.73E-13
B138	B157	PAB	IgG	-0.17115	8.69E-13
B138	B183	PAB	PTA	0.161295	1.67E-11
B138	B146	PAB	CysC	-0.15942	2.87E-11
B138	B182	PAB	TT	-0.15398	1.33E-10
B138	B193	PAB	K	-0.14947	4.57E-10
B138	B152	PAB	ACA-Ig(G-A-M)	0.145131	1.45E-09
B138	B159	PAB	β2-MG	0.1451	1.46E-09
B138	B181	PAB	APTT	-0.12974	6.56E-08
B138	B184	PAB	INR	-0.11944	6.67E-07
B139	B155	ADA	IgA	0.295388	0
B139	B156	ADA	IgE	0.413322	0
B139	B199	ADA	Mg	-0.21612	0
B139	B146	ADA	CysC	0.195943	2.22E-16
B139	B158	ADA	IgM	0.179299	6.59E-14
B139	B149	ADA	TGAb	0.173057	4.81E-13
B139	B157	ADA	IgG	0.153819	1.39E-10
B139	B148	ADA	Anti_TPO	0.144984	1.50E-09
B139	B183	ADA	PTA	-0.12827	9.24E-08
B14	B47	G-17	RDW	0.179684	5.82E-14
B14	B43	G-17	MCV	0.166724	3.35E-12
B14	B44	G-17	MCH	0.166069	4.07E-12
B14	B74	G-17	ApoA1	-0.16416	7.19E-12
B14	B46	G-17	RBC Count	-0.13216	3.71E-08
B140	B171	r-GT	EC	0.210073	0
B140	B141	r-GT	AST/ALT	-0.16922	1.57E-12
B140	B181	r-GT	APTT	-0.15232	2.11E-10
B140	B216	r-GT	BMI	0.142514	2.85E-09
B140	B189	r-GT	eGFB	0.141063	4.13E-09
B140	B222	r-GT	NS	0.123564	2.70E-07
B140	B215	r-GT	BMI	0.123363	2.82E-07
B141	B215	AST/ALT	BMI	-0.2117	0
B141	B216	AST/ALT	BMI	-0.25324	0
B141	B222	AST/ALT	NS	-0.21627	0
B141	B206	AST/ALT	Waist	-0.1939	4.44E-16
B141	B221	AST/ALT	Hei	-0.17506	2.56E-13
B141	B163	AST/ALT	SG	-0.16773	2.47E-12
B141	B210	AST/ALT	Hip	-0.15648	6.62E-11
B141	B192	AST/ALT	UA	-0.14477	1.59E-09
B141	B176	AST/ALT	Ucr	-0.14157	3.64E-09
B141	B189	AST/ALT	eGFB	-0.12242	3.48E-07
B142	B151	25-(OH)D	Anti-CCP	-0.16875	1.81E-12
B142	B154	25-(OH)D	RF	-0.16044	2.13E-11

B142	B144	25-(OH)D	Total-PINP	-0.1485	5.94E-10
B143	B144	β-CTx	Total-PINP	0.824272	0
B143	B145	β-CTx	BGP	0.812161	0
B143	B150	β-CTx	ALP	0.165681	4.58E-12
B143	B168	β-CTx	NMP22	0.149883	4.09E-10
B143	B151	β-CTx	Anti-CCP	0.125436	1.77E-07
B144	B145	Total-PINP	BGP	0.794003	0
B144	B151	Total-PINP	Anti-CCP	0.160659	2.01E-11
B144	B150	Total-PINP	ALP	0.139831	5.64E-09
B144	B168	Total-PINP	NMP22	0.134558	2.08E-08
B145	B150	BGP	ALP	0.157256	5.32E-11
B146	B197	CysC	AG	-0.13966	5.89E-09
B146	B189	CysC	eGFB	-0.12072	5.05E-07
B146	B193	CysC	K	0.118104	8.90E-07
B148	B149	Anti_TPO	TGAb	0.594659	0
B148	B155	Anti_TPO	IgA	0.151166	2.89E-10
B148	B198	Anti_TPO	P	0.125236	1.85E-07
B149	B155	TGAb	IgA	0.133584	2.63E-08
B149	B156	TGAb	IgE	0.131517	4.32E-08
B15	B101	CD3-CD19+(B cell)	anti-Sc70	0.503508	0
B15	B102	CD3-CD19+(B cell)	anti-Sm	0.503508	0
B15	B103	CD3-CD19+(B cell)	anti-SSA/Ro	0.503508	0
B15	B104	CD3-CD19+(B cell)	anti-SSB	0.503508	0
B15	B105	CD3-CD19+(B cell)	anti-U1RNP	0.503508	0
B15	B106	CD3-CD19+(B cell)	anti-P	0.503508	0
B15	B107	CD3-CD19+(B cell)	AnuA	0.503507	0
B15	B108	CD3-CD19+(B cell)	Anti-Cyclin	0.503508	0
B15	B109	CD3-CD19+(B cell)	AMA-M2	0.503508	0
B15	B110	CD3-CD19+(B cell)	AHA	0.503508	0
B15	B121	CD3-CD19+(B cell)	C3	-0.63292	0
B15	B122	CD3-CD19+(B cell)	C4	0.618635	0
B15	B134	CD3-CD19+(B cell)	CHE	-0.22803	0
B15	B138	CD3-CD19+(B cell)	PAB	-0.21923	0
B15	B151	CD3-CD19+(B cell)	Anti-CCP	0.322064	0
B15	B157	CD3-CD19+(B cell)	IgG	0.217577	0
B15	B17	CD3-CD19+(B cell)	CD3+CD16+CD56+NK	-0.28992	0
B15	B18	CD3-CD19+(B cell)	CD3+CD4+helper(inducer)T	0.647539	0
B15	B19	CD3-CD19+(B cell)	CD3+CD4+/CD3+CD8+	0.748441	0
B15	B21	CD3-CD19+(B cell)	CD3+CD4-CD8-	0.212241	0
B15	B22	CD3-CD19+(B cell)	CD3+CD8+(T)	-0.6321	0
B15	B23	CD3-CD19+(B cell)	CD3-CD16+CD56+(NK)	-0.35071	0
B15	B54	CD3-CD19+(B cell)	ESR	0.206402	0
B15	B95	CD3-CD19+(B cell)	anti-cenpb	0.503508	0
B15	B97	CD3-CD19+(B cell)	dsDNA	0.503508	0

B15	B98	CD3-CD19+(B cell)	anti-Jo-1	0.503508	0
B15	B99	CD3-CD19+(B cell)	anti-PM-Scl	0.503508	0
B15	B120	CD3-CD19+(B cell)	HCV-cAg	0.19201	8.88E-16
B15	B125	CD3-CD19+(B cell)	Cor	0.18866	2.89E-15
B15	B152	CD3-CD19+(B cell)	ACA-Ig(G-A-M)	-0.17317	4.63E-13
B15	B119	CD3-CD19+(B cell)	Hcv-rna	0.173137	4.69E-13
B15	B159	CD3-CD19+(B cell)	β2-MG	-0.17314	4.69E-13
B15	B94	CD3-CD19+(B cell)	ALDH2-Lys504Lys	0.173137	4.69E-13
B15	B135	CD3-CD19+(B cell)	TBA	0.155596	8.49E-11
B15	B181	CD3-CD19+(B cell)	APTT	0.154548	1.14E-10
B15	B55	CD3-CD19+(B cell)	TK	0.145308	1.38E-09
B15	B79	CD3-CD19+(B cell)	hb V -dna	0.140311	5.00E-09
B15	B58	CD3-CD19+(B cell)	HCT	-0.1392	6.61E-09
B15	B74	CD3-CD19+(B cell)	ApoA1	0.135284	1.74E-08
B15	B92	CD3-CD19+(B cell)	ALDH2-Glu504Glu	-0.12773	1.05E-07
B15	B183	CD3-CD19+(B cell)	PTA	-0.1271	1.21E-07
B15	B67	CD3-CD19+(B cell)	WBRSR(mPas_50)	-0.12263	3.32E-07
B15	B66	CD3-CD19+(B cell)	WBRSR(mPas_100)	-0.11976	6.23E-07
B15	B93	CD3-CD19+(B cell)	ALDH2-Glu504Lys	0.118986	7.36E-07
B151	B156	Anti-CCP	IgE	0.231514	0
B151	B157	Anti-CCP	IgG	0.241795	0
B151	B168	Anti-CCP	NMP22	-0.22189	0
B151	B152	Anti-CCP	ACA-Ig(G-A-M)	-0.15597	7.65E-11
B151	B159	Anti-CCP	β2-MG	-0.15594	7.72E-11
B151	B183	Anti-CCP	PTA	-0.12697	1.25E-07
B151	B193	Anti-CCP	K	0.123628	2.66E-07
B151	B197	Anti-CCP	AG	0.120424	5.39E-07
B152	B158	ACA-Ig(G-A-M)	IgM	0.493545	0
B152	B179	ACA-Ig(G-A-M)	α1-MG	0.233822	0
B152	B156	ACA-Ig(G-A-M)	IgE	-0.1829	2.04E-14
B152	B203	ACA-Ig(G-A-M)	DH	0.177736	1.09E-13
B152	B155	ACA-Ig(G-A-M)	IgA	-0.16507	5.49E-12
B152	B184	ACA-Ig(G-A-M)	INR	-0.14494	1.52E-09
B152	B181	ACA-Ig(G-A-M)	APTT	-0.13244	3.47E-08
B152	B186	ACA-Ig(G-A-M)	PFC	0.132025	3.83E-08
B152	B196	ACA-Ig(G-A-M)	TCO2	0.118869	7.55E-07
B153	B170	Glu	U-GLU	0.561095	0
B153	B206	Glu	Waist	0.220321	0
B153	B207	Glu	WHR	0.20817	0
B153	B220	Glu	SBP	0.20607	0
B153	B215	Glu	BMI	0.187585	4.22E-15
B153	B222	Glu	NS	0.164376	6.75E-12
B153	B216	Glu	BMI	0.145489	1.32E-09
B153	B191	Glu	Urea	0.126274	1.46E-07

B153	B175	Glu	ALB-U	0.12082	4.94E-07
B153	B163	Glu	SG	0.118206	8.70E-07
B155	B156	IgA	IgE	0.43841	0
B155	B196	IgA	TCO2	0.176142	1.82E-13
B155	B159	IgA	β2-MG	-0.16503	5.55E-12
B155	B199	IgA	Mg	-0.15137	2.73E-10
B155	B197	IgA	AG	-0.14728	8.20E-10
B155	B182	IgA	TT	0.1399	5.55E-09
B155	B179	IgA	α1-MG	-0.11872	7.79E-07
B156	B157	IgE	IgG	0.447624	0
B156	B159	IgE	β2-MG	-0.18286	2.04E-14
B156	B182	IgE	TT	0.140413	4.87E-09
B156	B224	IgE	pmh	0.123276	2.88E-07
B157	B181	IgG	APTT	0.215681	0
B157	B158	IgG	IgM	0.194657	4.44E-16
B157	B184	IgG	INR	0.127724	1.05E-07
B158	B159	IgM	β2-MG	0.493441	0
B158	B179	IgM	α1-MG	0.3265	0
B158	B196	IgM	TCO2	0.228037	0
B158	B178	IgM	Ig-U	0.18154	3.18E-14
B158	B183	IgM	PTA	-0.16442	6.66E-12
B158	B197	IgM	AG	-0.14663	9.75E-10
B158	B203	IgM	DH	0.143474	2.23E-09
B158	B198	IgM	P	-0.13629	1.36E-08
B158	B182	IgM	TT	-0.13121	4.64E-08
B159	B179	β2-MG	α1-MG	0.233773	0
B159	B203	β2-MG	DH	0.177699	1.10E-13
B159	B184	β2-MG	INR	-0.14491	1.53E-09
B159	B181	β2-MG	APTT	-0.13241	3.49E-08
B159	B186	β2-MG	PFC	0.131997	3.85E-08
B159	B196	β2-MG	TCO2	0.118844	7.59E-07
B16	B115	CD3+T	TSH TT3 TT4(TT3)	0.250048	0
B16	B119	CD3+T	Hcv-rna	-0.20794	0
B16	B124	CD3+T	ACTH	-0.54744	0
B16	B125	CD3+T	Cor	-0.30617	0
B16	B152	CD3+T	ACA-Ig(G-A-M)	0.207985	0
B16	B155	CD3+T	IgA	-0.40969	0
B16	B158	CD3+T	IgM	0.321015	0
B16	B159	CD3+T	β2-MG	0.207942	0
B16	B17	CD3+T	CD3+CD16+CD56+NK	0.627785	0
B16	B179	CD3+T	α1-MG	0.286162	0
B16	B18	CD3+T	CD3+CD4+helper(inducer)T	0.634562	0
B16	B181	CD3+T	APTT	0.236034	0
B16	B182	CD3+T	TT	-0.25966	0

B16	B183	CD3+T	PTA	-0.21784	0
B16	B20	CD3+T	CD3+CD4+CD8+	-0.50689	0
B16	B21	CD3+T	CD3+CD4-CD8-	0.390532	0
B16	B22	CD3+T	CD3+CD8+(T)	0.423953	0
B16	B23	CD3+T	CD3-CD16+CD56+(NK)	-0.93468	0
B16	B25	CD3+T	CK	0.201892	0
B16	B58	CD3+T	HCT	0.199656	0
B16	B94	CD3+T	ALDH2-Lys504Lys	-0.20794	0
B16	B157	CD3+T	IgG	0.196417	2.22E-16
B16	B66	CD3+T	WBRSR(mPas_100)	0.187815	4.00E-15
B16	B63	CD3+T	WBRSR(mPas_200)	0.185147	9.55E-15
B16	B178	CD3+T	Ig-U	0.180736	4.13E-14
B16	B67	CD3+T	WBRSR(mPas_50)	0.176867	1.44E-13
B16	B62	CD3+T	WBHSRVI	0.174668	2.90E-13
B16	B79	CD3+T	hb V -dna	-0.16971	1.35E-12
B16	B76	CD3+T	ApoB	0.161359	1.64E-11
B16	B92	CD3+T	ALDH2-Glu504Glu	0.151754	2.46E-10
B16	B61	CD3+T	WBHSRV	0.150836	3.16E-10
B16	B75	CD3+T	ApoA1/B	-0.14318	2.40E-09
B16	B93	CD3+T	ALDH2-Glu504Lys	-0.14124	3.95E-09
B16	B56	CD3+T	IR	0.135151	1.80E-08
B16	B185	CD3+T	PT	0.131542	4.29E-08
B16	B64	CD3+T	WBRSR(mPas_5)	0.128816	8.14E-08
B16	B73	CD3+T	ApoA	-0.12621	1.48E-07
B16	B194	CD3+T	CI	-0.12263	3.32E-07
B16	B186	CD3+T	PFC	0.117876	9.34E-07
B160	B191	PH	Urea	-0.14621	1.09E-09
B160	B192	PH	UA	-0.14607	1.13E-09
B160	B163	PH	SG	-0.13911	6.76E-09
B160	B190	PH	Cre	-0.13229	3.59E-08
B161	B221	LEU	Hei	-0.1869	5.33E-15
B161	B173	LEU	NTT	0.16002	2.41E-11
B161	B222	LEU	NS	-0.13573	1.56E-08
B163	B166	SG	Pro	0.33842	0
B163	B176	SG	Ucr	0.570032	0
B163	B216	SG	BMI	0.280659	0
B163	B221	SG	Hei	0.264325	0
B163	B222	SG	NS	0.294306	0
B163	B191	SG	Urea	0.192252	8.88E-16
B163	B215	SG	BMI	0.185957	7.33E-15
B163	B189	SG	eGFB	0.18558	8.44E-15
B163	B206	SG	Waist	0.170039	1.22E-12
B163	B210	SG	Hip	0.155204	9.48E-11
B163	B192	SG	UA	0.153848	1.38E-10

B163	B170	SG	U-GLU	0.150252	3.70E-10
B163	B220	SG	SBP	-0.13362	2.61E-08
B163	B165	SG	bilirubin	0.132135	3.73E-08
B164	B213	COM	CVA-R	-0.12949	6.96E-08
B165	B189	bilirubin	eGFB	0.169036	1.66E-12
B166	B176	Pro	Ucr	0.158293	3.96E-11
B166	B222	Pro	NS	0.13332	2.81E-08
B166	B175	Pro	ALB-U	0.12337	2.82E-07
B166	B216	Pro	BMI	0.122923	3.11E-07
B166	B190	Pro	Cre	0.122745	3.24E-07
B167	B173	PC	NTT	-0.1737	3.93E-13
B17	B114	CD3+CD16+CD56+NK	TSH TT3 TT4(TT4)	0.211004	0
B17	B115	CD3+CD16+CD56+NK	TSH TT3 TT4(TT3)	0.217132	0
B17	B157	CD3+CD16+CD56+NK	IgG	0.199016	0
B17	B19	CD3+CD16+CD56+NK	CD3+CD4+/CD3+CD8+	-0.22513	0
B17	B198	CD3+CD16+CD56+NK	P	0.311371	0
B17	B20	CD3+CD16+CD56+NK	CD3+CD4+CD8+	-0.29988	0
B17	B21	CD3+CD16+CD56+NK	CD3+CD4-CD8-	0.584192	0
B17	B22	CD3+CD16+CD56+NK	CD3+CD8+(T)	0.538831	0
B17	B23	CD3+CD16+CD56+NK	CD3-CD16+CD56+(NK)	-0.37322	0
B17	B148	CD3+CD16+CD56+NK	Anti_TPO	0.195192	4.44E-16
B17	B124	CD3+CD16+CD56+NK	ACTH	-0.19177	8.88E-16
B17	B194	CD3+CD16+CD56+NK	Cl	-0.17807	9.81E-14
B17	B136	CD3+CD16+CD56+NK	5'NT	0.169881	1.28E-12
B17	B69	CD3+CD16+CD56+NK	PV	0.160117	2.35E-11
B17	B25	CD3+CD16+CD56+NK	CK	0.16001	2.42E-11
B17	B57	CD3+CD16+CD56+NK	RBCAI	-0.14468	1.63E-09
B17	B156	CD3+CD16+CD56+NK	IgE	-0.13975	5.76E-09
B17	B200	CD3+CD16+CD56+NK	OP	-0.13824	8.40E-09
B17	B101	CD3+CD16+CD56+NK	anti-Sc70	0.135702	1.57E-08
B17	B102	CD3+CD16+CD56+NK	anti-Sm	0.135702	1.57E-08
B17	B103	CD3+CD16+CD56+NK	anti-SSA/Ro	0.135702	1.57E-08
B17	B104	CD3+CD16+CD56+NK	anti-SSB	0.135702	1.57E-08
B17	B105	CD3+CD16+CD56+NK	anti-U1RNP	0.135702	1.57E-08
B17	B106	CD3+CD16+CD56+NK	anti-P	0.135702	1.57E-08
B17	B107	CD3+CD16+CD56+NK	AnuA	0.135702	1.57E-08
B17	B108	CD3+CD16+CD56+NK	Anti-Cyclin	0.135702	1.57E-08
B17	B109	CD3+CD16+CD56+NK	AMA-M2	0.135702	1.57E-08
B17	B110	CD3+CD16+CD56+NK	AHA	0.135702	1.57E-08
B17	B95	CD3+CD16+CD56+NK	anti-cenpb	0.135702	1.57E-08
B17	B97	CD3+CD16+CD56+NK	dsDNA	0.135702	1.57E-08
B17	B98	CD3+CD16+CD56+NK	anti-Jo-1	0.135702	1.57E-08
B17	B99	CD3+CD16+CD56+NK	anti-PM-Scl	0.135702	1.57E-08
B17	B113	CD3+CD16+CD56+NK	FT3	0.135598	1.61E-08

B17	B183	CD3+CD16+CD56+NK	PTA	-0.13413	2.31E-08
B17	B142	CD3+CD16+CD56+NK	25-(OH)D	0.129874	6.36E-08
B17	B144	CD3+CD16+CD56+NK	Total-PINP	0.127245	1.17E-07
B17	B112	CD3+CD16+CD56+NK	FT4	0.118493	8.19E-07
B170	B171	U-GLU	EC	0.13283	3.16E-08
B174	B189	Urea/Cr	eGFB	0.207076	0
B174	B190	Urea/Cr	Cre	-0.40954	0
B174	B191	Urea/Cr	Urea	0.526654	0
B174	B192	Urea/Cr	UA	-0.24593	0
B174	B216	Urea/Cr	BMI	-0.27247	0
B174	B221	Urea/Cr	Hei	-0.34514	0
B174	B222	Urea/Cr	NS	-0.27831	0
B174	B209	Urea/Cr	Smoking	-0.16144	1.60E-11
B174	B204	Urea/Cr	Drinking	-0.15787	4.46E-11
B174	B206	Urea/Cr	Waist	-0.14995	4.01E-10
B174	B210	Urea/Cr	Hip	-0.14537	1.36E-09
B174	B200	Urea/Cr	OP	0.117695	9.71E-07
B175	B177	ALB-U	ACR	0.812916	0
B175	B190	ALB-U	Cre	0.246736	0
B175	B191	ALB-U	Urea	0.210051	0
B175	B227	ALB-U	OD	0.130894	5.00E-08
B176	B189	Ucr	eGFB	0.210985	0
B176	B220	Ucr	SBP	-0.18682	5.33E-15
B176	B201	Ucr	e-habits	-0.15891	3.32E-11
B176	B219	Ucr	OT	-0.13441	2.16E-08
B177	B190	ACR	Cre	0.287134	0
B177	B191	ACR	Urea	0.221665	0
B177	B189	ACR	eGFB	-0.15892	3.31E-11
B177	B227	ACR	OD	0.155517	8.68E-11
B178	B179	Ig-U	α1-MG	0.678946	0
B178	B180	Ig-U	Tf	0.826112	0
B179	B180	α1-MG	Tf	0.435963	0
B179	B200	α1-MG	OP	0.154607	1.12E-10
B18	B101	CD3+CD4+helper(inducer)T	anti-Sc70	0.235992	0
B18	B102	CD3+CD4+helper(inducer)T	anti-Sm	0.235992	0
B18	B103	CD3+CD4+helper(inducer)T	anti-SSA/Ro	0.235992	0
B18	B104	CD3+CD4+helper(inducer)T	anti-SSB	0.235992	0
B18	B105	CD3+CD4+helper(inducer)T	anti-U1RNP	0.235992	0
B18	B106	CD3+CD4+helper(inducer)T	anti-P	0.235992	0
B18	B107	CD3+CD4+helper(inducer)T	AnuA	0.235992	0
B18	B108	CD3+CD4+helper(inducer)T	Anti-Cyclin	0.235992	0
B18	B109	CD3+CD4+helper(inducer)T	AMA-M2	0.235992	0
B18	B110	CD3+CD4+helper(inducer)T	AHA	0.235992	0
B18	B121	CD3+CD4+helper(inducer)T	C3	-0.33585	0

B18	B122	CD3+CD4+helper(inducer)T	C4	0.331524	0
B18	B124	CD3+CD4+helper(inducer)T	ACTH	-0.33353	0
B18	B155	CD3+CD4+helper(inducer)T	IgA	-0.27576	0
B18	B157	CD3+CD4+helper(inducer)T	IgG	0.292349	0
B18	B181	CD3+CD4+helper(inducer)T	APTT	0.446673	0
B18	B19	CD3+CD4+helper(inducer)T	CD3+CD4+/CD3+CD8+	0.795837	0
B18	B20	CD3+CD4+helper(inducer)T	CD3+CD4+CD8+	-0.49817	0
B18	B21	CD3+CD4+helper(inducer)T	CD3+CD4-CD8-	0.313182	0
B18	B22	CD3+CD4+helper(inducer)T	CD3+CD8+(T)	-0.38197	0
B18	B23	CD3+CD4+helper(inducer)T	CD3-CD16+CD56+(NK)	-0.76304	0
B18	B95	CD3+CD4+helper(inducer)T	anti-cenpb	0.235992	0
B18	B97	CD3+CD4+helper(inducer)T	dsDNA	0.235992	0
B18	B98	CD3+CD4+helper(inducer)T	anti-Jo-1	0.235992	0
B18	B99	CD3+CD4+helper(inducer)T	anti-PM-Scl	0.235992	0
B18	B182	CD3+CD4+helper(inducer)T	TT	-0.185	1.02E-14
B18	B55	CD3+CD4+helper(inducer)T	TK	0.175358	2.33E-13
B18	B179	CD3+CD4+helper(inducer)T	α1-MG	0.160828	1.91E-11
B18	B151	CD3+CD4+helper(inducer)T	Anti-CCP	0.158136	4.14E-11
B18	B158	CD3+CD4+helper(inducer)T	IgM	0.156749	6.14E-11
B18	B56	CD3+CD4+helper(inducer)T	IR	0.144251	1.82E-09
B18	B57	CD3+CD4+helper(inducer)T	RBCAI	-0.139	6.95E-09
B18	B137	CD3+CD4+helper(inducer)T	AFU	0.136626	1.25E-08
B18	B76	CD3+CD4+helper(inducer)T	ApoB	0.129774	6.51E-08
B18	B184	CD3+CD4+helper(inducer)T	INR	0.127746	1.04E-07
B18	B178	CD3+CD4+helper(inducer)T	Ig-U	0.127609	1.08E-07
B18	B73	CD3+CD4+helper(inducer)T	ApoA	-0.123	3.06E-07
B18	B61	CD3+CD4+helper(inducer)T	WBHSRV	0.121932	3.87E-07
B180	B197	Tf	AG	0.211949	0
B180	B194	Tf	Cl	-0.17615	1.81E-13
B180	B198	Tf	P	-0.17111	8.79E-13
B180	B196	Tf	TCO2	-0.16875	1.82E-12
B180	B200	Tf	OP	0.129942	6.26E-08
B180	B199	Tf	Mg	-0.12634	1.44E-07
B181	B183	APTT	PTA	-0.26776	0
B181	B184	APTT	INR	0.494501	0
B181	B185	APTT	PT	0.377255	0
B181	B186	APTT	PFC	-0.1913	1.33E-15
B181	B193	APTT	K	-0.1229	3.12E-07
B182	B183	TT	PTA	-0.71523	0
B182	B184	TT	INR	-0.22139	0
B182	B185	TT	PT	-0.32822	0
B182	B193	TT	K	0.137874	9.21E-09
B183	B186	PTA	PFC	-0.28506	0
B183	B193	PTA	K	-0.20269	0

B184	B185	INR	PT	0.889451	0
B184	B193	INR	K	-0.11963	6.41E-07
B185	B186	PT	PFC	0.154056	1.30E-10
B189	B190	eGFB	Cre	-0.69281	0
B189	B191	eGFB	Urea	-0.42856	0
B189	B220	eGFB	SBP	-0.24708	0
B189	B192	eGFB	UA	-0.18601	7.11E-15
B189	B218	eGFB	DBP	0.146314	1.06E-09
B189	B219	eGFB	OT	-0.13624	1.38E-08
B189	B227	eGFB	OD	-0.13007	6.07E-08
B189	B209	eGFB	Smoking	-0.1213	4.45E-07
B19	B101	CD3+CD4+/CD3+CD8+	anti-Sc70	0.414177	0
B19	B102	CD3+CD4+/CD3+CD8+	anti-Sm	0.414177	0
B19	B103	CD3+CD4+/CD3+CD8+	anti-SSA/Ro	0.414177	0
B19	B104	CD3+CD4+/CD3+CD8+	anti-SSB	0.414177	0
B19	B105	CD3+CD4+/CD3+CD8+	anti-U1RNP	0.414177	0
B19	B106	CD3+CD4+/CD3+CD8+	anti-P	0.414177	0
B19	B107	CD3+CD4+/CD3+CD8+	AnuA	0.414176	0
B19	B108	CD3+CD4+/CD3+CD8+	Anti-Cyclin	0.414177	0
B19	B109	CD3+CD4+/CD3+CD8+	AMA-M2	0.414177	0
B19	B110	CD3+CD4+/CD3+CD8+	AHA	0.414177	0
B19	B119	CD3+CD4+/CD3+CD8+	Hcv-rna	0.319469	0
B19	B120	CD3+CD4+/CD3+CD8+	HCV-cAg	0.225309	0
B19	B121	CD3+CD4+/CD3+CD8+	C3	-0.33505	0
B19	B122	CD3+CD4+/CD3+CD8+	C4	0.308411	0
B19	B125	CD3+CD4+/CD3+CD8+	Cor	0.3612	0
B19	B151	CD3+CD4+/CD3+CD8+	Anti-CCP	0.2498	0
B19	B152	CD3+CD4+/CD3+CD8+	ACA-Ig(G-A-M)	-0.31953	0
B19	B157	CD3+CD4+/CD3+CD8+	IgG	0.28412	0
B19	B159	CD3+CD4+/CD3+CD8+	β2-MG	-0.31947	0
B19	B181	CD3+CD4+/CD3+CD8+	APTT	0.383382	0
B19	B20	CD3+CD4+/CD3+CD8+	CD3+CD4+CD8+	-0.23686	0
B19	B21	CD3+CD4+/CD3+CD8+	CD3+CD4-CD8-	0.313647	0
B19	B22	CD3+CD4+/CD3+CD8+	CD3+CD8+(T)	-0.84406	0
B19	B23	CD3+CD4+/CD3+CD8+	CD3-CD16+CD56+(NK)	-0.24149	0
B19	B55	CD3+CD4+/CD3+CD8+	TK	0.226431	0
B19	B79	CD3+CD4+/CD3+CD8+	hb V -dna	0.260055	0
B19	B92	CD3+CD4+/CD3+CD8+	ALDH2-Glu504Glu	-0.23408	0
B19	B93	CD3+CD4+/CD3+CD8+	ALDH2-Glu504Lys	0.217927	0
B19	B94	CD3+CD4+/CD3+CD8+	ALDH2-Lys504Lys	0.319469	0
B19	B95	CD3+CD4+/CD3+CD8+	anti-cenpb	0.414177	0
B19	B97	CD3+CD4+/CD3+CD8+	dsDNA	0.414177	0
B19	B98	CD3+CD4+/CD3+CD8+	anti-Jo-1	0.414177	0
B19	B99	CD3+CD4+/CD3+CD8+	anti-PM-Scl	0.414177	0

B19	B137	CD3+CD4+/CD3+CD8+	AFU	0.16206	1.33E-11
B19	B138	CD3+CD4+/CD3+CD8+	PAB	-0.154	1.33E-10
B19	B58	CD3+CD4+/CD3+CD8+	HCT	-0.15399	1.33E-10
B19	B199	CD3+CD4+/CD3+CD8+	Mg	-0.14533	1.37E-09
B19	B184	CD3+CD4+/CD3+CD8+	INR	0.134719	2.00E-08
B19	B134	CD3+CD4+/CD3+CD8+	CHE	-0.12728	1.16E-07
B19	B158	CD3+CD4+/CD3+CD8+	IgM	-0.12247	3.44E-07
B19	B34	CD3+CD4+/CD3+CD8+	TK	0.121468	4.29E-07
B190	B191	Cre	Urea	0.527548	0
B190	B192	Cre	UA	0.434368	0
B190	B204	Cre	Drinking	0.21324	0
B190	B206	Cre	Waist	0.257772	0
B190	B209	Cre	Smoking	0.214933	0
B190	B210	Cre	Hip	0.197378	0
B190	B216	Cre	BMI	0.309412	0
B190	B221	Cre	Hei	0.32023	0
B190	B222	Cre	NS	0.361433	0
B190	B215	Cre	BMI	0.184282	1.29E-14
B190	B207	Cre	WHR	0.177891	1.04E-13
B190	B227	Cre	OD	0.153525	1.51E-10
B191	B192	Urea	UA	0.178154	9.55E-14
B192	B204	UA	Drinking	0.232069	0
B192	B206	UA	Waist	0.38464	0
B192	B207	UA	WHR	0.261741	0
B192	B210	UA	Hip	0.270493	0
B192	B215	UA	BMI	0.31654	0
B192	B216	UA	BMI	0.424406	0
B192	B221	UA	Hei	0.350105	0
B192	B222	UA	NS	0.454056	0
B192	B209	UA	Smoking	0.182383	2.40E-14
B193	B200	K	OP	-0.1371	1.12E-08
B193	B196	K	TCO2	0.137059	1.13E-08
B194	B195	Cl	Na	0.442314	0
B194	B197	Cl	AG	-0.40625	0
B194	B200	Cl	OP	0.315895	0
B195	B197	Na	AG	0.217881	0
B195	B200	Na	OP	0.839185	0
B196	B197	TCO2	AG	-0.75857	0
B196	B199	TCO2	Mg	0.308633	0
B197	B199	AG	Mg	-0.2459	0
B197	B200	AG	OP	0.201614	0
B2	B113	Age	FT3	-0.266	0
B2	B126	Age	Alb	-0.36802	0
B2	B127	Age	A-G ratio	-0.22343	0

B2	B128	Age	ALT	-0.25272	0
B2	B141	Age	AST/ALT	0.257824	0
B2	B163	Age	SG	-0.31725	0
B2	B176	Age	Ucr	-0.39564	0
B2	B189	Age	eGFB	-0.61091	0
B2	B191	Age	Urea	0.216124	0
B2	B216	Age	BMI	-0.24167	0
B2	B218	Age	DBP	-0.22227	0
B2	B219	Age	OT	0.204399	0
B2	B220	Age	SBP	0.402249	0
B2	B221	Age	Hei	-0.28123	0
B2	B46	Age	RBC Count	-0.32003	0
B2	B47	Age	RDW	0.323647	0
B2	B51	Age	Hb	-0.31985	0
B2	B71	Age	TG	-0.18966	2.22E-15
B2	B118	Age	CEA1	0.183785	1.51E-14
B2	B70	Age	LDL-C	-0.18161	3.11E-14
B2	B77	Age	TC	-0.17607	1.86E-13
B2	B190	Age	Cre	0.175734	2.07E-13
B2	B140	Age	r-GT	-0.17506	2.56E-13
B2	B41	Age	MON％	0.169738	1.34E-12
B2	B45	Age	MCHC	-0.1696	1.40E-12
B2	B201	Age	e-habits	0.16904	1.66E-12
B2	B222	Age	NS	-0.16574	4.50E-12
B2	B52	Age	PLT/BPC	-0.15913	3.12E-11
B2	B138	Age	PAB	-0.14995	4.02E-10
B2	B43	Age	MCV	0.143808	2.04E-09
B2	B134	Age	CHE	-0.14101	4.18E-09
B2	B72	Age	HDL-C	0.139257	6.52E-09
B2	B153	Age	Glu	0.13729	1.06E-08
B2	B91	Age	TP	-0.13025	5.82E-08
B2	B210	Age	Hip	-0.12444	2.22E-07
B2	B27	Age	LDH	0.123156	2.95E-07
B2	B227	Age	OD	0.122456	3.45E-07
B2	B79	Age	hb V -dna	0.118474	8.22E-07
B2	B132	Age	Dbil	0.118431	8.29E-07
B2	B146	Age	CysC	0.118328	8.48E-07
B20	B151	CD3+CD4+CD8+	Anti-CCP	0.198094	0
B20	B181	CD3+CD4+CD8+	APTT	-0.45473	0
B20	B182	CD3+CD4+CD8+	TT	0.308778	0
B20	B184	CD3+CD4+CD8+	INR	-0.21951	0
B20	B21	CD3+CD4+CD8+	CD3+CD4-CD8-	-0.29869	0
B20	B23	CD3+CD4+CD8+	CD3-CD16+CD56+(NK)	0.402624	0
B20	B25	CD3+CD4+CD8+	CK	-0.44315	0

B20	B57	CD3+CD4+CD8+	RBCAI	0.234848	0
B20	B155	CD3+CD4+CD8+	IgA	0.196068	2.22E-16
B20	B125	CD3+CD4+CD8+	Cor	-0.18592	7.55E-15
B20	B101	CD3+CD4+CD8+	anti-Sc70	0.184252	1.29E-14
B20	B102	CD3+CD4+CD8+	anti-Sm	0.184252	1.29E-14
B20	B103	CD3+CD4+CD8+	anti-SSA/Ro	0.184252	1.29E-14
B20	B104	CD3+CD4+CD8+	anti-SSB	0.184252	1.29E-14
B20	B105	CD3+CD4+CD8+	anti-U1RNP	0.184252	1.29E-14
B20	B106	CD3+CD4+CD8+	anti-P	0.184252	1.29E-14
B20	B107	CD3+CD4+CD8+	AnuA	0.184252	1.29E-14
B20	B108	CD3+CD4+CD8+	Anti-Cyclin	0.184252	1.29E-14
B20	B109	CD3+CD4+CD8+	AMA-M2	0.184252	1.29E-14
B20	B110	CD3+CD4+CD8+	AHA	0.184252	1.29E-14
B20	B95	CD3+CD4+CD8+	anti-cenpb	0.184252	1.29E-14
B20	B97	CD3+CD4+CD8+	dsDNA	0.184252	1.29E-14
B20	B98	CD3+CD4+CD8+	anti-Jo-1	0.184252	1.29E-14
B20	B99	CD3+CD4+CD8+	anti-PM-Scl	0.184252	1.29E-14
B20	B183	CD3+CD4+CD8+	PTA	-0.18203	2.71E-14
B20	B152	CD3+CD4+CD8+	ACA-Ig(G-A-M)	0.169831	1.30E-12
B20	B119	CD3+CD4+CD8+	Hcv-rna	-0.1698	1.32E-12
B20	B159	CD3+CD4+CD8+	β2-MG	0.169795	1.32E-12
B20	B94	CD3+CD4+CD8+	ALDH2-Lys504Lys	-0.1698	1.32E-12
B20	B193	CD3+CD4+CD8+	K	0.157749	4.62E-11
B20	B186	CD3+CD4+CD8+	PFC	0.153386	1.57E-10
B20	B185	CD3+CD4+CD8+	PT	-0.15171	2.49E-10
B20	B195	CD3+CD4+CD8+	Na	0.150496	3.47E-10
B20	B156	CD3+CD4+CD8+	IgE	0.142264	3.04E-09
B20	B55	CD3+CD4+CD8+	TK	-0.14081	4.41E-09
B20	B199	CD3+CD4+CD8+	Mg	0.140415	4.87E-09
B20	B79	CD3+CD4+CD8+	hb V -dna	-0.13907	6.83E-09
B20	B56	CD3+CD4+CD8+	IR	-0.13854	7.80E-09
B20	B200	CD3+CD4+CD8+	OP	0.131168	4.69E-08
B20	B61	CD3+CD4+CD8+	WBHSRV	-0.12741	1.13E-07
B20	B92	CD3+CD4+CD8+	ALDH2-Glu504Glu	0.123226	2.91E-07
B20	B59	CD3+CD4+CD8+	WBRV	0.121556	4.21E-07
B20	B176	CD3+CD4+CD8+	Ucr	-0.11831	8.52E-07
B201	B204	e-habits	Drinking	0.266674	0
B201	B209	e-habits	Smoking	0.167425	2.71E-12
B202	B223	e-times	FH	0.565128	0
B204	B206	Drinking	Waist	0.215797	0
B204	B209	Drinking	Smoking	0.473199	0
B204	B216	Drinking	BMI	0.254788	0
B204	B221	Drinking	Hei	0.278206	0
B204	B222	Drinking	NS	0.291659	0

B204	B210	Drinking	Hip	0.160512	2.09E-11
B204	B207	Drinking	WHR	0.15006	3.90E-10
B204	B215	Drinking	BMI	0.13695	1.16E-08
B206	B207	Waist	WHR	0.699355	0
B206	B209	Waist	Smoking	0.207289	0
B206	B210	Waist	Hip	0.739727	0
B206	B215	Waist	BMI	0.819736	0
B206	B216	Waist	BMI	0.832963	0
B206	B221	Waist	Hei	0.429444	0
B206	B222	Waist	NS	0.747318	0
B206	B218	Waist	DBP	0.150164	3.79E-10
B206	B220	Waist	SBP	0.136262	1.37E-08
B207	B210	WHR	Hip	0.257019	0
B207	B215	WHR	BMI	0.455456	0
B207	B216	WHR	BMI	0.454879	0
B207	B221	WHR	Hei	0.226686	0
B207	B222	WHR	NS	0.488376	0
B207	B209	WHR	Smoking	0.149229	4.88E-10
B207	B220	WHR	SBP	0.133858	2.46E-08
B209	B216	Smoking	BMI	0.220757	0
B209	B221	Smoking	Hei	0.260486	0
B209	B222	Smoking	NS	0.297237	0
B209	B210	Smoking	Hip	0.125563	1.72E-07
B21	B101	CD3+CD4-CD8-	anti-Sc70	0.41225	0
B21	B102	CD3+CD4-CD8-	anti-Sm	0.41225	0
B21	B103	CD3+CD4-CD8-	anti-SSA/Ro	0.41225	0
B21	B104	CD3+CD4-CD8-	anti-SSB	0.41225	0
B21	B105	CD3+CD4-CD8-	anti-U1RNP	0.41225	0
B21	B106	CD3+CD4-CD8-	anti-P	0.41225	0
B21	B107	CD3+CD4-CD8-	AnuA	0.412249	0
B21	B108	CD3+CD4-CD8-	Anti-Cyclin	0.41225	0
B21	B109	CD3+CD4-CD8-	AMA-M2	0.41225	0
B21	B110	CD3+CD4-CD8-	AHA	0.41225	0
B21	B119	CD3+CD4-CD8-	Hcv-rna	0.463911	0
B21	B120	CD3+CD4-CD8-	HCV-cAg	0.240167	0
B21	B125	CD3+CD4-CD8-	Cor	0.53427	0
B21	B134	CD3+CD4-CD8-	CHE	-0.22053	0
B21	B136	CD3+CD4-CD8-	5'NT	0.27851	0
B21	B151	CD3+CD4-CD8-	Anti-CCP	0.27979	0
B21	B152	CD3+CD4-CD8-	ACA-Ig(G-A-M)	-0.464	0
B21	B157	CD3+CD4-CD8-	IgG	0.276216	0
B21	B158	CD3+CD4-CD8-	IgM	-0.38878	0
B21	B159	CD3+CD4-CD8-	β2-MG	-0.46391	0
B21	B184	CD3+CD4-CD8-	INR	0.198245	0

B21	B194	CD3+CD4-CD8-	Cl	-0.2029	0
B21	B197	CD3+CD4-CD8-	AG	0.239999	0
B21	B198	CD3+CD4-CD8-	P	0.233654	0
B21	B23	CD3+CD4-CD8-	CD3-CD16+CD56+(NK)	-0.25681	0
B21	B58	CD3+CD4-CD8-	HCT	-0.28226	0
B21	B79	CD3+CD4-CD8-	hb V -dna	0.378355	0
B21	B92	CD3+CD4-CD8-	ALDH2-Glu504Glu	-0.33891	0
B21	B93	CD3+CD4-CD8-	ALDH2-Glu504Lys	0.315446	0
B21	B94	CD3+CD4-CD8-	ALDH2-Lys504Lys	0.463911	0
B21	B95	CD3+CD4-CD8-	anti-cenpb	0.41225	0
B21	B97	CD3+CD4-CD8-	dsDNA	0.41225	0
B21	B98	CD3+CD4-CD8-	anti-Jo-1	0.41225	0
B21	B99	CD3+CD4-CD8-	anti-PM-Scl	0.41225	0
B21	B156	CD3+CD4-CD8-	IgE	0.193874	4.44E-16
B21	B196	CD3+CD4-CD8-	TCO2	-0.19198	8.88E-16
B21	B22	CD3+CD4-CD8-	CD3+CD8+(T)	-0.17942	6.35E-14
B21	B114	CD3+CD4-CD8-	TSH TT3 TT4(TT4)	0.178523	8.48E-14
B21	B183	CD3+CD4-CD8-	PTA	-0.17256	5.60E-13
B21	B81	CD3+CD4-CD8-	TSGF	0.167413	2.72E-12
B21	B62	CD3+CD4-CD8-	WBHSRVI	-0.16622	3.89E-12
B21	B75	CD3+CD4-CD8-	ApoA1/B	0.146481	1.01E-09
B21	B66	CD3+CD4-CD8-	WBRSR(mPas_100)	-0.14406	1.91E-09
B21	B203	CD3+CD4-CD8-	DH	-0.14174	3.47E-09
B21	B193	CD3+CD4-CD8-	K	0.141092	4.10E-09
B21	B67	CD3+CD4-CD8-	WBRSR(mPas_50)	-0.14072	4.50E-09
B21	B55	CD3+CD4-CD8-	TK	0.138477	7.93E-09
B21	B74	CD3+CD4-CD8-	ApoA1	0.137956	9.03E-09
B21	B63	CD3+CD4-CD8-	WBRSR(mPas_200)	-0.12637	1.43E-07
B21	B181	CD3+CD4-CD8-	APTT	0.124483	2.19E-07
B21	B76	CD3+CD4-CD8-	ApoB	-0.11945	6.66E-07
B21	B112	CD3+CD4-CD8-	FT4	0.118207	8.70E-07
B210	B215	Hip	BMI	0.749414	0
B210	B216	Hip	BMI	0.771012	0
B210	B221	Hip	Hei	0.409209	0
B210	B222	Hip	NS	0.61652	0
B210	B218	Hip	DBP	0.158072	4.22E-11
B211	B212	IOP-R	IOP-L	0.77683	0
B215	B216	BMI	BMI	0.827869	0
B215	B222	BMI	NS	0.672988	0
B215	B218	BMI	DBP	0.193681	4.44E-16
B215	B221	BMI	Hei	0.194239	4.44E-16
B216	B218	BMI	DBP	0.24158	0
B216	B221	BMI	Hei	0.70549	0
B216	B222	BMI	NS	0.825704	0

B218	B220	DBP	SBP	0.461762	0
B218	B222	DBP	NS	0.196799	2.22E-16
B218	B221	DBP	Hei	0.168409	2.01E-12
B22	B101	CD3+CD8+(T)	anti-Sc70	-0.37136	0
B22	B102	CD3+CD8+(T)	anti-Sm	-0.37136	0
B22	B103	CD3+CD8+(T)	anti-SSA/Ro	-0.37136	0
B22	B104	CD3+CD8+(T)	anti-SSB	-0.37136	0
B22	B105	CD3+CD8+(T)	anti-U1RNP	-0.37136	0
B22	B106	CD3+CD8+(T)	anti-P	-0.37136	0
B22	B107	CD3+CD8+(T)	AnuA	-0.37136	0
B22	B108	CD3+CD8+(T)	Anti-Cyclin	-0.37136	0
B22	B109	CD3+CD8+(T)	AMA-M2	-0.37136	0
B22	B110	CD3+CD8+(T)	AHA	-0.37136	0
B22	B115	CD3+CD8+(T)	TSH TT3 TT4(TT3)	0.244994	0
B22	B119	CD3+CD8+(T)	Hcv-rna	-0.47249	0
B22	B120	CD3+CD8+(T)	HCV-cAg	-0.25295	0
B22	B121	CD3+CD8+(T)	C3	0.211997	0
B22	B124	CD3+CD8+(T)	ACTH	-0.35063	0
B22	B125	CD3+CD8+(T)	Cor	-0.57676	0
B22	B151	CD3+CD8+(T)	Anti-CCP	-0.22884	0
B22	B152	CD3+CD8+(T)	ACA-Ig(G-A-M)	0.47258	0
B22	B156	CD3+CD8+(T)	IgE	-0.21348	0
B22	B158	CD3+CD8+(T)	IgM	0.348529	0
B22	B159	CD3+CD8+(T)	β2-MG	0.472481	0
B22	B179	CD3+CD8+(T)	α1-MG	0.208477	0
B22	B181	CD3+CD8+(T)	APTT	-0.23703	0
B22	B23	CD3+CD8+(T)	CD3-CD16+CD56+(NK)	-0.25854	0
B22	B55	CD3+CD8+(T)	TK	-0.22602	0
B22	B58	CD3+CD8+(T)	HCT	0.284287	0
B22	B79	CD3+CD8+(T)	hb V -dna	-0.38508	0
B22	B92	CD3+CD8+(T)	ALDH2-Glu504Glu	0.345549	0
B22	B93	CD3+CD8+(T)	ALDH2-Glu504Lys	-0.32166	0
B22	B94	CD3+CD8+(T)	ALDH2-Lys504Lys	-0.47249	0
B22	B95	CD3+CD8+(T)	anti-cenpb	-0.37136	0
B22	B97	CD3+CD8+(T)	dsDNA	-0.37136	0
B22	B98	CD3+CD8+(T)	anti-Jo-1	-0.37136	0
B22	B99	CD3+CD8+(T)	anti-PM-Scl	-0.37136	0
B22	B155	CD3+CD8+(T)	IgA	-0.18673	5.77E-15
B22	B138	CD3+CD8+(T)	PAB	0.182043	2.69E-14
B22	B66	CD3+CD8+(T)	WBRSR(mPas_100)	0.178676	8.06E-14
B22	B199	CD3+CD8+(T)	Mg	0.177844	1.05E-13
B22	B67	CD3+CD8+(T)	WBRSR(mPas_50)	0.175664	2.11E-13
B22	B122	CD3+CD8+(T)	C4	-0.1725	5.72E-13
B22	B81	CD3+CD8+(T)	TSGF	-0.17079	9.72E-13

B22	B182	CD3+CD8+(T)	TT	-0.15453	1.15E-10
B22	B157	CD3+CD8+(T)	IgG	-0.15205	2.27E-10
B22	B75	CD3+CD8+(T)	ApoA1/B	-0.15204	2.27E-10
B22	B63	CD3+CD8+(T)	WBRSR(mPas_200)	0.148377	6.13E-10
B22	B64	CD3+CD8+(T)	WBRSR(mPas_5)	0.14563	1.27E-09
B22	B203	CD3+CD8+(T)	DH	0.143906	1.99E-09
B22	B74	CD3+CD8+(T)	ApoA1	-0.1398	5.69E-09
B22	B137	CD3+CD8+(T)	AFU	-0.13944	6.22E-09
B22	B62	CD3+CD8+(T)	WBHSRVI	0.136924	1.17E-08
B22	B113	CD3+CD8+(T)	FT3	0.136037	1.45E-08
B22	B134	CD3+CD8+(T)	CHE	0.135434	1.68E-08
B22	B25	CD3+CD8+(T)	CK	0.124602	2.14E-07
B220	B221	SBP	Hei	-0.12401	2.44E-07
B221	B222	Hei	NS	0.600798	0
B23	B115	CD3-CD16+CD56+(NK)	TSH TT3 TT4(TT3)	-0.21814	0
B23	B119	CD3-CD16+CD56+(NK)	Hcv-rna	0.246572	0
B23	B121	CD3-CD16+CD56+(NK)	C3	0.226825	0
B23	B122	CD3-CD16+CD56+(NK)	C4	-0.24755	0
B23	B124	CD3-CD16+CD56+(NK)	ACTH	0.578942	0
B23	B125	CD3-CD16+CD56+(NK)	Cor	0.353449	0
B23	B152	CD3-CD16+CD56+(NK)	ACA-Ig(G-A-M)	-0.24662	0
B23	B155	CD3-CD16+CD56+(NK)	IgA	0.433744	0
B23	B158	CD3-CD16+CD56+(NK)	IgM	-0.38221	0
B23	B159	CD3-CD16+CD56+(NK)	β2-MG	-0.24657	0
B23	B179	CD3-CD16+CD56+(NK)	α1-MG	-0.30437	0
B23	B181	CD3-CD16+CD56+(NK)	APTT	-0.22842	0
B23	B182	CD3-CD16+CD56+(NK)	TT	0.249971	0
B23	B183	CD3-CD16+CD56+(NK)	PTA	0.235656	0
B23	B58	CD3-CD16+CD56+(NK)	HCT	-0.20716	0
B23	B76	CD3-CD16+CD56+(NK)	ApoB	-0.20225	0
B23	B79	CD3-CD16+CD56+(NK)	hb V -dna	0.201465	0
B23	B94	CD3-CD16+CD56+(NK)	ALDH2-Lys504Lys	0.246572	0
B23	B178	CD3-CD16+CD56+(NK)	Ig-U	-0.18817	3.55E-15
B23	B157	CD3-CD16+CD56+(NK)	IgG	-0.18045	4.53E-14
B23	B92	CD3-CD16+CD56+(NK)	ALDH2-Glu504Glu	-0.17962	5.95E-14
B23	B62	CD3-CD16+CD56+(NK)	WBHSRVI	-0.17669	1.52E-13
B23	B63	CD3-CD16+CD56+(NK)	WBRSR(mPas_200)	-0.16723	2.87E-12
B23	B93	CD3-CD16+CD56+(NK)	ALDH2-Glu504Lys	0.167148	2.95E-12
B23	B66	CD3-CD16+CD56+(NK)	WBRSR(mPas_100)	-0.16237	1.22E-11
B23	B73	CD3-CD16+CD56+(NK)	ApoA	0.159899	2.50E-11
B23	B186	CD3-CD16+CD56+(NK)	PFC	-0.15544	8.87E-11
B23	B75	CD3-CD16+CD56+(NK)	ApoA1/B	0.152418	2.05E-10
B23	B67	CD3-CD16+CD56+(NK)	WBRSR(mPas_50)	-0.15122	2.85E-10
B23	B25	CD3-CD16+CD56+(NK)	CK	-0.1509	3.10E-10

B23	B56	CD3-CD16+CD56+(NK)	IR	-0.13993	5.50E-09
B23	B61	CD3-CD16+CD56+(NK)	WBHSRV	-0.12967	6.68E-08
B23	B156	CD3-CD16+CD56+(NK)	IgE	0.125961	1.57E-07
B23	B185	CD3-CD16+CD56+(NK)	PT	-0.12525	1.85E-07
B24	B27	αHBDH	LDH	0.192947	6.66E-16
B24	B25	αHBDH	CK	0.169385	1.49E-12
B24	B74	αHBDH	ApoA1	0.1295	6.94E-08
B24	B76	αHBDH	ApoB	0.127235	1.17E-07
B25	B181	CK	APTT	0.249118	0
B25	B114	CK	TSH TT3 TT4(TT4)	0.183203	1.82E-14
B25	B134	CK	CHE	-0.16299	1.02E-11
B25	B196	CK	TCO2	0.158775	3.45E-11
B25	B34	CK	TK	0.1442	1.84E-09
B25	B136	CK	5'NT	-0.14051	4.75E-09
B25	B186	CK	PFC	-0.12898	7.83E-08
B25	B197	CK	AG	-0.12639	1.42E-07
B25	B182	CK	TT	-0.12426	2.30E-07
B25	B135	CK	TBA	0.12128	4.47E-07
B27	B38	LDH	BAND%	0.406565	0
B27	B37	LDH	BAND	-0.17353	4.15E-13
B27	B150	LDH	ALP	0.160651	2.01E-11
B27	B47	LDH	RDW	0.147995	6.79E-10
B27	B176	LDH	Ucr	-0.13186	3.97E-08
B28	B125	PRL	Cor	0.29953	0
B28	B29	PRL	E2	0.99974	0
B28	B30	PRL	LH	-0.96577	0
B28	B31	PRL	FSH	-1	0
B28	B32	PRL	Testo	0.99984	0
B28	B33	PRL	Prog	0.999776	0
B29	B125	E2	Cor	0.292876	0
B29	B30	E2	LH	-0.95961	0
B29	B31	E2	FSH	-0.99975	0
B29	B32	E2	Testo	0.999171	0
B29	B33	E2	Prog	0.999999	0
B3	B190	Hcy	Cre	0.185556	8.44E-15
B3	B189	Hcy	eGFB	-0.14968	4.32E-10
B3	B222	Hcy	NS	0.12267	3.29E-07
B3	B216	Hcy	BMI	0.122186	3.66E-07
B3	B221	Hcy	Hei	0.120689	5.09E-07
B30	B125	LH	Cor	-0.36406	0
B30	B31	LH	FSH	0.965667	0
B30	B32	LH	Testo	-0.97026	0
B30	B33	LH	Prog	-0.96007	0
B30	B143	LH	β-CTx	0.122239	3.62E-07

B30	B134	LH	CHE	0.121081	4.67E-07
B30	B145	LH	BGP	0.118718	7.80E-07
B31	B125	FSH	Cor	-0.29941	0
B31	B32	FSH	Testo	-0.99983	0
B31	B33	FSH	Prog	-0.99978	0
B32	B125	Testo	Cor	0.304643	0
B32	B33	Testo	Prog	0.999237	0
B33	B125	Prog	Cor	0.293361	0
B34	B101	TK	anti-Sc70	0.185289	9.10E-15
B34	B102	TK	anti-Sm	0.185289	9.10E-15
B34	B103	TK	anti-SSA/Ro	0.185289	9.10E-15
B34	B104	TK	anti-SSB	0.185289	9.10E-15
B34	B105	TK	anti-U1RNP	0.185289	9.10E-15
B34	B106	TK	anti-P	0.185289	9.10E-15
B34	B107	TK	AnuA	0.185289	9.10E-15
B34	B108	TK	Anti-Cyclin	0.185289	9.10E-15
B34	B109	TK	AMA-M2	0.185289	9.10E-15
B34	B110	TK	AHA	0.185289	9.10E-15
B34	B95	TK	anti-cenpb	0.185289	9.10E-15
B34	B97	TK	dsDNA	0.185289	9.10E-15
B34	B98	TK	anti-Jo-1	0.185289	9.10E-15
B34	B99	TK	anti-PM-Scl	0.185289	9.10E-15
B34	B193	TK	K	0.173809	3.80E-13
B34	B196	TK	TCO2	0.170099	1.20E-12
B34	B157	TK	IgG	0.169298	1.53E-12
B34	B197	TK	AG	-0.15786	4.47E-11
B34	B78	TK	HBcAb	0.155361	9.07E-11
B34	B93	TK	ALDH2-Glu504Lys	-0.15456	1.13E-10
B34	B114	TK	TSH TT3 TT4(TT4)	0.153617	1.47E-10
B34	B136	TK	5'NT	-0.1513	2.78E-10
B34	B92	TK	ALDH2-Glu504Glu	0.14825	6.34E-10
B34	B54	TK	ESR	0.137018	1.14E-08
B34	B138	TK	PAB	-0.13233	3.56E-08
B34	B200	TK	OP	-0.12401	2.44E-07
B35	B117	β2-MG	TT4	0.988317	0
B35	B202	β2-MG	e-times	0.634161	0
B36	B227	V-Lym%	OD	-0.1833	1.78E-14
B37	B38	BAND	BAND%	0.355575	0
B39	B44	Other cells	MCH	-0.16927	1.55E-12
B39	B43	Other cells	MCV	-0.16546	4.89E-12
B4	B181	WB-Ca	APTT	0.333728	0
B4	B184	WB-Ca	INR	0.406803	0
B4	B185	WB-Ca	PT	0.230644	0
B4	B5	WB-Ca	WB-Mg	0.260266	0

B4	B6	WB-Ca	WB-Fe	0.849105	0
B4	B7	WB-Ca	WB-Cop	0.820412	0
B4	B193	WB-Ca	K	-0.18583	7.55E-15
B4	B8	WB-Ca	WB-Zinc	0.185112	9.77E-15
B4	B136	WB-Ca	5'NT	0.169805	1.31E-12
B4	B186	WB-Ca	PFC	-0.16375	8.12E-12
B4	B182	WB-Ca	TT	-0.137	1.14E-08
B4	B157	WB-Ca	IgG	-0.12967	6.66E-08
B40	B206	WBC	Waist	0.200841	0
B40	B222	WBC	NS	0.215308	0
B40	B42	WBC	MON	0.678988	0
B40	B46	WBC	RBC Count	0.218887	0
B40	B51	WBC	Hb	0.218621	0
B40	B52	WBC	PLT/BPC	0.238041	0
B40	B72	WBC	HDL-C	-0.19798	0
B40	B216	WBC	BMI	0.196833	2.22E-16
B40	B53	WBC	GR%	0.193612	4.44E-16
B40	B192	WBC	UA	0.189147	2.44E-15
B40	B48	WBC	LYM%	-0.17902	7.22E-14
B40	B215	WBC	BMI	0.17884	7.64E-14
B40	B209	WBC	Smoking	0.172646	5.46E-13
B40	B207	WBC	WHR	0.147114	8.58E-10
B40	B41	WBC	MON %	-0.13979	5.70E-09
B40	B128	WBC	ALT	0.133982	2.39E-08
B40	B210	WBC	Hip	0.131868	3.97E-08
B40	B221	WBC	Hei	0.123132	2.97E-07
B41	B42	MON %	MON	0.603857	0
B41	B53	MON %	GR%	-0.21334	0
B41	B126	MON %	Alb	-0.17767	1.11E-13
B41	B190	MON %	Cre	0.168484	1.97E-12
B41	B189	MON %	eGFB	-0.164	7.54E-12
B41	B47	MON %	RDW	0.143145	2.42E-09
B41	B49	MON %	baso%	0.130222	5.86E-08
B41	B204	MON %	Drinking	0.121887	3.91E-07
B41	B209	MON %	Smoking	0.121759	4.02E-07
B42	B190	MON	Cre	0.207257	0
B42	B192	MON	UA	0.197821	0
B42	B206	MON	Waist	0.209904	0
B42	B209	MON	Smoking	0.222968	0
B42	B222	MON	NS	0.221241	0
B42	B216	MON	BMI	0.176438	1.65E-13
B42	B51	MON	Hb	0.173167	4.64E-13
B42	B207	MON	WHR	0.156828	6.00E-11
B42	B72	MON	HDL-C	-0.15578	8.06E-11

B42	B204	MON	Drinking	0.148508	5.92E-10
B42	B215	MON	BMI	0.147847	7.06E-10
B42	B46	MON	RBC Count	0.146086	1.13E-09
B42	B48	MON	LYM%	-0.1457	1.25E-09
B42	B189	MON	eGFB	-0.13212	3.74E-08
B42	B221	MON	Hei	0.129229	7.40E-08
B42	B210	MON	Hip	0.126748	1.31E-07
B42	B52	MON	PLT/BPC	0.122605	3.34E-07
B43	B44	MCV	MCH	0.871328	0
B43	B46	MCV	RBC Count	-0.54705	0
B43	B47	MCV	RDW	0.587097	0
B43	B52	MCV	PLT/BPC	-0.15471	1.09E-10
B43	B218	MCV	DBP	-0.12451	2.18E-07
B43	B189	MCV	eGFB	-0.12381	2.55E-07
B44	B45	MCH	MCHC	0.547262	0
B44	B46	MCH	RBC Count	-0.42046	0
B44	B47	MCH	RDW	0.377826	0
B44	B51	MCH	Hb	0.208038	0
B44	B52	MCH	PLT/BPC	-0.13391	2.43E-08
B45	B216	MCHC	BMI	0.227695	0
B45	B221	MCHC	Hei	0.28203	0
B45	B222	MCHC	NS	0.267349	0
B45	B47	MCHC	RDW	-0.24607	0
B45	B51	MCHC	Hb	0.446771	0
B45	B71	MCHC	TG	0.292903	0
B45	B72	MCHC	HDL-C	-0.19357	4.44E-16
B45	B192	MCHC	UA	0.188547	3.11E-15
B45	B129	MCHC	IBIL	0.180162	4.97E-14
B45	B133	MCHC	TBIL	0.175539	2.20E-13
B45	B163	MCHC	SG	0.155057	9.87E-11
B45	B174	MCHC	Urea/Cr	-0.1416	3.60E-09
B45	B206	MCHC	Waist	0.132368	3.52E-08
B45	B126	MCHC	Alb	0.126626	1.35E-07
B45	B141	MCHC	AST/ALT	-0.11893	7.45E-07
B46	B126	RBC Count	Alb	0.253137	0
B46	B141	RBC Count	AST/ALT	-0.21297	0
B46	B163	RBC Count	SG	0.264573	0
B46	B174	RBC Count	Urea/Cr	-0.24991	0
B46	B189	RBC Count	eGFB	0.203754	0
B46	B192	RBC Count	UA	0.197544	0
B46	B206	RBC Count	Waist	0.212292	0
B46	B215	RBC Count	BMI	0.208201	0
B46	B216	RBC Count	BMI	0.351322	0
B46	B218	RBC Count	DBP	0.274319	0

B46	B221	RBC Count	Hei	0.352341	0
B46	B222	RBC Count	NS	0.340002	0
B46	B47	RBC Count	RDW	-0.45641	0
B46	B51	RBC Count	Hb	0.788157	0
B46	B72	RBC Count	HDL-C	-0.22218	0
B46	B113	RBC Count	FT3	0.190221	1.78E-15
B46	B128	RBC Count	ALT	0.188405	3.11E-15
B46	B129	RBC Count	IBIL	0.182309	2.46E-14
B46	B191	RBC Count	Urea	-0.18068	4.22E-14
B46	B176	RBC Count	Ucr	0.177226	1.28E-13
B46	B210	RBC Count	Hip	0.170653	1.01E-12
B46	B133	RBC Count	TBIL	0.164194	7.12E-12
B46	B71	RBC Count	TG	0.152478	2.02E-10
B46	B207	RBC Count	WHR	0.144876	1.55E-09
B46	B91	RBC Count	TP	0.131257	4.59E-08
B46	B70	RBC Count	LDL-C	0.128661	8.44E-08
B46	B209	RBC Count	Smoking	0.123893	2.50E-07
B46	B161	RBC Count	LEU	-0.12338	2.81E-07
B46	B58	RBC Count	HCT	0.121012	4.74E-07
B46	B52	RBC Count	PLT/BPC	0.117644	9.81E-07
B47	B189	RDW	eGFB	-0.26237	0
B47	B51	RDW	Hb	-0.26061	0
B47	B52	RDW	PLT/BPC	-0.19351	4.44E-16
B47	B191	RDW	Urea	0.175583	2.17E-13
B47	B126	RDW	Alb	-0.15288	1.81E-10
B47	B218	RDW	DBP	-0.14292	2.57E-09
B47	B190	RDW	Cre	0.141588	3.61E-09
B47	B163	RDW	SG	-0.13306	2.99E-08
B47	B185	RDW	PT	0.132029	3.82E-08
B47	B221	RDW	Hei	-0.12557	1.72E-07
B47	B184	RDW	INR	0.124922	1.99E-07
B47	B176	RDW	Ucr	-0.12439	2.24E-07
B47	B72	RDW	HDL-C	0.121601	4.17E-07
B47	B216	RDW	BMI	-0.12025	5.60E-07
B48	B53	LYM%	GR%	-0.93483	0
B48	B77	LYM%	TC	0.158457	3.78E-11
B48	B192	LYM%	UA	-0.14214	3.14E-09
B48	B72	LYM%	HDL-C	0.13705	1.13E-08
B48	B190	LYM%	Cre	-0.13193	3.91E-08
B48	B189	LYM%	eGFB	0.12825	9.28E-08
B48	B207	LYM%	WHR	-0.1257	1.67E-07
B48	B206	LYM%	Waist	-0.12383	2.54E-07
B48	B153	LYM%	Glu	-0.11912	7.15E-07
B48	B91	LYM%	TP	0.118193	8.73E-07

B49	B50	baso%	eo%	0.258069	0
B49	B53	baso%	GR%	-0.22323	0
B5	B136	WB-Mg	5'NT	0.328978	0
B5	B155	WB-Mg	IgA	-0.20115	0
B5	B157	WB-Mg	IgG	-0.2474	0
B5	B6	WB-Mg	WB-Fe	0.719574	0
B5	B7	WB-Mg	WB-Cop	0.289189	0
B5	B8	WB-Mg	WB-Zinc	-0.57613	0
B5	B57	WB-Mg	RBCAI	-0.1722	6.28E-13
B5	B138	WB-Mg	PAB	0.169655	1.38E-12
B5	B158	WB-Mg	IgM	-0.16505	5.53E-12
B5	B58	WB-Mg	HCT	0.151616	2.55E-10
B5	B156	WB-Mg	IgE	-0.12976	6.53E-08
B5	B195	WB-Mg	Na	0.125514	1.74E-07
B5	B137	WB-Mg	AFU	0.125325	1.81E-07
B5	B66	WB-Mg	WBRSR(mPas_100)	0.12474	2.07E-07
B5	B63	WB-Mg	WBRSR(mPas_200)	0.121484	4.27E-07
B50	B53	eo%	GR%	-0.25287	0
B50	B222	eo%	NS	0.137345	1.05E-08
B50	B209	eo%	Smoking	0.123507	2.73E-07
B50	B204	eo%	Drinking	0.120411	5.41E-07
B51	B113	Hb	FT3	0.199217	0
B51	B126	Hb	Alb	0.255877	0
B51	B128	Hb	ALT	0.230937	0
B51	B129	Hb	IBIL	0.210682	0
B51	B133	Hb	TBIL	0.205164	0
B51	B141	Hb	AST/ALT	-0.2387	0
B51	B163	Hb	SG	0.294535	0
B51	B174	Hb	Urea/Cr	-0.29336	0
B51	B176	Hb	Ucr	0.197856	0
B51	B192	Hb	UA	0.258485	0
B51	B206	Hb	Waist	0.272896	0
B51	B210	Hb	Hip	0.20239	0
B51	B215	Hb	BMI	0.22554	0
B51	B216	Hb	BMI	0.422259	0
B51	B218	Hb	DBP	0.261166	0
B51	B221	Hb	Hei	0.457378	0
B51	B222	Hb	NS	0.438025	0
B51	B71	Hb	TG	0.214931	0
B51	B72	Hb	HDL-C	-0.249	0
B51	B207	Hb	WHR	0.194609	4.44E-16
B51	B189	Hb	eGFB	0.190374	1.78E-15
B51	B209	Hb	Smoking	0.188249	3.33E-15
B51	B204	Hb	Drinking	0.176515	1.61E-13

B51	B191	Hb	Urea	-0.15622	7.13E-11
B51	B161	Hb	LEU	-0.1518	2.43E-10
B51	B140	Hb	r-GT	0.143436	2.25E-09
B51	B127	Hb	A-G ratio	0.139592	5.99E-09
B51	B58	Hb	HCT	0.132908	3.10E-08
B51	B132	Hb	Dbil	0.119629	6.41E-07
B51	B190	Hb	Cre	0.118791	7.68E-07
B52	B70	PLT/BPC	LDL-C	0.165776	4.45E-12
B52	B132	PLT/BPC	Dbil	-0.13971	5.82E-09
B52	B186	PLT/BPC	PFC	0.122922	3.11E-07
B53	B72	GR%	HDL-C	-0.12039	5.44E-07
B54	B56	ESR	IR	-0.26436	0
B54	B58	ESR	HCT	-0.27016	0
B54	B60	ESR	WBRVI	-0.35469	0
B54	B62	ESR	WBHSRVI	-0.30281	0
B54	B64	ESR	WBRSR(mPas_5)	-0.21317	0
B54	B65	ESR	WBRSR(mPas_1)	-0.21145	0
B54	B69	ESR	PV	0.437617	0
B54	B138	ESR	PAB	-0.19002	1.78E-15
B54	B78	ESR	HBcAb	-0.18239	2.40E-14
B54	B157	ESR	IgG	0.179105	7.02E-14
B54	B67	ESR	WBRSR(mPas_50)	-0.17058	1.04E-12
B54	B66	ESR	WBRSR(mPas_100)	-0.16085	1.90E-11
B54	B121	ESR	C3	-0.15902	3.21E-11
B54	B57	ESR	RBCAI	-0.15766	4.74E-11
B54	B55	ESR	TK	-0.1545	1.15E-10
B54	B100	ESR	anti-Ro52	0.152839	1.83E-10
B54	B122	ESR	C4	0.151447	2.68E-10
B54	B134	ESR	CHE	-0.14327	2.35E-09
B54	B63	ESR	WBRSR(mPas_200)	-0.13617	1.40E-08
B54	B61	ESR	WBHSRV	-0.13047	5.52E-08
B54	B101	ESR	anti-Sc70	0.126492	1.39E-07
B54	B102	ESR	anti-Sm	0.126492	1.39E-07
B54	B103	ESR	anti-SSA/Ro	0.126492	1.39E-07
B54	B104	ESR	anti-SSB	0.126492	1.39E-07
B54	B105	ESR	anti-U1RNP	0.126492	1.39E-07
B54	B106	ESR	anti-P	0.126492	1.39E-07
B54	B107	ESR	AnuA	0.126492	1.39E-07
B54	B108	ESR	Anti-Cyclin	0.126492	1.39E-07
B54	B109	ESR	AMA-M2	0.126492	1.39E-07
B54	B110	ESR	AHA	0.126492	1.39E-07
B54	B95	ESR	anti-cenpb	0.126492	1.39E-07
B54	B97	ESR	dsDNA	0.126492	1.39E-07
B54	B98	ESR	anti-Jo-1	0.126492	1.39E-07

B54	B99	ESR	anti-PM-Scl	0.126492	1.39E-07
B54	B137	ESR	AFU	-0.12109	4.66E-07
B54	B151	ESR	Anti-CCP	0.120891	4.87E-07
B54	B135	ESR	TBA	0.117881	9.33E-07
B55	B100	TK	anti-Ro52	-0.21528	0
B55	B156	TK	IgE	0.245646	0
B55	B157	TK	IgG	0.200104	0
B55	B181	TK	APTT	0.220883	0
B55	B56	TK	IR	0.794046	0
B55	B59	TK	WBRV	0.398205	0
B55	B60	TK	WBRVI	0.435229	0
B55	B61	TK	WBHSRV	0.712537	0
B55	B62	TK	WBHSRVI	0.568087	0
B55	B63	TK	WBRSR(mPas_200)	0.483142	0
B55	B64	TK	WBRSR(mPas_5)	0.39548	0
B55	B65	TK	WBRSR(mPas_1)	0.320667	0
B55	B66	TK	WBRSR(mPas_100)	0.43621	0
B55	B67	TK	WBRSR(mPas_50)	0.435939	0
B55	B69	TK	PV	-0.31592	0
B55	B184	TK	INR	0.173131	4.69E-13
B55	B152	TK	ACA-Ig(G-A-M)	-0.17147	7.88E-13
B55	B119	TK	Hcv-rna	0.171431	7.96E-13
B55	B159	TK	β2-MG	-0.17143	7.97E-13
B55	B125	TK	Cor	0.166021	4.13E-12
B55	B138	TK	PAB	-0.16195	1.38E-11
B55	B73	TK	ApoA	-0.15382	1.39E-10
B55	B135	TK	TBA	0.139562	6.04E-09
B55	B79	TK	hb V -dna	0.138625	7.64E-09
B55	B185	TK	PT	0.13782	9.34E-09
B55	B94	TK	ALDH2-Lys504Lys	0.128796	8.18E-08
B55	B92	TK	ALDH2-Glu504Glu	-0.12486	2.01E-07
B55	B58	TK	HCT	0.122974	3.07E-07
B55	B93	TK	ALDH2-Glu504Lys	0.118577	8.04E-07
B56	B100	IR	anti-Ro52	-0.24122	0
B56	B58	IR	HCT	0.665741	0
B56	B59	IR	WBRV	0.433104	0
B56	B60	IR	WBRVI	0.740648	0
B56	B61	IR	WBHSRV	0.924881	0
B56	B62	IR	WBHSRVI	0.949802	0
B56	B63	IR	WBRSR(mPas_200)	0.850747	0
B56	B64	IR	WBRSR(mPas_5)	0.740152	0
B56	B65	IR	WBRSR(mPas_1)	0.620014	0
B56	B66	IR	WBRSR(mPas_100)	0.828273	0
B56	B67	IR	WBRSR(mPas_50)	0.826939	0

B56	B69	IR	PV	-0.2732	0
B56	B73	IR	ApoA	-0.23342	0
B56	B158	IR	IgM	0.147033	8.77E-10
B56	B115	IR	TSH TT3 TT4(TT3)	0.143194	2.39E-09
B56	B137	IR	AFU	0.142784	2.66E-09
B56	B181	IR	APTT	0.129557	6.85E-08
B56	B124	IR	ACTH	-0.11932	6.86E-07
B57	B100	RBCAI	anti-Ro52	0.576761	0
B57	B121	RBCAI	C3	0.272098	0
B57	B122	RBCAI	C4	-0.27038	0
B57	B156	RBCAI	IgE	0.255426	0
B57	B59	RBCAI	WBRV	0.752545	0
B57	B60	RBCAI	WBRVI	0.533065	0
B57	B64	RBCAI	WBRSR(mPas_5)	0.344074	0
B57	B65	RBCAI	WBRSR(mPas_1)	0.566048	0
B57	B151	RBCAI	Anti-CCP	0.181244	3.51E-14
B57	B198	RBCAI	P	-0.15723	5.35E-11
B57	B93	RBCAI	ALDH2-Glu504Lys	0.155972	7.64E-11
B57	B183	RBCAI	PTA	-0.1517	2.50E-10
B57	B92	RBCAI	ALDH2-Glu504Glu	-0.15034	3.62E-10
B57	B182	RBCAI	TT	0.142384	2.95E-09
B57	B194	RBCAI	Cl	-0.13052	5.47E-08
B57	B155	RBCAI	IgA	0.12532	1.82E-07
B57	B101	RBCAI	anti-Sc70	-0.12276	3.23E-07
B57	B102	RBCAI	anti-Sm	-0.12276	3.23E-07
B57	B103	RBCAI	anti-SSA/Ro	-0.12276	3.23E-07
B57	B104	RBCAI	anti-SSB	-0.12276	3.23E-07
B57	B105	RBCAI	anti-U1RNP	-0.12276	3.23E-07
B57	B106	RBCAI	anti-P	-0.12276	3.23E-07
B57	B107	RBCAI	AnuA	-0.12276	3.23E-07
B57	B108	RBCAI	Anti-Cyclin	-0.12276	3.23E-07
B57	B109	RBCAI	AMA-M2	-0.12276	3.23E-07
B57	B110	RBCAI	AHA	-0.12276	3.23E-07
B57	B95	RBCAI	anti-cenpb	-0.12276	3.23E-07
B57	B97	RBCAI	dsDNA	-0.12276	3.23E-07
B57	B98	RBCAI	anti-Jo-1	-0.12276	3.23E-07
B57	B99	RBCAI	anti-PM-Scl	-0.12276	3.23E-07
B57	B138	RBCAI	PAB	-0.11858	8.04E-07
B58	B119	HCT	Hcv-rna	-0.30186	0
B58	B124	HCT	ACTH	-0.26931	0
B58	B125	HCT	Cor	-0.3781	0
B58	B138	HCT	PAB	0.202058	0
B58	B152	HCT	ACA-Ig(G-A-M)	0.301919	0
B58	B156	HCT	IgE	-0.21054	0

B58	B157	HCT	IgG	-0.22905	0
B58	B158	HCT	IgM	0.269345	0
B58	B159	HCT	β2-MG	0.301856	0
B58	B59	HCT	WBRV	0.262724	0
B58	B60	HCT	WBRVI	0.698997	0
B58	B61	HCT	WBHSRV	0.674185	0
B58	B62	HCT	WBHSRVI	0.854004	0
B58	B63	HCT	WBRSR(mPas_200)	0.836296	0
B58	B64	HCT	WBRSR(mPas_5)	0.759868	0
B58	B65	HCT	WBRSR(mPas_1)	0.66472	0
B58	B66	HCT	WBRSR(mPas_100)	0.841793	0
B58	B67	HCT	WBRSR(mPas_50)	0.847132	0
B58	B73	HCT	ApoA	-0.22547	0
B58	B74	HCT	ApoA1	-0.20905	0
B58	B79	HCT	hb V -dna	-0.24645	0
B58	B92	HCT	ALDH2-Glu504Glu	0.268601	0
B58	B93	HCT	ALDH2-Glu504Lys	-0.25515	0
B58	B94	HCT	ALDH2-Lys504Lys	-0.27579	0
B58	B100	HCT	anti-Ro52	-0.19354	4.44E-16
B58	B115	HCT	TSH TT3 TT4(TT3)	0.186313	6.44E-15
B58	B184	HCT	INR	-0.17443	3.12E-13
B58	B134	HCT	CHE	0.171712	7.30E-13
B58	B137	HCT	AFU	0.162993	1.02E-11
B58	B151	HCT	Anti-CCP	-0.16257	1.15E-11
B58	B198	HCT	P	-0.15392	1.36E-10
B58	B179	HCT	α1-MG	0.153716	1.43E-10
B58	B155	HCT	IgA	-0.1514	2.71E-10
B58	B120	HCT	HCV-cAg	-0.14883	5.44E-10
B58	B222	HCT	NS	0.143848	2.02E-09
B58	B101	HCT	anti-Sc70	-0.14298	2.53E-09
B58	B102	HCT	anti-Sm	-0.14298	2.53E-09
B58	B103	HCT	anti-SSA/Ro	-0.14298	2.53E-09
B58	B104	HCT	anti-SSB	-0.14298	2.53E-09
B58	B105	HCT	anti-U1RNP	-0.14298	2.53E-09
B58	B106	HCT	anti-P	-0.14298	2.53E-09
B58	B107	HCT	AnuA	-0.14298	2.53E-09
B58	B108	HCT	Anti-Cyclin	-0.14298	2.53E-09
B58	B109	HCT	AMA-M2	-0.14298	2.53E-09
B58	B110	HCT	AHA	-0.14298	2.53E-09
B58	B95	HCT	anti-cenpb	-0.14298	2.53E-09
B58	B97	HCT	dsDNA	-0.14298	2.53E-09
B58	B98	HCT	anti-Jo-1	-0.14298	2.53E-09
B58	B99	HCT	anti-PM-Scl	-0.14298	2.53E-09
B58	B182	HCT	TT	-0.13956	6.04E-09

B58	B75	HCT	ApoA1/B	-0.13409	2.33E-08
B58	B180	HCT	Tf	0.1224	3.49E-07
B59	B100	WBRV	anti-Ro52	0.452814	0
B59	B121	WBRV	C3	0.362512	0
B59	B122	WBRV	C4	-0.36894	0
B59	B151	WBRV	Anti-CCP	0.226704	0
B59	B156	WBRV	IgE	0.339574	0
B59	B194	WBRV	Cl	-0.22172	0
B59	B60	WBRV	WBRVI	0.792257	0
B59	B61	WBRV	WBHSRV	0.560416	0
B59	B62	WBRV	WBHSRVI	0.380671	0
B59	B63	WBRV	WBRSR(mPas_200)	0.505491	0
B59	B64	WBRV	WBRSR(mPas_5)	0.787068	0
B59	B65	WBRV	WBRSR(mPas_1)	0.890295	0
B59	B66	WBRV	WBRSR(mPas_100)	0.548957	0
B59	B67	WBRV	WBRSR(mPas_50)	0.587319	0
B59	B69	WBRV	PV	0.26207	0
B59	B198	WBRV	P	-0.19084	1.33E-15
B59	B157	WBRV	IgG	0.178444	8.70E-14
B59	B183	WBRV	PTA	-0.13282	3.17E-08
B59	B138	WBRV	PAB	-0.13092	4.97E-08
B6	B136	WB-Fe	5'NT	0.266014	0
B6	B157	WB-Fe	IgG	-0.20162	0
B6	B184	WB-Fe	INR	0.231202	0
B6	B7	WB-Fe	WB-Cop	0.674072	0
B6	B8	WB-Fe	WB-Zinc	-0.25903	0
B6	B181	WB-Fe	APTT	0.189608	2.22E-15
B6	B155	WB-Fe	IgA	-0.16318	9.61E-12
B6	B138	WB-Fe	PAB	0.140644	4.60E-09
B6	B57	WB-Fe	RBCAI	-0.13967	5.88E-09
B6	B158	WB-Fe	IgM	-0.13497	1.88E-08
B6	B185	WB-Fe	PT	0.131094	4.77E-08
B6	B193	WB-Fe	K	-0.12603	1.55E-07
B6	B58	WB-Fe	HCT	0.123225	2.91E-07
B60	B121	WBRVI	C3	0.285609	0
B60	B122	WBRVI	C4	-0.27255	0
B60	B158	WBRVI	IgM	0.25423	0
B60	B198	WBRVI	P	-0.20807	0
B60	B61	WBRVI	WBHSRV	0.709266	0
B60	B62	WBRVI	WBHSRVI	0.783034	0
B60	B63	WBRVI	WBRSR(mPas_200)	0.724128	0
B60	B64	WBRVI	WBRSR(mPas_5)	0.902104	0
B60	B65	WBRVI	WBRSR(mPas_1)	0.93285	0
B60	B66	WBRVI	WBRSR(mPas_100)	0.760976	0

B60	B67	WBRVI	WBRSR(mPas_50)	0.79471	0
B60	B73	WBRVI	ApoA	-0.20415	0
B60	B94	WBRVI	ALDH2-Lys504Lys	-0.20395	0
B60	B125	WBRVI	Cor	-0.19184	8.88E-16
B60	B194	WBRVI	Cl	-0.17581	2.02E-13
B60	B124	WBRVI	ACTH	-0.16876	1.81E-12
B60	B152	WBRVI	ACA-Ig(G-A-M)	0.15707	5.60E-11
B60	B119	WBRVI	Hcv-rna	-0.15704	5.65E-11
B60	B159	WBRVI	β2-MG	0.157037	5.66E-11
B60	B134	WBRVI	CHE	0.155785	8.05E-11
B60	B101	WBRVI	anti-Sc70	-0.15327	1.62E-10
B60	B102	WBRVI	anti-Sm	-0.15327	1.62E-10
B60	B103	WBRVI	anti-SSA/Ro	-0.15327	1.62E-10
B60	B104	WBRVI	anti-SSB	-0.15327	1.62E-10
B60	B105	WBRVI	anti-U1RNP	-0.15327	1.62E-10
B60	B106	WBRVI	anti-P	-0.15327	1.62E-10
B60	B107	WBRVI	AnuA	-0.15327	1.62E-10
B60	B108	WBRVI	Anti-Cyclin	-0.15327	1.62E-10
B60	B109	WBRVI	AMA-M2	-0.15327	1.62E-10
B60	B110	WBRVI	AHA	-0.15327	1.62E-10
B60	B95	WBRVI	anti-cenpb	-0.15327	1.62E-10
B60	B97	WBRVI	dsDNA	-0.15327	1.62E-10
B60	B98	WBRVI	anti-Jo-1	-0.15327	1.62E-10
B60	B99	WBRVI	anti-PM-Scl	-0.15327	1.62E-10
B60	B137	WBRVI	AFU	0.146201	1.09E-09
B60	B69	WBRVI	PV	-0.14593	1.17E-09
B60	B156	WBRVI	IgE	0.141699	3.51E-09
B60	B114	WBRVI	TSH TT3 TT4(TT4)	-0.1375	1.01E-08
B60	B120	WBRVI	HCV-cAg	-0.13741	1.03E-08
B60	B79	WBRVI	hb V -dna	-0.1285	8.76E-08
B60	B100	WBRVI	anti-Ro52	0.124471	2.20E-07
B60	B115	WBRVI	TSH TT3 TT4(TT3)	0.124168	2.36E-07
B60	B222	WBRVI	NS	0.118516	8.14E-07
B61	B62	WBHSRV	WBHSRVI	0.892133	0
B61	B63	WBHSRV	WBRSR(mPas_200)	0.953616	0
B61	B64	WBHSRV	WBRSR(mPas_5)	0.851012	0
B61	B65	WBHSRV	WBRSR(mPas_1)	0.728448	0
B61	B66	WBHSRV	WBRSR(mPas_100)	0.935546	0
B61	B67	WBHSRV	WBRSR(mPas_50)	0.932497	0
B61	B73	WBHSRV	ApoA	-0.20364	0
B61	B122	WBHSRV	C4	-0.18217	2.58E-14
B61	B121	WBHSRV	C3	0.180356	4.66E-14
B61	B93	WBHSRV	ALDH2-Glu504Lys	-0.16561	4.68E-12
B61	B194	WBHSRV	Cl	-0.16299	1.02E-11

B61	B92	WBHSRV	ALDH2-Glu504Glu	0.162784	1.08E-11
B61	B181	WBHSRV	APTT	0.137529	1.00E-08
B61	B137	WBHSRV	AFU	0.126824	1.29E-07
B61	B74	WBHSRV	ApoA1	-0.12342	2.78E-07
B61	B100	WBHSRV	anti-Ro52	-0.11874	7.76E-07
B62	B100	WBHSRVI	anti-Ro52	-0.22557	0
B62	B125	WBHSRVI	Cor	-0.22629	0
B62	B158	WBHSRVI	IgM	0.210436	0
B62	B63	WBHSRVI	WBRSR(mPas_200)	0.911803	0
B62	B64	WBHSRVI	WBRSR(mPas_5)	0.805164	0
B62	B65	WBHSRVI	WBRSR(mPas_1)	0.679506	0
B62	B66	WBHSRVI	WBRSR(mPas_100)	0.904615	0
B62	B67	WBHSRVI	WBRSR(mPas_50)	0.903074	0
B62	B69	WBHSRVI	PV	-0.20917	0
B62	B73	WBHSRVI	ApoA	-0.24521	0
B62	B124	WBHSRVI	ACTH	-0.19197	8.88E-16
B62	B115	WBHSRVI	TSH TT3 TT4(TT3)	0.186015	7.11E-15
B62	B94	WBHSRVI	ALDH2-Lys504Lys	-0.17124	8.46E-13
B62	B74	WBHSRVI	ApoA1	-0.1707	9.99E-13
B62	B152	WBHSRVI	ACA-Ig(G-A-M)	0.161981	1.37E-11
B62	B119	WBHSRVI	Hcv-rna	-0.16195	1.38E-11
B62	B159	WBHSRVI	β2-MG	0.161946	1.38E-11
B62	B137	WBHSRVI	AFU	0.159364	2.91E-11
B62	B92	WBHSRVI	ALDH2-Glu504Glu	0.142957	2.54E-09
B62	B93	WBHSRVI	ALDH2-Glu504Lys	-0.13443	2.15E-08
B62	B79	WBHSRVI	hb V -dna	-0.13255	3.38E-08
B62	B222	WBHSRVI	NS	0.128463	8.84E-08
B62	B179	WBHSRVI	α1-MG	0.12393	2.48E-07
B62	B134	WBHSRVI	CHE	0.120498	5.30E-07
B63	B64	WBRSR(mPas_200)	WBRSR(mPas_5)	0.899864	0
B63	B65	WBRSR(mPas_200)	WBRSR(mPas_1)	0.771134	0
B63	B66	WBRSR(mPas_200)	WBRSR(mPas_100)	0.992718	0
B63	B67	WBRSR(mPas_200)	WBRSR(mPas_50)	0.988302	0
B63	B73	WBRSR(mPas_200)	ApoA	-0.2104	0
B63	B92	WBRSR(mPas_200)	ALDH2-Glu504Glu	0.258645	0
B63	B93	WBRSR(mPas_200)	ALDH2-Glu504Lys	-0.25772	0
B63	B69	WBRSR(mPas_200)	PV	0.196525	2.22E-16
B63	B125	WBRSR(mPas_200)	Cor	-0.18926	2.22E-15
B63	B124	WBRSR(mPas_200)	ACTH	-0.1766	1.57E-13
B63	B74	WBRSR(mPas_200)	ApoA1	-0.16804	2.25E-12
B63	B121	WBRSR(mPas_200)	C3	0.164956	5.68E-12
B63	B122	WBRSR(mPas_200)	C4	-0.15845	3.79E-11
B63	B158	WBRSR(mPas_200)	IgM	0.158078	4.21E-11
B63	B194	WBRSR(mPas_200)	Cl	-0.15287	1.81E-10

B63	B198	WBRSR(mPas_200)	P	-0.14133	3.86E-09
B63	B115	WBRSR(mPas_200)	TSH TT3 TT4(TT3)	0.134953	1.89E-08
B63	B137	WBRSR(mPas_200)	AFU	0.134552	2.08E-08
B63	B182	WBRSR(mPas_200)	TT	-0.12244	3.46E-07
B63	B222	WBRSR(mPas_200)	NS	0.120661	5.12E-07
B64	B121	WBRSR(mPas_5)	C3	0.304924	0
B64	B122	WBRSR(mPas_5)	C4	-0.29661	0
B64	B158	WBRSR(mPas_5)	IgM	0.206613	0
B64	B198	WBRSR(mPas_5)	P	-0.22719	0
B64	B65	WBRSR(mPas_5)	WBRSR(mPas_1)	0.964521	0
B64	B66	WBRSR(mPas_5)	WBRSR(mPas_100)	0.929791	0
B64	B67	WBRSR(mPas_5)	WBRSR(mPas_50)	0.945663	0
B64	B69	WBRSR(mPas_5)	PV	0.216858	0
B64	B194	WBRSR(mPas_5)	Cl	-0.19555	2.22E-16
B64	B73	WBRSR(mPas_5)	ApoA	-0.19485	4.44E-16
B64	B92	WBRSR(mPas_5)	ALDH2-Glu504Glu	0.191728	8.88E-16
B64	B125	WBRSR(mPas_5)	Cor	-0.19115	1.33E-15
B64	B93	WBRSR(mPas_5)	ALDH2-Glu504Lys	-0.18773	4.00E-15
B64	B124	WBRSR(mPas_5)	ACTH	-0.17466	2.91E-13
B64	B100	WBRSR(mPas_5)	anti-Ro52	0.147334	8.10E-10
B64	B137	WBRSR(mPas_5)	AFU	0.141971	3.28E-09
B64	B101	WBRSR(mPas_5)	anti-Sc70	-0.13822	8.46E-09
B64	B102	WBRSR(mPas_5)	anti-Sm	-0.13822	8.46E-09
B64	B103	WBRSR(mPas_5)	anti-SSA/Ro	-0.13822	8.46E-09
B64	B104	WBRSR(mPas_5)	anti-SSB	-0.13822	8.46E-09
B64	B105	WBRSR(mPas_5)	anti-U1RNP	-0.13822	8.46E-09
B64	B106	WBRSR(mPas_5)	anti-P	-0.13822	8.46E-09
B64	B107	WBRSR(mPas_5)	AnuA	-0.13822	8.46E-09
B64	B108	WBRSR(mPas_5)	Anti-Cyclin	-0.13822	8.46E-09
B64	B109	WBRSR(mPas_5)	AMA-M2	-0.13822	8.46E-09
B64	B110	WBRSR(mPas_5)	AHA	-0.13822	8.46E-09
B64	B95	WBRSR(mPas_5)	anti-cenpb	-0.13822	8.46E-09
B64	B97	WBRSR(mPas_5)	dsDNA	-0.13822	8.46E-09
B64	B98	WBRSR(mPas_5)	anti-Jo-1	-0.13822	8.46E-09
B64	B99	WBRSR(mPas_5)	anti-PM-Scl	-0.13822	8.46E-09
B64	B134	WBRSR(mPas_5)	CHE	0.136759	1.21E-08
B64	B222	WBRSR(mPas_5)	NS	0.120588	5.20E-07
B64	B156	WBRSR(mPas_5)	IgE	0.119644	6.39E-07
B64	B74	WBRSR(mPas_5)	ApoA1	-0.11768	9.75E-07
B65	B100	WBRSR(mPas_1)	anti-Ro52	0.272732	0
B65	B121	WBRSR(mPas_1)	C3	0.342131	0
B65	B122	WBRSR(mPas_1)	C4	-0.3346	0
B65	B158	WBRSR(mPas_1)	IgM	0.202338	0
B65	B194	WBRSR(mPas_1)	Cl	-0.22008	0

B65	B198	WBRSR(mPas_1)	P	-0.2364	0
B65	B66	WBRSR(mPas_1)	WBRSR(mPas_100)	0.814691	0
B65	B67	WBRSR(mPas_1)	WBRSR(mPas_50)	0.845969	0
B65	B69	WBRSR(mPas_1)	PV	0.214734	0
B65	B156	WBRSR(mPas_1)	IgE	0.167387	2.74E-12
B65	B73	WBRSR(mPas_1)	ApoA	-0.16448	6.54E-12
B65	B125	WBRSR(mPas_1)	Cor	-0.16161	1.52E-11
B65	B101	WBRSR(mPas_1)	anti-Sc70	-0.15047	3.49E-10
B65	B102	WBRSR(mPas_1)	anti-Sm	-0.15047	3.49E-10
B65	B103	WBRSR(mPas_1)	anti-SSA/Ro	-0.15047	3.49E-10
B65	B104	WBRSR(mPas_1)	anti-SSB	-0.15047	3.49E-10
B65	B105	WBRSR(mPas_1)	anti-U1RNP	-0.15047	3.49E-10
B65	B106	WBRSR(mPas_1)	anti-P	-0.15047	3.49E-10
B65	B107	WBRSR(mPas_1)	AnuA	-0.15047	3.49E-10
B65	B108	WBRSR(mPas_1)	Anti-Cyclin	-0.15047	3.49E-10
B65	B109	WBRSR(mPas_1)	AMA-M2	-0.15047	3.49E-10
B65	B110	WBRSR(mPas_1)	AHA	-0.15047	3.49E-10
B65	B95	WBRSR(mPas_1)	anti-cenpb	-0.15047	3.49E-10
B65	B97	WBRSR(mPas_1)	dsDNA	-0.15047	3.49E-10
B65	B98	WBRSR(mPas_1)	anti-Jo-1	-0.15047	3.49E-10
B65	B99	WBRSR(mPas_1)	anti-PM-Scl	-0.15047	3.49E-10
B65	B124	WBRSR(mPas_1)	ACTH	-0.15035	3.61E-10
B65	B134	WBRSR(mPas_1)	CHE	0.145052	1.48E-09
B65	B92	WBRSR(mPas_1)	ALDH2-Glu504Glu	0.131658	4.17E-08
B65	B93	WBRSR(mPas_1)	ALDH2-Glu504Lys	-0.12698	1.24E-07
B65	B137	WBRSR(mPas_1)	AFU	0.124004	2.44E-07
B65	B120	WBRSR(mPas_1)	HCV-cAg	-0.1211	4.65E-07
B66	B125	WBRSR(mPas_100)	Cor	-0.21061	0
B66	B67	WBRSR(mPas_100)	WBRSR(mPas_50)	0.99705	0
B66	B69	WBRSR(mPas_100)	PV	0.214928	0
B66	B73	WBRSR(mPas_100)	ApoA	-0.21067	0
B66	B92	WBRSR(mPas_100)	ALDH2-Glu504Glu	0.255946	0
B66	B93	WBRSR(mPas_100)	ALDH2-Glu504Lys	-0.25343	0
B66	B121	WBRSR(mPas_100)	C3	0.196454	2.22E-16
B66	B122	WBRSR(mPas_100)	C4	-0.18787	3.77E-15
B66	B124	WBRSR(mPas_100)	ACTH	-0.18597	7.33E-15
B66	B74	WBRSR(mPas_100)	ApoA1	-0.17962	5.95E-14
B66	B158	WBRSR(mPas_100)	IgM	0.167973	2.30E-12
B66	B198	WBRSR(mPas_100)	P	-0.1609	1.87E-11
B66	B194	WBRSR(mPas_100)	Cl	-0.15923	3.03E-11
B66	B137	WBRSR(mPas_100)	AFU	0.143606	2.15E-09
B66	B115	WBRSR(mPas_100)	TSH TT3 TT4(TT3)	0.143202	2.39E-09
B66	B182	WBRSR(mPas_100)	TT	-0.12729	1.16E-07
B66	B222	WBRSR(mPas_100)	NS	0.12359	2.68E-07

B66	B134	WBRSR(mPas_100)	CHE	0.120439	5.37E-07
B66	B101	WBRSR(mPas_100)	anti-Sc70	-0.11838	8.38E-07
B66	B102	WBRSR(mPas_100)	anti-Sm	-0.11838	8.38E-07
B66	B103	WBRSR(mPas_100)	anti-SSA/Ro	-0.11838	8.38E-07
B66	B104	WBRSR(mPas_100)	anti-SSB	-0.11838	8.38E-07
B66	B105	WBRSR(mPas_100)	anti-U1RNP	-0.11838	8.38E-07
B66	B106	WBRSR(mPas_100)	anti-P	-0.11838	8.38E-07
B66	B107	WBRSR(mPas_100)	AnuA	-0.11838	8.38E-07
B66	B108	WBRSR(mPas_100)	Anti-Cyclin	-0.11838	8.38E-07
B66	B109	WBRSR(mPas_100)	AMA-M2	-0.11838	8.38E-07
B66	B110	WBRSR(mPas_100)	AHA	-0.11838	8.38E-07
B66	B95	WBRSR(mPas_100)	anti-cenpb	-0.11838	8.38E-07
B66	B97	WBRSR(mPas_100)	dsDNA	-0.11838	8.38E-07
B66	B98	WBRSR(mPas_100)	anti-Jo-1	-0.11838	8.38E-07
B66	B99	WBRSR(mPas_100)	anti-PM-Scl	-0.11838	8.38E-07
B67	B121	WBRSR(mPas_50)	C3	0.21538	0
B67	B122	WBRSR(mPas_50)	C4	-0.20671	0
B67	B125	WBRSR(mPas_50)	Cor	-0.20839	0
B67	B69	WBRSR(mPas_50)	PV	0.207615	0
B67	B73	WBRSR(mPas_50)	ApoA	-0.20623	0
B67	B92	WBRSR(mPas_50)	ALDH2-Glu504Glu	0.243975	0
B67	B93	WBRSR(mPas_50)	ALDH2-Glu504Lys	-0.24108	0
B67	B124	WBRSR(mPas_50)	ACTH	-0.18367	1.58E-14
B67	B158	WBRSR(mPas_50)	IgM	0.174568	2.99E-13
B67	B74	WBRSR(mPas_50)	ApoA1	-0.17368	3.95E-13
B67	B194	WBRSR(mPas_50)	Cl	-0.17293	5.00E-13
B67	B198	WBRSR(mPas_50)	P	-0.16737	2.76E-12
B67	B137	WBRSR(mPas_50)	AFU	0.144055	1.91E-09
B67	B115	WBRSR(mPas_50)	TSH TT3 TT4(TT3)	0.140894	4.31E-09
B67	B134	WBRSR(mPas_50)	CHE	0.125187	1.87E-07
B67	B222	WBRSR(mPas_50)	NS	0.124953	1.97E-07
B69	B100	PV	anti-Ro52	0.374087	0
B69	B92	PV	ALDH2-Glu504Glu	0.263249	0
B69	B93	PV	ALDH2-Glu504Lys	-0.27866	0
B69	B94	PV	ALDH2-Lys504Lys	0.240579	0
B69	B151	PV	Anti-CCP	0.177098	1.34E-13
B69	B152	PV	ACA-Ig(G-A-M)	-0.15634	6.89E-11
B69	B119	PV	Hcv-rna	0.156308	6.95E-11
B69	B159	PV	β2-MG	-0.15631	6.95E-11
B69	B122	PV	C4	-0.15006	3.90E-10
B69	B121	PV	C3	0.136921	1.17E-08
B69	B194	PV	Cl	-0.13211	3.75E-08
B69	B79	PV	hb V -dna	0.127619	1.07E-07
B69	B158	PV	IgM	-0.12632	1.45E-07

B69	B76	PV	ApoB	-0.12577	1.64E-07
B7	B136	WB-Cop	5'NT	0.317561	0
B7	B157	WB-Cop	IgG	-0.23954	0
B7	B181	WB-Cop	APTT	0.254367	0
B7	B184	WB-Cop	INR	0.309115	0
B7	B8	WB-Cop	WB-Zinc	0.520338	0
B7	B155	WB-Cop	IgA	-0.19441	4.44E-16
B7	B185	WB-Cop	PT	0.175143	2.49E-13
B7	B57	WB-Cop	RBCAI	-0.16642	3.67E-12
B7	B138	WB-Cop	PAB	0.165365	5.03E-12
B7	B193	WB-Cop	K	-0.16293	1.03E-11
B7	B158	WB-Cop	IgM	-0.16002	2.42E-11
B7	B58	WB-Cop	HCT	0.146644	9.72E-10
B7	B156	WB-Cop	IgE	-0.12586	1.61E-07
B7	B186	WB-Cop	PFC	-0.12442	2.22E-07
B7	B137	WB-Cop	AFU	0.121204	4.55E-07
B7	B66	WB-Cop	WBRSR(mPas_100)	0.120415	5.40E-07
B70	B132	LDL-C	Dbil	-0.25579	0
B70	B76	LDL-C	ApoB	0.238165	0
B70	B77	LDL-C	TC	0.801317	0
B70	B189	LDL-C	eGFB	0.15226	2.14E-10
B70	B75	LDL-C	ApoA1/B	-0.15222	2.17E-10
B70	B72	LDL-C	HDL-C	0.134382	2.17E-08
B70	B153	LDL-C	Glu	-0.11878	7.70E-07
B71	B130	TG	GLB	0.343881	0
B71	B153	TG	Glu	0.198564	0
B71	B167	TG	PC	-0.19882	0
B71	B72	TG	HDL-C	-0.40662	0
B71	B77	TG	TC	0.347883	0
B71	B91	TG	TP	0.401001	0
B71	B192	TG	UA	0.191365	1.11E-15
B71	B222	TG	NS	0.177157	1.31E-13
B71	B126	TG	Alb	0.173547	4.12E-13
B71	B127	TG	A-G ratio	-0.16736	2.76E-12
B71	B216	TG	BMI	0.166489	3.59E-12
B71	B218	TG	DBP	0.16413	7.26E-12
B71	B140	TG	r-GT	0.161023	1.80E-11
B71	B163	TG	SG	0.154185	1.26E-10
B71	B128	TG	ALT	0.15071	3.27E-10
B71	B215	TG	BMI	0.141374	3.82E-09
B71	B141	TG	AST/ALT	-0.14114	4.05E-09
B71	B170	TG	U-GLU	0.136568	1.27E-08
B71	B206	TG	Waist	0.136176	1.40E-08
B71	B132	TG	Dbil	-0.12494	1.98E-07

B71	B166	TG	Pro	0.122761	3.22E-07
B71	B189	TG	eGFB	0.118108	8.89E-07
B72	B141	HDL-C	AST/ALT	0.23547	0
B72	B153	HDL-C	Glu	-0.21882	0
B72	B163	HDL-C	SG	-0.20164	0
B72	B190	HDL-C	Cre	-0.20417	0
B72	B192	HDL-C	UA	-0.35801	0
B72	B206	HDL-C	Waist	-0.41942	0
B72	B207	HDL-C	WHR	-0.28512	0
B72	B210	HDL-C	Hip	-0.3023	0
B72	B215	HDL-C	BMI	-0.3831	0
B72	B216	HDL-C	BMI	-0.44293	0
B72	B221	HDL-C	Hei	-0.3081	0
B72	B222	HDL-C	NS	-0.46646	0
B72	B77	HDL-C	TC	0.186903	5.33E-15
B72	B74	HDL-C	ApoA1	0.176484	1.63E-13
B72	B128	HDL-C	ALT	-0.16618	3.94E-12
B72	B174	HDL-C	Urea/Cr	0.157244	5.33E-11
B72	B209	HDL-C	Smoking	-0.15112	2.92E-10
B73	B137	ApoA	AFU	-0.26408	0
B73	B193	ApoA	K	0.174415	3.14E-13
B73	B138	ApoA	PAB	-0.14086	4.35E-09
B73	B134	ApoA	CHE	-0.13723	1.08E-08
B73	B125	ApoA	Cor	0.132503	3.41E-08
B73	B135	ApoA	TBA	-0.12889	8.00E-08
B74	B135	ApoA1	TBA	0.266957	0
B74	B195	ApoA1	Na	0.198073	0
B74	B197	ApoA1	AG	0.324219	0
B74	B75	ApoA1	ApoA1/B	0.508079	0
B74	B86	ApoA1	TBhCG	0.202895	0
B74	B196	ApoA1	TCO2	-0.17949	6.17E-14
B74	B76	ApoA1	ApoB	0.168426	2.00E-12
B74	B138	ApoA1	PAB	-0.16585	4.35E-12
B74	B152	ApoA1	ACA-Ig(G-A-M)	-0.14057	4.68E-09
B74	B119	ApoA1	Hcv-rna	0.140542	4.71E-09
B74	B159	ApoA1	β2-MG	-0.14054	4.72E-09
B74	B182	ApoA1	TT	0.135859	1.51E-08
B74	B199	ApoA1	Mg	-0.12749	1.11E-07
B74	B183	ApoA1	PTA	-0.12258	3.36E-07
B74	B88	ApoA1	CA 242	0.120271	5.57E-07
B74	B200	ApoA1	OP	0.119324	6.84E-07
B75	B119	ApoA1/B	Hcv-rna	0.197133	0
B75	B135	ApoA1/B	TBA	0.22158	0
B75	B152	ApoA1/B	ACA-Ig(G-A-M)	-0.19717	0

B75	B159	ApoA1/B	β2-MG	-0.19713	0
B75	B196	ApoA1/B	TCO2	-0.25946	0
B75	B197	ApoA1/B	AG	0.226304	0
B75	B198	ApoA1/B	P	0.210667	0
B75	B76	ApoA1/B	ApoB	-0.70614	0
B75	B158	ApoA1/B	IgM	-0.18474	1.11E-14
B75	B155	ApoA1/B	IgA	0.177159	1.31E-13
B75	B79	ApoA1/B	hb V -dna	0.17487	2.72E-13
B75	B199	ApoA1/B	Mg	-0.1628	1.07E-11
B75	B125	ApoA1/B	Cor	0.159877	2.51E-11
B75	B179	ApoA1/B	α1-MG	-0.14286	2.61E-09
B75	B156	ApoA1/B	IgE	0.14026	5.06E-09
B75	B94	ApoA1/B	ALDH2-Lys504Lys	0.13651	1.29E-08
B75	B138	ApoA1/B	PAB	-0.13638	1.33E-08
B75	B180	ApoA1/B	Tf	-0.13442	2.15E-08
B75	B134	ApoA1/B	CHE	-0.1312	4.65E-08
B75	B124	ApoA1/B	ACTH	0.120616	5.17E-07
B76	B158	ApoB	IgM	0.19607	2.22E-16
B76	B77	ApoB	TC	0.188946	2.66E-15
B76	B152	ApoB	ACA-Ig(G-A-M)	0.179356	6.48E-14
B76	B119	ApoB	Hcv-rna	-0.17932	6.55E-14
B76	B159	ApoB	β2-MG	0.179318	6.55E-14
B76	B79	ApoB	hb V -dna	-0.16494	5.71E-12
B76	B125	ApoB	Cor	-0.15986	2.53E-11
B76	B198	ApoB	P	-0.15797	4.34E-11
B76	B155	ApoB	IgA	-0.15678	6.08E-11
B76	B196	ApoB	TCO2	0.151163	2.89E-10
B76	B124	ApoB	ACTH	-0.14787	7.02E-10
B76	B115	ApoB	TSH TT3 TT4(TT3)	0.147397	7.96E-10
B76	B181	ApoB	APTT	0.138334	8.21E-09
B76	B94	ApoB	ALDH2-Lys504Lys	-0.13617	1.40E-08
B76	B88	ApoB	CA 242	0.123796	2.56E-07
B77	B130	TC	GLB	0.249616	0
B77	B132	TC	Dbil	-0.27348	0
B77	B91	TC	TP	0.267983	0
B77	B127	TC	A-G ratio	-0.16966	1.37E-12
B77	B206	TC	Waist	-0.15657	6.46E-11
B77	B189	TC	eGFB	0.146918	9.04E-10
B77	B216	TC	BMI	-0.13014	5.97E-08
B77	B222	TC	NS	-0.1239	2.50E-07
B78	B79	HBcAb	hb V -dna	0.18978	2.00E-15
B79	B101	hb V -dna	anti-Sc70	0.312626	0
B79	B102	hb V -dna	anti-Sm	0.312626	0
B79	B103	hb V -dna	anti-SSA/Ro	0.312626	0

B79	B104	hb V -dna	anti-SSB	0.312626	0
B79	B105	hb V -dna	anti-U1RNP	0.312626	0
B79	B106	hb V -dna	anti-P	0.312626	0
B79	B107	hb V -dna	AnuA	0.312626	0
B79	B108	hb V -dna	Anti-Cyclin	0.312626	0
B79	B109	hb V -dna	AMA-M2	0.312626	0
B79	B110	hb V -dna	AHA	0.312626	0
B79	B119	hb V -dna	Hcv-rna	0.611302	0
B79	B120	hb V -dna	HCV-cAg	0.276786	0
B79	B124	hb V -dna	ACTH	0.322904	0
B79	B125	hb V -dna	Cor	0.499478	0
B79	B134	hb V -dna	CHE	-0.21361	0
B79	B152	hb V -dna	ACA-Ig(G-A-M)	-0.61142	0
B79	B158	hb V -dna	IgM	-0.40409	0
B79	B159	hb V -dna	β2-MG	-0.6113	0
B79	B92	hb V -dna	ALDH2-Glu504Glu	-0.29786	0
B79	B93	hb V -dna	ALDH2-Glu504Lys	0.277306	0
B79	B94	hb V -dna	ALDH2-Lys504Lys	0.406584	0
B79	B95	hb V -dna	anti-cenpb	0.312626	0
B79	B97	hb V -dna	dsDNA	0.312626	0
B79	B98	hb V -dna	anti-Jo-1	0.312626	0
B79	B99	hb V -dna	anti-PM-Scl	0.312626	0
B79	B179	hb V -dna	α1-MG	-0.19057	1.55E-15
B79	B115	hb V -dna	TSH TT3 TT4(TT3)	-0.18123	3.51E-14
B79	B156	hb V -dna	IgE	0.148676	5.66E-10
B79	B81	hb V -dna	TSGF	0.147341	8.08E-10
B79	B155	hb V -dna	IgA	0.134674	2.02E-08
B79	B151	hb V -dna	Anti-CCP	0.125644	1.69E-07
B79	B203	hb V -dna	DH	-0.12321	2.92E-07
B79	B184	hb V -dna	INR	0.117779	9.54E-07
B8	B184	WB-Zinc	INR	0.210806	0
B8	B181	WB-Zinc	APTT	0.173829	3.77E-13
B8	B123	WB-Zinc	hs-CRP1	0.130822	5.09E-08
B8	B185	WB-Zinc	PT	0.119388	6.75E-07
B8	B86	WB-Zinc	TBhCG	-0.11806	8.98E-07
B81	B125	TSGF	Cor	0.22092	0
B81	B152	TSGF	ACA-Ig(G-A-M)	-0.18032	4.75E-14
B81	B119	TSGF	Hcv-rna	0.180279	4.80E-14
B81	B159	TSGF	β2-MG	-0.18028	4.80E-14
B81	B94	TSGF	ALDH2-Lys504Lys	0.180279	4.80E-14
B81	B124	TSGF	ACTH	0.142981	2.53E-09
B81	B158	TSGF	IgM	-0.13754	1.00E-08
B81	B92	TSGF	ALDH2-Glu504Glu	-0.13127	4.58E-08
B81	B93	TSGF	ALDH2-Glu504Lys	0.12215	3.69E-07

B82	B83	CEA	AFP	0.213956	0
B82	B85	CEA	SCC	0.338078	0
B82	B88	CEA	CA 242	0.383507	0
B82	B87	CEA	NSE	0.180443	4.53E-14
B82	B138	CEA	PAB	-0.16441	6.69E-12
B82	B84	CEA	C-PSA	0.160429	2.14E-11
B82	B196	CEA	TCO2	0.134492	2.11E-08
B82	B183	CEA	PTA	-0.13022	5.86E-08
B82	B181	CEA	APTT	0.128377	9.02E-08
B83	B137	AFP	AFU	0.210057	0
B83	B84	AFP	C-PSA	0.411867	0
B83	B87	AFP	NSE	0.404988	0
B83	B89	AFP	CA 72-4	0.142585	2.80E-09
B83	B86	AFP	TBhCG	-0.1369	1.17E-08
B84	B197	C-PSA	AG	-0.1973	0
B84	B87	C-PSA	NSE	0.3458	0
B84	B88	C-PSA	CA 242	0.273804	0
B84	B85	C-PSA	SCC	0.189116	2.66E-15
B84	B114	C-PSA	TSH TT3 TT4(TT4)	-0.15354	1.51E-10
B84	B196	C-PSA	TCO2	0.150333	3.62E-10
B85	B87	SCC	NSE	0.407628	0
B85	B88	SCC	CA 242	0.413618	0
B85	B89	SCC	CA 72-4	0.173786	3.82E-13
B85	B181	SCC	APTT	0.134294	2.22E-08
B85	B86	SCC	TBhCG	0.128594	8.57E-08
B86	B138	TBhCG	PAB	-0.24672	0
B86	B197	TBhCG	AG	0.237617	0
B86	B199	TBhCG	Mg	-0.22495	0
B86	B87	TBhCG	NSE	-0.24355	0
B86	B88	TBhCG	CA 242	0.358697	0
B86	B90	TBhCG	CYFRA 21-1	0.236773	0
B86	B139	TBhCG	ADA	0.185733	7.99E-15
B86	B115	TBhCG	TSH TT3 TT4(TT3)	0.165253	5.20E-12
B86	B136	TBhCG	5'NT	0.158556	3.67E-11
B86	B137	TBhCG	AFU	-0.15515	9.63E-11
B86	B196	TBhCG	TCO2	-0.15265	1.93E-10
B86	B181	TBhCG	APTT	0.145662	1.26E-09
B86	B114	TBhCG	TSH TT3 TT4(TT4)	0.141454	3.74E-09
B86	B89	TBhCG	CA 72-4	0.136049	1.45E-08
B86	B183	TBhCG	PTA	-0.12326	2.88E-07
B87	B88	NSE	CA 242	0.179368	6.44E-14
B87	B155	NSE	IgA	0.123634	2.65E-07
B88	B138	CA 242	PAB	-0.18141	3.31E-14
B88	B139	CA 242	ADA	0.161726	1.47E-11

B88	B136	CA 242	5'NT	0.130729	5.20E-08
B88	B137	CA 242	AFU	-0.11922	7.00E-07
B89	B137	CA 72-4	AFU	0.333274	0
B89	B138	CA 72-4	PAB	0.153954	1.34E-10
B89	B139	CA 72-4	ADA	-0.14598	1.16E-09
B89	B134	CA 72-4	CHE	0.122289	3.58E-07
B9	B35	RH	β2-MG	0.713974	0
B90	B184	CYFRA 21-1	INR	0.227589	0
B90	B185	CYFRA 21-1	PT	0.24048	0
B90	B123	CYFRA 21-1	hs-CRP1	0.153738	1.43E-10
B90	B187	CYFRA 21-1	NSE	0.119755	6.23E-07
B91	B126	TP	Alb	0.499596	0
B91	B127	TP	A-G ratio	-0.4909	0
B91	B130	TP	GLB	0.812667	0
B91	B188	TP	Ca	0.254618	0
B91	B218	TP	DBP	0.138361	8.16E-09
B92	B119	ALDH2-Glu504Glu	Hcv-rna	-0.36478	0
B92	B124	ALDH2-Glu504Glu	ACTH	-0.2894	0
B92	B125	ALDH2-Glu504Glu	Cor	-0.44727	0
B92	B152	ALDH2-Glu504Glu	ACA-Ig(G-A-M)	0.364851	0
B92	B158	ALDH2-Glu504Glu	IgM	0.358725	0
B92	B159	ALDH2-Glu504Glu	β2-MG	0.364774	0
B92	B93	ALDH2-Glu504Glu	ALDH2-Glu504Lys	-0.99847	0
B92	B101	ALDH2-Glu504Glu	anti-Sc70	-0.18845	3.11E-15
B92	B102	ALDH2-Glu504Glu	anti-Sm	-0.18845	3.11E-15
B92	B103	ALDH2-Glu504Glu	anti-SSA/Ro	-0.18845	3.11E-15
B92	B104	ALDH2-Glu504Glu	anti-SSB	-0.18845	3.11E-15
B92	B105	ALDH2-Glu504Glu	anti-U1RNP	-0.18845	3.11E-15
B92	B106	ALDH2-Glu504Glu	anti-P	-0.18845	3.11E-15
B92	B107	ALDH2-Glu504Glu	AnuA	-0.18845	3.11E-15
B92	B108	ALDH2-Glu504Glu	Anti-Cyclin	-0.18845	3.11E-15
B92	B109	ALDH2-Glu504Glu	AMA-M2	-0.18845	3.11E-15
B92	B110	ALDH2-Glu504Glu	AHA	-0.18845	3.11E-15
B92	B95	ALDH2-Glu504Glu	anti-cenpb	-0.18845	3.11E-15
B92	B97	ALDH2-Glu504Glu	dsDNA	-0.18845	3.11E-15
B92	B98	ALDH2-Glu504Glu	anti-Jo-1	-0.18845	3.11E-15
B92	B99	ALDH2-Glu504Glu	anti-PM-Scl	-0.18845	3.11E-15
B92	B114	ALDH2-Glu504Glu	TSH TT3 TT4(TT4)	-0.17109	8.84E-13
B92	B179	ALDH2-Glu504Glu	α1-MG	0.169721	1.35E-12
B92	B120	ALDH2-Glu504Glu	HCV-cAg	-0.16747	2.68E-12
B92	B94	ALDH2-Glu504Glu	ALDH2-Lys504Lys	-0.15928	2.98E-11
B92	B156	ALDH2-Glu504Glu	IgE	-0.13423	2.25E-08
B92	B134	ALDH2-Glu504Glu	CHE	0.124259	2.31E-07
B92	B155	ALDH2-Glu504Glu	IgA	-0.11991	6.03E-07

B93	B119	ALDH2-Glu504Lys	Hcv-rna	0.339553	0
B93	B124	ALDH2-Glu504Lys	ACTH	0.269373	0
B93	B125	ALDH2-Glu504Lys	Cor	0.416298	0
B93	B152	ALDH2-Glu504Lys	ACA-Ig(G-A-M)	-0.33962	0
B93	B158	ALDH2-Glu504Lys	IgM	-0.33373	0
B93	B159	ALDH2-Glu504Lys	β2-MG	-0.33955	0
B93	B101	ALDH2-Glu504Lys	anti-Sc70	0.175562	2.18E-13
B93	B102	ALDH2-Glu504Lys	anti-Sm	0.175562	2.18E-13
B93	B103	ALDH2-Glu504Lys	anti-SSA/Ro	0.175562	2.18E-13
B93	B104	ALDH2-Glu504Lys	anti-SSB	0.175562	2.18E-13
B93	B105	ALDH2-Glu504Lys	anti-U1RNP	0.175562	2.18E-13
B93	B106	ALDH2-Glu504Lys	anti-P	0.175562	2.18E-13
B93	B107	ALDH2-Glu504Lys	AnuA	0.175561	2.18E-13
B93	B108	ALDH2-Glu504Lys	Anti-Cyclin	0.175562	2.18E-13
B93	B109	ALDH2-Glu504Lys	AMA-M2	0.175562	2.18E-13
B93	B110	ALDH2-Glu504Lys	AHA	0.175562	2.18E-13
B93	B95	ALDH2-Glu504Lys	anti-cenpb	0.175562	2.18E-13
B93	B97	ALDH2-Glu504Lys	dsDNA	0.175562	2.18E-13
B93	B98	ALDH2-Glu504Lys	anti-Jo-1	0.175562	2.18E-13
B93	B99	ALDH2-Glu504Lys	anti-PM-Scl	0.175562	2.18E-13
B93	B114	ALDH2-Glu504Lys	TSH TT3 TT4(TT4)	0.166503	3.58E-12
B93	B179	ALDH2-Glu504Lys	α1-MG	-0.15794	4.38E-11
B93	B120	ALDH2-Glu504Lys	HCV-cAg	0.156061	7.45E-11
B93	B156	ALDH2-Glu504Lys	IgE	0.124988	1.96E-07
B94	B101	ALDH2-Lys504Lys	anti-Sc70	0.255109	0
B94	B102	ALDH2-Lys504Lys	anti-Sm	0.255109	0
B94	B103	ALDH2-Lys504Lys	anti-SSA/Ro	0.255109	0
B94	B104	ALDH2-Lys504Lys	anti-SSB	0.255109	0
B94	B105	ALDH2-Lys504Lys	anti-U1RNP	0.255109	0
B94	B106	ALDH2-Lys504Lys	anti-P	0.255109	0
B94	B107	ALDH2-Lys504Lys	AnuA	0.255109	0
B94	B108	ALDH2-Lys504Lys	Anti-Cyclin	0.255109	0
B94	B109	ALDH2-Lys504Lys	AMA-M2	0.255109	0
B94	B110	ALDH2-Lys504Lys	AHA	0.255109	0
B94	B115	ALDH2-Lys504Lys	TSH TT3 TT4(TT3)	-0.21858	0
B94	B119	ALDH2-Lys504Lys	Hcv-rna	0.498836	0
B94	B120	ALDH2-Lys504Lys	HCV-cAg	0.225864	0
B94	B124	ALDH2-Lys504Lys	ACTH	0.396008	0
B94	B125	ALDH2-Lys504Lys	Cor	0.612351	0
B94	B152	ALDH2-Lys504Lys	ACA-Ig(G-A-M)	-0.49894	0
B94	B158	ALDH2-Lys504Lys	IgM	-0.49386	0
B94	B159	ALDH2-Lys504Lys	β2-MG	-0.49883	0
B94	B179	ALDH2-Lys504Lys	α1-MG	-0.23286	0
B94	B95	ALDH2-Lys504Lys	anti-cenpb	0.255109	0

B94	B97	ALDH2-Lys504Lys	dsDNA	0.255109	0
B94	B98	ALDH2-Lys504Lys	anti-Jo-1	0.255109	0
B94	B99	ALDH2-Lys504Lys	anti-PM-Scl	0.255109	0
B94	B156	ALDH2-Lys504Lys	IgE	0.182861	2.04E-14
B94	B134	ALDH2-Lys504Lys	CHE	-0.16818	2.15E-12
B94	B155	ALDH2-Lys504Lys	IgA	0.16461	6.30E-12
B94	B151	ALDH2-Lys504Lys	Anti-CCP	0.155926	7.74E-11
B94	B203	ALDH2-Lys504Lys	DH	-0.15198	2.31E-10
B94	B184	ALDH2-Lys504Lys	INR	0.144909	1.53E-09
B94	B181	ALDH2-Lys504Lys	APTT	0.132409	3.49E-08
B94	B186	ALDH2-Lys504Lys	PFC	-0.132	3.85E-08
B94	B182	ALDH2-Lys504Lys	TT	0.125636	1.69E-07
B94	B199	ALDH2-Lys504Lys	Mg	-0.1191	7.18E-07
B95	B103	anti-cenpb	anti-SSA/Ro	1	0
B95	B104	anti-cenpb	anti-SSB	1	0
B95	B105	anti-cenpb	anti-U1RNP	1	0
B95	B107	anti-cenpb	AnuA	0.999999	0
B95	B108	anti-cenpb	Anti-Cyclin	1	0
B95	B110	anti-cenpb	AHA	1	0
B95	B119	anti-cenpb	Hcv-rna	0.384926	0
B95	B120	anti-cenpb	HCV-cAg	0.349051	0
B95	B121	anti-cenpb	C3	-0.27501	0
B95	B122	anti-cenpb	C4	0.242862	0
B95	B125	anti-cenpb	Cor	0.298639	0
B95	B134	anti-cenpb	CHE	-0.23432	0
B95	B151	anti-cenpb	Anti-CCP	0.245648	0
B95	B152	anti-cenpb	ACA-Ig(G-A-M)	-0.385	0
B95	B156	anti-cenpb	IgE	0.234142	0
B95	B157	anti-cenpb	IgG	0.328845	0
B95	B159	anti-cenpb	β2-MG	-0.38492	0
B95	B97	anti-cenpb	dsDNA	1	0
B95	B138	anti-cenpb	PAB	-0.18058	4.35E-14
B95	B154	anti-cenpb	RF	0.158265	3.99E-11
B95	B178	anti-cenpb	Ig-U	0.151079	2.96E-10
B95	B193	anti-cenpb	K	0.125601	1.70E-07
B95	B182	anti-cenpb	TT	0.124542	2.17E-07
B95	B183	anti-cenpb	PTA	-0.11862	7.97E-07
B96	B116	dsDNA (IIF)	TT3	-0.86844	0
B97	B101	dsDNA	anti-Sc70	1	0
B97	B102	dsDNA	anti-Sm	1	0
B97	B104	dsDNA	anti-SSB	1	0
B97	B106	dsDNA	anti-P	1	0
B97	B107	dsDNA	AnuA	0.999999	0
B97	B110	dsDNA	AHA	1	0

B97	B119	dsDNA	Hcv-rna	0.384926	0
B97	B120	dsDNA	HCV-cAg	0.349051	0
B97	B121	dsDNA	C3	-0.27501	0
B97	B122	dsDNA	C4	0.242862	0
B97	B125	dsDNA	Cor	0.298639	0
B97	B134	dsDNA	CHE	-0.23432	0
B97	B151	dsDNA	Anti-CCP	0.245648	0
B97	B152	dsDNA	ACA-Ig(G-A-M)	-0.38501	0
B97	B156	dsDNA	IgE	0.234142	0
B97	B157	dsDNA	IgG	0.328845	0
B97	B159	dsDNA	β2-MG	-0.38492	0
B97	B99	dsDNA	anti-PM-Scl	1	0
B97	B138	dsDNA	PAB	-0.18058	4.35E-14
B97	B154	dsDNA	RF	0.158265	3.99E-11
B97	B178	dsDNA	Ig-U	0.151079	2.96E-10
B97	B193	dsDNA	K	0.125601	1.70E-07
B97	B182	dsDNA	TT	0.124542	2.17E-07
B97	B183	dsDNA	PTA	-0.11862	7.97E-07
B98	B107	anti-Jo-1	AnuA	0.999999	0
B98	B119	anti-Jo-1	Hcv-rna	0.384926	0
B98	B120	anti-Jo-1	HCV-cAg	0.349051	0
B98	B121	anti-Jo-1	C3	-0.27501	0
B98	B122	anti-Jo-1	C4	0.242862	0
B98	B125	anti-Jo-1	Cor	0.298639	0
B98	B134	anti-Jo-1	CHE	-0.23432	0
B98	B151	anti-Jo-1	Anti-CCP	0.245648	0
B98	B152	anti-Jo-1	ACA-Ig(G-A-M)	-0.38501	0
B98	B156	anti-Jo-1	IgE	0.234142	0
B98	B157	anti-Jo-1	IgG	0.328845	0
B98	B159	anti-Jo-1	β2-MG	-0.38492	0
B98	B138	anti-Jo-1	PAB	-0.18058	4.35E-14
B98	B154	anti-Jo-1	RF	0.158265	3.99E-11
B98	B178	anti-Jo-1	Ig-U	0.151079	2.96E-10
B98	B193	anti-Jo-1	K	0.125601	1.70E-07
B98	B182	anti-Jo-1	TT	0.124542	2.17E-07
B98	B183	anti-Jo-1	PTA	-0.11862	7.97E-07
B99	B101	anti-PM-Scl	anti-Sc70	1	0
B99	B102	anti-PM-Scl	anti-Sm	1	0
B99	B104	anti-PM-Scl	anti-SSB	1	0
B99	B107	anti-PM-Scl	AnuA	0.999999	0
B99	B108	anti-PM-Scl	Anti-Cyclin	1	0
B99	B119	anti-PM-Scl	Hcv-rna	0.384926	0
B99	B120	anti-PM-Scl	HCV-cAg	0.349051	0
B99	B121	anti-PM-Scl	C3	-0.27501	0

B99	B122	anti-PM-Scl	C4	0.242862	0
B99	B125	anti-PM-Scl	Cor	0.298639	0
B99	B134	anti-PM-Scl	CHE	-0.23432	0
B99	B151	anti-PM-Scl	Anti-CCP	0.245648	0
B99	B152	anti-PM-Scl	ACA-Ig(G-A-M)	-0.38501	0
B99	B156	anti-PM-Scl	IgE	0.234142	0
B99	B157	anti-PM-Scl	IgG	0.328845	0
B99	B159	anti-PM-Scl	β2-MG	-0.38492	0
B99	B138	anti-PM-Scl	PAB	-0.18058	4.35E-14
B99	B154	anti-PM-Scl	RF	0.158265	3.99E-11
B99	B178	anti-PM-Scl	Ig-U	0.151079	2.96E-10
B99	B193	anti-PM-Scl	K	0.125601	1.70E-07
B99	B182	anti-PM-Scl	TT	0.124542	2.17E-07
B99	B183	anti-PM-Scl	PTA	-0.11862	7.97E-07

Supplementary Table 8. PEI Correlations in Coronary heart disease

Row	Column	Row	Column	Cor	*P*
B1	B161	Gender	LEU	0.224483458	0
B1	B163	Gender	SG	-0.303509972	0
B1	B174	Gender	Urea/Cr	0.360119848	0
B1	B190	Gender	Cre	-0.44189101	0
B1	B192	Gender	UA	-0.3679103	0
B1	B206	Gender	Waist	-0.231726663	0
B1	B209	Gender	Smoking	-0.233703562	0
B1	B216	Gender	BMI	-0.434121969	0
B1	B221	Gender	Hei	-0.640784165	0
B1	B222	Gender	NS	-0.563973997	0
B1	B45	Gender	MCHC	-0.271118031	0
B1	B46	Gender	RBC Count	-0.354552338	0
B1	B51	Gender	Hb	-0.479672263	0
B1	B72	Gender	HDL-C	0.353525548	0
B1	B77	Gender	TC	0.277378551	0
B1	B207	Gender	WHR	-0.220945924	4.44E-16
B1	B42	Gender	MON	-0.220061298	4.44E-16
B1	B141	Gender	AST/ALT	0.216882862	1.33E-15
B1	B205	Gender	KDA	0.211720674	6.66E-15
B1	B132	Gender	Dbil	-0.20649327	3.15E-14
B1	B130	Gender	GLB	0.18629387	8.22E-12
B1	B2	Gender	Age	0.182663651	2.10E-11
B1	B219	Gender	OT	-0.178847395	5.49E-11
B1	B204	Gender	Drinking	-0.178157582	6.52E-11
B1	B127	Gender	A-G ratio	-0.17775302	7.21E-11
B1	B128	Gender	ALT	-0.175778083	1.17E-10
B1	B220	Gender	SBP	0.175649265	1.21E-10
B1	B41	Gender	MON %	-0.17009921	4.63E-10

				7	
B1	B133	Gender	TBIL	-0.161908336	3.08E-09
B1	B70	Gender	LDL-C	0.154279223	1.66E-08
B1	B210	Gender	Hip	-0.144681217	1.23E-07
B1	B48	Gender	LYM%	0.14414447	1.37E-07
B1	B50	Gender	eo%	-0.143439195	1.58E-07
B1	B74	Gender	ApoA1	0.141575684	2.28E-07
B10	B11	PG I	PGI/PGII	0.261067925	0
B10	B12	PG I	PG II	0.457146812	0
B10	B165	PG I	bilirubin	0.410825967	0
B10	B227	PG I	OD	0.205903407	3.75E-14
B10	B14	PG I	G-17	0.17916346	5.08E-11
B10	B112	PG I	FT4	0.146588236	8.33E-08
B100	B119	anti-Ro52	Hcv-rna	0.161376293	3.48E-09
B100	B197	anti-Ro52	AG	-0.150214952	3.93E-08
B100	B115	anti-Ro52	TSH TT3 TT4(TT3)	0.142367131	1.95E-07
B101	B104	anti-Sc70	anti-SSB	0.999999993	0
B101	B107	anti-Sc70	AnuA	0.999999468	0
B101	B119	anti-Sc70	Hcv-rna	0.470017916	0
B101	B120	anti-Sc70	HCV-cAg	0.311176082	0
B101	B125	anti-Sc70	Cor	-0.319333509	0
B101	B152	anti-Sc70	ACA-Ig(G-A-M)	-0.263417922	0
B101	B154	anti-Sc70	RF	0.277811861	0
B101	B155	anti-Sc70	IgA	-0.244461163	0
B101	B158	anti-Sc70	IgM	0.292027928	0
B101	B159	anti-Sc70	β2-MG	-0.263404818	0
B101	B169	anti-Sc70	U-CRY	-0.263405908	0
B101	B197	anti-Sc70	AG	0.240927349	0
B101	B199	anti-Sc70	Mg	0.216390069	1.78E-15
B101	B137	anti-Sc70	AFU	0.204688407	5.33E-14
B101	B134	anti-Sc70	CHE	0.187404368	6.15E-12
B101	B200	anti-Sc70	OP	0.187095702	6.66E-12
B101	B195	anti-Sc70	Na	0.184163402	1.43E-11
B101	B193	anti-Sc70	K	0.182545786	2.16E-11
B101	B108	anti-Sc70	Anti-Cyclin	-0.18087605	3.30E-11

				3	
B101	B114	anti-Sc70	TSH TT3 TT4(TT4)	0.173261898	2.17E-10
B101	B138	anti-Sc70	PAB	0.164858125	1.57E-09
B101	B136	anti-Sc70	5'NT	0.151810374	2.81E-08
B101	B196	anti-Sc70	TCO2	-0.138358662	4.28E-07
B102	B103	anti-Sm	anti-SSA/Ro	0.999999995	0
B102	B104	anti-Sm	anti-SSB	0.999999993	0
B102	B105	anti-Sm	anti-U1RNP	0.999999995	0
B102	B107	anti-Sm	AnuA	0.9999996	0
B102	B109	anti-Sm	AMA-M2	0.999999997	0
B102	B110	anti-Sm	AHA	0.999999992	0
B102	B119	anti-Sm	Hcv-rna	0.470017904	0
B102	B120	anti-Sm	HCV-cAg	0.311176286	0
B102	B125	anti-Sm	Cor	-0.319333499	0
B102	B152	anti-Sm	ACA-Ig(G-A-M)	-0.26341618	0
B102	B154	anti-Sm	RF	0.277811854	0
B102	B155	anti-Sm	IgA	-0.244461157	0
B102	B158	anti-Sm	IgM	0.292027921	0
B102	B159	anti-Sm	β2-MG	-0.263403589	0
B102	B169	anti-Sm	U-CRY	-0.263405001	0
B102	B197	anti-Sm	AG	0.240927343	0
B102	B199	anti-Sm	Mg	0.216390066	1.78E-15
B102	B137	anti-Sm	AFU	0.204688403	5.33E-14
B102	B134	anti-Sm	CHE	0.187404363	6.15E-12
B102	B200	anti-Sm	OP	0.187095702	6.66E-12
B102	B195	anti-Sm	Na	0.184163403	1.43E-11
B102	B193	anti-Sm	K	0.182545783	2.16E-11
B102	B108	anti-Sm	Anti-Cyclin	-0.180876048	3.30E-11
B102	B114	anti-Sm	TSH TT3 TT4(TT4)	0.173261894	2.17E-10
B102	B138	anti-Sm	PAB	0.164858122	1.57E-09
B102	B136	anti-Sm	5'NT	0.151810371	2.81E-08
B102	B196	anti-Sm	TCO2	-0.138358658	4.28E-07
B103	B104	anti-SSA/Ro	anti-SSB	0.999999999	0
B103	B107	anti-SSA/Ro	AnuA	0.999999435	0
B103	B119	anti-SSA/Ro	Hcv-rna	0.470017907	0
B103	B120	anti-SSA/Ro	HCV-cAg	0.311176207	0
B103	B125	anti-SSA/Ro	Cor	-0.31933350	0

				3	
B103	B152	anti-SSA/Ro	ACA-Ig(G-A-M)	-0.26341622<br>8	0
B103	B154	anti-SSA/Ro	RF	0.277811855	0
B103	B155	anti-SSA/Ro	IgA	-0.24446115<br>9	0
B103	B158	anti-SSA/Ro	IgM	0.292027922	0
B103	B159	anti-SSA/Ro	β2-MG	-0.26340408<br>6	0
B103	B169	anti-SSA/Ro	U-CRY	-0.26340544<br>9	0
B103	B197	anti-SSA/Ro	AG	0.240927345	0
B103	B199	anti-SSA/Ro	Mg	0.216390065	1.78E-15
B103	B137	anti-SSA/Ro	AFU	0.204688404	5.33E-14
B103	B134	anti-SSA/Ro	CHE	0.187404364	6.15E-12
B103	B200	anti-SSA/Ro	OP	0.187095704	6.66E-12
B103	B195	anti-SSA/Ro	Na	0.184163407	1.43E-11
B103	B193	anti-SSA/Ro	K	0.182545784	2.16E-11
B103	B108	anti-SSA/Ro	Anti-Cyclin	-0.18087604<br>9	3.30E-11
B103	B114	anti-SSA/Ro	TSH TT3 TT4(TT4)	0.173261895	2.17E-10
B103	B138	anti-SSA/Ro	PAB	0.164858123	1.57E-09
B103	B136	anti-SSA/Ro	5'NT	0.151810372	2.81E-08
B103	B196	anti-SSA/Ro	TCO2	-0.13835865<br>9	4.28E-07
B104	B106	anti-SSB	anti-P	0.99999999	0
B104	B107	anti-SSB	AnuA	0.999999493	0
B104	B110	anti-SSB	AHA	0.999999994	0
B104	B119	anti-SSB	Hcv-rna	0.470017908	0
B104	B120	anti-SSB	HCV-cAg	0.311176126	0
B104	B125	anti-SSB	Cor	-0.31933350<br>3	0
B104	B152	anti-SSB	ACA-Ig(G-A-M)	-0.26341558<br>2	0
B104	B154	anti-SSB	RF	0.277811855	0
B104	B155	anti-SSB	IgA	-0.24446115<br>9	0
B104	B158	anti-SSB	IgM	0.292027922	0
B104	B159	anti-SSB	β2-MG	-0.26340444<br>4	0
B104	B169	anti-SSB	U-CRY	-0.26340569<br>7	0
B104	B197	anti-SSB	AG	0.240927345	0
B104	B199	anti-SSB	Mg	0.216390064	1.78E-15

B104	B137	anti-SSB	AFU	0.204688404	5.33E-14
B104	B134	anti-SSB	CHE	0.187404364	6.15E-12
B104	B200	anti-SSB	OP	0.187095705	6.66E-12
B104	B195	anti-SSB	Na	0.184163405	1.43E-11
B104	B193	anti-SSB	K	0.182545784	2.16E-11
B104	B108	anti-SSB	Anti-Cyclin	-0.18087605	3.30E-11
B104	B114	anti-SSB	TSH TT3 TT4(TT4)	0.173261895	2.17E-10
B104	B138	anti-SSB	PAB	0.164858123	1.57E-09
B104	B136	anti-SSB	5'NT	0.151810372	2.81E-08
B104	B196	anti-SSB	TCO2	-0.13835866	4.28E-07
B105	B107	anti-U1RNP	AnuA	0.999999418	0
B105	B119	anti-U1RNP	Hcv-rna	0.470017908	0
B105	B120	anti-U1RNP	HCV-cAg	0.311176192	0
B105	B125	anti-U1RNP	Cor	-0.319333502	0
B105	B152	anti-U1RNP	ACA-Ig(G-A-M)	-0.26341651	0
B105	B154	anti-U1RNP	RF	0.277811856	0
B105	B155	anti-U1RNP	IgA	-0.244461159	0
B105	B158	anti-U1RNP	IgM	0.292027923	0
B105	B159	anti-U1RNP	β2-MG	-0.263404289	0
B105	B169	anti-U1RNP	U-CRY	-0.263405283	0
B105	B197	anti-U1RNP	AG	0.240927345	0
B105	B199	anti-U1RNP	Mg	0.216390065	1.78E-15
B105	B137	anti-U1RNP	AFU	0.204688404	5.33E-14
B105	B134	anti-U1RNP	CHE	0.187404365	6.15E-12
B105	B200	anti-U1RNP	OP	0.187095705	6.66E-12
B105	B195	anti-U1RNP	Na	0.184163407	1.43E-11
B105	B193	anti-U1RNP	K	0.182545784	2.16E-11
B105	B108	anti-U1RNP	Anti-Cyclin	-0.18087605	3.30E-11
B105	B114	anti-U1RNP	TSH TT3 TT4(TT4)	0.173261895	2.17E-10
B105	B138	anti-U1RNP	PAB	0.164858123	1.57E-09
B105	B136	anti-U1RNP	5'NT	0.151810372	2.81E-08
B105	B196	anti-U1RNP	TCO2	-0.138358659	4.28E-07
B106	B107	anti-P	AnuA	0.999999631	0
B106	B119	anti-P	Hcv-rna	0.470017909	0
B106	B120	anti-P	HCV-cAg	0.311176275	0
B106	B125	anti-P	Cor	-0.3193335	0
B106	B152	anti-P	ACA-Ig(G-A-M)	-0.263416046	0
B106	B154	anti-P	RF	0.277811857	0

B106	B155	anti-P	IgA	-0.244461159	0
B106	B158	anti-P	IgM	0.292027924	0
B106	B159	anti-P	β2-MG	-0.26340441	0
B106	B169	anti-P	U-CRY	-0.263405089	0
B106	B197	anti-P	AG	0.240927346	0
B106	B199	anti-P	Mg	0.216390066	1.78E-15
B106	B137	anti-P	AFU	0.204688405	5.33E-14
B106	B134	anti-P	CHE	0.187404365	6.15E-12
B106	B200	anti-P	OP	0.187095699	6.66E-12
B106	B195	anti-P	Na	0.184163403	1.43E-11
B106	B193	anti-P	K	0.182545786	2.16E-11
B106	B108	anti-P	Anti-Cyclin	-0.180876051	3.30E-11
B106	B114	anti-P	TSH TT3 TT4(TT4)	0.173261896	2.17E-10
B106	B138	anti-P	PAB	0.164858124	1.57E-09
B106	B136	anti-P	5'NT	0.151810372	2.81E-08
B106	B196	anti-P	TCO2	-0.138358659	4.28E-07
B107	B109	AnuA	AMA-M2	0.999999447	0
B107	B110	AnuA	AHA	0.999999467	0
B107	B119	AnuA	Hcv-rna	0.47001764	0
B107	B120	AnuA	HCV-cAg	0.311176813	0
B107	B125	AnuA	Cor	-0.319333283	0
B107	B152	AnuA	ACA-Ig(G-A-M)	-0.263412426	0
B107	B154	AnuA	RF	0.277811698	0
B107	B155	AnuA	IgA	-0.244461016	0
B107	B158	AnuA	IgM	0.292027759	0
B107	B159	AnuA	β2-MG	-0.263402253	0
B107	B169	AnuA	U-CRY	-0.263401605	0
B107	B197	AnuA	AG	0.240927208	0
B107	B199	AnuA	Mg	0.21638995	1.78E-15
B107	B137	AnuA	AFU	0.204688287	5.33E-14
B107	B134	AnuA	CHE	0.187404261	6.15E-12
B107	B200	AnuA	OP	0.187095577	6.67E-12
B107	B195	AnuA	Na	0.184163278	1.43E-11
B107	B193	AnuA	K	0.182545683	2.16E-11
B107	B108	AnuA	Anti-Cyclin	-0.18087594	3.30E-11

				8	
B107	B114	AnuA	TSH TT3 TT4(TT4)	0.173261795	2.17E-10
B107	B138	AnuA	PAB	0.164858031	1.57E-09
B107	B136	AnuA	5'NT	0.151810285	2.81E-08
B107	B196	AnuA	TCO2	-0.138358582	4.28E-07
B108	B151	Anti-Cyclin	Anti-CCP	0.277090214	0
B108	B156	Anti-Cyclin	IgE	0.452888359	0
B108	B158	Anti-Cyclin	IgM	0.228221872	0
B108	B179	Anti-Cyclin	α1-MG	0.272315859	0
B108	B194	Anti-Cyclin	Cl	0.211036689	8.44E-15
B108	B157	Anti-Cyclin	IgG	0.192922904	1.42E-12
B108	B109	Anti-Cyclin	AMA-M2	-0.180876049	3.30E-11
B108	B110	Anti-Cyclin	AHA	-0.18087605	3.30E-11
B108	B195	Anti-Cyclin	Na	0.137596226	4.96E-07
B109	B119	AMA-M2	Hcv-rna	0.470017907	0
B109	B120	AMA-M2	HCV-cAg	0.311176226	0
B109	B125	AMA-M2	Cor	-0.319333502	0
B109	B152	AMA-M2	ACA-Ig(G-A-M)	-0.263416311	0
B109	B154	AMA-M2	RF	0.277811855	0
B109	B155	AMA-M2	IgA	-0.244461158	0
B109	B158	AMA-M2	IgM	0.292027922	0
B109	B159	AMA-M2	β2-MG	-0.263403875	0
B109	B169	AMA-M2	U-CRY	-0.263405201	0
B109	B197	AMA-M2	AG	0.240927345	0
B109	B199	AMA-M2	Mg	0.216390065	1.78E-15
B109	B137	AMA-M2	AFU	0.204688404	5.33E-14
B109	B134	AMA-M2	CHE	0.187404364	6.15E-12
B109	B200	AMA-M2	OP	0.187095704	6.66E-12
B109	B195	AMA-M2	Na	0.184163408	1.43E-11
B109	B193	AMA-M2	K	0.182545784	2.16E-11
B109	B114	AMA-M2	TSH TT3 TT4(TT4)	0.173261895	2.17E-10
B109	B138	AMA-M2	PAB	0.164858123	1.57E-09
B109	B136	AMA-M2	5'NT	0.151810372	2.81E-08
B109	B196	AMA-M2	TCO2	-0.138358658	4.28E-07
B11	B12	PGI/PGII	PG II	-0.527260379	0

B11	B139	PGI/PGII	ADA	0.179276844	4.93E-11
B11	B153	PGI/PGII	Glu	0.177111101	8.46E-11
B11	B137	PGI/PGII	AFU	0.155096173	1.39E-08
B11	B135	PGI/PGII	TBA	0.147746289	6.57E-08
B11	B14	PGI/PGII	G-17	-0.14382958	1.46E-07
B11	B200	PGI/PGII	OP	0.139675073	3.32E-07
B110	B119	AHA	Hcv-rna	0.470017908	0
B110	B120	AHA	HCV-cAg	0.311176178	0
B110	B125	AHA	Cor	-0.319333502	0
B110	B152	AHA	ACA-Ig(G-A-M)	-0.263416304	0
B110	B154	AHA	RF	0.277811856	0
B110	B155	AHA	IgA	-0.244461159	0
B110	B158	AHA	IgM	0.292027923	0
B110	B159	AHA	β2-MG	-0.263404134	0
B110	B169	AHA	U-CRY	-0.26340539	0
B110	B197	AHA	AG	0.240927346	0
B110	B199	AHA	Mg	0.216390065	1.78E-15
B110	B137	AHA	AFU	0.204688404	5.33E-14
B110	B134	AHA	CHE	0.187404365	6.15E-12
B110	B200	AHA	OP	0.187095703	6.66E-12
B110	B195	AHA	Na	0.184163406	1.43E-11
B110	B193	AHA	K	0.182545784	2.16E-11
B110	B114	AHA	TSH TT3 TT4(TT4)	0.173261895	2.17E-10
B110	B138	AHA	PAB	0.164858123	1.57E-09
B110	B136	AHA	5'NT	0.151810372	2.81E-08
B110	B196	AHA	TCO2	-0.138358659	4.28E-07
B111	B203	TSH	DH	0.177199096	8.27E-11
B111	B112	TSH	FT4	-0.164129984	1.86E-09
B111	B115	TSH	TSH TT3 TT4(TT3)	-0.158408039	6.74E-09
B111	B113	TSH	FT3	-0.145986901	9.42E-08
B112	B113	FT4	FT3	0.441320394	0
B112	B114	FT4	TSH TT3 TT4(TT4)	0.339311682	0
B112	B227	FT4	OD	0.18764734	5.77E-12
B112	B149	FT4	TGAb	0.180539099	3.59E-11
B113	B115	FT3	TSH TT3 TT4(TT3)	0.360796287	0
B114	B115	TSH TT3 TT4(TT4)	TSH TT3 TT4(TT3)	0.226830476	0

B115	B119	TSH TT3 TT4(TT3)	Hcv-rna	0.211830085	6.66E-15
B115	B194	TSH TT3 TT4(TT3)	Cl	0.17163294	3.21E-10
B115	B126	TSH TT3 TT4(TT3)	Alb	0.16958152	5.23E-10
B115	B139	TSH TT3 TT4(TT3)	ADA	-0.16817448<br>1	7.28E-10
B115	B134	TSH TT3 TT4(TT3)	CHE	0.162051053	2.98E-09
B115	B120	TSH TT3 TT4(TT3)	HCV-cAg	0.15336224	2.02E-08
B115	B195	TSH TT3 TT4(TT3)	Na	0.145095615	1.13E-07
B116	B167	TT3	PC	0.359380749	0
B116	B202	TT3	e-times	0.738141684	0
B117	B202	TT4	e-times	0.82486282	0
B119	B120	Hcv-rna	HCV-cAg	0.56199294	0
B119	B125	Hcv-rna	Cor	-0.42007021<br>2	0
B119	B134	Hcv-rna	CHE	0.256589275	0
B119	B138	Hcv-rna	PAB	0.326353159	0
B119	B139	Hcv-rna	ADA	-0.38608486<br>1	0
B119	B152	Hcv-rna	ACA-Ig(G-A-M)	-0.70183693<br>8	0
B119	B153	Hcv-rna	Glu	-0.26336467<br>8	0
B119	B155	Hcv-rna	IgA	-0.29702081<br>6	0
B119	B159	Hcv-rna	β2-MG	-0.70180544<br>6	0
B119	B169	Hcv-rna	U-CRY	-0.70180892<br>2	0
B119	B195	Hcv-rna	Na	0.357398754	0
B119	B194	Hcv-rna	Cl	0.21770731	1.11E-15
B119	B158	Hcv-rna	IgM	0.195964093	6.19E-13
B119	B197	Hcv-rna	AG	0.163037098	2.39E-09
B119	B157	Hcv-rna	IgG	-0.14589831<br>7	9.59E-08
B12	B84	PG II	C-PSA	0.224922884	0
B12	B111	PG II	TSH	0.205836484	3.82E-14
B12	B14	PG II	G-17	0.179834582	4.29E-11
B12	B165	PG II	bilirubin	0.144643986	1.24E-07
B12	B83	PG II	AFP	-0.13842571	4.23E-07
B12	B164	PG II	COM	-0.13621296<br>9	6.47E-07
B120	B125	HCV-cAg	Cor	-0.23881131<br>6	0
B120	B152	HCV-cAg	ACA-Ig(G-A-M)	-0.39683070	0

				6	
B120	B159	HCV-cAg	β2-MG	-0.396809587	0
B120	B169	HCV-cAg	U-CRY	-0.396809162	0
B120	B139	HCV-cAg	ADA	-0.222618568	2.22E-16
B120	B195	HCV-cAg	Na	0.210082147	1.11E-14
B120	B158	HCV-cAg	IgM	0.209094619	1.47E-14
B120	B138	HCV-cAg	PAB	0.190474809	2.73E-12
B120	B134	HCV-cAg	CHE	0.155565561	1.26E-08
B120	B154	HCV-cAg	RF	0.154455792	1.60E-08
B120	B156	HCV-cAg	IgE	-0.145518963	1.04E-07
B120	B194	HCV-cAg	Cl	0.139662235	3.33E-07
B121	B122	C3	C4	0.985295194	0
B121	B125	C3	Cor	-0.290116033	0
B121	B136	C3	5'NT	0.497710874	0
B121	B157	C3	IgG	-0.300293175	0
B121	B158	C3	IgM	-0.165119503	1.48E-09
B121	B124	C3	ACTH	-0.153965316	1.77E-08
B121	B155	C3	IgA	0.135240936	7.78E-07
B122	B125	C4	Cor	-0.361506618	0
B122	B136	C4	5'NT	0.60600754	0
B122	B157	C4	IgG	-0.318776806	0
B122	B124	C4	ACTH	-0.196401587	5.48E-13
B122	B158	C4	IgM	-0.135238041	7.78E-07
B123	B177	hs-CRP1	ACR	0.179215281	5.01E-11
B123	B213	hs-CRP1	CVA-R	0.153029595	2.17E-08
B124	B125	ACTH	Cor	0.371832818	0
B124	B136	ACTH	5'NT	-0.246827677	0
B124	B152	ACTH	ACA-Ig(G-A-M)	0.363606783	0
B124	B155	ACTH	IgA	-0.273864253	0
B124	B159	ACTH	β2-MG	0.363590621	0

B124	B169	ACTH	U-CRY	0.363592416	0
B124	B179	ACTH	α1-MG	0.3010837	0
B124	B182	ACTH	TT	-0.250885073	0
B124	B199	ACTH	Mg	-0.299417707	0
B124	B138	ACTH	PAB	0.192597437	1.55E-12
B124	B157	ACTH	IgG	-0.186135025	8.56E-12
B124	B151	ACTH	Anti-CCP	0.162358138	2.78E-09
B124	B195	ACTH	Na	-0.150389594	3.79E-08
B124	B148	ACTH	Anti_TPO	-0.139819814	3.22E-07
B125	B136	Cor	5'NT	-0.522170462	0
B125	B152	Cor	ACA-Ig(G-A-M)	0.364010052	0
B125	B159	Cor	β2-MG	0.363993846	0
B125	B169	Cor	U-CRY	0.363995638	0
B125	B199	Cor	Mg	-0.340340447	0
B125	B186	Cor	PFC	0.189790838	3.28E-12
B125	B181	Cor	APTT	-0.187361305	6.22E-12
B125	B154	Cor	RF	-0.174563408	1.58E-10
B125	B183	Cor	PTA	-0.172140031	2.84E-10
B125	B151	Cor	Anti-CCP	0.163941182	1.94E-09
B125	B179	Cor	α1-MG	0.159304919	5.52E-09
B125	B196	Cor	TCO2	0.153083144	2.14E-08
B125	B158	Cor	IgM	-0.151570166	2.95E-08
B125	B138	Cor	PAB	-0.136112463	6.59E-07
B126	B127	Alb	A-G ratio	0.514306614	0
B126	B188	Alb	Ca	0.403950605	0
B126	B189	Alb	eGFB	0.295765923	0
B126	B141	Alb	AST/ALT	-0.206196546	3.46E-14
B126	B216	Alb	BMI	0.199137655	2.57E-13
B126	B138	Alb	PAB	0.189613506	3.43E-12
B126	B221	Alb	Hei	0.183003349	1.92E-11
B126	B130	Alb	GLB	-0.17833783	6.24E-11

				9	
B126	B222	Alb	NS	0.166373658	1.11E-09
B126	B134	Alb	CHE	0.164352063	1.77E-09
B126	B139	Alb	ADA	-0.15685758<br>9	9.47E-09
B126	B128	Alb	ALT	0.152290919	2.54E-08
B126	B191	Alb	Urea	-0.13965912<br>8	3.33E-07
B127	B130	A-G ratio	GLB	-0.90446997<br>9	0
B127	B221	A-G ratio	Hei	0.225147005	0
B127	B189	A-G ratio	eGFB	0.220939194	4.44E-16
B127	B141	A-G ratio	AST/ALT	-0.20062962<br>4	1.69E-13
B127	B220	A-G ratio	SBP	-0.16709395<br>1	9.38E-10
B127	B216	A-G ratio	BMI	0.165588044	1.33E-09
B127	B222	A-G ratio	NS	0.158369577	6.80E-09
B128	B131	ALT	AST	0.675583817	0
B128	B140	ALT	r-GT	0.410977703	0
B128	B141	ALT	AST/ALT	-0.58456758<br>9	0
B128	B215	ALT	BMI	0.235433195	0
B128	B216	ALT	BMI	0.285474834	0
B128	B222	ALT	NS	0.233340901	0
B128	B206	ALT	Waist	0.220392971	4.44E-16
B128	B210	ALT	Hip	0.18601428	8.84E-12
B128	B221	ALT	Hei	0.177055566	8.57E-11
B128	B189	ALT	eGFB	0.168733687	6.39E-10
B128	B207	ALT	WHR	0.165173271	1.46E-09
B128	B213	ALT	CVA-R	0.141679147	2.24E-07
B129	B132	IBIL	Dbil	0.67701573	0
B129	B133	IBIL	TBIL	0.978991087	0
B129	B221	IBIL	Hei	0.135874519	6.90E-07
B130	B221	GLB	Hei	-0.1829539	1.95E-11
B130	B220	GLB	SBP	0.179341443	4.85E-11
B130	B178	GLB	Ig-U	0.156683974	9.84E-09
B130	B179	GLB	α1-MG	0.152370972	2.49E-08
B130	B180	GLB	Tf	0.149312848	4.75E-08
B130	B141	GLB	AST/ALT	0.143852492	1.45E-07
B130	B177	GLB	ACR	0.139366873	3.52E-07
B130	B218	GLB	DBP	0.139072717	3.73E-07
B131	B140	AST	r-GT	0.379401013	0
B131	B213	AST	CVA-R	0.319598905	0

B131	B150	AST	ALP	0.216519907	1.55E-15
B132	B133	Dbil	TBIL	0.812768155	0
B133	B221	TBIL	Hei	0.139478338	3.45E-07
B134	B138	CHE	PAB	0.541644109	0
B134	B139	CHE	ADA	-0.361097934	0
B134	B195	CHE	Na	0.220730772	4.44E-16
B134	B198	CHE	P	0.217863877	1.11E-15
B134	B153	CHE	Glu	-0.185749846	9.47E-12
B134	B193	CHE	K	0.139860482	3.20E-07
B135	B137	TBA	AFU	0.295682763	0
B135	B138	TBA	PAB	-0.290495465	0
B135	B139	TBA	ADA	0.35467039	0
B135	B194	TBA	Cl	0.182016021	2.47E-11
B135	B197	TBA	AG	-0.155982011	1.15E-08
B135	B198	TBA	P	-0.145864368	9.66E-08
B136	B157	5'NT	IgG	-0.214897818	2.66E-15
B136	B150	5'NT	ALP	0.206488242	3.18E-14
B136	B140	5'NT	r-GT	0.195577999	6.87E-13
B136	B137	5'NT	AFU	0.151863641	2.78E-08
B137	B139	AFU	ADA	0.400172241	0
B137	B153	AFU	Glu	0.273801255	0
B137	B200	AFU	OP	0.230390425	0
B137	B138	AFU	PAB	-0.222313427	2.22E-16
B137	B150	AFU	ALP	0.188832341	4.22E-12
B137	B155	AFU	IgA	-0.187371775	6.20E-12
B137	B199	AFU	Mg	0.169994635	4.74E-10
B138	B139	PAB	ADA	-0.709279996	0
B138	B153	PAB	Glu	-0.334026498	0
B138	B195	PAB	Na	0.246806345	0
B138	B200	PAB	OP	-0.153390654	2.01E-08
B138	B170	PAB	U-GLU	-0.14348572	1.56E-07
B139	B150	ADA	ALP	0.249067988	0
B139	B153	ADA	Glu	0.480663436	0

B139	B194	ADA	Cl	-0.239005322	0
B139	B195	ADA	Na	-0.31941111	0
B139	B196	ADA	TCO2	-0.275645108	0
B139	B200	ADA	OP	0.295684458	0
B139	B170	ADA	U-GLU	0.211702563	6.88E-15
B139	B146	ADA	CysC	0.179991052	4.12E-11
B139	B197	ADA	AG	0.148042644	6.18E-08
B14	B165	G-17	bilirubin	0.632681548	0
B14	B227	G-17	OD	0.248945729	0
B14	B123	G-17	hs-CRP1	0.155279957	1.34E-08
B14	B149	G-17	TGAb	0.15321584	2.08E-08
B14	B82	G-17	CEA	0.140975685	2.57E-07
B140	B150	r-GT	ALP	0.196468924	5.38E-13
B140	B141	r-GT	AST/ALT	-0.192759971	1.48E-12
B141	B206	AST/ALT	Waist	-0.27141692	0
B141	B215	AST/ALT	BMI	-0.276153474	0
B141	B216	AST/ALT	BMI	-0.360441152	0
B141	B221	AST/ALT	Hei	-0.263709661	0
B141	B222	AST/ALT	NS	-0.278076833	0
B141	B210	AST/ALT	Hip	-0.221537982	4.44E-16
B141	B189	AST/ALT	eGFB	-0.217508127	1.33E-15
B141	B207	AST/ALT	WHR	-0.213152568	4.44E-15
B142	B145	25-(OH)D	BGP	0.24374097	0
B142	B184	25-(OH)D	INR	0.335651334	0
B142	B185	25-(OH)D	PT	0.330334862	0
B142	B181	25-(OH)D	APTT	0.158693999	6.32E-09
B142	B201	25-(OH)D	e-habits	0.148795515	5.29E-08
B143	B144	β-CTx	Total-PINP	0.783055347	0
B143	B145	β-CTx	BGP	0.585470229	0
B144	B145	Total-PINP	BGP	0.670731251	0
B144	B150	Total-PINP	ALP	0.249377682	0
B145	B150	BGP	ALP	0.189591549	3.45E-12
B145	B184	BGP	INR	0.152922394	2.22E-08
B145	B227	BGP	OD	0.152383537	2.49E-08

B145	B185	BGP	PT	0.150577236	3.64E-08
B146	B190	CysC	Cre	0.205920689	3.73E-14
B146	B191	CysC	Urea	0.192100781	1.77E-12
B146	B189	CysC	eGFB	-0.18880088 3	4.26E-12
B148	B149	Anti_TPO	TGAb	0.489232853	0
B149	B165	TGAb	bilirubin	0.256008926	0
B15	B101	CD3-CD19+(B cell)	anti-Sc70	0.507137695	0
B15	B102	CD3-CD19+(B cell)	anti-Sm	0.507137683	0
B15	B103	CD3-CD19+(B cell)	anti-SSA/Ro	0.507137687	0
B15	B104	CD3-CD19+(B cell)	anti-SSB	0.507137687	0
B15	B105	CD3-CD19+(B cell)	anti-U1RNP	0.507137687	0
B15	B106	CD3-CD19+(B cell)	anti-P	0.507137689	0
B15	B107	CD3-CD19+(B cell)	AnuA	0.5071374	0
B15	B109	CD3-CD19+(B cell)	AMA-M2	0.507137686	0
B15	B110	CD3-CD19+(B cell)	AHA	0.507137688	0
B15	B119	CD3-CD19+(B cell)	Hcv-rna	0.642361166	0
B15	B120	CD3-CD19+(B cell)	HCV-cAg	0.340519736	0
B15	B121	CD3-CD19+(B cell)	C3	-0.34563901 2	0
B15	B122	CD3-CD19+(B cell)	C4	-0.28989544 6	0
B15	B124	CD3-CD19+(B cell)	ACTH	-0.26899272 6	0
B15	B125	CD3-CD19+(B cell)	Cor	-0.53171137 5	0
B15	B152	CD3-CD19+(B cell)	ACA-Ig(G-A-M)	-0.41232613 6	0
B15	B155	CD3-CD19+(B cell)	IgA	-0.27486599 7	0
B15	B158	CD3-CD19+(B cell)	IgM	0.296134198	0
B15	B159	CD3-CD19+(B cell)	β2-MG	-0.41230759 1	0
B15	B16	CD3-CD19+(B cell)	CD3+T	0.482134281	0
B15	B169	CD3-CD19+(B cell)	U-CRY	-0.41230964 1	0
B15	B18	CD3-CD19+(B cell)	CD3+CD4+helper(inducer) T	0.378926472	0
B15	B19	CD3-CD19+(B cell)	CD3+CD4+/CD3+CD8+	0.405798914	0
B15	B197	CD3-CD19+(B cell)	AG	0.241030558	0
B15	B22	CD3-CD19+(B cell)	CD3+CD8+(T)	0.42494134	0
B15	B28	CD3-CD19+(B cell)	PRL	0.237155653	0
B15	B29	CD3-CD19+(B cell)	E2	0.237155653	0
B15	B30	CD3-CD19+(B cell)	LH	-0.23715565	0

				4	
B15	B31	CD3-CD19+(B cell)	FSH	-0.237155653	0
B15	B32	CD3-CD19+(B cell)	Testo	0.237155653	0
B15	B33	CD3-CD19+(B cell)	Prog	0.237155653	0
B15	B79	CD3-CD19+(B cell)	hb V -dna	0.284326945	0
B15	B94	CD3-CD19+(B cell)	ALDH2-Lys504Lys	0.329615104	0
B15	B97	CD3-CD19+(B cell)	dsDNA	0.507137687	0
B15	B99	CD3-CD19+(B cell)	anti-PM-Scl	0.50713769	0
B15	B20	CD3-CD19+(B cell)	CD3+CD4+CD8+	0.214252989	3.11E-15
B15	B92	CD3-CD19+(B cell)	ALDH2-Glu504Glu	-0.20250422	9.95E-14
B15	B196	CD3-CD19+(B cell)	TCO2	-0.20205742	1.13E-13
B15	B199	CD3-CD19+(B cell)	Mg	0.18548082	1.02E-11
B15	B195	CD3-CD19+(B cell)	Na	0.185013722	1.15E-11
B15	B93	CD3-CD19+(B cell)	ALDH2-Glu504Lys	0.184377066	1.35E-11
B15	B100	CD3-CD19+(B cell)	anti-Ro52	0.173732141	1.93E-10
B15	B179	CD3-CD19+(B cell)	α1-MG	-0.161223942	3.60E-09
B15	B181	CD3-CD19+(B cell)	APTT	0.156465085	1.03E-08
B15	B183	CD3-CD19+(B cell)	PTA	0.143254204	1.64E-07
B150	B200	ALP	OP	0.189961631	3.13E-12
B150	B213	ALP	CVA-R	0.185020688	1.14E-11
B150	B153	ALP	Glu	0.164981702	1.53E-09
B150	B197	ALP	AG	0.136647838	5.95E-07
B151	B186	Anti-CCP	PFC	0.249789235	0
B151	B155	Anti-CCP	IgA	-0.180190496	3.92E-11
B152	B199	ACA-Ig(G-A-M)	Mg	-0.338684981	0
B152	B193	ACA-Ig(G-A-M)	K	-0.154289782	1.65E-08
B152	B179	ACA-Ig(G-A-M)	α1-MG	0.151070363	3.28E-08
B153	B170	Glu	U-GLU	0.446395089	0
B153	B194	Glu	Cl	-0.275098064	0
B153	B195	Glu	Na	-0.260061503	0
B153	B200	Glu	OP	0.299517666	0
B153	B196	Glu	TCO2	-0.164818762	1.59E-09
B154	B158	RF	IgM	0.182180847	2.37E-11
B154	B156	RF	IgE	-0.151376901	3.08E-08
B155	B157	IgA	IgG	0.314840025	0

B155	B200	IgA	OP	-0.208805741	1.60E-14
B155	B156	IgA	IgE	-0.203708971	7.06E-14
B155	B195	IgA	Na	-0.182002761	2.48E-11
B157	B158	IgG	IgM	0.180430207	3.69E-11
B157	B198	IgG	P	0.139391526	3.51E-07
B158	B195	IgM	Na	0.204190441	6.15E-14
B158	B197	IgM	AG	0.162274287	2.84E-09
B158	B200	IgM	OP	0.157599669	8.05E-09
B159	B169	β2-MG	U-CRY	0.999993797	0
B159	B199	β2-MG	Mg	-0.338669679	0
B159	B193	β2-MG	K	-0.154282871	1.66E-08
B159	B179	β2-MG	α1-MG	0.151063565	3.29E-08
B16	B101	CD3+T	anti-Sc70	0.242614894	0
B16	B102	CD3+T	anti-Sm	0.242614889	0
B16	B103	CD3+T	anti-SSA/Ro	0.242614891	0
B16	B104	CD3+T	anti-SSB	0.242614891	0
B16	B105	CD3+T	anti-U1RNP	0.242614891	0
B16	B106	CD3+T	anti-P	0.242614892	0
B16	B107	CD3+T	AnuA	0.242614751	0
B16	B109	CD3+T	AMA-M2	0.242614891	0
B16	B110	CD3+T	AHA	0.242614891	0
B16	B124	CD3+T	ACTH	-0.656136947	0
B16	B125	CD3+T	Cor	-0.500336355	0
B16	B152	CD3+T	ACA-Ig(G-A-M)	-0.32780374	0
B16	B155	CD3+T	IgA	0.23122311	0
B16	B157	CD3+T	IgG	0.406121345	0
B16	B159	CD3+T	β2-MG	-0.327788963	0
B16	B169	CD3+T	U-CRY	-0.327790592	0
B16	B17	CD3+T	CD3+CD16+CD56+NK	0.276495548	0
B16	B18	CD3+T	CD3+CD4+helper(inducer) T	0.947825442	0
B16	B19	CD3+T	CD3+CD4+/CD3+CD8+	0.410300003	0
B16	B199	CD3+T	Mg	0.365023457	0
B16	B20	CD3+T	CD3+CD4+CD8+	0.367581861	0
B16	B21	CD3+T	CD3+CD4-CD8-	0.423992737	0

B16	B22	CD3+T	CD3+CD8+(T)	0.954211859	0
B16	B79	CD3+T	hb V -dna	0.322801774	0
B16	B94	CD3+T	ALDH2-Lys504Lys	0.281956025	0
B16	B97	CD3+T	dsDNA	0.242614891	0
B16	B99	CD3+T	anti-PM-Scl	0.242614892	0
B16	B179	CD3+T	α1-MG	-0.221710131	4.44E-16
B16	B182	CD3+T	TT	0.208505688	1.78E-14
B16	B23	CD3+T	CD3-CD16+CD56+(NK)	-0.201841715	1.20E-13
B16	B121	CD3+T	C3	-0.201122037	1.47E-13
B16	B122	CD3+T	C4	-0.183889098	1.53E-11
B16	B158	CD3+T	IgM	0.180457252	3.67E-11
B16	B181	CD3+T	APTT	0.175336817	1.31E-10
B16	B92	CD3+T	ALDH2-Glu504Glu	-0.17197922	2.95E-10
B16	B73	CD3+T	ApoA	-0.159411806	5.39E-09
B16	B93	CD3+T	ALDH2-Glu504Lys	0.156493168	1.03E-08
B16	B62	CD3+T	WBHSRVI	-0.155812207	1.19E-08
B16	B200	CD3+T	OP	-0.152582386	2.38E-08
B16	B154	CD3+T	RF	0.150401011	3.78E-08
B16	B75	CD3+T	ApoA1/B	0.142991811	1.72E-07
B16	B58	CD3+T	HCT	-0.136474343	6.15E-07
B160	B191	PH	Urea	-0.213411298	4.00E-15
B160	B163	PH	SG	-0.170275506	4.44E-10
B160	B190	PH	Cre	-0.15074061	3.52E-08
B160	B192	PH	UA	-0.150684659	3.56E-08
B161	B173	LEU	NTT	0.18218184	2.37E-11
B161	B221	LEU	Hei	-0.168184074	7.27E-10
B163	B166	SG	Pro	0.278841086	0
B163	B176	SG	Ucr	0.300941415	0
B163	B191	SG	Urea	0.236318972	0
B163	B222	SG	NS	0.208398882	1.82E-14
B163	B216	SG	BMI	0.15652893	1.02E-08
B163	B190	SG	Cre	0.154269548	1.66E-08

B163	B221	SG	Hei	0.149728778	4.35E-08
B163	B205	SG	KDA	-0.135474784	7.44E-07
B163	B188	SG	Ca	-0.13451611	8.92E-07
B163	B192	SG	UA	0.134125508	9.59E-07
B165	B227	bilirubin	OD	0.402409468	0
B166	B176	Pro	Ucr	0.134929655	8.25E-07
B167	B202	PC	e-times	0.988683958	0
B169	B199	U-CRY	Mg	-0.338671316	0
B169	B193	U-CRY	K	-0.154283614	1.66E-08
B169	B179	U-CRY	α1-MG	0.151064321	3.29E-08
B17	B101	CD3+CD16+CD56+NK	anti-Sc70	0.374917967	0
B17	B102	CD3+CD16+CD56+NK	anti-Sm	0.37491796	0
B17	B103	CD3+CD16+CD56+NK	anti-SSA/Ro	0.374917962	0
B17	B104	CD3+CD16+CD56+NK	anti-SSB	0.374917963	0
B17	B105	CD3+CD16+CD56+NK	anti-U1RNP	0.374917962	0
B17	B106	CD3+CD16+CD56+NK	anti-P	0.374917964	0
B17	B107	CD3+CD16+CD56+NK	AnuA	0.374917759	0
B17	B109	CD3+CD16+CD56+NK	AMA-M2	0.374917962	0
B17	B110	CD3+CD16+CD56+NK	AHA	0.374917962	0
B17	B151	CD3+CD16+CD56+NK	Anti-CCP	0.304606065	0
B17	B186	CD3+CD16+CD56+NK	PFC	0.410000097	0
B17	B20	CD3+CD16+CD56+NK	CD3+CD4+CD8+	0.350232566	0
B17	B21	CD3+CD16+CD56+NK	CD3+CD4-CD8-	0.568994217	0
B17	B22	CD3+CD16+CD56+NK	CD3+CD8+(T)	0.278575485	0
B17	B23	CD3+CD16+CD56+NK	CD3-CD16+CD56+(NK)	-0.448508984	0
B17	B73	CD3+CD16+CD56+NK	ApoA	-0.263927353	0
B17	B81	CD3+CD16+CD56+NK	TSGF	-0.249636293	0
B17	B97	CD3+CD16+CD56+NK	dsDNA	0.374917963	0
B17	B99	CD3+CD16+CD56+NK	anti-PM-Scl	0.374917964	0
B17	B155	CD3+CD16+CD56+NK	IgA	-0.217292018	1.33E-15
B17	B158	CD3+CD16+CD56+NK	IgM	0.215941608	1.78E-15
B17	B18	CD3+CD16+CD56+NK	CD3+CD4+helper(inducer) T	0.206022395	3.64E-14
B17	B34	CD3+CD16+CD56+NK	TK	0.168754566	6.36E-10
B17	B199	CD3+CD16+CD56+NK	Mg	0.160203907	4.52E-09
B17	B135	CD3+CD16+CD56+NK	TBA	-0.158329219	6.86E-09

B17	B154	CD3+CD16+CD56+NK	RF	0.146302473	8.83E-08
B171	B172	EC	KB	-0.237892718	0
B173	B178	NTT	Ig-U	0.264394406	0
B173	B180	NTT	Tf	0.265865618	0
B173	B179	NTT	α1-MG	0.189046291	3.99E-12
B173	B177	NTT	ACR	0.140089073	3.06E-07
B174	B190	Urea/Cr	Cre	-0.42031173	0
B174	B191	Urea/Cr	Urea	0.640680359	0
B174	B192	Urea/Cr	UA	-0.224627775	0
B174	B216	Urea/Cr	BMI	-0.247815512	0
B174	B221	Urea/Cr	Hei	-0.327845559	0
B174	B222	Urea/Cr	NS	-0.26932264	0
B174	B189	Urea/Cr	eGFB	0.139689952	3.31E-07
B175	B177	ALB-U	ACR	0.928396406	0
B175	B178	ALB-U	Ig-U	0.492804645	0
B175	B179	ALB-U	α1-MG	0.360487579	0
B175	B180	ALB-U	Tf	0.493376972	0
B175	B193	ALB-U	K	0.216418468	1.78E-15
B177	B178	ACR	Ig-U	0.509307032	0
B177	B179	ACR	α1-MG	0.358794291	0
B177	B180	ACR	Tf	0.513929363	0
B177	B193	ACR	K	0.235759234	0
B178	B179	Ig-U	α1-MG	0.760738744	0
B178	B180	Ig-U	Tf	0.991249441	0
B178	B193	Ig-U	K	0.454901225	0
B178	B195	Ig-U	Na	-0.245402372	0
B178	B194	Ig-U	Cl	-0.2135711	4.00E-15
B179	B180	α1-MG	Tf	0.711709327	0
B179	B193	α1-MG	K	0.294970024	0
B179	B195	α1-MG	Na	-0.149694237	4.38E-08
B179	B182	α1-MG	TT	-0.147288752	7.22E-08
B18	B124	CD3+CD4+helper(inducer)T	ACTH	-0.786483604	0
B18	B125	CD3+CD4+helper(inducer)T	Cor	-0.610540292	0
B18	B136	CD3+CD4+helper(inducer)T	5'NT	0.24812374	0

B18	B152	CD3+CD4+helper(inducer) T	ACA-Ig(G-A-M)	-0.329073428	0
B18	B155	CD3+CD4+helper(inducer) T	IgA	0.305364428	0
B18	B157	CD3+CD4+helper(inducer) T	IgG	0.313880452	0
B18	B159	CD3+CD4+helper(inducer) T	β2-MG	-0.3290587	0
B18	B169	CD3+CD4+helper(inducer) T	U-CRY	-0.329060341	0
B18	B179	CD3+CD4+helper(inducer) T	α1-MG	-0.246599597	0
B18	B19	CD3+CD4+helper(inducer) T	CD3+CD4+/CD3+CD8+	0.619451408	0
B18	B199	CD3+CD4+helper(inducer) T	Mg	0.371020662	0
B18	B22	CD3+CD4+helper(inducer) T	CD3+CD8+(T)	0.826143564	0
B18	B79	CD3+CD4+helper(inducer) T	hb V -dna	0.283427786	0
B18	B94	CD3+CD4+helper(inducer) T	ALDH2-Lys504Lys	0.256366745	0
B18	B182	CD3+CD4+helper(inducer) T	TT	0.220417489	4.44E-16
B18	B28	CD3+CD4+helper(inducer) T	PRL	-0.203632254	7.22E-14
B18	B29	CD3+CD4+helper(inducer) T	E2	-0.203632255	7.22E-14
B18	B30	CD3+CD4+helper(inducer) T	LH	0.203632255	7.22E-14
B18	B31	CD3+CD4+helper(inducer) T	FSH	0.203632255	7.22E-14
B18	B32	CD3+CD4+helper(inducer) T	Testo	-0.203632255	7.22E-14
B18	B33	CD3+CD4+helper(inducer) T	Prog	-0.203632255	7.22E-14
B18	B21	CD3+CD4+helper(inducer) T	CD3+CD4-CD8-	0.19356722	1.19E-12
B18	B20	CD3+CD4+helper(inducer) T	CD3+CD4+CD8+	0.185439048	1.03E-11
B18	B62	CD3+CD4+helper(inducer) T	WBHSRVI	-0.167520728	8.49E-10
B18	B181	CD3+CD4+helper(inducer) T	APTT	0.165233888	1.44E-09

B18	B101	CD3+CD4+helper(inducer) T	anti-Sc70	0.157686644	7.90E-09
B18	B102	CD3+CD4+helper(inducer) T	anti-Sm	0.15768664	7.90E-09
B18	B103	CD3+CD4+helper(inducer) T	anti-SSA/Ro	0.157686641	7.90E-09
B18	B104	CD3+CD4+helper(inducer) T	anti-SSB	0.157686642	7.90E-09
B18	B105	CD3+CD4+helper(inducer) T	anti-U1RNP	0.157686641	7.90E-09
B18	B106	CD3+CD4+helper(inducer) T	anti-P	0.157686642	7.90E-09
B18	B107	CD3+CD4+helper(inducer) T	AnuA	0.157686554	7.90E-09
B18	B109	CD3+CD4+helper(inducer) T	AMA-M2	0.157686641	7.90E-09
B18	B110	CD3+CD4+helper(inducer) T	AHA	0.157686641	7.90E-09
B18	B97	CD3+CD4+helper(inducer) T	dsDNA	0.157686642	7.90E-09
B18	B99	CD3+CD4+helper(inducer) T	anti-PM-Scl	0.157686642	7.90E-09
B18	B200	CD3+CD4+helper(inducer) T	OP	-0.15655896	1.01E-08
B18	B92	CD3+CD4+helper(inducer) T	ALDH2-Glu504Glu	-0.155320241	1.32E-08
B18	B58	CD3+CD4+helper(inducer) T	HCT	-0.150699644	3.55E-08
B18	B60	CD3+CD4+helper(inducer) T	WBRVI	-0.149121541	4.94E-08
B18	B67	CD3+CD4+helper(inducer) T	WBRSR(mPas_50)	-0.143863821	1.45E-07
B18	B66	CD3+CD4+helper(inducer) T	WBRSR(mPas_100)	-0.143012651	1.72E-07
B18	B93	CD3+CD4+helper(inducer) T	ALDH2-Glu504Lys	0.141256608	2.43E-07
B18	B89	CD3+CD4+helper(inducer) T	CA 72-4	0.136273408	6.39E-07
B18	B63	CD3+CD4+helper(inducer) T	WBRSR(mPas_200)	-0.134698729	8.61E-07
B18	B154	CD3+CD4+helper(inducer) T	RF	0.13415799	9.54E-07
B18	B64	CD3+CD4+helper(inducer) T	WBRSR(mPas_5)	-0.134116253	9.61E-07

B180	B193	Tf	K	0.443930114	0
B180	B195	Tf	Na	-0.25243383<br>2	0
B180	B194	Tf	Cl	-0.21708580<br>1	1.33E-15
B181	B184	APTT	INR	0.900337373	0
B181	B185	APTT	PT	0.875319302	0
B181	B186	APTT	PFC	0.246760217	0
B181	B182	APTT	TT	0.221999489	4.44E-16
B181	B183	APTT	PTA	0.143778825	1.47E-07
B182	B183	TT	PTA	-0.42793955<br>5	0
B182	B185	TT	PT	0.148607509	5.50E-08
B184	B185	INR	PT	0.985546294	0
B184	B186	INR	PFC	0.175931283	1.13E-10
B185	B186	PT	PFC	0.215268929	2.22E-15
B186	B200	PFC	OP	0.194446353	9.37E-13
B189	B190	eGFB	Cre	-0.64145363<br>7	0
B189	B191	eGFB	Urea	-0.36744917<br>1	0
B189	B192	eGFB	UA	-0.23132452	0
B189	B220	eGFB	SBP	-0.18407976<br>4	1.46E-11
B189	B216	eGFB	BMI	0.173584957	2.00E-10
B189	B221	eGFB	Hei	0.137424626	5.13E-07
B19	B101	CD3+CD4+/CD3+CD8+	anti-Sc70	0.230182118	0
B19	B102	CD3+CD4+/CD3+CD8+	anti-Sm	0.230182114	0
B19	B103	CD3+CD4+/CD3+CD8+	anti-SSA/Ro	0.230182115	0
B19	B104	CD3+CD4+/CD3+CD8+	anti-SSB	0.230182115	0
B19	B105	CD3+CD4+/CD3+CD8+	anti-U1RNP	0.230182116	0
B19	B106	CD3+CD4+/CD3+CD8+	anti-P	0.230182116	0
B19	B107	CD3+CD4+/CD3+CD8+	AnuA	0.230181985	0
B19	B109	CD3+CD4+/CD3+CD8+	AMA-M2	0.230182116	0
B19	B110	CD3+CD4+/CD3+CD8+	AHA	0.230182116	0
B19	B119	CD3+CD4+/CD3+CD8+	Hcv-rna	0.329072038	0
B19	B121	CD3+CD4+/CD3+CD8+	C3	0.496834695	0
B19	B122	CD3+CD4+/CD3+CD8+	C4	0.587279114	0
B19	B124	CD3+CD4+/CD3+CD8+	ACTH	-0.65826845<br>2	0
B19	B125	CD3+CD4+/CD3+CD8+	Cor	-0.85664296<br>5	0
B19	B136	CD3+CD4+/CD3+CD8+	5'NT	0.675576383	0
B19	B152	CD3+CD4+/CD3+CD8+	ACA-Ig(G-A-M)	-0.38314563	0

				9	
B19	B159	CD3+CD4+/CD3+CD8+	β2-MG	-0.383128394	0
B19	B169	CD3+CD4+/CD3+CD8+	U-CRY	-0.383130275	0
B19	B199	CD3+CD4+/CD3+CD8+	Mg	0.295285379	0
B19	B20	CD3+CD4+/CD3+CD8+	CD3+CD4+CD8+	-0.379384457	0
B19	B21	CD3+CD4+/CD3+CD8+	CD3+CD4-CD8-	-0.418540969	0
B19	B23	CD3+CD4+/CD3+CD8+	CD3-CD16+CD56+(NK)	0.34330761	0
B19	B79	CD3+CD4+/CD3+CD8+	hb V -dna	0.239125914	0
B19	B89	CD3+CD4+/CD3+CD8+	CA 72-4	0.570267255	0
B19	B94	CD3+CD4+/CD3+CD8+	ALDH2-Lys504Lys	0.280490472	0
B19	B97	CD3+CD4+/CD3+CD8+	dsDNA	0.230182116	0
B19	B99	CD3+CD4+/CD3+CD8+	anti-PM-Scl	0.230182117	0
B19	B120	CD3+CD4+/CD3+CD8+	HCV-cAg	0.193526896	1.20E-12
B19	B179	CD3+CD4+/CD3+CD8+	α1-MG	-0.192197471	1.72E-12
B19	B151	CD3+CD4+/CD3+CD8+	Anti-CCP	-0.18353291	1.68E-11
B19	B92	CD3+CD4+/CD3+CD8+	ALDH2-Glu504Glu	-0.168576982	6.63E-10
B19	B196	CD3+CD4+/CD3+CD8+	TCO2	-0.161404596	3.45E-09
B19	B186	CD3+CD4+/CD3+CD8+	PFC	-0.15738008	8.45E-09
B19	B93	CD3+CD4+/CD3+CD8+	ALDH2-Glu504Lys	0.153211914	2.08E-08
B19	B182	CD3+CD4+/CD3+CD8+	TT	0.141441176	2.34E-07
B19	B157	CD3+CD4+/CD3+CD8+	IgG	-0.140605168	2.76E-07
B19	B197	CD3+CD4+/CD3+CD8+	AG	0.140419453	2.87E-07
B19	B22	CD3+CD4+/CD3+CD8+	CD3+CD8+(T)	0.135714017	7.11E-07
B190	B191	Cre	Urea	0.384690868	0
B190	B192	Cre	UA	0.479894966	0
B190	B221	Cre	Hei	0.272993903	0
B190	B222	Cre	NS	0.243896826	0
B190	B216	Cre	BMI	0.170342618	4.37E-10
B191	B192	Urea	UA	0.17023	4.48E-10
B192	B206	UA	Waist	0.272153166	0
B192	B207	UA	WHR	0.231886386	0
B192	B216	UA	BMI	0.303084029	0
B192	B221	UA	Hei	0.235744855	0
B192	B222	UA	NS	0.366601292	0
B192	B215	UA	BMI	0.220440006	4.44E-16
B192	B210	UA	Hip	0.199503272	2.32E-13

B193	B200	K	OP	0.182490476	2.19E-11
B193	B194	K	Cl	-0.144274109	1.33E-07
B194	B195	Cl	Na	0.566103596	0
B194	B197	Cl	AG	-0.30582502	0
B194	B198	Cl	P	-0.240789746	0
B195	B197	Na	AG	0.332558875	0
B195	B200	Na	OP	0.56360873	0
B196	B197	TCO2	AG	-0.785808366	0
B196	B200	TCO2	OP	-0.31425964	0
B196	B199	TCO2	Mg	-0.213330931	4.22E-15
B197	B198	AG	P	0.23178507	0
B197	B200	AG	OP	0.533768634	0
B198	B200	P	OP	0.162728791	2.56E-09
B2	B126	Age	Alb	-0.334984772	0
B2	B127	Age	A-G ratio	-0.286644723	0
B2	B128	Age	ALT	-0.331923794	0
B2	B141	Age	AST/ALT	0.407976607	0
B2	B189	Age	eGFB	-0.510702551	0
B2	B191	Age	Urea	0.236359411	0
B2	B210	Age	Hip	-0.225075398	0
B2	B215	Age	BMI	-0.295703922	0
B2	B216	Age	BMI	-0.43890545	0
B2	B220	Age	SBP	0.340665275	0
B2	B221	Age	Hei	-0.363615019	0
B2	B222	Age	NS	-0.310235134	0
B2	B46	Age	RBC Count	-0.340852168	0
B2	B47	Age	RDW	0.234245076	0
B2	B51	Age	Hb	-0.363951127	0
B2	B72	Age	HDL-C	0.235957305	0
B2	B206	Age	Waist	-0.19270450	1.50E-12

				3	
B2	B41	Age	MON %	0.18709544	6.67E-12
B2	B130	Age	GLB	0.186201654	8.42E-12
B2	B71	Age	TG	-0.184210382	1.41E-11
B2	B146	Age	CysC	0.175049951	1.40E-10
B2	B209	Age	Smoking	-0.157663926	7.94E-09
B2	B190	Age	Cre	0.149435749	4.63E-08
B2	B176	Age	Ucr	-0.145040807	1.14E-07
B2	B188	Age	Ca	-0.137746405	4.82E-07
B2	B45	Age	MCHC	-0.137010904	5.55E-07
B20	B101	CD3+CD4+CD8+	anti-Sc70	0.249193824	0
B20	B102	CD3+CD4+CD8+	anti-Sm	0.249193819	0
B20	B103	CD3+CD4+CD8+	anti-SSA/Ro	0.24919382	0
B20	B104	CD3+CD4+CD8+	anti-SSB	0.24919382	0
B20	B105	CD3+CD4+CD8+	anti-U1RNP	0.249193821	0
B20	B106	CD3+CD4+CD8+	anti-P	0.249193821	0
B20	B107	CD3+CD4+CD8+	AnuA	0.249193683	0
B20	B109	CD3+CD4+CD8+	AMA-M2	0.24919382	0
B20	B110	CD3+CD4+CD8+	AHA	0.249193821	0
B20	B121	CD3+CD4+CD8+	C3	-0.252807789	0
B20	B122	CD3+CD4+CD8+	C4	-0.307357228	0
B20	B136	CD3+CD4+CD8+	5'NT	-0.282796289	0
B20	B151	CD3+CD4+CD8+	Anti-CCP	0.235966863	0
B20	B157	CD3+CD4+CD8+	IgG	0.26713563	0
B20	B186	CD3+CD4+CD8+	PFC	0.318087921	0
B20	B21	CD3+CD4+CD8+	CD3+CD4-CD8-	0.758675072	0
B20	B22	CD3+CD4+CD8+	CD3+CD8+(T)	0.508350419	0
B20	B23	CD3+CD4+CD8+	CD3-CD16+CD56+(NK)	-0.452174555	0
B20	B89	CD3+CD4+CD8+	CA 72-4	-0.331194383	0
B20	B97	CD3+CD4+CD8+	dsDNA	0.249193821	0
B20	B99	CD3+CD4+CD8+	anti-PM-Scl	0.249193821	0
B20	B125	CD3+CD4+CD8+	Cor	0.214786281	2.66E-15
B20	B193	CD3+CD4+CD8+	K	0.212907537	4.88E-15
B20	B79	CD3+CD4+CD8+	hb V -dna	0.177150152	8.38E-11

B20	B75	CD3+CD4+CD8+	ApoA1/B	0.17615186	1.07E-10
B20	B54	CD3+CD4+CD8+	ESR	-0.174007875	1.81E-10
B20	B73	CD3+CD4+CD8+	ApoA	-0.169366986	5.50E-10
B20	B74	CD3+CD4+CD8+	ApoA1	0.161336155	3.51E-09
B20	B76	CD3+CD4+CD8+	ApoB	-0.158444475	6.68E-09
B201	B204	e-habits	Drinking	0.200097169	1.96E-13
B204	B209	Drinking	Smoking	0.401420404	0
B204	B221	Drinking	Hei	0.147702396	6.63E-08
B204	B216	Drinking	BMI	0.14095501	2.58E-07
B204	B222	Drinking	NS	0.137894525	4.68E-07
B205	B222	KDA	NS	-0.160859935	3.90E-09
B206	B207	Waist	WHR	0.798286207	0
B206	B210	Waist	Hip	0.783662237	0
B206	B215	Waist	BMI	0.819821101	0
B206	B216	Waist	BMI	0.790002746	0
B206	B221	Waist	Hei	0.295925409	0
B206	B222	Waist	NS	0.70822204	0
B206	B218	Waist	DBP	0.158191545	7.07E-09
B207	B210	WHR	Hip	0.266091147	0
B207	B215	WHR	BMI	0.535151035	0
B207	B216	WHR	BMI	0.481267621	0
B207	B222	WHR	NS	0.494331646	0
B209	B221	Smoking	Hei	0.177079016	8.52E-11
B209	B222	Smoking	NS	0.171840913	3.05E-10
B209	B216	Smoking	BMI	0.138822628	3.92E-07
B21	B101	CD3+CD4-CD8-	anti-Sc70	0.324574798	0
B21	B102	CD3+CD4-CD8-	anti-Sm	0.32457479	0
B21	B103	CD3+CD4-CD8-	anti-SSA/Ro	0.324574792	0
B21	B104	CD3+CD4-CD8-	anti-SSB	0.324574792	0
B21	B105	CD3+CD4-CD8-	anti-U1RNP	0.324574792	0
B21	B106	CD3+CD4-CD8-	anti-P	0.324574793	0
B21	B107	CD3+CD4-CD8-	AnuA	0.32457461	0
B21	B109	CD3+CD4-CD8-	AMA-M2	0.324574792	0
B21	B110	CD3+CD4-CD8-	AHA	0.324574793	0
B21	B121	CD3+CD4-CD8-	C3	-0.318406879	0
B21	B122	CD3+CD4-CD8-	C4	-0.387098289	0
B21	B125	CD3+CD4-CD8-	Cor	0.270447618	0
B21	B136	CD3+CD4-CD8-	5'NT	-0.34638569	0

				5	
B21	B157	CD3+CD4-CD8-	IgG	0.368405118	0
B21	B22	CD3+CD4-CD8-	CD3+CD8+(T)	0.553800874	0
B21	B23	CD3+CD4-CD8-	CD3-CD16+CD56+(NK)	-0.51747391<br>5	0
B21	B73	CD3+CD4-CD8-	ApoA	-0.39452940<br>4	0
B21	B89	CD3+CD4-CD8-	CA 72-4	-0.41703482	0
B21	B97	CD3+CD4-CD8-	dsDNA	0.324574792	0
B21	B99	CD3+CD4-CD8-	anti-PM-Scl	0.324574794	0
B21	B186	CD3+CD4-CD8-	PFC	0.185171808	1.10E-11
B21	B81	CD3+CD4-CD8-	TSGF	-0.18360552<br>1	1.65E-11
B21	B196	CD3+CD4-CD8-	TCO2	0.176343476	1.02E-10
B21	B54	CD3+CD4-CD8-	ESR	-0.17296043	2.33E-10
B21	B197	CD3+CD4-CD8-	AG	-0.16951983	5.31E-10
B21	B74	CD3+CD4-CD8-	ApoA1	0.164466081	1.72E-09
B21	B124	CD3+CD4-CD8-	ACTH	0.147199325	7.35E-08
B21	B75	CD3+CD4-CD8-	ApoA1/B	0.142979903	1.73E-07
B21	B79	CD3+CD4-CD8-	hb V -dna	0.140832946	2.64E-07
B21	B151	CD3+CD4-CD8-	Anti-CCP	0.136779675	5.80E-07
B21	B137	CD3+CD4-CD8-	AFU	0.134138078	9.57E-07
B210	B215	Hip	BMI	0.765432686	0
B210	B216	Hip	BMI	0.770932701	0
B210	B221	Hip	Hei	0.340482436	0
B210	B222	Hip	NS	0.636506585	0
B210	B218	Hip	DBP	0.154599328	1.55E-08
B211	B212	IOP-R	IOP-L	0.786535748	0
B215	B216	BMI	BMI	0.802099165	0
B215	B222	BMI	NS	0.636771224	0
B215	B218	BMI	DBP	0.173106707	2.25E-10
B216	B221	BMI	Hei	0.667990193	0
B216	B222	BMI	NS	0.788403369	0
B216	B219	BMI	OT	0.135723325	7.10E-07
B218	B220	DBP	SBP	0.535262534	0
B219	B222	OT	NS	0.177721691	7.27E-11
B219	B221	OT	Hei	0.141068123	2.52E-07
B22	B121	CD3+CD8+(T)	C3	-0.38242633<br>4	0
B22	B122	CD3+CD8+(T)	C4	-0.39758783<br>9	0
B22	B124	CD3+CD8+(T)	ACTH	-0.49629944<br>3	0
B22	B125	CD3+CD8+(T)	Cor	-0.30750141	0

				5	
B22	B152	CD3+CD8+(T)	ACA-Ig(G-A-M)	-0.245034752	0
B22	B157	CD3+CD8+(T)	IgG	0.474532321	0
B22	B159	CD3+CD8+(T)	β2-MG	-0.245023737	0
B22	B169	CD3+CD8+(T)	U-CRY	-0.245024951	0
B22	B199	CD3+CD8+(T)	Mg	0.310413673	0
B22	B23	CD3+CD8+(T)	CD3-CD16+CD56+(NK)	-0.262402466	0
B22	B79	CD3+CD8+(T)	hb V -dna	0.296462758	0
B22	B89	CD3+CD8+(T)	CA 72-4	-0.284043715	0
B22	B94	CD3+CD8+(T)	ALDH2-Lys504Lys	0.252388323	0
B22	B155	CD3+CD8+(T)	IgA	0.215095706	2.44E-15
B22	B136	CD3+CD8+(T)	5'NT	-0.200350914	1.83E-13
B22	B101	CD3+CD8+(T)	anti-Sc70	0.194931514	8.20E-13
B22	B102	CD3+CD8+(T)	anti-Sm	0.194931511	8.20E-13
B22	B103	CD3+CD8+(T)	anti-SSA/Ro	0.194931511	8.20E-13
B22	B104	CD3+CD8+(T)	anti-SSB	0.194931511	8.20E-13
B22	B105	CD3+CD8+(T)	anti-U1RNP	0.194931511	8.20E-13
B22	B106	CD3+CD8+(T)	anti-P	0.194931512	8.20E-13
B22	B109	CD3+CD8+(T)	AMA-M2	0.194931511	8.20E-13
B22	B110	CD3+CD8+(T)	AHA	0.194931511	8.20E-13
B22	B97	CD3+CD8+(T)	dsDNA	0.194931511	8.20E-13
B22	B99	CD3+CD8+(T)	anti-PM-Scl	0.194931512	8.20E-13
B22	B107	CD3+CD8+(T)	AnuA	0.194931404	8.21E-13
B22	B179	CD3+CD8+(T)	α1-MG	-0.19491153	8.25E-13
B22	B182	CD3+CD8+(T)	TT	0.184467613	1.32E-11
B22	B73	CD3+CD8+(T)	ApoA	-0.171588044	3.24E-10
B22	B200	CD3+CD8+(T)	OP	-0.167480158	8.57E-10
B22	B181	CD3+CD8+(T)	APTT	0.162745278	2.55E-09
B22	B75	CD3+CD8+(T)	ApoA1/B	0.161063512	3.73E-09
B22	B158	CD3+CD8+(T)	IgM	0.158792954	6.19E-09
B22	B92	CD3+CD8+(T)	ALDH2-Glu504Glu	-0.154403842	1.61E-08
B22	B93	CD3+CD8+(T)	ALDH2-Glu504Lys	0.14053434	2.80E-07
B22	B156	CD3+CD8+(T)	IgE	0.139939941	3.15E-07
B22	B154	CD3+CD8+(T)	RF	0.137325544	5.23E-07
B22	B76	CD3+CD8+(T)	ApoB	-0.13560418	7.26E-07

B22	B62	CD3+CD8+(T)	WBHSRVI	-0.134635392	8.72E-07
B220	B221	SBP	Hei	-0.224251263	0
B221	B222	Hei	NS	0.525319784	0
B23	B119	CD3-CD16+CD56+(NK)	Hcv-rna	0.358803356	0
B23	B121	CD3-CD16+CD56+(NK)	C3	0.344805531	0
B23	B122	CD3-CD16+CD56+(NK)	C4	0.301131931	0
B23	B125	CD3-CD16+CD56+(NK)	Cor	-0.316549559	0
B23	B151	CD3-CD16+CD56+(NK)	Anti-CCP	-0.229577919	0
B23	B152	CD3-CD16+CD56+(NK)	ACA-Ig(G-A-M)	-0.289022693	0
B23	B157	CD3-CD16+CD56+(NK)	IgG	-0.251414282	0
B23	B159	CD3-CD16+CD56+(NK)	β2-MG	-0.289009752	0
B23	B169	CD3-CD16+CD56+(NK)	U-CRY	-0.289011179	0
B23	B186	CD3-CD16+CD56+(NK)	PFC	-0.286438094	0
B23	B196	CD3-CD16+CD56+(NK)	TCO2	-0.284498931	0
B23	B197	CD3-CD16+CD56+(NK)	AG	0.285499145	0
B23	B200	CD3-CD16+CD56+(NK)	OP	0.236855387	0
B23	B54	CD3-CD16+CD56+(NK)	ESR	0.259816148	0
B23	B195	CD3-CD16+CD56+(NK)	Na	0.203546966	7.39E-14
B23	B94	CD3-CD16+CD56+(NK)	ALDH2-Lys504Lys	0.201978503	1.15E-13
B23	B199	CD3-CD16+CD56+(NK)	Mg	0.198926974	2.72E-13
B23	B79	CD3-CD16+CD56+(NK)	hbⅤ-dna	0.175422845	1.28E-10
B23	B69	CD3-CD16+CD56+(NK)	PV	0.169058269	5.92E-10
B23	B28	CD3-CD16+CD56+(NK)	PRL	0.158702426	6.31E-09
B23	B29	CD3-CD16+CD56+(NK)	E2	0.158702426	6.31E-09
B23	B30	CD3-CD16+CD56+(NK)	LH	-0.158702426	6.31E-09
B23	B31	CD3-CD16+CD56+(NK)	FSH	-0.158702426	6.31E-09
B23	B32	CD3-CD16+CD56+(NK)	Testo	0.158702426	6.31E-09
B23	B33	CD3-CD16+CD56+(NK)	Prog	0.158702426	6.31E-09
B23	B120	CD3-CD16+CD56+(NK)	HCV-cAg	0.158486399	6.62E-09
B23	B124	CD3-CD16+CD56+(NK)	ACTH	-0.141328748	2.40E-07
B23	B81	CD3-CD16+CD56+(NK)	TSGF	0.137786302	4.78E-07

B23	B73	CD3-CD16+CD56+(NK)	ApoA	0.137607704	4.95E-07
B24	B26	αHBDH	CKMB	0.390747925	0
B24	B27	αHBDH	LDH	0.623000507	0
B24	B28	αHBDH	PRL	-0.251974201	0
B24	B29	αHBDH	E2	-0.251974202	0
B24	B30	αHBDH	LH	0.251974202	0
B24	B31	αHBDH	FSH	0.251974202	0
B24	B32	αHBDH	Testo	-0.251974202	0
B24	B33	αHBDH	Prog	-0.251974202	0
B24	B74	αHBDH	ApoA1	0.243060392	0
B24	B25	αHBDH	CK	0.214738303	2.66E-15
B24	B135	αHBDH	TBA	0.21261425	5.33E-15
B24	B75	αHBDH	ApoA1/B	0.171687618	3.17E-10
B24	B125	αHBDH	Cor	0.141013563	2.55E-07
B24	B62	αHBDH	WBHSRVI	-0.140729535	2.70E-07
B24	B78	αHBDH	HBcAb	-0.136555477	6.06E-07
B24	B124	αHBDH	ACTH	-0.136317378	6.34E-07
B25	B135	CK	TBA	0.250614296	0
B25	B26	CK	CKMB	0.251327875	0
B25	B27	CK	LDH	0.195073314	7.89E-13
B25	B76	CK	ApoB	-0.161412682	3.45E-09
B25	B197	CK	AG	-0.146751477	8.06E-08
B25	B69	CK	PV	-0.137794229	4.78E-07
B26	B27	CKMB	LDH	0.266355104	0
B26	B73	CKMB	ApoA	0.27797223	0
B26	B199	CKMB	Mg	-0.153232241	2.08E-08
B26	B79	CKMB	hb V -dna	-0.135367165	7.59E-07
B27	B131	LDH	AST	0.198504902	3.06E-13
B27	B135	LDH	TBA	0.177443072	7.79E-11
B27	B74	LDH	ApoA1	0.153743018	1.86E-08
B27	B176	LDH	Ucr	-0.151925228	2.74E-08

B27	B28	LDH	PRL	-0.136407857	6.23E-07
B27	B29	LDH	E2	-0.136407857	6.23E-07
B27	B30	LDH	LH	0.136407858	6.23E-07
B27	B31	LDH	FSH	0.136407857	6.23E-07
B27	B32	LDH	Testo	-0.136407857	6.23E-07
B27	B33	LDH	Prog	-0.136407857	6.23E-07
B28	B101	PRL	anti-Sc70	0.265945926	0
B28	B102	PRL	anti-Sm	0.265945924	0
B28	B103	PRL	anti-SSA/Ro	0.265945922	0
B28	B104	PRL	anti-SSB	0.265945925	0
B28	B105	PRL	anti-U1RNP	0.265945923	0
B28	B106	PRL	anti-P	0.265945925	0
B28	B107	PRL	AnuA	0.265945811	0
B28	B109	PRL	AMA-M2	0.265945924	0
B28	B110	PRL	AHA	0.265945924	0
B28	B119	PRL	Hcv-rna	0.350513941	0
B28	B124	PRL	ACTH	0.631222824	0
B28	B125	PRL	Cor	-0.466514235	0
B28	B138	PRL	PAB	0.289685363	0
B28	B29	PRL	E2	1	0
B28	B31	PRL	FSH	-0.999999999	0
B28	B32	PRL	Testo	1	0
B28	B33	PRL	Prog	1	0
B28	B79	PRL	hb V -dna	0.352651028	0
B28	B92	PRL	ALDH2-Glu504Glu	-0.246566971	0
B28	B93	PRL	ALDH2-Glu504Lys	0.224218726	0
B28	B94	PRL	ALDH2-Lys504Lys	0.407476705	0
B28	B97	PRL	dsDNA	0.265945925	0
B28	B99	PRL	anti-PM-Scl	0.265945924	0
B28	B155	PRL	IgA	-0.218378638	8.88E-16
B28	B157	PRL	IgG	-0.20448832	5.64E-14
B28	B148	PRL	Anti_TPO	-0.200940676	1.55E-13
B28	B120	PRL	HCV-cAg	0.198178951	3.36E-13
B28	B65	PRL	WBRSR(mPas_1)	0.194378927	9.54E-13
B28	B64	PRL	WBRSR(mPas_5)	0.186668404	7.45E-12

B28	B134	PRL	CHE	0.169931466	4.81E-10
B28	B135	PRL	TBA	-0.169570293	5.24E-10
B28	B60	PRL	WBRVI	0.168473569	6.79E-10
B28	B195	PRL	Na	-0.164280193	1.80E-09
B28	B194	PRL	Cl	-0.163368793	2.21E-09
B28	B186	PRL	PFC	-0.159007624	5.90E-09
B28	B67	PRL	WBRSR(mPas_50)	0.155085019	1.39E-08
B28	B200	PRL	OP	-0.151974337	2.71E-08
B28	B63	PRL	WBRSR(mPas_200)	0.151188295	3.20E-08
B28	B75	PRL	ApoA1/B	-0.14855435	5.56E-08
B28	B66	PRL	WBRSR(mPas_100)	0.148437946	5.69E-08
B28	B183	PRL	PTA	0.143832465	1.46E-07
B28	B58	PRL	HCT	0.139647193	3.33E-07
B28	B182	PRL	TT	-0.138565733	4.12E-07
B29	B101	E2	anti-Sc70	0.265945928	0
B29	B102	E2	anti-Sm	0.265945921	0
B29	B103	E2	anti-SSA/Ro	0.265945922	0
B29	B104	E2	anti-SSB	0.265945923	0
B29	B105	E2	anti-U1RNP	0.265945922	0
B29	B106	E2	anti-P	0.265945924	0
B29	B107	E2	AnuA	0.265945778	0
B29	B109	E2	AMA-M2	0.265945923	0
B29	B110	E2	AHA	0.265945923	0
B29	B119	E2	Hcv-rna	0.350513941	0
B29	B124	E2	ACTH	0.631222824	0
B29	B125	E2	Cor	-0.466514236	0
B29	B138	E2	PAB	0.289685363	0
B29	B31	E2	FSH	-1	0
B29	B32	E2	Testo	1	0
B29	B79	E2	hb V -dna	0.352651028	0
B29	B92	E2	ALDH2-Glu504Glu	-0.246566971	0
B29	B93	E2	ALDH2-Glu504Lys	0.224218726	0
B29	B94	E2	ALDH2-Lys504Lys	0.407476703	0
B29	B97	E2	dsDNA	0.265945922	0
B29	B99	E2	anti-PM-Scl	0.265945923	0
B29	B155	E2	IgA	-0.21837863	8.88E-16

				8	
B29	B157	E2	IgG	-0.20448832	5.64E-14
B29	B148	E2	Anti_TPO	-0.200940676	1.55E-13
B29	B120	E2	HCV-cAg	0.19817895	3.36E-13
B29	B65	E2	WBRSR(mPas_1)	0.194378927	9.54E-13
B29	B64	E2	WBRSR(mPas_5)	0.186668404	7.45E-12
B29	B134	E2	CHE	0.169931466	4.81E-10
B29	B135	E2	TBA	-0.169570293	5.24E-10
B29	B60	E2	WBRVI	0.168473569	6.79E-10
B29	B195	E2	Na	-0.164280193	1.80E-09
B29	B194	E2	Cl	-0.163368794	2.21E-09
B29	B186	E2	PFC	-0.159007624	5.90E-09
B29	B67	E2	WBRSR(mPas_50)	0.155085019	1.39E-08
B29	B200	E2	OP	-0.151974338	2.71E-08
B29	B63	E2	WBRSR(mPas_200)	0.151188295	3.20E-08
B29	B75	E2	ApoA1/B	-0.14855435	5.56E-08
B29	B66	E2	WBRSR(mPas_100)	0.148437946	5.69E-08
B29	B183	E2	PTA	0.143832465	1.46E-07
B29	B58	E2	HCT	0.139647193	3.33E-07
B29	B182	E2	TT	-0.138565733	4.12E-07
B3	B84	Hcy	C-PSA	0.182945463	1.95E-11
B3	B14	Hcy	G-17	0.145268058	1.09E-07
B30	B101	LH	anti-Sc70	-0.26594593	0
B30	B102	LH	anti-Sm	-0.26594592	0
B30	B103	LH	anti-SSA/Ro	-0.265945924	0
B30	B104	LH	anti-SSB	-0.265945924	0
B30	B105	LH	anti-U1RNP	-0.265945926	0
B30	B106	LH	anti-P	-0.265945933	0
B30	B107	LH	AnuA	-0.265945718	0
B30	B109	LH	AMA-M2	-0.265945925	0
B30	B110	LH	AHA	-0.26594592	0

				6	
B30	B119	LH	Hcv-rna	-0.350513941	0
B30	B124	LH	ACTH	-0.631222826	0
B30	B125	LH	Cor	0.466514237	0
B30	B138	LH	PAB	-0.289685363	0
B30	B79	LH	hb V -dna	-0.352651029	0
B30	B92	LH	ALDH2-Glu504Glu	0.246566972	0
B30	B93	LH	ALDH2-Glu504Lys	-0.224218726	0
B30	B94	LH	ALDH2-Lys504Lys	-0.407476706	0
B30	B97	LH	dsDNA	-0.265945927	0
B30	B99	LH	anti-PM-Scl	-0.265945929	0
B30	B155	LH	IgA	0.218378639	8.88E-16
B30	B157	LH	IgG	0.20448832	5.64E-14
B30	B148	LH	Anti_TPO	0.200940677	1.55E-13
B30	B120	LH	HCV-cAg	-0.198178933	3.36E-13
B30	B65	LH	WBRSR(mPas_1)	-0.194378928	9.54E-13
B30	B64	LH	WBRSR(mPas_5)	-0.186668404	7.45E-12
B30	B134	LH	CHE	-0.169931467	4.81E-10
B30	B135	LH	TBA	0.169570294	5.24E-10
B30	B60	LH	WBRVI	-0.168473569	6.79E-10
B30	B195	LH	Na	0.164280192	1.80E-09
B30	B194	LH	Cl	0.163368794	2.21E-09
B30	B186	LH	PFC	0.159007625	5.90E-09
B30	B67	LH	WBRSR(mPas_50)	-0.155085019	1.39E-08
B30	B200	LH	OP	0.151974335	2.71E-08
B30	B63	LH	WBRSR(mPas_200)	-0.151188296	3.20E-08
B30	B75	LH	ApoA1/B	0.14855435	5.56E-08
B30	B66	LH	WBRSR(mPas_100)	-0.148437946	5.69E-08

B30	B183	LH	PTA	-0.143832465	1.46E-07
B30	B58	LH	HCT	-0.139647193	3.33E-07
B30	B182	LH	TT	0.138565733	4.12E-07
B31	B101	FSH	anti-Sc70	-0.265945926	0
B31	B102	FSH	anti-Sm	-0.265945917	0
B31	B103	FSH	anti-SSA/Ro	-0.265945922	0
B31	B104	FSH	anti-SSB	-0.265945922	0
B31	B105	FSH	anti-U1RNP	-0.265945922	0
B31	B106	FSH	anti-P	-0.265945921	0
B31	B107	FSH	AnuA	-0.26594575	0
B31	B109	FSH	AMA-M2	-0.26594592	0
B31	B110	FSH	AHA	-0.265945922	0
B31	B119	FSH	Hcv-rna	-0.35051394	0
B31	B124	FSH	ACTH	-0.631222824	0
B31	B125	FSH	Cor	0.466514237	0
B31	B138	FSH	PAB	-0.289685363	0
B31	B32	FSH	Testo	-0.999999999	0
B31	B33	FSH	Prog	-1	0
B31	B79	FSH	hb V -dna	-0.352651028	0
B31	B92	FSH	ALDH2-Glu504Glu	0.246566972	0
B31	B93	FSH	ALDH2-Glu504Lys	-0.224218726	0
B31	B94	FSH	ALDH2-Lys504Lys	-0.407476701	0
B31	B97	FSH	dsDNA	-0.265945921	0
B31	B99	FSH	anti-PM-Scl	-0.265945924	0
B31	B155	FSH	IgA	0.218378638	8.88E-16
B31	B157	FSH	IgG	0.20448832	5.64E-14
B31	B148	FSH	Anti_TPO	0.200940676	1.55E-13

B31	B120	FSH	HCV-cAg	-0.198178924	3.36E-13
B31	B65	FSH	WBRSR(mPas_1)	-0.194378927	9.54E-13
B31	B64	FSH	WBRSR(mPas_5)	-0.186668404	7.45E-12
B31	B134	FSH	CHE	-0.169931466	4.81E-10
B31	B135	FSH	TBA	0.169570293	5.24E-10
B31	B60	FSH	WBRVI	-0.168473568	6.79E-10
B31	B195	FSH	Na	0.164280193	1.80E-09
B31	B194	FSH	Cl	0.163368795	2.21E-09
B31	B186	FSH	PFC	0.159007625	5.90E-09
B31	B67	FSH	WBRSR(mPas_50)	-0.155085019	1.39E-08
B31	B200	FSH	OP	0.151974338	2.71E-08
B31	B63	FSH	WBRSR(mPas_200)	-0.151188295	3.20E-08
B31	B75	FSH	ApoA1/B	0.14855435	5.56E-08
B31	B66	FSH	WBRSR(mPas_100)	-0.148437946	5.69E-08
B31	B183	FSH	PTA	-0.143832465	1.46E-07
B31	B58	FSH	HCT	-0.139647193	3.33E-07
B31	B182	FSH	TT	0.138565733	4.12E-07
B32	B101	Testo	anti-Sc70	0.265945925	0
B32	B102	Testo	anti-Sm	0.265945922	0
B32	B103	Testo	anti-SSA/Ro	0.265945922	0
B32	B104	Testo	anti-SSB	0.265945921	0
B32	B105	Testo	anti-U1RNP	0.265945922	0
B32	B106	Testo	anti-P	0.265945924	0
B32	B107	Testo	AnuA	0.265945781	0
B32	B109	Testo	AMA-M2	0.265945922	0
B32	B110	Testo	AHA	0.265945922	0
B32	B119	Testo	Hcv-rna	0.350513941	0
B32	B124	Testo	ACTH	0.631222824	0
B32	B125	Testo	Cor	-0.466514236	0
B32	B138	Testo	PAB	0.289685363	0
B32	B33	Testo	Prog	1	0
B32	B79	Testo	hb V -dna	0.352651028	0
B32	B92	Testo	ALDH2-Glu504Glu	-0.24656697	0

				1	
B32	B93	Testo	ALDH2-Glu504Lys	0.224218726	0
B32	B94	Testo	ALDH2-Lys504Lys	0.407476701	0
B32	B97	Testo	dsDNA	0.265945922	0
B32	B99	Testo	anti-PM-Scl	0.265945924	0
B32	B155	Testo	IgA	-0.218378638	8.88E-16
B32	B157	Testo	IgG	-0.20448832	5.64E-14
B32	B148	Testo	Anti_TPO	-0.200940676	1.55E-13
B32	B120	Testo	HCV-cAg	0.198178942	3.36E-13
B32	B65	Testo	WBRSR(mPas_1)	0.194378927	9.54E-13
B32	B64	Testo	WBRSR(mPas_5)	0.186668404	7.45E-12
B32	B134	Testo	CHE	0.169931466	4.81E-10
B32	B135	Testo	TBA	-0.169570293	5.24E-10
B32	B60	Testo	WBRVI	0.168473568	6.79E-10
B32	B195	Testo	Na	-0.164280194	1.80E-09
B32	B194	Testo	Cl	-0.163368794	2.21E-09
B32	B186	Testo	PFC	-0.159007624	5.90E-09
B32	B67	Testo	WBRSR(mPas_50)	0.155085019	1.39E-08
B32	B200	Testo	OP	-0.151974338	2.71E-08
B32	B63	Testo	WBRSR(mPas_200)	0.151188295	3.20E-08
B32	B75	Testo	ApoA1/B	-0.14855435	5.56E-08
B32	B66	Testo	WBRSR(mPas_100)	0.148437946	5.69E-08
B32	B183	Testo	PTA	0.143832465	1.46E-07
B32	B58	Testo	HCT	0.139647193	3.33E-07
B32	B182	Testo	TT	-0.138565733	4.12E-07
B33	B101	Prog	anti-Sc70	0.265945927	0
B33	B102	Prog	anti-Sm	0.265945921	0
B33	B103	Prog	anti-SSA/Ro	0.265945922	0
B33	B104	Prog	anti-SSB	0.265945921	0
B33	B105	Prog	anti-U1RNP	0.265945922	0
B33	B106	Prog	anti-P	0.265945924	0
B33	B107	Prog	AnuA	0.265945767	0
B33	B109	Prog	AMA-M2	0.265945922	0
B33	B110	Prog	AHA	0.265945923	0
B33	B119	Prog	Hcv-rna	0.350513941	0
B33	B124	Prog	ACTH	0.631222824	0

B33	B125	Prog	Cor	-0.466514236	0
B33	B138	Prog	PAB	0.289685363	0
B33	B79	Prog	hb V -dna	0.352651028	0
B33	B92	Prog	ALDH2-Glu504Glu	-0.246566971	0
B33	B93	Prog	ALDH2-Glu504Lys	0.224218726	0
B33	B94	Prog	ALDH2-Lys504Lys	0.407476702	0
B33	B97	Prog	dsDNA	0.265945922	0
B33	B99	Prog	anti-PM-Scl	0.265945923	0
B33	B155	Prog	IgA	-0.218378638	8.88E-16
B33	B157	Prog	IgG	-0.20448832	5.64E-14
B33	B148	Prog	Anti_TPO	-0.200940677	1.55E-13
B33	B120	Prog	HCV-cAg	0.198178946	3.36E-13
B33	B65	Prog	WBRSR(mPas_1)	0.194378927	9.54E-13
B33	B64	Prog	WBRSR(mPas_5)	0.186668404	7.45E-12
B33	B134	Prog	CHE	0.169931466	4.81E-10
B33	B135	Prog	TBA	-0.169570293	5.24E-10
B33	B60	Prog	WBRVI	0.168473568	6.79E-10
B33	B195	Prog	Na	-0.164280193	1.80E-09
B33	B194	Prog	Cl	-0.163368795	2.21E-09
B33	B186	Prog	PFC	-0.159007624	5.90E-09
B33	B67	Prog	WBRSR(mPas_50)	0.155085019	1.39E-08
B33	B200	Prog	OP	-0.151974338	2.71E-08
B33	B63	Prog	WBRSR(mPas_200)	0.151188295	3.20E-08
B33	B75	Prog	ApoA1/B	-0.14855435	5.56E-08
B33	B66	Prog	WBRSR(mPas_100)	0.148437946	5.69E-08
B33	B183	Prog	PTA	0.143832465	1.46E-07
B33	B58	Prog	HCT	0.139647193	3.33E-07
B33	B182	Prog	TT	-0.138565733	4.12E-07
B35	B116	β2-MG	TT3	0.516508183	0
B35	B117	β2-MG	TT4	0.737331664	0
B35	B167	β2-MG	PC	0.863642472	0
B35	B202	β2-MG	e-times	0.873730964	0
B36	B223	V-Lym%	FH	0.569548914	0
B36	B38	V-Lym%	BAND%	-0.80450903	0

				9	
B37	B202	BAND	e-times	0.374516878	0
B38	B96	BAND%	dsDNA (IIF)	-0.491400677	0
B4	B121	WB-Ca	C3	0.57491285	0
B4	B122	WB-Ca	C4	0.698091611	0
B4	B124	WB-Ca	ACTH	-0.264549505	0
B4	B125	WB-Ca	Cor	-0.483483118	0
B4	B136	WB-Ca	5'NT	0.805945093	0
B4	B157	WB-Ca	IgG	-0.272858004	0
B4	B19	WB-Ca	CD3+CD4+/CD3+CD8+	0.701348555	0
B4	B20	WB-Ca	CD3+CD4+CD8+	-0.410751519	0
B4	B21	WB-Ca	CD3+CD4-CD8-	-0.517270745	0
B4	B22	WB-Ca	CD3+CD8+(T)	-0.332756241	0
B4	B5	WB-Ca	WB-Mg	0.43691822	0
B4	B6	WB-Ca	WB-Fe	0.300260988	0
B4	B7	WB-Ca	WB-Cop	-0.93813274	0
B4	B8	WB-Ca	WB-Zinc	0.324479811	0
B4	B89	WB-Ca	CA 72-4	0.746362142	0
B4	B18	WB-Ca	CD3+CD4+helper(inducer) T	0.190045546	3.06E-12
B4	B73	WB-Ca	ApoA	0.142622581	1.85E-07
B40	B42	WBC	MON	0.634209449	0
B40	B46	WBC	RBC Count	0.262408922	0
B40	B51	WBC	Hb	0.249246219	0
B40	B53	WBC	GR%	0.243822057	0
B40	B72	WBC	HDL-C	-0.216455575	1.78E-15
B40	B48	WBC	LYM%	-0.201664445	1.26E-13
B40	B222	WBC	NS	0.194258527	9.86E-13
B40	B41	WBC	MON %	-0.190171904	2.96E-12
B40	B52	WBC	PLT/BPC	0.179742947	4.39E-11
B40	B206	WBC	Waist	0.172756153	2.45E-10
B40	B216	WBC	BMI	0.170661309	4.05E-10
B40	B71	WBC	TG	0.165220675	1.45E-09
B40	B215	WBC	BMI	0.151523237	2.98E-08

B40	B207	WBC	WHR	0.147561507	6.82E-08
B40	B170	WBC	U-GLU	0.13783094	4.74E-07
B40	B209	WBC	Smoking	0.136849266	5.73E-07
B41	B42	MON％	MON	0.603700894	0
B41	B53	MON％	GR%	-0.245574375	0
B41	B126	MON％	Alb	-0.19133006	2.17E-12
B41	B49	MON％	baso%	0.188256349	4.91E-12
B41	B70	MON％	LDL-C	-0.170680033	4.03E-10
B41	B77	MON％	TC	-0.159036044	5.86E-09
B41	B190	MON％	Cre	0.15833692	6.84E-09
B41	B71	MON％	TG	-0.157429315	8.36E-09
B41	B189	MON％	eGFB	-0.153499782	1.96E-08
B41	B47	MON％	RDW	0.152940176	2.21E-08
B41	B46	MON％	RBC Count	-0.148086955	6.12E-08
B41	B43	MON％	MCV	0.137386164	5.17E-07
B41	B141	MON％	AST/ALT	0.134252357	9.37E-07
B42	B190	MON	Cre	0.170791244	3.92E-10
B42	B222	MON	NS	0.168697384	6.44E-10
B42	B192	MON	UA	0.162230657	2.87E-09
B42	B48	MON	LYM%	-0.157550357	8.14E-09
B42	B163	MON	SG	0.142397807	1.94E-07
B42	B72	MON	HDL-C	-0.138360609	4.28E-07
B43	B44	MCV	MCH	0.91190825	0
B43	B46	MCV	RBC Count	-0.6037356	0
B43	B47	MCV	RDW	0.598577924	0
B43	B52	MCV	PLT/BPC	-0.193747024	1.13E-12
B43	B216	MCV	BMI	-0.150072225	4.05E-08
B43	B215	MCV	BMI	-0.142001183	2.10E-07
B43	B210	MCV	Hip	-0.136861311	5.71E-07
B44	B45	MCH	MCHC	0.507600478	0
B44	B46	MCH	RBC Count	-0.513487569	0

B44	B47	MCH	RDW	0.431554274	0
B44	B51	MCH	Hb	0.186628536	7.53E-12
B44	B52	MCH	PLT/BPC	-0.179119198	5.13E-11
B44	B132	MCH	Dbil	0.165513162	1.35E-09
B44	B133	MCH	TBIL	0.141031208	2.54E-07
B45	B216	MCHC	BMI	0.258649692	0
B45	B221	MCHC	Hei	0.271294338	0
B45	B222	MCHC	NS	0.237094467	0
B45	B51	MCHC	Hb	0.427531085	0
B45	B47	MCHC	RDW	-0.211573217	7.11E-15
B45	B129	MCHC	IBIL	0.170950743	3.78E-10
B45	B133	MCHC	TBIL	0.1686529	6.51E-10
B45	B141	MCHC	AST/ALT	-0.16803522	7.53E-10
B45	B206	MCHC	Waist	0.163800488	2.01E-09
B45	B210	MCHC	Hip	0.160627549	4.11E-09
B45	B127	MCHC	A-G ratio	0.157398751	8.41E-09
B45	B174	MCHC	Urea/Cr	-0.151117499	3.25E-08
B45	B192	MCHC	UA	0.151021164	3.32E-08
B45	B189	MCHC	eGFB	0.145495085	1.04E-07
B45	B215	MCHC	BMI	0.138447113	4.21E-07
B46	B126	RBC Count	Alb	0.28099964	0
B46	B141	RBC Count	AST/ALT	-0.317598578	0
B46	B206	RBC Count	Waist	0.29708114	0
B46	B210	RBC Count	Hip	0.261828379	0
B46	B215	RBC Count	BMI	0.273541391	0
B46	B216	RBC Count	BMI	0.398383565	0
B46	B221	RBC Count	Hei	0.332222038	0
B46	B222	RBC Count	NS	0.372277133	0
B46	B47	RBC Count	RDW	-0.466595773	0
B46	B51	RBC Count	Hb	0.737672818	0
B46	B72	RBC Count	HDL-C	-0.259428932	0
B46	B207	RBC Count	WHR	0.211430698	7.55E-15
B46	B189	RBC Count	eGFB	0.20951006	1.31E-14
B46	B128	RBC Count	ALT	0.202473716	1.00E-13
B46	B174	RBC Count	Urea/Cr	-0.188143521	5.06E-12
B46	B58	RBC Count	HCT	0.154386721	1.62E-08
B46	B127	RBC Count	A-G ratio	0.151760373	2.84E-08

B46	B218	RBC Count	DBP	0.149608975	4.46E-08
B47	B216	RDW	BMI	-0.23402407 4	0
B47	B51	RDW	Hb	-0.21491897 7	2.66E-15
B47	B210	RDW	Hip	-0.21291659 7	4.88E-15
B47	B126	RDW	Alb	-0.20919585 9	1.44E-14
B47	B141	RDW	AST/ALT	0.20630867	3.33E-14
B47	B189	RDW	eGFB	-0.19996244 7	2.04E-13
B47	B215	RDW	BMI	-0.19686544 5	4.83E-13
B47	B72	RDW	HDL-C	0.164829331	1.58E-09
B47	B222	RDW	NS	-0.15584888	1.18E-08
B47	B52	RDW	PLT/BPC	-0.15569073 2	1.22E-08
B47	B206	RDW	Waist	-0.14895178 5	5.12E-08
B47	B221	RDW	Hei	-0.14743246	7.01E-08
B48	B53	LYM%	GR%	-0.92947878 2	0
B49	B50	baso%	eo%	0.324882463	0
B49	B53	baso%	GR%	-0.1720913	2.87E-10
B5	B136	WB-Mg	5'NT	0.254649603	0
B5	B6	WB-Mg	WB-Fe	0.716625456	0
B5	B7	WB-Mg	WB-Cop	-0.68566311	0
B5	B89	WB-Mg	CA 72-4	0.236421604	0
B5	B122	WB-Mg	C4	0.223591072	2.22E-16
B5	B19	WB-Mg	CD3+CD4+/CD3+CD8+	0.222940104	2.22E-16
B5	B8	WB-Mg	WB-Zinc	0.188066815	5.17E-12
B5	B121	WB-Mg	C3	0.184459868	1.32E-11
B5	B21	WB-Mg	CD3+CD4-CD8-	-0.16702063 1	9.54E-10
B5	B224	WB-Mg	pmh	-0.16653491	1.07E-09
B5	B125	WB-Mg	Cor	-0.15290325 9	2.23E-08
B50	B53	eo%	GR%	-0.21824506 4	8.88E-16
B51	B126	Hb	Alb	0.321826951	0
B51	B128	Hb	ALT	0.230023707	0
B51	B141	Hb	AST/ALT	-0.34955777 6	0

B51	B174	Hb	Urea/Cr	-0.249715498	0
B51	B189	Hb	eGFB	0.225808539	0
B51	B206	Hb	Waist	0.314155964	0
B51	B207	Hb	WHR	0.231631883	0
B51	B210	Hb	Hip	0.271731413	0
B51	B215	Hb	BMI	0.277033953	0
B51	B216	Hb	BMI	0.448746236	0
B51	B221	Hb	Hei	0.423147517	0
B51	B222	Hb	NS	0.438021785	0
B51	B72	Hb	HDL-C	-0.283097623	0
B51	B127	Hb	A-G ratio	0.219552734	6.66E-16
B51	B129	Hb	IBIL	0.216837321	1.33E-15
B51	B58	Hb	HCT	0.210507577	9.77E-15
B51	B133	Hb	TBIL	0.204100965	6.31E-14
B51	B163	Hb	SG	0.189884253	3.20E-12
B51	B62	Hb	WBHSRVI	0.182955372	1.94E-11
B51	B67	Hb	WBRSR(mPas_50)	0.163684857	2.06E-09
B51	B66	Hb	WBRSR(mPas_100)	0.163541638	2.13E-09
B51	B192	Hb	UA	0.16302205	2.40E-09
B51	B60	Hb	WBRVI	0.161235749	3.59E-09
B51	B63	Hb	WBRSR(mPas_200)	0.160288994	4.44E-09
B51	B205	Hb	KDA	-0.156722573	9.76E-09
B51	B64	Hb	WBRSR(mPas_5)	0.153294916	2.05E-08
B51	B209	Hb	Smoking	0.1486724	5.42E-08
B51	B191	Hb	Urea	-0.14747851	6.94E-08
B51	B218	Hb	DBP	0.139312155	3.56E-07
B52	B132	PLT/BPC	Dbil	-0.178108736	6.60E-11
B54	B123	ESR	hs-CRP1	0.247127303	0
B54	B58	ESR	HCT	-0.374796276	0
B54	B59	ESR	WBRV	0.237010774	0
B54	B60	ESR	WBRVI	-0.263611968	0
B54	B62	ESR	WBHSRVI	-0.307051595	0
B54	B69	ESR	PV	0.638075941	0
B54	B74	ESR	ApoA1	0.220513148	4.44E-16
B54	B56	ESR	IR	-0.201497268	1.32E-13
B54	B73	ESR	ApoA	0.188117499	5.10E-12

B54	B158	ESR	IgM	0.166083328	1.19E-09
B54	B76	ESR	ApoB	0.160177282	4.55E-09
B54	B200	ESR	OP	0.139435769	3.48E-07
B54	B195	ESR	Na	0.135883271	6.88E-07
B55	B56	TK	IR	0.761707435	0
B55	B57	TK	RBCAI	-0.25307444 4	0
B55	B58	TK	HCT	-0.35011458	0
B55	B59	TK	WBRV	0.53418906	0
B55	B61	TK	WBHSRV	0.794237532	0
B55	B62	TK	WBHSRVI	0.383469533	0
B55	B63	TK	WBRSR(mPas_200)	0.422002715	0
B55	B64	TK	WBRSR(mPas_5)	0.317045998	0
B55	B66	TK	WBRSR(mPas_100)	0.419644574	0
B55	B67	TK	WBRSR(mPas_50)	0.413659435	0
B55	B60	TK	WBRVI	0.217166833	1.33E-15
B55	B65	TK	WBRSR(mPas_1)	0.214618741	2.89E-15
B55	B108	TK	Anti-Cyclin	-0.17829343 7	6.31E-11
B55	B98	TK	anti-Jo-1	-0.16487048 5	1.57E-09
B55	B101	TK	anti-Sc70	-0.13433768 6	9.22E-07
B55	B102	TK	anti-Sm	-0.13433768 1	9.22E-07
B55	B103	TK	anti-SSA/Ro	-0.13433768 1	9.22E-07
B55	B104	TK	anti-SSB	-0.13433768 1	9.22E-07
B55	B105	TK	anti-U1RNP	-0.13433768 2	9.22E-07
B55	B106	TK	anti-P	-0.13433768 3	9.22E-07
B55	B107	TK	AnuA	-0.13433759 5	9.22E-07
B55	B109	TK	AMA-M2	-0.13433768 1	9.22E-07
B55	B110	TK	AHA	-0.13433768 2	9.22E-07
B55	B97	TK	dsDNA	-0.13433768 3	9.22E-07
B55	B99	TK	anti-PM-Scl	-0.13433768 3	9.22E-07
B56	B57	IR	RBCAI	-0.34469284	0

				9	
B56	B58	IR	HCT	0.307733243	0
B56	B59	IR	WBRV	0.409847393	0
B56	B60	IR	WBRVI	0.653547886	0
B56	B61	IR	WBHSRV	0.937511417	0
B56	B62	IR	WBHSRVI	0.886568951	0
B56	B63	IR	WBRSR(mPas_200)	0.859402842	0
B56	B64	IR	WBRSR(mPas_5)	0.72202142	0
B56	B65	IR	WBRSR(mPas_1)	0.556585838	0
B56	B66	IR	WBRSR(mPas_100)	0.855006462	0
B56	B67	IR	WBRSR(mPas_50)	0.847969677	0
B56	B69	IR	PV	-0.272716943	0
B56	B124	IR	ACTH	0.163481782	2.16E-09
B56	B108	IR	Anti-Cyclin	-0.158986742	5.93E-09
B56	B98	IR	anti-Jo-1	-0.148968929	5.10E-08
B57	B108	RBCAI	Anti-Cyclin	0.414159852	0
B57	B59	RBCAI	WBRV	0.563628746	0
B57	B60	RBCAI	WBRVI	0.330543637	0
B57	B61	RBCAI	WBHSRV	-0.296357109	0
B57	B62	RBCAI	WBHSRVI	-0.322111796	0
B57	B63	RBCAI	WBRSR(mPas_200)	-0.296003691	0
B57	B65	RBCAI	WBRSR(mPas_1)	0.428713544	0
B57	B66	RBCAI	WBRSR(mPas_100)	-0.24314403	0
B57	B98	RBCAI	anti-Jo-1	0.415567104	0
B57	B67	RBCAI	WBRSR(mPas_50)	-0.194834708	8.42E-13
B57	B156	RBCAI	IgE	0.187557566	5.91E-12
B57	B69	RBCAI	PV	0.178722986	5.67E-11
B57	B64	RBCAI	WBRSR(mPas_5)	0.155777824	1.20E-08
B57	B58	RBCAI	HCT	-0.145769748	9.84E-08
B57	B151	RBCAI	Anti-CCP	0.139862111	3.20E-07
B58	B59	HCT	WBRV	-0.226886514	0
B58	B60	HCT	WBRVI	0.615173877	0
B58	B62	HCT	WBHSRVI	0.698521836	0
B58	B63	HCT	WBRSR(mPas_200)	0.603037355	0
B58	B64	HCT	WBRSR(mPas_5)	0.555311328	0

B58	B65	HCT	WBRSR(mPas_1)	0.470438242	0
B58	B66	HCT	WBRSR(mPas_100)	0.592623216	0
B58	B67	HCT	WBRSR(mPas_50)	0.591700969	0
B58	B69	HCT	PV	-0.354748855	0
B58	B124	HCT	ACTH	0.184977871	1.16E-11
B58	B61	HCT	WBHSRV	0.163979844	1.93E-09
B58	B74	HCT	ApoA1	-0.146587323	8.33E-08
B59	B108	WBRV	Anti-Cyclin	0.292039538	0
B59	B60	WBRV	WBRVI	0.54531672	0
B59	B61	WBRV	WBHSRV	0.571476498	0
B59	B63	WBRV	WBRSR(mPas_200)	0.367529507	0
B59	B64	WBRV	WBRSR(mPas_5)	0.641857016	0
B59	B65	WBRV	WBRSR(mPas_1)	0.745722597	0
B59	B66	WBRV	WBRSR(mPas_100)	0.410180709	0
B59	B67	WBRV	WBRSR(mPas_50)	0.443150139	0
B59	B69	WBRV	PV	0.398961769	0
B59	B98	WBRV	anti-Jo-1	0.301166569	0
B59	B62	WBRV	WBHSRVI	0.188248835	4.92E-12
B59	B151	WBRV	Anti-CCP	0.181822004	2.60E-11
B59	B101	WBRV	anti-Sc70	-0.181600759	2.75E-11
B59	B102	WBRV	anti-Sm	-0.181600753	2.75E-11
B59	B103	WBRV	anti-SSA/Ro	-0.181600755	2.75E-11
B59	B104	WBRV	anti-SSB	-0.181600755	2.75E-11
B59	B105	WBRV	anti-U1RNP	-0.181600756	2.75E-11
B59	B106	WBRV	anti-P	-0.181600755	2.75E-11
B59	B107	WBRV	AnuA	-0.181600644	2.75E-11
B59	B109	WBRV	AMA-M2	-0.181600755	2.75E-11
B59	B110	WBRV	AHA	-0.181600756	2.75E-11
B59	B97	WBRV	dsDNA	-0.181600756	2.75E-11
B59	B99	WBRV	anti-PM-Scl	-0.181600757	2.75E-11
B59	B74	WBRV	ApoA1	0.137628885	4.93E-07

B6	B7	WB-Fe	WB-Cop	-0.36897988 2	0
B6	B8	WB-Fe	WB-Zinc	0.789531561	0
B6	B224	WB-Fe	pmh	-0.20366862 2	7.15E-14
B60	B124	WBRVI	ACTH	0.232576514	0
B60	B61	WBRVI	WBHSRV	0.55960072	0
B60	B62	WBRVI	WBHSRVI	0.781407997	0
B60	B63	WBRVI	WBRSR(mPas_200)	0.717530896	0
B60	B64	WBRVI	WBRSR(mPas_5)	0.892227104	0
B60	B65	WBRVI	WBRSR(mPas_1)	0.909630533	0
B60	B66	WBRVI	WBRSR(mPas_100)	0.747131233	0
B60	B67	WBRVI	WBRSR(mPas_50)	0.77419617	0
B60	B69	WBRVI	PV	-0.27926956 2	0
B61	B62	WBHSRV	WBHSRVI	0.763421612	0
B61	B63	WBHSRV	WBRSR(mPas_200)	0.872982607	0
B61	B64	WBHSRV	WBRSR(mPas_5)	0.764229341	0
B61	B65	WBHSRV	WBRSR(mPas_1)	0.610094759	0
B61	B66	WBHSRV	WBRSR(mPas_100)	0.873599978	0
B61	B67	WBHSRV	WBRSR(mPas_50)	0.868262785	0
B61	B124	WBHSRV	ACTH	0.137632547	4.93E-07
B62	B63	WBHSRVI	WBRSR(mPas_200)	0.920035429	0
B62	B64	WBHSRVI	WBRSR(mPas_5)	0.795649183	0
B62	B65	WBHSRVI	WBRSR(mPas_1)	0.63114906	0
B62	B66	WBHSRVI	WBRSR(mPas_100)	0.915977078	0
B62	B67	WBHSRVI	WBRSR(mPas_50)	0.910212771	0
B62	B69	WBHSRVI	PV	-0.38197929	0
B62	B124	WBHSRVI	ACTH	0.211764767	6.66E-15
B62	B89	WBHSRVI	CA 72-4	0.13866979	4.03E-07
B62	B216	WBHSRVI	BMI	0.137961155	4.63E-07
B62	B74	WBHSRVI	ApoA1	-0.13727221 1	5.28E-07
B63	B64	WBRSR(mPas_200)	WBRSR(mPas_5)	0.894228477	0
B63	B65	WBRSR(mPas_200)	WBRSR(mPas_1)	0.730646783	0
B63	B66	WBRSR(mPas_200)	WBRSR(mPas_100)	0.995694386	0
B63	B67	WBRSR(mPas_200)	WBRSR(mPas_50)	0.990528331	0
B63	B124	WBRSR(mPas_200)	ACTH	0.195205331	7.61E-13
B64	B65	WBRSR(mPas_5)	WBRSR(mPas_1)	0.95824965	0
B64	B66	WBRSR(mPas_5)	WBRSR(mPas_100)	0.918128653	0
B64	B67	WBRSR(mPas_5)	WBRSR(mPas_50)	0.93600549	0
B64	B124	WBRSR(mPas_5)	ACTH	0.215693133	2.22E-15
B64	B98	WBRSR(mPas_5)	anti-Jo-1	0.179683023	4.46E-11
B64	B108	WBRSR(mPas_5)	Anti-Cyclin	0.175302504	1.32E-10

B65	B108	WBRSR(mPas_1)	Anti-Cyclin	0.283958695	0
B65	B66	WBRSR(mPas_1)	WBRSR(mPas_100)	0.767276722	0
B65	B67	WBRSR(mPas_1)	WBRSR(mPas_50)	0.797468972	0
B65	B98	WBRSR(mPas_1)	anti-Jo-1	0.288531072	0
B65	B124	WBRSR(mPas_1)	ACTH	0.205677097	4.00E-14
B65	B151	WBRSR(mPas_1)	Anti-CCP	0.153171249	2.10E-08
B65	B69	WBRSR(mPas_1)	PV	0.139511676	3.42E-07
B66	B67	WBRSR(mPas_100)	WBRSR(mPas_50)	0.998365536	0
B66	B124	WBRSR(mPas_100)	ACTH	0.199234836	2.50E-13
B67	B124	WBRSR(mPas_50)	ACTH	0.203764568	6.95E-14
B69	B108	PV	Anti-Cyclin	0.317531981	0
B69	B98	PV	anti-Jo-1	0.312759401	0
B69	B158	PV	IgM	0.219505486	6.66E-16
B69	B74	PV	ApoA1	0.207803364	2.18E-14
B69	B76	PV	ApoB	0.205115559	4.71E-14
B69	B77	PV	TC	0.153431421	1.99E-08
B69	B156	PV	IgE	0.141231337	2.44E-07
B69	B70	PV	LDL-C	0.139508962	3.43E-07
B69	B130	PV	GLB	0.135626938	7.23E-07
B7	B121	WB-Cop	C3	-0.53891266	0
B7	B122	WB-Cop	C4	-0.654263108	0
B7	B124	WB-Cop	ACTH	0.24781699	0
B7	B125	WB-Cop	Cor	0.452553674	0
B7	B136	WB-Cop	5'NT	-0.754318312	0
B7	B157	WB-Cop	IgG	0.255859406	0
B7	B19	WB-Cop	CD3+CD4+/CD3+CD8+	-0.656816491	0
B7	B20	WB-Cop	CD3+CD4+CD8+	0.385291105	0
B7	B21	WB-Cop	CD3+CD4-CD8-	0.485190855	0
B7	B22	WB-Cop	CD3+CD8+(T)	0.312174073	0
B7	B89	WB-Cop	CA 72-4	-0.698728278	0
B7	B18	WB-Cop	CD3+CD4+helper(inducer) T	-0.177728809	7.26E-11
B7	B8	WB-Cop	WB-Zinc	-0.16815063	7.33E-10
B7	B73	WB-Cop	ApoA	-0.134558332	8.84E-07
B70	B132	LDL-C	Dbil	-0.262177907	0
B70	B76	LDL-C	ApoB	0.30813815	0
B70	B77	LDL-C	TC	0.915712898	0
B70	B91	LDL-C	TP	0.219443674	6.66E-16

B70	B130	LDL-C	GLB	0.212089962	6.22E-15
B70	B75	LDL-C	ApoA1/B	-0.206001948	3.64E-14
B70	B218	LDL-C	DBP	0.186783008	7.23E-12
B70	B219	LDL-C	OT	-0.174335472	1.67E-10
B70	B127	LDL-C	A-G ratio	-0.162194221	2.89E-09
B70	B72	LDL-C	HDL-C	0.160173109	4.55E-09
B70	B220	LDL-C	SBP	0.153427366	1.99E-08
B70	B174	LDL-C	Urea/Cr	0.135529844	7.36E-07
B71	B206	TG	Waist	0.234784433	0
B71	B215	TG	BMI	0.25468564	0
B71	B72	TG	HDL-C	-0.393880003	0
B71	B216	TG	BMI	0.220602391	4.44E-16
B71	B77	TG	TC	0.220560996	4.44E-16
B71	B132	TG	Dbil	-0.20483375	5.11E-14
B71	B140	TG	r-GT	0.199437992	2.36E-13
B71	B210	TG	Hip	0.198448452	3.11E-13
B71	B128	TG	ALT	0.197702792	3.83E-13
B71	B218	TG	DBP	0.193944721	1.07E-12
B71	B192	TG	UA	0.19330185	1.28E-12
B71	B222	TG	NS	0.189212362	3.82E-12
B71	B207	TG	WHR	0.180735669	3.42E-11
B71	B141	TG	AST/ALT	-0.180237396	3.88E-11
B71	B126	TG	Alb	0.174478454	1.61E-10
B71	B91	TG	TP	0.174319095	1.68E-10
B72	B141	HDL-C	AST/ALT	0.273055824	0
B72	B190	HDL-C	Cre	-0.240158005	0
B72	B192	HDL-C	UA	-0.332798549	0
B72	B206	HDL-C	Waist	-0.383096046	0
B72	B207	HDL-C	WHR	-0.324555086	0
B72	B210	HDL-C	Hip	-0.285378249	0
B72	B215	HDL-C	BMI	-0.365942658	0
B72	B216	HDL-C	BMI	-0.439348745	0

B72	B221	HDL-C	Hei	-0.286991502	0
B72	B222	HDL-C	NS	-0.441362524	0
B72	B74	HDL-C	ApoA1	0.26633343	0
B72	B77	HDL-C	TC	0.377963385	0
B72	B128	HDL-C	ALT	-0.212109178	6.22E-15
B72	B174	HDL-C	Urea/Cr	0.19795435	3.57E-13
B72	B163	HDL-C	SG	-0.186593306	7.60E-12
B72	B219	HDL-C	OT	-0.172555156	2.57E-10
B72	B153	HDL-C	Glu	-0.156827332	9.54E-09
B72	B75	HDL-C	ApoA1/B	0.141943474	2.12E-07
B72	B209	HDL-C	Smoking	-0.135526604	7.37E-07
B73	B194	ApoA	Cl	-0.172671106	2.50E-10
B73	B75	ApoA	ApoA1/B	-0.168163976	7.30E-10
B73	B111	ApoA	TSH	0.166729938	1.02E-09
B73	B157	ApoA	IgG	-0.166400347	1.10E-09
B73	B101	ApoA	anti-Sc70	-0.158305153	6.89E-09
B73	B102	ApoA	anti-Sm	-0.158305149	6.89E-09
B73	B103	ApoA	anti-SSA/Ro	-0.15830515	6.89E-09
B73	B104	ApoA	anti-SSB	-0.15830515	6.89E-09
B73	B105	ApoA	anti-U1RNP	-0.158305151	6.89E-09
B73	B106	ApoA	anti-P	-0.158305151	6.89E-09
B73	B107	ApoA	AnuA	-0.15830506	6.89E-09
B73	B109	ApoA	AMA-M2	-0.15830515	6.89E-09
B73	B110	ApoA	AHA	-0.158305151	6.89E-09
B73	B97	ApoA	dsDNA	-0.158305151	6.89E-09
B73	B99	ApoA	anti-PM-Scl	-0.158305151	6.89E-09
B73	B81	ApoA	TSGF	0.14816273	6.03E-08

B74	B137	ApoA1	AFU	-0.267435469	0
B74	B139	ApoA1	ADA	-0.293372003	0
B74	B75	ApoA1	ApoA1/B	0.389390152	0
B74	B76	ApoA1	ApoB	0.217387989	1.33E-15
B74	B134	ApoA1	CHE	0.196672847	5.09E-13
B74	B77	ApoA1	TC	0.185014843	1.15E-11
B74	B174	ApoA1	Urea/Cr	0.181937898	2.52E-11
B74	B153	ApoA1	Glu	-0.174908648	1.45E-10
B74	B196	ApoA1	TCO2	0.167376948	8.78E-10
B74	B138	ApoA1	PAB	0.164578455	1.68E-09
B74	B195	ApoA1	Na	0.154844886	1.47E-08
B74	B190	ApoA1	Cre	-0.145576429	1.02E-07
B74	B115	ApoA1	TSH TT3 TT4(TT3)	0.145415408	1.06E-07
B74	B199	ApoA1	Mg	-0.135489842	7.42E-07
B75	B76	ApoA1/B	ApoB	-0.748127075	0
B75	B200	ApoA1/B	OP	-0.183497869	1.69E-11
B75	B137	ApoA1/B	AFU	-0.173175997	2.21E-10
B75	B197	ApoA1/B	AG	-0.170081765	4.64E-10
B75	B196	ApoA1/B	TCO2	0.167512986	8.50E-10
B75	B77	ApoA1/B	TC	-0.158327181	6.86E-09
B75	B136	ApoA1/B	5'NT	-0.150959517	3.36E-08
B75	B134	ApoA1/B	CHE	-0.139217452	3.63E-07
B76	B134	ApoB	CHE	0.275135269	0
B76	B77	ApoB	TC	0.302459998	0
B76	B138	ApoB	PAB	0.198313533	3.23E-13
B76	B197	ApoB	AG	0.168197119	7.25E-10
B76	B195	ApoB	Na	0.153325779	2.03E-08
B76	B136	ApoB	5'NT	0.152423191	2.47E-08
B77	B132	TC	Dbil	-0.264984406	0
B77	B91	TC	TP	0.227779001	0
B77	B130	TC	GLB	0.207988359	2.04E-14

B77	B222	TC	NS	-0.20093637<br>8	1.55E-13
B77	B219	TC	OT	-0.20028364<br>4	1.87E-13
B77	B221	TC	Hei	-0.19111566<br>7	2.30E-12
B77	B218	TC	DBP	0.186625932	7.54E-12
B77	B174	TC	Urea/Cr	0.183180102	1.84E-11
B77	B190	TC	Cre	-0.17936878<br>7	4.82E-11
B77	B220	TC	SBP	0.178102758	6.61E-11
B77	B216	TC	BMI	-0.16776046	8.03E-10
B77	B127	TC	A-G ratio	-0.14995545<br>9	4.15E-08
B78	B138	HBcAb	PAB	0.136705311	5.89E-07
B79	B101	hb V -dna	anti-Sc70	0.373111398	0
B79	B102	hb V -dna	anti-Sm	0.37311139	0
B79	B103	hb V -dna	anti-SSA/Ro	0.373111392	0
B79	B104	hb V -dna	anti-SSB	0.373111392	0
B79	B105	hb V -dna	anti-U1RNP	0.373111392	0
B79	B106	hb V -dna	anti-P	0.373111394	0
B79	B107	hb V -dna	AnuA	0.37311118	0
B79	B109	hb V -dna	AMA-M2	0.373111392	0
B79	B110	hb V -dna	AHA	0.373111393	0
B79	B119	hb V -dna	Hcv-rna	0.368999017	0
B79	B120	hb V -dna	HCV-cAg	0.275634178	0
B79	B125	hb V -dna	Cor	-0.42263104<br>1	0
B79	B134	hb V -dna	CHE	0.230787727	0
B79	B138	hb V -dna	PAB	0.295011835	0
B79	B139	hb V -dna	ADA	-0.36616068<br>5	0
B79	B153	hb V -dna	Glu	-0.29811406<br>1	0
B79	B92	hb V -dna	ALDH2-Glu504Glu	-0.2633534	0
B79	B93	hb V -dna	ALDH2-Glu504Lys	0.239714513	0
B79	B94	hb V -dna	ALDH2-Lys504Lys	0.430097024	0
B79	B97	hb V -dna	dsDNA	0.373111393	0
B79	B99	hb V -dna	anti-PM-Scl	0.373111394	0
B79	B154	hb V -dna	RF	0.192377417	1.64E-12
B79	B115	hb V -dna	TSH TT3 TT4(TT3)	0.182705061	2.07E-11
B79	B170	hb V -dna	U-GLU	-0.17612759<br>8	1.08E-10
B79	B152	hb V -dna	ACA-Ig(G-A-M)	-0.17331652	2.14E-10

B79	B159	hb V -dna	β2-MG	-0.173308748	2.14E-10
B79	B169	hb V -dna	U-CRY	-0.173309616	2.14E-10
B79	B200	hb V -dna	OP	-0.169560974	5.25E-10
B79	B158	hb V -dna	IgM	0.160605022	4.13E-09
B79	B195	hb V -dna	Na	0.151443386	3.04E-08
B79	B135	hb V -dna	TBA	-0.135570157	7.31E-07
B8	B224	WB-Zinc	pmh	-0.140619096	2.76E-07
B80	B118	anti-HP IgG	CEA1	0.147899736	6.36E-08
B81	B108	TSGF	Anti-Cyclin	-0.214473127	3.11E-15
B81	B98	TSGF	anti-Jo-1	-0.210970497	8.44E-15
B81	B157	TSGF	IgG	-0.171055471	3.68E-10
B81	B156	TSGF	IgE	-0.163745335	2.03E-09
B82	B84	CEA	C-PSA	0.275011549	0
B82	B88	CEA	CA 242	0.416593368	0
B82	B86	CEA	TBhCG	-0.177888436	6.97E-11
B83	B86	AFP	TBhCG	0.285547501	0
B83	B88	AFP	CA 242	0.273228492	0
B83	B134	AFP	CHE	0.136826145	5.75E-07
B84	B86	C-PSA	TBhCG	-0.363799755	0
B84	B90	C-PSA	CYFRA 21-1	0.236684117	0
B84	B88	C-PSA	CA 242	0.203982843	6.53E-14
B84	B87	C-PSA	NSE	0.192255498	1.69E-12
B85	B87	SCC	NSE	0.214045891	3.55E-15
B85	B90	SCC	CYFRA 21-1	0.209850278	1.20E-14
B85	B161	SCC	LEU	0.170841407	3.88E-10
B86	B87	TBhCG	NSE	-0.218500154	8.88E-16
B87	B198	NSE	P	0.149873714	4.22E-08
B87	B88	NSE	CA 242	0.149544834	4.52E-08
B89	B121	CA 72-4	C3	0.462065795	0
B89	B122	CA 72-4	C4	0.561205526	0
B89	B125	CA 72-4	Cor	-0.387140716	0

B89	B136	CA 72-4	5'NT	0.67189087	0
B89	B157	CA 72-4	IgG	-0.225977477	0
B89	B124	CA 72-4	ACTH	-0.212370599	5.77E-15
B9	B223	RH	FH	0.994134847	0
B90	B187	CYFRA 21-1	NSE	0.143847142	1.45E-07
B91	B126	TP	Alb	0.483222986	0
B91	B127	TP	A-G ratio	-0.474608267	0
B91	B130	TP	GLB	0.775273184	0
B91	B188	TP	Ca	0.351529697	0
B91	B218	TP	DBP	0.208698568	1.67E-14
B91	B220	TP	SBP	0.142673631	1.84E-07
B92	B125	ALDH2-Glu504Glu	Cor	0.294431822	0
B92	B93	ALDH2-Glu504Glu	ALDH2-Glu504Lys	-0.99909976	0
B92	B94	ALDH2-Glu504Glu	ALDH2-Lys504Lys	0.338130403	0
B92	B101	ALDH2-Glu504Glu	anti-Sc70	-0.200522819	1.74E-13
B92	B102	ALDH2-Glu504Glu	anti-Sm	-0.200522813	1.74E-13
B92	B103	ALDH2-Glu504Glu	anti-SSA/Ro	-0.200522815	1.74E-13
B92	B104	ALDH2-Glu504Glu	anti-SSB	-0.200522816	1.74E-13
B92	B105	ALDH2-Glu504Glu	anti-U1RNP	-0.200522815	1.74E-13
B92	B106	ALDH2-Glu504Glu	anti-P	-0.200522816	1.74E-13
B92	B107	ALDH2-Glu504Glu	AnuA	-0.200522686	1.74E-13
B92	B109	ALDH2-Glu504Glu	AMA-M2	-0.200522815	1.74E-13
B92	B110	ALDH2-Glu504Glu	AHA	-0.200522816	1.74E-13
B92	B97	ALDH2-Glu504Glu	dsDNA	-0.200522815	1.74E-13
B92	B99	ALDH2-Glu504Glu	anti-PM-Scl	-0.200522817	1.74E-13
B92	B199	ALDH2-Glu504Glu	Mg	-0.191593467	2.02E-12
B92	B119	ALDH2-Glu504Glu	Hcv-rna	-0.175108829	1.38E-10
B92	B186	ALDH2-Glu504Glu	PFC	0.142423506	1.93E-07

B93	B125	ALDH2-Glu504Lys	Cor	-0.267679994	0
B93	B94	ALDH2-Glu504Lys	ALDH2-Lys504Lys	-0.377749793	0
B93	B101	ALDH2-Glu504Lys	anti-Sc70	0.182732703	2.06E-11
B93	B102	ALDH2-Glu504Lys	anti-Sm	0.182732699	2.06E-11
B93	B103	ALDH2-Glu504Lys	anti-SSA/Ro	0.182732699	2.06E-11
B93	B104	ALDH2-Glu504Lys	anti-SSB	0.182732699	2.06E-11
B93	B105	ALDH2-Glu504Lys	anti-U1RNP	0.182732699	2.06E-11
B93	B106	ALDH2-Glu504Lys	anti-P	0.182732701	2.06E-11
B93	B107	ALDH2-Glu504Lys	AnuA	0.182732597	2.06E-11
B93	B109	ALDH2-Glu504Lys	AMA-M2	0.182732699	2.06E-11
B93	B110	ALDH2-Glu504Lys	AHA	0.182732699	2.06E-11
B93	B97	ALDH2-Glu504Lys	dsDNA	0.182732699	2.06E-11
B93	B99	ALDH2-Glu504Lys	anti-PM-Scl	0.1827327	2.06E-11
B93	B199	ALDH2-Glu504Lys	Mg	0.1822279	2.34E-11
B93	B119	ALDH2-Glu504Lys	Hcv-rna	0.159514932	5.27E-09
B93	B186	ALDH2-Glu504Lys	PFC	-0.136513998	6.11E-07
B94	B101	ALDH2-Lys504Lys	anti-Sc70	0.322847803	0
B94	B102	ALDH2-Lys504Lys	anti-Sm	0.322847808	0
B94	B103	ALDH2-Lys504Lys	anti-SSA/Ro	0.32284781	0
B94	B104	ALDH2-Lys504Lys	anti-SSB	0.322847812	0
B94	B105	ALDH2-Lys504Lys	anti-U1RNP	0.322847807	0
B94	B106	ALDH2-Lys504Lys	anti-P	0.322847814	0
B94	B107	ALDH2-Lys504Lys	AnuA	0.322847715	0
B94	B109	ALDH2-Lys504Lys	AMA-M2	0.322847812	0
B94	B110	ALDH2-Lys504Lys	AHA	0.322847812	0
B94	B119	ALDH2-Lys504Lys	Hcv-rna	0.283227722	0
B94	B125	ALDH2-Lys504Lys	Cor	-0.488025133	0
B94	B97	ALDH2-Lys504Lys	dsDNA	0.322847813	0
B94	B99	ALDH2-Lys504Lys	anti-PM-Scl	0.32284781	0
B94	B154	ALDH2-Lys504Lys	RF	0.214913133	2.66E-15
B94	B152	ALDH2-Lys504Lys	ACA-Ig(G-A-M)	-0.201195032	1.44E-13
B94	B159	ALDH2-Lys504Lys	β2-MG	-0.201185641	1.45E-13
B94	B169	ALDH2-Lys504Lys	U-CRY	-0.201186688	1.45E-13
B94	B183	ALDH2-Lys504Lys	PTA	0.176948173	8.80E-11
B94	B120	ALDH2-Lys504Lys	HCV-cAg	0.155883595	1.17E-08
B94	B138	ALDH2-Lys504Lys	PAB	0.151653733	2.90E-08
B94	B196	ALDH2-Lys504Lys	TCO2	-0.14285837	1.77E-07

				8	
B94	B199	ALDH2-Lys504Lys	Mg	0.139155536	3.67E-07
B95	B101	anti-cenpb	anti-Sc70	-0.236295827	0
B95	B102	anti-cenpb	anti-Sm	-0.236295821	0
B95	B103	anti-cenpb	anti-SSA/Ro	-0.236295824	0
B95	B104	anti-cenpb	anti-SSB	-0.236295824	0
B95	B105	anti-cenpb	anti-U1RNP	-0.236295824	0
B95	B106	anti-cenpb	anti-P	-0.236295824	0
B95	B107	anti-cenpb	AnuA	-0.236295686	0
B95	B109	anti-cenpb	AMA-M2	-0.236295823	0
B95	B110	anti-cenpb	AHA	-0.236295824	0
B95	B97	anti-cenpb	dsDNA	-0.236295824	0
B95	B99	anti-cenpb	anti-PM-Scl	-0.236295825	0
B95	B146	anti-cenpb	CysC	0.202173583	1.09E-13
B96	B223	dsDNA (IIF)	FH	0.150618808	3.61E-08
B97	B101	dsDNA	anti-Sc70	0.999999999	0
B97	B102	dsDNA	anti-Sm	0.999999997	0
B97	B104	dsDNA	anti-SSB	0.999999989	0
B97	B106	dsDNA	anti-P	0.999999995	0
B97	B107	dsDNA	AnuA	0.999999389	0
B97	B109	dsDNA	AMA-M2	0.999999999	0
B97	B110	dsDNA	AHA	0.999999998	0
B97	B119	dsDNA	Hcv-rna	0.470017908	0
B97	B120	dsDNA	HCV-cAg	0.311176271	0
B97	B125	dsDNA	Cor	-0.319333504	0
B97	B152	dsDNA	ACA-Ig(G-A-M)	-0.263417303	0
B97	B154	dsDNA	RF	0.277811856	0
B97	B155	dsDNA	IgA	-0.244461159	0
B97	B158	dsDNA	IgM	0.292027923	0
B97	B159	dsDNA	β2-MG	-0.26340422	0

				7	
B97	B169	dsDNA	U-CRY	-0.26340567 6	0
B97	B197	dsDNA	AG	0.240927346	0
B97	B98	dsDNA	anti-Jo-1	-0.25528155 4	0
B97	B99	dsDNA	anti-PM-Scl	0.999999992	0
B97	B199	dsDNA	Mg	0.216390065	1.78E-15
B97	B137	dsDNA	AFU	0.204688404	5.33E-14
B97	B134	dsDNA	CHE	0.187404365	6.15E-12
B97	B200	dsDNA	OP	0.1870957	6.66E-12
B97	B195	dsDNA	Na	0.1841634	1.43E-11
B97	B193	dsDNA	K	0.182545784	2.16E-11
B97	B108	dsDNA	Anti-Cyclin	-0.18087605	3.30E-11
B97	B114	dsDNA	TSH TT3 TT4(TT4)	0.173261895	2.17E-10
B97	B138	dsDNA	PAB	0.164858123	1.57E-09
B97	B136	dsDNA	5'NT	0.151810372	2.81E-08
B97	B196	dsDNA	TCO2	-0.13835865 9	4.28E-07
B98	B101	anti-Jo-1	anti-Sc70	-0.25528155 8	0
B98	B102	anti-Jo-1	anti-Sm	-0.25528155 2	0
B98	B103	anti-Jo-1	anti-SSA/Ro	-0.25528155 3	0
B98	B104	anti-Jo-1	anti-SSB	-0.25528155 4	0
B98	B105	anti-Jo-1	anti-U1RNP	-0.25528155 4	0
B98	B106	anti-Jo-1	anti-P	-0.25528155 5	0
B98	B107	anti-Jo-1	AnuA	-0.25528140 9	0
B98	B108	anti-Jo-1	Anti-Cyclin	0.99709348	0
B98	B109	anti-Jo-1	AMA-M2	-0.25528155 3	0
B98	B110	anti-Jo-1	AHA	-0.25528155 4	0
B98	B151	anti-Jo-1	Anti-CCP	0.280418647	0
B98	B156	anti-Jo-1	IgE	0.451351353	0
B98	B179	anti-Jo-1	α1-MG	0.265734301	0
B98	B99	anti-Jo-1	anti-PM-Scl	-0.25528155 5	0
B98	B194	anti-Jo-1	Cl	0.209835414	1.20E-14

B98	B158	anti-Jo-1	IgM	0.201738653	1.24E-13
B98	B157	anti-Jo-1	IgG	0.197601309	3.94E-13
B98	B154	anti-Jo-1	RF	-0.150311112	3.85E-08
B99	B101	anti-PM-Scl	anti-Sc70	0.999999993	0
B99	B104	anti-PM-Scl	anti-SSB	0.999999995	0
B99	B107	anti-PM-Scl	AnuA	0.999999418	0
B99	B119	anti-PM-Scl	Hcv-rna	0.47001791	0
B99	B120	anti-PM-Scl	HCV-cAg	0.311176223	0
B99	B125	anti-PM-Scl	Cor	-0.319333505	0
B99	B152	anti-PM-Scl	ACA-Ig(G-A-M)	-0.263417164	0
B99	B154	anti-PM-Scl	RF	0.277811857	0
B99	B155	anti-PM-Scl	IgA	-0.244461161	0
B99	B158	anti-PM-Scl	IgM	0.292027925	0
B99	B159	anti-PM-Scl	β2-MG	-0.263404441	0
B99	B169	anti-PM-Scl	U-CRY	-0.263405654	0
B99	B197	anti-PM-Scl	AG	0.240927347	0
B99	B199	anti-PM-Scl	Mg	0.216390066	1.78E-15
B99	B137	anti-PM-Scl	AFU	0.204688405	5.33E-14
B99	B134	anti-PM-Scl	CHE	0.187404365	6.15E-12
B99	B200	anti-PM-Scl	OP	0.187095705	6.66E-12
B99	B195	anti-PM-Scl	Na	0.184163407	1.43E-11
B99	B193	anti-PM-Scl	K	0.182545784	2.16E-11
B99	B108	anti-PM-Scl	Anti-Cyclin	-0.180876051	3.30E-11
B99	B114	anti-PM-Scl	TSH TT3 TT4(TT4)	0.173261896	2.17E-10
B99	B138	anti-PM-Scl	PAB	0.164858124	1.57E-09
B99	B136	anti-PM-Scl	5'NT	0.151810372	2.81E-08
B99	B196	anti-PM-Scl	TCO2	-0.13835866	4.28E-07

Supplementary Table 9. PEI Correlations in Coronary+Diabetes

Row	Column	Row	Column	Cor	*P*
B1	B221	Gender	Hei	-0.57222	0
B1	B51	Gender	Hb	-0.47231	0
B1	B222	Gender	NS	-0.45835	4.44E-16
B1	B174	Gender	Urea/Cr	0.432889	3.24E-14
B1	B190	Gender	Cre	-0.39981	3.59E-12
B1	B220	Gender	SBP	0.382085	3.65E-11
B1	B46	Gender	RBC Count	-0.38091	4.23E-11
B1	B205	Gender	KDA	0.362514	4.03E-10
B1	B163	Gender	SG	-0.31881	4.93E-08
B1	B45	Gender	MCHC	-0.30481	1.97E-07
B1	B72	Gender	HDL-C	0.30013	3.09E-07
B1	B161	Gender	LEU	0.287662	9.79E-07
B10	B12	PG I	PG II	0.775037	0
B100	B101	anti-Ro52	anti-Sc70	1	0
B100	B103	anti-Ro52	anti-SSA/Ro	1	0
B100	B105	anti-Ro52	anti-U1RNP	1	0
B100	B106	anti-Ro52	anti-P	1	0
B100	B107	anti-Ro52	AnuA	1	0
B100	B110	anti-Ro52	AHA	1	0
B100	B125	anti-Ro52	Cor	1	0
B100	B151	anti-Ro52	Anti-CCP	0.508198	0
B100	B158	anti-Ro52	IgM	0.497089	0
B100	B178	anti-Ro52	Ig-U	0.6975	0
B100	B179	anti-Ro52	α1-MG	0.724317	0
B100	B180	anti-Ro52	Tf	0.705838	0
B100	B181	anti-Ro52	APTT	1	0
B100	B182	anti-Ro52	TT	-0.99982	0
B100	B183	anti-Ro52	PTA	0.999999	0
B100	B185	anti-Ro52	PT	0.999261	0
B100	B194	anti-Ro52	CI	0.804248	0
B100	B197	anti-Ro52	AG	-0.67006	0
B100	B138	anti-Ro52	PAB	-0.41317	5.70E-13
B100	B137	anti-Ro52	AFU	-0.28849	9.08E-07
B101	B102	anti-Sc70	anti-Sm	1	0
B101	B103	anti-Sc70	anti-SSA/Ro	1	0
B101	B105	anti-Sc70	anti-U1RNP	1	0
B101	B106	anti-Sc70	anti-P	1	0
B101	B107	anti-Sc70	AnuA	1	0
B101	B108	anti-Sc70	Anti-Cyclin	1	0
B101	B109	anti-Sc70	AMA-M2	1	0
B101	B110	anti-Sc70	AHA	1	0
B101	B124	anti-Sc70	ACTH	-1	0

B101	B125	anti-Sc70	Cor	1	0
B101	B151	anti-Sc70	Anti-CCP	0.508198	0
B101	B158	anti-Sc70	IgM	0.497089	0
B101	B178	anti-Sc70	Ig-U	0.6975	0
B101	B179	anti-Sc70	α1-MG	0.724317	0
B101	B180	anti-Sc70	Tf	0.705838	0
B101	B181	anti-Sc70	APTT	1	0
B101	B182	anti-Sc70	TT	-0.99982	0
B101	B183	anti-Sc70	PTA	0.999999	0
B101	B184	anti-Sc70	INR	1	0
B101	B185	anti-Sc70	PT	0.999261	0
B101	B194	anti-Sc70	CI	0.804248	0
B101	B197	anti-Sc70	AG	-0.67006	0
B101	B138	anti-Sc70	PAB	-0.41317	5.70E-13
B101	B137	anti-Sc70	AFU	-0.28849	9.08E-07
B102	B104	anti-Sm	anti-SSB	1	0
B102	B106	anti-Sm	anti-P	1	0
B102	B107	anti-Sm	AnuA	1	0
B102	B108	anti-Sm	Anti-Cyclin	1	0
B102	B151	anti-Sm	Anti-CCP	0.508198	0
B102	B158	anti-Sm	IgM	0.497089	0
B102	B178	anti-Sm	Ig-U	0.6975	0
B102	B179	anti-Sm	α1-MG	0.724317	0
B102	B180	anti-Sm	Tf	0.705838	0
B102	B182	anti-Sm	TT	-0.99982	0
B102	B183	anti-Sm	PTA	0.999999	0
B102	B185	anti-Sm	PT	0.999261	0
B102	B194	anti-Sm	CI	0.804248	0
B102	B197	anti-Sm	AG	-0.67006	0
B102	B138	anti-Sm	PAB	-0.41317	5.70E-13
B102	B137	anti-Sm	AFU	-0.28849	9.08E-07
B103	B105	anti-SSA/Ro	anti-U1RNP	1	0
B103	B106	anti-SSA/Ro	anti-P	1	0
B103	B107	anti-SSA/Ro	AnuA	1	0
B103	B124	anti-SSA/Ro	ACTH	-1	0
B103	B125	anti-SSA/Ro	Cor	1	0
B103	B151	anti-SSA/Ro	Anti-CCP	0.508198	0
B103	B158	anti-SSA/Ro	IgM	0.497089	0
B103	B178	anti-SSA/Ro	Ig-U	0.6975	0
B103	B179	anti-SSA/Ro	α1-MG	0.724317	0
B103	B180	anti-SSA/Ro	Tf	0.705838	0
B103	B181	anti-SSA/Ro	APTT	1	0
B103	B182	anti-SSA/Ro	TT	-0.99982	0
B103	B183	anti-SSA/Ro	PTA	0.999999	0

B103	B185	anti-SSA/Ro	PT	0.999261	0
B103	B194	anti-SSA/Ro	CI	0.804248	0
B103	B197	anti-SSA/Ro	AG	-0.67006	0
B103	B138	anti-SSA/Ro	PAB	-0.41317	5.70E-13
B103	B137	anti-SSA/Ro	AFU	-0.28849	9.08E-07
B104	B107	anti-SSB	AnuA	1	0
B104	B151	anti-SSB	Anti-CCP	0.508198	0
B104	B158	anti-SSB	IgM	0.497089	0
B104	B178	anti-SSB	Ig-U	0.6975	0
B104	B179	anti-SSB	α1-MG	0.724317	0
B104	B180	anti-SSB	Tf	0.705838	0
B104	B182	anti-SSB	TT	-0.99982	0
B104	B183	anti-SSB	PTA	0.999999	0
B104	B185	anti-SSB	PT	0.999261	0
B104	B194	anti-SSB	CI	0.804248	0
B104	B197	anti-SSB	AG	-0.67006	0
B104	B138	anti-SSB	PAB	-0.41317	5.70E-13
B104	B137	anti-SSB	AFU	-0.28849	9.08E-07
B105	B106	anti-U1RNP	anti-P	1	0
B105	B107	anti-U1RNP	AnuA	1	0
B105	B109	anti-U1RNP	AMA-M2	1	0
B105	B110	anti-U1RNP	AHA	1	0
B105	B124	anti-U1RNP	ACTH	-1	0
B105	B125	anti-U1RNP	Cor	1	0
B105	B151	anti-U1RNP	Anti-CCP	0.508198	0
B105	B158	anti-U1RNP	IgM	0.497089	0
B105	B178	anti-U1RNP	Ig-U	0.6975	0
B105	B179	anti-U1RNP	α1-MG	0.724317	0
B105	B180	anti-U1RNP	Tf	0.705838	0
B105	B181	anti-U1RNP	APTT	1	0
B105	B182	anti-U1RNP	TT	-0.99982	0
B105	B183	anti-U1RNP	PTA	0.999999	0
B105	B184	anti-U1RNP	INR	1	0
B105	B185	anti-U1RNP	PT	0.999261	0
B105	B194	anti-U1RNP	CI	0.804248	0
B105	B197	anti-U1RNP	AG	-0.67006	0
B105	B138	anti-U1RNP	PAB	-0.41317	5.70E-13
B105	B137	anti-U1RNP	AFU	-0.28849	9.08E-07
B106	B107	anti-P	AnuA	1	0
B106	B108	anti-P	Anti-Cyclin	1	0
B106	B110	anti-P	AHA	1	0
B106	B124	anti-P	ACTH	-1	0
B106	B125	anti-P	Cor	1	0
B106	B151	anti-P	Anti-CCP	0.508198	0

B106	B158	anti-P	IgM	0.497089	0
B106	B178	anti-P	Ig-U	0.6975	0
B106	B179	anti-P	α1-MG	0.724317	0
B106	B180	anti-P	Tf	0.705838	0
B106	B181	anti-P	APTT	1	0
B106	B182	anti-P	TT	-0.99982	0
B106	B183	anti-P	PTA	0.999999	0
B106	B184	anti-P	INR	1	0
B106	B185	anti-P	PT	0.999261	0
B106	B194	anti-P	CI	0.804248	0
B106	B197	anti-P	AG	-0.67006	0
B106	B138	anti-P	PAB	-0.41317	5.70E-13
B106	B137	anti-P	AFU	-0.28849	9.08E-07
B107	B108	AnuA	Anti-Cyclin	1	0
B107	B109	AnuA	AMA-M2	1	0
B107	B110	AnuA	AHA	1	0
B107	B124	AnuA	ACTH	-1	0
B107	B125	AnuA	Cor	1	0
B107	B151	AnuA	Anti-CCP	0.508198	0
B107	B158	AnuA	IgM	0.497089	0
B107	B178	AnuA	Ig-U	0.6975	0
B107	B179	AnuA	α1-MG	0.724317	0
B107	B180	AnuA	Tf	0.705838	0
B107	B181	AnuA	APTT	1	0
B107	B182	AnuA	TT	-0.99982	0
B107	B183	AnuA	PTA	0.999999	0
B107	B184	AnuA	INR	1	0
B107	B185	AnuA	PT	0.999261	0
B107	B186	AnuA	PFC	-1	0
B107	B194	AnuA	CI	0.804248	0
B107	B197	AnuA	AG	-0.67006	0
B107	B138	AnuA	PAB	-0.41317	5.70E-13
B107	B137	AnuA	AFU	-0.28849	9.08E-07
B108	B151	Anti-Cyclin	Anti-CCP	0.508198	0
B108	B158	Anti-Cyclin	IgM	0.497089	0
B108	B178	Anti-Cyclin	Ig-U	0.6975	0
B108	B179	Anti-Cyclin	α1-MG	0.724317	0
B108	B180	Anti-Cyclin	Tf	0.705838	0
B108	B182	Anti-Cyclin	TT	-0.99982	0
B108	B183	Anti-Cyclin	PTA	0.999999	0
B108	B185	Anti-Cyclin	PT	0.999261	0
B108	B194	Anti-Cyclin	CI	0.804248	0
B108	B197	Anti-Cyclin	AG	-0.67006	0
B108	B138	Anti-Cyclin	PAB	-0.41317	5.70E-13

B108	B137	Anti-Cyclin	AFU	-0.28849	9.08E-07
B109	B151	AMA-M2	Anti-CCP	0.508198	0
B109	B158	AMA-M2	IgM	0.497089	0
B109	B178	AMA-M2	Ig-U	0.6975	0
B109	B179	AMA-M2	α1-MG	0.724317	0
B109	B180	AMA-M2	Tf	0.705838	0
B109	B181	AMA-M2	APTT	1	0
B109	B182	AMA-M2	TT	-0.99982	0
B109	B183	AMA-M2	PTA	0.999999	0
B109	B185	AMA-M2	PT	0.999261	0
B109	B194	AMA-M2	Cl	0.804248	0
B109	B197	AMA-M2	AG	-0.67006	0
B109	B138	AMA-M2	PAB	-0.41317	5.70E-13
B109	B137	AMA-M2	AFU	-0.28849	9.08E-07
B11	B12	PGI/PGII	PG II	-0.57651	0
B11	B14	PGI/PGII	G-17	-0.43246	3.46E-14
B110	B124	AHA	ACTH	-1	0
B110	B125	AHA	Cor	1	0
B110	B151	AHA	Anti-CCP	0.508198	0
B110	B158	AHA	IgM	0.497089	0
B110	B178	AHA	Ig-U	0.6975	0
B110	B179	AHA	α1-MG	0.724317	0
B110	B180	AHA	Tf	0.705838	0
B110	B181	AHA	APTT	1	0
B110	B182	AHA	TT	-0.99982	0
B110	B183	AHA	PTA	0.999999	0
B110	B184	AHA	INR	1	0
B110	B185	AHA	PT	0.999261	0
B110	B194	AHA	Cl	0.804248	0
B110	B197	AHA	AG	-0.67006	0
B110	B138	AHA	PAB	-0.41317	5.70E-13
B110	B137	AHA	AFU	-0.28849	9.08E-07
B111	B112	TSH	FT4	-0.33144	1.33E-08
B111	B194	TSH	Cl	-0.30527	1.89E-07
B112	B114	FT4	TSH TT3 TT4(TT4)	0.4702	0
B112	B113	FT4	FT3	0.386444	2.09E-11
B112	B148	FT4	Anti_TPO	0.305724	1.81E-07
B113	B114	FT3	TSH TT3 TT4(TT4)	0.525263	0
B113	B115	FT3	TSH TT3 TT4(TT3)	0.631155	0
B113	B148	FT3	Anti_TPO	0.551591	0
B113	B165	FT3	bilirubin	-0.46145	4.44E-16
B114	B115	TSH TT3 TT4(TT4)	TSH TT3 TT4(TT3)	0.79744	0
B114	B148	TSH TT3 TT4(TT4)	Anti_TPO	0.334264	9.81E-09
B115	B165	TSH TT3 TT4(TT3)	bilirubin	-0.50974	0

B115	B148	TSH TT3 TT4(TT3)	Anti_TPO	0.428799	5.95E-14
B119	B152	Hcv-rna	ACA-Ig(G-A-M)	-0.99999	0
B119	B169	Hcv-rna	U-CRY	-1	0
B119	B120	Hcv-rna	HCV-cAg	0.404574	1.88E-12
B12	B14	PG II	G-17	0.409985	8.92E-13
B120	B156	HCV-cAg	IgE	-0.56004	0
B120	B121	HCV-cAg	C3	-0.40457	1.88E-12
B120	B122	HCV-cAg	C4	-0.40457	1.88E-12
B120	B152	HCV-cAg	ACA-Ig(G-A-M)	-0.40457	1.88E-12
B120	B159	HCV-cAg	β2-MG	-0.40457	1.88E-12
B120	B169	HCV-cAg	U-CRY	-0.40457	1.88E-12
B120	B150	HCV-cAg	ALP	-0.31865	5.01E-08
B121	B152	C3	ACA-Ig(G-A-M)	0.999986	0
B121	B169	C3	U-CRY	0.999999	0
B122	B152	C4	ACA-Ig(G-A-M)	0.999989	0
B122	B169	C4	U-CRY	1	0
B124	B125	ACTH	Cor	-1	0
B124	B151	ACTH	Anti-CCP	-0.5082	0
B124	B158	ACTH	IgM	-0.49709	0
B124	B178	ACTH	Ig-U	-0.6975	0
B124	B179	ACTH	α1-MG	-0.72432	0
B124	B180	ACTH	Tf	-0.70584	0
B124	B182	ACTH	TT	0.999822	0
B124	B183	ACTH	PTA	-1	0
B124	B184	ACTH	INR	-1	0
B124	B185	ACTH	PT	-0.99926	0
B124	B194	ACTH	Cl	-0.80425	0
B124	B197	ACTH	AG	0.670061	0
B124	B138	ACTH	PAB	0.413173	5.70E-13
B124	B137	ACTH	AFU	0.288494	9.08E-07
B125	B151	Cor	Anti-CCP	0.508198	0
B125	B158	Cor	IgM	0.497089	0
B125	B178	Cor	Ig-U	0.6975	0
B125	B179	Cor	α1-MG	0.724317	0
B125	B180	Cor	Tf	0.705838	0
B125	B181	Cor	APTT	1	0
B125	B182	Cor	TT	-0.99982	0
B125	B183	Cor	PTA	0.999999	0
B125	B185	Cor	PT	0.999261	0
B125	B194	Cor	Cl	0.804248	0
B125	B197	Cor	AG	-0.67006	0
B125	B138	Cor	PAB	-0.41317	5.70E-13
B125	B137	Cor	AFU	-0.28849	9.08E-07
B126	B127	Alb	A-G ratio	0.523231	0

B126	B188	Alb	Ca	0.365475	2.83E-10
B127	B130	A-G ratio	GLB	-0.91855	0
B128	B131	ALT	AST	0.697656	0
B128	B141	ALT	AST/ALT	-0.62781	0
B128	B140	ALT	r-GT	0.395249	6.61E-12
B129	B132	IBIL	Dbil	0.593097	0
B129	B133	IBIL	TBIL	0.970461	0
B131	B140	AST	r-GT	0.44286	7.11E-15
B131	B165	AST	bilirubin	0.291125	7.14E-07
B132	B133	Dbil	TBIL	0.769691	0
B134	B199	CHE	Mg	-0.455	8.88E-16
B134	B200	CHE	OP	0.455672	8.88E-16
B134	B198	CHE	P	0.362124	4.22E-10
B134	B193	CHE	K	0.349133	1.90E-09
B134	B196	CHE	TCO2	0.342543	3.98E-09
B134	B139	CHE	ADA	0.31029	1.16E-07
B135	B197	TBA	AG	0.382989	3.25E-11
B135	B139	TBA	ADA	0.297926	3.80E-07
B136	B139	5'NT	ADA	0.467451	0
B136	B140	5'NT	r-GT	0.612235	0
B136	B137	5'NT	AFU	0.463506	2.22E-16
B136	B196	5'NT	TCO2	0.428994	5.80E-14
B136	B200	5'NT	OP	0.408371	1.12E-12
B136	B150	5'NT	ALP	0.401645	2.80E-12
B136	B138	5'NT	PAB	-0.38577	2.28E-11
B136	B198	5'NT	P	-0.3091	1.30E-07
B137	B139	AFU	ADA	0.766162	0
B137	B194	AFU	Cl	-0.52484	0
B137	B200	AFU	OP	0.676973	0
B137	B140	AFU	r-GT	0.409662	9.33E-13
B137	B197	AFU	AG	0.372788	1.17E-10
B137	B181	AFU	APTT	-0.28849	9.08E-07
B137	B184	AFU	INR	-0.28849	9.08E-07
B137	B186	AFU	PFC	0.288494	9.08E-07
B137	B183	AFU	PTA	-0.28848	9.09E-07
B137	B182	AFU	TT	0.288196	9.33E-07
B137	B185	AFU	PT	-0.28778	9.69E-07
B138	B197	PAB	AG	0.419548	2.30E-13
B138	B181	PAB	APTT	-0.41317	5.70E-13
B138	B183	PAB	PTA	-0.41317	5.70E-13
B138	B184	PAB	INR	-0.41317	5.70E-13
B138	B186	PAB	PFC	0.413173	5.70E-13
B138	B182	PAB	TT	0.413118	5.75E-13
B138	B185	PAB	PT	-0.4129	5.92E-13

B138	B198	PAB	P	0.405393	1.68E-12
B138	B194	PAB	Cl	-0.36041	5.17E-10
B138	B179	PAB	α1-MG	-0.30141	2.73E-07
B138	B180	PAB	Tf	-0.29384	5.56E-07
B138	B178	PAB	Ig-U	-0.29042	7.62E-07
B139	B194	ADA	Cl	-0.4914	0
B139	B200	ADA	OP	0.894141	0
B139	B140	ADA	r-GT	0.430385	4.71E-14
B139	B150	ADA	ALP	0.353673	1.13E-09
B139	B153	ADA	Glu	0.348209	2.11E-09
B14	B118	G-17	CEA1	0.398482	4.30E-12
B14	B80	G-17	anti-HP IgG	0.343525	3.57E-09
B140	B200	r-GT	OP	0.404194	1.98E-12
B140	B227	r-GT	OD	0.337128	7.20E-09
B140	B153	r-GT	Glu	0.303585	2.22E-07
B142	B144	25-(OH)D	Total-PINP	-0.44357	6.22E-15
B142	B151	25-(OH)D	Anti-CCP	-0.37692	6.98E-11
B142	B168	25-(OH)D	NMP22	-0.29844	3.62E-07
B143	B144	β-CTx	Total-PINP	0.912455	0
B143	B145	β-CTx	BGP	0.716386	0
B143	B154	β-CTx	RF	-0.47963	0
B143	B175	β-CTx	ALB-U	-0.29217	6.49E-07
B144	B145	Total-PINP	BGP	0.682211	0
B144	B154	Total-PINP	RF	-0.52378	0
B144	B175	Total-PINP	ALB-U	-0.37378	1.03E-10
B144	B151	Total-PINP	Anti-CCP	0.323049	3.19E-08
B148	B149	Anti_TPO	TGAb	0.448847	2.66E-15
B15	B156	CD3-CD19+(B cell)	IgE	-0.75293	0
B15	B16	CD3-CD19+(B cell)	CD3+T	0.762273	0
B15	B22	CD3-CD19+(B cell)	CD3+CD8+(T)	0.934563	0
B15	B19	CD3-CD19+(B cell)	CD3+CD4+/CD3+CD8+	-0.45738	6.66E-16
B15	B120	CD3-CD19+(B cell)	HCV-cAg	0.381032	4.17E-11
B15	B158	CD3-CD19+(B cell)	IgM	0.35971	5.61E-10
B15	B100	CD3-CD19+(B cell)	anti-Ro52	0.315889	6.62E-08
B15	B101	CD3-CD19+(B cell)	anti-Sc70	0.315889	6.62E-08
B15	B102	CD3-CD19+(B cell)	anti-Sm	0.315889	6.62E-08
B15	B103	CD3-CD19+(B cell)	anti-SSA/Ro	0.315889	6.62E-08
B15	B104	CD3-CD19+(B cell)	anti-SSB	0.315889	6.62E-08
B15	B105	CD3-CD19+(B cell)	anti-U1RNP	0.315889	6.62E-08
B15	B106	CD3-CD19+(B cell)	anti-P	0.315889	6.62E-08
B15	B107	CD3-CD19+(B cell)	AnuA	0.315889	6.62E-08
B15	B108	CD3-CD19+(B cell)	Anti-Cyclin	0.315889	6.62E-08
B15	B109	CD3-CD19+(B cell)	AMA-M2	0.315889	6.62E-08
B15	B110	CD3-CD19+(B cell)	AHA	0.315889	6.62E-08

B15	B124	CD3-CD19+(B cell)	ACTH	-0.31589	6.62E-08
B15	B125	CD3-CD19+(B cell)	Cor	0.315889	6.62E-08
B15	B181	CD3-CD19+(B cell)	APTT	0.315889	6.62E-08
B15	B184	CD3-CD19+(B cell)	INR	0.315889	6.62E-08
B15	B186	CD3-CD19+(B cell)	PFC	-0.31589	6.62E-08
B15	B20	CD3-CD19+(B cell)	CD3+CD4+CD8+	0.315889	6.62E-08
B15	B21	CD3-CD19+(B cell)	CD3+CD4-CD8-	-0.31589	6.62E-08
B15	B79	CD3-CD19+(B cell)	hb V -dna	0.315889	6.62E-08
B15	B92	CD3-CD19+(B cell)	ALDH2-Glu504Glu	0.315889	6.62E-08
B15	B93	CD3-CD19+(B cell)	ALDH2-Glu504Lys	-0.31589	6.62E-08
B15	B94	CD3-CD19+(B cell)	ALDH2-Lys504Lys	0.315889	6.62E-08
B15	B95	CD3-CD19+(B cell)	anti-cenpb	0.315889	6.62E-08
B15	B97	CD3-CD19+(B cell)	dsDNA	0.315889	6.62E-08
B15	B98	CD3-CD19+(B cell)	anti-Jo-1	0.315889	6.62E-08
B15	B99	CD3-CD19+(B cell)	anti-PM-Scl	0.315889	6.62E-08
B15	B183	CD3-CD19+(B cell)	PTA	0.315885	6.63E-08
B15	B182	CD3-CD19+(B cell)	TT	-0.31577	6.70E-08
B15	B185	CD3-CD19+(B cell)	PT	0.315524	6.87E-08
B150	B193	ALP	K	0.344866	3.07E-09
B150	B200	ALP	OP	0.322082	3.53E-08
B150	B175	ALP	ALB-U	0.303947	2.14E-07
B151	B181	Anti-CCP	APTT	0.508198	0
B151	B182	Anti-CCP	TT	-0.50801	0
B151	B183	Anti-CCP	PTA	0.508192	0
B151	B184	Anti-CCP	INR	0.508198	0
B151	B185	Anti-CCP	PT	0.507623	0
B151	B186	Anti-CCP	PFC	-0.5082	0
B151	B158	Anti-CCP	IgM	0.417143	3.25E-13
B151	B155	Anti-CCP	IgA	0.401477	2.87E-12
B151	B194	Anti-CCP	Cl	0.401039	3.04E-12
B151	B179	Anti-CCP	α1-MG	0.364509	3.18E-10
B151	B154	Anti-CCP	RF	-0.35782	7.00E-10
B151	B180	Anti-CCP	Tf	0.355003	9.71E-10
B151	B178	Anti-CCP	Ig-U	0.350723	1.59E-09
B151	B197	Anti-CCP	AG	-0.33963	5.48E-09
B152	B159	ACA-Ig(G-A-M)	β2-MG	0.999992	0
B152	B169	ACA-Ig(G-A-M)	U-CRY	0.999992	0
B153	B170	Glu	U-GLU	0.62779	0
B153	B195	Glu	Na	-0.35044	1.64E-09
B153	B200	Glu	OP	0.34853	2.04E-09
B155	B158	IgA	IgM	0.353613	1.14E-09
B156	B158	IgE	IgM	-0.38655	2.06E-11
B156	B212	IgE	IOP-L	0.289585	8.22E-07
B158	B181	IgM	APTT	0.497089	0

B158	B182	IgM	TT	-0.49688	0
B158	B183	IgM	PTA	0.497081	0
B158	B184	IgM	INR	0.497089	0
B158	B185	IgM	PT	0.496468	0
B158	B186	IgM	PFC	-0.49709	0
B158	B194	IgM	Cl	0.394324	7.47E-12
B158	B179	IgM	α1-MG	0.354984	9.73E-10
B158	B180	IgM	Tf	0.345638	2.82E-09
B158	B178	IgM	Ig-U	0.341432	4.49E-09
B158	B197	IgM	AG	-0.3112	1.06E-07
B159	B169	β2-MG	U-CRY	0.999997	0
B16	B156	CD3+T	IgE	-0.78173	0
B16	B18	CD3+T	CD3+CD4+helper(inducer)T	0.824237	0
B16	B22	CD3+T	CD3+CD8+(T)	0.482099	0
B16	B23	CD3+T	CD3-CD16+CD56+(NK)	-0.83239	0
B16	B120	CD3+T	HCV-cAg	0.378604	5.66E-11
B16	B100	CD3+T	anti-Ro52	-0.37332	1.09E-10
B16	B101	CD3+T	anti-Sc70	-0.37332	1.09E-10
B16	B102	CD3+T	anti-Sm	-0.37332	1.09E-10
B16	B103	CD3+T	anti-SSA/Ro	-0.37332	1.09E-10
B16	B104	CD3+T	anti-SSB	-0.37332	1.09E-10
B16	B105	CD3+T	anti-U1RNP	-0.37332	1.09E-10
B16	B106	CD3+T	anti-P	-0.37332	1.09E-10
B16	B107	CD3+T	AnuA	-0.37332	1.09E-10
B16	B108	CD3+T	Anti-Cyclin	-0.37332	1.09E-10
B16	B109	CD3+T	AMA-M2	-0.37332	1.09E-10
B16	B110	CD3+T	AHA	-0.37332	1.09E-10
B16	B124	CD3+T	ACTH	0.373321	1.09E-10
B16	B125	CD3+T	Cor	-0.37332	1.09E-10
B16	B181	CD3+T	APTT	-0.37332	1.09E-10
B16	B182	CD3+T	TT	0.373317	1.09E-10
B16	B183	CD3+T	PTA	-0.37332	1.09E-10
B16	B184	CD3+T	INR	-0.37332	1.09E-10
B16	B186	CD3+T	PFC	0.373321	1.09E-10
B16	B20	CD3+T	CD3+CD4+CD8+	-0.37332	1.09E-10
B16	B21	CD3+T	CD3+CD4-CD8-	0.373321	1.09E-10
B16	B79	CD3+T	hbＶ-dna	-0.37332	1.09E-10
B16	B92	CD3+T	ALDH2-Glu504Glu	-0.37332	1.09E-10
B16	B93	CD3+T	ALDH2-Glu504Lys	0.373321	1.09E-10
B16	B94	CD3+T	ALDH2-Lys504Lys	-0.37332	1.09E-10
B16	B95	CD3+T	anti-cenpb	-0.37332	1.09E-10
B16	B97	CD3+T	dsDNA	-0.37332	1.09E-10
B16	B98	CD3+T	anti-Jo-1	-0.37332	1.09E-10
B16	B99	CD3+T	anti-PM-Scl	-0.37332	1.09E-10

B16	B185	CD3+T	PT	-0.37317	1.11E-10
B16	B81	CD3+T	TSGF	0.368619	1.94E-10
B16	B157	CD3+T	IgG	-0.33995	5.29E-09
B16	B155	CD3+T	IgA	0.302949	2.36E-07
B161	B173	LEU	NTT	0.464776	2.22E-16
B163	B170	SG	U-GLU	0.372608	1.19E-10
B163	B176	SG	Ucr	0.3334	1.08E-08
B163	B166	SG	Pro	0.307529	1.52E-07
B168	B176	NMP22	Ucr	0.303471	2.24E-07
B17	B116	CD3+CD16+CD56+NK	TT3	-0.98074	0
B17	B28	CD3+CD16+CD56+NK	PRL	-0.68803	0
B174	B191	Urea/Cr	Urea	0.477027	0
B174	B190	Urea/Cr	Cre	-0.42169	1.69E-13
B174	B221	Urea/Cr	Hei	-0.34741	2.31E-09
B175	B177	ALB-U	ACR	0.743122	0
B175	B176	ALB-U	Ucr	0.334717	9.34E-09
B178	B179	Ig-U	α1-MG	0.999268	0
B178	B180	Ig-U	Tf	0.999932	0
B178	B181	Ig-U	APTT	0.6975	0
B178	B182	Ig-U	TT	-0.69733	0
B178	B183	Ig-U	PTA	0.697497	0
B178	B184	Ig-U	INR	0.6975	0
B178	B185	Ig-U	PT	0.696886	0
B178	B186	Ig-U	PFC	-0.6975	0
B178	B194	Ig-U	Cl	0.557176	0
B178	B197	Ig-U	AG	-0.46693	0
B179	B180	α1-MG	Tf	0.999646	0
B179	B181	α1-MG	APTT	0.724317	0
B179	B182	α1-MG	TT	-0.72408	0
B179	B183	α1-MG	PTA	0.72431	0
B179	B184	α1-MG	INR	0.724317	0
B179	B185	α1-MG	PT	0.723567	0
B179	B186	α1-MG	PFC	-0.72432	0
B179	B194	α1-MG	Cl	0.578783	0
B179	B197	α1-MG	AG	-0.48492	0
B18	B100	CD3+CD4+helper(inducer)T	anti-Ro52	-0.83301	0
B18	B101	CD3+CD4+helper(inducer)T	anti-Sc70	-0.83301	0
B18	B102	CD3+CD4+helper(inducer)T	anti-Sm	-0.83301	0
B18	B103	CD3+CD4+helper(inducer)T	anti-SSA/Ro	-0.83301	0
B18	B104	CD3+CD4+helper(inducer)T	anti-SSB	-0.83301	0
B18	B105	CD3+CD4+helper(inducer)T	anti-U1RNP	-0.83301	0
B18	B106	CD3+CD4+helper(inducer)T	anti-P	-0.83301	0
B18	B107	CD3+CD4+helper(inducer)T	AnuA	-0.83301	0
B18	B108	CD3+CD4+helper(inducer)T	Anti-Cyclin	-0.83301	0

B18	B109	CD3+CD4+helper(inducer)T	AMA-M2	-0.83301	0
B18	B110	CD3+CD4+helper(inducer)T	AHA	-0.83301	0
B18	B124	CD3+CD4+helper(inducer)T	ACTH	0.833011	0
B18	B125	CD3+CD4+helper(inducer)T	Cor	-0.83301	0
B18	B156	CD3+CD4+helper(inducer)T	IgE	-0.50694	0
B18	B178	CD3+CD4+helper(inducer)T	Ig-U	-0.58245	0
B18	B179	CD3+CD4+helper(inducer)T	α1-MG	-0.60473	0
B18	B180	CD3+CD4+helper(inducer)T	Tf	-0.58938	0
B18	B181	CD3+CD4+helper(inducer)T	APTT	-0.83301	0
B18	B182	CD3+CD4+helper(inducer)T	TT	0.832901	0
B18	B183	CD3+CD4+helper(inducer)T	PTA	-0.83301	0
B18	B184	CD3+CD4+helper(inducer)T	INR	-0.83301	0
B18	B185	CD3+CD4+helper(inducer)T	PT	-0.83247	0
B18	B186	CD3+CD4+helper(inducer)T	PFC	0.833011	0
B18	B19	CD3+CD4+helper(inducer)T	CD3+CD4+/CD3+CD8+	0.738519	0
B18	B194	CD3+CD4+helper(inducer)T	CI	-0.65147	0
B18	B197	CD3+CD4+helper(inducer)T	AG	0.578919	0
B18	B20	CD3+CD4+helper(inducer)T	CD3+CD4+CD8+	-0.83301	0
B18	B21	CD3+CD4+helper(inducer)T	CD3+CD4-CD8-	0.833011	0
B18	B23	CD3+CD4+helper(inducer)T	CD3-CD16+CD56+(NK)	-0.99989	0
B18	B79	CD3+CD4+helper(inducer)T	hbV-dna	-0.83301	0
B18	B81	CD3+CD4+helper(inducer)T	TSGF	0.554403	0
B18	B92	CD3+CD4+helper(inducer)T	ALDH2-Glu504Glu	-0.83301	0
B18	B93	CD3+CD4+helper(inducer)T	ALDH2-Glu504Lys	0.833011	0
B18	B94	CD3+CD4+helper(inducer)T	ALDH2-Lys504Lys	-0.83301	0
B18	B95	CD3+CD4+helper(inducer)T	anti-cenpb	-0.83301	0
B18	B97	CD3+CD4+helper(inducer)T	dsDNA	-0.83301	0
B18	B98	CD3+CD4+helper(inducer)T	anti-Jo-1	-0.83301	0
B18	B99	CD3+CD4+helper(inducer)T	anti-PM-Scl	-0.83301	0
B18	B151	CD3+CD4+helper(inducer)T	Anti-CCP	-0.42623	8.70E-14
B18	B55	CD3+CD4+helper(inducer)T	TK	-0.41853	2.66E-13
B18	B54	CD3+CD4+helper(inducer)T	ESR	-0.41504	4.38E-13
B18	B138	CD3+CD4+helper(inducer)T	PAB	0.377217	6.73E-11
B18	B158	CD3+CD4+helper(inducer)T	IgM	-0.29589	4.60E-07
B180	B181	Tf	APTT	0.705838	0
B180	B182	Tf	TT	-0.70566	0
B180	B183	Tf	PTA	0.705835	0
B180	B184	Tf	INR	0.705838	0
B180	B185	Tf	PT	0.705219	0
B180	B186	Tf	PFC	-0.70584	0
B180	B194	Tf	CI	0.563926	0
B180	B197	Tf	AG	-0.47252	0
B181	B182	APTT	TT	-0.99982	0
B181	B183	APTT	PTA	0.999999	0

B181	B185	APTT	PT	0.999261	0
B181	B194	APTT	CI	0.804248	0
B181	B197	APTT	AG	-0.67006	0
B182	B183	TT	PTA	-0.99984	0
B182	B184	TT	INR	-0.99982	0
B182	B185	TT	PT	-0.99981	0
B182	B186	TT	PFC	0.999822	0
B182	B194	TT	CI	-0.80474	0
B182	B197	TT	AG	0.66989	0
B183	B184	PTA	INR	0.999999	0
B183	B185	PTA	PT	0.999304	0
B183	B186	PTA	PFC	-1	0
B183	B194	PTA	CI	0.804286	0
B183	B197	PTA	AG	-0.67006	0
B184	B185	INR	PT	0.999261	0
B184	B194	INR	CI	0.804248	0
B184	B197	INR	AG	-0.67006	0
B185	B186	PT	PFC	-0.99926	0
B185	B194	PT	CI	0.804938	0
B185	B197	PT	AG	-0.66946	0
B186	B194	PFC	CI	-0.80425	0
B186	B197	PFC	AG	0.670061	0
B189	B190	eGFB	Cre	-0.72456	0
B189	B191	eGFB	Urea	-0.49177	0
B189	B192	eGFB	UA	-0.32414	2.85E-08
B19	B100	CD3+CD4+/CD3+CD8+	anti-Ro52	-0.98822	0
B19	B101	CD3+CD4+/CD3+CD8+	anti-Sc70	-0.98822	0
B19	B102	CD3+CD4+/CD3+CD8+	anti-Sm	-0.98822	0
B19	B103	CD3+CD4+/CD3+CD8+	anti-SSA/Ro	-0.98822	0
B19	B104	CD3+CD4+/CD3+CD8+	anti-SSB	-0.98822	0
B19	B105	CD3+CD4+/CD3+CD8+	anti-U1RNP	-0.98822	0
B19	B106	CD3+CD4+/CD3+CD8+	anti-P	-0.98822	0
B19	B107	CD3+CD4+/CD3+CD8+	AnuA	-0.98822	0
B19	B108	CD3+CD4+/CD3+CD8+	Anti-Cyclin	-0.98822	0
B19	B109	CD3+CD4+/CD3+CD8+	AMA-M2	-0.98822	0
B19	B110	CD3+CD4+/CD3+CD8+	AHA	-0.98822	0
B19	B124	CD3+CD4+/CD3+CD8+	ACTH	0.988218	0
B19	B125	CD3+CD4+/CD3+CD8+	Cor	-0.98822	0
B19	B151	CD3+CD4+/CD3+CD8+	Anti-CCP	-0.50141	0
B19	B158	CD3+CD4+/CD3+CD8+	IgM	-0.52393	0
B19	B178	CD3+CD4+/CD3+CD8+	Ig-U	-0.68889	0
B19	B179	CD3+CD4+/CD3+CD8+	α1-MG	-0.71541	0
B19	B180	CD3+CD4+/CD3+CD8+	Tf	-0.69713	0
B19	B181	CD3+CD4+/CD3+CD8+	APTT	-0.98822	0

B19	B182	CD3+CD4+/CD3+CD8+	TT	0.988032	0
B19	B183	CD3+CD4+/CD3+CD8+	PTA	-0.98822	0
B19	B184	CD3+CD4+/CD3+CD8+	INR	-0.98822	0
B19	B185	CD3+CD4+/CD3+CD8+	PT	-0.98747	0
B19	B186	CD3+CD4+/CD3+CD8+	PFC	0.988218	0
B19	B194	CD3+CD4+/CD3+CD8+	Cl	-0.79988	0
B19	B197	CD3+CD4+/CD3+CD8+	AG	0.656425	0
B19	B20	CD3+CD4+/CD3+CD8+	CD3+CD4+CD8+	-0.98822	0
B19	B21	CD3+CD4+/CD3+CD8+	CD3+CD4-CD8-	0.988218	0
B19	B22	CD3+CD4+/CD3+CD8+	CD3+CD8+(T)	-0.74385	0
B19	B23	CD3+CD4+/CD3+CD8+	CD3-CD16+CD56+(NK)	-0.72863	0
B19	B54	CD3+CD4+/CD3+CD8+	ESR	-0.48943	0
B19	B55	CD3+CD4+/CD3+CD8+	TK	-0.49564	0
B19	B79	CD3+CD4+/CD3+CD8+	hbV-dna	-0.98822	0
B19	B81	CD3+CD4+/CD3+CD8+	TSGF	0.514626	0
B19	B92	CD3+CD4+/CD3+CD8+	ALDH2-Glu504Glu	-0.98822	0
B19	B93	CD3+CD4+/CD3+CD8+	ALDH2-Glu504Lys	0.988218	0
B19	B94	CD3+CD4+/CD3+CD8+	ALDH2-Lys504Lys	-0.98822	0
B19	B95	CD3+CD4+/CD3+CD8+	anti-cenpb	-0.98822	0
B19	B97	CD3+CD4+/CD3+CD8+	dsDNA	-0.98822	0
B19	B98	CD3+CD4+/CD3+CD8+	anti-Jo-1	-0.98822	0
B19	B99	CD3+CD4+/CD3+CD8+	anti-PM-Scl	-0.98822	0
B19	B138	CD3+CD4+/CD3+CD8+	PAB	0.399165	3.92E-12
B19	B137	CD3+CD4+/CD3+CD8+	AFU	0.300417	3.00E-07
B190	B191	Cre	Urea	0.556065	0
B190	B192	Cre	UA	0.426496	8.39E-14
B193	B196	K	TCO2	0.429013	5.77E-14
B193	B197	K	AG	-0.38685	1.98E-11
B194	B197	Cl	AG	-0.66428	0
B194	B200	Cl	OP	-0.41438	4.81E-13
B195	B198	Na	P	0.586244	0
B195	B196	Na	TCO2	0.458762	4.44E-16
B196	B197	TCO2	AG	-0.40744	1.27E-12
B197	B198	AG	P	0.35507	9.64E-10
B2	B189	Age	eGFB	-0.57178	0
B2	B51	Age	Hb	-0.43089	4.35E-14
B2	B220	Age	SBP	0.411746	6.97E-13
B2	B46	Age	RBC Count	-0.37315	1.11E-10
B2	B221	Age	Hei	-0.32417	2.85E-08
B2	B126	Age	Alb	-0.32206	3.53E-08
B2	B128	Age	ALT	-0.31066	1.11E-07
B2	B222	Age	NS	-0.30976	1.22E-07
B2	B216	Age	BMI	-0.30188	2.61E-07
B20	B101	CD3+CD4+CD8+	anti-Sc70	1	0

B20	B103	CD3+CD4+CD8+	anti-SSA/Ro	1	0
B20	B105	CD3+CD4+CD8+	anti-U1RNP	1	0
B20	B107	CD3+CD4+CD8+	AnuA	1	0
B20	B110	CD3+CD4+CD8+	AHA	1	0
B20	B124	CD3+CD4+CD8+	ACTH	-1	0
B20	B125	CD3+CD4+CD8+	Cor	1	0
B20	B151	CD3+CD4+CD8+	Anti-CCP	0.508198	0
B20	B158	CD3+CD4+CD8+	IgM	0.497089	0
B20	B178	CD3+CD4+CD8+	Ig-U	0.6975	0
B20	B179	CD3+CD4+CD8+	α1-MG	0.724317	0
B20	B180	CD3+CD4+CD8+	Tf	0.705838	0
B20	B182	CD3+CD4+CD8+	TT	-0.99982	0
B20	B183	CD3+CD4+CD8+	PTA	0.999999	0
B20	B185	CD3+CD4+CD8+	PT	0.999261	0
B20	B194	CD3+CD4+CD8+	Cl	0.804248	0
B20	B197	CD3+CD4+CD8+	AG	-0.67006	0
B20	B22	CD3+CD4+CD8+	CD3+CD8+(T)	0.632798	0
B20	B23	CD3+CD4+CD8+	CD3-CD16+CD56+(NK)	0.824875	0
B20	B54	CD3+CD4+CD8+	ESR	0.495827	0
B20	B55	CD3+CD4+CD8+	TK	0.501718	0
B20	B79	CD3+CD4+CD8+	hb V -dna	1	0
B20	B81	CD3+CD4+CD8+	TSGF	-0.54814	0
B20	B92	CD3+CD4+CD8+	ALDH2-Glu504Glu	1	0
B20	B93	CD3+CD4+CD8+	ALDH2-Glu504Lys	-1	0
B20	B94	CD3+CD4+CD8+	ALDH2-Lys504Lys	1	0
B20	B97	CD3+CD4+CD8+	dsDNA	1	0
B20	B98	CD3+CD4+CD8+	anti-Jo-1	1	0
B20	B99	CD3+CD4+CD8+	anti-PM-Scl	1	0
B20	B138	CD3+CD4+CD8+	PAB	-0.41317	5.70E-13
B20	B137	CD3+CD4+CD8+	AFU	-0.28849	9.08E-07
B201	B204	e-habits	Drinking	0.303398	2.26E-07
B206	B207	Waist	WHR	0.631412	0
B206	B210	Waist	Hip	0.720887	0
B206	B215	Waist	BMI	0.770044	0
B206	B216	Waist	BMI	0.756643	0
B206	B222	Waist	NS	0.669739	0
B207	B222	WHR	NS	0.353821	1.11E-09
B21	B105	CD3+CD4-CD8-	anti-U1RNP	-1	0
B21	B107	CD3+CD4-CD8-	AnuA	-1	0
B21	B110	CD3+CD4-CD8-	AHA	-1	0
B21	B151	CD3+CD4-CD8-	Anti-CCP	-0.5082	0
B21	B158	CD3+CD4-CD8-	IgM	-0.49709	0
B21	B178	CD3+CD4-CD8-	Ig-U	-0.6975	0
B21	B179	CD3+CD4-CD8-	α1-MG	-0.72432	0

B21	B180	CD3+CD4-CD8-	Tf	-0.70584	0
B21	B182	CD3+CD4-CD8-	TT	0.999822	0
B21	B183	CD3+CD4-CD8-	PTA	-1	0
B21	B185	CD3+CD4-CD8-	PT	-0.99926	0
B21	B194	CD3+CD4-CD8-	Cl	-0.80425	0
B21	B197	CD3+CD4-CD8-	AG	0.670061	0
B21	B22	CD3+CD4-CD8-	CD3+CD8+(T)	-0.6328	0
B21	B23	CD3+CD4-CD8-	CD3-CD16+CD56+(NK)	-0.82488	0
B21	B54	CD3+CD4-CD8-	ESR	-0.49583	0
B21	B55	CD3+CD4-CD8-	TK	-0.50172	0
B21	B81	CD3+CD4-CD8-	TSGF	0.548139	0
B21	B94	CD3+CD4-CD8-	ALDH2-Lys504Lys	-1	0
B21	B97	CD3+CD4-CD8-	dsDNA	-1	0
B21	B98	CD3+CD4-CD8-	anti-Jo-1	-1	0
B21	B99	CD3+CD4-CD8-	anti-PM-Scl	-1	0
B21	B138	CD3+CD4-CD8-	PAB	0.413173	5.70E-13
B21	B137	CD3+CD4-CD8-	AFU	0.288494	9.08E-07
B210	B215	Hip	BMI	0.80334	0
B210	B216	Hip	BMI	0.788474	0
B210	B222	Hip	NS	0.533358	0
B211	B212	IOP-R	IOP-L	0.672445	0
B213	B214	CVA-R	CVA-L	0.777644	0
B215	B216	BMI	BMI	0.770646	0
B215	B222	BMI	NS	0.571705	0
B216	B221	BMI	Hei	0.621133	0
B216	B222	BMI	NS	0.719286	0
B218	B220	DBP	SBP	0.472626	0
B22	B100	CD3+CD8+(T)	anti-Ro52	0.632798	0
B22	B101	CD3+CD8+(T)	anti-Sc70	0.632798	0
B22	B102	CD3+CD8+(T)	anti-Sm	0.632798	0
B22	B103	CD3+CD8+(T)	anti-SSA/Ro	0.632798	0
B22	B104	CD3+CD8+(T)	anti-SSB	0.632798	0
B22	B105	CD3+CD8+(T)	anti-U1RNP	0.632798	0
B22	B106	CD3+CD8+(T)	anti-P	0.632798	0
B22	B107	CD3+CD8+(T)	AnuA	0.632798	0
B22	B108	CD3+CD8+(T)	Anti-Cyclin	0.632798	0
B22	B109	CD3+CD8+(T)	AMA-M2	0.632798	0
B22	B110	CD3+CD8+(T)	AHA	0.632798	0
B22	B124	CD3+CD8+(T)	ACTH	-0.6328	0
B22	B125	CD3+CD8+(T)	Cor	0.632798	0
B22	B156	CD3+CD8+(T)	IgE	-0.58943	0
B22	B158	CD3+CD8+(T)	IgM	0.479969	0
B22	B181	CD3+CD8+(T)	APTT	0.632798	0
B22	B182	CD3+CD8+(T)	TT	-0.63263	0

B22	B183	CD3+CD8+(T)	PTA	0.632795	0
B22	B184	CD3+CD8+(T)	INR	0.632798	0
B22	B185	CD3+CD8+(T)	PT	0.632224	0
B22	B186	CD3+CD8+(T)	PFC	-0.6328	0
B22	B194	CD3+CD8+(T)	Cl	0.534791	0
B22	B79	CD3+CD8+(T)	hb V -dna	0.632798	0
B22	B92	CD3+CD8+(T)	ALDH2-Glu504Glu	0.632798	0
B22	B93	CD3+CD8+(T)	ALDH2-Glu504Lys	-0.6328	0
B22	B94	CD3+CD8+(T)	ALDH2-Lys504Lys	0.632798	0
B22	B95	CD3+CD8+(T)	anti-cenpb	0.632798	0
B22	B97	CD3+CD8+(T)	dsDNA	0.632798	0
B22	B98	CD3+CD8+(T)	anti-Jo-1	0.632798	0
B22	B99	CD3+CD8+(T)	anti-PM-Scl	0.632798	0
B22	B179	CD3+CD8+(T)	α1-MG	0.456435	8.88E-16
B22	B180	CD3+CD8+(T)	Tf	0.44468	5.33E-15
B22	B178	CD3+CD8+(T)	Ig-U	0.439381	1.20E-14
B22	B197	CD3+CD8+(T)	AG	-0.39497	6.86E-12
B22	B151	CD3+CD8+(T)	Anti-CCP	0.31754	5.61E-08
B22	B55	CD3+CD8+(T)	TK	0.316652	6.13E-08
B22	B54	CD3+CD8+(T)	ESR	0.310941	1.08E-07
B22	B120	CD3+CD8+(T)	HCV-cAg	0.307639	1.50E-07
B221	B222	Hei	NS	0.432367	3.51E-14
B227	B228	OD	OS	0.396424	5.66E-12
B23	B100	CD3-CD16+CD56+(NK)	anti-Ro52	0.824875	0
B23	B101	CD3-CD16+CD56+(NK)	anti-Sc70	0.824875	0
B23	B102	CD3-CD16+CD56+(NK)	anti-Sm	0.824875	0
B23	B103	CD3-CD16+CD56+(NK)	anti-SSA/Ro	0.824875	0
B23	B104	CD3-CD16+CD56+(NK)	anti-SSB	0.824875	0
B23	B105	CD3-CD16+CD56+(NK)	anti-U1RNP	0.824875	0
B23	B106	CD3-CD16+CD56+(NK)	anti-P	0.824875	0
B23	B107	CD3-CD16+CD56+(NK)	AnuA	0.824875	0
B23	B108	CD3-CD16+CD56+(NK)	Anti-Cyclin	0.824875	0
B23	B109	CD3-CD16+CD56+(NK)	AMA-M2	0.824875	0
B23	B110	CD3-CD16+CD56+(NK)	AHA	0.824875	0
B23	B124	CD3-CD16+CD56+(NK)	ACTH	-0.82488	0
B23	B125	CD3-CD16+CD56+(NK)	Cor	0.824875	0
B23	B156	CD3-CD16+CD56+(NK)	IgE	0.516238	0
B23	B178	CD3-CD16+CD56+(NK)	Ig-U	0.576808	0
B23	B179	CD3-CD16+CD56+(NK)	α1-MG	0.598867	0
B23	B180	CD3-CD16+CD56+(NK)	Tf	0.583669	0
B23	B181	CD3-CD16+CD56+(NK)	APTT	0.824875	0
B23	B182	CD3-CD16+CD56+(NK)	TT	-0.82477	0
B23	B183	CD3-CD16+CD56+(NK)	PTA	0.824877	0
B23	B184	CD3-CD16+CD56+(NK)	INR	0.824875	0

B23	B185	CD3-CD16+CD56+(NK)	PT	0.824344	0
B23	B186	CD3-CD16+CD56+(NK)	PFC	-0.82488	0
B23	B194	CD3-CD16+CD56+(NK)	CI	0.644522	0
B23	B197	CD3-CD16+CD56+(NK)	AG	-0.57392	0
B23	B79	CD3-CD16+CD56+(NK)	hb V -dna	0.824875	0
B23	B81	CD3-CD16+CD56+(NK)	TSGF	-0.55207	0
B23	B92	CD3-CD16+CD56+(NK)	ALDH2-Glu504Glu	0.824875	0
B23	B93	CD3-CD16+CD56+(NK)	ALDH2-Glu504Lys	-0.82488	0
B23	B94	CD3-CD16+CD56+(NK)	ALDH2-Lys504Lys	0.824875	0
B23	B95	CD3-CD16+CD56+(NK)	anti-cenpb	0.824875	0
B23	B97	CD3-CD16+CD56+(NK)	dsDNA	0.824875	0
B23	B98	CD3-CD16+CD56+(NK)	anti-Jo-1	0.824875	0
B23	B99	CD3-CD16+CD56+(NK)	anti-PM-Scl	0.824875	0
B23	B151	CD3-CD16+CD56+(NK)	Anti-CCP	0.422155	1.58E-13
B23	B55	CD3-CD16+CD56+(NK)	TK	0.414464	4.75E-13
B23	B54	CD3-CD16+CD56+(NK)	ESR	0.411053	7.68E-13
B23	B138	CD3-CD16+CD56+(NK)	PAB	-0.37458	9.35E-11
B23	B158	CD3-CD16+CD56+(NK)	IgM	0.289272	8.46E-07
B24	B26	αHBDH	CKMB	0.762881	0
B24	B27	αHBDH	LDH	0.624897	0
B24	B134	αHBDH	CHE	0.31918	4.75E-08
B25	B58	CK	HCT	-0.47032	0
B25	B62	CK	WBHSRVI	-0.36863	1.93E-10
B25	B63	CK	WBRSR(mPas_200)	-0.3461	2.67E-09
B25	B66	CK	WBRSR(mPas_100)	-0.32519	2.56E-08
B25	B67	CK	WBRSR(mPas_50)	-0.3012	2.79E-07
B26	B27	CKMB	LDH	0.465097	2.22E-16
B27	B200	LDH	OP	0.451274	1.78E-15
B27	B139	LDH	ADA	0.440671	9.77E-15
B27	B88	LDH	CA 242	0.415229	4.27E-13
B27	B82	LDH	CEA	0.390215	1.28E-11
B27	B134	LDH	CHE	0.385252	2.44E-11
B27	B75	LDH	ApoA1/B	0.368923	1.87E-10
B27	B87	LDH	NSE	0.303496	2.24E-07
B27	B137	LDH	AFU	0.300984	2.85E-07
B28	B202	PRL	e-times	-0.77621	0
B28	B31	PRL	FSH	-0.80403	0
B29	B116	E2	TT3	-0.48807	0
B29	B223	E2	FH	-0.70598	0
B29	B202	E2	e-times	-0.37418	9.82E-11
B30	B167	LH	PC	0.920567	0
B30	B32	LH	Testo	0.453743	1.33E-15
B31	B35	FSH	β2-MG	-0.56575	0
B32	B167	Testo	PC	0.431174	4.17E-14

B33	B116	Prog	TT3	-0.68362	0
B33	B35	Prog	β2-MG	-0.89583	0
B35	B202	β2-MG	e-times	0.82734	0
B36	B167	V-Lym%	PC	0.777374	0
B36	B96	V-Lym%	dsDNA (IIF)	0.781452	0
B37	B139	BAND	ADA	-0.49733	0
B37	B38	BAND	BAND%	-1	0
B37	B71	BAND	TG	-0.62386	0
B37	B82	BAND	CEA	-0.521	0
B37	B90	BAND	CYFRA 21-1	-0.53967	0
B37	B88	BAND	CA 242	-0.46151	4.44E-16
B37	B87	BAND	NSE	-0.44548	4.66E-15
B37	B200	BAND	OP	-0.43491	2.40E-14
B37	B85	BAND	SCC	0.412518	6.25E-13
B37	B198	BAND	P	0.331499	1.32E-08
B37	B195	BAND	Na	0.32272	3.30E-08
B37	B194	BAND	Cl	0.31422	7.83E-08
B37	B196	BAND	TCO2	0.305274	1.89E-07
B37	B199	BAND	Mg	-0.30011	3.09E-07
B38	B139	BAND%	ADA	0.497328	0
B38	B71	BAND%	TG	0.623858	0
B38	B82	BAND%	CEA	0.521003	0
B38	B90	BAND%	CYFRA 21-1	0.539673	0
B38	B88	BAND%	CA 242	0.461513	4.44E-16
B38	B87	BAND%	NSE	0.445478	4.66E-15
B38	B200	BAND%	OP	0.434906	2.40E-14
B38	B85	BAND%	SCC	-0.41252	6.25E-13
B38	B198	BAND%	P	-0.3315	1.32E-08
B38	B195	BAND%	Na	-0.32272	3.30E-08
B38	B194	BAND%	Cl	-0.31422	7.83E-08
B38	B196	BAND%	TCO2	-0.30527	1.89E-07
B38	B199	BAND%	Mg	0.300114	3.09E-07
B39	B44	Other cells	MCH	-0.31145	1.03E-07
B39	B43	Other cells	MCV	-0.29903	3.42E-07
B4	B17	WB-Ca	CD3+CD16+CD56+NK	-0.55141	0
B4	B29	WB-Ca	E2	-0.54621	0
B40	B42	WBC	MON	0.754101	0
B40	B209	WBC	Smoking	0.316292	6.36E-08
B41	B42	MON %	MON	0.561751	0
B42	B51	MON	Hb	0.293758	5.60E-07
B43	B44	MCV	MCH	0.89385	0
B43	B47	MCV	RDW	0.523748	0
B43	B46	MCV	RBC Count	-0.46029	4.44E-16
B44	B45	MCH	MCHC	0.531605	0

B44	B51	MCH	Hb	0.327252	2.06E-08
B44	B46	MCH	RBC Count	-0.32007	4.34E-08
B45	B51	MCHC	Hb	0.501964	0
B45	B47	MCHC	RDW	-0.39545	6.44E-12
B46	B51	RBC Count	Hb	0.785559	0
B46	B47	RBC Count	RDW	-0.40994	8.98E-13
B46	B189	RBC Count	eGFB	0.354091	1.08E-09
B46	B222	RBC Count	NS	0.349375	1.85E-09
B46	B218	RBC Count	DBP	0.317728	5.50E-08
B46	B191	RBC Count	Urea	-0.29984	3.17E-07
B46	B70	RBC Count	LDL-C	0.290069	7.87E-07
B46	B126	RBC Count	Alb	0.289128	8.57E-07
B48	B53	LYM%	GR%	-0.94198	0
B5	B96	WB-Mg	dsDNA (IIF)	0.802896	0
B5	B36	WB-Mg	V-Lym%	0.380936	4.22E-11
B5	B167	WB-Mg	PC	0.367825	2.13E-10
B51	B174	Hb	Urea/Cr	-0.34296	3.79E-09
B51	B126	Hb	Alb	0.327038	2.11E-08
B51	B222	Hb	NS	0.324412	2.77E-08
B51	B221	Hb	Hei	0.314338	7.74E-08
B51	B128	Hb	ALT	0.311104	1.07E-07
B51	B218	Hb	DBP	0.311019	1.08E-07
B51	B141	Hb	AST/ALT	-0.29704	4.13E-07
B51	B189	Hb	eGFB	0.295381	4.82E-07
B52	B132	PLT/BPC	Dbil	-0.342	4.22E-09
B54	B100	ESR	anti-Ro52	0.495827	0
B54	B101	ESR	anti-Sc70	0.495827	0
B54	B102	ESR	anti-Sm	0.495827	0
B54	B103	ESR	anti-SSA/Ro	0.495827	0
B54	B104	ESR	anti-SSB	0.495827	0
B54	B105	ESR	anti-U1RNP	0.495827	0
B54	B106	ESR	anti-P	0.495827	0
B54	B107	ESR	AnuA	0.495827	0
B54	B108	ESR	Anti-Cyclin	0.495827	0
B54	B109	ESR	AMA-M2	0.495827	0
B54	B110	ESR	AHA	0.495827	0
B54	B124	ESR	ACTH	-0.49583	0
B54	B125	ESR	Cor	0.495827	0
B54	B181	ESR	APTT	0.495827	0
B54	B182	ESR	TT	-0.49567	0
B54	B183	ESR	PTA	0.495823	0
B54	B184	ESR	INR	0.495827	0
B54	B185	ESR	PT	0.495321	0
B54	B186	ESR	PFC	-0.49583	0

B54	B55	ESR	TK	0.586123	0
B54	B57	ESR	RBCAI	0.485173	0
B54	B59	ESR	WBRV	0.556399	0
B54	B60	ESR	WBRVI	0.512089	0
B54	B61	ESR	WBHSRV	0.538617	0
B54	B64	ESR	WBRSR(mPas_5)	0.527444	0
B54	B65	ESR	WBRSR(mPas_1)	0.529017	0
B54	B79	ESR	hbV-dna	0.495827	0
B54	B92	ESR	ALDH2-Glu504Glu	0.495827	0
B54	B93	ESR	ALDH2-Glu504Lys	-0.49583	0
B54	B94	ESR	ALDH2-Lys504Lys	0.495827	0
B54	B95	ESR	anti-cenpb	0.495827	0
B54	B97	ESR	dsDNA	0.495827	0
B54	B98	ESR	anti-Jo-1	0.495827	0
B54	B99	ESR	anti-PM-Scl	0.495827	0
B54	B56	ESR	IR	0.455914	8.88E-16
B54	B67	ESR	WBRSR(mPas_50)	0.439019	1.29E-14
B54	B69	ESR	PV	0.431432	4.04E-14
B54	B66	ESR	WBRSR(mPas_100)	0.401023	3.05E-12
B54	B194	ESR	CI	0.394606	7.20E-12
B54	B63	ESR	WBRSR(mPas_200)	0.389679	1.37E-11
B54	B134	ESR	CHE	-0.38162	3.87E-11
B54	B179	ESR	α1-MG	0.356638	8.04E-10
B54	B197	ESR	AG	-0.34931	1.86E-09
B54	B180	ESR	Tf	0.347396	2.31E-09
B54	B178	ESR	Ig-U	0.343232	3.68E-09
B55	B100	TK	anti-Ro52	0.501718	0
B55	B101	TK	anti-Sc70	0.501718	0
B55	B102	TK	anti-Sm	0.501718	0
B55	B103	TK	anti-SSA/Ro	0.501718	0
B55	B104	TK	anti-SSB	0.501718	0
B55	B105	TK	anti-U1RNP	0.501718	0
B55	B106	TK	anti-P	0.501718	0
B55	B107	TK	AnuA	0.501718	0
B55	B108	TK	Anti-Cyclin	0.501718	0
B55	B109	TK	AMA-M2	0.501718	0
B55	B110	TK	AHA	0.501718	0
B55	B124	TK	ACTH	-0.50172	0
B55	B125	TK	Cor	0.501718	0
B55	B181	TK	APTT	0.501718	0
B55	B182	TK	TT	-0.50161	0
B55	B183	TK	PTA	0.501716	0
B55	B184	TK	INR	0.501718	0
B55	B185	TK	PT	0.501306	0

B55	B186	TK	PFC	-0.50172	0
B55	B56	TK	IR	0.804792	0
B55	B57	TK	RBCAI	0.607399	0
B55	B59	TK	WBRV	0.701451	0
B55	B60	TK	WBRVI	0.726182	0
B55	B61	TK	WBHSRV	0.731167	0
B55	B62	TK	WBHSRVI	0.502569	0
B55	B64	TK	WBRSR(mPas_5)	0.663832	0
B55	B65	TK	WBRSR(mPas_1)	0.672237	0
B55	B66	TK	WBRSR(mPas_100)	0.494996	0
B55	B67	TK	WBRSR(mPas_50)	0.545568	0
B55	B79	TK	hbV-dna	0.501718	0
B55	B92	TK	ALDH2-Glu504Glu	0.501718	0
B55	B93	TK	ALDH2-Glu504Lys	-0.50172	0
B55	B94	TK	ALDH2-Lys504Lys	0.501718	0
B55	B95	TK	anti-cenpb	0.501718	0
B55	B97	TK	dsDNA	0.501718	0
B55	B98	TK	anti-Jo-1	0.501718	0
B55	B99	TK	anti-PM-Scl	0.501718	0
B55	B63	TK	WBRSR(mPas_200)	0.454335	1.11E-15
B55	B194	TK	Cl	0.407317	1.29E-12
B55	B179	TK	α1-MG	0.362663	3.96E-10
B55	B180	TK	Tf	0.353369	1.17E-09
B55	B178	TK	Ig-U	0.349176	1.89E-09
B55	B197	TK	AG	-0.3173	5.74E-08
B56	B58	IR	HCT	0.540861	0
B56	B59	IR	WBRV	0.544279	0
B56	B60	IR	WBRVI	0.689051	0
B56	B61	IR	WBHSRV	0.947507	0
B56	B62	IR	WBHSRVI	0.917159	0
B56	B63	IR	WBRSR(mPas_200)	0.858743	0
B56	B64	IR	WBRSR(mPas_5)	0.744933	0
B56	B65	IR	WBRSR(mPas_1)	0.648998	0
B56	B66	IR	WBRSR(mPas_100)	0.855832	0
B56	B67	IR	WBRSR(mPas_50)	0.851482	0
B56	B57	IR	RBCAI	0.386609	2.05E-11
B57	B59	RBCAI	WBRV	0.979235	0
B57	B60	RBCAI	WBRVI	0.925271	0
B57	B61	RBCAI	WBHSRV	0.516753	0
B57	B64	RBCAI	WBRSR(mPas_5)	0.841729	0
B57	B65	RBCAI	WBRSR(mPas_1)	0.928554	0
B57	B67	RBCAI	WBRSR(mPas_50)	0.533653	0
B57	B69	RBCAI	PV	0.490685	0
B57	B66	RBCAI	WBRSR(mPas_100)	0.420134	2.12E-13

B57	B63	RBCAI	WBRSR(mPas_200)	0.290649	7.46E-07
B58	B61	HCT	WBHSRV	0.548502	0
B58	B62	HCT	WBHSRVI	0.825157	0
B58	B63	HCT	WBRSR(mPas_200)	0.791246	0
B58	B66	HCT	WBRSR(mPas_100)	0.72401	0
B58	B67	HCT	WBRSR(mPas_50)	0.645415	0
B58	B64	HCT	WBRSR(mPas_5)	0.30206	2.57E-07
B59	B60	WBRV	WBRVI	0.97241	0
B59	B61	WBRV	WBHSRV	0.671367	0
B59	B64	WBRV	WBRSR(mPas_5)	0.920091	0
B59	B65	WBRV	WBRSR(mPas_1)	0.976095	0
B59	B66	WBRV	WBRSR(mPas_100)	0.569521	0
B59	B67	WBRV	WBRSR(mPas_50)	0.669803	0
B59	B69	WBRV	PV	0.532643	0
B59	B63	WBRV	WBRSR(mPas_200)	0.453576	1.33E-15
B59	B62	WBRV	WBHSRVI	0.332709	1.16E-08
B6	B167	WB-Fe	PC	0.745356	0
B6	B8	WB-Fe	WB-Zinc	-0.7746	0
B6	B96	WB-Fe	dsDNA (IIF)	0.650791	0
B60	B61	WBRVI	WBHSRV	0.787469	0
B60	B62	WBRVI	WBHSRVI	0.525773	0
B60	B63	WBRVI	WBRSR(mPas_200)	0.608836	0
B60	B64	WBRVI	WBRSR(mPas_5)	0.972042	0
B60	B65	WBRVI	WBRSR(mPas_1)	0.992859	0
B60	B66	WBRVI	WBRSR(mPas_100)	0.709654	0
B60	B67	WBRVI	WBRSR(mPas_50)	0.792962	0
B60	B69	WBRVI	PV	0.496335	0
B61	B62	WBHSRV	WBHSRVI	0.895667	0
B61	B63	WBHSRV	WBRSR(mPas_200)	0.934937	0
B61	B64	WBHSRV	WBRSR(mPas_5)	0.876001	0
B61	B65	WBHSRV	WBRSR(mPas_1)	0.783025	0
B61	B66	WBHSRV	WBRSR(mPas_100)	0.953067	0
B61	B67	WBHSRV	WBRSR(mPas_50)	0.96081	0
B61	B69	WBHSRV	PV	0.46712	0
B62	B63	WBHSRVI	WBRSR(mPas_200)	0.951452	0
B62	B64	WBHSRVI	WBRSR(mPas_5)	0.650411	0
B62	B65	WBHSRVI	WBRSR(mPas_1)	0.505226	0
B62	B66	WBHSRVI	WBRSR(mPas_100)	0.922524	0
B62	B67	WBHSRVI	WBRSR(mPas_50)	0.883256	0
B63	B64	WBRSR(mPas_200)	WBRSR(mPas_5)	0.759795	0
B63	B65	WBRSR(mPas_200)	WBRSR(mPas_1)	0.622145	0
B63	B66	WBRSR(mPas_200)	WBRSR(mPas_100)	0.988507	0
B63	B67	WBRSR(mPas_200)	WBRSR(mPas_50)	0.962291	0
B63	B69	WBRSR(mPas_200)	PV	0.524439	0

B64	B65	WBRSR(mPas_5)	WBRSR(mPas_1)	0.981623	0
B64	B66	WBRSR(mPas_5)	WBRSR(mPas_100)	0.842925	0
B64	B67	WBRSR(mPas_5)	WBRSR(mPas_50)	0.905252	0
B64	B69	WBRSR(mPas_5)	PV	0.624037	0
B65	B66	WBRSR(mPas_1)	WBRSR(mPas_100)	0.724895	0
B65	B67	WBRSR(mPas_1)	WBRSR(mPas_50)	0.807675	0
B65	B69	WBRSR(mPas_1)	PV	0.593781	0
B66	B67	WBRSR(mPas_100)	WBRSR(mPas_50)	0.991627	0
B66	B69	WBRSR(mPas_100)	PV	0.580898	0
B67	B69	WBRSR(mPas_50)	PV	0.610737	0
B7	B17	WB-Cop	CD3+CD16+CD56+NK	0.60404	0
B7	B29	WB-Cop	E2	0.472377	0
B70	B77	LDL-C	TC	0.833984	0
B71	B82	TG	CEA	0.631337	0
B71	B87	TG	NSE	0.500126	0
B71	B88	TG	CA 242	0.638937	0
B71	B90	TG	CYFRA 21-1	0.560132	0
B71	B153	TG	Glu	0.454584	1.11E-15
B71	B77	TG	TC	0.453829	1.33E-15
B71	B200	TG	OP	0.437363	1.64E-14
B71	B75	TG	ApoA1/B	0.435793	2.09E-14
B71	B139	TG	ADA	0.433517	2.93E-14
B71	B195	TG	Na	-0.40409	2.01E-12
B71	B85	TG	SCC	-0.34408	3.35E-09
B71	B170	TG	U-GLU	0.317934	5.39E-08
B71	B140	TG	r-GT	0.317719	5.51E-08
B72	B222	HDL-C	NS	-0.30335	2.27E-07
B72	B77	HDL-C	TC	0.300693	2.93E-07
B73	B76	ApoA	ApoB	0.432548	3.42E-14
B73	B74	ApoA	ApoA1	0.408813	1.05E-12
B73	B135	ApoA	TBA	-0.37755	6.46E-11
B73	B139	ApoA	ADA	-0.35927	5.91E-10
B73	B200	ApoA	OP	-0.35891	6.16E-10
B74	B76	ApoA1	ApoB	0.526752	0
B75	B76	ApoA1/B	ApoB	-0.62308	0
B75	B82	ApoA1/B	CEA	0.715016	0
B75	B88	ApoA1/B	CA 242	0.755482	0
B75	B90	ApoA1/B	CYFRA 21-1	0.46551	2.22E-16
B75	B87	ApoA1/B	NSE	0.429149	5.66E-14
B75	B195	ApoA1/B	Na	-0.42815	6.57E-14
B75	B200	ApoA1/B	OP	0.427737	6.97E-14
B75	B153	ApoA1/B	Glu	0.296258	4.44E-07
B76	B200	ApoB	OP	-0.44876	2.66E-15
B76	B82	ApoB	CEA	-0.39938	3.81E-12

B76	B88	ApoB	CA 242	-0.36106	4.78E-10
B76	B139	ApoB	ADA	-0.337	7.29E-09
B76	B90	ApoB	CYFRA 21-1	-0.33334	1.08E-08
B76	B137	ApoB	AFU	-0.32044	4.18E-08
B77	B132	TC	Dbil	-0.32053	4.14E-08
B77	B130	TC	GLB	0.307623	1.50E-07
B78	B134	HBcAb	CHE	-0.36985	1.67E-10
B78	B176	HBcAb	Ucr	0.365831	2.71E-10
B78	B113	HBcAb	FT3	0.339037	5.85E-09
B79	B101	hb V -dna	anti-Sc70	1	0
B79	B103	hb V -dna	anti-SSA/Ro	1	0
B79	B105	hb V -dna	anti-U1RNP	1	0
B79	B106	hb V -dna	anti-P	1	0
B79	B107	hb V -dna	AnuA	1	0
B79	B110	hb V -dna	AHA	1	0
B79	B124	hb V -dna	ACTH	-1	0
B79	B125	hb V -dna	Cor	1	0
B79	B151	hb V -dna	Anti-CCP	0.508198	0
B79	B158	hb V -dna	IgM	0.497089	0
B79	B178	hb V -dna	Ig-U	0.6975	0
B79	B179	hb V -dna	α1-MG	0.724317	0
B79	B180	hb V -dna	Tf	0.705838	0
B79	B182	hb V -dna	TT	-0.99982	0
B79	B183	hb V -dna	PTA	0.999999	0
B79	B185	hb V -dna	PT	0.999261	0
B79	B194	hb V -dna	Cl	0.804248	0
B79	B197	hb V -dna	AG	-0.67006	0
B79	B81	hb V -dna	TSGF	-0.54814	0
B79	B93	hb V -dna	ALDH2-Glu504Lys	-1	0
B79	B94	hb V -dna	ALDH2-Lys504Lys	1	0
B79	B97	hb V -dna	dsDNA	1	0
B79	B98	hb V -dna	anti-Jo-1	1	0
B79	B99	hb V -dna	anti-PM-Scl	1	0
B79	B138	hb V -dna	PAB	-0.41317	5.70E-13
B79	B137	hb V -dna	AFU	-0.28849	9.08E-07
B81	B100	TSGF	anti-Ro52	-0.54814	0
B81	B101	TSGF	anti-Sc70	-0.54814	0
B81	B102	TSGF	anti-Sm	-0.54814	0
B81	B103	TSGF	anti-SSA/Ro	-0.54814	0
B81	B104	TSGF	anti-SSB	-0.54814	0
B81	B105	TSGF	anti-U1RNP	-0.54814	0
B81	B106	TSGF	anti-P	-0.54814	0
B81	B107	TSGF	AnuA	-0.54814	0
B81	B108	TSGF	Anti-Cyclin	-0.54814	0

B81	B109	TSGF	AMA-M2	-0.54814	0
B81	B110	TSGF	AHA	-0.54814	0
B81	B124	TSGF	ACTH	0.548139	0
B81	B125	TSGF	Cor	-0.54814	0
B81	B181	TSGF	APTT	-0.54814	0
B81	B182	TSGF	TT	0.548017	0
B81	B183	TSGF	PTA	-0.54814	0
B81	B184	TSGF	INR	-0.54814	0
B81	B185	TSGF	PT	-0.54768	0
B81	B186	TSGF	PFC	0.548139	0
B81	B92	TSGF	ALDH2-Glu504Glu	-0.54814	0
B81	B93	TSGF	ALDH2-Glu504Lys	0.548139	0
B81	B94	TSGF	ALDH2-Lys504Lys	-0.54814	0
B81	B95	TSGF	anti-cenpb	-0.54814	0
B81	B97	TSGF	dsDNA	-0.54814	0
B81	B98	TSGF	anti-Jo-1	-0.54814	0
B81	B99	TSGF	anti-PM-Scl	-0.54814	0
B81	B194	TSGF	Cl	-0.43222	3.57E-14
B81	B179	TSGF	α1-MG	-0.39616	5.86E-12
B81	B180	TSGF	Tf	-0.386	2.21E-11
B81	B178	TSGF	Ig-U	-0.38142	3.97E-11
B81	B197	TSGF	AG	0.373521	1.06E-10
B81	B156	TSGF	IgE	-0.31286	8.96E-08
B82	B195	CEA	Na	-0.51874	0
B82	B200	CEA	OP	0.484182	0
B82	B87	CEA	NSE	0.599168	0
B82	B88	CEA	CA 242	0.858919	0
B82	B90	CEA	CYFRA 21-1	0.542552	0
B82	B139	CEA	ADA	0.44322	6.66E-15
B82	B86	CEA	TBhCG	-0.38146	3.95E-11
B82	B85	CEA	SCC	-0.37542	8.42E-11
B82	B198	CEA	P	-0.35544	9.23E-10
B82	B153	CEA	Glu	0.338824	5.98E-09
B84	B86	C-PSA	TBhCG	-0.35365	1.13E-09
B84	B165	C-PSA	bilirubin	0.32718	2.08E-08
B84	B89	C-PSA	CA 72-4	0.325591	2.45E-08
B85	B86	SCC	TBhCG	0.442602	7.33E-15
B85	B88	SCC	CA 242	-0.35147	1.46E-09
B85	B165	SCC	bilirubin	0.306871	1.62E-07
B86	B89	TBhCG	CA 72-4	-0.40344	2.20E-12
B86	B137	TBhCG	AFU	-0.31819	5.25E-08
B86	B200	TBhCG	OP	-0.29569	4.68E-07
B87	B88	NSE	CA 242	0.621019	0
B87	B90	NSE	CYFRA 21-1	0.591324	0

B87	B195	NSE	Na	-0.42952	5.35E-14
B87	B89	NSE	CA 72-4	0.42371	1.26E-13
B87	B139	NSE	ADA	0.414106	5.00E-13
B87	B200	NSE	OP	0.358038	6.83E-10
B88	B195	CA 242	Na	-0.52697	0
B88	B200	CA 242	OP	0.496184	0
B88	B90	CA 242	CYFRA 21-1	0.566104	0
B88	B139	CA 242	ADA	0.456174	8.88E-16
B88	B153	CA 242	Glu	0.350841	1.57E-09
B88	B198	CA 242	P	-0.3175	5.63E-08
B88	B137	CA 242	AFU	0.292215	6.46E-07
B89	B165	CA 72-4	bilirubin	0.899226	0
B89	B115	CA 72-4	TSH TT3 TT4(TT3)	-0.43619	1.95E-14
B89	B113	CA 72-4	FT3	-0.40306	2.31E-12
B89	B90	CA 72-4	CYFRA 21-1	0.326614	2.21E-08
B9	B29	RH	E2	-0.54545	0
B9	B31	RH	FSH	-0.74574	0
B9	B33	RH	Prog	-0.40234	2.55E-12
B90	B195	CYFRA 21-1	Na	-0.39667	5.47E-12
B90	B139	CYFRA 21-1	ADA	0.381761	3.80E-11
B90	B200	CYFRA 21-1	OP	0.375982	7.85E-11
B90	B194	CYFRA 21-1	Cl	-0.34712	2.39E-09
B91	B127	TP	A-G ratio	-0.52419	0
B91	B130	TP	GLB	0.790798	0
B91	B126	TP	Alb	0.424231	1.17E-13
B92	B101	ALDH2-Glu504Glu	anti-Sc70	1	0
B92	B103	ALDH2-Glu504Glu	anti-SSA/Ro	1	0
B92	B105	ALDH2-Glu504Glu	anti-U1RNP	1	0
B92	B106	ALDH2-Glu504Glu	anti-P	1	0
B92	B107	ALDH2-Glu504Glu	AnuA	1	0
B92	B110	ALDH2-Glu504Glu	AHA	1	0
B92	B151	ALDH2-Glu504Glu	Anti-CCP	0.508198	0
B92	B158	ALDH2-Glu504Glu	IgM	0.497089	0
B92	B178	ALDH2-Glu504Glu	Ig-U	0.6975	0
B92	B179	ALDH2-Glu504Glu	α1-MG	0.724317	0
B92	B180	ALDH2-Glu504Glu	Tf	0.705838	0
B92	B182	ALDH2-Glu504Glu	TT	-0.99982	0
B92	B183	ALDH2-Glu504Glu	PTA	0.999999	0
B92	B185	ALDH2-Glu504Glu	PT	0.999261	0
B92	B194	ALDH2-Glu504Glu	Cl	0.804248	0
B92	B197	ALDH2-Glu504Glu	AG	-0.67006	0
B92	B94	ALDH2-Glu504Glu	ALDH2-Lys504Lys	1	0
B92	B97	ALDH2-Glu504Glu	dsDNA	1	0
B92	B98	ALDH2-Glu504Glu	anti-Jo-1	1	0

B92	B99	ALDH2-Glu504Glu	anti-PM-Scl	1	0
B92	B138	ALDH2-Glu504Glu	PAB	-0.41317	5.70E-13
B92	B137	ALDH2-Glu504Glu	AFU	-0.28849	9.08E-07
B93	B101	ALDH2-Glu504Lys	anti-Sc70	-1	0
B93	B103	ALDH2-Glu504Lys	anti-SSA/Ro	-1	0
B93	B105	ALDH2-Glu504Lys	anti-U1RNP	-1	0
B93	B106	ALDH2-Glu504Lys	anti-P	-1	0
B93	B107	ALDH2-Glu504Lys	AnuA	-1	0
B93	B110	ALDH2-Glu504Lys	AHA	-1	0
B93	B151	ALDH2-Glu504Lys	Anti-CCP	-0.5082	0
B93	B158	ALDH2-Glu504Lys	IgM	-0.49709	0
B93	B178	ALDH2-Glu504Lys	Ig-U	-0.6975	0
B93	B179	ALDH2-Glu504Lys	α1-MG	-0.72432	0
B93	B180	ALDH2-Glu504Lys	Tf	-0.70584	0
B93	B182	ALDH2-Glu504Lys	TT	0.999822	0
B93	B183	ALDH2-Glu504Lys	PTA	-1	0
B93	B184	ALDH2-Glu504Lys	INR	-1	0
B93	B185	ALDH2-Glu504Lys	PT	-0.99926	0
B93	B194	ALDH2-Glu504Lys	CI	-0.80425	0
B93	B197	ALDH2-Glu504Lys	AG	0.670061	0
B93	B94	ALDH2-Glu504Lys	ALDH2-Lys504Lys	-1	0
B93	B97	ALDH2-Glu504Lys	dsDNA	-1	0
B93	B98	ALDH2-Glu504Lys	anti-Jo-1	-1	0
B93	B99	ALDH2-Glu504Lys	anti-PM-Scl	-1	0
B93	B138	ALDH2-Glu504Lys	PAB	0.413173	5.70E-13
B93	B137	ALDH2-Glu504Lys	AFU	0.288494	9.08E-07
B94	B100	ALDH2-Lys504Lys	anti-Ro52	1	0
B94	B101	ALDH2-Lys504Lys	anti-Sc70	1	0
B94	B103	ALDH2-Lys504Lys	anti-SSA/Ro	1	0
B94	B105	ALDH2-Lys504Lys	anti-U1RNP	1	0
B94	B106	ALDH2-Lys504Lys	anti-P	1	0
B94	B107	ALDH2-Lys504Lys	AnuA	1	0
B94	B109	ALDH2-Lys504Lys	AMA-M2	1	0
B94	B110	ALDH2-Lys504Lys	AHA	1	0
B94	B124	ALDH2-Lys504Lys	ACTH	-1	0
B94	B125	ALDH2-Lys504Lys	Cor	1	0
B94	B151	ALDH2-Lys504Lys	Anti-CCP	0.508198	0
B94	B158	ALDH2-Lys504Lys	IgM	0.497089	0
B94	B178	ALDH2-Lys504Lys	Ig-U	0.6975	0
B94	B179	ALDH2-Lys504Lys	α1-MG	0.724317	0
B94	B180	ALDH2-Lys504Lys	Tf	0.705838	0
B94	B181	ALDH2-Lys504Lys	APTT	1	0
B94	B182	ALDH2-Lys504Lys	TT	-0.99982	0
B94	B183	ALDH2-Lys504Lys	PTA	0.999999	0

B94	B184	ALDH2-Lys504Lys	INR	1	0
B94	B185	ALDH2-Lys504Lys	PT	0.999261	0
B94	B194	ALDH2-Lys504Lys	CI	0.804248	0
B94	B197	ALDH2-Lys504Lys	AG	-0.67006	0
B94	B95	ALDH2-Lys504Lys	anti-cenpb	1	0
B94	B97	ALDH2-Lys504Lys	dsDNA	1	0
B94	B98	ALDH2-Lys504Lys	anti-Jo-1	1	0
B94	B99	ALDH2-Lys504Lys	anti-PM-Scl	1	0
B94	B138	ALDH2-Lys504Lys	PAB	-0.41317	5.70E-13
B94	B137	ALDH2-Lys504Lys	AFU	-0.28849	9.08E-07
B95	B101	anti-cenpb	anti-Sc70	1	0
B95	B106	anti-cenpb	anti-P	1	0
B95	B107	anti-cenpb	AnuA	1	0
B95	B109	anti-cenpb	AMA-M2	1	0
B95	B151	anti-cenpb	Anti-CCP	0.508198	0
B95	B158	anti-cenpb	IgM	0.497089	0
B95	B178	anti-cenpb	Ig-U	0.6975	0
B95	B179	anti-cenpb	α1-MG	0.724317	0
B95	B180	anti-cenpb	Tf	0.705838	0
B95	B182	anti-cenpb	TT	-0.99982	0
B95	B183	anti-cenpb	PTA	0.999999	0
B95	B185	anti-cenpb	PT	0.999261	0
B95	B194	anti-cenpb	CI	0.804248	0
B95	B197	anti-cenpb	AG	-0.67006	0
B95	B98	anti-cenpb	anti-Jo-1	1	0
B95	B138	anti-cenpb	PAB	-0.41317	5.70E-13
B95	B137	anti-cenpb	AFU	-0.28849	9.08E-07
B96	B167	dsDNA (IIF)	PC	0.781504	0
B97	B100	dsDNA	anti-Ro52	1	0
B97	B103	dsDNA	anti-SSA/Ro	1	0
B97	B105	dsDNA	anti-U1RNP	1	0
B97	B106	dsDNA	anti-P	1	0
B97	B107	dsDNA	AnuA	1	0
B97	B108	dsDNA	Anti-Cyclin	1	0
B97	B109	dsDNA	AMA-M2	1	0
B97	B110	dsDNA	AHA	1	0
B97	B124	dsDNA	ACTH	-1	0
B97	B125	dsDNA	Cor	1	0
B97	B151	dsDNA	Anti-CCP	0.508198	0
B97	B158	dsDNA	IgM	0.497089	0
B97	B178	dsDNA	Ig-U	0.6975	0
B97	B179	dsDNA	α1-MG	0.724317	0
B97	B180	dsDNA	Tf	0.705838	0
B97	B181	dsDNA	APTT	1	0

B97	B182	dsDNA	TT	-0.99982	0
B97	B183	dsDNA	PTA	0.999999	0
B97	B184	dsDNA	INR	1	0
B97	B185	dsDNA	PT	0.999261	0
B97	B186	dsDNA	PFC	-1	0
B97	B194	dsDNA	CI	0.804248	0
B97	B197	dsDNA	AG	-0.67006	0
B97	B138	dsDNA	PAB	-0.41317	5.70E-13
B97	B137	dsDNA	AFU	-0.28849	9.08E-07
B98	B100	anti-Jo-1	anti-Ro52	1	0
B98	B101	anti-Jo-1	anti-Sc70	1	0
B98	B102	anti-Jo-1	anti-Sm	1	0
B98	B103	anti-Jo-1	anti-SSA/Ro	1	0
B98	B104	anti-Jo-1	anti-SSB	1	0
B98	B105	anti-Jo-1	anti-U1RNP	1	0
B98	B107	anti-Jo-1	AnuA	1	0
B98	B108	anti-Jo-1	Anti-Cyclin	1	0
B98	B109	anti-Jo-1	AMA-M2	1	0
B98	B110	anti-Jo-1	AHA	1	0
B98	B124	anti-Jo-1	ACTH	-1	0
B98	B125	anti-Jo-1	Cor	1	0
B98	B151	anti-Jo-1	Anti-CCP	0.508198	0
B98	B158	anti-Jo-1	IgM	0.497089	0
B98	B178	anti-Jo-1	Ig-U	0.6975	0
B98	B179	anti-Jo-1	α1-MG	0.724317	0
B98	B180	anti-Jo-1	Tf	0.705838	0
B98	B181	anti-Jo-1	APTT	1	0
B98	B182	anti-Jo-1	TT	-0.99982	0
B98	B183	anti-Jo-1	PTA	0.999999	0
B98	B184	anti-Jo-1	INR	1	0
B98	B185	anti-Jo-1	PT	0.999261	0
B98	B186	anti-Jo-1	PFC	-1	0
B98	B194	anti-Jo-1	CI	0.804248	0
B98	B197	anti-Jo-1	AG	-0.67006	0
B98	B99	anti-Jo-1	anti-PM-Scl	1	0
B98	B138	anti-Jo-1	PAB	-0.41317	5.70E-13
B98	B137	anti-Jo-1	AFU	-0.28849	9.08E-07
B99	B100	anti-PM-Scl	anti-Ro52	1	0
B99	B103	anti-PM-Scl	anti-SSA/Ro	1	0
B99	B105	anti-PM-Scl	anti-U1RNP	1	0
B99	B107	anti-PM-Scl	AnuA	1	0
B99	B108	anti-PM-Scl	Anti-Cyclin	1	0
B99	B109	anti-PM-Scl	AMA-M2	1	0
B99	B110	anti-PM-Scl	AHA	1	0

B99	B124	anti-PM-Scl	ACTH	-1	0
B99	B125	anti-PM-Scl	Cor	1	0
B99	B151	anti-PM-Scl	Anti-CCP	0.508198	0
B99	B158	anti-PM-Scl	IgM	0.497089	0
B99	B178	anti-PM-Scl	Ig-U	0.6975	0
B99	B179	anti-PM-Scl	α1-MG	0.724317	0
B99	B180	anti-PM-Scl	Tf	0.705838	0
B99	B181	anti-PM-Scl	APTT	1	0
B99	B182	anti-PM-Scl	TT	-0.99982	0
B99	B183	anti-PM-Scl	PTA	0.999999	0
B99	B184	anti-PM-Scl	INR	1	0
B99	B185	anti-PM-Scl	PT	0.999261	0
B99	B186	anti-PM-Scl	PFC	-1	0
B99	B194	anti-PM-Scl	Cl	0.804248	0
B99	B197	anti-PM-Scl	AG	-0.67006	0
B99	B138	anti-PM-Scl	PAB	-0.41317	5.70E-13
B99	B137	anti-PM-Scl	AFU	-0.28849	9.08E-07

Supplementary Table 10. PEI Correlations in Rhinallergosis

Row	Column	Row	Column	Cor	*P*
B1	B190	Gender	Cre	-0.730474206	0
B1	B192	Gender	UA	-0.672096816	0
B1	B206	Gender	Waist	-0.632182121	0
B1	B207	Gender	WHR	-0.642272664	0
B1	B216	Gender	BMI	-0.656737407	0
B1	B221	Gender	Hei	-0.645853302	0
B1	B222	Gender	NS	-0.775250534	0
B1	B51	Gender	Hb	-0.653142121	0
B1	B72	Gender	HDL-C	0.520393778	3.33E-12
B1	B46	Gender	RBC Count	-0.486547047	1.20E-10
B1	B215	Gender	BMI	-0.424098683	3.45E-08
B1	B210	Gender	Hip	-0.416884126	6.18E-08
B1	B220	Gender	SBP	-0.400924434	2.14E-07
B1	B141	Gender	AST/ALT	0.396517966	2.99E-07
B1	B128	Gender	ALT	-0.385850465	6.54E-07
B1	B163	Gender	SG	-0.384058046	7.44E-07
B1	B204	Gender	Drinking	-0.381626539	8.86E-07
B10	B14	PG I	G-17	-0.576591808	3.33E-15
B10	B11	PG I	PGI/PGII	0.515479843	5.74E-12
B100	B101	anti-Ro52	anti-Sc70	0.999999998	0
B100	B103	anti-Ro52	anti-SSA/Ro	1	0
B100	B105	anti-Ro52	anti-U1RNP	1	0
B100	B106	anti-Ro52	anti-P	1	0
B100	B109	anti-Ro52	AMA-M2	1	0
B100	B151	anti-Ro52	Anti-CCP	0.7048221	0
B100	B156	anti-Ro52	IgE	-0.903553226	0
B100	B183	anti-Ro52	PTA	-0.935351263	0
B100	B155	anti-Ro52	IgA	0.55642635	4.64E-14
B100	B175	anti-Ro52	ALB-U	0.538122697	4.33E-13
B100	B158	anti-Ro52	IgM	-0.492296831	6.70E-11
B100	B198	anti-Ro52	P	-0.484721524	1.44E-10
B100	B185	anti-Ro52	PT	0.471649437	5.14E-10
B100	B184	anti-Ro52	INR	0.461953764	1.28E-09
B100	B181	anti-Ro52	APTT	0.459306489	1.63E-09
B100	B168	anti-Ro52	NMP22	0.401609664	2.03E-07
B100	B157	anti-Ro52	IgG	0.400110805	2.28E-07
B100	B176	anti-Ro52	Ucr	0.387593593	5.77E-07
B101	B102	anti-Sc70	anti-Sm	0.999999998	0
B101	B103	anti-Sc70	anti-SSA/Ro	0.999999997	0
B101	B104	anti-Sc70	anti-SSB	0.999999997	0
B101	B105	anti-Sc70	anti-U1RNP	0.999999999	0
B101	B106	anti-Sc70	anti-P	0.999999996	0

B101	B108	anti-Sc70	Anti-Cyclin	0.999999998	0
B101	B109	anti-Sc70	AMA-M2	0.999999998	0
B101	B110	anti-Sc70	AHA	0.999999998	0
B101	B151	anti-Sc70	Anti-CCP	0.704822098	0
B101	B156	anti-Sc70	IgE	-0.903553223	0
B101	B183	anti-Sc70	PTA	-0.93535126	0
B101	B155	anti-Sc70	IgA	0.556426348	4.64E-14
B101	B175	anti-Sc70	ALB-U	0.538122696	4.33E-13
B101	B158	anti-Sc70	IgM	-0.49229683	6.70E-11
B101	B198	anti-Sc70	P	-0.484721523	1.44E-10
B101	B185	anti-Sc70	PT	0.471649436	5.14E-10
B101	B184	anti-Sc70	INR	0.461953763	1.28E-09
B101	B181	anti-Sc70	APTT	0.459306487	1.63E-09
B101	B168	anti-Sc70	NMP22	0.401609665	2.03E-07
B101	B157	anti-Sc70	IgG	0.400110804	2.28E-07
B101	B176	anti-Sc70	Ucr	0.387593591	5.77E-07
B102	B103	anti-Sm	anti-SSA/Ro	0.999999999	0
B102	B104	anti-Sm	anti-SSB	0.999999998	0
B102	B106	anti-Sm	anti-P	0.999999999	0
B102	B108	anti-Sm	Anti-Cyclin	0.999999999	0
B102	B109	anti-Sm	AMA-M2	1	0
B102	B151	anti-Sm	Anti-CCP	0.7048221	0
B102	B156	anti-Sm	IgE	-0.903553226	0
B102	B183	anti-Sm	PTA	-0.935351263	0
B102	B155	anti-Sm	IgA	0.55642635	4.64E-14
B102	B175	anti-Sm	ALB-U	0.538122697	4.33E-13
B102	B158	anti-Sm	IgM	-0.492296831	6.70E-11
B102	B198	anti-Sm	P	-0.484721524	1.44E-10
B102	B185	anti-Sm	PT	0.471649436	5.14E-10
B102	B184	anti-Sm	INR	0.461953764	1.28E-09
B102	B181	anti-Sm	APTT	0.459306489	1.63E-09
B102	B168	anti-Sm	NMP22	0.401609664	2.03E-07
B102	B157	anti-Sm	IgG	0.400110805	2.28E-07
B102	B176	anti-Sm	Ucr	0.387593592	5.77E-07
B103	B105	anti-SSA/Ro	anti-U1RNP	1	0
B103	B109	anti-SSA/Ro	AMA-M2	1	0
B103	B110	anti-SSA/Ro	AHA	1	0
B103	B151	anti-SSA/Ro	Anti-CCP	0.7048221	0
B103	B156	anti-SSA/Ro	IgE	-0.903553226	0
B103	B183	anti-SSA/Ro	PTA	-0.935351263	0
B103	B155	anti-SSA/Ro	IgA	0.55642635	4.64E-14
B103	B175	anti-SSA/Ro	ALB-U	0.538122697	4.33E-13
B103	B158	anti-SSA/Ro	IgM	-0.492296831	6.70E-11
B103	B198	anti-SSA/Ro	P	-0.484721524	1.44E-10

B103	B185	anti-SSA/Ro	PT	0.471649437	5.14E-10
B103	B184	anti-SSA/Ro	INR	0.461953764	1.28E-09
B103	B181	anti-SSA/Ro	APTT	0.459306489	1.63E-09
B103	B168	anti-SSA/Ro	NMP22	0.401609664	2.03E-07
B103	B157	anti-SSA/Ro	IgG	0.400110805	2.28E-07
B103	B176	anti-SSA/Ro	Ucr	0.387593593	5.77E-07
B104	B151	anti-SSB	Anti-CCP	0.704822102	0
B104	B156	anti-SSB	IgE	-0.903553229	0
B104	B183	anti-SSB	PTA	-0.935351266	0
B104	B155	anti-SSB	IgA	0.556426351	4.64E-14
B104	B175	anti-SSB	ALB-U	0.538122699	4.33E-13
B104	B158	anti-SSB	IgM	-0.492296833	6.69E-11
B104	B198	anti-SSB	P	-0.484721525	1.44E-10
B104	B185	anti-SSB	PT	0.471649437	5.14E-10
B104	B184	anti-SSB	INR	0.461953765	1.28E-09
B104	B181	anti-SSB	APTT	0.45930649	1.63E-09
B104	B168	anti-SSB	NMP22	0.401609664	2.03E-07
B104	B157	anti-SSB	IgG	0.400110806	2.28E-07
B104	B176	anti-SSB	Ucr	0.387593594	5.77E-07
B105	B110	anti-U1RNP	AHA	1	0
B105	B151	anti-U1RNP	Anti-CCP	0.7048221	0
B105	B156	anti-U1RNP	IgE	-0.903553226	0
B105	B183	anti-U1RNP	PTA	-0.935351263	0
B105	B155	anti-U1RNP	IgA	0.55642635	4.64E-14
B105	B175	anti-U1RNP	ALB-U	0.538122697	4.33E-13
B105	B158	anti-U1RNP	IgM	-0.492296831	6.70E-11
B105	B198	anti-U1RNP	P	-0.484721524	1.44E-10
B105	B185	anti-U1RNP	PT	0.471649436	5.14E-10
B105	B184	anti-U1RNP	INR	0.461953764	1.28E-09
B105	B181	anti-U1RNP	APTT	0.459306489	1.63E-09
B105	B168	anti-U1RNP	NMP22	0.401609665	2.03E-07
B105	B157	anti-U1RNP	IgG	0.400110805	2.28E-07
B105	B176	anti-U1RNP	Ucr	0.387593592	5.77E-07
B106	B108	anti-P	Anti-Cyclin	1	0
B106	B110	anti-P	AHA	1	0
B106	B151	anti-P	Anti-CCP	0.7048221	0
B106	B156	anti-P	IgE	-0.903553227	0
B106	B183	anti-P	PTA	-0.935351264	0
B106	B155	anti-P	IgA	0.55642635	4.64E-14
B106	B175	anti-P	ALB-U	0.538122698	4.33E-13
B106	B158	anti-P	IgM	-0.492296832	6.70E-11
B106	B198	anti-P	P	-0.484721524	1.44E-10
B106	B185	anti-P	PT	0.471649437	5.14E-10
B106	B184	anti-P	INR	0.461953764	1.28E-09

B106	B181	anti-P	APTT	0.459306489	1.63E-09
B106	B168	anti-P	NMP22	0.401609665	2.03E-07
B106	B157	anti-P	IgG	0.400110805	2.28E-07
B106	B176	anti-P	Ucr	0.387593593	5.77E-07
B107	B151	AnuA	Anti-CCP	0.704822115	0
B107	B156	AnuA	IgE	-0.903553245	0
B107	B183	AnuA	PTA	-0.935351283	0
B107	B155	AnuA	IgA	0.556426361	4.64E-14
B107	B175	AnuA	ALB-U	0.538122709	4.33E-13
B107	B158	AnuA	IgM	-0.492296842	6.69E-11
B107	B198	AnuA	P	-0.484721535	1.44E-10
B107	B185	AnuA	PT	0.471649445	5.14E-10
B107	B184	AnuA	INR	0.461953777	1.28E-09
B107	B181	AnuA	APTT	0.459306499	1.63E-09
B107	B168	AnuA	NMP22	0.401609685	2.03E-07
B107	B157	AnuA	IgG	0.400110814	2.28E-07
B107	B176	AnuA	Ucr	0.387593601	5.77E-07
B108	B151	Anti-Cyclin	Anti-CCP	0.7048221	0
B108	B156	Anti-Cyclin	IgE	-0.903553226	0
B108	B183	Anti-Cyclin	PTA	-0.935351263	0
B108	B155	Anti-Cyclin	IgA	0.55642635	4.64E-14
B108	B175	Anti-Cyclin	ALB-U	0.538122697	4.33E-13
B108	B158	Anti-Cyclin	IgM	-0.492296831	6.70E-11
B108	B198	Anti-Cyclin	P	-0.484721524	1.44E-10
B108	B185	Anti-Cyclin	PT	0.471649436	5.14E-10
B108	B184	Anti-Cyclin	INR	0.461953764	1.28E-09
B108	B181	Anti-Cyclin	APTT	0.459306489	1.63E-09
B108	B168	Anti-Cyclin	NMP22	0.401609665	2.03E-07
B108	B157	Anti-Cyclin	IgG	0.400110805	2.28E-07
B108	B176	Anti-Cyclin	Ucr	0.387593593	5.77E-07
B109	B110	AMA-M2	AHA	1	0
B109	B151	AMA-M2	Anti-CCP	0.7048221	0
B109	B156	AMA-M2	IgE	-0.903553226	0
B109	B183	AMA-M2	PTA	-0.935351263	0
B109	B155	AMA-M2	IgA	0.55642635	4.64E-14
B109	B175	AMA-M2	ALB-U	0.538122697	4.33E-13
B109	B158	AMA-M2	IgM	-0.492296831	6.70E-11
B109	B198	AMA-M2	P	-0.484721524	1.44E-10
B109	B185	AMA-M2	PT	0.471649437	5.14E-10
B109	B184	AMA-M2	INR	0.461953764	1.28E-09
B109	B181	AMA-M2	APTT	0.459306489	1.63E-09
B109	B168	AMA-M2	NMP22	0.401609665	2.03E-07
B109	B157	AMA-M2	IgG	0.400110805	2.28E-07
B109	B176	AMA-M2	Ucr	0.387593592	5.77E-07

B11	B14	PGI/PGII	G-17	-0.674597955	0
B11	B12	PGI/PGII	PG II	-0.489670856	8.74E-11
B110	B151	AHA	Anti-CCP	0.7048221	0
B110	B156	AHA	IgE	-0.903553226	0
B110	B183	AHA	PTA	-0.935351263	0
B110	B155	AHA	IgA	0.55642635	4.64E-14
B110	B175	AHA	ALB-U	0.538122697	4.33E-13
B110	B158	AHA	IgM	-0.492296831	6.70E-11
B110	B198	AHA	P	-0.484721524	1.44E-10
B110	B185	AHA	PT	0.471649437	5.14E-10
B110	B184	AHA	INR	0.461953764	1.28E-09
B110	B181	AHA	APTT	0.459306489	1.63E-09
B110	B168	AHA	NMP22	0.401609665	2.03E-07
B110	B157	AHA	IgG	0.400110805	2.28E-07
B110	B176	AHA	Ucr	0.387593592	5.77E-07
B111	B148	TSH	Anti_TPO	0.87003874	0
B111	B149	TSH	TGAb	0.816950733	0
B112	B114	FT4	TSH TT3 TT4(TT4)	0.437362613	1.14E-08
B113	B115	FT3	TSH TT3 TT4(TT3)	0.466435778	8.41E-10
B114	B193	TSH TT3 TT4(TT4)	K	-0.588727142	6.66E-16
B114	B134	TSH TT3 TT4(TT4)	CHE	0.536951247	4.98E-13
B114	B185	TSH TT3 TT4(TT4)	PT	-0.485109177	1.38E-10
B114	B181	TSH TT3 TT4(TT4)	APTT	-0.436845608	1.19E-08
B115	B134	TSH TT3 TT4(TT3)	CHE	0.456541437	2.10E-09
B115	B152	TSH TT3 TT4(TT3)	ACA-Ig(G-A-M)	-0.42028932	4.70E-08
B115	B194	TSH TT3 TT4(TT3)	Cl	-0.400591138	2.20E-07
B115	B131	TSH TT3 TT4(TT3)	AST	0.399243822	2.43E-07
B115	B197	TSH TT3 TT4(TT3)	AG	0.398188601	2.63E-07
B116	B124	TT3	ACTH	0.513552591	7.10E-12
B117	B125	TT4	Cor	-0.914137926	0
B117	B202	TT4	e-times	-0.593913871	4.44E-16
B117	B180	TT4	Tf	-0.476042484	3.37E-10
B119	B121	Hcv-rna	C3	-0.999999984	0
B119	B169	Hcv-rna	U-CRY	-0.99999944	0
B119	B179	Hcv-rna	α1-MG	-0.999999966	0
B121	B169	C3	U-CRY	0.999999432	0
B122	B159	C4	β2-MG	0.999999731	0
B122	B169	C4	U-CRY	0.999999715	0
B123	B156	hs-CRP1	IgE	-0.38988918	4.88E-07
B125	B223	Cor	FH	-0.534296709	6.80E-13
B125	B178	Cor	Ig-U	-0.451446949	3.32E-09
B126	B127	Alb	A-G ratio	0.545800046	1.73E-13
B127	B130	A-G ratio	GLB	-0.918735946	0
B128	B131	ALT	AST	0.701116767	0

B128	B141	ALT	AST/ALT	-0.654655193	0
B128	B216	ALT	BMI	0.609555706	0
B128	B215	ALT	BMI	0.59309447	4.44E-16
B128	B206	ALT	Waist	0.545366428	1.82E-13
B128	B210	ALT	Hip	0.525381598	1.90E-12
B128	B222	ALT	NS	0.513252177	7.33E-12
B128	B140	ALT	r-GT	0.423615491	3.59E-08
B128	B207	ALT	WHR	0.413649219	7.99E-08
B129	B132	IBIL	Dbil	0.679156728	0
B129	B133	IBIL	TBIL	0.976858959	0
B131	B215	AST	BMI	0.444540491	6.11E-09
B131	B206	AST	Waist	0.419988119	4.82E-08
B131	B216	AST	BMI	0.406567872	1.39E-07
B131	B207	AST	WHR	0.401877387	1.99E-07
B132	B133	Dbil	TBIL	0.820341578	0
B134	B193	CHE	K	-0.692059016	0
B134	B136	CHE	5'NT	0.387006046	6.02E-07
B135	B136	TBA	5'NT	-0.6808099	0
B135	B196	TBA	TCO2	0.647416217	0
B135	B198	TBA	P	-0.649487971	0
B135	B137	TBA	AFU	0.53834308	4.22E-13
B135	B194	TBA	Cl	-0.533768098	7.23E-13
B135	B139	TBA	ADA	0.52284771	2.53E-12
B136	B138	5'NT	PAB	0.519188303	3.81E-12
B136	B196	5'NT	TCO2	-0.411191733	9.70E-08
B137	B139	AFU	ADA	0.727550678	0
B137	B194	AFU	Cl	-0.799829167	0
B137	B198	AFU	P	-0.586990126	8.88E-16
B137	B196	AFU	TCO2	0.584532779	1.11E-15
B137	B200	AFU	OP	0.525971846	1.78E-12
B137	B197	AFU	AG	0.465415351	9.25E-10
B138	B150	PAB	ALP	0.412286239	8.90E-08
B138	B197	PAB	AG	0.391090098	4.47E-07
B139	B198	ADA	P	-0.557346717	4.13E-14
B139	B194	ADA	Cl	-0.556894487	4.37E-14
B139	B196	ADA	TCO2	0.415506736	6.90E-08
B14	B44	G-17	MCH	-0.412427117	8.80E-08
B141	B216	AST/ALT	BMI	-0.481976085	1.89E-10
B141	B210	AST/ALT	Hip	-0.460257138	1.49E-09
B141	B222	AST/ALT	NS	-0.451137336	3.41E-09
B141	B215	AST/ALT	BMI	-0.449099606	4.09E-09
B141	B206	AST/ALT	Waist	-0.419433405	5.04E-08
B142	B143	25-(OH)D	β-CTx	0.999999291	0
B142	B151	25-(OH)D	Anti-CCP	-0.7048221	0

B142	B168	25-(OH)D	NMP22	-0.401609664	2.03E-07
B143	B144	β-CTx	Total-PINP	0.999999291	0
B143	B151	β-CTx	Anti-CCP	-0.704816154	0
B143	B168	β-CTx	NMP22	-0.40159333	2.04E-07
B144	B151	Total-PINP	Anti-CCP	-0.7048221	0
B144	B168	Total-PINP	NMP22	-0.401609664	2.03E-07
B145	B171	BGP	EC	0.843661488	0
B146	B194	CysC	Cl	-0.397608531	2.75E-07
B148	B149	Anti_TPO	TGAb	0.960804957	0
B148	B214	Anti_TPO	CVA-L	-0.38989287	4.88E-07
B15	B100	CD3-CD19+(B cell)	anti-Ro52	1	0
B15	B101	CD3-CD19+(B cell)	anti-Sc70	0.999999997	0
B15	B102	CD3-CD19+(B cell)	anti-Sm	0.999999999	0
B15	B105	CD3-CD19+(B cell)	anti-U1RNP	1	0
B15	B109	CD3-CD19+(B cell)	AMA-M2	1	0
B15	B110	CD3-CD19+(B cell)	AHA	1	0
B15	B151	CD3-CD19+(B cell)	Anti-CCP	0.7048221	0
B15	B156	CD3-CD19+(B cell)	IgE	-0.903553226	0
B15	B16	CD3-CD19+(B cell)	CD3+T	-1	0
B15	B18	CD3-CD19+(B cell)	CD3+CD4+helper(inducer)T	-1	0
B15	B183	CD3-CD19+(B cell)	PTA	-0.935351263	0
B15	B19	CD3-CD19+(B cell)	CD3+CD4+/CD3+CD8+	-1	0
B15	B20	CD3-CD19+(B cell)	CD3+CD4+CD8+	1	0
B15	B57	CD3-CD19+(B cell)	RBCAI	0.673254212	0
B15	B90	CD3-CD19+(B cell)	CYFRA 21-1	-0.720217082	0
B15	B98	CD3-CD19+(B cell)	anti-Jo-1	0.999999997	0
B15	B99	CD3-CD19+(B cell)	anti-PM-Scl	0.999999999	0
B15	B25	CD3-CD19+(B cell)	CK	0.5935606	4.44E-16
B15	B59	CD3-CD19+(B cell)	WBRV	0.571774066	6.44E-15
B15	B155	CD3-CD19+(B cell)	IgA	0.55642635	4.64E-14
B15	B175	CD3-CD19+(B cell)	ALB-U	0.538122697	4.33E-13
B15	B158	CD3-CD19+(B cell)	IgM	-0.492296831	6.70E-11
B15	B60	CD3-CD19+(B cell)	WBRVI	0.490164144	8.32E-11
B15	B54	CD3-CD19+(B cell)	ESR	0.486953983	1.15E-10
B15	B198	CD3-CD19+(B cell)	P	-0.484721524	1.44E-10
B15	B185	CD3-CD19+(B cell)	PT	0.471649437	5.14E-10
B15	B184	CD3-CD19+(B cell)	INR	0.461953764	1.28E-09
B15	B181	CD3-CD19+(B cell)	APTT	0.459306489	1.63E-09
B15	B65	CD3-CD19+(B cell)	WBRSR(mPas_1)	0.450002865	3.78E-09
B15	B58	CD3-CD19+(B cell)	HCT	0.423424837	3.64E-08
B15	B168	CD3-CD19+(B cell)	NMP22	0.401609664	2.03E-07
B15	B157	CD3-CD19+(B cell)	IgG	0.400110805	2.28E-07
B15	B176	CD3-CD19+(B cell)	Ucr	0.387593593	5.77E-07
B150	B197	ALP	AG	0.444918983	5.91E-09

B150	B194	ALP	Cl	-0.419423234	5.04E-08
B151	B156	Anti-CCP	IgE	-0.636306618	0
B151	B183	Anti-CCP	PTA	-0.66156937	0
B151	B175	Anti-CCP	ALB-U	0.594784271	2.22E-16
B151	B168	Anti-CCP	NMP22	0.569802512	8.44E-15
B151	B155	Anti-CCP	IgA	0.386941597	6.04E-07
B152	B184	ACA-Ig(G-A-M)	INR	0.613320015	0
B153	B220	Glu	SBP	0.420870213	4.48E-08
B154	B157	RF	IgG	0.585357938	8.88E-16
B155	B156	IgA	IgE	-0.69776581	0
B155	B183	IgA	PTA	-0.5503322	9.90E-14
B156	B183	IgE	PTA	0.868039722	0
B156	B181	IgE	APTT	-0.494514492	5.34E-11
B156	B185	IgE	PT	-0.492402198	6.62E-11
B156	B175	IgE	ALB-U	-0.484538328	1.46E-10
B156	B198	IgE	P	0.438501072	1.03E-08
B156	B184	IgE	INR	-0.437642311	1.11E-08
B157	B158	IgG	IgM	-0.730819563	0
B157	B182	IgG	TT	-0.467473771	7.63E-10
B157	B186	IgG	PFC	0.443192413	6.88E-09
B158	B183	IgM	PTA	0.538267316	4.26E-13
B158	B181	IgM	APTT	-0.509191177	1.14E-11
B158	B185	IgM	PT	-0.474476901	3.92E-10
B159	B169	β2-MG	U-CRY	0.999999092	0
B16	B100	CD3+T	anti-Ro52	-1	0
B16	B101	CD3+T	anti-Sc70	-0.999999996	0
B16	B102	CD3+T	anti-Sm	-0.999999999	0
B16	B103	CD3+T	anti-SSA/Ro	-1	0
B16	B105	CD3+T	anti-U1RNP	-1	0
B16	B109	CD3+T	AMA-M2	-1	0
B16	B110	CD3+T	AHA	-1	0
B16	B151	CD3+T	Anti-CCP	-0.7048221	0
B16	B156	CD3+T	IgE	0.903553226	0
B16	B17	CD3+T	CD3+CD16+CD56+NK	-1	0
B16	B18	CD3+T	CD3+CD4+helper(inducer)T	1	0
B16	B183	CD3+T	PTA	0.935351263	0
B16	B19	CD3+T	CD3+CD4+/CD3+CD8+	1	0
B16	B20	CD3+T	CD3+CD4+CD8+	-1	0
B16	B21	CD3+T	CD3+CD4-CD8-	-1	0
B16	B23	CD3+T	CD3-CD16+CD56+(NK)	-1	0
B16	B34	CD3+T	TK	-1	0
B16	B57	CD3+T	RBCAI	-0.673254212	0
B16	B90	CD3+T	CYFRA 21-1	0.720217082	0
B16	B98	CD3+T	anti-Jo-1	-0.999999997	0

B16	B99	CD3+T	anti-PM-Scl	-0.999999999	0
B16	B25	CD3+T	CK	-0.5935606	4.44E-16
B16	B59	CD3+T	WBRV	-0.571774066	6.44E-15
B16	B155	CD3+T	IgA	-0.55642635	4.64E-14
B16	B175	CD3+T	ALB-U	-0.538122697	4.33E-13
B16	B158	CD3+T	IgM	0.492296831	6.70E-11
B16	B60	CD3+T	WBRVI	-0.490164144	8.32E-11
B16	B54	CD3+T	ESR	-0.486953983	1.15E-10
B16	B198	CD3+T	P	0.484721524	1.44E-10
B16	B185	CD3+T	PT	-0.471649437	5.14E-10
B16	B184	CD3+T	INR	-0.461953764	1.28E-09
B16	B181	CD3+T	APTT	-0.459306489	1.63E-09
B16	B65	CD3+T	WBRSR(mPas_1)	-0.450002865	3.78E-09
B16	B58	CD3+T	HCT	-0.423424837	3.64E-08
B16	B168	CD3+T	NMP22	-0.401609664	2.03E-07
B16	B157	CD3+T	IgG	-0.400110805	2.28E-07
B16	B176	CD3+T	Ucr	-0.387593593	5.77E-07
B163	B166	SG	Pro	0.442841356	7.09E-09
B168	B200	NMP22	OP	0.535140384	6.16E-13
B168	B195	NMP22	Na	0.480593065	2.16E-10
B168	B183	NMP22	PTA	-0.38241168	8.37E-07
B169	B179	U-CRY	α1-MG	0.999999915	0
B169	B187	U-CRY	NSE	-0.999999748	0
B17	B100	CD3+CD16+CD56+NK	anti-Ro52	1	0
B17	B101	CD3+CD16+CD56+NK	anti-Sc70	0.999999997	0
B17	B102	CD3+CD16+CD56+NK	anti-Sm	1	0
B17	B105	CD3+CD16+CD56+NK	anti-U1RNP	1	0
B17	B109	CD3+CD16+CD56+NK	AMA-M2	1	0
B17	B110	CD3+CD16+CD56+NK	AHA	1	0
B17	B151	CD3+CD16+CD56+NK	Anti-CCP	0.7048221	0
B17	B156	CD3+CD16+CD56+NK	IgE	-0.903553226	0
B17	B183	CD3+CD16+CD56+NK	PTA	-0.935351263	0
B17	B19	CD3+CD16+CD56+NK	CD3+CD4+/CD3+CD8+	-1	0
B17	B21	CD3+CD16+CD56+NK	CD3+CD4-CD8-	1	0
B17	B57	CD3+CD16+CD56+NK	RBCAI	0.673254212	0
B17	B90	CD3+CD16+CD56+NK	CYFRA 21-1	-0.720217082	0
B17	B98	CD3+CD16+CD56+NK	anti-Jo-1	0.999999997	0
B17	B99	CD3+CD16+CD56+NK	anti-PM-Scl	0.999999999	0
B17	B25	CD3+CD16+CD56+NK	CK	0.5935606	4.44E-16
B17	B59	CD3+CD16+CD56+NK	WBRV	0.571774066	6.44E-15
B17	B155	CD3+CD16+CD56+NK	IgA	0.55642635	4.64E-14
B17	B175	CD3+CD16+CD56+NK	ALB-U	0.538122697	4.33E-13
B17	B158	CD3+CD16+CD56+NK	IgM	-0.492296831	6.70E-11
B17	B60	CD3+CD16+CD56+NK	WBRVI	0.490164144	8.32E-11

B17	B54	CD3+CD16+CD56+NK	ESR	0.486953983	1.15E-10
B17	B198	CD3+CD16+CD56+NK	P	-0.484721524	1.44E-10
B17	B185	CD3+CD16+CD56+NK	PT	0.471649437	5.14E-10
B17	B184	CD3+CD16+CD56+NK	INR	0.461953764	1.28E-09
B17	B181	CD3+CD16+CD56+NK	APTT	0.459306489	1.63E-09
B17	B65	CD3+CD16+CD56+NK	WBRSR(mPas_1)	0.450002865	3.78E-09
B17	B58	CD3+CD16+CD56+NK	HCT	0.423424837	3.64E-08
B17	B168	CD3+CD16+CD56+NK	NMP22	0.401609664	2.03E-07
B17	B157	CD3+CD16+CD56+NK	IgG	0.400110805	2.28E-07
B17	B176	CD3+CD16+CD56+NK	Ucr	0.387593593	5.77E-07
B170	B173	U-GLU	NTT	1	0
B174	B191	Urea/Cr	Urea	0.654289272	0
B174	B190	Urea/Cr	Cre	-0.398592719	2.56E-07
B175	B183	ALB-U	PTA	-0.510579374	9.80E-12
B175	B198	ALB-U	P	-0.382053618	8.59E-07
B178	B180	Ig-U	Tf	-0.783645156	0
B18	B100	CD3+CD4+helper(inducer)T	anti-Ro52	-1	0
B18	B101	CD3+CD4+helper(inducer)T	anti-Sc70	-0.999999997	0
B18	B102	CD3+CD4+helper(inducer)T	anti-Sm	-0.999999999	0
B18	B105	CD3+CD4+helper(inducer)T	anti-U1RNP	-1	0
B18	B109	CD3+CD4+helper(inducer)T	AMA-M2	-1	0
B18	B110	CD3+CD4+helper(inducer)T	AHA	-1	0
B18	B151	CD3+CD4+helper(inducer)T	Anti-CCP	-0.7048221	0
B18	B156	CD3+CD4+helper(inducer)T	IgE	0.903553226	0
B18	B183	CD3+CD4+helper(inducer)T	PTA	0.935351263	0
B18	B19	CD3+CD4+helper(inducer)T	CD3+CD4+/CD3+CD8+	1	0
B18	B20	CD3+CD4+helper(inducer)T	CD3+CD4+CD8+	-1	0
B18	B23	CD3+CD4+helper(inducer)T	CD3-CD16+CD56+(NK)	-1	0
B18	B34	CD3+CD4+helper(inducer)T	TK	-1	0
B18	B57	CD3+CD4+helper(inducer)T	RBCAI	-0.673254212	0
B18	B90	CD3+CD4+helper(inducer)T	CYFRA 21-1	0.720217082	0
B18	B98	CD3+CD4+helper(inducer)T	anti-Jo-1	-0.999999997	0
B18	B99	CD3+CD4+helper(inducer)T	anti-PM-Scl	-0.999999999	0
B18	B25	CD3+CD4+helper(inducer)T	CK	-0.5935606	4.44E-16
B18	B59	CD3+CD4+helper(inducer)T	WBRV	-0.571774066	6.44E-15
B18	B155	CD3+CD4+helper(inducer)T	IgA	-0.55642635	4.64E-14
B18	B175	CD3+CD4+helper(inducer)T	ALB-U	-0.538122697	4.33E-13
B18	B158	CD3+CD4+helper(inducer)T	IgM	0.492296831	6.70E-11
B18	B60	CD3+CD4+helper(inducer)T	WBRVI	-0.490164144	8.32E-11
B18	B54	CD3+CD4+helper(inducer)T	ESR	-0.486953983	1.15E-10
B18	B198	CD3+CD4+helper(inducer)T	P	0.484721524	1.44E-10
B18	B185	CD3+CD4+helper(inducer)T	PT	-0.471649437	5.14E-10
B18	B184	CD3+CD4+helper(inducer)T	INR	-0.461953764	1.28E-09
B18	B181	CD3+CD4+helper(inducer)T	APTT	-0.459306489	1.63E-09

B18	B65	CD3+CD4+helper(inducer)T	WBRSR(mPas_1)	-0.450002865	3.78E-09
B18	B58	CD3+CD4+helper(inducer)T	HCT	-0.423424837	3.64E-08
B18	B168	CD3+CD4+helper(inducer)T	NMP22	-0.401609664	2.03E-07
B18	B157	CD3+CD4+helper(inducer)T	IgG	-0.400110805	2.28E-07
B18	B176	CD3+CD4+helper(inducer)T	Ucr	-0.387593593	5.77E-07
B180	B223	Tf	FH	-0.620684829	0
B180	B225	Tf	hPBG	-0.784381436	0
B181	B182	APTT	TT	0.666986375	0
B181	B183	APTT	PTA	-0.642674629	0
B181	B185	APTT	PT	0.897972248	0
B181	B186	APTT	PFC	-0.615454491	0
B181	B184	APTT	INR	0.459067764	1.67E-09
B182	B186	TT	PFC	-0.990845633	0
B182	B185	TT	PT	0.384800228	7.06E-07
B183	B185	PTA	PT	-0.512121503	8.29E-12
B183	B198	PTA	P	0.451107418	3.42E-09
B184	B185	INR	PT	0.797196839	0
B189	B190	eGFB	Cre	-0.641678937	0
B189	B219	eGFB	OT	-0.458575056	1.74E-09
B19	B100	CD3+CD4+/CD3+CD8+	anti-Ro52	-1	0
B19	B101	CD3+CD4+/CD3+CD8+	anti-Sc70	-0.999999997	0
B19	B102	CD3+CD4+/CD3+CD8+	anti-Sm	-0.999999999	0
B19	B103	CD3+CD4+/CD3+CD8+	anti-SSA/Ro	-1	0
B19	B105	CD3+CD4+/CD3+CD8+	anti-U1RNP	-1	0
B19	B109	CD3+CD4+/CD3+CD8+	AMA-M2	-1	0
B19	B110	CD3+CD4+/CD3+CD8+	AHA	-1	0
B19	B151	CD3+CD4+/CD3+CD8+	Anti-CCP	-0.7048221	0
B19	B156	CD3+CD4+/CD3+CD8+	IgE	0.903553226	0
B19	B183	CD3+CD4+/CD3+CD8+	PTA	0.935351263	0
B19	B20	CD3+CD4+/CD3+CD8+	CD3+CD4+CD8+	-1	0
B19	B21	CD3+CD4+/CD3+CD8+	CD3+CD4-CD8-	-1	0
B19	B23	CD3+CD4+/CD3+CD8+	CD3-CD16+CD56+(NK)	-1	0
B19	B34	CD3+CD4+/CD3+CD8+	TK	-1	0
B19	B57	CD3+CD4+/CD3+CD8+	RBCAI	-0.673254212	0
B19	B90	CD3+CD4+/CD3+CD8+	CYFRA 21-1	0.720217082	0
B19	B98	CD3+CD4+/CD3+CD8+	anti-Jo-1	-0.999999997	0
B19	B99	CD3+CD4+/CD3+CD8+	anti-PM-Scl	-0.999999999	0
B19	B25	CD3+CD4+/CD3+CD8+	CK	-0.5935606	4.44E-16
B19	B59	CD3+CD4+/CD3+CD8+	WBRV	-0.571774066	6.44E-15
B19	B155	CD3+CD4+/CD3+CD8+	IgA	-0.55642635	4.64E-14
B19	B175	CD3+CD4+/CD3+CD8+	ALB-U	-0.538122697	4.33E-13
B19	B158	CD3+CD4+/CD3+CD8+	IgM	0.492296831	6.70E-11
B19	B60	CD3+CD4+/CD3+CD8+	WBRVI	-0.490164144	8.32E-11
B19	B54	CD3+CD4+/CD3+CD8+	ESR	-0.486953983	1.15E-10

B19	B198	CD3+CD4+/CD3+CD8+	P	0.484721524	1.44E-10
B19	B185	CD3+CD4+/CD3+CD8+	PT	-0.471649437	5.14E-10
B19	B184	CD3+CD4+/CD3+CD8+	INR	-0.461953764	1.28E-09
B19	B181	CD3+CD4+/CD3+CD8+	APTT	-0.459306489	1.63E-09
B19	B65	CD3+CD4+/CD3+CD8+	WBRSR(mPas_1)	-0.450002865	3.78E-09
B19	B58	CD3+CD4+/CD3+CD8+	HCT	-0.423424837	3.64E-08
B19	B168	CD3+CD4+/CD3+CD8+	NMP22	-0.401609664	2.03E-07
B19	B157	CD3+CD4+/CD3+CD8+	IgG	-0.400110805	2.28E-07
B19	B176	CD3+CD4+/CD3+CD8+	Ucr	-0.387593593	5.77E-07
B190	B192	Cre	UA	0.582511213	1.55E-15
B190	B222	Cre	NS	0.54748259	1.41E-13
B190	B221	Cre	Hei	0.449827544	3.84E-09
B190	B216	Cre	BMI	0.431898953	1.81E-08
B190	B207	Cre	WHR	0.416023127	6.62E-08
B190	B191	Cre	Urea	0.407081471	1.34E-07
B190	B206	Cre	Waist	0.399064822	2.47E-07
B192	B222	UA	NS	0.583597208	1.33E-15
B192	B216	UA	BMI	0.579396181	2.22E-15
B192	B206	UA	Waist	0.551924177	8.13E-14
B192	B221	UA	Hei	0.495853452	4.65E-11
B192	B207	UA	WHR	0.488180308	1.02E-10
B192	B210	UA	Hip	0.449990516	3.78E-09
B192	B215	UA	BMI	0.423567698	3.60E-08
B194	B196	Cl	TCO2	-0.572970007	5.33E-15
B194	B197	Cl	AG	-0.517715404	4.49E-12
B194	B198	Cl	P	0.51761687	4.54E-12
B195	B200	Na	OP	0.861881501	0
B195	B197	Na	AG	0.533047188	7.86E-13
B196	B198	TCO2	P	-0.87937918	0
B196	B200	TCO2	OP	0.418356505	5.49E-08
B197	B200	AG	OP	0.589632778	4.44E-16
B198	B200	P	OP	-0.389341278	5.08E-07
B2	B189	Age	eGFB	-0.660327006	0
B2	B219	Age	OT	0.48143245	1.99E-10
B2	B153	Age	Glu	0.47314488	4.45E-10
B2	B207	Age	WHR	0.446938084	4.95E-09
B20	B100	CD3+CD4+CD8+	anti-Ro52	1	0
B20	B101	CD3+CD4+CD8+	anti-Sc70	0.999999997	0
B20	B102	CD3+CD4+CD8+	anti-Sm	1	0
B20	B105	CD3+CD4+CD8+	anti-U1RNP	1	0
B20	B109	CD3+CD4+CD8+	AMA-M2	1	0
B20	B110	CD3+CD4+CD8+	AHA	1	0
B20	B151	CD3+CD4+CD8+	Anti-CCP	0.7048221	0
B20	B156	CD3+CD4+CD8+	IgE	-0.903553226	0

B20	B183	CD3+CD4+CD8+	PTA	-0.935351263	0
B20	B34	CD3+CD4+CD8+	TK	1	0
B20	B57	CD3+CD4+CD8+	RBCAI	0.673254212	0
B20	B90	CD3+CD4+CD8+	CYFRA 21-1	-0.720217082	0
B20	B98	CD3+CD4+CD8+	anti-Jo-1	0.999999997	0
B20	B99	CD3+CD4+CD8+	anti-PM-Scl	0.999999999	0
B20	B25	CD3+CD4+CD8+	CK	0.5935606	4.44E-16
B20	B59	CD3+CD4+CD8+	WBRV	0.571774066	6.44E-15
B20	B155	CD3+CD4+CD8+	IgA	0.55642635	4.64E-14
B20	B175	CD3+CD4+CD8+	ALB-U	0.538122697	4.33E-13
B20	B158	CD3+CD4+CD8+	IgM	-0.492296831	6.70E-11
B20	B60	CD3+CD4+CD8+	WBRVI	0.490164144	8.32E-11
B20	B54	CD3+CD4+CD8+	ESR	0.486953983	1.15E-10
B20	B198	CD3+CD4+CD8+	P	-0.484721524	1.44E-10
B20	B185	CD3+CD4+CD8+	PT	0.471649437	5.14E-10
B20	B184	CD3+CD4+CD8+	INR	0.461953764	1.28E-09
B20	B181	CD3+CD4+CD8+	APTT	0.459306489	1.63E-09
B20	B65	CD3+CD4+CD8+	WBRSR(mPas_1)	0.450002865	3.78E-09
B20	B58	CD3+CD4+CD8+	HCT	0.423424837	3.64E-08
B20	B168	CD3+CD4+CD8+	NMP22	0.401609664	2.03E-07
B20	B157	CD3+CD4+CD8+	IgG	0.400110805	2.28E-07
B20	B176	CD3+CD4+CD8+	Ucr	0.387593593	5.77E-07
B202	B225	e-times	hPBG	-0.873326063	0
B202	B223	e-times	FH	-0.431244346	1.91E-08
B204	B209	Drinking	Smoking	0.442334442	7.41E-09
B206	B207	Waist	WHR	0.874619494	0
B206	B210	Waist	Hip	0.792914657	0
B206	B215	Waist	BMI	0.855027013	0
B206	B216	Waist	BMI	0.867953182	0
B206	B222	Waist	NS	0.770658776	0
B206	B221	Waist	Hei	0.448362523	4.37E-09
B207	B215	WHR	BMI	0.668817718	0
B207	B216	WHR	BMI	0.652972857	0
B207	B222	WHR	NS	0.68318672	0
B207	B210	WHR	Hip	0.452264295	3.09E-09
B21	B100	CD3+CD4-CD8-	anti-Ro52	1	0
B21	B101	CD3+CD4-CD8-	anti-Sc70	0.999999997	0
B21	B102	CD3+CD4-CD8-	anti-Sm	0.999999999	0
B21	B103	CD3+CD4-CD8-	anti-SSA/Ro	1	0
B21	B105	CD3+CD4-CD8-	anti-U1RNP	1	0
B21	B109	CD3+CD4-CD8-	AMA-M2	1	0
B21	B110	CD3+CD4-CD8-	AHA	1	0
B21	B151	CD3+CD4-CD8-	Anti-CCP	0.7048221	0
B21	B156	CD3+CD4-CD8-	IgE	-0.903553226	0

B21	B183	CD3+CD4-CD8-	PTA	-0.935351263	0
B21	B23	CD3+CD4-CD8-	CD3-CD16+CD56+(NK)	1	0
B21	B57	CD3+CD4-CD8-	RBCAI	0.673254212	0
B21	B90	CD3+CD4-CD8-	CYFRA 21-1	-0.720217082	0
B21	B98	CD3+CD4-CD8-	anti-Jo-1	0.999999997	0
B21	B99	CD3+CD4-CD8-	anti-PM-Scl	0.999999999	0
B21	B25	CD3+CD4-CD8-	CK	0.5935606	4.44E-16
B21	B59	CD3+CD4-CD8-	WBRV	0.571774066	6.44E-15
B21	B155	CD3+CD4-CD8-	IgA	0.55642635	4.64E-14
B21	B175	CD3+CD4-CD8-	ALB-U	0.538122697	4.33E-13
B21	B158	CD3+CD4-CD8-	IgM	-0.492296831	6.70E-11
B21	B60	CD3+CD4-CD8-	WBRVI	0.490164144	8.32E-11
B21	B54	CD3+CD4-CD8-	ESR	0.486953983	1.15E-10
B21	B198	CD3+CD4-CD8-	P	-0.484721524	1.44E-10
B21	B185	CD3+CD4-CD8-	PT	0.471649437	5.14E-10
B21	B184	CD3+CD4-CD8-	INR	0.461953764	1.28E-09
B21	B181	CD3+CD4-CD8-	APTT	0.459306489	1.63E-09
B21	B65	CD3+CD4-CD8-	WBRSR(mPas_1)	0.450002865	3.78E-09
B21	B58	CD3+CD4-CD8-	HCT	0.423424837	3.64E-08
B21	B168	CD3+CD4-CD8-	NMP22	0.401609664	2.03E-07
B21	B157	CD3+CD4-CD8-	IgG	0.400110805	2.28E-07
B21	B176	CD3+CD4-CD8-	Ucr	0.387593593	5.77E-07
B210	B215	Hip	BMI	0.776312553	0
B210	B216	Hip	BMI	0.82466008	0
B210	B222	Hip	NS	0.656636781	0
B210	B221	Hip	Hei	0.473952504	4.12E-10
B211	B212	IOP-R	IOP-L	0.871437527	0
B213	B214	CVA-R	CVA-L	0.723999171	0
B215	B216	BMI	BMI	0.832590375	0
B215	B222	BMI	NS	0.659158344	0
B216	B221	BMI	Hei	0.70400546	0
B216	B222	BMI	NS	0.794399592	0
B218	B220	DBP	SBP	0.734518467	0
B22	B101	CD3+CD8+(T)	anti-Sc70	0.999999997	0
B22	B102	CD3+CD8+(T)	anti-Sm	0.999999999	0
B22	B105	CD3+CD8+(T)	anti-U1RNP	1	0
B22	B106	CD3+CD8+(T)	anti-P	1	0
B22	B109	CD3+CD8+(T)	AMA-M2	1	0
B22	B151	CD3+CD8+(T)	Anti-CCP	0.7048221	0
B22	B156	CD3+CD8+(T)	IgE	-0.903553226	0
B22	B183	CD3+CD8+(T)	PTA	-0.935351263	0
B22	B57	CD3+CD8+(T)	RBCAI	0.673254212	0
B22	B90	CD3+CD8+(T)	CYFRA 21-1	-0.720217082	0
B22	B98	CD3+CD8+(T)	anti-Jo-1	0.999999997	0

B22	B99	CD3+CD8+(T)	anti-PM-Scl	0.999999999	0
B22	B25	CD3+CD8+(T)	CK	0.5935606	4.44E-16
B22	B59	CD3+CD8+(T)	WBRV	0.571774066	6.44E-15
B22	B155	CD3+CD8+(T)	IgA	0.55642635	4.64E-14
B22	B175	CD3+CD8+(T)	ALB-U	0.538122697	4.33E-13
B22	B158	CD3+CD8+(T)	IgM	-0.492296831	6.70E-11
B22	B60	CD3+CD8+(T)	WBRVI	0.490164144	8.32E-11
B22	B54	CD3+CD8+(T)	ESR	0.486953983	1.15E-10
B22	B198	CD3+CD8+(T)	P	-0.484721524	1.44E-10
B22	B185	CD3+CD8+(T)	PT	0.471649437	5.14E-10
B22	B184	CD3+CD8+(T)	INR	0.461953764	1.28E-09
B22	B181	CD3+CD8+(T)	APTT	0.459306489	1.63E-09
B22	B65	CD3+CD8+(T)	WBRSR(mPas_1)	0.450002865	3.78E-09
B22	B58	CD3+CD8+(T)	HCT	0.423424837	3.64E-08
B22	B168	CD3+CD8+(T)	NMP22	0.401609665	2.03E-07
B22	B157	CD3+CD8+(T)	IgG	0.400110805	2.28E-07
B22	B176	CD3+CD8+(T)	Ucr	0.387593593	5.77E-07
B221	B222	Hei	NS	0.578653593	2.66E-15
B227	B228	OD	OS	0.719740153	0
B23	B100	CD3-CD16+CD56+(NK)	anti-Ro52	1	0
B23	B101	CD3-CD16+CD56+(NK)	anti-Sc70	0.999999997	0
B23	B102	CD3-CD16+CD56+(NK)	anti-Sm	0.999999999	0
B23	B105	CD3-CD16+CD56+(NK)	anti-U1RNP	1	0
B23	B109	CD3-CD16+CD56+(NK)	AMA-M2	1	0
B23	B110	CD3-CD16+CD56+(NK)	AHA	1	0
B23	B151	CD3-CD16+CD56+(NK)	Anti-CCP	0.7048221	0
B23	B156	CD3-CD16+CD56+(NK)	IgE	-0.903553226	0
B23	B183	CD3-CD16+CD56+(NK)	PTA	-0.935351263	0
B23	B57	CD3-CD16+CD56+(NK)	RBCAI	0.673254212	0
B23	B90	CD3-CD16+CD56+(NK)	CYFRA 21-1	-0.720217082	0
B23	B98	CD3-CD16+CD56+(NK)	anti-Jo-1	0.999999997	0
B23	B99	CD3-CD16+CD56+(NK)	anti-PM-Scl	0.999999999	0
B23	B25	CD3-CD16+CD56+(NK)	CK	0.5935606	4.44E-16
B23	B59	CD3-CD16+CD56+(NK)	WBRV	0.571774066	6.44E-15
B23	B155	CD3-CD16+CD56+(NK)	IgA	0.55642635	4.64E-14
B23	B175	CD3-CD16+CD56+(NK)	ALB-U	0.538122697	4.33E-13
B23	B158	CD3-CD16+CD56+(NK)	IgM	-0.492296831	6.70E-11
B23	B60	CD3-CD16+CD56+(NK)	WBRVI	0.490164144	8.32E-11
B23	B54	CD3-CD16+CD56+(NK)	ESR	0.486953983	1.15E-10
B23	B198	CD3-CD16+CD56+(NK)	P	-0.484721524	1.44E-10
B23	B185	CD3-CD16+CD56+(NK)	PT	0.471649437	5.14E-10
B23	B184	CD3-CD16+CD56+(NK)	INR	0.461953764	1.28E-09
B23	B181	CD3-CD16+CD56+(NK)	APTT	0.459306489	1.63E-09
B23	B65	CD3-CD16+CD56+(NK)	WBRSR(mPas_1)	0.450002865	3.78E-09

B23	B58	CD3-CD16+CD56+(NK)	HCT	0.423424837	3.64E-08
B23	B168	CD3-CD16+CD56+(NK)	NMP22	0.401609664	2.03E-07
B23	B157	CD3-CD16+CD56+(NK)	IgG	0.400110805	2.28E-07
B23	B176	CD3-CD16+CD56+(NK)	Ucr	0.387593593	5.77E-07
B24	B26	αHBDH	CKMB	0.932085975	0
B24	B27	αHBDH	LDH	0.623270507	0
B24	B146	αHBDH	CysC	0.505558108	1.68E-11
B25	B100	CK	anti-Ro52	0.5935606	4.44E-16
B25	B101	CK	anti-Sc70	0.593560598	4.44E-16
B25	B102	CK	anti-Sm	0.5935606	4.44E-16
B25	B103	CK	anti-SSA/Ro	0.5935606	4.44E-16
B25	B104	CK	anti-SSB	0.593560601	4.44E-16
B25	B105	CK	anti-U1RNP	0.5935606	4.44E-16
B25	B106	CK	anti-P	0.5935606	4.44E-16
B25	B107	CK	AnuA	0.593560613	4.44E-16
B25	B108	CK	Anti-Cyclin	0.5935606	4.44E-16
B25	B109	CK	AMA-M2	0.5935606	4.44E-16
B25	B110	CK	AHA	0.5935606	4.44E-16
B25	B34	CK	TK	0.5935606	4.44E-16
B25	B95	CK	anti-cenpb	0.5935606	4.44E-16
B25	B97	CK	dsDNA	0.5935606	4.44E-16
B25	B98	CK	anti-Jo-1	0.593560598	4.44E-16
B25	B99	CK	anti-PM-Scl	0.593560599	4.44E-16
B25	B176	CK	Ucr	0.556651349	4.51E-14
B25	B183	CK	PTA	-0.555299892	5.33E-14
B25	B199	CK	Mg	0.485772964	1.29E-10
B25	B81	CK	TSGF	0.465386889	9.28E-10
B25	B156	CK	IgE	-0.442492479	7.31E-09
B25	B60	CK	WBRVI	0.420243874	4.72E-08
B25	B151	CK	Anti-CCP	0.418196062	5.56E-08
B25	B69	CK	PV	-0.415127572	7.11E-08
B25	B155	CK	IgA	0.392225399	4.11E-07
B25	B146	CK	CysC	0.385339417	6.79E-07
B26	B27	CKMB	LDH	0.622732428	0
B26	B146	CKMB	CysC	0.4809585	2.08E-10
B27	B146	LDH	CysC	0.544013853	2.14E-13
B28	B117	PRL	TT4	-0.777777778	0
B28	B180	PRL	Tf	-0.72659116	0
B28	B92	PRL	ALDH2-Glu504Glu	-0.905821627	0
B28	B202	PRL	e-times	-0.564218177	1.73E-14
B28	B178	PRL	Ig-U	-0.486533633	1.20E-10
B28	B225	PRL	hPBG	-0.45677344	2.05E-09
B29	B125	E2	Cor	-0.745601135	0
B29	B178	E2	Ig-U	-0.911407491	0

B29	B202	E2	e-times	-0.637193093	0
B29	B223	E2	FH	-0.603451429	0
B29	B225	E2	hPBG	-0.837707817	0
B29	B117	E2	TT4	-0.529812943	1.14E-12
B3	B26	Hcy	CKMB	0.604655101	0
B3	B146	Hcy	CysC	0.59009587	4.44E-16
B3	B84	Hcy	C-PSA	0.581424821	1.78E-15
B3	B24	Hcy	αHBDH	0.530540732	1.05E-12
B3	B88	Hcy	CA 242	-0.514337787	6.51E-12
B3	B89	Hcy	CA 72-4	0.512285366	8.15E-12
B3	B168	Hcy	NMP22	0.473860973	4.16E-10
B3	B82	Hcy	CEA	-0.414121816	7.70E-08
B3	B100	Hcy	anti-Ro52	0.399542724	2.38E-07
B3	B101	Hcy	anti-Sc70	0.399542723	2.38E-07
B3	B102	Hcy	anti-Sm	0.399542724	2.38E-07
B3	B103	Hcy	anti-SSA/Ro	0.399542724	2.38E-07
B3	B104	Hcy	anti-SSB	0.399542725	2.38E-07
B3	B105	Hcy	anti-U1RNP	0.399542724	2.38E-07
B3	B106	Hcy	anti-P	0.399542724	2.38E-07
B3	B107	Hcy	AnuA	0.399542733	2.38E-07
B3	B108	Hcy	Anti-Cyclin	0.399542724	2.38E-07
B3	B109	Hcy	AMA-M2	0.399542724	2.38E-07
B3	B110	Hcy	AHA	0.399542724	2.38E-07
B3	B15	Hcy	CD3-CD19+(B cell)	0.399542724	2.38E-07
B3	B16	Hcy	CD3+T	-0.399542724	2.38E-07
B3	B17	Hcy	CD3+CD16+CD56+NK	0.399542724	2.38E-07
B3	B18	Hcy	CD3+CD4+helper(inducer)T	-0.399542724	2.38E-07
B3	B19	Hcy	CD3+CD4+/CD3+CD8+	-0.399542724	2.38E-07
B3	B20	Hcy	CD3+CD4+CD8+	0.399542724	2.38E-07
B3	B21	Hcy	CD3+CD4-CD8-	0.399542724	2.38E-07
B3	B22	Hcy	CD3+CD8+(T)	0.399542724	2.38E-07
B3	B23	Hcy	CD3-CD16+CD56+(NK)	0.399542724	2.38E-07
B3	B34	Hcy	TK	0.399542724	2.38E-07
B3	B95	Hcy	anti-cenpb	0.399542725	2.38E-07
B3	B97	Hcy	dsDNA	0.399542724	2.38E-07
B3	B98	Hcy	anti-Jo-1	0.399542723	2.38E-07
B3	B99	Hcy	anti-PM-Scl	0.399542724	2.38E-07
B3	B27	Hcy	LDH	0.39547999	3.23E-07
B30	B145	LH	BGP	0.89597867	0
B30	B171	LH	EC	0.666972969	0
B30	B35	LH	β2-MG	0.688247202	0
B30	B38	LH	BAND%	0.79381612	0
B30	B94	LH	ALDH2-Lys504Lys	0.535303379	6.04E-13
B30	B33	LH	Prog	0.480761974	2.12E-10

B31	B117	FSH	TT4	-0.635052896	0
B31	B225	FSH	hPBG	-0.817629818	0
B31	B180	FSH	Tf	-0.583030591	1.33E-15
B31	B125	FSH	Cor	-0.471404521	5.26E-10
B31	B92	FSH	ALDH2-Glu504Glu	-0.416025147	6.62E-08
B32	B145	Testo	BGP	0.597614305	2.22E-16
B32	B38	Testo	BAND%	0.5143445	6.51E-12
B32	B94	Testo	ALDH2-Lys504Lys	0.433554985	1.57E-08
B33	B94	Prog	ALDH2-Lys504Lys	0.724791515	0
B33	B37	Prog	BAND	0.549442256	1.11E-13
B33	B93	Prog	ALDH2-Glu504Lys	0.545132838	1.87E-13
B33	B36	Prog	V-Lym%	0.507237499	1.40E-11
B34	B100	TK	anti-Ro52	1	0
B34	B101	TK	anti-Sc70	0.999999997	0
B34	B102	TK	anti-Sm	1	0
B34	B105	TK	anti-U1RNP	1	0
B34	B109	TK	AMA-M2	1	0
B34	B110	TK	AHA	1	0
B34	B151	TK	Anti-CCP	0.7048221	0
B34	B156	TK	IgE	-0.903553226	0
B34	B183	TK	PTA	-0.935351263	0
B34	B57	TK	RBCAI	0.673254212	0
B34	B90	TK	CYFRA 21-1	-0.720217082	0
B34	B98	TK	anti-Jo-1	0.999999997	0
B34	B99	TK	anti-PM-Scl	0.999999999	0
B34	B59	TK	WBRV	0.571774066	6.44E-15
B34	B155	TK	IgA	0.55642635	4.64E-14
B34	B175	TK	ALB-U	0.538122697	4.33E-13
B34	B158	TK	IgM	-0.492296831	6.70E-11
B34	B60	TK	WBRVI	0.490164144	8.32E-11
B34	B54	TK	ESR	0.486953983	1.15E-10
B34	B198	TK	P	-0.484721524	1.44E-10
B34	B185	TK	PT	0.471649437	5.14E-10
B34	B184	TK	INR	0.461953764	1.28E-09
B34	B181	TK	APTT	0.459306489	1.63E-09
B34	B65	TK	WBRSR(mPas_1)	0.450002865	3.78E-09
B34	B58	TK	HCT	0.423424837	3.64E-08
B34	B168	TK	NMP22	0.401609664	2.03E-07
B34	B157	TK	IgG	0.400110805	2.28E-07
B34	B176	TK	Ucr	0.387593593	5.77E-07
B35	B124	β2-MG	ACTH	0.951189731	0
B35	B171	β2-MG	EC	0.945905303	0
B35	B36	β2-MG	V-Lym%	0.818934617	0
B35	B38	β2-MG	BAND%	0.967805339	0

B35	B94	β2-MG	ALDH2-Lys504Lys	-0.684210526	0
B35	B116	β2-MG	TT3	0.449921271	3.80E-09
B35	B93	β2-MG	ALDH2-Glu504Lys	0.390199486	4.77E-07
B36	B124	V-Lym%	ACTH	0.778962199	0
B36	B37	V-Lym%	BAND	-0.738548946	0
B36	B145	V-Lym%	BGP	-0.583874208	1.33E-15
B36	B38	V-Lym%	BAND%	0.502518908	2.32E-11
B36	B171	V-Lym%	EC	0.447811076	4.59E-09
B37	B38	BAND	BAND%	-0.748455199	0
B38	B94	BAND%	ALDH2-Lys504Lys	0.718049122	0
B38	B124	BAND%	ACTH	0.534522484	6.62E-13
B39	B78	Other cells	HBcAb	0.888189409	0
B39	B184	Other cells	INR	0.410087608	1.06E-07
B4	B33	WB-Ca	Prog	0.638273567	0
B4	B36	WB-Ca	V-Lym%	0.994729463	0
B4	B37	WB-Ca	BAND	0.606091527	0
B4	B9	WB-Ca	RH	0.785714286	0
B4	B94	WB-Ca	ALDH2-Lys504Lys	0.55618773	4.77E-14
B4	B171	WB-Ca	EC	0.416496564	6.37E-08
B40	B42	WBC	MON	0.662710799	0
B40	B52	WBC	PLT/BPC	0.396185433	3.06E-07
B41	B42	MON %	MON	0.681625394	0
B43	B44	MCV	MCH	0.927882323	0
B43	B47	MCV	RDW	0.553916452	6.35E-14
B43	B46	MCV	RBC Count	-0.427523178	2.60E-08
B43	B51	MCV	Hb	0.383605289	7.69E-07
B44	B45	MCH	MCHC	0.665965631	0
B44	B51	MCH	Hb	0.534867766	6.36E-13
B45	B51	MCHC	Hb	0.593785636	4.44E-16
B45	B221	MCHC	Hei	0.386854209	6.08E-07
B46	B51	RBC Count	Hb	0.617771182	0
B46	B216	RBC Count	BMI	0.426842781	2.75E-08
B46	B222	RBC Count	NS	0.397583531	2.76E-07
B46	B72	RBC Count	HDL-C	-0.391832687	4.23E-07
B47	B52	RDW	PLT/BPC	-0.409858048	1.08E-07
B48	B53	LYM%	GR%	-0.901082556	0
B5	B116	WB-Mg	TT3	0.980580676	0
B5	B30	WB-Mg	LH	0.6	0
B5	B7	WB-Mg	WB-Cop	0.896717661	0
B5	B171	WB-Mg	EC	0.533578375	7.39E-13
B5	B124	WB-Mg	ACTH	0.480079359	2.27E-10
B5	B9	WB-Mg	RH	0.459619408	1.58E-09
B50	B53	eo%	GR%	-0.42464886	3.30E-08
B51	B216	Hb	BMI	0.60870164	0

B51	B222	Hb	NS	0.596050737	2.22E-16
B51	B221	Hb	Hei	0.555502135	5.20E-14
B51	B206	Hb	Waist	0.505548499	1.68E-11
B51	B128	Hb	ALT	0.47684554	3.11E-10
B51	B192	Hb	UA	0.436363781	1.24E-08
B51	B207	Hb	WHR	0.43259909	1.70E-08
B51	B215	Hb	BMI	0.422192602	4.03E-08
B51	B210	Hb	Hip	0.420201984	4.73E-08
B51	B190	Hb	Cre	0.413015653	8.40E-08
B51	B163	Hb	SG	0.409572191	1.10E-07
B51	B141	Hb	AST/ALT	-0.388311016	5.47E-07
B54	B59	ESR	WBRV	0.653536948	0
B54	B69	ESR	PV	0.775779336	0
B54	B182	ESR	TT	-0.563927758	1.78E-14
B54	B186	ESR	PFC	0.532587861	8.30E-13
B54	B100	ESR	anti-Ro52	0.486953983	1.15E-10
B54	B101	ESR	anti-Sc70	0.486953981	1.15E-10
B54	B102	ESR	anti-Sm	0.486953982	1.15E-10
B54	B103	ESR	anti-SSA/Ro	0.486953983	1.15E-10
B54	B104	ESR	anti-SSB	0.486953984	1.15E-10
B54	B105	ESR	anti-U1RNP	0.486953982	1.15E-10
B54	B106	ESR	anti-P	0.486953983	1.15E-10
B54	B107	ESR	AnuA	0.486953993	1.15E-10
B54	B108	ESR	Anti-Cyclin	0.486953983	1.15E-10
B54	B109	ESR	AMA-M2	0.486953983	1.15E-10
B54	B110	ESR	AHA	0.486953982	1.15E-10
B54	B95	ESR	anti-cenpb	0.486953983	1.15E-10
B54	B97	ESR	dsDNA	0.486953983	1.15E-10
B54	B98	ESR	anti-Jo-1	0.486953981	1.15E-10
B54	B99	ESR	anti-PM-Scl	0.486953982	1.15E-10
B54	B198	ESR	P	-0.413539252	8.06E-08
B54	B175	ESR	ALB-U	0.40546693	1.51E-07
B54	B157	ESR	IgG	0.396311532	3.03E-07
B54	B156	ESR	IgE	-0.395455498	3.23E-07
B54	B74	ESR	ApoA1	0.391156952	4.44E-07
B55	B56	TK	IR	0.754875937	0
B55	B59	TK	WBRV	0.642649442	0
B55	B61	TK	WBHSRV	0.725202895	0
B55	B62	TK	WBHSRVI	0.648399763	0
B55	B63	TK	WBRSR(mPas_200)	0.635797941	0
B55	B64	TK	WBRSR(mPas_5)	0.615046872	0
B55	B66	TK	WBRSR(mPas_100)	0.631759993	0
B55	B67	TK	WBRSR(mPas_50)	0.631564019	0
B55	B60	TK	WBRVI	0.589817092	4.44E-16

B55	B65	TK	WBRSR(mPas_1)	0.593692492	4.44E-16
B55	B57	TK	RBCAI	-0.478585737	2.63E-10
B55	B58	TK	HCT	0.469338227	6.40E-10
B55	B82	TK	CEA	-0.448671862	4.25E-09
B55	B85	TK	SCC	0.409745881	1.09E-07
B56	B181	IR	APTT	0.631205858	0
B56	B58	IR	HCT	0.928594544	0
B56	B59	IR	WBRV	0.791785242	0
B56	B60	IR	WBRVI	0.955359382	0
B56	B61	IR	WBHSRV	0.950615995	0
B56	B62	IR	WBHSRVI	0.988681151	0
B56	B63	IR	WBRSR(mPas_200)	0.944744621	0
B56	B64	IR	WBRSR(mPas_5)	0.94920596	0
B56	B65	IR	WBRSR(mPas_1)	0.938865572	0
B56	B66	IR	WBRSR(mPas_100)	0.951796663	0
B56	B67	IR	WBRSR(mPas_50)	0.952262054	0
B56	B185	IR	PT	0.556329141	4.71E-14
B56	B57	IR	RBCAI	-0.486331958	1.22E-10
B56	B182	IR	TT	0.439752697	9.26E-09
B56	B186	IR	PFC	-0.409324358	1.12E-07
B56	B183	IR	PTA	-0.399697336	2.35E-07
B57	B100	RBCAI	anti-Ro52	0.673254212	0
B57	B101	RBCAI	anti-Sc70	0.67325421	0
B57	B102	RBCAI	anti-Sm	0.673254212	0
B57	B103	RBCAI	anti-SSA/Ro	0.673254212	0
B57	B104	RBCAI	anti-SSB	0.673254213	0
B57	B105	RBCAI	anti-U1RNP	0.673254212	0
B57	B106	RBCAI	anti-P	0.673254212	0
B57	B107	RBCAI	AnuA	0.673254228	0
B57	B108	RBCAI	Anti-Cyclin	0.673254212	0
B57	B109	RBCAI	AMA-M2	0.673254212	0
B57	B110	RBCAI	AHA	0.673254212	0
B57	B95	RBCAI	anti-cenpb	0.673254212	0
B57	B97	RBCAI	dsDNA	0.673254212	0
B57	B98	RBCAI	anti-Jo-1	0.67325421	0
B57	B99	RBCAI	anti-PM-Scl	0.673254212	0
B57	B156	RBCAI	IgE	-0.580427933	2.00E-15
B57	B90	RBCAI	CYFRA 21-1	-0.579403025	2.22E-15
B57	B61	RBCAI	WBHSRV	-0.561143131	2.55E-14
B57	B183	RBCAI	PTA	-0.554065171	6.24E-14
B57	B66	RBCAI	WBRSR(mPas_100)	-0.533783385	7.22E-13
B57	B63	RBCAI	WBRSR(mPas_200)	-0.522143781	2.74E-12
B57	B67	RBCAI	WBRSR(mPas_50)	-0.520820913	3.18E-12
B57	B151	RBCAI	Anti-CCP	0.470864386	5.53E-10

B57	B62	RBCAI	WBHSRVI	-0.467385839	7.69E-10
B57	B182	RBCAI	TT	-0.44356452	6.66E-09
B57	B64	RBCAI	WBRSR(mPas_5)	-0.43388162	1.53E-08
B57	B186	RBCAI	PFC	0.421300532	4.33E-08
B57	B157	RBCAI	IgG	0.396282707	3.04E-07
B58	B181	HCT	APTT	0.801570407	0
B58	B185	HCT	PT	0.712066125	0
B58	B59	HCT	WBRV	0.761164617	0
B58	B60	HCT	WBRVI	0.979121342	0
B58	B61	HCT	WBHSRV	0.879164402	0
B58	B62	HCT	WBHSRVI	0.969210789	0
B58	B63	HCT	WBRSR(mPas_200)	0.921718972	0
B58	B64	HCT	WBRSR(mPas_5)	0.947871464	0
B58	B65	HCT	WBRSR(mPas_1)	0.955395958	0
B58	B66	HCT	WBRSR(mPas_100)	0.929961591	0
B58	B67	HCT	WBRSR(mPas_50)	0.932370716	0
B58	B183	HCT	PTA	-0.567117229	1.18E-14
B58	B182	HCT	TT	0.506162736	1.58E-11
B58	B186	HCT	PFC	-0.470353637	5.81E-10
B58	B156	HCT	IgE	-0.444802333	5.98E-09
B58	B100	HCT	anti-Ro52	0.423424837	3.64E-08
B58	B101	HCT	anti-Sc70	0.423424835	3.64E-08
B58	B102	HCT	anti-Sm	0.423424836	3.64E-08
B58	B103	HCT	anti-SSA/Ro	0.423424837	3.64E-08
B58	B104	HCT	anti-SSB	0.423424838	3.64E-08
B58	B105	HCT	anti-U1RNP	0.423424837	3.64E-08
B58	B106	HCT	anti-P	0.423424837	3.64E-08
B58	B107	HCT	AnuA	0.423424846	3.64E-08
B58	B108	HCT	Anti-Cyclin	0.423424837	3.64E-08
B58	B109	HCT	AMA-M2	0.423424837	3.64E-08
B58	B110	HCT	AHA	0.423424837	3.64E-08
B58	B95	HCT	anti-cenpb	0.423424837	3.64E-08
B58	B97	HCT	dsDNA	0.423424837	3.64E-08
B58	B98	HCT	anti-Jo-1	0.423424836	3.64E-08
B58	B99	HCT	anti-PM-Scl	0.423424836	3.64E-08
B58	B158	HCT	IgM	-0.4200254	4.80E-08
B59	B60	WBRV	WBRVI	0.785802654	0
B59	B61	WBRV	WBHSRV	0.883529281	0
B59	B62	WBRV	WBHSRVI	0.766621119	0
B59	B63	WBRV	WBRSR(mPas_200)	0.875785951	0
B59	B64	WBRV	WBRSR(mPas_5)	0.892262875	0
B59	B65	WBRV	WBRSR(mPas_1)	0.90995803	0
B59	B66	WBRV	WBRSR(mPas_100)	0.856566108	0
B59	B67	WBRV	WBRSR(mPas_50)	0.862799664	0

B59	B183	WBRV	PTA	-0.581174313	1.78E-15
B59	B100	WBRV	anti-Ro52	0.571774066	6.44E-15
B59	B101	WBRV	anti-Sc70	0.571774064	6.44E-15
B59	B102	WBRV	anti-Sm	0.571774066	6.44E-15
B59	B103	WBRV	anti-SSA/Ro	0.571774066	6.44E-15
B59	B104	WBRV	anti-SSB	0.571774068	6.44E-15
B59	B105	WBRV	anti-U1RNP	0.571774066	6.44E-15
B59	B106	WBRV	anti-P	0.571774066	6.44E-15
B59	B107	WBRV	AnuA	0.571774077	6.44E-15
B59	B108	WBRV	Anti-Cyclin	0.571774066	6.44E-15
B59	B109	WBRV	AMA-M2	0.571774066	6.44E-15
B59	B110	WBRV	AHA	0.571774066	6.44E-15
B59	B95	WBRV	anti-cenpb	0.571774067	6.44E-15
B59	B97	WBRV	dsDNA	0.571774066	6.44E-15
B59	B98	WBRV	anti-Jo-1	0.571774065	6.44E-15
B59	B99	WBRV	anti-PM-Scl	0.571774066	6.44E-15
B59	B156	WBRV	IgE	-0.533428991	7.52E-13
B59	B69	WBRV	PV	0.508980562	1.17E-11
B59	B181	WBRV	APTT	0.426967726	2.72E-08
B59	B185	WBRV	PT	0.40828593	1.22E-07
B59	B151	WBRV	Anti-CCP	0.404533446	1.63E-07
B6	B180	WB-Fe	Tf	-0.637792804	0
B6	B29	WB-Fe	E2	-0.561951487	2.31E-14
B6	B28	WB-Fe	PRL	-0.510688231	9.69E-12
B60	B181	WBRVI	APTT	0.78301838	0
B60	B183	WBRVI	PTA	-0.615571695	0
B60	B185	WBRVI	PT	0.70219741	0
B60	B61	WBRVI	WBHSRV	0.875192264	0
B60	B62	WBRVI	WBHSRVI	0.971532803	0
B60	B63	WBRVI	WBRSR(mPas_200)	0.899303498	0
B60	B64	WBRVI	WBRSR(mPas_5)	0.932383701	0
B60	B65	WBRVI	WBRSR(mPas_1)	0.946566336	0
B60	B66	WBRVI	WBRSR(mPas_100)	0.906716902	0
B60	B67	WBRVI	WBRSR(mPas_50)	0.910525265	0
B60	B156	WBRVI	IgE	-0.500143067	2.98E-11
B60	B100	WBRVI	anti-Ro52	0.490164144	8.32E-11
B60	B101	WBRVI	anti-Sc70	0.490164143	8.32E-11
B60	B102	WBRVI	anti-Sm	0.490164144	8.32E-11
B60	B103	WBRVI	anti-SSA/Ro	0.490164144	8.32E-11
B60	B104	WBRVI	anti-SSB	0.490164146	8.32E-11
B60	B105	WBRVI	anti-U1RNP	0.490164144	8.32E-11
B60	B106	WBRVI	anti-P	0.490164145	8.32E-11
B60	B107	WBRVI	AnuA	0.490164156	8.32E-11
B60	B108	WBRVI	Anti-Cyclin	0.490164144	8.32E-11

B60	B109	WBRVI	AMA-M2	0.490164144	8.32E-11
B60	B110	WBRVI	AHA	0.490164144	8.32E-11
B60	B95	WBRVI	anti-cenpb	0.490164145	8.32E-11
B60	B97	WBRVI	dsDNA	0.490164145	8.32E-11
B60	B98	WBRVI	anti-Jo-1	0.490164143	8.32E-11
B60	B99	WBRVI	anti-PM-Scl	0.490164144	8.32E-11
B60	B182	WBRVI	TT	0.439252619	9.67E-09
B60	B158	WBRVI	IgM	-0.435136474	1.37E-08
B60	B186	WBRVI	PFC	-0.407031957	1.34E-07
B60	B184	WBRVI	INR	0.380086499	9.88E-07
B61	B62	WBHSRV	WBHSRVI	0.938423877	0
B61	B63	WBHSRV	WBRSR(mPas_200)	0.991329285	0
B61	B64	WBHSRV	WBRSR(mPas_5)	0.97941114	0
B61	B65	WBHSRV	WBRSR(mPas_1)	0.960754975	0
B61	B66	WBHSRV	WBRSR(mPas_100)	0.989051386	0
B61	B67	WBHSRV	WBRSR(mPas_50)	0.988877746	0
B61	B181	WBHSRV	APTT	0.484147635	1.52E-10
B61	B185	WBHSRV	PT	0.428670374	2.36E-08
B61	B69	WBHSRV	PV	0.414931192	7.22E-08
B62	B181	WBHSRVI	APTT	0.697129785	0
B62	B185	WBHSRVI	PT	0.612879625	0
B62	B63	WBHSRVI	WBRSR(mPas_200)	0.951716347	0
B62	B64	WBHSRVI	WBRSR(mPas_5)	0.960325259	0
B62	B65	WBHSRVI	WBRSR(mPas_1)	0.95186254	0
B62	B66	WBHSRVI	WBRSR(mPas_100)	0.961165043	0
B62	B67	WBHSRVI	WBRSR(mPas_50)	0.961520804	0
B62	B182	WBHSRVI	TT	0.498003339	3.72E-11
B62	B186	WBHSRVI	PFC	-0.463805643	1.08E-09
B62	B183	WBHSRVI	PTA	-0.427831783	2.54E-08
B63	B64	WBRSR(mPas_200)	WBRSR(mPas_5)	0.994351325	0
B63	B65	WBRSR(mPas_200)	WBRSR(mPas_1)	0.980095382	0
B63	B66	WBRSR(mPas_200)	WBRSR(mPas_100)	0.999001218	0
B63	B67	WBRSR(mPas_200)	WBRSR(mPas_50)	0.999186937	0
B63	B181	WBRSR(mPas_200)	APTT	0.558198963	3.71E-14
B63	B185	WBRSR(mPas_200)	PT	0.49416294	5.53E-11
B63	B69	WBRSR(mPas_200)	PV	0.404772855	1.60E-07
B64	B181	WBRSR(mPas_5)	APTT	0.611871437	0
B64	B65	WBRSR(mPas_5)	WBRSR(mPas_1)	0.995399827	0
B64	B66	WBRSR(mPas_5)	WBRSR(mPas_100)	0.993341621	0
B64	B67	WBRSR(mPas_5)	WBRSR(mPas_50)	0.99499354	0
B64	B185	WBRSR(mPas_5)	PT	0.547257711	1.44E-13
B64	B183	WBRSR(mPas_5)	PTA	-0.465573535	9.12E-10
B65	B181	WBRSR(mPas_1)	APTT	0.645768069	0
B65	B66	WBRSR(mPas_1)	WBRSR(mPas_100)	0.977776782	0

B65	B67	WBRSR(mPas_1)	WBRSR(mPas_50)	0.980873646	0
B65	B185	WBRSR(mPas_1)	PT	0.583001602	1.33E-15
B65	B183	WBRSR(mPas_1)	PTA	-0.544523134	2.01E-13
B65	B156	WBRSR(mPas_1)	IgE	-0.451685312	3.25E-09
B65	B100	WBRSR(mPas_1)	anti-Ro52	0.450002865	3.78E-09
B65	B101	WBRSR(mPas_1)	anti-Sc70	0.450002864	3.78E-09
B65	B102	WBRSR(mPas_1)	anti-Sm	0.450002865	3.78E-09
B65	B103	WBRSR(mPas_1)	anti-SSA/Ro	0.450002865	3.78E-09
B65	B104	WBRSR(mPas_1)	anti-SSB	0.450002866	3.78E-09
B65	B105	WBRSR(mPas_1)	anti-U1RNP	0.450002865	3.78E-09
B65	B106	WBRSR(mPas_1)	anti-P	0.450002865	3.78E-09
B65	B107	WBRSR(mPas_1)	AnuA	0.450002874	3.78E-09
B65	B108	WBRSR(mPas_1)	Anti-Cyclin	0.450002865	3.78E-09
B65	B109	WBRSR(mPas_1)	AMA-M2	0.450002865	3.78E-09
B65	B110	WBRSR(mPas_1)	AHA	0.450002865	3.78E-09
B65	B95	WBRSR(mPas_1)	anti-cenpb	0.450002866	3.78E-09
B65	B97	WBRSR(mPas_1)	dsDNA	0.450002865	3.78E-09
B65	B98	WBRSR(mPas_1)	anti-Jo-1	0.450002864	3.78E-09
B65	B99	WBRSR(mPas_1)	anti-PM-Scl	0.450002865	3.78E-09
B66	B67	WBRSR(mPas_100)	WBRSR(mPas_50)	0.999876245	0
B66	B181	WBRSR(mPas_100)	APTT	0.567743662	1.09E-14
B66	B185	WBRSR(mPas_100)	PT	0.501063211	2.70E-11
B66	B182	WBRSR(mPas_100)	TT	0.38978406	4.91E-07
B67	B181	WBRSR(mPas_50)	APTT	0.572046227	6.22E-15
B67	B185	WBRSR(mPas_50)	PT	0.505954957	1.61E-11
B67	B182	WBRSR(mPas_50)	TT	0.383312356	7.85E-07
B69	B176	PV	Ucr	-0.437773342	1.10E-08
B69	B74	PV	ApoA1	0.400587806	2.20E-07
B7	B124	WB-Cop	ACTH	0.978399181	0
B7	B33	WB-Cop	Prog	0.703849392	0
B7	B94	WB-Cop	ALDH2-Lys504Lys	0.844926112	0
B7	B36	WB-Cop	V-Lym%	0.571601983	6.66E-15
B7	B30	WB-Cop	LH	0.480384461	2.21E-10
B7	B37	WB-Cop	BAND	0.400320385	2.24E-07
B70	B77	LDL-C	TC	0.787478139	0
B71	B140	TG	r-GT	0.474847367	3.78E-10
B71	B72	TG	HDL-C	-0.462187317	1.25E-09
B71	B207	TG	WHR	0.413425066	8.14E-08
B71	B77	TG	TC	0.381330446	9.04E-07
B72	B216	HDL-C	BMI	-0.517135185	4.79E-12
B72	B206	HDL-C	Waist	-0.476238126	3.30E-10
B72	B222	HDL-C	NS	-0.453377628	2.79E-09
B72	B215	HDL-C	BMI	-0.443219935	6.86E-09
B72	B192	HDL-C	UA	-0.431655671	1.84E-08

B72	B141	HDL-C	AST/ALT	0.423385755	3.65E-08
B72	B207	HDL-C	WHR	-0.409716864	1.09E-07
B72	B210	HDL-C	Hip	-0.38937128	5.06E-07
B72	B190	HDL-C	Cre	-0.386151352	6.40E-07
B72	B128	HDL-C	ALT	-0.382198207	8.50E-07
B73	B193	ApoA	K	-0.544186079	2.10E-13
B73	B134	ApoA	CHE	0.497565324	3.89E-11
B73	B75	ApoA	ApoA1/B	0.449204682	4.05E-09
B73	B114	ApoA	TSH TT3 TT4(TT4)	0.446470887	5.16E-09
B74	B198	ApoA1	P	-0.622342131	0
B74	B135	ApoA1	TBA	0.595853834	2.22E-16
B74	B79	ApoA1	hb V -dna	-0.566667787	1.24E-14
B74	B75	ApoA1	ApoA1/B	0.534624838	6.54E-13
B74	B196	ApoA1	TCO2	0.502442416	2.34E-11
B74	B194	ApoA1	Cl	-0.449298461	4.02E-09
B74	B150	ApoA1	ALP	0.388595461	5.36E-07
B75	B76	ApoA1/B	ApoB	-0.800440872	0
B75	B198	ApoA1/B	P	-0.551897781	8.17E-14
B75	B135	ApoA1/B	TBA	0.509493311	1.10E-11
B75	B196	ApoA1/B	TCO2	0.45010983	3.74E-09
B75	B199	ApoA1/B	Mg	0.437005778	1.17E-08
B75	B197	ApoA1/B	AG	-0.415681505	6.80E-08
B76	B199	ApoB	Mg	-0.62222023	0
B76	B197	ApoB	AG	0.565743498	1.42E-14
B76	B138	ApoB	PAB	0.480328932	2.22E-10
B76	B87	ApoB	NSE	0.447438573	4.74E-09
B76	B150	ApoB	ALP	0.393469514	3.75E-07
B76	B85	ApoB	SCC	-0.380725394	9.44E-07
B79	B135	hb V -dna	TBA	-0.454374906	2.55E-09
B79	B112	hb V -dna	FT4	-0.387441038	5.83E-07
B8	B125	WB-Zinc	Cor	0.603022689	0
B8	B180	WB-Zinc	Tf	-0.824322106	0
B8	B29	WB-Zinc	E2	-0.691714464	0
B8	B96	WB-Zinc	dsDNA (IIF)	0.639602149	0
B82	B86	CEA	TBhCG	-0.598505808	0
B82	B87	CEA	NSE	-0.639696363	0
B82	B89	CEA	CA 72-4	-0.790443854	0
B82	B197	CEA	AG	-0.525462331	1.88E-12
B82	B84	CEA	C-PSA	-0.506954954	1.45E-11
B82	B115	CEA	TSH TT3 TT4(TT3)	-0.439790272	9.23E-09
B82	B88	CEA	CA 242	0.412229427	8.94E-08
B83	B90	AFP	CYFRA 21-1	0.552268908	7.79E-14
B83	B200	AFP	OP	0.530158413	1.10E-12
B83	B195	AFP	Na	0.511071315	9.30E-12

B84	B85	C-PSA	SCC	-0.637762019	0
B84	B86	C-PSA	TBhCG	0.60622951	0
B84	B88	C-PSA	CA 242	-0.922835511	0
B84	B89	C-PSA	CA 72-4	0.790532091	0
B84	B197	C-PSA	AG	0.582805651	1.33E-15
B84	B168	C-PSA	NMP22	0.568536022	9.77E-15
B84	B200	C-PSA	OP	0.444693619	6.03E-09
B85	B197	SCC	AG	-0.646464351	0
B85	B88	SCC	CA 242	0.816293757	0
B85	B115	SCC	TSH TT3 TT4(TT3)	-0.537041643	4.92E-13
B85	B194	SCC	Cl	0.440621886	8.59E-09
B86	B168	TBhCG	NMP22	0.600219258	0
B86	B89	TBhCG	CA 72-4	0.925788656	0
B86	B90	TBhCG	CYFRA 21-1	0.599785707	0
B86	B200	TBhCG	OP	0.522105903	2.75E-12
B86	B195	TBhCG	Na	0.517652052	4.52E-12
B86	B88	TBhCG	CA 242	-0.435820159	1.30E-08
B87	B115	NSE	TSH TT3 TT4(TT3)	0.625799513	0
B87	B197	NSE	AG	0.555096708	5.48E-14
B87	B194	NSE	Cl	-0.437011322	1.17E-08
B88	B197	CA 242	AG	-0.748593379	0
B88	B89	CA 242	CA 72-4	-0.619992658	0
B88	B200	CA 242	OP	-0.525664669	1.84E-12
B88	B168	CA 242	NMP22	-0.482809704	1.74E-10
B88	B194	CA 242	Cl	0.413401505	8.15E-08
B88	B195	CA 242	Na	-0.395165241	3.30E-07
B89	B168	CA 72-4	NMP22	0.569013526	9.33E-15
B89	B90	CA 72-4	CYFRA 21-1	0.484806446	1.42E-10
B89	B197	CA 72-4	AG	0.462778319	1.18E-09
B89	B200	CA 72-4	OP	0.448130234	4.46E-09
B89	B195	CA 72-4	Na	0.38574515	6.59E-07
B9	B124	RH	ACTH	0.6172134	0
B9	B36	RH	V-Lym%	0.957427108	0
B9	B94	RH	ALDH2-Lys504Lys	0.405553553	1.50E-07
B90	B100	CYFRA 21-1	anti-Ro52	-0.720217082	0
B90	B101	CYFRA 21-1	anti-Sc70	-0.72021708	0
B90	B102	CYFRA 21-1	anti-Sm	-0.720217082	0
B90	B103	CYFRA 21-1	anti-SSA/Ro	-0.720217082	0
B90	B104	CYFRA 21-1	anti-SSB	-0.720217084	0
B90	B105	CYFRA 21-1	anti-U1RNP	-0.720217082	0
B90	B106	CYFRA 21-1	anti-P	-0.720217082	0
B90	B107	CYFRA 21-1	AnuA	-0.720217097	0
B90	B108	CYFRA 21-1	Anti-Cyclin	-0.720217082	0
B90	B109	CYFRA 21-1	AMA-M2	-0.720217082	0

B90	B110	CYFRA 21-1	AHA	-0.720217082	0
B90	B156	CYFRA 21-1	IgE	0.651658316	0
B90	B183	CYFRA 21-1	PTA	0.66976743	0
B90	B95	CYFRA 21-1	anti-cenpb	-0.720217083	0
B90	B97	CYFRA 21-1	dsDNA	-0.720217082	0
B90	B98	CYFRA 21-1	anti-Jo-1	-0.72021708	0
B90	B99	CYFRA 21-1	anti-PM-Scl	-0.720217081	0
B90	B151	CYFRA 21-1	Anti-CCP	-0.513118653	7.44E-12
B90	B155	CYFRA 21-1	IgA	-0.409556575	1.10E-07
B90	B175	CYFRA 21-1	ALB-U	-0.404773635	1.60E-07
B91	B130	TP	GLB	0.769458428	0
B91	B127	TP	A-G ratio	-0.493930493	5.66E-11
B91	B126	TP	Alb	0.433703152	1.55E-08
B92	B117	ALDH2-Glu504Glu	TT4	-0.603881085	0
B92	B180	ALDH2-Glu504Glu	Tf	-0.944689404	0
B92	B202	ALDH2-Glu504Glu	e-times	-0.877579922	0
B93	B145	ALDH2-Glu504Lys	BGP	0.657375735	0
B93	B171	ALDH2-Glu504Lys	EC	0.870863572	0
B94	B171	ALDH2-Lys504Lys	EC	0.612056372	0
B95	B101	anti-cenpb	anti-Sc70	0.999999996	0
B95	B106	anti-cenpb	anti-P	0.999999998	0
B95	B151	anti-cenpb	Anti-CCP	0.704822101	0
B95	B156	anti-cenpb	IgE	-0.903553227	0
B95	B183	anti-cenpb	PTA	-0.935351264	0
B95	B98	anti-cenpb	anti-Jo-1	0.999999998	0
B95	B155	anti-cenpb	IgA	0.556426351	4.64E-14
B95	B175	anti-cenpb	ALB-U	0.538122698	4.33E-13
B95	B158	anti-cenpb	IgM	-0.492296832	6.70E-11
B95	B198	anti-cenpb	P	-0.484721525	1.44E-10
B95	B185	anti-cenpb	PT	0.471649437	5.14E-10
B95	B184	anti-cenpb	INR	0.461953765	1.28E-09
B95	B181	anti-cenpb	APTT	0.459306489	1.63E-09
B95	B168	anti-cenpb	NMP22	0.401609665	2.03E-07
B95	B157	anti-cenpb	IgG	0.400110805	2.28E-07
B95	B176	anti-cenpb	Ucr	0.387593593	5.77E-07
B97	B101	dsDNA	anti-Sc70	0.999999998	0
B97	B109	dsDNA	AMA-M2	1	0
B97	B151	dsDNA	Anti-CCP	0.7048221	0
B97	B156	dsDNA	IgE	-0.903553226	0
B97	B183	dsDNA	PTA	-0.935351263	0
B97	B98	dsDNA	anti-Jo-1	0.999999998	0
B97	B155	dsDNA	IgA	0.55642635	4.64E-14
B97	B175	dsDNA	ALB-U	0.538122697	4.33E-13
B97	B158	dsDNA	IgM	-0.492296831	6.70E-11

B97	B198	dsDNA	P	-0.484721524	1.44E-10
B97	B185	dsDNA	PT	0.471649437	5.14E-10
B97	B184	dsDNA	INR	0.461953764	1.28E-09
B97	B181	dsDNA	APTT	0.459306489	1.63E-09
B97	B168	dsDNA	NMP22	0.401609664	2.03E-07
B97	B157	dsDNA	IgG	0.400110805	2.28E-07
B97	B176	dsDNA	Ucr	0.387593593	5.77E-07
B98	B100	anti-Jo-1	anti-Ro52	0.999999997	0
B98	B101	anti-Jo-1	anti-Sc70	1	0
B98	B102	anti-Jo-1	anti-Sm	0.999999999	0
B98	B103	anti-Jo-1	anti-SSA/Ro	0.999999997	0
B98	B104	anti-Jo-1	anti-SSB	0.999999998	0
B98	B105	anti-Jo-1	anti-U1RNP	0.999999998	0
B98	B106	anti-Jo-1	anti-P	0.999999998	0
B98	B108	anti-Jo-1	Anti-Cyclin	0.999999998	0
B98	B109	anti-Jo-1	AMA-M2	0.999999997	0
B98	B110	anti-Jo-1	AHA	0.999999997	0
B98	B151	anti-Jo-1	Anti-CCP	0.704822098	0
B98	B156	anti-Jo-1	IgE	-0.903553224	0
B98	B183	anti-Jo-1	PTA	-0.935351261	0
B98	B99	anti-Jo-1	anti-PM-Scl	0.999999997	0
B98	B155	anti-Jo-1	IgA	0.556426348	4.64E-14
B98	B175	anti-Jo-1	ALB-U	0.538122696	4.33E-13
B98	B158	anti-Jo-1	IgM	-0.49229683	6.70E-11
B98	B198	anti-Jo-1	P	-0.484721523	1.44E-10
B98	B185	anti-Jo-1	PT	0.471649435	5.14E-10
B98	B184	anti-Jo-1	INR	0.461953763	1.28E-09
B98	B181	anti-Jo-1	APTT	0.459306488	1.63E-09
B98	B168	anti-Jo-1	NMP22	0.401609666	2.03E-07
B98	B157	anti-Jo-1	IgG	0.400110804	2.28E-07
B98	B176	anti-Jo-1	Ucr	0.387593591	5.77E-07
B99	B100	anti-PM-Scl	anti-Ro52	0.999999999	0
B99	B103	anti-PM-Scl	anti-SSA/Ro	0.999999999	0
B99	B108	anti-PM-Scl	Anti-Cyclin	1	0
B99	B110	anti-PM-Scl	AHA	1	0
B99	B151	anti-PM-Scl	Anti-CCP	0.7048221	0
B99	B156	anti-PM-Scl	IgE	-0.903553225	0
B99	B183	anti-PM-Scl	PTA	-0.935351262	0
B99	B155	anti-PM-Scl	IgA	0.55642635	4.64E-14
B99	B175	anti-PM-Scl	ALB-U	0.538122697	4.33E-13
B99	B158	anti-PM-Scl	IgM	-0.492296831	6.70E-11
B99	B198	anti-PM-Scl	P	-0.484721524	1.44E-10
B99	B185	anti-PM-Scl	PT	0.471649437	5.14E-10
B99	B184	anti-PM-Scl	INR	0.461953764	1.28E-09

B99	B181	anti-PM-Scl	APTT	0.459306489	1.63E-09
B99	B168	anti-PM-Scl	NMP22	0.401609666	2.03E-07
B99	B157	anti-PM-Scl	IgG	0.400110805	2.28E-07
B99	B176	anti-PM-Scl	Ucr	0.387593592	5.77E-07

Supplementary Table 11. PEI Correlations in Hypothyroidism

Row	Column	Row	Column	Cor	*P*
B1	B163	Gender	SG	-0.2932978	0
B1	B174	Gender	Urea/Cr	0.26229326	0
B1	B189	Gender	eGFB	0.2216233	0
B1	B190	Gender	Cre	-0.3224045	0
B1	B192	Gender	UA	-0.4492026	0
B1	B204	Gender	Drinking	-0.4458063	0
B1	B206	Gender	Waist	-0.4121516	0
B1	B207	Gender	WHR	-0.4001778	0
B1	B209	Gender	Smoking	-0.4627832	0
B1	B210	Gender	Hip	-0.2378583	0
B1	B216	Gender	BMI	-0.5070998	0
B1	B221	Gender	Hei	-0.5772929	0
B1	B222	Gender	NS	-0.6517958	0
B1	B42	Gender	MON	-0.2016853	0
B1	B45	Gender	MCHC	-0.2103364	0
B1	B46	Gender	RBC Count	-0.392497	0
B1	B51	Gender	Hb	-0.4968018	0
B1	B72	Gender	HDL-C	0.2333749	0
B1	B215	Gender	BMI	-0.1986516	4.44E-16
B1	B128	Gender	ALT	-0.1914466	3.55E-15
B1	B218	Gender	DBP	-0.1818718	8.08E-14
B1	B166	Gender	Pro	-0.1762402	4.69E-13
B1	B84	Gender	C-PSA	-0.1620117	3.10E-11
B1	B141	Gender	AST/ALT	0.15541088	1.92E-10
B1	B94	Gender	ALDH2-Lys504Lys	0.15240508	4.29E-10
B1	B140	Gender	r-GT	-0.1519831	4.80E-10
B1	B133	Gender	TBIL	-0.1502997	7.46E-10
B1	B71	Gender	TG	-0.1458477	2.35E-09
B1	B129	Gender	IBIL	-0.1446851	3.15E-09
B1	B41	Gender	MON％	-0.1399206	1.03E-08
B1	B2	Gender	Age	-0.1381718	1.57E-08
B1	B220	Gender	SBP	-0.135408	3.03E-08
B1	B191	Gender	Urea	-0.1341079	4.10E-08
B1	B44	Gender	MCH	-0.1321422	6.47E-08
B1	B132	Gender	Dbil	-0.1304053	9.63E-08
B1	B118	Gender	CEA1	-0.1234945	4.45E-07
B10	B11	PG I	PGI/PGII	0.30500541	0
B10	B12	PG I	PG II	0.49137292	0
B10	B14	PG I	G-17	-0.1742848	8.52E-13
B100	B101	anti-Ro52	anti-Sc70	-0.3185303	0
B100	B102	anti-Ro52	anti-Sm	-0.3185303	0
B100	B103	anti-Ro52	anti-SSA/Ro	0.70410455	0

B100	B104	anti-Ro52	anti-SSB	0.56083724	0
B100	B105	anti-Ro52	anti-U1RNP	-0.3185303	0
B100	B107	anti-Ro52	AnuA	-0.3185301	0
B100	B108	anti-Ro52	Anti-Cyclin	-0.3185303	0
B100	B157	anti-Ro52	IgG	0.20041427	0
B100	B180	anti-Ro52	Tf	-0.2434742	0
B100	B196	anti-Ro52	TCO2	0.23141832	0
B100	B197	anti-Ro52	AG	-0.255022	0
B100	B194	anti-Ro52	Cl	0.17604091	4.98E-13
B100	B178	anti-Ro52	Ig-U	-0.1593497	6.52E-11
B100	B179	anti-Ro52	α1-MG	-0.1550516	2.11E-10
B100	B193	anti-Ro52	K	-0.1504767	7.13E-10
B100	B138	anti-Ro52	PAB	-0.136584	2.29E-08
B100	B134	anti-Ro52	CHE	-0.1343484	3.88E-08
B100	B155	anti-Ro52	IgA	0.13268723	5.71E-08
B100	B136	anti-Ro52	5'NT	-0.128511	1.48E-07
B101	B103	anti-Sc70	anti-SSA/Ro	-0.3344827	0
B101	B107	anti-Sc70	AnuA	0.99999939	0
B101	B125	anti-Sc70	Cor	0.26774054	0
B101	B135	anti-Sc70	TBA	0.24449504	0
B101	B156	anti-Sc70	IgE	0.29730773	0
B101	B178	anti-Sc70	Ig-U	0.34819819	0
B101	B179	anti-Sc70	α1-MG	0.34866525	0
B101	B180	anti-Sc70	Tf	0.42439565	0
B101	B121	anti-Sc70	C3	-0.1954528	8.88E-16
B101	B122	anti-Sc70	C4	-0.195453	8.88E-16
B101	B159	anti-Sc70	β2-MG	-0.1954519	8.88E-16
B101	B169	anti-Sc70	U-CRY	-0.1954546	8.88E-16
B101	B184	anti-Sc70	INR	0.1931741	2.00E-15
B101	B106	anti-Sc70	anti-P	-0.19276	2.22E-15
B101	B154	anti-Sc70	RF	-0.1785002	2.33E-13
B101	B139	anti-Sc70	ADA	0.16693577	7.57E-12
B101	B119	anti-Sc70	Hcv-rna	0.16323416	2.19E-11
B101	B151	anti-Sc70	Anti-CCP	0.15767897	1.03E-10
B101	B104	anti-Sc70	anti-SSB	-0.1552047	2.03E-10
B101	B158	anti-Sc70	IgM	0.14873463	1.12E-09
B101	B181	anti-Sc70	APTT	-0.1409302	8.01E-09
B101	B137	anti-Sc70	AFU	0.13753181	1.83E-08
B101	B138	anti-Sc70	PAB	0.12878441	1.39E-07
B102	B103	anti-Sm	anti-SSA/Ro	-0.3344827	0
B102	B105	anti-Sm	anti-U1RNP	0.99999999	0
B102	B107	anti-Sm	AnuA	0.99999949	0
B102	B108	anti-Sm	Anti-Cyclin	0.99999999	0
B102	B125	anti-Sm	Cor	0.26774053	0

B102	B135	anti-Sm	TBA	0.24449504	0
B102	B156	anti-Sm	IgE	0.29730772	0
B102	B178	anti-Sm	Ig-U	0.34819818	0
B102	B179	anti-Sm	α1-MG	0.34866524	0
B102	B180	anti-Sm	Tf	0.42439564	0
B102	B121	anti-Sm	C3	-0.1954523	8.88E-16
B102	B122	anti-Sm	C4	-0.1954531	8.88E-16
B102	B159	anti-Sm	β2-MG	-0.1954505	8.88E-16
B102	B169	anti-Sm	U-CRY	-0.1954535	8.88E-16
B102	B184	anti-Sm	INR	0.1931741	2.00E-15
B102	B106	anti-Sm	anti-P	-0.19276	2.22E-15
B102	B154	anti-Sm	RF	-0.1785001	2.33E-13
B102	B139	anti-Sm	ADA	0.16693576	7.57E-12
B102	B119	anti-Sm	Hcv-rna	0.16323415	2.19E-11
B102	B151	anti-Sm	Anti-CCP	0.15767896	1.03E-10
B102	B104	anti-Sm	anti-SSB	-0.1552047	2.03E-10
B102	B158	anti-Sm	IgM	0.14873462	1.12E-09
B102	B181	anti-Sm	APTT	-0.1409302	8.01E-09
B102	B137	anti-Sm	AFU	0.1375318	1.83E-08
B102	B138	anti-Sm	PAB	0.12878441	1.39E-07
B103	B104	anti-SSA/Ro	anti-SSB	0.61143268	0
B103	B105	anti-SSA/Ro	anti-U1RNP	-0.3344827	0
B103	B107	anti-SSA/Ro	AnuA	-0.3344825	0
B103	B108	anti-SSA/Ro	Anti-Cyclin	-0.3344827	0
B103	B180	anti-SSA/Ro	Tf	-0.209224	0
B103	B199	anti-SSA/Ro	Mg	0.19617216	6.66E-16
B103	B157	anti-SSA/Ro	IgG	0.18682514	1.64E-14
B103	B138	anti-SSA/Ro	PAB	-0.1508511	6.46E-10
B103	B194	anti-SSA/Ro	Cl	0.15074574	6.64E-10
B103	B176	anti-SSA/Ro	Ucr	0.1425359	5.39E-09
B103	B186	anti-SSA/Ro	PFC	-0.1362468	2.48E-08
B103	B197	anti-SSA/Ro	AG	-0.1337711	4.44E-08
B103	B125	anti-SSA/Ro	Cor	-0.1329353	5.39E-08
B103	B156	anti-SSA/Ro	IgE	-0.1328801	5.46E-08
B103	B198	anti-SSA/Ro	P	0.12514342	3.11E-07
B104	B155	anti-SSB	IgA	0.21776694	0
B104	B157	anti-SSB	IgG	0.33826943	0
B104	B194	anti-SSB	Cl	0.20569998	0
B104	B198	anti-SSB	P	0.27117125	0
B104	B197	anti-SSB	AG	-0.1847552	3.20E-14
B104	B105	anti-SSB	anti-U1RNP	-0.1552047	2.03E-10
B104	B107	anti-SSB	AnuA	-0.1552046	2.03E-10
B104	B108	anti-SSB	Anti-Cyclin	-0.1552047	2.03E-10
B104	B158	anti-SSB	IgM	0.12687822	2.13E-07

B105	B107	anti-U1RNP	AnuA	0.99999928	0
B105	B108	anti-U1RNP	Anti-Cyclin	1	0
B105	B125	anti-U1RNP	Cor	0.26774053	0
B105	B135	anti-U1RNP	TBA	0.24449504	0
B105	B156	anti-U1RNP	IgE	0.29730773	0
B105	B178	anti-U1RNP	Ig-U	0.34819819	0
B105	B179	anti-U1RNP	α1-MG	0.34866524	0
B105	B180	anti-U1RNP	Tf	0.42439565	0
B105	B121	anti-U1RNP	C3	-0.1954525	8.88E-16
B105	B122	anti-U1RNP	C4	-0.195453	8.88E-16
B105	B159	anti-U1RNP	β2-MG	-0.1954514	8.88E-16
B105	B169	anti-U1RNP	U-CRY	-0.195454	8.88E-16
B105	B184	anti-U1RNP	INR	0.1931741	2.00E-15
B105	B106	anti-U1RNP	anti-P	-0.19276	2.22E-15
B105	B154	anti-U1RNP	RF	-0.1785002	2.33E-13
B105	B139	anti-U1RNP	ADA	0.16693577	7.57E-12
B105	B119	anti-U1RNP	Hcv-rna	0.16323416	2.19E-11
B105	B151	anti-U1RNP	Anti-CCP	0.15767896	1.03E-10
B105	B158	anti-U1RNP	IgM	0.14873462	1.12E-09
B105	B181	anti-U1RNP	APTT	-0.1409302	8.01E-09
B105	B137	anti-U1RNP	AFU	0.1375318	1.83E-08
B105	B138	anti-U1RNP	PAB	0.12878441	1.39E-07
B106	B157	anti-P	IgG	0.3091613	0
B106	B178	anti-P	Ig-U	-0.2486824	0
B106	B179	anti-P	α1-MG	-0.2580017	0
B106	B180	anti-P	Tf	-0.2392454	0
B106	B107	anti-P	AnuA	-0.1927599	2.22E-15
B106	B108	anti-P	Anti-Cyclin	-0.19276	2.22E-15
B107	B108	AnuA	Anti-Cyclin	0.99999926	0
B107	B125	AnuA	Cor	0.26774036	0
B107	B135	AnuA	TBA	0.24449486	0
B107	B156	AnuA	IgE	0.29730752	0
B107	B178	AnuA	Ig-U	0.34819794	0
B107	B179	AnuA	α1-MG	0.348665	0
B107	B180	AnuA	Tf	0.42439535	0
B107	B121	AnuA	C3	-0.1954527	8.88E-16
B107	B122	AnuA	C4	-0.1954528	8.88E-16
B107	B159	AnuA	β2-MG	-0.1954492	8.88E-16
B107	B169	AnuA	U-CRY	-0.1954536	8.88E-16
B107	B184	AnuA	INR	0.19317398	2.00E-15
B107	B154	AnuA	RF	-0.1785	2.33E-13
B107	B139	AnuA	ADA	0.16693565	7.57E-12
B107	B119	AnuA	Hcv-rna	0.16323404	2.19E-11
B107	B151	AnuA	Anti-CCP	0.15767885	1.03E-10

B107	B158	AnuA	IgM	0.14873452	1.12E-09
B107	B181	AnuA	APTT	-0.1409301	8.01E-09
B107	B137	AnuA	AFU	0.1375317	1.83E-08
B107	B138	AnuA	PAB	0.12878432	1.39E-07
B108	B125	Anti-Cyclin	Cor	0.26774053	0
B108	B135	Anti-Cyclin	TBA	0.24449504	0
B108	B156	Anti-Cyclin	IgE	0.29730773	0
B108	B178	Anti-Cyclin	Ig-U	0.34819819	0
B108	B179	Anti-Cyclin	α1-MG	0.34866524	0
B108	B180	Anti-Cyclin	Tf	0.42439565	0
B108	B121	Anti-Cyclin	C3	-0.1954526	8.88E-16
B108	B122	Anti-Cyclin	C4	-0.1954532	8.88E-16
B108	B159	Anti-Cyclin	β2-MG	-0.1954512	8.88E-16
B108	B169	Anti-Cyclin	U-CRY	-0.1954547	8.88E-16
B108	B184	Anti-Cyclin	INR	0.19317409	2.00E-15
B108	B154	Anti-Cyclin	RF	-0.1785002	2.33E-13
B108	B139	Anti-Cyclin	ADA	0.16693576	7.57E-12
B108	B119	Anti-Cyclin	Hcv-rna	0.16323416	2.19E-11
B108	B151	Anti-Cyclin	Anti-CCP	0.15767896	1.03E-10
B108	B158	Anti-Cyclin	IgM	0.14873462	1.12E-09
B108	B181	Anti-Cyclin	APTT	-0.1409302	8.01E-09
B108	B137	Anti-Cyclin	AFU	0.1375318	1.83E-08
B108	B138	Anti-Cyclin	PAB	0.12878441	1.39E-07
B109	B194	AMA-M2	Cl	-0.2564007	0
B109	B200	AMA-M2	OP	-0.1646234	1.47E-11
B109	B137	AMA-M2	AFU	0.15891578	7.36E-11
B109	B135	AMA-M2	TBA	0.13196973	6.74E-08
B109	B195	AMA-M2	Na	-0.1265361	2.29E-07
B11	B12	PGI/PGII	PG II	-0.4359501	0
B11	B14	PGI/PGII	G-17	-0.1861793	2.02E-14
B110	B145	AHA	BGP	-0.2385151	0
B110	B182	AHA	TT	-0.3000954	0
B110	B183	AHA	PTA	0.36807865	0
B110	B143	AHA	β-CTx	0.14045352	9.01E-09
B110	B151	AHA	Anti-CCP	-0.1362611	2.47E-08
B110	B194	AHA	Cl	0.13034051	9.78E-08
B110	B155	AHA	IgA	-0.1261841	2.48E-07
B110	B199	AHA	Mg	0.12473497	3.40E-07
B111	B112	TSH	FT4	-0.4603166	0
B111	B113	TSH	FT3	-0.3467826	0
B111	B114	TSH	TSH TT3 TT4(TT4)	-0.3598568	0
B111	B115	TSH	TSH TT3 TT4(TT3)	-0.3332098	0
B111	B148	TSH	Anti_TPO	0.20599664	0
B111	B149	TSH	TGAb	0.17080419	2.43E-12

B111	B185	TSH	PT	-0.1606495	4.54E-11
B111	B183	TSH	PTA	0.1563651	1.48E-10
B111	B184	TSH	INR	-0.1355076	2.95E-08
B111	B204	TSH	Drinking	0.12919253	1.27E-07
B112	B113	FT4	FT3	0.21784508	0
B112	B114	FT4	TSH TT3 TT4(TT4)	0.60802314	0
B112	B115	FT4	TSH TT3 TT4(TT3)	0.15174551	5.11E-10
B112	B185	FT4	PT	0.13167384	7.21E-08
B112	B184	FT4	INR	0.12662952	2.25E-07
B113	B115	FT3	TSH TT3 TT4(TT3)	0.47695754	0
B113	B148	FT3	Anti_TPO	-0.1686624	4.57E-12
B113	B149	FT3	TGAb	-0.1518481	4.97E-10
B113	B114	FT3	TSH TT3 TT4(TT4)	0.13572553	2.81E-08
B113	B165	FT3	bilirubin	0.125624	2.80E-07
B114	B115	TSH TT3 TT4(TT4)	TSH TT3 TT4(TT3)	0.21064944	0
B114	B185	TSH TT3 TT4(TT4)	PT	0.16052999	4.70E-11
B114	B184	TSH TT3 TT4(TT4)	INR	0.15017374	7.71E-10
B114	B183	TSH TT3 TT4(TT4)	PTA	-0.148593	1.16E-09
B115	B149	TSH TT3 TT4(TT3)	TGAb	-0.1579154	9.69E-11
B115	B148	TSH TT3 TT4(TT3)	Anti_TPO	-0.1490629	1.03E-09
B115	B136	TSH TT3 TT4(TT3)	5'NT	0.12655294	2.28E-07
B116	B117	TT3	TT4	1	0
B116	B149	TT3	TGAb	-0.1660342	9.83E-12
B117	B149	TT4	TGAb	-0.1660342	9.83E-12
B118	B189	CEA1	eGFB	-0.1956132	8.88E-16
B118	B209	CEA1	Smoking	0.15684453	1.30E-10
B118	B190	CEA1	Cre	0.12426915	3.76E-07
B119	B120	Hcv-rna	HCV-cAg	0.46415872	0
B119	B121	Hcv-rna	C3	-0.8446383	0
B119	B122	Hcv-rna	C4	-0.8446403	0
B119	B124	Hcv-rna	ACTH	0.27490152	0
B119	B137	Hcv-rna	AFU	0.28984077	0
B119	B159	Hcv-rna	β2-MG	-0.8446325	0
B119	B169	Hcv-rna	U-CRY	-0.8446447	0
B119	B138	Hcv-rna	PAB	0.17784475	2.86E-13
B119	B125	Hcv-rna	Cor	0.17164912	1.89E-12
B119	B197	Hcv-rna	AG	-0.1705621	2.61E-12
B119	B195	Hcv-rna	Na	-0.170224	2.88E-12
B119	B198	Hcv-rna	P	-0.1635085	2.03E-11
B119	B199	Hcv-rna	Mg	0.15267172	4.00E-10
B12	B26	PG II	CKMB	0.25070277	0
B12	B80	PG II	anti-HP IgG	0.12484326	3.32E-07
B120	B121	HCV-cAg	C3	-0.392049	0
B120	B122	HCV-cAg	C4	-0.3920502	0

B120	B159	HCV-cAg	β2-MG	-0.3920467	0
B120	B169	HCV-cAg	U-CRY	-0.3920506	0
B120	B137	HCV-cAg	AFU	0.176001	5.04E-13
B121	B159	C3	β2-MG	0.99999606	0
B121	B198	C3	P	0.20598207	0
B121	B137	C3	AFU	-0.1935644	1.78E-15
B121	B197	C3	AG	0.15863487	7.95E-11
B121	B138	C3	PAB	-0.1496715	8.79E-10
B121	B194	C3	Cl	-0.1468888	1.80E-09
B121	B195	C3	Na	0.145285	2.71E-09
B121	B199	C3	Mg	-0.12976	1.12E-07
B121	B124	C3	ACTH	-0.1247748	3.37E-07
B121	B158	C3	IgM	0.12327742	4.66E-07
B122	B159	C4	β2-MG	0.99999854	0
B122	B169	C4	U-CRY	0.99999759	0
B122	B198	C4	P	0.20598257	0
B122	B137	C4	AFU	-0.1935648	1.78E-15
B122	B197	C4	AG	0.15863524	7.95E-11
B122	B138	C4	PAB	-0.1496719	8.79E-10
B122	B194	C4	Cl	-0.1468892	1.80E-09
B122	B195	C4	Na	0.1452853	2.71E-09
B122	B199	C4	Mg	-0.1297603	1.12E-07
B122	B124	C4	ACTH	-0.1247752	3.37E-07
B122	B158	C4	IgM	0.12327771	4.66E-07
B123	B182	hs-CRP1	TT	-0.1544025	2.52E-10
B123	B186	hs-CRP1	PFC	0.14537558	2.65E-09
B124	B125	ACTH	Cor	0.7162258	0
B124	B138	ACTH	PAB	0.23392131	0
B124	B156	ACTH	IgE	0.34882002	0
B124	B137	ACTH	AFU	0.1800311	1.44E-13
B124	B134	ACTH	CHE	0.17422837	8.67E-13
B124	B186	ACTH	PFC	0.17157802	1.93E-12
B124	B182	ACTH	TT	-0.1305272	9.37E-08
B124	B178	ACTH	Ig-U	0.12911021	1.29E-07
B124	B196	ACTH	TCO2	-0.1273804	1.90E-07
B124	B159	ACTH	β2-MG	-0.124774	3.37E-07
B124	B169	ACTH	U-CRY	-0.1247758	3.37E-07
B125	B178	Cor	Ig-U	0.20612019	0
B125	B184	Cor	INR	0.23769401	0
B125	B179	Cor	α1-MG	0.19706423	4.44E-16
B125	B138	Cor	PAB	0.17968426	1.61E-13
B125	B135	Cor	TBA	0.17483294	7.21E-13
B125	B199	Cor	Mg	-0.1652123	1.25E-11
B125	B198	Cor	P	-0.1364226	2.38E-08

B125	B180	Cor	Tf	0.13548544	2.97E-08
B125	B137	Cor	AFU	0.12187326	6.30E-07
B126	B127	Alb	A-G ratio	0.44329327	0
B126	B188	Alb	Ca	0.40099163	0
B126	B189	Alb	eGFB	0.17998443	1.47E-13
B127	B130	A-G ratio	GLB	-0.8983219	0
B128	B131	ALT	AST	0.75778207	0
B128	B140	ALT	r-GT	0.42162058	0
B128	B141	ALT	AST/ALT	-0.5828154	0
B128	B192	ALT	UA	0.25670266	0
B128	B206	ALT	Waist	0.31240565	0
B128	B207	ALT	WHR	0.28200355	0
B128	B210	ALT	Hip	0.2053167	0
B128	B215	ALT	BMI	0.29178949	0
B128	B216	ALT	BMI	0.28344158	0
B128	B222	ALT	NS	0.28410545	0
B128	B204	ALT	Drinking	0.15001302	8.05E-10
B128	B220	ALT	SBP	0.14170685	6.62E-09
B128	B218	ALT	DBP	0.1222007	5.87E-07
B129	B132	IBIL	Dbil	0.68400614	0
B129	B133	IBIL	TBIL	0.98330863	0
B129	B210	IBIL	Hip	-0.1315539	7.41E-08
B130	B188	GLB	Ca	0.12483094	3.33E-07
B131	B140	AST	r-GT	0.37009095	0
B131	B189	AST	eGFB	-0.1844174	3.57E-14
B131	B207	AST	WHR	0.179269	1.83E-13
B131	B206	AST	Waist	0.17864496	2.23E-13
B131	B222	AST	NS	0.15559956	1.82E-10
B131	B220	AST	SBP	0.15519191	2.03E-10
B131	B215	AST	BMI	0.15487939	2.21E-10
B131	B192	AST	UA	0.14221622	5.84E-09
B131	B141	AST	AST/ALT	-0.1422078	5.85E-09
B131	B216	AST	BMI	0.12281652	5.15E-07
B132	B133	Dbil	TBIL	0.80515208	0
B132	B210	Dbil	Hip	-0.157025	1.24E-10
B132	B215	Dbil	BMI	-0.1565175	1.42E-10
B133	B210	TBIL	Hip	-0.1458042	2.38E-09
B134	B136	CHE	5'NT	0.38546351	0
B134	B138	CHE	PAB	0.34423965	0
B134	B150	CHE	ALP	0.27536691	0
B134	B193	CHE	K	0.1663667	8.93E-12
B134	B152	CHE	ACA-Ig(G-A-M)	0.15267483	3.99E-10
B134	B196	CHE	TCO2	-0.1360788	2.58E-08
B134	B137	CHE	AFU	0.12392262	4.06E-07

B135	B179	TBA	α1-MG	0.2005377	0
B135	B138	TBA	PAB	0.18955785	6.66E-15
B135	B178	TBA	Ig-U	0.17747746	3.20E-13
B135	B180	TBA	Tf	0.17299896	1.26E-12
B135	B184	TBA	INR	0.17266041	1.39E-12
B135	B152	TBA	ACA-Ig(G-A-M)	0.15787505	9.80E-11
B135	B182	TBA	TT	0.14796166	1.37E-09
B135	B146	TBA	CysC	0.13438664	3.85E-08
B136	B138	5'NT	PAB	0.45183805	0
B136	B150	5'NT	ALP	0.21157264	0
B136	B158	5'NT	IgM	0.23370719	0
B136	B194	5'NT	Cl	-0.2328053	0
B136	B197	5'NT	AG	0.26326577	0
B136	B199	5'NT	Mg	0.18253344	6.55E-14
B136	B181	5'NT	APTT	-0.1770606	3.64E-13
B136	B200	5'NT	OP	0.16775007	5.97E-12
B136	B196	5'NT	TCO2	-0.1538344	2.93E-10
B136	B178	5'NT	Ig-U	-0.1301533	1.02E-07
B137	B199	AFU	Mg	0.20873923	0
B137	B159	AFU	β2-MG	-0.1935631	1.78E-15
B137	B169	AFU	U-CRY	-0.1935659	1.78E-15
B137	B156	AFU	IgE	0.12932412	1.23E-07
B138	B196	PAB	TCO2	-0.1844382	3.55E-14
B138	B197	PAB	AG	0.17592922	5.16E-13
B138	B169	PAB	U-CRY	-0.1496727	8.79E-10
B138	B159	PAB	β2-MG	-0.1496705	8.80E-10
B138	B200	PAB	OP	0.14220554	5.85E-09
B138	B150	PAB	ALP	0.12548806	2.89E-07
B139	B194	ADA	Cl	-0.2738199	0
B139	B157	ADA	IgG	0.18196727	7.84E-14
B139	B155	ADA	IgA	0.17581362	5.34E-13
B139	B156	ADA	IgE	0.1473967	1.58E-09
B139	B197	ADA	AG	0.1417439	6.56E-09
B139	B146	ADA	CysC	0.13391247	4.30E-08
B140	B141	r-GT	AST/ALT	-0.2263782	0
B140	B206	r-GT	Waist	0.22706987	0
B140	B207	r-GT	WHR	0.20669139	0
B140	B215	r-GT	BMI	0.20147701	0
B140	B216	r-GT	BMI	0.20757204	0
B140	B222	r-GT	NS	0.22381615	0
B140	B192	r-GT	UA	0.17330042	1.15E-12
B140	B220	r-GT	SBP	0.15157381	5.34E-10
B140	B210	r-GT	Hip	0.15146866	5.49E-10
B140	B218	r-GT	DBP	0.13277535	5.59E-08

B140	B166	r-GT	Pro	0.1302435	9.99E-08
B140	B153	r-GT	Glu	0.12025963	8.86E-07
B141	B192	AST/ALT	UA	-0.2167204	0
B141	B206	AST/ALT	Waist	-0.2860861	0
B141	B207	AST/ALT	WHR	-0.2552961	0
B141	B215	AST/ALT	BMI	-0.2737384	0
B141	B216	AST/ALT	BMI	-0.2650887	0
B141	B222	AST/ALT	NS	-0.2362241	0
B141	B210	AST/ALT	Hip	-0.1987911	4.44E-16
B141	B153	AST/ALT	Glu	-0.1270299	2.05E-07
B142	B143	25-(OH)D	β-CTx	-0.2344676	0
B142	B144	25-(OH)D	Total-PINP	-0.2117557	0
B142	B151	25-(OH)D	Anti-CCP	-0.2711152	0
B143	B144	β-CTx	Total-PINP	0.75244221	0
B143	B145	β-CTx	BGP	0.3875566	0
B144	B145	Total-PINP	BGP	0.47539046	0
B146	B189	CysC	eGFB	-0.193011	2.22E-15
B148	B149	Anti_TPO	TGAb	0.51298912	0
B15	B101	CD3-CD19+(B cell)	anti-Sc70	0.68607375	0
B15	B102	CD3-CD19+(B cell)	anti-Sm	0.68607372	0
B15	B105	CD3-CD19+(B cell)	anti-U1RNP	0.68607373	0
B15	B107	CD3-CD19+(B cell)	AnuA	0.68607325	0
B15	B108	CD3-CD19+(B cell)	Anti-Cyclin	0.68607373	0
B15	B125	CD3-CD19+(B cell)	Cor	0.27874228	0
B15	B151	CD3-CD19+(B cell)	Anti-CCP	0.22904968	0
B15	B154	CD3-CD19+(B cell)	RF	-0.3821013	0
B15	B156	CD3-CD19+(B cell)	IgE	0.23808324	0
B15	B157	CD3-CD19+(B cell)	IgG	0.25580479	0
B15	B16	CD3-CD19+(B cell)	CD3+T	0.22107974	0
B15	B17	CD3-CD19+(B cell)	CD3+CD16+CD56+NK	0.54898906	0
B15	B181	CD3-CD19+(B cell)	APTT	-0.3211687	0
B15	B19	CD3-CD19+(B cell)	CD3+CD4+/CD3+CD8+	-0.2489407	0
B15	B20	CD3-CD19+(B cell)	CD3+CD4+CD8+	-0.3255677	0
B15	B22	CD3-CD19+(B cell)	CD3+CD8+(T)	0.49604469	0
B15	B23	CD3-CD19+(B cell)	CD3-CD16+CD56+(NK)	-0.4664635	0
B15	B79	CD3-CD19+(B cell)	hb V -dna	0.2744815	0
B15	B92	CD3-CD19+(B cell)	ALDH2-Glu504Glu	-0.2088476	0
B15	B94	CD3-CD19+(B cell)	ALDH2-Lys504Lys	0.31321827	0
B15	B97	CD3-CD19+(B cell)	dsDNA	0.68607373	0
B15	B98	CD3-CD19+(B cell)	anti-Jo-1	0.68607375	0
B15	B99	CD3-CD19+(B cell)	anti-PM-Scl	0.68607374	0
B15	B93	CD3-CD19+(B cell)	ALDH2-Glu504Lys	0.19453401	1.33E-15
B15	B139	CD3-CD19+(B cell)	ADA	0.19232211	2.66E-15
B15	B81	CD3-CD19+(B cell)	TSGF	-0.174058	9.13E-13

B15	B155	CD3-CD19+(B cell)	IgA	0.16077006	4.39E-11
B15	B135	CD3-CD19+(B cell)	TBA	0.15984746	5.68E-11
B15	B121	CD3-CD19+(B cell)	C3	-0.1555827	1.83E-10
B15	B122	CD3-CD19+(B cell)	C4	-0.1555831	1.83E-10
B15	B159	CD3-CD19+(B cell)	β2-MG	-0.1555816	1.83E-10
B15	B169	CD3-CD19+(B cell)	U-CRY	-0.1555839	1.83E-10
B15	B158	CD3-CD19+(B cell)	IgM	0.15520381	2.03E-10
B15	B180	CD3-CD19+(B cell)	Tf	0.1547707	2.28E-10
B15	B124	CD3-CD19+(B cell)	ACTH	0.13291003	5.42E-08
B15	B21	CD3-CD19+(B cell)	CD3+CD4-CD8-	0.13224869	6.32E-08
B15	B119	CD3-CD19+(B cell)	Hcv-rna	0.13006042	1.04E-07
B15	B109	CD3-CD19+(B cell)	AMA-M2	0.12123064	7.22E-07
B151	B155	Anti-CCP	IgA	0.13274621	5.63E-08
B153	B170	Glu	U-GLU	0.25446642	0
B153	B220	Glu	SBP	0.21926049	0
B153	B206	Glu	Waist	0.18354816	4.73E-14
B153	B215	Glu	BMI	0.1772303	3.46E-13
B153	B207	Glu	WHR	0.1641641	1.68E-11
B153	B189	Glu	eGFB	-0.1378502	1.69E-08
B153	B222	Glu	NS	0.1286131	1.44E-07
B153	B218	Glu	DBP	0.12777927	1.74E-07
B153	B216	Glu	BMI	0.12770034	1.77E-07
B153	B210	Glu	Hip	0.12643148	2.35E-07
B154	B157	RF	IgG	-0.1869768	1.55E-14
B155	B157	IgA	IgG	0.22446037	0
B155	B158	IgA	IgM	0.14418595	3.57E-09
B155	B185	IgA	PT	0.12377331	4.19E-07
B156	B186	IgE	PFC	0.22805413	0
B156	B157	IgE	IgG	0.19026357	5.33E-15
B156	B182	IgE	TT	-0.1783868	2.42E-13
B157	B158	IgG	IgM	0.17608389	4.92E-13
B157	B198	IgG	P	0.12021953	8.94E-07
B158	B186	IgM	PFC	0.15027247	7.52E-10
B158	B181	IgM	APTT	-0.126161	2.49E-07
B158	B159	IgM	β2-MG	0.12327657	4.66E-07
B158	B169	IgM	U-CRY	0.12327834	4.66E-07
B159	B169	β2-MG	U-CRY	0.99998655	0
B159	B198	β2-MG	P	0.20598064	0
B159	B197	β2-MG	AG	0.15863377	7.95E-11
B159	B194	β2-MG	Cl	-0.1468877	1.80E-09
B159	B195	β2-MG	Na	0.14528391	2.71E-09
B159	B199	β2-MG	Mg	-0.1297591	1.12E-07
B16	B125	CD3+T	Cor	0.22845464	0
B16	B18	CD3+T	CD3+CD4+helper(inducer)T	0.83173009	0

B16	B181	CD3+T	APTT	-0.2887294	0
B16	B182	CD3+T	TT	-0.2364736	0
B16	B19	CD3+T	CD3+CD4+/CD3+CD8+	0.27711508	0
B16	B193	CD3+T	K	-0.2073335	0
B16	B20	CD3+T	CD3+CD4+CD8+	0.38236656	0
B16	B21	CD3+T	CD3+CD4-CD8-	-0.5489844	0
B16	B22	CD3+T	CD3+CD8+(T)	0.27411402	0
B16	B23	CD3+T	CD3-CD16+CD56+(NK)	-0.9431253	0
B16	B92	CD3+T	ALDH2-Glu504Glu	0.26987876	0
B16	B93	CD3+T	ALDH2-Glu504Lys	-0.275253	0
B16	B199	CD3+T	Mg	-0.1853228	2.66E-14
B16	B124	CD3+T	ACTH	0.1688048	4.38E-12
B16	B137	CD3+T	AFU	-0.1529407	3.72E-10
B16	B155	CD3+T	IgA	0.14782886	1.42E-09
B16	B81	CD3+T	TSGF	-0.1409645	7.95E-09
B16	B103	CD3+T	anti-SSA/Ro	-0.1328397	5.51E-08
B16	B198	CD3+T	P	-0.1297596	1.12E-07
B16	B121	CD3+T	C3	0.12429716	3.74E-07
B16	B122	CD3+T	C4	0.12429742	3.74E-07
B16	B159	CD3+T	β2-MG	0.12429628	3.74E-07
B16	B169	CD3+T	U-CRY	0.12429797	3.74E-07
B16	B123	CD3+T	hs-CRP1	0.12358195	4.37E-07
B16	B151	CD3+T	Anti-CCP	0.12212929	5.96E-07
B16	B179	CD3+T	α1-MG	0.12004249	9.27E-07
B160	B191	PH	Urea	-0.1491062	1.02E-09
B160	B163	PH	SG	-0.1391751	1.23E-08
B160	B192	PH	UA	-0.122748	5.22E-07
B161	B173	LEU	NTT	0.14966118	8.82E-10
B163	B165	SG	bilirubin	0.24317059	0
B163	B166	SG	Pro	0.38615875	0
B163	B176	SG	Ucr	0.25809519	0
B163	B191	SG	Urea	0.23945331	0
B163	B222	SG	NS	0.23067029	0
B163	B192	SG	UA	0.20016109	2.22E-16
B163	B216	SG	BMI	0.19605707	8.88E-16
B163	B221	SG	Hei	0.17495834	6.94E-13
B163	B190	SG	Cre	0.15593696	1.66E-10
B163	B170	SG	U-GLU	0.14640215	2.04E-09
B163	B206	SG	Waist	0.14140143	7.14E-09
B163	B207	SG	WHR	0.13660341	2.28E-08
B163	B209	SG	Smoking	0.13362513	4.59E-08
B165	B192	bilirubin	UA	0.12041241	8.58E-07
B166	B222	Pro	NS	0.12913172	1.28E-07
B166	B192	Pro	UA	0.12377949	4.18E-07

B167	B223	PC	FH	0.7144725	0
B169	B198	U-CRY	P	0.20598362	0
B169	B197	U-CRY	AG	0.15863605	7.95E-11
B169	B194	U-CRY	Cl	-0.14689	1.80E-09
B169	B195	U-CRY	Na	0.14528582	2.71E-09
B169	B199	U-CRY	Mg	-0.1297609	1.11E-07
B17	B101	CD3+CD16+CD56+NK	anti-Sc70	0.39230095	0
B17	B102	CD3+CD16+CD56+NK	anti-Sm	0.39230094	0
B17	B105	CD3+CD16+CD56+NK	anti-U1RNP	0.39230095	0
B17	B107	CD3+CD16+CD56+NK	AnuA	0.39230068	0
B17	B108	CD3+CD16+CD56+NK	Anti-Cyclin	0.39230095	0
B17	B110	CD3+CD16+CD56+NK	AHA	-0.2129242	0
B17	B154	CD3+CD16+CD56+NK	RF	-0.3823145	0
B17	B157	CD3+CD16+CD56+NK	IgG	0.24330987	0
B17	B158	CD3+CD16+CD56+NK	IgM	0.25456847	0
B17	B18	CD3+CD16+CD56+NK	CD3+CD4+helper(inducer)T	-0.2164953	0
B17	B181	CD3+CD16+CD56+NK	APTT	-0.2714965	0
B17	B183	CD3+CD16+CD56+NK	PTA	-0.2653238	0
B17	B19	CD3+CD16+CD56+NK	CD3+CD4+/CD3+CD8+	-0.207906	0
B17	B21	CD3+CD16+CD56+NK	CD3+CD4-CD8-	0.5053338	0
B17	B22	CD3+CD16+CD56+NK	CD3+CD8+(T)	0.27752058	0
B17	B79	CD3+CD16+CD56+NK	hb V -dna	0.24014783	0
B17	B94	CD3+CD16+CD56+NK	ALDH2-Lys504Lys	0.32890827	0
B17	B97	CD3+CD16+CD56+NK	dsDNA	0.39230095	0
B17	B98	CD3+CD16+CD56+NK	anti-Jo-1	0.39230096	0
B17	B99	CD3+CD16+CD56+NK	anti-PM-Scl	0.39230095	0
B17	B23	CD3+CD16+CD56+NK	CD3-CD16+CD56+(NK)	-0.1965728	6.66E-16
B17	B180	CD3+CD16+CD56+NK	Tf	0.1843055	3.71E-14
B17	B185	CD3+CD16+CD56+NK	PT	-0.1784952	2.34E-13
B17	B198	CD3+CD16+CD56+NK	P	0.17148247	1.98E-12
B17	B176	CD3+CD16+CD56+NK	Ucr	-0.1607652	4.40E-11
B17	B151	CD3+CD16+CD56+NK	Anti-CCP	0.13811787	1.59E-08
B17	B184	CD3+CD16+CD56+NK	INR	-0.1329725	5.34E-08
B17	B195	CD3+CD16+CD56+NK	Na	-0.1295715	1.16E-07
B172	B184	KB	INR	0.24815098	0
B172	B185	KB	PT	0.23891924	0
B172	B182	KB	TT	0.17339109	1.12E-12
B172	B186	KB	PFC	-0.1552569	2.00E-10
B174	B189	Urea/Cr	eGFB	0.20199201	0
B174	B190	Urea/Cr	Cre	-0.297067	0
B174	B191	Urea/Cr	Urea	0.67017031	0
B174	B192	Urea/Cr	UA	-0.2006114	0
B174	B221	Urea/Cr	Hei	-0.209151	0
B174	B222	Urea/Cr	NS	-0.1796417	1.63E-13

B174	B216	Urea/Cr	BMI	-0.1481332	1.31E-09
B175	B176	ALB-U	Ucr	0.24162513	0
B175	B177	ALB-U	ACR	0.95123285	0
B178	B179	Ig-U	α1-MG	0.91023718	0
B178	B180	Ig-U	Tf	0.81852579	0
B179	B180	α1-MG	Tf	0.83172071	0
B179	B194	α1-MG	Cl	0.14185911	6.38E-09
B179	B184	α1-MG	INR	0.13444585	3.79E-08
B18	B101	CD3+CD4+helper(inducer)T	anti-Sc70	-0.2490083	0
B18	B102	CD3+CD4+helper(inducer)T	anti-Sm	-0.2490083	0
B18	B105	CD3+CD4+helper(inducer)T	anti-U1RNP	-0.2490083	0
B18	B107	CD3+CD4+helper(inducer)T	AnuA	-0.2490081	0
B18	B108	CD3+CD4+helper(inducer)T	Anti-Cyclin	-0.2490083	0
B18	B154	CD3+CD4+helper(inducer)T	RF	0.20885673	0
B18	B182	CD3+CD4+helper(inducer)T	TT	-0.2521065	0
B18	B19	CD3+CD4+helper(inducer)T	CD3+CD4+/CD3+CD8+	0.70927038	0
B18	B20	CD3+CD4+helper(inducer)T	CD3+CD4+CD8+	0.3555002	0
B18	B21	CD3+CD4+helper(inducer)T	CD3+CD4-CD8-	-0.5792123	0
B18	B22	CD3+CD4+helper(inducer)T	CD3+CD8+(T)	-0.2339493	0
B18	B23	CD3+CD4+helper(inducer)T	CD3-CD16+CD56+(NK)	-0.7459191	0
B18	B92	CD3+CD4+helper(inducer)T	ALDH2-Glu504Glu	0.26552008	0
B18	B93	CD3+CD4+helper(inducer)T	ALDH2-Glu504Lys	-0.2587733	0
B18	B94	CD3+CD4+helper(inducer)T	ALDH2-Lys504Lys	-0.218704	0
B18	B97	CD3+CD4+helper(inducer)T	dsDNA	-0.2490083	0
B18	B98	CD3+CD4+helper(inducer)T	anti-Jo-1	-0.2490083	0
B18	B99	CD3+CD4+helper(inducer)T	anti-PM-Scl	-0.2490083	0
B18	B110	CD3+CD4+helper(inducer)T	AHA	0.19792037	4.44E-16
B18	B79	CD3+CD4+helper(inducer)T	hb V -dna	-0.1938597	1.55E-15
B18	B181	CD3+CD4+helper(inducer)T	APTT	-0.1828706	5.86E-14
B18	B137	CD3+CD4+helper(inducer)T	AFU	-0.1731982	1.18E-12
B18	B193	CD3+CD4+helper(inducer)T	K	-0.1726687	1.39E-12
B18	B121	CD3+CD4+helper(inducer)T	C3	0.1571251	1.20E-10
B18	B122	CD3+CD4+helper(inducer)T	C4	0.15712546	1.20E-10
B18	B159	CD3+CD4+helper(inducer)T	β2-MG	0.15712402	1.20E-10
B18	B169	CD3+CD4+helper(inducer)T	U-CRY	0.15712627	1.20E-10
B18	B180	CD3+CD4+helper(inducer)T	Tf	-0.1505544	6.98E-10
B18	B194	CD3+CD4+helper(inducer)T	Cl	0.14208777	6.03E-09
B18	B58	CD3+CD4+helper(inducer)T	HCT	-0.1418406	6.41E-09
B18	B67	CD3+CD4+helper(inducer)T	WBRSR(mPas_50)	-0.1359838	2.64E-08
B18	B66	CD3+CD4+helper(inducer)T	WBRSR(mPas_100)	-0.1347897	3.50E-08
B18	B158	CD3+CD4+helper(inducer)T	IgM	-0.1335538	4.67E-08
B18	B64	CD3+CD4+helper(inducer)T	WBRSR(mPas_5)	-0.1333288	4.92E-08
B18	B119	CD3+CD4+helper(inducer)T	Hcv-rna	-0.1324646	6.01E-08
B18	B134	CD3+CD4+helper(inducer)T	CHE	-0.1316451	7.26E-08

B18	B62	CD3+CD4+helper(inducer)T	WBHSRVI	-0.1315191	7.47E-08
B18	B60	CD3+CD4+helper(inducer)T	WBRVI	-0.1290275	1.32E-07
B18	B63	CD3+CD4+helper(inducer)T	WBRSR(mPas_200)	-0.1277382	1.76E-07
B18	B65	CD3+CD4+helper(inducer)T	WBRSR(mPas_1)	-0.127401	1.89E-07
B18	B123	CD3+CD4+helper(inducer)T	hs-CRP1	0.12537802	2.96E-07
B18	B139	CD3+CD4+helper(inducer)T	ADA	-0.1228832	5.08E-07
B180	B193	Tf	K	0.13640703	2.39E-08
B181	B183	APTT	PTA	0.2596008	0
B181	B184	APTT	INR	0.2258926	0
B181	B185	APTT	PT	0.3180133	0
B181	B186	APTT	PFC	-0.3325534	0
B181	B193	APTT	K	0.15080935	6.53E-10
B181	B182	APTT	TT	0.13098257	8.45E-08
B182	B184	TT	INR	0.60046692	0
B182	B185	TT	PT	0.55537741	0
B182	B186	TT	PFC	-0.5722458	0
B183	B193	PTA	K	0.15886036	7.47E-11
B184	B185	INR	PT	0.91166924	0
B184	B186	INR	PFC	-0.529424	0
B184	B199	INR	Mg	-0.145119	2.83E-09
B185	B186	PT	PFC	-0.4293931	0
B185	B194	PT	Cl	-0.1269233	2.10E-07
B188	B192	Ca	UA	0.1255809	2.83E-07
B189	B190	eGFB	Cre	-0.5104604	0
B189	B191	eGFB	Urea	-0.3514174	0
B189	B192	eGFB	UA	-0.2812525	0
B189	B206	eGFB	Waist	-0.249505	0
B189	B207	eGFB	WHR	-0.2844988	0
B189	B220	eGFB	SBP	-0.318098	0
B189	B222	eGFB	NS	-0.1742918	8.50E-13
B189	B219	eGFB	OT	-0.1540347	2.78E-10
B189	B215	eGFB	BMI	-0.1534254	3.27E-10
B189	B227	eGFB	OD	0.14160343	6.79E-09
B189	B201	eGFB	e-habits	-0.1364556	2.36E-08
B189	B216	eGFB	BMI	-0.1351661	3.20E-08
B189	B209	eGFB	Smoking	-0.1348556	3.44E-08
B189	B218	eGFB	DBP	-0.1337971	4.41E-08
B19	B101	CD3+CD4+/CD3+CD8+	anti-Sc70	-0.3766767	0
B19	B102	CD3+CD4+/CD3+CD8+	anti-Sm	-0.3766767	0
B19	B103	CD3+CD4+/CD3+CD8+	anti-SSA/Ro	0.20841984	0
B19	B105	CD3+CD4+/CD3+CD8+	anti-U1RNP	-0.3766767	0
B19	B107	CD3+CD4+/CD3+CD8+	AnuA	-0.3766764	0
B19	B108	CD3+CD4+/CD3+CD8+	Anti-Cyclin	-0.3766767	0
B19	B124	CD3+CD4+/CD3+CD8+	ACTH	-0.2809772	0

B19	B125	CD3+CD4+/CD3+CD8+	Cor	-0.4470642	0
B19	B182	CD3+CD4+/CD3+CD8+	TT	-0.2088825	0
B19	B184	CD3+CD4+/CD3+CD8+	INR	-0.2466338	0
B19	B22	CD3+CD4+/CD3+CD8+	CD3+CD8+(T)	-0.8014754	0
B19	B23	CD3+CD4+/CD3+CD8+	CD3-CD16+CD56+(NK)	-0.2720074	0
B19	B60	CD3+CD4+/CD3+CD8+	WBRVI	-0.2018404	0
B19	B61	CD3+CD4+/CD3+CD8+	WBHSRV	-0.2170218	0
B19	B62	CD3+CD4+/CD3+CD8+	WBHSRVI	-0.2549625	0
B19	B63	CD3+CD4+/CD3+CD8+	WBRSR(mPas_200)	-0.2539727	0
B19	B64	CD3+CD4+/CD3+CD8+	WBRSR(mPas_5)	-0.2283135	0
B19	B65	CD3+CD4+/CD3+CD8+	WBRSR(mPas_1)	-0.2037966	0
B19	B66	CD3+CD4+/CD3+CD8+	WBRSR(mPas_100)	-0.252677	0
B19	B67	CD3+CD4+/CD3+CD8+	WBRSR(mPas_50)	-0.250167	0
B19	B79	CD3+CD4+/CD3+CD8+	hb V -dna	-0.3398078	0
B19	B81	CD3+CD4+/CD3+CD8+	TSGF	0.26057891	0
B19	B92	CD3+CD4+/CD3+CD8+	ALDH2-Glu504Glu	0.26478486	0
B19	B93	CD3+CD4+/CD3+CD8+	ALDH2-Glu504Lys	-0.2558123	0
B19	B94	CD3+CD4+/CD3+CD8+	ALDH2-Lys504Lys	-0.2532826	0
B19	B97	CD3+CD4+/CD3+CD8+	dsDNA	-0.3766767	0
B19	B98	CD3+CD4+/CD3+CD8+	anti-Jo-1	-0.3766767	0
B19	B99	CD3+CD4+/CD3+CD8+	anti-PM-Scl	-0.3766767	0
B19	B56	CD3+CD4+/CD3+CD8+	IR	-0.1987692	4.44E-16
B19	B110	CD3+CD4+/CD3+CD8+	AHA	0.19561198	8.88E-16
B19	B139	CD3+CD4+/CD3+CD8+	ADA	-0.1848643	3.11E-14
B19	B156	CD3+CD4+/CD3+CD8+	IgE	-0.1820605	7.59E-14
B19	B135	CD3+CD4+/CD3+CD8+	TBA	-0.1769752	3.74E-13
B19	B180	CD3+CD4+/CD3+CD8+	Tf	-0.1722681	1.57E-12
B19	B58	CD3+CD4+/CD3+CD8+	HCT	-0.1638544	1.84E-11
B19	B154	CD3+CD4+/CD3+CD8+	RF	0.161248	3.84E-11
B19	B54	CD3+CD4+/CD3+CD8+	ESR	0.15166946	5.21E-10
B19	B186	CD3+CD4+/CD3+CD8+	PFC	-0.1465907	1.95E-09
B19	B185	CD3+CD4+/CD3+CD8+	PT	-0.1411652	7.57E-09
B19	B158	CD3+CD4+/CD3+CD8+	IgM	-0.1398954	1.03E-08
B19	B194	CD3+CD4+/CD3+CD8+	Cl	0.13742147	1.88E-08
B19	B183	CD3+CD4+/CD3+CD8+	PTA	-0.1354881	2.97E-08
B19	B176	CD3+CD4+/CD3+CD8+	Ucr	0.13237712	6.13E-08
B19	B138	CD3+CD4+/CD3+CD8+	PAB	-0.1296882	1.13E-07
B19	B178	CD3+CD4+/CD3+CD8+	Ig-U	-0.1257828	2.71E-07
B190	B191	Cre	Urea	0.36251539	0
B190	B192	Cre	UA	0.29951046	0
B190	B221	Cre	Hei	0.20424686	0
B190	B222	Cre	NS	0.19710431	4.44E-16
B190	B207	Cre	WHR	0.19272671	2.22E-15
B190	B206	Cre	Waist	0.18019546	1.37E-13

B190	B216	Cre	BMI	0.17528842	6.27E-13
B190	B209	Cre	Smoking	0.14881289	1.10E-09
B190	B204	Cre	Drinking	0.13190647	6.84E-08
B191	B207	Urea	WHR	0.17178095	1.81E-12
B191	B192	Urea	UA	0.16536293	1.19E-11
B191	B206	Urea	Waist	0.13968118	1.09E-08
B191	B220	Urea	SBP	0.12830593	1.55E-07
B192	B204	UA	Drinking	0.25408153	0
B192	B206	UA	Waist	0.40844848	0
B192	B207	UA	WHR	0.37303285	0
B192	B209	UA	Smoking	0.21173004	0
B192	B210	UA	Hip	0.26899673	0
B192	B215	UA	BMI	0.32737937	0
B192	B216	UA	BMI	0.41380461	0
B192	B221	UA	Hei	0.24620425	0
B192	B222	UA	NS	0.43409989	0
B192	B220	UA	SBP	0.15287483	3.79E-10
B192	B218	UA	DBP	0.15282335	3.84E-10
B193	B198	K	P	0.234722	0
B194	B197	Cl	AG	-0.4469758	0
B194	B198	Cl	P	0.19987365	2.22E-16
B194	B200	Cl	OP	0.14093205	8.01E-09
B194	B195	Cl	Na	0.11970862	9.95E-07
B195	B197	Na	AG	0.5228123	0
B195	B199	Na	Mg	0.24124103	0
B195	B200	Na	OP	0.93268589	0
B196	B197	TCO2	AG	-0.7124255	0
B196	B198	TCO2	P	0.13291871	5.41E-08
B196	B200	TCO2	OP	-0.1248134	3.34E-07
B197	B200	AG	OP	0.50093376	0
B197	B198	AG	P	-0.1507546	6.63E-10
B198	B199	P	Mg	0.16646766	8.67E-12
B198	B200	P	OP	0.12580041	2.69E-07
B199	B200	Mg	OP	0.22541906	0
B2	B118	Age	CEA1	0.23330884	0
B2	B126	Age	Alb	-0.2347371	0
B2	B131	Age	AST	0.21198826	0
B2	B153	Age	Glu	0.21592789	0
B2	B189	Age	eGFB	-0.7026477	0
B2	B191	Age	Urea	0.25907329	0
B2	B206	Age	Waist	0.31243783	0
B2	B207	Age	WHR	0.36671545	0
B2	B215	Age	BMI	0.20533556	0
B2	B219	Age	OT	0.25046376	0

B2	B220	Age	SBP	0.44748115	0
B2	B227	Age	OD	-0.2168498	0
B2	B47	Age	RDW	0.21934585	0
B2	B77	Age	TC	0.26450423	0
B2	B70	Age	LDL-C	0.18746997	1.33E-14
B2	B43	Age	MCV	0.18394795	4.17E-14
B2	B52	Age	PLT/BPC	-0.1771751	3.52E-13
B2	B222	Age	NS	0.1679627	5.61E-12
B2	B146	Age	CysC	0.16708907	7.24E-12
B2	B190	Age	Cre	0.16612201	9.58E-12
B2	B44	Age	MCH	0.16390638	1.81E-11
B2	B218	Age	DBP	0.15990331	5.59E-11
B2	B201	Age	e-habits	0.15882785	7.54E-11
B2	B91	Age	TP	-0.1537764	2.98E-10
B2	B221	Age	Hei	-0.1483478	1.24E-09
B2	B27	Age	LDH	0.1366668	2.25E-08
B2	B71	Age	TG	0.13506117	3.28E-08
B2	B192	Age	UA	0.13317816	5.10E-08
B2	B53	Age	GR%	-0.1292266	1.26E-07
B2	B224	Age	pmh	0.12840583	1.51E-07
B2	B205	Age	KDA	0.12405589	3.94E-07
B20	B119	CD3+CD4+CD8+	Hcv-rna	-0.35532	0
B20	B121	CD3+CD4+CD8+	C3	0.42068475	0
B20	B122	CD3+CD4+CD8+	C4	0.42068574	0
B20	B159	CD3+CD4+CD8+	β2-MG	0.42068185	0
B20	B169	CD3+CD4+CD8+	U-CRY	0.42068791	0
B20	B21	CD3+CD4+CD8+	CD3+CD4-CD8-	-0.3885244	0
B20	B22	CD3+CD4+CD8+	CD3+CD8+(T)	0.28043463	0
B20	B23	CD3+CD4+CD8+	CD3-CD16+CD56+(NK)	-0.2176143	0
B20	B101	CD3+CD4+CD8+	anti-Sc70	-0.1993207	2.22E-16
B20	B102	CD3+CD4+CD8+	anti-Sm	-0.1993207	2.22E-16
B20	B105	CD3+CD4+CD8+	anti-U1RNP	-0.1993207	2.22E-16
B20	B107	CD3+CD4+CD8+	AnuA	-0.1993206	2.22E-16
B20	B108	CD3+CD4+CD8+	Anti-Cyclin	-0.1993207	2.22E-16
B20	B97	CD3+CD4+CD8+	dsDNA	-0.1993207	2.22E-16
B20	B98	CD3+CD4+CD8+	anti-Jo-1	-0.1993207	2.22E-16
B20	B99	CD3+CD4+CD8+	anti-PM-Scl	-0.1993207	2.22E-16
B20	B120	CD3+CD4+CD8+	HCV-cAg	-0.1648898	1.37E-11
B20	B195	CD3+CD4+CD8+	Na	0.16161289	3.47E-11
B20	B200	CD3+CD4+CD8+	OP	0.15040277	7.27E-10
B20	B82	CD3+CD4+CD8+	CEA	-0.1381712	1.57E-08
B20	B125	CD3+CD4+CD8+	Cor	0.13707246	2.04E-08
B20	B211	CD3+CD4+CD8+	IOP-R	-0.1310827	8.26E-08
B20	B193	CD3+CD4+CD8+	K	-0.1304016	9.64E-08

B20	B150	CD3+CD4+CD8+	ALP	-0.1271455	2.00E-07
B202	B223	e-times	FH	0.55316819	0
B204	B206	Drinking	Waist	0.23636881	0
B204	B207	Drinking	WHR	0.21517746	0
B204	B209	Drinking	Smoking	0.39260708	0
B204	B216	Drinking	BMI	0.29967354	0
B204	B221	Drinking	Hei	0.28766813	0
B204	B222	Drinking	NS	0.36235843	0
B204	B210	Drinking	Hip	0.14969493	8.74E-10
B204	B215	Drinking	BMI	0.14885704	1.09E-09
B206	B207	Waist	WHR	0.84733896	0
B206	B210	Waist	Hip	0.75456699	0
B206	B215	Waist	BMI	0.80663512	0
B206	B216	Waist	BMI	0.80867433	0
B206	B218	Waist	DBP	0.24041706	0
B206	B220	Waist	SBP	0.30103419	0
B206	B221	Waist	Hei	0.26945363	0
B206	B222	Waist	NS	0.73499351	0
B206	B209	Waist	Smoking	0.17946521	1.73E-13
B207	B210	WHR	Hip	0.29529312	0
B207	B215	WHR	BMI	0.55197834	0
B207	B216	WHR	BMI	0.53007933	0
B207	B220	WHR	SBP	0.27374197	0
B207	B222	WHR	NS	0.58570468	0
B207	B218	WHR	DBP	0.19239514	2.66E-15
B207	B209	WHR	Smoking	0.17729491	3.39E-13
B207	B221	WHR	Hei	0.13708164	2.03E-08
B209	B216	Smoking	BMI	0.2129169	0
B209	B221	Smoking	Hei	0.2570443	0
B209	B222	Smoking	NS	0.30425923	0
B21	B125	CD3+CD4-CD8-	Cor	-0.2154162	0
B21	B154	CD3+CD4-CD8-	RF	-0.4944637	0
B21	B22	CD3+CD4-CD8-	CD3+CD8+(T)	-0.2237589	0
B21	B23	CD3+CD4-CD8-	CD3-CD16+CD56+(NK)	0.41984298	0
B21	B94	CD3+CD4-CD8-	ALDH2-Lys504Lys	0.1854506	2.58E-14
B21	B121	CD3+CD4-CD8-	C3	-0.1824873	6.64E-14
B21	B122	CD3+CD4-CD8-	C4	-0.1824877	6.64E-14
B21	B169	CD3+CD4-CD8-	U-CRY	-0.1824887	6.64E-14
B21	B159	CD3+CD4-CD8-	β2-MG	-0.1824861	6.66E-14
B21	B103	CD3+CD4-CD8-	anti-SSA/Ro	0.17367892	1.02E-12
B21	B93	CD3+CD4-CD8-	ALDH2-Glu504Lys	-0.1549293	2.18E-10
B21	B158	CD3+CD4-CD8-	IgM	0.15427737	2.60E-10
B21	B119	CD3+CD4-CD8-	Hcv-rna	0.15415878	2.69E-10
B21	B185	CD3+CD4-CD8-	PT	-0.14756	1.52E-09

B21	B199	CD3+CD4-CD8-	Mg	0.14749427	1.54E-09
B21	B76	CD3+CD4-CD8-	ApoB	0.14379419	3.94E-09
B21	B137	CD3+CD4-CD8-	AFU	0.14015812	9.68E-09
B21	B92	CD3+CD4-CD8-	ALDH2-Glu504Glu	0.13931925	1.19E-08
B21	B184	CD3+CD4-CD8-	INR	-0.1375689	1.81E-08
B21	B101	CD3+CD4-CD8-	anti-Sc70	0.13497247	3.35E-08
B21	B102	CD3+CD4-CD8-	anti-Sm	0.13497246	3.35E-08
B21	B105	CD3+CD4-CD8-	anti-U1RNP	0.13497247	3.35E-08
B21	B107	CD3+CD4-CD8-	AnuA	0.13497237	3.35E-08
B21	B108	CD3+CD4-CD8-	Anti-Cyclin	0.13497247	3.35E-08
B21	B97	CD3+CD4-CD8-	dsDNA	0.13497247	3.35E-08
B21	B98	CD3+CD4-CD8-	anti-Jo-1	0.13497247	3.35E-08
B21	B99	CD3+CD4-CD8-	anti-PM-Scl	0.13497247	3.35E-08
B21	B124	CD3+CD4-CD8-	ACTH	-0.1344394	3.80E-08
B21	B193	CD3+CD4-CD8-	K	0.13076749	8.87E-08
B21	B110	CD3+CD4-CD8-	AHA	-0.127386	1.90E-07
B210	B215	Hip	BMI	0.77540194	0
B210	B216	Hip	BMI	0.80062573	0
B210	B220	Hip	SBP	0.20414046	0
B210	B221	Hip	Hei	0.31099488	0
B210	B222	Hip	NS	0.59711062	0
B210	B218	Hip	DBP	0.19192024	3.11E-15
B211	B212	IOP-R	IOP-L	0.80360747	0
B215	B216	BMI	BMI	0.81248972	0
B215	B218	BMI	DBP	0.23199854	0
B215	B220	BMI	SBP	0.28464517	0
B215	B222	BMI	NS	0.62409917	0
B216	B218	BMI	DBP	0.2354885	0
B216	B220	BMI	SBP	0.22370123	0
B216	B221	BMI	Hei	0.5869468	0
B216	B222	BMI	NS	0.77106421	0
B218	B220	DBP	SBP	0.67987439	0
B218	B222	DBP	NS	0.22244743	0
B22	B101	CD3+CD8+(T)	anti-Sc70	0.54445175	0
B22	B102	CD3+CD8+(T)	anti-Sm	0.54445174	0
B22	B103	CD3+CD8+(T)	anti-SSA/Ro	-0.2708374	0
B22	B105	CD3+CD8+(T)	anti-U1RNP	0.54445174	0
B22	B107	CD3+CD8+(T)	AnuA	0.54445136	0
B22	B108	CD3+CD8+(T)	Anti-Cyclin	0.54445175	0
B22	B124	CD3+CD8+(T)	ACTH	0.31369181	0
B22	B125	CD3+CD8+(T)	Cor	0.56655073	0
B22	B135	CD3+CD8+(T)	TBA	0.21874534	0
B22	B139	CD3+CD8+(T)	ADA	0.20612261	0
B22	B156	CD3+CD8+(T)	IgE	0.23751085	0

B22	B178	CD3+CD8+(T)	Ig-U	0.20636177	0
B22	B184	CD3+CD8+(T)	INR	0.3136025	0
B22	B199	CD3+CD8+(T)	Mg	-0.2189041	0
B22	B23	CD3+CD8+(T)	CD3-CD16+CD56+(NK)	-0.2552459	0
B22	B56	CD3+CD8+(T)	IR	0.22036796	0
B22	B61	CD3+CD8+(T)	WBHSRV	0.23672109	0
B22	B62	CD3+CD8+(T)	WBHSRVI	0.22626464	0
B22	B63	CD3+CD8+(T)	WBRSR(mPas_200)	0.2348534	0
B22	B66	CD3+CD8+(T)	WBRSR(mPas_100)	0.22150684	0
B22	B67	CD3+CD8+(T)	WBRSR(mPas_50)	0.21766041	0
B22	B79	CD3+CD8+(T)	hb V -dna	0.38791829	0
B22	B81	CD3+CD8+(T)	TSGF	-0.2973793	0
B22	B94	CD3+CD8+(T)	ALDH2-Lys504Lys	0.36184291	0
B22	B97	CD3+CD8+(T)	dsDNA	0.54445175	0
B22	B98	CD3+CD8+(T)	anti-Jo-1	0.54445176	0
B22	B99	CD3+CD8+(T)	anti-PM-Scl	0.54445175	0
B22	B64	CD3+CD8+(T)	WBRSR(mPas_5)	0.19535705	8.88E-16
B22	B151	CD3+CD8+(T)	Anti-CCP	0.18298007	5.68E-14
B22	B92	CD3+CD8+(T)	ALDH2-Glu504Glu	-0.180599	1.21E-13
B22	B180	CD3+CD8+(T)	Tf	0.1793568	1.79E-13
B22	B110	CD3+CD8+(T)	AHA	-0.1751273	6.59E-13
B22	B65	CD3+CD8+(T)	WBRSR(mPas_1)	0.17016129	2.94E-12
B22	B185	CD3+CD8+(T)	PT	0.16869386	4.53E-12
B22	B60	CD3+CD8+(T)	WBRVI	0.16677528	7.93E-12
B22	B93	CD3+CD8+(T)	ALDH2-Glu504Lys	0.16241715	2.76E-11
B22	B54	CD3+CD8+(T)	ESR	-0.1533458	3.34E-10
B22	B154	CD3+CD8+(T)	RF	-0.1450794	2.85E-09
B22	B59	CD3+CD8+(T)	WBRV	0.13614773	2.54E-08
B22	B179	CD3+CD8+(T)	α1-MG	0.12909632	1.30E-07
B22	B176	CD3+CD8+(T)	Ucr	-0.128163	1.60E-07
B22	B157	CD3+CD8+(T)	IgG	0.12535191	2.97E-07
B22	B194	CD3+CD8+(T)	Cl	-0.1210922	7.43E-07
B22	B186	CD3+CD8+(T)	PFC	0.11974744	9.87E-07
B220	B222	SBP	NS	0.23071909	0
B221	B222	Hei	NS	0.44767716	0
B23	B125	CD3-CD16+CD56+(NK)	Cor	-0.2244492	0
B23	B181	CD3-CD16+CD56+(NK)	APTT	0.40902807	0
B23	B182	CD3-CD16+CD56+(NK)	TT	0.28792473	0
B23	B193	CD3-CD16+CD56+(NK)	K	0.20930706	0
B23	B93	CD3-CD16+CD56+(NK)	ALDH2-Glu504Lys	0.19171153	3.11E-15
B23	B101	CD3-CD16+CD56+(NK)	anti-Sc70	-0.1913519	3.55E-15
B23	B102	CD3-CD16+CD56+(NK)	anti-Sm	-0.1913519	3.55E-15
B23	B105	CD3-CD16+CD56+(NK)	anti-U1RNP	-0.1913519	3.55E-15
B23	B107	CD3-CD16+CD56+(NK)	AnuA	-0.1913517	3.55E-15

B23	B108	CD3-CD16+CD56+(NK)	Anti-Cyclin	-0.1913519	3.55E-15
B23	B97	CD3-CD16+CD56+(NK)	dsDNA	-0.1913519	3.55E-15
B23	B98	CD3-CD16+CD56+(NK)	anti-Jo-1	-0.1913519	3.55E-15
B23	B99	CD3-CD16+CD56+(NK)	anti-PM-Scl	-0.1913519	3.55E-15
B23	B92	CD3-CD16+CD56+(NK)	ALDH2-Glu504Glu	-0.1883342	9.99E-15
B23	B124	CD3-CD16+CD56+(NK)	ACTH	-0.1762374	4.69E-13
B23	B151	CD3-CD16+CD56+(NK)	Anti-CCP	-0.1604408	4.82E-11
B23	B81	CD3-CD16+CD56+(NK)	TSGF	0.15744556	1.10E-10
B23	B155	CD3-CD16+CD56+(NK)	IgA	-0.1410096	7.86E-09
B23	B183	CD3-CD16+CD56+(NK)	PTA	0.13279114	5.57E-08
B23	B137	CD3-CD16+CD56+(NK)	AFU	0.12814204	1.60E-07
B23	B199	CD3-CD16+CD56+(NK)	Mg	0.12556406	2.84E-07
B24	B25	αHBDH	CK	0.37894246	0
B24	B26	αHBDH	CKMB	0.20819467	0
B24	B27	αHBDH	LDH	0.71620766	0
B24	B74	αHBDH	ApoA1	0.24628549	0
B24	B139	αHBDH	ADA	0.17842462	2.39E-13
B24	B58	αHBDH	HCT	-0.1451626	2.79E-09
B24	B62	αHBDH	WBHSRVI	-0.1266801	2.22E-07
B24	B75	αHBDH	ApoA1/B	0.12584779	2.67E-07
B24	B135	αHBDH	TBA	0.12259315	5.40E-07
B24	B73	αHBDH	ApoA	0.12188524	6.28E-07
B24	B146	αHBDH	CysC	0.12121236	7.25E-07
B25	B27	CK	LDH	0.33677399	0
B25	B26	CK	CKMB	0.18468397	3.29E-14
B25	B73	CK	ApoA	0.15178039	5.06E-10
B25	B110	CK	AHA	0.14879016	1.11E-09
B25	B76	CK	ApoB	0.14859236	1.16E-09
B25	B54	CK	ESR	0.13187335	6.89E-08
B25	B75	CK	ApoA1/B	-0.1251203	3.13E-07
B25	B69	CK	PV	0.125099	3.14E-07
B25	B174	CK	Urea/Cr	-0.1241716	3.84E-07
B25	B136	CK	5'NT	0.12378375	4.18E-07
B26	B27	CKMB	LDH	0.16573268	1.07E-11
B27	B74	LDH	ApoA1	0.19968895	2.22E-16
B27	B136	LDH	5'NT	0.17038957	2.74E-12
B27	B197	LDH	AG	0.16620122	9.36E-12
B27	B196	LDH	TCO2	-0.1649933	1.33E-11
B27	B139	LDH	ADA	0.15770432	1.03E-10
B27	B220	LDH	SBP	0.15756077	1.07E-10
B27	B131	LDH	AST	0.15403198	2.78E-10
B27	B150	LDH	ALP	0.14442936	3.36E-09
B27	B189	LDH	eGFB	-0.1369454	2.10E-08
B27	B134	LDH	CHE	0.13030329	9.86E-08

B28	B30	PRL	LH	0.24065489	0
B28	B75	PRL	ApoA1/B	-0.1433276	4.43E-09
B28	B33	PRL	Prog	0.13727069	1.94E-08
B29	B30	E2	LH	-0.5142006	0
B29	B31	E2	FSH	-0.6130249	0
B29	B32	E2	Testo	0.35861151	0
B29	B33	E2	Prog	0.31193541	0
B29	B73	E2	ApoA	-0.1706354	2.55E-12
B29	B194	E2	Cl	-0.1605977	4.61E-11
B29	B198	E2	P	-0.1426122	5.29E-09
B29	B74	E2	ApoA1	-0.1351961	3.18E-08
B29	B136	E2	5'NT	0.12149497	6.83E-07
B3	B146	Hcy	CysC	0.15122392	5.86E-10
B3	B25	Hcy	CK	0.14644839	2.02E-09
B3	B24	Hcy	αHBDH	0.14599729	2.26E-09
B3	B125	Hcy	Cor	0.12567722	2.77E-07
B3	B199	Hcy	Mg	-0.1253773	2.96E-07
B3	B50	Hcy	eo%	0.12403057	3.96E-07
B30	B31	LH	FSH	0.91366083	0
B30	B32	LH	Testo	-0.3882613	0
B30	B33	LH	Prog	-0.3496809	0
B30	B73	LH	ApoA	0.15979677	5.76E-11
B30	B161	LH	LEU	0.12596742	2.60E-07
B31	B32	FSH	Testo	-0.5250149	0
B31	B33	FSH	Prog	-0.4395265	0
B31	B73	FSH	ApoA	0.19195932	3.11E-15
B31	B194	FSH	Cl	0.161053	4.06E-11
B31	B193	FSH	K	0.13629079	2.45E-08
B31	B198	FSH	P	0.1350396	3.30E-08
B32	B33	Testo	Prog	0.81738285	0
B32	B194	Testo	Cl	-0.1898532	6.22E-15
B32	B198	Testo	P	-0.1700053	3.08E-12
B32	B73	Testo	ApoA	-0.1603355	4.96E-11
B32	B136	Testo	5'NT	0.14600284	2.26E-09
B32	B74	Testo	ApoA1	-0.1383853	1.49E-08
B32	B134	Testo	CHE	0.12860182	1.45E-07
B32	B193	Testo	K	-0.1247751	3.37E-07
B32	B138	Testo	PAB	0.12041603	8.57E-07
B33	B73	Prog	ApoA	-0.1782381	2.53E-13
B33	B194	Prog	Cl	-0.16369	1.93E-11
B33	B198	Prog	P	-0.1431525	4.63E-09
B33	B74	Prog	ApoA1	-0.1371911	1.98E-08
B33	B136	Prog	5'NT	0.12109845	7.42E-07
B34	B145	TK	BGP	0.25358268	0

B34	B144	TK	Total-PINP	0.15182729	5.00E-10
B34	B120	TK	HCV-cAg	-0.1443877	3.40E-09
B34	B87	TK	NSE	0.13813469	1.58E-08
B34	B88	TK	CA 242	0.12077938	7.94E-07
B35	B202	β2-MG	e-times	0.64908514	0
B37	B38	BAND	BAND%	0.86093306	0
B39	B103	Other cells	anti-SSA/Ro	0.2056132	0
B39	B104	Other cells	anti-SSB	0.32782863	0
B39	B40	Other cells	WBC	0.22612436	0
B39	B100	Other cells	anti-Ro52	0.18875952	8.66E-15
B4	B5	WB-Ca	WB-Mg	-0.4113526	0
B4	B7	WB-Ca	WB-Cop	-0.693474	0
B4	B73	WB-Ca	ApoA	0.14510162	2.84E-09
B40	B41	WBC	MON %	-0.2214992	0
B40	B42	WBC	MON	0.59888889	0
B40	B52	WBC	PLT/BPC	0.20458388	0
B40	B53	WBC	GR%	0.20478241	0
B40	B46	WBC	RBC Count	0.1752712	6.31E-13
B40	B48	WBC	LYM%	-0.1666593	8.20E-12
B40	B51	WBC	Hb	0.15871524	7.77E-11
B40	B71	WBC	TG	0.158238	8.87E-11
B40	B206	WBC	Waist	0.13139554	7.69E-08
B40	B130	WBC	GLB	0.13030496	9.86E-08
B40	B207	WBC	WHR	0.12711118	2.02E-07
B40	B49	WBC	baso%	-0.126765	2.18E-07
B40	B72	WBC	HDL-C	-0.1228655	5.10E-07
B40	B222	WBC	NS	0.12114672	7.35E-07
B41	B42	MON %	MON	0.57259544	0
B41	B53	MON %	GR%	-0.2556312	0
B42	B51	MON	Hb	0.22902169	0
B42	B192	MON	UA	0.19246873	2.66E-15
B42	B46	MON	RBC Count	0.19238613	2.66E-15
B42	B222	MON	NS	0.17337443	1.12E-12
B42	B128	MON	ALT	0.16174711	3.34E-11
B42	B206	MON	Waist	0.15158477	5.33E-10
B42	B165	MON	bilirubin	0.14980292	8.50E-10
B42	B131	MON	AST	0.14406329	3.69E-09
B42	B207	MON	WHR	0.14222487	5.83E-09
B42	B127	MON	A-G ratio	-0.1406375	8.61E-09
B42	B48	MON	LYM%	-0.1388265	1.34E-08
B42	B130	MON	GLB	0.13593893	2.67E-08
B42	B209	MON	Smoking	0.12804176	1.64E-07
B42	B71	MON	TG	0.12654257	2.29E-07
B42	B216	MON	BMI	0.12399961	3.99E-07

B42	B52	MON	PLT/BPC	0.1200956	9.17E-07
B43	B44	MCV	MCH	0.93623723	0
B43	B45	MCV	MCHC	0.36960758	0
B43	B46	MCV	RBC Count	-0.4941775	0
B43	B47	MCV	RDW	0.44056572	0
B43	B51	MCV	Hb	0.3384339	0
B43	B52	MCV	PLT/BPC	-0.2855881	0
B43	B189	MCV	eGFB	-0.159232	6.74E-11
B44	B45	MCH	MCHC	0.67023677	0
B44	B46	MCH	RBC Count	-0.3852863	0
B44	B47	MCH	RDW	0.26655108	0
B44	B51	MCH	Hb	0.49013207	0
B44	B52	MCH	PLT/BPC	-0.2509054	0
B44	B189	MCH	eGFB	-0.1587505	7.70E-11
B44	B54	MCH	ESR	-0.12352	4.43E-07
B45	B47	MCHC	RDW	-0.2369142	0
B45	B51	MCHC	Hb	0.59078055	0
B45	B192	MCHC	UA	0.18212777	7.46E-14
B45	B133	MCHC	TBIL	0.15866193	7.89E-11
B45	B129	MCHC	IBIL	0.15654765	1.41E-10
B45	B54	MCHC	ESR	-0.1375583	1.81E-08
B45	B132	MCHC	Dbil	0.12573918	2.73E-07
B45	B58	MCHC	HCT	0.12246231	5.55E-07
B45	B222	MCHC	NS	0.12220407	5.87E-07
B46	B126	RBC Count	Alb	0.21662443	0
B46	B128	RBC Count	ALT	0.21981334	0
B46	B163	RBC Count	SG	0.21549941	0
B46	B192	RBC Count	UA	0.30753199	0
B46	B204	RBC Count	Drinking	0.20483257	0
B46	B216	RBC Count	BMI	0.2405709	0
B46	B218	RBC Count	DBP	0.22324214	0
B46	B221	RBC Count	Hei	0.26869054	0
B46	B222	RBC Count	NS	0.27278347	0
B46	B47	RBC Count	RDW	-0.4225922	0
B46	B51	RBC Count	Hb	0.60307934	0
B46	B72	RBC Count	HDL-C	-0.1994591	2.22E-16
B46	B141	RBC Count	AST/ALT	-0.1907583	4.44E-15
B46	B206	RBC Count	Waist	0.17426625	8.57E-13
B46	B188	RBC Count	Ca	0.16354405	2.01E-11
B46	B209	RBC Count	Smoking	0.15511162	2.08E-10
B46	B207	RBC Count	WHR	0.15509774	2.09E-10
B46	B91	RBC Count	TP	0.15333906	3.35E-10
B46	B165	RBC Count	bilirubin	0.15257404	4.10E-10
B46	B71	RBC Count	TG	0.14454777	3.26E-09

B46	B174	RBC Count	Urea/Cr	-0.1437866	3.95E-09
B46	B52	RBC Count	PLT/BPC	0.12983587	1.10E-07
B46	B140	RBC Count	r-GT	0.12565857	2.78E-07
B46	B166	RBC Count	Pro	0.12250693	5.50E-07
B46	B176	RBC Count	Ucr	0.12220677	5.87E-07
B47	B52	RDW	PLT/BPC	-0.2181827	0
B47	B189	RDW	eGFB	-0.1690943	4.02E-12
B47	B112	RDW	FT4	-0.1679779	5.58E-12
B47	B51	RDW	Hb	-0.1593123	6.59E-11
B47	B72	RDW	HDL-C	0.15837428	8.54E-11
B47	B111	RDW	TSH	0.15436545	2.54E-10
B47	B118	RDW	CEA1	0.14702622	1.74E-09
B47	B126	RDW	Alb	-0.1290886	1.30E-07
B47	B77	RDW	TC	0.12655077	2.28E-07
B48	B53	LYM%	GR%	-0.9507621	0
B49	B50	baso%	eo%	0.28341193	0
B49	B53	baso%	GR%	-0.1802886	1.33E-13
B49	B177	baso%	ACR	0.124816	3.34E-07
B5	B6	WB-Mg	WB-Fe	0.66484304	0
B5	B7	WB-Mg	WB-Cop	-0.31341	0
B5	B8	WB-Mg	WB-Zinc	-0.8114666	0
B5	B58	WB-Mg	HCT	0.19481305	1.11E-15
B5	B73	WB-Mg	ApoA	-0.170532	2.63E-12
B5	B62	WB-Mg	WBHSRVI	0.15725577	1.16E-10
B5	B57	WB-Mg	RBCAI	-0.1488251	1.10E-09
B5	B63	WB-Mg	WBRSR(mPas_200)	0.14249442	5.45E-09
B5	B66	WB-Mg	WBRSR(mPas_100)	0.13047711	9.48E-08
B5	B43	WB-Mg	MCV	0.12412284	3.89E-07
B5	B67	WB-Mg	WBRSR(mPas_50)	0.12228193	5.77E-07
B50	B53	eo%	GR%	-0.2863998	0
B50	B190	eo%	Cre	0.14876266	1.11E-09
B50	B189	eo%	eGFB	-0.1310091	8.40E-08
B50	B156	eo%	IgE	0.12739729	1.89E-07
B51	B128	Hb	ALT	0.22238779	0
B51	B141	Hb	AST/ALT	-0.2438774	0
B51	B163	Hb	SG	0.2223385	0
B51	B192	Hb	UA	0.37124237	0
B51	B204	Hb	Drinking	0.23985776	0
B51	B206	Hb	Waist	0.22830562	0
B51	B207	Hb	WHR	0.22753752	0
B51	B209	Hb	Smoking	0.21747925	0
B51	B216	Hb	BMI	0.27422621	0
B51	B218	Hb	DBP	0.23300799	0
B51	B221	Hb	Hei	0.29564228	0

B51	B222	Hb	NS	0.326488	0
B51	B126	Hb	Alb	0.19138501	3.55E-15
B51	B174	Hb	Urea/Cr	-0.1867712	1.67E-14
B51	B71	Hb	TG	0.18378248	4.40E-14
B51	B190	Hb	Cre	0.1827569	6.08E-14
B51	B165	Hb	bilirubin	0.17767473	3.01E-13
B51	B58	Hb	HCT	0.1735626	1.06E-12
B51	B188	Hb	Ca	0.17130596	2.09E-12
B51	B133	Hb	TBIL	0.15303946	3.62E-10
B51	B129	Hb	IBIL	0.15256254	4.11E-10
B51	B84	Hb	C-PSA	0.15087125	6.43E-10
B51	B72	Hb	HDL-C	-0.1446793	3.16E-09
B51	B189	Hb	eGFB	-0.1377287	1.74E-08
B51	B166	Hb	Pro	0.13339419	4.85E-08
B51	B220	Hb	SBP	0.13154106	7.43E-08
B51	B55	Hb	TK	-0.1255524	2.85E-07
B51	B62	Hb	WBHSRVI	0.11987586	9.60E-07
B51	B210	Hb	Hip	0.11975353	9.85E-07
B52	B189	PLT/BPC	eGFB	0.17042872	2.71E-12
B52	B132	PLT/BPC	Dbil	-0.1667448	8.00E-12
B52	B133	PLT/BPC	TBIL	-0.1295119	1.18E-07
B54	B106	ESR	anti-P	0.30162618	0
B54	B55	ESR	TK	0.28770607	0
B54	B58	ESR	HCT	-0.4381305	0
B54	B59	ESR	WBRV	0.34791283	0
B54	B60	ESR	WBRVI	-0.2507557	0
B54	B62	ESR	WBHSRVI	-0.3395407	0
B54	B69	ESR	PV	0.45805377	0
B54	B61	ESR	WBHSRV	0.16868532	4.54E-12
B54	B195	ESR	Na	-0.1574253	1.11E-10
B54	B157	ESR	IgG	0.13825467	1.54E-08
B54	B179	ESR	α1-MG	-0.1298473	1.09E-07
B54	B101	ESR	anti-Sc70	-0.1295783	1.16E-07
B54	B102	ESR	anti-Sm	-0.1295783	1.16E-07
B54	B105	ESR	anti-U1RNP	-0.1295783	1.16E-07
B54	B107	ESR	AnuA	-0.1295782	1.16E-07
B54	B108	ESR	Anti-Cyclin	-0.1295783	1.16E-07
B54	B97	ESR	dsDNA	-0.1295783	1.16E-07
B54	B98	ESR	anti-Jo-1	-0.1295783	1.16E-07
B54	B99	ESR	anti-PM-Scl	-0.1295783	1.16E-07
B54	B178	ESR	Ig-U	-0.1272658	1.95E-07
B54	B182	ESR	TT	-0.1264486	2.34E-07
B54	B94	ESR	ALDH2-Lys504Lys	-0.1262799	2.43E-07
B54	B200	ESR	OP	-0.1262065	2.46E-07

B54	B180	ESR	Tf	-0.1250915	3.15E-07
B55	B56	TK	IR	0.76740052	0
B55	B58	TK	HCT	-0.601382	0
B55	B59	TK	WBRV	0.57771287	0
B55	B61	TK	WBHSRV	0.6670247	0
B55	B83	TK	AFP	0.32591476	0
B55	B86	TK	TBhCG	0.32517757	0
B55	B69	TK	PV	-0.158778	7.64E-11
B56	B110	IR	AHA	-0.225677	0
B56	B59	IR	WBRV	0.63603961	0
B56	B60	IR	WBRVI	0.55503376	0
B56	B61	IR	WBHSRV	0.91354725	0
B56	B62	IR	WBHSRVI	0.69412865	0
B56	B63	IR	WBRSR(mPas_200)	0.55159827	0
B56	B64	IR	WBRSR(mPas_5)	0.49750478	0
B56	B65	IR	WBRSR(mPas_1)	0.44968532	0
B56	B66	IR	WBRSR(mPas_100)	0.55608854	0
B56	B67	IR	WBRSR(mPas_50)	0.55146005	0
B56	B106	IR	anti-P	-0.1411722	7.55E-09
B56	B86	IR	TBhCG	0.1370312	2.06E-08
B56	B83	IR	AFP	0.13675002	2.20E-08
B56	B69	IR	PV	-0.1363823	2.40E-08
B56	B138	IR	PAB	0.13044292	9.55E-08
B56	B156	IR	IgE	0.12682716	2.15E-07
B56	B125	IR	Cor	0.1204944	8.43E-07
B57	B156	RBCAI	IgE	-0.2574271	0
B57	B59	RBCAI	WBRV	0.59050293	0
B57	B60	RBCAI	WBRVI	0.60112625	0
B57	B64	RBCAI	WBRSR(mPas_5)	0.47379047	0
B57	B65	RBCAI	WBRSR(mPas_1)	0.60800108	0
B57	B66	RBCAI	WBRSR(mPas_100)	0.21228259	0
B57	B67	RBCAI	WBRSR(mPas_50)	0.25977591	0
B57	B94	RBCAI	ALDH2-Lys504Lys	0.18262461	6.35E-14
B57	B58	RBCAI	HCT	0.17995291	1.48E-13
B57	B63	RBCAI	WBRSR(mPas_200)	0.17518772	6.47E-13
B57	B69	RBCAI	PV	0.1664757	8.65E-12
B57	B81	RBCAI	TSGF	-0.15115	5.97E-10
B57	B92	RBCAI	ALDH2-Glu504Glu	-0.1493841	9.48E-10
B57	B83	RBCAI	AFP	-0.143516	4.23E-09
B57	B86	RBCAI	TBhCG	-0.1430331	4.77E-09
B57	B93	RBCAI	ALDH2-Glu504Lys	0.14178767	6.49E-09
B57	B73	RBCAI	ApoA	0.13275584	5.62E-08
B57	B180	RBCAI	Tf	0.12185428	6.32E-07
B58	B60	HCT	WBRVI	0.68272539	0

B58	B62	HCT	WBHSRVI	0.74137645	0
B58	B63	HCT	WBRSR(mPas_200)	0.74075723	0
B58	B64	HCT	WBRSR(mPas_5)	0.70454054	0
B58	B65	HCT	WBRSR(mPas_1)	0.66263434	0
B58	B66	HCT	WBRSR(mPas_100)	0.72854253	0
B58	B67	HCT	WBRSR(mPas_50)	0.72685471	0
B58	B83	HCT	AFP	-0.3044795	0
B58	B86	HCT	TBhCG	-0.30212	0
B58	B110	HCT	AHA	-0.1690959	4.02E-12
B58	B135	HCT	TBA	-0.1525845	4.09E-10
B58	B106	HCT	anti-P	-0.1506943	6.73E-10
B58	B137	HCT	AFU	0.13482864	3.47E-08
B58	B134	HCT	CHE	0.12228113	5.77E-07
B59	B60	WBRV	WBRVI	0.59185858	0
B59	B61	WBRV	WBHSRV	0.78707406	0
B59	B62	WBRV	WBHSRVI	0.33717856	0
B59	B63	WBRV	WBRSR(mPas_200)	0.47442326	0
B59	B64	WBRV	WBRSR(mPas_5)	0.62582685	0
B59	B65	WBRV	WBRSR(mPas_1)	0.67581168	0
B59	B66	WBRV	WBRSR(mPas_100)	0.50582408	0
B59	B67	WBRV	WBRSR(mPas_50)	0.53154798	0
B59	B69	WBRV	PV	0.39518276	0
B59	B81	WBRV	TSGF	-0.1254075	2.94E-07
B6	B7	WB-Fe	WB-Cop	-0.5035603	0
B6	B8	WB-Fe	WB-Zinc	-0.426845	0
B6	B43	WB-Fe	MCV	0.1512009	5.89E-10
B6	B73	WB-Fe	ApoA	-0.1353497	3.07E-08
B6	B44	WB-Fe	MCH	0.13217182	6.43E-08
B60	B110	WBRVI	AHA	-0.2235675	0
B60	B61	WBRVI	WBHSRV	0.58782718	0
B60	B62	WBRVI	WBHSRVI	0.85054902	0
B60	B63	WBRVI	WBRSR(mPas_200)	0.81309301	0
B60	B64	WBRVI	WBRSR(mPas_5)	0.91619329	0
B60	B65	WBRVI	WBRSR(mPas_1)	0.93500705	0
B60	B66	WBRVI	WBRSR(mPas_100)	0.82839077	0
B60	B67	WBRVI	WBRSR(mPas_50)	0.84926039	0
B60	B106	WBRVI	anti-P	-0.1783984	2.41E-13
B60	B83	WBRVI	AFP	-0.1707495	2.47E-12
B60	B86	WBRVI	TBhCG	-0.1690013	4.14E-12
B60	B176	WBRVI	Ucr	0.12913794	1.28E-07
B60	B81	WBRVI	TSGF	-0.1226502	5.34E-07
B60	B182	WBRVI	TT	0.12079139	7.92E-07
B61	B110	WBHSRV	AHA	-0.210857	0
B61	B62	WBHSRV	WBHSRVI	0.67569473	0

B61	B63	WBHSRV	WBRSR(mPas_200)	0.71753223	0
B61	B64	WBHSRV	WBRSR(mPas_5)	0.67612387	0
B61	B65	WBHSRV	WBRSR(mPas_1)	0.62606014	0
B61	B66	WBHSRV	WBRSR(mPas_100)	0.72655937	0
B61	B67	WBHSRV	WBRSR(mPas_50)	0.72369145	0
B61	B69	WBHSRV	PV	0.2683029	0
B61	B124	WBHSRV	ACTH	0.1653959	1.18E-11
B61	B156	WBHSRV	IgE	0.15147843	5.48E-10
B61	B136	WBHSRV	5'NT	0.14400214	3.74E-09
B61	B138	WBHSRV	PAB	0.13516117	3.21E-08
B61	B125	WBHSRV	Cor	0.13060085	9.21E-08
B62	B110	WBHSRVI	AHA	-0.2699924	0
B62	B63	WBHSRVI	WBRSR(mPas_200)	0.89690615	0
B62	B64	WBHSRVI	WBRSR(mPas_5)	0.82822294	0
B62	B65	WBHSRVI	WBRSR(mPas_1)	0.76267785	0
B62	B66	WBHSRVI	WBRSR(mPas_100)	0.89131569	0
B62	B67	WBHSRVI	WBRSR(mPas_50)	0.88595002	0
B62	B106	WBHSRVI	anti-P	-0.1995472	2.22E-16
B62	B124	WBHSRVI	ACTH	0.16135319	3.73E-11
B62	B134	WBHSRVI	CHE	0.15683409	1.30E-10
B62	B138	WBHSRVI	PAB	0.15145405	5.51E-10
B62	B156	WBHSRVI	IgE	0.13959064	1.11E-08
B62	B176	WBHSRVI	Ucr	0.13086889	8.67E-08
B62	B83	WBHSRVI	AFP	-0.1254669	2.90E-07
B62	B86	WBHSRVI	TBhCG	-0.1238598	4.11E-07
B62	B94	WBHSRVI	ALDH2-Lys504Lys	-0.1215686	6.72E-07
B63	B110	WBRSR(mPas_200)	AHA	-0.2287775	0
B63	B64	WBRSR(mPas_200)	WBRSR(mPas_5)	0.9481393	0
B63	B65	WBRSR(mPas_200)	WBRSR(mPas_1)	0.8852284	0
B63	B66	WBRSR(mPas_200)	WBRSR(mPas_100)	0.99750844	0
B63	B67	WBRSR(mPas_200)	WBRSR(mPas_50)	0.99424194	0
B63	B69	WBRSR(mPas_200)	PV	0.40563884	0
B63	B124	WBRSR(mPas_200)	ACTH	0.1953651	8.88E-16
B63	B83	WBRSR(mPas_200)	AFP	-0.1806054	1.21E-13
B63	B86	WBRSR(mPas_200)	TBhCG	-0.1783281	2.46E-13
B63	B134	WBRSR(mPas_200)	CHE	0.15606211	1.61E-10
B63	B156	WBRSR(mPas_200)	IgE	0.15169416	5.18E-10
B63	B138	WBRSR(mPas_200)	PAB	0.14778129	1.43E-09
B63	B136	WBRSR(mPas_200)	5'NT	0.14613328	2.19E-09
B63	B176	WBRSR(mPas_200)	Ucr	0.12562372	2.80E-07
B64	B110	WBRSR(mPas_5)	AHA	-0.2113925	0
B64	B65	WBRSR(mPas_5)	WBRSR(mPas_1)	0.98673087	0
B64	B66	WBRSR(mPas_5)	WBRSR(mPas_100)	0.96050739	0
B64	B67	WBRSR(mPas_5)	WBRSR(mPas_50)	0.97294383	0

B64	B69	WBRSR(mPas_5)	PV	0.42600654	0
B64	B83	WBRSR(mPas_5)	AFP	-0.2004771	0
B64	B86	WBRSR(mPas_5)	TBhCG	-0.1983891	4.44E-16
B64	B124	WBRSR(mPas_5)	ACTH	0.14551352	2.56E-09
B64	B136	WBRSR(mPas_5)	5'NT	0.14095179	7.97E-09
B64	B134	WBRSR(mPas_5)	CHE	0.13456285	3.69E-08
B64	B176	WBRSR(mPas_5)	Ucr	0.12949469	1.18E-07
B64	B81	WBRSR(mPas_5)	TSGF	-0.1233974	4.54E-07
B65	B66	WBRSR(mPas_1)	WBRSR(mPas_100)	0.90354225	0
B65	B67	WBRSR(mPas_1)	WBRSR(mPas_50)	0.92348346	0
B65	B69	WBRSR(mPas_1)	PV	0.41515823	0
B65	B83	WBRSR(mPas_1)	AFP	-0.2031649	0
B65	B86	WBRSR(mPas_1)	TBhCG	-0.2010401	0
B65	B110	WBRSR(mPas_1)	AHA	-0.1913515	3.55E-15
B65	B81	WBRSR(mPas_1)	TSGF	-0.1426656	5.22E-09
B65	B136	WBRSR(mPas_1)	5'NT	0.13042773	9.58E-08
B65	B176	WBRSR(mPas_1)	Ucr	0.12414468	3.87E-07
B66	B110	WBRSR(mPas_100)	AHA	-0.2313186	0
B66	B67	WBRSR(mPas_100)	WBRSR(mPas_50)	0.99876546	0
B66	B69	WBRSR(mPas_100)	PV	0.41801796	0
B66	B124	WBRSR(mPas_100)	ACTH	0.19496486	1.11E-15
B66	B83	WBRSR(mPas_100)	AFP	-0.1820668	7.59E-14
B66	B86	WBRSR(mPas_100)	TBhCG	-0.179821	1.54E-13
B66	B134	WBRSR(mPas_100)	CHE	0.16561322	1.11E-11
B66	B136	WBRSR(mPas_100)	5'NT	0.14838872	1.23E-09
B66	B156	WBRSR(mPas_100)	IgE	0.14227701	5.75E-09
B66	B138	WBRSR(mPas_100)	PAB	0.1392241	1.21E-08
B66	B94	WBRSR(mPas_100)	ALDH2-Lys504Lys	-0.1294645	1.19E-07
B66	B176	WBRSR(mPas_100)	Ucr	0.12913357	1.28E-07
B66	B135	WBRSR(mPas_100)	TBA	-0.1200933	9.18E-07
B67	B110	WBRSR(mPas_50)	AHA	-0.2302073	0
B67	B69	WBRSR(mPas_50)	PV	0.42056381	0
B67	B124	WBRSR(mPas_50)	ACTH	0.1873715	1.38E-14
B67	B83	WBRSR(mPas_50)	AFP	-0.1867696	1.67E-14
B67	B86	WBRSR(mPas_50)	TBhCG	-0.1845326	3.46E-14
B67	B134	WBRSR(mPas_50)	CHE	0.16191715	3.18E-11
B67	B136	WBRSR(mPas_50)	5'NT	0.14803636	1.34E-09
B67	B138	WBRSR(mPas_50)	PAB	0.13265649	5.75E-08
B67	B176	WBRSR(mPas_50)	Ucr	0.13006388	1.04E-07
B67	B156	WBRSR(mPas_50)	IgE	0.12605989	2.55E-07
B67	B135	WBRSR(mPas_50)	TBA	-0.1200514	9.26E-07
B69	B106	PV	anti-P	0.44939311	0
B69	B83	PV	AFP	-0.1726844	1.38E-12
B69	B86	PV	TBhCG	-0.17027	2.84E-12

B69	B91	PV	TP	0.15399597	2.81E-10
B69	B157	PV	IgG	0.14419912	3.56E-09
B69	B158	PV	IgM	0.13009175	1.03E-07
B7	B8	WB-Cop	WB-Zinc	0.59563632	0
B7	B199	WB-Cop	Mg	0.15130497	5.73E-10
B7	B193	WB-Cop	K	0.12835022	1.53E-07
B70	B132	LDL-C	Dbil	-0.2105849	0
B70	B189	LDL-C	eGFB	-0.2012124	0
B70	B75	LDL-C	ApoA1/B	-0.2012752	0
B70	B76	LDL-C	ApoB	0.26972037	0
B70	B77	LDL-C	TC	0.8956847	0
B70	B206	LDL-C	Waist	0.19450766	1.33E-15
B70	B215	LDL-C	BMI	0.17318074	1.19E-12
B70	B207	LDL-C	WHR	0.17292974	1.28E-12
B70	B71	LDL-C	TG	0.1714046	2.03E-12
B70	B218	LDL-C	DBP	0.16494534	1.34E-11
B70	B192	LDL-C	UA	0.16383743	1.85E-11
B70	B220	LDL-C	SBP	0.14773866	1.45E-09
B70	B188	LDL-C	Ca	0.14573799	2.42E-09
B70	B111	LDL-C	TSH	0.14220449	5.86E-09
B70	B210	LDL-C	Hip	0.13932095	1.19E-08
B70	B153	LDL-C	Glu	0.13377521	4.44E-08
B70	B216	LDL-C	BMI	0.12913532	1.28E-07
B70	B222	LDL-C	NS	0.124241	3.79E-07
B71	B128	TG	ALT	0.28167523	0
B71	B131	TG	AST	0.20975923	0
B71	B132	TG	Dbil	-0.2087297	0
B71	B140	TG	r-GT	0.24722868	0
B71	B192	TG	UA	0.33577712	0
B71	B206	TG	Waist	0.32678238	0
B71	B207	TG	WHR	0.31759929	0
B71	B215	TG	BMI	0.32901905	0
B71	B216	TG	BMI	0.28291637	0
B71	B218	TG	DBP	0.21392909	0
B71	B222	TG	NS	0.26516514	0
B71	B72	TG	HDL-C	-0.4327736	0
B71	B77	TG	TC	0.29751064	0
B71	B91	TG	TP	0.21158108	0
B71	B130	TG	GLB	0.19833445	4.44E-16
B71	B210	TG	Hip	0.19710925	4.44E-16
B71	B141	TG	AST/ALT	-0.1849412	3.02E-14
B71	B220	TG	SBP	0.16191552	3.18E-11
B71	B153	TG	Glu	0.15559304	1.83E-10
B71	B134	TG	CHE	0.14327275	4.49E-09

B71	B189	TG	eGFB	-0.1405343	8.83E-09
B71	B127	TG	A-G ratio	-0.1404083	9.11E-09
B71	B150	TG	ALP	0.12355328	4.39E-07
B71	B74	TG	ApoA1	-0.1212573	7.18E-07
B72	B192	HDL-C	UA	-0.3283451	0
B72	B206	HDL-C	Waist	-0.3080484	0
B72	B207	HDL-C	WHR	-0.2657916	0
B72	B210	HDL-C	Hip	-0.2313509	0
B72	B215	HDL-C	BMI	-0.3106156	0
B72	B216	HDL-C	BMI	-0.3559139	0
B72	B222	HDL-C	NS	-0.3279948	0
B72	B74	HDL-C	ApoA1	0.27341745	0
B72	B77	HDL-C	TC	0.25295145	0
B72	B132	HDL-C	Dbil	0.18189763	8.02E-14
B72	B221	HDL-C	Hei	-0.1796147	1.65E-13
B72	B141	HDL-C	AST/ALT	0.16387398	1.83E-11
B72	B75	HDL-C	ApoA1/B	0.16252453	2.68E-11
B72	B128	HDL-C	ALT	-0.1612631	3.83E-11
B72	B133	HDL-C	TBIL	0.13856448	1.42E-08
B72	B140	HDL-C	r-GT	-0.1237507	4.21E-07
B72	B134	HDL-C	CHE	-0.1207147	8.05E-07
B73	B193	ApoA	K	0.14004429	9.95E-09
B73	B90	ApoA	CYFRA 21-1	0.13390042	4.31E-08
B73	B137	ApoA	AFU	0.12650831	2.31E-07
B73	B76	ApoA	ApoB	0.12155575	6.74E-07
B74	B75	ApoA1	ApoA1/B	0.5877379	0
B74	B138	ApoA1	PAB	-0.1527352	3.93E-10
B74	B198	ApoA1	P	0.13739703	1.89E-08
B74	B200	ApoA1	OP	0.12849125	1.48E-07
B75	B134	ApoA1/B	CHE	-0.2295618	0
B75	B76	ApoA1/B	ApoB	-0.7877488	0
B75	B150	ApoA1/B	ALP	-0.1497107	8.71E-10
B75	B136	ApoA1/B	5'NT	-0.147721	1.46E-09
B75	B77	ApoA1/B	TC	-0.1389592	1.30E-08
B76	B134	ApoB	CHE	0.25921783	0
B76	B136	ApoB	5'NT	0.22690049	0
B76	B77	ApoB	TC	0.23831296	0
B76	B197	ApoB	AG	0.18747623	1.33E-14
B76	B198	ApoB	P	0.16337052	2.11E-11
B76	B200	ApoB	OP	0.15583669	1.71E-10
B76	B193	ApoB	K	0.15140962	5.58E-10
B76	B196	ApoB	TCO2	-0.136988	2.08E-08
B76	B155	ApoB	IgA	-0.1311275	8.17E-08
B76	B150	ApoB	ALP	0.1244281	3.64E-07

B76	B211	ApoB	IOP-R	0.12347154	4.47E-07
B76	B156	ApoB	IgE	0.12064584	8.17E-07
B77	B189	TC	eGFB	-0.2661557	0
B77	B132	TC	Dbil	-0.1883932	9.77E-15
B77	B188	TC	Ca	0.17920973	1.87E-13
B77	B111	TC	TSH	0.17458105	7.79E-13
B77	B220	TC	SBP	0.17456856	7.82E-13
B77	B218	TC	DBP	0.16083988	4.31E-11
B77	B207	TC	WHR	0.14853057	1.18E-09
B77	B206	TC	Waist	0.14482368	3.04E-09
B77	B140	TC	r-GT	0.14460224	3.22E-09
B77	B91	TC	TP	0.13515235	3.21E-08
B77	B153	TC	Glu	0.13152716	7.46E-08
B77	B131	TC	AST	0.12958146	1.16E-07
B77	B215	TC	BMI	0.12923958	1.25E-07
B77	B191	TC	Urea	0.12490735	3.28E-07
B78	B154	HBcAb	RF	0.2299056	0
B78	B80	HBcAb	anti-HP IgG	-0.1464926	1.99E-09
B79	B101	hb V -dna	anti-Sc70	0.36623607	0
B79	B102	hb V -dna	anti-Sm	0.36623606	0
B79	B105	hb V -dna	anti-U1RNP	0.36623606	0
B79	B107	hb V -dna	AnuA	0.3662358	0
B79	B108	hb V -dna	Anti-Cyclin	0.36623606	0
B79	B119	hb V -dna	Hcv-rna	0.41630606	0
B79	B121	hb V -dna	C3	-0.2446759	0
B79	B122	hb V -dna	C4	-0.2446765	0
B79	B124	hb V -dna	ACTH	0.25750111	0
B79	B125	hb V -dna	Cor	0.41391598	0
B79	B137	hb V -dna	AFU	0.21246597	0
B79	B159	hb V -dna	β2-MG	-0.2446742	0
B79	B169	hb V -dna	U-CRY	-0.2446778	0
B79	B178	hb V -dna	Ig-U	0.28435161	0
B79	B179	hb V -dna	α1-MG	0.31682626	0
B79	B180	hb V -dna	Tf	0.43471341	0
B79	B81	hb V -dna	TSGF	-0.2984419	0
B79	B94	hb V -dna	ALDH2-Lys504Lys	0.5218244	0
B79	B97	hb V -dna	dsDNA	0.36623606	0
B79	B98	hb V -dna	anti-Jo-1	0.36623607	0
B79	B99	hb V -dna	anti-PM-Scl	0.36623607	0
B79	B197	hb V -dna	AG	-0.1977522	4.44E-16
B79	B120	hb V -dna	HCV-cAg	0.18647142	1.84E-14
B79	B135	hb V -dna	TBA	0.18402383	4.06E-14
B79	B196	hb V -dna	TCO2	0.17510968	6.63E-13
B79	B181	hb V -dna	APTT	-0.1717766	1.81E-12

B79	B184	hb V -dna	INR	0.15953293	6.20E-11
B79	B92	hb V -dna	ALDH2-Glu504Glu	-0.1584854	8.28E-11
B79	B176	hb V -dna	Ucr	-0.1406633	8.56E-09
B79	B93	hb V -dna	ALDH2-Glu504Lys	0.12949829	1.18E-07
B79	B183	hb V -dna	PTA	0.12645038	2.34E-07
B8	B58	WB-Zinc	HCT	-0.1854144	2.60E-14
B8	B62	WB-Zinc	WBHSRVI	-0.1331586	5.12E-08
B81	B125	TSGF	Cor	-0.3300932	0
B81	B179	TSGF	α1-MG	-0.228452	0
B81	B180	TSGF	Tf	-0.2456794	0
B81	B92	TSGF	ALDH2-Glu504Glu	0.20304701	0
B81	B94	TSGF	ALDH2-Lys504Lys	-0.2487227	0
B81	B120	TSGF	HCV-cAg	0.19769959	4.44E-16
B81	B181	TSGF	APTT	0.1972075	4.44E-16
B81	B93	TSGF	ALDH2-Glu504Lys	-0.1926902	2.22E-15
B81	B156	TSGF	IgE	0.18574095	2.33E-14
B81	B199	TSGF	Mg	0.15309279	3.57E-10
B81	B135	TSGF	TBA	-0.1529992	3.66E-10
B81	B184	TSGF	INR	-0.1417861	6.49E-09
B81	B101	TSGF	anti-Sc70	-0.1382216	1.55E-08
B81	B102	TSGF	anti-Sm	-0.1382216	1.55E-08
B81	B105	TSGF	anti-U1RNP	-0.1382216	1.55E-08
B81	B107	TSGF	AnuA	-0.1382215	1.55E-08
B81	B108	TSGF	Anti-Cyclin	-0.1382216	1.55E-08
B81	B97	TSGF	dsDNA	-0.1382216	1.55E-08
B81	B98	TSGF	anti-Jo-1	-0.1382216	1.55E-08
B81	B99	TSGF	anti-PM-Scl	-0.1382216	1.55E-08
B81	B178	TSGF	Ig-U	-0.13477	3.51E-08
B81	B124	TSGF	ACTH	-0.1279605	1.67E-07
B82	B83	CEA	AFP	0.24636568	0
B82	B84	CEA	C-PSA	0.53629725	0
B82	B85	CEA	SCC	0.35187384	0
B82	B86	CEA	TBhCG	0.22014833	0
B82	B89	CEA	CA 72-4	0.2157008	0
B82	B90	CEA	CYFRA 21-1	0.23288259	0
B82	B114	CEA	TSH TT3 TT4(TT4)	-0.1236645	4.29E-07
B83	B86	AFP	TBhCG	0.99709962	0
B84	B85	C-PSA	SCC	0.28709107	0
B84	B90	C-PSA	CYFRA 21-1	0.25868345	0
B84	B114	C-PSA	TSH TT3 TT4(TT4)	-0.1384693	1.46E-08
B84	B192	C-PSA	UA	0.12574519	2.73E-07
B84	B209	C-PSA	Smoking	0.12508138	3.15E-07
B85	B87	SCC	NSE	0.24316567	0
B85	B90	SCC	CYFRA 21-1	0.13721847	1.97E-08

B9	B137	RH	AFU	-0.193383	1.78E-15
B9	B150	RH	ALP	0.16205818	3.06E-11
B9	B138	RH	PAB	0.15292227	3.74E-10
B9	B134	RH	CHE	0.13876508	1.36E-08
B90	B109	CYFRA 21-1	AMA-M2	0.15610618	1.59E-10
B90	B137	CYFRA 21-1	AFU	0.12300729	4.94E-07
B91	B126	TP	Alb	0.54395769	0
B91	B127	TP	A-G ratio	-0.4913383	0
B91	B130	TP	GLB	0.80438174	0
B91	B188	TP	Ca	0.34357935	0
B91	B192	TP	UA	0.1231953	4.75E-07
B92	B125	ALDH2-Glu504Glu	Cor	-0.2093103	0
B92	B184	ALDH2-Glu504Glu	INR	-0.2139305	0
B92	B225	ALDH2-Glu504Glu	hPBG	-0.216276	0
B92	B93	ALDH2-Glu504Glu	ALDH2-Glu504Lys	-0.9983775	0
B92	B94	ALDH2-Glu504Glu	ALDH2-Lys504Lys	-0.4507836	0
B92	B180	ALDH2-Glu504Glu	Tf	-0.1656057	1.11E-11
B92	B176	ALDH2-Glu504Glu	Ucr	0.16159977	3.48E-11
B92	B135	ALDH2-Glu504Glu	TBA	-0.1554307	1.91E-10
B92	B196	ALDH2-Glu504Glu	TCO2	-0.1489041	1.07E-09
B92	B179	ALDH2-Glu504Glu	α1-MG	-0.1473162	1.62E-09
B92	B186	ALDH2-Glu504Glu	PFC	0.1430673	4.73E-09
B92	B183	ALDH2-Glu504Glu	PTA	-0.1382339	1.54E-08
B92	B197	ALDH2-Glu504Glu	AG	0.13043988	9.56E-08
B92	B101	ALDH2-Glu504Glu	anti-Sc70	-0.126844	2.14E-07
B92	B102	ALDH2-Glu504Glu	anti-Sm	-0.126844	2.14E-07
B92	B105	ALDH2-Glu504Glu	anti-U1RNP	-0.126844	2.14E-07
B92	B107	ALDH2-Glu504Glu	AnuA	-0.1268439	2.14E-07
B92	B108	ALDH2-Glu504Glu	Anti-Cyclin	-0.126844	2.14E-07
B92	B97	ALDH2-Glu504Glu	dsDNA	-0.126844	2.14E-07
B92	B98	ALDH2-Glu504Glu	anti-Jo-1	-0.126844	2.14E-07
B92	B99	ALDH2-Glu504Glu	anti-PM-Scl	-0.126844	2.14E-07
B93	B184	ALDH2-Glu504Lys	INR	0.20301985	0
B93	B225	ALDH2-Glu504Lys	hPBG	0.20530349	0
B93	B94	ALDH2-Glu504Lys	ALDH2-Lys504Lys	0.39922455	0
B93	B125	ALDH2-Glu504Lys	Cor	0.19870874	4.44E-16
B93	B176	ALDH2-Glu504Lys	Ucr	-0.1565437	1.41E-10
B93	B135	ALDH2-Glu504Lys	TBA	0.14768882	1.47E-09
B93	B180	ALDH2-Glu504Lys	Tf	0.14559318	2.51E-09
B93	B196	ALDH2-Glu504Lys	TCO2	0.14120421	7.49E-09
B93	B186	ALDH2-Glu504Lys	PFC	-0.1357127	2.82E-08
B93	B179	ALDH2-Glu504Lys	α1-MG	0.13423319	3.99E-08
B93	B183	ALDH2-Glu504Lys	PTA	0.13110273	8.22E-08
B93	B197	ALDH2-Glu504Lys	AG	-0.1236942	4.26E-07

B94	B101	ALDH2-Lys504Lys	anti-Sc70	0.41886119	0
B94	B102	ALDH2-Lys504Lys	anti-Sm	0.4188612	0
B94	B105	ALDH2-Lys504Lys	anti-U1RNP	0.41886119	0
B94	B107	ALDH2-Lys504Lys	AnuA	0.41886096	0
B94	B108	ALDH2-Lys504Lys	Anti-Cyclin	0.41886118	0
B94	B125	ALDH2-Lys504Lys	Cor	0.25522481	0
B94	B178	ALDH2-Lys504Lys	Ig-U	0.22866047	0
B94	B179	ALDH2-Lys504Lys	α1-MG	0.26775524	0
B94	B180	ALDH2-Lys504Lys	Tf	0.38416472	0
B94	B184	ALDH2-Lys504Lys	INR	0.26203444	0
B94	B225	ALDH2-Lys504Lys	hPBG	0.26400212	0
B94	B97	ALDH2-Lys504Lys	dsDNA	0.4188612	0
B94	B98	ALDH2-Lys504Lys	anti-Jo-1	0.4188612	0
B94	B99	ALDH2-Lys504Lys	anti-PM-Scl	0.4188612	0
B94	B135	ALDH2-Lys504Lys	TBA	0.18747681	1.33E-14
B94	B196	ALDH2-Lys504Lys	TCO2	0.18404199	4.04E-14
B94	B156	ALDH2-Lys504Lys	IgE	-0.1822061	7.26E-14
B94	B186	ALDH2-Lys504Lys	PFC	-0.1761472	4.83E-13
B94	B183	ALDH2-Lys504Lys	PTA	0.1705894	2.59E-12
B94	B197	ALDH2-Lys504Lys	AG	-0.1612302	3.86E-11
B94	B158	ALDH2-Lys504Lys	IgM	0.15737891	1.12E-10
B94	B222	ALDH2-Lys504Lys	NS	-0.1511486	5.98E-10
B94	B176	ALDH2-Lys504Lys	Ucr	-0.1479972	1.36E-09
B94	B134	ALDH2-Lys504Lys	CHE	-0.1296921	1.13E-07
B94	B199	ALDH2-Lys504Lys	Mg	-0.1290765	1.30E-07
B94	B193	ALDH2-Lys504Lys	K	0.12704368	2.05E-07
B94	B209	ALDH2-Lys504Lys	Smoking	-0.1228171	5.15E-07
B94	B216	ALDH2-Lys504Lys	BMI	-0.1205447	8.34E-07
B95	B100	anti-cenpb	anti-Ro52	0.56791038	0
B95	B178	anti-cenpb	Ig-U	-0.2626861	0
B95	B179	anti-cenpb	α1-MG	-0.2551582	0
B95	B180	anti-cenpb	Tf	-0.2197689	0
B95	B196	anti-cenpb	TCO2	0.31117951	0
B95	B197	anti-cenpb	AG	-0.2057588	0
B95	B101	anti-cenpb	anti-Sc70	-0.1861109	2.07E-14
B95	B102	anti-cenpb	anti-Sm	-0.1861109	2.07E-14
B95	B105	anti-cenpb	anti-U1RNP	-0.1861109	2.07E-14
B95	B107	anti-cenpb	AnuA	-0.1861107	2.07E-14
B95	B108	anti-cenpb	Anti-Cyclin	-0.1861109	2.07E-14
B95	B97	anti-cenpb	dsDNA	-0.1861109	2.07E-14
B95	B98	anti-cenpb	anti-Jo-1	-0.1861109	2.07E-14
B95	B99	anti-cenpb	anti-PM-Scl	-0.1861109	2.07E-14
B95	B224	anti-cenpb	pmh	0.15893548	7.32E-11
B95	B137	anti-cenpb	AFU	-0.1250606	3.17E-07

B97	B100	dsDNA	anti-Ro52	-0.3185303	0
B97	B101	dsDNA	anti-Sc70	1	0
B97	B102	dsDNA	anti-Sm	1	0
B97	B103	dsDNA	anti-SSA/Ro	-0.3344827	0
B97	B107	dsDNA	AnuA	0.99999919	0
B97	B125	dsDNA	Cor	0.26774053	0
B97	B135	dsDNA	TBA	0.24449504	0
B97	B156	dsDNA	IgE	0.29730773	0
B97	B178	dsDNA	Ig-U	0.34819819	0
B97	B179	dsDNA	α1-MG	0.34866524	0
B97	B180	dsDNA	Tf	0.42439565	0
B97	B99	dsDNA	anti-PM-Scl	0.99999999	0
B97	B121	dsDNA	C3	-0.1954527	8.88E-16
B97	B122	dsDNA	C4	-0.1954533	8.88E-16
B97	B159	dsDNA	β2-MG	-0.1954515	8.88E-16
B97	B169	dsDNA	U-CRY	-0.1954547	8.88E-16
B97	B184	dsDNA	INR	0.1931741	2.00E-15
B97	B106	dsDNA	anti-P	-0.19276	2.22E-15
B97	B154	dsDNA	RF	-0.1785002	2.33E-13
B97	B139	dsDNA	ADA	0.16693577	7.57E-12
B97	B119	dsDNA	Hcv-rna	0.16323416	2.19E-11
B97	B151	dsDNA	Anti-CCP	0.15767896	1.03E-10
B97	B104	dsDNA	anti-SSB	-0.1552047	2.03E-10
B97	B158	dsDNA	IgM	0.14873462	1.12E-09
B97	B181	dsDNA	APTT	-0.1409302	8.01E-09
B97	B137	dsDNA	AFU	0.1375318	1.83E-08
B97	B138	dsDNA	PAB	0.12878441	1.39E-07
B98	B100	anti-Jo-1	anti-Ro52	-0.3185303	0
B98	B103	anti-Jo-1	anti-SSA/Ro	-0.3344827	0
B98	B107	anti-Jo-1	AnuA	0.99999916	0
B98	B125	anti-Jo-1	Cor	0.26774054	0
B98	B135	anti-Jo-1	TBA	0.24449505	0
B98	B156	anti-Jo-1	IgE	0.29730774	0
B98	B178	anti-Jo-1	Ig-U	0.3481982	0
B98	B179	anti-Jo-1	α1-MG	0.34866525	0
B98	B180	anti-Jo-1	Tf	0.42439566	0
B98	B121	anti-Jo-1	C3	-0.1954526	8.88E-16
B98	B122	anti-Jo-1	C4	-0.1954531	8.88E-16
B98	B159	anti-Jo-1	β2-MG	-0.1954518	8.88E-16
B98	B169	anti-Jo-1	U-CRY	-0.1954546	8.88E-16
B98	B184	anti-Jo-1	INR	0.1931741	2.00E-15
B98	B106	anti-Jo-1	anti-P	-0.19276	2.22E-15
B98	B154	anti-Jo-1	RF	-0.1785002	2.33E-13
B98	B139	anti-Jo-1	ADA	0.16693577	7.57E-12

B98	B119	anti-Jo-1	Hcv-rna	0.16323416	2.19E-11
B98	B151	anti-Jo-1	Anti-CCP	0.15767897	1.03E-10
B98	B104	anti-Jo-1	anti-SSB	-0.1552047	2.03E-10
B98	B158	anti-Jo-1	IgM	0.14873463	1.12E-09
B98	B181	anti-Jo-1	APTT	-0.1409302	8.01E-09
B98	B137	anti-Jo-1	AFU	0.13753181	1.83E-08
B98	B138	anti-Jo-1	PAB	0.12878442	1.39E-07
B99	B100	anti-PM-Scl	anti-Ro52	-0.3185303	0
B99	B101	anti-PM-Scl	anti-Sc70	1	0
B99	B103	anti-PM-Scl	anti-SSA/Ro	-0.3344827	0
B99	B107	anti-PM-Scl	AnuA	0.99999928	0
B99	B108	anti-PM-Scl	Anti-Cyclin	0.99999999	0
B99	B125	anti-PM-Scl	Cor	0.26774054	0
B99	B135	anti-PM-Scl	TBA	0.24449504	0
B99	B156	anti-PM-Scl	IgE	0.29730773	0
B99	B178	anti-PM-Scl	Ig-U	0.34819819	0
B99	B179	anti-PM-Scl	α1-MG	0.34866524	0
B99	B180	anti-PM-Scl	Tf	0.42439565	0
B99	B121	anti-PM-Scl	C3	-0.1954526	8.88E-16
B99	B122	anti-PM-Scl	C4	-0.1954533	8.88E-16
B99	B159	anti-PM-Scl	β2-MG	-0.1954515	8.88E-16
B99	B169	anti-PM-Scl	U-CRY	-0.1954547	8.88E-16
B99	B184	anti-PM-Scl	INR	0.1931741	2.00E-15
B99	B106	anti-PM-Scl	anti-P	-0.19276	2.22E-15
B99	B154	anti-PM-Scl	RF	-0.1785002	2.33E-13
B99	B139	anti-PM-Scl	ADA	0.16693577	7.57E-12
B99	B119	anti-PM-Scl	Hcv-rna	0.16323416	2.19E-11
B99	B151	anti-PM-Scl	Anti-CCP	0.15767896	1.03E-10
B99	B104	anti-PM-Scl	anti-SSB	-0.1552047	2.03E-10
B99	B158	anti-PM-Scl	IgM	0.14873463	1.12E-09
B99	B181	anti-PM-Scl	APTT	-0.1409302	8.01E-09
B99	B137	anti-PM-Scl	AFU	0.1375318	1.83E-08
B99	B138	anti-PM-Scl	PAB	0.12878441	1.39E-07

Supplementary Table 12. PEI Correlations in Hyperthyroidism

Row	Column	Row	Column	Cor	*P*
B1	B141	Gender	AST/ALT	0.325837418	0
B1	B163	Gender	SG	-0.47370468	0
B1	B174	Gender	Urea/Cr	0.321845394	0
B1	B190	Gender	Cre	-0.66322191	0
B1	B192	Gender	UA	-0.53069188	0
B1	B204	Gender	Drinking	-0.39666751	0
B1	B206	Gender	Waist	-0.52645017	0
B1	B209	Gender	Smoking	-0.49301441	0
B1	B210	Gender	Hip	-0.44400475	0
B1	B215	Gender	BMI	-0.32879682	0
B1	B216	Gender	BMI	-0.62804215	0
B1	B221	Gender	Hei	-0.67020916	0
B1	B222	Gender	NS	-0.74772688	0
B1	B42	Gender	MON	-0.2940062	0
B1	B46	Gender	RBC Count	-0.57046018	0
B1	B51	Gender	Hb	-0.67753058	0
B1	B45	Gender	MCHC	-0.29144508	2.22E-16
B1	B72	Gender	HDL-C	0.285079822	8.88E-16
B1	B128	Gender	ALT	-0.27368421	1.20E-14
B1	B127	Gender	A-G ratio	-0.24488045	6.16E-12
B1	B218	Gender	DBP	-0.24054071	1.48E-11
B1	B166	Gender	Pro	-0.22575594	2.55E-10
B1	B71	Gender	TG	-0.20873412	5.35E-09
B1	B130	Gender	GLB	0.207500561	6.61E-09
B1	B41	Gender	MON%	-0.20374665	1.25E-08
B1	B191	Gender	Urea	-0.19603457	4.41E-08
B10	B12	PG I	PG II	0.614289527	0
B100	B103	anti-Ro52	anti-SSA/Ro	-0.66109405	0
B100	B104	anti-Ro52	anti-SSB	0.999999994	0
B100	B106	anti-Ro52	anti-P	0.999999994	0
B100	B107	anti-Ro52	AnuA	0.99999973	0
B100	B109	anti-Ro52	AMA-M2	1	0
B100	B171	anti-Ro52	EC	-0.30105168	0
B100	B173	anti-Ro52	NTT	-0.29660015	0
B100	B179	anti-Ro52	α1-MG	0.285253143	8.88E-16
B100	B158	anti-Ro52	IgM	0.281658571	1.78E-15
B100	B139	anti-Ro52	ADA	-0.22290662	4.32E-10
B100	B199	anti-Ro52	Mg	0.207016524	7.18E-09
B100	B135	anti-Ro52	TBA	0.178387519	6.62E-07
B100	B193	anti-Ro52	K	-0.17780783	7.20E-07
B100	B157	anti-Ro52	IgG	-0.17568129	9.80E-07
B101	B102	anti-Sc70	anti-Sm	0.999999988	0

B101	B103	anti-Sc70	anti-SSA/Ro	-0.66109406	0
B101	B105	anti-Sc70	anti-U1RNP	0.999999999	0
B101	B107	anti-Sc70	AnuA	0.999999522	0
B101	B108	anti-Sc70	Anti-Cyclin	0.999999989	0
B101	B171	anti-Sc70	EC	-0.30105168	0
B101	B173	anti-Sc70	NTT	-0.29660015	0
B101	B179	anti-Sc70	α1-MG	0.285253148	8.88E-16
B101	B158	anti-Sc70	IgM	0.281658576	1.78E-15
B101	B139	anti-Sc70	ADA	-0.22290662	4.32E-10
B101	B199	anti-Sc70	Mg	0.207016527	7.18E-09
B101	B135	anti-Sc70	TBA	0.178387522	6.62E-07
B101	B193	anti-Sc70	K	-0.17780784	7.20E-07
B101	B157	anti-Sc70	IgG	-0.17568129	9.80E-07
B102	B103	anti-Sm	anti-SSA/Ro	-0.66109406	0
B102	B107	anti-Sm	AnuA	0.999999833	0
B102	B171	anti-Sm	EC	-0.30105168	0
B102	B173	anti-Sm	NTT	-0.29660015	0
B102	B179	anti-Sm	α1-MG	0.285253146	8.88E-16
B102	B158	anti-Sm	IgM	0.281658574	1.78E-15
B102	B139	anti-Sm	ADA	-0.22290662	4.32E-10
B102	B199	anti-Sm	Mg	0.207016527	7.18E-09
B102	B135	anti-Sm	TBA	0.178387521	6.62E-07
B102	B193	anti-Sm	K	-0.17780784	7.20E-07
B102	B157	anti-Sm	IgG	-0.17568129	9.80E-07
B103	B104	anti-SSA/Ro	anti-SSB	-0.66109405	0
B103	B105	anti-SSA/Ro	anti-U1RNP	-0.66109405	0
B103	B106	anti-SSA/Ro	anti-P	-0.66109404	0
B103	B107	anti-SSA/Ro	AnuA	-0.66109387	0
B103	B108	anti-SSA/Ro	Anti-Cyclin	-0.66109405	0
B103	B109	anti-SSA/Ro	AMA-M2	-0.66109405	0
B103	B110	anti-SSA/Ro	AHA	-0.66109405	0
B103	B139	anti-SSA/Ro	ADA	0.414422377	0
B103	B173	anti-SSA/Ro	NTT	0.327126499	0
B103	B179	anti-SSA/Ro	α1-MG	-0.39454072	0
B103	B199	anti-SSA/Ro	Mg	-0.27510782	8.66E-15
B103	B134	anti-SSA/Ro	CHE	-0.25678747	5.14E-13
B103	B149	anti-SSA/Ro	TGAb	0.247924341	3.31E-12
B103	B150	anti-SSA/Ro	ALP	0.233329181	6.07E-11
B103	B198	anti-SSA/Ro	P	0.221774954	5.32E-10
B103	B197	anti-SSA/Ro	AG	0.212922277	2.59E-09
B103	B196	anti-SSA/Ro	TCO2	-0.20859179	5.49E-09
B103	B157	anti-SSA/Ro	IgG	0.204832735	1.04E-08
B103	B193	anti-SSA/Ro	K	0.199046014	2.71E-08
B104	B105	anti-SSB	anti-U1RNP	0.999999992	0

B104	B106	anti-SSB	anti-P	0.999999998	0
B104	B107	anti-SSB	AnuA	0.999999775	0
B104	B108	anti-SSB	Anti-Cyclin	0.99999999	0
B104	B109	anti-SSB	AMA-M2	0.999999996	0
B104	B110	anti-SSB	AHA	0.999999993	0
B104	B171	anti-SSB	EC	-0.30105168	0
B104	B173	anti-SSB	NTT	-0.29660015	0
B104	B179	anti-SSB	α1-MG	0.285253141	8.88E-16
B104	B158	anti-SSB	IgM	0.281658569	1.78E-15
B104	B139	anti-SSB	ADA	-0.22290661	4.32E-10
B104	B199	anti-SSB	Mg	0.207016523	7.18E-09
B104	B135	anti-SSB	TBA	0.178387518	6.62E-07
B104	B193	anti-SSB	K	-0.17780783	7.20E-07
B104	B157	anti-SSB	IgG	-0.17568129	9.80E-07
B105	B106	anti-U1RNP	anti-P	0.999999987	0
B105	B107	anti-U1RNP	AnuA	0.999999711	0
B105	B109	anti-U1RNP	AMA-M2	0.999999996	0
B105	B171	anti-U1RNP	EC	-0.30105168	0
B105	B173	anti-U1RNP	NTT	-0.29660015	0
B105	B179	anti-U1RNP	α1-MG	0.285253143	8.88E-16
B105	B158	anti-U1RNP	IgM	0.281658571	1.78E-15
B105	B139	anti-U1RNP	ADA	-0.22290662	4.32E-10
B105	B199	anti-U1RNP	Mg	0.207016524	7.18E-09
B105	B135	anti-U1RNP	TBA	0.178387519	6.62E-07
B105	B193	anti-U1RNP	K	-0.17780783	7.20E-07
B105	B157	anti-U1RNP	IgG	-0.17568129	9.80E-07
B106	B108	anti-P	Anti-Cyclin	0.999999993	0
B106	B109	anti-P	AMA-M2	0.999999993	0
B106	B110	anti-P	AHA	0.999999992	0
B106	B171	anti-P	EC	-0.30105167	0
B106	B173	anti-P	NTT	-0.29660014	0
B106	B179	anti-P	α1-MG	0.285253141	8.88E-16
B106	B158	anti-P	IgM	0.281658568	1.78E-15
B106	B139	anti-P	ADA	-0.22290661	4.32E-10
B106	B199	anti-P	Mg	0.207016524	7.18E-09
B106	B135	anti-P	TBA	0.178387517	6.62E-07
B106	B193	anti-P	K	-0.17780783	7.20E-07
B106	B157	anti-P	IgG	-0.17568129	9.80E-07
B107	B108	AnuA	Anti-Cyclin	0.999999687	0
B107	B109	AnuA	AMA-M2	0.999999735	0
B107	B110	AnuA	AHA	0.99999977	0
B107	B171	AnuA	EC	-0.3010516	0
B107	B173	AnuA	NTT	-0.29660007	0
B107	B179	AnuA	α1-MG	0.285253067	8.88E-16

B107	B158	AnuA	IgM	0.281658499	1.78E-15
B107	B139	AnuA	ADA	-0.22290656	4.32E-10
B107	B199	AnuA	Mg	0.207016466	7.18E-09
B107	B135	AnuA	TBA	0.178387472	6.62E-07
B107	B193	AnuA	K	-0.17780779	7.20E-07
B107	B157	AnuA	IgG	-0.17568124	9.80E-07
B108	B110	Anti-Cyclin	AHA	0.999999997	0
B108	B171	Anti-Cyclin	EC	-0.30105168	0
B108	B173	Anti-Cyclin	NTT	-0.29660015	0
B108	B179	Anti-Cyclin	α1-MG	0.285253144	8.88E-16
B108	B158	Anti-Cyclin	IgM	0.281658572	1.78E-15
B108	B139	Anti-Cyclin	ADA	-0.22290662	4.32E-10
B108	B199	Anti-Cyclin	Mg	0.207016524	7.18E-09
B108	B135	Anti-Cyclin	TBA	0.178387519	6.62E-07
B108	B193	Anti-Cyclin	K	-0.17780784	7.20E-07
B108	B157	Anti-Cyclin	IgG	-0.17568129	9.80E-07
B109	B171	AMA-M2	EC	-0.30105168	0
B109	B173	AMA-M2	NTT	-0.29660015	0
B109	B179	AMA-M2	α1-MG	0.285253143	8.88E-16
B109	B158	AMA-M2	IgM	0.281658571	1.78E-15
B109	B139	AMA-M2	ADA	-0.22290662	4.32E-10
B109	B199	AMA-M2	Mg	0.207016524	7.18E-09
B109	B135	AMA-M2	TBA	0.178387519	6.62E-07
B109	B193	AMA-M2	K	-0.17780783	7.20E-07
B109	B157	AMA-M2	IgG	-0.17568129	9.80E-07
B11	B12	PGI/PGII	PG II	-0.4519117	0
B11	B14	PGI/PGII	G-17	-0.26219205	1.59E-13
B11	B196	PGI/PGII	TCO2	0.254617295	8.16E-13
B11	B197	PGI/PGII	AG	-0.19682067	3.89E-08
B110	B171	AHA	EC	-0.30105168	0
B110	B173	AHA	NTT	-0.29660015	0
B110	B179	AHA	α1-MG	0.285253144	8.88E-16
B110	B158	AHA	IgM	0.281658572	1.78E-15
B110	B139	AHA	ADA	-0.22290662	4.32E-10
B110	B199	AHA	Mg	0.207016524	7.18E-09
B110	B135	AHA	TBA	0.178387519	6.62E-07
B110	B193	AHA	K	-0.17780783	7.20E-07
B110	B157	AHA	IgG	-0.17568129	9.80E-07
B111	B123	TSH	hs-CRP1	0.355789066	0
B111	B132	TSH	Dbil	0.345918066	0
B111	B165	TSH	bilirubin	0.297198777	0
B111	B112	TSH	FT4	-0.27704928	5.55E-15
B111	B133	TSH	TBIL	0.194883831	5.31E-08
B111	B131	TSH	AST	0.179420476	5.69E-07

B111	B130	TSH	GLB	0.178108961	6.89E-07
B112	B113	FT4	FT3	0.855980391	0
B112	B114	FT4	TSH TT3 TT4(TT4)	0.591225565	0
B112	B115	FT4	TSH TT3 TT4(TT3)	0.543938823	0
B112	B174	FT4	Urea/Cr	0.293490531	0
B112	B145	FT4	BGP	0.280214981	2.66E-15
B112	B143	FT4	β-CTx	0.253618464	1.01E-12
B112	B144	FT4	Total-PINP	0.252549567	1.26E-12
B112	B149	FT4	TGAb	0.232679893	6.88E-11
B112	B148	FT4	Anti_TPO	0.196468051	4.12E-08
B112	B190	FT4	Cre	-0.17598587	9.38E-07
B113	B114	FT3	TSH TT3 TT4(TT4)	0.469429751	0
B113	B115	FT3	TSH TT3 TT4(TT3)	0.571696482	0
B113	B174	FT3	Urea/Cr	0.300519373	0
B113	B145	FT3	BGP	0.274975697	8.88E-15
B113	B144	FT3	Total-PINP	0.251369205	1.62E-12
B113	B143	FT3	β-CTx	0.246912795	4.07E-12
B113	B149	FT3	TGAb	0.225933301	2.47E-10
B113	B137	FT3	AFU	-0.20818622	5.88E-09
B113	B199	FT3	Mg	-0.17567927	9.80E-07
B114	B115	TSH TT3 TT4(TT4)	TSH TT3 TT4(TT3)	0.798713449	0
B114	B137	TSH TT3 TT4(TT4)	AFU	-0.26521343	8.19E-14
B114	B199	TSH TT3 TT4(TT4)	Mg	-0.23710561	2.91E-11
B114	B138	TSH TT3 TT4(TT4)	PAB	-0.23682215	3.08E-11
B114	B149	TSH TT3 TT4(TT4)	TGAb	0.210291181	4.09E-09
B114	B148	TSH TT3 TT4(TT4)	Anti_TPO	0.193822857	6.28E-08
B114	B174	TSH TT3 TT4(TT4)	Urea/Cr	0.190761985	1.02E-07
B115	B137	TSH TT3 TT4(TT3)	AFU	-0.29855747	0
B115	B199	TSH TT3 TT4(TT3)	Mg	-0.27913543	3.55E-15
B115	B138	TSH TT3 TT4(TT3)	PAB	-0.26681252	5.73E-14
B115	B149	TSH TT3 TT4(TT3)	TGAb	0.202115724	1.63E-08
B115	B174	TSH TT3 TT4(TT3)	Urea/Cr	0.193125853	7.02E-08
B117	B167	TT4	PC	0.295954092	0
B117	B223	TT4	FH	0.848365617	0
B118	B123	CEA1	hs-CRP1	0.199335416	2.58E-08
B118	B132	CEA1	Dbil	0.182257836	3.74E-07
B119	B120	Hcv-rna	HCV-cAg	0.374988321	0
B119	B122	Hcv-rna	C4	-0.99999947	0
B119	B152	Hcv-rna	ACA-Ig(G-A-M)	-0.99996911	0
B119	B169	Hcv-rna	U-CRY	-0.99999274	0
B12	B80	PG II	anti-HP IgG	0.289830134	2.22E-16
B12	B14	PG II	G-17	0.288217638	4.44E-16
B120	B121	HCV-cAg	C3	-0.37498915	0
B120	B122	HCV-cAg	C4	-0.37498851	0

B120	B152	HCV-cAg	ACA-Ig(G-A-M)	-0.37497813	0
B120	B159	HCV-cAg	β2-MG	-0.37499177	0
B120	B169	HCV-cAg	U-CRY	-0.37498701	0
B121	B122	C3	C4	0.999999539	0
B121	B152	C3	ACA-Ig(G-A-M)	0.999974627	0
B121	B169	C3	U-CRY	0.999995258	0
B122	B152	C4	ACA-Ig(G-A-M)	0.999973235	0
B122	B159	C4	β2-MG	0.999998584	0
B123	B131	hs-CRP1	AST	0.458078796	0
B123	B132	hs-CRP1	Dbil	0.730082968	0
B123	B133	hs-CRP1	TBIL	0.358254947	0
B123	B140	hs-CRP1	r-GT	0.47808406	0
B123	B165	hs-CRP1	bilirubin	0.630486503	0
B123	B126	hs-CRP1	Alb	-0.2865132	4.44E-16
B123	B188	hs-CRP1	Ca	-0.20872702	5.36E-09
B123	B130	hs-CRP1	GLB	0.190756397	1.02E-07
B124	B125	ACTH	Cor	-0.65419532	0
B125	B156	Cor	IgE	0.192336905	7.95E-08
B126	B127	Alb	A-G ratio	0.455174888	0
B126	B188	Alb	Ca	0.316346988	0
B126	B132	Alb	Dbil	-0.22890682	1.41E-10
B126	B165	Alb	bilirubin	-0.22361441	3.79E-10
B126	B131	Alb	AST	-0.18627118	2.04E-07
B127	B130	A-G ratio	GLB	-0.90397717	0
B127	B221	A-G ratio	Hei	0.235292855	4.15E-11
B127	B222	A-G ratio	NS	0.200839332	2.02E-08
B127	B131	A-G ratio	AST	-0.18723712	1.76E-07
B128	B131	ALT	AST	0.670298342	0
B128	B140	ALT	r-GT	0.347307831	0
B128	B141	ALT	AST/ALT	-0.51382551	0
B128	B192	ALT	UA	0.278868919	3.55E-15
B128	B216	ALT	BMI	0.240341182	1.54E-11
B128	B206	ALT	Waist	0.23418686	5.14E-11
B128	B222	ALT	NS	0.219701999	7.75E-10
B128	B221	ALT	Hei	0.185089978	2.44E-07
B128	B215	ALT	BMI	0.184140007	2.82E-07
B128	B163	ALT	SG	0.18282922	3.43E-07
B129	B132	IBIL	Dbil	0.477419469	0
B129	B133	IBIL	TBIL	0.937383712	0
B130	B131	GLB	AST	0.238106936	2.39E-11
B130	B221	GLB	Hei	-0.20738672	6.74E-09
B130	B132	GLB	Dbil	0.197243876	3.63E-08
B131	B132	AST	Dbil	0.495840231	0
B131	B133	AST	TBIL	0.302475673	0

B131	B140	AST	r-GT	0.489017331	0
B131	B165	AST	bilirubin	0.36657622	0
B131	B177	AST	ACR	0.192064909	8.30E-08
B131	B173	AST	NTT	0.190951132	9.88E-08
B131	B175	AST	ALB-U	0.177045324	8.05E-07
B132	B133	Dbil	TBIL	0.753504186	0
B132	B140	Dbil	r-GT	0.446441165	0
B132	B165	Dbil	bilirubin	0.596053471	0
B133	B165	TBIL	bilirubin	0.306463593	0
B133	B140	TBIL	r-GT	0.280763062	2.22E-15
B133	B228	TBIL	OS	-0.18106055	4.46E-07
B134	B196	CHE	TCO2	0.282874961	1.33E-15
B134	B139	CHE	ADA	-0.27347846	1.27E-14
B134	B138	CHE	PAB	0.222921388	4.31E-10
B134	B200	CHE	OP	0.192582802	7.65E-08
B134	B195	CHE	Na	0.186369659	2.01E-07
B135	B136	TBA	5'NT	0.401503455	0
B135	B198	TBA	P	-0.63309753	0
B135	B200	TBA	OP	0.278195447	4.22E-15
B135	B194	TBA	Cl	-0.2432697	8.54E-12
B135	B199	TBA	Mg	0.19495745	5.24E-08
B136	B198	5'NT	P	-0.43803806	0
B136	B140	5'NT	r-GT	0.248439558	2.97E-12
B137	B199	AFU	Mg	0.385124044	0
B137	B138	AFU	PAB	0.198378621	3.02E-08
B138	B139	PAB	ADA	-0.34770626	0
B138	B199	PAB	Mg	0.406632552	0
B138	B193	PAB	K	0.276765411	5.77E-15
B138	B171	PAB	EC	0.224112653	3.46E-10
B138	B196	PAB	TCO2	0.217145528	1.23E-09
B138	B158	PAB	IgM	-0.21660017	1.35E-09
B138	B195	PAB	Na	0.204054261	1.18E-08
B138	B198	PAB	P	-0.18646453	1.98E-07
B138	B200	PAB	OP	0.179681458	5.47E-07
B139	B193	ADA	K	0.333193234	0
B139	B198	ADA	P	0.306729588	0
B139	B196	ADA	TCO2	-0.28133897	2.00E-15
B139	B197	ADA	AG	0.271529477	1.98E-14
B139	B150	ADA	ALP	0.228197706	1.61E-10
B139	B149	ADA	TGAb	0.183985416	2.88E-07
B14	B34	G-17	TK	-0.28662686	4.44E-16
B14	B85	G-17	SCC	-0.18765533	1.65E-07
B140	B165	r-GT	bilirubin	0.344221954	0
B140	B192	r-GT	UA	0.209507568	4.69E-09

B140	B141	r-GT	AST/ALT	-0.19107973	9.69E-08
B140	B206	r-GT	Waist	0.182439968	3.63E-07
B140	B145	r-GT	BGP	0.177427656	7.61E-07
B141	B216	AST/ALT	BMI	-0.32112622	0
B141	B206	AST/ALT	Waist	-0.2870654	4.44E-16
B141	B222	AST/ALT	NS	-0.28167751	1.78E-15
B141	B221	AST/ALT	Hei	-0.27761484	4.88E-15
B141	B192	AST/ALT	UA	-0.24857344	2.89E-12
B141	B210	AST/ALT	Hip	-0.22565886	2.60E-10
B141	B215	AST/ALT	BMI	-0.21826877	1.00E-09
B141	B218	AST/ALT	DBP	-0.17814574	6.85E-07
B142	B151	25-(OH)D	Anti-CCP	-0.37661338	0
B142	B143	25-(OH)D	β-CTx	0.21126171	3.46E-09
B142	B173	25-(OH)D	NTT	0.208036193	6.03E-09
B142	B165	25-(OH)D	bilirubin	-0.20672209	7.55E-09
B142	B168	25-(OH)D	NMP22	-0.18575873	2.20E-07
B143	B144	β-CTx	Total-PINP	0.895902823	0
B143	B145	β-CTx	BGP	0.883209723	0
B143	B149	β-CTx	TGAb	0.184058537	2.85E-07
B144	B145	Total-PINP	BGP	0.939814318	0
B144	B168	Total-PINP	NMP22	-0.2406817	1.44E-11
B144	B149	Total-PINP	TGAb	0.1857808	2.20E-07
B145	B149	BGP	TGAb	0.205113059	9.90E-09
B148	B149	Anti_TPO	TGAb	0.636893318	0
B149	B185	TGAb	PT	0.203950226	1.20E-08
B149	B184	TGAb	INR	0.200193229	2.25E-08
B149	B150	TGAb	ALP	0.178468955	6.54E-07
B15	B16	CD3-CD19+(B cell)	CD3+T	-0.45069499	0
B15	B17	CD3-CD19+(B cell)	CD3+CD16+CD56+NK	-0.63205007	0
B15	B19	CD3-CD19+(B cell)	CD3+CD4+/CD3+CD8+	0.353797565	0
B15	B197	CD3-CD19+(B cell)	AG	0.340023329	0
B15	B20	CD3-CD19+(B cell)	CD3+CD4+CD8+	-0.5312214	0
B15	B22	CD3-CD19+(B cell)	CD3+CD8+(T)	-0.34427819	0
B15	B100	CD3-CD19+(B cell)	anti-Ro52	0.261346919	1.92E-13
B15	B101	CD3-CD19+(B cell)	anti-Sc70	0.261346923	1.92E-13
B15	B102	CD3-CD19+(B cell)	anti-Sm	0.261346922	1.92E-13
B15	B104	CD3-CD19+(B cell)	anti-SSB	0.261346918	1.92E-13
B15	B105	CD3-CD19+(B cell)	anti-U1RNP	0.261346919	1.92E-13
B15	B106	CD3-CD19+(B cell)	anti-P	0.261346917	1.92E-13
B15	B107	CD3-CD19+(B cell)	AnuA	0.26134685	1.92E-13
B15	B108	CD3-CD19+(B cell)	Anti-Cyclin	0.261346919	1.92E-13
B15	B109	CD3-CD19+(B cell)	AMA-M2	0.261346918	1.92E-13
B15	B110	CD3-CD19+(B cell)	AHA	0.261346919	1.92E-13
B15	B95	CD3-CD19+(B cell)	anti-cenpb	0.261346917	1.92E-13

B15	B97	CD3-CD19+(B cell)	dsDNA	0.261346916	1.92E-13
B15	B98	CD3-CD19+(B cell)	anti-Jo-1	0.261346924	1.92E-13
B15	B99	CD3-CD19+(B cell)	anti-PM-Scl	0.26134692	1.92E-13
B15	B138	CD3-CD19+(B cell)	PAB	-0.24367094	7.88E-12
B15	B200	CD3-CD19+(B cell)	OP	0.227728421	1.76E-10
B15	B158	CD3-CD19+(B cell)	IgM	0.215666118	1.60E-09
B15	B135	CD3-CD19+(B cell)	TBA	0.208351037	5.72E-09
B15	B196	CD3-CD19+(B cell)	TCO2	-0.20504964	1.00E-08
B15	B195	CD3-CD19+(B cell)	Na	0.20434864	1.13E-08
B15	B164	CD3-CD19+(B cell)	COM	-0.19221959	8.10E-08
B15	B57	CD3-CD19+(B cell)	RBCAI	-0.17640898	8.82E-07
B150	B195	ALP	Na	-0.23252218	7.09E-11
B150	B200	ALP	OP	-0.18560995	2.25E-07
B151	B154	Anti-CCP	RF	0.316113776	0
B151	B168	Anti-CCP	NMP22	0.28186116	1.78E-15
B151	B175	Anti-CCP	ALB-U	0.259268795	3.02E-13
B151	B165	Anti-CCP	bilirubin	0.224129164	3.45E-10
B151	B177	Anti-CCP	ACR	0.20742436	6.70E-09
B151	B173	Anti-CCP	NTT	-0.18297974	3.35E-07
B152	B159	ACA-Ig(G-A-M)	β2-MG	0.999978385	0
B153	B170	Glu	U-GLU	0.309788707	0
B153	B220	Glu	SBP	0.246192983	4.72E-12
B153	B206	Glu	Waist	0.195266557	4.99E-08
B154	B173	RF	NTT	-0.26646964	6.20E-14
B154	B156	RF	IgE	0.255610792	6.61E-13
B154	B157	RF	IgG	-0.24618281	4.73E-12
B154	B183	RF	PTA	0.195567188	4.76E-08
B154	B158	RF	IgM	0.189602015	1.22E-07
B154	B182	RF	TT	-0.18158411	4.13E-07
B155	B157	IgA	IgG	0.490069448	0
B155	B158	IgA	IgM	0.509141775	0
B156	B193	IgE	K	-0.33072155	0
B156	B173	IgE	NTT	-0.2543743	8.60E-13
B157	B173	IgG	NTT	0.302574725	0
B158	B183	IgM	PTA	0.243232037	8.61E-12
B158	B182	IgM	TT	-0.22234724	4.79E-10
B158	B186	IgM	PFC	0.213477182	2.35E-09
B16	B19	CD3+T	CD3+CD4+/CD3+CD8+	-0.43358127	0
B16	B197	CD3+T	AG	-0.32256386	0
B16	B22	CD3+T	CD3+CD8+(T)	0.486163841	0
B16	B23	CD3+T	CD3-CD16+CD56+(NK)	-0.7960172	0
B16	B200	CD3+T	OP	-0.28591855	6.66E-16
B16	B138	CD3+T	PAB	0.28464763	8.88E-16
B16	B21	CD3+T	CD3+CD4-CD8-	0.280958834	2.22E-15

B16	B135	CD3+T	TBA	-0.25857081	3.50E-13
B16	B18	CD3+T	CD3+CD4+helper(inducer)T	0.254300173	8.73E-13
B16	B156	CD3+T	IgE	-0.25085664	1.80E-12
B16	B195	CD3+T	Na	-0.25013814	2.09E-12
B16	B20	CD3+T	CD3+CD4+CD8+	-0.22609141	2.40E-10
B16	B171	CD3+T	EC	0.215295352	1.71E-09
B16	B196	CD3+T	TCO2	0.188559511	1.43E-07
B16	B73	CD3+T	ApoA	0.180488832	4.86E-07
B163	B166	SG	Pro	0.421919185	0
B163	B190	SG	Cre	0.342284255	0
B163	B191	SG	Urea	0.315939404	0
B163	B216	SG	BMI	0.307904842	0
B163	B221	SG	Hei	0.344306291	0
B163	B222	SG	NS	0.357734843	0
B163	B206	SG	Waist	0.260057163	2.54E-13
B163	B192	SG	UA	0.226465053	2.23E-10
B163	B176	SG	Ucr	0.219086702	8.66E-10
B163	B209	SG	Smoking	0.205932493	8.63E-09
B163	B210	SG	Hip	0.196416373	4.15E-08
B166	B190	Pro	Cre	0.182432046	3.64E-07
B166	B221	Pro	Hei	0.176044221	9.30E-07
B166	B222	Pro	NS	0.175549928	9.99E-07
B167	B223	PC	FH	0.293940161	0
B17	B18	CD3+CD16+CD56+NK	CD3+CD4+helper(inducer)T	-0.33616565	0
B17	B19	CD3+CD16+CD56+NK	CD3+CD4+/CD3+CD8+	-0.58678119	0
B17	B20	CD3+CD16+CD56+NK	CD3+CD4+CD8+	0.709306717	0
B17	B21	CD3+CD16+CD56+NK	CD3+CD4-CD8-	-0.3723861	0
B17	B22	CD3+CD16+CD56+NK	CD3+CD8+(T)	0.631627392	0
B17	B23	CD3+CD16+CD56+NK	CD3-CD16+CD56+(NK)	0.33844168	0
B17	B34	CD3+CD16+CD56+NK	TK	0.341748786	0
B17	B193	CD3+CD16+CD56+NK	K	0.256591294	5.36E-13
B17	B164	CD3+CD16+CD56+NK	COM	0.253880236	9.54E-13
B17	B158	CD3+CD16+CD56+NK	IgM	-0.2065253	7.80E-09
B17	B138	CD3+CD16+CD56+NK	PAB	0.179565929	5.57E-07
B173	B193	NTT	K	0.202207165	1.61E-08
B174	B190	Urea/Cr	Cre	-0.54785908	0
B174	B191	Urea/Cr	Urea	0.619619055	0
B174	B221	Urea/Cr	Hei	-0.27470626	9.55E-15
B174	B222	Urea/Cr	NS	-0.26617815	6.62E-14
B174	B189	Urea/Cr	eGFB	0.25026561	2.04E-12
B174	B192	Urea/Cr	UA	-0.23433287	5.00E-11
B174	B216	Urea/Cr	BMI	-0.229973	1.15E-10
B174	B209	Urea/Cr	Smoking	-0.20506916	9.98E-09
B174	B210	Urea/Cr	Hip	-0.19853367	2.95E-08

B175	B176	ALB-U	Ucr	0.553118943	0
B175	B177	ALB-U	ACR	0.684695063	0
B176	B207	Ucr	WHR	-0.18387939	2.93E-07
B177	B178	ACR	Ig-U	0.399738107	0
B177	B179	ACR	α1-MG	0.311138845	0
B177	B180	ACR	Tf	0.388771067	0
B177	B207	ACR	WHR	0.198561867	2.93E-08
B178	B179	Ig-U	α1-MG	0.935848691	0
B178	B180	Ig-U	Tf	0.998283348	0
B178	B195	Ig-U	Na	-0.3476602	0
B178	B198	Ig-U	P	-0.33257487	0
B178	B194	Ig-U	Cl	-0.25785697	4.09E-13
B178	B200	Ig-U	OP	-0.22327163	4.04E-10
B179	B180	α1-MG	Tf	0.950139408	0
B179	B195	α1-MG	Na	-0.29981706	0
B179	B198	α1-MG	P	-0.37886954	0
B179	B207	α1-MG	WHR	-0.26676426	5.82E-14
B179	B194	α1-MG	Cl	-0.24895877	2.67E-12
B18	B100	CD3+CD4+helper(inducer)T	anti-Ro52	-0.3406733	0
B18	B101	CD3+CD4+helper(inducer)T	anti-Sc70	-0.34067331	0
B18	B102	CD3+CD4+helper(inducer)T	anti-Sm	-0.34067331	0
B18	B103	CD3+CD4+helper(inducer)T	anti-SSA/Ro	0.656567531	0
B18	B104	CD3+CD4+helper(inducer)T	anti-SSB	-0.3406733	0
B18	B105	CD3+CD4+helper(inducer)T	anti-U1RNP	-0.3406733	0
B18	B106	CD3+CD4+helper(inducer)T	anti-P	-0.3406733	0
B18	B107	CD3+CD4+helper(inducer)T	AnuA	-0.34067322	0
B18	B108	CD3+CD4+helper(inducer)T	Anti-Cyclin	-0.34067331	0
B18	B109	CD3+CD4+helper(inducer)T	AMA-M2	-0.3406733	0
B18	B110	CD3+CD4+helper(inducer)T	AHA	-0.34067331	0
B18	B139	CD3+CD4+helper(inducer)T	ADA	0.332512628	0
B18	B19	CD3+CD4+helper(inducer)T	CD3+CD4+/CD3+CD8+	0.655565755	0
B18	B22	CD3+CD4+helper(inducer)T	CD3+CD8+(T)	-0.51131103	0
B18	B23	CD3+CD4+helper(inducer)T	CD3-CD16+CD56+(NK)	-0.57814357	0
B18	B95	CD3+CD4+helper(inducer)T	anti-cenpb	-0.3406733	0
B18	B97	CD3+CD4+helper(inducer)T	dsDNA	-0.3406733	0
B18	B98	CD3+CD4+helper(inducer)T	anti-Jo-1	-0.34067331	0
B18	B99	CD3+CD4+helper(inducer)T	anti-PM-Scl	-0.3406733	0
B18	B179	CD3+CD4+helper(inducer)T	α1-MG	-0.22299576	4.25E-10
B18	B194	CD3+CD4+helper(inducer)T	Cl	-0.19652713	4.08E-08
B180	B195	Tf	Na	-0.34103068	0
B180	B198	Tf	P	-0.33805831	0
B180	B194	Tf	Cl	-0.26918076	3.38E-14
B180	B200	Tf	OP	-0.21576324	1.57E-09
B181	B182	APTT	TT	-0.32584637	0

B181	B184	APTT	INR	0.578421572	0
B181	B185	APTT	PT	0.688605045	0
B181	B186	APTT	PFC	-0.79579335	0
B181	B183	APTT	PTA	0.240714021	1.43E-11
B182	B183	TT	PTA	-0.30485813	0
B182	B184	TT	INR	-0.64040194	0
B182	B185	TT	PT	-0.53603432	0
B183	B184	PTA	INR	0.432610688	0
B183	B185	PTA	PT	0.380206094	0
B183	B186	PTA	PFC	-0.240197	1.58E-11
B184	B185	INR	PT	0.977961883	0
B184	B186	INR	PFC	-0.54523131	0
B185	B186	PT	PFC	-0.64453424	0
B189	B190	eGFB	Cre	-0.60814849	0
B189	B191	eGFB	Urea	-0.26906692	3.46E-14
B189	B206	eGFB	Waist	-0.23357619	5.79E-11
B189	B215	eGFB	BMI	-0.22373509	3.71E-10
B189	B192	eGFB	UA	-0.20398801	1.20E-08
B189	B220	eGFB	SBP	-0.2038483	1.22E-08
B189	B222	eGFB	NS	-0.19974384	2.42E-08
B19	B100	CD3+CD4+/CD3+CD8+	anti-Ro52	-0.29869143	0
B19	B101	CD3+CD4+/CD3+CD8+	anti-Sc70	-0.29869143	0
B19	B102	CD3+CD4+/CD3+CD8+	anti-Sm	-0.29869143	0
B19	B103	CD3+CD4+/CD3+CD8+	anti-SSA/Ro	0.406731378	0
B19	B104	CD3+CD4+/CD3+CD8+	anti-SSB	-0.29869142	0
B19	B105	CD3+CD4+/CD3+CD8+	anti-U1RNP	-0.29869143	0
B19	B106	CD3+CD4+/CD3+CD8+	anti-P	-0.29869142	0
B19	B107	CD3+CD4+/CD3+CD8+	AnuA	-0.29869135	0
B19	B108	CD3+CD4+/CD3+CD8+	Anti-Cyclin	-0.29869143	0
B19	B109	CD3+CD4+/CD3+CD8+	AMA-M2	-0.29869143	0
B19	B110	CD3+CD4+/CD3+CD8+	AHA	-0.29869143	0
B19	B138	CD3+CD4+/CD3+CD8+	PAB	-0.31885961	0
B19	B139	CD3+CD4+/CD3+CD8+	ADA	0.299503674	0
B19	B156	CD3+CD4+/CD3+CD8+	IgE	0.316035596	0
B19	B20	CD3+CD4+/CD3+CD8+	CD3+CD4+CD8+	-0.30558848	0
B19	B22	CD3+CD4+/CD3+CD8+	CD3+CD8+(T)	-0.96286334	0
B19	B95	CD3+CD4+/CD3+CD8+	anti-cenpb	-0.29869142	0
B19	B97	CD3+CD4+/CD3+CD8+	dsDNA	-0.29869142	0
B19	B98	CD3+CD4+/CD3+CD8+	anti-Jo-1	-0.29869143	0
B19	B99	CD3+CD4+/CD3+CD8+	anti-PM-Scl	-0.29869143	0
B19	B34	CD3+CD4+/CD3+CD8+	TK	-0.25553254	6.72E-13
B19	B171	CD3+CD4+/CD3+CD8+	EC	-0.23138245	8.82E-11
B19	B197	CD3+CD4+/CD3+CD8+	AG	0.22668673	2.14E-10
B19	B55	CD3+CD4+/CD3+CD8+	TK	0.206082632	8.41E-09

B19	B158	CD3+CD4+/CD3+CD8+	IgM	0.191339518	9.30E-08
B19	B193	CD3+CD4+/CD3+CD8+	K	-0.18294699	3.37E-07
B190	B192	Cre	UA	0.515739845	0
B190	B204	Cre	Drinking	0.341853085	0
B190	B206	Cre	Waist	0.414481993	0
B190	B209	Cre	Smoking	0.361782862	0
B190	B210	Cre	Hip	0.379291746	0
B190	B215	Cre	BMI	0.321941187	0
B190	B216	Cre	BMI	0.496673548	0
B190	B221	Cre	Hei	0.436858453	0
B190	B222	Cre	NS	0.549195986	0
B190	B191	Cre	Urea	0.257192711	4.71E-13
B191	B206	Urea	Waist	0.200793639	2.03E-08
B192	B206	UA	Waist	0.499450653	0
B192	B210	UA	Hip	0.404256929	0
B192	B215	UA	BMI	0.402815787	0
B192	B216	UA	BMI	0.525555577	0
B192	B221	UA	Hei	0.392422595	0
B192	B222	UA	NS	0.523938062	0
B192	B204	UA	Drinking	0.278164105	4.44E-15
B192	B209	UA	Smoking	0.203185537	1.37E-08
B194	B195	Cl	Na	0.309969928	0
B194	B197	Cl	AG	-0.24054021	1.48E-11
B194	B198	Cl	P	0.187256757	1.75E-07
B194	B200	Cl	OP	0.181089696	4.44E-07
B195	B197	Na	AG	0.377406652	0
B195	B200	Na	OP	0.896157741	0
B196	B197	TCO2	AG	-0.82834408	0
B196	B199	TCO2	Mg	-0.19397322	6.14E-08
B197	B200	AG	OP	0.294735475	0
B197	B199	AG	Mg	0.187959654	1.57E-07
B198	B199	P	Mg	-0.31088665	0
B2	B153	Age	Glu	0.311101739	0
B2	B189	Age	eGFB	-0.64933678	0
B2	B220	Age	SBP	0.346908745	0
B2	B43	Age	MCV	0.298284629	0
B2	B47	Age	RDW	0.348998023	0
B2	B52	Age	PLT/BPC	-0.26919932	3.38E-14
B2	B126	Age	Alb	-0.26525038	8.13E-14
B2	B219	Age	OT	0.257108465	4.80E-13
B2	B46	Age	RBC Count	-0.2362646	3.43E-11
B2	B77	Age	TC	0.233953394	5.38E-11
B2	B227	Age	OD	-0.23127994	9.00E-11
B2	B44	Age	MCH	0.224507026	3.22E-10

B2	B221	Age	Hei	-0.22333977	3.99E-10
B2	B228	Age	OS	-0.21859666	9.46E-10
B2	B206	Age	Waist	0.215382494	1.68E-09
B2	B191	Age	Urea	0.21042167	4.00E-09
B2	B70	Age	LDL-C	0.177942803	7.06E-07
B20	B164	CD3+CD4+CD8+	COM	0.348935773	0
B20	B21	CD3+CD4+CD8+	CD3+CD4-CD8-	-0.63374153	0
B20	B22	CD3+CD4+CD8+	CD3+CD8+(T)	0.308015767	0
B20	B23	CD3+CD4+CD8+	CD3-CD16+CD56+(NK)	0.574050822	0
B20	B161	CD3+CD4+CD8+	LEU	0.229360173	1.30E-10
B20	B87	CD3+CD4+CD8+	NSE	-0.2123356	2.87E-09
B204	B209	Drinking	Smoking	0.404511543	0
B204	B216	Drinking	BMI	0.318800804	0
B204	B221	Drinking	Hei	0.302570354	0
B204	B222	Drinking	NS	0.33296055	0
B204	B206	Drinking	Waist	0.262150261	1.61E-13
B204	B210	Drinking	Hip	0.247691965	3.47E-12
B204	B215	Drinking	BMI	0.191955168	8.44E-08
B206	B210	Waist	Hip	0.776324217	0
B206	B215	Waist	BMI	0.796460944	0
B206	B216	Waist	BMI	0.831090816	0
B206	B220	Waist	SBP	0.30696212	0
B206	B221	Waist	Hei	0.425655252	0
B206	B222	Waist	NS	0.743938554	0
B206	B218	Waist	DBP	0.270565002	2.46E-14
B206	B209	Waist	Smoking	0.24369015	7.85E-12
B206	B207	Waist	WHR	0.22007991	7.24E-10
B209	B222	Smoking	NS	0.360957639	0
B209	B221	Smoking	Hei	0.283611433	1.33E-15
B209	B216	Smoking	BMI	0.268769807	3.69E-14
B209	B210	Smoking	Hip	0.182583255	3.56E-07
B21	B198	CD3+CD4-CD8-	P	0.308452975	0
B21	B23	CD3+CD4-CD8-	CD3-CD16+CD56+(NK)	-0.44158433	0
B21	B22	CD3+CD4-CD8-	CD3+CD8+(T)	-0.25172786	1.50E-12
B21	B164	CD3+CD4-CD8-	COM	-0.20863464	5.44E-09
B21	B197	CD3+CD4-CD8-	AG	-0.20191072	1.69E-08
B21	B194	CD3+CD4-CD8-	Cl	0.188569851	1.43E-07
B210	B215	Hip	BMI	0.751987374	0
B210	B216	Hip	BMI	0.837949596	0
B210	B221	Hip	Hei	0.494110207	0
B210	B222	Hip	NS	0.668783266	0
B210	B218	Hip	DBP	0.263154711	1.29E-13
B210	B220	Hip	SBP	0.210200031	4.16E-09
B211	B212	IOP-R	IOP-L	0.797642859	0

B213	B214	CVA-R	CVA-L	0.742748637	0
B213	B228	CVA-R	OS	-0.17916958	5.90E-07
B214	B228	CVA-L	OS	-0.18665127	1.92E-07
B215	B216	BMI	BMI	0.82273833	0
B215	B222	BMI	NS	0.639314006	0
B215	B218	BMI	DBP	0.282006954	1.78E-15
B215	B220	BMI	SBP	0.28184145	1.78E-15
B216	B218	BMI	DBP	0.326969218	0
B216	B221	BMI	Hei	0.681320176	0
B216	B222	BMI	NS	0.799814215	0
B216	B220	BMI	SBP	0.267816065	4.57E-14
B218	B220	DBP	SBP	0.668038492	0
B218	B222	DBP	NS	0.28462791	8.88E-16
B218	B221	DBP	Hei	0.208134483	5.93E-09
B22	B138	CD3+CD8+(T)	PAB	0.342578812	0
B22	B156	CD3+CD8+(T)	IgE	-0.34681166	0
B22	B34	CD3+CD8+(T)	TK	0.288694411	4.44E-16
B22	B171	CD3+CD8+(T)	EC	0.258805011	3.33E-13
B22	B139	CD3+CD8+(T)	ADA	-0.25006074	2.13E-12
B22	B103	CD3+CD8+(T)	anti-SSA/Ro	-0.23316021	6.27E-11
B22	B55	CD3+CD8+(T)	TK	-0.22739041	1.88E-10
B22	B158	CD3+CD8+(T)	IgM	-0.22063491	6.54E-10
B22	B193	CD3+CD8+(T)	K	0.189641328	1.21E-07
B22	B100	CD3+CD8+(T)	anti-Ro52	0.18416581	2.80E-07
B22	B101	CD3+CD8+(T)	anti-Sc70	0.184165812	2.80E-07
B22	B102	CD3+CD8+(T)	anti-Sm	0.184165812	2.80E-07
B22	B104	CD3+CD8+(T)	anti-SSB	0.184165809	2.80E-07
B22	B105	CD3+CD8+(T)	anti-U1RNP	0.18416581	2.80E-07
B22	B106	CD3+CD8+(T)	anti-P	0.184165809	2.80E-07
B22	B107	CD3+CD8+(T)	AnuA	0.184165762	2.80E-07
B22	B108	CD3+CD8+(T)	Anti-Cyclin	0.18416581	2.80E-07
B22	B109	CD3+CD8+(T)	AMA-M2	0.18416581	2.80E-07
B22	B110	CD3+CD8+(T)	AHA	0.184165811	2.80E-07
B22	B95	CD3+CD8+(T)	anti-cenpb	0.184165809	2.80E-07
B22	B97	CD3+CD8+(T)	dsDNA	0.184165809	2.80E-07
B22	B98	CD3+CD8+(T)	anti-Jo-1	0.184165812	2.80E-07
B22	B99	CD3+CD8+(T)	anti-PM-Scl	0.18416581	2.80E-07
B220	B222	SBP	NS	0.2501207	2.10E-12
B221	B222	Hei	NS	0.576417327	0
B227	B228	OD	OS	0.823836625	0
B23	B103	CD3-CD16+CD56+(NK)	anti-SSA/Ro	-0.39446951	0
B23	B135	CD3-CD16+CD56+(NK)	TBA	0.219896019	7.48E-10
B23	B200	CD3-CD16+CD56+(NK)	OP	0.200343204	2.19E-08
B23	B164	CD3-CD16+CD56+(NK)	COM	0.197564396	3.45E-08

B24	B26	αHBDH	CKMB	0.528244613	0
B24	B27	αHBDH	LDH	0.816539224	0
B24	B132	αHBDH	Dbil	0.257256845	4.65E-13
B24	B47	αHBDH	RDW	0.243139576	8.77E-12
B24	B25	αHBDH	CK	0.225481449	2.68E-10
B24	B131	αHBDH	AST	0.210214701	4.15E-09
B25	B26	CK	CKMB	0.326845338	0
B25	B27	CK	LDH	0.236478099	3.29E-11
B26	B27	CKMB	LDH	0.46005364	0
B26	B149	CKMB	TGAb	0.21118749	3.51E-09
B26	B132	CKMB	Dbil	0.185090845	2.44E-07
B26	B131	CKMB	AST	0.175960727	9.41E-07
B27	B131	LDH	AST	0.228606685	1.49E-10
B27	B47	LDH	RDW	0.186705104	1.91E-07
B28	B139	PRL	ADA	0.346967777	0
B28	B29	PRL	E2	0.793082169	0
B28	B31	PRL	FSH	-0.57555326	0
B28	B92	PRL	ALDH2-Glu504Glu	0.538568014	0
B28	B93	PRL	ALDH2-Glu504Lys	-0.53856801	0
B28	B94	PRL	ALDH2-Lys504Lys	-0.53856801	0
B28	B138	PRL	PAB	-0.28030079	2.66E-15
B28	B33	PRL	Prog	0.253116099	1.12E-12
B28	B39	PRL	Other cells	-0.2203986	6.83E-10
B28	B156	PRL	IgE	-0.21567959	1.59E-09
B28	B30	PRL	LH	-0.17884605	6.19E-07
B29	B138	E2	PAB	-0.35912027	0
B29	B139	E2	ADA	0.444585851	0
B29	B31	E2	FSH	-0.47535163	0
B29	B92	E2	ALDH2-Glu504Glu	0.689359117	0
B29	B93	E2	ALDH2-Glu504Lys	-0.68935912	0
B29	B94	E2	ALDH2-Lys504Lys	-0.68935911	0
B29	B156	E2	IgE	-0.27588767	7.11E-15
B29	B33	E2	Prog	0.241390664	1.25E-11
B29	B198	E2	P	0.204213031	1.15E-08
B29	B194	E2	Cl	0.185923017	2.15E-07
B29	B32	E2	Testo	-0.17901784	6.03E-07
B29	B39	E2	Other cells	-0.17623682	9.04E-07
B3	B123	Hcy	hs-CRP1	0.337662309	0
B3	B132	Hcy	Dbil	0.320430719	0
B3	B24	Hcy	αHBDH	0.252263156	1.34E-12
B3	B165	Hcy	bilirubin	0.227965571	1.69E-10
B3	B27	Hcy	LDH	0.227044226	2.01E-10
B3	B47	Hcy	RDW	0.209005801	5.11E-09
B3	B131	Hcy	AST	0.177071292	8.02E-07

B30	B31	LH	FSH	0.872222492	0
B30	B33	LH	Prog	-0.29809925	0
B30	B173	LH	NTT	0.210520952	3.93E-09
B31	B33	FSH	Prog	-0.36839696	0
B31	B92	FSH	ALDH2-Glu504Glu	-0.29669961	0
B31	B93	FSH	ALDH2-Glu504Lys	0.296699612	0
B31	B94	FSH	ALDH2-Lys504Lys	0.29669961	0
B31	B173	FSH	NTT	0.214448826	1.98E-09
B31	B139	FSH	ADA	-0.1916835	8.81E-08
B31	B39	FSH	Other cells	0.175835174	9.58E-07
B32	B33	Testo	Prog	0.779485975	0
B32	B92	Testo	ALDH2-Glu504Glu	-0.25930635	2.99E-13
B32	B93	Testo	ALDH2-Glu504Lys	0.25930635	2.99E-13
B32	B94	Testo	ALDH2-Lys504Lys	0.259306348	2.99E-13
B33	B173	Prog	NTT	-0.25504185	7.46E-13
B34	B193	TK	K	0.377638141	0
B34	B85	TK	SCC	0.227962206	1.69E-10
B34	B156	TK	IgE	-0.22710487	1.98E-10
B34	B87	TK	NSE	0.205898221	8.68E-09
B34	B145	TK	BGP	-0.19871512	2.86E-08
B34	B197	TK	AG	-0.18494693	2.49E-07
B34	B144	TK	Total-PINP	-0.18299442	3.35E-07
B34	B143	TK	β-CTx	-0.17755774	7.47E-07
B35	B167	β2-MG	PC	0.480572312	0
B35	B37	β2-MG	BAND	0.846787565	0
B35	B96	β2-MG	dsDNA (IIF)	0.771415561	0
B36	B116	V-Lym%	TT3	0.317363237	0
B39	B79	Other cells	hb V -dna	0.356399017	0
B39	B75	Other cells	ApoA1/B	-0.26985239	2.89E-14
B39	B76	Other cells	ApoB	0.209304084	4.85E-09
B39	B154	Other cells	RF	0.205463888	9.34E-09
B39	B145	Other cells	BGP	-0.20135933	1.85E-08
B39	B175	Other cells	ALB-U	0.194924582	5.27E-08
B39	B144	Other cells	Total-PINP	-0.18331082	3.19E-07
B39	B143	Other cells	β-CTx	-0.18045603	4.88E-07
B4	B5	WB-Ca	WB-Mg	0.945885434	0
B4	B6	WB-Ca	WB-Fe	0.973106048	0
B4	B112	WB-Ca	FT4	0.207471537	6.64E-09
B4	B7	WB-Ca	WB-Cop	0.187889218	1.59E-07
B40	B42	WBC	MON	0.647252762	0
B40	B48	WBC	LYM%	-0.32131988	0
B40	B53	WBC	GR%	0.331446569	0
B40	B206	WBC	Waist	0.241098441	1.32E-11
B40	B216	WBC	BMI	0.226747536	2.12E-10

B40	B51	WBC	Hb	0.222124606	4.99E-10
B40	B52	WBC	PLT/BPC	0.211818268	3.14E-09
B40	B215	WBC	BMI	0.208182217	5.88E-09
B40	B209	WBC	Smoking	0.204589552	1.08E-08
B40	B222	WBC	NS	0.203134206	1.38E-08
B40	B210	WBC	Hip	0.202738425	1.47E-08
B40	B46	WBC	RBC Count	0.186061119	2.10E-07
B40	B72	WBC	HDL-C	-0.17884575	6.19E-07
B41	B42	MON％	MON	0.649491808	0
B41	B53	MON％	GR%	-0.2668274	5.73E-14
B42	B209	MON	Smoking	0.287527218	4.44E-16
B42	B51	MON	Hb	0.263168132	1.29E-13
B42	B123	MON	hs-CRP1	0.245543474	5.39E-12
B42	B72	MON	HDL-C	-0.23858274	2.18E-11
B42	B206	MON	Waist	0.226147804	2.37E-10
B42	B216	MON	BMI	0.218936942	8.90E-10
B42	B48	MON	LYM%	-0.21852349	9.59E-10
B42	B222	MON	NS	0.213869195	2.19E-09
B42	B140	MON	r-GT	0.210472505	3.97E-09
B42	B221	MON	Hei	0.197582296	3.44E-08
B42	B46	MON	RBC Count	0.193686931	6.42E-08
B42	B192	MON	UA	0.191917234	8.49E-08
B42	B58	MON	HCT	0.185809038	2.19E-07
B42	B131	MON	AST	0.181649002	4.09E-07
B42	B69	MON	PV	-0.17832111	6.68E-07
B42	B190	MON	Cre	0.178243601	6.76E-07
B42	B210	MON	Hip	0.176675268	8.49E-07
B43	B189	MCV	eGFB	-0.30806825	0
B43	B44	MCV	MCH	0.907616211	0
B43	B46	MCV	RBC Count	-0.51234081	0
B43	B47	MCV	RDW	0.495853842	0
B43	B45	MCV	MCHC	0.26266172	1.44E-13
B43	B77	MCV	TC	0.225659139	2.60E-10
B43	B52	MCV	PLT/BPC	-0.19805459	3.18E-08
B43	B70	MCV	LDL-C	0.196797276	3.90E-08
B43	B51	MCV	Hb	0.192251837	8.06E-08
B44	B45	MCH	MCHC	0.641376328	0
B44	B46	MCH	RBC Count	-0.35310216	0
B44	B47	MCH	RDW	0.305218724	0
B44	B51	MCH	Hb	0.408788772	0
B44	B189	MCH	eGFB	-0.2722022	1.69E-14
B44	B190	MCH	Cre	0.226866291	2.07E-10
B44	B52	MCH	PLT/BPC	-0.20531425	9.57E-09
B44	B209	MCH	Smoking	0.191724636	8.76E-08

B44	B77	MCH	TC	0.179652839	5.50E-07
B45	B51	MCHC	Hb	0.59082528	0
B45	B190	MCHC	Cre	0.23081079	9.84E-11
B45	B141	MCHC	AST/ALT	-0.22459243	3.17E-10
B45	B47	MCHC	RDW	-0.21950682	8.03E-10
B45	B209	MCHC	Smoking	0.194897274	5.30E-08
B45	B222	MCHC	NS	0.194455548	5.68E-08
B45	B221	MCHC	Hei	0.181863172	3.96E-07
B45	B216	MCHC	BMI	0.179303976	5.79E-07
B46	B141	RBC Count	AST/ALT	-0.33055272	0
B46	B163	RBC Count	SG	0.31583356	0
B46	B190	RBC Count	Cre	0.313722025	0
B46	B192	RBC Count	UA	0.420686272	0
B46	B206	RBC Count	Waist	0.337568754	0
B46	B210	RBC Count	Hip	0.294039978	0
B46	B216	RBC Count	BMI	0.429131945	0
B46	B221	RBC Count	Hei	0.462019395	0
B46	B222	RBC Count	NS	0.444174895	0
B46	B47	RBC Count	RDW	-0.41399313	0
B46	B51	RBC Count	Hb	0.699524485	0
B46	B218	RBC Count	DBP	0.282896869	1.33E-15
B46	B126	RBC Count	Alb	0.274651449	9.77E-15
B46	B127	RBC Count	A-G ratio	0.239004654	2.00E-11
B46	B128	RBC Count	ALT	0.228760499	1.45E-10
B46	B209	RBC Count	Smoking	0.225547888	2.65E-10
B46	B215	RBC Count	BMI	0.222769936	4.43E-10
B46	B72	RBC Count	HDL-C	-0.21922088	8.45E-10
B46	B204	RBC Count	Drinking	0.213161635	2.48E-09
B47	B189	RDW	eGFB	-0.30716828	0
B47	B111	RDW	TSH	0.263590151	1.17E-13
B47	B112	RDW	FT4	-0.24843181	2.98E-12
B47	B132	RDW	Dbil	0.244548562	6.59E-12
B47	B77	RDW	TC	0.238709201	2.12E-11
B47	B131	RDW	AST	0.23310689	6.34E-11
B47	B123	RDW	hs-CRP1	0.212386531	2.84E-09
B47	B130	RDW	GLB	0.20564873	9.05E-09
B47	B127	RDW	A-G ratio	-0.1983568	3.03E-08
B47	B113	RDW	FT3	-0.19658721	4.04E-08
B47	B221	RDW	Hei	-0.19543667	4.86E-08
B47	B70	RDW	LDL-C	0.187865683	1.60E-07
B47	B52	RDW	PLT/BPC	-0.18620796	2.06E-07
B47	B141	RDW	AST/ALT	0.179583091	5.55E-07
B48	B53	LYM%	GR%	-0.94590657	0
B49	B50	baso%	eo%	0.29776153	0

B49	B53	baso%	GR%	-0.17979503	5.38E-07
B49	B190	baso%	Cre	0.175637588	9.86E-07
B5	B6	WB-Mg	WB-Fe	0.995198051	0
B5	B7	WB-Mg	WB-Cop	0.496443154	0
B5	B8	WB-Mg	WB-Zinc	0.274549432	9.77E-15
B5	B78	WB-Mg	HBcAb	-0.20806435	6.00E-09
B5	B112	WB-Mg	FT4	0.18829976	1.49E-07
B50	B165	eo%	bilirubin	-0.27234808	1.64E-14
B50	B53	eo%	GR%	-0.25028149	2.03E-12
B51	B141	Hb	AST/ALT	-0.35465245	0
B51	B163	Hb	SG	0.338470429	0
B51	B190	Hb	Cre	0.485907	0
B51	B192	Hb	UA	0.41466518	0
B51	B204	Hb	Drinking	0.324514643	0
B51	B206	Hb	Waist	0.369265867	0
B51	B209	Hb	Smoking	0.375460985	0
B51	B210	Hb	Hip	0.317918819	0
B51	B216	Hb	BMI	0.468117623	0
B51	B221	Hb	Hei	0.483059479	0
B51	B222	Hb	NS	0.511494621	0
B51	B126	Hb	Alb	0.27936192	3.11E-15
B51	B174	Hb	Urea/Cr	-0.27960223	3.11E-15
B51	B128	Hb	ALT	0.278145203	4.44E-15
B51	B218	Hb	DBP	0.274620616	9.77E-15
B51	B215	Hb	BMI	0.253499979	1.03E-12
B51	B127	Hb	A-G ratio	0.251201082	1.68E-12
B51	B71	Hb	TG	0.209891715	4.39E-09
B51	B166	Hb	Pro	0.179036881	6.02E-07
B51	B62	Hb	WBHSRVI	0.176479625	8.73E-07
B52	B189	PLT/BPC	eGFB	0.176329148	8.92E-07
B54	B56	ESR	IR	-0.51592531	0
B54	B58	ESR	HCT	-0.35865271	0
B54	B60	ESR	WBRVI	-0.5224656	0
B54	B61	ESR	WBHSRV	-0.33423326	0
B54	B62	ESR	WBHSRVI	-0.51142634	0
B54	B63	ESR	WBRSR(mPas_200)	-0.32152764	0
B54	B64	ESR	WBRSR(mPas_5)	-0.40125561	0
B54	B65	ESR	WBRSR(mPas_1)	-0.40252247	0
B54	B66	ESR	WBRSR(mPas_100)	-0.3475621	0
B54	B67	ESR	WBRSR(mPas_50)	-0.36395032	0
B54	B69	ESR	PV	0.585339698	0
B54	B79	ESR	hb V -dna	-0.28361006	1.33E-15
B54	B182	ESR	TT	-0.27019359	2.69E-14
B54	B156	ESR	IgE	0.258110147	3.87E-13

B54	B111	ESR	TSH	0.23665683	3.18E-11
B54	B59	ESR	WBRV	-0.228548	1.51E-10
B54	B125	ESR	Cor	0.224203067	3.40E-10
B54	B55	ESR	TK	-0.22241864	4.73E-10
B54	B81	ESR	TSGF	0.221713142	5.38E-10
B54	B75	ESR	ApoA1/B	0.196319313	4.22E-08
B54	B57	ESR	RBCAI	-0.19616996	4.32E-08
B55	B56	TK	IR	0.558048855	0
B55	B58	TK	HCT	-0.41201262	0
B55	B59	TK	WBRV	0.537112439	0
B55	B61	TK	WBHSRV	0.500025604	0
B55	B69	TK	PV	-0.26369065	1.15E-13
B55	B60	TK	WBRVI	0.210334699	4.06E-09
B55	B136	TK	5'NT	-0.19632295	4.21E-08
B55	B182	TK	TT	0.178224324	6.78E-07
B56	B182	IR	TT	0.296731276	0
B56	B58	IR	HCT	0.506579134	0
B56	B59	IR	WBRV	0.46812105	0
B56	B60	IR	WBRVI	0.766175403	0
B56	B61	IR	WBHSRV	0.911990931	0
B56	B62	IR	WBHSRVI	0.893615015	0
B56	B63	IR	WBRSR(mPas_200)	0.766215074	0
B56	B64	IR	WBRSR(mPas_5)	0.773183109	0
B56	B65	IR	WBRSR(mPas_1)	0.678534872	0
B56	B66	IR	WBRSR(mPas_100)	0.796728953	0
B56	B67	IR	WBRSR(mPas_50)	0.803873014	0
B56	B69	IR	PV	-0.43057919	0
B57	B100	RBCAI	anti-Ro52	-0.30683775	0
B57	B101	RBCAI	anti-Sc70	-0.30683776	0
B57	B102	RBCAI	anti-Sm	-0.30683775	0
B57	B104	RBCAI	anti-SSB	-0.30683775	0
B57	B105	RBCAI	anti-U1RNP	-0.30683775	0
B57	B106	RBCAI	anti-P	-0.30683775	0
B57	B107	RBCAI	AnuA	-0.30683767	0
B57	B108	RBCAI	Anti-Cyclin	-0.30683775	0
B57	B109	RBCAI	AMA-M2	-0.30683775	0
B57	B110	RBCAI	AHA	-0.30683775	0
B57	B136	RBCAI	5'NT	0.447927009	0
B57	B59	RBCAI	WBRV	0.789469281	0
B57	B60	RBCAI	WBRVI	0.570550029	0
B57	B65	RBCAI	WBRSR(mPas_1)	0.518240627	0
B57	B95	RBCAI	anti-cenpb	-0.30683775	0
B57	B97	RBCAI	dsDNA	-0.30683775	0
B57	B98	RBCAI	anti-Jo-1	-0.30683776	0

B57	B99	RBCAI	anti-PM-Scl	-0.30683775	0
B57	B64	RBCAI	WBRSR(mPas_5)	0.263118609	1.30E-13
B57	B69	RBCAI	PV	-0.2290386	1.38E-10
B57	B171	RBCAI	EC	0.194946944	5.25E-08
B58	B134	HCT	CHE	0.308802914	0
B58	B60	HCT	WBRVI	0.592404372	0
B58	B61	HCT	WBHSRV	0.4948534	0
B58	B62	HCT	WBHSRVI	0.830221429	0
B58	B63	HCT	WBRSR(mPas_200)	0.836169256	0
B58	B64	HCT	WBRSR(mPas_5)	0.763537211	0
B58	B65	HCT	WBRSR(mPas_1)	0.632635154	0
B58	B66	HCT	WBRSR(mPas_100)	0.834728621	0
B58	B67	HCT	WBRSR(mPas_50)	0.828352232	0
B58	B194	HCT	CI	-0.2399335	1.67E-11
B59	B60	WBRV	WBRVI	0.695987962	0
B59	B61	WBRV	WBHSRV	0.504052707	0
B59	B64	WBRV	WBRSR(mPas_5)	0.557786328	0
B59	B65	WBRV	WBRSR(mPas_1)	0.722047272	0
B59	B66	WBRV	WBRSR(mPas_100)	0.299545247	0
B59	B67	WBRV	WBRSR(mPas_50)	0.357311238	0
B59	B136	WBRV	5'NT	0.287708939	4.44E-16
B59	B62	WBRV	WBHSRVI	0.251927711	1.44E-12
B59	B63	WBRV	WBRSR(mPas_200)	0.243832076	7.62E-12
B59	B100	WBRV	anti-Ro52	-0.19831357	3.05E-08
B59	B101	WBRV	anti-Sc70	-0.19831358	3.05E-08
B59	B102	WBRV	anti-Sm	-0.19831358	3.05E-08
B59	B104	WBRV	anti-SSB	-0.19831357	3.05E-08
B59	B105	WBRV	anti-U1RNP	-0.19831357	3.05E-08
B59	B106	WBRV	anti-P	-0.19831357	3.05E-08
B59	B107	WBRV	AnuA	-0.19831352	3.05E-08
B59	B108	WBRV	Anti-Cyclin	-0.19831357	3.05E-08
B59	B109	WBRV	AMA-M2	-0.19831357	3.05E-08
B59	B110	WBRV	AHA	-0.19831357	3.05E-08
B59	B95	WBRV	anti-cenpb	-0.19831357	3.05E-08
B59	B97	WBRV	dsDNA	-0.19831357	3.05E-08
B59	B98	WBRV	anti-Jo-1	-0.19831358	3.05E-08
B59	B99	WBRV	anti-PM-Scl	-0.19831357	3.05E-08
B6	B7	WB-Fe	WB-Cop	0.409091112	0
B6	B112	WB-Fe	FT4	0.196252109	4.26E-08
B6	B8	WB-Fe	WB-Zinc	0.1791106	5.95E-07
B60	B136	WBRVI	5'NT	0.291883456	0
B60	B61	WBRVI	WBHSRV	0.64707978	0
B60	B62	WBRVI	WBHSRVI	0.794327568	0
B60	B63	WBRVI	WBRSR(mPas_200)	0.64631546	0

B60	B64	WBRVI	WBRSR(mPas_5)	0.875924544	0
B60	B65	WBRVI	WBRSR(mPas_1)	0.923559518	0
B60	B66	WBRVI	WBRSR(mPas_100)	0.70802383	0
B60	B67	WBRVI	WBRSR(mPas_50)	0.751748454	0
B60	B69	WBRVI	PV	-0.44620318	0
B60	B140	WBRVI	r-GT	0.183459676	3.12E-07
B61	B62	WBHSRV	WBHSRVI	0.814460802	0
B61	B63	WBHSRV	WBRSR(mPas_200)	0.868088572	0
B61	B64	WBHSRV	WBRSR(mPas_5)	0.819182142	0
B61	B65	WBHSRV	WBRSR(mPas_1)	0.707044118	0
B61	B66	WBHSRV	WBRSR(mPas_100)	0.876040536	0
B61	B67	WBHSRV	WBRSR(mPas_50)	0.876449711	0
B61	B182	WBHSRV	TT	0.245742045	5.17E-12
B61	B194	WBHSRV	CI	-0.199195	2.64E-08
B61	B135	WBHSRV	TBA	0.178682635	6.34E-07
B62	B63	WBHSRVI	WBRSR(mPas_200)	0.903372348	0
B62	B64	WBHSRVI	WBRSR(mPas_5)	0.872379257	0
B62	B65	WBHSRVI	WBRSR(mPas_1)	0.742711315	0
B62	B66	WBHSRVI	WBRSR(mPas_100)	0.926674077	0
B62	B67	WBHSRVI	WBRSR(mPas_50)	0.927557645	0
B62	B69	WBHSRVI	PV	-0.38662854	0
B62	B134	WBHSRVI	CHE	0.262956287	1.35E-13
B62	B182	WBHSRVI	TT	0.261038308	2.05E-13
B63	B64	WBRSR(mPas_200)	WBRSR(mPas_5)	0.900815154	0
B63	B65	WBRSR(mPas_200)	WBRSR(mPas_1)	0.749793961	0
B63	B66	WBRSR(mPas_200)	WBRSR(mPas_100)	0.993599967	0
B63	B67	WBRSR(mPas_200)	WBRSR(mPas_50)	0.985915058	0
B63	B194	WBRSR(mPas_200)	CI	-0.29140005	2.22E-16
B63	B134	WBRSR(mPas_200)	CHE	0.235777911	3.78E-11
B63	B182	WBRSR(mPas_200)	TT	0.196087308	4.38E-08
B63	B75	WBRSR(mPas_200)	ApoA1/B	-0.18513784	2.42E-07
B63	B135	WBRSR(mPas_200)	TBA	0.180969263	4.52E-07
B64	B65	WBRSR(mPas_5)	WBRSR(mPas_1)	0.9581316	0
B64	B66	WBRSR(mPas_5)	WBRSR(mPas_100)	0.928233561	0
B64	B67	WBRSR(mPas_5)	WBRSR(mPas_50)	0.950267496	0
B64	B136	WBRSR(mPas_5)	5'NT	0.239347537	1.87E-11
B64	B194	WBRSR(mPas_5)	CI	-0.22785677	1.72E-10
B64	B134	WBRSR(mPas_5)	CHE	0.183007916	3.34E-07
B65	B136	WBRSR(mPas_1)	5'NT	0.349219406	0
B65	B66	WBRSR(mPas_1)	WBRSR(mPas_100)	0.792158501	0
B65	B67	WBRSR(mPas_1)	WBRSR(mPas_50)	0.831694194	0
B65	B194	WBRSR(mPas_1)	CI	-0.1756314	9.87E-07
B66	B67	WBRSR(mPas_100)	WBRSR(mPas_50)	0.997466292	0
B66	B194	WBRSR(mPas_100)	CI	-0.2767149	6.00E-15

B66	B134	WBRSR(mPas_100)	CHE	0.23729014	2.81E-11
B66	B182	WBRSR(mPas_100)	TT	0.206312012	8.09E-09
B67	B194	WBRSR(mPas_50)	Cl	-0.27041492	2.55E-14
B67	B134	WBRSR(mPas_50)	CHE	0.230598136	1.02E-10
B67	B182	WBRSR(mPas_50)	TT	0.203578505	1.28E-08
B69	B194	PV	Cl	-0.22186866	5.23E-10
B69	B193	PV	K	0.197544637	3.46E-08
B69	B111	PV	TSH	0.192719907	7.48E-08
B69	B135	PV	TBA	0.186472086	1.98E-07
B69	B199	PV	Mg	0.184837771	2.53E-07
B69	B154	PV	RF	0.179113353	5.95E-07
B7	B78	WB-Cop	HBcAb	-0.38833911	0
B7	B8	WB-Cop	WB-Zinc	0.971010112	0
B7	B156	WB-Cop	IgE	0.280583073	2.44E-15
B7	B81	WB-Cop	TSGF	0.246173808	4.74E-12
B7	B39	WB-Cop	Other cells	0.205068515	9.98E-09
B7	B154	WB-Cop	RF	0.186777445	1.89E-07
B7	B157	WB-Cop	IgG	-0.17984912	5.34E-07
B70	B77	LDL-C	TC	0.856266163	0
B70	B76	LDL-C	ApoB	0.286174136	6.66E-16
B70	B189	LDL-C	eGFB	-0.26471085	9.15E-14
B70	B91	LDL-C	TP	0.240744983	1.42E-11
B70	B112	LDL-C	FT4	-0.22529031	2.78E-10
B70	B190	LDL-C	Cre	0.208000529	6.07E-09
B70	B215	LDL-C	BMI	0.203266405	1.35E-08
B70	B206	LDL-C	Waist	0.188324445	1.49E-07
B70	B126	LDL-C	Alb	0.180665376	4.73E-07
B70	B132	LDL-C	Dbil	-0.17980337	5.38E-07
B70	B75	LDL-C	ApoA1/B	-0.17940029	5.70E-07
B71	B206	TG	Waist	0.349446424	0
B71	B215	TG	BMI	0.326707897	0
B71	B216	TG	BMI	0.302178237	0
B71	B77	TG	TC	0.336817947	0
B71	B222	TG	NS	0.289636968	2.22E-16
B71	B192	TG	UA	0.281079757	2.22E-15
B71	B190	TG	Cre	0.235700919	3.83E-11
B71	B72	TG	HDL-C	-0.23231163	7.38E-11
B71	B210	TG	Hip	0.219454251	8.11E-10
B71	B189	TG	eGFB	-0.21414075	2.09E-09
B71	B218	TG	DBP	0.194575543	5.57E-08
B71	B141	TG	AST/ALT	-0.19148117	9.10E-08
B72	B206	HDL-C	Waist	-0.32780986	0
B72	B216	HDL-C	BMI	-0.34192246	0
B72	B222	HDL-C	NS	-0.33862857	0

B72	B74	HDL-C	ApoA1	0.370311791	0
B72	B77	HDL-C	TC	0.37901466	0
B72	B192	HDL-C	UA	-0.27106903	2.20E-14
B72	B215	HDL-C	BMI	-0.26851474	3.91E-14
B72	B221	HDL-C	Hei	-0.2580383	3.93E-13
B72	B92	HDL-C	ALDH2-Glu504Glu	0.253697794	9.92E-13
B72	B93	HDL-C	ALDH2-Glu504Lys	-0.25369779	9.92E-13
B72	B94	HDL-C	ALDH2-Lys504Lys	-0.25369779	9.92E-13
B72	B210	HDL-C	Hip	-0.24167173	1.18E-11
B72	B209	HDL-C	Smoking	-0.18955615	1.23E-07
B72	B163	HDL-C	SG	-0.1885427	1.44E-07
B72	B128	HDL-C	ALT	-0.18135433	4.27E-07
B72	B141	HDL-C	AST/ALT	0.180641879	4.75E-07
B73	B199	ApoA	Mg	0.259326793	2.98E-13
B73	B76	ApoA	ApoB	0.247317466	3.75E-12
B73	B74	ApoA	ApoA1	0.229985178	1.15E-10
B73	B90	ApoA	CYFRA 21-1	0.206090199	8.40E-09
B73	B88	ApoA	CA 242	0.181000756	4.50E-07
B74	B123	ApoA1	hs-CRP1	-0.43072709	0
B74	B132	ApoA1	Dbil	-0.36553098	0
B74	B165	ApoA1	bilirubin	-0.30147568	0
B74	B75	ApoA1	ApoA1/B	0.459245211	0
B74	B92	ApoA1	ALDH2-Glu504Glu	0.370511268	0
B74	B93	ApoA1	ALDH2-Glu504Lys	-0.37051127	0
B74	B94	ApoA1	ALDH2-Lys504Lys	-0.37051127	0
B74	B140	ApoA1	r-GT	-0.25445298	8.45E-13
B74	B131	ApoA1	AST	-0.24842291	2.98E-12
B74	B77	ApoA1	TC	0.230158945	1.11E-10
B74	B133	ApoA1	TBIL	-0.21086364	3.71E-09
B74	B126	ApoA1	Alb	0.191339507	9.30E-08
B74	B188	ApoA1	Ca	0.187653052	1.65E-07
B75	B115	ApoA1/B	TSH TT3 TT4(TT3)	0.337589066	0
B75	B76	ApoA1/B	ApoB	-0.67368507	0
B75	B79	ApoA1/B	hb V -dna	-0.28232143	1.55E-15
B75	B114	ApoA1/B	TSH TT3 TT4(TT4)	0.269998612	2.80E-14
B75	B194	ApoA1/B	Cl	0.263410166	1.22E-13
B75	B112	ApoA1/B	FT4	0.227636917	1.79E-10
B75	B125	ApoA1/B	Cor	0.222613812	4.56E-10
B75	B123	ApoA1/B	hs-CRP1	-0.22246438	4.69E-10
B75	B81	ApoA1/B	TSGF	0.221442027	5.65E-10
B75	B193	ApoA1/B	K	-0.22016569	7.13E-10
B75	B113	ApoA1/B	FT3	0.21791379	1.07E-09
B75	B138	ApoA1/B	PAB	-0.21286129	2.62E-09
B75	B179	ApoA1/B	α1-MG	-0.21175179	3.18E-09

B75	B211	ApoA1/B	IOP-R	-0.20337682	1.33E-08
B75	B92	ApoA1/B	ALDH2-Glu504Glu	0.197189135	3.66E-08
B75	B93	ApoA1/B	ALDH2-Glu504Lys	-0.19718914	3.66E-08
B75	B94	ApoA1/B	ALDH2-Lys504Lys	-0.19718913	3.66E-08
B75	B199	ApoA1/B	Mg	-0.18009334	5.15E-07
B76	B77	ApoB	TC	0.300966954	0
B76	B194	ApoB	Cl	-0.25268159	1.23E-12
B76	B138	ApoB	PAB	0.242222012	1.05E-11
B76	B115	ApoB	TSH TT3 TT4(TT3)	-0.21571632	1.58E-09
B76	B196	ApoB	TCO2	0.214237139	2.06E-09
B76	B212	ApoB	IOP-L	0.195312401	4.96E-08
B76	B114	ApoB	TSH TT3 TT4(TT4)	-0.19457567	5.57E-08
B76	B112	ApoB	FT4	-0.18448263	2.67E-07
B77	B189	TC	eGFB	-0.32715349	0
B77	B91	TC	TP	0.266581427	6.04E-14
B77	B112	TC	FT4	-0.22598048	2.45E-10
B77	B219	TC	OT	0.201647151	1.77E-08
B77	B126	TC	Alb	0.199562231	2.49E-08
B77	B132	TC	Dbil	-0.19398516	6.12E-08
B77	B190	TC	Cre	0.186497552	1.97E-07
B77	B113	TC	FT3	-0.18636171	2.01E-07
B78	B143	HBcAb	β-CTx	0.341589039	0
B78	B144	HBcAb	Total-PINP	0.353602701	0
B78	B145	HBcAb	BGP	0.382640924	0
B78	B83	HBcAb	AFP	0.226027971	2.42E-10
B78	B79	HBcAb	hbＶ-dna	0.217102793	1.24E-09
B79	B81	hbＶ-dna	TSGF	-0.26701442	5.48E-14
B79	B125	hbＶ-dna	Cor	-0.26055636	2.28E-13
B79	B156	hbＶ-dna	IgE	-0.24314368	8.76E-12
B8	B78	WB-Zinc	HBcAb	-0.37287466	0
B8	B156	WB-Zinc	IgE	0.269452115	3.18E-14
B8	B81	WB-Zinc	TSGF	0.2363008	3.41E-11
B8	B39	WB-Zinc	Other cells	0.195631161	4.71E-08
B8	B154	WB-Zinc	RF	0.177590414	7.43E-07
B81	B156	TSGF	IgE	0.314083323	0
B81	B125	TSGF	Cor	0.211265663	3.46E-09
B82	B84	CEA	C-PSA	0.417378722	0
B82	B85	CEA	SCC	0.285853664	6.66E-16
B82	B87	CEA	NSE	0.237482575	2.70E-11
B82	B89	CEA	CA 72-4	0.225910439	2.48E-10
B82	B88	CEA	CA 242	0.221611937	5.48E-10
B82	B86	CEA	TBhCG	0.213257356	2.44E-09
B83	B85	AFP	SCC	0.400347479	0
B83	B86	AFP	TBhCG	0.282371158	1.55E-15

B83	B89	AFP	CA 72-4	0.28111785	2.22E-15
B84	B85	C-PSA	SCC	0.467840608	0
B84	B86	C-PSA	TBhCG	0.67051875	0
B84	B87	C-PSA	NSE	0.444282084	0
B84	B89	C-PSA	CA 72-4	0.288266636	4.44E-16
B85	B86	SCC	TBhCG	0.42387002	0
B85	B87	SCC	NSE	0.299251518	0
B85	B89	SCC	CA 72-4	0.359136884	0
B85	B183	SCC	PTA	-0.18768255	1.64E-07
B86	B87	TBhCG	NSE	0.481942871	0
B86	B89	TBhCG	CA 72-4	0.430080469	0
B86	B196	TBhCG	TCO2	0.176797185	8.34E-07
B90	B120	CYFRA 21-1	HCV-cAg	-0.23305973	6.40E-11
B91	B126	TP	Alb	0.523184698	0
B91	B127	TP	A-G ratio	-0.49132356	0
B91	B130	TP	GLB	0.784033798	0
B91	B188	TP	Ca	0.239317977	1.88E-11
B91	B209	TP	Smoking	-0.17672909	8.42E-07
B92	B93	ALDH2-Glu504Glu	ALDH2-Glu504Lys	-1	0
B92	B94	ALDH2-Glu504Glu	ALDH2-Lys504Lys	-0.99999999	0
B92	B156	ALDH2-Glu504Glu	IgE	-0.19910174	2.68E-08
B93	B94	ALDH2-Glu504Lys	ALDH2-Lys504Lys	0.999999994	0
B93	B156	ALDH2-Glu504Lys	IgE	0.199101741	2.68E-08
B94	B156	ALDH2-Lys504Lys	IgE	0.19910174	2.68E-08
B95	B100	anti-cenpb	anti-Ro52	0.999999993	0
B95	B103	anti-cenpb	anti-SSA/Ro	-0.66109405	0
B95	B104	anti-cenpb	anti-SSB	0.999999993	0
B95	B105	anti-cenpb	anti-U1RNP	0.999999995	0
B95	B107	anti-cenpb	AnuA	0.999999767	0
B95	B108	anti-cenpb	Anti-Cyclin	0.999999997	0
B95	B109	anti-cenpb	AMA-M2	0.999999993	0
B95	B110	anti-cenpb	AHA	0.999999995	0
B95	B171	anti-cenpb	EC	-0.30105168	0
B95	B173	anti-cenpb	NTT	-0.29660015	0
B95	B97	anti-cenpb	dsDNA	0.999999997	0
B95	B179	anti-cenpb	α1-MG	0.285253141	8.88E-16
B95	B158	anti-cenpb	IgM	0.281658569	1.78E-15
B95	B139	anti-cenpb	ADA	-0.22290661	4.32E-10
B95	B199	anti-cenpb	Mg	0.207016523	7.18E-09
B95	B135	anti-cenpb	TBA	0.178387518	6.62E-07
B95	B193	anti-cenpb	K	-0.17780783	7.20E-07
B95	B157	anti-cenpb	IgG	-0.17568129	9.80E-07
B97	B100	dsDNA	anti-Ro52	0.999999993	0
B97	B102	dsDNA	anti-Sm	0.999999996	0

B97	B103	dsDNA	anti-SSA/Ro	-0.66109405	0
B97	B104	dsDNA	anti-SSB	0.999999997	0
B97	B105	dsDNA	anti-U1RNP	0.999999992	0
B97	B106	dsDNA	anti-P	0.999999995	0
B97	B107	dsDNA	AnuA	0.999999837	0
B97	B108	dsDNA	Anti-Cyclin	0.999999997	0
B97	B109	dsDNA	AMA-M2	0.99999999	0
B97	B110	dsDNA	AHA	0.999999995	0
B97	B171	dsDNA	EC	-0.30105167	0
B97	B173	dsDNA	NTT	-0.29660014	0
B97	B99	dsDNA	anti-PM-Scl	0.999999997	0
B97	B179	dsDNA	α1-MG	0.285253141	8.88E-16
B97	B158	dsDNA	IgM	0.281658569	1.78E-15
B97	B139	dsDNA	ADA	-0.22290661	4.32E-10
B97	B199	dsDNA	Mg	0.207016523	7.18E-09
B97	B135	dsDNA	TBA	0.178387517	6.62E-07
B97	B193	dsDNA	K	-0.17780783	7.20E-07
B97	B157	dsDNA	IgG	-0.17568129	9.80E-07
B98	B101	anti-Jo-1	anti-Sc70	0.999999965	0
B98	B102	anti-Jo-1	anti-Sm	0.999999992	0
B98	B103	anti-Jo-1	anti-SSA/Ro	-0.66109407	0
B98	B107	anti-Jo-1	AnuA	0.999999555	0
B98	B108	anti-Jo-1	Anti-Cyclin	0.999999998	0
B98	B171	anti-Jo-1	EC	-0.30105168	0
B98	B173	anti-Jo-1	NTT	-0.29660015	0
B98	B99	anti-Jo-1	anti-PM-Scl	0.999999999	0
B98	B179	anti-Jo-1	α1-MG	0.285253149	8.88E-16
B98	B158	anti-Jo-1	IgM	0.281658576	1.78E-15
B98	B139	anti-Jo-1	ADA	-0.22290662	4.32E-10
B98	B199	anti-Jo-1	Mg	0.207016526	7.18E-09
B98	B135	anti-Jo-1	TBA	0.178387522	6.62E-07
B98	B193	anti-Jo-1	K	-0.17780784	7.20E-07
B98	B157	anti-Jo-1	IgG	-0.17568129	9.80E-07
B99	B101	anti-PM-Scl	anti-Sc70	0.999999987	0
B99	B103	anti-PM-Scl	anti-SSA/Ro	-0.66109405	0
B99	B104	anti-PM-Scl	anti-SSB	0.999999997	0
B99	B106	anti-PM-Scl	anti-P	0.999999988	0
B99	B107	anti-PM-Scl	AnuA	0.999999707	0
B99	B109	anti-PM-Scl	AMA-M2	0.999999999	0
B99	B171	anti-PM-Scl	EC	-0.30105168	0
B99	B173	anti-PM-Scl	NTT	-0.29660015	0
B99	B179	anti-PM-Scl	α1-MG	0.285253144	8.88E-16
B99	B158	anti-PM-Scl	IgM	0.281658572	1.78E-15
B99	B139	anti-PM-Scl	ADA	-0.22290662	4.32E-10

B99	B199	anti-PM-Scl	Mg	0.207016524	7.18E-09
B99	B135	anti-PM-Scl	TBA	0.17838752	6.62E-07
B99	B193	anti-PM-Scl	K	-0.17780784	7.20E-07
B99	B157	anti-PM-Scl	IgG	-0.17568129	9.80E-07

Supplementary Table 13. PEI Correlations in Cervical spondylopathy

Row	Column	Row	Column	Cor	*P*
B1	B190	Gender	Cre	-0.66340451	0
B1	B192	Gender	UA	-0.60573664	0
B1	B216	Gender	BMI	-0.52700024	0
B1	B221	Gender	Hei	-0.5609765	0
B1	B222	Gender	NS	-0.6893586	0
B1	B46	Gender	RBC Count	-0.45117102	0
B1	B51	Gender	Hb	-0.58557155	0
B1	B206	Gender	Waist	-0.43154684	8.88E-16
B1	B163	Gender	SG	-0.40707091	4.02E-14
B1	B209	Gender	Smoking	-0.38209899	1.71E-12
B1	B174	Gender	Urea/Cr	0.358302493	4.57E-11
B1	B72	Gender	HDL-C	0.335272777	8.59E-10
B1	B204	Gender	Drinking	-0.3245909	3.09E-09
B1	B128	Gender	ALT	-0.30172694	4.07E-08
B1	B215	Gender	BMI	-0.28635324	2.04E-07
B1	B45	Gender	MCHC	-0.28570787	2.18E-07
B1	B218	Gender	DBP	-0.28486881	2.37E-07
B1	B210	Gender	Hip	-0.27962808	4.01E-07
B1	B207	Gender	WHR	-0.27884504	4.34E-07
B10	B12	PG I	PG II	0.619206966	0
B10	B14	PG I	G-17	0.463184577	0
B10	B55	PG I	TK	0.289058078	1.55E-07
B10	B61	PG I	WBHSRV	0.275319006	6.13E-07
B100	B103	anti-Ro52	anti-SSA/Ro	1	0
B100	B105	anti-Ro52	anti-U1RNP	1	0
B100	B106	anti-Ro52	anti-P	0.999999999	0
B100	B107	anti-Ro52	AnuA	0.999999986	0
B100	B151	anti-Ro52	Anti-CCP	0.665704468	0
B100	B156	anti-Ro52	IgE	0.614367186	0
B100	B178	anti-Ro52	Ig-U	0.524633851	0
B100	B180	anti-Ro52	Tf	0.531153079	0
B100	B184	anti-Ro52	INR	0.452826162	0
B100	B193	anti-Ro52	K	0.459514228	0
B100	B154	anti-Ro52	RF	0.419728548	5.33E-15
B100	B199	anti-Ro52	Mg	0.402787859	7.82E-14
B100	B179	anti-Ro52	α1-MG	0.397881945	1.66E-13
B100	B183	anti-Ro52	PTA	0.375742668	4.22E-12
B100	B157	anti-Ro52	IgG	0.340998994	4.24E-10
B100	B158	anti-Ro52	IgM	-0.28134467	3.38E-07
B100	B119	anti-Ro52	Hcv-rna	0.281326061	3.39E-07
B100	B121	anti-Ro52	C3	-0.28132607	3.39E-07
B100	B122	anti-Ro52	C4	-0.28132616	3.39E-07

B100	B152	anti-Ro52	ACA-Ig(G-A-M)	-0.2813268	3.39E-07
B100	B159	anti-Ro52	β2-MG	-0.28132641	3.39E-07
B100	B169	anti-Ro52	U-CRY	-0.28132577	3.39E-07
B101	B102	anti-Sc70	anti-Sm	0.999999998	0
B101	B106	anti-Sc70	anti-P	0.999999999	0
B101	B107	anti-Sc70	AnuA	0.999999968	0
B101	B108	anti-Sc70	Anti-Cyclin	1	0
B101	B151	anti-Sc70	Anti-CCP	0.665704468	0
B101	B156	anti-Sc70	IgE	0.614367187	0
B101	B178	anti-Sc70	Ig-U	0.524633852	0
B101	B180	anti-Sc70	Tf	0.53115308	0
B101	B184	anti-Sc70	INR	0.452826163	0
B101	B193	anti-Sc70	K	0.459514229	0
B101	B154	anti-Sc70	RF	0.419728548	5.33E-15
B101	B199	anti-Sc70	Mg	0.40278786	7.82E-14
B101	B179	anti-Sc70	α1-MG	0.397881946	1.66E-13
B101	B183	anti-Sc70	PTA	0.375742668	4.22E-12
B101	B157	anti-Sc70	IgG	0.340998994	4.24E-10
B101	B158	anti-Sc70	IgM	-0.28134467	3.38E-07
B101	B119	anti-Sc70	Hcv-rna	0.281326061	3.39E-07
B101	B121	anti-Sc70	C3	-0.28132608	3.39E-07
B101	B122	anti-Sc70	C4	-0.28132624	3.39E-07
B101	B152	anti-Sc70	ACA-Ig(G-A-M)	-0.28132699	3.39E-07
B101	B159	anti-Sc70	β2-MG	-0.28132642	3.39E-07
B101	B169	anti-Sc70	U-CRY	-0.28132573	3.39E-07
B102	B104	anti-Sm	anti-SSB	0.999999999	0
B102	B106	anti-Sm	anti-P	1	0
B102	B107	anti-Sm	AnuA	0.999999998	0
B102	B108	anti-Sm	Anti-Cyclin	0.999999999	0
B102	B151	anti-Sm	Anti-CCP	0.665704468	0
B102	B156	anti-Sm	IgE	0.614367187	0
B102	B178	anti-Sm	Ig-U	0.524633852	0
B102	B180	anti-Sm	Tf	0.531153079	0
B102	B184	anti-Sm	INR	0.452826163	0
B102	B193	anti-Sm	K	0.459514229	0
B102	B154	anti-Sm	RF	0.419728548	5.33E-15
B102	B199	anti-Sm	Mg	0.40278786	7.82E-14
B102	B179	anti-Sm	α1-MG	0.397881945	1.66E-13
B102	B183	anti-Sm	PTA	0.375742668	4.22E-12
B102	B157	anti-Sm	IgG	0.340998994	4.24E-10
B102	B158	anti-Sm	IgM	-0.28134467	3.38E-07
B102	B119	anti-Sm	Hcv-rna	0.281326061	3.39E-07
B102	B121	anti-Sm	C3	-0.28132608	3.39E-07
B102	B122	anti-Sm	C4	-0.28132619	3.39E-07

B102	B152	anti-Sm	ACA-Ig(G-A-M)	-0.28132698	3.39E-07
B102	B159	anti-Sm	β2-MG	-0.28132649	3.39E-07
B102	B169	anti-Sm	U-CRY	-0.28132578	3.39E-07
B103	B105	anti-SSA/Ro	anti-U1RNP	1	0
B103	B106	anti-SSA/Ro	anti-P	1	0
B103	B107	anti-SSA/Ro	AnuA	0.999999991	0
B103	B151	anti-SSA/Ro	Anti-CCP	0.665704467	0
B103	B156	anti-SSA/Ro	IgE	0.614367186	0
B103	B178	anti-SSA/Ro	Ig-U	0.524633851	0
B103	B180	anti-SSA/Ro	Tf	0.531153079	0
B103	B184	anti-SSA/Ro	INR	0.452826162	0
B103	B193	anti-SSA/Ro	K	0.459514228	0
B103	B154	anti-SSA/Ro	RF	0.419728548	5.33E-15
B103	B199	anti-SSA/Ro	Mg	0.402787859	7.82E-14
B103	B179	anti-SSA/Ro	α1-MG	0.397881945	1.66E-13
B103	B183	anti-SSA/Ro	PTA	0.375742668	4.22E-12
B103	B157	anti-SSA/Ro	IgG	0.340998994	4.24E-10
B103	B158	anti-SSA/Ro	IgM	-0.28134467	3.38E-07
B103	B119	anti-SSA/Ro	Hcv-rna	0.281326061	3.39E-07
B103	B121	anti-SSA/Ro	C3	-0.28132607	3.39E-07
B103	B122	anti-SSA/Ro	C4	-0.28132616	3.39E-07
B103	B152	anti-SSA/Ro	ACA-Ig(G-A-M)	-0.28132677	3.39E-07
B103	B159	anti-SSA/Ro	β2-MG	-0.28132639	3.39E-07
B103	B169	anti-SSA/Ro	U-CRY	-0.28132575	3.39E-07
B104	B107	anti-SSB	AnuA	0.999999989	0
B104	B151	anti-SSB	Anti-CCP	0.665704468	0
B104	B156	anti-SSB	IgE	0.614367186	0
B104	B178	anti-SSB	Ig-U	0.524633852	0
B104	B180	anti-SSB	Tf	0.531153079	0
B104	B184	anti-SSB	INR	0.452826162	0
B104	B193	anti-SSB	K	0.459514229	0
B104	B154	anti-SSB	RF	0.419728548	5.33E-15
B104	B199	anti-SSB	Mg	0.40278786	7.82E-14
B104	B179	anti-SSB	α1-MG	0.397881945	1.66E-13
B104	B183	anti-SSB	PTA	0.375742668	4.22E-12
B104	B157	anti-SSB	IgG	0.340998994	4.24E-10
B104	B158	anti-SSB	IgM	-0.28134467	3.38E-07
B104	B119	anti-SSB	Hcv-rna	0.281326061	3.39E-07
B104	B121	anti-SSB	C3	-0.28132606	3.39E-07
B104	B122	anti-SSB	C4	-0.28132617	3.39E-07
B104	B152	anti-SSB	ACA-Ig(G-A-M)	-0.28132684	3.39E-07
B104	B159	anti-SSB	β2-MG	-0.28132639	3.39E-07
B104	B169	anti-SSB	U-CRY	-0.28132574	3.39E-07
B105	B106	anti-U1RNP	anti-P	0.999999999	0

B105	B107	anti-U1RNP	AnuA	0.99999999	0
B105	B109	anti-U1RNP	AMA-M2	1	0
B105	B110	anti-U1RNP	AHA	1	0
B105	B151	anti-U1RNP	Anti-CCP	0.665704467	0
B105	B156	anti-U1RNP	IgE	0.614367186	0
B105	B178	anti-U1RNP	Ig-U	0.524633851	0
B105	B180	anti-U1RNP	Tf	0.531153079	0
B105	B184	anti-U1RNP	INR	0.452826163	0
B105	B193	anti-U1RNP	K	0.459514228	0
B105	B154	anti-U1RNP	RF	0.419728548	5.33E-15
B105	B199	anti-U1RNP	Mg	0.402787859	7.82E-14
B105	B179	anti-U1RNP	α1-MG	0.397881945	1.66E-13
B105	B183	anti-U1RNP	PTA	0.375742668	4.22E-12
B105	B157	anti-U1RNP	IgG	0.340998994	4.24E-10
B105	B158	anti-U1RNP	IgM	-0.28134467	3.38E-07
B105	B119	anti-U1RNP	Hcv-rna	0.281326061	3.39E-07
B105	B121	anti-U1RNP	C3	-0.28132607	3.39E-07
B105	B122	anti-U1RNP	C4	-0.28132617	3.39E-07
B105	B152	anti-U1RNP	ACA-Ig(G-A-M)	-0.28132675	3.39E-07
B105	B159	anti-U1RNP	β2-MG	-0.2813264	3.39E-07
B105	B169	anti-U1RNP	U-CRY	-0.28132577	3.39E-07
B106	B107	anti-P	AnuA	0.99999998	0
B106	B108	anti-P	Anti-Cyclin	1	0
B106	B110	anti-P	AHA	0.999999999	0
B106	B151	anti-P	Anti-CCP	0.665704467	0
B106	B156	anti-P	IgE	0.614367186	0
B106	B178	anti-P	Ig-U	0.524633851	0
B106	B180	anti-P	Tf	0.531153079	0
B106	B184	anti-P	INR	0.452826162	0
B106	B193	anti-P	K	0.459514229	0
B106	B154	anti-P	RF	0.419728548	5.33E-15
B106	B199	anti-P	Mg	0.402787859	7.82E-14
B106	B179	anti-P	α1-MG	0.397881945	1.66E-13
B106	B183	anti-P	PTA	0.375742668	4.22E-12
B106	B157	anti-P	IgG	0.340998994	4.24E-10
B106	B158	anti-P	IgM	-0.28134467	3.38E-07
B106	B119	anti-P	Hcv-rna	0.281326061	3.39E-07
B106	B121	anti-P	C3	-0.28132609	3.39E-07
B106	B122	anti-P	C4	-0.28132622	3.39E-07
B106	B152	anti-P	ACA-Ig(G-A-M)	-0.28132684	3.39E-07
B106	B159	anti-P	β2-MG	-0.2813264	3.39E-07
B106	B169	anti-P	U-CRY	-0.28132571	3.39E-07
B107	B108	AnuA	Anti-Cyclin	0.999999987	0
B107	B109	AnuA	AMA-M2	0.999999986	0

B107	B110	AnuA	AHA	0.999999991	0
B107	B151	AnuA	Anti-CCP	0.66570446	0
B107	B156	AnuA	IgE	0.614367179	0
B107	B178	AnuA	Ig-U	0.524633845	0
B107	B180	AnuA	Tf	0.531153072	0
B107	B184	AnuA	INR	0.452826157	0
B107	B193	AnuA	K	0.459514223	0
B107	B154	AnuA	RF	0.419728543	5.33E-15
B107	B199	AnuA	Mg	0.402787855	7.82E-14
B107	B179	AnuA	α1-MG	0.39788194	1.66E-13
B107	B183	AnuA	PTA	0.375742664	4.22E-12
B107	B157	AnuA	IgG	0.34099899	4.24E-10
B107	B158	AnuA	IgM	-0.28134466	3.38E-07
B107	B119	AnuA	Hcv-rna	0.281326058	3.39E-07
B107	B121	AnuA	C3	-0.28132601	3.39E-07
B107	B122	AnuA	C4	-0.28132645	3.39E-07
B107	B152	AnuA	ACA-Ig(G-A-M)	-0.28132692	3.39E-07
B107	B159	AnuA	β2-MG	-0.28132645	3.39E-07
B107	B169	AnuA	U-CRY	-0.28132579	3.39E-07
B108	B110	Anti-Cyclin	AHA	1	0
B108	B151	Anti-Cyclin	Anti-CCP	0.665704468	0
B108	B156	Anti-Cyclin	IgE	0.614367186	0
B108	B178	Anti-Cyclin	Ig-U	0.524633851	0
B108	B180	Anti-Cyclin	Tf	0.531153079	0
B108	B184	Anti-Cyclin	INR	0.452826163	0
B108	B193	Anti-Cyclin	K	0.459514229	0
B108	B154	Anti-Cyclin	RF	0.419728548	5.33E-15
B108	B199	Anti-Cyclin	Mg	0.402787859	7.82E-14
B108	B179	Anti-Cyclin	α1-MG	0.397881945	1.66E-13
B108	B183	Anti-Cyclin	PTA	0.375742668	4.22E-12
B108	B157	Anti-Cyclin	IgG	0.340998994	4.24E-10
B108	B158	Anti-Cyclin	IgM	-0.28134467	3.38E-07
B108	B119	Anti-Cyclin	Hcv-rna	0.281326061	3.39E-07
B108	B121	Anti-Cyclin	C3	-0.28132607	3.39E-07
B108	B122	Anti-Cyclin	C4	-0.28132616	3.39E-07
B108	B152	Anti-Cyclin	ACA-Ig(G-A-M)	-0.28132677	3.39E-07
B108	B159	Anti-Cyclin	β2-MG	-0.28132642	3.39E-07
B108	B169	Anti-Cyclin	U-CRY	-0.28132576	3.39E-07
B109	B151	AMA-M2	Anti-CCP	0.665704468	0
B109	B156	AMA-M2	IgE	0.614367186	0
B109	B178	AMA-M2	Ig-U	0.524633851	0
B109	B180	AMA-M2	Tf	0.531153079	0
B109	B184	AMA-M2	INR	0.452826162	0
B109	B193	AMA-M2	K	0.459514229	0

B109	B154	AMA-M2	RF	0.419728548	5.33E-15
B109	B199	AMA-M2	Mg	0.402787859	7.82E-14
B109	B179	AMA-M2	α1-MG	0.397881945	1.66E-13
B109	B183	AMA-M2	PTA	0.375742668	4.22E-12
B109	B157	AMA-M2	IgG	0.340998994	4.24E-10
B109	B158	AMA-M2	IgM	-0.28134467	3.38E-07
B109	B119	AMA-M2	Hcv-rna	0.281326061	3.39E-07
B109	B121	AMA-M2	C3	-0.28132607	3.39E-07
B109	B122	AMA-M2	C4	-0.28132617	3.39E-07
B109	B152	AMA-M2	ACA-Ig(G-A-M)	-0.28132684	3.39E-07
B109	B159	AMA-M2	β2-MG	-0.28132642	3.39E-07
B109	B169	AMA-M2	U-CRY	-0.28132578	3.39E-07
B11	B12	PGI/PGII	PG II	-0.49176828	0
B110	B151	AHA	Anti-CCP	0.665704467	0
B110	B156	AHA	IgE	0.614367186	0
B110	B178	AHA	Ig-U	0.524633851	0
B110	B180	AHA	Tf	0.531153079	0
B110	B184	AHA	INR	0.452826162	0
B110	B193	AHA	K	0.459514228	0
B110	B154	AHA	RF	0.419728548	5.33E-15
B110	B199	AHA	Mg	0.402787859	7.82E-14
B110	B179	AHA	α1-MG	0.397881945	1.66E-13
B110	B183	AHA	PTA	0.375742668	4.22E-12
B110	B157	AHA	IgG	0.340998994	4.24E-10
B110	B158	AHA	IgM	-0.28134467	3.38E-07
B110	B119	AHA	Hcv-rna	0.281326061	3.39E-07
B110	B121	AHA	C3	-0.28132607	3.39E-07
B110	B122	AHA	C4	-0.28132617	3.39E-07
B110	B152	AHA	ACA-Ig(G-A-M)	-0.28132685	3.39E-07
B110	B159	AHA	β2-MG	-0.28132641	3.39E-07
B110	B169	AHA	U-CRY	-0.28132575	3.39E-07
B113	B115	FT3	TSH TT3 TT4(TT3)	0.449146784	0
B114	B115	TSH TT3 TT4(TT4)	TSH TT3 TT4(TT3)	0.578239605	0
B114	B195	TSH TT3 TT4(TT4)	Na	0.545521849	0
B114	B135	TSH TT3 TT4(TT4)	TBA	0.373579392	5.72E-12
B114	B197	TSH TT3 TT4(TT4)	AG	0.370917378	8.28E-12
B114	B200	TSH TT3 TT4(TT4)	OP	0.354853258	7.21E-11
B114	B187	TSH TT3 TT4(TT4)	NSE	0.310615769	1.53E-08
B115	B195	TSH TT3 TT4(TT3)	Na	0.404865188	5.66E-14
B115	B187	TSH TT3 TT4(TT3)	NSE	0.300315282	4.74E-08
B115	B119	TSH TT3 TT4(TT3)	Hcv-rna	0.274628703	6.56E-07
B115	B121	TSH TT3 TT4(TT3)	C3	-0.27462871	6.56E-07
B115	B122	TSH TT3 TT4(TT3)	C4	-0.2746288	6.56E-07
B115	B152	TSH TT3 TT4(TT3)	ACA-Ig(G-A-M)	-0.27462943	6.56E-07

B115	B159	TSH TT3 TT4(TT3)	β2-MG	-0.27462903	6.56E-07
B115	B169	TSH TT3 TT4(TT3)	U-CRY	-0.27462841	6.56E-07
B117	B167	TT4	PC	0.955636965	0
B119	B148	Hcv-rna	Anti_TPO	-0.45472746	0
B119	B169	Hcv-rna	U-CRY	-0.99999893	0
B119	B120	Hcv-rna	HCV-cAg	0.357558491	5.05E-11
B12	B14	PG II	G-17	0.411104796	2.13E-14
B120	B121	HCV-cAg	C3	-0.35755848	5.05E-11
B120	B122	HCV-cAg	C4	-0.35755871	5.05E-11
B120	B152	HCV-cAg	ACA-Ig(G-A-M)	-0.35755786	5.05E-11
B120	B159	HCV-cAg	β2-MG	-0.3575586	5.05E-11
B120	B169	HCV-cAg	U-CRY	-0.35755781	5.05E-11
B120	B135	HCV-cAg	TBA	-0.31644216	7.94E-09
B121	B148	C3	Anti_TPO	0.454727474	0
B121	B169	C3	U-CRY	0.999998943	0
B122	B148	C4	Anti_TPO	0.454727616	0
B122	B169	C4	U-CRY	0.999999453	0
B123	B186	hs-CRP1	PFC	0.448626831	0
B123	B177	hs-CRP1	ACR	0.405253439	5.33E-14
B123	B194	hs-CRP1	Cl	-0.36515016	1.83E-11
B123	B181	hs-CRP1	APTT	-0.34261594	3.46E-10
B123	B199	hs-CRP1	Mg	-0.31923125	5.77E-09
B124	B181	ACTH	APTT	-0.27280423	7.83E-07
B125	B187	Cor	NSE	0.698307366	0
B125	B198	Cor	P	0.474811123	0
B125	B199	Cor	Mg	-0.34906423	1.53E-10
B125	B193	Cor	K	-0.29978458	5.02E-08
B125	B205	Cor	KDA	0.295118666	8.24E-08
B126	B127	Alb	A-G ratio	0.442527588	0
B126	B188	Alb	Ca	0.397133176	1.86E-13
B127	B130	A-G ratio	GLB	-0.89766643	0
B128	B131	ALT	AST	0.664598327	0
B128	B140	ALT	r-GT	0.462344814	0
B128	B141	ALT	AST/ALT	-0.56501874	0
B128	B192	ALT	UA	0.384959383	1.13E-12
B128	B222	ALT	NS	0.35357227	8.52E-11
B128	B216	ALT	BMI	0.341640439	3.91E-10
B128	B163	ALT	SG	0.275409353	6.08E-07
B129	B132	IBIL	Dbil	0.651186167	0
B129	B133	IBIL	TBIL	0.978419962	0
B131	B140	AST	r-GT	0.447765374	0
B131	B192	AST	UA	0.3086149	1.92E-08
B132	B133	Dbil	TBIL	0.79386761	0
B132	B135	Dbil	TBA	0.282973	2.87E-07

B134	B135	CHE	TBA	-0.41380577	1.38E-14
B134	B137	CHE	AFU	0.383388064	1.42E-12
B135	B197	TBA	AG	0.559320366	0
B135	B200	TBA	OP	0.515839739	0
B135	B198	TBA	P	-0.43932369	2.22E-16
B135	B193	TBA	K	-0.38830528	6.92E-13
B135	B195	TBA	Na	0.355944846	6.24E-11
B135	B196	TBA	TCO2	-0.32056282	4.94E-09
B135	B146	TBA	CysC	0.314629861	9.76E-09
B135	B194	TBA	Cl	-0.31140224	1.40E-08
B136	B150	5'NT	ALP	0.601534733	0
B136	B153	5'NT	Glu	0.47586359	0
B136	B140	5'NT	r-GT	0.339940577	4.83E-10
B137	B138	AFU	PAB	0.365113135	1.83E-11
B137	B197	AFU	AG	0.327625183	2.16E-09
B137	B199	AFU	Mg	-0.29147372	1.21E-07
B137	B195	AFU	Na	0.287105454	1.89E-07
B138	B200	PAB	OP	0.385728684	1.01E-12
B138	B198	PAB	P	-0.32840378	1.97E-09
B138	B176	PAB	Ucr	0.282787253	2.93E-07
B139	B200	ADA	OP	0.393560818	3.18E-13
B139	B150	ADA	ALP	0.286990572	1.91E-07
B139	B197	ADA	AG	0.281263565	3.41E-07
B14	B61	G-17	WBHSRV	0.437839677	2.22E-16
B14	B55	G-17	TK	0.424371674	2.44E-15
B14	B56	G-17	IR	0.421106508	4.22E-15
B14	B59	G-17	WBRV	0.35101503	1.19E-10
B14	B93	G-17	ALDH2-Glu504Lys	0.349638306	1.42E-10
B14	B92	G-17	ALDH2-Glu504Glu	-0.3328717	1.15E-09
B14	B62	G-17	WBHSRVI	0.307975416	2.06E-08
B14	B66	G-17	WBRSR(mPas_100)	0.30545146	2.71E-08
B14	B63	G-17	WBRSR(mPas_200)	0.304947563	2.87E-08
B14	B185	G-17	PT	-0.30015817	4.82E-08
B14	B67	G-17	WBRSR(mPas_50)	0.296447487	7.16E-08
B14	B22	G-17	CD3+CD8+(T)	-0.27937151	4.12E-07
B14	B19	G-17	CD3+CD4+/CD3+CD8+	0.272123793	8.36E-07
B14	B64	G-17	WBRSR(mPas_5)	0.270725485	9.56E-07
B140	B192	r-GT	UA	0.303854592	3.23E-08
B140	B141	r-GT	AST/ALT	-0.29477788	8.54E-08
B140	B150	r-GT	ALP	0.294217624	9.05E-08
B140	B153	r-GT	Glu	0.292676552	1.06E-07
B140	B222	r-GT	NS	0.286448991	2.02E-07
B141	B216	AST/ALT	BMI	-0.38337395	1.42E-12
B141	B221	AST/ALT	Hei	-0.34669278	2.07E-10

B141	B192	AST/ALT	UA	-0.31191796	1.33E-08
B141	B222	AST/ALT	NS	-0.31147964	1.39E-08
B141	B210	AST/ALT	Hip	-0.31108149	1.46E-08
B141	B206	AST/ALT	Waist	-0.2737038	7.17E-07
B142	B144	25-(OH)D	Total-PINP	-0.74438861	0
B142	B151	25-(OH)D	Anti-CCP	-0.5231091	0
B142	B168	25-(OH)D	NMP22	0.913089867	0
B142	B154	25-(OH)D	RF	-0.27263202	7.96E-07
B143	B145	β-CTx	BGP	0.724602378	0
B143	B144	β-CTx	Total-PINP	0.4290153	1.11E-15
B144	B145	Total-PINP	BGP	0.607179761	0
B144	B168	Total-PINP	NMP22	-0.75829061	0
B144	B151	Total-PINP	Anti-CCP	0.389128721	6.13E-13
B148	B152	Anti_TPO	ACA-Ig(G-A-M)	0.454728667	0
B148	B159	Anti_TPO	β2-MG	0.454728001	0
B148	B169	Anti_TPO	U-CRY	0.454726971	0
B15	B100	CD3-CD19+(B cell)	anti-Ro52	0.487717811	0
B15	B101	CD3-CD19+(B cell)	anti-Sc70	0.487717811	0
B15	B102	CD3-CD19+(B cell)	anti-Sm	0.487717811	0
B15	B103	CD3-CD19+(B cell)	anti-SSA/Ro	0.487717811	0
B15	B104	CD3-CD19+(B cell)	anti-SSB	0.487717811	0
B15	B105	CD3-CD19+(B cell)	anti-U1RNP	0.48771781	0
B15	B106	CD3-CD19+(B cell)	anti-P	0.487717811	0
B15	B107	CD3-CD19+(B cell)	AnuA	0.487717805	0
B15	B108	CD3-CD19+(B cell)	Anti-Cyclin	0.487717811	0
B15	B109	CD3-CD19+(B cell)	AMA-M2	0.487717811	0
B15	B110	CD3-CD19+(B cell)	AHA	0.487717811	0
B15	B135	CD3-CD19+(B cell)	TBA	-0.69234486	0
B15	B151	CD3-CD19+(B cell)	Anti-CCP	0.49437704	0
B15	B156	CD3-CD19+(B cell)	IgE	0.452397262	0
B15	B16	CD3-CD19+(B cell)	CD3+T	-0.6910061	0
B15	B178	CD3-CD19+(B cell)	Ig-U	0.485411482	0
B15	B180	CD3-CD19+(B cell)	Tf	0.441073948	0
B15	B193	CD3-CD19+(B cell)	K	0.507089668	0
B15	B195	CD3-CD19+(B cell)	Na	-0.46655867	0
B15	B197	CD3-CD19+(B cell)	AG	-0.55984251	0
B15	B20	CD3-CD19+(B cell)	CD3+CD4+CD8+	0.455674377	0
B15	B200	CD3-CD19+(B cell)	OP	-0.52288446	0
B15	B22	CD3-CD19+(B cell)	CD3+CD8+(T)	-0.58622157	0
B15	B57	CD3-CD19+(B cell)	RBCAI	0.524412223	0
B15	B94	CD3-CD19+(B cell)	ALDH2-Lys504Lys	0.466351143	0
B15	B95	CD3-CD19+(B cell)	anti-cenpb	0.487717811	0
B15	B97	CD3-CD19+(B cell)	dsDNA	0.48771781	0
B15	B98	CD3-CD19+(B cell)	anti-Jo-1	0.48771781	0

B15	B99	CD3-CD19+(B cell)	anti-PM-Scl	0.48771781	0
B15	B199	CD3-CD19+(B cell)	Mg	0.422765115	3.11E-15
B15	B179	CD3-CD19+(B cell)	α1-MG	0.41387299	1.38E-14
B15	B23	CD3-CD19+(B cell)	CD3-CD16+CD56+(NK)	0.409203162	2.89E-14
B15	B79	CD3-CD19+(B cell)	hb V -dna	0.394760849	2.65E-13
B15	B194	CD3-CD19+(B cell)	Cl	0.386462279	9.07E-13
B15	B114	CD3-CD19+(B cell)	TSH TT3 TT4(TT4)	-0.38624108	9.37E-13
B15	B55	CD3-CD19+(B cell)	TK	-0.34066035	4.42E-10
B15	B90	CD3-CD19+(B cell)	CYFRA 21-1	-0.33801211	6.13E-10
B15	B154	CD3-CD19+(B cell)	RF	0.324653455	3.07E-09
B15	B184	CD3-CD19+(B cell)	INR	0.316267256	8.10E-09
B15	B56	CD3-CD19+(B cell)	IR	-0.31003622	1.64E-08
B15	B198	CD3-CD19+(B cell)	P	0.30964857	1.71E-08
B15	B17	CD3-CD19+(B cell)	CD3+CD16+CD56+NK	0.308075605	2.03E-08
B15	B19	CD3-CD19+(B cell)	CD3+CD4+/CD3+CD8+	0.300363731	4.71E-08
B15	B83	CD3-CD19+(B cell)	AFP	-0.28677498	1.96E-07
B15	B183	CD3-CD19+(B cell)	PTA	0.279638139	4.01E-07
B150	B153	ALP	Glu	0.359739508	3.78E-11
B150	B200	ALP	OP	0.298955366	5.48E-08
B151	B154	Anti-CCP	RF	0.50928063	0
B151	B156	Anti-CCP	IgE	0.622239933	0
B151	B178	Anti-CCP	Ig-U	0.460682227	0
B151	B184	Anti-CCP	INR	0.46257367	0
B151	B180	Anti-CCP	Tf	0.420935839	4.44E-15
B151	B168	Anti-CCP	NMP22	-0.3972012	1.84E-13
B151	B193	Anti-CCP	K	0.380584508	2.12E-12
B151	B183	Anti-CCP	PTA	0.378651096	2.80E-12
B151	B157	Anti-CCP	IgG	0.358453581	4.48E-11
B151	B179	Anti-CCP	α1-MG	0.336905115	7.03E-10
B151	B199	Anti-CCP	Mg	0.325633257	2.73E-09
B151	B158	Anti-CCP	IgM	-0.29343417	9.83E-08
B153	B170	Glu	U-GLU	0.444498444	0
B153	B220	Glu	SBP	0.314211484	1.02E-08
B153	B215	Glu	BMI	0.298798194	5.57E-08
B153	B206	Glu	Waist	0.289727228	1.44E-07
B154	B156	RF	IgE	0.608316769	0
B154	B182	RF	TT	-0.37603067	4.05E-12
B154	B184	RF	INR	0.37338812	5.87E-12
B154	B178	RF	Ig-U	0.339237894	5.27E-10
B154	B180	RF	Tf	0.333644662	1.05E-09
B154	B199	RF	Mg	0.285067747	2.33E-07
B154	B183	RF	PTA	0.279655876	4.00E-07
B154	B179	RF	α1-MG	0.275954542	5.76E-07
B155	B182	IgA	TT	0.584830956	0

B155	B157	IgA	IgG	0.415779691	1.02E-14
B155	B158	IgA	IgM	-0.35239011	9.94E-11
B155	B178	IgA	Ig-U	0.306070311	2.54E-08
B155	B180	IgA	Tf	0.28150289	3.33E-07
B156	B178	IgE	Ig-U	0.453416948	0
B156	B182	IgE	TT	-0.50835567	0
B156	B183	IgE	PTA	0.451718941	0
B156	B184	IgE	INR	0.664103269	0
B156	B180	IgE	Tf	0.414679398	1.20E-14
B156	B185	IgE	PT	0.373203868	6.02E-12
B156	B179	IgE	α1-MG	0.318785879	6.07E-09
B156	B193	IgE	K	0.318606915	6.20E-09
B157	B158	IgG	IgM	-0.5827547	0
B157	B178	IgG	Ig-U	0.381510969	1.86E-12
B157	B184	IgG	INR	0.375728659	4.23E-12
B157	B180	IgG	Tf	0.349876956	1.38E-10
B157	B179	IgG	α1-MG	0.34099007	4.24E-10
B157	B181	IgG	APTT	-0.28118696	3.44E-07
B158	B182	IgM	TT	-0.34987854	1.37E-10
B158	B179	IgM	α1-MG	-0.31302641	1.17E-08
B158	B178	IgM	Ig-U	-0.27745067	4.98E-07
B159	B169	β2-MG	U-CRY	0.999997962	0
B16	B125	CD3+T	Cor	0.529680402	0
B16	B193	CD3+T	K	-0.44076975	0
B16	B194	CD3+T	Cl	-0.45596137	0
B16	B199	CD3+T	Mg	-0.59099587	0
B16	B23	CD3+T	CD3-CD16+CD56+(NK)	-0.94028915	0
B16	B34	CD3+T	TK	-0.4869878	0
B16	B57	CD3+T	RBCAI	-0.43841022	2.22E-16
B16	B22	CD3+T	CD3+CD8+(T)	0.433309079	4.44E-16
B16	B90	CD3+T	CYFRA 21-1	0.424036526	2.66E-15
B16	B17	CD3+T	CD3+CD16+CD56+NK	-0.42247717	3.33E-15
B16	B197	CD3+T	AG	0.41368817	1.42E-14
B16	B179	CD3+T	α1-MG	-0.40382079	6.66E-14
B16	B83	CD3+T	AFP	0.393170664	3.37E-13
B16	B137	CD3+T	AFU	0.375717207	4.23E-12
B16	B181	CD3+T	APTT	-0.34946051	1.45E-10
B16	B195	CD3+T	Na	0.345751944	2.33E-10
B16	B178	CD3+T	Ig-U	-0.3411854	4.14E-10
B16	B19	CD3+T	CD3+CD4+/CD3+CD8+	-0.33639561	7.48E-10
B16	B187	CD3+T	NSE	0.318768392	6.08E-09
B16	B200	CD3+T	OP	0.317753517	6.84E-09
B16	B114	CD3+T	TSH TT3 TT4(TT4)	0.315217087	9.13E-09
B16	B186	CD3+T	PFC	0.310625606	1.53E-08

B16	B180	CD3+T	Tf	-0.30805621	2.04E-08
B16	B151	CD3+T	Anti-CCP	-0.28802629	1.72E-07
B16	B135	CD3+T	TBA	0.279041904	4.25E-07
B16	B60	CD3+T	WBRVI	-0.2790053	4.27E-07
B16	B154	CD3+T	RF	-0.27239318	8.14E-07
B161	B173	LEU	NTT	0.370335845	8.97E-12
B163	B222	SG	NS	0.351398056	1.13E-10
B163	B166	SG	Pro	0.348904827	1.56E-10
B163	B216	SG	BMI	0.321288519	4.55E-09
B163	B176	SG	Ucr	0.308315868	1.98E-08
B163	B190	SG	Cre	0.299142756	5.37E-08
B163	B191	SG	Urea	0.289274626	1.51E-07
B163	B221	SG	Hei	0.275924621	5.78E-07
B163	B192	SG	UA	0.270463551	9.80E-07
B17	B123	CD3+CD16+CD56+NK	hs-CRP1	-0.50744763	0
B17	B181	CD3+CD16+CD56+NK	APTT	0.574618957	0
B17	B186	CD3+CD16+CD56+NK	PFC	-0.83312517	0
B17	B193	CD3+CD16+CD56+NK	K	0.608847387	0
B17	B194	CD3+CD16+CD56+NK	Cl	0.73597467	0
B17	B197	CD3+CD16+CD56+NK	AG	-0.460943	0
B17	B199	CD3+CD16+CD56+NK	Mg	0.69970468	0
B17	B20	CD3+CD16+CD56+NK	CD3+CD4+CD8+	-0.67631864	0
B17	B83	CD3+CD16+CD56+NK	AFP	-0.93046442	0
B17	B84	CD3+CD16+CD56+NK	C-PSA	0.465903548	0
B17	B89	CD3+CD16+CD56+NK	CA 72-4	0.517486102	0
B17	B23	CD3+CD16+CD56+NK	CD3-CD16+CD56+(NK)	0.436946596	4.44E-16
B17	B69	CD3+CD16+CD56+NK	PV	-0.41143938	2.02E-14
B17	B100	CD3+CD16+CD56+NK	anti-Ro52	0.40501587	5.53E-14
B17	B101	CD3+CD16+CD56+NK	anti-Sc70	0.405015869	5.53E-14
B17	B102	CD3+CD16+CD56+NK	anti-Sm	0.40501587	5.53E-14
B17	B103	CD3+CD16+CD56+NK	anti-SSA/Ro	0.40501587	5.53E-14
B17	B104	CD3+CD16+CD56+NK	anti-SSB	0.40501587	5.53E-14
B17	B105	CD3+CD16+CD56+NK	anti-U1RNP	0.40501587	5.53E-14
B17	B106	CD3+CD16+CD56+NK	anti-P	0.40501587	5.53E-14
B17	B107	CD3+CD16+CD56+NK	AnuA	0.405015865	5.53E-14
B17	B108	CD3+CD16+CD56+NK	Anti-Cyclin	0.40501587	5.53E-14
B17	B109	CD3+CD16+CD56+NK	AMA-M2	0.40501587	5.53E-14
B17	B110	CD3+CD16+CD56+NK	AHA	0.40501587	5.53E-14
B17	B95	CD3+CD16+CD56+NK	anti-cenpb	0.40501587	5.53E-14
B17	B97	CD3+CD16+CD56+NK	dsDNA	0.40501587	5.53E-14
B17	B98	CD3+CD16+CD56+NK	anti-Jo-1	0.405015869	5.53E-14
B17	B99	CD3+CD16+CD56+NK	anti-PM-Scl	0.40501587	5.53E-14
B17	B18	CD3+CD16+CD56+NK	CD3+CD4+helper(inducer)T	-0.39882617	1.43E-13
B17	B151	CD3+CD16+CD56+NK	Anti-CCP	0.397040687	1.88E-13

B17	B86	CD3+CD16+CD56+NK	TBhCG	0.374513269	5.02E-12
B17	B85	CD3+CD16+CD56+NK	SCC	0.358793438	4.29E-11
B17	B156	CD3+CD16+CD56+NK	IgE	0.288804758	1.59E-07
B174	B190	Urea/Cr	Cre	-0.5055585	0
B174	B191	Urea/Cr	Urea	0.627824022	0
B174	B221	Urea/Cr	Hei	-0.30774658	2.11E-08
B174	B192	Urea/Cr	UA	-0.30044165	4.67E-08
B175	B177	ALB-U	ACR	0.87197617	0
B175	B196	ALB-U	TCO2	-0.27382525	7.09E-07
B178	B179	Ig-U	α1-MG	0.830582277	0
B178	B180	Ig-U	Tf	0.956429859	0
B178	B184	Ig-U	INR	0.480135305	0
B179	B180	α1-MG	Tf	0.8899402	0
B18	B134	CD3+CD4+helper(inducer)T	CHE	0.473265022	0
B18	B135	CD3+CD4+helper(inducer)T	TBA	-0.79886481	0
B18	B19	CD3+CD4+helper(inducer)T	CD3+CD4+/CD3+CD8+	0.878428861	0
B18	B20	CD3+CD4+helper(inducer)T	CD3+CD4+CD8+	0.677781207	0
B18	B22	CD3+CD4+helper(inducer)T	CD3+CD8+(T)	-0.86073875	0
B18	B151	CD3+CD4+helper(inducer)T	Anti-CCP	-0.4049726	5.57E-14
B18	B100	CD3+CD4+helper(inducer)T	anti-Ro52	-0.40395404	6.53E-14
B18	B101	CD3+CD4+helper(inducer)T	anti-Sc70	-0.40395405	6.53E-14
B18	B102	CD3+CD4+helper(inducer)T	anti-Sm	-0.40395405	6.53E-14
B18	B103	CD3+CD4+helper(inducer)T	anti-SSA/Ro	-0.40395404	6.53E-14
B18	B104	CD3+CD4+helper(inducer)T	anti-SSB	-0.40395404	6.53E-14
B18	B105	CD3+CD4+helper(inducer)T	anti-U1RNP	-0.40395404	6.53E-14
B18	B106	CD3+CD4+helper(inducer)T	anti-P	-0.40395404	6.53E-14
B18	B107	CD3+CD4+helper(inducer)T	AnuA	-0.40395404	6.53E-14
B18	B108	CD3+CD4+helper(inducer)T	Anti-Cyclin	-0.40395404	6.53E-14
B18	B109	CD3+CD4+helper(inducer)T	AMA-M2	-0.40395404	6.53E-14
B18	B110	CD3+CD4+helper(inducer)T	AHA	-0.40395404	6.53E-14
B18	B95	CD3+CD4+helper(inducer)T	anti-cenpb	-0.40395404	6.53E-14
B18	B97	CD3+CD4+helper(inducer)T	dsDNA	-0.40395404	6.53E-14
B18	B98	CD3+CD4+helper(inducer)T	anti-Jo-1	-0.40395404	6.53E-14
B18	B99	CD3+CD4+helper(inducer)T	anti-PM-Scl	-0.40395404	6.53E-14
B18	B21	CD3+CD4+helper(inducer)T	CD3+CD4-CD8-	-0.39952873	1.29E-13
B18	B198	CD3+CD4+helper(inducer)T	P	0.37543057	4.41E-12
B18	B83	CD3+CD4+helper(inducer)T	AFP	0.371069618	8.10E-12
B18	B57	CD3+CD4+helper(inducer)T	RBCAI	-0.35720577	5.29E-11
B18	B156	CD3+CD4+helper(inducer)T	IgE	-0.35467844	7.37E-11
B18	B43	CD3+CD4+helper(inducer)T	MCV	0.313130611	1.16E-08
B18	B120	CD3+CD4+helper(inducer)T	HCV-cAg	0.311789821	1.34E-08
B18	B44	CD3+CD4+helper(inducer)T	MCH	0.30363349	3.31E-08
B18	B75	CD3+CD4+helper(inducer)T	ApoA1/B	-0.29582146	7.65E-08
B18	B178	CD3+CD4+helper(inducer)T	Ig-U	-0.28254846	3.00E-07

B18	B196	CD3+CD4+helper(inducer)T	TCO2	0.271722228	8.69E-07
B180	B184	Tf	INR	0.443717828	0
B181	B183	APTT	PTA	-0.57113504	0
B181	B186	APTT	PFC	-0.86875573	0
B181	B194	APTT	Cl	0.534408118	0
B181	B199	APTT	Mg	0.4512556	0
B181	B193	APTT	K	0.37700598	3.53E-12
B181	B185	APTT	PT	0.290252331	1.37E-07
B182	B183	TT	PTA	-0.52237114	0
B182	B184	TT	INR	-0.42501378	2.22E-15
B183	B185	PTA	PT	-0.31950466	5.59E-09
B183	B186	PTA	PFC	0.302397815	3.78E-08
B184	B185	INR	PT	0.828685871	0
B186	B193	PFC	K	-0.48508307	0
B186	B194	PFC	Cl	-0.63646627	0
B186	B199	PFC	Mg	-0.5547354	0
B186	B197	PFC	AG	0.381366391	1.90E-12
B187	B198	NSE	P	0.334342977	9.62E-10
B189	B190	eGFB	Cre	-0.57422485	0
B189	B220	eGFB	SBP	-0.35133577	1.14E-10
B189	B191	eGFB	Urea	-0.29900574	5.45E-08
B19	B135	CD3+CD4+/CD3+CD8+	TBA	-0.7153298	0
B19	B20	CD3+CD4+/CD3+CD8+	CD3+CD4+CD8+	0.54932376	0
B19	B21	CD3+CD4+/CD3+CD8+	CD3+CD4-CD8-	-0.53069429	0
B19	B22	CD3+CD4+/CD3+CD8+	CD3+CD8+(T)	-0.93000006	0
B19	B34	CD3+CD4+/CD3+CD8+	TK	0.488457537	0
B19	B134	CD3+CD4+/CD3+CD8+	CHE	0.374747732	4.85E-12
B19	B100	CD3+CD4+/CD3+CD8+	anti-Ro52	-0.355961	6.23E-11
B19	B101	CD3+CD4+/CD3+CD8+	anti-Sc70	-0.355961	6.23E-11
B19	B102	CD3+CD4+/CD3+CD8+	anti-Sm	-0.355961	6.23E-11
B19	B103	CD3+CD4+/CD3+CD8+	anti-SSA/Ro	-0.355961	6.23E-11
B19	B104	CD3+CD4+/CD3+CD8+	anti-SSB	-0.355961	6.23E-11
B19	B105	CD3+CD4+/CD3+CD8+	anti-U1RNP	-0.355961	6.23E-11
B19	B106	CD3+CD4+/CD3+CD8+	anti-P	-0.355961	6.23E-11
B19	B107	CD3+CD4+/CD3+CD8+	AnuA	-0.355961	6.23E-11
B19	B108	CD3+CD4+/CD3+CD8+	Anti-Cyclin	-0.355961	6.23E-11
B19	B109	CD3+CD4+/CD3+CD8+	AMA-M2	-0.355961	6.23E-11
B19	B110	CD3+CD4+/CD3+CD8+	AHA	-0.355961	6.23E-11
B19	B95	CD3+CD4+/CD3+CD8+	anti-cenpb	-0.355961	6.23E-11
B19	B97	CD3+CD4+/CD3+CD8+	dsDNA	-0.355961	6.23E-11
B19	B98	CD3+CD4+/CD3+CD8+	anti-Jo-1	-0.355961	6.23E-11
B19	B99	CD3+CD4+/CD3+CD8+	anti-PM-Scl	-0.355961	6.23E-11
B19	B125	CD3+CD4+/CD3+CD8+	Cor	-0.34450879	2.73E-10
B19	B151	CD3+CD4+/CD3+CD8+	Anti-CCP	-0.3422218	3.63E-10

B19	B114	CD3+CD4+/CD3+CD8+	TSH TT3 TT4(TT4)	-0.32596209	2.63E-09
B19	B156	CD3+CD4+/CD3+CD8+	IgE	-0.30011663	4.84E-08
B19	B94	CD3+CD4+/CD3+CD8+	ALDH2-Lys504Lys	-0.29887977	5.53E-08
B19	B124	CD3+CD4+/CD3+CD8+	ACTH	-0.29652432	7.10E-08
B19	B120	CD3+CD4+/CD3+CD8+	HCV-cAg	0.283067379	2.85E-07
B190	B192	Cre	UA	0.582171275	0
B190	B222	Cre	NS	0.437410458	2.22E-16
B190	B221	Cre	Hei	0.309129824	1.81E-08
B190	B191	Cre	Urea	0.308474079	1.95E-08
B190	B216	Cre	BMI	0.299070398	5.41E-08
B190	B206	Cre	Waist	0.276771921	5.32E-07
B192	B222	UA	NS	0.529607775	0
B192	B216	UA	BMI	0.438804968	2.22E-16
B192	B221	UA	Hei	0.407618392	3.69E-14
B192	B206	UA	Waist	0.376271794	3.92E-12
B192	B215	UA	BMI	0.284548048	2.45E-07
B192	B207	UA	WHR	0.28229958	3.08E-07
B193	B194	K	Cl	0.63801903	0
B193	B199	K	Mg	0.658969849	0
B193	B197	K	AG	-0.35518322	6.90E-11
B194	B197	Cl	AG	-0.44933149	0
B194	B199	Cl	Mg	0.610651521	0
B194	B196	Cl	TCO2	-0.31642884	7.95E-09
B195	B197	Na	AG	0.743430912	0
B195	B200	Na	OP	0.673850214	0
B195	B196	Na	TCO2	-0.3719814	7.14E-12
B195	B198	Na	P	-0.28710205	1.89E-07
B196	B197	TCO2	AG	-0.60457206	0
B197	B199	AG	Mg	-0.58307264	0
B197	B200	AG	OP	0.631187863	0
B197	B198	AG	P	-0.2834388	2.74E-07
B198	B200	P	OP	-0.42463839	2.22E-15
B198	B199	P	Mg	-0.29993671	4.93E-08
B2	B189	Age	eGFB	-0.57056915	0
B2	B220	Age	SBP	0.429708939	8.88E-16
B2	B47	Age	RDW	0.382583998	1.59E-12
B2	B221	Age	Hei	-0.34415099	2.85E-10
B2	B191	Age	Urea	0.287772538	1.77E-07
B2	B126	Age	Alb	-0.2798959	3.91E-07
B20	B135	CD3+CD4+CD8+	TBA	-0.61526221	0
B20	B181	CD3+CD4+CD8+	APTT	-0.53217528	0
B20	B186	CD3+CD4+CD8+	PFC	0.608919991	0
B20	B22	CD3+CD4+CD8+	CD3+CD8+(T)	-0.61335159	0
B20	B83	CD3+CD4+CD8+	AFP	0.629204477	0

B20	B198	CD3+CD4+CD8+	P	0.426301522	1.78E-15
B20	B69	CD3+CD4+CD8+	PV	0.42644748	1.78E-15
B20	B123	CD3+CD4+CD8+	hs-CRP1	0.368698624	1.12E-11
B20	B89	CD3+CD4+CD8+	CA 72-4	-0.35944365	3.93E-11
B20	B134	CD3+CD4+CD8+	CHE	0.355083574	6.99E-11
B20	B199	CD3+CD4+CD8+	Mg	-0.3494611	1.45E-10
B20	B194	CD3+CD4+CD8+	Cl	-0.3372181	6.77E-10
B20	B184	CD3+CD4+CD8+	INR	0.327657303	2.15E-09
B20	B84	CD3+CD4+CD8+	C-PSA	-0.32202775	4.17E-09
B20	B196	CD3+CD4+CD8+	TCO2	0.312444725	1.25E-08
B20	B155	CD3+CD4+CD8+	IgA	0.304328384	3.07E-08
B20	B94	CD3+CD4+CD8+	ALDH2-Lys504Lys	0.287993637	1.73E-07
B20	B62	CD3+CD4+CD8+	WBHSRVI	-0.28247101	3.02E-07
B20	B34	CD3+CD4+CD8+	TK	-0.27670661	5.35E-07
B204	B209	Drinking	Smoking	0.482504471	0
B204	B221	Drinking	Hei	0.291909612	1.15E-07
B206	B207	Waist	WHR	0.560327769	0
B206	B210	Waist	Hip	0.686047819	0
B206	B215	Waist	BMI	0.886917023	0
B206	B216	Waist	BMI	0.832474216	0
B206	B222	Waist	NS	0.758881315	0
B206	B218	Waist	DBP	0.353625314	8.46E-11
B206	B220	Waist	SBP	0.317914676	6.71E-09
B206	B221	Waist	Hei	0.287568639	1.80E-07
B207	B215	WHR	BMI	0.437764615	2.22E-16
B207	B222	WHR	NS	0.433147548	4.44E-16
B207	B216	WHR	BMI	0.375596485	4.31E-12
B209	B222	Smoking	NS	0.30124367	4.29E-08
B21	B100	CD3+CD4-CD8-	anti-Ro52	0.638459265	0
B21	B101	CD3+CD4-CD8-	anti-Sc70	0.638459266	0
B21	B102	CD3+CD4-CD8-	anti-Sm	0.638459266	0
B21	B103	CD3+CD4-CD8-	anti-SSA/Ro	0.638459265	0
B21	B104	CD3+CD4-CD8-	anti-SSB	0.638459265	0
B21	B105	CD3+CD4-CD8-	anti-U1RNP	0.638459265	0
B21	B106	CD3+CD4-CD8-	anti-P	0.638459265	0
B21	B107	CD3+CD4-CD8-	AnuA	0.638459257	0
B21	B108	CD3+CD4-CD8-	Anti-Cyclin	0.638459265	0
B21	B109	CD3+CD4-CD8-	AMA-M2	0.638459265	0
B21	B110	CD3+CD4-CD8-	AHA	0.638459265	0
B21	B151	CD3+CD4-CD8-	Anti-CCP	0.646429098	0
B21	B156	CD3+CD4-CD8-	IgE	0.595568957	0
B21	B34	CD3+CD4-CD8-	TK	-0.57098546	0
B21	B79	CD3+CD4-CD8-	hb V -dna	0.577643304	0
B21	B95	CD3+CD4-CD8-	anti-cenpb	0.638459265	0

B21	B97	CD3+CD4-CD8-	dsDNA	0.638459264	0
B21	B98	CD3+CD4-CD8-	anti-Jo-1	0.638459264	0
B21	B99	CD3+CD4-CD8-	anti-PM-Scl	0.638459264	0
B21	B138	CD3+CD4-CD8-	PAB	0.420899121	4.44E-15
B21	B157	CD3+CD4-CD8-	IgG	0.41633003	9.33E-15
B21	B184	CD3+CD4-CD8-	INR	0.408907601	3.02E-14
B21	B158	CD3+CD4-CD8-	IgM	-0.40258619	8.06E-14
B21	B57	CD3+CD4-CD8-	RBCAI	0.366276945	1.57E-11
B21	B183	CD3+CD4-CD8-	PTA	0.359717767	3.79E-11
B21	B124	CD3+CD4-CD8-	ACTH	0.358018145	4.75E-11
B21	B39	CD3+CD4-CD8-	Other cells	0.330375603	1.55E-09
B21	B81	CD3+CD4-CD8-	TSGF	-0.32897941	1.84E-09
B21	B176	CD3+CD4-CD8-	Ucr	0.323618957	3.46E-09
B21	B22	CD3+CD4-CD8-	CD3+CD8+(T)	0.314857621	9.51E-09
B21	B55	CD3+CD4-CD8-	TK	-0.31154544	1.38E-08
B21	B76	CD3+CD4-CD8-	ApoB	0.30567245	2.65E-08
B21	B23	CD3+CD4-CD8-	CD3-CD16+CD56+(NK)	-0.30163881	4.11E-08
B21	B178	CD3+CD4-CD8-	Ig-U	0.294274351	9.00E-08
B21	B56	CD3+CD4-CD8-	IR	-0.27800056	4.71E-07
B21	B193	CD3+CD4-CD8-	K	0.275202711	6.20E-07
B210	B215	Hip	BMI	0.705810086	0
B210	B216	Hip	BMI	0.746899498	0
B210	B222	Hip	NS	0.570596761	0
B210	B221	Hip	Hei	0.377718456	3.19E-12
B210	B218	Hip	DBP	0.273096994	7.61E-07
B211	B212	IOP-R	IOP-L	0.757291722	0
B215	B216	BMI	BMI	0.836550562	0
B215	B222	BMI	NS	0.716129486	0
B215	B218	BMI	DBP	0.380879002	2.03E-12
B215	B220	BMI	SBP	0.331588326	1.34E-09
B216	B221	BMI	Hei	0.64852655	0
B216	B222	BMI	NS	0.811615675	0
B216	B218	BMI	DBP	0.359576853	3.86E-11
B218	B220	DBP	SBP	0.651986042	0
B218	B222	DBP	NS	0.350128823	1.33E-10
B22	B135	CD3+CD8+(T)	TBA	0.901971578	0
B22	B197	CD3+CD8+(T)	AG	0.448072081	0
B22	B200	CD3+CD8+(T)	OP	0.396108173	2.17E-13
B22	B114	CD3+CD8+(T)	TSH TT3 TT4(TT4)	0.385310802	1.07E-12
B22	B134	CD3+CD8+(T)	CHE	-0.38524714	1.08E-12
B22	B193	CD3+CD8+(T)	K	-0.34691664	2.01E-10
B22	B43	CD3+CD8+(T)	MCV	-0.33956108	5.06E-10
B22	B120	CD3+CD8+(T)	HCV-cAg	-0.33684183	7.09E-10
B22	B44	CD3+CD8+(T)	MCH	-0.32746101	2.20E-09

B22	B198	CD3+CD8+(T)	P	-0.31283238	1.20E-08
B22	B194	CD3+CD8+(T)	Cl	-0.28980735	1.43E-07
B22	B34	CD3+CD8+(T)	TK	-0.27858946	4.45E-07
B22	B195	CD3+CD8+(T)	Na	0.27395709	7.00E-07
B221	B222	Hei	NS	0.483491593	0
B23	B125	CD3-CD16+CD56+(NK)	Cor	-0.6030885	0
B23	B181	CD3-CD16+CD56+(NK)	APTT	0.47414844	0
B23	B199	CD3-CD16+CD56+(NK)	Mg	0.574732147	0
B23	B34	CD3-CD16+CD56+(NK)	TK	0.667636574	0
B23	B194	CD3-CD16+CD56+(NK)	Cl	0.422330795	3.55E-15
B23	B83	CD3-CD16+CD56+(NK)	AFP	-0.40659546	4.33E-14
B23	B90	CD3-CD16+CD56+(NK)	CYFRA 21-1	-0.38279534	1.54E-12
B23	B187	CD3-CD16+CD56+(NK)	NSE	-0.37413165	5.29E-12
B23	B186	CD3-CD16+CD56+(NK)	PFC	-0.35562442	6.51E-11
B23	B137	CD3-CD16+CD56+(NK)	AFU	-0.35548526	6.63E-11
B23	B193	CD3-CD16+CD56+(NK)	K	0.351958081	1.05E-10
B23	B57	CD3-CD16+CD56+(NK)	RBCAI	0.315029594	9.33E-09
B23	B76	CD3-CD16+CD56+(NK)	ApoB	-0.31095916	1.48E-08
B23	B179	CD3-CD16+CD56+(NK)	α1-MG	0.308065852	2.04E-08
B24	B26	αHBDH	CKMB	0.549364557	0
B24	B27	αHBDH	LDH	0.662601313	0
B24	B90	αHBDH	CYFRA 21-1	0.275351663	6.11E-07
B25	B39	CK	Other cells	0.447485202	0
B25	B57	CK	RBCAI	0.364212542	2.07E-11
B25	B26	CK	CKMB	0.312810015	1.20E-08
B25	B69	CK	PV	0.292006289	1.14E-07
B26	B27	CKMB	LDH	0.450162754	0
B27	B213	LDH	CVA-R	0.494212905	0
B27	B176	LDH	Ucr	-0.28583905	2.15E-07
B28	B164	PRL	COM	-0.58343433	0
B28	B30	PRL	LH	1	0
B28	B32	PRL	Testo	-1	0
B29	B164	E2	COM	0.58343433	0
B29	B30	E2	LH	-1	0
B29	B32	E2	Testo	1	0
B3	B85	Hcy	SCC	0.328987096	1.84E-09
B3	B84	Hcy	C-PSA	0.272195382	8.30E-07
B30	B164	LH	COM	-0.58343433	0
B30	B31	LH	FSH	1	0
B30	B32	LH	Testo	-1	0
B30	B33	LH	Prog	-1	0
B31	B164	FSH	COM	-0.58343433	0
B31	B32	FSH	Testo	-1	0
B32	B164	Testo	COM	0.58343433	0

B32	B33	Testo	Prog	1	0
B33	B164	Prog	COM	0.58343433	0
B34	B124	TK	ACTH	-0.5210076	0
B34	B125	TK	Cor	-0.52169085	0
B34	B181	TK	APTT	0.557683407	0
B34	B79	TK	hb V -dna	-0.35037167	1.29E-10
B34	B187	TK	NSE	-0.34684582	2.03E-10
B34	B94	TK	ALDH2-Lys504Lys	-0.34608381	2.23E-10
B34	B199	TK	Mg	0.333453356	1.07E-09
B34	B158	TK	IgM	0.318130245	6.55E-09
B34	B69	TK	PV	-0.31217438	1.29E-08
B34	B92	TK	ALDH2-Glu504Glu	0.293175219	1.01E-07
B34	B93	TK	ALDH2-Glu504Lys	-0.27604734	5.71E-07
B34	B198	TK	P	-0.27539219	6.09E-07
B35	B116	β2-MG	TT3	-0.90093746	0
B35	B202	β2-MG	e-times	-0.54272042	0
B35	B37	β2-MG	BAND	-0.38376129	1.34E-12
B36	B167	V-Lym%	PC	0.707174992	0
B36	B96	V-Lym%	dsDNA (IIF)	0.679873454	0
B39	B57	Other cells	RBCAI	0.425113928	2.22E-15
B39	B180	Other cells	Tf	0.378815166	2.73E-12
B39	B60	Other cells	WBRVI	0.373841177	5.51E-12
B39	B78	Other cells	HBcAb	0.366008462	1.62E-11
B39	B65	Other cells	WBRSR(mPas_1)	0.357706376	4.95E-11
B39	B179	Other cells	α1-MG	0.354896324	7.17E-11
B39	B178	Other cells	Ig-U	0.349114533	1.52E-10
B39	B64	Other cells	WBRSR(mPas_5)	0.326679533	2.42E-09
B39	B59	Other cells	WBRV	0.302040361	3.93E-08
B39	B151	Other cells	Anti-CCP	0.280584068	3.65E-07
B39	B100	Other cells	anti-Ro52	0.275282728	6.15E-07
B39	B101	Other cells	anti-Sc70	0.275282729	6.15E-07
B39	B102	Other cells	anti-Sm	0.275282729	6.15E-07
B39	B103	Other cells	anti-SSA/Ro	0.275282728	6.15E-07
B39	B104	Other cells	anti-SSB	0.275282729	6.15E-07
B39	B105	Other cells	anti-U1RNP	0.275282728	6.15E-07
B39	B106	Other cells	anti-P	0.275282728	6.15E-07
B39	B107	Other cells	AnuA	0.275282725	6.15E-07
B39	B108	Other cells	Anti-Cyclin	0.275282729	6.15E-07
B39	B109	Other cells	AMA-M2	0.275282728	6.15E-07
B39	B110	Other cells	AHA	0.275282728	6.15E-07
B39	B95	Other cells	anti-cenpb	0.275282729	6.15E-07
B39	B97	Other cells	dsDNA	0.275282728	6.15E-07
B39	B98	Other cells	anti-Jo-1	0.275282728	6.15E-07
B39	B99	Other cells	anti-PM-Scl	0.275282728	6.15E-07

B4	B137	WB-Ca	AFU	0.452850717	0
B4	B138	WB-Ca	PAB	0.52045028	0
B4	B16	WB-Ca	CD3+T	0.659409978	0
B4	B21	WB-Ca	CD3+CD4-CD8-	0.514334406	0
B4	B23	WB-Ca	CD3-CD16+CD56+(NK)	-0.62509392	0
B4	B5	WB-Ca	WB-Mg	1	0
B4	B6	WB-Ca	WB-Fe	1	0
B4	B7	WB-Ca	WB-Cop	1	0
B4	B76	WB-Ca	ApoB	0.443255337	0
B4	B8	WB-Ca	WB-Zinc	1	0
B4	B94	WB-Ca	ALDH2-Lys504Lys	-0.49762845	0
B4	B15	WB-Ca	CD3-CD19+(B cell)	-0.39394978	3.00E-13
B4	B179	WB-Ca	α1-MG	-0.38747121	7.82E-13
B4	B178	WB-Ca	Ig-U	-0.38072155	2.08E-12
B4	B57	WB-Ca	RBCAI	-0.3714923	7.64E-12
B4	B85	WB-Ca	SCC	-0.36823304	1.20E-11
B4	B92	WB-Ca	ALDH2-Glu504Glu	0.364342135	2.04E-11
B4	B75	WB-Ca	ApoA1/B	-0.35273649	9.50E-11
B4	B180	WB-Ca	Tf	-0.34443905	2.75E-10
B4	B93	WB-Ca	ALDH2-Glu504Lys	-0.33929588	5.23E-10
B4	B90	WB-Ca	CYFRA 21-1	0.312457974	1.25E-08
B4	B176	WB-Ca	Ucr	0.295282965	8.09E-08
B4	B200	WB-Ca	OP	0.291810316	1.16E-07
B4	B134	WB-Ca	CHE	0.29147904	1.21E-07
B4	B195	WB-Ca	Na	0.290527589	1.33E-07
B4	B198	WB-Ca	P	-0.27659081	5.42E-07
B40	B42	WBC	MON	0.622408679	0
B41	B42	MON %	MON	0.702632156	0
B41	B190	MON %	Cre	0.271142705	9.18E-07
B42	B190	MON	Cre	0.330006996	1.62E-09
B43	B44	MCV	MCH	0.902288532	0
B43	B46	MCV	RBC Count	-0.61867737	0
B43	B47	MCV	RDW	0.508239105	0
B43	B135	MCV	TBA	-0.38774998	7.51E-13
B44	B45	MCH	MCHC	0.533881408	0
B44	B46	MCH	RBC Count	-0.48676848	0
B44	B135	MCH	TBA	-0.35367891	8.40E-11
B44	B47	MCH	RDW	0.314817957	9.55E-09
B45	B51	MCHC	Hb	0.514496546	0
B45	B222	MCHC	NS	0.327193704	2.27E-09
B45	B221	MCHC	Hei	0.32026516	5.12E-09
B45	B216	MCHC	BMI	0.317173426	7.31E-09
B45	B71	MCHC	TG	0.292574968	1.08E-07
B45	B47	MCHC	RDW	-0.27814951	4.65E-07

B46	B51	RBC Count	Hb	0.722785008	0
B46	B47	RBC Count	RDW	-0.43915076	2.22E-16
B46	B222	RBC Count	NS	0.386499263	9.02E-13
B46	B190	RBC Count	Cre	0.338915235	5.49E-10
B46	B216	RBC Count	BMI	0.334120218	9.88E-10
B46	B72	RBC Count	HDL-C	-0.32221178	4.08E-09
B46	B218	RBC Count	DBP	0.305989548	2.56E-08
B46	B192	RBC Count	UA	0.303840612	3.24E-08
B46	B221	RBC Count	Hei	0.303109262	3.50E-08
B48	B53	LYM%	GR%	-0.93400012	0
B49	B50	baso%	eo%	0.296701494	6.97E-08
B5	B137	WB-Mg	AFU	0.452850717	0
B5	B138	WB-Mg	PAB	0.52045028	0
B5	B16	WB-Mg	CD3+T	0.659409978	0
B5	B21	WB-Mg	CD3+CD4-CD8-	0.514334406	0
B5	B23	WB-Mg	CD3-CD16+CD56+(NK)	-0.62509392	0
B5	B7	WB-Mg	WB-Cop	1	0
B5	B76	WB-Mg	ApoB	0.443255337	0
B5	B8	WB-Mg	WB-Zinc	1	0
B5	B94	WB-Mg	ALDH2-Lys504Lys	-0.49762844	0
B5	B15	WB-Mg	CD3-CD19+(B cell)	-0.39394978	3.00E-13
B5	B179	WB-Mg	α1-MG	-0.38747121	7.82E-13
B5	B178	WB-Mg	Ig-U	-0.38072155	2.08E-12
B5	B57	WB-Mg	RBCAI	-0.37149229	7.64E-12
B5	B85	WB-Mg	SCC	-0.36823304	1.20E-11
B5	B92	WB-Mg	ALDH2-Glu504Glu	0.364342135	2.04E-11
B5	B75	WB-Mg	ApoA1/B	-0.35273649	9.50E-11
B5	B180	WB-Mg	Tf	-0.34443905	2.75E-10
B5	B93	WB-Mg	ALDH2-Glu504Lys	-0.33929588	5.23E-10
B5	B90	WB-Mg	CYFRA 21-1	0.312457974	1.25E-08
B5	B176	WB-Mg	Ucr	0.295282965	8.09E-08
B5	B200	WB-Mg	OP	0.291810317	1.16E-07
B5	B134	WB-Mg	CHE	0.29147904	1.21E-07
B5	B195	WB-Mg	Na	0.29052759	1.33E-07
B5	B198	WB-Mg	P	-0.27659081	5.42E-07
B50	B53	eo%	GR%	-0.28346989	2.73E-07
B51	B216	Hb	BMI	0.454530105	0
B51	B221	Hb	Hei	0.457770411	0
B51	B222	Hb	NS	0.513800953	0
B51	B190	Hb	Cre	0.402104869	8.68E-14
B51	B192	Hb	UA	0.395251083	2.46E-13
B51	B163	Hb	SG	0.391164038	4.54E-13
B51	B72	Hb	HDL-C	-0.34565108	2.36E-10
B51	B174	Hb	Urea/Cr	-0.33483236	9.06E-10

B51	B206	Hb	Waist	0.328333112	1.98E-09
B51	B218	Hb	DBP	0.306676962	2.37E-08
B51	B141	Hb	AST/ALT	-0.29085825	1.29E-07
B51	B58	Hb	HCT	0.274760562	6.47E-07
B52	B70	PLT/BPC	LDL-C	0.276891526	5.26E-07
B54	B175	ESR	ALB-U	0.606123921	0
B54	B177	ESR	ACR	0.680428239	0
B54	B58	ESR	HCT	-0.56901985	0
B54	B62	ESR	WBHSRVI	-0.5342105	0
B54	B69	ESR	PV	0.464598039	0
B54	B60	ESR	WBRVI	-0.41043548	2.35E-14
B54	B155	ESR	IgA	0.392378335	3.79E-13
B54	B123	ESR	hs-CRP1	0.377115972	3.48E-12
B54	B63	ESR	WBRSR(mPas_200)	-0.338929	5.48E-10
B54	B56	ESR	IR	-0.32708725	2.30E-09
B54	B66	ESR	WBRSR(mPas_100)	-0.32209425	4.14E-09
B54	B67	ESR	WBRSR(mPas_50)	-0.31642585	7.96E-09
B55	B100	TK	anti-Ro52	-0.48238707	0
B55	B101	TK	anti-Sc70	-0.48238707	0
B55	B102	TK	anti-Sm	-0.48238707	0
B55	B103	TK	anti-SSA/Ro	-0.48238707	0
B55	B104	TK	anti-SSB	-0.48238707	0
B55	B105	TK	anti-U1RNP	-0.48238707	0
B55	B106	TK	anti-P	-0.48238707	0
B55	B107	TK	AnuA	-0.48238706	0
B55	B108	TK	Anti-Cyclin	-0.48238707	0
B55	B109	TK	AMA-M2	-0.48238707	0
B55	B110	TK	AHA	-0.48238707	0
B55	B151	TK	Anti-CCP	-0.47494673	0
B55	B156	TK	IgE	-0.58443017	0
B55	B184	TK	INR	-0.75603691	0
B55	B185	TK	PT	-0.66802379	0
B55	B56	TK	IR	0.913873027	0
B55	B59	TK	WBRV	0.53584953	0
B55	B61	TK	WBHSRV	0.866322572	0
B55	B62	TK	WBHSRVI	0.620980942	0
B55	B63	TK	WBRSR(mPas_200)	0.489437186	0
B55	B66	TK	WBRSR(mPas_100)	0.487134729	0
B55	B67	TK	WBRSR(mPas_50)	0.455711952	0
B55	B79	TK	hb V -dna	-0.50011517	0
B55	B92	TK	ALDH2-Glu504Glu	-0.55736182	0
B55	B93	TK	ALDH2-Glu504Lys	0.592658712	0
B55	B94	TK	ALDH2-Lys504Lys	-0.56067198	0
B55	B95	TK	anti-cenpb	-0.48238707	0

B55	B97	TK	dsDNA	-0.48238707	0
B55	B98	TK	anti-Jo-1	-0.48238707	0
B55	B99	TK	anti-PM-Scl	-0.48238706	0
B55	B178	TK	Ig-U	-0.43948821	2.22E-16
B55	B180	TK	Tf	-0.40928355	2.84E-14
B55	B57	TK	RBCAI	-0.34866845	1.61E-10
B55	B81	TK	TSGF	0.331992741	1.28E-09
B55	B154	TK	RF	-0.32796641	2.07E-09
B55	B64	TK	WBRSR(mPas_5)	0.317538557	7.01E-09
B55	B182	TK	TT	0.298260569	5.90E-08
B55	B183	TK	PTA	-0.29021103	1.37E-07
B56	B100	IR	anti-Ro52	-0.45070213	0
B56	B101	IR	anti-Sc70	-0.45070213	0
B56	B102	IR	anti-Sm	-0.45070213	0
B56	B103	IR	anti-SSA/Ro	-0.45070213	0
B56	B104	IR	anti-SSB	-0.45070213	0
B56	B105	IR	anti-U1RNP	-0.45070213	0
B56	B106	IR	anti-P	-0.45070213	0
B56	B107	IR	AnuA	-0.45070213	0
B56	B108	IR	Anti-Cyclin	-0.45070213	0
B56	B109	IR	AMA-M2	-0.45070213	0
B56	B110	IR	AHA	-0.45070213	0
B56	B151	IR	Anti-CCP	-0.4439355	0
B56	B156	IR	IgE	-0.50552841	0
B56	B178	IR	Ig-U	-0.4474319	0
B56	B184	IR	INR	-0.73734556	0
B56	B185	IR	PT	-0.68086459	0
B56	B58	IR	HCT	0.520497407	0
B56	B59	IR	WBRV	0.702513463	0
B56	B60	IR	WBRVI	0.555886674	0
B56	B61	IR	WBHSRV	0.966021408	0
B56	B62	IR	WBHSRVI	0.880283392	0
B56	B63	IR	WBRSR(mPas_200)	0.761781409	0
B56	B64	IR	WBRSR(mPas_5)	0.599241935	0
B56	B65	IR	WBRSR(mPas_1)	0.530191926	0
B56	B66	IR	WBRSR(mPas_100)	0.756306412	0
B56	B67	IR	WBRSR(mPas_50)	0.731033291	0
B56	B79	IR	hb V -dna	-0.51325512	0
B56	B92	IR	ALDH2-Glu504Glu	-0.61637245	0
B56	B93	IR	ALDH2-Glu504Lys	0.65265477	0
B56	B94	IR	ALDH2-Lys504Lys	-0.57061899	0
B56	B95	IR	anti-cenpb	-0.45070213	0
B56	B97	IR	dsDNA	-0.45070213	0
B56	B98	IR	anti-Jo-1	-0.45070213	0

B56	B99	IR	anti-PM-Scl	-0.45070213	0
B56	B180	IR	Tf	-0.41817552	6.88E-15
B56	B81	IR	TSGF	0.340114654	4.73E-10
B57	B100	RBCAI	anti-Ro52	0.484457407	0
B57	B101	RBCAI	anti-Sc70	0.484457408	0
B57	B102	RBCAI	anti-Sm	0.484457407	0
B57	B103	RBCAI	anti-SSA/Ro	0.484457407	0
B57	B104	RBCAI	anti-SSB	0.484457407	0
B57	B105	RBCAI	anti-U1RNP	0.484457407	0
B57	B106	RBCAI	anti-P	0.484457407	0
B57	B107	RBCAI	AnuA	0.484457401	0
B57	B108	RBCAI	Anti-Cyclin	0.484457407	0
B57	B109	RBCAI	AMA-M2	0.484457407	0
B57	B110	RBCAI	AHA	0.484457407	0
B57	B151	RBCAI	Anti-CCP	0.495287936	0
B57	B178	RBCAI	Ig-U	0.517143354	0
B57	B179	RBCAI	α1-MG	0.48953876	0
B57	B180	RBCAI	Tf	0.468563991	0
B57	B58	RBCAI	HCT	0.458750924	0
B57	B59	RBCAI	WBRV	0.553994626	0
B57	B60	RBCAI	WBRVI	0.703072189	0
B57	B64	RBCAI	WBRSR(mPas_5)	0.607991695	0
B57	B65	RBCAI	WBRSR(mPas_1)	0.677527692	0
B57	B94	RBCAI	ALDH2-Lys504Lys	0.455319477	0
B57	B95	RBCAI	anti-cenpb	0.484457407	0
B57	B97	RBCAI	dsDNA	0.484457406	0
B57	B98	RBCAI	anti-Jo-1	0.484457407	0
B57	B99	RBCAI	anti-PM-Scl	0.484457406	0
B57	B79	RBCAI	hb V -dna	0.438467924	2.22E-16
B57	B69	RBCAI	PV	0.434666678	4.44E-16
B57	B67	RBCAI	WBRSR(mPas_50)	0.425345731	2.22E-15
B57	B158	RBCAI	IgM	-0.41064455	2.29E-14
B57	B66	RBCAI	WBRSR(mPas_100)	0.376110437	4.01E-12
B57	B156	RBCAI	IgE	0.371925905	7.20E-12
B57	B63	RBCAI	WBRSR(mPas_200)	0.36780602	1.27E-11
B57	B157	RBCAI	IgG	0.362721666	2.53E-11
B57	B90	RBCAI	CYFRA 21-1	-0.35833631	4.55E-11
B57	B155	RBCAI	IgA	0.354419713	7.63E-11
B57	B154	RBCAI	RF	0.314934631	9.43E-09
B57	B184	RBCAI	INR	0.270775909	9.51E-07
B58	B59	HCT	WBRV	0.611133444	0
B58	B60	HCT	WBRVI	0.853327718	0
B58	B61	HCT	WBHSRV	0.542280492	0
B58	B62	HCT	WBHSRVI	0.815656052	0

B58	B63	HCT	WBRSR(mPas_200)	0.811170945	0
B58	B64	HCT	WBRSR(mPas_5)	0.78572606	0
B58	B65	HCT	WBRSR(mPas_1)	0.787191	0
B58	B66	HCT	WBRSR(mPas_100)	0.802778804	0
B58	B67	HCT	WBRSR(mPas_50)	0.808796848	0
B58	B92	HCT	ALDH2-Glu504Glu	-0.44063063	0
B58	B93	HCT	ALDH2-Glu504Lys	0.453504359	0
B58	B155	HCT	IgA	-0.42360857	2.66E-15
B58	B185	HCT	PT	-0.29544211	7.96E-08
B59	B185	WBRV	PT	-0.55635177	0
B59	B60	WBRV	WBRVI	0.856565903	0
B59	B61	WBRV	WBHSRV	0.82222459	0
B59	B62	WBRV	WBHSRVI	0.738334888	0
B59	B63	WBRV	WBRSR(mPas_200)	0.871901034	0
B59	B64	WBRV	WBRSR(mPas_5)	0.911434055	0
B59	B65	WBRV	WBRSR(mPas_1)	0.879366271	0
B59	B66	WBRV	WBRSR(mPas_100)	0.879377723	0
B59	B67	WBRV	WBRSR(mPas_50)	0.891242559	0
B59	B92	WBRV	ALDH2-Glu504Glu	-0.66502903	0
B59	B93	WBRV	ALDH2-Glu504Lys	0.67686024	0
B59	B69	WBRV	PV	0.438705305	2.22E-16
B59	B184	WBRV	INR	-0.38577432	1.00E-12
B59	B158	WBRV	IgM	-0.35187844	1.06E-10
B59	B124	WBRV	ACTH	0.285872389	2.14E-07
B59	B179	WBRV	α1-MG	0.28438483	2.49E-07
B6	B137	WB-Fe	AFU	0.452850717	0
B6	B138	WB-Fe	PAB	0.52045028	0
B6	B16	WB-Fe	CD3+T	0.659409978	0
B6	B21	WB-Fe	CD3+CD4-CD8-	0.514334406	0
B6	B23	WB-Fe	CD3-CD16+CD56+(NK)	-0.62509392	0
B6	B7	WB-Fe	WB-Cop	1	0
B6	B76	WB-Fe	ApoB	0.443255337	0
B6	B8	WB-Fe	WB-Zinc	1	0
B6	B94	WB-Fe	ALDH2-Lys504Lys	-0.49762844	0
B6	B15	WB-Fe	CD3-CD19+(B cell)	-0.39394978	3.00E-13
B6	B179	WB-Fe	α1-MG	-0.38747121	7.82E-13
B6	B178	WB-Fe	Ig-U	-0.38072155	2.08E-12
B6	B57	WB-Fe	RBCAI	-0.37149229	7.64E-12
B6	B85	WB-Fe	SCC	-0.36823304	1.20E-11
B6	B92	WB-Fe	ALDH2-Glu504Glu	0.364342135	2.04E-11
B6	B75	WB-Fe	ApoA1/B	-0.35273649	9.50E-11
B6	B180	WB-Fe	Tf	-0.34443905	2.75E-10
B6	B93	WB-Fe	ALDH2-Glu504Lys	-0.33929588	5.23E-10
B6	B90	WB-Fe	CYFRA 21-1	0.312457974	1.25E-08

B6	B176	WB-Fe	Ucr	0.295282965	8.09E-08
B6	B200	WB-Fe	OP	0.291810317	1.16E-07
B6	B134	WB-Fe	CHE	0.29147904	1.21E-07
B6	B195	WB-Fe	Na	0.29052759	1.33E-07
B6	B198	WB-Fe	P	-0.27659081	5.42E-07
B60	B61	WBRVI	WBHSRV	0.625919965	0
B60	B62	WBRVI	WBHSRVI	0.813815846	0
B60	B63	WBRVI	WBRSR(mPas_200)	0.882598303	0
B60	B64	WBRVI	WBRSR(mPas_5)	0.946030663	0
B60	B65	WBRVI	WBRSR(mPas_1)	0.965621928	0
B60	B66	WBRVI	WBRSR(mPas_100)	0.88344806	0
B60	B67	WBRVI	WBRSR(mPas_50)	0.903585544	0
B60	B92	WBRVI	ALDH2-Glu504Glu	-0.46509955	0
B60	B93	WBRVI	ALDH2-Glu504Lys	0.471235996	0
B60	B185	WBRVI	PT	-0.34038153	4.57E-10
B60	B199	WBRVI	Mg	0.273326099	7.44E-07
B61	B156	WBHSRV	IgE	-0.44240159	0
B61	B184	WBHSRV	INR	-0.65004671	0
B61	B185	WBHSRV	PT	-0.67389552	0
B61	B62	WBHSRV	WBHSRVI	0.86833035	0
B61	B63	WBHSRV	WBRSR(mPas_200)	0.851806091	0
B61	B64	WBHSRV	WBRSR(mPas_5)	0.723031509	0
B61	B65	WBHSRV	WBRSR(mPas_1)	0.646731945	0
B61	B66	WBHSRV	WBRSR(mPas_100)	0.851208047	0
B61	B67	WBHSRV	WBRSR(mPas_50)	0.831425963	0
B61	B92	WBHSRV	ALDH2-Glu504Glu	-0.65447069	0
B61	B93	WBHSRV	ALDH2-Glu504Lys	0.687104331	0
B61	B94	WBHSRV	ALDH2-Lys504Lys	-0.50009272	0
B61	B79	WBHSRV	hb V -dna	-0.41338243	1.47E-14
B61	B100	WBHSRV	anti-Ro52	-0.40094254	1.04E-13
B61	B101	WBHSRV	anti-Sc70	-0.40094254	1.04E-13
B61	B102	WBHSRV	anti-Sm	-0.40094254	1.04E-13
B61	B103	WBHSRV	anti-SSA/Ro	-0.40094254	1.04E-13
B61	B104	WBHSRV	anti-SSB	-0.40094254	1.04E-13
B61	B105	WBHSRV	anti-U1RNP	-0.40094254	1.04E-13
B61	B106	WBHSRV	anti-P	-0.40094254	1.04E-13
B61	B107	WBHSRV	AnuA	-0.40094254	1.04E-13
B61	B108	WBHSRV	Anti-Cyclin	-0.40094254	1.04E-13
B61	B109	WBHSRV	AMA-M2	-0.40094254	1.04E-13
B61	B110	WBHSRV	AHA	-0.40094254	1.04E-13
B61	B95	WBHSRV	anti-cenpb	-0.40094254	1.04E-13
B61	B97	WBHSRV	dsDNA	-0.40094254	1.04E-13
B61	B98	WBHSRV	anti-Jo-1	-0.40094254	1.04E-13
B61	B99	WBHSRV	anti-PM-Scl	-0.40094254	1.04E-13

B61	B151	WBHSRV	Anti-CCP	-0.39008491	5.32E-13
B61	B178	WBHSRV	Ig-U	-0.35003561	1.35E-10
B61	B180	WBHSRV	Tf	-0.32960749	1.70E-09
B61	B81	WBHSRV	TSGF	0.286518233	2.01E-07
B62	B184	WBHSRVI	INR	-0.54090551	0
B62	B185	WBHSRVI	PT	-0.50594891	0
B62	B63	WBHSRVI	WBRSR(mPas_200)	0.92009837	0
B62	B64	WBHSRVI	WBRSR(mPas_5)	0.807806445	0
B62	B65	WBHSRVI	WBRSR(mPas_1)	0.77760547	0
B62	B66	WBHSRVI	WBRSR(mPas_100)	0.912968149	0
B62	B67	WBHSRVI	WBRSR(mPas_50)	0.900415643	0
B62	B92	WBHSRVI	ALDH2-Glu504Glu	-0.48695632	0
B62	B93	WBHSRVI	ALDH2-Glu504Lys	0.515923919	0
B62	B94	WBHSRVI	ALDH2-Lys504Lys	-0.45625559	0
B62	B79	WBHSRVI	hb V -dna	-0.41150801	2.00E-14
B62	B178	WBHSRVI	Ig-U	-0.35914237	4.09E-11
B62	B155	WBHSRVI	IgA	-0.34348134	3.10E-10
B62	B180	WBHSRVI	Tf	-0.33648776	7.40E-10
B62	B100	WBHSRVI	anti-Ro52	-0.30946045	1.74E-08
B62	B101	WBHSRVI	anti-Sc70	-0.30946045	1.74E-08
B62	B102	WBHSRVI	anti-Sm	-0.30946045	1.74E-08
B62	B103	WBHSRVI	anti-SSA/Ro	-0.30946045	1.74E-08
B62	B104	WBHSRVI	anti-SSB	-0.30946045	1.74E-08
B62	B105	WBHSRVI	anti-U1RNP	-0.30946045	1.74E-08
B62	B106	WBHSRVI	anti-P	-0.30946045	1.74E-08
B62	B107	WBHSRVI	AnuA	-0.30946045	1.74E-08
B62	B108	WBHSRVI	Anti-Cyclin	-0.30946045	1.74E-08
B62	B109	WBHSRVI	AMA-M2	-0.30946045	1.74E-08
B62	B110	WBHSRVI	AHA	-0.30946045	1.74E-08
B62	B95	WBHSRVI	anti-cenpb	-0.30946045	1.74E-08
B62	B97	WBHSRVI	dsDNA	-0.30946045	1.74E-08
B62	B98	WBHSRVI	anti-Jo-1	-0.30946045	1.74E-08
B62	B99	WBHSRVI	anti-PM-Scl	-0.30946045	1.74E-08
B62	B156	WBHSRVI	IgE	-0.30840065	1.96E-08
B62	B151	WBHSRVI	Anti-CCP	-0.30488052	2.89E-08
B62	B81	WBHSRVI	TSGF	0.270867887	9.43E-07
B63	B185	WBRSR(mPas_200)	PT	-0.45548261	0
B63	B64	WBRSR(mPas_200)	WBRSR(mPas_5)	0.949475069	0
B63	B65	WBRSR(mPas_200)	WBRSR(mPas_1)	0.913976765	0
B63	B66	WBRSR(mPas_200)	WBRSR(mPas_100)	0.99861291	0
B63	B67	WBRSR(mPas_200)	WBRSR(mPas_50)	0.996425316	0
B63	B92	WBRSR(mPas_200)	ALDH2-Glu504Glu	-0.50859719	0
B63	B93	WBRSR(mPas_200)	ALDH2-Glu504Lys	0.529174643	0
B63	B184	WBRSR(mPas_200)	INR	-0.35989678	3.70E-11

B63	B69	WBRSR(mPas_200)	PV	0.338866835	5.52E-10
B63	B94	WBRSR(mPas_200)	ALDH2-Lys504Lys	-0.30274265	3.65E-08
B64	B65	WBRSR(mPas_5)	WBRSR(mPas_1)	0.984730042	0
B64	B66	WBRSR(mPas_5)	WBRSR(mPas_100)	0.954939113	0
B64	B67	WBRSR(mPas_5)	WBRSR(mPas_50)	0.968542421	0
B64	B69	WBRSR(mPas_5)	PV	0.452306682	0
B64	B92	WBRSR(mPas_5)	ALDH2-Glu504Glu	-0.49238576	0
B64	B93	WBRSR(mPas_5)	ALDH2-Glu504Lys	0.501674797	0
B64	B185	WBRSR(mPas_5)	PT	-0.36976331	9.71E-12
B65	B66	WBRSR(mPas_1)	WBRSR(mPas_100)	0.919015368	0
B65	B67	WBRSR(mPas_1)	WBRSR(mPas_50)	0.937672755	0
B65	B93	WBRSR(mPas_1)	ALDH2-Glu504Lys	0.420343261	4.88E-15
B65	B92	WBRSR(mPas_1)	ALDH2-Glu504Glu	-0.41298964	1.58E-14
B65	B69	WBRSR(mPas_1)	PV	0.410797816	2.22E-14
B65	B185	WBRSR(mPas_1)	PT	-0.3078096	2.09E-08
B66	B185	WBRSR(mPas_100)	PT	-0.45463781	0
B66	B67	WBRSR(mPas_100)	WBRSR(mPas_50)	0.99850823	0
B66	B92	WBRSR(mPas_100)	ALDH2-Glu504Glu	-0.51032169	0
B66	B93	WBRSR(mPas_100)	ALDH2-Glu504Lys	0.530620321	0
B66	B184	WBRSR(mPas_100)	INR	-0.36097927	3.20E-11
B66	B69	WBRSR(mPas_100)	PV	0.357887479	4.83E-11
B66	B94	WBRSR(mPas_100)	ALDH2-Lys504Lys	-0.29750912	6.40E-08
B67	B185	WBRSR(mPas_50)	PT	-0.44165738	0
B67	B92	WBRSR(mPas_50)	ALDH2-Glu504Glu	-0.50979087	0
B67	B93	WBRSR(mPas_50)	ALDH2-Glu504Lys	0.528155573	0
B67	B69	WBRSR(mPas_50)	PV	0.375601019	4.30E-12
B67	B184	WBRSR(mPas_50)	INR	-0.3355482	8.30E-10
B69	B123	PV	hs-CRP1	0.744715887	0
B69	B177	PV	ACR	0.474885813	0
B69	B181	PV	APTT	-0.44739694	0
B69	B186	PV	PFC	0.44573522	0
B69	B83	PV	AFP	0.382809593	1.54E-12
B69	B194	PV	Cl	-0.38129091	1.92E-12
B69	B157	PV	IgG	0.340155443	4.70E-10
B69	B175	PV	ALB-U	0.33261087	1.19E-09
B69	B178	PV	Ig-U	0.331914848	1.29E-09
B69	B179	PV	α1-MG	0.329419199	1.74E-09
B69	B79	PV	hb V -dna	0.327917634	2.08E-09
B69	B180	PV	Tf	0.30190306	3.99E-08
B69	B184	PV	INR	0.292347037	1.10E-07
B69	B155	PV	IgA	0.273204023	7.53E-07
B7	B137	WB-Cop	AFU	0.452850717	0
B7	B138	WB-Cop	PAB	0.52045028	0
B7	B16	WB-Cop	CD3+T	0.659409978	0

B7	B21	WB-Cop	CD3+CD4-CD8-	0.514334406	0
B7	B23	WB-Cop	CD3-CD16+CD56+(NK)	-0.62509392	0
B7	B76	WB-Cop	ApoB	0.443255337	0
B7	B8	WB-Cop	WB-Zinc	1	0
B7	B94	WB-Cop	ALDH2-Lys504Lys	-0.49762844	0
B7	B15	WB-Cop	CD3-CD19+(B cell)	-0.39394978	3.00E-13
B7	B179	WB-Cop	α1-MG	-0.38747121	7.82E-13
B7	B178	WB-Cop	Ig-U	-0.38072155	2.08E-12
B7	B57	WB-Cop	RBCAI	-0.3714923	7.64E-12
B7	B85	WB-Cop	SCC	-0.36823304	1.20E-11
B7	B92	WB-Cop	ALDH2-Glu504Glu	0.364342135	2.04E-11
B7	B75	WB-Cop	ApoA1/B	-0.35273649	9.50E-11
B7	B180	WB-Cop	Tf	-0.34443905	2.75E-10
B7	B93	WB-Cop	ALDH2-Glu504Lys	-0.33929588	5.23E-10
B7	B90	WB-Cop	CYFRA 21-1	0.312457974	1.25E-08
B7	B176	WB-Cop	Ucr	0.295282965	8.09E-08
B7	B200	WB-Cop	OP	0.291810316	1.16E-07
B7	B134	WB-Cop	CHE	0.29147904	1.21E-07
B7	B195	WB-Cop	Na	0.29052759	1.33E-07
B7	B198	WB-Cop	P	-0.27659081	5.42E-07
B70	B77	LDL-C	TC	0.755437877	0
B71	B136	TG	5'NT	0.443158291	0
B71	B72	TG	HDL-C	-0.47491012	0
B71	B150	TG	ALP	0.378885339	2.70E-12
B71	B77	TG	TC	0.367596049	1.31E-11
B71	B153	TG	Glu	0.359626941	3.84E-11
B71	B91	TG	TP	0.352810433	9.41E-11
B71	B140	TG	r-GT	0.343501692	3.10E-10
B71	B206	TG	Waist	0.302559475	3.72E-08
B71	B222	TG	NS	0.299361347	5.25E-08
B71	B215	TG	BMI	0.28916963	1.53E-07
B71	B216	TG	BMI	0.28368688	2.67E-07
B72	B206	HDL-C	Waist	-0.47757782	0
B72	B216	HDL-C	BMI	-0.51852346	0
B72	B222	HDL-C	NS	-0.50554224	0
B72	B215	HDL-C	BMI	-0.42961007	8.88E-16
B72	B192	HDL-C	UA	-0.40379493	6.68E-14
B72	B221	HDL-C	Hei	-0.35641631	5.87E-11
B72	B210	HDL-C	Hip	-0.3411968	4.13E-10
B72	B227	HDL-C	OD	0.281476334	3.34E-07
B72	B141	HDL-C	AST/ALT	0.278233133	4.61E-07
B73	B124	ApoA	ACTH	0.562557594	0
B73	B76	ApoA	ApoB	0.282507549	3.01E-07
B74	B75	ApoA1	ApoA1/B	0.547071489	0

B74	B198	ApoA1	P	0.29589142	7.59E-08
B75	B76	ApoA1/B	ApoB	-0.71059925	0
B75	B134	ApoA1/B	CHE	-0.42764818	1.33E-15
B75	B135	ApoA1/B	TBA	0.341571462	3.94E-10
B75	B124	ApoA1/B	ACTH	-0.3282914	1.99E-09
B76	B124	ApoB	ACTH	0.425023673	2.22E-15
B76	B134	ApoB	CHE	0.39705872	1.88E-13
B76	B85	ApoB	SCC	-0.33870682	5.63E-10
B76	B138	ApoB	PAB	0.330205465	1.59E-09
B77	B150	TC	ALP	0.309442637	1.75E-08
B77	B136	TC	5'NT	0.298928159	5.50E-08
B77	B91	TC	TP	0.276405283	5.51E-07
B77	B153	TC	Glu	0.272670836	7.93E-07
B79	B100	hb V -dna	anti-Ro52	0.725623807	0
B79	B101	hb V -dna	anti-Sc70	0.725623808	0
B79	B102	hb V -dna	anti-Sm	0.725623808	0
B79	B103	hb V -dna	anti-SSA/Ro	0.725623807	0
B79	B104	hb V -dna	anti-SSB	0.725623808	0
B79	B105	hb V -dna	anti-U1RNP	0.725623807	0
B79	B106	hb V -dna	anti-P	0.725623807	0
B79	B107	hb V -dna	AnuA	0.725623799	0
B79	B108	hb V -dna	Anti-Cyclin	0.725623807	0
B79	B109	hb V -dna	AMA-M2	0.725623807	0
B79	B110	hb V -dna	AHA	0.725623807	0
B79	B119	hb V -dna	Hcv-rna	0.629446092	0
B79	B121	hb V -dna	C3	-0.62944611	0
B79	B122	hb V -dna	C4	-0.62944631	0
B79	B151	hb V -dna	Anti-CCP	0.549392706	0
B79	B152	hb V -dna	ACA-Ig(G-A-M)	-0.62944776	0
B79	B156	hb V -dna	IgE	0.541949567	0
B79	B157	hb V -dna	IgG	0.440503925	0
B79	B159	hb V -dna	β2-MG	-0.62944684	0
B79	B169	hb V -dna	U-CRY	-0.62944542	0
B79	B178	hb V -dna	Ig-U	0.498776722	0
B79	B180	hb V -dna	Tf	0.456474597	0
B79	B184	hb V -dna	INR	0.573640307	0
B79	B95	hb V -dna	anti-cenpb	0.725623808	0
B79	B97	hb V -dna	dsDNA	0.725623806	0
B79	B98	hb V -dna	anti-Jo-1	0.725623806	0
B79	B99	hb V -dna	anti-PM-Scl	0.725623806	0
B79	B94	hb V -dna	ALDH2-Lys504Lys	0.439368322	2.22E-16
B79	B155	hb V -dna	IgA	0.36157392	2.96E-11
B79	B179	hb V -dna	α1-MG	0.352195286	1.02E-10
B79	B81	hb V -dna	TSGF	-0.35064557	1.25E-10

B79	B158	hb V -dna	IgM	-0.31427123	1.02E-08
B79	B185	hb V -dna	PT	0.313267756	1.14E-08
B79	B183	hb V -dna	PTA	0.306927622	2.31E-08
B79	B181	hb V -dna	APTT	-0.30643365	2.44E-08
B8	B137	WB-Zinc	AFU	0.452850717	0
B8	B138	WB-Zinc	PAB	0.52045028	0
B8	B16	WB-Zinc	CD3+T	0.659409978	0
B8	B21	WB-Zinc	CD3+CD4-CD8-	0.514334406	0
B8	B23	WB-Zinc	CD3-CD16+CD56+(NK)	-0.62509392	0
B8	B76	WB-Zinc	ApoB	0.443255337	0
B8	B94	WB-Zinc	ALDH2-Lys504Lys	-0.49762845	0
B8	B15	WB-Zinc	CD3-CD19+(B cell)	-0.39394978	3.00E-13
B8	B179	WB-Zinc	α1-MG	-0.38747121	7.82E-13
B8	B178	WB-Zinc	Ig-U	-0.38072155	2.08E-12
B8	B57	WB-Zinc	RBCAI	-0.3714923	7.64E-12
B8	B85	WB-Zinc	SCC	-0.36823304	1.20E-11
B8	B92	WB-Zinc	ALDH2-Glu504Glu	0.364342135	2.04E-11
B8	B75	WB-Zinc	ApoA1/B	-0.35273649	9.50E-11
B8	B180	WB-Zinc	Tf	-0.34443905	2.75E-10
B8	B93	WB-Zinc	ALDH2-Glu504Lys	-0.33929588	5.23E-10
B8	B90	WB-Zinc	CYFRA 21-1	0.312457974	1.25E-08
B8	B176	WB-Zinc	Ucr	0.295282965	8.09E-08
B8	B200	WB-Zinc	OP	0.291810316	1.16E-07
B8	B134	WB-Zinc	CHE	0.29147904	1.21E-07
B8	B195	WB-Zinc	Na	0.29052759	1.33E-07
B8	B198	WB-Zinc	P	-0.27659081	5.42E-07
B80	B227	anti-HP IgG	OD	0.322921151	3.76E-09
B81	B100	TSGF	anti-Ro52	-0.43125203	8.88E-16
B81	B101	TSGF	anti-Sc70	-0.43125203	8.88E-16
B81	B102	TSGF	anti-Sm	-0.43125203	8.88E-16
B81	B103	TSGF	anti-SSA/Ro	-0.43125203	8.88E-16
B81	B104	TSGF	anti-SSB	-0.43125203	8.88E-16
B81	B105	TSGF	anti-U1RNP	-0.43125203	8.88E-16
B81	B106	TSGF	anti-P	-0.43125203	8.88E-16
B81	B107	TSGF	AnuA	-0.43125203	8.88E-16
B81	B108	TSGF	Anti-Cyclin	-0.43125203	8.88E-16
B81	B109	TSGF	AMA-M2	-0.43125203	8.88E-16
B81	B110	TSGF	AHA	-0.43125203	8.88E-16
B81	B95	TSGF	anti-cenpb	-0.43125203	8.88E-16
B81	B97	TSGF	dsDNA	-0.43125203	8.88E-16
B81	B98	TSGF	anti-Jo-1	-0.43125203	8.88E-16
B81	B99	TSGF	anti-PM-Scl	-0.43125203	8.88E-16
B81	B180	TSGF	Tf	-0.34985053	1.38E-10
B81	B184	TSGF	INR	-0.34630417	2.17E-10

B81	B178	TSGF	Ig-U	-0.34367174	3.03E-10
B81	B151	TSGF	Anti-CCP	-0.32207176	4.15E-09
B81	B158	TSGF	IgM	0.315768681	8.58E-09
B81	B94	TSGF	ALDH2-Lys504Lys	-0.29245471	1.09E-07
B81	B157	TSGF	IgG	-0.2706861	9.59E-07
B82	B88	CEA	CA 242	0.460104663	0
B82	B200	CEA	OP	0.433875678	4.44E-16
B82	B87	CEA	NSE	-0.32878394	1.88E-09
B82	B195	CEA	Na	0.29463942	8.66E-08
B83	B123	AFP	hs-CRP1	0.452510401	0
B83	B181	AFP	APTT	-0.53464925	0
B83	B186	AFP	PFC	0.775203324	0
B83	B193	AFP	K	-0.59387799	0
B83	B194	AFP	Cl	-0.68821901	0
B83	B199	AFP	Mg	-0.65113069	0
B83	B197	AFP	AG	0.412532719	1.69E-14
B83	B89	AFP	CA 72-4	-0.40780974	3.57E-14
B83	B100	AFP	anti-Ro52	-0.37696214	3.55E-12
B83	B101	AFP	anti-Sc70	-0.37696214	3.55E-12
B83	B102	AFP	anti-Sm	-0.37696214	3.55E-12
B83	B103	AFP	anti-SSA/Ro	-0.37696214	3.55E-12
B83	B104	AFP	anti-SSB	-0.37696214	3.55E-12
B83	B105	AFP	anti-U1RNP	-0.37696214	3.55E-12
B83	B106	AFP	anti-P	-0.37696214	3.55E-12
B83	B107	AFP	AnuA	-0.37696214	3.55E-12
B83	B108	AFP	Anti-Cyclin	-0.37696214	3.55E-12
B83	B109	AFP	AMA-M2	-0.37696214	3.55E-12
B83	B110	AFP	AHA	-0.37696214	3.55E-12
B83	B95	AFP	anti-cenpb	-0.37696214	3.55E-12
B83	B97	AFP	dsDNA	-0.37696214	3.55E-12
B83	B98	AFP	anti-Jo-1	-0.37696214	3.55E-12
B83	B99	AFP	anti-PM-Scl	-0.37696214	3.55E-12
B83	B151	AFP	Anti-CCP	-0.3695418	1.00E-11
B83	B86	AFP	TBhCG	-0.34216834	3.66E-10
B84	B85	C-PSA	SCC	0.476247364	0
B84	B86	C-PSA	TBhCG	0.682517908	0
B84	B88	C-PSA	CA 242	-0.46121772	0
B84	B89	C-PSA	CA 72-4	0.811662665	0
B84	B186	C-PSA	PFC	-0.38735061	7.96E-13
B84	B194	C-PSA	Cl	0.340332318	4.60E-10
B84	B199	C-PSA	Mg	0.319516186	5.58E-09
B84	B193	C-PSA	K	0.277510201	4.95E-07
B85	B186	SCC	PFC	-0.34642373	2.14E-10
B85	B87	SCC	NSE	0.328513462	1.94E-09

B85	B138	SCC	PAB	-0.29993395	4.94E-08
B85	B200	SCC	OP	-0.29597409	7.52E-08
B86	B89	TBhCG	CA 72-4	0.887323378	0
B86	B88	TBhCG	CA 242	-0.34742837	1.88E-10
B86	B186	TBhCG	PFC	-0.31065351	1.53E-08
B86	B193	TBhCG	K	0.301843309	4.02E-08
B86	B87	TBhCG	NSE	-0.2920283	1.14E-07
B86	B194	TBhCG	Cl	0.281758987	3.25E-07
B87	B89	NSE	CA 72-4	-0.49080507	0
B87	B161	NSE	LEU	0.362926624	2.47E-11
B88	B111	CA 242	TSH	0.382893533	1.52E-12
B88	B89	CA 242	CA 72-4	-0.38272012	1.56E-12
B89	B186	CA 72-4	PFC	-0.43003128	8.88E-16
B89	B194	CA 72-4	Cl	0.377360764	3.36E-12
B89	B199	CA 72-4	Mg	0.3532433	8.89E-11
B89	B193	CA 72-4	K	0.306669234	2.37E-08
B89	B181	CA 72-4	APTT	0.298815402	5.56E-08
B9	B38	RH	BAND%	-0.50251891	0
B90	B100	CYFRA 21-1	anti-Ro52	-0.38294936	1.51E-12
B90	B101	CYFRA 21-1	anti-Sc70	-0.38294936	1.51E-12
B90	B102	CYFRA 21-1	anti-Sm	-0.38294936	1.51E-12
B90	B103	CYFRA 21-1	anti-SSA/Ro	-0.38294936	1.51E-12
B90	B104	CYFRA 21-1	anti-SSB	-0.38294936	1.51E-12
B90	B105	CYFRA 21-1	anti-U1RNP	-0.38294936	1.51E-12
B90	B106	CYFRA 21-1	anti-P	-0.38294936	1.51E-12
B90	B107	CYFRA 21-1	AnuA	-0.38294936	1.51E-12
B90	B108	CYFRA 21-1	Anti-Cyclin	-0.38294936	1.51E-12
B90	B109	CYFRA 21-1	AMA-M2	-0.38294936	1.51E-12
B90	B110	CYFRA 21-1	AHA	-0.38294936	1.51E-12
B90	B95	CYFRA 21-1	anti-cenpb	-0.38294936	1.51E-12
B90	B97	CYFRA 21-1	dsDNA	-0.38294936	1.51E-12
B90	B98	CYFRA 21-1	anti-Jo-1	-0.38294936	1.51E-12
B90	B99	CYFRA 21-1	anti-PM-Scl	-0.38294936	1.51E-12
B90	B178	CYFRA 21-1	Ig-U	-0.36222547	2.71E-11
B90	B180	CYFRA 21-1	Tf	-0.36213819	2.74E-11
B90	B179	CYFRA 21-1	α1-MG	-0.35593868	6.25E-11
B90	B134	CYFRA 21-1	CHE	0.354298641	7.75E-11
B90	B199	CYFRA 21-1	Mg	-0.30469114	2.95E-08
B90	B154	CYFRA 21-1	RF	-0.28547013	2.23E-07
B90	B151	CYFRA 21-1	Anti-CCP	-0.28382542	2.64E-07
B91	B126	TP	Alb	0.536914334	0
B91	B127	TP	A-G ratio	-0.49207434	0
B91	B130	TP	GLB	0.797848216	0
B91	B188	TP	Ca	0.331280484	1.39E-09

B92	B184	ALDH2-Glu504Glu	INR	0.443743864	0
B92	B185	ALDH2-Glu504Glu	PT	0.635909382	0
B92	B93	ALDH2-Glu504Glu	ALDH2-Glu504Lys	-0.99848738	0
B92	B124	ALDH2-Glu504Glu	ACTH	-0.37261755	6.54E-12
B92	B196	ALDH2-Glu504Glu	TCO2	-0.28557816	2.21E-07
B92	B137	ALDH2-Glu504Glu	AFU	0.285309647	2.27E-07
B93	B184	ALDH2-Glu504Lys	INR	-0.47172352	0
B93	B185	ALDH2-Glu504Lys	PT	-0.65556721	0
B93	B124	ALDH2-Glu504Lys	ACTH	0.365312443	1.79E-11
B93	B196	ALDH2-Glu504Lys	TCO2	0.284593763	2.44E-07
B93	B137	ALDH2-Glu504Lys	AFU	-0.27710825	5.15E-07
B94	B178	ALDH2-Lys504Lys	Ig-U	0.561320416	0
B94	B180	ALDH2-Lys504Lys	Tf	0.514330776	0
B94	B184	ALDH2-Lys504Lys	INR	0.444188575	0
B94	B100	ALDH2-Lys504Lys	anti-Ro52	0.399120174	1.37E-13
B94	B101	ALDH2-Lys504Lys	anti-Sc70	0.399120174	1.37E-13
B94	B102	ALDH2-Lys504Lys	anti-Sm	0.399120174	1.37E-13
B94	B103	ALDH2-Lys504Lys	anti-SSA/Ro	0.399120174	1.37E-13
B94	B104	ALDH2-Lys504Lys	anti-SSB	0.399120176	1.37E-13
B94	B105	ALDH2-Lys504Lys	anti-U1RNP	0.399120174	1.37E-13
B94	B106	ALDH2-Lys504Lys	anti-P	0.399120174	1.37E-13
B94	B107	ALDH2-Lys504Lys	AnuA	0.399120178	1.37E-13
B94	B108	ALDH2-Lys504Lys	Anti-Cyclin	0.399120175	1.37E-13
B94	B109	ALDH2-Lys504Lys	AMA-M2	0.399120173	1.37E-13
B94	B110	ALDH2-Lys504Lys	AHA	0.399120173	1.37E-13
B94	B95	ALDH2-Lys504Lys	anti-cenpb	0.399120174	1.37E-13
B94	B97	ALDH2-Lys504Lys	dsDNA	0.399120174	1.37E-13
B94	B98	ALDH2-Lys504Lys	anti-Jo-1	0.399120174	1.37E-13
B94	B99	ALDH2-Lys504Lys	anti-PM-Scl	0.399120175	1.37E-13
B94	B156	ALDH2-Lys504Lys	IgE	0.388677474	6.55E-13
B94	B151	ALDH2-Lys504Lys	Anti-CCP	0.388283322	6.95E-13
B94	B179	ALDH2-Lys504Lys	α1-MG	0.351375372	1.13E-10
B94	B204	ALDH2-Lys504Lys	Drinking	-0.31798911	6.65E-09
B94	B181	ALDH2-Lys504Lys	APTT	-0.31639598	7.98E-09
B94	B154	ALDH2-Lys504Lys	RF	0.304899056	2.88E-08
B94	B125	ALDH2-Lys504Lys	Cor	0.296747287	6.93E-08
B94	B198	ALDH2-Lys504Lys	P	0.296433808	7.17E-08
B95	B101	anti-cenpb	anti-Sc70	0.999999999	0
B95	B106	anti-cenpb	anti-P	0.999999999	0
B95	B107	anti-cenpb	AnuA	0.99999999	0
B95	B108	anti-cenpb	Anti-Cyclin	1	0
B95	B109	anti-cenpb	AMA-M2	1	0
B95	B151	anti-cenpb	Anti-CCP	0.665704468	0
B95	B156	anti-cenpb	IgE	0.614367186	0

B95	B178	anti-cenpb	Ig-U	0.524633852	0
B95	B180	anti-cenpb	Tf	0.531153079	0
B95	B184	anti-cenpb	INR	0.452826163	0
B95	B193	anti-cenpb	K	0.459514229	0
B95	B98	anti-cenpb	anti-Jo-1	0.999999999	0
B95	B154	anti-cenpb	RF	0.419728548	5.33E-15
B95	B199	anti-cenpb	Mg	0.402787859	7.82E-14
B95	B179	anti-cenpb	α1-MG	0.397881945	1.66E-13
B95	B183	anti-cenpb	PTA	0.375742668	4.22E-12
B95	B157	anti-cenpb	IgG	0.340998994	4.24E-10
B95	B158	anti-cenpb	IgM	-0.28134467	3.38E-07
B95	B119	anti-cenpb	Hcv-rna	0.281326061	3.39E-07
B95	B121	anti-cenpb	C3	-0.28132607	3.39E-07
B95	B122	anti-cenpb	C4	-0.2813262	3.39E-07
B95	B152	anti-cenpb	ACA-Ig(G-A-M)	-0.28132685	3.39E-07
B95	B159	anti-cenpb	β2-MG	-0.28132641	3.39E-07
B95	B169	anti-cenpb	U-CRY	-0.28132578	3.39E-07
B96	B167	dsDNA (IIF)	PC	0.948223744	0
B97	B100	dsDNA	anti-Ro52	0.999999999	0
B97	B103	dsDNA	anti-SSA/Ro	0.999999999	0
B97	B105	dsDNA	anti-U1RNP	0.999999999	0
B97	B108	dsDNA	Anti-Cyclin	0.999999999	0
B97	B109	dsDNA	AMA-M2	0.999999999	0
B97	B110	dsDNA	AHA	0.999999999	0
B97	B151	dsDNA	Anti-CCP	0.665704467	0
B97	B156	dsDNA	IgE	0.614367185	0
B97	B178	dsDNA	Ig-U	0.524633851	0
B97	B180	dsDNA	Tf	0.531153078	0
B97	B184	dsDNA	INR	0.452826162	0
B97	B193	dsDNA	K	0.459514228	0
B97	B99	dsDNA	anti-PM-Scl	1	0
B97	B154	dsDNA	RF	0.419728547	5.33E-15
B97	B199	dsDNA	Mg	0.402787859	7.82E-14
B97	B179	dsDNA	α1-MG	0.397881945	1.66E-13
B97	B183	dsDNA	PTA	0.375742667	4.22E-12
B97	B157	dsDNA	IgG	0.340998994	4.24E-10
B97	B158	dsDNA	IgM	-0.28134467	3.38E-07
B97	B119	dsDNA	Hcv-rna	0.281326061	3.39E-07
B97	B121	dsDNA	C3	-0.28132606	3.39E-07
B97	B122	dsDNA	C4	-0.28132615	3.39E-07
B97	B152	dsDNA	ACA-Ig(G-A-M)	-0.28132666	3.39E-07
B97	B159	dsDNA	β2-MG	-0.28132637	3.39E-07
B97	B169	dsDNA	U-CRY	-0.2813257	3.39E-07
B98	B100	anti-Jo-1	anti-Ro52	0.999999999	0

B98	B101	anti-Jo-1	anti-Sc70	0.999999998	0
B98	B102	anti-Jo-1	anti-Sm	0.999999999	0
B98	B103	anti-Jo-1	anti-SSA/Ro	0.999999999	0
B98	B104	anti-Jo-1	anti-SSB	0.999999999	0
B98	B105	anti-Jo-1	anti-U1RNP	0.999999999	0
B98	B107	anti-Jo-1	AnuA	0.999999995	0
B98	B109	anti-Jo-1	AMA-M2	0.999999997	0
B98	B110	anti-Jo-1	AHA	0.999999999	0
B98	B151	anti-Jo-1	Anti-CCP	0.665704467	0
B98	B156	anti-Jo-1	IgE	0.614367185	0
B98	B178	anti-Jo-1	Ig-U	0.524633851	0
B98	B180	anti-Jo-1	Tf	0.531153078	0
B98	B184	anti-Jo-1	INR	0.452826162	0
B98	B193	anti-Jo-1	K	0.459514228	0
B98	B99	anti-Jo-1	anti-PM-Scl	1	0
B98	B154	anti-Jo-1	RF	0.419728547	5.33E-15
B98	B199	anti-Jo-1	Mg	0.402787859	7.82E-14
B98	B179	anti-Jo-1	α1-MG	0.397881945	1.66E-13
B98	B183	anti-Jo-1	PTA	0.375742667	4.22E-12
B98	B157	anti-Jo-1	IgG	0.340998993	4.24E-10
B98	B158	anti-Jo-1	IgM	-0.28134467	3.38E-07
B98	B119	anti-Jo-1	Hcv-rna	0.28132606	3.39E-07
B98	B121	anti-Jo-1	C3	-0.28132608	3.39E-07
B98	B122	anti-Jo-1	C4	-0.28132621	3.39E-07
B98	B152	anti-Jo-1	ACA-Ig(G-A-M)	-0.28132689	3.39E-07
B98	B159	anti-Jo-1	β2-MG	-0.28132646	3.39E-07
B98	B169	anti-Jo-1	U-CRY	-0.28132575	3.39E-07
B99	B100	anti-PM-Scl	anti-Ro52	0.999999999	0
B99	B103	anti-PM-Scl	anti-SSA/Ro	0.999999999	0
B99	B105	anti-PM-Scl	anti-U1RNP	0.999999999	0
B99	B107	anti-PM-Scl	AnuA	0.99999999	0
B99	B109	anti-PM-Scl	AMA-M2	0.999999999	0
B99	B110	anti-PM-Scl	AHA	1	0
B99	B151	anti-PM-Scl	Anti-CCP	0.665704467	0
B99	B156	anti-PM-Scl	IgE	0.614367185	0
B99	B178	anti-PM-Scl	Ig-U	0.524633851	0
B99	B180	anti-PM-Scl	Tf	0.531153078	0
B99	B184	anti-PM-Scl	INR	0.452826162	0
B99	B193	anti-PM-Scl	K	0.459514228	0
B99	B154	anti-PM-Scl	RF	0.419728547	5.33E-15
B99	B199	anti-PM-Scl	Mg	0.402787859	7.82E-14
B99	B179	anti-PM-Scl	α1-MG	0.397881945	1.66E-13
B99	B183	anti-PM-Scl	PTA	0.375742667	4.22E-12
B99	B157	anti-PM-Scl	IgG	0.340998993	4.24E-10

B99	B158	anti-PM-Scl	IgM	-0.28134467	3.38E-07
B99	B119	anti-PM-Scl	Hcv-rna	0.28132606	3.39E-07
B99	B121	anti-PM-Scl	C3	-0.28132607	3.39E-07
B99	B122	anti-PM-Scl	C4	-0.28132615	3.39E-07
B99	B152	anti-PM-Scl	ACA-Ig(G-A-M)	-0.28132668	3.39E-07
B99	B159	anti-PM-Scl	β2-MG	-0.28132635	3.39E-07
B99	B169	anti-PM-Scl	U-CRY	-0.28132572	3.39E-07

Supplementary Table 14. PEI Correlations in Rheumatoid arthritis

Row	Column	Row	Column	Cor	*P*
B1	B190	Gender	Cre	-0.55999211	0
B1	B192	Gender	UA	-0.50031825	0
B1	B206	Gender	Waist	-0.42215999	0
B1	B207	Gender	WHR	-0.44331733	0
B1	B209	Gender	Smoking	-0.51914003	0
B1	B216	Gender	BMI	-0.44426835	0
B1	B221	Gender	Hei	-0.46529749	0
B1	B222	Gender	NS	-0.63605267	0
B1	B51	Gender	Hb	-0.45575253	0
B1	B72	Gender	HDL-C	0.409170049	0
B1	B46	Gender	RBC Count	-0.39632222	2.22E-16
B1	B163	Gender	SG	-0.36107627	1.22E-13
B1	B174	Gender	Urea/Cr	0.320705999	6.35E-11
B1	B204	Gender	Drinking	-0.30083701	9.98E-10
B1	B40	Gender	WBC	-0.28584033	6.98E-09
B1	B2	Gender	Age	-0.26007114	1.52E-07
B1	B42	Gender	MON	-0.24437571	8.55E-07
B10	B14	PG I	G-17	0.472619969	0
B10	B12	PG I	PG II	0.352387385	5.08E-13
B10	B191	PG I	Urea	0.251331733	4.04E-07
B100	B120	anti-Ro52	HCV-cAg	-0.40546819	0
B100	B204	anti-Ro52	Drinking	0.359976665	1.47E-13
B100	B224	anti-Ro52	pmh	0.308194415	3.69E-10
B100	B103	anti-Ro52	anti-SSA/Ro	0.294488569	2.30E-09
B100	B101	anti-Ro52	anti-Sc70	-0.24927397	5.05E-07
B100	B102	anti-Ro52	anti-Sm	-0.24927397	5.05E-07
B100	B105	anti-Ro52	anti-U1RNP	-0.24927397	5.05E-07
B100	B106	anti-Ro52	anti-P	-0.24927397	5.05E-07
B100	B107	anti-Ro52	AnuA	-0.24927397	5.05E-07
B100	B108	anti-Ro52	Anti-Cyclin	-0.24927397	5.05E-07
B100	B109	anti-Ro52	AMA-M2	-0.24927397	5.05E-07
B100	B110	anti-Ro52	AHA	-0.24927397	5.05E-07
B101	B102	anti-Sc70	anti-Sm	0.999999999	0
B101	B103	anti-Sc70	anti-SSA/Ro	-0.67410508	0
B101	B106	anti-Sc70	anti-P	1	0
B101	B107	anti-Sc70	AnuA	0.999999967	0
B101	B121	anti-Sc70	C3	0.329995431	1.63E-11
B101	B122	anti-Sc70	C4	0.329969907	1.64E-11
B101	B104	anti-Sc70	anti-SSB	-0.31611978	1.22E-10
B101	B155	anti-Sc70	IgA	0.2516837	3.88E-07
B101	B157	anti-Sc70	IgG	-0.25117744	4.11E-07
B102	B103	anti-Sm	anti-SSA/Ro	-0.67410508	0

B102	B108	anti-Sm	Anti-Cyclin	1	0
B102	B121	anti-Sm	C3	0.329995431	1.63E-11
B102	B122	anti-Sm	C4	0.329969907	1.64E-11
B102	B104	anti-Sm	anti-SSB	-0.31611978	1.22E-10
B102	B155	anti-Sm	IgA	0.251683699	3.88E-07
B102	B157	anti-Sm	IgG	-0.25117744	4.11E-07
B103	B104	anti-SSA/Ro	anti-SSB	0.52279203	0
B103	B105	anti-SSA/Ro	anti-U1RNP	-0.67410508	0
B103	B106	anti-SSA/Ro	anti-P	-0.67410508	0
B103	B107	anti-SSA/Ro	AnuA	-0.67410508	0
B103	B108	anti-SSA/Ro	Anti-Cyclin	-0.67410508	0
B103	B109	anti-SSA/Ro	AMA-M2	-0.67410508	0
B103	B110	anti-SSA/Ro	AHA	-0.67410508	0
B103	B121	anti-SSA/Ro	C3	-0.53057894	0
B103	B122	anti-SSA/Ro	C4	-0.53058105	0
B103	B157	anti-SSA/Ro	IgG	0.320995151	6.09E-11
B104	B105	anti-SSB	anti-U1RNP	-0.31611978	1.22E-10
B104	B106	anti-SSB	anti-P	-0.31611978	1.22E-10
B104	B107	anti-SSB	AnuA	-0.31611979	1.22E-10
B104	B108	anti-SSB	Anti-Cyclin	-0.31611978	1.22E-10
B104	B109	anti-SSB	AMA-M2	-0.31611978	1.22E-10
B104	B110	anti-SSB	AHA	-0.31611978	1.22E-10
B104	B173	anti-SSB	NTT	0.313147017	1.86E-10
B105	B106	anti-U1RNP	anti-P	0.999999999	0
B105	B108	anti-U1RNP	Anti-Cyclin	1	0
B105	B109	anti-U1RNP	AMA-M2	1	0
B105	B110	anti-U1RNP	AHA	1	0
B105	B121	anti-U1RNP	C3	0.32999543	1.63E-11
B105	B122	anti-U1RNP	C4	0.329969907	1.64E-11
B105	B155	anti-U1RNP	IgA	0.251683699	3.88E-07
B105	B157	anti-U1RNP	IgG	-0.25117744	4.11E-07
B106	B107	anti-P	AnuA	0.999999993	0
B106	B108	anti-P	Anti-Cyclin	1	0
B106	B109	anti-P	AMA-M2	1	0
B106	B110	anti-P	AHA	0.999999999	0
B106	B121	anti-P	C3	0.32999543	1.63E-11
B106	B122	anti-P	C4	0.329969907	1.64E-11
B106	B155	anti-P	IgA	0.251683699	3.88E-07
B106	B157	anti-P	IgG	-0.25117744	4.11E-07
B107	B121	AnuA	C3	0.329995432	1.63E-11
B107	B122	AnuA	C4	0.329969906	1.64E-11
B107	B155	AnuA	IgA	0.251683699	3.88E-07
B107	B157	AnuA	IgG	-0.25117744	4.11E-07
B108	B110	Anti-Cyclin	AHA	1	0

B108	B121	Anti-Cyclin	C3	0.32999543	1.63E-11
B108	B122	Anti-Cyclin	C4	0.329969907	1.64E-11
B108	B155	Anti-Cyclin	IgA	0.251683699	3.88E-07
B108	B157	Anti-Cyclin	IgG	-0.25117744	4.11E-07
B109	B121	AMA-M2	C3	0.32999543	1.63E-11
B109	B122	AMA-M2	C4	0.329969907	1.64E-11
B109	B155	AMA-M2	IgA	0.251683699	3.88E-07
B109	B157	AMA-M2	IgG	-0.25117744	4.11E-07
B11	B12	PGI/PGII	PG II	-0.56076253	0
B11	B14	PGI/PGII	G-17	-0.31267385	1.98E-10
B11	B149	PGI/PGII	TGAb	-0.255659	2.50E-07
B110	B121	AHA	C3	0.32999543	1.63E-11
B110	B122	AHA	C4	0.329969907	1.64E-11
B110	B155	AHA	IgA	0.251683699	3.88E-07
B110	B157	AHA	IgG	-0.25117744	4.11E-07
B112	B114	FT4	TSH TT3 TT4(TT4)	0.373660874	1.44E-14
B113	B115	FT3	TSH TT3 TT4(TT3)	0.527283159	0
B113	B134	FT3	CHE	-0.28482608	7.93E-09
B113	B196	FT3	TCO2	-0.26216811	1.20E-07
B114	B198	TSH TT3 TT4(TT4)	P	-0.50617096	0
B114	B115	TSH TT3 TT4(TT4)	TSH TT3 TT4(TT3)	0.302610103	7.87E-10
B114	B194	TSH TT3 TT4(TT4)	Cl	0.246790828	6.60E-07
B115	B134	TSH TT3 TT4(TT3)	CHE	-0.38636903	1.55E-15
B115	B196	TSH TT3 TT4(TT3)	TCO2	-0.37464821	1.20E-14
B115	B200	TSH TT3 TT4(TT3)	OP	0.324210269	3.82E-11
B115	B138	TSH TT3 TT4(TT3)	PAB	-0.269816	4.93E-08
B116	B117	TT3	TT4	-0.52774674	0
B119	B120	Hcv-rna	HCV-cAg	0.405656033	0
B119	B124	Hcv-rna	ACTH	0.635038757	0
B119	B169	Hcv-rna	U-CRY	-0.99999452	0
B119	B179	Hcv-rna	α1-MG	0.413808817	0
B119	B198	Hcv-rna	P	-0.28987342	4.18E-09
B119	B150	Hcv-rna	ALP	-0.2452647	7.77E-07
B12	B14	PG II	G-17	0.412678199	0
B12	B149	PG II	TGAb	0.352155232	5.27E-13
B120	B152	HCV-cAg	ACA-Ig(G-A-M)	-0.40566747	0
B120	B159	HCV-cAg	β2-MG	-0.40565655	0
B120	B169	HCV-cAg	U-CRY	-0.40565295	0
B120	B224	HCV-cAg	pmh	-0.28144004	1.21E-08
B120	B139	HCV-cAg	ADA	-0.27077664	4.40E-08
B120	B124	HCV-cAg	ACTH	0.261410263	1.31E-07
B121	B122	C3	C4	0.99998187	0
B121	B158	C3	IgM	0.346244154	1.35E-12
B121	B205	C3	KDA	-0.29766287	1.52E-09

B122	B158	C4	IgM	0.34629298	1.34E-12
B122	B205	C4	KDA	-0.29757726	1.54E-09
B123	B184	hs-CRP1	INR	-0.39849014	0
B123	B186	hs-CRP1	PFC	0.608178075	0
B123	B150	hs-CRP1	ALP	0.348181955	9.95E-13
B123	B156	hs-CRP1	IgE	0.336060521	6.55E-12
B123	B125	hs-CRP1	Cor	-0.32273278	4.74E-11
B123	B179	hs-CRP1	α1-MG	-0.30907698	3.26E-10
B123	B157	hs-CRP1	IgG	0.284074071	8.71E-09
B123	B181	hs-CRP1	APTT	0.275773746	2.42E-08
B124	B125	ACTH	Cor	0.720676179	0
B124	B152	ACTH	ACA-Ig(G-A-M)	-0.63506467	0
B124	B159	ACTH	β2-MG	-0.63504099	0
B124	B169	ACTH	U-CRY	-0.63503528	0
B124	B181	ACTH	APTT	0.459914164	0
B124	B198	ACTH	P	-0.40091199	0
B124	B194	ACTH	Cl	-0.38626289	1.55E-15
B124	B184	ACTH	INR	-0.36417276	7.28E-14
B124	B179	ACTH	α1-MG	0.317382298	1.02E-10
B124	B193	ACTH	K	-0.30289233	7.58E-10
B124	B186	ACTH	PFC	0.25856407	1.81E-07
B124	B200	ACTH	OP	-0.2544029	2.88E-07
B124	B197	ACTH	AG	0.248827415	5.30E-07
B124	B158	ACTH	IgM	0.244928772	8.06E-07
B125	B135	Cor	TBA	0.421042482	0
B125	B194	Cor	Cl	-0.4046803	0
B125	B181	Cor	APTT	0.347934134	1.03E-12
B125	B197	Cor	AG	0.340093677	3.53E-12
B125	B182	Cor	TT	0.332019545	1.21E-11
B125	B150	Cor	ALP	-0.30968441	3.00E-10
B125	B179	Cor	α1-MG	0.284717588	8.04E-09
B125	B157	Cor	IgG	-0.28079399	1.31E-08
B125	B199	Cor	Mg	0.27373739	3.09E-08
B125	B156	Cor	IgE	-0.26598509	7.73E-08
B125	B198	Cor	P	-0.25927097	1.67E-07
B126	B127	Alb	A-G ratio	0.682301807	0
B126	B130	Alb	GLB	-0.45441572	0
B126	B188	Alb	Ca	0.509760393	0
B127	B130	A-G ratio	GLB	-0.92384873	0
B128	B131	ALT	AST	0.760359693	0
B128	B140	ALT	r-GT	0.498060348	0
B128	B141	ALT	AST/ALT	-0.60651243	0
B129	B132	IBIL	Dbil	0.656486907	0
B129	B133	IBIL	TBIL	0.976739304	0

B131	B140	AST	r-GT	0.382482716	3.11E-15
B132	B133	Dbil	TBIL	0.802818964	0
B134	B137	CHE	AFU	-0.45322181	0
B134	B138	CHE	PAB	0.56125778	0
B134	B199	CHE	Mg	0.394573486	4.44E-16
B134	B225	CHE	hPBG	0.33387409	9.12E-12
B134	B193	CHE	K	0.332965914	1.05E-11
B134	B136	CHE	5'NT	0.304357936	6.22E-10
B135	B139	TBA	ADA	0.40827134	0
B135	B194	TBA	Cl	-0.5196916	0
B135	B225	TBA	hPBG	-0.71502761	0
B135	B199	TBA	Mg	0.387811206	1.11E-15
B135	B138	TBA	PAB	-0.30689384	4.40E-10
B135	B193	TBA	K	-0.29575183	1.95E-09
B136	B197	5'NT	AG	0.460095273	0
B136	B193	5'NT	K	0.347352851	1.14E-12
B136	B195	5'NT	Na	0.337856724	4.98E-12
B136	B196	5'NT	TCO2	-0.2949023	2.18E-09
B136	B200	5'NT	OP	0.246873773	6.55E-07
B137	B200	AFU	OP	0.294808692	2.21E-09
B137	B225	AFU	hPBG	-0.28381012	9.00E-09
B138	B139	PAB	ADA	-0.52537711	0
B138	B225	PAB	hPBG	0.324885593	3.47E-11
B138	B155	PAB	IgA	0.244593766	8.35E-07
B139	B225	ADA	hPBG	-0.53197929	0
B139	B196	ADA	TCO2	0.285957264	6.88E-09
B140	B141	r-GT	AST/ALT	-0.26439342	9.29E-08
B141	B216	AST/ALT	BMI	-0.26432881	9.36E-08
B141	B215	AST/ALT	BMI	-0.24831923	5.60E-07
B142	B143	25-(OH)D	β-CTx	0.303805838	6.70E-10
B143	B144	β-CTx	Total-PINP	0.792209477	0
B143	B145	β-CTx	BGP	0.549546646	0
B143	B151	β-CTx	Anti-CCP	0.299942955	1.12E-09
B144	B145	Total-PINP	BGP	0.682277615	0
B144	B151	Total-PINP	Anti-CCP	0.248161398	5.70E-07
B145	B199	BGP	Mg	0.313957594	1.66E-10
B145	B195	BGP	Na	0.264662274	9.01E-08
B146	B154	CysC	RF	0.471073182	0
B146	B190	CysC	Cre	0.250445881	4.45E-07
B148	B149	Anti_TPO	TGAb	0.511349684	0
B15	B138	CD3-CD19+(B cell)	PAB	0.541531759	0
B15	B139	CD3-CD19+(B cell)	ADA	-0.40195918	0
B15	B16	CD3-CD19+(B cell)	CD3+T	0.838584399	0
B15	B17	CD3-CD19+(B cell)	CD3+CD16+CD56+NK	0.789619196	0

B15	B18	CD3-CD19+(B cell)	CD3+CD4+helper(inducer)T	-0.94804226	0
B15	B19	CD3-CD19+(B cell)	CD3+CD4+/CD3+CD8+	-0.99206548	0
B15	B21	CD3-CD19+(B cell)	CD3+CD4-CD8-	0.982074448	0
B15	B22	CD3-CD19+(B cell)	CD3+CD8+(T)	0.949664592	0
B15	B23	CD3-CD19+(B cell)	CD3-CD16+CD56+(NK)	-0.99578801	0
B15	B194	CD3-CD19+(B cell)	Cl	-0.36052421	1.34E-13
B15	B200	CD3-CD19+(B cell)	OP	-0.35562353	3.00E-13
B15	B58	CD3-CD19+(B cell)	HCT	0.346334463	1.33E-12
B15	B195	CD3-CD19+(B cell)	Na	-0.34623246	1.35E-12
B15	B193	CD3-CD19+(B cell)	K	-0.33387629	9.12E-12
B15	B196	CD3-CD19+(B cell)	TCO2	0.295524667	2.01E-09
B15	B69	CD3-CD19+(B cell)	PV	0.288098295	5.24E-09
B15	B63	CD3-CD19+(B cell)	WBRSR(mPas_200)	0.272329153	3.66E-08
B15	B54	CD3-CD19+(B cell)	ESR	-0.26915028	5.33E-08
B15	B66	CD3-CD19+(B cell)	WBRSR(mPas_100)	0.263523657	1.03E-07
B15	B67	CD3-CD19+(B cell)	WBRSR(mPas_50)	0.262726839	1.13E-07
B15	B101	CD3-CD19+(B cell)	anti-Sc70	0.256331751	2.32E-07
B15	B102	CD3-CD19+(B cell)	anti-Sm	0.25633175	2.32E-07
B15	B105	CD3-CD19+(B cell)	anti-U1RNP	0.25633175	2.32E-07
B15	B106	CD3-CD19+(B cell)	anti-P	0.25633175	2.32E-07
B15	B107	CD3-CD19+(B cell)	AnuA	0.256331751	2.32E-07
B15	B108	CD3-CD19+(B cell)	Anti-Cyclin	0.25633175	2.32E-07
B15	B109	CD3-CD19+(B cell)	AMA-M2	0.25633175	2.32E-07
B15	B110	CD3-CD19+(B cell)	AHA	0.25633175	2.32E-07
B15	B95	CD3-CD19+(B cell)	anti-cenpb	0.25633175	2.32E-07
B15	B97	CD3-CD19+(B cell)	dsDNA	0.25633175	2.32E-07
B15	B98	CD3-CD19+(B cell)	anti-Jo-1	0.25633175	2.32E-07
B15	B99	CD3-CD19+(B cell)	anti-PM-Scl	0.25633175	2.32E-07
B15	B120	CD3-CD19+(B cell)	HCV-cAg	0.243972609	8.92E-07
B150	B186	ALP	PFC	0.437443385	0
B150	B179	ALP	α1-MG	-0.35266648	4.85E-13
B150	B193	ALP	K	0.263881379	9.86E-08
B150	B184	ALP	INR	-0.24543065	7.64E-07
B150	B152	ALP	ACA-Ig(G-A-M)	0.245274696	7.77E-07
B150	B159	ALP	β2-MG	0.245265556	7.77E-07
B150	B169	ALP	U-CRY	0.24526336	7.78E-07
B152	B179	ACA-Ig(G-A-M)	α1-MG	-0.41382569	0
B152	B198	ACA-Ig(G-A-M)	P	0.289885257	4.18E-09
B155	B158	IgA	IgM	0.45923468	0
B155	B156	IgA	IgE	0.388569918	8.88E-16
B155	B157	IgA	IgG	0.339180494	4.06E-12
B155	B196	IgA	TCO2	0.25963957	1.60E-07
B156	B157	IgE	IgG	0.451869186	0
B156	B158	IgE	IgM	0.463155439	0

B156	B186	IgE	PFC	0.415572851	0
B156	B184	IgE	INR	-0.25881671	1.76E-07
B157	B158	IgG	IgM	0.579199818	0
B157	B186	IgG	PFC	0.384795593	2.00E-15
B158	B186	IgM	PFC	0.374448466	1.24E-14
B158	B184	IgM	INR	-0.32803584	2.18E-11
B158	B181	IgM	APTT	0.314247899	1.59E-10
B159	B169	β2-MG	U-CRY	0.999993991	0
B159	B179	β2-MG	α1-MG	-0.41381027	0
B159	B198	β2-MG	P	0.289874433	4.18E-09
B16	B139	CD3+T	ADA	-0.59452548	0
B16	B18	CD3+T	CD3+CD4+helper(inducer)T	-0.62169737	0
B16	B19	CD3+T	CD3+CD4+/CD3+CD8+	-0.90042055	0
B16	B195	CD3+T	Na	-0.45702251	0
B16	B20	CD3+T	CD3+CD4+CD8+	0.675954982	0
B16	B200	CD3+T	OP	-0.4149932	0
B16	B21	CD3+T	CD3+CD4-CD8-	0.720866417	0
B16	B22	CD3+T	CD3+CD8+(T)	0.967033733	0
B16	B23	CD3+T	CD3-CD16+CD56+(NK)	-0.7851046	0
B16	B120	CD3+T	HCV-cAg	0.381169441	4.00E-15
B16	B138	CD3+T	PAB	0.379237697	5.33E-15
B16	B17	CD3+T	CD3+CD16+CD56+NK	0.327891948	2.23E-11
B16	B194	CD3+T	Cl	-0.32721974	2.46E-11
B16	B224	CD3+T	pmh	-0.2840429	8.74E-09
B16	B150	CD3+T	ALP	-0.25323696	3.27E-07
B16	B50	CD3+T	eo%	-0.24338643	9.49E-07
B161	B173	LEU	NTT	0.354971264	3.34E-13
B163	B166	SG	Pro	0.347718911	1.07E-12
B163	B176	SG	Ucr	0.303747965	6.75E-10
B163	B222	SG	NS	0.302508433	7.98E-10
B163	B190	SG	Cre	0.290334571	3.94E-09
B163	B216	SG	BMI	0.255713074	2.49E-07
B163	B165	SG	bilirubin	0.245244822	7.79E-07
B167	B223	PC	FH	0.286125156	6.73E-09
B169	B179	U-CRY	α1-MG	-0.41380655	0
B169	B198	U-CRY	P	0.289871835	4.18E-09
B17	B138	CD3+CD16+CD56+NK	PAB	0.511946769	0
B17	B18	CD3+CD16+CD56+NK	CD3+CD4+helper(inducer)T	-0.94380489	0
B17	B19	CD3+CD16+CD56+NK	CD3+CD4+/CD3+CD8+	-0.70621114	0
B17	B20	CD3+CD16+CD56+NK	CD3+CD4+CD8+	-0.474561	0
B17	B21	CD3+CD16+CD56+NK	CD3+CD4-CD8-	0.891123886	0
B17	B22	CD3+CD16+CD56+NK	CD3+CD8+(T)	0.557652708	0
B17	B23	CD3+CD16+CD56+NK	CD3-CD16+CD56+(NK)	-0.84255132	0
B17	B58	CD3+CD16+CD56+NK	HCT	0.442692608	0

B17	B69	CD3+CD16+CD56+NK	PV	0.362902569	9.02E-14
B17	B54	CD3+CD16+CD56+NK	ESR	-0.34493555	1.66E-12
B17	B63	CD3+CD16+CD56+NK	WBRSR(mPas_200)	0.344786351	1.70E-12
B17	B196	CD3+CD16+CD56+NK	TCO2	0.343004147	2.25E-12
B17	B66	CD3+CD16+CD56+NK	WBRSR(mPas_100)	0.333404254	9.79E-12
B17	B67	CD3+CD16+CD56+NK	WBRSR(mPas_50)	0.332738217	1.08E-11
B17	B101	CD3+CD16+CD56+NK	anti-Sc70	0.329940539	1.65E-11
B17	B102	CD3+CD16+CD56+NK	anti-Sm	0.32994054	1.65E-11
B17	B105	CD3+CD16+CD56+NK	anti-U1RNP	0.329940539	1.65E-11
B17	B106	CD3+CD16+CD56+NK	anti-P	0.32994054	1.65E-11
B17	B107	CD3+CD16+CD56+NK	AnuA	0.329940543	1.65E-11
B17	B108	CD3+CD16+CD56+NK	Anti-Cyclin	0.329940539	1.65E-11
B17	B109	CD3+CD16+CD56+NK	AMA-M2	0.329940539	1.65E-11
B17	B110	CD3+CD16+CD56+NK	AHA	0.329940539	1.65E-11
B17	B95	CD3+CD16+CD56+NK	anti-cenpb	0.329940539	1.65E-11
B17	B97	CD3+CD16+CD56+NK	dsDNA	0.32994054	1.65E-11
B17	B98	CD3+CD16+CD56+NK	anti-Jo-1	0.329940539	1.65E-11
B17	B99	CD3+CD16+CD56+NK	anti-PM-Scl	0.32994054	1.65E-11
B17	B193	CD3+CD16+CD56+NK	K	-0.31452771	1.53E-10
B17	B55	CD3+CD16+CD56+NK	TK	-0.30842143	3.57E-10
B17	B76	CD3+CD16+CD56+NK	ApoB	-0.26519772	8.47E-08
B17	B199	CD3+CD16+CD56+NK	Mg	-0.26297417	1.09E-07
B17	B62	CD3+CD16+CD56+NK	WBHSRVI	0.259444526	1.64E-07
B17	B194	CD3+CD16+CD56+NK	Cl	-0.25664255	2.24E-07
B17	B134	CD3+CD16+CD56+NK	CHE	0.249506934	4.93E-07
B174	B190	Urea/Cr	Cre	-0.42236983	0
B174	B191	Urea/Cr	Urea	0.67114201	0
B174	B221	Urea/Cr	Hei	-0.27859334	1.72E-08
B174	B192	Urea/Cr	UA	-0.25431785	2.91E-07
B175	B177	ALB-U	ACR	0.920396331	0
B175	B178	ALB-U	Ig-U	0.982665144	0
B175	B179	ALB-U	α1-MG	0.586600138	0
B175	B180	ALB-U	Tf	0.983615954	0
B176	B179	Ucr	α1-MG	0.29384277	2.51E-09
B177	B178	ACR	Ig-U	0.885455395	0
B177	B179	ACR	α1-MG	0.489960637	0
B177	B180	ACR	Tf	0.886170264	0
B178	B179	Ig-U	α1-MG	0.642894384	0
B178	B180	Ig-U	Tf	0.999202816	0
B179	B180	α1-MG	Tf	0.638036378	0
B179	B186	α1-MG	PFC	-0.42182008	0
B179	B184	α1-MG	INR	0.262389914	1.17E-07
B18	B138	CD3+CD4+helper(inducer)T	PAB	-0.557126	0
B18	B19	CD3+CD4+helper(inducer)T	CD3+CD4+/CD3+CD8+	0.900522177	0

B18	B21	CD3+CD4+helper(inducer)T	CD3+CD4-CD8-	-0.99101622	0
B18	B22	CD3+CD4+helper(inducer)T	CD3+CD8+(T)	-0.80065754	0
B18	B23	CD3+CD4+helper(inducer)T	CD3-CD16+CD56+(NK)	0.973218359	0
B18	B58	CD3+CD4+helper(inducer)T	HCT	-0.41607884	0
B18	B69	CD3+CD4+helper(inducer)T	PV	-0.34334058	2.13E-12
B18	B193	CD3+CD4+helper(inducer)T	K	0.34291616	2.28E-12
B18	B196	CD3+CD4+helper(inducer)T	TCO2	-0.33702351	5.66E-12
B18	B194	CD3+CD4+helper(inducer)T	Cl	0.32725667	2.45E-11
B18	B63	CD3+CD4+helper(inducer)T	WBRSR(mPas_200)	-0.32545372	3.19E-11
B18	B54	CD3+CD4+helper(inducer)T	ESR	0.323818833	4.05E-11
B18	B66	CD3+CD4+helper(inducer)T	WBRSR(mPas_100)	-0.31480929	1.47E-10
B18	B67	CD3+CD4+helper(inducer)T	WBRSR(mPas_50)	-0.31403476	1.64E-10
B18	B101	CD3+CD4+helper(inducer)T	anti-Sc70	-0.30913959	3.24E-10
B18	B102	CD3+CD4+helper(inducer)T	anti-Sm	-0.30913959	3.24E-10
B18	B105	CD3+CD4+helper(inducer)T	anti-U1RNP	-0.30913959	3.24E-10
B18	B106	CD3+CD4+helper(inducer)T	anti-P	-0.30913959	3.24E-10
B18	B107	CD3+CD4+helper(inducer)T	AnuA	-0.30913959	3.24E-10
B18	B108	CD3+CD4+helper(inducer)T	Anti-Cyclin	-0.30913959	3.24E-10
B18	B109	CD3+CD4+helper(inducer)T	AMA-M2	-0.30913959	3.24E-10
B18	B110	CD3+CD4+helper(inducer)T	AHA	-0.30913959	3.24E-10
B18	B95	CD3+CD4+helper(inducer)T	anti-cenpb	-0.30913959	3.24E-10
B18	B97	CD3+CD4+helper(inducer)T	dsDNA	-0.30913959	3.24E-10
B18	B98	CD3+CD4+helper(inducer)T	anti-Jo-1	-0.30913959	3.24E-10
B18	B99	CD3+CD4+helper(inducer)T	anti-PM-Scl	-0.30913959	3.24E-10
B18	B55	CD3+CD4+helper(inducer)T	TK	0.289457111	4.41E-09
B18	B200	CD3+CD4+helper(inducer)T	OP	0.268951268	5.46E-08
B18	B62	CD3+CD4+helper(inducer)T	WBHSRVI	-0.24468841	8.27E-07
B181	B186	APTT	PFC	0.651464386	0
B181	B183	APTT	PTA	-0.30408668	6.45E-10
B181	B194	APTT	Cl	-0.28521455	7.55E-09
B181	B184	APTT	INR	-0.28506102	7.70E-09
B182	B183	TT	PTA	-0.82693263	0
B182	B185	TT	PT	-0.70056512	0
B182	B184	TT	INR	-0.39703352	2.22E-16
B183	B184	PTA	INR	0.508207886	0
B183	B185	PTA	PT	0.640585688	0
B183	B186	PTA	PFC	-0.39160653	4.44E-16
B184	B185	INR	PT	0.765610785	0
B184	B186	INR	PFC	-0.77319186	0
B185	B186	PT	PFC	-0.32909928	1.86E-11
B189	B190	eGFB	Cre	-0.67626556	0
B189	B207	eGFB	WHR	-0.33813669	4.77E-12
B189	B191	eGFB	Urea	-0.33230102	1.16E-11
B189	B192	eGFB	UA	-0.32157249	5.61E-11

B189	B206	eGFB	Waist	-0.29074816	3.74E-09
B189	B220	eGFB	SBP	-0.25472313	2.78E-07
B19	B138	CD3+CD4+/CD3+CD8+	PAB	-0.51995363	0
B19	B139	CD3+CD4+/CD3+CD8+	ADA	0.458183768	0
B19	B21	CD3+CD4+/CD3+CD8+	CD3+CD4-CD8-	-0.95058435	0
B19	B22	CD3+CD4+/CD3+CD8+	CD3+CD8+(T)	-0.98151428	0
B19	B23	CD3+CD4+/CD3+CD8+	CD3-CD16+CD56+(NK)	0.976360002	0
B19	B195	CD3+CD4+/CD3+CD8+	Na	0.38195102	3.33E-15
B19	B200	CD3+CD4+/CD3+CD8+	OP	0.379750627	4.88E-15
B19	B194	CD3+CD4+/CD3+CD8+	Cl	0.363407674	8.30E-14
B19	B193	CD3+CD4+/CD3+CD8+	K	0.320799571	6.27E-11
B19	B58	CD3+CD4+/CD3+CD8+	HCT	-0.30891429	3.34E-10
B19	B20	CD3+CD4+/CD3+CD8+	CD3+CD4+CD8+	-0.28805848	5.27E-09
B19	B120	CD3+CD4+/CD3+CD8+	HCV-cAg	-0.28278736	1.02E-08
B19	B196	CD3+CD4+/CD3+CD8+	TCO2	-0.27071277	4.43E-08
B19	B69	CD3+CD4+/CD3+CD8+	PV	-0.25806676	1.91E-07
B19	B150	CD3+CD4+/CD3+CD8+	ALP	0.249046708	5.18E-07
B19	B63	CD3+CD4+/CD3+CD8+	WBRSR(mPas_200)	-0.24358338	9.29E-07
B190	B192	Cre	UA	0.537050478	0
B190	B222	Cre	NS	0.453394612	0
B190	B206	Cre	Waist	0.345205753	1.59E-12
B190	B191	Cre	Urea	0.34427762	1.84E-12
B190	B216	Cre	BMI	0.340215146	3.47E-12
B190	B207	Cre	WHR	0.335337932	7.31E-12
B190	B209	Cre	Smoking	0.302514972	7.97E-10
B190	B221	Cre	Hei	0.278041825	1.84E-08
B192	B206	UA	Waist	0.460315903	0
B192	B207	UA	WHR	0.45202991	0
B192	B216	UA	BMI	0.416081734	0
B192	B222	UA	NS	0.466348841	0
B192	B215	UA	BMI	0.315669814	1.30E-10
B192	B209	UA	Smoking	0.291109405	3.57E-09
B192	B221	UA	Hei	0.275813577	2.41E-08
B193	B199	K	Mg	0.3332757	9.99E-12
B193	B196	K	TCO2	-0.30302026	7.45E-10
B193	B194	K	Cl	0.256142315	2.37E-07
B194	B197	Cl	AG	-0.45405849	0
B194	B225	Cl	hPBG	0.338073995	4.82E-12
B194	B200	Cl	OP	0.251846916	3.82E-07
B195	B196	Na	TCO2	-0.41410286	0
B195	B197	Na	AG	0.628311413	0
B195	B200	Na	OP	0.925017526	0
B196	B197	TCO2	AG	-0.71900391	0
B196	B200	TCO2	OP	-0.51404569	0

B196	B225	TCO2	hPBG	-0.26429093	9.40E-08
B197	B200	AG	OP	0.609953103	0
B197	B199	AG	Mg	0.266981699	6.88E-08
B198	B200	P	OP	0.278056523	1.83E-08
B2	B189	Age	eGFB	-0.60382522	0
B2	B207	Age	WHR	0.441091561	0
B2	B220	Age	SBP	0.481872534	0
B2	B206	Age	Waist	0.351577234	5.78E-13
B2	B190	Age	Cre	0.323069148	4.52E-11
B2	B191	Age	Urea	0.293251984	2.71E-09
B2	B77	Age	TC	0.288099109	5.24E-09
B2	B70	Age	LDL-C	0.287293842	5.81E-09
B2	B192	Age	UA	0.254008025	3.01E-07
B20	B139	CD3+CD4+CD8+	ADA	-0.53254946	0
B20	B22	CD3+CD4+CD8+	CD3+CD8+(T)	0.466010006	0
B20	B120	CD3+CD4+CD8+	HCV-cAg	0.360016524	1.46E-13
B20	B195	CD3+CD4+CD8+	Na	-0.35900494	1.72E-13
B20	B76	CD3+CD4+CD8+	ApoB	0.277363315	1.99E-08
B20	B224	CD3+CD4+CD8+	pmh	-0.27629216	2.27E-08
B20	B200	CD3+CD4+CD8+	OP	-0.27021318	4.71E-08
B204	B209	Drinking	Smoking	0.468109181	0
B204	B222	Drinking	NS	0.329524412	1.75E-11
B206	B207	Waist	WHR	0.855610835	0
B206	B210	Waist	Hip	0.708112018	0
B206	B215	Waist	BMI	0.795076864	0
B206	B216	Waist	BMI	0.81079271	0
B206	B222	Waist	NS	0.734711855	0
B206	B209	Waist	Smoking	0.323086486	4.50E-11
B206	B221	Waist	Hei	0.312395222	2.06E-10
B207	B215	WHR	BMI	0.570095537	0
B207	B216	WHR	BMI	0.531534844	0
B207	B222	WHR	NS	0.594068499	0
B207	B209	WHR	Smoking	0.31257051	2.01E-10
B207	B220	WHR	SBP	0.287447681	5.70E-09
B207	B210	WHR	Hip	0.245233412	7.80E-07
B209	B222	Smoking	NS	0.401900359	0
B209	B216	Smoking	BMI	0.312062684	2.16E-10
B209	B221	Smoking	Hei	0.258429821	1.84E-07
B21	B138	CD3+CD4-CD8-	PAB	0.557734144	0
B21	B22	CD3+CD4-CD8-	CD3+CD8+(T)	0.873592269	0
B21	B23	CD3+CD4-CD8-	CD3-CD16+CD56+(NK)	-0.99522008	0
B21	B58	CD3+CD4-CD8-	HCT	0.392109696	4.44E-16
B21	B194	CD3+CD4-CD8-	Cl	-0.34544965	1.53E-12
B21	B193	CD3+CD4-CD8-	K	-0.34352527	2.07E-12

B21	B69	CD3+CD4-CD8-	PV	0.324532548	3.65E-11
B21	B196	CD3+CD4-CD8-	TCO2	0.323911733	3.99E-11
B21	B200	CD3+CD4-CD8-	OP	-0.30884485	3.37E-10
B21	B63	CD3+CD4-CD8-	WBRSR(mPas_200)	0.307305945	4.16E-10
B21	B139	CD3+CD4-CD8-	ADA	-0.30567541	5.20E-10
B21	B54	CD3+CD4-CD8-	ESR	-0.30500097	5.70E-10
B21	B66	CD3+CD4-CD8-	WBRSR(mPas_100)	0.297297696	1.60E-09
B21	B67	CD3+CD4-CD8-	WBRSR(mPas_50)	0.296503841	1.77E-09
B21	B101	CD3+CD4-CD8-	anti-Sc70	0.290915191	3.66E-09
B21	B102	CD3+CD4-CD8-	anti-Sm	0.290915191	3.66E-09
B21	B105	CD3+CD4-CD8-	anti-U1RNP	0.29091519	3.66E-09
B21	B106	CD3+CD4-CD8-	anti-P	0.29091519	3.66E-09
B21	B107	CD3+CD4-CD8-	AnuA	0.290915192	3.66E-09
B21	B108	CD3+CD4-CD8-	Anti-Cyclin	0.29091519	3.66E-09
B21	B109	CD3+CD4-CD8-	AMA-M2	0.29091519	3.66E-09
B21	B110	CD3+CD4-CD8-	AHA	0.290915191	3.66E-09
B21	B95	CD3+CD4-CD8-	anti-cenpb	0.290915191	3.66E-09
B21	B97	CD3+CD4-CD8-	dsDNA	0.29091519	3.66E-09
B21	B98	CD3+CD4-CD8-	anti-Jo-1	0.29091519	3.66E-09
B21	B99	CD3+CD4-CD8-	anti-PM-Scl	0.29091519	3.66E-09
B21	B195	CD3+CD4-CD8-	Na	-0.28235494	1.08E-08
B21	B55	CD3+CD4-CD8-	TK	-0.27260032	3.54E-08
B210	B215	Hip	BMI	0.720045334	0
B210	B216	Hip	BMI	0.794202744	0
B210	B222	Hip	NS	0.560520694	0
B210	B221	Hip	Hei	0.398160285	2.22E-16
B211	B212	IOP-R	IOP-L	0.834584827	0
B213	B214	CVA-R	CVA-L	0.787837042	0
B215	B216	BMI	BMI	0.790990592	0
B215	B222	BMI	NS	0.611567091	0
B215	B220	BMI	SBP	0.247324141	6.24E-07
B216	B221	BMI	Hei	0.628805017	0
B216	B222	BMI	NS	0.766049413	0
B218	B220	DBP	SBP	0.65328488	0
B22	B138	CD3+CD8+(T)	PAB	0.471212757	0
B22	B139	CD3+CD8+(T)	ADA	-0.52977116	0
B22	B195	CD3+CD8+(T)	Na	-0.42465178	0
B22	B200	CD3+CD8+(T)	OP	-0.40487278	0
B22	B23	CD3+CD8+(T)	CD3-CD16+CD56+(NK)	-0.91694242	0
B22	B194	CD3+CD8+(T)	Cl	-0.35668982	2.52E-13
B22	B120	CD3+CD8+(T)	HCV-cAg	0.33323241	1.01E-11
B22	B193	CD3+CD8+(T)	K	-0.29108758	3.58E-09
B22	B150	CD3+CD8+(T)	ALP	-0.25719724	2.11E-07
B22	B224	CD3+CD8+(T)	pmh	-0.24555225	7.54E-07

B221	B222	Hei	NS	0.466854263	0
B23	B138	CD3-CD16+CD56+(NK)	PAB	-0.5518536	0
B23	B58	CD3-CD16+CD56+(NK)	HCT	-0.37016111	2.64E-14
B23	B139	CD3-CD16+CD56+(NK)	ADA	0.356937282	2.42E-13
B23	B194	CD3-CD16+CD56+(NK)	Cl	0.354816717	3.42E-13
B23	B193	CD3-CD16+CD56+(NK)	K	0.340074529	3.54E-12
B23	B200	CD3-CD16+CD56+(NK)	OP	0.334472674	8.34E-12
B23	B195	CD3-CD16+CD56+(NK)	Na	0.316722193	1.12E-10
B23	B196	CD3-CD16+CD56+(NK)	TCO2	-0.31066448	2.62E-10
B23	B69	CD3-CD16+CD56+(NK)	PV	-0.30711889	4.27E-10
B23	B63	CD3-CD16+CD56+(NK)	WBRSR(mPas_200)	-0.29056975	3.83E-09
B23	B54	CD3-CD16+CD56+(NK)	ESR	0.287801618	5.45E-09
B23	B66	CD3-CD16+CD56+(NK)	WBRSR(mPas_100)	-0.28113953	1.25E-08
B23	B67	CD3-CD16+CD56+(NK)	WBRSR(mPas_50)	-0.28034056	1.38E-08
B23	B101	CD3-CD16+CD56+(NK)	anti-Sc70	-0.27430891	2.89E-08
B23	B102	CD3-CD16+CD56+(NK)	anti-Sm	-0.27430891	2.89E-08
B23	B105	CD3-CD16+CD56+(NK)	anti-U1RNP	-0.27430891	2.89E-08
B23	B106	CD3-CD16+CD56+(NK)	anti-P	-0.27430891	2.89E-08
B23	B107	CD3-CD16+CD56+(NK)	AnuA	-0.27430891	2.89E-08
B23	B108	CD3-CD16+CD56+(NK)	Anti-Cyclin	-0.27430891	2.89E-08
B23	B109	CD3-CD16+CD56+(NK)	AMA-M2	-0.27430891	2.89E-08
B23	B110	CD3-CD16+CD56+(NK)	AHA	-0.27430891	2.89E-08
B23	B95	CD3-CD16+CD56+(NK)	anti-cenpb	-0.27430891	2.89E-08
B23	B97	CD3-CD16+CD56+(NK)	dsDNA	-0.27430891	2.89E-08
B23	B98	CD3-CD16+CD56+(NK)	anti-Jo-1	-0.27430891	2.89E-08
B23	B99	CD3-CD16+CD56+(NK)	anti-PM-Scl	-0.27430891	2.89E-08
B23	B55	CD3-CD16+CD56+(NK)	TK	0.25720035	2.11E-07
B24	B26	αHBDH	CKMB	0.479133327	0
B24	B27	αHBDH	LDH	0.659238805	0
B24	B83	αHBDH	AFP	0.370990967	2.29E-14
B24	B197	αHBDH	AG	-0.30969944	3.00E-10
B24	B186	αHBDH	PFC	0.299148049	1.25E-09
B24	B82	αHBDH	CEA	0.281316787	1.23E-08
B24	B137	αHBDH	AFU	-0.26887839	5.51E-08
B24	B194	αHBDH	Cl	0.258831474	1.75E-07
B25	B121	CK	C3	-0.48440609	0
B25	B122	CK	C4	-0.48449447	0
B25	B26	CK	CKMB	0.324867569	3.48E-11
B25	B103	CK	anti-SSA/Ro	0.273474939	3.19E-08
B25	B123	CK	hs-CRP1	-0.26481006	8.85E-08
B26	B83	CKMB	AFP	0.558016035	0
B26	B89	CKMB	CA 72-4	0.348999923	8.74E-13
B26	B137	CKMB	AFU	-0.34207839	2.60E-12
B26	B198	CKMB	P	-0.34120883	2.97E-12

B26	B27	CKMB	LDH	0.313361575	1.80E-10
B27	B150	LDH	ALP	0.31363401	1.73E-10
B27	B131	LDH	AST	0.289146308	4.59E-09
B27	B137	LDH	AFU	-0.28297433	9.99E-09
B27	B83	LDH	AFP	0.271085381	4.24E-08
B28	B119	PRL	Hcv-rna	0.518975263	0
B28	B120	PRL	HCV-cAg	0.493886247	0
B28	B152	PRL	ACA-Ig(G-A-M)	-0.51899644	0
B28	B159	PRL	β2-MG	-0.51897708	0
B28	B169	PRL	U-CRY	-0.51897242	0
B28	B29	PRL	E2	0.987541777	0
B28	B30	PRL	LH	-0.60218313	0
B28	B31	PRL	FSH	-0.95902679	0
B28	B32	PRL	Testo	0.989482462	0
B28	B33	PRL	Prog	0.987111234	0
B28	B79	PRL	hb V -dna	0.364365765	7.06E-14
B28	B124	PRL	ACTH	0.33177432	1.25E-11
B28	B74	PRL	ApoA1	-0.29252425	2.97E-09
B28	B82	PRL	CEA	-0.27788024	1.87E-08
B28	B198	PRL	P	-0.27243987	3.61E-08
B29	B119	E2	Hcv-rna	0.559040365	0
B29	B120	E2	HCV-cAg	0.462455791	0
B29	B152	E2	ACA-Ig(G-A-M)	-0.55906316	0
B29	B159	E2	β2-MG	-0.55904232	0
B29	B169	E2	U-CRY	-0.55903731	0
B29	B30	E2	LH	-0.71864792	0
B29	B31	E2	FSH	-0.98147259	0
B29	B32	E2	Testo	0.983565549	0
B29	B33	E2	Prog	0.99995357	0
B29	B79	E2	hb V -dna	0.392690684	4.44E-16
B29	B124	E2	ACTH	0.357185788	2.33E-13
B29	B74	E2	ApoA1	-0.33132461	1.34E-11
B29	B198	E2	P	-0.30754605	4.03E-10
B29	B89	E2	CA 72-4	-0.2907363	3.74E-09
B29	B199	E2	Mg	0.258339383	1.85E-07
B29	B82	E2	CEA	-0.25093223	4.22E-07
B30	B119	LH	Hcv-rna	-0.44171801	0
B30	B152	LH	ACA-Ig(G-A-M)	0.441736005	0
B30	B159	LH	β2-MG	0.441719543	0
B30	B169	LH	U-CRY	0.441715588	0
B30	B199	LH	Mg	-0.41704917	0
B30	B31	LH	FSH	0.726380565	0
B30	B32	LH	Testo	-0.60998655	0
B30	B33	LH	Prog	-0.71907902	0

B30	B89	LH	CA 72-4	0.598506088	0
B30	B134	LH	CHE	-0.39526767	4.44E-16
B30	B74	LH	ApoA1	0.372418957	1.78E-14
B30	B198	LH	P	0.344529151	1.77E-12
B30	B79	LH	hb V -dna	-0.3104985	2.68E-10
B30	B136	LH	5'NT	-0.28838035	5.06E-09
B30	B124	LH	ACTH	-0.28199939	1.13E-08
B30	B75	LH	ApoA1/B	0.28192704	1.14E-08
B31	B119	FSH	Hcv-rna	-0.70691575	0
B31	B120	FSH	HCV-cAg	-0.48278222	0
B31	B124	FSH	ACTH	-0.45077245	0
B31	B152	FSH	ACA-Ig(G-A-M)	0.706944568	0
B31	B159	FSH	β2-MG	0.706918217	0
B31	B169	FSH	U-CRY	0.706911881	0
B31	B32	FSH	Testo	-0.98286426	0
B31	B33	FSH	Prog	-0.983266	0
B31	B79	FSH	hb V -dna	-0.49743246	0
B31	B74	FSH	ApoA1	0.362114053	1.03E-13
B31	B198	FSH	P	0.331866734	1.23E-11
B31	B179	FSH	α1-MG	-0.29169203	3.31E-09
B31	B89	FSH	CA 72-4	0.258507562	1.82E-07
B32	B119	Testo	Hcv-rna	0.636880971	0
B32	B120	Testo	HCV-cAg	0.51621376	0
B32	B124	Testo	ACTH	0.406428052	0
B32	B152	Testo	ACA-Ig(G-A-M)	-0.63690695	0
B32	B159	Testo	β2-MG	-0.63688319	0
B32	B169	Testo	U-CRY	-0.63687748	0
B32	B33	Testo	Prog	0.984531319	0
B32	B79	Testo	hb V -dna	0.447846554	0
B32	B74	Testo	ApoA1	-0.31785229	9.55E-11
B32	B198	Testo	P	-0.29271769	2.90E-09
B32	B179	Testo	α1-MG	0.262652216	1.14E-07
B32	B82	Testo	CEA	-0.25024475	4.55E-07
B33	B119	Prog	Hcv-rna	0.567004201	0
B33	B120	Prog	HCV-cAg	0.464144194	0
B33	B152	Prog	ACA-Ig(G-A-M)	-0.56702733	0
B33	B159	Prog	β2-MG	-0.56700618	0
B33	B169	Prog	U-CRY	-0.5670011	0
B33	B79	Prog	hb V -dna	0.398328638	0
B33	B124	Prog	ACTH	0.36222891	1.01E-13
B33	B74	Prog	ApoA1	-0.3330175	1.04E-11
B33	B198	Prog	P	-0.3089025	3.34E-10
B33	B89	Prog	CA 72-4	-0.28887158	4.75E-09
B33	B199	Prog	Mg	0.256019282	2.41E-07

B33	B82	Prog	CEA	-0.24920207	5.09E-07
B34	B92	TK	ALDH2-Glu504Glu	-0.32122147	5.90E-11
B34	B93	TK	ALDH2-Glu504Lys	0.321221468	5.90E-11
B34	B94	TK	ALDH2-Lys504Lys	-0.32122147	5.90E-11
B34	B178	TK	Ig-U	-0.29566325	1.98E-09
B34	B180	TK	Tf	-0.29263795	2.93E-09
B34	B175	TK	ALB-U	-0.28595836	6.88E-09
B34	B125	TK	Cor	0.281889163	1.14E-08
B34	B177	TK	ACR	-0.26981638	4.93E-08
B34	B124	TK	ACTH	0.247741848	5.96E-07
B34	B74	TK	ApoA1	-0.24752028	6.11E-07
B35	B116	β2-MG	TT3	-0.82869432	0
B36	B167	V-Lym%	PC	0.555103175	0
B36	B223	V-Lym%	FH	0.694876798	0
B36	B37	V-Lym%	BAND	0.457495711	0
B36	B96	V-Lym%	dsDNA (IIF)	0.646996639	0
B37	B167	BAND	PC	-0.36629484	5.11E-14
B39	B78	Other cells	HBcAb	0.85253165	0
B39	B101	Other cells	anti-Sc70	0.362171569	1.02E-13
B39	B102	Other cells	anti-Sm	0.362171569	1.02E-13
B39	B105	Other cells	anti-U1RNP	0.362171569	1.02E-13
B39	B106	Other cells	anti-P	0.362171568	1.02E-13
B39	B107	Other cells	AnuA	0.36217157	1.02E-13
B39	B108	Other cells	Anti-Cyclin	0.362171569	1.02E-13
B39	B109	Other cells	AMA-M2	0.362171569	1.02E-13
B39	B110	Other cells	AHA	0.362171568	1.02E-13
B39	B95	Other cells	anti-cenpb	0.362171569	1.02E-13
B39	B97	Other cells	dsDNA	0.362171568	1.02E-13
B39	B98	Other cells	anti-Jo-1	0.362171568	1.02E-13
B39	B99	Other cells	anti-PM-Scl	0.362171568	1.02E-13
B39	B103	Other cells	anti-SSA/Ro	-0.34545047	1.53E-12
B4	B167	WB-Ca	PC	0.493215425	0
B4	B223	WB-Ca	FH	0.664291572	0
B40	B42	WBC	MON	0.68466748	0
B40	B53	WBC	GR%	0.372367616	1.80E-14
B40	B52	WBC	PLT/BPC	0.355249222	3.19E-13
B40	B46	WBC	RBC Count	0.329626635	1.72E-11
B40	B48	WBC	LYM%	-0.30429244	6.27E-10
B40	B207	WBC	WHR	0.300971259	9.80E-10
B40	B127	WBC	A-G ratio	-0.26915614	5.33E-08
B40	B192	WBC	UA	0.260679515	1.42E-07
B40	B206	WBC	Waist	0.259165419	1.69E-07
B41	B42	MON %	MON	0.530601006	0
B41	B53	MON %	GR%	-0.24846944	5.51E-07

B43	B44	MCV	MCH	0.934518255	0
B43	B46	MCV	RBC Count	-0.49702277	0
B43	B47	MCV	RDW	0.40859818	0
B43	B45	MCV	MCHC	0.344825273	1.69E-12
B43	B52	MCV	PLT/BPC	-0.33223689	1.17E-11
B43	B51	MCV	Hb	0.289795631	4.22E-09
B44	B45	MCH	MCHC	0.654671834	0
B44	B51	MCH	Hb	0.44491138	0
B44	B46	MCH	RBC Count	-0.38240145	3.11E-15
B44	B52	MCH	PLT/BPC	-0.3459287	1.42E-12
B45	B51	MCHC	Hb	0.565733803	0
B45	B126	MCHC	Alb	0.30093388	9.85E-10
B45	B127	MCHC	A-G ratio	0.276715287	2.16E-08
B45	B216	MCHC	BMI	0.251628801	3.91E-07
B46	B47	RBC Count	RDW	-0.40633826	0
B46	B51	RBC Count	Hb	0.646167053	0
B46	B222	RBC Count	NS	0.321774554	5.45E-11
B46	B192	RBC Count	UA	0.314311344	1.58E-10
B46	B216	RBC Count	BMI	0.311100188	2.47E-10
B46	B206	RBC Count	Waist	0.301873891	8.69E-10
B46	B207	RBC Count	WHR	0.279122672	1.61E-08
B46	B209	RBC Count	Smoking	0.253730806	3.10E-07
B46	B221	RBC Count	Hei	0.250655007	4.35E-07
B46	B174	RBC Count	Urea/Cr	-0.24306124	9.82E-07
B47	B126	RDW	Alb	-0.25766426	2.00E-07
B48	B53	LYM%	GR%	-0.91990002	0
B48	B126	LYM%	Alb	0.29223409	3.09E-09
B48	B127	LYM%	A-G ratio	0.252148646	3.69E-07
B49	B50	baso%	eo%	0.279490587	1.54E-08
B49	B53	baso%	GR%	-0.24883922	5.30E-07
B5	B167	WB-Mg	PC	-0.76302703	0
B5	B37	WB-Mg	BAND	-0.7200823	0
B5	B6	WB-Mg	WB-Fe	0.470013944	0
B5	B96	WB-Mg	dsDNA (IIF)	0.272772363	3.47E-08
B50	B53	eo%	GR%	-0.35766757	2.15E-13
B51	B216	Hb	BMI	0.438923747	0
B51	B222	Hb	NS	0.431144862	0
B51	B221	Hb	Hei	0.390115374	8.88E-16
B51	B206	Hb	Waist	0.366627036	4.82E-14
B51	B192	Hb	UA	0.354544678	3.58E-13
B51	B190	Hb	Cre	0.327858086	2.24E-11
B51	B209	Hb	Smoking	0.315703007	1.30E-10
B51	B126	Hb	Alb	0.310592825	2.65E-10
B51	B163	Hb	SG	0.309535078	3.06E-10

B51	B210	Hb	Hip	0.299720425	1.16E-09
B51	B207	Hb	WHR	0.283782393	9.03E-09
B51	B141	Hb	AST/ALT	-0.2743576	2.87E-08
B51	B215	Hb	BMI	0.26238389	1.17E-07
B51	B129	Hb	IBIL	0.259200107	1.68E-07
B53	B126	GR%	Alb	-0.26113841	1.35E-07
B53	B127	GR%	A-G ratio	-0.25138198	4.02E-07
B54	B55	ESR	TK	0.451299091	0
B54	B58	ESR	HCT	-0.60175735	0
B54	B60	ESR	WBRVI	-0.32791475	2.22E-11
B54	B69	ESR	PV	0.319171	7.92E-11
B55	B114	TK	TSH TT3 TT4(TT4)	0.419044692	0
B55	B56	TK	IR	0.806816132	0
B55	B57	TK	RBCAI	-0.69504111	0
B55	B58	TK	HCT	-0.70229403	0
B55	B60	TK	WBRVI	-0.68152992	0
B55	B61	TK	WBHSRV	0.683682579	0
B55	B64	TK	WBRSR(mPas_5)	-0.45071559	0
B55	B65	TK	WBRSR(mPas_1)	-0.61250009	0
B55	B59	TK	WBRV	-0.3576802	2.14E-13
B55	B196	TK	TCO2	-0.26519127	8.47E-08
B55	B156	TK	IgE	0.262350619	1.18E-07
B55	B62	TK	WBHSRVI	0.245437659	7.63E-07
B56	B114	IR	TSH TT3 TT4(TT4)	0.442262537	0
B56	B156	IR	IgE	0.40650249	0
B56	B57	IR	RBCAI	-0.80071054	0
B56	B59	IR	WBRV	-0.41684703	0
B56	B60	IR	WBRVI	-0.51013545	0
B56	B61	IR	WBHSRV	0.93849919	0
B56	B62	IR	WBHSRVI	0.770195153	0
B56	B63	IR	WBRSR(mPas_200)	0.659357974	0
B56	B66	IR	WBRSR(mPas_100)	0.630403687	0
B56	B67	IR	WBRSR(mPas_50)	0.543434419	0
B56	B65	IR	WBRSR(mPas_1)	-0.3896099	8.88E-16
B57	B114	RBCAI	TSH TT3 TT4(TT4)	-0.41166058	0
B57	B156	RBCAI	IgE	-0.46874507	0
B57	B59	RBCAI	WBRV	0.834843898	0
B57	B60	RBCAI	WBRVI	0.876672785	0
B57	B61	RBCAI	WBHSRV	-0.70751906	0
B57	B62	RBCAI	WBHSRVI	-0.54463124	0
B57	B63	RBCAI	WBRSR(mPas_200)	-0.40915147	0
B57	B64	RBCAI	WBRSR(mPas_5)	0.494550505	0
B57	B65	RBCAI	WBRSR(mPas_1)	0.775376649	0
B57	B66	RBCAI	WBRSR(mPas_100)	-0.35165239	5.71E-13

B57	B58	RBCAI	HCT	0.259632505	1.60E-07
B58	B60	HCT	WBRVI	0.596779414	0
B58	B62	HCT	WBHSRVI	0.506520018	0
B58	B63	HCT	WBRSR(mPas_200)	0.544878434	0
B58	B64	HCT	WBRSR(mPas_5)	0.702149714	0
B58	B65	HCT	WBRSR(mPas_1)	0.611138697	0
B58	B66	HCT	WBRSR(mPas_100)	0.53474365	0
B58	B67	HCT	WBRSR(mPas_50)	0.586139119	0
B58	B69	HCT	PV	0.271401283	4.09E-08
B58	B76	HCT	ApoB	-0.24701832	6.44E-07
B59	B156	WBRV	IgE	-0.40218827	0
B59	B60	WBRV	WBRVI	0.847648301	0
B59	B64	WBRV	WBRSR(mPas_5)	0.726194067	0
B59	B65	WBRV	WBRSR(mPas_1)	0.879048689	0
B59	B69	WBRV	PV	0.358782974	1.79E-13
B59	B62	WBRV	WBHSRVI	-0.27282226	3.45E-08
B59	B114	WBRV	TSH TT3 TT4(TT4)	-0.26532188	8.35E-08
B6	B167	WB-Fe	PC	0.418405566	0
B6	B8	WB-Fe	WB-Zinc	0.487565206	0
B60	B64	WBRVI	WBRSR(mPas_5)	0.814614693	0
B60	B65	WBRVI	WBRSR(mPas_1)	0.954959449	0
B60	B61	WBRVI	WBHSRV	-0.37522913	1.11E-14
B60	B156	WBRVI	IgE	-0.35946041	1.60E-13
B60	B114	WBRVI	TSH TT3 TT4(TT4)	-0.33465557	8.11E-12
B61	B62	WBHSRV	WBHSRVI	0.803279548	0
B61	B63	WBHSRV	WBRSR(mPas_200)	0.813287253	0
B61	B66	WBHSRV	WBRSR(mPas_100)	0.806137567	0
B61	B67	WBHSRV	WBRSR(mPas_50)	0.742007867	0
B61	B69	WBHSRV	PV	0.551329051	0
B61	B114	WBHSRV	TSH TT3 TT4(TT4)	0.395300314	4.44E-16
B61	B156	WBHSRV	IgE	0.37416385	1.33E-14
B61	B92	WBHSRV	ALDH2-Glu504Glu	-0.25647477	2.29E-07
B61	B93	WBHSRV	ALDH2-Glu504Lys	0.256474772	2.29E-07
B61	B94	WBHSRV	ALDH2-Lys504Lys	-0.25647477	2.29E-07
B62	B63	WBHSRVI	WBRSR(mPas_200)	0.933823504	0
B62	B66	WBHSRVI	WBRSR(mPas_100)	0.90306135	0
B62	B67	WBHSRVI	WBRSR(mPas_50)	0.858426388	0
B62	B69	WBHSRVI	PV	0.39388528	4.44E-16
B62	B64	WBHSRVI	WBRSR(mPas_5)	0.378789292	5.77E-15
B62	B156	WBHSRVI	IgE	0.378506969	6.22E-15
B62	B114	WBHSRVI	TSH TT3 TT4(TT4)	0.273966345	3.01E-08
B63	B64	WBRSR(mPas_200)	WBRSR(mPas_5)	0.583769506	0
B63	B66	WBRSR(mPas_200)	WBRSR(mPas_100)	0.995022667	0
B63	B67	WBRSR(mPas_200)	WBRSR(mPas_50)	0.976946727	0

B63	B69	WBRSR(mPas_200)	PV	0.69196705	0
B63	B156	WBRSR(mPas_200)	IgE	0.305017718	5.69E-10
B63	B65	WBRSR(mPas_200)	WBRSR(mPas_1)	0.250687028	4.33E-07
B64	B65	WBRSR(mPas_5)	WBRSR(mPas_1)	0.931877813	0
B64	B66	WBRSR(mPas_5)	WBRSR(mPas_100)	0.636368702	0
B64	B67	WBRSR(mPas_5)	WBRSR(mPas_50)	0.73330688	0
B64	B69	WBRSR(mPas_5)	PV	0.653689838	0
B65	B67	WBRSR(mPas_1)	WBRSR(mPas_50)	0.437092901	0
B65	B69	WBRSR(mPas_1)	PV	0.454704271	0
B65	B66	WBRSR(mPas_1)	WBRSR(mPas_100)	0.31356686	1.75E-10
B65	B156	WBRSR(mPas_1)	IgE	-0.30898422	3.31E-10
B65	B114	WBRSR(mPas_1)	TSH TT3 TT4(TT4)	-0.28713521	5.93E-09
B66	B67	WBRSR(mPas_100)	WBRSR(mPas_50)	0.991053055	0
B66	B69	WBRSR(mPas_100)	PV	0.741290403	0
B66	B156	WBRSR(mPas_100)	IgE	0.27547767	2.51E-08
B67	B69	WBRSR(mPas_50)	PV	0.770733747	0
B69	B92	PV	ALDH2-Glu504Glu	-0.31454597	1.53E-10
B69	B93	PV	ALDH2-Glu504Lys	0.314545972	1.53E-10
B69	B94	PV	ALDH2-Lys504Lys	-0.31454597	1.53E-10
B69	B125	PV	Cor	0.276845225	2.12E-08
B69	B171	PV	EC	-0.25647596	2.29E-07
B69	B124	PV	ACTH	0.243668891	9.21E-07
B7	B116	WB-Cop	TT3	-0.78920046	0
B7	B9	WB-Cop	RH	-0.82105805	0
B70	B77	LDL-C	TC	0.861614842	0
B70	B76	LDL-C	ApoB	0.272023742	3.79E-08
B70	B206	LDL-C	Waist	0.249142193	5.12E-07
B71	B72	TG	HDL-C	-0.36193992	1.06E-13
B71	B206	TG	Waist	0.343114007	2.21E-12
B71	B192	TG	UA	0.335175835	7.49E-12
B71	B207	TG	WHR	0.327018726	2.54E-11
B71	B77	TG	TC	0.320338174	6.70E-11
B71	B215	TG	BMI	0.275122326	2.62E-08
B71	B222	TG	NS	0.272877008	3.43E-08
B71	B216	TG	BMI	0.256053129	2.40E-07
B72	B192	HDL-C	UA	-0.41787824	0
B72	B77	HDL-C	TC	0.413839498	0
B72	B222	HDL-C	NS	-0.34242985	2.46E-12
B72	B207	HDL-C	WHR	-0.32312084	4.48E-11
B72	B206	HDL-C	Waist	-0.31304036	1.88E-10
B72	B216	HDL-C	BMI	-0.30247797	8.01E-10
B72	B209	HDL-C	Smoking	-0.26423414	9.46E-08
B72	B190	HDL-C	Cre	-0.25796966	1.93E-07
B72	B174	HDL-C	Urea/Cr	0.251248857	4.07E-07

B72	B221	HDL-C	Hei	-0.24767399	6.01E-07
B73	B123	ApoA	hs-CRP1	0.462947668	0
B73	B186	ApoA	PFC	0.471328571	0
B73	B184	ApoA	INR	-0.35081722	6.53E-13
B73	B156	ApoA	IgE	0.27386286	3.05E-08
B73	B125	ApoA	Cor	-0.26637777	7.38E-08
B73	B76	ApoA	ApoB	0.257039762	2.15E-07
B74	B124	ApoA1	ACTH	-0.42943326	0
B74	B179	ApoA1	α1-MG	-0.42334969	0
B74	B75	ApoA1	ApoA1/B	0.534878825	0
B74	B125	ApoA1	Cor	-0.37398506	1.38E-14
B74	B119	ApoA1	Hcv-rna	-0.33221058	1.17E-11
B74	B152	ApoA1	ACA-Ig(G-A-M)	0.332224108	1.17E-11
B74	B159	ApoA1	β2-MG	0.332211733	1.17E-11
B74	B169	ApoA1	U-CRY	0.332208757	1.17E-11
B74	B198	ApoA1	P	0.308605739	3.48E-10
B74	B176	ApoA1	Ucr	-0.27894199	1.64E-08
B74	B92	ApoA1	ALDH2-Glu504Glu	0.269738274	4.98E-08
B74	B93	ApoA1	ALDH2-Glu504Lys	-0.26973827	4.98E-08
B74	B94	ApoA1	ALDH2-Lys504Lys	0.269738272	4.98E-08
B74	B123	ApoA1	hs-CRP1	0.255078303	2.67E-07
B74	B150	ApoA1	ALP	0.248687551	5.38E-07
B74	B199	ApoA1	Mg	-0.24673255	6.64E-07
B75	B76	ApoA1/B	ApoB	-0.8840219	0
B75	B148	ApoA1/B	Anti_TPO	0.268566099	5.71E-08
B76	B148	ApoB	Anti_TPO	-0.26189131	1.24E-07
B76	B77	ApoB	TC	0.243668058	9.21E-07
B77	B188	TC	Ca	0.273228542	3.29E-08
B78	B103	HBcAb	anti-SSA/Ro	-0.26463462	9.04E-08
B78	B104	HBcAb	anti-SSB	-0.25947482	1.63E-07
B79	B119	hb V -dna	Hcv-rna	0.706210978	0
B79	B125	hb V -dna	Cor	-0.59665199	0
B79	B135	hb V -dna	TBA	-0.47547986	0
B79	B152	hb V -dna	ACA-Ig(G-A-M)	-0.70623977	0
B79	B159	hb V -dna	β2-MG	-0.70621344	0
B79	B169	hb V -dna	U-CRY	-0.70620711	0
B79	B181	hb V -dna	APTT	-0.43488164	0
B79	B92	hb V -dna	ALDH2-Glu504Glu	0.706211306	0
B79	B93	hb V -dna	ALDH2-Glu504Lys	-0.70621131	0
B79	B94	hb V -dna	ALDH2-Lys504Lys	0.7062113	0
B79	B184	hb V -dna	INR	0.355785889	2.92E-13
B79	B197	hb V -dna	AG	-0.33404829	8.89E-12
B79	B81	hb V -dna	TSGF	-0.31632257	1.19E-10
B79	B194	hb V -dna	Cl	0.291576852	3.36E-09

B79	B120	hb V -dna	HCV-cAg	0.282787295	1.02E-08
B79	B196	hb V -dna	TCO2	0.270712789	4.43E-08
B79	B186	hb V -dna	PFC	-0.26904517	5.40E-08
B79	B199	hb V -dna	Mg	-0.26150129	1.30E-07
B79	B179	hb V -dna	α1-MG	0.260742568	1.41E-07
B8	B96	WB-Zinc	dsDNA (IIF)	0.545705156	0
B81	B92	TSGF	ALDH2-Glu504Glu	-0.44998593	0
B81	B93	TSGF	ALDH2-Glu504Lys	0.449985933	0
B81	B94	TSGF	ALDH2-Lys504Lys	-0.44998593	0
B81	B125	TSGF	Cor	0.396522209	2.22E-16
B81	B124	TSGF	ACTH	0.349211559	8.45E-13
B81	B181	TSGF	APTT	0.27684532	2.12E-08
B82	B85	CEA	SCC	0.458328619	0
B82	B86	CEA	TBhCG	-0.53661017	0
B82	B87	CEA	NSE	0.510663492	0
B82	B88	CEA	CA 242	0.628465589	0
B82	B83	CEA	AFP	0.331068091	1.39E-11
B82	B194	CEA	Cl	0.294499471	2.30E-09
B83	B89	AFP	CA 72-4	0.488725542	0
B83	B137	AFP	AFU	-0.33369399	9.38E-12
B83	B84	AFP	C-PSA	0.263477615	1.03E-07
B83	B198	AFP	P	-0.25243085	3.58E-07
B85	B194	SCC	Cl	0.282207732	1.10E-08
B85	B197	SCC	AG	-0.26085688	1.39E-07
B85	B224	SCC	pmh	0.252097865	3.71E-07
B87	B88	NSE	CA 242	0.643778248	0
B87	B90	NSE	CYFRA 21-1	0.525918948	0
B88	B90	CA 242	CYFRA 21-1	0.429367372	0
B89	B134	CA 72-4	CHE	-0.30686154	4.42E-10
B89	B199	CA 72-4	Mg	-0.28069651	1.33E-08
B90	B151	CYFRA 21-1	Anti-CCP	0.249408012	4.98E-07
B91	B127	TP	A-G ratio	-0.51909936	0
B91	B130	TP	GLB	0.766534869	0
B91	B188	TP	Ca	0.283822478	8.99E-09
B92	B124	ALDH2-Glu504Glu	ACTH	-0.77295096	0
B92	B125	ALDH2-Glu504Glu	Cor	-0.87884206	0
B92	B135	ALDH2-Glu504Glu	TBA	-0.43789181	0
B92	B181	ALDH2-Glu504Glu	APTT	-0.61649705	0
B92	B184	ALDH2-Glu504Glu	INR	0.503368653	0
B92	B194	ALDH2-Glu504Glu	Cl	0.460793281	0
B92	B94	ALDH2-Glu504Glu	ALDH2-Lys504Lys	0.999999992	0
B92	B197	ALDH2-Glu504Glu	AG	-0.38983807	8.88E-16
B92	B186	ALDH2-Glu504Glu	PFC	-0.38244907	3.11E-15
B92	B158	ALDH2-Glu504Glu	IgM	-0.32866191	1.99E-11

B92	B199	ALDH2-Glu504Glu	Mg	-0.31517355	1.40E-10
B92	B198	ALDH2-Glu504Glu	P	0.281615372	1.18E-08
B93	B124	ALDH2-Glu504Lys	ACTH	0.772950962	0
B93	B125	ALDH2-Glu504Lys	Cor	0.878842064	0
B93	B135	ALDH2-Glu504Lys	TBA	0.437891811	0
B93	B181	ALDH2-Glu504Lys	APTT	0.616497047	0
B93	B184	ALDH2-Glu504Lys	INR	-0.50336865	0
B93	B194	ALDH2-Glu504Lys	Cl	-0.46079328	0
B93	B94	ALDH2-Glu504Lys	ALDH2-Lys504Lys	-0.99999999	0
B93	B197	ALDH2-Glu504Lys	AG	0.389838067	8.88E-16
B93	B186	ALDH2-Glu504Lys	PFC	0.382449069	3.11E-15
B93	B158	ALDH2-Glu504Lys	IgM	0.328661908	1.99E-11
B93	B199	ALDH2-Glu504Lys	Mg	0.315173549	1.40E-10
B93	B198	ALDH2-Glu504Lys	P	-0.28161537	1.18E-08
B94	B124	ALDH2-Lys504Lys	ACTH	-0.77295096	0
B94	B125	ALDH2-Lys504Lys	Cor	-0.87884206	0
B94	B135	ALDH2-Lys504Lys	TBA	-0.43789181	0
B94	B181	ALDH2-Lys504Lys	APTT	-0.61649704	0
B94	B184	ALDH2-Lys504Lys	INR	0.503368649	0
B94	B194	ALDH2-Lys504Lys	Cl	0.460793278	0
B94	B197	ALDH2-Lys504Lys	AG	-0.38983806	8.88E-16
B94	B186	ALDH2-Lys504Lys	PFC	-0.38244907	3.11E-15
B94	B158	ALDH2-Lys504Lys	IgM	-0.32866191	1.99E-11
B94	B199	ALDH2-Lys504Lys	Mg	-0.31517355	1.40E-10
B94	B198	ALDH2-Lys504Lys	P	0.28161537	1.18E-08
B95	B103	anti-cenpb	anti-SSA/Ro	-0.67410508	0
B95	B106	anti-cenpb	anti-P	0.999999999	0
B95	B108	anti-cenpb	Anti-Cyclin	1	0
B95	B109	anti-cenpb	AMA-M2	1	0
B95	B97	anti-cenpb	dsDNA	1	0
B95	B98	anti-cenpb	anti-Jo-1	0.999999998	0
B95	B99	anti-cenpb	anti-PM-Scl	1	0
B95	B121	anti-cenpb	C3	0.32999543	1.63E-11
B95	B122	anti-cenpb	C4	0.329969907	1.64E-11
B95	B104	anti-cenpb	anti-SSB	-0.31611978	1.22E-10
B95	B155	anti-cenpb	IgA	0.251683699	3.88E-07
B95	B157	anti-cenpb	IgG	-0.25117744	4.11E-07
B95	B100	anti-cenpb	anti-Ro52	-0.24927397	5.05E-07
B97	B103	dsDNA	anti-SSA/Ro	-0.67410508	0
B97	B105	dsDNA	anti-U1RNP	0.999999998	0
B97	B108	dsDNA	Anti-Cyclin	0.999999999	0
B97	B109	dsDNA	AMA-M2	0.999999999	0
B97	B110	dsDNA	AHA	0.999999998	0
B97	B99	dsDNA	anti-PM-Scl	0.999999999	0

B97	B121	dsDNA	C3	0.32999543	1.63E-11
B97	B122	dsDNA	C4	0.329969907	1.64E-11
B97	B104	dsDNA	anti-SSB	-0.31611978	1.22E-10
B97	B155	dsDNA	IgA	0.251683699	3.88E-07
B97	B157	dsDNA	IgG	-0.25117744	4.11E-07
B97	B100	dsDNA	anti-Ro52	-0.24927397	5.05E-07
B98	B101	anti-Jo-1	anti-Sc70	0.999999997	0
B98	B102	anti-Jo-1	anti-Sm	0.999999999	0
B98	B103	anti-Jo-1	anti-SSA/Ro	-0.67410508	0
B98	B105	anti-Jo-1	anti-U1RNP	0.999999998	0
B98	B109	anti-Jo-1	AMA-M2	0.999999997	0
B98	B110	anti-Jo-1	AHA	0.999999999	0
B98	B121	anti-Jo-1	C3	0.32999543	1.63E-11
B98	B122	anti-Jo-1	C4	0.329969906	1.64E-11
B98	B104	anti-Jo-1	anti-SSB	-0.31611978	1.22E-10
B98	B155	anti-Jo-1	IgA	0.251683699	3.88E-07
B98	B157	anti-Jo-1	IgG	-0.25117744	4.11E-07
B98	B100	anti-Jo-1	anti-Ro52	-0.24927397	5.05E-07
B99	B103	anti-PM-Scl	anti-SSA/Ro	-0.67410508	0
B99	B105	anti-PM-Scl	anti-U1RNP	0.999999999	0
B99	B106	anti-PM-Scl	anti-P	1	0
B99	B109	anti-PM-Scl	AMA-M2	0.999999998	0
B99	B110	anti-PM-Scl	AHA	0.999999999	0
B99	B121	anti-PM-Scl	C3	0.32999543	1.63E-11
B99	B122	anti-PM-Scl	C4	0.329969907	1.64E-11
B99	B104	anti-PM-Scl	anti-SSB	-0.31611978	1.22E-10
B99	B155	anti-PM-Scl	IgA	0.251683699	3.88E-07
B99	B157	anti-PM-Scl	IgG	-0.25117744	4.11E-07
B99	B100	anti-PM-Scl	anti-Ro52	-0.24927397	5.05E-07

Supplementary Table 15. PEI Correlations in Chronic rhinitis

Row	Column	Row	Column	Cor	*P*
B1	B163	Gender	SG	-0.54351	0
B1	B190	Gender	Cre	-0.68471	0
B1	B192	Gender	UA	-0.51787	0
B1	B206	Gender	Waist	-0.58224	0
B1	B207	Gender	WHR	-0.52517	0
B1	B210	Gender	Hip	-0.46255	0
B1	B216	Gender	BMI	-0.6616	0
B1	B221	Gender	Hei	-0.66505	0
B1	B222	Gender	NS	-0.70413	0
B1	B46	Gender	RBC Count	-0.61346	0
B1	B51	Gender	Hb	-0.69961	0
B1	B72	Gender	HDL-C	0.442135	0
B1	B215	Gender	BMI	-0.40381	5.55E-14
B1	B141	Gender	AST/ALT	0.341137	3.67E-10
B1	B204	Gender	Drinking	-0.32342	3.16E-09
B1	B174	Gender	Urea/Cr	0.305185	2.53E-08
B1	B128	Gender	ALT	-0.30009	4.40E-08
B1	B191	Gender	Urea	-0.28565	2.01E-07
B1	B45	Gender	MCHC	-0.28059	3.35E-07
B1	B218	Gender	DBP	-0.27417	6.32E-07
B10	B12	PG I	PG II	0.579791	0
B10	B11	PG I	PGI/PGII	0.270641	8.90E-07
B100	B103	anti-Ro52	anti-SSA/Ro	1	0
B100	B105	anti-Ro52	anti-U1RNP	1	0
B100	B106	anti-Ro52	anti-P	1	0
B100	B107	anti-Ro52	AnuA	1	0
B100	B151	anti-Ro52	Anti-CCP	-0.62933	0
B100	B154	anti-Ro52	RF	0.392478	3.15E-13
B100	B178	anti-Ro52	Ig-U	0.288523	1.49E-07
B101	B102	anti-Sc70	anti-Sm	1	0
B101	B106	anti-Sc70	anti-P	1	0
B101	B107	anti-Sc70	AnuA	1	0
B101	B108	anti-Sc70	Anti-Cyclin	1	0
B101	B151	anti-Sc70	Anti-CCP	-0.62933	0
B101	B154	anti-Sc70	RF	0.392478	3.15E-13
B101	B178	anti-Sc70	Ig-U	0.288523	1.49E-07
B102	B104	anti-Sm	anti-SSB	1	0
B102	B106	anti-Sm	anti-P	1	0
B102	B107	anti-Sm	AnuA	1	0
B102	B108	anti-Sm	Anti-Cyclin	1	0
B102	B151	anti-Sm	Anti-CCP	-0.62933	0
B102	B154	anti-Sm	RF	0.392478	3.15E-13

B102	B178	anti-Sm	Ig-U	0.288523	1.49E-07
B103	B105	anti-SSA/Ro	anti-U1RNP	1	0
B103	B106	anti-SSA/Ro	anti-P	1	0
B103	B107	anti-SSA/Ro	AnuA	1	0
B103	B151	anti-SSA/Ro	Anti-CCP	-0.62933	0
B103	B154	anti-SSA/Ro	RF	0.392478	3.15E-13
B103	B178	anti-SSA/Ro	Ig-U	0.288523	1.49E-07
B104	B107	anti-SSB	AnuA	1	0
B104	B151	anti-SSB	Anti-CCP	-0.62933	0
B104	B154	anti-SSB	RF	0.392478	3.15E-13
B104	B178	anti-SSB	Ig-U	0.288523	1.49E-07
B105	B106	anti-U1RNP	anti-P	1	0
B105	B107	anti-U1RNP	AnuA	1	0
B105	B109	anti-U1RNP	AMA-M2	1	0
B105	B110	anti-U1RNP	AHA	1	0
B105	B151	anti-U1RNP	Anti-CCP	-0.62933	0
B105	B154	anti-U1RNP	RF	0.392478	3.15E-13
B105	B178	anti-U1RNP	Ig-U	0.288523	1.49E-07
B106	B107	anti-P	AnuA	1	0
B106	B108	anti-P	Anti-Cyclin	1	0
B106	B110	anti-P	AHA	1	0
B106	B151	anti-P	Anti-CCP	-0.62933	0
B106	B154	anti-P	RF	0.392478	3.15E-13
B106	B178	anti-P	Ig-U	0.288523	1.49E-07
B107	B108	AnuA	Anti-Cyclin	1	0
B107	B109	AnuA	AMA-M2	1	0
B107	B110	AnuA	AHA	1	0
B107	B151	AnuA	Anti-CCP	-0.62933	0
B107	B154	AnuA	RF	0.392478	3.15E-13
B107	B178	AnuA	Ig-U	0.288523	1.49E-07
B108	B110	Anti-Cyclin	AHA	1	0
B108	B151	Anti-Cyclin	Anti-CCP	-0.62933	0
B108	B154	Anti-Cyclin	RF	0.392478	3.15E-13
B108	B178	Anti-Cyclin	Ig-U	0.288523	1.49E-07
B109	B151	AMA-M2	Anti-CCP	-0.62933	0
B109	B154	AMA-M2	RF	0.392478	3.15E-13
B109	B178	AMA-M2	Ig-U	0.288523	1.49E-07
B11	B12	PGI/PGII	PG II	-0.50773	0
B11	B85	PGI/PGII	SCC	0.371621	6.45E-12
B11	B200	PGI/PGII	OP	-0.29386	8.57E-08
B11	B195	PGI/PGII	Na	-0.28343	2.52E-07
B11	B87	PGI/PGII	NSE	-0.27192	7.87E-07
B11	B90	PGI/PGII	CYFRA 21-1	-0.27026	9.23E-07
B110	B151	AHA	Anti-CCP	-0.62933	0

B110	B154	AHA	RF	0.392478	3.15E-13
B110	B178	AHA	Ig-U	0.288523	1.49E-07
B112	B113	FT4	FT3	0.870398	0
B112	B114	FT4	TSH TT3 TT4(TT4)	0.553524	0
B112	B115	FT4	TSH TT3 TT4(TT3)	0.492649	0
B113	B115	FT3	TSH TT3 TT4(TT3)	0.394364	2.37E-13
B113	B114	FT3	TSH TT3 TT4(TT4)	0.362545	2.25E-11
B114	B115	TSH TT3 TT4(TT4)	TSH TT3 TT4(TT3)	0.878867	0
B114	B148	TSH TT3 TT4(TT4)	Anti_TPO	0.380804	1.75E-12
B115	B148	TSH TT3 TT4(TT3)	Anti_TPO	0.466372	0
B116	B142	TT3	25-(OH)D	-0.73366	0
B117	B145	TT4	BGP	-0.82624	0
B117	B171	TT4	EC	0.59222	0
B118	B170	CEA1	U-GLU	0.283509	2.50E-07
B119	B120	Hcv-rna	HCV-cAg	0.492851	0
B119	B121	Hcv-rna	C3	-0.81521	0
B119	B122	Hcv-rna	C4	-0.81521	0
B119	B143	Hcv-rna	β-CTx	-0.81522	0
B119	B159	Hcv-rna	β2-MG	-0.81521	0
B119	B169	Hcv-rna	U-CRY	-0.8152	0
B120	B121	HCV-cAg	C3	-0.40178	7.64E-14
B120	B122	HCV-cAg	C4	-0.40178	7.64E-14
B120	B143	HCV-cAg	β-CTx	-0.40178	7.64E-14
B120	B159	HCV-cAg	β2-MG	-0.40178	7.64E-14
B120	B169	HCV-cAg	U-CRY	-0.40178	7.64E-14
B121	B169	C3	U-CRY	0.999999	0
B122	B169	C4	U-CRY	0.999999	0
B123	B154	hs-CRP1	RF	0.406961	3.40E-14
B124	B180	ACTH	Tf	-0.49456	0
B124	B187	ACTH	NSE	-0.6133	0
B124	B185	ACTH	PT	-0.38174	1.53E-12
B124	B179	ACTH	α1-MG	0.351614	9.60E-11
B124	B186	ACTH	PFC	-0.31672	6.90E-09
B124	B183	ACTH	PTA	-0.30721	2.02E-08
B124	B156	ACTH	IgE	-0.2954	7.27E-08
B125	B180	Cor	Tf	0.494563	0
B125	B187	Cor	NSE	0.613304	0
B125	B185	Cor	PT	0.381739	1.53E-12
B125	B179	Cor	α1-MG	-0.35161	9.60E-11
B125	B186	Cor	PFC	0.316722	6.90E-09
B125	B183	Cor	PTA	0.307213	2.02E-08
B125	B156	Cor	IgE	0.295403	7.27E-08
B126	B127	Alb	A-G ratio	0.431677	4.44E-16
B126	B188	Alb	Ca	0.434188	4.44E-16

B127	B130	A-G ratio	GLB	-0.82835	0
B128	B131	ALT	AST	0.56791	0
B128	B140	ALT	r-GT	0.545343	0
B128	B141	ALT	AST/ALT	-0.51182	0
B128	B216	ALT	BMI	0.383826	1.13E-12
B128	B222	ALT	NS	0.358311	3.97E-11
B128	B206	ALT	Waist	0.357793	4.25E-11
B128	B215	ALT	BMI	0.348559	1.43E-10
B128	B192	ALT	UA	0.348024	1.53E-10
B128	B210	ALT	Hip	0.318888	5.37E-09
B128	B207	ALT	WHR	0.281273	3.13E-07
B129	B132	IBIL	Dbil	0.718275	0
B129	B133	IBIL	TBIL	0.98139	0
B131	B184	AST	INR	-0.39726	1.53E-13
B132	B133	Dbil	TBIL	0.838407	0
B134	B138	CHE	PAB	0.3848	9.81E-13
B134	B198	CHE	P	-0.35725	4.57E-11
B134	B137	CHE	AFU	0.337531	5.75E-10
B134	B196	CHE	TCO2	0.319225	5.17E-09
B134	B135	CHE	TBA	-0.28957	1.34E-07
B135	B187	TBA	NSE	-0.48054	0
B136	B195	5'NT	Na	-0.46029	0
B136	B197	5'NT	AG	-0.37993	1.99E-12
B136	B200	5'NT	OP	-0.31923	5.17E-09
B136	B196	5'NT	TCO2	0.288084	1.56E-07
B137	B197	AFU	AG	0.394698	2.25E-13
B137	B139	AFU	ADA	0.365337	1.54E-11
B137	B194	AFU	Cl	-0.32216	3.67E-09
B137	B193	AFU	K	-0.27409	6.38E-07
B138	B196	PAB	TCO2	0.41056	1.91E-14
B138	B139	PAB	ADA	-0.39385	2.56E-13
B139	B197	ADA	AG	0.413938	1.11E-14
B139	B196	ADA	TCO2	-0.36808	1.06E-11
B14	B193	G-17	K	0.580689	0
B14	B200	G-17	OP	0.388098	6.04E-13
B14	B195	G-17	Na	0.282204	2.85E-07
B140	B206	r-GT	Waist	0.338973	4.81E-10
B140	B141	r-GT	AST/ALT	-0.33526	7.61E-10
B140	B207	r-GT	WHR	0.324825	2.68E-09
B140	B216	r-GT	BMI	0.32162	3.91E-09
B140	B192	r-GT	UA	0.310201	1.45E-08
B140	B222	r-GT	NS	0.293377	9.01E-08
B140	B215	r-GT	BMI	0.2907	1.19E-07
B141	B192	AST/ALT	UA	-0.36141	2.62E-11

B141	B216	AST/ALT	BMI	-0.35223	8.86E-11
B141	B206	AST/ALT	Waist	-0.34176	3.39E-10
B141	B173	AST/ALT	NTT	0.320652	4.38E-09
B141	B222	AST/ALT	NS	-0.31748	6.32E-09
B141	B215	AST/ALT	BMI	-0.30309	3.18E-08
B141	B207	AST/ALT	WHR	-0.3026	3.35E-08
B141	B210	AST/ALT	Hip	-0.27142	8.26E-07
B142	B223	25-(OH)D	FH	-0.66667	0
B144	B171	Total-PINP	EC	0.601929	0
B145	B167	BGP	PC	0.830105	0
B145	B171	BGP	EC	0.43093	6.66E-16
B146	B194	CysC	Cl	0.427149	1.33E-15
B146	B196	CysC	TCO2	-0.27633	5.12E-07
B149	B161	TGAb	LEU	0.310471	1.40E-08
B149	B168	TGAb	NMP22	-0.3058	2.36E-08
B15	B100	CD3-CD19+(B cell)	anti-Ro52	0.486695	0
B15	B101	CD3-CD19+(B cell)	anti-Sc70	0.486695	0
B15	B102	CD3-CD19+(B cell)	anti-Sm	0.486695	0
B15	B103	CD3-CD19+(B cell)	anti-SSA/Ro	0.486695	0
B15	B104	CD3-CD19+(B cell)	anti-SSB	0.486695	0
B15	B105	CD3-CD19+(B cell)	anti-U1RNP	0.486695	0
B15	B106	CD3-CD19+(B cell)	anti-P	0.486695	0
B15	B107	CD3-CD19+(B cell)	AnuA	0.486695	0
B15	B108	CD3-CD19+(B cell)	Anti-Cyclin	0.486695	0
B15	B109	CD3-CD19+(B cell)	AMA-M2	0.486695	0
B15	B110	CD3-CD19+(B cell)	AHA	0.486695	0
B15	B139	CD3-CD19+(B cell)	ADA	-0.48595	0
B15	B16	CD3-CD19+(B cell)	CD3+T	-0.99728	0
B15	B17	CD3-CD19+(B cell)	CD3+CD16+CD56+NK	0.995002	0
B15	B18	CD3-CD19+(B cell)	CD3+CD4+helper(inducer)T	0.606256	0
B15	B19	CD3-CD19+(B cell)	CD3+CD4+/CD3+CD8+	0.727366	0
B15	B194	CD3-CD19+(B cell)	Cl	-0.44496	0
B15	B20	CD3-CD19+(B cell)	CD3+CD4+CD8+	0.995002	0
B15	B21	CD3-CD19+(B cell)	CD3+CD4-CD8-	0.995002	0
B15	B22	CD3-CD19+(B cell)	CD3+CD8+(T)	-0.7891	0
B15	B23	CD3-CD19+(B cell)	CD3-CD16+CD56+(NK)	0.777566	0
B15	B95	CD3-CD19+(B cell)	anti-cenpb	0.486695	0
B15	B97	CD3-CD19+(B cell)	dsDNA	0.486695	0
B15	B98	CD3-CD19+(B cell)	anti-Jo-1	0.486695	0
B15	B99	CD3-CD19+(B cell)	anti-PM-Scl	0.486695	0
B15	B196	CD3-CD19+(B cell)	TCO2	0.436496	2.22E-16
B15	B198	CD3-CD19+(B cell)	P	-0.42113	3.55E-15
B15	B200	CD3-CD19+(B cell)	OP	-0.38913	5.18E-13
B15	B168	CD3-CD19+(B cell)	NMP22	0.347119	1.72E-10

B15	B138	CD3-CD19+(B cell)	PAB	0.326999	2.07E-09
B15	B146	CD3-CD19+(B cell)	CysC	-0.32395	2.97E-09
B15	B156	CD3-CD19+(B cell)	IgE	0.28827	1.53E-07
B15	B193	CD3-CD19+(B cell)	K	-0.28606	1.93E-07
B151	B154	Anti-CCP	RF	-0.34423	2.48E-10
B154	B156	RF	IgE	0.400827	8.84E-14
B154	B184	RF	INR	0.351678	9.52E-11
B154	B182	RF	TT	-0.29816	5.42E-08
B155	B198	IgA	P	-0.29516	7.47E-08
B156	B184	IgE	INR	0.371871	6.23E-12
B156	B186	IgE	PFC	0.290467	1.22E-07
B156	B182	IgE	TT	-0.27192	7.87E-07
B159	B169	β2-MG	U-CRY	0.999998	0
B16	B100	CD3+T	anti-Ro52	-0.51477	0
B16	B101	CD3+T	anti-Sc70	-0.51477	0
B16	B102	CD3+T	anti-Sm	-0.51477	0
B16	B103	CD3+T	anti-SSA/Ro	-0.51477	0
B16	B104	CD3+T	anti-SSB	-0.51477	0
B16	B105	CD3+T	anti-U1RNP	-0.51477	0
B16	B106	CD3+T	anti-P	-0.51477	0
B16	B107	CD3+T	AnuA	-0.51477	0
B16	B108	CD3+T	Anti-Cyclin	-0.51477	0
B16	B109	CD3+T	AMA-M2	-0.51477	0
B16	B110	CD3+T	AHA	-0.51477	0
B16	B139	CD3+T	ADA	0.481512	0
B16	B17	CD3+T	CD3+CD16+CD56+NK	-0.98495	0
B16	B18	CD3+T	CD3+CD4+helper(inducer)T	-0.66317	0
B16	B19	CD3+T	CD3+CD4+/CD3+CD8+	-0.77593	0
B16	B194	CD3+T	Cl	0.440277	0
B16	B20	CD3+T	CD3+CD4+CD8+	-0.98495	0
B16	B21	CD3+T	CD3+CD4-CD8-	-0.98495	0
B16	B22	CD3+T	CD3+CD8+(T)	0.832191	0
B16	B23	CD3+T	CD3-CD16+CD56+(NK)	-0.82176	0
B16	B95	CD3+T	anti-cenpb	-0.51477	0
B16	B97	CD3+T	dsDNA	-0.51477	0
B16	B98	CD3+T	anti-Jo-1	-0.51477	0
B16	B99	CD3+T	anti-PM-Scl	-0.51477	0
B16	B196	CD3+T	TCO2	-0.43209	4.44E-16
B16	B198	CD3+T	P	0.416855	7.11E-15
B16	B200	CD3+T	OP	0.38504	9.47E-13
B16	B168	CD3+T	NMP22	-0.34303	2.89E-10
B16	B138	CD3+T	PAB	-0.32382	3.02E-09
B16	B146	CD3+T	CysC	0.320622	4.39E-09
B16	B193	CD3+T	K	0.28301	2.63E-07

B16	B156	CD3+T	IgE	-0.27834	4.19E-07
B161	B173	LEU	NTT	0.413424	1.20E-14
B163	B166	SG	Pro	0.467708	0
B163	B190	SG	Cre	0.497867	0
B163	B216	SG	BMI	0.380977	1.71E-12
B163	B222	SG	NS	0.376617	3.19E-12
B163	B192	SG	UA	0.3593	3.48E-11
B163	B221	SG	Hei	0.359242	3.51E-11
B163	B191	SG	Urea	0.34924	1.31E-10
B163	B206	SG	Waist	0.298967	4.97E-08
B163	B207	SG	WHR	0.278147	4.27E-07
B168	B198	NMP22	P	-0.31963	4.93E-09
B17	B100	CD3+CD16+CD56+NK	anti-Ro52	0.444401	0
B17	B101	CD3+CD16+CD56+NK	anti-Sc70	0.444401	0
B17	B102	CD3+CD16+CD56+NK	anti-Sm	0.444401	0
B17	B103	CD3+CD16+CD56+NK	anti-SSA/Ro	0.444401	0
B17	B104	CD3+CD16+CD56+NK	anti-SSB	0.444401	0
B17	B105	CD3+CD16+CD56+NK	anti-U1RNP	0.444401	0
B17	B106	CD3+CD16+CD56+NK	anti-P	0.444401	0
B17	B107	CD3+CD16+CD56+NK	AnuA	0.444401	0
B17	B108	CD3+CD16+CD56+NK	Anti-Cyclin	0.444401	0
B17	B109	CD3+CD16+CD56+NK	AMA-M2	0.444401	0
B17	B110	CD3+CD16+CD56+NK	AHA	0.444401	0
B17	B139	CD3+CD16+CD56+NK	ADA	-0.48774	0
B17	B18	CD3+CD16+CD56+NK	CD3+CD4+helper(inducer)T	0.523814	0
B17	B19	CD3+CD16+CD56+NK	CD3+CD4+/CD3+CD8+	0.655205	0
B17	B194	CD3+CD16+CD56+NK	Cl	-0.44745	0
B17	B22	CD3+CD16+CD56+NK	CD3+CD8+(T)	-0.72382	0
B17	B23	CD3+CD16+CD56+NK	CD3-CD16+CD56+(NK)	0.710891	0
B17	B95	CD3+CD16+CD56+NK	anti-cenpb	0.444401	0
B17	B97	CD3+CD16+CD56+NK	dsDNA	0.444401	0
B17	B98	CD3+CD16+CD56+NK	anti-Jo-1	0.444401	0
B17	B99	CD3+CD16+CD56+NK	anti-PM-Scl	0.444401	0
B17	B196	CD3+CD16+CD56+NK	TCO2	0.438683	2.22E-16
B17	B198	CD3+CD16+CD56+NK	P	-0.42328	2.44E-15
B17	B200	CD3+CD16+CD56+NK	OP	-0.39131	3.75E-13
B17	B168	CD3+CD16+CD56+NK	NMP22	0.349653	1.24E-10
B17	B138	CD3+CD16+CD56+NK	PAB	0.328466	1.74E-09
B17	B146	CD3+CD16+CD56+NK	CysC	-0.32566	2.43E-09
B17	B156	CD3+CD16+CD56+NK	IgE	0.299231	4.83E-08
B17	B193	CD3+CD16+CD56+NK	K	-0.28772	1.62E-07
B17	B195	CD3+CD16+CD56+NK	Na	-0.2707	8.85E-07
B170	B173	U-GLU	NTT	0.311905	1.19E-08
B174	B191	Urea/Cr	Urea	0.628182	0

B174	B190	Urea/Cr	Cre	-0.41186	1.55E-14
B174	B221	Urea/Cr	Hei	-0.30146	3.80E-08
B175	B177	ALB-U	ACR	0.425819	1.55E-15
B175	B176	ALB-U	Ucr	0.372752	5.51E-12
B175	B186	ALB-U	PFC	-0.30854	1.74E-08
B175	B185	ALB-U	PT	0.28369	2.45E-07
B176	B177	Ucr	ACR	-0.44914	0
B176	B179	Ucr	α1-MG	0.320637	4.39E-09
B177	B179	ACR	α1-MG	-0.38681	7.31E-13
B178	B180	Ig-U	Tf	0.713474	0
B179	B180	α1-MG	Tf	-0.28444	2.27E-07
B18	B100	CD3+CD4+helper(inducer)T	anti-Ro52	0.612527	0
B18	B101	CD3+CD4+helper(inducer)T	anti-Sc70	0.612527	0
B18	B102	CD3+CD4+helper(inducer)T	anti-Sm	0.612527	0
B18	B103	CD3+CD4+helper(inducer)T	anti-SSA/Ro	0.612527	0
B18	B104	CD3+CD4+helper(inducer)T	anti-SSB	0.612527	0
B18	B105	CD3+CD4+helper(inducer)T	anti-U1RNP	0.612527	0
B18	B106	CD3+CD4+helper(inducer)T	anti-P	0.612527	0
B18	B107	CD3+CD4+helper(inducer)T	AnuA	0.612527	0
B18	B108	CD3+CD4+helper(inducer)T	Anti-Cyclin	0.612527	0
B18	B109	CD3+CD4+helper(inducer)T	AMA-M2	0.612527	0
B18	B110	CD3+CD4+helper(inducer)T	AHA	0.612527	0
B18	B178	CD3+CD4+helper(inducer)T	Ig-U	0.55863	0
B18	B180	CD3+CD4+helper(inducer)T	Tf	0.447747	0
B18	B19	CD3+CD4+helper(inducer)T	CD3+CD4+/CD3+CD8+	0.986724	0
B18	B20	CD3+CD4+helper(inducer)T	CD3+CD4+CD8+	0.523814	0
B18	B21	CD3+CD4+helper(inducer)T	CD3+CD4-CD8-	0.523814	0
B18	B22	CD3+CD4+helper(inducer)T	CD3+CD8+(T)	-0.9669	0
B18	B23	CD3+CD4+helper(inducer)T	CD3-CD16+CD56+(NK)	0.97147	0
B18	B92	CD3+CD4+helper(inducer)T	ALDH2-Glu504Glu	-0.59906	0
B18	B93	CD3+CD4+helper(inducer)T	ALDH2-Glu504Lys	0.599057	0
B18	B94	CD3+CD4+helper(inducer)T	ALDH2-Lys504Lys	0.599057	0
B18	B95	CD3+CD4+helper(inducer)T	anti-cenpb	0.612527	0
B18	B97	CD3+CD4+helper(inducer)T	dsDNA	0.612527	0
B18	B98	CD3+CD4+helper(inducer)T	anti-Jo-1	0.612527	0
B18	B99	CD3+CD4+helper(inducer)T	anti-PM-Scl	0.612527	0
B18	B201	CD3+CD4+helper(inducer)T	e-habits	-0.30709	2.05E-08
B18	B154	CD3+CD4+helper(inducer)T	RF	0.289714	1.32E-07
B18	B24	CD3+CD4+helper(inducer)T	αHBDH	0.285775	1.98E-07
B180	B187	Tf	NSE	0.301135	3.93E-08
B181	B183	APTT	PTA	0.738304	0
B181	B184	APTT	INR	0.448265	0
B181	B185	APTT	PT	0.705202	0
B181	B186	APTT	PFC	-0.50427	0

B181	B182	APTT	TT	0.288457	1.51E-07
B182	B186	TT	PFC	-0.81694	0
B182	B184	TT	INR	-0.36012	3.12E-11
B183	B184	PTA	INR	0.785348	0
B183	B185	PTA	PT	0.817879	0
B184	B185	INR	PT	0.778069	0
B185	B186	PT	PFC	-0.36982	8.30E-12
B187	B227	NSE	OD	0.338432	5.14E-10
B189	B190	eGFB	Cre	-0.52861	0
B19	B100	CD3+CD4+/CD3+CD8+	anti-Ro52	0.627951	0
B19	B101	CD3+CD4+/CD3+CD8+	anti-Sc70	0.627951	0
B19	B102	CD3+CD4+/CD3+CD8+	anti-Sm	0.627951	0
B19	B103	CD3+CD4+/CD3+CD8+	anti-SSA/Ro	0.627951	0
B19	B104	CD3+CD4+/CD3+CD8+	anti-SSB	0.627951	0
B19	B105	CD3+CD4+/CD3+CD8+	anti-U1RNP	0.627951	0
B19	B106	CD3+CD4+/CD3+CD8+	anti-P	0.627951	0
B19	B107	CD3+CD4+/CD3+CD8+	AnuA	0.627951	0
B19	B108	CD3+CD4+/CD3+CD8+	Anti-Cyclin	0.627951	0
B19	B109	CD3+CD4+/CD3+CD8+	AMA-M2	0.627951	0
B19	B110	CD3+CD4+/CD3+CD8+	AHA	0.627951	0
B19	B178	CD3+CD4+/CD3+CD8+	Ig-U	0.494595	0
B19	B20	CD3+CD4+/CD3+CD8+	CD3+CD4+CD8+	0.655205	0
B19	B21	CD3+CD4+/CD3+CD8+	CD3+CD4-CD8-	0.655205	0
B19	B22	CD3+CD4+/CD3+CD8+	CD3+CD8+(T)	-0.9955	0
B19	B23	CD3+CD4+/CD3+CD8+	CD3-CD16+CD56+(NK)	0.99709	0
B19	B92	CD3+CD4+/CD3+CD8+	ALDH2-Glu504Glu	-0.53043	0
B19	B93	CD3+CD4+/CD3+CD8+	ALDH2-Glu504Lys	0.53043	0
B19	B94	CD3+CD4+/CD3+CD8+	ALDH2-Lys504Lys	0.53043	0
B19	B95	CD3+CD4+/CD3+CD8+	anti-cenpb	0.627951	0
B19	B97	CD3+CD4+/CD3+CD8+	dsDNA	0.627951	0
B19	B98	CD3+CD4+/CD3+CD8+	anti-Jo-1	0.627951	0
B19	B99	CD3+CD4+/CD3+CD8+	anti-PM-Scl	0.627951	0
B19	B180	CD3+CD4+/CD3+CD8+	Tf	0.395889	1.88E-13
B19	B139	CD3+CD4+/CD3+CD8+	ADA	-0.32443	2.81E-09
B19	B201	CD3+CD4+/CD3+CD8+	e-habits	-0.31482	8.58E-09
B19	B194	CD3+CD4+/CD3+CD8+	Cl	-0.29124	1.13E-07
B19	B196	CD3+CD4+/CD3+CD8+	TCO2	0.287466	1.67E-07
B19	B154	CD3+CD4+/CD3+CD8+	RF	0.286866	1.77E-07
B19	B198	CD3+CD4+/CD3+CD8+	P	-0.27709	4.75E-07
B190	B192	Cre	UA	0.498771	0
B190	B216	Cre	BMI	0.487162	0
B190	B221	Cre	Hei	0.467129	0
B190	B222	Cre	NS	0.508297	0
B190	B206	Cre	Waist	0.405061	4.57E-14

B190	B191	Cre	Urea	0.401101	8.48E-14
B190	B207	Cre	WHR	0.361009	2.77E-11
B190	B210	Cre	Hip	0.329611	1.51E-09
B190	B215	Cre	BMI	0.313742	9.70E-09
B192	B206	UA	Waist	0.466739	0
B192	B216	UA	BMI	0.524312	0
B192	B222	UA	NS	0.531205	0
B192	B210	UA	Hip	0.423064	2.66E-15
B192	B221	UA	Hei	0.420098	4.00E-15
B192	B215	UA	BMI	0.395487	2.00E-13
B192	B207	UA	WHR	0.366589	1.30E-11
B192	B204	UA	Drinking	0.3147	8.70E-09
B192	B218	UA	DBP	0.272358	7.54E-07
B193	B195	K	Na	0.563333	0
B193	B200	K	OP	0.659558	0
B193	B197	K	AG	0.274893	5.89E-07
B194	B196	Cl	TCO2	-0.51003	0
B194	B199	Cl	Mg	0.375295	3.85E-12
B195	B197	Na	AG	0.490043	0
B195	B200	Na	OP	0.937903	0
B195	B198	Na	P	-0.33241	1.08E-09
B196	B197	TCO2	AG	-0.6518	0
B196	B199	TCO2	Mg	-0.29047	1.22E-07
B197	B200	AG	OP	0.440121	0
B198	B200	P	OP	-0.28446	2.27E-07
B2	B189	Age	eGFB	-0.50502	0
B2	B47	Age	RDW	0.339152	4.70E-10
B2	B126	Age	Alb	-0.3306	1.34E-09
B2	B207	Age	WHR	0.31187	1.20E-08
B2	B153	Age	Glu	0.27308	7.03E-07
B20	B100	CD3+CD4+CD8+	anti-Ro52	0.444401	0
B20	B101	CD3+CD4+CD8+	anti-Sc70	0.444401	0
B20	B102	CD3+CD4+CD8+	anti-Sm	0.444401	0
B20	B103	CD3+CD4+CD8+	anti-SSA/Ro	0.444401	0
B20	B104	CD3+CD4+CD8+	anti-SSB	0.444401	0
B20	B105	CD3+CD4+CD8+	anti-U1RNP	0.444401	0
B20	B106	CD3+CD4+CD8+	anti-P	0.444401	0
B20	B107	CD3+CD4+CD8+	AnuA	0.444401	0
B20	B108	CD3+CD4+CD8+	Anti-Cyclin	0.444401	0
B20	B109	CD3+CD4+CD8+	AMA-M2	0.444401	0
B20	B110	CD3+CD4+CD8+	AHA	0.444401	0
B20	B139	CD3+CD4+CD8+	ADA	-0.48774	0
B20	B194	CD3+CD4+CD8+	Cl	-0.44745	0
B20	B22	CD3+CD4+CD8+	CD3+CD8+(T)	-0.72382	0

B20	B23	CD3+CD4+CD8+	CD3-CD16+CD56+(NK)	0.710891	0
B20	B95	CD3+CD4+CD8+	anti-cenpb	0.444401	0
B20	B97	CD3+CD4+CD8+	dsDNA	0.444401	0
B20	B98	CD3+CD4+CD8+	anti-Jo-1	0.444401	0
B20	B99	CD3+CD4+CD8+	anti-PM-Scl	0.444401	0
B20	B196	CD3+CD4+CD8+	TCO2	0.438683	2.22E-16
B20	B198	CD3+CD4+CD8+	P	-0.42328	2.44E-15
B20	B200	CD3+CD4+CD8+	OP	-0.39131	3.75E-13
B20	B168	CD3+CD4+CD8+	NMP22	0.349653	1.24E-10
B20	B138	CD3+CD4+CD8+	PAB	0.328466	1.74E-09
B20	B146	CD3+CD4+CD8+	CysC	-0.32566	2.43E-09
B20	B156	CD3+CD4+CD8+	IgE	0.299231	4.83E-08
B20	B193	CD3+CD4+CD8+	K	-0.28772	1.62E-07
B20	B195	CD3+CD4+CD8+	Na	-0.2707	8.85E-07
B204	B222	Drinking	NS	0.308284	1.79E-08
B204	B206	Drinking	Waist	0.282212	2.85E-07
B204	B207	Drinking	WHR	0.278885	3.97E-07
B204	B209	Drinking	Smoking	0.27652	5.02E-07
B204	B216	Drinking	BMI	0.270157	9.33E-07
B206	B207	Waist	WHR	0.871097	0
B206	B210	Waist	Hip	0.804774	0
B206	B215	Waist	BMI	0.82188	0
B206	B216	Waist	BMI	0.846927	0
B206	B221	Waist	Hei	0.442832	0
B206	B222	Waist	NS	0.779891	0
B206	B220	Waist	SBP	0.334219	8.65E-10
B206	B218	Waist	DBP	0.329201	1.59E-09
B207	B215	WHR	BMI	0.613408	0
B207	B216	WHR	BMI	0.610514	0
B207	B222	WHR	NS	0.623749	0
B207	B210	WHR	Hip	0.413063	1.29E-14
B207	B218	WHR	DBP	0.302349	3.45E-08
B207	B221	WHR	Hei	0.298915	5.00E-08
B21	B100	CD3+CD4-CD8-	anti-Ro52	0.444401	0
B21	B101	CD3+CD4-CD8-	anti-Sc70	0.444401	0
B21	B102	CD3+CD4-CD8-	anti-Sm	0.444401	0
B21	B103	CD3+CD4-CD8-	anti-SSA/Ro	0.444401	0
B21	B104	CD3+CD4-CD8-	anti-SSB	0.444401	0
B21	B105	CD3+CD4-CD8-	anti-U1RNP	0.444401	0
B21	B106	CD3+CD4-CD8-	anti-P	0.444401	0
B21	B107	CD3+CD4-CD8-	AnuA	0.444401	0
B21	B108	CD3+CD4-CD8-	Anti-Cyclin	0.444401	0
B21	B109	CD3+CD4-CD8-	AMA-M2	0.444401	0
B21	B110	CD3+CD4-CD8-	AHA	0.444401	0

B21	B139	CD3+CD4-CD8-	ADA	-0.48774	0
B21	B194	CD3+CD4-CD8-	Cl	-0.44745	0
B21	B22	CD3+CD4-CD8-	CD3+CD8+(T)	-0.72382	0
B21	B23	CD3+CD4-CD8-	CD3-CD16+CD56+(NK)	0.710891	0
B21	B95	CD3+CD4-CD8-	anti-cenpb	0.444401	0
B21	B97	CD3+CD4-CD8-	dsDNA	0.444401	0
B21	B98	CD3+CD4-CD8-	anti-Jo-1	0.444401	0
B21	B99	CD3+CD4-CD8-	anti-PM-Scl	0.444401	0
B21	B196	CD3+CD4-CD8-	TCO2	0.438683	2.22E-16
B21	B198	CD3+CD4-CD8-	P	-0.42328	2.44E-15
B21	B200	CD3+CD4-CD8-	OP	-0.39131	3.75E-13
B21	B168	CD3+CD4-CD8-	NMP22	0.349653	1.24E-10
B21	B138	CD3+CD4-CD8-	PAB	0.328466	1.74E-09
B21	B146	CD3+CD4-CD8-	CysC	-0.32566	2.43E-09
B21	B156	CD3+CD4-CD8-	IgE	0.299231	4.83E-08
B21	B193	CD3+CD4-CD8-	K	-0.28772	1.62E-07
B21	B195	CD3+CD4-CD8-	Na	-0.2707	8.85E-07
B210	B215	Hip	BMI	0.784583	0
B210	B216	Hip	BMI	0.837874	0
B210	B221	Hip	Hei	0.470654	0
B210	B222	Hip	NS	0.697292	0
B210	B220	Hip	SBP	0.339506	4.50E-10
B211	B212	IOP-R	IOP-L	0.542019	0
B211	B227	IOP-R	OD	0.558799	0
B212	B225	IOP-L	hPBG	0.278502	4.13E-07
B213	B214	CVA-R	CVA-L	0.766352	0
B215	B216	BMI	BMI	0.824366	0
B215	B222	BMI	NS	0.724876	0
B215	B220	BMI	SBP	0.358288	3.98E-11
B215	B218	BMI	DBP	0.297426	5.86E-08
B216	B221	BMI	Hei	0.69468	0
B216	B222	BMI	NS	0.823286	0
B216	B220	BMI	SBP	0.36009	3.13E-11
B216	B218	BMI	DBP	0.333962	8.93E-10
B218	B220	DBP	SBP	0.679355	0
B218	B222	DBP	NS	0.29657	6.42E-08
B22	B100	CD3+CD8+(T)	anti-Ro52	-0.62926	0
B22	B101	CD3+CD8+(T)	anti-Sc70	-0.62926	0
B22	B102	CD3+CD8+(T)	anti-Sm	-0.62926	0
B22	B103	CD3+CD8+(T)	anti-SSA/Ro	-0.62926	0
B22	B104	CD3+CD8+(T)	anti-SSB	-0.62926	0
B22	B105	CD3+CD8+(T)	anti-U1RNP	-0.62926	0
B22	B106	CD3+CD8+(T)	anti-P	-0.62926	0
B22	B107	CD3+CD8+(T)	AnuA	-0.62926	0

B22	B108	CD3+CD8+(T)	Anti-Cyclin	-0.62926	0
B22	B109	CD3+CD8+(T)	AMA-M2	-0.62926	0
B22	B110	CD3+CD8+(T)	AHA	-0.62926	0
B22	B178	CD3+CD8+(T)	Ig-U	-0.45119	0
B22	B23	CD3+CD8+(T)	CD3-CD16+CD56+(NK)	-0.99983	0
B22	B92	CD3+CD8+(T)	ALDH2-Glu504Glu	0.483911	0
B22	B93	CD3+CD8+(T)	ALDH2-Glu504Lys	-0.48391	0
B22	B94	CD3+CD8+(T)	ALDH2-Lys504Lys	-0.48391	0
B22	B95	CD3+CD8+(T)	anti-cenpb	-0.62926	0
B22	B97	CD3+CD8+(T)	dsDNA	-0.62926	0
B22	B98	CD3+CD8+(T)	anti-Jo-1	-0.62926	0
B22	B99	CD3+CD8+(T)	anti-PM-Scl	-0.62926	0
B22	B180	CD3+CD8+(T)	Tf	-0.3608	2.85E-11
B22	B139	CD3+CD8+(T)	ADA	0.357475	4.44E-11
B22	B194	CD3+CD8+(T)	Cl	0.322112	3.69E-09
B22	B196	CD3+CD8+(T)	TCO2	-0.31756	6.26E-09
B22	B201	CD3+CD8+(T)	e-habits	0.315475	7.96E-09
B22	B198	CD3+CD8+(T)	P	0.306156	2.27E-08
B22	B200	CD3+CD8+(T)	OP	0.28173	2.99E-07
B22	B154	CD3+CD8+(T)	RF	-0.28169	3.00E-07
B220	B222	SBP	NS	0.313356	1.01E-08
B221	B222	Hei	NS	0.524854	0
B23	B100	CD3-CD16+CD56+(NK)	anti-Ro52	0.62945	0
B23	B101	CD3-CD16+CD56+(NK)	anti-Sc70	0.62945	0
B23	B102	CD3-CD16+CD56+(NK)	anti-Sm	0.62945	0
B23	B103	CD3-CD16+CD56+(NK)	anti-SSA/Ro	0.62945	0
B23	B104	CD3-CD16+CD56+(NK)	anti-SSB	0.62945	0
B23	B105	CD3-CD16+CD56+(NK)	anti-U1RNP	0.62945	0
B23	B106	CD3-CD16+CD56+(NK)	anti-P	0.62945	0
B23	B107	CD3-CD16+CD56+(NK)	AnuA	0.62945	0
B23	B108	CD3-CD16+CD56+(NK)	Anti-Cyclin	0.62945	0
B23	B109	CD3-CD16+CD56+(NK)	AMA-M2	0.62945	0
B23	B110	CD3-CD16+CD56+(NK)	AHA	0.62945	0
B23	B178	CD3-CD16+CD56+(NK)	Ig-U	0.460014	0
B23	B92	CD3-CD16+CD56+(NK)	ALDH2-Glu504Glu	-0.49337	0
B23	B93	CD3-CD16+CD56+(NK)	ALDH2-Glu504Lys	0.493366	0
B23	B94	CD3-CD16+CD56+(NK)	ALDH2-Lys504Lys	0.493366	0
B23	B95	CD3-CD16+CD56+(NK)	anti-cenpb	0.62945	0
B23	B97	CD3-CD16+CD56+(NK)	dsDNA	0.62945	0
B23	B98	CD3-CD16+CD56+(NK)	anti-Jo-1	0.62945	0
B23	B99	CD3-CD16+CD56+(NK)	anti-PM-Scl	0.62945	0
B23	B180	CD3-CD16+CD56+(NK)	Tf	0.367927	1.08E-11
B23	B139	CD3-CD16+CD56+(NK)	ADA	-0.35126	1.01E-10
B23	B194	CD3-CD16+CD56+(NK)	Cl	-0.31629	7.25E-09

B23	B201	CD3-CD16+CD56+(NK)	e-habits	-0.31557	7.87E-09
B23	B196	CD3-CD16+CD56+(NK)	TCO2	0.311892	1.20E-08
B23	B198	CD3-CD16+CD56+(NK)	P	-0.30068	4.13E-08
B23	B154	CD3-CD16+CD56+(NK)	RF	0.282906	2.65E-07
B23	B200	CD3-CD16+CD56+(NK)	OP	-0.27664	4.96E-07
B24	B27	αHBDH	LDH	0.595163	0
B24	B124	αHBDH	ACTH	0.424802	1.78E-15
B24	B125	αHBDH	Cor	-0.4248	1.78E-15
B24	B178	αHBDH	Ig-U	0.310727	1.36E-08
B24	B198	αHBDH	P	-0.30587	2.34E-08
B24	B79	αHBDH	hb V -dna	-0.2867	1.80E-07
B25	B119	CK	Hcv-rna	-0.56336	0
B25	B26	CK	CKMB	0.636663	0
B25	B34	CK	TK	-0.48711	0
B25	B79	CK	hb V -dna	-0.50801	0
B25	B138	CK	PAB	-0.40851	2.66E-14
B26	B119	CKMB	Hcv-rna	-0.38715	6.95E-13
B26	B138	CKMB	PAB	-0.38599	8.24E-13
B26	B79	CKMB	hb V -dna	-0.30901	1.65E-08
B27	B139	LDH	ADA	0.335072	7.79E-10
B28	B202	PRL	e-times	-0.86658	0
B28	B29	PRL	E2	-0.95229	0
B28	B33	PRL	Prog	-0.38656	7.59E-13
B29	B116	E2	TT3	-0.51232	0
B29	B202	E2	e-times	-0.53182	0
B29	B35	E2	β2-MG	-0.88589	0
B29	B37	E2	BAND	0.456633	0
B29	B38	E2	BAND%	0.611577	0
B30	B145	LH	BGP	0.531162	0
B30	B167	LH	PC	0.919239	0
B30	B171	LH	EC	0.686199	0
B30	B32	LH	Testo	0.436564	2.22E-16
B30	B144	LH	Total-PINP	0.4	1.00E-13
B31	B35	FSH	β2-MG	-0.8761	0
B32	B144	Testo	Total-PINP	-0.60634	0
B33	B116	Prog	TT3	-0.67488	0
B33	B35	Prog	β2-MG	-0.92984	0
B34	B124	TK	ACTH	-0.52035	0
B34	B125	TK	Cor	0.520349	0
B34	B79	TK	hb V -dna	0.578798	0
B34	B89	TK	CA 72-4	-0.42459	2.00E-15
B34	B136	TK	5'NT	0.38399	1.10E-12
B34	B92	TK	ALDH2-Glu504Glu	-0.36369	1.93E-11
B34	B93	TK	ALDH2-Glu504Lys	0.363687	1.93E-11

B34	B94	TK	ALDH2-Lys504Lys	0.363687	1.93E-11
B34	B74	TK	ApoA1	0.336984	6.15E-10
B34	B82	TK	CEA	-0.3261	2.30E-09
B34	B187	TK	NSE	0.316734	6.89E-09
B34	B75	TK	ApoA1/B	0.303227	3.13E-08
B34	B119	TK	Hcv-rna	0.280834	3.27E-07
B35	B116	β2-MG	TT3	-0.89474	0
B35	B202	β2-MG	e-times	-0.62439	0
B35	B142	β2-MG	25-(OH)D	-0.42146	3.11E-15
B35	B37	β2-MG	BAND	-0.39736	1.51E-13
B36	B118	V-Lym%	CEA1	-0.53073	0
B36	B170	V-Lym%	U-GLU	-0.50361	0
B36	B42	V-Lym%	MON	-0.2793	3.81E-07
B36	B153	V-Lym%	Glu	-0.27699	4.79E-07
B39	B151	Other cells	Anti-CCP	-0.32477	2.70E-09
B4	B29	WB-Ca	E2	-0.49751	0
B4	B35	WB-Ca	β2-MG	-0.36345	1.99E-11
B40	B42	WBC	MON	0.562033	0
B41	B42	MON %	MON	0.712927	0
B41	B190	MON %	Cre	0.284264	2.31E-07
B42	B192	MON	UA	0.314032	9.38E-09
B42	B190	MON	Cre	0.27533	5.64E-07
B43	B44	MCV	MCH	0.86322	0
B43	B46	MCV	RBC Count	-0.60398	0
B43	B47	MCV	RDW	0.634344	0
B44	B45	MCH	MCHC	0.495116	0
B44	B46	MCH	RBC Count	-0.47162	0
B44	B47	MCH	RDW	0.39738	1.50E-13
B45	B51	MCHC	Hb	0.480258	0
B45	B71	MCHC	TG	0.34837	1.46E-10
B45	B47	MCHC	RDW	-0.30649	2.19E-08
B46	B221	RBC Count	Hei	0.479235	0
B46	B47	RBC Count	RDW	-0.49734	0
B46	B51	RBC Count	Hb	0.756488	0
B46	B190	RBC Count	Cre	0.437282	2.22E-16
B46	B216	RBC Count	BMI	0.437987	2.22E-16
B46	B72	RBC Count	HDL-C	-0.40877	2.55E-14
B46	B222	RBC Count	NS	0.39825	1.31E-13
B46	B163	RBC Count	SG	0.389887	4.64E-13
B46	B192	RBC Count	UA	0.387733	6.38E-13
B46	B206	RBC Count	Waist	0.364965	1.62E-11
B46	B207	RBC Count	WHR	0.326339	2.24E-09
B46	B141	RBC Count	AST/ALT	-0.32109	4.16E-09
B46	B126	RBC Count	Alb	0.299126	4.89E-08

B46	B210	RBC Count	Hip	0.296716	6.33E-08
B46	B128	RBC Count	ALT	0.284418	2.28E-07
B47	B72	RDW	HDL-C	0.274493	6.13E-07
B48	B53	LYM%	GR%	-0.88873	0
B5	B144	WB-Mg	Total-PINP	-0.97991	0
B5	B96	WB-Mg	dsDNA (IIF)	0.835672	0
B5	B171	WB-Mg	EC	0.335754	7.16E-10
B50	B53	eo%	GR%	-0.39532	2.05E-13
B51	B190	Hb	Cre	0.564461	0
B51	B192	Hb	UA	0.441822	0
B51	B216	Hb	BMI	0.494474	0
B51	B221	Hb	Hei	0.541014	0
B51	B222	Hb	NS	0.501976	0
B51	B163	Hb	SG	0.435476	4.44E-16
B51	B206	Hb	Waist	0.405196	4.49E-14
B51	B126	Hb	Alb	0.395553	1.98E-13
B51	B72	Hb	HDL-C	-0.39046	4.26E-13
B51	B207	Hb	WHR	0.364503	1.72E-11
B51	B210	Hb	Hip	0.320818	4.29E-09
B51	B141	Hb	AST/ALT	-0.32034	4.54E-09
B51	B174	Hb	Urea/Cr	-0.30505	2.56E-08
B51	B204	Hb	Drinking	0.289919	1.29E-07
B51	B70	Hb	LDL-C	0.277341	4.63E-07
B54	B56	ESR	IR	-0.68814	0
B54	B57	ESR	RBCAI	-0.46707	0
B54	B58	ESR	HCT	-0.60691	0
B54	B59	ESR	WBRV	-0.53409	0
B54	B60	ESR	WBRVI	-0.74095	0
B54	B61	ESR	WBHSRV	-0.4915	0
B54	B62	ESR	WBHSRVI	-0.67502	0
B54	B63	ESR	WBRSR(mPas_200)	-0.47071	0
B54	B64	ESR	WBRSR(mPas_5)	-0.57069	0
B54	B65	ESR	WBRSR(mPas_1)	-0.60121	0
B54	B66	ESR	WBRSR(mPas_100)	-0.4871	0
B54	B67	ESR	WBRSR(mPas_50)	-0.50751	0
B54	B69	ESR	PV	0.425817	1.55E-15
B54	B55	ESR	TK	-0.41953	4.44E-15
B54	B81	ESR	TSGF	0.368934	9.37E-12
B54	B157	ESR	IgG	0.308752	1.70E-08
B54	B184	ESR	INR	0.283097	2.60E-07
B55	B56	TK	IR	0.667938	0
B55	B61	TK	WBHSRV	0.490637	0
B55	B62	TK	WBHSRVI	0.450018	0
B55	B187	TK	NSE	-0.43143	6.66E-16

B55	B135	TK	TBA	0.390374	4.31E-13
B55	B69	TK	PV	-0.38609	8.12E-13
B55	B124	TK	ACTH	0.310149	1.46E-08
B55	B125	TK	Cor	-0.31015	1.46E-08
B56	B58	IR	HCT	0.779295	0
B56	B59	IR	WBRV	0.525351	0
B56	B60	IR	WBRVI	0.812865	0
B56	B61	IR	WBHSRV	0.876785	0
B56	B62	IR	WBHSRVI	0.964233	0
B56	B63	IR	WBRSR(mPas_200)	0.806988	0
B56	B64	IR	WBRSR(mPas_5)	0.781872	0
B56	B65	IR	WBRSR(mPas_1)	0.725993	0
B56	B66	IR	WBRSR(mPas_100)	0.830883	0
B56	B67	IR	WBRSR(mPas_50)	0.82884	0
B56	B184	IR	INR	-0.32694	2.08E-09
B56	B69	IR	PV	-0.30563	2.41E-08
B56	B182	IR	TT	0.298984	4.96E-08
B56	B154	IR	RF	-0.29661	6.40E-08
B57	B135	RBCAI	TBA	-0.54596	0
B57	B187	RBCAI	NSE	0.442304	0
B57	B59	RBCAI	WBRV	0.725881	0
B57	B60	RBCAI	WBRVI	0.626906	0
B57	B64	RBCAI	WBRSR(mPas_5)	0.451388	0
B57	B65	RBCAI	WBRSR(mPas_1)	0.594047	0
B57	B58	RBCAI	HCT	0.373437	5.00E-12
B57	B90	RBCAI	CYFRA 21-1	-0.31114	1.30E-08
B58	B59	HCT	WBRV	0.662019	0
B58	B60	HCT	WBRVI	0.893316	0
B58	B61	HCT	WBHSRV	0.78676	0
B58	B62	HCT	WBHSRVI	0.913695	0
B58	B63	HCT	WBRSR(mPas_200)	0.886588	0
B58	B64	HCT	WBRSR(mPas_5)	0.925693	0
B58	B65	HCT	WBRSR(mPas_1)	0.904395	0
B58	B66	HCT	WBRSR(mPas_100)	0.906289	0
B58	B67	HCT	WBRSR(mPas_50)	0.916304	0
B59	B60	WBRV	WBRVI	0.799996	0
B59	B61	WBRV	WBHSRV	0.67875	0
B59	B62	WBRV	WBHSRVI	0.591439	0
B59	B63	WBRV	WBRSR(mPas_200)	0.726293	0
B59	B64	WBRV	WBRSR(mPas_5)	0.862532	0
B59	B65	WBRV	WBRSR(mPas_1)	0.91201	0
B59	B66	WBRV	WBRSR(mPas_100)	0.724987	0
B59	B67	WBRV	WBRSR(mPas_50)	0.750626	0
B59	B135	WBRV	TBA	-0.42729	1.33E-15

B59	B187	WBRV	NSE	0.306149	2.27E-08
B59	B69	WBRV	PV	0.279913	3.59E-07
B59	B151	WBRV	Anti-CCP	-0.27375	6.59E-07
B6	B167	WB-Fe	PC	0.532901	0
B60	B61	WBRVI	WBHSRV	0.719481	0
B60	B62	WBRVI	WBHSRVI	0.890608	0
B60	B63	WBRVI	WBRSR(mPas_200)	0.766732	0
B60	B64	WBRVI	WBRSR(mPas_5)	0.90263	0
B60	B65	WBRVI	WBRSR(mPas_1)	0.933549	0
B60	B66	WBRVI	WBRSR(mPas_100)	0.801374	0
B60	B67	WBRVI	WBRSR(mPas_50)	0.824893	0
B60	B157	WBRVI	IgG	-0.28724	1.71E-07
B60	B154	WBRVI	RF	-0.27245	7.47E-07
B61	B62	WBHSRV	WBHSRVI	0.879602	0
B61	B63	WBHSRV	WBRSR(mPas_200)	0.966437	0
B61	B64	WBHSRV	WBRSR(mPas_5)	0.890929	0
B61	B65	WBHSRV	WBRSR(mPas_1)	0.813323	0
B61	B66	WBHSRV	WBRSR(mPas_100)	0.9625	0
B61	B67	WBHSRV	WBRSR(mPas_50)	0.954863	0
B62	B63	WBHSRVI	WBRSR(mPas_200)	0.877479	0
B62	B64	WBHSRVI	WBRSR(mPas_5)	0.878532	0
B62	B65	WBHSRVI	WBRSR(mPas_1)	0.833025	0
B62	B66	WBHSRVI	WBRSR(mPas_100)	0.90436	0
B62	B67	WBHSRVI	WBRSR(mPas_50)	0.906996	0
B62	B154	WBHSRVI	RF	-0.30279	3.28E-08
B62	B184	WBHSRVI	INR	-0.28636	1.87E-07
B62	B182	WBHSRVI	TT	0.281505	3.06E-07
B63	B64	WBRSR(mPas_200)	WBRSR(mPas_5)	0.95202	0
B63	B65	WBRSR(mPas_200)	WBRSR(mPas_1)	0.888221	0
B63	B66	WBRSR(mPas_200)	WBRSR(mPas_100)	0.996585	0
B63	B67	WBRSR(mPas_200)	WBRSR(mPas_50)	0.993896	0
B64	B65	WBRSR(mPas_5)	WBRSR(mPas_1)	0.985809	0
B64	B66	WBRSR(mPas_5)	WBRSR(mPas_100)	0.961735	0
B64	B67	WBRSR(mPas_5)	WBRSR(mPas_50)	0.973528	0
B65	B66	WBRSR(mPas_1)	WBRSR(mPas_100)	0.902179	0
B65	B67	WBRSR(mPas_1)	WBRSR(mPas_50)	0.921486	0
B66	B67	WBRSR(mPas_100)	WBRSR(mPas_50)	0.998857	0
B69	B139	PV	ADA	0.338534	5.08E-10
B69	B135	PV	TBA	-0.30747	1.96E-08
B69	B154	PV	RF	0.293379	9.01E-08
B69	B187	PV	NSE	0.275264	5.68E-07
B7	B202	WB-Cop	e-times	0.7698	0
B7	B28	WB-Cop	PRL	-0.92916	0
B70	B77	LDL-C	TC	0.830768	0

B70	B216	LDL-C	BMI	0.311031	1.32E-08
B70	B222	LDL-C	NS	0.307548	1.95E-08
B70	B206	LDL-C	Waist	0.305998	2.31E-08
B70	B215	LDL-C	BMI	0.277502	4.56E-07
B71	B130	TG	GLB	0.603397	0
B71	B91	TG	TP	0.633478	0
B71	B72	TG	HDL-C	-0.36347	1.98E-11
B71	B77	TG	TC	0.272361	7.54E-07
B72	B192	HDL-C	UA	-0.46586	0
B72	B206	HDL-C	Waist	-0.4574	0
B72	B216	HDL-C	BMI	-0.47092	0
B72	B222	HDL-C	NS	-0.45727	0
B72	B207	HDL-C	WHR	-0.40508	4.57E-14
B72	B215	HDL-C	BMI	-0.39834	1.30E-13
B72	B210	HDL-C	Hip	-0.36331	2.03E-11
B72	B141	HDL-C	AST/ALT	0.357732	4.29E-11
B72	B163	HDL-C	SG	-0.33369	9.23E-10
B72	B221	HDL-C	Hei	-0.32672	2.14E-09
B72	B190	HDL-C	Cre	-0.32029	4.56E-09
B72	B128	HDL-C	ALT	-0.30503	2.57E-08
B72	B74	HDL-C	ApoA1	0.285498	2.04E-07
B73	B197	ApoA	AG	-0.4638	0
B73	B196	ApoA	TCO2	0.433761	4.44E-16
B73	B80	ApoA	anti-HP IgG	0.358822	3.71E-11
B73	B199	ApoA	Mg	-0.33982	4.33E-10
B73	B76	ApoA	ApoB	0.318984	5.31E-09
B73	B79	ApoA	hb V -dna	-0.31396	9.46E-09
B73	B124	ApoA	ACTH	0.312959	1.06E-08
B73	B125	ApoA	Cor	-0.31296	1.06E-08
B73	B195	ApoA	Na	-0.29358	8.82E-08
B73	B120	ApoA	HCV-cAg	-0.29049	1.22E-07
B74	B111	ApoA1	TSH	0.568371	0
B74	B75	ApoA1	ApoA1/B	0.743359	0
B74	B179	ApoA1	α1-MG	-0.3193	5.13E-09
B74	B86	ApoA1	TBhCG	0.29397	8.46E-08
B75	B111	ApoA1/B	TSH	0.531163	0
B75	B76	ApoA1/B	ApoB	-0.71769	0
B76	B136	ApoB	5'NT	0.305357	2.48E-08
B76	B89	ApoB	CA 72-4	0.277113	4.74E-07
B78	B179	HBcAb	α1-MG	0.448025	0
B78	B151	HBcAb	Anti-CCP	-0.29176	1.07E-07
B79	B124	hb V -dna	ACTH	-0.58967	0
B79	B125	hb V -dna	Cor	0.589668	0
B79	B92	hb V -dna	ALDH2-Glu504Glu	-0.41376	1.15E-14

B79	B93	hb V -dna	ALDH2-Glu504Lys	0.413758	1.15E-14
B79	B94	hb V -dna	ALDH2-Lys504Lys	0.413758	1.15E-14
B79	B187	hb V -dna	NSE	0.359986	3.17E-11
B79	B119	hb V -dna	Hcv-rna	0.310037	1.47E-08
B79	B180	hb V -dna	Tf	0.286223	1.89E-07
B79	B136	hb V -dna	5'NT	0.277688	4.47E-07
B8	B145	WB-Zinc	BGP	-0.36148	2.60E-11
B81	B124	TSGF	ACTH	0.328442	1.74E-09
B81	B125	TSGF	Cor	-0.32844	1.74E-09
B82	B84	CEA	C-PSA	0.568462	0
B82	B89	CEA	CA 72-4	0.451178	0
B82	B194	CEA	Cl	0.391901	3.43E-13
B82	B196	CEA	TCO2	-0.27632	5.12E-07
B84	B199	C-PSA	Mg	0.369544	8.61E-12
B84	B196	C-PSA	TCO2	-0.27	9.47E-07
B85	B88	SCC	CA 242	0.325437	2.49E-09
B86	B90	TBhCG	CYFRA 21-1	0.64706	0
B86	B88	TBhCG	CA 242	0.424259	2.22E-15
B86	B199	TBhCG	Mg	-0.28822	1.54E-07
B87	B137	NSE	AFU	0.48171	0
B87	B120	NSE	HCV-cAg	-0.35053	1.11E-10
B87	B194	NSE	Cl	-0.28456	2.24E-07
B88	B157	CA 242	IgG	0.329408	1.55E-09
B90	B135	CYFRA 21-1	TBA	0.337916	5.48E-10
B91	B126	TP	Alb	0.452619	0
B91	B127	TP	A-G ratio	-0.53508	0
B91	B130	TP	GLB	0.881668	0
B92	B124	ALDH2-Glu504Glu	ACTH	0.705998	0
B92	B125	ALDH2-Glu504Glu	Cor	-0.706	0
B92	B180	ALDH2-Glu504Glu	Tf	-0.72526	0
B92	B94	ALDH2-Glu504Glu	ALDH2-Lys504Lys	-1	0
B92	B187	ALDH2-Glu504Glu	NSE	-0.43196	4.44E-16
B92	B100	ALDH2-Glu504Glu	anti-Ro52	-0.30974	1.52E-08
B92	B101	ALDH2-Glu504Glu	anti-Sc70	-0.30974	1.52E-08
B92	B102	ALDH2-Glu504Glu	anti-Sm	-0.30974	1.52E-08
B92	B103	ALDH2-Glu504Glu	anti-SSA/Ro	-0.30974	1.52E-08
B92	B104	ALDH2-Glu504Glu	anti-SSB	-0.30974	1.52E-08
B92	B105	ALDH2-Glu504Glu	anti-U1RNP	-0.30974	1.52E-08
B92	B106	ALDH2-Glu504Glu	anti-P	-0.30974	1.52E-08
B92	B107	ALDH2-Glu504Glu	AnuA	-0.30974	1.52E-08
B92	B108	ALDH2-Glu504Glu	Anti-Cyclin	-0.30974	1.52E-08
B92	B109	ALDH2-Glu504Glu	AMA-M2	-0.30974	1.52E-08
B92	B110	ALDH2-Glu504Glu	AHA	-0.30974	1.52E-08
B92	B95	ALDH2-Glu504Glu	anti-cenpb	-0.30974	1.52E-08

B92	B97	ALDH2-Glu504Glu	dsDNA	-0.30974	1.52E-08
B92	B98	ALDH2-Glu504Glu	anti-Jo-1	-0.30974	1.52E-08
B92	B99	ALDH2-Glu504Glu	anti-PM-Scl	-0.30974	1.52E-08
B92	B178	ALDH2-Glu504Glu	Ig-U	-0.3068	2.12E-08
B92	B154	ALDH2-Glu504Glu	RF	-0.28724	1.71E-07
B93	B124	ALDH2-Glu504Lys	ACTH	-0.706	0
B93	B125	ALDH2-Glu504Lys	Cor	0.705998	0
B93	B180	ALDH2-Glu504Lys	Tf	0.725264	0
B93	B94	ALDH2-Glu504Lys	ALDH2-Lys504Lys	1	0
B93	B187	ALDH2-Glu504Lys	NSE	0.431963	4.44E-16
B93	B100	ALDH2-Glu504Lys	anti-Ro52	0.309737	1.52E-08
B93	B101	ALDH2-Glu504Lys	anti-Sc70	0.309737	1.52E-08
B93	B102	ALDH2-Glu504Lys	anti-Sm	0.309737	1.52E-08
B93	B103	ALDH2-Glu504Lys	anti-SSA/Ro	0.309737	1.52E-08
B93	B104	ALDH2-Glu504Lys	anti-SSB	0.309737	1.52E-08
B93	B105	ALDH2-Glu504Lys	anti-U1RNP	0.309737	1.52E-08
B93	B106	ALDH2-Glu504Lys	anti-P	0.309737	1.52E-08
B93	B107	ALDH2-Glu504Lys	AnuA	0.309737	1.52E-08
B93	B108	ALDH2-Glu504Lys	Anti-Cyclin	0.309737	1.52E-08
B93	B109	ALDH2-Glu504Lys	AMA-M2	0.309737	1.52E-08
B93	B110	ALDH2-Glu504Lys	AHA	0.309737	1.52E-08
B93	B95	ALDH2-Glu504Lys	anti-cenpb	0.309737	1.52E-08
B93	B97	ALDH2-Glu504Lys	dsDNA	0.309737	1.52E-08
B93	B98	ALDH2-Glu504Lys	anti-Jo-1	0.309737	1.52E-08
B93	B99	ALDH2-Glu504Lys	anti-PM-Scl	0.309737	1.52E-08
B93	B178	ALDH2-Glu504Lys	Ig-U	0.306795	2.12E-08
B93	B154	ALDH2-Glu504Lys	RF	0.287243	1.71E-07
B94	B124	ALDH2-Lys504Lys	ACTH	-0.706	0
B94	B125	ALDH2-Lys504Lys	Cor	0.705998	0
B94	B180	ALDH2-Lys504Lys	Tf	0.725264	0
B94	B187	ALDH2-Lys504Lys	NSE	0.431963	4.44E-16
B94	B100	ALDH2-Lys504Lys	anti-Ro52	0.309737	1.52E-08
B94	B101	ALDH2-Lys504Lys	anti-Sc70	0.309737	1.52E-08
B94	B102	ALDH2-Lys504Lys	anti-Sm	0.309737	1.52E-08
B94	B103	ALDH2-Lys504Lys	anti-SSA/Ro	0.309737	1.52E-08
B94	B104	ALDH2-Lys504Lys	anti-SSB	0.309737	1.52E-08
B94	B105	ALDH2-Lys504Lys	anti-U1RNP	0.309737	1.52E-08
B94	B106	ALDH2-Lys504Lys	anti-P	0.309737	1.52E-08
B94	B107	ALDH2-Lys504Lys	AnuA	0.309737	1.52E-08
B94	B108	ALDH2-Lys504Lys	Anti-Cyclin	0.309737	1.52E-08
B94	B109	ALDH2-Lys504Lys	AMA-M2	0.309737	1.52E-08
B94	B110	ALDH2-Lys504Lys	AHA	0.309737	1.52E-08
B94	B95	ALDH2-Lys504Lys	anti-cenpb	0.309737	1.52E-08
B94	B97	ALDH2-Lys504Lys	dsDNA	0.309737	1.52E-08

B94	B98	ALDH2-Lys504Lys	anti-Jo-1	0.309737	1.52E-08
B94	B99	ALDH2-Lys504Lys	anti-PM-Scl	0.309737	1.52E-08
B94	B178	ALDH2-Lys504Lys	Ig-U	0.306795	2.12E-08
B94	B154	ALDH2-Lys504Lys	RF	0.287243	1.71E-07
B95	B101	anti-cenpb	anti-Sc70	1	0
B95	B106	anti-cenpb	anti-P	1	0
B95	B107	anti-cenpb	AnuA	1	0
B95	B108	anti-cenpb	Anti-Cyclin	1	0
B95	B109	anti-cenpb	AMA-M2	1	0
B95	B151	anti-cenpb	Anti-CCP	-0.62933	0
B95	B98	anti-cenpb	anti-Jo-1	1	0
B95	B154	anti-cenpb	RF	0.392478	3.15E-13
B95	B178	anti-cenpb	Ig-U	0.288523	1.49E-07
B96	B167	dsDNA (IIF)	PC	0.954786	0
B97	B100	dsDNA	anti-Ro52	1	0
B97	B103	dsDNA	anti-SSA/Ro	1	0
B97	B105	dsDNA	anti-U1RNP	1	0
B97	B108	dsDNA	Anti-Cyclin	1	0
B97	B109	dsDNA	AMA-M2	1	0
B97	B110	dsDNA	AHA	1	0
B97	B151	dsDNA	Anti-CCP	-0.62933	0
B97	B99	dsDNA	anti-PM-Scl	1	0
B97	B154	dsDNA	RF	0.392478	3.15E-13
B97	B178	dsDNA	Ig-U	0.288523	1.49E-07
B98	B100	anti-Jo-1	anti-Ro52	1	0
B98	B101	anti-Jo-1	anti-Sc70	1	0
B98	B102	anti-Jo-1	anti-Sm	1	0
B98	B103	anti-Jo-1	anti-SSA/Ro	1	0
B98	B104	anti-Jo-1	anti-SSB	1	0
B98	B105	anti-Jo-1	anti-U1RNP	1	0
B98	B107	anti-Jo-1	AnuA	1	0
B98	B109	anti-Jo-1	AMA-M2	1	0
B98	B110	anti-Jo-1	AHA	1	0
B98	B151	anti-Jo-1	Anti-CCP	-0.62933	0
B98	B99	anti-Jo-1	anti-PM-Scl	1	0
B98	B154	anti-Jo-1	RF	0.392478	3.15E-13
B98	B178	anti-Jo-1	Ig-U	0.288523	1.49E-07
B99	B100	anti-PM-Scl	anti-Ro52	1	0
B99	B103	anti-PM-Scl	anti-SSA/Ro	1	0
B99	B105	anti-PM-Scl	anti-U1RNP	1	0
B99	B106	anti-PM-Scl	anti-P	1	0
B99	B107	anti-PM-Scl	AnuA	1	0
B99	B109	anti-PM-Scl	AMA-M2	1	0
B99	B110	anti-PM-Scl	AHA	1	0

B99	B151	anti-PM-Scl	Anti-CCP	-0.62933	0
B99	B154	anti-PM-Scl	RF	0.392478	3.15E-13
B99	B178	anti-PM-Scl	Ig-U	0.288523	1.49E-07

Supplementary Table 16. PEI Correlations in Nephropathy

Row	Column	Row	Column	Cor	*P*
B1	B192	Gender	UA	-0.43349803	0
B1	B206	Gender	Waist	-0.50917524	0
B1	B207	Gender	WHR	-0.44638652	0
B1	B209	Gender	Smoking	-0.33960109	0
B1	B210	Gender	Hip	-0.3802898	0
B1	B216	Gender	BMI	-0.58729438	0
B1	B221	Gender	Hei	-0.65373775	0
B1	B222	Gender	NS	-0.60058387	0
B1	B46	Gender	RBC Count	-0.43865444	0
B1	B51	Gender	Hb	-0.54324201	0
B1	B174	Gender	Urea/Cr	0.331987769	2.22E-16
B1	B42	Gender	MON	-0.32035903	3.11E-15
B1	B72	Gender	HDL-C	0.315452713	8.88E-15
B1	B215	Gender	BMI	-0.30264881	1.15E-13
B1	B163	Gender	SG	-0.29787673	2.88E-13
B1	B45	Gender	MCHC	-0.29130371	9.94E-13
B1	B141	Gender	AST/ALT	0.265651016	9.26E-11
B1	B204	Gender	Drinking	-0.25697298	3.87E-10
B1	B40	Gender	WBC	-0.23056764	2.18E-08
B1	B140	Gender	r-GT	-0.22805989	3.12E-08
B1	B128	Gender	ALT	-0.21822647	1.22E-07
B10	B12	PG I	PG II	0.62879399	0
B10	B145	PG I	BGP	-0.31589593	8.22E-15
B10	B11	PG I	PGI/PGII	0.28877045	1.59E-12
B10	B144	PG I	Total-PINP	-0.27012187	4.35E-11
B10	B189	PG I	eGFB	-0.23350567	1.42E-08
B10	B191	PG I	Urea	0.213518371	2.30E-07
B10	B143	PG I	β-CTx	-0.21027781	3.53E-07
B10	B228	PG I	OS	0.209178274	4.07E-07
B100	B103	anti-Ro52	anti-SSA/Ro	1	0
B100	B104	anti-Ro52	anti-SSB	0.999999998	0
B100	B105	anti-Ro52	anti-U1RNP	1	0
B100	B106	anti-Ro52	anti-P	0.999999998	0
B100	B107	anti-Ro52	AnuA	0.999999975	0
B100	B124	anti-Ro52	ACTH	-0.40646943	0
B100	B125	anti-Ro52	Cor	0.406469422	0
B100	B156	anti-Ro52	IgE	0.379583052	0
B100	B178	anti-Ro52	Ig-U	-0.39099799	0
B100	B180	anti-Ro52	Tf	-0.37436038	0
B100	B184	anti-Ro52	INR	0.310198726	2.58E-14
B100	B185	anti-Ro52	PT	0.293544549	6.54E-13
B100	B214	anti-Ro52	CVA-L	-0.28581501	2.73E-12

B100	B154	anti-Ro52	RF	0.285144149	3.08E-12
B100	B175	anti-Ro52	ALB-U	-0.25006036	1.16E-09
B100	B183	anti-Ro52	PTA	-0.24874269	1.43E-09
B100	B177	anti-Ro52	ACR	-0.24423985	2.86E-09
B100	B196	anti-Ro52	TCO2	0.244131093	2.91E-09
B100	B199	anti-Ro52	Mg	0.234108713	1.30E-08
B100	B195	anti-Ro52	Na	0.228135163	3.09E-08
B100	B182	anti-Ro52	TT	0.219111153	1.08E-07
B100	B151	anti-Ro52	Anti-CCP	0.209182419	4.07E-07
B101	B102	anti-Sc70	anti-Sm	0.999999998	0
B101	B107	anti-Sc70	AnuA	0.999999862	0
B101	B124	anti-Sc70	ACTH	-0.40646945	0
B101	B125	anti-Sc70	Cor	0.406469428	0
B101	B156	anti-Sc70	IgE	0.379583056	0
B101	B178	anti-Sc70	Ig-U	-0.390998	0
B101	B180	anti-Sc70	Tf	-0.37436039	0
B101	B184	anti-Sc70	INR	0.310198725	2.58E-14
B101	B185	anti-Sc70	PT	0.293544547	6.54E-13
B101	B214	anti-Sc70	CVA-L	-0.28581502	2.73E-12
B101	B154	anti-Sc70	RF	0.285144152	3.08E-12
B101	B175	anti-Sc70	ALB-U	-0.25006036	1.16E-09
B101	B183	anti-Sc70	PTA	-0.24874269	1.43E-09
B101	B177	anti-Sc70	ACR	-0.24423985	2.86E-09
B101	B196	anti-Sc70	TCO2	0.244131094	2.91E-09
B101	B199	anti-Sc70	Mg	0.234108715	1.30E-08
B101	B195	anti-Sc70	Na	0.228135159	3.09E-08
B101	B182	anti-Sc70	TT	0.219111154	1.08E-07
B101	B151	anti-Sc70	Anti-CCP	0.209182422	4.07E-07
B102	B104	anti-Sm	anti-SSB	1	0
B102	B107	anti-Sm	AnuA	0.999999997	0
B102	B124	anti-Sm	ACTH	-0.40646944	0
B102	B125	anti-Sm	Cor	0.406469426	0
B102	B156	anti-Sm	IgE	0.379583054	0
B102	B178	anti-Sm	Ig-U	-0.39099799	0
B102	B180	anti-Sm	Tf	-0.37436039	0
B102	B184	anti-Sm	INR	0.310198727	2.58E-14
B102	B185	anti-Sm	PT	0.293544548	6.54E-13
B102	B214	anti-Sm	CVA-L	-0.28581501	2.73E-12
B102	B154	anti-Sm	RF	0.28514415	3.08E-12
B102	B175	anti-Sm	ALB-U	-0.25006036	1.16E-09
B102	B183	anti-Sm	PTA	-0.24874269	1.43E-09
B102	B177	anti-Sm	ACR	-0.24423985	2.86E-09
B102	B196	anti-Sm	TCO2	0.244131093	2.91E-09
B102	B199	anti-Sm	Mg	0.234108714	1.30E-08

B102	B195	anti-Sm	Na	0.228135159	3.09E-08
B102	B182	anti-Sm	TT	0.219111153	1.08E-07
B102	B151	anti-Sm	Anti-CCP	0.20918242	4.07E-07
B103	B104	anti-SSA/Ro	anti-SSB	0.999999998	0
B103	B105	anti-SSA/Ro	anti-U1RNP	1	0
B103	B106	anti-SSA/Ro	anti-P	0.999999998	0
B103	B107	anti-SSA/Ro	AnuA	0.999999985	0
B103	B108	anti-SSA/Ro	Anti-Cyclin	1	0
B103	B124	anti-SSA/Ro	ACTH	-0.40646943	0
B103	B125	anti-SSA/Ro	Cor	0.406469422	0
B103	B156	anti-SSA/Ro	IgE	0.379583052	0
B103	B178	anti-SSA/Ro	Ig-U	-0.39099799	0
B103	B180	anti-SSA/Ro	Tf	-0.37436038	0
B103	B184	anti-SSA/Ro	INR	0.310198726	2.58E-14
B103	B185	anti-SSA/Ro	PT	0.293544549	6.54E-13
B103	B214	anti-SSA/Ro	CVA-L	-0.28581501	2.73E-12
B103	B154	anti-SSA/Ro	RF	0.285144149	3.08E-12
B103	B175	anti-SSA/Ro	ALB-U	-0.25006036	1.16E-09
B103	B183	anti-SSA/Ro	PTA	-0.24874269	1.43E-09
B103	B177	anti-SSA/Ro	ACR	-0.24423985	2.86E-09
B103	B196	anti-SSA/Ro	TCO2	0.244131093	2.91E-09
B103	B199	anti-SSA/Ro	Mg	0.234108713	1.30E-08
B103	B195	anti-SSA/Ro	Na	0.228135162	3.09E-08
B103	B182	anti-SSA/Ro	TT	0.219111153	1.08E-07
B103	B151	anti-SSA/Ro	Anti-CCP	0.209182419	4.07E-07
B104	B105	anti-SSB	anti-U1RNP	0.999999997	0
B104	B107	anti-SSB	AnuA	0.999999974	0
B104	B108	anti-SSB	Anti-Cyclin	0.999999998	0
B104	B109	anti-SSB	AMA-M2	0.999999998	0
B104	B110	anti-SSB	AHA	0.999999999	0
B104	B124	anti-SSB	ACTH	-0.40646942	0
B104	B125	anti-SSB	Cor	0.406469423	0
B104	B156	anti-SSB	IgE	0.379583052	0
B104	B178	anti-SSB	Ig-U	-0.39099799	0
B104	B180	anti-SSB	Tf	-0.37436038	0
B104	B184	anti-SSB	INR	0.310198725	2.58E-14
B104	B185	anti-SSB	PT	0.293544549	6.54E-13
B104	B214	anti-SSB	CVA-L	-0.28581501	2.73E-12
B104	B154	anti-SSB	RF	0.285144148	3.08E-12
B104	B175	anti-SSB	ALB-U	-0.25006036	1.16E-09
B104	B183	anti-SSB	PTA	-0.24874269	1.43E-09
B104	B177	anti-SSB	ACR	-0.24423985	2.86E-09
B104	B196	anti-SSB	TCO2	0.244131092	2.91E-09
B104	B199	anti-SSB	Mg	0.234108713	1.30E-08

B104	B195	anti-SSB	Na	0.228135162	3.09E-08
B104	B182	anti-SSB	TT	0.219111153	1.08E-07
B104	B151	anti-SSB	Anti-CCP	0.209182419	4.07E-07
B105	B106	anti-U1RNP	anti-P	0.999999995	0
B105	B107	anti-U1RNP	AnuA	0.999999973	0
B105	B108	anti-U1RNP	Anti-Cyclin	1	0
B105	B109	anti-U1RNP	AMA-M2	0.999999998	0
B105	B124	anti-U1RNP	ACTH	-0.40646943	0
B105	B125	anti-U1RNP	Cor	0.406469422	0
B105	B156	anti-U1RNP	IgE	0.379583052	0
B105	B178	anti-U1RNP	Ig-U	-0.39099799	0
B105	B180	anti-U1RNP	Tf	-0.37436038	0
B105	B184	anti-U1RNP	INR	0.310198726	2.58E-14
B105	B185	anti-U1RNP	PT	0.293544549	6.54E-13
B105	B214	anti-U1RNP	CVA-L	-0.28581501	2.73E-12
B105	B154	anti-U1RNP	RF	0.285144149	3.08E-12
B105	B175	anti-U1RNP	ALB-U	-0.25006036	1.16E-09
B105	B183	anti-U1RNP	PTA	-0.24874269	1.43E-09
B105	B177	anti-U1RNP	ACR	-0.24423985	2.86E-09
B105	B196	anti-U1RNP	TCO2	0.244131093	2.91E-09
B105	B199	anti-U1RNP	Mg	0.234108713	1.30E-08
B105	B195	anti-U1RNP	Na	0.228135162	3.09E-08
B105	B182	anti-U1RNP	TT	0.219111153	1.08E-07
B105	B151	anti-U1RNP	Anti-CCP	0.209182419	4.07E-07
B106	B108	anti-P	Anti-Cyclin	0.999999999	0
B106	B109	anti-P	AMA-M2	0.999999998	0
B106	B110	anti-P	AHA	0.999999997	0
B106	B124	anti-P	ACTH	-0.40646943	0
B106	B125	anti-P	Cor	0.406469426	0
B106	B156	anti-P	IgE	0.379583052	0
B106	B178	anti-P	Ig-U	-0.39099799	0
B106	B180	anti-P	Tf	-0.37436038	0
B106	B184	anti-P	INR	0.310198726	2.58E-14
B106	B185	anti-P	PT	0.293544547	6.54E-13
B106	B214	anti-P	CVA-L	-0.28581501	2.73E-12
B106	B154	anti-P	RF	0.285144149	3.08E-12
B106	B175	anti-P	ALB-U	-0.25006036	1.16E-09
B106	B183	anti-P	PTA	-0.24874269	1.43E-09
B106	B177	anti-P	ACR	-0.24423985	2.86E-09
B106	B196	anti-P	TCO2	0.244131092	2.91E-09
B106	B199	anti-P	Mg	0.234108713	1.30E-08
B106	B195	anti-P	Na	0.228135162	3.09E-08
B106	B182	anti-P	TT	0.219111154	1.08E-07
B106	B151	anti-P	Anti-CCP	0.209182419	4.07E-07

B107	B108	AnuA	Anti-Cyclin	0.999999962	0
B107	B109	AnuA	AMA-M2	0.999999972	0
B107	B110	AnuA	AHA	0.999999987	0
B107	B124	AnuA	ACTH	-0.40646949	0
B107	B125	AnuA	Cor	0.406469424	0
B107	B156	AnuA	IgE	0.379583045	0
B107	B178	AnuA	Ig-U	-0.39099798	0
B107	B180	AnuA	Tf	-0.37436038	0
B107	B184	AnuA	INR	0.31019871	2.58E-14
B107	B185	AnuA	PT	0.29354453	6.54E-13
B107	B214	AnuA	CVA-L	-0.28581501	2.73E-12
B107	B154	AnuA	RF	0.285144144	3.08E-12
B107	B175	AnuA	ALB-U	-0.25006035	1.16E-09
B107	B183	AnuA	PTA	-0.24874269	1.43E-09
B107	B177	AnuA	ACR	-0.24423984	2.86E-09
B107	B196	AnuA	TCO2	0.244131087	2.91E-09
B107	B199	AnuA	Mg	0.234108706	1.30E-08
B107	B195	AnuA	Na	0.228135131	3.09E-08
B107	B182	AnuA	TT	0.219111147	1.08E-07
B107	B151	AnuA	Anti-CCP	0.209182416	4.07E-07
B108	B110	Anti-Cyclin	AHA	0.999999999	0
B108	B124	Anti-Cyclin	ACTH	-0.40646943	0
B108	B125	Anti-Cyclin	Cor	0.406469423	0
B108	B156	Anti-Cyclin	IgE	0.379583053	0
B108	B178	Anti-Cyclin	Ig-U	-0.39099799	0
B108	B180	Anti-Cyclin	Tf	-0.37436038	0
B108	B184	Anti-Cyclin	INR	0.310198725	2.58E-14
B108	B185	Anti-Cyclin	PT	0.293544549	6.54E-13
B108	B214	Anti-Cyclin	CVA-L	-0.28581501	2.73E-12
B108	B154	Anti-Cyclin	RF	0.285144149	3.08E-12
B108	B175	Anti-Cyclin	ALB-U	-0.25006036	1.16E-09
B108	B183	Anti-Cyclin	PTA	-0.24874269	1.43E-09
B108	B177	Anti-Cyclin	ACR	-0.24423985	2.86E-09
B108	B196	Anti-Cyclin	TCO2	0.244131093	2.91E-09
B108	B199	Anti-Cyclin	Mg	0.234108713	1.30E-08
B108	B195	Anti-Cyclin	Na	0.22813516	3.09E-08
B108	B182	Anti-Cyclin	TT	0.219111153	1.08E-07
B108	B151	Anti-Cyclin	Anti-CCP	0.20918242	4.07E-07
B109	B124	AMA-M2	ACTH	-0.40646943	0
B109	B125	AMA-M2	Cor	0.406469423	0
B109	B156	AMA-M2	IgE	0.379583052	0
B109	B178	AMA-M2	Ig-U	-0.39099799	0
B109	B180	AMA-M2	Tf	-0.37436038	0
B109	B184	AMA-M2	INR	0.310198725	2.58E-14

B109	B185	AMA-M2	PT	0.293544549	6.54E-13
B109	B214	AMA-M2	CVA-L	-0.28581501	2.73E-12
B109	B154	AMA-M2	RF	0.285144149	3.08E-12
B109	B175	AMA-M2	ALB-U	-0.25006036	1.16E-09
B109	B183	AMA-M2	PTA	-0.24874269	1.43E-09
B109	B177	AMA-M2	ACR	-0.24423985	2.86E-09
B109	B196	AMA-M2	TCO2	0.244131093	2.91E-09
B109	B199	AMA-M2	Mg	0.234108713	1.30E-08
B109	B195	AMA-M2	Na	0.228135162	3.09E-08
B109	B182	AMA-M2	TT	0.219111153	1.08E-07
B109	B151	AMA-M2	Anti-CCP	0.209182419	4.07E-07
B11	B12	PGI/PGII	PG II	-0.40370454	0
B11	B80	PGI/PGII	anti-HP IgG	-0.28218686	5.26E-12
B11	B14	PGI/PGII	G-17	-0.25087086	1.02E-09
B11	B175	PGI/PGII	ALB-U	0.219229463	1.07E-07
B110	B124	AHA	ACTH	-0.40646943	0
B110	B125	AHA	Cor	0.406469423	0
B110	B156	AHA	IgE	0.379583053	0
B110	B178	AHA	Ig-U	-0.39099799	0
B110	B180	AHA	Tf	-0.37436038	0
B110	B184	AHA	INR	0.310198725	2.58E-14
B110	B185	AHA	PT	0.293544548	6.54E-13
B110	B214	AHA	CVA-L	-0.28581501	2.73E-12
B110	B154	AHA	RF	0.285144149	3.08E-12
B110	B175	AHA	ALB-U	-0.25006036	1.16E-09
B110	B183	AHA	PTA	-0.24874269	1.43E-09
B110	B177	AHA	ACR	-0.24423985	2.86E-09
B110	B196	AHA	TCO2	0.244131093	2.91E-09
B110	B199	AHA	Mg	0.234108713	1.30E-08
B110	B195	AHA	Na	0.228135162	3.09E-08
B110	B182	AHA	TT	0.219111152	1.08E-07
B110	B151	AHA	Anti-CCP	0.20918242	4.07E-07
B112	B114	FT4	TSH TT3 TT4(TT4)	0.381034742	0
B112	B113	FT4	FT3	0.221525213	7.79E-08
B112	B115	FT4	TSH TT3 TT4(TT3)	0.216804003	1.48E-07
B112	B227	FT4	OD	0.21434938	2.06E-07
B113	B115	FT3	TSH TT3 TT4(TT3)	0.532646094	0
B113	B114	FT3	TSH TT3 TT4(TT4)	0.299745193	2.01E-13
B114	B115	TSH TT3 TT4(TT4)	TSH TT3 TT4(TT3)	0.539614912	0
B117	B167	TT4	PC	-0.34434328	0
B118	B189	CEA1	eGFB	-0.34663862	0
B118	B191	CEA1	Urea	0.371629149	0
B118	B190	CEA1	Cre	0.330611796	4.44E-16
B118	B220	CEA1	SBP	0.215207727	1.84E-07

B119	B120	Hcv-rna	HCV-cAg	0.34921508	0
B119	B134	Hcv-rna	CHE	0.384008425	0
B119	B139	Hcv-rna	ADA	-0.34453101	0
B119	B169	Hcv-rna	U-CRY	-0.99999344	0
B12	B14	PG II	G-17	0.35424304	0
B12	B80	PG II	anti-HP IgG	0.302441466	1.19E-13
B120	B121	HCV-cAg	C3	-0.34921535	0
B120	B122	HCV-cAg	C4	-0.34921542	0
B120	B152	HCV-cAg	ACA-Ig(G-A-M)	-0.34922522	0
B120	B159	HCV-cAg	β2-MG	-0.34921688	0
B120	B169	HCV-cAg	U-CRY	-0.34921278	0
B120	B157	HCV-cAg	IgG	-0.30379152	9.19E-14
B121	B134	C3	CHE	-0.38400851	0
B121	B139	C3	ADA	0.344531076	0
B121	B169	C3	U-CRY	0.999994911	0
B122	B134	C4	CHE	-0.3840085	0
B122	B139	C4	ADA	0.344531069	0
B122	B169	C4	U-CRY	0.999996977	0
B124	B151	ACTH	Anti-CCP	-0.37877757	0
B124	B182	ACTH	TT	0.335197768	0
B124	B184	ACTH	INR	-0.70503348	0
B124	B185	ACTH	PT	-0.7059417	0
B124	B186	ACTH	PFC	-0.38380101	0
B124	B222	ACTH	NS	0.597437357	0
B124	B156	ACTH	IgE	-0.30619534	5.73E-14
B124	B183	ACTH	PTA	-0.29856274	2.53E-13
B124	B155	ACTH	IgA	-0.29356881	6.51E-13
B124	B181	ACTH	APTT	0.275574994	1.69E-11
B124	B135	ACTH	TBA	-0.23820546	7.12E-09
B124	B196	ACTH	TCO2	-0.2154783	1.77E-07
B124	B198	ACTH	P	0.205188628	6.81E-07
B125	B151	Cor	Anti-CCP	0.378777569	0
B125	B182	Cor	TT	-0.33519777	0
B125	B184	Cor	INR	0.705033474	0
B125	B185	Cor	PT	0.705941694	0
B125	B186	Cor	PFC	0.383801003	0
B125	B222	Cor	NS	-0.59743735	0
B125	B156	Cor	IgE	0.306195333	5.73E-14
B125	B183	Cor	PTA	0.298562739	2.53E-13
B125	B155	Cor	IgA	0.293568811	6.51E-13
B125	B181	Cor	APTT	-0.27557499	1.69E-11
B125	B135	Cor	TBA	0.238205454	7.12E-09
B125	B196	Cor	TCO2	0.2154783	1.77E-07
B125	B198	Cor	P	-0.20518863	6.81E-07

B126	B127	Alb	A-G ratio	0.533434841	0
B126	B188	Alb	Ca	0.390742561	0
B126	B189	Alb	eGFB	0.260646454	2.12E-10
B126	B191	Alb	Urea	-0.23468199	1.20E-08
B126	B133	Alb	TBIL	0.209921394	3.70E-07
B127	B130	A-G ratio	GLB	-0.85894528	0
B127	B132	A-G ratio	Dbil	0.238337418	6.98E-09
B127	B221	A-G ratio	Hei	0.220895566	8.50E-08
B127	B133	A-G ratio	TBIL	0.216240676	1.60E-07
B128	B131	ALT	AST	0.745136276	0
B128	B140	ALT	r-GT	0.487747558	0
B128	B141	ALT	AST/ALT	-0.61226664	0
B128	B215	ALT	BMI	0.344283981	0
B128	B216	ALT	BMI	0.369281074	0
B128	B206	ALT	Waist	0.327033137	8.88E-16
B128	B222	ALT	NS	0.295046584	4.93E-13
B128	B210	ALT	Hip	0.292615173	7.78E-13
B128	B171	ALT	EC	0.286224673	2.53E-12
B128	B207	ALT	WHR	0.248227074	1.55E-09
B128	B150	ALT	ALP	0.216526036	1.54E-07
B129	B132	IBIL	Dbil	0.658446552	0
B129	B133	IBIL	TBIL	0.980188567	0
B129	B191	IBIL	Urea	-0.26424896	1.17E-10
B129	B190	IBIL	Cre	-0.22712479	3.56E-08
B130	B209	GLB	Smoking	-0.21147888	3.01E-07
B131	B140	AST	r-GT	0.351456762	0
B131	B215	AST	BMI	0.249747954	1.22E-09
B131	B206	AST	Waist	0.242588886	3.68E-09
B131	B150	AST	ALP	0.232474552	1.65E-08
B131	B171	AST	EC	0.230055338	2.34E-08
B131	B216	AST	BMI	0.225417775	4.53E-08
B131	B207	AST	WHR	0.222043321	7.25E-08
B131	B141	AST	AST/ALT	-0.20918703	4.07E-07
B132	B133	Dbil	TBIL	0.794349882	0
B133	B191	TBIL	Urea	-0.24931043	1.31E-09
B133	B190	TBIL	Cre	-0.21488195	1.92E-07
B134	B152	CHE	ACA-Ig(G-A-M)	-0.38402169	0
B134	B159	CHE	β2-MG	-0.38400957	0
B134	B169	CHE	U-CRY	-0.38400591	0
B134	B168	CHE	NMP22	0.245481544	2.37E-09
B134	B199	CHE	Mg	0.238839525	6.48E-09
B134	B137	CHE	AFU	0.226556351	3.86E-08
B134	B136	CHE	5'NT	0.210973385	3.22E-07
B135	B198	TBA	P	-0.25761973	3.48E-10

B135	B193	TBA	K	-0.21673915	1.50E-07
B135	B138	TBA	PAB	-0.20556661	6.49E-07
B135	B179	TBA	α1-MG	-0.20399075	7.93E-07
B136	B157	5'NT	IgG	0.368989751	0
B136	B194	5'NT	Cl	-0.4308252	0
B136	B150	5'NT	ALP	0.211963561	2.83E-07
B136	B196	5'NT	TCO2	0.204928896	7.04E-07
B137	B139	AFU	ADA	0.340306645	0
B137	B197	AFU	AG	-0.29191801	8.86E-13
B137	B196	AFU	TCO2	0.289516375	1.38E-12
B138	B196	PAB	TCO2	-0.33857247	0
B138	B197	PAB	AG	0.406007493	0
B138	B200	PAB	OP	0.433357268	0
B138	B199	PAB	Mg	0.319981334	3.55E-15
B138	B195	PAB	Na	0.23133961	1.95E-08
B138	B193	PAB	K	0.213070952	2.44E-07
B138	B198	PAB	P	0.209503675	3.90E-07
B139	B152	ADA	ACA-Ig(G-A-M)	0.344542885	0
B139	B159	ADA	β2-MG	0.344532027	0
B139	B169	ADA	U-CRY	0.344528752	0
B139	B200	ADA	OP	0.410790525	0
B139	B195	ADA	Na	0.286401434	2.45E-12
B139	B197	ADA	AG	0.239979399	5.46E-09
B140	B141	r-GT	AST/ALT	-0.34086476	0
B140	B216	r-GT	BMI	0.254972784	5.33E-10
B140	B206	r-GT	Waist	0.22964843	2.49E-08
B140	B215	r-GT	BMI	0.215775129	1.70E-07
B141	B216	AST/ALT	BMI	-0.34302606	0
B141	B215	AST/ALT	BMI	-0.29103407	1.04E-12
B141	B206	AST/ALT	Waist	-0.27733172	1.25E-11
B141	B210	AST/ALT	Hip	-0.26359225	1.31E-10
B141	B222	AST/ALT	NS	-0.26334455	1.36E-10
B141	B221	AST/ALT	Hei	-0.21875998	1.14E-07
B142	B143	25-(OH)D	β-CTx	-0.69450994	0
B142	B144	25-(OH)D	Total-PINP	-0.62122397	0
B142	B145	25-(OH)D	BGP	-0.51416787	0
B143	B144	β-CTx	Total-PINP	0.631584686	0
B143	B145	β-CTx	BGP	0.861494257	0
B143	B183	β-CTx	PTA	-0.33777889	0
B143	B182	β-CTx	TT	0.324075635	1.33E-15
B143	B186	β-CTx	PFC	-0.30837782	3.73E-14
B144	B145	Total-PINP	BGP	0.752932347	0
B144	B151	Total-PINP	Anti-CCP	-0.21415919	2.11E-07
B145	B183	BGP	PTA	-0.20717357	5.28E-07

B146	B189	CysC	eGFB	-0.26951401	4.82E-11
B146	B191	CysC	Urea	0.235881935	1.00E-08
B146	B192	CysC	UA	0.230651556	2.15E-08
B146	B200	CysC	OP	0.215661798	1.73E-07
B146	B213	CysC	CVA-R	0.209657489	3.83E-07
B148	B149	Anti_TPO	TGAb	0.465801936	0
B15	B100	CD3-CD19+(B cell)	anti-Ro52	0.652229026	0
B15	B101	CD3-CD19+(B cell)	anti-Sc70	0.652229033	0
B15	B102	CD3-CD19+(B cell)	anti-Sm	0.652229029	0
B15	B103	CD3-CD19+(B cell)	anti-SSA/Ro	0.652229026	0
B15	B104	CD3-CD19+(B cell)	anti-SSB	0.652229025	0
B15	B105	CD3-CD19+(B cell)	anti-U1RNP	0.652229026	0
B15	B106	CD3-CD19+(B cell)	anti-P	0.652229025	0
B15	B107	CD3-CD19+(B cell)	AnuA	0.652229014	0
B15	B108	CD3-CD19+(B cell)	Anti-Cyclin	0.652229027	0
B15	B109	CD3-CD19+(B cell)	AMA-M2	0.652229026	0
B15	B110	CD3-CD19+(B cell)	AHA	0.652229027	0
B15	B124	CD3-CD19+(B cell)	ACTH	-0.83524692	0
B15	B125	CD3-CD19+(B cell)	Cor	0.835246914	0
B15	B151	CD3-CD19+(B cell)	Anti-CCP	0.409716483	0
B15	B156	CD3-CD19+(B cell)	IgE	0.379800516	0
B15	B16	CD3-CD19+(B cell)	CD3+T	0.454281176	0
B15	B17	CD3-CD19+(B cell)	CD3+CD16+CD56+NK	-0.44494478	0
B15	B18	CD3-CD19+(B cell)	CD3+CD4+helper(inducer)T	0.539497712	0
B15	B184	CD3-CD19+(B cell)	INR	0.616597635	0
B15	B185	CD3-CD19+(B cell)	PT	0.598813108	0
B15	B20	CD3-CD19+(B cell)	CD3+CD4+CD8+	-0.82864423	0
B15	B222	CD3-CD19+(B cell)	NS	-0.47002389	0
B15	B23	CD3-CD19+(B cell)	CD3-CD16+CD56+(NK)	-0.81883527	0
B15	B54	CD3-CD19+(B cell)	ESR	-0.34659567	0
B15	B79	CD3-CD19+(B cell)	hb V -dna	0.414678647	0
B15	B81	CD3-CD19+(B cell)	TSGF	-0.35217837	0
B15	B92	CD3-CD19+(B cell)	ALDH2-Glu504Glu	-0.83524691	0
B15	B93	CD3-CD19+(B cell)	ALDH2-Glu504Lys	0.835246914	0
B15	B94	CD3-CD19+(B cell)	ALDH2-Lys504Lys	0.835246903	0
B15	B95	CD3-CD19+(B cell)	anti-cenpb	0.652229026	0
B15	B97	CD3-CD19+(B cell)	dsDNA	0.652229024	0
B15	B98	CD3-CD19+(B cell)	anti-Jo-1	0.652229028	0
B15	B99	CD3-CD19+(B cell)	anti-PM-Scl	-0.83130518	0
B15	B61	CD3-CD19+(B cell)	WBHSRV	0.330241289	4.44E-16
B15	B181	CD3-CD19+(B cell)	APTT	-0.31150533	2.00E-14
B15	B59	CD3-CD19+(B cell)	WBRV	0.300466174	1.75E-13
B15	B26	CD3-CD19+(B cell)	CKMB	0.2857997	2.74E-12
B15	B63	CD3-CD19+(B cell)	WBRSR(mPas_200)	0.271664623	3.34E-11

B15	B56	CD3-CD19+(B cell)	IR	0.270368299	4.17E-11
B15	B64	CD3-CD19+(B cell)	WBRSR(mPas_5)	0.267958684	6.28E-11
B15	B199	CD3-CD19+(B cell)	Mg	0.257813304	3.37E-10
B15	B66	CD3-CD19+(B cell)	WBRSR(mPas_100)	0.251794324	8.85E-10
B15	B67	CD3-CD19+(B cell)	WBRSR(mPas_50)	0.247365883	1.77E-09
B15	B24	CD3-CD19+(B cell)	αHBDH	0.245164308	2.49E-09
B15	B34	CD3-CD19+(B cell)	TK	0.241561047	4.30E-09
B15	B65	CD3-CD19+(B cell)	WBRSR(mPas_1)	0.235824074	1.01E-08
B15	B19	CD3-CD19+(B cell)	CD3+CD4+/CD3+CD8+	0.233339949	1.46E-08
B15	B55	CD3-CD19+(B cell)	TK	0.228292721	3.02E-08
B15	B75	CD3-CD19+(B cell)	ApoA1/B	0.220264005	9.26E-08
B15	B25	CD3-CD19+(B cell)	CK	0.219376316	1.05E-07
B15	B62	CD3-CD19+(B cell)	WBHSRVI	0.218843553	1.13E-07
B15	B74	CD3-CD19+(B cell)	ApoA1	0.210073482	3.62E-07
B15	B115	CD3-CD19+(B cell)	TSH TT3 TT4(TT3)	0.203401932	8.54E-07
B15	B154	CD3-CD19+(B cell)	RF	0.202360097	9.74E-07
B150	B171	ALP	EC	0.55447731	0
B151	B154	Anti-CCP	RF	0.383871842	0
B151	B184	Anti-CCP	INR	0.289700823	1.34E-12
B151	B185	Anti-CCP	PT	0.274223957	2.14E-11
B151	B179	Anti-CCP	α1-MG	0.254707981	5.56E-10
B151	B183	Anti-CCP	PTA	-0.23152826	1.90E-08
B151	B182	Anti-CCP	TT	0.203834308	8.09E-07
B153	B206	Glu	Waist	0.246662232	1.97E-09
B153	B170	Glu	U-GLU	0.223875976	5.62E-08
B153	B207	Glu	WHR	0.213606064	2.28E-07
B153	B215	Glu	BMI	0.208121713	4.67E-07
B154	B156	RF	IgE	0.335003609	0
B154	B179	RF	α1-MG	0.576643114	0
B154	B181	RF	APTT	-0.23364593	1.39E-08
B154	B184	RF	INR	0.219717427	9.99E-08
B154	B185	RF	PT	0.214250122	2.09E-07
B154	B180	RF	Tf	0.20305682	8.92E-07
B155	B156	IgA	IgE	-0.34407235	0
B155	B157	IgA	IgG	0.387559626	0
B155	B185	IgA	PT	0.274364903	2.09E-11
B155	B186	IgA	PFC	0.273605913	2.39E-11
B155	B184	IgA	INR	0.267849691	6.40E-11
B155	B182	IgA	TT	-0.26078147	2.08E-10
B155	B183	IgA	PTA	0.249001839	1.37E-09
B155	B158	IgA	IgM	-0.21052242	3.42E-07
B156	B158	IgE	IgM	0.425746685	0
B156	B181	IgE	APTT	-0.42726479	0
B156	B184	IgE	INR	0.466622582	0

B156	B185	IgE	PT	0.463727144	0
B156	B157	IgE	IgG	-0.29749835	3.10E-13
B156	B228	IgE	OS	0.273104352	2.60E-11
B157	B228	IgG	OS	0.204309826	7.61E-07
B157	B158	IgG	IgM	-0.20288561	9.12E-07
B158	B182	IgM	TT	-0.46541891	0
B158	B183	IgM	PTA	0.470475157	0
B158	B186	IgM	PFC	0.459501084	0
B159	B169	β2-MG	U-CRY	0.999998858	0
B16	B124	CD3+T	ACTH	-0.75337271	0
B16	B125	CD3+T	Cor	0.753372702	0
B16	B155	CD3+T	IgA	0.462805037	0
B16	B18	CD3+T	CD3+CD4+helper(inducer)T	0.378820343	0
B16	B182	CD3+T	TT	-0.48271116	0
B16	B183	CD3+T	PTA	0.464678714	0
B16	B184	CD3+T	INR	0.515717161	0
B16	B185	CD3+T	PT	0.527423099	0
B16	B186	CD3+T	PFC	0.508227595	0
B16	B19	CD3+T	CD3+CD4+/CD3+CD8+	-0.52588974	0
B16	B20	CD3+T	CD3+CD4+CD8+	-0.78557986	0
B16	B21	CD3+T	CD3+CD4-CD8-	0.863099247	0
B16	B22	CD3+T	CD3+CD8+(T)	0.723172611	0
B16	B222	CD3+T	NS	-0.47156642	0
B16	B23	CD3+T	CD3-CD16+CD56+(NK)	-0.76223585	0
B16	B69	CD3+T	PV	0.390184986	0
B16	B74	CD3+T	ApoA1	0.390530891	0
B16	B79	CD3+T	hb V -dna	0.379051246	0
B16	B92	CD3+T	ALDH2-Glu504Glu	-0.7533727	0
B16	B93	CD3+T	ALDH2-Glu504Lys	0.753372702	0
B16	B94	CD3+T	ALDH2-Lys504Lys	0.753372693	0
B16	B99	CD3+T	anti-PM-Scl	-0.75951936	0
B16	B75	CD3+T	ApoA1/B	0.326082305	8.88E-16
B16	B81	CD3+T	TSGF	-0.31507139	9.77E-15
B16	B54	CD3+T	ESR	0.289292399	1.44E-12
B16	B178	CD3+T	Ig-U	0.272972563	2.66E-11
B16	B198	CD3+T	P	-0.27142261	3.48E-11
B16	B17	CD3+T	CD3+CD16+CD56+NK	0.265138075	1.01E-10
B16	B180	CD3+T	Tf	0.257158098	3.75E-10
B16	B76	CD3+T	ApoB	-0.238906	6.42E-09
B16	B151	CD3+T	Anti-CCP	0.229140954	2.67E-08
B16	B34	CD3+T	TK	0.224723113	4.99E-08
B16	B139	CD3+T	ADA	0.204714508	7.23E-07
B16	B135	CD3+T	TBA	0.203067699	8.91E-07
B160	B163	PH	SG	-0.20369665	8.23E-07

B163	B216	SG	BMI	0.272146839	3.07E-11
B163	B176	SG	Ucr	0.270882013	3.82E-11
B163	B221	SG	Hei	0.256265842	4.33E-10
B163	B210	SG	Hip	0.217041574	1.44E-07
B163	B206	SG	Waist	0.203244506	8.71E-07
B164	B172	COM	KB	-0.21502012	1.88E-07
B167	B223	PC	FH	-0.22048193	8.99E-08
B168	B175	NMP22	ALB-U	0.524405949	0
B168	B177	NMP22	ACR	0.367054024	0
B168	B194	NMP22	Cl	0.373754818	0
B168	B196	NMP22	TCO2	-0.47114471	0
B168	B199	NMP22	Mg	-0.25232796	8.13E-10
B168	B200	NMP22	OP	0.23812539	7.21E-09
B17	B100	CD3+CD16+CD56+NK	anti-Ro52	-0.40646942	0
B17	B101	CD3+CD16+CD56+NK	anti-Sc70	-0.40646943	0
B17	B102	CD3+CD16+CD56+NK	anti-Sm	-0.40646942	0
B17	B103	CD3+CD16+CD56+NK	anti-SSA/Ro	-0.40646942	0
B17	B104	CD3+CD16+CD56+NK	anti-SSB	-0.40646942	0
B17	B105	CD3+CD16+CD56+NK	anti-U1RNP	-0.40646942	0
B17	B106	CD3+CD16+CD56+NK	anti-P	-0.40646942	0
B17	B107	CD3+CD16+CD56+NK	AnuA	-0.40646941	0
B17	B108	CD3+CD16+CD56+NK	Anti-Cyclin	-0.40646942	0
B17	B109	CD3+CD16+CD56+NK	AMA-M2	-0.40646942	0
B17	B110	CD3+CD16+CD56+NK	AHA	-0.40646942	0
B17	B143	CD3+CD16+CD56+NK	β-CTx	-0.37235035	0
B17	B151	CD3+CD16+CD56+NK	Anti-CCP	-0.37877757	0
B17	B158	CD3+CD16+CD56+NK	IgM	0.43544915	0
B17	B18	CD3+CD16+CD56+NK	CD3+CD4+helper(inducer)T	-0.54777916	0
B17	B182	CD3+CD16+CD56+NK	TT	-0.8705931	0
B17	B183	CD3+CD16+CD56+NK	PTA	0.907361612	0
B17	B186	CD3+CD16+CD56+NK	PFC	0.828469925	0
B17	B19	CD3+CD16+CD56+NK	CD3+CD4+/CD3+CD8+	-0.64029469	0
B17	B21	CD3+CD16+CD56+NK	CD3+CD4-CD8-	0.62943436	0
B17	B22	CD3+CD16+CD56+NK	CD3+CD8+(T)	0.509389507	0
B17	B54	CD3+CD16+CD56+NK	ESR	0.803575963	0
B17	B55	CD3+CD16+CD56+NK	TK	-0.5857551	0
B17	B56	CD3+CD16+CD56+NK	IR	-0.68004681	0
B17	B59	CD3+CD16+CD56+NK	WBRV	-0.41330162	0
B17	B60	CD3+CD16+CD56+NK	WBRVI	-0.42620366	0
B17	B61	CD3+CD16+CD56+NK	WBHSRV	-0.57716015	0
B17	B62	CD3+CD16+CD56+NK	WBHSRVI	-0.53629716	0
B17	B63	CD3+CD16+CD56+NK	WBRSR(mPas_200)	-0.37946254	0
B17	B64	CD3+CD16+CD56+NK	WBRSR(mPas_5)	-0.38585494	0
B17	B65	CD3+CD16+CD56+NK	WBRSR(mPas_1)	-0.33674866	0

B17	B66	CD3+CD16+CD56+NK	WBRSR(mPas_100)	-0.37015563	0
B17	B67	CD3+CD16+CD56+NK	WBRSR(mPas_50)	-0.36438948	0
B17	B69	CD3+CD16+CD56+NK	PV	0.517730344	0
B17	B95	CD3+CD16+CD56+NK	anti-cenpb	-0.40646942	0
B17	B97	CD3+CD16+CD56+NK	dsDNA	-0.40646942	0
B17	B98	CD3+CD16+CD56+NK	anti-Jo-1	-0.40646942	0
B17	B20	CD3+CD16+CD56+NK	CD3+CD4+CD8+	0.296585411	3.68E-13
B17	B26	CD3+CD16+CD56+NK	CKMB	-0.2956089	4.43E-13
B17	B74	CD3+CD16+CD56+NK	ApoA1	0.258922692	2.82E-10
B17	B199	CD3+CD16+CD56+NK	Mg	-0.23214646	1.73E-08
B17	B145	CD3+CD16+CD56+NK	BGP	-0.22795763	3.16E-08
B17	B195	CD3+CD16+CD56+NK	Na	-0.22586999	4.25E-08
B17	B24	CD3+CD16+CD56+NK	αHBDH	-0.20645747	5.79E-07
B170	B178	U-GLU	Ig-U	0.379812166	0
B170	B180	U-GLU	Tf	0.358300232	0
B170	B214	U-GLU	CVA-L	0.537378487	0
B170	B171	U-GLU	EC	0.315904388	8.22E-15
B170	B191	U-GLU	Urea	0.270426178	4.13E-11
B170	B190	U-GLU	Cre	0.251391471	9.43E-10
B170	B189	U-GLU	eGFB	-0.22030846	9.21E-08
B174	B189	Urea/Cr	eGFB	0.444390195	0
B174	B190	Urea/Cr	Cre	-0.50823022	0
B174	B218	Urea/Cr	DBP	-0.23673986	8.85E-09
B174	B220	Urea/Cr	SBP	-0.2217208	7.58E-08
B174	B221	Urea/Cr	Hei	-0.20973392	3.79E-07
B175	B177	ALB-U	ACR	0.78821998	0
B175	B178	ALB-U	Ig-U	0.531428512	0
B175	B180	ALB-U	Tf	0.503037511	0
B175	B196	ALB-U	TCO2	-0.37329977	0
B175	B214	ALB-U	CVA-L	0.375198032	0
B175	B179	ALB-U	α1-MG	0.245759422	2.27E-09
B175	B199	ALB-U	Mg	-0.22703287	3.61E-08
B175	B200	ALB-U	OP	0.220908555	8.48E-08
B176	B177	Ucr	ACR	-0.2361647	9.64E-09
B176	B183	Ucr	PTA	0.204552702	7.38E-07
B177	B178	ACR	Ig-U	0.438258891	0
B177	B180	ACR	Tf	0.436152295	0
B177	B227	ACR	OD	0.551575426	0
B177	B214	ACR	CVA-L	0.307753073	4.22E-14
B177	B196	ACR	TCO2	-0.29803566	2.79E-13
B177	B179	ACR	α1-MG	0.263292543	1.37E-10
B178	B179	Ig-U	α1-MG	0.480327637	0
B178	B180	Ig-U	Tf	0.95290513	0
B178	B214	Ig-U	CVA-L	0.709359068	0

B178	B193	Ig-U	K	0.326065457	8.88E-16
B178	B194	Ig-U	Cl	-0.26272476	1.51E-10
B178	B195	Ig-U	Na	-0.21846639	1.18E-07
B179	B180	α1-MG	Tf	0.718842707	0
B179	B214	α1-MG	CVA-L	0.329486073	4.44E-16
B179	B211	α1-MG	IOP-R	0.227892216	3.19E-08
B18	B124	CD3+CD4+helper(inducer)T	ACTH	-0.42491543	0
B18	B125	CD3+CD4+helper(inducer)T	Cor	0.424915423	0
B18	B151	CD3+CD4+helper(inducer)T	Anti-CCP	0.413833135	0
B18	B178	CD3+CD4+helper(inducer)T	Ig-U	-0.41914635	0
B18	B180	CD3+CD4+helper(inducer)T	Tf	-0.39705923	0
B18	B182	CD3+CD4+helper(inducer)T	TT	0.334565536	0
B18	B183	CD3+CD4+helper(inducer)T	PTA	-0.37038676	0
B18	B184	CD3+CD4+helper(inducer)T	INR	0.334531609	0
B18	B19	CD3+CD4+helper(inducer)T	CD3+CD4+/CD3+CD8+	0.536969528	0
B18	B195	CD3+CD4+helper(inducer)T	Na	0.379093156	0
B18	B20	CD3+CD4+helper(inducer)T	CD3+CD4+CD8+	-0.7770318	0
B18	B23	CD3+CD4+helper(inducer)T	CD3-CD16+CD56+(NK)	-0.41854658	0
B18	B54	CD3+CD4+helper(inducer)T	ESR	-0.38513283	0
B18	B55	CD3+CD4+helper(inducer)T	TK	0.38654304	0
B18	B56	CD3+CD4+helper(inducer)T	IR	0.392670534	0
B18	B61	CD3+CD4+helper(inducer)T	WBHSRV	0.385916118	0
B18	B92	CD3+CD4+helper(inducer)T	ALDH2-Glu504Glu	-0.42491542	0
B18	B93	CD3+CD4+helper(inducer)T	ALDH2-Glu504Lys	0.424915423	0
B18	B94	CD3+CD4+helper(inducer)T	ALDH2-Lys504Lys	0.424915418	0
B18	B99	CD3+CD4+helper(inducer)T	anti-PM-Scl	-0.42377117	0
B18	B22	CD3+CD4+helper(inducer)T	CD3+CD8+(T)	-0.33038903	4.44E-16
B18	B185	CD3+CD4+helper(inducer)T	PT	0.312191009	1.73E-14
B18	B59	CD3+CD4+helper(inducer)T	WBRV	0.300263475	1.82E-13
B18	B214	CD3+CD4+helper(inducer)T	CVA-L	-0.29510209	4.88E-13
B18	B186	CD3+CD4+helper(inducer)T	PFC	-0.29074932	1.10E-12
B18	B198	CD3+CD4+helper(inducer)T	P	-0.28418725	3.67E-12
B18	B62	CD3+CD4+helper(inducer)T	WBHSRVI	0.273006733	2.65E-11
B18	B155	CD3+CD4+helper(inducer)T	IgA	0.268411022	5.82E-11
B18	B137	CD3+CD4+helper(inducer)T	AFU	-0.26296997	1.45E-10
B18	B100	CD3+CD4+helper(inducer)T	anti-Ro52	0.262550685	1.55E-10
B18	B101	CD3+CD4+helper(inducer)T	anti-Sc70	0.262550687	1.55E-10
B18	B102	CD3+CD4+helper(inducer)T	anti-Sm	0.262550686	1.55E-10
B18	B103	CD3+CD4+helper(inducer)T	anti-SSA/Ro	0.262550685	1.55E-10
B18	B104	CD3+CD4+helper(inducer)T	anti-SSB	0.262550684	1.55E-10
B18	B105	CD3+CD4+helper(inducer)T	anti-U1RNP	0.262550684	1.55E-10
B18	B106	CD3+CD4+helper(inducer)T	anti-P	0.262550685	1.55E-10
B18	B107	CD3+CD4+helper(inducer)T	AnuA	0.262550677	1.55E-10
B18	B108	CD3+CD4+helper(inducer)T	Anti-Cyclin	0.262550684	1.55E-10

B18	B109	CD3+CD4+helper(inducer)T	AMA-M2	0.262550685	1.55E-10
B18	B110	CD3+CD4+helper(inducer)T	AHA	0.262550684	1.55E-10
B18	B95	CD3+CD4+helper(inducer)T	anti-cenpb	0.262550684	1.55E-10
B18	B97	CD3+CD4+helper(inducer)T	dsDNA	0.262550684	1.55E-10
B18	B98	CD3+CD4+helper(inducer)T	anti-Jo-1	0.262550684	1.55E-10
B18	B222	CD3+CD4+helper(inducer)T	NS	-0.25566035	4.78E-10
B18	B63	CD3+CD4+helper(inducer)T	WBRSR(mPas_200)	0.240817608	4.82E-09
B18	B26	CD3+CD4+helper(inducer)T	CKMB	0.240114699	5.35E-09
B18	B64	CD3+CD4+helper(inducer)T	WBRSR(mPas_5)	0.236774775	8.81E-09
B18	B193	CD3+CD4+helper(inducer)T	K	-0.23655411	9.10E-09
B18	B175	CD3+CD4+helper(inducer)T	ALB-U	-0.23556364	1.05E-08
B18	B66	CD3+CD4+helper(inducer)T	WBRSR(mPas_100)	0.231726743	1.84E-08
B18	B158	CD3+CD4+helper(inducer)T	IgM	-0.22952431	2.53E-08
B18	B179	CD3+CD4+helper(inducer)T	α1-MG	-0.22843223	2.96E-08
B18	B67	CD3+CD4+helper(inducer)T	WBRSR(mPas_50)	0.227767443	3.25E-08
B18	B181	CD3+CD4+helper(inducer)T	APTT	-0.21822306	1.22E-07
B18	B60	CD3+CD4+helper(inducer)T	WBRVI	0.21721023	1.40E-07
B18	B79	CD3+CD4+helper(inducer)T	hb V -dna	0.211405199	3.04E-07
B18	B65	CD3+CD4+helper(inducer)T	WBRSR(mPas_1)	0.20432608	7.60E-07
B18	B143	CD3+CD4+helper(inducer)T	β-CTx	0.203978528	7.94E-07
B180	B214	Tf	CVA-L	0.672841433	0
B180	B193	Tf	K	0.307148502	4.75E-14
B180	B194	Tf	Cl	-0.2496517	1.24E-09
B180	B195	Tf	Na	-0.20650891	5.75E-07
B181	B184	APTT	INR	-0.87239029	0
B181	B185	APTT	PT	-0.86582407	0
B181	B186	APTT	PFC	-0.34008634	0
B181	B182	APTT	TT	0.273535486	2.42E-11
B182	B183	TT	PTA	-0.99645978	0
B182	B184	TT	INR	-0.43657144	0
B182	B185	TT	PT	-0.47387643	0
B182	B186	TT	PFC	-0.9965913	0
B182	B222	TT	NS	0.237818819	7.54E-09
B183	B184	PTA	INR	0.363510654	0
B183	B185	PTA	PT	0.401663282	0
B183	B186	PTA	PFC	0.987003714	0
B183	B222	PTA	NS	-0.21685055	1.47E-07
B184	B185	INR	PT	0.998619253	0
B184	B186	INR	PFC	0.507494613	0
B184	B222	INR	NS	-0.42896966	0
B185	B186	PT	PFC	0.542524014	0
B185	B222	PT	NS	-0.43031764	0
B186	B222	PFC	NS	-0.2664064	8.16E-11
B189	B190	eGFB	Cre	-0.67346969	0

B189	B191	eGFB	Urea	-0.72840104	0
B189	B220	eGFB	SBP	-0.3646374	0
B189	B192	eGFB	UA	-0.23525281	1.10E-08
B19	B100	CD3+CD4+/CD3+CD8+	anti-Ro52	0.605358308	0
B19	B101	CD3+CD4+/CD3+CD8+	anti-Sc70	0.605358314	0
B19	B102	CD3+CD4+/CD3+CD8+	anti-Sm	0.605358311	0
B19	B103	CD3+CD4+/CD3+CD8+	anti-SSA/Ro	0.605358308	0
B19	B104	CD3+CD4+/CD3+CD8+	anti-SSB	0.605358307	0
B19	B105	CD3+CD4+/CD3+CD8+	anti-U1RNP	0.605358308	0
B19	B106	CD3+CD4+/CD3+CD8+	anti-P	0.605358308	0
B19	B107	CD3+CD4+/CD3+CD8+	AnuA	0.605358296	0
B19	B108	CD3+CD4+/CD3+CD8+	Anti-Cyclin	0.605358308	0
B19	B109	CD3+CD4+/CD3+CD8+	AMA-M2	0.605358308	0
B19	B110	CD3+CD4+/CD3+CD8+	AHA	0.605358309	0
B19	B175	CD3+CD4+/CD3+CD8+	ALB-U	-0.40542736	0
B19	B178	CD3+CD4+/CD3+CD8+	Ig-U	-0.73568162	0
B19	B180	CD3+CD4+/CD3+CD8+	Tf	-0.69710211	0
B19	B182	CD3+CD4+/CD3+CD8+	TT	0.599522562	0
B19	B183	CD3+CD4+/CD3+CD8+	PTA	-0.6183569	0
B19	B186	CD3+CD4+/CD3+CD8+	PFC	-0.57863106	0
B19	B21	CD3+CD4+/CD3+CD8+	CD3+CD4-CD8-	-0.50894277	0
B19	B214	CD3+CD4+/CD3+CD8+	CVA-L	-0.52163543	0
B19	B22	CD3+CD4+/CD3+CD8+	CD3+CD8+(T)	-0.93899878	0
B19	B54	CD3+CD4+/CD3+CD8+	ESR	-0.54646247	0
B19	B56	CD3+CD4+/CD3+CD8+	IR	0.417478694	0
B19	B61	CD3+CD4+/CD3+CD8+	WBHSRV	0.338003249	0
B19	B62	CD3+CD4+/CD3+CD8+	WBHSRVI	0.351149389	0
B19	B69	CD3+CD4+/CD3+CD8+	PV	-0.3698906	0
B19	B95	CD3+CD4+/CD3+CD8+	anti-cenpb	0.605358308	0
B19	B97	CD3+CD4+/CD3+CD8+	dsDNA	0.605358306	0
B19	B98	CD3+CD4+/CD3+CD8+	anti-Jo-1	0.60535831	0
B19	B179	CD3+CD4+/CD3+CD8+	α1-MG	-0.32957816	4.44E-16
B19	B55	CD3+CD4+/CD3+CD8+	TK	0.331308818	4.44E-16
B19	B74	CD3+CD4+/CD3+CD8+	ApoA1	-0.32922047	4.44E-16
B19	B177	CD3+CD4+/CD3+CD8+	ACR	-0.32371297	1.78E-15
B19	B158	CD3+CD4+/CD3+CD8+	IgM	-0.29211704	8.54E-13
B19	B60	CD3+CD4+/CD3+CD8+	WBRVI	0.282001969	5.43E-12
B19	B195	CD3+CD4+/CD3+CD8+	Na	0.277529717	1.20E-11
B19	B170	CD3+CD4+/CD3+CD8+	U-GLU	-0.27449904	2.04E-11
B19	B194	CD3+CD4+/CD3+CD8+	Cl	0.262741193	1.50E-10
B19	B64	CD3+CD4+/CD3+CD8+	WBRSR(mPas_5)	0.245021913	2.54E-09
B19	B143	CD3+CD4+/CD3+CD8+	β-CTx	0.238524395	6.79E-09
B19	B63	CD3+CD4+/CD3+CD8+	WBRSR(mPas_200)	0.237303952	8.14E-09
B19	B59	CD3+CD4+/CD3+CD8+	WBRV	0.236504903	9.17E-09

B19	B66	CD3+CD4+/CD3+CD8+	WBRSR(mPas_100)	0.2314018	1.93E-08
B19	B67	CD3+CD4+/CD3+CD8+	WBRSR(mPas_50)	0.22784213	3.22E-08
B19	B199	CD3+CD4+/CD3+CD8+	Mg	0.223154118	6.22E-08
B19	B65	CD3+CD4+/CD3+CD8+	WBRSR(mPas_1)	0.215037007	1.88E-07
B190	B191	Cre	Urea	0.815616248	0
B190	B220	Cre	SBP	0.248092077	1.58E-09
B191	B220	Urea	SBP	0.23326252	1.47E-08
B191	B192	Urea	UA	0.231235139	1.98E-08
B192	B206	UA	Waist	0.393839569	0
B192	B207	UA	WHR	0.336076201	0
B192	B216	UA	BMI	0.372091011	0
B192	B222	UA	NS	0.355803644	0
B192	B210	UA	Hip	0.306335748	5.60E-14
B192	B215	UA	BMI	0.293314865	6.83E-13
B192	B221	UA	Hei	0.276260362	1.50E-11
B193	B198	K	P	0.58890567	0
B193	B199	K	Mg	0.386893118	0
B193	B214	K	CVA-L	0.226592124	3.84E-08
B193	B197	K	AG	-0.21657109	1.53E-07
B194	B196	Cl	TCO2	-0.54914653	0
B194	B200	Cl	OP	0.328745048	4.44E-16
B194	B195	Cl	Na	0.298206348	2.70E-13
B194	B197	Cl	AG	-0.29356449	6.51E-13
B194	B199	Cl	Mg	-0.2152783	1.82E-07
B195	B197	Na	AG	0.550662561	0
B195	B200	Na	OP	0.833230506	0
B195	B196	Na	TCO2	-0.31168659	1.91E-14
B196	B197	TCO2	AG	-0.50344039	0
B196	B200	TCO2	OP	-0.60328111	0
B196	B199	TCO2	Mg	0.21551309	1.76E-07
B196	B222	TCO2	NS	-0.2147912	1.94E-07
B197	B200	AG	OP	0.670758646	0
B197	B198	AG	P	-0.20223696	9.89E-07
B2	B189	Age	eGFB	-0.47906498	0
B2	B191	Age	Urea	0.394728268	0
B2	B207	Age	WHR	0.36556453	0
B2	B220	Age	SBP	0.372568358	0
B2	B118	Age	CEA1	0.307884034	4.11E-14
B2	B47	Age	RDW	0.297654469	3.01E-13
B2	B153	Age	Glu	0.285334594	2.98E-12
B2	B206	Age	Waist	0.277603813	1.19E-11
B2	B126	Age	Alb	-0.27213687	3.08E-11
B2	B46	Age	RBC Count	-0.2680526	6.18E-11
B2	B52	Age	PLT/BPC	-0.26649189	8.04E-11

B2	B50	Age	eo%	0.253110227	7.18E-10
B2	B43	Age	MCV	0.239641258	5.75E-09
B2	B219	Age	OT	0.219315005	1.05E-07
B2	B221	Age	Hei	-0.21780607	1.30E-07
B20	B124	CD3+CD4+CD8+	ACTH	0.832497393	0
B20	B125	CD3+CD4+CD8+	Cor	-0.83249739	0
B20	B151	CD3+CD4+CD8+	Anti-CCP	-0.42924206	0
B20	B155	CD3+CD4+CD8+	IgA	-0.37711648	0
B20	B184	CD3+CD4+CD8+	INR	-0.60628594	0
B20	B185	CD3+CD4+CD8+	PT	-0.59471724	0
B20	B21	CD3+CD4+CD8+	CD3+CD4-CD8-	-0.53323707	0
B20	B222	CD3+CD4+CD8+	NS	0.494031374	0
B20	B23	CD3+CD4+CD8+	CD3-CD16+CD56+(NK)	0.826375671	0
B20	B79	CD3+CD4+CD8+	hb V -dna	-0.4157639	0
B20	B81	CD3+CD4+CD8+	TSGF	0.349757509	0
B20	B92	CD3+CD4+CD8+	ALDH2-Glu504Glu	0.832497387	0
B20	B93	CD3+CD4+CD8+	ALDH2-Glu504Lys	-0.83249739	0
B20	B94	CD3+CD4+CD8+	ALDH2-Lys504Lys	-0.83249738	0
B20	B99	CD3+CD4+CD8+	anti-PM-Scl	0.83330331	0
B20	B198	CD3+CD4+CD8+	P	0.31771807	5.77E-15
B20	B181	CD3+CD4+CD8+	APTT	0.284838305	3.26E-12
B20	B61	CD3+CD4+CD8+	WBHSRV	-0.27433594	2.10E-11
B20	B100	CD3+CD4+CD8+	anti-Ro52	-0.26923869	5.05E-11
B20	B101	CD3+CD4+CD8+	anti-Sc70	-0.26923869	5.05E-11
B20	B102	CD3+CD4+CD8+	anti-Sm	-0.26923869	5.05E-11
B20	B103	CD3+CD4+CD8+	anti-SSA/Ro	-0.26923869	5.05E-11
B20	B104	CD3+CD4+CD8+	anti-SSB	-0.26923868	5.05E-11
B20	B105	CD3+CD4+CD8+	anti-U1RNP	-0.26923869	5.05E-11
B20	B106	CD3+CD4+CD8+	anti-P	-0.26923868	5.05E-11
B20	B107	CD3+CD4+CD8+	AnuA	-0.26923868	5.05E-11
B20	B108	CD3+CD4+CD8+	Anti-Cyclin	-0.26923869	5.05E-11
B20	B109	CD3+CD4+CD8+	AMA-M2	-0.26923869	5.05E-11
B20	B110	CD3+CD4+CD8+	AHA	-0.26923869	5.05E-11
B20	B95	CD3+CD4+CD8+	anti-cenpb	-0.26923869	5.05E-11
B20	B97	CD3+CD4+CD8+	dsDNA	-0.26923868	5.05E-11
B20	B98	CD3+CD4+CD8+	anti-Jo-1	-0.26923869	5.05E-11
B20	B75	CD3+CD4+CD8+	ApoA1/B	-0.25841831	3.06E-10
B20	B22	CD3+CD4+CD8+	CD3+CD8+(T)	-0.25722029	3.71E-10
B20	B59	CD3+CD4+CD8+	WBRV	-0.25289791	7.43E-10
B20	B195	CD3+CD4+CD8+	Na	-0.2506836	1.05E-09
B20	B34	CD3+CD4+CD8+	TK	-0.24410426	2.92E-09
B20	B26	CD3+CD4+CD8+	CKMB	-0.24074918	4.87E-09
B20	B74	CD3+CD4+CD8+	ApoA1	-0.23434061	1.26E-08
B20	B76	CD3+CD4+CD8+	ApoB	0.223229251	6.15E-08

B20	B55	CD3+CD4+CD8+	TK	-0.21582733	1.69E-07
B20	B24	CD3+CD4+CD8+	αHBDH	-0.21160912	2.96E-07
B20	B56	CD3+CD4+CD8+	IR	-0.20320397	8.76E-07
B20	B25	CD3+CD4+CD8+	CK	-0.20304093	8.94E-07
B204	B209	Drinking	Smoking	0.426604408	0
B204	B221	Drinking	Hei	0.229257234	2.63E-08
B206	B207	Waist	WHR	0.859220488	0
B206	B210	Waist	Hip	0.772854052	0
B206	B215	Waist	BMI	0.833088268	0
B206	B216	Waist	BMI	0.82407878	0
B206	B221	Waist	Hei	0.353370253	0
B206	B222	Waist	NS	0.64698412	0
B206	B220	Waist	SBP	0.21336123	2.35E-07
B207	B210	WHR	Hip	0.348849707	0
B207	B215	WHR	BMI	0.597711834	0
B207	B216	WHR	BMI	0.55069757	0
B207	B222	WHR	NS	0.495892349	0
B207	B220	WHR	SBP	0.245745841	2.27E-09
B209	B221	Smoking	Hei	0.225333213	4.58E-08
B21	B124	CD3+CD4-CD8-	ACTH	-0.56643612	0
B21	B125	CD3+CD4-CD8-	Cor	0.56643611	0
B21	B155	CD3+CD4-CD8-	IgA	0.458627742	0
B21	B182	CD3+CD4-CD8-	TT	-0.73698786	0
B21	B183	CD3+CD4-CD8-	PTA	0.739300257	0
B21	B184	CD3+CD4-CD8-	INR	0.360430627	0
B21	B185	CD3+CD4-CD8-	PT	0.387057116	0
B21	B186	CD3+CD4-CD8-	PFC	0.738012851	0
B21	B22	CD3+CD4-CD8-	CD3+CD8+(T)	0.622364293	0
B21	B222	CD3+CD4-CD8-	NS	-0.3711591	0
B21	B23	CD3+CD4-CD8-	CD3-CD16+CD56+(NK)	-0.57822826	0
B21	B54	CD3+CD4-CD8-	ESR	0.544072057	0
B21	B55	CD3+CD4-CD8-	TK	-0.34040641	0
B21	B56	CD3+CD4-CD8-	IR	-0.42680809	0
B21	B62	CD3+CD4-CD8-	WBHSRVI	-0.35654054	0
B21	B69	CD3+CD4-CD8-	PV	0.520122765	0
B21	B74	CD3+CD4-CD8-	ApoA1	0.367844536	0
B21	B92	CD3+CD4-CD8-	ALDH2-Glu504Glu	-0.56643611	0
B21	B93	CD3+CD4-CD8-	ALDH2-Glu504Lys	0.56643611	0
B21	B94	CD3+CD4-CD8-	ALDH2-Lys504Lys	0.566436103	0
B21	B99	CD3+CD4-CD8-	anti-PM-Scl	-0.57228414	0
B21	B158	CD3+CD4-CD8-	IgM	0.321049934	2.66E-15
B21	B61	CD3+CD4-CD8-	WBHSRV	-0.29292944	7.34E-13
B21	B79	CD3+CD4-CD8-	hb V -dna	0.285630917	2.82E-12
B21	B75	CD3+CD4-CD8-	ApoA1/B	0.281225309	6.24E-12

B21	B60	CD3+CD4-CD8-	WBRVI	-0.27399405	2.23E-11
B21	B100	CD3+CD4-CD8-	anti-Ro52	-0.24309786	3.41E-09
B21	B101	CD3+CD4-CD8-	anti-Sc70	-0.24309786	3.41E-09
B21	B102	CD3+CD4-CD8-	anti-Sm	-0.24309786	3.41E-09
B21	B103	CD3+CD4-CD8-	anti-SSA/Ro	-0.24309786	3.41E-09
B21	B104	CD3+CD4-CD8-	anti-SSB	-0.24309786	3.41E-09
B21	B105	CD3+CD4-CD8-	anti-U1RNP	-0.24309786	3.41E-09
B21	B106	CD3+CD4-CD8-	anti-P	-0.24309786	3.41E-09
B21	B107	CD3+CD4-CD8-	AnuA	-0.24309785	3.41E-09
B21	B108	CD3+CD4-CD8-	Anti-Cyclin	-0.24309786	3.41E-09
B21	B109	CD3+CD4-CD8-	AMA-M2	-0.24309786	3.41E-09
B21	B110	CD3+CD4-CD8-	AHA	-0.24309786	3.41E-09
B21	B95	CD3+CD4-CD8-	anti-cenpb	-0.24309786	3.41E-09
B21	B97	CD3+CD4-CD8-	dsDNA	-0.24309785	3.41E-09
B21	B98	CD3+CD4-CD8-	anti-Jo-1	-0.24309786	3.41E-09
B21	B81	CD3+CD4-CD8-	TSGF	-0.23656498	9.08E-09
B21	B143	CD3+CD4-CD8-	β-CTx	-0.23436602	1.26E-08
B21	B198	CD3+CD4-CD8-	P	-0.22461718	5.07E-08
B21	B135	CD3+CD4-CD8-	TBA	0.214165414	2.11E-07
B210	B215	Hip	BMI	0.791524842	0
B210	B216	Hip	BMI	0.825726288	0
B210	B221	Hip	Hei	0.414697493	0
B210	B222	Hip	NS	0.573116707	0
B211	B212	IOP-R	IOP-L	0.850240995	0
B215	B216	BMI	BMI	0.82722191	0
B215	B222	BMI	NS	0.559510828	0
B216	B221	BMI	Hei	0.665239	0
B216	B222	BMI	NS	0.699313278	0
B218	B220	DBP	SBP	0.6478366	0
B22	B100	CD3+CD8+(T)	anti-Ro52	-0.40441919	0
B22	B101	CD3+CD8+(T)	anti-Sc70	-0.40441919	0
B22	B102	CD3+CD8+(T)	anti-Sm	-0.40441919	0
B22	B103	CD3+CD8+(T)	anti-SSA/Ro	-0.40441919	0
B22	B104	CD3+CD8+(T)	anti-SSB	-0.40441919	0
B22	B105	CD3+CD8+(T)	anti-U1RNP	-0.40441919	0
B22	B106	CD3+CD8+(T)	anti-P	-0.40441919	0
B22	B107	CD3+CD8+(T)	AnuA	-0.40441918	0
B22	B108	CD3+CD8+(T)	Anti-Cyclin	-0.40441919	0
B22	B109	CD3+CD8+(T)	AMA-M2	-0.40441919	0
B22	B110	CD3+CD8+(T)	AHA	-0.40441919	0
B22	B124	CD3+CD8+(T)	ACTH	-0.42712165	0
B22	B125	CD3+CD8+(T)	Cor	0.427121644	0
B22	B175	CD3+CD8+(T)	ALB-U	0.405092814	0
B22	B178	CD3+CD8+(T)	Ig-U	0.740596897	0

B22	B179	CD3+CD8+(T)	α1-MG	0.361502717	0
B22	B180	CD3+CD8+(T)	Tf	0.700614733	0
B22	B182	CD3+CD8+(T)	TT	-0.58631489	0
B22	B183	CD3+CD8+(T)	PTA	0.589259569	0
B22	B186	CD3+CD8+(T)	PFC	0.585639363	0
B22	B214	CD3+CD8+(T)	CVA-L	0.522225531	0
B22	B23	CD3+CD8+(T)	CD3-CD16+CD56+(NK)	-0.43799411	0
B22	B54	CD3+CD8+(T)	ESR	0.446551758	0
B22	B56	CD3+CD8+(T)	IR	-0.33909859	0
B22	B69	CD3+CD8+(T)	PV	0.409025446	0
B22	B74	CD3+CD8+(T)	ApoA1	0.411419791	0
B22	B92	CD3+CD8+(T)	ALDH2-Glu504Glu	-0.42712164	0
B22	B93	CD3+CD8+(T)	ALDH2-Glu504Lys	0.427121644	0
B22	B94	CD3+CD8+(T)	ALDH2-Lys504Lys	0.427121638	0
B22	B95	CD3+CD8+(T)	anti-cenpb	-0.40441919	0
B22	B97	CD3+CD8+(T)	dsDNA	-0.40441919	0
B22	B98	CD3+CD8+(T)	anti-Jo-1	-0.40441919	0
B22	B99	CD3+CD8+(T)	anti-PM-Scl	-0.43428023	0
B22	B177	CD3+CD8+(T)	ACR	0.325491868	1.11E-15
B22	B185	CD3+CD8+(T)	PT	0.291779891	9.09E-13
B22	B158	CD3+CD8+(T)	IgM	0.290792956	1.09E-12
B22	B62	CD3+CD8+(T)	WBHSRVI	-0.28827656	1.74E-12
B22	B170	CD3+CD8+(T)	U-GLU	0.27789519	1.13E-11
B22	B75	CD3+CD8+(T)	ApoA1/B	0.275943246	1.59E-11
B22	B194	CD3+CD8+(T)	Cl	-0.27247256	2.90E-11
B22	B184	CD3+CD8+(T)	INR	0.270218899	4.27E-11
B22	B222	CD3+CD8+(T)	NS	-0.26792658	6.31E-11
B22	B55	CD3+CD8+(T)	TK	-0.26421988	1.18E-10
B22	B155	CD3+CD8+(T)	IgA	0.245026172	2.54E-09
B22	B61	CD3+CD8+(T)	WBHSRV	-0.23500065	1.14E-08
B22	B60	CD3+CD8+(T)	WBRVI	-0.22357283	5.86E-08
B22	B79	CD3+CD8+(T)	hbV-dna	0.216802857	1.48E-07
B22	B195	CD3+CD8+(T)	Na	-0.20867311	4.35E-07
B22	B139	CD3+CD8+(T)	ADA	0.204702256	7.24E-07
B221	B222	Hei	NS	0.48476734	0
B23	B100	CD3-CD16+CD56+(NK)	anti-Ro52	-0.38898959	0
B23	B101	CD3-CD16+CD56+(NK)	anti-Sc70	-0.38898959	0
B23	B102	CD3-CD16+CD56+(NK)	anti-Sm	-0.38898959	0
B23	B103	CD3-CD16+CD56+(NK)	anti-SSA/Ro	-0.38898959	0
B23	B104	CD3-CD16+CD56+(NK)	anti-SSB	-0.38898959	0
B23	B105	CD3-CD16+CD56+(NK)	anti-U1RNP	-0.38898959	0
B23	B106	CD3-CD16+CD56+(NK)	anti-P	-0.38898959	0
B23	B107	CD3-CD16+CD56+(NK)	AnuA	-0.38898958	0
B23	B108	CD3-CD16+CD56+(NK)	Anti-Cyclin	-0.38898959	0

B23	B109	CD3-CD16+CD56+(NK)	AMA-M2	-0.38898959	0
B23	B110	CD3-CD16+CD56+(NK)	AHA	-0.38898959	0
B23	B124	CD3-CD16+CD56+(NK)	ACTH	0.999545676	0
B23	B125	CD3-CD16+CD56+(NK)	Cor	-0.99954567	0
B23	B151	CD3-CD16+CD56+(NK)	Anti-CCP	-0.37681798	0
B23	B182	CD3-CD16+CD56+(NK)	TT	0.351772154	0
B23	B184	CD3-CD16+CD56+(NK)	INR	-0.70353199	0
B23	B185	CD3-CD16+CD56+(NK)	PT	-0.70523844	0
B23	B186	CD3-CD16+CD56+(NK)	PFC	-0.39954346	0
B23	B222	CD3-CD16+CD56+(NK)	NS	0.598768851	0
B23	B69	CD3-CD16+CD56+(NK)	PV	-0.3519771	0
B23	B74	CD3-CD16+CD56+(NK)	ApoA1	-0.3950888	0
B23	B79	CD3-CD16+CD56+(NK)	hb V -dna	-0.49876789	0
B23	B81	CD3-CD16+CD56+(NK)	TSGF	0.420157296	0
B23	B92	CD3-CD16+CD56+(NK)	ALDH2-Glu504Glu	0.999545669	0
B23	B93	CD3-CD16+CD56+(NK)	ALDH2-Glu504Lys	-0.99954567	0
B23	B94	CD3-CD16+CD56+(NK)	ALDH2-Lys504Lys	-0.99954566	0
B23	B95	CD3-CD16+CD56+(NK)	anti-cenpb	-0.38898959	0
B23	B97	CD3-CD16+CD56+(NK)	dsDNA	-0.38898959	0
B23	B98	CD3-CD16+CD56+(NK)	anti-Jo-1	-0.38898959	0
B23	B99	CD3-CD16+CD56+(NK)	anti-PM-Scl	0.9996962	0
B23	B75	CD3-CD16+CD56+(NK)	ApoA1/B	-0.32738135	8.88E-16
B23	B183	CD3-CD16+CD56+(NK)	PTA	-0.3158576	8.22E-15
B23	B156	CD3-CD16+CD56+(NK)	IgE	-0.29812646	2.75E-13
B23	B155	CD3-CD16+CD56+(NK)	IgA	-0.29782065	2.91E-13
B23	B34	CD3-CD16+CD56+(NK)	TK	-0.29250989	7.94E-13
B23	B181	CD3-CD16+CD56+(NK)	APTT	0.271965706	3.17E-11
B23	B135	CD3-CD16+CD56+(NK)	TBA	-0.23991083	5.52E-09
B23	B196	CD3-CD16+CD56+(NK)	TCO2	-0.21559493	1.75E-07
B23	B76	CD3-CD16+CD56+(NK)	ApoB	0.214256913	2.09E-07
B23	B198	CD3-CD16+CD56+(NK)	P	0.203892484	8.03E-07
B23	B25	CD3-CD16+CD56+(NK)	CK	-0.20220769	9.93E-07
B24	B27	αHBDH	LDH	0.458458122	0
B24	B25	αHBDH	CK	0.322018208	2.22E-15
B24	B55	αHBDH	TK	0.239735348	5.67E-09
B24	B26	αHBDH	CKMB	0.235619288	1.04E-08
B24	B138	αHBDH	PAB	0.227140758	3.55E-08
B24	B200	αHBDH	OP	0.215873872	1.68E-07
B25	B59	CK	WBRV	0.263097217	1.42E-10
B25	B124	CK	ACTH	-0.20438884	7.54E-07
B25	B125	CK	Cor	0.204388838	7.54E-07
B25	B92	CK	ALDH2-Glu504Glu	-0.20438884	7.54E-07
B25	B93	CK	ALDH2-Glu504Lys	0.204388838	7.54E-07
B25	B94	CK	ALDH2-Lys504Lys	0.204388836	7.54E-07

B25	B99	CK	anti-PM-Scl	-0.20383367	8.09E-07
B25	B131	CK	AST	0.202595682	9.45E-07
B26	B59	CKMB	WBRV	0.317894879	5.33E-15
B26	B60	CKMB	WBRVI	0.291272723	1.00E-12
B26	B64	CKMB	WBRSR(mPas_5)	0.285864822	2.70E-12
B26	B65	CKMB	WBRSR(mPas_1)	0.275550784	1.70E-11
B26	B62	CKMB	WBHSRVI	0.274385079	2.08E-11
B26	B56	CKMB	IR	0.272549085	2.87E-11
B26	B61	CKMB	WBHSRV	0.265540922	9.43E-11
B26	B57	CKMB	RBCAI	0.253122368	7.17E-10
B26	B67	CKMB	WBRSR(mPas_50)	0.24997216	1.18E-09
B26	B63	CKMB	WBRSR(mPas_200)	0.246931097	1.89E-09
B26	B66	CKMB	WBRSR(mPas_100)	0.246347908	2.07E-09
B26	B183	CKMB	PTA	-0.21327234	2.38E-07
B27	B171	LDH	EC	0.27279742	2.75E-11
B27	B150	LDH	ALP	0.223598845	5.84E-08
B27	B195	LDH	Na	0.223211962	6.17E-08
B27	B200	LDH	OP	0.208015456	4.74E-07
B28	B29	PRL	E2	-1	0
B28	B33	PRL	Prog	1	0
B29	B30	E2	LH	1	0
B29	B31	E2	FSH	1	0
B29	B32	E2	Testo	-1	0
B29	B33	E2	Prog	-1	0
B3	B34	Hcy	TK	-0.3685843	0
B3	B25	Hcy	CK	0.275430547	1.74E-11
B3	B24	Hcy	αHBDH	0.245422863	2.39E-09
B3	B187	Hcy	NSE	0.223196847	6.18E-08
B3	B26	Hcy	CKMB	-0.22135475	7.97E-08
B30	B31	LH	FSH	1	0
B30	B32	LH	Testo	-1	0
B31	B32	FSH	Testo	-1	0
B32	B33	Testo	Prog	1	0
B34	B168	TK	NMP22	-0.61157281	0
B34	B187	TK	NSE	-0.46404793	0
B34	B175	TK	ALB-U	-0.31284161	1.51E-14
B34	B194	TK	Cl	-0.30956245	2.93E-14
B34	B99	TK	anti-PM-Scl	-0.29256328	7.86E-13
B34	B124	TK	ACTH	-0.29249118	7.97E-13
B34	B125	TK	Cor	0.292491173	7.97E-13
B34	B92	TK	ALDH2-Glu504Glu	-0.29249117	7.97E-13
B34	B93	TK	ALDH2-Glu504Lys	0.292491173	7.97E-13
B34	B94	TK	ALDH2-Lys504Lys	0.29249117	7.97E-13
B34	B222	TK	NS	-0.25069532	1.05E-09

B34	B75	TK	ApoA1/B	0.246941664	1.89E-09
B34	B196	TK	TCO2	0.234275141	1.27E-08
B34	B76	TK	ApoB	-0.23240432	1.67E-08
B34	B199	TK	Mg	0.231537355	1.89E-08
B34	B177	TK	ACR	-0.21522983	1.83E-07
B34	B185	TK	PT	0.203712926	8.21E-07
B34	B184	TK	INR	0.203465657	8.47E-07
B36	B116	V-Lym%	TT3	-0.86772183	0
B37	B38	BAND	BAND%	1	0
B37	B40	BAND	WBC	0.281639001	5.80E-12
B37	B120	BAND	HCV-cAg	-0.24671753	1.96E-09
B38	B40	BAND%	WBC	0.281639001	5.80E-12
B38	B120	BAND%	HCV-cAg	-0.24671753	1.96E-09
B39	B43	Other cells	MCV	-0.24445933	2.77E-09
B39	B44	Other cells	MCH	-0.23359143	1.41E-08
B4	B167	WB-Ca	PC	-0.2754307	1.74E-11
B40	B42	WBC	MON	0.696732425	0
B40	B52	WBC	PLT/BPC	0.258148603	3.19E-10
B40	B46	WBC	RBC Count	0.236079721	9.76E-09
B40	B53	WBC	GR%	0.219781448	9.90E-08
B40	B192	WBC	UA	0.21830067	1.21E-07
B40	B206	WBC	Waist	0.210519238	3.42E-07
B40	B48	WBC	LYM%	-0.20685096	5.50E-07
B41	B42	MON %	MON	0.587866152	0
B41	B53	MON %	GR%	-0.23999598	5.45E-09
B42	B206	MON	Waist	0.27128583	3.56E-11
B42	B192	MON	UA	0.269185335	5.10E-11
B42	B216	MON	BMI	0.246710793	1.96E-09
B42	B207	MON	WHR	0.228121414	3.09E-08
B42	B210	MON	Hip	0.21223217	2.73E-07
B42	B215	MON	BMI	0.211968848	2.83E-07
B42	B222	MON	NS	0.211844155	2.87E-07
B42	B174	MON	Urea/Cr	-0.20370888	8.22E-07
B43	B44	MCV	MCH	0.920034243	0
B43	B46	MCV	RBC Count	-0.49321115	0
B43	B47	MCV	RDW	0.520019028	0
B43	B52	MCV	PLT/BPC	-0.28699424	2.20E-12
B43	B45	MCV	MCHC	0.279455112	8.56E-12
B44	B45	MCH	MCHC	0.631003592	0
B44	B51	MCH	Hb	0.350876571	0
B44	B46	MCH	RBC Count	-0.33174042	4.44E-16
B44	B47	MCH	RDW	0.311043455	2.18E-14
B44	B52	MCH	PLT/BPC	-0.27412894	2.18E-11
B44	B209	MCH	Smoking	0.210458423	3.45E-07

B45	B51	MCHC	Hb	0.588066406	0
B45	B47	MCHC	RDW	-0.28288721	4.64E-12
B45	B216	MCHC	BMI	0.265399475	9.66E-11
B45	B221	MCHC	Hei	0.264590033	1.11E-10
B45	B71	MCHC	TG	0.261938875	1.72E-10
B45	B163	MCHC	SG	0.241391791	4.42E-09
B45	B222	MCHC	NS	0.209321937	4.00E-07
B46	B189	RBC Count	eGFB	0.391448458	0
B46	B190	RBC Count	Cre	-0.35280409	0
B46	B191	RBC Count	Urea	-0.47116127	0
B46	B216	RBC Count	BMI	0.383190143	0
B46	B221	RBC Count	Hei	0.42939358	0
B46	B47	RBC Count	RDW	-0.51256639	0
B46	B51	RBC Count	Hb	0.760141745	0
B46	B129	RBC Count	IBIL	0.306084828	5.86E-14
B46	B222	RBC Count	NS	0.290559643	1.14E-12
B46	B133	RBC Count	TBIL	0.288423696	1.69E-12
B46	B126	RBC Count	Alb	0.285396418	2.94E-12
B46	B210	RBC Count	Hip	0.278737559	9.72E-12
B46	B72	RBC Count	HDL-C	-0.26482068	1.06E-10
B46	B128	RBC Count	ALT	0.262405821	1.59E-10
B46	B206	RBC Count	Waist	0.258332451	3.10E-10
B46	B141	RBC Count	AST/ALT	-0.24951905	1.27E-09
B46	B163	RBC Count	SG	0.242010822	4.02E-09
B46	B140	RBC Count	r-GT	0.213069522	2.44E-07
B47	B191	RDW	Urea	0.301904911	1.33E-13
B47	B51	RDW	Hb	-0.28954655	1.38E-12
B47	B190	RDW	Cre	0.289462301	1.40E-12
B47	B189	RDW	eGFB	-0.28520949	3.05E-12
B47	B170	RDW	U-GLU	0.267145552	7.20E-11
B47	B118	RDW	CEA1	0.257795435	3.38E-10
B47	B52	RDW	PLT/BPC	-0.23437405	1.25E-08
B47	B214	RDW	CVA-L	0.206588736	5.69E-07
B48	B53	LYM%	GR%	-0.92322274	0
B48	B189	LYM%	eGFB	0.323514843	1.78E-15
B48	B191	LYM%	Urea	-0.29843564	2.59E-13
B48	B190	LYM%	Cre	-0.25012339	1.15E-09
B49	B50	baso%	eo%	0.27492726	1.90E-11
B5	B116	WB-Mg	TT3	-0.92518727	0
B5	B7	WB-Mg	WB-Cop	-0.49039971	0
B5	B9	WB-Mg	RH	0.990867389	0
B50	B53	eo%	GR%	-0.22897236	2.74E-08
B50	B189	eo%	eGFB	-0.21470485	1.97E-07
B50	B191	eo%	Urea	0.204394788	7.53E-07

B51	B141	Hb	AST/ALT	-0.3357761	0
B51	B189	Hb	eGFB	0.353179472	0
B51	B191	Hb	Urea	-0.42291926	0
B51	B210	Hb	Hip	0.348640533	0
B51	B216	Hb	BMI	0.461791423	0
B51	B221	Hb	Hei	0.480219036	0
B51	B222	Hb	NS	0.363019592	0
B51	B206	Hb	Waist	0.333377848	2.22E-16
B51	B190	Hb	Cre	-0.32674297	8.88E-16
B51	B163	Hb	SG	0.316789546	6.66E-15
B51	B128	Hb	ALT	0.29195266	8.81E-13
B51	B126	Hb	Alb	0.276074693	1.55E-11
B51	B209	Hb	Smoking	0.275162846	1.82E-11
B51	B129	Hb	IBIL	0.272377092	2.95E-11
B51	B133	Hb	TBIL	0.26679118	7.65E-11
B51	B204	Hb	Drinking	0.262740639	1.50E-10
B51	B72	Hb	HDL-C	-0.25617492	4.40E-10
B51	B215	Hb	BMI	0.253247653	7.02E-10
B51	B140	Hb	r-GT	0.211889611	2.86E-07
B51	B207	Hb	WHR	0.211816579	2.88E-07
B51	B71	Hb	TG	0.211457273	3.02E-07
B52	B70	PLT/BPC	LDL-C	0.266237083	8.39E-11
B53	B189	GR%	eGFB	-0.21752314	1.35E-07
B54	B100	ESR	anti-Ro52	-0.35777954	0
B54	B101	ESR	anti-Sc70	-0.35777955	0
B54	B102	ESR	anti-Sm	-0.35777954	0
B54	B103	ESR	anti-SSA/Ro	-0.35777954	0
B54	B104	ESR	anti-SSB	-0.35777954	0
B54	B105	ESR	anti-U1RNP	-0.35777954	0
B54	B106	ESR	anti-P	-0.35777954	0
B54	B107	ESR	AnuA	-0.35777954	0
B54	B108	ESR	Anti-Cyclin	-0.35777954	0
B54	B109	ESR	AMA-M2	-0.35777954	0
B54	B110	ESR	AHA	-0.35777954	0
B54	B158	ESR	IgM	0.368120618	0
B54	B182	ESR	TT	-0.79117819	0
B54	B183	ESR	PTA	0.806082601	0
B54	B186	ESR	PFC	0.769830444	0
B54	B55	ESR	TK	-0.34927235	0
B54	B56	ESR	IR	-0.46472201	0
B54	B62	ESR	WBHSRVI	-0.41634492	0
B54	B69	ESR	PV	0.619740956	0
B54	B95	ESR	anti-cenpb	-0.35777954	0
B54	B97	ESR	dsDNA	-0.35777954	0

B54	B98	ESR	anti-Jo-1	-0.35777954	0
B54	B60	ESR	WBRVI	-0.32618767	8.88E-16
B54	B61	ESR	WBHSRV	-0.31880603	4.44E-15
B54	B74	ESR	ApoA1	0.301790878	1.36E-13
B54	B143	ESR	β-CTx	-0.29915105	2.26E-13
B54	B151	ESR	Anti-CCP	-0.246847	1.92E-09
B54	B58	ESR	HCT	-0.23295808	1.54E-08
B54	B63	ESR	WBRSR(mPas_200)	-0.22081241	8.59E-08
B54	B66	ESR	WBRSR(mPas_100)	-0.21787073	1.28E-07
B54	B67	ESR	WBRSR(mPas_50)	-0.21057443	3.39E-07
B55	B182	TK	TT	0.487150171	0
B55	B183	TK	PTA	-0.51160066	0
B55	B186	TK	PFC	-0.45926206	0
B55	B56	TK	IR	0.714137659	0
B55	B58	TK	HCT	-0.36073573	0
B55	B61	TK	WBHSRV	0.574572044	0
B55	B69	TK	PV	-0.50307192	0
B55	B151	TK	Anti-CCP	0.297930415	2.85E-13
B55	B74	TK	ApoA1	-0.27045061	4.11E-11
B55	B158	TK	IgM	-0.2505835	1.07E-09
B55	B62	TK	WBHSRVI	0.246396039	2.06E-09
B55	B57	TK	RBCAI	-0.24490815	2.59E-09
B55	B59	TK	WBRV	0.234964473	1.15E-08
B55	B143	TK	β-CTx	0.218014285	1.26E-07
B55	B176	TK	Ucr	-0.21225045	2.72E-07
B56	B182	IR	TT	0.59259083	0
B56	B183	IR	PTA	-0.61744996	0
B56	B186	IR	PFC	-0.56398298	0
B56	B58	IR	HCT	0.387009813	0
B56	B59	IR	WBRV	0.757864902	0
B56	B60	IR	WBRVI	0.702598253	0
B56	B61	IR	WBHSRV	0.944259308	0
B56	B62	IR	WBHSRVI	0.853468086	0
B56	B63	IR	WBRSR(mPas_200)	0.726666053	0
B56	B64	IR	WBRSR(mPas_5)	0.655446283	0
B56	B65	IR	WBRSR(mPas_1)	0.626336903	0
B56	B66	IR	WBRSR(mPas_100)	0.72880854	0
B56	B67	IR	WBRSR(mPas_50)	0.72301139	0
B56	B69	IR	PV	-0.34793021	0
B56	B57	IR	RBCAI	0.320873383	2.89E-15
B56	B158	IR	IgM	-0.30316947	1.04E-13
B56	B151	IR	Anti-CCP	0.281169474	6.31E-12
B56	B198	IR	P	-0.26617891	8.48E-11
B56	B74	IR	ApoA1	-0.25483061	5.46E-10

B56	B143	IR	β-CTx	0.25328871	6.98E-10
B56	B100	IR	anti-Ro52	0.233241509	1.48E-08
B56	B101	IR	anti-Sc70	0.233241511	1.48E-08
B56	B102	IR	anti-Sm	0.23324151	1.48E-08
B56	B103	IR	anti-SSA/Ro	0.233241509	1.48E-08
B56	B104	IR	anti-SSB	0.233241509	1.48E-08
B56	B105	IR	anti-U1RNP	0.233241509	1.48E-08
B56	B106	IR	anti-P	0.233241509	1.48E-08
B56	B107	IR	AnuA	0.233241502	1.48E-08
B56	B108	IR	Anti-Cyclin	0.233241509	1.48E-08
B56	B109	IR	AMA-M2	0.233241509	1.48E-08
B56	B110	IR	AHA	0.233241509	1.48E-08
B56	B95	IR	anti-cenpb	0.233241509	1.48E-08
B56	B97	IR	dsDNA	0.233241508	1.48E-08
B56	B98	IR	anti-Jo-1	0.233241509	1.48E-08
B56	B199	IR	Mg	0.210690696	3.34E-07
B57	B58	RBCAI	HCT	0.776911499	0
B57	B59	RBCAI	WBRV	0.78698974	0
B57	B60	RBCAI	WBRVI	0.851811229	0
B57	B62	RBCAI	WBHSRVI	0.61872553	0
B57	B63	RBCAI	WBRSR(mPas_200)	0.581037781	0
B57	B64	RBCAI	WBRSR(mPas_5)	0.770537663	0
B57	B65	RBCAI	WBRSR(mPas_1)	0.817218372	0
B57	B66	RBCAI	WBRSR(mPas_100)	0.593696259	0
B57	B67	RBCAI	WBRSR(mPas_50)	0.62169477	0
B57	B61	RBCAI	WBHSRV	0.318120976	5.33E-15
B58	B59	HCT	WBRV	0.693900477	0
B58	B60	HCT	WBRVI	0.880199687	0
B58	B61	HCT	WBHSRV	0.477872163	0
B58	B62	HCT	WBHSRVI	0.803910992	0
B58	B63	HCT	WBRSR(mPas_200)	0.83180133	0
B58	B64	HCT	WBRSR(mPas_5)	0.850019694	0
B58	B65	HCT	WBRSR(mPas_1)	0.895921359	0
B58	B66	HCT	WBRSR(mPas_100)	0.836337631	0
B58	B67	HCT	WBRSR(mPas_50)	0.84730172	0
B59	B60	WBRV	WBRVI	0.923377477	0
B59	B61	WBRV	WBHSRV	0.812608034	0
B59	B62	WBRV	WBHSRVI	0.867018297	0
B59	B63	WBRV	WBRSR(mPas_200)	0.862669752	0
B59	B64	WBRV	WBRSR(mPas_5)	0.925952013	0
B59	B65	WBRV	WBRSR(mPas_1)	0.927849972	0
B59	B66	WBRV	WBRSR(mPas_100)	0.866392596	0
B59	B67	WBRV	WBRSR(mPas_50)	0.878546793	0
B59	B183	WBRV	PTA	-0.32583742	8.88E-16

B59	B182	WBRV	TT	0.304350344	8.24E-14
B59	B186	WBRV	PFC	-0.27881009	9.59E-12
B59	B151	WBRV	Anti-CCP	0.221080688	8.28E-08
B59	B199	WBRV	Mg	0.21523034	1.83E-07
B59	B100	WBRV	anti-Ro52	0.211045998	3.19E-07
B59	B101	WBRV	anti-Sc70	0.211046	3.19E-07
B59	B102	WBRV	anti-Sm	0.211045999	3.19E-07
B59	B103	WBRV	anti-SSA/Ro	0.211045998	3.19E-07
B59	B104	WBRV	anti-SSB	0.211045998	3.19E-07
B59	B105	WBRV	anti-U1RNP	0.211045998	3.19E-07
B59	B106	WBRV	anti-P	0.211045998	3.19E-07
B59	B107	WBRV	AnuA	0.211045995	3.19E-07
B59	B108	WBRV	Anti-Cyclin	0.211045998	3.19E-07
B59	B109	WBRV	AMA-M2	0.211045998	3.19E-07
B59	B110	WBRV	AHA	0.211045998	3.19E-07
B59	B95	WBRV	anti-cenpb	0.211045998	3.19E-07
B59	B97	WBRV	dsDNA	0.211045998	3.19E-07
B59	B98	WBRV	anti-Jo-1	0.211045999	3.19E-07
B6	B167	WB-Fe	PC	0.477921261	0
B6	B8	WB-Fe	WB-Zinc	0.795810417	0
B60	B182	WBRVI	TT	0.383477833	0
B60	B183	WBRVI	PTA	-0.39718131	0
B60	B186	WBRVI	PFC	-0.3671487	0
B60	B61	WBRVI	WBHSRV	0.722472145	0
B60	B62	WBRVI	WBHSRVI	0.937353682	0
B60	B63	WBRVI	WBRSR(mPas_200)	0.885705212	0
B60	B64	WBRVI	WBRSR(mPas_5)	0.949092527	0
B60	B65	WBRVI	WBRSR(mPas_1)	0.968480531	0
B60	B66	WBRVI	WBRSR(mPas_100)	0.895167163	0
B60	B67	WBRVI	WBRSR(mPas_50)	0.908560847	0
B61	B182	WBHSRV	TT	0.452614787	0
B61	B183	WBHSRV	PTA	-0.47987026	0
B61	B186	WBHSRV	PFC	-0.42109411	0
B61	B62	WBHSRV	WBHSRVI	0.879129407	0
B61	B63	WBHSRV	WBRSR(mPas_200)	0.86155592	0
B61	B64	WBHSRV	WBRSR(mPas_5)	0.758212846	0
B61	B65	WBHSRV	WBRSR(mPas_1)	0.726168206	0
B61	B66	WBHSRV	WBRSR(mPas_100)	0.858340074	0
B61	B67	WBHSRV	WBRSR(mPas_50)	0.84908748	0
B61	B151	WBHSRV	Anti-CCP	0.284724488	3.33E-12
B61	B100	WBHSRV	anti-Ro52	0.245220545	2.46E-09
B61	B101	WBHSRV	anti-Sc70	0.245220547	2.46E-09
B61	B102	WBHSRV	anti-Sm	0.245220546	2.46E-09
B61	B103	WBHSRV	anti-SSA/Ro	0.245220545	2.46E-09

B61	B104	WBHSRV	anti-SSB	0.245220544	2.46E-09
B61	B105	WBHSRV	anti-U1RNP	0.245220544	2.46E-09
B61	B106	WBHSRV	anti-P	0.245220544	2.46E-09
B61	B107	WBHSRV	AnuA	0.24522054	2.46E-09
B61	B108	WBHSRV	Anti-Cyclin	0.245220545	2.46E-09
B61	B109	WBHSRV	AMA-M2	0.245220544	2.46E-09
B61	B110	WBHSRV	AHA	0.245220545	2.46E-09
B61	B95	WBHSRV	anti-cenpb	0.245220544	2.46E-09
B61	B97	WBHSRV	dsDNA	0.245220543	2.46E-09
B61	B98	WBHSRV	anti-Jo-1	0.245220545	2.46E-09
B61	B199	WBHSRV	Mg	0.243969786	2.98E-09
B61	B158	WBHSRV	IgM	-0.23473425	1.19E-08
B61	B198	WBHSRV	P	-0.22783765	3.22E-08
B61	B143	WBHSRV	β-CTx	0.215040939	1.88E-07
B61	B136	WBHSRV	5'NT	0.209920227	3.70E-07
B62	B182	WBHSRVI	TT	0.482965524	0
B62	B183	WBHSRVI	PTA	-0.50059357	0
B62	B186	WBHSRVI	PFC	-0.46259818	0
B62	B63	WBHSRVI	WBRSR(mPas_200)	0.941711911	0
B62	B64	WBHSRVI	WBRSR(mPas_5)	0.908791364	0
B62	B65	WBHSRVI	WBRSR(mPas_1)	0.907202534	0
B62	B66	WBHSRVI	WBRSR(mPas_100)	0.947568545	0
B62	B67	WBHSRVI	WBRSR(mPas_50)	0.948994576	0
B62	B158	WBHSRVI	IgM	-0.24594181	2.21E-09
B62	B100	WBHSRVI	anti-Ro52	0.21353833	2.30E-07
B62	B101	WBHSRVI	anti-Sc70	0.213538332	2.30E-07
B62	B102	WBHSRVI	anti-Sm	0.213538331	2.30E-07
B62	B103	WBHSRVI	anti-SSA/Ro	0.21353833	2.30E-07
B62	B104	WBHSRVI	anti-SSB	0.21353833	2.30E-07
B62	B105	WBHSRVI	anti-U1RNP	0.21353833	2.30E-07
B62	B106	WBHSRVI	anti-P	0.21353833	2.30E-07
B62	B107	WBHSRVI	AnuA	0.213538325	2.30E-07
B62	B108	WBHSRVI	Anti-Cyclin	0.21353833	2.30E-07
B62	B109	WBHSRVI	AMA-M2	0.21353833	2.30E-07
B62	B110	WBHSRVI	AHA	0.21353833	2.30E-07
B62	B95	WBHSRVI	anti-cenpb	0.21353833	2.30E-07
B62	B97	WBHSRVI	dsDNA	0.213538329	2.30E-07
B62	B98	WBHSRVI	anti-Jo-1	0.213538331	2.30E-07
B62	B199	WBHSRVI	Mg	0.210385671	3.48E-07
B62	B198	WBHSRVI	P	-0.20959437	3.86E-07
B63	B64	WBRSR(mPas_200)	WBRSR(mPas_5)	0.939822626	0
B63	B65	WBRSR(mPas_200)	WBRSR(mPas_1)	0.937762619	0
B63	B66	WBRSR(mPas_200)	WBRSR(mPas_100)	0.998580515	0
B63	B67	WBRSR(mPas_200)	WBRSR(mPas_50)	0.997281508	0

B63	B183	WBRSR(mPas_200)	PTA	-0.30475621	7.62E-14
B63	B182	WBRSR(mPas_200)	TT	0.285137132	3.09E-12
B63	B186	WBRSR(mPas_200)	PFC	-0.26261349	1.54E-10
B63	B199	WBRSR(mPas_200)	Mg	0.23328489	1.47E-08
B63	B69	WBRSR(mPas_200)	PV	0.217443282	1.36E-07
B63	B100	WBRSR(mPas_200)	anti-Ro52	0.215055274	1.88E-07
B63	B101	WBRSR(mPas_200)	anti-Sc70	0.215055276	1.88E-07
B63	B102	WBRSR(mPas_200)	anti-Sm	0.215055275	1.88E-07
B63	B103	WBRSR(mPas_200)	anti-SSA/Ro	0.215055274	1.88E-07
B63	B104	WBRSR(mPas_200)	anti-SSB	0.215055274	1.88E-07
B63	B105	WBRSR(mPas_200)	anti-U1RNP	0.215055274	1.88E-07
B63	B106	WBRSR(mPas_200)	anti-P	0.215055274	1.88E-07
B63	B107	WBRSR(mPas_200)	AnuA	0.215055268	1.88E-07
B63	B108	WBRSR(mPas_200)	Anti-Cyclin	0.215055274	1.88E-07
B63	B109	WBRSR(mPas_200)	AMA-M2	0.215055274	1.88E-07
B63	B110	WBRSR(mPas_200)	AHA	0.215055274	1.88E-07
B63	B95	WBRSR(mPas_200)	anti-cenpb	0.215055274	1.88E-07
B63	B97	WBRSR(mPas_200)	dsDNA	0.215055273	1.88E-07
B63	B98	WBRSR(mPas_200)	anti-Jo-1	0.215055274	1.88E-07
B63	B197	WBRSR(mPas_200)	AG	0.210459004	3.45E-07
B64	B65	WBRSR(mPas_5)	WBRSR(mPas_1)	0.986180234	0
B64	B66	WBRSR(mPas_5)	WBRSR(mPas_100)	0.946195383	0
B64	B67	WBRSR(mPas_5)	WBRSR(mPas_50)	0.955187913	0
B64	B183	WBRSR(mPas_5)	PTA	-0.32242606	2.22E-15
B64	B182	WBRSR(mPas_5)	TT	0.30477369	7.59E-14
B64	B186	WBRSR(mPas_5)	PFC	-0.28391203	3.85E-12
B64	B100	WBRSR(mPas_5)	anti-Ro52	0.217682444	1.32E-07
B64	B101	WBRSR(mPas_5)	anti-Sc70	0.217682446	1.32E-07
B64	B102	WBRSR(mPas_5)	anti-Sm	0.217682444	1.32E-07
B64	B103	WBRSR(mPas_5)	anti-SSA/Ro	0.217682444	1.32E-07
B64	B104	WBRSR(mPas_5)	anti-SSB	0.217682443	1.32E-07
B64	B105	WBRSR(mPas_5)	anti-U1RNP	0.217682444	1.32E-07
B64	B106	WBRSR(mPas_5)	anti-P	0.217682443	1.32E-07
B64	B107	WBRSR(mPas_5)	AnuA	0.217682439	1.32E-07
B64	B108	WBRSR(mPas_5)	Anti-Cyclin	0.217682444	1.32E-07
B64	B109	WBRSR(mPas_5)	AMA-M2	0.217682444	1.32E-07
B64	B110	WBRSR(mPas_5)	AHA	0.217682444	1.32E-07
B64	B95	WBRSR(mPas_5)	anti-cenpb	0.217682444	1.32E-07
B64	B97	WBRSR(mPas_5)	dsDNA	0.217682443	1.32E-07
B64	B98	WBRSR(mPas_5)	anti-Jo-1	0.217682444	1.32E-07
B64	B199	WBRSR(mPas_5)	Mg	0.203869795	8.05E-07
B65	B66	WBRSR(mPas_1)	WBRSR(mPas_100)	0.943457258	0
B65	B67	WBRSR(mPas_1)	WBRSR(mPas_50)	0.954484131	0
B65	B183	WBRSR(mPas_1)	PTA	-0.28356081	4.11E-12

B65	B182	WBRSR(mPas_1)	TT	0.268600127	5.63E-11
B65	B186	WBRSR(mPas_1)	PFC	-0.25072599	1.05E-09
B66	B67	WBRSR(mPas_100)	WBRSR(mPas_50)	0.999257543	0
B66	B183	WBRSR(mPas_100)	PTA	-0.3058221	6.17E-14
B66	B182	WBRSR(mPas_100)	TT	0.288188238	1.77E-12
B66	B186	WBRSR(mPas_100)	PFC	-0.2676089	6.66E-11
B66	B199	WBRSR(mPas_100)	Mg	0.220083691	9.50E-08
B66	B69	WBRSR(mPas_100)	PV	0.203804006	8.12E-07
B66	B197	WBRSR(mPas_100)	AG	0.203238147	8.72E-07
B67	B183	WBRSR(mPas_50)	PTA	-0.30223605	1.24E-13
B67	B182	WBRSR(mPas_50)	TT	0.285112482	3.10E-12
B67	B186	WBRSR(mPas_50)	PFC	-0.26504143	1.03E-10
B67	B199	WBRSR(mPas_50)	Mg	0.21758675	1.33E-07
B67	B197	WBRSR(mPas_50)	AG	0.203696023	8.23E-07
B69	B124	PV	ACTH	-0.34296199	0
B69	B125	PV	Cor	0.342961985	0
B69	B182	PV	TT	-0.5891094	0
B69	B183	PV	PTA	0.591356415	0
B69	B186	PV	PFC	0.587227626	0
B69	B74	PV	ApoA1	0.492944696	0
B69	B92	PV	ALDH2-Glu504Glu	-0.34296199	0
B69	B93	PV	ALDH2-Glu504Lys	0.342961985	0
B69	B94	PV	ALDH2-Lys504Lys	0.34296198	0
B69	B99	PV	anti-PM-Scl	-0.34541515	0
B69	B158	PV	IgM	0.291501681	9.58E-13
B69	B138	PV	PAB	0.280031596	7.73E-12
B69	B185	PV	PT	0.278472137	1.02E-11
B69	B184	PV	INR	0.256721627	4.03E-10
B69	B176	PV	Ucr	0.223778407	5.70E-08
B7	B116	WB-Cop	TT3	-0.52991409	0
B7	B202	WB-Cop	e-times	0.361157559	0
B7	B9	WB-Cop	RH	0.571832802	0
B70	B77	LDL-C	TC	0.819840031	0
B70	B132	LDL-C	Dbil	-0.27984858	7.98E-12
B71	B72	TG	HDL-C	-0.38508766	0
B71	B140	TG	r-GT	0.320220727	3.33E-15
B71	B77	TG	TC	0.299871532	1.97E-13
B71	B130	TG	GLB	0.257649531	3.46E-10
B71	B91	TG	TP	0.245286358	2.44E-09
B72	B206	HDL-C	Waist	-0.42956682	0
B72	B207	HDL-C	WHR	-0.37349804	0
B72	B215	HDL-C	BMI	-0.38945447	0
B72	B216	HDL-C	BMI	-0.42539111	0
B72	B210	HDL-C	Hip	-0.3296776	4.44E-16

B72	B222	HDL-C	NS	-0.32517469	1.33E-15
B72	B192	HDL-C	UA	-0.28821983	1.76E-12
B72	B141	HDL-C	AST/ALT	0.259922741	2.39E-10
B72	B221	HDL-C	Hei	-0.23962559	5.76E-09
B72	B153	HDL-C	Glu	-0.2064031	5.83E-07
B73	B119	ApoA	Hcv-rna	-0.30853729	3.60E-14
B73	B121	ApoA	C3	0.308537355	3.60E-14
B73	B122	ApoA	C4	0.308537349	3.60E-14
B73	B152	ApoA	ACA-Ig(G-A-M)	0.308547929	3.60E-14
B73	B159	ApoA	β2-MG	0.308538208	3.60E-14
B73	B169	ApoA	U-CRY	0.308535268	3.60E-14
B73	B139	ApoA	ADA	0.277854849	1.14E-11
B73	B198	ApoA	P	0.221554865	7.76E-08
B74	B124	ApoA1	ACTH	-0.39026158	0
B74	B125	ApoA1	Cor	0.39026158	0
B74	B182	ApoA1	TT	-0.35606129	0
B74	B183	ApoA1	PTA	0.350170401	0
B74	B186	ApoA1	PFC	0.362779097	0
B74	B75	ApoA1	ApoA1/B	0.334445524	0
B74	B84	ApoA1	C-PSA	-0.39253316	0
B74	B89	ApoA1	CA 72-4	-0.42396847	0
B74	B92	ApoA1	ALDH2-Glu504Glu	-0.39026158	0
B74	B93	ApoA1	ALDH2-Glu504Lys	0.39026158	0
B74	B94	ApoA1	ALDH2-Lys504Lys	0.390261575	0
B74	B99	ApoA1	anti-PM-Scl	-0.392132	0
B74	B194	ApoA1	Cl	-0.32511755	1.33E-15
B74	B196	ApoA1	TCO2	0.301954345	1.31E-13
B74	B185	ApoA1	PT	0.270748746	3.90E-11
B74	B222	ApoA1	NS	-0.26749385	6.79E-11
B74	B184	ApoA1	INR	0.25753661	3.53E-10
B74	B137	ApoA1	AFU	-0.2494217	1.28E-09
B74	B200	ApoA1	OP	-0.20675597	5.57E-07
B75	B76	ApoA1/B	ApoB	-0.81851747	0
B75	B99	ApoA1/B	anti-PM-Scl	-0.32721789	8.88E-16
B75	B124	ApoA1/B	ACTH	-0.32530829	1.11E-15
B75	B125	ApoA1/B	Cor	0.325308291	1.11E-15
B75	B92	ApoA1/B	ALDH2-Glu504Glu	-0.32530829	1.11E-15
B75	B93	ApoA1/B	ALDH2-Glu504Lys	0.325308291	1.11E-15
B75	B94	ApoA1/B	ALDH2-Lys504Lys	0.325308287	1.11E-15
B75	B196	ApoA1/B	TCO2	0.31401784	1.20E-14
B75	B138	ApoA1/B	PAB	-0.31094313	2.22E-14
B75	B137	ApoA1/B	AFU	-0.27089387	3.81E-11
B75	B222	ApoA1/B	NS	-0.26223916	1.63E-10
B75	B168	ApoA1/B	NMP22	-0.2482871	1.53E-09

B75	B194	ApoA1/B	Cl	-0.24043974	5.10E-09
B75	B185	ApoA1/B	PT	0.23115874	2.00E-08
B75	B89	ApoA1/B	CA 72-4	-0.22233223	6.97E-08
B75	B184	ApoA1/B	INR	0.221594822	7.71E-08
B75	B200	ApoA1/B	OP	-0.22100286	8.37E-08
B75	B186	ApoA1/B	PFC	0.219501953	1.03E-07
B75	B182	ApoA1/B	TT	-0.21121311	3.12E-07
B75	B135	ApoA1/B	TBA	0.204270315	7.65E-07
B76	B138	ApoB	PAB	0.349324961	0
B76	B198	ApoB	P	0.293203788	6.97E-13
B76	B168	ApoB	NMP22	0.272071541	3.11E-11
B76	B77	ApoB	TC	0.215795464	1.70E-07
B76	B99	ApoB	anti-PM-Scl	0.215743281	1.71E-07
B76	B124	ApoB	ACTH	0.213961145	2.17E-07
B76	B125	ApoB	Cor	-0.21396114	2.17E-07
B76	B92	ApoB	ALDH2-Glu504Glu	0.213961144	2.17E-07
B76	B93	ApoB	ALDH2-Glu504Lys	-0.21396114	2.17E-07
B76	B94	ApoB	ALDH2-Lys504Lys	-0.21396114	2.17E-07
B77	B132	TC	Dbil	-0.30290975	1.09E-13
B79	B124	hb V -dna	ACTH	-0.49888327	0
B79	B125	hb V -dna	Cor	0.498883269	0
B79	B179	hb V -dna	α1-MG	-0.39051216	0
B79	B184	hb V -dna	INR	0.349710655	0
B79	B185	hb V -dna	PT	0.350150262	0
B79	B92	hb V -dna	ALDH2-Glu504Glu	-0.49888327	0
B79	B93	hb V -dna	ALDH2-Glu504Lys	0.498883269	0
B79	B94	hb V -dna	ALDH2-Lys504Lys	0.498883262	0
B79	B99	hb V -dna	anti-PM-Scl	-0.49891786	0
B79	B222	hb V -dna	NS	-0.28310868	4.45E-12
B79	B154	hb V -dna	RF	-0.25720213	3.72E-10
B79	B138	hb V -dna	PAB	0.253355248	6.91E-10
B79	B81	hb V -dna	TSGF	-0.21176151	2.90E-07
B8	B117	WB-Zinc	TT4	0.463586325	0
B80	B88	anti-HP IgG	CA 242	0.239949172	5.49E-09
B81	B124	TSGF	ACTH	0.420405592	0
B81	B125	TSGF	Cor	-0.42040559	0
B81	B92	TSGF	ALDH2-Glu504Glu	0.420405589	0
B81	B93	TSGF	ALDH2-Glu504Lys	-0.42040559	0
B81	B94	TSGF	ALDH2-Lys504Lys	-0.42040558	0
B81	B99	TSGF	anti-PM-Scl	0.42034409	0
B81	B185	TSGF	PT	-0.29782812	2.91E-13
B81	B184	TSGF	INR	-0.29743936	3.13E-13
B81	B222	TSGF	NS	0.237970755	7.37E-09
B81	B136	TSGF	5'NT	-0.22769712	3.28E-08

B82	B85	CEA	SCC	0.459651936	0
B82	B89	CEA	CA 72-4	0.321021387	2.89E-15
B82	B87	CEA	NSE	0.314272057	1.15E-14
B82	B137	CEA	AFU	0.29890651	2.37E-13
B82	B195	CEA	Na	-0.28629432	2.50E-12
B82	B200	CEA	OP	-0.23327554	1.47E-08
B82	B198	CEA	P	0.212704732	2.56E-07
B82	B228	CEA	OS	-0.20642959	5.81E-07
B82	B193	CEA	K	0.205160771	6.83E-07
B83	B86	AFP	TBhCG	0.450014916	0
B83	B85	AFP	SCC	0.227197828	3.52E-08
B83	B84	AFP	C-PSA	0.212653522	2.58E-07
B83	B197	AFP	AG	0.209137343	4.09E-07
B84	B135	C-PSA	TBA	0.387659969	0
B84	B89	C-PSA	CA 72-4	0.740224407	0
B84	B193	C-PSA	K	-0.29379477	6.24E-13
B84	B137	C-PSA	AFU	0.271075249	3.69E-11
B84	B204	C-PSA	Drinking	0.249547862	1.26E-09
B84	B85	C-PSA	SCC	0.231823274	1.82E-08
B84	B198	C-PSA	P	-0.21965615	1.01E-07
B85	B195	SCC	Na	-0.25292917	7.39E-10
B85	B155	SCC	IgA	0.241751522	4.18E-09
B86	B88	TBhCG	CA 242	0.393453325	0
B86	B137	TBhCG	AFU	-0.25282543	7.51E-10
B86	B89	TBhCG	CA 72-4	-0.21379696	2.22E-07
B86	B90	TBhCG	CYFRA 21-1	0.209173999	4.08E-07
B86	B228	TBhCG	OS	-0.20755757	5.02E-07
B87	B228	NSE	OS	-0.24528477	2.44E-09
B88	B138	CA 242	PAB	-0.33836412	0
B88	B197	CA 242	AG	-0.35036834	0
B88	B196	CA 242	TCO2	0.295791543	4.28E-13
B88	B200	CA 242	OP	-0.25322983	7.04E-10
B88	B199	CA 242	Mg	-0.23134013	1.95E-08
B89	B135	CA 72-4	TBA	0.338403278	0
B89	B137	CA 72-4	AFU	0.486872212	0
B89	B204	CA 72-4	Drinking	0.224046981	5.49E-08
B9	B202	RH	e-times	-0.83333333	0
B90	B189	CYFRA 21-1	eGFB	-0.22127561	8.06E-08
B91	B126	TP	Alb	0.619420802	0
B91	B130	TP	GLB	0.722411878	0
B91	B188	TP	Ca	0.318762105	4.44E-15
B91	B127	TP	A-G ratio	-0.30689329	5.00E-14
B92	B100	ALDH2-Glu504Glu	anti-Ro52	-0.40646942	0
B92	B101	ALDH2-Glu504Glu	anti-Sc70	-0.40646943	0

B92	B102	ALDH2-Glu504Glu	anti-Sm	-0.40646943	0
B92	B103	ALDH2-Glu504Glu	anti-SSA/Ro	-0.40646942	0
B92	B104	ALDH2-Glu504Glu	anti-SSB	-0.40646942	0
B92	B105	ALDH2-Glu504Glu	anti-U1RNP	-0.40646942	0
B92	B106	ALDH2-Glu504Glu	anti-P	-0.40646942	0
B92	B107	ALDH2-Glu504Glu	AnuA	-0.40646943	0
B92	B108	ALDH2-Glu504Glu	Anti-Cyclin	-0.40646942	0
B92	B109	ALDH2-Glu504Glu	AMA-M2	-0.40646942	0
B92	B110	ALDH2-Glu504Glu	AHA	-0.40646942	0
B92	B125	ALDH2-Glu504Glu	Cor	-1	0
B92	B151	ALDH2-Glu504Glu	Anti-CCP	-0.37877757	0
B92	B182	ALDH2-Glu504Glu	TT	0.335197767	0
B92	B184	ALDH2-Glu504Glu	INR	-0.70503348	0
B92	B185	ALDH2-Glu504Glu	PT	-0.7059417	0
B92	B186	ALDH2-Glu504Glu	PFC	-0.383801	0
B92	B222	ALDH2-Glu504Glu	NS	0.597437353	0
B92	B94	ALDH2-Glu504Glu	ALDH2-Lys504Lys	-0.99999999	0
B92	B95	ALDH2-Glu504Glu	anti-cenpb	-0.40646942	0
B92	B97	ALDH2-Glu504Glu	dsDNA	-0.40646942	0
B92	B98	ALDH2-Glu504Glu	anti-Jo-1	-0.40646942	0
B92	B99	ALDH2-Glu504Glu	anti-PM-Scl	0.999935487	0
B92	B156	ALDH2-Glu504Glu	IgE	-0.30619533	5.73E-14
B92	B183	ALDH2-Glu504Glu	PTA	-0.29856274	2.53E-13
B92	B155	ALDH2-Glu504Glu	IgA	-0.29356881	6.51E-13
B92	B181	ALDH2-Glu504Glu	APTT	0.275574992	1.69E-11
B92	B135	ALDH2-Glu504Glu	TBA	-0.23820545	7.12E-09
B92	B196	ALDH2-Glu504Glu	TCO2	-0.2154783	1.77E-07
B92	B198	ALDH2-Glu504Glu	P	0.205188626	6.81E-07
B93	B100	ALDH2-Glu504Lys	anti-Ro52	0.406469422	0
B93	B101	ALDH2-Glu504Lys	anti-Sc70	0.406469425	0
B93	B102	ALDH2-Glu504Lys	anti-Sm	0.406469424	0
B93	B103	ALDH2-Glu504Lys	anti-SSA/Ro	0.406469422	0
B93	B104	ALDH2-Glu504Lys	anti-SSB	0.406469423	0
B93	B105	ALDH2-Glu504Lys	anti-U1RNP	0.406469423	0
B93	B106	ALDH2-Glu504Lys	anti-P	0.406469423	0
B93	B107	ALDH2-Glu504Lys	AnuA	0.406469411	0
B93	B108	ALDH2-Glu504Lys	Anti-Cyclin	0.406469423	0
B93	B109	ALDH2-Glu504Lys	AMA-M2	0.406469423	0
B93	B110	ALDH2-Glu504Lys	AHA	0.406469423	0
B93	B151	ALDH2-Glu504Lys	Anti-CCP	0.378777569	0
B93	B182	ALDH2-Glu504Lys	TT	-0.33519777	0
B93	B184	ALDH2-Glu504Lys	INR	0.705033474	0
B93	B185	ALDH2-Glu504Lys	PT	0.705941693	0
B93	B186	ALDH2-Glu504Lys	PFC	0.383801003	0

B93	B222	ALDH2-Glu504Lys	NS	-0.59743735	0
B93	B94	ALDH2-Glu504Lys	ALDH2-Lys504Lys	0.999999988	0
B93	B95	ALDH2-Glu504Lys	anti-cenpb	0.406469422	0
B93	B97	ALDH2-Glu504Lys	dsDNA	0.406469422	0
B93	B98	ALDH2-Glu504Lys	anti-Jo-1	0.406469423	0
B93	B99	ALDH2-Glu504Lys	anti-PM-Scl	-0.99993549	0
B93	B156	ALDH2-Glu504Lys	IgE	0.306195333	5.73E-14
B93	B183	ALDH2-Glu504Lys	PTA	0.298562739	2.53E-13
B93	B155	ALDH2-Glu504Lys	IgA	0.293568811	6.51E-13
B93	B181	ALDH2-Glu504Lys	APTT	-0.27557499	1.69E-11
B93	B135	ALDH2-Glu504Lys	TBA	0.238205454	7.12E-09
B93	B196	ALDH2-Glu504Lys	TCO2	0.2154783	1.77E-07
B93	B198	ALDH2-Glu504Lys	P	-0.20518863	6.81E-07
B94	B100	ALDH2-Lys504Lys	anti-Ro52	0.406469418	0
B94	B101	ALDH2-Lys504Lys	anti-Sc70	0.406469422	0
B94	B102	ALDH2-Lys504Lys	anti-Sm	0.406469417	0
B94	B103	ALDH2-Lys504Lys	anti-SSA/Ro	0.406469417	0
B94	B104	ALDH2-Lys504Lys	anti-SSB	0.406469437	0
B94	B105	ALDH2-Lys504Lys	anti-U1RNP	0.406469421	0
B94	B106	ALDH2-Lys504Lys	anti-P	0.406469431	0
B94	B107	ALDH2-Lys504Lys	AnuA	0.406469516	0
B94	B108	ALDH2-Lys504Lys	Anti-Cyclin	0.406469423	0
B94	B109	ALDH2-Lys504Lys	AMA-M2	0.406469418	0
B94	B110	ALDH2-Lys504Lys	AHA	0.406469416	0
B94	B124	ALDH2-Lys504Lys	ACTH	-0.99999999	0
B94	B125	ALDH2-Lys504Lys	Cor	0.999999992	0
B94	B151	ALDH2-Lys504Lys	Anti-CCP	0.378777564	0
B94	B182	ALDH2-Lys504Lys	TT	-0.33519776	0
B94	B184	ALDH2-Lys504Lys	INR	0.705033464	0
B94	B185	ALDH2-Lys504Lys	PT	0.705941685	0
B94	B186	ALDH2-Lys504Lys	PFC	0.383800999	0
B94	B222	ALDH2-Lys504Lys	NS	-0.59743734	0
B94	B95	ALDH2-Lys504Lys	anti-cenpb	0.406469425	0
B94	B97	ALDH2-Lys504Lys	dsDNA	0.406469427	0
B94	B98	ALDH2-Lys504Lys	anti-Jo-1	0.406469424	0
B94	B99	ALDH2-Lys504Lys	anti-PM-Scl	-0.99993547	0
B94	B156	ALDH2-Lys504Lys	IgE	0.306195329	5.73E-14
B94	B183	ALDH2-Lys504Lys	PTA	0.298562735	2.53E-13
B94	B155	ALDH2-Lys504Lys	IgA	0.293568807	6.51E-13
B94	B181	ALDH2-Lys504Lys	APTT	-0.27557499	1.69E-11
B94	B135	ALDH2-Lys504Lys	TBA	0.238205451	7.12E-09
B94	B196	ALDH2-Lys504Lys	TCO2	0.215478297	1.77E-07
B94	B198	ALDH2-Lys504Lys	P	-0.20518862	6.81E-07
B95	B100	anti-cenpb	anti-Ro52	0.999999999	0

B95	B103	anti-cenpb	anti-SSA/Ro	0.999999999	0
B95	B104	anti-cenpb	anti-SSB	0.999999997	0
B95	B105	anti-cenpb	anti-U1RNP	0.999999999	0
B95	B107	anti-cenpb	AnuA	0.999999983	0
B95	B108	anti-cenpb	Anti-Cyclin	0.999999999	0
B95	B109	anti-cenpb	AMA-M2	0.999999998	0
B95	B110	anti-cenpb	AHA	1	0
B95	B124	anti-cenpb	ACTH	-0.40646943	0
B95	B125	anti-cenpb	Cor	0.406469423	0
B95	B156	anti-cenpb	IgE	0.379583052	0
B95	B178	anti-cenpb	Ig-U	-0.39099799	0
B95	B180	anti-cenpb	Tf	-0.37436038	0
B95	B97	anti-cenpb	dsDNA	0.999999999	0
B95	B99	anti-cenpb	anti-PM-Scl	-0.39606511	0
B95	B184	anti-cenpb	INR	0.310198725	2.58E-14
B95	B185	anti-cenpb	PT	0.293544549	6.54E-13
B95	B214	anti-cenpb	CVA-L	-0.28581501	2.73E-12
B95	B154	anti-cenpb	RF	0.285144149	3.08E-12
B95	B175	anti-cenpb	ALB-U	-0.25006036	1.16E-09
B95	B183	anti-cenpb	PTA	-0.24874269	1.43E-09
B95	B177	anti-cenpb	ACR	-0.24423985	2.86E-09
B95	B196	anti-cenpb	TCO2	0.244131093	2.91E-09
B95	B199	anti-cenpb	Mg	0.234108713	1.30E-08
B95	B195	anti-cenpb	Na	0.228135163	3.09E-08
B95	B182	anti-cenpb	TT	0.219111153	1.08E-07
B95	B151	anti-cenpb	Anti-CCP	0.209182419	4.07E-07
B96	B225	dsDNA (IIF)	hPBG	-0.36115756	0
B97	B100	dsDNA	anti-Ro52	0.999999997	0
B97	B103	dsDNA	anti-SSA/Ro	0.999999996	0
B97	B105	dsDNA	anti-U1RNP	0.999999996	0
B97	B108	dsDNA	Anti-Cyclin	0.999999998	0
B97	B109	dsDNA	AMA-M2	0.999999996	0
B97	B110	dsDNA	AHA	0.999999996	0
B97	B124	dsDNA	ACTH	-0.40646943	0
B97	B125	dsDNA	Cor	0.406469422	0
B97	B156	dsDNA	IgE	0.379583051	0
B97	B178	dsDNA	Ig-U	-0.39099799	0
B97	B180	dsDNA	Tf	-0.37436038	0
B97	B99	dsDNA	anti-PM-Scl	-0.3960651	0
B97	B184	dsDNA	INR	0.310198724	2.58E-14
B97	B185	dsDNA	PT	0.293544547	6.54E-13
B97	B214	dsDNA	CVA-L	-0.28581501	2.73E-12
B97	B154	dsDNA	RF	0.285144148	3.08E-12
B97	B175	dsDNA	ALB-U	-0.25006036	1.16E-09

B97	B183	dsDNA	PTA	-0.24874269	1.43E-09
B97	B177	dsDNA	ACR	-0.24423985	2.86E-09
B97	B196	dsDNA	TCO2	0.244131092	2.91E-09
B97	B199	dsDNA	Mg	0.234108712	1.30E-08
B97	B195	dsDNA	Na	0.228135161	3.09E-08
B97	B182	dsDNA	TT	0.219111152	1.08E-07
B97	B151	dsDNA	Anti-CCP	0.209182419	4.07E-07
B98	B101	anti-Jo-1	anti-Sc70	0.999999989	0
B98	B102	anti-Jo-1	anti-Sm	0.999999996	0
B98	B105	anti-Jo-1	anti-U1RNP	0.999999999	0
B98	B107	anti-Jo-1	AnuA	0.99999994	0
B98	B109	anti-Jo-1	AMA-M2	0.999999999	0
B98	B110	anti-Jo-1	AHA	0.999999999	0
B98	B124	anti-Jo-1	ACTH	-0.40646943	0
B98	B125	anti-Jo-1	Cor	0.406469423	0
B98	B156	anti-Jo-1	IgE	0.379583053	0
B98	B178	anti-Jo-1	Ig-U	-0.39099799	0
B98	B180	anti-Jo-1	Tf	-0.37436038	0
B98	B99	anti-Jo-1	anti-PM-Scl	-0.39606511	0
B98	B184	anti-Jo-1	INR	0.310198724	2.58E-14
B98	B185	anti-Jo-1	PT	0.293544545	6.54E-13
B98	B214	anti-Jo-1	CVA-L	-0.28581501	2.73E-12
B98	B154	anti-Jo-1	RF	0.28514415	3.08E-12
B98	B175	anti-Jo-1	ALB-U	-0.25006036	1.16E-09
B98	B183	anti-Jo-1	PTA	-0.24874269	1.43E-09
B98	B177	anti-Jo-1	ACR	-0.24423985	2.86E-09
B98	B196	anti-Jo-1	TCO2	0.244131093	2.91E-09
B98	B199	anti-Jo-1	Mg	0.234108713	1.30E-08
B98	B195	anti-Jo-1	Na	0.228135157	3.09E-08
B98	B182	anti-Jo-1	TT	0.219111153	1.08E-07
B98	B151	anti-Jo-1	Anti-CCP	0.20918242	4.07E-07
B99	B100	anti-PM-Scl	anti-Ro52	-0.39606511	0
B99	B101	anti-PM-Scl	anti-Sc70	-0.39606511	0
B99	B102	anti-PM-Scl	anti-Sm	-0.39606511	0
B99	B103	anti-PM-Scl	anti-SSA/Ro	-0.39606511	0
B99	B104	anti-PM-Scl	anti-SSB	-0.39606511	0
B99	B105	anti-PM-Scl	anti-U1RNP	-0.39606511	0
B99	B106	anti-PM-Scl	anti-P	-0.39606511	0
B99	B107	anti-PM-Scl	AnuA	-0.3960651	0
B99	B108	anti-PM-Scl	Anti-Cyclin	-0.39606511	0
B99	B109	anti-PM-Scl	AMA-M2	-0.39606511	0
B99	B110	anti-PM-Scl	AHA	-0.39606511	0
B99	B124	anti-PM-Scl	ACTH	0.999935495	0
B99	B125	anti-PM-Scl	Cor	-0.99993549	0

B99	B151	anti-PM-Scl	Anti-CCP	-0.37806662	0
B99	B182	anti-PM-Scl	TT	0.339593995	0
B99	B184	anti-PM-Scl	INR	-0.70469429	0
B99	B185	anti-PM-Scl	PT	-0.70581409	0
B99	B186	anti-PM-Scl	PFC	-0.3879812	0
B99	B222	anti-PM-Scl	NS	0.59809483	0
B99	B183	anti-PM-Scl	PTA	-0.30314459	1.04E-13
B99	B156	anti-PM-Scl	IgE	-0.30300386	1.07E-13
B99	B155	anti-PM-Scl	IgA	-0.29630482	3.89E-13
B99	B181	anti-PM-Scl	APTT	0.274662507	1.99E-11
B99	B135	anti-PM-Scl	TBA	-0.23732104	8.12E-09
B99	B196	anti-PM-Scl	TCO2	-0.21351821	2.30E-07
B99	B198	anti-PM-Scl	P	0.206251861	5.94E-07

Supplementary Table 17.PEI Correlations in Diabetes

Row	Column	Row	Column	Cor	*P*
B1	B126	Gender	Alb	-0.094115831	0
B1	B127	Gender	A-G ratio	-0.145731403	0
B1	B128	Gender	ALT	-0.081382156	0
B1	B129	Gender	IBIL	-0.099340521	0
B1	B130	Gender	GLB	0.124953029	0
B1	B132	Gender	Dbil	-0.11101206	0
B1	B133	Gender	TBIL	-0.11099021	0
B1	B140	Gender	r-GT	-0.09344472	0
B1	B141	Gender	AST/ALT	0.121756985	0
B1	B148	Gender	Anti_TPO	0.081956341	0
B1	B161	Gender	LEU	0.225163153	0
B1	B163	Gender	SG	-0.333109931	0
B1	B166	Gender	Pro	-0.154635363	0
B1	B170	Gender	U-GLU	-0.144332905	0
B1	B173	Gender	NTT	0.157765641	0
B1	B174	Gender	Urea/Cr	0.364584609	0
B1	B176	Gender	Ucr	-0.166167979	0
B1	B190	Gender	Cre	-0.368049155	0
B1	B191	Gender	Urea	-0.081734077	0
B1	B192	Gender	UA	-0.262956124	0
B1	B204	Gender	Drinking	-0.260139157	0
B1	B206	Gender	Waist	-0.288783125	0
B1	B209	Gender	Smoking	-0.308687138	0
B1	B210	Gender	Hip	-0.198150138	0
B1	B215	Gender	BMI	-0.100119361	0
B1	B216	Gender	BMI	-0.458499075	0
B1	B218	Gender	DBP	-0.113132465	0
B1	B219	Gender	OT	0.125745637	0
B1	B220	Gender	SBP	0.076914388	0
B1	B221	Gender	Hei	-0.642868778	0
B1	B222	Gender	NS	-0.608769157	0
B1	B3	Gender	Hcy	-0.105404684	0
B1	B40	Gender	WBC	-0.115820861	0
B1	B41	Gender	MON %	-0.175471487	0
B1	B42	Gender	MON	-0.218767418	0
B1	B44	Gender	MCH	-0.140983187	0
B1	B45	Gender	MCHC	-0.26266774	0
B1	B46	Gender	RBC Count	-0.376448818	0
B1	B48	Gender	LYM%	0.08463569	0
B1	B50	Gender	eo%	-0.096821769	0
B1	B51	Gender	Hb	-0.49464612	0
B1	B52	Gender	PLT/BPC	0.093423037	0

B1	B58	Gender	HCT	-0.14135428	0
B1	B60	Gender	WBRVI	-0.110166995	0
B1	B62	Gender	WBHSRVI	-0.108805252	0
B1	B63	Gender	WBRSR(mPas_200)	-0.093735344	0
B1	B65	Gender	WBRSR(mPas_1)	-0.097584756	0
B1	B66	Gender	WBRSR(mPas_100)	-0.092345803	0
B1	B67	Gender	WBRSR(mPas_50)	-0.095110163	0
B1	B72	Gender	HDL-C	0.274746234	0
B1	B74	Gender	ApoA1	0.138504758	0
B1	B77	Gender	TC	0.097068786	0
B1	B54	Gender	ESR	0.073699162	1.11E-15
B1	B2	Gender	Age	0.070981707	1.22E-14
B1	B113	Gender	FT3	-0.067836736	1.70E-13
B1	B149	Gender	TGAb	0.063304689	6.10E-12
B1	B56	Gender	IR	-0.062668328	9.90E-12
B1	B172	Gender	KB	-0.060667653	4.39E-11
B1	B75	Gender	ApoA1/B	0.060581649	4.68E-11
B1	B71	Gender	TG	-0.060249282	5.96E-11
B1	B138	Gender	PAB	-0.059426716	1.08E-10
B1	B198	Gender	P	0.057966866	3.05E-10
B1	B153	Gender	Glu	-0.05697617	6.07E-10
B1	B27	Gender	LDH	0.056583363	7.95E-10
B1	B24	Gender	αHBDH	0.056579087	7.98E-10
B1	B91	Gender	TP	0.05577717	1.38E-09
B1	B25	Gender	CK	-0.054689005	2.85E-09
B1	B114	Gender	TSH TT3 TT4(TT4)	0.054494968	3.25E-09
B1	B205	Gender	KDA	0.053043632	8.37E-09
B1	B61	Gender	WBHSRV	-0.05200241	1.63E-08
B1	B111	Gender	TSH	0.05193736	1.70E-08
B1	B84	Gender	C-PSA	-0.047667019	2.26E-07
B1	B49	Gender	baso%	-0.046567763	4.27E-07
B1	B189	Gender	eGFB	0.046243131	5.13E-07
B1	B179	Gender	α1-MG	-0.045323533	8.59E-07
B10	B11	PG I	PGI/PGII	0.165564845	0
B10	B119	PG I	Hcv-rna	-0.092059861	0
B10	B12	PG I	PG II	0.543757539	0
B10	B143	PG I	β-CTx	-0.096524665	0
B10	B145	PG I	BGP	-0.106710914	0
B10	B15	PG I	CD3-CD19+(B cell)	-0.077186201	0
B10	B175	PG I	ALB-U	0.289761478	0
B10	B177	PG I	ACR	0.235078333	0
B10	B189	PG I	eGFB	-0.09912661	0
B10	B190	PG I	Cre	0.102115992	0
B10	B191	PG I	Urea	0.085957831	0

B10	B80	PG I	anti-HP IgG	0.083290261	0
B10	B144	PG I	Total-PINP	-0.07363458	1.33E-15
B10	B123	PG I	hs-CRP1	0.068479424	1.00E-13
B10	B186	PG I	PFC	0.068204143	1.26E-13
B10	B107	PG I	AnuA	-0.067446002	2.33E-13
B10	B101	PG I	anti-Sc70	-0.066956741	3.47E-13
B10	B104	PG I	anti-SSB	-0.066956729	3.47E-13
B10	B98	PG I	anti-Jo-1	-0.066956723	3.47E-13
B10	B151	PG I	Anti-CCP	0.065144244	1.47E-12
B10	B18	PG I	CD3+CD4+helper(inducer)T	0.062996367	7.72E-12
B10	B125	PG I	Cor	0.056888008	6.45E-10
B10	B47	PG I	RDW	0.054618425	2.99E-09
B10	B16	PG I	CD3+T	0.052641652	1.08E-08
B10	B24	PG I	αHBDH	0.050742543	3.58E-08
B10	B25	PG I	CK	0.050692469	3.69E-08
B10	B79	PG I	hb V -dna	-0.047537137	2.44E-07
B10	B126	PG I	Alb	-0.046722878	3.90E-07
B10	B192	PG I	UA	0.046519656	4.38E-07
B10	B120	PG I	HCV-cAg	-0.045399169	8.24E-07
B100	B101	anti-Ro52	anti-Sc70	0.09482229	0
B100	B104	anti-Ro52	anti-SSB	0.094822278	0
B100	B107	anti-Ro52	AnuA	0.092893593	0
B100	B157	anti-Ro52	IgG	0.079460027	0
B100	B182	anti-Ro52	TT	-0.06027973	5.83E-11
B100	B193	anti-Ro52	K	0.052154086	1.48E-08
B100	B124	anti-Ro52	ACTH	-0.050831261	3.39E-08
B100	B137	anti-Ro52	AFU	-0.046961108	3.41E-07
B100	B119	anti-Ro52	Hcv-rna	-0.045990699	5.92E-07
B101	B102	anti-Sc70	anti-Sm	0.110763142	0
B101	B103	anti-Sc70	anti-SSA/Ro	0.094458136	0
B101	B106	anti-Sc70	anti-P	0.110785287	0
B101	B107	anti-Sc70	AnuA	0.994375158	0
B101	B119	anti-Sc70	Hcv-rna	0.150439903	0
B101	B120	anti-Sc70	HCV-cAg	0.126183922	0
B101	B121	anti-Sc70	C3	-0.094750552	0
B101	B122	anti-Sc70	C4	-0.086964903	0
B101	B134	anti-Sc70	CHE	-0.099159467	0
B101	B137	anti-Sc70	AFU	-0.077017833	0
B101	B138	anti-Sc70	PAB	-0.07599806	0
B101	B139	anti-Sc70	ADA	-0.086100026	0
B101	B151	anti-Sc70	Anti-CCP	-0.123677072	0
B101	B155	anti-Sc70	IgA	-0.07859844	0
B101	B157	anti-Sc70	IgG	0.143501857	0
B101	B181	anti-Sc70	APTT	0.126973236	0

B101	B184	anti-Sc70	INR	0.140026311	0
B101	B185	anti-Sc70	PT	0.076376763	0
B101	B186	anti-Sc70	PFC	-0.08709384	0
B101	B194	anti-Sc70	Cl	0.12305288	0
B101	B195	anti-Sc70	Na	0.122660759	0
B101	B198	anti-Sc70	P	-0.094561464	0
B101	B199	anti-Sc70	Mg	0.114914253	0
B101	B200	anti-Sc70	OP	-0.165117545	0
B101	B183	anti-Sc70	PTA	0.072236214	4.00E-15
B101	B152	anti-Sc70	ACA-Ig(G-A-M)	0.066754191	4.08E-13
B101	B179	anti-Sc70	α1-MG	-0.065045271	1.59E-12
B101	B212	anti-Sc70	IOP-L	-0.061301285	2.75E-11
B101	B124	anti-Sc70	ACTH	-0.054617221	2.99E-09
B101	B193	anti-Sc70	K	0.05201209	1.62E-08
B101	B112	anti-Sc70	FT4	-0.048806322	1.16E-07
B101	B220	anti-Sc70	SBP	0.04795154	1.92E-07
B101	B150	anti-Sc70	ALP	-0.047571286	2.39E-07
B101	B189	anti-Sc70	eGFB	-0.047256567	2.87E-07
B101	B211	anti-Sc70	IOP-R	-0.046863291	3.60E-07
B102	B104	anti-Sm	anti-SSB	0.110763147	0
B102	B107	anti-Sm	AnuA	0.109310812	0
B102	B140	anti-Sm	r-GT	0.118774779	0
B102	B155	anti-Sm	IgA	0.127839175	0
B102	B179	anti-Sm	α1-MG	0.098510446	0
B102	B157	anti-Sm	IgG	0.06964853	3.77E-14
B102	B158	anti-Sm	IgM	0.059000807	1.47E-10
B102	B115	anti-Sm	TSH TT3 TT4(TT3)	-0.054272634	3.76E-09
B102	B178	anti-Sm	Ig-U	0.050999672	3.05E-08
B103	B104	anti-SSA/Ro	anti-SSB	0.094458122	0
B103	B107	anti-SSA/Ro	AnuA	0.092371516	0
B103	B108	anti-SSA/Ro	Anti-Cyclin	0.626575473	0
B103	B141	anti-SSA/Ro	AST/ALT	0.068280597	1.18E-13
B103	B157	anti-SSA/Ro	IgG	0.066327519	5.75E-13
B104	B106	anti-SSB	anti-P	0.110785259	0
B104	B107	anti-SSB	AnuA	0.994376258	0
B104	B119	anti-SSB	Hcv-rna	0.150439883	0
B104	B120	anti-SSB	HCV-cAg	0.12618479	0
B104	B121	anti-SSB	C3	-0.094750482	0
B104	B122	anti-SSB	C4	-0.086964863	0
B104	B134	anti-SSB	CHE	-0.099159449	0
B104	B137	anti-SSB	AFU	-0.07701782	0
B104	B138	anti-SSB	PAB	-0.075998044	0
B104	B139	anti-SSB	ADA	-0.08610001	0
B104	B151	anti-SSB	Anti-CCP	-0.123677049	0

B104	B155	anti-SSB	IgA	-0.078598424	0
B104	B157	anti-SSB	IgG	0.143501842	0
B104	B181	anti-SSB	APTT	0.126973217	0
B104	B184	anti-SSB	INR	0.140026295	0
B104	B185	anti-SSB	PT	0.076376755	0
B104	B186	anti-SSB	PFC	-0.087093817	0
B104	B194	anti-SSB	Cl	0.123052885	0
B104	B195	anti-SSB	Na	0.1226608	0
B104	B198	anti-SSB	P	-0.094561437	0
B104	B199	anti-SSB	Mg	0.114914249	0
B104	B200	anti-SSB	OP	-0.165117511	0
B104	B183	anti-SSB	PTA	0.072236204	4.00E-15
B104	B152	anti-SSB	ACA-Ig(G-A-M)	0.06675424	4.08E-13
B104	B179	anti-SSB	α1-MG	-0.06504526	1.59E-12
B104	B212	anti-SSB	IOP-L	-0.061301273	2.75E-11
B104	B124	anti-SSB	ACTH	-0.054617211	2.99E-09
B104	B193	anti-SSB	K	0.052012096	1.62E-08
B104	B112	anti-SSB	FT4	-0.048806311	1.16E-07
B104	B220	anti-SSB	SBP	0.047951534	1.92E-07
B104	B150	anti-SSB	ALP	-0.047571276	2.39E-07
B104	B189	anti-SSB	eGFB	-0.047256558	2.87E-07
B104	B211	anti-SSB	IOP-R	-0.046863283	3.60E-07
B105	B119	anti-U1RNP	Hcv-rna	-0.084963313	0
B105	B125	anti-U1RNP	Cor	0.109565661	0
B105	B196	anti-U1RNP	TCO2	0.056127644	1.09E-09
B105	B179	anti-U1RNP	α1-MG	0.051636505	2.05E-08
B105	B158	anti-U1RNP	IgM	0.050233638	4.89E-08
B106	B107	anti-P	AnuA	0.109332722	0
B106	B157	anti-P	IgG	0.08368434	0
B106	B123	anti-P	hs-CRP1	0.073384458	1.55E-15
B106	B155	anti-P	IgA	0.069168591	5.66E-14
B107	B119	AnuA	Hcv-rna	0.151385073	0
B107	B120	AnuA	HCV-cAg	0.122875342	0
B107	B121	AnuA	C3	-0.095282408	0
B107	B122	AnuA	C4	-0.087444475	0
B107	B134	AnuA	CHE	-0.10458179	0
B107	B138	AnuA	PAB	-0.084602301	0
B107	B139	AnuA	ADA	-0.086218483	0
B107	B151	AnuA	Anti-CCP	-0.124321867	0
B107	B157	AnuA	IgG	0.145517111	0
B107	B181	AnuA	APTT	0.127680792	0
B107	B184	AnuA	INR	0.140811983	0
B107	B185	AnuA	PT	0.076786554	0
B107	B186	AnuA	PFC	-0.08761144	0

B107	B194	AnuA	Cl	0.124650008	0
B107	B195	AnuA	Na	0.124763441	0
B107	B198	AnuA	P	-0.093804267	0
B107	B199	AnuA	Mg	0.108597029	0
B107	B200	AnuA	OP	-0.16232695	0
B107	B137	AnuA	AFU	-0.073949171	8.88E-16
B107	B183	AnuA	PTA	0.072651555	2.89E-15
B107	B155	AnuA	IgA	-0.072107587	4.66E-15
B107	B152	AnuA	ACA-Ig(G-A-M)	0.067136596	3.00E-13
B107	B179	AnuA	α1-MG	-0.063648249	4.69E-12
B107	B212	AnuA	IOP-L	-0.06337118	5.80E-12
B107	B124	AnuA	ACTH	-0.054937091	2.42E-09
B107	B150	AnuA	ALP	-0.050769582	3.52E-08
B107	B220	AnuA	SBP	0.048932252	1.07E-07
B107	B211	AnuA	IOP-R	-0.04795949	1.91E-07
B107	B112	AnuA	FT4	-0.047045475	3.25E-07
B107	B189	AnuA	eGFB	-0.04678867	3.76E-07
B107	B193	AnuA	K	0.046693334	3.97E-07
B108	B109	Anti-Cyclin	AMA-M2	0.146688438	0
B109	B182	AMA-M2	TT	0.102773861	0
B109	B195	AMA-M2	Na	-0.063625262	4.77E-12
B109	B119	AMA-M2	Hcv-rna	-0.054104454	4.20E-09
B109	B204	AMA-M2	Drinking	0.050062658	5.43E-08
B109	B155	AMA-M2	IgA	0.047715273	2.20E-07
B109	B157	AMA-M2	IgG	-0.047386031	2.67E-07
B109	B151	AMA-M2	Anti-CCP	-0.045329198	8.56E-07
B11	B12	PGI/PGII	PG II	-0.498291336	0
B11	B14	PGI/PGII	G-17	-0.234989476	0
B11	B175	PGI/PGII	ALB-U	0.11913415	0
B11	B177	PGI/PGII	ACR	0.097007614	0
B11	B80	PGI/PGII	anti-HP IgG	-0.119502957	0
B11	B34	PGI/PGII	TK	0.070837419	1.38E-14
B11	B145	PGI/PGII	BGP	-0.051394021	2.39E-08
B110	B119	AHA	Hcv-rna	-0.138791252	0
B110	B134	AHA	CHE	0.070314478	2.18E-14
B110	B138	AHA	PAB	0.069955001	2.93E-14
B110	B120	AHA	HCV-cAg	-0.06702877	3.27E-13
B110	B197	AHA	AG	0.054032248	4.40E-09
B110	B196	AHA	TCO2	-0.045970141	5.98E-07
B111	B112	TSH	FT4	-0.219643397	0
B111	B113	TSH	FT3	-0.130398835	0
B111	B148	TSH	Anti_TPO	0.200964715	0
B111	B149	TSH	TGAb	0.132053942	0
B111	B189	TSH	eGFB	-0.050036872	5.52E-08

B111	B198	TSH	P	0.049236109	8.95E-08
B111	B114	TSH	TSH TT3 TT4(TT4)	-0.045289237	8.76E-07
B112	B113	FT4	FT3	0.250976763	0
B112	B114	FT4	TSH TT3 TT4(TT4)	0.452266153	0
B112	B115	FT4	TSH TT3 TT4(TT3)	0.087293655	0
B112	B153	FT4	Glu	0.073258724	1.78E-15
B112	B188	FT4	Ca	0.06195375	1.69E-11
B112	B120	FT4	HCV-cAg	-0.059124283	1.34E-10
B112	B189	FT4	eGFB	0.057253095	5.01E-10
B112	B150	FT4	ALP	0.054767309	2.71E-09
B112	B194	FT4	Cl	-0.050226563	4.91E-08
B113	B114	FT3	TSH TT3 TT4(TT4)	0.104717097	0
B113	B115	FT3	TSH TT3 TT4(TT3)	0.358200366	0
B113	B126	FT3	Alb	0.092343097	0
B113	B145	FT3	BGP	0.080801966	0
B113	B149	FT3	TGAb	0.064735221	2.02E-12
B113	B189	FT3	eGFB	0.062780944	9.09E-12
B113	B222	FT3	NS	0.059984732	7.22E-11
B113	B123	FT3	hs-CRP1	-0.052543674	1.15E-08
B113	B209	FT3	Smoking	0.051274653	2.57E-08
B113	B216	FT3	BMI	0.050526912	4.09E-08
B113	B188	FT3	Ca	0.049440475	7.92E-08
B113	B134	FT3	CHE	0.049283165	8.70E-08
B113	B141	FT3	AST/ALT	-0.049236072	8.95E-08
B113	B206	FT3	Waist	0.046644196	4.08E-07
B113	B144	FT3	Total-PINP	0.046410547	4.67E-07
B113	B128	FT3	ALT	0.046112207	5.52E-07
B113	B119	FT3	Hcv-rna	-0.045600254	7.36E-07
B113	B143	FT3	β-CTx	0.04538352	8.31E-07
B114	B115	TSH TT3 TT4(TT4)	TSH TT3 TT4(TT3)	0.325671044	0
B114	B150	TSH TT3 TT4(TT4)	ALP	0.059692141	8.93E-11
B114	B139	TSH TT3 TT4(TT4)	ADA	0.057049229	5.77E-10
B114	B186	TSH TT3 TT4(TT4)	PFC	0.054746578	2.75E-09
B114	B123	TSH TT3 TT4(TT4)	hs-CRP1	0.05320768	7.53E-09
B114	B197	TSH TT3 TT4(TT4)	AG	0.052924042	9.04E-09
B114	B204	TSH TT3 TT4(TT4)	Drinking	-0.048426736	1.45E-07
B114	B188	TSH TT3 TT4(TT4)	Ca	0.048390098	1.48E-07
B114	B194	TSH TT3 TT4(TT4)	Cl	-0.047844555	2.04E-07
B114	B120	TSH TT3 TT4(TT4)	HCV-cAg	-0.047232993	2.91E-07
B115	B134	TSH TT3 TT4(TT3)	CHE	0.076412018	0
B115	B126	TSH TT3 TT4(TT3)	Alb	0.057481141	4.28E-10
B115	B125	TSH TT3 TT4(TT3)	Cor	-0.045195609	9.22E-07
B116	B117	TT3	TT4	0.594770853	0
B118	B150	CEA1	ALP	0.229329471	0

B118	B219	CEA1	OT	0.067693087	1.91E-13
B119	B120	Hcv-rna	HCV-cAg	0.505246111	0
B119	B151	Hcv-rna	Anti-CCP	-0.096563033	0
B119	B183	Hcv-rna	PTA	0.129101314	0
B119	B186	Hcv-rna	PFC	-0.08750318	0
B119	B194	Hcv-rna	CI	0.129000852	0
B119	B200	Hcv-rna	OP	-0.114737538	0
B119	B184	Hcv-rna	INR	0.069489405	4.33E-14
B119	B199	Hcv-rna	Mg	0.068321963	1.14E-13
B119	B169	Hcv-rna	U-CRY	-0.065438676	1.16E-12
B119	B137	Hcv-rna	AFU	-0.065284508	1.31E-12
B119	B182	Hcv-rna	TT	-0.05705892	5.73E-10
B119	B135	Hcv-rna	TBA	-0.055938192	1.23E-09
B119	B121	Hcv-rna	C3	-0.054494779	3.25E-09
B119	B156	Hcv-rna	IgE	0.05295574	8.86E-09
B119	B181	Hcv-rna	APTT	0.049894481	6.01E-08
B119	B146	Hcv-rna	CysC	-0.047113163	3.12E-07
B119	B196	Hcv-rna	TCO2	-0.045127055	9.58E-07
B12	B14	PG II	G-17	0.208731128	0
B12	B80	PG II	anti-HP IgG	0.166607769	0
B12	B119	PG II	Hcv-rna	-0.069196564	5.53E-14
B12	B189	PG II	eGFB	-0.068373592	1.09E-13
B12	B186	PG II	PFC	0.064367821	2.69E-12
B12	B25	PG II	CK	0.062167831	1.44E-11
B12	B190	PG II	Cre	0.052747596	1.01E-08
B12	B47	PG II	RDW	0.052281871	1.36E-08
B12	B15	PG II	CD3-CD19+(B cell)	-0.049355211	8.33E-08
B12	B183	PG II	PTA	-0.046603752	4.18E-07
B12	B151	PG II	Anti-CCP	0.045095972	9.75E-07
B120	B121	HCV-cAg	C3	-0.091990789	0
B120	B134	HCV-cAg	CHE	-0.082257943	0
B120	B183	HCV-cAg	PTA	0.080303431	0
B120	B200	HCV-cAg	OP	-0.085758236	0
B120	B224	HCV-cAg	pmh	-0.134184958	0
B120	B194	HCV-cAg	CI	0.074118626	8.88E-16
B120	B122	HCV-cAg	C4	-0.057383392	4.58E-10
B120	B163	HCV-cAg	SG	-0.054082243	4.26E-09
B120	B189	HCV-cAg	eGFB	-0.053829236	5.03E-09
B120	B151	HCV-cAg	Anti-CCP	-0.053189104	7.62E-09
B120	B146	HCV-cAg	CysC	-0.050164775	5.10E-08
B120	B155	HCV-cAg	IgA	-0.046519238	4.39E-07
B120	B222	HCV-cAg	NS	-0.046093749	5.58E-07
B120	B126	HCV-cAg	Alb	-0.04592541	6.14E-07
B121	B122	C3	C4	0.915742313	0

B121	B228	C3	OS	-0.07228222	4.00E-15
B121	B227	C3	OD	-0.071143069	1.07E-14
B121	B157	C3	IgG	-0.05553465	1.62E-09
B121	B134	C3	CHE	0.053480788	6.31E-09
B122	B228	C4	OS	-0.056848148	6.63E-10
B122	B227	C4	OD	-0.055364842	1.82E-09
B122	B134	C4	CHE	0.053231354	7.42E-09
B123	B136	hs-CRP1	5'NT	0.07927594	0
B123	B138	hs-CRP1	PAB	-0.099772647	0
B123	B139	hs-CRP1	ADA	0.088487414	0
B123	B169	hs-CRP1	U-CRY	0.206797891	0
B123	B175	hs-CRP1	ALB-U	0.11435067	0
B123	B177	hs-CRP1	ACR	0.106257616	0
B123	B127	hs-CRP1	A-G ratio	-0.074321807	6.66E-16
B123	B130	hs-CRP1	GLB	0.069655581	3.77E-14
B123	B155	hs-CRP1	IgA	0.066164588	6.54E-13
B123	B126	hs-CRP1	Alb	-0.06456283	2.31E-12
B123	B194	hs-CRP1	Cl	-0.057760662	3.52E-10
B123	B186	hs-CRP1	PFC	0.053372158	6.77E-09
B123	B153	hs-CRP1	Glu	0.050554763	4.02E-08
B123	B195	hs-CRP1	Na	-0.0495951	7.21E-08
B123	B150	hs-CRP1	ALP	0.049145903	9.45E-08
B123	B199	hs-CRP1	Mg	-0.048795907	1.16E-07
B124	B125	ACTH	Cor	0.476475727	0
B124	B196	ACTH	TCO2	0.056503972	8.40E-10
B124	B183	ACTH	PTA	-0.056326563	9.48E-10
B124	B181	ACTH	APTT	-0.052687478	1.05E-08
B124	B200	ACTH	OP	0.047216808	2.94E-07
B125	B151	Cor	Anti-CCP	0.144695375	0
B125	B181	Cor	APTT	-0.0793659	0
B125	B182	Cor	TT	-0.100135988	0
B125	B186	Cor	PFC	0.20583999	0
B125	B138	Cor	PAB	0.073112152	1.78E-15
B125	B139	Cor	ADA	-0.063431229	5.54E-12
B125	B183	Cor	PTA	-0.059372734	1.12E-10
B125	B196	Cor	TCO2	0.05502892	2.28E-09
B125	B197	Cor	AG	-0.054352161	3.57E-09
B125	B150	Cor	ALP	-0.048918453	1.08E-07
B125	B136	Cor	5'NT	-0.048551751	1.35E-07
B126	B127	Alb	A-G ratio	0.496530408	0
B126	B128	Alb	ALT	0.08210408	0
B126	B129	Alb	IBIL	0.124681794	0
B126	B130	Alb	GLB	-0.16367333	0
B126	B133	Alb	TBIL	0.110072174	0

B126	B134	Alb	CHE	0.089039799	0
B126	B138	Alb	PAB	0.107937531	0
B126	B141	Alb	AST/ALT	-0.138423483	0
B126	B188	Alb	Ca	0.416879599	0
B126	B189	Alb	eGFB	0.23961717	0
B126	B192	Alb	UA	0.089274033	0
B126	B216	Alb	BMI	0.112134079	0
B126	B218	Alb	DBP	0.149477192	0
B126	B221	Alb	Hei	0.115950089	0
B126	B222	Alb	NS	0.108031258	0
B126	B177	Alb	ACR	-0.075055765	4.44E-16
B126	B175	Alb	ALB-U	-0.073506476	1.33E-15
B126	B191	Alb	Urea	-0.069758486	3.46E-14
B126	B163	Alb	SG	0.069270557	5.20E-14
B126	B190	Alb	Cre	-0.066156656	6.59E-13
B126	B160	Alb	PH	-0.061022312	3.38E-11
B126	B184	Alb	INR	-0.058615524	1.93E-10
B126	B161	Alb	LEU	-0.055797572	1.36E-09
B126	B224	Alb	pmh	0.05557047	1.58E-09
B126	B139	Alb	ADA	-0.055221416	2.00E-09
B126	B215	Alb	BMI	0.054934495	2.42E-09
B126	B185	Alb	PT	-0.054869158	2.53E-09
B126	B181	Alb	APTT	-0.054810476	2.63E-09
B126	B229	Alb	slit-lamp	-0.054292416	3.71E-09
B126	B194	Alb	Cl	-0.049733852	6.63E-08
B126	B212	Alb	IOP-L	0.047480987	2.52E-07
B126	B204	Alb	Drinking	0.046158788	5.38E-07
B127	B129	A-G ratio	IBIL	0.133510943	0
B127	B130	A-G ratio	GLB	-0.896251792	0
B127	B131	A-G ratio	AST	-0.081930425	0
B127	B132	A-G ratio	Dbil	0.077190354	0
B127	B133	A-G ratio	TBIL	0.128491623	0
B127	B139	A-G ratio	ADA	-0.082066925	0
B127	B141	A-G ratio	AST/ALT	-0.080931356	0
B127	B146	A-G ratio	CysC	-0.079347961	0
B127	B153	A-G ratio	Glu	-0.111847188	0
B127	B155	A-G ratio	IgA	-0.091089436	0
B127	B157	A-G ratio	IgG	-0.095370226	0
B127	B189	A-G ratio	eGFB	0.165972164	0
B127	B204	A-G ratio	Drinking	0.085767361	0
B127	B209	A-G ratio	Smoking	0.112339717	0
B127	B216	A-G ratio	BMI	0.080188298	0
B127	B220	A-G ratio	SBP	-0.133506395	0
B127	B221	A-G ratio	Hei	0.168866173	0

B127	B222	A-G ratio	NS	0.079935365	0
B127	B191	A-G ratio	Urea	-0.068733355	8.13E-14
B127	B138	A-G ratio	PAB	0.067750802	1.82E-13
B127	B161	A-G ratio	LEU	-0.0662527	6.10E-13
B127	B140	A-G ratio	r-GT	-0.065700294	9.46E-13
B127	B177	A-G ratio	ACR	-0.063837669	4.05E-12
B127	B170	A-G ratio	U-GLU	-0.061425674	2.51E-11
B127	B150	A-G ratio	ALP	-0.060091012	6.69E-11
B127	B163	A-G ratio	SG	0.052559799	1.14E-08
B127	B178	A-G ratio	Ig-U	-0.049222459	9.03E-08
B127	B188	A-G ratio	Ca	0.047726818	2.19E-07
B127	B158	A-G ratio	IgM	-0.045165572	9.38E-07
B128	B131	ALT	AST	0.839704026	0
B128	B132	ALT	Dbil	0.247248388	0
B128	B133	ALT	TBIL	0.12031803	0
B128	B140	ALT	r-GT	0.325376302	0
B128	B141	ALT	AST/ALT	-0.384997413	0
B128	B165	ALT	bilirubin	0.188321606	0
B128	B189	ALT	eGFB	0.094108084	0
B128	B192	ALT	UA	0.09701503	0
B128	B206	ALT	Waist	0.122039507	0
B128	B210	ALT	Hip	0.096240229	0
B128	B215	ALT	BMI	0.136293864	0
B128	B216	ALT	BMI	0.146439356	0
B128	B222	ALT	NS	0.118980841	0
B128	B221	ALT	Hei	0.073578064	1.33E-15
B128	B129	ALT	IBIL	0.062747158	9.32E-12
B128	B136	ALT	5'NT	0.062321566	1.29E-11
B128	B139	ALT	ADA	0.056767454	7.01E-10
B128	B218	ALT	DBP	0.054642375	2.94E-09
B128	B137	ALT	AFU	0.053685543	5.52E-09
B128	B130	ALT	GLB	0.051499334	2.23E-08
B128	B163	ALT	SG	0.051309503	2.52E-08
B128	B150	ALT	ALP	0.045893614	6.25E-07
B129	B130	IBIL	GLB	-0.111101093	0
B129	B131	IBIL	AST	0.083140716	0
B129	B132	IBIL	Dbil	0.621324441	0
B129	B133	IBIL	TBIL	0.974978086	0
B129	B163	IBIL	SG	0.078786703	0
B129	B209	IBIL	Smoking	-0.082572874	0
B129	B221	IBIL	Hei	0.067318384	2.59E-13
B129	B174	IBIL	Urea/Cr	-0.063507679	5.22E-12
B129	B176	IBIL	Ucr	0.057214812	5.15E-10
B129	B224	IBIL	pmh	-0.053874612	4.88E-09

B129	B150	IBIL	ALP	-0.053483127	6.30E-09
B130	B131	GLB	AST	0.102089095	0
B130	B133	GLB	TBIL	-0.102203578	0
B130	B139	GLB	ADA	0.077513083	0
B130	B140	GLB	r-GT	0.106473556	0
B130	B146	GLB	CysC	0.093925074	0
B130	B153	GLB	Glu	0.140254837	0
B130	B155	GLB	IgA	0.10697984	0
B130	B157	GLB	IgG	0.092856584	0
B130	B188	GLB	Ca	0.114431464	0
B130	B189	GLB	eGFB	-0.086292637	0
B130	B209	GLB	Smoking	-0.119421375	0
B130	B220	GLB	SBP	0.137007578	0
B130	B221	GLB	Hei	-0.142748976	0
B130	B150	GLB	ALP	0.072110054	4.66E-15
B130	B194	GLB	Cl	-0.071977091	5.33E-15
B130	B204	GLB	Drinking	-0.069548831	4.13E-14
B130	B170	GLB	U-GLU	0.067434873	2.35E-13
B130	B158	GLB	IgM	0.065111123	1.51E-12
B130	B192	GLB	UA	0.064563227	2.31E-12
B130	B161	GLB	LEU	0.057030845	5.85E-10
B130	B136	GLB	5'NT	0.054550053	3.13E-09
B130	B218	GLB	DBP	0.052587325	1.12E-08
B130	B191	GLB	Urea	0.052380858	1.28E-08
B130	B177	GLB	ACR	0.049941042	5.85E-08
B130	B216	GLB	BMI	-0.049299447	8.62E-08
B130	B222	GLB	NS	-0.048735008	1.21E-07
B130	B174	GLB	Urea/Cr	0.048609457	1.30E-07
B130	B160	GLB	PH	-0.048555592	1.34E-07
B130	B215	GLB	BMI	0.048364492	1.50E-07
B130	B178	GLB	Ig-U	0.048167638	1.69E-07
B130	B182	GLB	TT	0.047805577	2.09E-07
B130	B132	GLB	Dbil	-0.047741825	2.17E-07
B131	B132	AST	Dbil	0.2919091	0
B131	B133	AST	TBIL	0.149206675	0
B131	B140	AST	r-GT	0.348564231	0
B131	B165	AST	bilirubin	0.223779562	0
B131	B192	AST	UA	0.072350186	3.77E-15
B131	B139	AST	ADA	0.065993605	7.50E-13
B131	B135	AST	TBA	0.064288285	2.86E-12
B131	B137	AST	AFU	0.06187508	1.80E-11
B131	B136	AST	5'NT	0.058423798	2.21E-10
B131	B141	AST	AST/ALT	-0.056980201	6.05E-10
B131	B215	AST	BMI	0.054348067	3.58E-09

B132	B133	Dbil	TBIL	0.779759999	0
B132	B140	Dbil	r-GT	0.12597885	0
B132	B165	Dbil	bilirubin	0.148546435	0
B132	B184	Dbil	INR	0.096828849	0
B132	B185	Dbil	PT	0.092662611	0
B132	B209	Dbil	Smoking	-0.06818649	1.27E-13
B132	B221	Dbil	Hei	0.06088012	3.76E-11
B132	B189	Dbil	eGFB	-0.059501141	1.02E-10
B132	B174	Dbil	Urea/Cr	-0.056286775	9.74E-10
B132	B181	Dbil	APTT	0.054627412	2.97E-09
B132	B224	Dbil	pmh	-0.053638956	5.69E-09
B132	B204	Dbil	Drinking	0.051280468	2.56E-08
B133	B209	TBIL	Smoking	-0.08531537	0
B133	B163	TBIL	SG	0.07384683	8.88E-16
B133	B221	TBIL	Hei	0.071172547	1.04E-14
B133	B174	TBIL	Urea/Cr	-0.066592236	4.65E-13
B133	B165	TBIL	bilirubin	0.063742577	4.36E-12
B133	B140	TBIL	r-GT	0.059590572	9.60E-11
B133	B224	TBIL	pmh	-0.058312892	2.39E-10
B133	B176	TBIL	Ucr	0.055235976	1.98E-09
B133	B150	TBIL	ALP	-0.048028292	1.83E-07
B134	B136	CHE	5'NT	0.125452723	0
B134	B137	CHE	AFU	0.097672439	0
B134	B138	CHE	PAB	0.313210998	0
B134	B188	CHE	Ca	0.077861455	0
B134	B194	CHE	Cl	-0.214421049	0
B134	B195	CHE	Na	-0.118117354	0
B134	B197	CHE	AG	0.110449858	0
B134	B200	CHE	OP	0.096078236	0
B134	B150	CHE	ALP	0.072914716	2.22E-15
B134	B157	CHE	IgG	-0.070322681	2.13E-14
B134	B215	CHE	BMI	0.069432181	4.53E-14
B134	B199	CHE	Mg	-0.068350396	1.11E-13
B134	B139	CHE	ADA	-0.065697086	9.49E-13
B134	B216	CHE	BMI	0.060116591	6.56E-11
B134	B141	CHE	AST/ALT	-0.05777616	3.48E-10
B134	B155	CHE	IgA	0.056598163	7.87E-10
B134	B206	CHE	Waist	0.05619327	1.04E-09
B134	B193	CHE	K	0.055374471	1.81E-09
B134	B222	CHE	NS	0.053809738	5.09E-09
B134	B152	CHE	ACA-Ig(G-A-M)	0.052158333	1.47E-08
B134	B212	CHE	IOP-L	0.050404366	4.40E-08
B134	B198	CHE	P	0.048882316	1.11E-07
B134	B184	CHE	INR	-0.04685353	3.62E-07

B134	B153	CHE	Glu	0.045594984	7.39E-07
B135	B137	TBA	AFU	0.133413143	0
B135	B139	TBA	ADA	0.075523914	2.22E-16
B135	B196	TBA	TCO2	0.072782408	2.66E-15
B135	B198	TBA	P	-0.068972742	6.66E-14
B135	B138	TBA	PAB	-0.0604717	5.07E-11
B135	B150	TBA	ALP	0.059859099	7.91E-11
B135	B181	TBA	APTT	0.05681133	6.80E-10
B135	B200	TBA	OP	0.047021979	3.29E-07
B135	B197	TBA	AG	-0.046537838	4.34E-07
B135	B199	TBA	Mg	-0.046147107	5.42E-07
B136	B137	5'NT	AFU	0.120122202	0
B136	B139	5'NT	ADA	0.183702751	0
B136	B140	5'NT	r-GT	0.189492724	0
B136	B150	5'NT	ALP	0.295049958	0
B136	B186	5'NT	PFC	0.112363583	0
B136	B194	5'NT	Cl	-0.121414941	0
B136	B195	5'NT	Na	-0.093533445	0
B136	B197	5'NT	AG	0.07528829	4.44E-16
B136	B151	5'NT	Anti-CCP	0.0568385	6.68E-10
B136	B193	5'NT	K	-0.05677711	6.96E-10
B136	B196	5'NT	TCO2	-0.055731544	1.42E-09
B136	B153	5'NT	Glu	0.055388062	1.79E-09
B136	B146	5'NT	CysC	0.055007977	2.31E-09
B136	B157	5'NT	IgG	-0.049884745	6.05E-08
B136	B182	5'NT	TT	0.049800079	6.37E-08
B136	B138	5'NT	PAB	-0.046453392	4.55E-07
B136	B176	5'NT	Ucr	0.045660257	7.12E-07
B136	B170	5'NT	U-GLU	0.045121179	9.61E-07
B137	B139	AFU	ADA	0.259208	0
B137	B150	AFU	ALP	0.118204812	0
B137	B194	AFU	Cl	-0.109059676	0
B137	B196	AFU	TCO2	0.079779426	0
B137	B199	AFU	Mg	-0.107625741	0
B137	B200	AFU	OP	0.274833152	0
B137	B170	AFU	U-GLU	0.06062916	4.52E-11
B137	B204	AFU	Drinking	0.058225686	2.54E-10
B137	B140	AFU	r-GT	0.057116368	5.51E-10
B137	B153	AFU	Glu	0.055658507	1.49E-09
B137	B152	AFU	ACA-Ig(G-A-M)	-0.048898976	1.10E-07
B138	B139	PAB	ADA	-0.330954142	0
B138	B150	PAB	ALP	-0.129373925	0
B138	B157	PAB	IgG	-0.086745142	0
B138	B188	PAB	Ca	0.076837548	0

B138	B192	PAB	UA	0.090189344	0
B138	B194	PAB	Cl	-0.087842202	0
B138	B197	PAB	AG	0.155706778	0
B138	B204	PAB	Drinking	0.068601289	9.06E-14
B138	B200	PAB	OP	0.06756558	2.12E-13
B138	B158	PAB	IgM	-0.065143227	1.47E-12
B138	B181	PAB	APTT	-0.064004162	3.57E-12
B138	B184	PAB	INR	-0.063642543	4.71E-12
B138	B199	PAB	Mg	0.061965747	1.68E-11
B138	B195	PAB	Na	0.060735557	4.18E-11
B138	B196	PAB	TCO2	-0.060624179	4.53E-11
B138	B216	PAB	BMI	0.058962694	1.51E-10
B138	B222	PAB	NS	0.056616658	7.77E-10
B138	B185	PAB	PT	-0.055984128	1.20E-09
B138	B221	PAB	Hei	0.053967319	4.59E-09
B138	B148	PAB	Anti_TPO	-0.047222166	2.93E-07
B139	B150	ADA	ALP	0.219149164	0
B139	B155	ADA	IgA	0.087067794	0
B139	B182	ADA	TT	0.09025426	0
B139	B186	ADA	PFC	0.233212149	0
B139	B194	ADA	Cl	-0.236119898	0
B139	B195	ADA	Na	-0.161971685	0
B139	B199	ADA	Mg	-0.152224563	0
B139	B200	ADA	OP	0.166402871	0
B139	B153	ADA	Glu	0.064719289	2.05E-12
B139	B197	ADA	AG	0.062796135	8.98E-12
B139	B140	ADA	r-GT	0.058974423	1.49E-10
B139	B212	ADA	IOP-L	0.05858885	1.97E-10
B139	B170	ADA	U-GLU	0.05512893	2.13E-09
B139	B157	ADA	IgG	0.046540767	4.33E-07
B14	B93	G-17	ALDH2-Glu504Lys	0.078152312	0
B14	B92	G-17	ALDH2-Glu504Glu	-0.073164982	1.78E-15
B14	B81	G-17	TSGF	0.069349715	4.86E-14
B14	B20	G-17	CD3+CD4+CD8+	0.065915704	7.98E-13
B14	B80	G-17	anti-HP IgG	0.055219277	2.00E-09
B14	B138	G-17	PAB	-0.054891173	2.50E-09
B14	B150	G-17	ALP	0.05217293	1.46E-08
B14	B23	G-17	CD3-CD16+CD56+(NK)	-0.049572104	7.31E-08
B140	B141	r-GT	AST/ALT	-0.13284524	0
B140	B150	r-GT	ALP	0.083221662	0
B140	B153	r-GT	Glu	0.118641463	0
B140	B163	r-GT	SG	0.091717671	0
B140	B165	r-GT	bilirubin	0.158598418	0
B140	B170	r-GT	U-GLU	0.114717807	0

B140	B176	r-GT	Ucr	0.095602896	0
B140	B189	r-GT	eGFB	0.098150686	0
B140	B192	r-GT	UA	0.079262781	0
B140	B204	r-GT	Drinking	0.133214396	0
B140	B206	r-GT	Waist	0.123096261	0
B140	B210	r-GT	Hip	0.083332826	0
B140	B215	r-GT	BMI	0.106164571	0
B140	B216	r-GT	BMI	0.129096772	0
B140	B218	r-GT	DBP	0.094087423	0
B140	B221	r-GT	Hei	0.082797095	0
B140	B222	r-GT	NS	0.136083199	0
B140	B209	r-GT	Smoking	0.072512824	3.11E-15
B140	B172	r-GT	KB	0.065102988	1.52E-12
B140	B179	r-GT	α1-MG	0.062152566	1.46E-11
B140	B191	r-GT	Urea	-0.053177863	7.68E-09
B140	B186	r-GT	PFC	0.046365428	4.79E-07
B140	B166	r-GT	Pro	0.046008361	5.86E-07
B140	B174	r-GT	Urea/Cr	-0.045116866	9.63E-07
B141	B153	AST/ALT	Glu	-0.108662443	0
B141	B163	AST/ALT	SG	-0.108888174	0
B141	B170	AST/ALT	U-GLU	-0.086535331	0
B141	B189	AST/ALT	eGFB	-0.136937964	0
B141	B192	AST/ALT	UA	-0.089405788	0
B141	B206	AST/ALT	Waist	-0.154261034	0
B141	B210	AST/ALT	Hip	-0.11163458	0
B141	B215	AST/ALT	BMI	-0.167306221	0
B141	B216	AST/ALT	BMI	-0.200008335	0
B141	B221	AST/ALT	Hei	-0.128382674	0
B141	B222	AST/ALT	NS	-0.157542093	0
B141	B218	AST/ALT	DBP	-0.072881924	2.22E-15
B141	B220	AST/ALT	SBP	0.065201611	1.40E-12
B141	B176	AST/ALT	Ucr	-0.052622017	1.10E-08
B141	B188	AST/ALT	Ca	-0.050867977	3.31E-08
B141	B160	AST/ALT	PH	0.048446202	1.43E-07
B141	B224	AST/ALT	pmh	0.045315626	8.63E-07
B142	B143	25-(OH)D	β-CTx	-0.162459542	0
B142	B144	25-(OH)D	Total-PINP	-0.1066258	0
B142	B151	25-(OH)D	Anti-CCP	-0.108794785	0
B142	B173	25-(OH)D	NTT	0.053591593	5.87E-09
B142	B176	25-(OH)D	Ucr	-0.052830888	9.60E-09
B142	B145	25-(OH)D	BGP	-0.052121802	1.51E-08
B143	B144	β-CTx	Total-PINP	0.830006212	0
B143	B145	β-CTx	BGP	0.606028461	0
B143	B150	β-CTx	ALP	0.077129894	0

B143	B151	β-CTx	Anti-CCP	0.083865835	0
B143	B167	β-CTx	PC	0.066050433	7.17E-13
B143	B168	β-CTx	NMP22	-0.064405684	2.61E-12
B143	B183	β-CTx	PTA	-0.053781522	5.19E-09
B143	B176	β-CTx	Ucr	0.048652493	1.27E-07
B144	B145	Total-PINP	BGP	0.670825076	0
B144	B150	Total-PINP	ALP	0.0981478	0
B144	B151	Total-PINP	Anti-CCP	0.140267004	0
B144	B183	Total-PINP	PTA	-0.076652745	0
B144	B167	Total-PINP	PC	0.069520285	4.22E-14
B144	B168	Total-PINP	NMP22	-0.062763537	9.21E-12
B145	B150	BGP	ALP	0.10807254	0
B145	B167	BGP	PC	0.083742913	0
B145	B183	BGP	PTA	-0.117629279	0
B145	B182	BGP	TT	0.056722196	7.23E-10
B145	B203	BGP	DH	0.047105472	3.14E-07
B146	B182	CysC	TT	0.105068777	0
B146	B189	CysC	eGFB	-0.160940935	0
B146	B190	CysC	Cre	0.123408848	0
B146	B191	CysC	Urea	0.070706355	1.55E-14
B146	B155	CysC	IgA	0.070225714	2.33E-14
B146	B174	CysC	Urea/Cr	-0.063664422	4.63E-12
B146	B192	CysC	UA	0.062961692	7.92E-12
B146	B163	CysC	SG	-0.050207711	4.97E-08
B146	B152	CysC	ACA-Ig(G-A-M)	-0.04955728	7.38E-08
B146	B200	CysC	OP	0.047764802	2.14E-07
B146	B198	CysC	P	-0.046794083	3.75E-07
B148	B149	Anti_TPO	TGAb	0.392114459	0
B148	B222	Anti_TPO	NS	-0.061901786	1.76E-11
B148	B181	Anti_TPO	APTT	0.056091393	1.11E-09
B148	B221	Anti_TPO	Hei	-0.053556313	6.01E-09
B148	B216	Anti_TPO	BMI	-0.052076628	1.55E-08
B148	B176	Anti_TPO	Ucr	-0.048352617	1.52E-07
B149	B222	TGAb	NS	-0.053118678	7.98E-09
B149	B221	TGAb	Hei	-0.051338743	2.47E-08
B15	B101	CD3-CD19+(B cell)	anti-Sc70	0.364725366	0
B15	B103	CD3-CD19+(B cell)	anti-SSA/Ro	0.078369868	0
B15	B104	CD3-CD19+(B cell)	anti-SSB	0.364725304	0
B15	B105	CD3-CD19+(B cell)	anti-U1RNP	-0.102732383	0
B15	B107	CD3-CD19+(B cell)	AnuA	0.366829111	0
B15	B119	CD3-CD19+(B cell)	Hcv-rna	0.119864426	0
B15	B120	CD3-CD19+(B cell)	HCV-cAg	0.128562754	0
B15	B138	CD3-CD19+(B cell)	PAB	-0.08742482	0
B15	B155	CD3-CD19+(B cell)	IgA	0.11782687	0

B15	B157	CD3-CD19+(B cell)	IgG	0.181660918	0
B15	B158	CD3-CD19+(B cell)	IgM	0.113373509	0
B15	B16	CD3-CD19+(B cell)	CD3+T	-0.349822511	0
B15	B17	CD3-CD19+(B cell)	CD3+CD16+CD56+NK	-0.162919801	0
B15	B181	CD3-CD19+(B cell)	APTT	0.141318463	0
B15	B183	CD3-CD19+(B cell)	PTA	0.132189333	0
B15	B184	CD3-CD19+(B cell)	INR	0.115478801	0
B15	B19	CD3-CD19+(B cell)	CD3+CD4+/CD3+CD8+	0.143914863	0
B15	B20	CD3-CD19+(B cell)	CD3+CD4+CD8+	-0.16236123	0
B15	B200	CD3-CD19+(B cell)	OP	-0.107138405	0
B15	B21	CD3-CD19+(B cell)	CD3+CD4-CD8-	-0.172248326	0
B15	B22	CD3-CD19+(B cell)	CD3+CD8+(T)	-0.29538826	0
B15	B79	CD3-CD19+(B cell)	hb V -dna	0.140413696	0
B15	B96	CD3-CD19+(B cell)	dsDNA (IIF)	0.125350753	0
B15	B98	CD3-CD19+(B cell)	anti-Jo-1	0.364725276	0
B15	B194	CD3-CD19+(B cell)	Cl	0.070840085	1.38E-14
B15	B151	CD3-CD19+(B cell)	Anti-CCP	-0.070351524	2.09E-14
B15	B90	CD3-CD19+(B cell)	CYFRA 21-1	-0.069675854	3.69E-14
B15	B57	CD3-CD19+(B cell)	RBCAI	-0.06839042	1.08E-13
B15	B106	CD3-CD19+(B cell)	anti-P	0.067893497	1.62E-13
B15	B102	CD3-CD19+(B cell)	anti-Sm	0.067885497	1.63E-13
B15	B136	CD3-CD19+(B cell)	5'NT	-0.0670597	3.19E-13
B15	B94	CD3-CD19+(B cell)	ALDH2-Lys504Lys	0.066075702	7.03E-13
B15	B60	CD3-CD19+(B cell)	WBRVI	-0.064078324	3.37E-12
B15	B31	CD3-CD19+(B cell)	FSH	-0.063543004	5.08E-12
B15	B95	CD3-CD19+(B cell)	anti-cenpb	0.061040919	3.34E-11
B15	B59	CD3-CD19+(B cell)	WBRV	-0.059444159	1.07E-10
B15	B65	CD3-CD19+(B cell)	WBRSR(mPas_1)	-0.059387507	1.11E-10
B15	B212	CD3-CD19+(B cell)	IOP-L	-0.057154511	5.37E-10
B15	B122	CD3-CD19+(B cell)	C4	-0.056472209	8.58E-10
B15	B185	CD3-CD19+(B cell)	PT	0.0551429	2.11E-09
B15	B93	CD3-CD19+(B cell)	ALDH2-Glu504Lys	-0.053655378	5.63E-09
B15	B115	CD3-CD19+(B cell)	TSH TT3 TT4(TT3)	0.051206779	2.68E-08
B15	B139	CD3-CD19+(B cell)	ADA	-0.048830774	1.14E-07
B15	B29	CD3-CD19+(B cell)	E2	0.048602482	1.31E-07
B15	B18	CD3-CD19+(B cell)	CD3+CD4+helper(inducer)T	-0.048359918	1.51E-07
B15	B186	CD3-CD19+(B cell)	PFC	-0.048299975	1.56E-07
B15	B124	CD3-CD19+(B cell)	ACTH	-0.04772244	2.19E-07
B15	B54	CD3-CD19+(B cell)	ESR	0.046293491	4.99E-07
B150	B153	ALP	Glu	0.086222518	0
B150	B170	ALP	U-GLU	0.088043359	0
B150	B194	ALP	Cl	-0.107517107	0
B150	B200	ALP	OP	0.104668445	0
B150	B186	ALP	PFC	0.067007756	3.33E-13

B150	B195	ALP	Na	-0.06334839	5.90E-12
B150	B189	ALP	eGFB	0.055251277	1.96E-09
B150	B199	ALP	Mg	-0.050215676	4.94E-08
B150	B174	ALP	Urea/Cr	0.047516478	2.47E-07
B150	B190	ALP	Cre	-0.04693737	3.45E-07
B151	B157	Anti-CCP	IgG	0.081069552	0
B151	B183	Anti-CCP	PTA	-0.168138053	0
B151	B186	Anti-CCP	PFC	0.082836286	0
B151	B152	Anti-CCP	ACA-Ig(G-A-M)	0.073986997	8.88E-16
B151	B182	Anti-CCP	TT	-0.060587963	4.65E-11
B151	B155	Anti-CCP	IgA	0.059781288	8.37E-11
B151	B194	Anti-CCP	Cl	-0.05417684	4.00E-09
B151	B156	Anti-CCP	IgE	0.053201996	7.56E-09
B151	B158	Anti-CCP	IgM	0.048588034	1.32E-07
B151	B168	Anti-CCP	NMP22	-0.047072045	3.20E-07
B151	B179	Anti-CCP	α1-MG	0.045126717	9.58E-07
B152	B200	ACA-Ig(G-A-M)	OP	-0.048322506	1.54E-07
B153	B160	Glu	PH	-0.140239199	0
B153	B163	Glu	SG	0.333646115	0
B153	B166	Glu	Pro	0.104487774	0
B153	B170	Glu	U-GLU	0.640971164	0
B153	B172	Glu	KB	0.201293372	0
B153	B174	Glu	Urea/Cr	0.172239274	0
B153	B188	Glu	Ca	0.084574579	0
B153	B189	Glu	eGFB	0.129067827	0
B153	B190	Glu	Cre	-0.093903693	0
B153	B192	Glu	UA	-0.130506112	0
B153	B194	Glu	Cl	-0.076018155	0
B153	B195	Glu	Na	-0.092657492	0
B153	B206	Glu	Waist	0.084258837	0
B153	B218	Glu	DBP	0.081509543	0
B153	B222	Glu	NS	0.077359746	0
B153	B216	Glu	BMI	0.073923182	8.88E-16
B153	B209	Glu	Smoking	0.066005309	7.43E-13
B153	B204	Glu	Drinking	0.064682439	2.11E-12
B153	B191	Glu	Urea	0.061801296	1.90E-11
B153	B215	Glu	BMI	0.058528481	2.05E-10
B153	B179	Glu	α1-MG	0.052869654	9.36E-09
B153	B200	Glu	OP	0.049741185	6.60E-08
B153	B219	Glu	OT	-0.049455532	7.85E-08
B153	B221	Glu	Hei	0.049205663	9.12E-08
B153	B225	Glu	hPBG	0.048174817	1.68E-07
B154	B157	RF	IgG	0.082065732	0
B155	B157	IgA	IgG	0.188006599	0

B155	B182	IgA	TT	0.084108475	0
B155	B194	IgA	Cl	-0.084358584	0
B155	B156	IgA	IgE	0.071273225	9.55E-15
B155	B195	IgA	Na	-0.068368276	1.10E-13
B155	B178	IgA	Ig-U	0.056733773	7.17E-10
B155	B186	IgA	PFC	0.04868215	1.25E-07
B155	B180	IgA	Tf	0.047242642	2.90E-07
B155	B170	IgA	U-GLU	0.046623004	4.13E-07
B156	B158	IgE	IgM	-0.059891125	7.73E-11
B157	B158	IgG	IgM	0.098018984	0
B157	B200	IgG	OP	-0.128973452	0
B157	B184	IgG	INR	0.049714243	6.71E-08
B157	B199	IgG	Mg	0.047843578	2.04E-07
B159	B186	β2-MG	PFC	0.052221081	1.42E-08
B159	B181	β2-MG	APTT	-0.045325652	8.58E-07
B16	B101	CD3+T	anti-Sc70	-0.121056924	0
B16	B104	CD3+T	anti-SSB	-0.121056889	0
B16	B107	CD3+T	AnuA	-0.130646618	0
B16	B108	CD3+T	Anti-Cyclin	0.086971045	0
B16	B121	CD3+T	C3	0.232797363	0
B16	B122	CD3+T	C4	0.223536338	0
B16	B152	CD3+T	ACA-Ig(G-A-M)	-0.086388218	0
B16	B155	CD3+T	IgA	-0.08634152	0
B16	B157	CD3+T	IgG	-0.107651359	0
B16	B17	CD3+T	CD3+CD16+CD56+NK	0.202889622	0
B16	B18	CD3+T	CD3+CD4+helper(inducer)T	0.392382531	0
B16	B19	CD3+T	CD3+CD4+/CD3+CD8+	-0.21752396	0
B16	B193	CD3+T	K	-0.077229238	0
B16	B20	CD3+T	CD3+CD4+CD8+	0.200174466	0
B16	B21	CD3+T	CD3+CD4-CD8-	0.320225356	0
B16	B22	CD3+T	CD3+CD8+(T)	0.573832381	0
B16	B23	CD3+T	CD3-CD16+CD56+(NK)	-0.844743191	0
B16	B95	CD3+T	anti-cenpb	-0.092187338	0
B16	B98	CD3+T	anti-Jo-1	-0.121056872	0
B16	B99	CD3+T	anti-PM-Scl	0.076350756	0
B16	B105	CD3+T	anti-U1RNP	0.074761967	4.44E-16
B16	B181	CD3+T	APTT	-0.072450739	3.55E-15
B16	B79	CD3+T	hb V -dna	-0.070922453	1.29E-14
B16	B74	CD3+T	ApoA1	-0.06854504	9.48E-14
B16	B124	CD3+T	ACTH	0.066998221	3.35E-13
B16	B31	CD3+T	FSH	0.061877937	1.79E-11
B16	B200	CD3+T	OP	0.059190576	1.28E-10
B16	B212	CD3+T	IOP-L	0.051394329	2.39E-08
B16	B184	CD3+T	INR	-0.050790733	3.47E-08

B16	B29	CD3+T	E2	-0.048486981	1.40E-07
B160	B163	PH	SG	-0.132155739	0
B160	B170	PH	U-GLU	-0.124360712	0
B160	B191	PH	Urea	-0.119409062	0
B160	B192	PH	UA	-0.090003147	0
B160	B188	PH	Ca	-0.067424428	2.38E-13
B160	B174	PH	Urea/Cr	-0.062897824	8.32E-12
B160	B172	PH	KB	-0.058652864	1.88E-10
B160	B206	PH	Waist	-0.058403517	2.24E-10
B161	B173	LEU	NTT	0.199866777	0
B161	B216	LEU	BMI	-0.089633497	0
B161	B221	LEU	Hei	-0.154354806	0
B161	B222	LEU	NS	-0.115783754	0
B161	B174	LEU	Urea/Cr	0.074197503	8.88E-16
B161	B209	LEU	Smoking	-0.067723103	1.86E-13
B161	B204	LEU	Drinking	-0.06408317	3.35E-12
B161	B163	LEU	SG	-0.061504113	2.37E-11
B161	B189	LEU	eGFB	-0.046003161	5.87E-07
B163	B166	SG	Pro	0.303321381	0
B163	B170	SG	U-GLU	0.389458195	0
B163	B172	SG	KB	0.201680095	0
B163	B174	SG	Urea/Cr	0.127218059	0
B163	B176	SG	Ucr	0.265943159	0
B163	B189	SG	eGFB	0.135366891	0
B163	B191	SG	Urea	0.182995106	0
B163	B204	SG	Drinking	0.125087882	0
B163	B206	SG	Waist	0.150292224	0
B163	B209	SG	Smoking	0.158015157	0
B163	B210	SG	Hip	0.100734538	0
B163	B215	SG	BMI	0.097317456	0
B163	B216	SG	BMI	0.220839347	0
B163	B220	SG	SBP	-0.106016069	0
B163	B221	SG	Hei	0.246427884	0
B163	B222	SG	NS	0.254602945	0
B163	B179	SG	α1-MG	0.075084982	4.44E-16
B163	B219	SG	OT	-0.068119746	1.35E-13
B163	B173	SG	NTT	-0.063540094	5.10E-12
B163	B218	SG	DBP	0.057447711	4.38E-10
B163	B201	SG	e-habits	-0.05215532	1.48E-08
B163	B200	SG	OP	0.046728348	3.89E-07
B165	B176	bilirubin	Ucr	0.060276704	5.84E-11
B165	B179	bilirubin	α1-MG	0.05987408	7.83E-11
B166	B170	Pro	U-GLU	0.179955831	0
B166	B172	Pro	KB	0.135624847	0

B166	B176	Pro	Ucr	0.135112922	0
B166	B190	Pro	Cre	0.097176899	0
B166	B191	Pro	Urea	0.103857261	0
B166	B209	Pro	Smoking	0.078659842	0
B166	B216	Pro	BMI	0.078991785	0
B166	B221	Pro	Hei	0.088779781	0
B166	B222	Pro	NS	0.113190445	0
B166	B206	Pro	Waist	0.072724461	2.66E-15
B166	B218	Pro	DBP	0.057365523	4.64E-10
B169	B171	U-CRY	EC	-0.120950806	0
B169	B204	U-CRY	Drinking	0.058364318	2.30E-10
B17	B134	CD3+CD16+CD56+NK	CHE	-0.081946334	0
B17	B164	CD3+CD16+CD56+NK	COM	-0.075856114	0
B17	B18	CD3+CD16+CD56+NK	CD3+CD4+helper(inducer)T	-0.129103371	0
B17	B183	CD3+CD16+CD56+NK	PTA	0.140396682	0
B17	B19	CD3+CD16+CD56+NK	CD3+CD4+/CD3+CD8+	-0.160473332	0
B17	B20	CD3+CD16+CD56+NK	CD3+CD4+CD8+	0.267605298	0
B17	B21	CD3+CD16+CD56+NK	CD3+CD4-CD8-	0.470964736	0
B17	B22	CD3+CD16+CD56+NK	CD3+CD8+(T)	0.163610009	0
B17	B23	CD3+CD16+CD56+NK	CD3-CD16+CD56+(NK)	-0.096667457	0
B17	B99	CD3+CD16+CD56+NK	anti-PM-Scl	-0.065419678	1.18E-12
B17	B95	CD3+CD16+CD56+NK	anti-cenpb	-0.063250528	6.36E-12
B17	B107	CD3+CD16+CD56+NK	AnuA	0.058628164	1.91E-10
B17	B101	CD3+CD16+CD56+NK	anti-Sc70	0.058304011	2.40E-10
B17	B104	CD3+CD16+CD56+NK	anti-SSB	0.058304007	2.40E-10
B17	B98	CD3+CD16+CD56+NK	anti-Jo-1	0.058304011	2.40E-10
B17	B115	CD3+CD16+CD56+NK	TSH TT3 TT4(TT3)	-0.046853523	3.62E-07
B17	B155	CD3+CD16+CD56+NK	IgA	-0.045452664	8.00E-07
B170	B172	U-GLU	KB	0.210319242	0
B170	B174	U-GLU	Urea/Cr	0.116547319	0
B170	B189	U-GLU	eGFB	0.13451806	0
B170	B192	U-GLU	UA	-0.138146692	0
B170	B204	U-GLU	Drinking	0.08905184	0
B170	B206	U-GLU	Waist	0.083020258	0
B170	B209	U-GLU	Smoking	0.11807666	0
B170	B216	U-GLU	BMI	0.094470728	0
B170	B221	U-GLU	Hei	0.114378847	0
B170	B222	U-GLU	NS	0.122354753	0
B170	B194	U-GLU	Cl	-0.071757384	6.22E-15
B170	B218	U-GLU	DBP	0.066176283	6.48E-13
B170	B195	U-GLU	Na	-0.064524204	2.38E-12
B170	B191	U-GLU	Urea	0.063293842	6.15E-12
B170	B176	U-GLU	Ucr	0.061080558	3.24E-11
B170	B219	U-GLU	OT	-0.06104322	3.33E-11

B170	B179	U-GLU	α1-MG	0.060395714	5.36E-11
B170	B200	U-GLU	OP	0.060131358	6.49E-11
B170	B201	U-GLU	e-habits	-0.05239895	1.27E-08
B170	B190	U-GLU	Cre	-0.049058728	9.96E-08
B172	B189	KB	eGFB	0.093614076	0
B172	B176	KB	Ucr	0.074149032	8.88E-16
B172	B174	KB	Urea/Cr	0.067991362	1.50E-13
B172	B221	KB	Hei	0.04621509	5.21E-07
B173	B216	NTT	BMI	-0.076610987	0
B173	B221	NTT	Hei	-0.119898592	0
B173	B222	NTT	NS	-0.085497027	0
B173	B174	NTT	Urea/Cr	0.07110658	1.11E-14
B173	B209	NTT	Smoking	-0.045541253	7.61E-07
B174	B189	Urea/Cr	eGFB	0.248752006	0
B174	B190	Urea/Cr	Cre	-0.385194229	0
B174	B191	Urea/Cr	Urea	0.55512888	0
B174	B192	Urea/Cr	UA	-0.22358618	0
B174	B200	Urea/Cr	OP	0.101306641	0
B174	B204	Urea/Cr	Drinking	-0.098143515	0
B174	B206	Urea/Cr	Waist	-0.112561934	0
B174	B209	Urea/Cr	Smoking	-0.122874024	0
B174	B210	Urea/Cr	Hip	-0.107627107	0
B174	B216	Urea/Cr	BMI	-0.191321997	0
B174	B221	Urea/Cr	Hei	-0.271923902	0
B174	B222	Urea/Cr	NS	-0.247527922	0
B174	B176	Urea/Cr	Ucr	-0.069218551	5.42E-14
B174	B218	Urea/Cr	DBP	-0.057036721	5.82E-10
B175	B177	ALB-U	ACR	0.908810884	0
B175	B178	ALB-U	Ig-U	0.103266206	0
B175	B180	ALB-U	Tf	0.078293447	0
B175	B191	ALB-U	Urea	0.09415116	0
B175	B190	ALB-U	Cre	0.063517338	5.18E-12
B175	B176	ALB-U	Ucr	0.057979175	3.02E-10
B175	B189	ALB-U	eGFB	-0.053580747	5.91E-09
B176	B179	Ucr	α1-MG	0.171220983	0
B176	B216	Ucr	BMI	0.120432541	0
B176	B221	Ucr	Hei	0.143257973	0
B176	B222	Ucr	NS	0.131589345	0
B176	B206	Ucr	Waist	0.071764604	6.22E-15
B176	B209	Ucr	Smoking	0.064310872	2.81E-12
B176	B183	Ucr	PTA	0.053252037	7.32E-09
B176	B220	Ucr	SBP	-0.052606567	1.11E-08
B176	B210	Ucr	Hip	0.052232885	1.41E-08
B176	B190	Ucr	Cre	0.050311354	4.66E-08

B177	B178	ACR	Ig-U	0.234648217	0
B177	B180	ACR	Tf	0.192755825	0
B177	B191	ACR	Urea	0.111725163	0
B177	B190	ACR	Cre	0.072210587	4.22E-15
B177	B179	ACR	α1-MG	0.071085783	1.11E-14
B177	B189	ACR	eGFB	-0.061182539	3.01E-11
B178	B179	Ig-U	α1-MG	0.555096349	0
B178	B180	Ig-U	Tf	0.903851033	0
B179	B180	α1-MG	Tf	0.414259968	0
B179	B194	α1-MG	Cl	-0.048845335	1.13E-07
B179	B195	α1-MG	Na	-0.048602002	1.31E-07
B18	B101	CD3+CD4+helper(inducer)T	anti-Sc70	-0.197470932	0
B18	B104	CD3+CD4+helper(inducer)T	anti-SSB	-0.197470885	0
B18	B107	CD3+CD4+helper(inducer)T	AnuA	-0.201640354	0
B18	B121	CD3+CD4+helper(inducer)T	C3	0.147428078	0
B18	B122	CD3+CD4+helper(inducer)T	C4	0.122256862	0
B18	B136	CD3+CD4+helper(inducer)T	5'NT	0.151891212	0
B18	B151	CD3+CD4+helper(inducer)T	Anti-CCP	0.102288522	0
B18	B152	CD3+CD4+helper(inducer)T	ACA-Ig(G-A-M)	-0.127254575	0
B18	B183	CD3+CD4+helper(inducer)T	PTA	-0.088345938	0
B18	B186	CD3+CD4+helper(inducer)T	PFC	0.111643493	0
B18	B19	CD3+CD4+helper(inducer)T	CD3+CD4+/CD3+CD8+	0.694187689	0
B18	B20	CD3+CD4+helper(inducer)T	CD3+CD4+CD8+	0.0848274	0
B18	B22	CD3+CD4+helper(inducer)T	CD3+CD8+(T)	-0.386989251	0
B18	B23	CD3+CD4+helper(inducer)T	CD3-CD16+CD56+(NK)	-0.425732317	0
B18	B98	CD3+CD4+helper(inducer)T	anti-Jo-1	-0.197470859	0
B18	B157	CD3+CD4+helper(inducer)T	IgG	-0.071749602	6.22E-15
B18	B181	CD3+CD4+helper(inducer)T	APTT	-0.071780357	6.22E-15
B18	B184	CD3+CD4+helper(inducer)T	INR	-0.06602756	7.30E-13
B18	B139	CD3+CD4+helper(inducer)T	ADA	0.06484659	1.85E-12
B18	B90	CD3+CD4+helper(inducer)T	CYFRA 21-1	0.063332835	5.97E-12
B18	B193	CD3+CD4+helper(inducer)T	K	-0.063320408	6.03E-12
B18	B195	CD3+CD4+helper(inducer)T	Na	-0.062598832	1.04E-11
B18	B150	CD3+CD4+helper(inducer)T	ALP	0.059568421	9.76E-11
B18	B97	CD3+CD4+helper(inducer)T	dsDNA	-0.058544256	2.03E-10
B18	B138	CD3+CD4+helper(inducer)T	PAB	0.055197958	2.03E-09
B18	B79	CD3+CD4+helper(inducer)T	hb V -dna	-0.05506369	2.22E-09
B18	B105	CD3+CD4+helper(inducer)T	anti-U1RNP	-0.052166115	1.47E-08
B18	B212	CD3+CD4+helper(inducer)T	IOP-L	0.051449045	2.30E-08
B18	B198	CD3+CD4+helper(inducer)T	P	0.051251653	2.61E-08
B18	B99	CD3+CD4+helper(inducer)T	anti-PM-Scl	0.049236401	8.95E-08
B18	B211	CD3+CD4+helper(inducer)T	IOP-R	0.047825521	2.06E-07
B18	B81	CD3+CD4+helper(inducer)T	TSGF	-0.046445918	4.57E-07
B181	B184	APTT	INR	0.436917944	0

B181	B185	APTT	PT	0.428607543	0
B181	B182	APTT	TT	0.074023973	8.88E-16
B181	B200	APTT	OP	-0.07346535	1.33E-15
B181	B194	APTT	Cl	0.06875106	7.99E-14
B182	B183	TT	PTA	-0.276339651	0
B182	B185	TT	PT	0.100800231	0
B182	B194	TT	Cl	-0.097638303	0
B182	B197	TT	AG	0.101209687	0
B182	B186	TT	PFC	-0.073903993	8.88E-16
B182	B196	TT	TCO2	-0.047225884	2.92E-07
B183	B186	PTA	PFC	-0.085042357	0
B183	B194	PTA	Cl	0.092475005	0
B183	B197	PTA	AG	-0.081439247	0
B183	B184	PTA	INR	0.055354143	1.83E-09
B183	B200	PTA	OP	-0.054160204	4.05E-09
B183	B193	PTA	K	-0.049972583	5.74E-08
B184	B185	INR	PT	0.973513942	0
B184	B186	INR	PFC	-0.09220699	0
B184	B200	INR	OP	-0.070742593	1.51E-14
B184	B194	INR	Cl	0.061223508	2.92E-11
B186	B194	PFC	Cl	-0.154511136	0
B186	B195	PFC	Na	-0.092159925	0
B188	B192	Ca	UA	0.114766602	0
B188	B218	Ca	DBP	0.092044258	0
B188	B194	Ca	Cl	-0.070090217	2.62E-14
B188	B193	Ca	K	0.064848587	1.85E-12
B188	B198	Ca	P	0.050726133	3.61E-08
B188	B197	Ca	AG	0.046422952	4.63E-07
B189	B190	eGFB	Cre	-0.600987795	0
B189	B191	eGFB	Urea	-0.359454859	0
B189	B192	eGFB	UA	-0.179633416	0
B189	B215	eGFB	BMI	0.081141672	0
B189	B216	eGFB	BMI	0.111549753	0
B189	B218	eGFB	DBP	0.113710447	0
B189	B220	eGFB	SBP	-0.204870922	0
B189	B221	eGFB	Hei	0.081997218	0
B189	B224	eGFB	pmh	0.086356055	0
B189	B229	eGFB	slit-lamp	-0.107611735	0
B189	B211	eGFB	IOP-R	0.071277508	9.55E-15
B189	B219	eGFB	OT	-0.069751418	3.46E-14
B189	B201	eGFB	e-habits	-0.064518827	2.39E-12
B189	B212	eGFB	IOP-L	0.060898353	3.71E-11
B189	B210	eGFB	Hip	0.060789971	4.01E-11
B189	B204	eGFB	Drinking	0.055105482	2.16E-09

B189	B194	eGFB	Cl	-0.054325464	3.63E-09
B189	B209	eGFB	Smoking	0.048259435	1.60E-07
B189	B203	eGFB	DH	0.047583291	2.38E-07
B189	B228	eGFB	OS	-0.046106431	5.54E-07
B19	B101	CD3+CD4+/CD3+CD8+	anti-Sc70	-0.118157459	0
B19	B104	CD3+CD4+/CD3+CD8+	anti-SSB	-0.11815744	0
B19	B105	CD3+CD4+/CD3+CD8+	anti-U1RNP	-0.087571282	0
B19	B107	CD3+CD4+/CD3+CD8+	AnuA	-0.116900042	0
B19	B136	CD3+CD4+/CD3+CD8+	5'NT	0.098884887	0
B19	B151	CD3+CD4+/CD3+CD8+	Anti-CCP	0.110896153	0
B19	B155	CD3+CD4+/CD3+CD8+	IgA	0.087237159	0
B19	B183	CD3+CD4+/CD3+CD8+	PTA	-0.08769574	0
B19	B20	CD3+CD4+/CD3+CD8+	CD3+CD4+CD8+	-0.147350557	0
B19	B22	CD3+CD4+/CD3+CD8+	CD3+CD8+(T)	-0.814433793	0
B19	B23	CD3+CD4+/CD3+CD8+	CD3-CD16+CD56+(NK)	0.107331521	0
B19	B98	CD3+CD4+/CD3+CD8+	anti-Jo-1	-0.11815743	0
B19	B81	CD3+CD4+/CD3+CD8+	TSGF	-0.072414697	3.55E-15
B19	B186	CD3+CD4+/CD3+CD8+	PFC	0.069122463	5.86E-14
B19	B125	CD3+CD4+/CD3+CD8+	Cor	-0.066606152	4.60E-13
B19	B100	CD3+CD4+/CD3+CD8+	anti-Ro52	-0.064984891	1.66E-12
B19	B139	CD3+CD4+/CD3+CD8+	ADA	0.062921508	8.17E-12
B19	B95	CD3+CD4+/CD3+CD8+	anti-cenpb	0.058890016	1.59E-10
B19	B138	CD3+CD4+/CD3+CD8+	PAB	0.056418903	8.90E-10
B19	B97	CD3+CD4+/CD3+CD8+	dsDNA	-0.05525604	1.96E-09
B19	B90	CD3+CD4+/CD3+CD8+	CYFRA 21-1	0.053858349	4.93E-09
B19	B195	CD3+CD4+/CD3+CD8+	Na	-0.050141173	5.18E-08
B19	B108	CD3+CD4+/CD3+CD8+	Anti-Cyclin	-0.046654459	4.06E-07
B19	B31	CD3+CD4+/CD3+CD8+	FSH	-0.045374983	8.35E-07
B190	B191	Cre	Urea	0.423101839	0
B190	B192	Cre	UA	0.291791173	0
B190	B206	Cre	Waist	0.115795574	0
B190	B209	Cre	Smoking	0.086883773	0
B190	B216	Cre	BMI	0.142250794	0
B190	B221	Cre	Hei	0.214858621	0
B190	B222	Cre	NS	0.222205557	0
B190	B210	Cre	Hip	0.075329681	2.22E-16
B190	B204	Cre	Drinking	0.056802964	6.84E-10
B191	B192	Urea	UA	0.114912694	0
B191	B200	Urea	OP	0.084707916	0
B191	B218	Urea	DBP	-0.069561218	4.09E-14
B192	B204	UA	Drinking	0.09021393	0
B192	B206	UA	Waist	0.231345842	0
B192	B210	UA	Hip	0.168825735	0
B192	B215	UA	BMI	0.191150847	0

B192	B216	UA	BMI	0.272067628	0
B192	B218	UA	DBP	0.087018164	0
B192	B221	UA	Hei	0.216265314	0
B192	B222	UA	NS	0.259196604	0
B193	B198	K	P	0.09989593	0
B193	B200	K	OP	0.075349943	2.22E-16
B193	B196	K	TCO2	0.067878171	1.64E-13
B193	B197	K	AG	-0.047136766	3.08E-07
B194	B195	Cl	Na	0.405944577	0
B194	B196	Cl	TCO2	-0.140723475	0
B194	B197	Cl	AG	-0.350415461	0
B194	B198	Cl	P	-0.143142857	0
B194	B199	Cl	Mg	0.135354924	0
B194	B212	Cl	IOP-L	-0.069322102	4.97E-14
B195	B196	Na	TCO2	0.090227828	0
B195	B197	Na	AG	0.27330461	0
B195	B199	Na	Mg	0.153128498	0
B195	B200	Na	OP	0.503896135	0
B196	B197	TCO2	AG	-0.676805732	0
B196	B200	TCO2	OP	0.07754742	0
B197	B198	AG	P	0.081443521	0
B197	B200	AG	OP	0.272443199	0
B198	B199	P	Mg	-0.076367456	0
B199	B200	Mg	OP	-0.138126876	0
B199	B216	Mg	BMI	-0.046565876	4.27E-07
B2	B126	Age	Alb	-0.280277706	0
B2	B127	Age	A-G ratio	-0.199873842	0
B2	B128	Age	ALT	-0.145854177	0
B2	B130	Age	GLB	0.110150619	0
B2	B132	Age	Dbil	0.085484865	0
B2	B134	Age	CHE	-0.079644869	0
B2	B140	Age	r-GT	-0.148916375	0
B2	B141	Age	AST/ALT	0.229420774	0
B2	B146	Age	CysC	0.113967838	0
B2	B153	Age	Glu	-0.115690049	0
B2	B163	Age	SG	-0.180787447	0
B2	B170	Age	U-GLU	-0.12598849	0
B2	B172	Age	KB	-0.100113	0
B2	B176	Age	Ucr	-0.086718773	0
B2	B188	Age	Ca	-0.109955382	0
B2	B189	Age	eGFB	-0.612158214	0
B2	B190	Age	Cre	0.178043621	0
B2	B191	Age	Urea	0.175274314	0
B2	B201	Age	e-habits	0.08462389	0

B2	B203	Age	DH	-0.082187271	0
B2	B204	Age	Drinking	-0.12243786	0
B2	B209	Age	Smoking	-0.112135824	0
B2	B210	Age	Hip	-0.120107044	0
B2	B211	Age	IOP-R	-0.104765576	0
B2	B212	Age	IOP-L	-0.09160839	0
B2	B215	Age	BMI	-0.1659732	0
B2	B216	Age	BMI	-0.285598708	0
B2	B218	Age	DBP	-0.171378565	0
B2	B219	Age	OT	0.122648313	0
B2	B220	Age	SBP	0.304827433	0
B2	B221	Age	Hei	-0.261514504	0
B2	B222	Age	NS	-0.179151668	0
B2	B228	Age	OS	0.081009733	0
B2	B229	Age	slit-lamp	0.160003398	0
B2	B24	Age	αHBDH	0.080775895	0
B2	B27	Age	LDH	0.089671163	0
B2	B41	Age	MON％	0.192804193	0
B2	B42	Age	MON	0.082570733	0
B2	B43	Age	MCV	0.169653754	0
B2	B45	Age	MCHC	-0.186510455	0
B2	B46	Age	RBC Count	-0.314116779	0
B2	B47	Age	RDW	0.279217127	0
B2	B51	Age	Hb	-0.29423118	0
B2	B52	Age	PLT/BPC	-0.111379054	0
B2	B71	Age	TG	-0.204349791	0
B2	B72	Age	HDL-C	0.170604297	0
B2	B74	Age	ApoA1	0.088782319	0
B2	B107	Age	AnuA	0.074304398	6.66E-16
B2	B101	Age	anti-Sc70	0.073704559	1.11E-15
B2	B104	Age	anti-SSB	0.073704548	1.11E-15
B2	B98	Age	anti-Jo-1	0.073704542	1.11E-15
B2	B77	Age	TC	-0.071973686	5.33E-15
B2	B62	Age	WBHSRVI	-0.070959006	1.24E-14
B2	B227	Age	OD	0.070306356	2.18E-14
B2	B91	Age	TP	-0.070206482	2.35E-14
B2	B40	Age	WBC	-0.070005689	2.80E-14
B2	B67	Age	WBRSR(mPas_50)	-0.069652443	3.77E-14
B2	B173	Age	NTT	0.068655774	8.66E-14
B2	B58	Age	HCT	-0.067993272	1.49E-13
B2	B66	Age	WBRSR(mPas_100)	-0.067371825	2.48E-13
B2	B138	Age	PAB	-0.067344004	2.54E-13
B2	B161	Age	LEU	0.066058255	7.12E-13
B2	B63	Age	WBRSR(mPas_200)	-0.064216068	3.03E-12

B2	B50	Age	eo%	0.06396994	3.66E-12
B2	B60	Age	WBRVI	-0.063914522	3.82E-12
B2	B65	Age	WBRSR(mPas_1)	-0.063320932	6.03E-12
B2	B44	Age	MCH	0.060260138	5.91E-11
B2	B194	Age	Cl	0.056525333	8.28E-10
B2	B75	Age	ApoA1/B	0.054339355	3.60E-09
B2	B195	Age	Na	0.053532077	6.10E-09
B2	B54	Age	ESR	0.053138867	7.87E-09
B2	B120	Age	HCV-cAg	0.053026929	8.46E-09
B2	B192	Age	UA	-0.05145116	2.30E-08
B2	B61	Age	WBHSRV	-0.05139253	2.39E-08
B2	B149	Age	TGAb	0.051089577	2.88E-08
B2	B3	Age	Hcy	0.050868853	3.31E-08
B2	B56	Age	IR	-0.050529834	4.08E-08
B2	B157	Age	IgG	0.047481921	2.52E-07
B2	B12	Age	PG II	0.046050872	5.72E-07
B2	B73	Age	ApoA	0.045961625	6.01E-07
B20	B101	CD3+CD4+CD8+	anti-Sc70	-0.076216462	0
B20	B104	CD3+CD4+CD8+	anti-SSB	-0.076216446	0
B20	B107	CD3+CD4+CD8+	AnuA	-0.076651767	0
B20	B151	CD3+CD4+CD8+	Anti-CCP	-0.090690405	0
B20	B152	CD3+CD4+CD8+	ACA-Ig(G-A-M)	-0.619483501	0
B20	B157	CD3+CD4+CD8+	IgG	-0.10821899	0
B20	B183	CD3+CD4+CD8+	PTA	0.120973521	0
B20	B21	CD3+CD4+CD8+	CD3+CD4-CD8-	-0.126969142	0
B20	B22	CD3+CD4+CD8+	CD3+CD8+(T)	0.271773292	0
B20	B23	CD3+CD4+CD8+	CD3-CD16+CD56+(NK)	-0.138265877	0
B20	B92	CD3+CD4+CD8+	ALDH2-Glu504Glu	-0.106746622	0
B20	B93	CD3+CD4+CD8+	ALDH2-Glu504Lys	0.110915296	0
B20	B98	CD3+CD4+CD8+	anti-Jo-1	-0.076216438	0
B20	B197	CD3+CD4+CD8+	AG	-0.074303061	6.66E-16
B20	B158	CD3+CD4+CD8+	IgM	-0.055405176	1.77E-09
B20	B99	CD3+CD4+CD8+	anti-PM-Scl	0.055207931	2.02E-09
B20	B155	CD3+CD4+CD8+	IgA	-0.050827877	3.39E-08
B200	B211	OP	IOP-R	0.071957658	5.33E-15
B200	B212	OP	IOP-L	0.061805779	1.89E-11
B201	B204	e-habits	Drinking	0.084321341	0
B201	B224	e-habits	pmh	0.103459958	0
B201	B205	e-habits	KDA	0.045230456	9.05E-07
B202	B223	e-times	FH	-0.32389084	0
B203	B209	DH	Smoking	0.065362113	1.24E-12
B203	B204	DH	Drinking	0.054800607	2.65E-09
B204	B206	Drinking	Waist	0.128894686	0
B204	B209	Drinking	Smoking	0.308817863	0

B204	B210	Drinking	Hip	0.085604909	0
B204	B216	Drinking	BMI	0.179387249	0
B204	B218	Drinking	DBP	0.099073617	0
B204	B221	Drinking	Hei	0.20834939	0
B204	B222	Drinking	NS	0.220359991	0
B204	B215	Drinking	BMI	0.07371782	1.11E-15
B204	B219	Drinking	OT	-0.046096375	5.57E-07
B205	B222	KDA	NS	-0.045964827	6.00E-07
B206	B207	Waist	WHR	0.100657118	0
B206	B209	Waist	Smoking	0.105557222	0
B206	B210	Waist	Hip	0.758147537	0
B206	B215	Waist	BMI	0.802340738	0
B206	B216	Waist	BMI	0.794663956	0
B206	B218	Waist	DBP	0.181866181	0
B206	B220	Waist	SBP	0.125140313	0
B206	B221	Waist	Hei	0.334200237	0
B206	B222	Waist	NS	0.668027611	0
B207	B215	WHR	BMI	0.057116629	5.51E-10
B207	B222	WHR	NS	0.051021284	3.01E-08
B207	B216	WHR	BMI	0.049948128	5.82E-08
B209	B216	Smoking	BMI	0.157130246	0
B209	B220	Smoking	SBP	-0.099276573	0
B209	B221	Smoking	Hei	0.257746335	0
B209	B222	Smoking	NS	0.218265917	0
B209	B219	Smoking	OT	-0.065935942	7.85E-13
B209	B210	Smoking	Hip	0.045488774	7.84E-07
B21	B124	CD3+CD4-CD8-	ACTH	0.243600281	0
B21	B152	CD3+CD4-CD8-	ACA-Ig(G-A-M)	0.080320743	0
B21	B195	CD3+CD4-CD8-	Na	0.081637995	0
B21	B200	CD3+CD4-CD8-	OP	0.098837166	0
B21	B23	CD3+CD4-CD8-	CD3-CD16+CD56+(NK)	-0.216209984	0
B21	B92	CD3+CD4-CD8-	ALDH2-Glu504Glu	-0.094508941	0
B21	B93	CD3+CD4-CD8-	ALDH2-Glu504Lys	0.100000257	0
B21	B138	CD3+CD4-CD8-	PAB	0.07289794	2.22E-15
B21	B81	CD3+CD4-CD8-	TSGF	0.056979074	6.06E-10
B21	B181	CD3+CD4-CD8-	APTT	-0.054587795	3.05E-09
B21	B183	CD3+CD4-CD8-	PTA	0.050949292	3.15E-08
B210	B215	Hip	BMI	0.733358186	0
B210	B216	Hip	BMI	0.766699379	0
B210	B218	Hip	DBP	0.154114398	0
B210	B221	Hip	Hei	0.37331522	0
B210	B222	Hip	NS	0.585630193	0
B210	B220	Hip	SBP	0.059045035	1.42E-10
B211	B212	IOP-R	IOP-L	0.778622209	0

B211	B218	IOP-R	DBP	0.090513666	0
B211	B220	IOP-R	SBP	0.046055986	5.70E-07
B211	B213	IOP-R	CVA-R	0.045450561	8.01E-07
B212	B218	IOP-L	DBP	0.079192678	0
B215	B216	BMI	BMI	0.789955773	0
B215	B218	BMI	DBP	0.209655418	0
B215	B220	BMI	SBP	0.116393268	0
B215	B221	BMI	Hei	0.090992087	0
B215	B222	BMI	NS	0.572796595	0
B216	B218	BMI	DBP	0.222228047	0
B216	B221	BMI	Hei	0.676936	0
B216	B222	BMI	NS	0.751353162	0
B216	B219	BMI	OT	-0.068432705	1.04E-13
B218	B220	DBP	SBP	0.565182993	0
B218	B221	DBP	Hei	0.113168952	0
B218	B222	DBP	NS	0.198624464	0
B218	B219	DBP	OT	-0.051912388	1.72E-08
B219	B221	OT	Hei	-0.08668641	0
B219	B222	OT	NS	-0.073854247	8.88E-16
B22	B105	CD3+CD8+(T)	anti-U1RNP	0.119124995	0
B22	B121	CD3+CD8+(T)	C3	0.078085501	0
B22	B122	CD3+CD8+(T)	C4	0.084715223	0
B22	B155	CD3+CD8+(T)	IgA	-0.10307405	0
B22	B157	CD3+CD8+(T)	IgG	-0.077784129	0
B22	B23	CD3+CD8+(T)	CD3-CD16+CD56+(NK)	-0.403679279	0
B22	B29	CD3+CD8+(T)	E2	-0.076020754	0
B22	B31	CD3+CD8+(T)	FSH	0.096638542	0
B22	B95	CD3+CD8+(T)	anti-cenpb	-0.097588698	0
B22	B97	CD3+CD8+(T)	dsDNA	0.082936797	0
B22	B108	CD3+CD8+(T)	Anti-Cyclin	0.074398434	6.66E-16
B22	B152	CD3+CD8+(T)	ACA-Ig(G-A-M)	-0.071091482	1.11E-14
B22	B33	CD3+CD8+(T)	Prog	-0.062278175	1.33E-11
B22	B100	CD3+CD8+(T)	anti-Ro52	0.060180791	6.27E-11
B22	B138	CD3+CD8+(T)	PAB	-0.049556428	7.38E-08
B22	B81	CD3+CD8+(T)	TSGF	0.048231735	1.63E-07
B22	B136	CD3+CD8+(T)	5'NT	-0.046765216	3.81E-07
B22	B99	CD3+CD8+(T)	anti-PM-Scl	0.046625354	4.13E-07
B220	B221	SBP	Hei	-0.132365631	0
B221	B222	Hei	NS	0.540482297	0
B227	B228	OD	OS	0.129484288	0
B227	B229	OD	slit-lamp	0.057928754	3.13E-10
B23	B101	CD3-CD16+CD56+(NK)	anti-Sc70	0.15682674	0
B23	B104	CD3-CD16+CD56+(NK)	anti-SSB	0.15682672	0
B23	B107	CD3-CD16+CD56+(NK)	AnuA	0.15770509	0

B23	B121	CD3-CD16+CD56+(NK)	C3	-0.280423681	0
B23	B122	CD3-CD16+CD56+(NK)	C4	-0.229017703	0
B23	B152	CD3-CD16+CD56+(NK)	ACA-Ig(G-A-M)	0.093059967	0
B23	B193	CD3-CD16+CD56+(NK)	K	0.116517702	0
B23	B98	CD3-CD16+CD56+(NK)	anti-Jo-1	0.156826712	0
B23	B99	CD3-CD16+CD56+(NK)	anti-PM-Scl	-0.07978312	0
B23	B157	CD3-CD16+CD56+(NK)	IgG	0.074199427	8.88E-16
B23	B96	CD3-CD16+CD56+(NK)	dsDNA (IIF)	0.068083546	1.39E-13
B23	B74	CD3-CD16+CD56+(NK)	ApoA1	0.067697953	1.90E-13
B23	B79	CD3-CD16+CD56+(NK)	hb V -dna	0.066744169	4.11E-13
B23	B124	CD3-CD16+CD56+(NK)	ACTH	-0.06468781	2.10E-12
B23	B200	CD3-CD16+CD56+(NK)	OP	-0.064655652	2.15E-12
B23	B109	CD3-CD16+CD56+(NK)	AMA-M2	-0.06437644	2.67E-12
B23	B212	CD3-CD16+CD56+(NK)	IOP-L	-0.057255267	5.01E-10
B23	B211	CD3-CD16+CD56+(NK)	IOP-R	-0.050981744	3.08E-08
B23	B181	CD3-CD16+CD56+(NK)	APTT	0.048434541	1.44E-07
B23	B120	CD3-CD16+CD56+(NK)	HCV-cAg	0.048244172	1.62E-07
B23	B184	CD3-CD16+CD56+(NK)	INR	0.048133873	1.72E-07
B23	B72	CD3-CD16+CD56+(NK)	HDL-C	0.046666751	4.03E-07
B23	B199	CD3-CD16+CD56+(NK)	Mg	0.04628988	5.00E-07
B24	B127	αHBDH	A-G ratio	-0.076129079	0
B24	B130	αHBDH	GLB	0.076669917	0
B24	B131	αHBDH	AST	0.092969362	0
B24	B175	αHBDH	ALB-U	0.07595509	0
B24	B25	αHBDH	CK	0.210571384	0
B24	B26	αHBDH	CKMB	0.147753188	0
B24	B27	αHBDH	LDH	0.728811674	0
B24	B69	αHBDH	PV	0.08128962	0
B24	B74	αHBDH	ApoA1	0.134522921	0
B24	B75	αHBDH	ApoA1/B	0.090421668	0
B24	B47	αHBDH	RDW	0.070799167	1.42E-14
B24	B177	αHBDH	ACR	0.068391164	1.08E-13
B24	B221	αHBDH	Hei	-0.065254851	1.35E-12
B24	B72	αHBDH	HDL-C	0.064932343	1.73E-12
B24	B220	αHBDH	SBP	0.063032389	7.51E-12
B24	B141	αHBDH	AST/ALT	0.06057441	4.70E-11
B24	B58	αHBDH	HCT	-0.058775737	1.72E-10
B24	B91	αHBDH	TP	0.056987147	6.02E-10
B24	B148	αHBDH	Anti_TPO	0.05349364	6.26E-09
B24	B176	αHBDH	Ucr	-0.051195714	2.70E-08
B24	B79	αHBDH	hb V -dna	0.050120695	5.24E-08
B24	B46	αHBDH	RBC Count	-0.049655117	6.95E-08
B24	B216	αHBDH	BMI	-0.045276851	8.82E-07
B25	B123	CK	hs-CRP1	-0.079284703	0

B25	B26	CK	CKMB	0.212429696	0
B25	B27	CK	LDH	0.202650293	0
B25	B89	CK	CA 72-4	0.084298682	0
B25	B189	CK	eGFB	-0.068685058	8.44E-14
B25	B190	CK	Cre	0.065132473	1.48E-12
B25	B192	CK	UA	0.061866514	1.81E-11
B25	B114	CK	TSH TT3 TT4(TT4)	-0.060276063	5.85E-11
B25	B57	CK	RBCAI	0.059830928	8.07E-11
B25	B153	CK	Glu	-0.055983642	1.20E-09
B25	B131	CK	AST	0.052128438	1.50E-08
B25	B78	CK	HBcAb	-0.047006696	3.32E-07
B26	B27	CKMB	LDH	0.120903843	0
B26	B145	CKMB	BGP	-0.066410145	5.38E-13
B26	B183	CKMB	PTA	0.056880813	6.48E-10
B26	B34	CKMB	TK	0.049610911	7.14E-08
B26	B115	CKMB	TSH TT3 TT4(TT3)	0.047255881	2.87E-07
B27	B118	LDH	CEA1	0.092661653	0
B27	B127	LDH	A-G ratio	-0.098499235	0
B27	B130	LDH	GLB	0.09907058	0
B27	B131	LDH	AST	0.146680648	0
B27	B138	LDH	PAB	-0.076617135	0
B27	B139	LDH	ADA	0.124426123	0
B27	B150	LDH	ALP	0.087000161	0
B27	B221	LDH	Hei	-0.075959801	0
B27	B69	LDH	PV	0.078289937	0
B27	B74	LDH	ApoA1	0.105386965	0
B27	B47	LDH	RDW	0.072314603	4.00E-15
B27	B91	LDH	TP	0.071572186	7.55E-15
B27	B220	LDH	SBP	0.071471671	7.99E-15
B27	B177	LDH	ACR	0.066245888	6.13E-13
B27	B72	LDH	HDL-C	0.065725413	9.28E-13
B27	B185	LDH	PT	0.065616449	1.01E-12
B27	B75	LDH	ApoA1/B	0.065397427	1.20E-12
B27	B184	LDH	INR	0.06427292	2.90E-12
B27	B186	LDH	PFC	0.063551745	5.05E-12
B27	B128	LDH	ALT	0.060366427	5.47E-11
B27	B176	LDH	Ucr	-0.060271734	5.86E-11
B27	B175	LDH	ALB-U	0.057887602	3.22E-10
B27	B189	LDH	eGFB	-0.056129562	1.08E-09
B27	B141	LDH	AST/ALT	0.055527494	1.63E-09
B27	B140	LDH	r-GT	0.053382282	6.73E-09
B27	B182	LDH	TT	0.047298375	2.80E-07
B27	B46	LDH	RBC Count	-0.045788862	6.63E-07
B27	B41	LDH	MON％	0.045485835	7.85E-07

B28	B29	PRL	E2	-0.149561775	0
B28	B30	PRL	LH	-0.274985206	0
B28	B31	PRL	FSH	-0.160989545	0
B28	B33	PRL	Prog	-0.187837538	0
B28	B32	PRL	Testo	-0.072268188	4.00E-15
B28	B34	PRL	TK	-0.049478224	7.74E-08
B28	B74	PRL	ApoA1	-0.047815822	2.08E-07
B29	B125	E2	Cor	0.079989177	0
B29	B30	E2	LH	-0.30263891	0
B29	B31	E2	FSH	-0.538598758	0
B29	B32	E2	Testo	-0.316313048	0
B29	B33	E2	Prog	0.484462645	0
B29	B173	E2	NTT	-0.059514368	1.01E-10
B29	B205	E2	KDA	-0.056261805	9.91E-10
B29	B75	E2	ApoA1/B	0.046001842	5.88E-07
B3	B174	Hcy	Urea/Cr	-0.091592138	0
B3	B189	Hcy	eGFB	-0.13845564	0
B3	B190	Hcy	Cre	0.13098271	0
B3	B192	Hcy	UA	0.105928358	0
B3	B222	Hcy	NS	0.077971958	0
B3	B176	Hcy	Ucr	0.061766407	1.95E-11
B3	B47	Hcy	RDW	0.061394701	2.57E-11
B3	B221	Hcy	Hei	0.058812131	1.68E-10
B3	B216	Hcy	BMI	0.057876111	3.25E-10
B3	B206	Hcy	Waist	0.057519036	4.17E-10
B3	B191	Hcy	Urea	0.054173917	4.01E-09
B3	B25	Hcy	CK	0.052271406	1.37E-08
B3	B209	Hcy	Smoking	0.051347345	2.46E-08
B3	B111	Hcy	TSH	0.049773674	6.47E-08
B3	B175	Hcy	ALB-U	0.047437637	2.59E-07
B3	B42	Hcy	MON	0.046492692	4.45E-07
B3	B44	Hcy	MCH	0.046244644	5.13E-07
B3	B146	Hcy	CysC	0.045570546	7.49E-07
B30	B31	LH	FSH	0.79088348	0
B30	B32	LH	Testo	-0.280747392	0
B30	B33	LH	Prog	-0.066973591	3.42E-13
B31	B32	FSH	Testo	-0.340823656	0
B31	B33	FSH	Prog	-0.164004706	0
B31	B125	FSH	Cor	-0.073056	2.00E-15
B31	B76	FSH	ApoB	0.056184083	1.04E-09
B31	B75	FSH	ApoA1/B	-0.054132056	4.12E-09
B31	B173	FSH	NTT	0.050347441	4.56E-08
B31	B77	FSH	TC	0.045898194	6.23E-07
B32	B121	Testo	C3	-0.139848725	0

B32	B33	Testo	Prog	-0.263255959	0
B33	B205	Prog	KDA	-0.067687919	1.92E-13
B34	B173	TK	NTT	0.069329408	4.95E-14
B34	B81	TK	TSGF	-0.066630976	4.51E-13
B34	B107	TK	AnuA	0.063873882	3.94E-12
B34	B101	TK	anti-Sc70	0.063484371	5.32E-12
B34	B104	TK	anti-SSB	0.06348436	5.32E-12
B34	B98	TK	anti-Jo-1	0.063484355	5.32E-12
B34	B151	TK	Anti-CCP	0.061352403	2.65E-11
B34	B200	TK	OP	-0.060107859	6.61E-11
B34	B74	TK	ApoA1	0.059219447	1.25E-10
B34	B139	TK	ADA	-0.057178566	5.28E-10
B34	B134	TK	CHE	-0.052784828	9.89E-09
B35	B159	β2-MG	β2-MG	0.978773458	0
B35	B186	β2-MG	PFC	0.052893004	9.22E-09
B35	B181	β2-MG	APTT	-0.046001934	5.88E-07
B36	B184	V-Lym%	INR	-0.15327275	0
B36	B185	V-Lym%	PT	-0.15135838	0
B36	B37	V-Lym%	BAND	0.100138926	0
B36	B181	V-Lym%	APTT	-0.075707442	2.22E-16
B36	B38	V-Lym%	BAND%	0.05933494	1.15E-10
B36	B66	V-Lym%	WBRSR(mPas_100)	-0.050862045	3.32E-08
B36	B67	V-Lym%	WBRSR(mPas_50)	-0.050370818	4.50E-08
B36	B63	V-Lym%	WBRSR(mPas_200)	-0.049772319	6.48E-08
B36	B62	V-Lym%	WBHSRVI	-0.049243912	8.91E-08
B36	B58	V-Lym%	HCT	-0.047979356	1.89E-07
B36	B132	V-Lym%	Dbil	-0.047698413	2.22E-07
B36	B179	V-Lym%	α1-MG	-0.04678942	3.76E-07
B37	B38	BAND	BAND%	0.636249318	0
B37	B90	BAND	CYFRA 21-1	-0.060907317	3.68E-11
B38	B85	BAND%	SCC	0.234301572	0
B38	B199	BAND%	Mg	-0.064146063	3.19E-12
B38	B193	BAND%	K	-0.060427835	5.23E-11
B38	B139	BAND%	ADA	0.048312543	1.55E-07
B38	B137	BAND%	AFU	0.047866937	2.02E-07
B38	B138	BAND%	PAB	-0.045339952	8.51E-07
B38	B41	BAND%	MON %	0.045169874	9.36E-07
B39	B43	Other cells	MCV	-0.102353391	0
B39	B44	Other cells	MCH	-0.110183498	0
B39	B45	Other cells	MCHC	-0.064005031	3.56E-12
B39	B51	Other cells	Hb	-0.051214403	2.67E-08
B39	B46	Other cells	RBC Count	0.049799956	6.37E-08
B4	B121	WB-Ca	C3	0.10960052	0
B4	B122	WB-Ca	C4	-0.118566899	0

B4	B29	WB-Ca	E2	0.21663564	0
B4	B32	WB-Ca	Testo	-0.298579259	0
B4	B33	WB-Ca	Prog	0.169363392	0
B4	B5	WB-Ca	WB-Mg	0.394819998	0
B4	B6	WB-Ca	WB-Fe	0.264356179	0
B4	B7	WB-Ca	WB-Cop	0.671242091	0
B4	B8	WB-Ca	WB-Zinc	0.541498414	0
B4	B158	WB-Ca	IgM	0.071468044	7.99E-15
B40	B127	WBC	A-G ratio	-0.101486507	0
B40	B129	WBC	IBIL	-0.109268794	0
B40	B130	WBC	GLB	0.124703251	0
B40	B132	WBC	Dbil	-0.126962692	0
B40	B133	WBC	TBIL	-0.12314777	0
B40	B166	WBC	Pro	0.077854342	0
B40	B188	WBC	Ca	0.078175987	0
B40	B192	WBC	UA	0.092047831	0
B40	B206	WBC	Waist	0.143707779	0
B40	B209	WBC	Smoking	0.216585951	0
B40	B210	WBC	Hip	0.098087442	0
B40	B215	WBC	BMI	0.10463833	0
B40	B216	WBC	BMI	0.13179181	0
B40	B221	WBC	Hei	0.089958957	0
B40	B222	WBC	NS	0.13703924	0
B40	B41	WBC	MON％	-0.141830021	0
B40	B42	WBC	MON	0.663961179	0
B40	B46	WBC	RBC Count	0.177861575	0
B40	B48	WBC	LYM%	-0.189527578	0
B40	B51	WBC	Hb	0.160154653	0
B40	B52	WBC	PLT/BPC	0.245250551	0
B40	B53	WBC	GR%	0.198158985	0
B40	B71	WBC	TG	0.104308302	0
B40	B72	WBC	HDL-C	-0.167792479	0
B40	B91	WBC	TP	0.116256906	0
B40	B174	WBC	Urea/Cr	-0.068921795	6.95E-14
B40	B218	WBC	DBP	0.068745407	8.04E-14
B40	B163	WBC	SG	0.066495476	5.02E-13
B40	B170	WBC	U-GLU	0.066100914	6.88E-13
B40	B153	WBC	Glu	0.06569149	9.53E-13
B40	B112	WBC	FT4	0.06110839	3.17E-11
B40	B49	WBC	baso%	-0.060875785	3.77E-11
B40	B123	WBC	hs-CRP1	0.060684544	4.34E-11
B40	B160	WBC	PH	-0.055681985	1.47E-09
B40	B43	WBC	MCV	-0.054753461	2.73E-09
B40	B63	WBC	WBRSR(mPas_200)	0.053524124	6.13E-09

B40	B190	WBC	Cre	0.052740616	1.02E-08
B40	B175	WBC	ALB-U	0.051208785	2.68E-08
B40	B67	WBC	WBRSR(mPas_50)	0.050422333	4.36E-08
B40	B66	WBC	WBRSR(mPas_100)	0.049828261	6.26E-08
B40	B47	WBC	RDW	-0.047130375	3.09E-07
B40	B70	WBC	LDL-C	0.045320456	8.61E-07
B40	B58	WBC	HCT	0.045250533	8.95E-07
B41	B126	MON %	Alb	-0.127435929	0
B41	B131	MON %	AST	0.10239394	0
B41	B174	MON %	Urea/Cr	-0.084372858	0
B41	B189	MON %	eGFB	-0.15937412	0
B41	B190	MON %	Cre	0.104549872	0
B41	B42	MON %	MON	0.602822676	0
B41	B43	MON %	MCV	0.088418762	0
B41	B47	MON %	RDW	0.090152574	0
B41	B49	MON %	baso%	0.140395366	0
B41	B53	MON %	GR%	-0.23774016	0
B41	B71	MON %	TG	-0.097556242	0
B41	B132	MON %	Dbil	0.07431835	6.66E-16
B41	B222	MON %	NS	0.074004478	8.88E-16
B41	B50	MON %	eo%	0.072128338	4.44E-15
B41	B204	MON %	Drinking	0.071651361	6.88E-15
B41	B44	MON %	MCH	0.071372686	8.88E-15
B41	B221	MON %	Hei	0.069479735	4.35E-14
B41	B127	MON %	A-G ratio	-0.069316774	5.00E-14
B41	B77	MON %	TC	-0.06568153	9.61E-13
B41	B188	MON %	Ca	-0.062432344	1.18E-11
B41	B128	MON %	ALT	0.059478616	1.04E-10
B41	B192	MON %	UA	0.053503328	6.22E-09
B41	B91	MON %	TP	-0.052242078	1.40E-08
B41	B191	MON %	Urea	0.050484286	4.19E-08
B41	B211	MON %	IOP-R	-0.04601865	5.82E-07
B41	B153	MON %	Glu	-0.045873451	6.32E-07
B41	B209	MON %	Smoking	0.045498446	7.79E-07
B42	B126	MON	Alb	-0.08133427	0
B42	B127	MON	A-G ratio	-0.132128633	0
B42	B130	MON	GLB	0.121286734	0
B42	B163	MON	SG	0.084812057	0
B42	B166	MON	Pro	0.09140822	0
B42	B174	MON	Urea/Cr	-0.119221791	0
B42	B189	MON	eGFB	-0.090819524	0
B42	B190	MON	Cre	0.117624577	0
B42	B192	MON	UA	0.106859808	0
B42	B206	MON	Waist	0.144033603	0

B42	B209	MON	Smoking	0.206885525	0
B42	B210	MON	Hip	0.088160566	0
B42	B216	MON	BMI	0.120948741	0
B42	B221	MON	Hei	0.12275573	0
B42	B222	MON	NS	0.163983355	0
B42	B46	MON	RBC Count	0.134115177	0
B42	B48	MON	LYM%	-0.151214074	0
B42	B50	MON	eo%	0.083840711	0
B42	B51	MON	Hb	0.151598896	0
B42	B52	MON	PLT/BPC	0.179647665	0
B42	B72	MON	HDL-C	-0.111700728	0
B42	B133	MON	TBIL	-0.067873241	1.65E-13
B42	B129	MON	IBIL	-0.067246094	2.74E-13
B42	B123	MON	hs-CRP1	0.067238439	2.76E-13
B42	B215	MON	BMI	0.062538137	1.09E-11
B42	B49	MON	baso%	0.060361128	5.49E-11
B42	B91	MON	TP	0.060208879	6.14E-11
B42	B176	MON	Ucr	0.060101441	6.64E-11
B42	B58	MON	HCT	0.05845065	2.17E-10
B42	B204	MON	Drinking	0.058003213	2.97E-10
B42	B175	MON	ALB-U	0.055884526	1.28E-09
B42	B132	MON	Dbil	-0.050620569	3.86E-08
B42	B131	MON	AST	0.050496852	4.16E-08
B42	B63	MON	WBRSR(mPas_200)	0.047903505	1.97E-07
B42	B128	MON	ALT	0.046676403	4.01E-07
B42	B113	MON	FT3	0.045650049	7.16E-07
B43	B153	MCV	Glu	-0.098815555	0
B43	B170	MCV	U-GLU	-0.086500696	0
B43	B189	MCV	eGFB	-0.14480876	0
B43	B190	MCV	Cre	0.083709184	0
B43	B44	MCV	MCH	0.897794217	0
B43	B45	MCV	MCHC	0.152351087	0
B43	B46	MCV	RBC Count	-0.570217837	0
B43	B47	MCV	RDW	0.591627875	0
B43	B51	MCV	Hb	0.099558711	0
B43	B52	MCV	PLT/BPC	-0.152501837	0
B43	B72	MCV	HDL-C	0.108876808	0
B43	B141	MCV	AST/ALT	0.066922039	3.57E-13
B43	B218	MCV	DBP	-0.064816164	1.90E-12
B43	B53	MCV	GR%	-0.06130889	2.74E-11
B43	B209	MCV	Smoking	0.057505264	4.21E-10
B43	B204	MCV	Drinking	0.054195769	3.95E-09
B43	B191	MCV	Urea	0.052329755	1.32E-08
B43	B71	MCV	TG	-0.051319815	2.50E-08

B43	B91	MCV	TP	-0.04778489	2.11E-07
B43	B74	MCV	ApoA1	0.046040026	5.75E-07
B44	B190	MCH	Cre	0.076675183	0
B44	B204	MCH	Drinking	0.104348881	0
B44	B209	MCH	Smoking	0.105713195	0
B44	B221	MCH	Hei	0.090851106	0
B44	B222	MCH	NS	0.080465509	0
B44	B45	MCH	MCHC	0.569629807	0
B44	B46	MCH	RBC Count	-0.435420966	0
B44	B47	MCH	RDW	0.376964073	0
B44	B51	MCH	Hb	0.318036093	0
B44	B52	MCH	PLT/BPC	-0.136725816	0
B44	B174	MCH	Urea/Cr	-0.075175465	4.44E-16
B44	B127	MCH	A-G ratio	0.0742044	8.88E-16
B44	B140	MCH	r-GT	0.06646858	5.13E-13
B44	B130	MCH	GLB	-0.062663687	9.93E-12
B44	B53	MCH	GR%	-0.062423195	1.19E-11
B44	B163	MCH	SG	0.060935431	3.61E-11
B44	B189	MCH	eGFB	-0.060327101	5.63E-11
B44	B216	MCH	BMI	0.059852516	7.95E-11
B44	B71	MCH	TG	0.057506358	4.20E-10
B44	B48	MCH	LYM%	0.05062827	3.84E-08
B44	B206	MCH	Waist	0.048209327	1.65E-07
B44	B58	MCH	HCT	0.047220119	2.93E-07
B44	B133	MCH	TBIL	0.046547555	4.32E-07
B45	B126	MCHC	Alb	0.142988772	0
B45	B127	MCHC	A-G ratio	0.123346222	0
B45	B129	MCHC	IBIL	0.134700637	0
B45	B133	MCHC	TBIL	0.12234495	0
B45	B140	MCHC	r-GT	0.105072106	0
B45	B141	MCHC	AST/ALT	-0.113487444	0
B45	B153	MCHC	Glu	0.194164666	0
B45	B163	MCHC	SG	0.188015215	0
B45	B170	MCHC	U-GLU	0.17499088	0
B45	B174	MCHC	Urea/Cr	-0.095105755	0
B45	B176	MCHC	Ucr	0.086769777	0
B45	B189	MCHC	eGFB	0.135339201	0
B45	B204	MCHC	Drinking	0.120784207	0
B45	B206	MCHC	Waist	0.119906216	0
B45	B209	MCHC	Smoking	0.12046989	0
B45	B210	MCHC	Hip	0.095928119	0
B45	B215	MCHC	BMI	0.098669666	0
B45	B216	MCHC	BMI	0.214611498	0
B45	B218	MCHC	DBP	0.130829347	0

B45	B221	MCHC	Hei	0.230614221	0
B45	B222	MCHC	NS	0.229288133	0
B45	B46	MCHC	RBC Count	0.086124142	0
B45	B47	MCHC	RDW	-0.268816332	0
B45	B51	MCHC	Hb	0.533707614	0
B45	B56	MCHC	IR	0.078536959	0
B45	B58	MCHC	HCT	0.079855331	0
B45	B61	MCHC	WBHSRV	0.082584522	0
B45	B62	MCHC	WBHSRVI	0.096971964	0
B45	B63	MCHC	WBRSR(mPas_200)	0.097210755	0
B45	B66	MCHC	WBRSR(mPas_100)	0.094116339	0
B45	B67	MCHC	WBRSR(mPas_50)	0.094062944	0
B45	B71	MCHC	TG	0.22345131	0
B45	B72	MCHC	HDL-C	-0.157699289	0
B45	B74	MCHC	ApoA1	-0.080357247	0
B45	B172	MCHC	KB	0.074705853	4.44E-16
B45	B60	MCHC	WBRVI	0.074570645	4.44E-16
B45	B65	MCHC	WBRSR(mPas_1)	0.07480544	4.44E-16
B45	B130	MCHC	GLB	-0.071403454	8.44E-15
B45	B128	MCHC	ALT	0.068507481	9.77E-14
B45	B219	MCHC	OT	-0.066128233	6.74E-13
B45	B192	MCHC	UA	0.061328429	2.70E-11
B45	B77	MCHC	TC	0.06049398	4.99E-11
B45	B166	MCHC	Pro	0.059811598	8.19E-11
B45	B191	MCHC	Urea	-0.055545876	1.61E-09
B45	B54	MCHC	ESR	-0.053101174	8.07E-09
B45	B161	MCHC	LEU	-0.052875891	9.32E-09
B45	B193	MCHC	K	-0.052239464	1.40E-08
B45	B195	MCHC	Na	-0.051929011	1.70E-08
B45	B132	MCHC	Dbil	0.051831169	1.81E-08
B45	B194	MCHC	Cl	-0.050748315	3.56E-08
B45	B173	MCHC	NTT	-0.05020249	4.98E-08
B45	B138	MCHC	PAB	0.049120376	9.60E-08
B46	B113	RBC Count	FT3	0.112664261	0
B46	B126	RBC Count	Alb	0.259401385	0
B46	B127	RBC Count	A-G ratio	0.125704856	0
B46	B128	RBC Count	ALT	0.110708627	0
B46	B129	RBC Count	IBIL	0.127155621	0
B46	B133	RBC Count	TBIL	0.113562609	0
B46	B134	RBC Count	CHE	0.080069597	0
B46	B141	RBC Count	AST/ALT	-0.219488539	0
B46	B153	RBC Count	Glu	0.181772643	0
B46	B161	RBC Count	LEU	-0.114222203	0
B46	B163	RBC Count	SG	0.263860701	0

B46	B166	RBC Count	Pro	0.085708078	0
B46	B170	RBC Count	U-GLU	0.214888807	0
B46	B172	RBC Count	KB	0.078636194	0
B46	B173	RBC Count	NTT	-0.081473889	0
B46	B174	RBC Count	Urea/Cr	-0.121679266	0
B46	B176	RBC Count	Ucr	0.106512371	0
B46	B188	RBC Count	Ca	0.137765394	0
B46	B189	RBC Count	eGFB	0.255249351	0
B46	B191	RBC Count	Urea	-0.118320979	0
B46	B204	RBC Count	Drinking	0.114404004	0
B46	B206	RBC Count	Waist	0.185161952	0
B46	B209	RBC Count	Smoking	0.150952968	0
B46	B210	RBC Count	Hip	0.160655826	0
B46	B215	RBC Count	BMI	0.147919282	0
B46	B216	RBC Count	BMI	0.303312551	0
B46	B218	RBC Count	DBP	0.210504141	0
B46	B219	RBC Count	OT	-0.089569333	0
B46	B221	RBC Count	Hei	0.320245019	0
B46	B222	RBC Count	NS	0.328511036	0
B46	B47	RBC Count	RDW	-0.492306441	0
B46	B51	RBC Count	Hb	0.706125505	0
B46	B52	RBC Count	PLT/BPC	0.079324574	0
B46	B56	RBC Count	IR	0.095371956	0
B46	B58	RBC Count	HCT	0.189896129	0
B46	B59	RBC Count	WBRV	0.077253968	0
B46	B60	RBC Count	WBRVI	0.155714266	0
B46	B61	RBC Count	WBHSRV	0.101297768	0
B46	B62	RBC Count	WBHSRVI	0.158071783	0
B46	B63	RBC Count	WBRSR(mPas_200)	0.158602515	0
B46	B65	RBC Count	WBRSR(mPas_1)	0.157218462	0
B46	B66	RBC Count	WBRSR(mPas_100)	0.159722255	0
B46	B67	RBC Count	WBRSR(mPas_50)	0.163021804	0
B46	B70	RBC Count	LDL-C	0.11141218	0
B46	B71	RBC Count	TG	0.124388999	0
B46	B72	RBC Count	HDL-C	-0.189425615	0
B46	B74	RBC Count	ApoA1	-0.087549647	0
B46	B75	RBC Count	ApoA1/B	-0.083397811	0
B46	B77	RBC Count	TC	0.078782338	0
B46	B91	RBC Count	TP	0.115776261	0
B46	B54	RBC Count	ESR	-0.0730571	2.00E-15
B46	B115	RBC Count	TSH TT3 TT4(TT3)	0.071420428	8.44E-15
B46	B140	RBC Count	r-GT	0.071338924	8.88E-15
B46	B192	RBC Count	UA	0.067996337	1.49E-13
B46	B64	RBC Count	WBRSR(mPas_5)	0.067310591	2.60E-13

B46	B111	RBC Count	TSH	-0.06339006	5.72E-12
B46	B120	RBC Count	HCV-cAg	-0.059702572	8.86E-11
B46	B194	RBC Count	Cl	-0.057381567	4.58E-10
B46	B149	RBC Count	TGAb	-0.055732947	1.42E-09
B46	B76	RBC Count	ApoB	0.049984274	5.69E-08
B46	B220	RBC Count	SBP	-0.049657145	6.95E-08
B46	B138	RBC Count	PAB	0.049487208	7.70E-08
B46	B49	RBC Count	baso%	0.048780382	1.18E-07
B46	B160	RBC Count	PH	-0.048468951	1.41E-07
B46	B146	RBC Count	CysC	-0.046037039	5.76E-07
B46	B130	RBC Count	GLB	-0.045619322	7.29E-07
B47	B126	RDW	Alb	-0.112121616	0
B47	B132	RDW	Dbil	0.086906834	0
B47	B134	RDW	CHE	-0.082631165	0
B47	B141	RDW	AST/ALT	0.10766492	0
B47	B153	RDW	Glu	-0.169621273	0
B47	B163	RDW	SG	-0.105215667	0
B47	B170	RDW	U-GLU	-0.142170104	0
B47	B189	RDW	eGFB	-0.223101323	0
B47	B190	RDW	Cre	0.117341416	0
B47	B191	RDW	Urea	0.104349989	0
B47	B216	RDW	BMI	-0.117881337	0
B47	B218	RDW	DBP	-0.104486299	0
B47	B221	RDW	Hei	-0.105031873	0
B47	B222	RDW	NS	-0.081704669	0
B47	B51	RDW	Hb	-0.2323588	0
B47	B52	RDW	PLT/BPC	-0.197411689	0
B47	B71	RDW	TG	-0.076688601	0
B47	B72	RDW	HDL-C	0.123079108	0
B47	B131	RDW	AST	0.073683409	1.33E-15
B47	B74	RDW	ApoA1	0.07308625	2.00E-15
B47	B146	RDW	CysC	0.072011869	5.11E-15
B47	B181	RDW	APTT	0.069824473	3.26E-14
B47	B215	RDW	BMI	-0.069085877	6.06E-14
B47	B188	RDW	Ca	-0.063992672	3.60E-12
B47	B127	RDW	A-G ratio	-0.062653012	1.00E-11
B47	B75	RDW	ApoA1/B	0.059242622	1.23E-10
B47	B70	RDW	LDL-C	-0.05894451	1.53E-10
B47	B195	RDW	Na	0.055233984	1.98E-09
B47	B63	RDW	WBRSR(mPas_200)	-0.054151344	4.07E-09
B47	B111	RDW	TSH	0.053131109	7.91E-09
B47	B219	RDW	OT	0.052366028	1.29E-08
B47	B112	RDW	FT4	-0.051417514	2.35E-08
B47	B77	RDW	TC	-0.051052733	2.95E-08

B47	B66	RDW	WBRSR(mPas_100)	-0.050063877	5.43E-08
B47	B67	RDW	WBRSR(mPas_50)	-0.049888568	6.04E-08
B47	B61	RDW	WBHSRV	-0.049852997	6.17E-08
B47	B194	RDW	CI	0.049238529	8.94E-08
B47	B62	RDW	WBHSRVI	-0.048702522	1.23E-07
B47	B209	RDW	Smoking	0.047540906	2.44E-07
B47	B211	RDW	IOP-R	-0.046586234	4.22E-07
B47	B210	RDW	Hip	-0.046339526	4.86E-07
B48	B132	LYM%	Dbil	-0.078065014	0
B48	B153	LYM%	Glu	-0.076931307	0
B48	B53	LYM%	GR%	-0.936046118	0
B48	B70	LYM%	LDL-C	0.086981462	0
B48	B72	LYM%	HDL-C	0.093117081	0
B48	B77	LYM%	TC	0.095053731	0
B48	B189	LYM%	eGFB	0.073837522	8.88E-16
B48	B91	LYM%	TP	0.070963105	1.24E-14
B48	B190	LYM%	Cre	-0.070041914	2.71E-14
B48	B170	LYM%	U-GLU	-0.065605497	1.02E-12
B48	B166	LYM%	Pro	-0.065068366	1.56E-12
B48	B123	LYM%	hs-CRP1	-0.061906109	1.76E-11
B48	B209	LYM%	Smoking	-0.058535218	2.04E-10
B48	B133	LYM%	TBIL	-0.058492225	2.10E-10
B48	B163	LYM%	SG	-0.050395877	4.43E-08
B48	B126	LYM%	Alb	0.047490697	2.51E-07
B48	B130	LYM%	GLB	0.046758052	3.83E-07
B48	B129	LYM%	IBIL	-0.045525317	7.68E-07
B49	B50	baso%	eo%	0.269393443	0
B49	B52	baso%	PLT/BPC	0.130099514	0
B49	B53	baso%	GR%	-0.176206167	0
B49	B129	baso%	IBIL	-0.053622052	5.75E-09
B49	B133	baso%	TBIL	-0.050984789	3.08E-08
B49	B140	baso%	r-GT	0.049124954	9.57E-08
B49	B221	baso%	Hei	0.047672072	2.26E-07
B5	B121	WB-Mg	C3	0.08115709	0
B5	B122	WB-Mg	C4	-0.12528822	0
B5	B29	WB-Mg	E2	0.129770899	0
B5	B32	WB-Mg	Testo	-0.107503107	0
B5	B33	WB-Mg	Prog	0.104072151	0
B5	B6	WB-Mg	WB-Fe	0.79870952	0
B5	B7	WB-Mg	WB-Cop	0.173416642	0
B5	B8	WB-Mg	WB-Zinc	0.247679333	0
B5	B120	WB-Mg	HCV-cAg	0.07522903	4.44E-16
B5	B158	WB-Mg	IgM	0.069078232	6.08E-14
B5	B31	WB-Mg	FSH	-0.047174083	3.01E-07

B50	B189	eo%	eGFB	-0.097600767	0
B50	B190	eo%	Cre	0.108493101	0
B50	B209	eo%	Smoking	0.077767835	0
B50	B53	eo%	GR%	-0.274165774	0
B50	B174	eo%	Urea/Cr	-0.069160686	5.68E-14
B50	B221	eo%	Hei	0.056772474	6.99E-10
B50	B222	eo%	NS	0.055797769	1.36E-09
B50	B126	eo%	Alb	-0.055787289	1.37E-09
B50	B192	eo%	UA	0.050572856	3.97E-08
B51	B113	Hb	FT3	0.122136469	0
B51	B126	Hb	Alb	0.301834817	0
B51	B127	Hb	A-G ratio	0.185868129	0
B51	B128	Hb	ALT	0.115005147	0
B51	B129	Hb	IBIL	0.161785317	0
B51	B130	Hb	GLB	-0.093637461	0
B51	B133	Hb	TBIL	0.148437205	0
B51	B134	Hb	CHE	0.08213438	0
B51	B138	Hb	PAB	0.082075221	0
B51	B140	Hb	r-GT	0.120091935	0
B51	B141	Hb	AST/ALT	-0.228096437	0
B51	B153	Hb	Glu	0.194668395	0
B51	B161	Hb	LEU	-0.133341374	0
B51	B163	Hb	SG	0.326585828	0
B51	B166	Hb	Pro	0.105692674	0
B51	B170	Hb	U-GLU	0.229908083	0
B51	B172	Hb	KB	0.105994055	0
B51	B173	Hb	NTT	-0.10111626	0
[truncated: 403,625 more chars]
